# Metastatic recurrence in colorectal cancer arises from residual EMP1+ cells

**DOI:** 10.1038/s41586-022-05402-9

**Published:** 2022-11-09

**Authors:** Adrià Cañellas-Socias, Carme Cortina, Xavier Hernando-Momblona, Sergio Palomo-Ponce, Eoghan J. Mulholland, Gemma Turon, Lidia Mateo, Sefora Conti, Olga Roman, Marta Sevillano, Felipe Slebe, Diana Stork, Adrià Caballé-Mestres, Antonio Berenguer-Llergo, Adrián Álvarez-Varela, Nicola Fenderico, Laura Novellasdemunt, Laura Jiménez-Gracia, Tamara Sipka, Lidia Bardia, Patricia Lorden, Julien Colombelli, Holger Heyn, Xavier Trepat, Sabine Tejpar, Elena Sancho, Daniele V.F. Tauriello, Simon Leedham, Camille Stephan-Otto Attolini, Eduard Batlle

**Affiliations:** 1 Institute for Research in Biomedicine (IRB Barcelona), The Barcelona Institute of Science and Technology (BIST), Barcelona, Spain; 2 Centro de Investigación Biomédica en Red de Cáncer (CIBERONC), Barcelona, Spain; 3 Gastrointestinal Stem Cell Biology Lab, Wellcome Centre Human Genetics, University of Oxford, Oxford, UK; 4 Institute for Bioengineering of Catalonia (IBEC), The Barcelona Institute of Science and Technology (BIST), Barcelona, Spain; 5 CNAG-CRG, Centre for Genomic Regulation, The Barcelona Institute of Science and Technology (BIST), Barcelona, Spain; 6 Universitat Pompeu Fabra (UPF), Barcelona, Spain; 7 Facultat de Medicina, Universitat de Barcelona, Barcelona, Spain; 8 Centro de Investigación Biomédica en Red en Bioingeniería, Biomateriales y Nanomedicina (CIBER-BBN), Barcelona, Spain; 9 Institució Catalana de Recerca i Estudis Avançats (ICREA), Barcelona, Spain; 10 Digestive Oncology, Department of Oncology, Katholieke Universiteit Leuven, Leuven, Belgium; 11 Department of Cell Biology, Radboud Institute for Molecular Life Sciences, Radboud University Medical Center, Nijmegen, The Netherlands; 12 Translational Gastroenterology Unit, John Radcliffe Hospital, University of Oxford and Oxford National Institute for Health Research Biomedical Research Centre, Oxford, UK

## Abstract

30-40% of colorectal cancer (CRC) patients undergoing curative resection of the primary tumor will develop metastases in the following years^1^. Therapies to prevent disease relapse remain an unmet medical need. Here we uncover the identity and features of the residual tumor cells responsible for CRC relapse. Analysis of single-cell transcriptomes of CRC patient samples revealed that the majority of poor prognosis genes are expressed by a unique tumor cell population that we named High Relapse Cells (HRCs). We established a human-like mouse model of microsatellite stable CRC that undergoes metastatic relapse following surgical resection of the primary tumor. Residual HRCs occult in mouse livers after primary CRC surgery gave rise to multiple cell types over time, including Lgr5+ stemlike tumor cells^2–4^, and caused overt metastatic disease. Using *Emp1* (epithelial membrane protein 1) as a marker gene for HRCs, we tracked and selectively eliminated this cell population. Genetic ablation of Emp1-high cells prevented metastatic recurrence and mice remained disease-free after surgery. We also discovered that HRC-rich micrometastases were T-cell infiltrated yet became progressively immune-excluded during outgrowth. Treatment with neoadjuvant immunotherapy eliminated residual metastatic cells and saved mice from relapsing after surgery. Together, our findings reveal the cell-state dynamics of the residual disease in CRC and anticipate that therapies targeting HRCs may help avoid metastatic relapse.

## The CRC poor prognosis transcriptome

**Extended Data Fig. 1 |:**
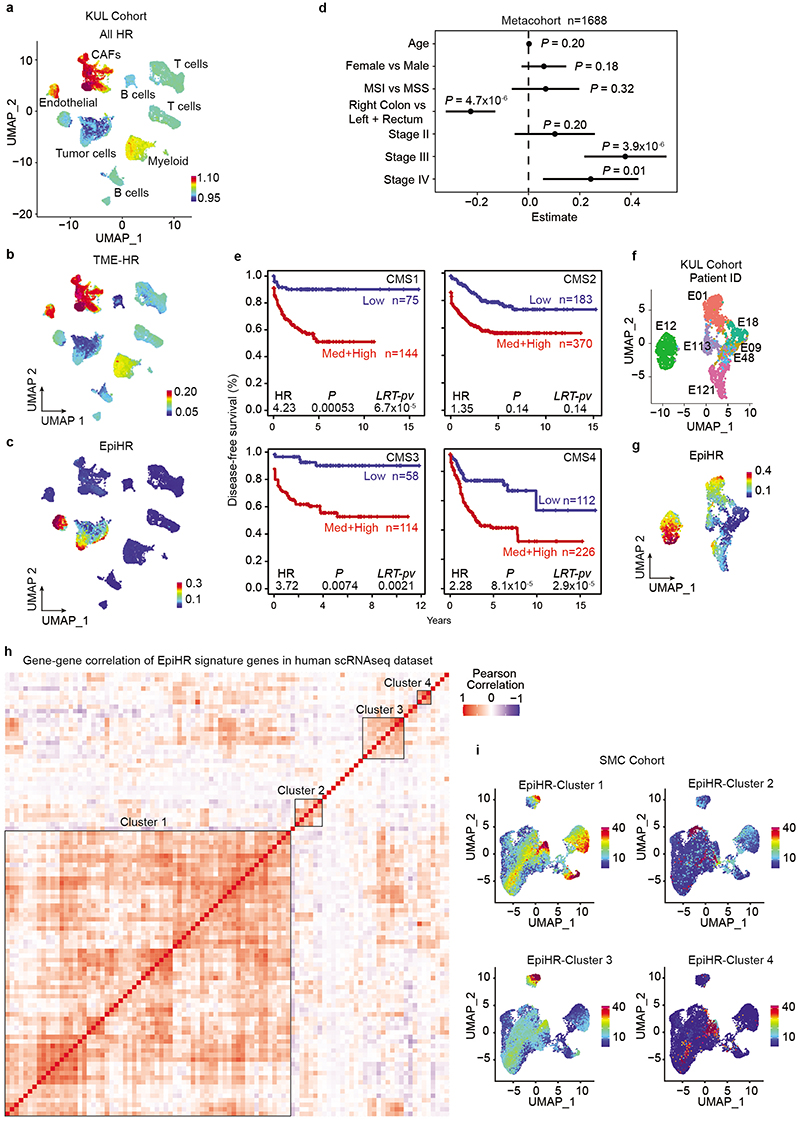
The EpiHR geneset marks a defined tumor cell population across CRCs. **a-c**, UMAP layout of whole tumors (stroma + epithelium cells) from 7 CRC patients in the KUL dataset. Colored by **(a)** gene expression of all high hazard ratio genes (AllHR), **(b)** tumor microenvironment-specific HR genes (TME-HR), and **(c**) epithelial-specific HR genes (EpiHR). **d,** Association between clinical variables and the EpiHR signature in the CRC meta cohort was assessed by fitting a linear model for each variable independently. Technical factors (dataset and center, as described in extended methods) were included as covariates. Lines show the left and right confidence intervals. n= 1688 patients. **e**. Kaplan-Meier survival curves indicating relapse-free survival according to EpiHR gene signature expression for CRC patients classified by CMS. Two-sided Wald test. **f-g,** UMAP layout of 2718 CRC tumor cells from the KUL cohort colored by **f)** patient ID and **g)** expression of the EpiHR signature. **h,** Heatmap showing Pearson correlation scores in gene expression among EpiHR signature genes in patients from the SMC cohort. Note that most genes belong to one coherent subset (Cluster 1). Gene lists are detailed in Supplementary Table 2. **i,** UMAP layout of human CRC tumor cells colored by the expression of genes belonging to Clusters 1, 2, 3 and 4 identified in (**h**).

**Fig. 1 |:**
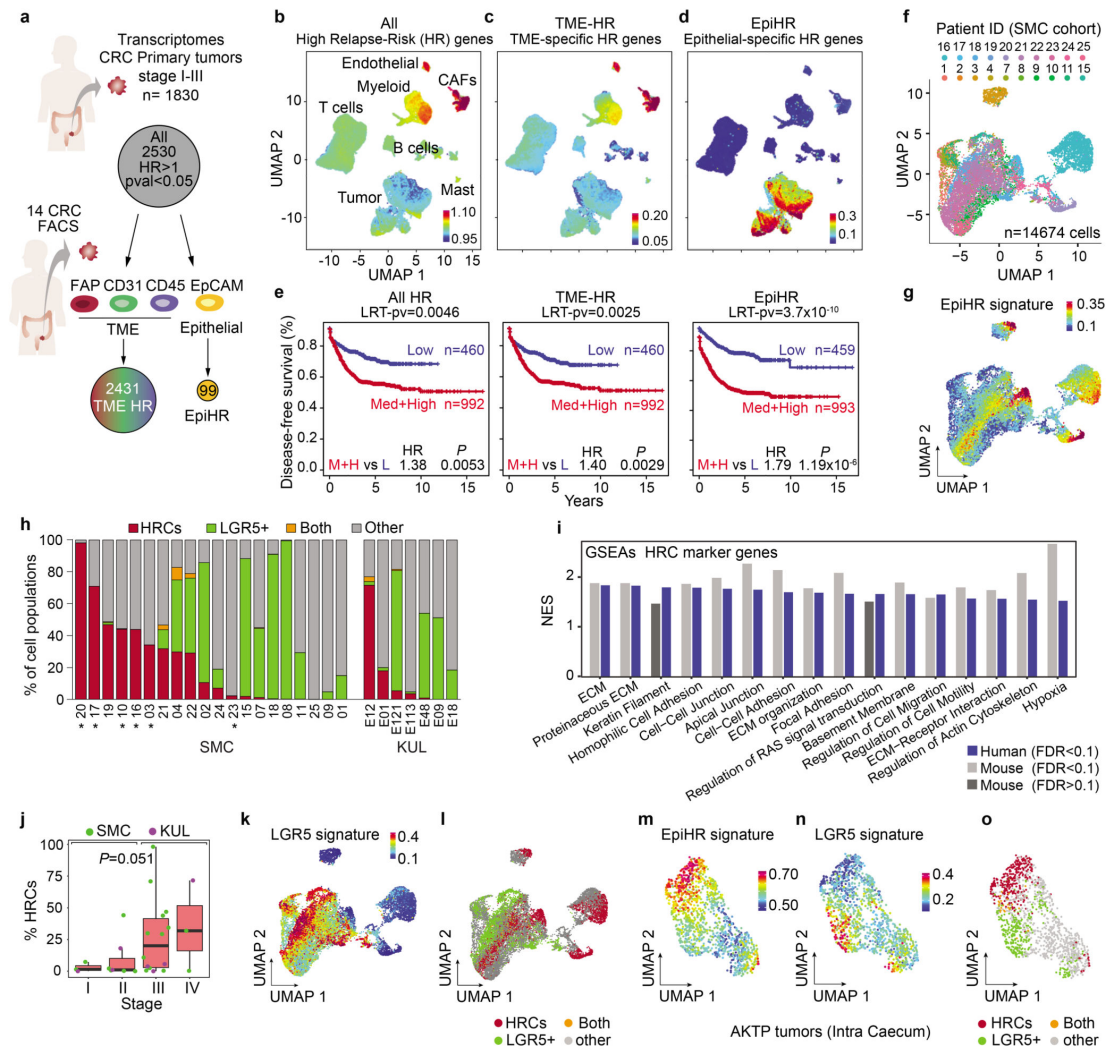
Identification of poor prognosis epithelial CRC cells **a,** Identification of TME-HR and EpiHR signatures in a metacohort of 7 pooled human stage I-III CRC datasets (n= 1830 patients, Supplementary Table 1). **b-d,** UMAP layout of whole tumors (stroma + epithelium cells) belonging to the SMC dataset, colored by AllHR **(b)**, TME-HR **(c)** and epithelial-specific HR genes **(d)**. **e,** Kaplan-Meier survival curves indicating relapse-free survival. Two-sided Wald test. Likelihood Ratio Test p-values (LRT-pv) are specified. **f,g**, UMAP layout of 14674 CRC tumor cells (SMC cohort) colored by patient ID **(f)** and expression of the EpiHR signature **(g)**. **h,** Barplot quantifying the sub-population composition of each patient in the SMC (left) and KUL (right) datasets. Patient ID is detailed. Patients with low WNT signature scores are marked with an “*”. **i,** Selected Hallmarks, GOSLIM and KEGG gene signatures enriched in HRCs compared to the rest of tumor cells in human and mouse CRC samples. **j,** Boxplot representing the proportion of HRCs in each clinical stage. Box plots have whiskers of maximum 1.5 times the interquartile range. Boxes represent first, second (median) and third quartiles. n= 3, 7, 14, 3, patients from left to right. Two-sided Kruskal-Wallis test. **k,** UMAP of tumor cells colored by expression of the LGR5 signature. **l,** UMAP of tumor cells labelled according to their classification as HRCs, LGR5+, double positive or other cells. **m-o,** Primary tumors were generated in the caecum of c57BL/6J mice by injecting syngeneic AKTP MTOs. UMAPs depicting GFP+ tumor cells dissociated from primary tumors colored by expression levels of (**m**) EpiHR signature, (**n**) Lgr5 signature and (**o**) their classification as HRCs, Lgr5+, double positive or other cells.

Surgical resection of the primary CRC effectively cures most patients diagnosed with locoregional disease^1^. However, about 5% of AJCC Stage I, 15% of Stage II and 40% of Stage III patients will develop metastases over the following years^1^. We and others have previously shown that the vast majority of genes that predict high risk of disease relapse in CRC are expressed by cells of the tumor microenvironment (TME), particularly by cancer-associated fibroblasts (CAFs)^5–7^. To further investigate this finding, we sought to map the expression of the poor prognosis CRC transcriptome at the single-cell level. Using a large pooled transcriptomic cohort of primary CRC samples (n=1830 stage I-III CRC, Supplementary Table 1), we identified 2530 genes that predicted disease relapse (HR>1, p-val<0.05) (Fig. 1a). Subsequently, the expression of this poor prognosis geneset was analyzed in two independent single cell RNA sequencing (scRNAseq) CRC datasets that included both tumor epithelial and microenvironment cells; 20 patients corresponding to the Samsung Medical Center (SMC) cohort and 7 patients from the Katholieke Universiteit Leuven (KUL) cohort^8^ (Fig. 1b-d and Extended Data Fig. 1a-c). Supporting our previous findings, CAFs, endothelial cells and, to a lower extent, myeloid cells expressed the highest levels of poor prognosis genes (Fig. 1b and Extended Data Fig. 1a). However, detailed analysis of the recurrence geneset in specific cell populations purified from primary CRC patient samples (tumor cells/EPCAM+, leukocytes/CD45+, endothelial cells/CD31+ and CAFs/FAP+)^6^ revealed that 99 out of the 2530 genes were upregulated in epithelial tumor cells compared to TME cells (Fig. 1a and Supplementary Table 2). Indeed, these 99 recurrence-associated genes (from now on named EpiHR for Epithelial-specific high-risk geneset), showed epithelial tumor cell-restricted expression patterns in the SMC (Fig. 1d) and KUL (Extended Data Fig. 1c) scRNAseq cohorts. EpiHR expression levels predicted recurrence with an accuracy equivalent to the subset of poor prognosis genes expressed in the tumor microenvironment (Fig. 1e). In multivariate analysis including the two signatures and clinical variables, the TME-HR and EpiHR genesets were independent prognostic factors (EpiHR: HR (+1 SD) = 2.26, p-val=1.2x10^7^; TME-HR: HR (+1 SD) =1.74, p-val=8x10^-4^). The EpiHR signature was significantly associated with right-side colon cancer and to AJCC Stages III-IV (Extended Data Fig. 1d). In addition, it stratified CRC patients within each consensus molecular subtype^9^ (CMS) into high and low risk of relapse (Extended Data Fig. 1e). Thus, the EpiHR geneset encodes determinants of disease relapse with epithelial tumor cell-specific expression.

## Identification of EpiHR+ tumor cells

**Extended Data Fig. 2 |:**
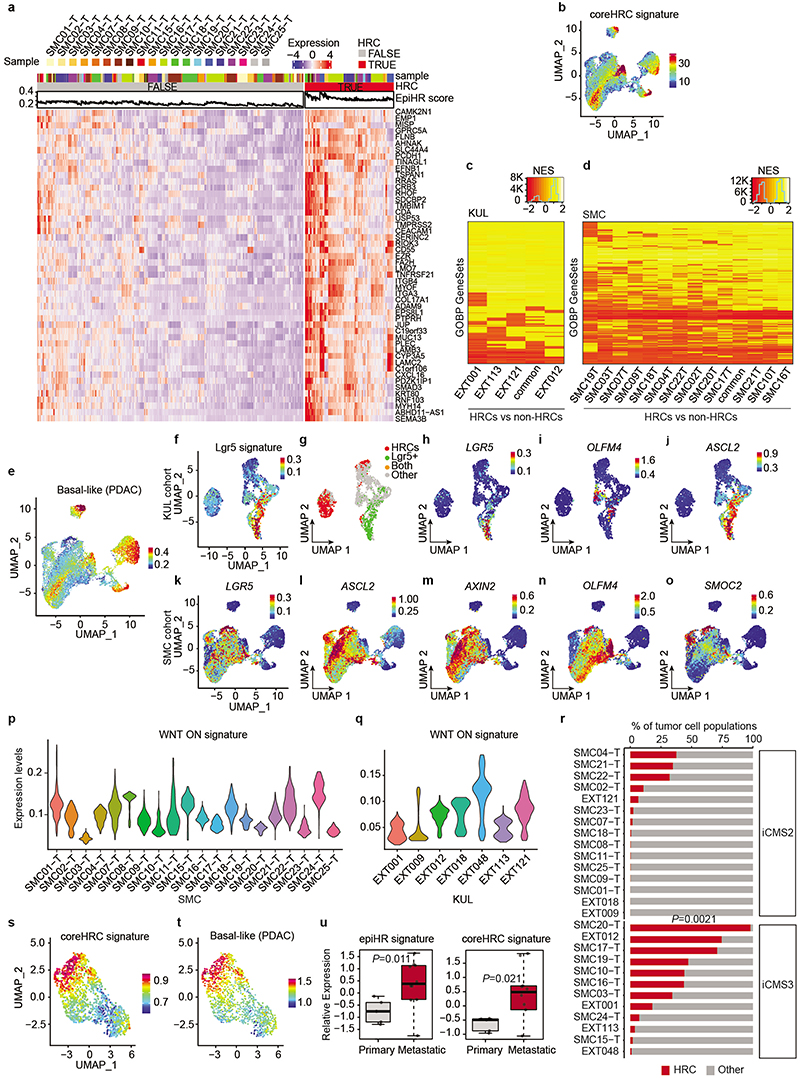
Characterization of HRC features. **a,** Heatmap showing scaled expression of the top 50 most correlated genes with the EpiHR signature across SMC patients. Tumor cells are divided as non-HRCs (left, FALSE) and HRCs (right, TRUE). The EpiHR signature score for each individual cell is plotted above the heatmap. **b,** UMAP layout of CRC tumor cells colored by the expression of the coreHRC signature. The coreHRC signature is defined as the top 100 genes with better correlation with the EpiHR signature. **c-d,** Heatmap showing Normalized Enrichment Scores (NES) for Genesets in Gene Ontology Biological Processes (GOBP) in HRCs from different patients in the KUL **(c)** and SMC (**d**) cohorts. Only GOBP genesets with NES scores above 0.5 are shown. Genesets and patients are ordered by hierarchical clustering. **e,** UMAP layout of human CRC tumor cells in the SMC cohort painted with the Basal cell state signature in Pancreatic Ductal Adenocarcinoma (PDAC) by Raghavan et al^10^. **f,** UMAP layout of tumor cells from the KUL cohort showing the expression of the Lgr5 signature. **g,** UMAP of same tumor cells labelled according to their classification into HRCs, Lgr5+, double positive or other cells. **h-j,** UMAP layout of same tumor cells showing gene expression levels of canonical intestinal stem cell genes *LGR5, OLFM4* and *ASCL2*. **k-o,** UMAPs of tumor cells in the SMC dataset showing gene expression levels of canonical intestinal stem cell genes *LGR5, ASCL2, AXIN2, OLFM4*, and *SMOC2*. **p,q,** Violin plots showing WNT-ON signature expression levels in epithelial tumor cells from patients in the SMC (**p**) and KUL (**q**) cohorts. **r,** Barplot quantifying the HRC composition of each patient (combined SMC and KUL datasets). Patients are classified as iCMS2 or iCMS3 according to Joanito et al^11^. Two-sided Kruskal-Wallis test. **s,t,** UMAPs of mouse CRC AKTP tumor cells colored according to (**s**) the coreHRC signature and (**t**) the Basal cell state signature in Pancreatic cancer by Raghavan et al.^10^ **u,** Gene expression levels of EpiHR (left) and coreHRC (right) signatures in MTOs derived from primary tumors or from liver metastases. Boxes represent the first, second (median) and third quartiles. Whiskers indicate maximum and minimum values. Welch two-sided t-test. n= 5 (primary) 10 (metastatic).

Representation of tumor epithelial CRC cells using Uniform Manifold Approximation and Projections (UMAPs) showed that 18 out of 27 CRCs contained cells labeled with the EpiHR geneset in proportions ranging from 1.4% to 98.1% (Fig. 1f-h and Extended Data Fig. 1f,g). We named this tumor cell population HRCs (for High Relapse Cells). Analysis of the EpiHR signature revealed a large number of highly correlated genes exhibiting overlapping expression in HRCs (Gene clusters 1 and 3 in Extended Data Fig. 1h,i and Supplementary Table 2). The EpiHR only contains epithelial-specific genes, reflecting only a fraction of the HRC transcriptome. We further identified a core gene expression program upregulated in HRCs of most tumor samples (Extended Data Fig. 2a-b and Supplementary Table 2). HRCs belonging to different CRCs displayed a shared enrichment pattern of annotated genesets, implying that they co-opt a similar phenotype and play equivalent functions (Extended Data Fig. 2c-d). Differential expression analysis of HRCs versus non-HRCs revealed prominent enrichment in genes related to hypoxia, cell-to-cell adhesion, extracellular matrix, actin cytoskeleton and regulation of cell migration (Fig. 1i). Amongst them (Supplementary Table 2), the collagen sensing receptor DDR1, the integrins α2, α3 and β4 or the protease PLAUR have been repeatedly associated with tumor cell invasion, extravasation and metastasis in multiple cancer types. Incidentally, we also discovered that the signature of basal-like pancreatic cancer cells^10^ marked both human and mouse HRCs suggesting that they adopt a state akin to the most aggressive subtype of pancreatic cancer (Extended Data Fig. 2e and Supplementary Table 3). Further reinforcing our observations, most scRNAseq samples containing abundant EpiHR+ cells were stage III and IV CRCs (Fig. 1j – p=0.051 Stage I+II versus III+IV).

Widespread evidence has demonstrated that CRC growth is driven by a subset of LGR5+ stem cell-like tumor cells^2–4^. However, our analyses revealed that HRCs represent a distinct cell population, shown by their mutually exclusive distribution in UMAPs (Fig. 1k-l, Extended Data Fig. 2f-g). Quantification showed that only one tumor sample exhibited a significant number of HRCs co-expressing the LGR5 signature (7.79% sample SMC04, Fig. 1h) whereas five others included a minimal fraction (<3%) of cells marked by both *LGR5* and the HRC gene programs. The expression patterns of several WNT/intestinal stem cell marker genes confirmed that HRCs were not LGR5+ stem-like tumor cells (Extended Data Fig. 2h-o). Indeed, a subset of CRCs exhibited marginal WNT target gene expression levels (Extended Data Fig. 2p-q) yet contained HRCs (identified with an * in Fig. 1h). Furthermore, HRCs represented a subset of cells in both epithelial intrinsic consensus molecular CRC subtypes iCMS2 and iCMS3^11^ (Extended Data Fig. 2r).

We previously described that mice bearing mutations in *Apc, Kras, Tgfbr2 and p53* (AKTP) in Lgr5+ ISCs develop human-like metastatic CRCs^12^. We analyzed CRCs generated by implantation of AKTP mouse tumor organoids (MTOs) in the caecum of c57BL/6 mice by scRNAseq (Fig. 1m-o). Mirroring the observations in human tumor samples, we found that mouse CRCs contained abundant HRCs and that this population did not express the Lgr5+ ISC-like expression program (Fig. 1m-o and Extended Data Fig. 2s). HRCs were enriched in similar gene categories in both species, including the pancreatic basal-like signature, implying functional equivalence (Fig. 1i and Extended Data Fig. 2s-t). Bulk RNA sequencing analysis of our MTO biobank^12^ showed elevated EpiHR and coreHRC gene signatures in AKTP MTOs derived from metastases compared to those established from primary CRCs (Extended Data Fig. 2u).

## Dynamics of metastatic cell states

**Extended Data Fig. 3 |:**
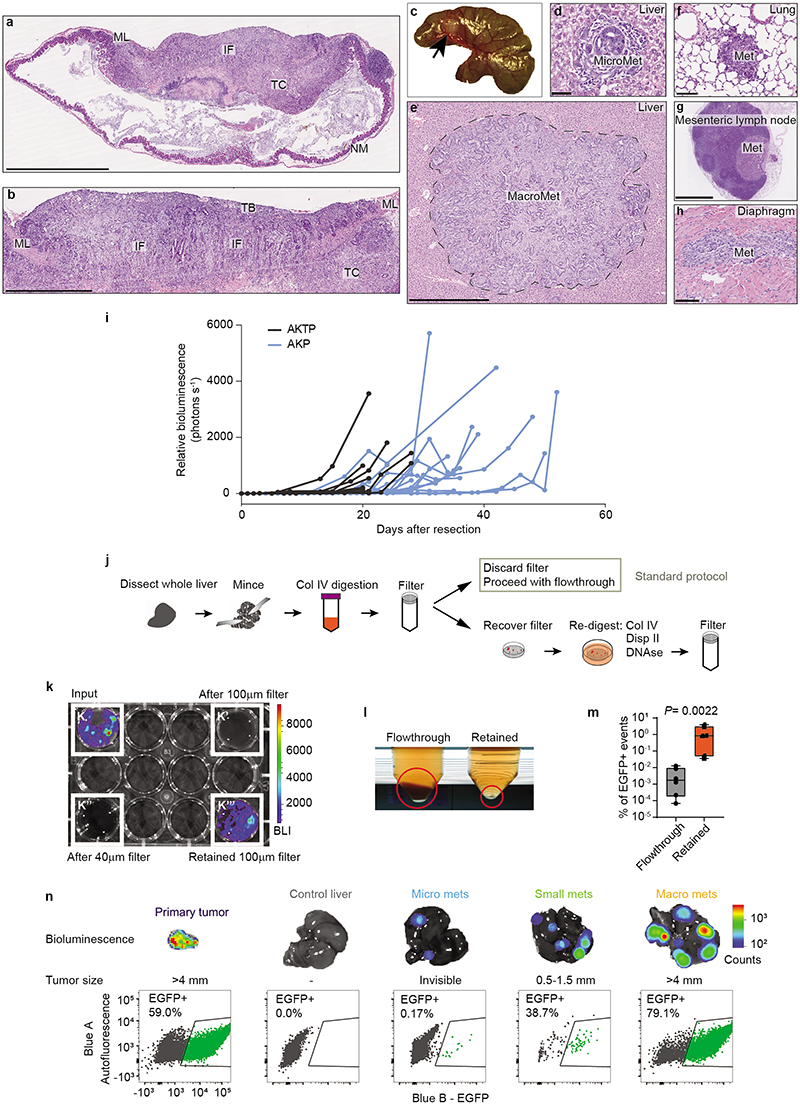
Analysis of the CRC relapse mouse models and purification of DTCs. **a,** Representative micrograph of hematoxylin- and eosin (HE)-stained adenocarcinoma with subserosal invasion (T4) generated by injection of an AKTP MTO in the mouse caecum. Tumor center (TC), invasive fronts (IF), muscle layer (ML) and normal mucosa (NM) are indicated. Scale bar, 2.5 mm. **b,** Representative image of a different T4 tumor penetrating the muscle layer (ML) and reaching the serosa layer. TB: Tumor buds. Scale bar, 1mm. **c,** Picture of a caecum 21 days after injection and imaged at the time of surgery showing a primary tumor (arrow) in the distal part. **d-e,** Haematoxilin-Eosin (HE) staining of micrometastases and large metastases observed in the liver of orthotopic isografted mouse. Scale bars, 50 μm and 1 mm, respectively. In **e,** tumoral tissue is surrounded by dashed lines. **f-h,** HE staining of lung, lymph node and diaphragm metastases from orthotopic isografted mice. Scale bars, 100 μm (**f** and **h**) 1 mm (**g**). **i,** Graph showing liver longitudinal BLI measurements (photons per second), normalized to the day of primary tumor resection. Points and lines represent individual mice. n= 9 (AKTP), 24 (AKP) mice. **j,** Schematic representation of a novel tissue-dissociation strategy that enables recovery of DTCs from livers. Whole livers are dissected and minced thoroughly. After a mild collagenase IV digestion, samples are filtered through 100 μm meshes. The filter retained sample is highly enriched in tumor cells. Remaining tissue in the filter is re-digested with a stronger enzymatic cocktail to fully digest it, and then re-filtered. **k,** Representative bioluminescent image of a whole liver sample containing luciferase+ tumor cells before enzymatic digestion (B, input), after filtering through 100 μm (B’) and 40 μm (B”) meshes (previous protocol), and after recovering and redigesting tissue retained in the 100 μm filter (B”’). **l,** Image showing the large cell pellet containing liver cells after 1 mild digestion and the small pellet in the retained and re-digested sample enriched in DTCs. **m,** Percentage of GFP+ cells measured by flow cytometry in samples with 1 round of digestion compared to re-digested samples. Boxes represent the first, second (median) and third quartiles. Whiskers indicate maximum and minimum values. Paired two-sided Wilcoxon test on percentages. n=6 independent paired samples examined in 2 independent experiments. **n,** Representative bioluminescent images, tumor burden and flow-cytometry plots of the 4 different stages analyzed by single-cell Smart-sequencing described in Fig. 2. Micrometastases samples were DTCs collected from livers with absent or low bioluminescence in which metastases were not visible. For small metastases samples, metastatic nodules were visible but small in size (<1.5mm). Macrometastases samples were metastatic nodules larger than 4mm.

**Fig. 2 |:**
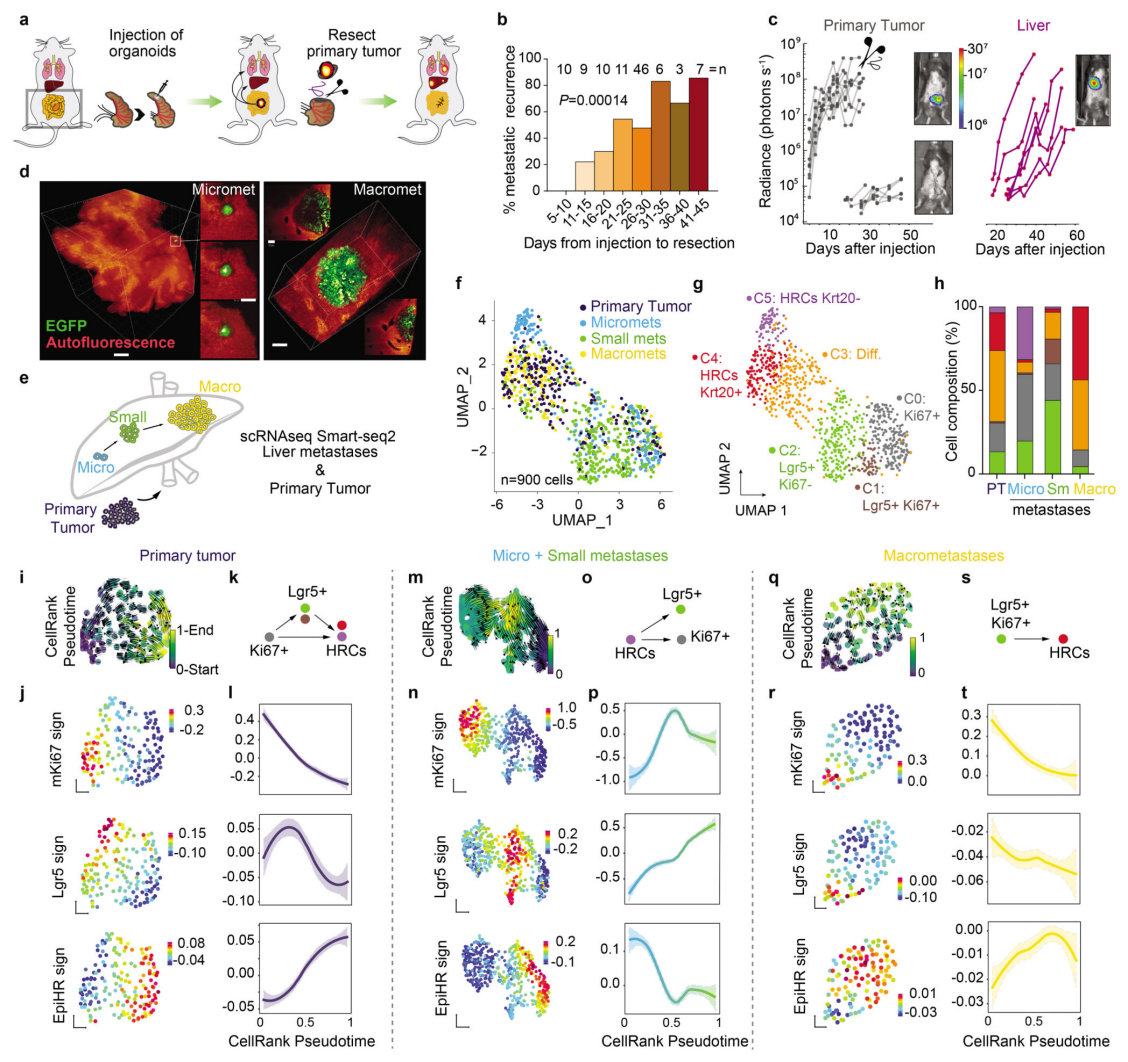
Spatiotemporal dynamics of CRC metastases resolved by scRNAseq **a,** Schematic representation of the mouse model of CRC metastatic relapse developed herein. **b,** Percentage of metastatic recurrence depending on time to primary tumor resection. Number of mice are detailed above the barplot. P-value for generalized linear model. **c,** Intravital bioluminescence imaging (BLI) quantification (photons s^-1^) of a representative experiment. Grey points and lines represent bioluminescence in the lower thorax of individual mice and purple points in the liver. Representative images of bioluminescence in the same mouse before, after surgery and upon liver metastases formation are shown. **d,** Representative images of whole livers containing GFP-expressing tumor cells obtained using lightsheet microscopy. Scale bars, left image (300 μm on Maximum Intensity Projections (MIP, and selected single plane insets 50 μm); right (100 μm on MIP and single plane insets 50 μm). **e,** Illustration of the longitudinal single cell RNA-expression analysis of tumor cells along the metastatic cascade. **f, g,** UMAP layout of 900 tumor cells isolated from 7 different mice colored by (**f**) metastatic stage and (**g**) Seurat clusters. **h,** Barplot showing Seurat cluster composition by sample stage. **i, m, q,** Vector fields representing RNA velocity projected on UMAPs of primary tumors (**i**), micro + small metastases (**m**) and macrometastases (**q**). Colored by the pseudotime estimated for each cell with scVelo. **j, n, r,** UMAPs with cells separated in primary tumors (**j**), micro+small metastases (**n**) and macrometastases (**r**) and colored by gene expression of mKi67, Lgr5 and EpiHR gene signatures. **k, o, s,** Schematics showing distinct hierarchical behavior during the different stages of metastasis formation. **l, p, t,** Smoothed mKi67, Lgr5 and EpiHR gene signature expression trends fitted with Generalized Additive Models as a function of pseudotime in primary tumors (**l**), micro+small (**p**) and large metastases (**t**).

We developed a new mouse model of metastatic relapse that allowed us to investigate the contribution of HRCs to metastatic recurrence. In brief, we innovated classical needle-based orthotopic injections by relocating them to the tip of the caecum, which allowed complete surgical excision of singular invasive CRCs (Fig. 2a, Extended Data Fig. 3a-c and Supplementary Video). Dual GFP/Luciferase-labelled AKTP MTOs grew rapidly in the caecum of c57BL/6 mice, colonized the adjacent mucosa and generated invasive cancers (Extended Data Fig. 3a,b). Bioluminescence imaging (BLI) revealed that mice remained free of primary disease after surgical resection yet, over the following days, they relapsed in the form of liver metastases (Fig. 2b,c and Extended Data Fig. 3d-e). Occasionally, we also observed metastases in mesenteric lymph nodes, lungs, peritoneum and diaphragm (Extended Data Fig. 3f-h). Primary tumor resection shortly after implantation cured all mice, whereas surgery at later points resulted in increased proportions of mice developing metastatic recurrences (Fig. 2b). In experiments of early primary tumor resection (day 11-15), faint bioluminescence could be detected ex vivo in some livers immediately after surgery, implying the presence of residual disseminated tumor cells. Lightsheet 3D fluorescence imaging revealed 3 to 10-cell micrometastases at the time of resection (Fig. 2d). Based on these observations, we established 4-5 weeks post-implantation as the optimal timepoint to enable a complete surgical resection of the primary CRC. We also developed a CRC relapse model based on the implantation of AKP MTOs in the caecum. These triple mutant CRCs exhibited delayed kinetics of metastatic recurrence after surgical extirpation of the primary CRC (Extended Data Fig. 3i).

We next sought to profile tumor cells along the process of relapse. Isolation of residual tumor cells from large organs has been historically a major hurdle in cancer research^13^. We devised a tissue dissociation strategy that enriched for residual tumor cells from whole liver samples (Extended Data Fig. 3j). In brief, we discovered that during tissue preparation for FACS, the vast majority of luciferase+ tumor cells were retained in 100 μm filters after mild enzymatic digestions, whereas most parenchymal liver cells flowed through in these conditions (Extended Data Fig. 3k,l). By redigesting cells retained in the filter, we obtained a 400-fold GFP-Luciferase+ enriched metastatic tumor cell preparation (Extended Data Fig. 3m). This step allowed purification of residual tumor cells from individual livers exhibiting absent or very low *ex vivo* bioluminescence (Extended Data Fig. 3n). By means of this approach, we profiled by Smart-seq2 scRNAseq 900 GFP+ tumor cells derived from livers collected at different time points after implantation of MTOs as well as from their corresponding primary CRCs (Fig. 2e). We confirmed the presence of micrometastases, small metastases or macrometastases by bioluminescence measurements in the resected livers (Extended Data Fig. 3n).

**Extended Data Fig. 4 |:**
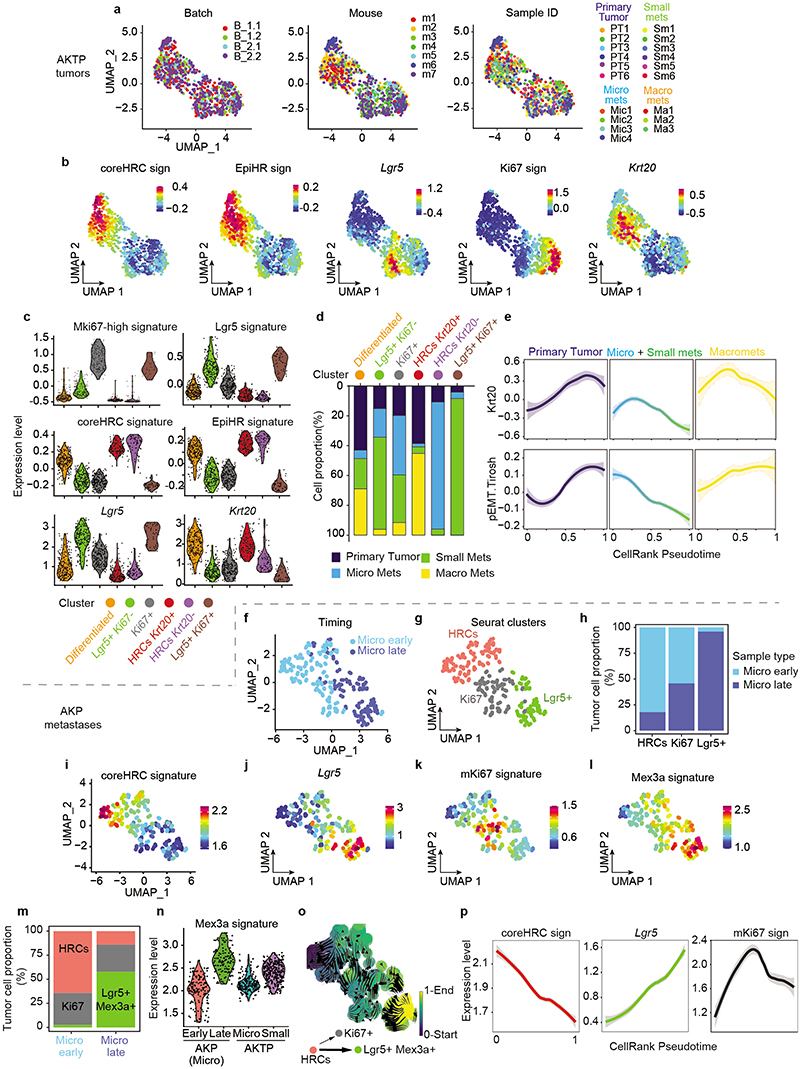
Additional description of residual AKTP and AKP metastatic cells. **a,** UMAPs of colorectal primary tumors and liver metastases at different stages (micro, small and large) colored according to sequencing batch, mouse ID, and sample ID. **b,** UMAPs showing the expression levels of coreHRC, EpiHR, and mKi67 gene signatures and *Lgr5* and *Krt20* genes. **c,** Violin plots showing expression of relevant genes used to define the 6 different Seurat clusters. **d,** Fraction of cells (y axis) from each Seurat cluster (x axis) present in the different sample types: Primary Tumor, micro-, small- and macro- metastases according to the indicated color code. Note the “HRCs Krt20-” are mostly exclusive from micro metastases samples, whereas Lgr5+ cells are highly enriched in small metastases samples. **e,** Smoothed *Krt20* gene and partial EMT gene signature^17^ expression trends fitted with Generalized Additive Models as a function of pseudotime in primary tumors, micro+small and large metastases. **f,g,** UMAP of AKP liver micrometastases colored according to timing of profiling and Seurat clusters. **h,** Barplot showing proportion of different Seurat tumor cell types captured in AKP early vs late micrometastases. **i-l,** UMAPs showing the expression levels of the coreHRC, mKi67 and Mex3a gene signatures and *Lgr5* mRNA in AKP metastases. **m,** Barplot showing Seurat cluster distribution across AKP early and late micrometastases. **n**. Violin plots showing expression levels of the Mex3a signature^16^ in AKP early and late micrometastases versus AKTP micro and small metastases. **o,** Vector fields representing RNA velocity projected on UMAPs of AKP micrometastases. Colored by the pseudotime estimated for each cell with scVelo. **p,** Smoothed coreHRC, mKi67, and Lgr5 gene signature expression trends in the early and late AKP micrometastasis dataset fitted with Generalized Additive Models as a function of CellRank pseudotime.

UMAP representation showed that tumor cells from primary tumors and metastases overlapped to a large extent (Fig. 2e,f and Extended Data Fig. 4). Hierarchical clustering analysis identified six cell clusters (Fig. 2g and Extended Data Fig. 4b, c). Cluster 0 included cells that expressed elevated levels of proliferation and biosynthesis-encoding genes (Fig. 2g and Extended Data Fig. 4b, c). Lgr5+ ISC-like tumor cells occupied two different clusters depending on high (cluster 1) and low (cluster 2) expression levels of the Ki67 proliferative signature^14^ (Fig. 2g and Extended Data Fig. 4c). Tumor cells of cluster 3 upregulated the differentiation marker *Krt20*. Clusters 4 and 5 were largely enriched in HRCs. Some cells in cluster 4 expressed *Krt20* suggesting that HRCs can also undergo differentiation (Fig. 2g and Extended Data Fig. 4b, c). Quantification of cell types revealed a dynamic distribution of cell populations across the metastatic relapse process (Fig. 2h). Primary CRCs and macrometastases exhibited similar distribution of cell populations, including proliferative cells, Lgr5+ ISC-like cells, Krt20+ differentiated tumor cells and HRCs, most of which were also Krt20+ (Fig. 2h). In contrast, micrometastases were largely enriched in undifferentiated (Krt20-) HRCs and also contained abundant proliferative cells (Fig. 2h). Small metastases were mainly formed by Lgr5+ ISC-like cells in both Ki67+ and Ki67- states and contained fewer HRCs than micrometastases (Fig. 2h and Extended Data Fig 4d).

The computational trajectory inference algorithm CellRank^15^ predicted diverse hierarchical organizations during metastatic progression. In primary CRCs, proliferative Lgr5-neg tumor cells gave rise to Lgr5+ cells and HRCs (Fig. 2i-l). In macrometastases, the apex of the hierarchy was occupied by proliferative Lgr5+ ISC-like cells, which generated HRCs over time (Fig. 2q-t). In contrast, the algorithm prognosticated that the cells that initiate the cellular hierarchy of micrometastases corresponded to undifferentiated HRCs in cluster 5 (Fig. 2m-p and Extended Data Fig 4e). This cell population gave rise to Lgr5+ ISC-like and proliferative tumor cell progeny (Fig. 2m-p), which are abundant in small metastases (Fig. 2h and Extended Data Fig. 4d).

We made equivalent observations in AKP CRCs (Extended Data Fig. 4f-p). Abundant HRCs characterized liver AKP lesions profiled at early time points (day 35 post-implantation), whereas micrometastases collected later (day 70) contained a larger proportion of Lgr5+ cells (Extended Data Fig. 4f-k). Micrometastatic Lgr5+ cells in the AKP model expressed high levels of the signature of latent Mex3a+ cells (Extended Data Fig. 4l-n), which is in agreement with our previous study on the specification of this population in triple mutant CRCs^16^. CellRank also predicted HRCs as the origin of micrometastasis in the AKP CRCs (Extended Data Fig. 4o,p).

## Characterization of HRC features

**Extended Data Fig. 5 |:**
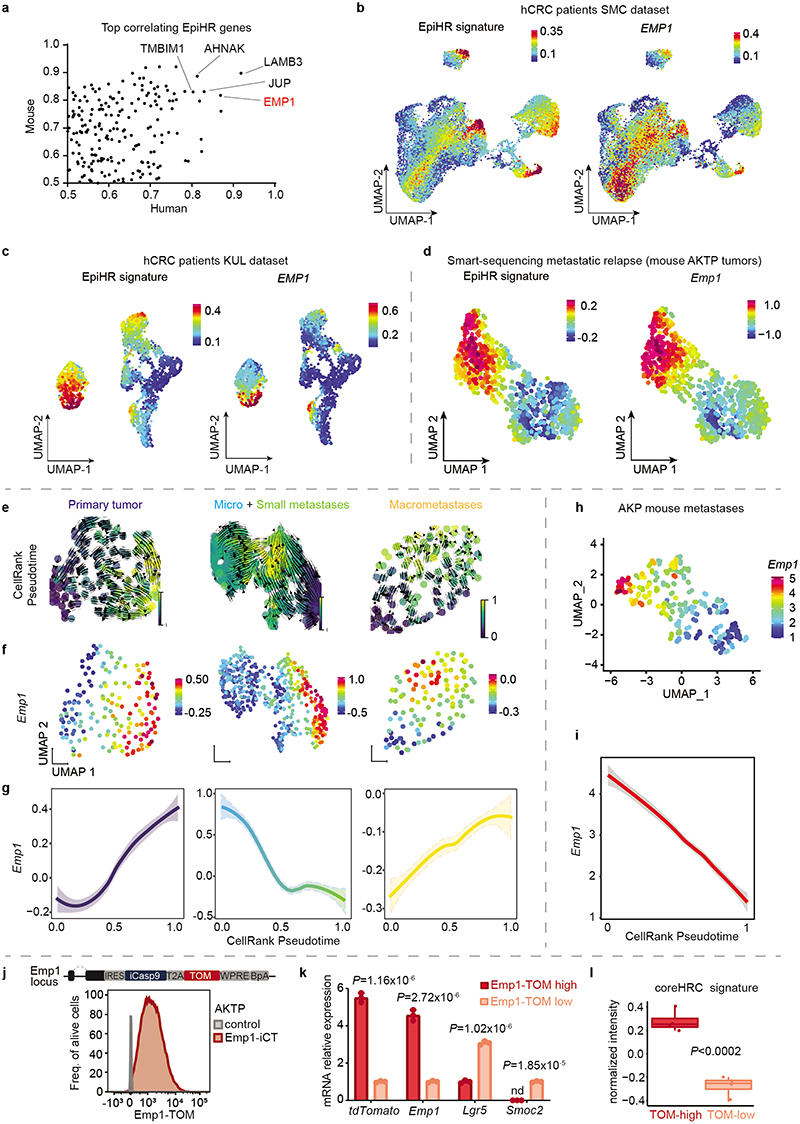
Epithelial membrane protein 1 (EMP1) marks HRCs. **a,** Scatter plot showing the correlation value between individual genes in the human SMC cohort (x axis) and in mouse primary tumors (y axis) with the EpiHR signature. Genes with correlation scores higher than 0.8 in both datasets are highlighted. **b,** UMAP of tumor cells from CRC patients in the SMC dataset colored according to the expression of EpiHR signature (left) and of *EMP1* gene (right). **c,** As in **b,** for CRC tumor cells from the KUL datasets. **d,** UMAP representation of Smart-sequencing single cell data of AKTP mouse tumor cells along metastatic relapse sequence colored by the EpiHR signature (left) and *Emp1* gene (right). **e,** Vector fields representing RNA velocity projected on AKTP primary CRC, micro+small and macro metastases UMAPs, colored by the pseudotime estimated for each cell with scVelo. **f,** AKTP tumor cell UMAPs colored by *Emp1* gene expression. **g,** Smoothed *Emp1* gene expression trends fitted with Generalized Additive Models as a function of pseudotime in AKTP primary tumor, micro+small and macro metastases samples. **h,** UMAP representation of AKP micrometastases colored by *Emp1* gene expression. **i,** Smoothed *Emp1* gene expression trends fitted with Generalized Additive Models as a function of pseudotime in AKP micrometastases samples. **j,** Representative flow cytometry plot of TOM expression in *wild-type* and Emp1-iCT AKTP MTOs. **k,** Relative mRNA expression in Emp1-TOM^high^ and Emp1- TOM^low^ sorted cell populations from Emp1-iCT AKTP MTOs *in vitro*. Two-sided t-test after normalizing by *Ppia*. n=3 technical replicates. Mean +/- SD. **l,** Boxplot showing normalized intensity of coreHRC signature expression in Emp1-TOM^high^ and Emp1-TOM^low^ cells dissociated from primary tumors 4 weeks post-implantation. Box plots have whiskers of maximum 1.5 times the interquartile range; boxes represent first, second (median) and third quartiles. n=4 mice per condition. ROAST-GSA adjusted p-values are shown.

**Fig. 3 |:**
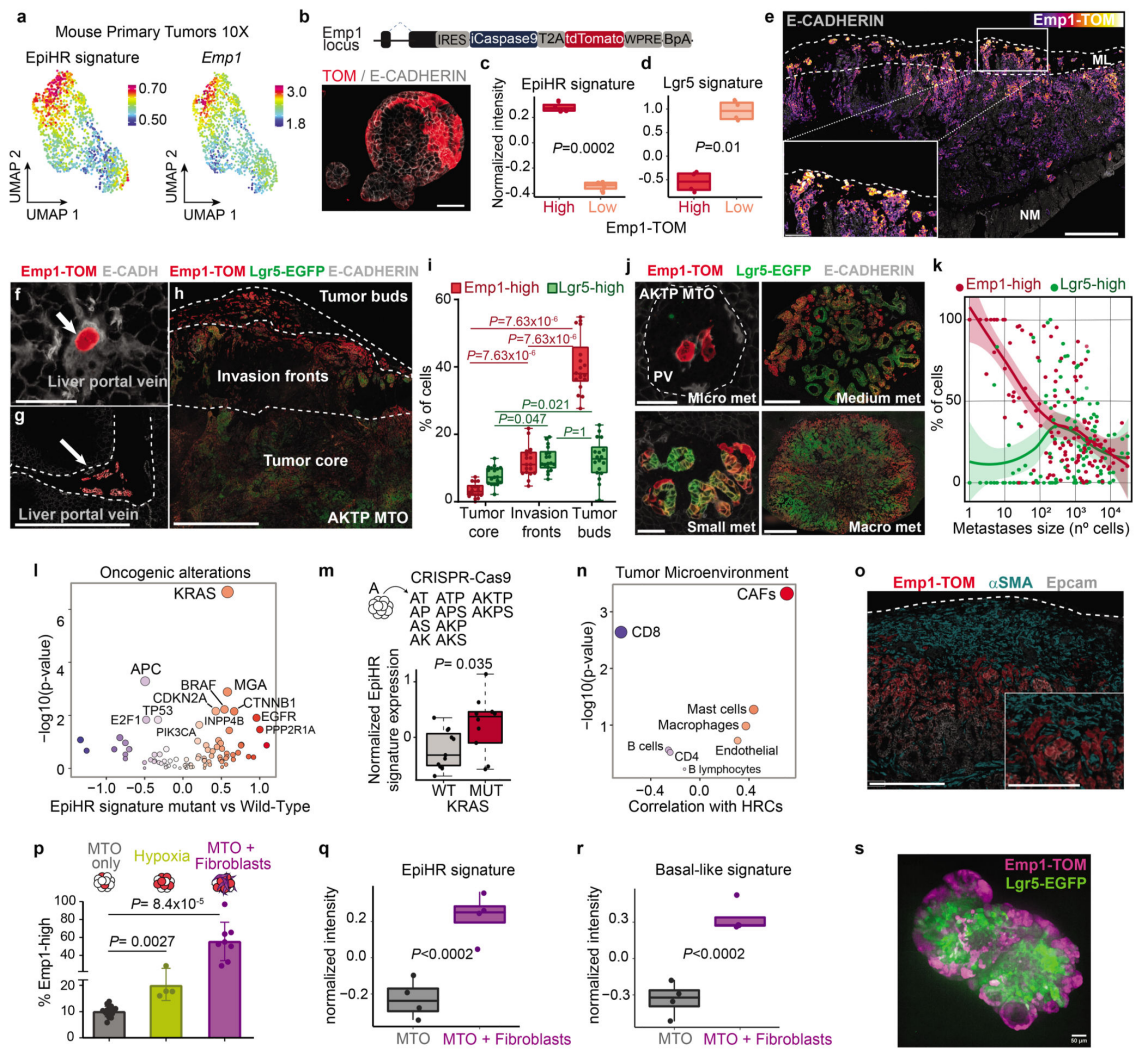
Emp1 marks cells enriched in invasion fronts and micrometastases. **a,** UMAP depicting EpiHR and *Emp1* levels. **b,** TOM and E-CADH immunostaining in Emp1-iCasp9-tdTomato AKTP MTOs. Scale bar, 50 μm. **c-d,** Expression (normalized intensity) of EpiHR (**c**) and Lgr5 (**d**) signatures in Emp1-high and Emp1-low cells 4 weeks post-implantation. n=4 mice per condition. ROAST-GSA adjusted p-values. **e-h,** Representative images of Emp1-TOM+ and/or Lgr5-GFP+ cells in primary CRCs **(e,h)** and liver metastases **(f,g)**. Dashed lines in panels **e,h** encompass tumor buds and invasion fronts or label the portal vein in **g**. NM: Normal Mucosa; ML: Muscle Layer. Scale bars, 500 μm (e,g, h); 100 μm (e, inset); 50 μm (f). **i,** Percentage of Lgr5-GFP^high^ and Emp1-TOM^high^ cells in different CRC regions. Whiskers are maximum and minimum values; n= 855,330 cells from 18 mice. Two-sided Paired Wilcoxon test. **j,** Examples of liver metastases of increasing size. Scale bars, 50 μm (micro and small), 250 μm (medium), 500 μm (macro). **k,** Percentage of Emp1-TOM^high^ and Lgr5-GFP^high^ cells per metastasis size. n= 318276 cells from 137 liver metastases from 17 different mice. LOESS model with a 95% confidence interval. **l,** EpiHR levels versus driver mutations. Difference of medians (x axis) versus p-value of Wilcoxon test (y axis) are shown. **m,** Normalized intensity of EpiHR signature expression in CTOs of different genotypes. n= 6 (WT) and 5 (Kras G12D) CTOs; 2 technical replicates per genotype. P-value for two-sided T-test. **n,** Association between HRC frequency and TME cell populations in SMC patients. Pearson correlation coefficients and p-values are shown. **o,** Representative patterns of TOM, α-SMA and EPCAM in primary CRCs. Dashed lines delimit the caecum. Scale bar: 250 μm; 125 μm (inset). **p,** Percentage of Emp1-TOM^high^ cells (mean ± SD) in the indicated conditions; n=17 (control), 4 (hypoxia) and 8 (fibroblast co-culture). Two-sided Wilcoxon test. **q-r,** Expression (normalized intensity) of EpiHR or basal-like signatures. n=4 biological replicates. ROAST-GSA adjusted p-values. **s,** Representative confocal images of AKTP MTOs co-cultured with colon fibroblasts. Scale bar, 50 μm. Boxes in panels c, d, i, m, q and r indicate first, second (median) and third quartiles. Whiskers in panels m,q and r indicate maximum of 1.5 times the interquartile range.

A comparison of human and mouse scRNAseq datasets revealed that a large subset of core genes were expressed consistently by HRCs of the two species (Extended Data Fig. 5a). Among them, we focused on EMP1 because it was expressed at high levels in HRCs and exhibited a large degree of overlap with the expression of the EpiHR geneset in both human (Extended Data Fig. 5b-c) and AKTP mouse CRCs (Fig. 3a and Extended Data Fig. 5d). Echoing our results with the EpiHR signature, CellRank predicted that the cell origin of metastatic relapse in AKTP (Extended Data Fig. 5e-g) and AKP (Extended Data Fig. 5h,i) tumors expressed elevated *Emp1* levels.

We thus leveraged *Emp1* to track HRCs during disease relapse. To this end, we knocked-in an inducible-Caspase9-tdTomato (iCT) cassette^2^ into the *Emp1* locus of AKTP MTOs using CRISPR-Cas9 (Fig. 3b). Inspection of knock-in MTOs revealed high tdTomato (TOM) expression in a subset of tumor cells (Fig. 3b). We next inoculated Emp1-iCT AKTP MTOs into the caecum of c57BL/6 mice. TOM expression in dissociated epithelial tumor cells measured by flow cytometry revealed heterogeneity in *Emp1* expression (Extended Data Fig. 5j). TOM-high cells purified by Fluorescence-activated single cell sorting (FACS) showed large upregulation of *Emp1* expression (Extended Data Fig. 5k). In contrast, TOM-low cells were characterized by expression of intestinal stem cell (ISC)-specific genes such as *Lgr5* and *Smoc2* (Extended Data Fig. 5k). Gene expression profiling confirmed elevated levels of the EpiHR and coreHRC signatures in Emp1-TOM-high cells, whereas the WNT/Lgr5+ ISC program was downregulated in these cells (Fig. 3c,d and Extended Data Fig. 5l).

**Extended Data Fig. 6 |:**
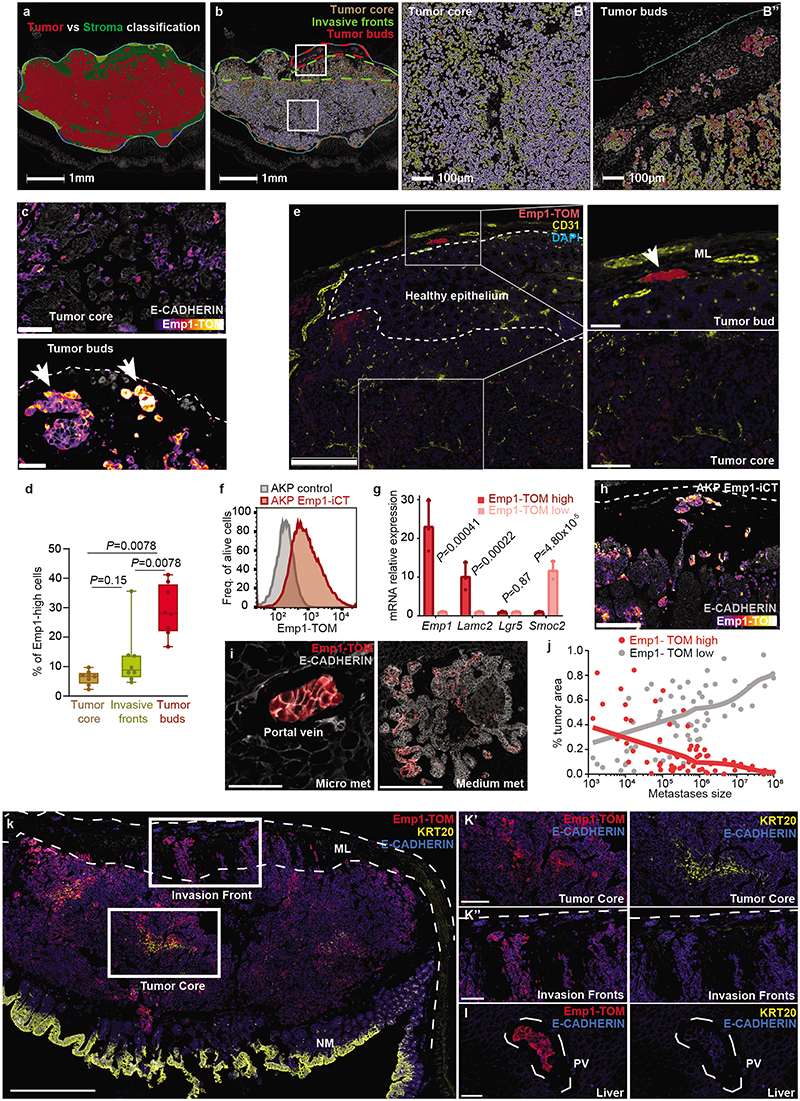
HRCs are enriched in invasion fronts and micrometastases. **a,** Primary tumor outlined by cyan line and colored in 4 different regions identified with HALO image analysis classifier (tumor-red, stroma-green, background-yellow, necrosis-blue). Scale bar, 1mm. **b,** TOM cell intensity analysis in the tumor area after segmentation into individual cells. B’ and B” show magnified regions corresponding to tumor core (B’) and invasion fronts + tumor buds (B”). Scale bars, 1mm (B), 100 μm (B’ and B”). **c,** Representative immunostaining of TOM and E-CADH in the tumor core and in tumor buds of primary tumors derived from Emp1-iCT MTOs 4 weeks post implantation in the caecum. TOM fluorescence is shown with mpl-inferno LUT. The dashed line delimits the caecum edge. Arrows point to tumor buds. Scale bars, 100 μm (tumor core) 50 μm (tumor buds). **d,** Quantification of Emp1-TOM^high^ (defined as cells in percentile 90 for TOM expression) in the tumor core (submucosal area), invasion fronts (inside muscular layer) and isolated glands (over muscular layer). Boxes represent the first, second (median) and third quartiles. Whiskers indicate maximum and minimum values. Two-sided Wilcoxon test on percentages. n= 8 mice. **e,** Immunofluorescence of TOM, CD31 and DAPI in primary tumors. Amplified insets show the tumor core and invasive glands intermingled in mucosal layers (ML) next to blood vessels. Dashed lines outline healthy intestinal epithelium. Scale bars, 250 μm, 100 μm (tumor core) and 50 μm (tumor buds). **f,** Representative flow cytometry plot of TOM expression in *wild-type* and Emp1-iCT AKP MTOs. **g,** Relative mRNA expression in Emp1-TOM^high^ and Emp1-TOM^low^ sorted cell populations from Emp1-iCT AKP MTOs. Two-sided t-test after normalizing by *Ppia*. n=3 technical replicates. Mean +/- SD. **h,** Representative immunostaining for TOM and E-CADHERIN in Emp1-iCT AKP tumors implanted in the caecum 4 weeks post-implantation. Emp1-TOM fluorescence is shown with an mpl-inferno LUT. Dashed lines delimit the edge of the caecum. Scale bar: 250 μm. **i,** Representative images of TOM and E-CADHERIN staining in micro (left) and medium (right) size metastases. Scale bars: 50 μm and 250 μm. **j,** Percentage of tumor area containing TOM-high and low fluorescent pixels versus metastases size (in pixels). Each dot represents an individual metastasis. **k,** TOM, KRT20 and E- CADHERIN staining in primary tumors generated by Emp1-iCasp9-tdTomato AKTP MTOs. Dashed lines encompass invasion fronts and tumor buds. KRT20 staining is observed in normal mucosa (NM) and to a lesser extent in the tumor core. Tumor cell clusters invading the muscular layer (ML) express high levels of TOM and no KRT20. Amplified insets show an example of tumor core (K’) and invasion fronts (K”) with TOM (left) and KRT20 (right) stainings. Scale bars, 500 μm (k) and 100 μm (K’ and K”). **l,** Immunofluorescence of TOM and E-CADHERIN (left) and KRT20 and E-CADHERIN (right) in a cluster of tumor cells that enter the liver through a portal vein (PV, delimited with dashed lines). Scale bar, 50 μm.

Inspection of tissue sections evidenced that cancer cells invading the muscular layer were strongly labeled by the fluorescent reporter (Fig. 3e). In particular, isolated tumor buds and larger clusters in contact with the stroma at the edges on invasion fronts exhibited the highest TOM expression (Inset in Fig. 3e, Extended Data Fig. 6a-d). Emp1-TOM^high^ cell clusters were often found in proximity to peripheral blood vessels in mouse primary CRCs, suggesting a connection with hematogenous or lymphatic dissemination (Extended Data Fig. 6e). CRCs generated by implantation of AKP MTOs in the caecum also exhibited Emp1-TOM^high^ invasion fronts and tumor buds that overexpressed HRC-marker genes (Extended Data Fig. 6f-h).

**Extended Data Fig. 7 |:**
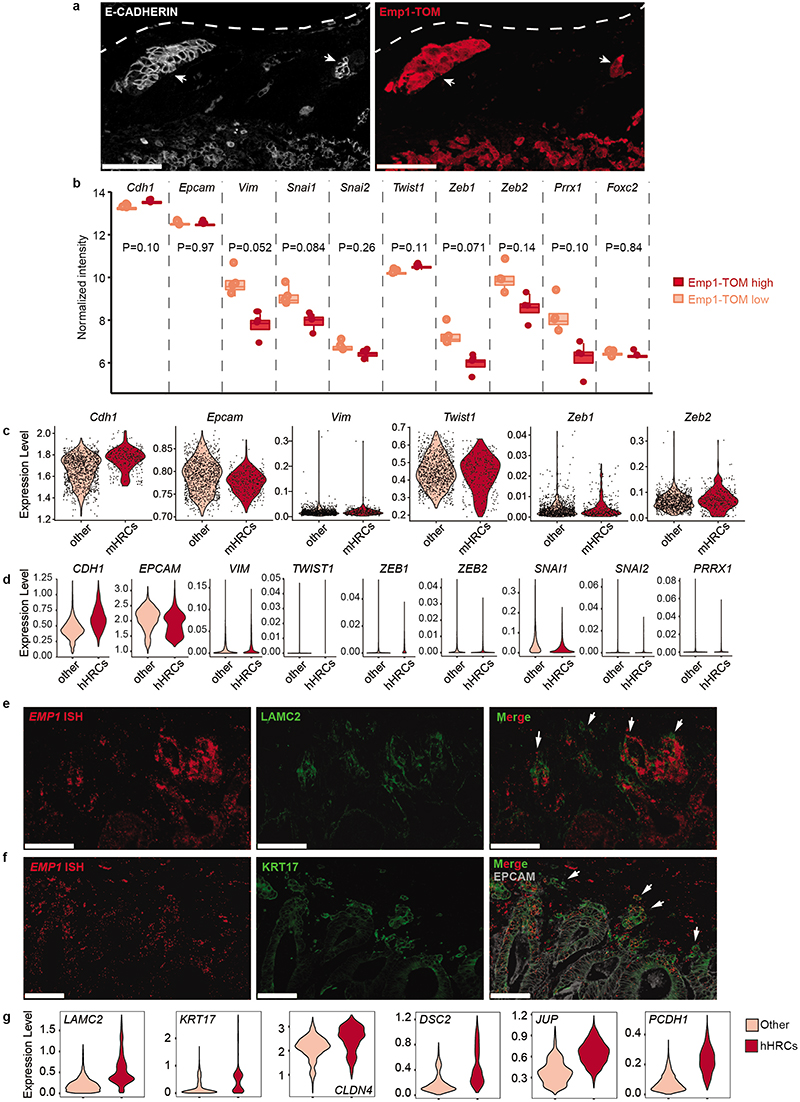
HRCs retain an epithelial phenotype. **a,** Immunostaining of E-CADHERIN and TOM in Emp1-iCT primary tumors 4 weeks post-implantation of MTOs. Arrows point at examples of E-CADHERIN+ invasion fronts and tumor buds. Dashed lines show the caecum edge. Scale bars, 100 μm. **b,** Boxplot showing normalized expression of genes related to EMT in Emp1-TOM^high^ versus Emp1- TOM^low^ cells. Box plots have whiskers of maximum 1.5 times the interquartile range; Boxes represent first, second (median) and third quartiles. P-value for differential expression with Linear Model for Microarray Analysis (limma). n=4 biological replicates. **c-d,** Violin plots showing expression of selected EMT-related genes in HRCs versus the rest of other cells in mouse epithelial primary tumor cells (**c**) and human tumor cells from the SMC cohort (**d**). Genes present in **b** not shown (Snai1 and Snai2) were undetected in (**c**). **e,** Representative example of *EMP1* mRNA FISH combined with LAMC2 immunofluorescence on human primary CRC tissue section showing an overlapping pattern of expression of *EMP1* and LAMC2 in invasion fronts and tumor buds (arrows). Scale bar, 100 μm. **f,** Representative example of *EMP1* mRNA FISH combined with KRT17 and EPCAM immunofluorescence on human primary CRC tissue sections showing an overlapping pattern of expression of *EMP1* and KRT17 in invading fronts and tumor cell clusters (arrows). Scale bars, 100 μm. **g,** Violin plots showing enrichment of *LAMC2, KRT17* and several cell-to-cell adhesion genes in HRCs (SMC cohort).

We also examined livers of mice bearing AKTP tumors at various time points post-orthotopic MTO implantation. In samples collected at early time points, micrometastatic lesions trapped within portal veins and liver sinusoids were populated entirely by Emp1-TOM^high^ cells (Fig. 3f-g and Extended Data Fig. 6i-j). Fitting the scRNAseq analyses, we identified two Emp1-TOM+ subsets; one expressed KRT20 and was located mainly in the tumor cores, whereas the other was positioned at invasion fronts, lacked KRT20 and expressed the highest levels of TOM reporter (Extended Data Fig. 6k). Liver Emp1-TOM^high^ micrometastases were also KRT20 negative (Extended Data Fig. 6l). Of note, Emp1-TOM^high^ tumor buds and micrometastases were labeled with EPCAM and E-CADHERIN, implying that they retained an epithelial organization (Extended Data Fig. 6 and Extended Data Fig. 7a). Analysis of epithelial-to-mesenchymal transition (EMT) master transcription factors showed equivalent expression levels in Emp1-TOM^high^ and Emp1-TOM^low^ cells (Extended Data Fig. 7b). scRNAseq also supported the lack of canonical EMT markers in human and mouse HRCs (Extended Data Fig. 7c-d). However, we noticed that the coreHRC signature genes *LAMA3, LAMC2, ITGA2* and *PLAUR* belong to a recently characterized partial EMT gene expression module^17^. HRCs in mouse primary CRCs and micrometastases upregulated this partial EMT signature (Extended Data Fig. 4e). Of note, LAMC2 has been previously identified as a specific marker for tumor budding in multiple tumor types, including CRC^18^, and we confirmed that its expression correlated with *EMP1* mRNA in CRC patient samples (Extended Data Fig. 7e,g). KRT17, a widely used marker of the basal pancreatic cancer subtype^19^, also marked EMP1^high^ invasion fronts and tumor buds (Extended Data Fig. 7f,g). Gene Set Enrichment Analysis (GSEA) also revealed homophilic cell adhesion and apical junctions as central features of HRCs (Fig. 1l). Fittingly, *EMP1* encodes a component of the tight junctions^20,21^ and there were multiple other constituents of adherent junction, tight junction and desmosome complexes upregulated in the core HRC program such as *PCDH1, DSC2, CLND4* and *JUP* (Extended Data Fig. 7g and Supplementary Table 2). The latter encodes Plakoglobin which mediates circulation of tumor cells as clusters and confers enhanced metastatic capacity to breast cancer cells ^22^.

**Extended Data Fig. 8 |:**
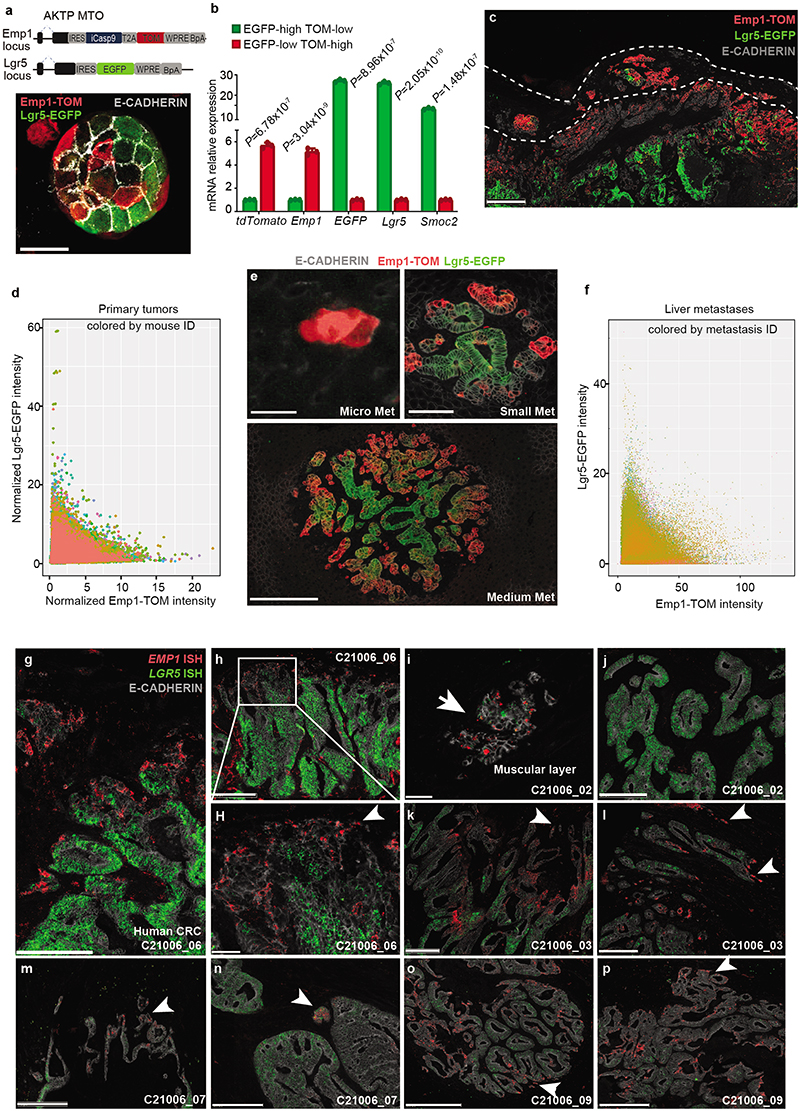
Emp1 and Lgr5 mark distinct tumor cell populations. **a,** Emp1-iCasp9-tdTomato and Lgr5-EGFP alleles introduced in AKTP MTOs. Confocal imaging of TOM, EGFP and E-CADHERIN immunostaining in edited MTOs. Single z-plane. Scale bar, 10 μm. **b,** Relative mRNA expression in EGFP-^high^/TOM-^low^ and EGFP-low/TOM-high sorted cells dissociated from subcutaneous AKTP Emp1-iCT Lgr5-EGFP tumors. Two-sided t-test after normalizing by *PPIA*. Mean +/- SD. n= 3 technical replicates. **c,** Immunostaining of TOM, EGFP and E-CADHERIN in Emp1-iCT Lgr5-EGFP primary tumors 4 weeks post-implantation of MTOs in the caecum. Dashed lines encompass tumor buds. Scale bar, 250 μm. **d,** Scatter plot showing normalized Emp1-TOM intensity versus normalized Lgr5-EGFP intensity in 855,330 cells from 18 different primary tumors. Note the absence of double positive cells (TOM and EGFP high). **e,** Representative immunofluorescence staining of TOM, EGFP and E-CADHERIN in liver metastases of increasing size (micro, small, medium) generated from the mouse CRC relapse model. Scale bars, 25 μm (micro) 100 μm (small) 250 μm (medium). **f,** Scatter plot showing TOM intensity versus EGFP intensity in 318,276 cells from 137 different liver metastases. Note the absence of double positive cells (TOM and EGFP high). **g-p,** Examples of dual *EMP1 and LGR5* mRNA ISH combined with E-CADHERIN immunofluorescence on human primary CRC tissue sections demonstrating a mutually exclusive pattern of expression of *EMP1* and *LGR5*. Note that *EMP1* expression is elevated in invasion fronts and tumor cell buds (white arrows). Scale bars, 500 μm (l, p) 250 μm (g, h, i, j, m, n, o) 50 μm (H’, k).

To further explore the relationship between *Emp1* and *Lgr5* expression, we engineered AKTP MTOs bearing both Emp1-iCT and Lgr5-EGFP knock-in reporter cassettes (Extended Data Fig. 8a-f). Confocal imaging of dual labeled MTOs showed a mutually exclusive pattern of expression of the two reporters (Extended Data Fig. 8a) and RT-qPCR analysis confirmed upregulation of *Emp1* and *Lgr5* in sorted TOM+ and EGFP+ cells, respectively (Extended data Fig. 8b). Primary CRCs generated from inoculation of dual labeled MTOs in the caecum also exhibited a mutually exclusive expression pattern of Emp1-TOM and Lgr5-EGFP reporters (Fig. 3h and Extended Data Fig. 8c-d). Emp1-TOM^high^ cells were largely enriched at tumor buds which, in contrast, contained few Lgr5-EGFP+ cells (Fig. 3i). RNA fluorescence in situ hybridization (FISH) analysis on human CRC patient samples also showed that *EMP1* expression was elevated at tumor invasion fronts, whereas *LGR5* marked the tumor cores in most cases (examples in Extended Data Fig. 8g-p).

Finally, we analyzed the livers of mice bearing primary CRCs. Lgr5-EGFP fluorescence was absent in disseminated tumor cells (DTCs) and micrometastases, but was progressively gained during metastatic outgrowth, in a marked antithetic pattern to Emp1-TOM expression (Fig. 3j-k and Extended data Fig. 8e-f). Together with CellRank bioinformatic predictions, these observations suggest that HRCs are endowed with the ability to migrate and disseminate to foreign organs where they initiate metastatic outgrowth and subsequently give rise to non-HRC populations.

## Determinants of the HRC population

**Extended Data Fig. 9 |:**
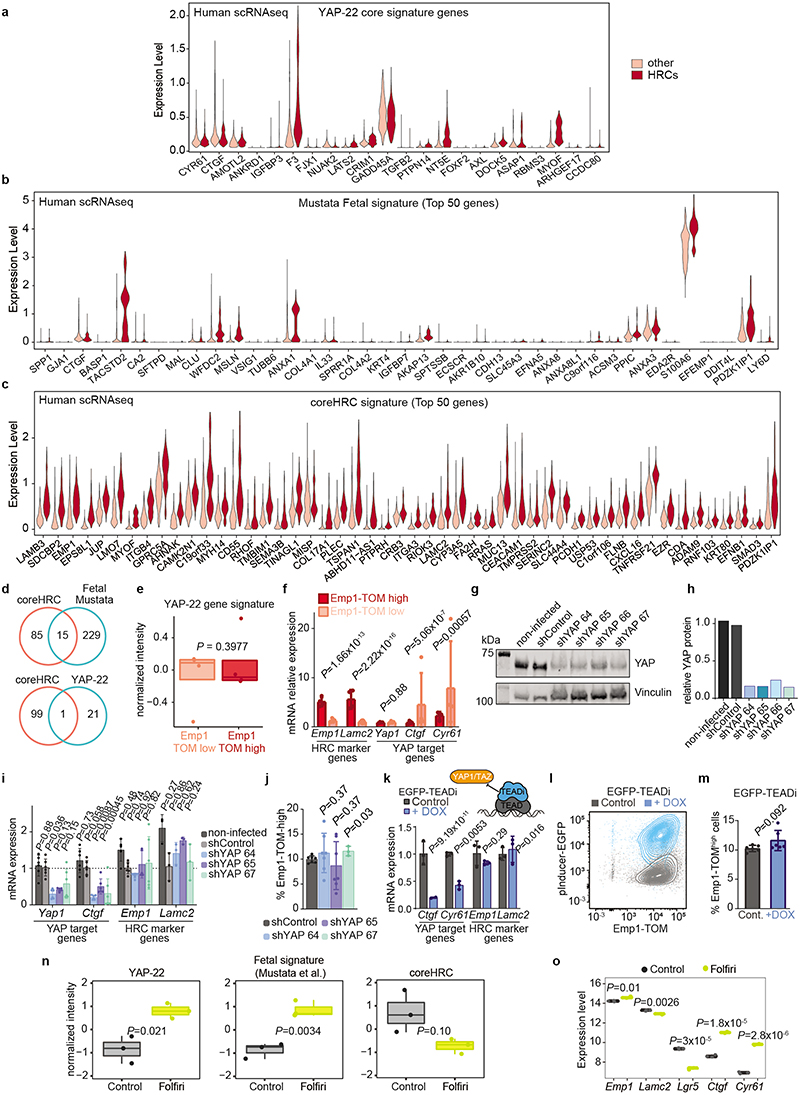
YAP/TAZ signaling is not required for HRC specification. **a-c,** Violin plots comparing the expression of genes belonging to the YAP-22 core signature^32^ **(a)**, the top 50 genes from the Fetal intestine progenitor signature^31^ **(b)** and the top 50 genes of the coreHRC signature **(c)** in HRCs vs other cells in the SMC scRNAseq cohort. **d**, Venn Diagram showing genes that overlap between the coreHRC signature and YAP-22 or Fetal intestine progenitor signatures. **e**. Boxplot showing normalized intensity of YAP-22 signature expression in Emp1-TOM^high^ and Emp1-TOM^low^ cells dissociated from primary tumors 4 weeks post-implantation. Box plots have whiskers of maximum 1.5 times the interquartile range; boxes represent first, second (median) and third quartiles. n=4 mice per condition. ROAST-GSA adjusted p-values are shown. **f**. Relative mRNA expression measured by RT-qPCR in Emp1-TOM^high^ and Emp1-TOM^low^ sorted cell populations from AKTP Emp1-iCT primary CRCs. Two-sided t-test after normalizing by *PPIA*. n=4 biological replicates. Mean +/- SD. **g,** Western blot for YAP and VINCULIN in non-infected, shControl and shYap infected AKTP organoids. **h,** Western blot quantification of YAP normalized by Vinculin. **i,** Relative mRNA expression (mean ± SD) in MTOs infected with shControl plasmid compared to uninfected MTOs and MTOs infected with three different shYAP plasmids. Analyzed with a mixed effects linear model after normalizing by *PPIA* housekeeping gene. n= 2 biological replicates with 3 technical replicates. **j,** Percentage (mean ± SD) of Emp1-TOM^high^ cells in organoids infected with shControl or shYAP plasmids. Two-sided Wilcoxon t-test. n= 3 (sh67) 7 (all other) measurements examined over 4 independent experiments. **k,** Relative mRNA expression (mean ± SD) in MTOs infected with pInducer GFP-TEADi plasmid treated or untreated with doxycycline (DOX). GFP+ cells were sorted in DOX treated organoids, whereas alive cells were sorted in untreated MTOs. n= 3 technical replicates. Analyzed with a mixed effects linear model after normalizing by *PPIA* housekeeping gene. **l,** Representative flow cytometry plot showing Emp1-TOM fluorescence versus pInducer GFP-TEADi fluorescence in TEADi MTOs untreated or treated with DOX. **m,** Quantification (mean ± SD) of Emp1-TOM^high^ in TEADi MTOs untreated or treated with DOX for 5 days. Two-sided Wilcoxon t-test. n= 2 biological replicates with 3 technical replicates. **n,** Boxplot showing expression levels (normalized intensity) of YAP-22, Fetal and coreHRC signature genes in control MTOs versus MTOs treated with chemotherapy (folfiri) for 4 days. Boxes represent the first, second (median) and third quartiles. Whiskers indicate maximum and minimum values. n=3 biological replicates per condition. Two-sided t-test. **o,** Boxplot showing the expression levels of relevant genes in control MTOs versus MTOs treated with chemotherapy (folfiri) for 4 days. Boxes represent the first, second (median) and third quartiles. Whiskers indicate maximum and minimum values. n=3 biological replicates per condition. Two-sided t-test.

The transcription factor YAP opposes the activity of the WNT pathway^23,24^ and a YAP-driven gene program has been associated with tumor cell plasticity^25,26^, regeneration^27^ and metastasis formation in CRC^28^. In addition, YAP promotes the conversion of LGR5+ CRCs cells to a fetal intestine-like progenitor state during chemotherapy^16,25,29,30^. Extended Data Fig. 9a,b shows the expression of the top 50 upregulated fetal intestine progenitor genes from Mustata et al.^31^, and the broadly used YAP_22 target gene signature^32^. As a reference, we also show the expression of the Top 50 HRC core genes (Extended Data Fig. 9c). Only 3 out of 22 YAP core signature genes were upregulated in HRCs of CRC patients, whereas most bonafide YAP target genes, including *CTGF* and *CYR61*, were not (Extended Data Fig. 9a). HRCs only upregulated a few canonical markers of the regenerative fetal-like intestinal population, including *ANXA1* or *TACSTD2*, but the majority of them were either not expressed or expressed at low levels by HRCs (Extended Data Fig. 9b). We also found little overlap between the coreHRC and YAP_22 or fetal intestinal progenitor signatures (Extended Data Fig. 9d). Emp1-TOM+ cells isolated from mouse primary CRCs were neither enriched in YAP target genes (Extended Data Fig. 9e-f). Therefore, there are only marginal similarities between YAP+/fetal intestinal progenitors and the HRC state. To obtain further insights into this question, we knocked down YAP levels using shRNAs in MTOs or blocked YAP-driven transcription by over-expressing an inducible dominant-negative TEAD transcription factor that inhibits both YAP and TAZ activity^33^ (Extended Data Fig. 9g-m). These genetic manipulations decreased the levels of the canonical YAP-target genes *Ctgf* and *Cyr61*, but did not affect the expression of core HRC genes *Emp1* or *Lamc2* in MTOs, neither the abundance of Emp1-Tom-high cells (Extended Data Fig. 9g-m). As a control for these experiments, we used chemotherapy (Folfiri), which upregulated the YAP_22 signature, including *Ctgf* and *Cyr61*, and the intestinal fetal progenitor program but did not induce the HRC state (Extended Data Fig. 9n,o).

**Extended Data Fig. 10 |:**
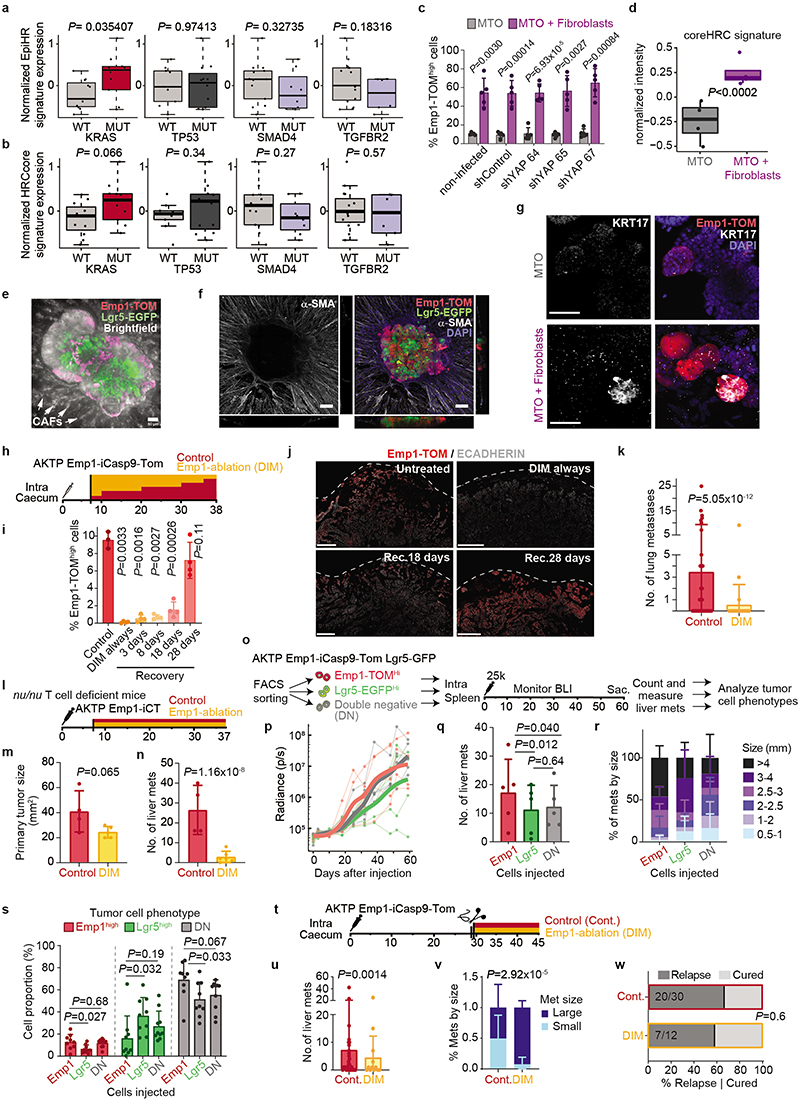
KRAS mutations and CAFs specify the HRC population. **a-b,** Normalized intensity of EpiHR and coreHRC signature expression in CTOs grouped by gain of function mutation in Kras g12d and loss of function mutations in *p53, Smad4 and Tgfbr2*. Box plots have whiskers of maximum and minimum values; boxes represent first, second (median) and third quartiles. n= 6 (WT) 5 (MUT) CTOs; 2 technical replicates. P-values for two-sided T-tests. **c,** Percentage of Emp1^high^ tumor cells (defined as the top 10% of the TOM population in control MTOs, mean ± SD) in parental (non-infected), control shRNA or shRNAs targeting YAP1. n=5 biological replicates. P-value for two-sided t-test. **d,** Normalized intensity of the coreHRC signature expression in control MTOs versus MTOs co-cultured with colon fibroblasts. Box plots have whiskers of maximum 1.5 times the interquartile range; boxes represent first, second (median) and third quartiles. n= 4 biological replicates. ROAST-GSA adjusted p-value is shown. **e,** Representative images of MTOs Emp1-iCT Lgr5-EGFP co-cultured with colon fibroblasts. Maximum intensity projection of confocal stacks, step 4 μm, z stack 120 μm. Scale bar, 50 μm. **f,** Immunostaining of α-SMA Emp1-iCT Lgr5-EGFP MTOs co-cultured with colon fibroblasts for 2 days. Scale bars, 100 μm. **g,** Immunostaining of KRT17 in 4-days grown MTO Emp1-iCT organoids: fibroblast co-cultures and organoids alone control cultures. Scale bars, 50 μm. **h,** Ablation by dimerizer (DIM) treatment and surgery schedule of mice with AKTP Emp1-iCT primary tumors to assess the recovery of HRCs upon treatment cessation. **i,** Percentage (mean ± SD) of Emp1-TOM^high^ cells (defined as top 10% in control animals) in untreated mice versus mice treated with DIM, with treatment discontinued at various timepoints post-injection. Two-sided T-test. n= 3 (control) 4 (rest) mice. **j,** Representative immunostainings showing effective Emp1-TOM^high^ cell ablation in DIM-treated primary tumors and recovery upon treatment cessation. Dashed lines delimitate the caecum edge. Scale bars, 250 μm. **k,** Lung metastases (mean ± SD) generated by MTO Emp1-iCT up to one month after primary tumor resection, treated with vehicle or DIM as in Fig. 4a. Each dot is a mouse; n= 34 (control) 29 (DIM). P-value for generalized linear model with negative binomial family. **l,** Inducible ablation schedule of nude mice *(nu/nu)* implanted with AKTP Emp1-iCT primary tumors. Resection was not possible due to local spreading of tumors to neighboring tissue. **m,** Primary tumor area (mean ± SD) measured at sacrifice. Each dot is a mouse; n= 4 (control), 5 (DIM) mice. P-value for linear model. **n,** Liver metastases (mean ± SD) generated by MTO Emp1-iCT. Each dot is a mouse; n= 4 (control), 6 (DIM) mice. P-value for generalized linear model. **o,** Schematics of an experiment to analyze the potential of Emp1+, Lgr5+ or double negative (DN) cells to colonize the liver and generate metastases. 25,000 FACS-sorted Emp1-TOM-high, Lgr5-EGFP-high or double negative cells were injected intrasplenically. **p,** Metastatic growth measured by BLI. **q,** Liver metastases (mean ± SD) generated by Emp1-high, Lgr5-high or double negative cells. Each dot is a mouse; n= 5 mice. P-value for generalized linear model. **r,** Distribution of liver metastasis diameters (mean ± SD). n=5 mice per condition. **s,** Percentage (mean ± SD) of Emp1-TOM^high^, Lgr5-EGFP^high^ and double negative tumor cells in metastases generated by the injection of Emp1-TOM^high^, Lgr5-EGFP^high^ and double negative cells, n= 9 (Emp1 and Lgr5) and 10 (DN) mice. Two-sided t-test. **t,** Experimental setup showing inducible HRC ablation after surgery of primary AKPT CRCs. **u,** Liver metastases (mean ± SD) generated by MTO Emp1-iCT in mice treated with vehicle or DIM 1 day after primary tumor resection and until experimental endpoints. Each dot is a mouse; n=30 (control) 12 (DIM) mice P-value for generalized linear model. **v,** Percentage (mean ± SD) of small (diameter equal or smaller than 1 mm^2^) and big metastases (bigger than 1 mm^2^) in mice treated with vehicle or DIM 1 day after primary tumor resection and until experimental endpoints. Mixed effects linear model after boxcox transformation with mouse as random effect, n= 20 (control) and 7 (DIM) mice. **w,** Percentage of mice that developed liver metastases in control and Emp1-ablated mice. Analyzed with a generalized linear model.

Association analyses in the TGCA COAD cohort revealed a strong correlation between activating KRAS mutations and EpiHR expression levels (Fig. 3l). Using a series of mouse organoids with compound mutations in main CRC driver genes engineered by means of CRISPR/Cas9 (CRISPR Tumor Organoids or CTOs), we confirmed that genotypes containing KRAS G12D mutations exhibited upregulation of coreHRC and EpiHR gene signatures (Fig. 3m and Extended Data Fig. 10a,b). We next searched for associations between HRCs and TME composition using the scRNAseq CRC patient dataset. This analysis exposed a direct correlation between CAFs and HRCs abundance (Fig. 3n). Consistent with this finding, α-SMA+ CAFs surrounded Emp1-TOM^high^ cells in primary AKTP CRCs (Fig. 3o). Co-culture of AKTP MTOs with CAFs augmented 6-fold Emp1-TOM+ cell numbers (Fig. 3p), but YAP knockdown failed to block this effect (Extended Data Fig. 10c). Expression profiling of MTO cells isolated from co-cultures demonstrated upregulation of EpiHR, coreHRC and basal pancreatic cancer signature levels (Fig. 3q-r and Extended Data Fig. 10d). Furthermore, it was evident an arrangement of tumor cell types reminiscent of the *in vivo* organization; Lgr5-EGFP cells were positioned at the organoid center, whereas Emp1-TOM+ cells relocated to the boundaries in contact with the fibroblast population (Fig. 3s and Extended Data Fig. 10e). Fibroblasts surrounding MTOs appeared activated, as shown by the expression of α-SMA (Extended Data Fig. 10f). We also noticed that some organoids gained expression of the pancreatic cancer basal cell marker KRT17 (Extended Data Fig. 10g).

## Relapse by residual EMP1+ cells

**Fig. 4 |:**
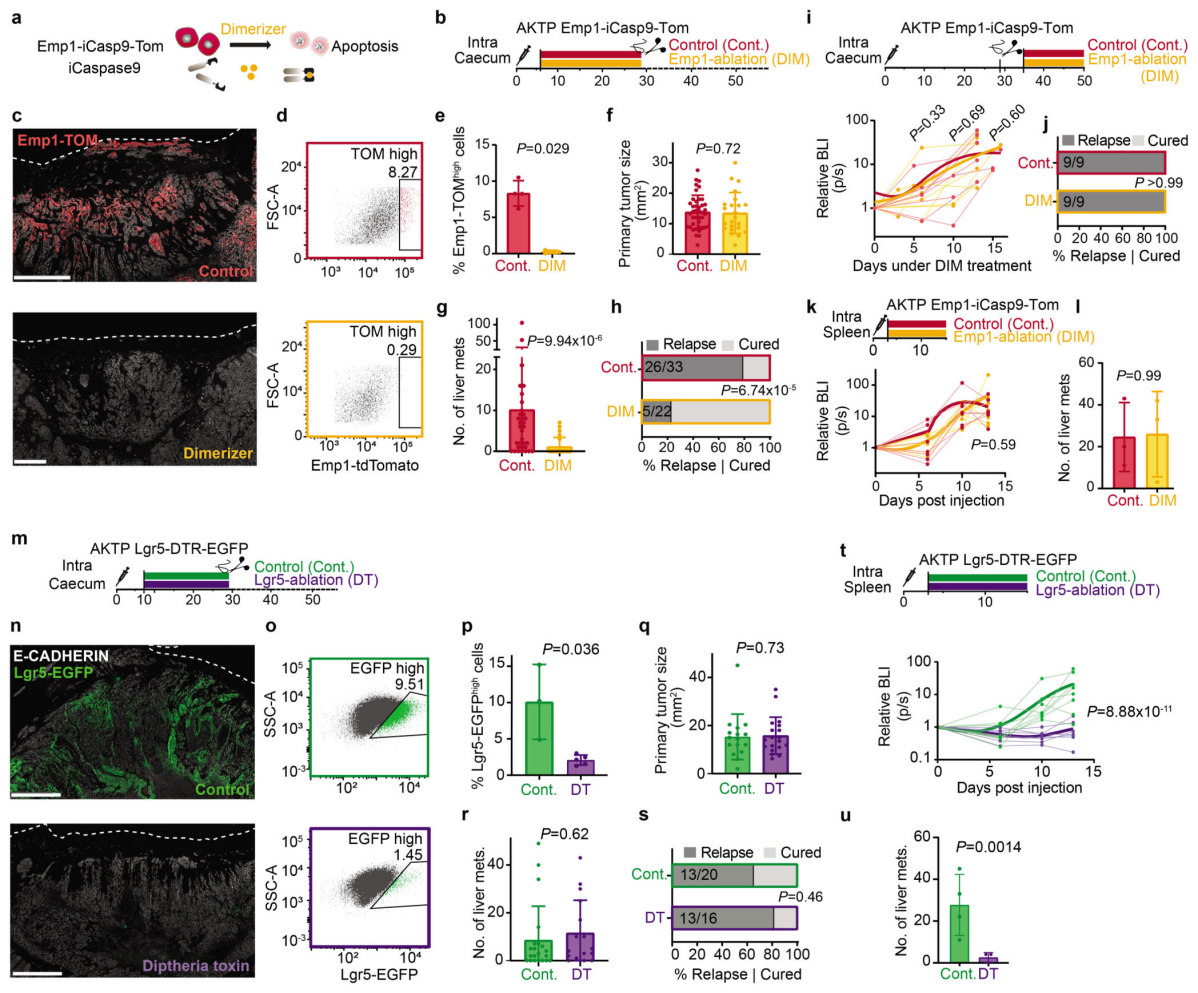
Emp1-high cells are the origin of metastatic relapse. **a,** Emp1-iCaspase9-mediated cell ablation. **b,** Experimental timeline detailing inducible Emp1+cell ablation and surgery. **c,** Emp1-TOM+ cells in primary tumors after ablation. Lines mark the caecum borders. Scale bars, 500 μm (C), 250 μm (C’). **d,** Representative flow cytometry plot of TOM fluorescence in EPCAM+ cells. **e,** Percentage of Emp1-TOM^high^ tumor cells (mean ± SD) at the time of surgery. Every dot is a mouse, n=4 in both groups. Two-sided Wilcoxon test. **f,** Primary tumor area (mean ± SD) after resection. Each dot is a mouse, n= 33 (control) and 22 (DIM) mice. P-value for linear model after boxcox transformation. **g,** Liver metastases (mean ± SD) at experimental endpoints. Each dot is a mouse, n as in panel **f**. **h,** Percentage of mice that relapse with liver metastases. Two-sided fisher test. **i,** Experimental timeline detailing late ablation of Emp1+ cells and metastatic growth by BLI monitoring. n=9 mice per each group. **j,** Percentage of mice that relapse with liver metastases. Two-sided fisher test. **k,** Metastatic growth by BLI monitoring upon ablation of Emp1 cells 3 days after intrasplenic inoculation of Emp1-iCasp9-Tom organoids, n= 3 mice per each group. **l,** Number of liver metastases in **k**. Mean ± SD. n= 3 mice per group. **m,** Experimental timeline. **n,** Representative stainings of Lgr5-DTR-EGFP+ cells in primary CRCs. Dashed lines outline the serosa. Scale bars, 100 μm. **o,** Representative flow cytometry plot of Lgr5-EGFP fluorescence. **p,** Percentage of Lgr5-GFP^high^ tumor cells (mean ± SD). n=3 (Cont.) and 5 (DT) mice respectively. P-value for generalized linear model**. q,** Primary tumor area (mean ± SD) measured after resection. n=20 mice in control and 16 in DT. Two-sided Wilcoxon test. **r,** Liver metastases (mean ± SD) at experimental endpoints. Each dot is a mouse, n as in (**r**). **s,** Percentage of mice that relapse with liver metastases. Two-sided fisher test. **t,** Liver metastasis growth monitored by BLI after intrasplenic inoculation of MTOs. **u,** Number of liver metastases (Mean ± SD) in (**t)**. n=7 mice in control and n=8 in DT treated in (**t,u)**. Points and lines of BLI measurements in panels **i, k** and **t,** represent individual mice, trend lines (bold) show a LOESS model and P-values were calculated with mixed effects linear model. P-values comparing in panels **g, r, l** and **u** were calculated using generalized linear model.

We next leveraged the inducible Caspase9 cassette inserted in the *Emp1* locus to perform cell ablation experiments in intact tumors (Fig. 4a,b)^2,34^. Inoculation of mice with AP20187 dimerized the chimeric Caspase9 expressed under the *Emp1* locus and specifically killed cells expressing the highest levels of Emp1-TOM reporter (Fig. 4c-e). This effect was reversible, shown by a slow but progressive recovery of the Emp1-TOM-high cell population at invasion fronts upon AP20187 dimerizer (DIM) treatment cessation (Extended Data Fig. 10h-j). DIM treatment was only administered during primary tumor growth but was ceased the day before primary CRC resection (Fig. 4b). Macrometastases were not yet present when DIM treatment finished; therefore, this experimental setting aimed at ablating Emp1^high^ cells in the primary tumor and possibly in incipient metastatic lesions. Remarkably, while DIM treatment did not affect primary tumor growth (Fig. 4f), the majority of mice showed no signs of liver and lung metastatic recurrence and were disease-free at experimental endpoints (Fig. 4g,h and Extended Data Fig. 10k). We made equivalent observations in AKTP CRC growing in nude mice, thus ruling out that genetic cell ablation strategy impaired metastatic progression through activation of the adaptive immune system (Extended Data Fig. 10l-n). When Emp1^high^ cell ablation started one week after primary CRC resection, we observed no changes in metastatic progression and all mice suffered metastatic relapse (Fig. 4i,j). Emp1^high^ cell ablation neither halted metastasis formation when MTOs were directly inoculated in the liver through the spleen (Fig. 4k,l). Reinforcing this observation, we found that intrasplenic inoculation of Emp1^high^ cells isolated from MTOs generated more and larger liver metastases than either Lgr5^high^ cells or Emp1^low^/Lgr5^low^ cells but the difference was not substantial, suggesting that all these tumor cell populations hold metastasis initiating capacity in this assay (Extended Data Fig. 10o-r). Moreover, metastases produced by these three cell populations contained the other tumor cell types owing to extensive cell plasticity (Extended Data Fig. 10s). Overall, these data demonstrate that Emp1^high^ HRCs drive metastatic relapse after primary CRC resection, yet they are dispensable after metastatic seeding is completed. This model is further supported by the observation that HRC ablation just after surgery and during metastatic outgrowth decreased the number of small size metastasis very significantly, but did not modify the frequency of large metastases (Extended Data Fig. 10t-w).

**Extended Data Fig. 11 |:**
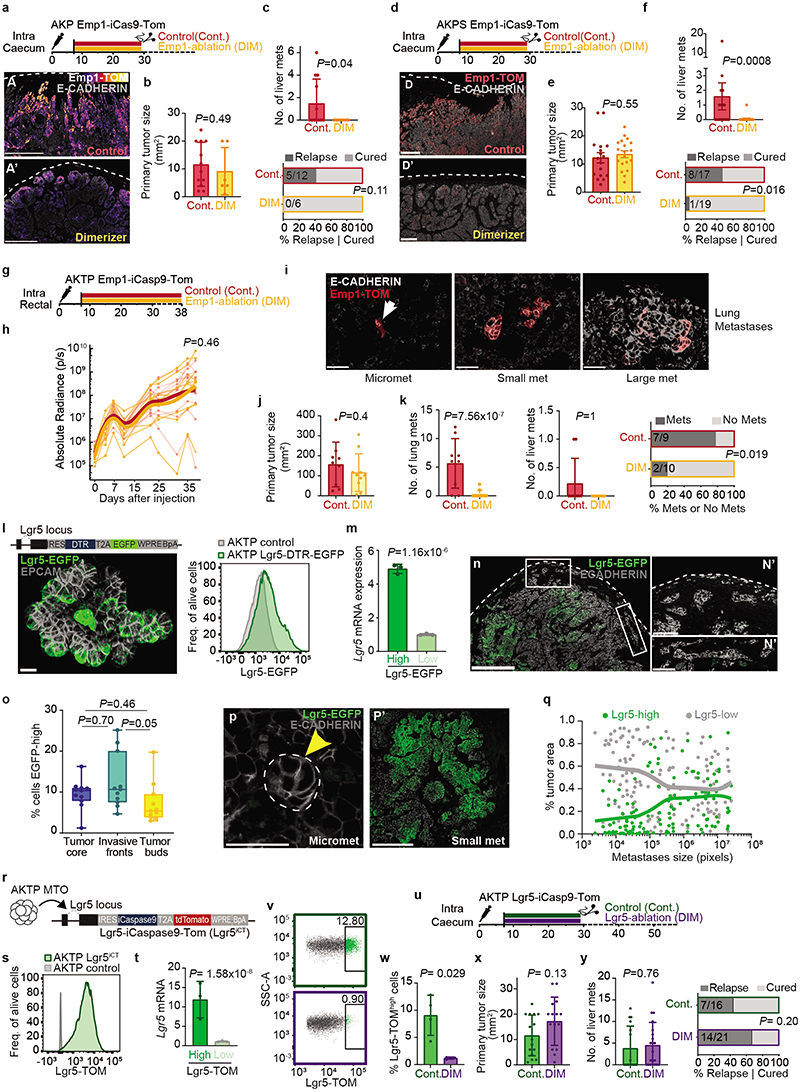
Metastatic relapse in different mouse CRC models arises from HRCs. **a,** Inducible ablation and surgery schedule of mice with AKP Emp1-iCT primary tumors. Panels A and A’ show immunostaining of TOM and E-CADHERIN demonstrating effective ablation of Emp1-high cells in primary CRCs. Dashed lines delimitate the caecum edge. Scale bars, 500 μm. **b,** Primary tumor area (mean ± SD) measured after resection. Each dot is a mouse, n= 12 (control) and 6 (DIM) mice. P-value for linear model after boxcox transformation. **c,** Liver metastases (mean ± SD) generated by MTO AKP Emp1-iCT up to one month after primary tumor resection. Each dot is a mouse, n= 12 (control) and 6 (DIM) mice. P-value for generalized linear model with negative binomial family. Bottom panel indicates the percentage of mice that developed liver metastases in the same experiment. Analyzed with a two-sided fisher test. **d,** Inducible ablation and surgery schedule of mice with AKPS Emp1-iCT primary tumors. Panels D and D’ show immunostaining of TOM demonstrating effective ablation of Emp1-TOM^high^ cells in DIM-treated primary tumors. Dashed lines delimitate the caecum edge. Scale bars, 250 μm. **e,** Primary tumor area (mean ± SD) measured after resection. Each dot is a mouse, n= 17 (control) and 19 (DIM) mice. P-value for linear model after boxcox transformation. **f,** Liver metastases (mean ± SD) generated by MTO AKPS Emp1-iCT up to one month after primary tumor resection. Each dot is a mouse, n as in panel **e**. P-value for generalized linear model with negative binomial family. Bottom panel indicate the percentage of mice that developed liver metastases in the same experiment. Analyzed with a two-sided fisher test. **g,** Inducible ablation schedule of mice implanted with AKTP Emp1-iCT MTOs in the rectum. **h,** Longitudinal intravital BLI quantification of AKTP MTOs implanted in the rectum. **i,** Representative TOM and E-CADHERIN immunostainings of lungs from mice bearing AKTP rectal tumors. Lung metastases of increasing size are shown. Note that TOM expression is higher in micrometastases and progressively reduced. Scale bars, 50 μm. **j,** Primary rectal tumor area (mean ± SD) measured at sacrifice. Each dot is a mouse, n= 9 (control) and 10 (DIM) mice. P-value for linear model after boxcox transformation. **k,** Lung (left panel) and liver (middle panel) metastases (mean ± SD) generated by MTO Emp1-iCT injected in the rectum. Each dot is a mouse, n as in panel **j**. P-value for generalized linear model with Poisson family. Right panel shows the percentage of mice that developed metastases in the same experiment. Analyzed with a two-sided fisher test. **l,** CRISPR-Cas9 targeting strategy to introduce an DTR-GFP cassette into the Lgr5 locus of MTOs. Confocal imaging of immunostaining for EGFP and EPCAM in Lgr5-DTR-EGFP organoids. Scale bar, 30μm. Right panel shows a representative flow cytometry plot of EGFP expression in wild-type and Lgr5-EGFP organoids. **m,** Relative *Lgr5* mRNA expression (mean ± SD) of Lgr5-EGFP^high^ versus -low cells isolated from Lgr5-DTR-EGFP subcutaneous tumors. n=3 biological replicates. Two-sided t-test normalizing to *B2M*. **n,** Immunofluorescence showing EGFP and E-CADHERIN in primary tumors. Insets (N’ and N”) correspond to invasion fronts and tumor buds lacking EGFP expression at higher magnification. Scale bars, 500 μm (D) and 100 μm (D’ and D”). **o,** Quantification of Lgr5-EGFP^high^ cells (defined as cells in percentile 90 for EGFP expression) in the tumor core, invasion fronts and tumor buds. Boxes represent the first, second (median) and third quartiles. Whiskers indicate maximum and minimum values. Paired two-sided Wilcoxon test on percentages. n= 11 mice. **p,** Representative images of Lgr5-EGFP staining in micro (P) and small (P’) metastases. Dashed lines and the yellow arrow surround a micrometastasis. Scale bars: (F) 50 μm; (F’) 250 μm. **q,** Percentage of tumor area containing Lgr5-EGFP^high^ and Lgr5-EGFP^low^ cells versus metastases size. Each dot represents an individual metastasis. **r,** CRISPR-Cas9 targeting strategy to introduce an iCaspase-9-TOM cassette into the LGR5 locus of AKTP MTOs. **s,** Representative flow cytometry plot of TOM expression in Lgr5-iCasp9-tdTomato organoids. **t,** Quantification of *Lgr5* mRNA (mean ± SD) by RT-qPCR in Lgr5-TOM^high^ and Lgr5-TOM^low^ cells dissociated from primary tumors grown for 4 weeks. n=3 primary tumors. Analyzed with a mixed effects linear model. **u,** Timing of inducible ablation and surgery in mice implanted with AKTP Lgr5-iCasp9-TOM primary tumors. **v,** Representative flow cytometry plot of Lgr5-TOM fluorescence in controls versus dimerizer-treated mice. DAPI-/EPCAM+ cells are shown. **w,** Percentage (mean ± SD) of Lgr5^high^ tumor cells (defined as the top 10% of the TOM+ population) in control and treated mice. n=4 mice each group. Two-sided Wilcoxon test. **x,** Primary tumor area measured after resection. n= 15 mice each group. Mean with SD, p-value of linear model after boxcox transformation. **y,** Liver metastases counted at experimental endpoints after primary tumor resection. n= 16 (control) and 21 (Lgr5-ablation) mice. Mean ± SD. Analyzed with a linear model with negative binomial family. Left panel show the percentage of mice that developed liver metastases in control and Lgr5-ablated tumors in the same experiment. Two-sided Fisher test.

Despite the slow-growing metastatic lesions generated by the AKP CRCs, HRC ablation before primary tumor surgery also prevented metastatic recurrence in these triple mutant models (Extended Data Fig. 11a-c). In addition, we demonstrated that HRCs mediated recurrence in CRCs bearing Smad4 mutations (AKPS) (Extended Data Fig. 11d-f). To extend our observations beyond the colon-to-liver metastasis axis, we implanted AKTP MTOs in the rectum, which generated invasive rectal cancers that, as in humans, metastasized preferentially to the lungs (Extended Data Fig. 11g-k). Inspection of lung metastases revealed that, akin to liver metastases, micrometastases were mostly Emp1^high^, whereas larger metastases decreased the percentage of Emp1^high^ cells (Extended Data Fig. 11i). Although we could not surgically resect mouse rectal tumors, ablation of Emp1^high^ cells caused a 20-fold reduction in lung metastasis burden in this model (Extended Data Fig. 11k).

Using a diphtheria toxin receptor (DTR)-based ablation strategy in CRC models, it was previously shown that Lgr5+ CRC cells are necessary for liver metastasis formation^3^. To assess the role of Lgr5+ tumor cells in our relapse models, we knocked-in a DTR cassette into the *Lgr5* locus of AKTP MTOs (Extended Data Fig. 11l-m). Inoculation of this MTO line into the caecum further validated our previous observations that invasion fronts, tumor buds and micrometastases seldom contain Lgr5+ cells (Extended Data Fig. 11n-q). More importantly, treatment with Diphtheria toxin (DT) before surgical removal of the primary CRC effectively eliminated Lgr5+ cells (Fig. 4m-p), yet it did not prevent disease relapse and mice developed overt liver metastatic disease (Fig. 4q-s). Thus, Lgr5+ cells are dispensable for dissemination and metastatic colonization. We validated these results using an independent AKTP MTO line engineered with an iCaspase9-tdTomato (iCT) cassette knocked-in in the *Lgr5* locus (Extended Data Fig. 11r-t). Again, effective ablation of Lgr5+ cells in the iCT model (Extended Data Fig. 11u-w) neither altered CRC tumor growth (Extended Data Fig. 11x) nor prevented metastatic recurrence (Extended Data Fig. 11y). On the other hand, ablation of Lgr5+ cells after direct inoculation of MTOs in the liver through the portal vein halted metastasis formation (Fig. 4t, u), further supporting a requirement for Lgr5+ cells during metastatic outgrowth^3^.

## Neoadjuvant immunotherapy avoids relapse

**Extended Data Fig. 12 |:**
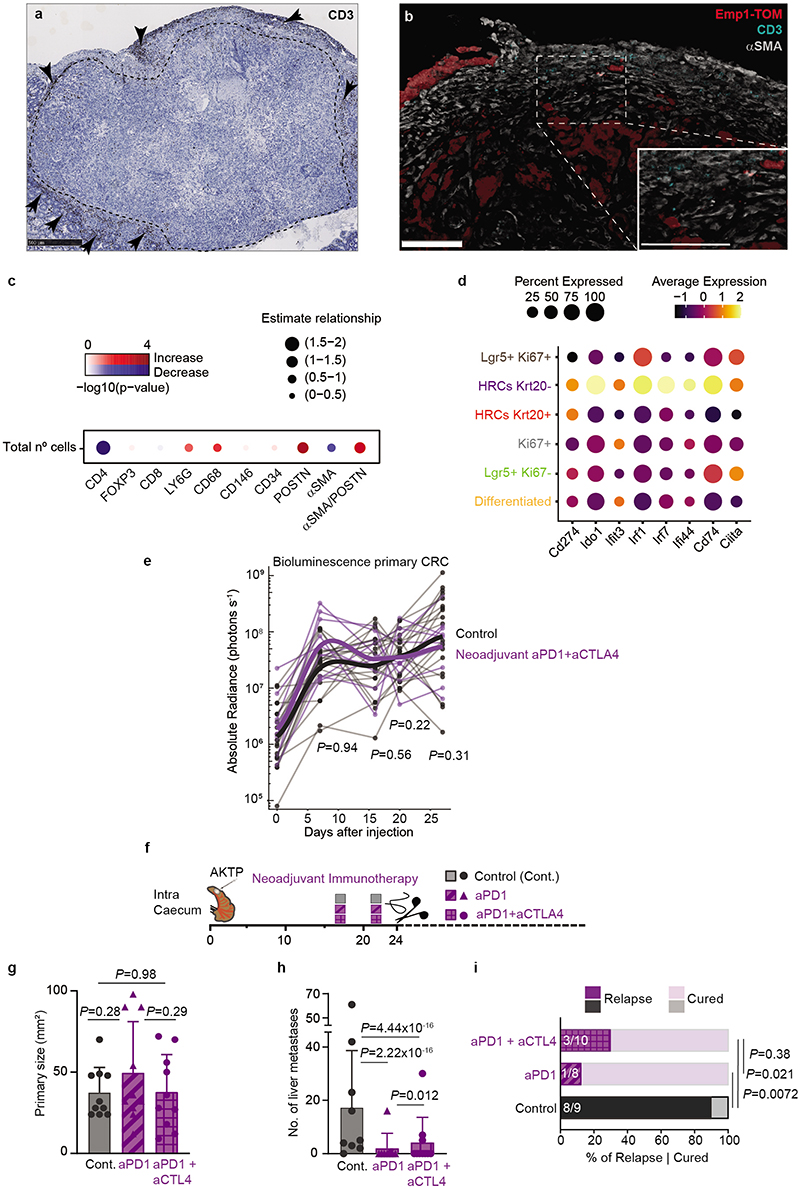
Immune checkpoint immunotherapies prevent metastatic relapse. **a**, Representative image of CD3+ cell distribution in primary AKTP CRC showing T cell exclusion. Arrows point to T cells located at the tumor periphery. **b,** Representative immunostaining of Emp1-TOM, CD3 and α-SMA in primary tumors. Scale bars, 100 μm. **c,** Dotplot summarizing regression models applied to multiplex immunofluorescence data. Effects of the total number of cells on the composition of every cell population are represented by different point sizes (defining the magnitude of the effect) and colors (showing both the sign of the effect in blue(-)/red(+) and the statistical significance by color intensity). **d,** Dotplot showing examples of interferon response genes across 6 tumor scRNAseq cell clusters as defined in Fig. 2g. **e,** Bioluminescence monitoring of the effect of the neoadjuvant immunotherapy regime used in Fig. 5k on primary tumor growth. Points and lines represent individual mice, trend lines (bold) show a LOESS model. n= 19 (control) 10 (PD1+CTLA4) mice. Mixed effects linear model with data normalized to time 0 and mouse as random effect. **f,** Schematics of an experiment comparing metastatic relapse in untreated mice and mice treated with neoadjuvant treatment with anti-PD1 monotherapy or anti-PD1+/anti-CTLA4 combined therapy. **g,** Primary tumor area (mean ± SD) measured after resection in the experiment described in **f**. Each dot is an individual mice. n= 10 mice each group. Linear model. **h**, Liver metastases (mean ± SD) generated by AKTP primary tumors up to one month after primary tumor resection in the experiment described in **f**. Each dot is an individual mice. n= 9 (control), 8 (PD1), 10 (PD1/CTLA4). Generalized linear model of Poisson family. **i**, Percentage of mice that developed liver metastases or remained metastases-free at experimental endpoints (4 weeks after resection) in control and immunotherapy-treated tumors. n= 9 (control), 8 (PD1), 10 (PD1/CTLA4). Generalized linear model with beta-binomial distribution.

**Fig. 5 |:**
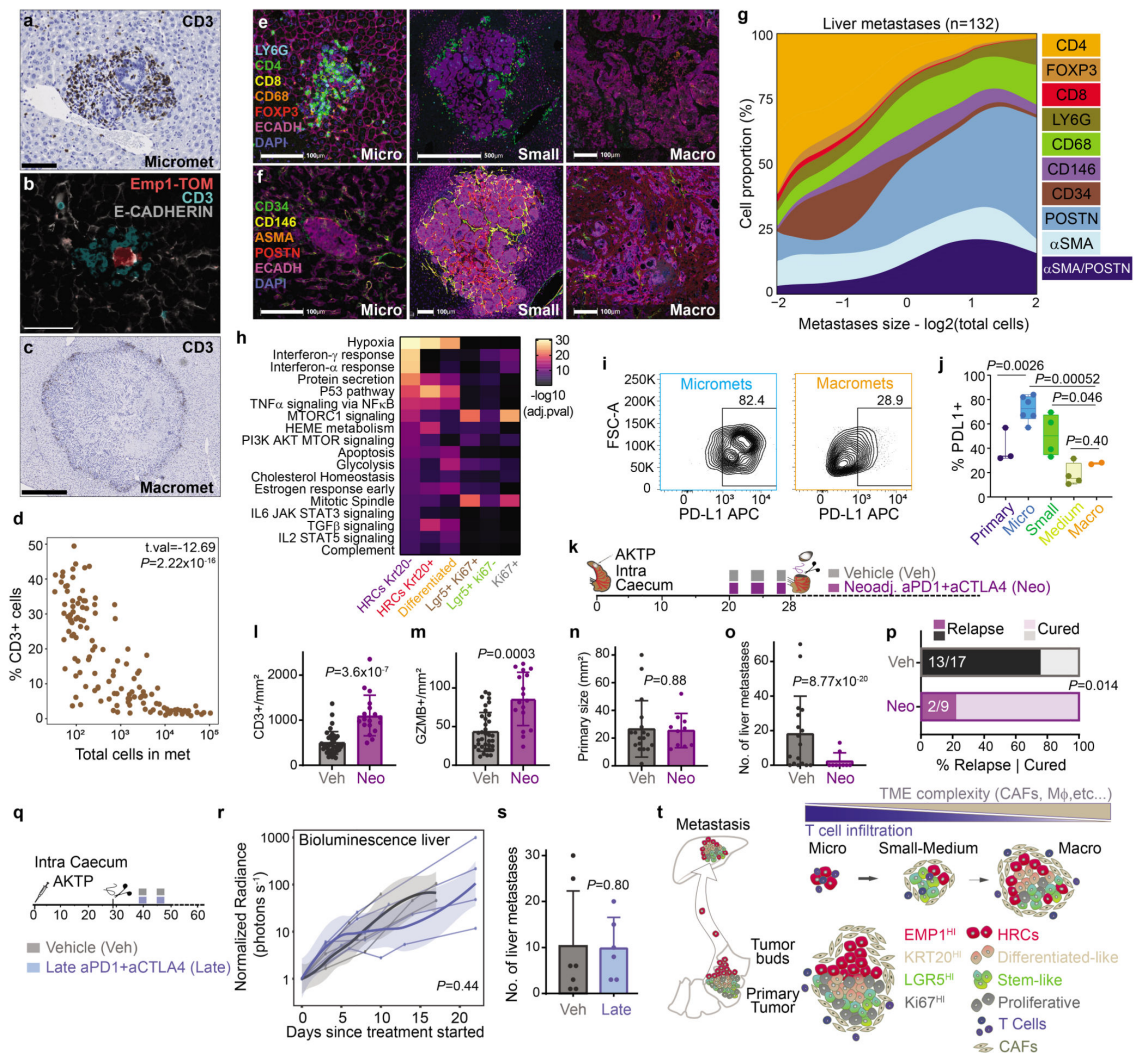
Neoadjuvant immunotherapy prevents metastatic relapse in CRC. **a-c**, Representative images of T cell distribution (CD3+) in liver micrometastasis (**a,b**) and macrometastases (**c**) present in the AKTP CRC relapse model. Scale bars, 100 μm (a), 50 μm (b) and 500 μm (c). **d**, Percentage of CD3+ T Cells (referred to total TME cells) versus metastases size. n=12 mice, 133 metastases. Linear model with mouse as fixed effect. **e-f,** Representative examples of multiplex immunofluorescence of immune (**e**) and stromal (**f**) markers in metastases. Scale bars, 100 μm (micro and macro), 500 μm (small). **g**, Proportional stacked area graph showing TME cell types in metastasis of different sizes; n= 132 metastasis of 20 mice. **h,** Heatmap showing Hallmark GSEA in cell populations described in Fig. 2g. **i,** Representative flow cytometry contour plot showing PD-L1 expression in tumor cells of micro- and macro-metastases. **j,** Percentage of PD-L1+ tumor cells by flow cytometry. Whiskers encompass the smallest and largest value. Boxes represent first, second (median) and third quartiles. n= 3 (primary), 6 (micro), 4 (small and medium), 2 (macro). Wilcoxon t-test. **k**, Experimental timeline for neoadjuvant immunotherapy treatment. **l-m,** CD3+ (**l**) and GZMB+ (**m**) cell densities in primary CRCs (means ± SD); n= 19 (control), 10 (neoadjuvant). Mixed effects linear model. **n**, Primary CRC area (mean ± SD) after resection. Each dot is a mouse; n= 18 (control), 10 (treated). P-value for linear model. **o**, Liver metastases (mean ± SD) at experimental endpoints. Each dot is a mouse; n= 17 (control), 9 (treated). P-value for generalized linear model. **p**, Percentage of mice that developed liver metastases at experimental endpoints; n as in **o**. P-value for generalized linear model. **q**, Experimental timeline for late immunotherapy. **r**, Liver metastasis growth measured by normalized BLI. Points and lines represent individual mice, trend lines show a LOESS model. n= 4 (control), 5 (late immunotherapy). Analyzed with a linear model. **s,** Liver metastases (mean ± SD) generated by AKTP primary tumors. Dots are an individual mouse. n= 7 (control), 6 (late immunotherapy). P-value for generalized linear model. **t,** Proposed model for metastatic dissemination of CRC and TME co-evolution.

We previously showed that mouse primary AKTP CRCs are models of microsatellite stable (MSS)/mismatch repair proficient CRCs characterized by a relatively low mutational burden, abundant stroma cells and T cell exclusion^12^. Transplantation of AKTP MTOs in caecum also gave rise to T-cell excluded CRCs (Extended Data Fig. 12a). CAFs surrounded *Emp1*-expressing invasion fronts and tumor buds, but T cells did not reach these structures and remained at the tumor periphery (Extended Data Fig. 12b). In contrast, liver micrometastases generated by these primary CRCs exhibited high CD3+ cell density (Fig. 5a). The lack of T cell exclusion was evident and we could visualize T cell-HRC interactions in these early lesions (Fig. 5b). However, metastatic outgrowth was accompanied by a gradual decline of CD3+ cells and the relocalization of T cells to the periphery (Fig. 5c,d). Multiplex immunohistochemistry showed that most infiltrating T cells were CD4+/FOXP3- (Examples in Fig. 5e and quantification in Fig. 5g and Extended Data Fig. 12c). T cell exclusion coincided with progressive CAFs (α-SMA+ and/or POSTN+) and Macrophage (CD68+) recruitment to the metastatic TME (Fig. 5f,g and Extended Data Fig. 12c).

scRNASeq revealed elevated expression of interferon-alpha and -gamma target genes in undifferentiated (Krt20-) HRCs (Fig. 5h and Supplementary Table 4), >95% of which belong to micrometastatic lesions, suggestive of an ongoing inflammatory response as HRCs reach the liver. Indeed, undifferentiated HRCs upregulate multiple interferon response genes compared to the other tumor cell populations in primary CRCs and large metastases (Extended Data Fig. 12d). It was also apparent that HRCs in micrometastases expressed elevated *Cd274* (that encodes PD-L1) and *Ido1* levels, two negative regulators of the immune response (Extended Data Fig. 12d). Flow cytometry confirmed elevated PD-L1 expression at the surface of micrometastatic CRC cells and a progressive decline over subsequent outgrowth (Fig. 5i,j). Based on these findings, we hypothesized that at the onset of organ colonization, lack of a mature TME exposes HRCs to the adaptive immune system, yet disseminated cells bypass immune attack through the expression of immune-modulatory molecules. To test the susceptibility of micrometastatic disease to immunotherapy, we treated mice with anti-PD1 in combination with anti-CTLA4 antibodies in the neoadjuvant setting, i.e., before the primary tumor was extirpated by surgery (Fig. 5k-p). This approach increased CD8+ cytotoxic T cell numbers in the primary CRC (Fig. 5l,m) but we did not observe alteration of the tumor growth rate (Extended Data Fig. 12e) or curative effects on the primary disease at experimental endpoints (Fig. 5n). Yet, remarkably, most of these mice did not develop metastasis after surgical removal of the primary CRC and remained disease-free at experimental endpoints (Fig. 5o,p). We obtained similar results with neoadjuvant anti-PD1 monotherapy (Extended Data Fig. 12f-i). In contrast, the same regime applied two weeks after surgery (i.e., late anti-PD1/CTLA4 immunotherapy) did not stop metastatic outgrowth (Fig. 5q-s), which is in line with the failure of checkpoint immunotherapy observed in patients with metastatic MSS CRC^35–37^. Hence, during a temporal window after metastatic colonization, immunotherapy is effective in eliminating the residual disease and preventing subsequent metastatic relapse.

## Discussion

HRCs represent a defined cell state in a large proportion of patient samples implying a common origin and mechanism of metastatic recurrence across CRC genotypes and molecular subtypes. Pioneering work by de Sauvage and colleagues revealed that Lgr5+ cancer stem cells are dispensable for primary CRC growth yet necessary for metastasis formation in experimental models^3^. Subsequently, Van Rheenen and colleagues proposed that metastases are initiated by disseminated differentiated tumor cells that, through plasticity, produce Lgr5+ cancer stem cells upon reaching the liver^38^. Massagué and colleagues also provided evidence that expression of the adhesion molecule L1CAM in LGR5+ and LGR5- cells is important for the regenerative burst that follows metastatic colonization^39^. We unequivocally show that HRCs are a subset of LGR5 negative cells yet they are neither differentiated nor stem-like but rather co-opt a distinct state that enables migration and colonization of foreign organs. KRAS mutations, cross-talk with stromal fibroblasts and other environmental stimuli such as hypoxia directly instruct HRCs at invasion fronts. The finding that HRCs are enriched in tumor buds strengthens the well-established association of these anatomic structures with poor prognosis^40,41^. In addition, a defining feature of the HRC state is the upregulation of genes encoding cell-to-cell adhesion molecules. We therefore speculate that HRCs may extravasate as cell clusters and colonize foreign organs as oligocellular structures rather than as single cells as previously shown for other cancer types^22,42^. Our data fit with a model whereby HRCs disseminate out of the primary tumor prior to surgical resection and the HRC state is subsequently retained in residual tumor cells lodged in foreign organs. Reacquisition of the Lgr5+ stem cell and proliferation programs occur at a later phase and is necessary for metastatic outgrowth (Fig. 5t). Ablation of HRCs in primary tumors prevents the vast majority of metastatic relapses. Yet, it is formally possible that other tumor cell populations, including Lgr5+ cells, may generate metastasis if they reach foreign organs, as suggested by experiments of direct inoculation of tumor cells into the blood stream performed herein and elsewhere^3,28,38,39^. Our data also indicate that YAP activity is not required for the specification of HRCs in primary CRCs, although do not rule out a role for YAP during the metastatic cascade as previously proposed^28^.

Immune-checkpoint therapy does not exert therapeutic benefits in MSS/mismatch repair-proficient overtly metastatic CRCs^35–37^. Besides the lower neoantigen burden of MSS CRCs, data in experimental models and patient samples indicate that the TME of MSS CRCs excludes and limits the activity of T-cells^12,43^. Our findings reveal that residual metastatic cells lodged in foreign organs lack a mature TME and are susceptible to the attack of the adaptive immune system upon immunotherapy treatment. This window of vulnerability could be exploited to prevent metastatic relapse. Our results back up current efforts to use neoadjuvant immunotherapy in early-stage CRC patients^44^. This and other therapeutic strategies capable of eliminating HRCs may prevent disease relapse if applied before metastatic disease is overt. The CRC relapse mouse model described herein may serve as a powerful pre-clinical platform for testing such therapies.

## Methods

### MTOs and integration of cassettes using CRISPR

We previously described^12^ the establishment of MTOs from primary tumors arising in GEMMs with compound genetic alterations (Apc, K-ras, Tp53, Tgfbr2). MTOs were cultured as detailed by Tauriello et al.^12^ and checked bimonthly for mycoplasma contamination. 20 bp small guide RNAs (sgRNA) were designed to cut 9-11 base pairs after the STOP codons using the http://crispr.mit.edu web tool and were cloned into pX330-IRFP hSp-enhanced-Cas9 plasmid^4^. The sgRNA sequence for Emp1 was “AAATAAGCCGAATACGCTCA” and for Lgr5 “GTCTCTAGTGACTATGAGAG”. 1kb 5’ and 3’ homology arms were sequentially cloned into pShuttle vectors containing the inducible Caspase9-tdTomato cassette^34^, a kind gift from Toshiro Sato. IRES-DTR-T2A-EGFP-WPRE-BGHpA sequence was cloned between Lgr5 homology arms flanking the gene stop codon. EGFP was cloned after the IRES sequence to generate the LGR5-IRES-EGFP-WPRE-BGHpA donor. CRISPR-Cas9 knock-in editing was carried out as described previously^4^. AKTP-MTO#93 Emp1-iCT was generated from a single tdTomato+ cell (clone#14). MTO#93 Emp1-iCT Lgr5-EGFP was first generated from a single tdTomato+ cell (clone#49), then nucleofected with the Lgr5-EGFP construct and stablished from a pool of sorted EGFP+ cells. MTO#93 Lgr5-iCT was generated by a single tdTomato+ cell (clone#2). MTO#93 Lgr5-DTR-EGFP was generated by sorting a pool of EGFP+ cells. Triple mutant AKP MTO#54^12^ Emp1-iCT was generated by sorting a pool of tdTomato+ cells. CRISPR-Cas9 gene editing was subsequently used to introduce a Smad4 mutation in AKP Emp1-iCT to generate AKPS MTOs as previously described^45^. Correct integration of the knockin cassettes was checked by PCR and sequencing of the genomic regions. Correct expression of the reporter cassette was tested by RT-qPCR or by sorting cells from tumors as described in the text. Genotyping primers are detailed in Supplementary Table 5.

### Generation of CRISPR-derived Tumor Organoids (CTOs)

To study the effect of gene driver mutations on the HRC phenotype, we sequentially introduced mutations in Apc, Kras, Tp53, Tgfbr2 and/or Smad4 in normal mucosa colonic organoids using CRISPR-Cas9 as described elsewhere^16,45,46^. Compound mutations were introduced in organoids derived from one c57BL/6J mouse, which allowed us to assess the impact of individual mutations on gene expression without other confounding variables. We named these organoids CRISPR-derived Tumor Organoids (CTOs) to distinguish them from Mouse Tumor Organoids (MTOs), that were stablished from GEMMs.

### Lentivirus production and MTO infection

For bioluminescent tracking, MTOs were infected with a lentivirus encoding an EGFP-firefly luciferase fusion reporter construct under the control of the PGK promoter. For YAP inhibition experiments, AKTP organoids were infected with shControl (SHC002) or shYAP1 (TRCN0000095864, TRCN0000095865, TRCN0000095866, TRCN0000095867) Mission Sigma-Aldrich lentiviral constructs. We also use a pInducer20 EGFP-TEADi vector, a gift from Ramiro Iglesias-Bartolome (Addgene plasmid #140145; http://n2t.net/addgene:140145; RRID:Addgene_140145), that blocks the activity of both YAP and TAZ^33^.

### Animal experimentation approval and maintenance

Experiments with mice were approved by the Animal Care and Use Committee of Barcelona Science Park under protocol CEEA-PCB-14-000053. Mice were maintained in a specific-pathogen-free (SPF) facility with a 12-h light–dark cycle, under controlled temperature and humidity (18-23°C and 40-60% respectively) and given ad libitum access to standard diet and water. All mice were closely monitored by authors, facility technicians and by an external veterinary scientist responsible for animal welfare. Authors monitored primary tumor and metastasis growth using intravital bioluminescence at least once a week.

### Inoculation of MTOs into the caecum and rectum

For all injections, c57BL/6J mice were purchased from Janvier Labs at six weeks of age and injected at 7 to 9 weeks of age. Sex always matched the origin of the tumor. Intra-caecum injections were used for the generation of primary tumors. Organoids were harvested and incubated for 30 minutes with cold HBSS to break down BME (Bio-Techne, 3533-010-02), without disrupting their structure. Cells were then counted and resuspended in 70% BME in HBSS for injection at a concentration of 0.1 X 10^6^ cells in 10 μl per mouse. Full organoids were injected with a 30G syringe into the submucosal wall of the distal caecum while looking through binocular lens. We introduced a significant modification to previous protocols^47^ by moving the injection site to the apex of the caecum, which allowed posterior surgical resection. For liver colonization studies we used intrasplenic injections of organoids as described before^12^. Rectal injections were performed following a previously described procedure^48^. Maximum tumor volume of 300mm^3^ allowed by the animal experimentation committee was never exceeded in these experiments except in experiments shown in Extended Data Fig 11j, where 1 mouse out of 19 developed larger tumor due to very fast tumor growth between the last measurement and the day of sacrifice.

### Primary tumor resection

Mice were anesthetized with isoflurane and placed in dorsal recumbency. The abdomen was shaved and sterilized with povidone-iodine surgical solution. A small midline incision - slightly to the left- was performed to open the skin and peritoneum and expose the abdominal area. We placed a sterile surgical drape on top of the abdomen with a circular hole above the incision and sprawled the caecum over the drape using cotton swabs and saline to keep it hydrated. After confirming the presence of a primary tumor, Kelly forceps were used to first knot the surgical suture into the caecum wall, in between the ileocecal junction and the primary tumor. This provided a grip for subsequent caecum ligation. After ligation, the apical caecum containing the primary tumor was excised and any remaining caecal tissue was trimmed. After resection and organ fixation, we measured primary tumor size using a caliper. We provide a video of the surgery, which usually lasted from 5 to 10 minutes (Supplementary video 1). Fitness of mice was monitored weekly throughout the experiment. Mice were euthanized four weeks after resection and metastasis were scored macroscopically.

### Pharmacological treatments

For iCasp9-inducible ablation experiments, animals were treated with dimerizer (AP20187, Medchem express, HY-13992) via intraperitoneal injection at 2.5 mg/kg 3 times per week (Emp1-iCasp9) or 5 mg/kg every day (Lgr5-iCasp9). For DTR-inducible ablation mice were treated with 16.7 μg/kg of diphtheria toxin (Sigma-Aldrich, 322326) three times per week. For immunotherapy experiments, 2 shots of 250 μg of anti-mouse CTLA-4 (C2444, Leinco) and/or anti-mouse PD-1 (P372, Leinco) were administered via intraperitoneal injection 5 days apart.

### Tumor dissociation for flow cytometry

Primary tumors and micro-dissected liver metastases were chopped with razor blades. Subsequent enzymatic digestion was performed with 200 U/ml collagenase IV (Sigma Aldrich, C5138) in HBSS (Lenovo) for 30 min at 37 °C, in a shaking water bath or a gentleMACS Dissociator (Miltenyi Biotec). Digested tissue fragments were then filtered through 100- and 40-μm meshes, washed, and treated for 5 minutes with ammonium chloride. Single-cell preparations were first blocked with anti-CD16/32 (clone 93; eBioscience) and then stained with APC anti-EPCAM (Biolegend, 118214) and BV605 anti-CD45 (Biolegend, 103155) antibodies at 1:200 concentration. In experiments measuring PD-L1 expression in tumor cells, cells were stained with FITC anti-EPCAM (Santa Cruz, clone G8.8, 53532) and APC anti-PDL1 (BD, 564715, clone MIH5) at 1:200 concentration. Finally, cells were resuspended in HBSS with 0.5% FBS and DAPI (Sigma Aldrich, D9542).

### Collection of primary CRC and metastasis samples

Metastatic seeding in the CRC relapse models is not synchronized; as it occurs in patients, tumor cells are shed from the primary tumor continuously during weeks until the day of resection. As a result, metastases expand for different periods in the liver and lung, and exhibit different sizes at experimental timepoints depending on when HRCs colonized the organ. We thus decided to classify according to size instead to time. Four different CRC samples were collected; Primary, Micrometastasis, Small metastasis and Macrometastases. Micrometastases and small metastases were collected from liver of mice 29 to 31 days post primary tumor implantations (i.e. at the time of primary CRC resection). Micrometastases samples were DTCs collected from livers with absent or residual bioluminescence ex vivo in which metastases were not visible. For small metastases, metastatic nodules were visible but small in size (<1.5mm). Macrometastases samples were derived from metastatic nodules larger than 4mm in livers from mice 64 to 66 days after primary tumor implantation. Primary tumor samples were paired with micro, small or macrometastases samples and were collected from both timepoints (29 to 31 and 64 to 66 days post primary tumor implantation).

### Isolation of liver residual DTCs

For isolation of low numbers of tumor cells from mice without visible metastases, whole livers were thoroughly minced with razor blades. After an initial 30-minute digestion with 200 U/ml collagenase IV (Sigma-Aldrich, C5138), samples were filtered through 100 μm meshes. Although most cells flowed through, a small fraction of the sample –highly enriched for tumor cells- was retained in the filter (Extended data Fig. 3k,l). Filters were then laid into a 6-well plate and covered with HBSS containing 200 U/ml collagenase IV, 200 μg/ml Dispase II (Sigma-Aldrich, D4693), 40 μg/ml DNase I (Sigma-Aldrich, 10104159001) and 13 μM rock inhibitor (Medchem Express, HY-10583). After re-digestion in a water bath for 30 minutes al 37 °C, most cells now seeped through the filters. The protocol continued with washing, ammonium chloride treatment and antibody staining as described above. DAPI- EGFP+ CD45- cells were gated to sort tumor cells.

### Histology and tissue staining

Standard hematoxylin/eosin (HE) and antibody staining were performed on 4-μm tissue sections using standard procedures, as described previously^12^. Details can be found in Supplementary Table 6. The following secondary antibodies were used: donkey anti-goat conjugated to Alexa 488/568/647 (Life Technologies A11055, A11057, A21447), donkey anti-rabbit conjugated to Alexa 488/568/647 (Life Technologies A21206, A10042, A31573) and donkey anti-mouse conjugated to Alexa 488/568/647 (Life Technologies A-21202, A10037, A31571) at RT. Digital scanned bright-field and fluorescent images were acquired with a NanoZoomer-2.0 HT C9600 scanner (Hamamatsu, Photonics, France). All images were visualized with the NDP.view 2 U123888-01 software (Hamamatsu, Photonics, France) with a gamma correction set at 1.8 in the image control panel.

### Co-culture of MTOs and colon fibroblasts

To address the role of fibroblasts in inducing HRCs, we seeded 5000 AKTP Emp1-iCT MTO cells with or without 100k mouse colon fibroblasts in BME (Bio-Techne, 3533-010-02) and supplemented them with MTO cell culture media^12^ without noggin nor galunisertib. Co-cultures were analyzed 7 days after by flow cytometry and confocal microscopy. For Fig. 3s and Extended Data Fig. 10e, MTOs and mouse colon fibroblasts were also co-cultured in hanging drops as previously described^49^.

### In Situ Hybridization on CRC patient samples

Paraffin-embedded tissue sections (2-3 μm in thickness) of human CRC primary tumors were air dried and further dried at 60 °C over-night prior any staining. To compare *EMP1* and *LGR5* expression, sections were hybridized with RNAscope® Probe Hs-LGR5 (ref: 311021, Bio-Techne R&D Systems) in C1 channel, a custom-made RNAscope® Probe Hs-EMP1 (, Bio-Techne R&D Systems, 895051-C2) in C2 channel and an Alexa568-conjugated antibody against E-CADHERIN. To compare the expression of *EMP1* with other HRC-marker genes, sections were hybridized with RNAscope® Probe Hs-EMP1 mRNA in channel 1 (Bio-Techne R&D Systems, 895051-C1) and stained with the relevant antibodies. FISH probe were detected using the RNAscope® Multiplex Fluorescent Detection Reagents Kit v2 (Bio-Techne R&D Systems, 323110).

### Multiplex immunofluorescence

Multiplex immunofluorescence staining was performed on 4-μm-thick formalin-fixed paraffin embedded sections using the OPAL protocol (Akoya Biosciences, Marlborough, MA) on the Leica BOND RXm autostainer (Leica Microsystems, Wetzlar, Germany). Six consecutive staining cycles were performed using the following primary antibody-Opal fluorophore pairings. Stroma panel: CD34 (1:3000, ab81289; Abcam)–Opal 520; CD146 (1:500, ab75769; Abcam)–Opal 570; α-SMA (1:1000, ab5694; Abcam)–Opal 620; PERIOSTIN (1:1000; Abcam, ab227049)–Opal 690; and E-E-CADHERIN (1:500; Cell Signaling, 3195)–Opal 650. Immune panel: (1) LY6G (1:300; BD Pharmingen, 551459)–Opal 540; (2) CD4 (1:500; Abcam, ab183685)–Opal 520; (3) CD8 (1:800; Cell Signaling, 98941)– Opal 570; (4) CD68 (1:1200, Abcam, ab125212)–Opal 620; (5) FOXP3 (1:400; Cell Signaling, 126553)–Opal 650; and (6) E-CADHERIN (1:500; Cell Signaling, 3195)–Opal 690. Primary antibodies were incubated for 60 minutes and detected using the BOND Polymer Refine Detection System (Leica Biosystems, Buffalo Grove, IL, DS9800) according to the manufacturer’s instructions, substituting DAB for the Opal fluorophores, with a 10 minute incubation time and without adding hematoxylin. Whole-slide scans and multispectral images (MSI) were obtained on the Akoya Biosciences Vectra Polaris. Batch analysis of the MSIs from each case was performed with the inForm 2.4.8 software provided. Finally, batched analyzed MSIs were fused in HALO (Indica Labs, Albuquerque, NM) to produce a spectrally unmixed reconstructed whole-tissue image, ready for analysis.

### Statistical analyses multiplex immunofluorescence

HALO ® IMAGE ANALYSIS PLATFORM software was used for quantification of cell phenotypes within metastases and primary tumors. Two matrices of counts (distinguishing immune and stromal panels) with the total number of cells per metastasis/primary (in rows) assigned to each cell population (in columns) were obtained. Multiple positives were present in both immune and stromal panels. These cases observed in the immune panel only represented the 0.7% of the total assignations and were removed from the analysis. For the stromal panel, aα-SMA and POSTN double positives were kept and labeled in a distinct category aα-SMA/POSTN. The rest of multiple positives, that accumulated the 7% of the total cells, were amalgamated and labeled as “stromal others”. For all metastases, information regarding metastasis size (n° of total cells in log2 scale), metastatic burden (defining micro, small and big metastases) and organ site were considered for subsequent analysis.

For the proportional stacked area graph, only the percentages of CD4, FOXP3, CD8, LY6G and CD68 for the immune panel, and the percentages of CD146, CD34, POSTN, α-SMA and α- SMA/POSTN for the stromal panel were considered. The cell composition of all measured metastases (in percentages) was averaged out using the R function aggregate, taking as grouping elements both metastasis size and metastatic burden. Linear mixed effects models were fitted independently for every cell population using the CLR-transformed values as response variable, the metastatic burden and the metastasis size as fixed effects, and the tumor Id (the tumor identifier) as random effect to consider the dependence between metastases for the same tumor.

### Quantification of tdTomato and EGFP fluorescence intensity

For the quantification of percentages of Emp1-tdTomato high and Lgr5-EGFP-high in tumor sections we used HALO® IMAGE ANALYSIS PLATFORM. Briefly, the epithelial tumor area was classified apart from the stroma, background and necrosis using a random forest algorithm. Single cells were detected and tdTomato/EGFP fluorescence intensity was measured for every cell. In primary tumors, Tomato-high and EGFP-high tumor cells were defined as the cells in the 90^th^ percentile of each sample. In liver metastases, Tomato-high and EGFP-high tumors cells were defined as the cells in the 90^th^ percentile for all metastases measured. For Emp1-iCT liver metastases (in Extended Data Fig. 6j) and Lgr5-DTR-EGFP liver metastases (in Extended Data Fig. 11q), tumor cell fluorescence-intensity was analyzed using ImageJ with a custom-made macro. Tumor cells were first detected and isolated using E-CADHERIN to create a mask. TdTomato or EGFP intensity was calculated for every pixel inside the masked area. Then, we plotted the percentage of fluorescence high and low pixels as a function of the area of the metastases (measured in pixels).

### Gene expression analysis by RT-qPCR

RT-qPCR and Microarrays were used to compare subpopulations of Emp1-high and -low, Lgr5-high and -low cells dissociated from MTO#93 organoids grown *in vitro* or *in vivo*. Subpopulations were defined in flow cytometry as the top/bottom 10% in fluorescence expression. RNA from 2000 cells was extracted and retrotranscribed to cDNA as described previously^50^. To analyze gene expression changes RT-qPCR was performed using 5 ng of cDNA per each real-time qPCR well. Real-time qPCRs were performed with TaqMan Universal PCR Master Mix (Applied Biosystems, 4369016) or PowerUp SYBR Green Master Mix (Applied Biosystems, 100029284) in triplicates, following manufacturer’s instructions. Gene expression levels were normalized using the housekeeping genes *PPIA* or *B2M*. The following TaqMan assays were used: TdTomato-BGHPA (custom made probe; F: GGGCATGGCACCGGCAGCACC, R: CCTACTTGTACAGCTCGTCCATGCC), MmPPIA (Mm002342430_g1), MmB2m (Mm00437762_m1), EGFP (Mr04097229mr), MmEmp1 (Mm00515678_m1), MmLgr5 (Mm0043889_m1), MmSmoc2 (Mm00491553_m1), MmLamC2 (Mm00500494), Ctgf (Mm01192033_g1). The following Sybr primers were used:_MmYAP (F: ACCCTCGTTTTGCCATGAAC, R: TGTGCTGGGATTGATATTCCGTA), MmCyr61 (F: AGGTCTGCGCTAAACAACTCA, R: ATATTCACAGGGTCTGCCTTCT).

### Western blotting

Cells were harvested in Cell Recovery Solution (Corning, 354257), pelleted and lysed in RIPA buffer (Tris-HCl pH 7.4, 150 mM NaCl, 0.5% Sodium deoxycolate, 0.1% SDS, 1 mM EDTA, 10 mM NaF, 1 mM PMSF, 1% Triton X-100) supplemented with protease and phosphatase inhibitors (Sigma Aldrich). Primary antibodies we incubated overnight at 4°C at the following dilutions: YAP/TAZ (Cell Signalling, CS93622) 1:1000, TAZ (Cell Signalling, 72804), 1:20000 vinculin (Sigma Aldrich, 9264). Anti-Rabbit IgG HRP (NA934V) and anti-mouse IgG HRP (NXA931V) secondary antibodies were diluted 1:5000 and incubated for 1h at RT with the PVDF membranes. Membranes were visualized using Hyper Processor (Amersham Pharmacia Biotech).

### RNA sequencing and analysis

We used RNA sequencing to profile CTOs with different genotypes and to profile the effects of chemotherapy treatment on MTOs. For the first dataset, 5,000 cells from CTOs with multiple genotypes (AT, AP, AS, AK, ATP, APS, AKP, AKS, AKPT, AKPS) were seeded in BME (Bio-Techne, 3533-010-02) and cultured in full stem cell medium^4^. Organoids were harvested 7-10 days after seeding. For chemotherapy experiments, MTO54 organoids were dissociated with TryplE express (, Thermo Fisher, 12604039) and single cells were resuspended in BME in 6 well plates in complete stem cell medium. Two days later, the media was removed and fresh media containing Folfiri (5FU at 50 μg/μl (Sigma-Aldrich, F6627) plus SN38 at 100 nM (MedChem, HY-13704)) or control (MTO stem cell media^12^) was added for two days.

RNA was extracted and sequenced as we previously described^16^. RNAseq reads from datasets (CTOs or chemotherapy treatment) were aligned with STAR (v2.5.2)^51^ with default parameters to the Mus musculus reference genome built with annotations version GENCODE_mmusculus_vM25. SAM files were converted to BAM and sorted using Sambamba (v0.7.1)^52^. Count matrices were generated using the R (v4.0.5) package Rsubread (v2.4.3)^53^ with the GENCODE_mmusculus_vM25 custom annotation. Data from parental versus metastatic MTOs was processed as described in Tauriello et al^12^. Genewise differential expression in the chemotherapy dataset between controls and Folfiri treatment was performed using the R package DESeq2 (v1.30.1)^54^. Normalized values for plots were obtained via the *rlog* function of the same package. Signature scores were defined as the scaled mean of all genes in the signature after scaling the expression matrix. Comparison of signature scores between conditions was assessed using a t-test.

### Microarray expression analyses

Microarrays GeneChip Mapping 250K Nsp Assay Kit, Affymetrix) were used to compare Emp1-high and Emp1-low cells from MTO#93 Emp1-iCT primary tumors grown for 4 weeks; and to compare MTO#93 Emp1-iCT MTOs grown in vitro alone or in combination with mouse colon fibroblasts for 7 days. Samples were processed with oligo v1.46.0^55^ (fitProbeLevelModel: background = TRUE, normalize = TRUE, target = “core”, method = “plm”). Raw cel files were normalized with RMA method^56^ (default paramaters). Probesets were annotated with Clariom_S_Mouse_HT-na36-mm10-transcript Affymetrix databases. Standard quality controls were considered to identify abnormal samples^57^. No samples were excluded due to quality issues.

Differential expression analysis of Emp1-high vs -low data was performed using a linear model with empirical shrinkage (limma R package)^58^, taking into account the paired data setting. Differential expression analysis of co-culture data was performed using the same regression method, but considering as adjustment variable the Eklund metric pm.iqr^59^ to reduce the influence of technical variation. Benjamini-Hochberg FDR was used for multiple comparisons correction. Gene set analysis was used to explore the enrichment in custom gene sets. The limma’s rotation-based approach for enrichment^60^ was considered to represent the null distribution. The maxmean enrichment statistic proposed in^61^, under restandardization, was used for competitive testing. Gene signatures (Supplementary Table 7) z-scores^62^ were used to measure pathway activity. For doing so, normalized expression was adjusted for biological replicate, centered and scaled genewise according to the mean and the standard deviation computed across samples. Gene signature z-score was summarized by taking the average of its constituent genes. In addition, a global signature was computed using all genes and used for a priori centering of signature scores. This strategy has been proved to be useful to avoid systematic biases. Only the expression of the most variable probe sets per gene were considered for gene set analyses.

### 10X mouse single cell RNA sequencing analysis

CellRanger^63^ (v4.0.0) was used to align reads to a custom refdata-gex-mm10-2020-A transcriptome including the EGFP and Luciferase genes. Gene expression was analysed with Seurat (v4.0.3)^64–67^. A total of 1,330 cells having <20% mitochondrial content and >3,000 detected genes were considered. Ribosomal reads (17% of the total) were removed. Mitochondrial content was regressed out during SCT normalization^68^.

SCT transformed counts were smoothed with MAGIC (v.2.0.3)^69^. Gene signature expression was summarized by taking the average MAGIC score of its constituent genes. HRCs and Lgr5+ cell populations were defined by having a score above the 75th percentile. FindAllMarkers was used to identify differentially expressed genes from raw counts. The HRCs cell population was compared to the rest in order to identify mice HRC markers. Testing was limited to genes detected in >10% of cells and showing >0.25 log-fold difference. Functional enrichment analysis was performed using the GSEApreranked^70^ method ranking genes by the log2 average fold change. The significance threshold was set at 5% Benjamini-Hochberg FDR.

To select for marker genes to track HRCs in mouse tumors, we computed the correlation scores for all genes with the EpiHR signature in the SMC human dataset and the mouse 10X dataset. We used MAGIC score for genes and signature scores as defined previously.

### Mouse SMART-seq_v2 single cell RNA sequencing analysis

Smart-seq2 reads were aligned to the UCSC_GRCm38.mm10 genome with zUMIs^71^ and analyzed with Seurat^64–67^ (v4.0.3). Four technical batches of AKTP at different stages were merged into a single object and two technical batches of AKP micrometastases were merged into another object. A total of 1,057 cells having <20% mitochondrial content and >20,000 UMIs were considered for AKTP, whereas 414 cells with <20% mitochondrial content and >100,000 UMIs were isolated from AKP micrometastases. Ribosomal reads were removed. Mitochondrial content was regressed out during SCT normalization. SCT transformed counts were further imputed and smoothed with MAGIC (v.2.0.3)^69^. Gene signature expression (Supplementary Table 7) was summarized by taking the average MAGIC score of its constituent genes. Non-epithelial cells were removed and normalized again. In order to improve the integration of the four AKTP batches, the IntegrateData function was used with pre-computed anchors based on 3,000 features. The integrated dataset was SCT normalized, smoothed with MAGIC (v.2.0.3)^69^, and clustered with FindClusters Seurat function (resolution = 1.2). FindMarkers was used to identify differentially expressed genes from raw counts. CellRank^15^ were used to uncover the cell-state dynamics of CRC metastasis from RNA velocity estimates^72,73^. Gene expression or signature expression was represented as a function of latent time with R^74^. Additionally, the integration, normalization, imputation, and trajectory analysis were performed independently for the subset of cells harvested from primary tumors, incipient metastasis, and macrometastases.

### Creation of CRC transcriptomic Meta-cohort

Public CRC transcriptomic datasets were downloaded from GEO^75^ and NCI GDC commons^76^, pre-processed and homogenized into a unique Meta-cohort including 1830 samples from: TCGA^77^, GSE38832^78^, GSE44076^79^, GSE33113^80^, GSE14333^81^, GSE39582^82^ and GSE37892^83^ (Supplementary Table 1). The last four datasets include disease-free survival information with a median follow-up of 3.7 years and clinical information across all datasets is gender, age, stage and location of primary tumor (Supplementary Table 1). When not available, MSI status was imputed using the transcriptomic signature reported in^84^ through density-based non-parametric clustering^85,86^. Signature scores were computed as the scaled mean of the genes in the signature after scaling the expression matrix.

Microarray data were processed separately using RMA^57^. Information about sample processing and hybridization was retrieved from the CEL files. For TCGA, the Legacy version was used for expression with clinical annotation from October 2016. Genes were annotated using Ensembl Biomart database (GRCh37)^87,88^. Duplicated samples across platforms were removed from the GA dataset, as well as samples from other locations than colon or rectum. RSEM^89^ expressions were already log2-transformed and quantile normalized. Samples TCGA-A6-2679-01A and TCGA-AA-A004-01A were excluded due to an anomalous expression distribution among all samples.

Each microarray series was corrected by Eklund metrics^59^, center and scanning date using a mixed-effect linear model^90^. Age, gender, stage, site and MSI were also included in these models. TCGA sets were corrected for occurrences of combinations of center and plate identifier (random effect). For microarrays, probesets were summarized at the gene-level using the first principal component from probesets in that gene. This component was then centered and scaled to the weighted mean of the means and standard deviations of the probesets. The sign of the component was changed to the sign of the probeset contributing the most. All datasets were merged after genewise standardization to the GSE39582 series according to the distribution of gender, age, MSI and stage using undersampling: a sub-sample of the same number of patients and the same distribution according to these clinical variables was selected from the GSE39582 series for each dataset. Expression values were truncated to the maximum and minimum values observed in the reference dataset.

### Gene screening for association with relapse

Each gene in the Meta-cohort was evaluated for linear association of its expression with recurrence using a frailty Cox proportional hazards model^91,92^, including dataset and technical variables. Statistical significance was assessed by means of a Wald test. Hazard Ratios (HR) and their corresponding 95% confidence intervals were computed as a measure of association. The 2530 genes with HR>1 and p<0.05 were included in the all_HR signature.

We used the GSE39397 dataset^6^ which includes expression profiles of epithelial cancer cells (EPCAM+), CAFs (FAP+), leukocytes (CD45+) and endothelial cells (CD31+) isolated by FACs from dissociated primary CRCs (n=14), to classify genes according to their expressions in these populations. The EpiHR signature contains genes from the allHR signature which are upregulated (Fold change >1; p<0,05) in EPCAM+ cells compared to the three TME populations (FAP+, CD31+ and CD45+). AllHR genes that did not pass this cut-off comprised the TME-HR signature.

Signature scores were computed as the scaled mean of the genes in the signature after scaling the expression matrix. The association with recurrence of the EpiHR signature in the whole dataset as well as the subclasses of CMS samples was assessed as described above. The likelihood ratio test comparing EpiHR and AllHR p-value was computed with the *drop1* R function. Kaplan Meier plots were generated using the *survfit* and *plot* functions.

Association between clinical variables and the EpiHR signature in the CRC metacohort was assessed by fitting a linear model for each variable of interest independently. Technical factors (dataset and center, as described in extended methods) were included as covariates.

### Association between oncogenic alterations and the EpiHR score

Annotations of oncogenic alterations for the TCGA samples were downloaded from ^93^. A Wilcoxon test comparing the expression of mutated vs wild-type samples was performed independently for every alteration. The difference of expression medians was used as a measure of the impact of each mutation in the gene expression.

### Patient 10X single-cell analysis

Count matrices were downloaded from arrayExpress (E-MTAB-8107 for samples EXT001, EXT002, EXT003, EXT009, EXT010, EXT011, EXT012, EXT013, EXT014, EXT018, EXT019, EXT020, EXT021, EXT022, EXT023, EXT024, EXT025, EXT026, EXT027, EXT028) and GEO (GSE132465 for all SMC..-T samples)^8^. The remaining EXT samples were processed as referred in E-MTAB-8107 and deposited in ArrayExpress under accession number E-MTAB-9934. Cells with mitochondrial content higher than 20%, less than 1000 counts, more than 6000 or less than 200 genes were discarded. Ribosomal genes were also removed from the matrix to avoid technical biases during normalization. Samples with less than 500 cells and not classified as core tumor were discarded from further analyses. The Korea (SMC samples) and Leuven (EXT samples) cohorts were processed independently following the R package Seurat V3 recommendations^67^: samples were combined and normalized using the SCTtransform function regressing mitochondrial percentage, with the method “glmGamPoi”, min.cells=1 and return.only.genes=FALSE in order to keep the maximum number of genes. Dimensionality reduction and visualization were performed using RunPCA and RunUMAP, with 26 principal components. Expression was imputed and smoothened using the MAGIC algorithm^69^. The expression of the EPCAM gene was used to define the connected components corresponding to epithelial cells.

The epithelial component of each cohort was processed as follows: cells with less than 1000/3000 (SMC/KUL) counts and less than 200/1250 genes detected were removed from the dataset. No further normalization was applied. RunPCA, RunUMAP, FindNeighbors and FindClusters were applied, with 5/7 principal components and resolution of 0.7. Expression was imputed and smoothened using the MAGIC algorithm. Signature scores were computed as the mean value of the MAGIC expression per cell for all genes in the signature. The EpiHR and Lgr5 cell populations were defined as cells with the corresponding signature expression above the 75 percentile. Population markers were found using *FindMarkers*. Functional enrichment was computed through the Gene Set Enrichment Analysis implementation in^94^ with genes ordered by fold change. Samples in the SMC and KUL datasets were annotated according to their iCMS class^11^.

To identify the core gene expression program upregulated in HRCs, we computed the correlation scores for all genes with the EpiHR signature in the SMC and KUL human dataset. The resulting list was ranked by the average correlation across all samples (Supplementary Table 2). The coreHRC signature was defined as the top 100 genes in the SMC dataset.

### Association of HRCs and tumor microenvironment populations

We classified clusters according to the expression of known markers of microenvironment components^8^. For each population we estimated the association between its percentage per sample and the percentage of HRCs. Spearman correlation coefficient and p-value were used to assess said association.

### Clustering of EpiHR genes according to expression correlation

We computed the Pearson correlation coefficient for all pairwise combinations of genes in the EpiHR signature. We then applied hierarchical clustering *(hclus* in R with method “complete”) and defined 6 clusters via the *cutree* function. Upon visual inspection we decided to merge the three clusters with higher correlation, resulting in Cluster 1 (Extended Data Fig. 1h).

### Ethics oversight

Samples of primary CRC from patients used for IF and ISH analysis were obtained from the Hospital Clinic de Barcelona-IDIBAPS Biobank (B.0000575), which is integrated in the Spanish National Biobanks Network. Samples were donated by patients under informed consent and they were processed following standard operating procedures with the appropriate approval of the Ethics and Scientific Committee of the Hospital Clinic de Barcelona (register code: HCB/2020/1478) and according to the guidelines of the European Network of Research Ethics Committees, following European, national and local laws.

### Statistics

No statistical test was used to determined sample size upfront. Instead sample size was determined empirically according to previous knowledge of the variation in the experimental setup ^4,12,34^. A minimum of four mice were quantified in each experiment and each condition. For the majority of in vitro experiments, we used n=>3 according to previous experience with similar experiments. Additional information is detailed in the reporting summary. Automated blind quantifications and blind data analysis were done whenever possible. The sample size typically results in standard error <25% of the mean. No data from in vitro or in vivo experiments were excluded, except for the CRC relapse model, where a small fraction of mice, typically 1 in every 10 in each experiment, were not included in the follow up due to invasion into the ileocaecal junction, which impeded successful surgery. Occasionally, mice bearing CRCs were sacrificed 1-2 days after surgery due to significant weight loss or unhealthy aspect, according to protocols approved by the animal experimental committees. Data distributions were assumed to be normal but this was not formally tested. Transformations were applied whenever needed. For percentage data, normality was assumed for values far form 0 or 100. Statistical analyses were performed using R software package (v.4.0.5) and GraphPad Prism (v.7.03)

### Reporting summary

Further information on research design is available in the Nature Research Reporting Summary linked to this article.

## Extended Data

## Supplementary Material

Supplemenatry tables

Supplementary Data

## Data Availability

All data relevant to this study are available from the corresponding author upon reasonable request. Expression arrays and RNA-seq data are available at Gene Expression Omnibus (GEO). The accession number for gene expression sequencing experiments reported in this paper are GEO: GSE190055 (Arrays Emp1-high vs Emp1-low AKTP tumor cells), GSE208139 (Arrays MTOs co-cultured with fibroblasts), GSE207974 (RNAseq chemotherapy) and GSE207668 (RNAseq CTOs). Count matrices for single cell RNAseq experiments were deposited in ArrayExpress under accession number E-MTAB-11284 (10X AKTP primary tumors) E-MTAB-11302 (Smart-seq metastatic progression) and E-MTAB-11981 (Smart-seq AKP micromets). Additional metadata and processed data files, including UMAP embeddings and gene signature scores, are available at Synapse (syn35000645). Source data are provided with this paper.
