# Supplementary material for: Metastatic recurrence in colorectal cancer arises from residual EMP1+ cells
Source: Nature. Author manuscript; Available in PMC 2024 Dec 8. (PMC7616986; doi:10.1038/s41586-022-05402-9)

Supplementary Data 1. Uncropped Western Blot related to Ext.Fig.9g

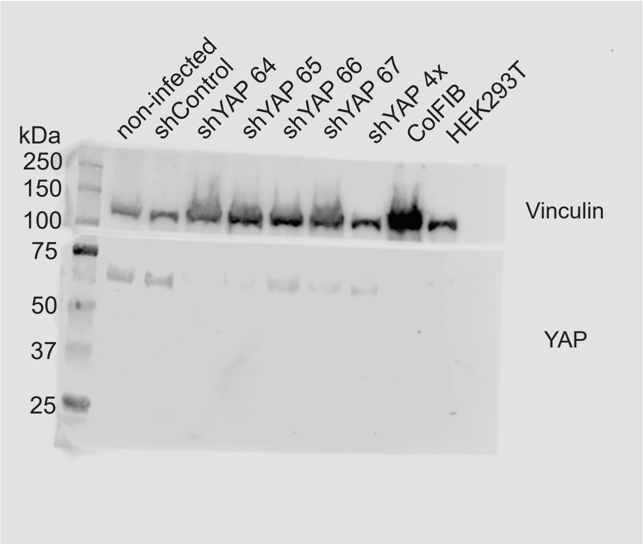

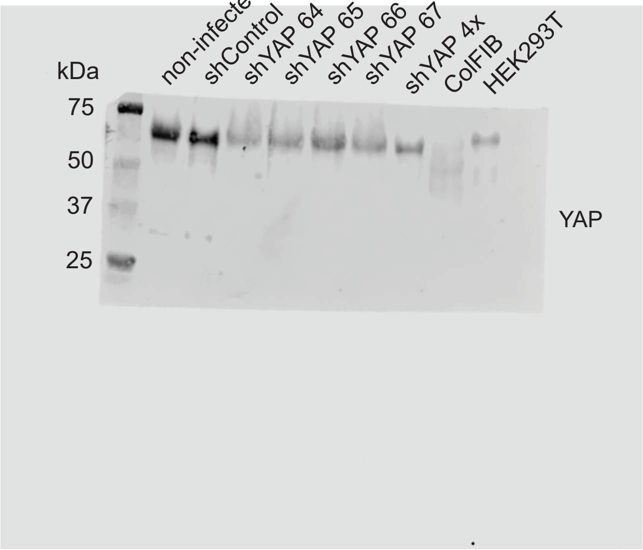

Supplementary Data 2. Example of gating strategy

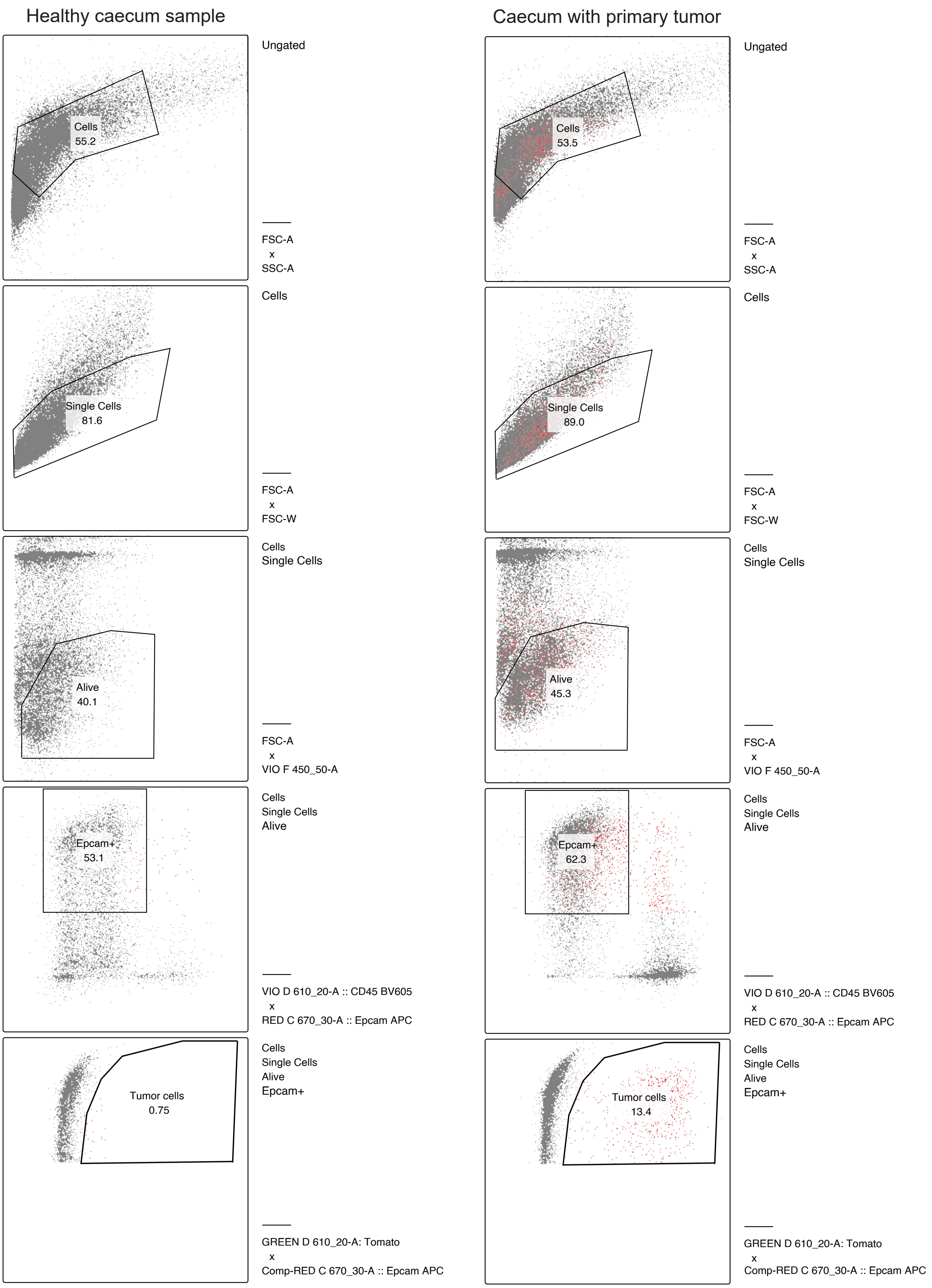

Supplement: Supplementary Data [file EMS181817-supplement-Supplementary_Data.pdf]
