# Supplementary material for: Metastatic recurrence in colorectal cancer arises from residual EMP1+ cells
Source: Nature. Author manuscript; Available in PMC 2024 Dec 8. (PMC7616986; doi:10.1038/s41586-022-05402-9)
Supplement: Supplemenatry tables [file EMS181817-supplement-Supplemenatry_tables.pdf]

Metastatic recurrence in colorectal cancer arises from residual EMP1+ cells

Adrià Cañellas-Socias[1,2], Carme Cortina[1,2], Xavier Hernando-Momblona[1,2], Sergio Palomo-Ponce[1,2], Eoghan J. Mulholland[3] , Gemma Turon[1], Lidia Mateo[1], Sefora Conti[4], Olga Roman[1], Marta Sevillano[1,2], Felipe Slebe[1], Diana Stork[1], Adrià Caballé-Mestres[1], Antonio Berenguer-Llergo[1], Adrián Álvarez-Varela[1,2], Nicola Fenderico[1], Laura Novellasdemunt[1], Laura Jiménez-Gracia[5], Tamara Sipka[1], Lidia Bardia[1], Patricia Lorden[5], Julien Colombelli[1], Holger Heyn[5,6], Xavier Trepat[4,7,8,9], Sabine Tejpar[10], Elena Sancho[1,2], Daniele V.F. Tauriello[1,11], Simon Leedham[3,12], Camille Stephan-Otto Attolini[1], Eduard Batlle[1,2,9]

1. Institute for Research in Biomedicine (IRB Barcelona), The Barcelona Institute of Science and Technology (BIST), Barcelona, Spain.
2. Centro de Investigación Biomédica en Red de Cáncer (CIBERONC), Barcelona, Spain.
3. Gastrointestinal Stem Cell Biology Lab, Wellcome Centre Human Genetics, University of Oxford, Oxford, UK.
4. Institute for Bioengineering of Catalonia (IBEC), The Barcelona Institute of Science and Technology (BIST), Barcelona, Spain.
5. CNAG-CRG, Centre for Genomic Regulation, The Barcelona Institute of Science and Technology (BIST), Barcelona, Spain.
6. Universitat Pompeu Fabra (UPF), Barcelona, Spain.
7. Facultat de Medicina, Universitat de Barcelona, Barcelona, Spain.
8. Centro de Investigación Biomédica en Red en Bioingeniería, Biomateriales y Nanomedicina (CIBER-BBN), Barcelona, Spain.
9. Institució Catalana de Recerca i Estudis Avançats (ICREA), Barcelona, Spain.
10. Digestive Oncology, Department of Oncology, Katholieke Universiteit Leuven, Leuven, Belgium.
11. Department of Cell Biology, Radboud Institute for Molecular Life Sciences, Radboud University Medical Center, Nijmegen, The Netherlands.
12. Translational Gastroenterology Unit, John Radcliffe Hospital, University of Oxford and Oxford National Institute for Health Research Biomedical Research Centre, Oxford, UK.

Corresponding author: Eduard Batlle

30-40% of colorectal cancer (CRC) patients undergoing curative resection of the primary tumor will develop metastases in the following years[1]. Therapies to prevent disease relapse remain an unmet medical need. Here we uncover the identity and features of the residual tumor cells responsible for CRC relapse. Analysis of single-cell transcriptomes of CRC patient samples revealed that the majority of poor prognosis genes are expressed by a unique tumor cell population that we named High Relapse Cells (HRCs). We established a human-like mouse model of microsatellite stable CRC that undergoes metastatic relapse following surgical resection of the primary tumor. Residual HRCs occult in mouse livers after primary CRC surgery gave rise to multiple cell types over time, including Lgr5+ stem-like tumor cells[2–4], and caused overt metastatic disease. Using *Emp1* (epithelial membrane

protein 1) as a marker gene for HRCs, we tracked and selectively eliminated this cell population. Genetic ablation of Emp1-high cells prevented metastatic recurrence and mice remained disease-free after surgery. We also discovered that HRC-rich micrometastases were T-cell infiltrated yet became progressively immune-excluded during outgrowth. Treatment with neoadjuvant immunotherapy eliminated residual metastatic cells and saved mice from relapsing after surgery. Together, our findings reveal the cell-state dynamics of the residual disease in CRC and anticipate that therapies targeting HRCs may help avoid metastatic relapse.

MAIN

The CRC poor prognosis transcriptome

Surgical resection of the primary CRC effectively cures most patients diagnosed with locoregional disease[1]. However, about 5% of AJCC Stage I, 15% of Stage II and 40% of Stage III patients will develop metastases over the following years[1]. We and others have previously shown that the vast majority of genes that predict high risk of disease relapse in CRC are expressed by cells of the tumor microenvironment (TME), particularly by cancer-associated fibroblasts (CAFs)[5–7]. To further investigate this finding, we sought to map the expression of the poor prognosis CRC transcriptome at the single-cell level. Using a large pooled transcriptomic cohort of primary CRC samples (n=1830 stage I-III CRC, Supplementary Table 1), we identified 2530 genes that predicted disease relapse (HR>1, p-val<0.05) (Fig. 1a). Subsequently, the expression of this poor prognosis geneset was analyzed in two independent single cell RNA sequencing (scRNAseq) CRC datasets that included both tumor epithelial and microenvironment cells; 20 patients corresponding to the Samsung Medical Center (SMC) cohort and 7 patients from the Katholieke Universiteit Leuven (KUL) cohort[8] (Fig. 1b-d and Extended Data Fig. 1a-c). Supporting our previous findings, CAFs, endothelial cells and, to a lower extent, myeloid cells expressed the highest levels of poor prognosis genes (Fig. 1b and Extended Data Fig. 1a). However, detailed analysis of the recurrence geneset in specific cell populations purified from primary CRC patient samples (tumor cells/EPCAM+, leukocytes/CD45+, endothelial cells/CD31+ and CAFs/FAP+)[6] revealed that 99 out of the 2530 genes were upregulated in epithelial tumor cells compared to TME cells (Fig. 1a and Supplementary Table 2). Indeed, these 99 recurrence-associated genes (from now on named EpiHR for Epithelial-specific high-risk geneset), showed epithelial tumor cell-restricted expression patterns in the SMC (Fig. 1d) and KUL (Extended Data Fig. 1c) scRNAseq cohorts. EpiHR expression levels predicted recurrence with an accuracy equivalent to the subset of poor prognosis genes expressed in the tumor microenvironment (Fig. 1e). In multivariate

analysis including the two signatures and clinical variables, the TME-HR and EpiHR genesets were independent prognostic factors (EpiHR: HR (+1 SD) = 2.26, p-val=1.2×10^{7}; TME-HR: HR (+1 SD) =1.74, p-val=8×10^{-4}). The EpiHR signature was significantly associated with right-side colon cancer and to AJCC Stages III-IV (Extended Data Fig. 1d). In addition, it stratified CRC patients within each consensus molecular subtype[9] (CMS) into high and low risk of relapse (Extended Data Fig. 1e). Thus, the EpiHR geneset encodes determinants of disease relapse with epithelial tumor cell-specific expression.

Identification of EpiHR+ tumor cells

Representation of tumor epithelial CRC cells using Uniform Manifold Approximation and Projections (UMAPs) showed that 18 out of 27 CRCs contained cells labeled with the EpiHR geneset in proportions ranging from 1.4% to 98.1% (Fig. 1f-h and Extended Data Fig. 1f,g). We named this tumor cell population HRCs (for High Relapse Cells). Analysis of the EpiHR signature revealed a large number of highly correlated genes exhibiting overlapping expression in HRCs (Gene clusters 1 and 3 in Extended Data Fig. 1h,i and Supplementary Table 2). The EpiHR only contains epithelial-specific genes, reflecting only a fraction of the HRC transcriptome. We further identified a core gene expression program upregulated in HRCs of most tumor samples (Extended Data Fig. 2a-b and Supplementary Table 2). HRCs belonging to different CRCs displayed a shared enrichment pattern of annotated genesets, implying that they co-opt a similar phenotype and play equivalent functions (Extended Data Fig. 2c-d). Differential expression analysis of HRCs versus non-HRCs revealed prominent enrichment in genes related to hypoxia, cell-to-cell adhesion, extracellular matrix, actin cytoskeleton and regulation of cell migration (Fig. 1i). Amongst them (Supplementary Table 2), the collagen sensing receptor DDR1, the integrins α2, α3 and β4 or the protease PLAUR have been repeatedly associated with tumor cell invasion, extravasation and metastasis in multiple cancer types. Incidentally, we also discovered that the signature of basal-like pancreatic cancer cells[10] marked both human and mouse HRCs suggesting that they adopt a state akin to the most aggressive subtype of pancreatic cancer (Extended Data Fig. 2e and Supplementary Table 3). Further reinforcing our observations, most scRNAseq samples containing abundant EpiHR+ cells were stage III and IV CRCs (Fig. 1j – p=0.051 Stage I+II versus III+IV).

Widespread evidence has demonstrated that CRC growth is driven by a subset of LGR5+ stem cell-like tumor cells[2–4]. However, our analyses revealed that HRCs represent a distinct cell population, shown by their mutually exclusive distribution in UMAPs (Fig. 1k-l, Extended Data Fig.

2f-g). Quantification showed that only one tumor sample exhibited a significant number of HRCs co-expressing the LGR5 signature (7.79% sample SMC04, Fig. 1h) whereas five others included a minimal fraction (<3%) of cells marked by both *LGR5* and the HRC gene programs. The expression patterns of several WNT/intestinal stem cell marker genes confirmed that HRCs were not LGR5+ stem-like tumor cells (Extended Data Fig. 2h-o). Indeed, a subset of CRCs exhibited marginal WNT target gene expression levels (Extended Data Fig. 2p-q) yet contained HRCs (identified with an * in Fig. 1h). Furthermore, HRCs represented a subset of cells in both epithelial intrinsic consensus molecular CRC subtypes iCMS2 and iCMS3[11] (Extended Data Fig. 2r).

We previously described that mice bearing mutations in *Apc, Kras, Tgfbr2 and p53* (AKTP) in Lgr5+ ISCs develop human-like metastatic CRCs[12]. We analyzed CRCs generated by implantation of AKTP mouse tumor organoids (MTOs) in the caecum of c57BL/6 mice by scRNAseq (Fig. 1m-o). Mirroring the observations in human tumor samples, we found that mouse CRCs contained abundant HRCs and that this population did not express the Lgr5+ ISC-like expression program (Fig. 1m-o and Extended Data Fig. 2s). HRCs were enriched in similar gene categories in both species, including the pancreatic basal-like signature, implying functional equivalence (Fig. 1i and Extended Data Fig. 2s-t). Bulk RNA sequencing analysis of our MTO biobank[12] showed elevated EpiHR and coreHRC gene signatures in AKTP MTOs derived from metastases compared to those established from primary CRCs (Extended Data Fig. 2u).

Dynamics of metastatic cell states

We developed a new mouse model of metastatic relapse that allowed us to investigate the contribution of HRCs to metastatic recurrence. In brief, we innovated classical needle-based orthotopic injections by relocating them to the tip of the caecum, which allowed complete surgical excision of singular invasive CRCs (Fig. 2a, Extended Data Fig. 3a-c and Supplementary Video). Dual GFP/Luciferase-labelled AKTP MTOs grew rapidly in the caecum of c57BL/6 mice, colonized the adjacent mucosa and generated invasive cancers (Extended Data Fig. 3a,b). Bioluminescence imaging (BLI) revealed that mice remained free of primary disease after surgical resection yet, over the following days, they relapsed in the form of liver metastases (Fig. 2b,c and Extended Data Fig. 3d-e). Occasionally, we also observed metastases in mesenteric lymph nodes, lungs, peritoneum and diaphragm (Extended Data Fig. 3f-h). Primary tumor resection shortly after implantation cured all mice, whereas surgery at later points resulted in increased proportions of mice developing metastatic recurrences (Fig. 2b). In experiments of early primary tumor resection (day 11-15), faint bioluminescence could be detected ex vivo in some livers

immediately after surgery, implying the presence of residual disseminated tumor cells. Lightsheet 3D fluorescence imaging revealed 3 to 10-cell micrometastases at the time of resection (Fig. 2d). Based on these observations, we established 4-5 weeks post-implantation as the optimal timepoint to enable a complete surgical resection of the primary CRC. We also developed a CRC relapse model based on the implantation of AKP MTOs in the caecum. These triple mutant CRCs exhibited delayed kinetics of metastatic recurrence after surgical extirpation of the primary CRC (Extended Data Fig. 3i).

We next sought to profile tumor cells along the process of relapse. Isolation of residual tumor cells from large organs has been historically a major hurdle in cancer research[13]. We devised a tissue dissociation strategy that enriched for residual tumor cells from whole liver samples (Extended Data Fig. 3j). In brief, we discovered that during tissue preparation for FACS, the vast majority of luciferase+ tumor cells were retained in 100 μm filters after mild enzymatic digestions, whereas most parenchymal liver cells flowed through in these conditions (Extended Data Fig. 3k,l). By re-digesting cells retained in the filter, we obtained a 400-fold GFP-Luciferase+ enriched metastatic tumor cell preparation (Extended Data Fig. 3m). This step allowed purification of residual tumor cells from individual livers exhibiting absent or very low *ex vivo* bioluminescence (Extended Data Fig. 3n). By means of this approach, we profiled by Smart-seq2 scRNAseq 900 GFP+ tumor cells derived from livers collected at different time points after implantation of MTOs as well as from their corresponding primary CRCs (Fig. 2e). We confirmed the presence of micrometastases, small metastases or macrometastases by bioluminescence measurements in the resected livers (Extended Data Fig. 3n).

UMAP representation showed that tumor cells from primary tumors and metastases overlapped to a large extent (Fig. 2e,f and Extended Data Fig. 4). Hierarchical clustering analysis identified six cell clusters (Fig. 2g and Extended Data Fig. 4b, c). Cluster 0 included cells that expressed elevated levels of proliferation and biosynthesis-encoding genes (Fig. 2g and Extended Data Fig. 4b, c). Lgr5+ ISC-like tumor cells occupied two different clusters depending on high (cluster 1) and low (cluster 2) expression levels of the Ki67 proliferative signature[14] (Fig. 2g and Extended Data Fig. 4c). Tumor cells of cluster 3 upregulated the differentiation marker *Krt20*. Clusters 4 and 5 were largely enriched in HRCs. Some cells in cluster 4 expressed *Krt20* suggesting that HRCs can also undergo differentiation (Fig. 2g and Extended Data Fig. 4b, c). Quantification of cell types revealed a dynamic distribution of cell populations across the metastatic relapse process (Fig. 2h). Primary CRCs and macrometastases exhibited similar distribution of cell populations,

including proliferative cells, Lgr5+ ISC-like cells, Krt20+ differentiated tumor cells and HRCs, most of which were also Krt20+ (Fig. 2h). In contrast, micrometastases were largely enriched in undifferentiated (Krt20-) HRCs and also contained abundant proliferative cells (Fig. 2h). Small metastases were mainly formed by Lgr5+ ISC-like cells in both Ki67+ and Ki67- states and contained fewer HRCs than micrometastases (Fig. 2h and Extended Data Fig 4d).

The computational trajectory inference algorithm CellRank[15] predicted diverse hierarchical organizations during metastatic progression. In primary CRCs, proliferative Lgr5-neg tumor cells gave rise to Lgr5+ cells and HRCs (Fig. 2i-l). In macrometastases, the apex of the hierarchy was occupied by proliferative Lgr5+ ISC-like cells, which generated HRCs over time (Fig. 2q-t). In contrast, the algorithm prognosticated that the cells that initiate the cellular hierarchy of micrometastases corresponded to undifferentiated HRCs in cluster 5 (Fig. 2m-p and Extended Data Fig 4e). This cell population gave rise to Lgr5+ ISC-like and proliferative tumor cell progeny (Fig. 2m-p), which are abundant in small metastases (Fig. 2h and Extended Data Fig. 4d).

We made equivalent observations in AKP CRCs (Extended Data Fig. 4f-p). Abundant HRCs characterized liver AKP lesions profiled at early time points (day 35 post-implantation), whereas micrometastases collected later (day 70) contained a larger proportion of Lgr5+ cells (Extended Data Fig. 4f-k). Micrometastatic Lgr5+ cells in the AKP model expressed high levels of the signature of latent Mex3a+ cells (Extended Data Fig. 4l-n), which is in agreement with our previous study on the specification of this population in triple mutant CRCs[16]. CellRank also predicted HRCs as the origin of micrometastasis in the AKP CRCs (Extended Data Fig. 4o,p).

Characterization of HRC features

A comparison of human and mouse scRNAseq datasets revealed that a large subset of core genes were expressed consistently by HRCs of the two species (Extended Data Fig. 5a). Among them, we focused on EMP1 because it was expressed at high levels in HRCs and exhibited a large degree of overlap with the expression of the EpiHR geneset in both human (Extended Data Fig. 5b-c) and AKTP mouse CRCs (Fig. 3a and Extended Data Fig. 5d). Echoing our results with the EpiHR signature, CellRank predicted that the cell origin of metastatic relapse in AKTP (Extended Data Fig. 5e-g) and AKP (Extended Data Fig. 5h,i) tumors expressed elevated *Emp1* levels.

We thus leveraged *Emp1* to track HRCs during disease relapse. To this end, we knocked-in an inducible-Caspase9-tdTomato (iCT) cassette[2] into the *Emp1* locus of AKTP MTOs using CRISPR-Cas9 (Fig. 3b). Inspection of knock-in MTOs revealed high tdTomato (TOM) expression in a subset of tumor cells (Fig. 3b). We next inoculated Emp1-iCT AKTP MTOs into the caecum of c57BL/6 mice. TOM expression in dissociated epithelial tumor cells measured by flow cytometry revealed heterogeneity in *Emp1* expression (Extended Data Fig. 5j). TOM-high cells purified by Fluorescence-activated single cell sorting (FACS) showed large upregulation of *Emp1* expression (Extended Data Fig. 5k). In contrast, TOM-low cells were characterized by expression of intestinal stem cell (ISC)-specific genes such as *Lgr5* and *Smoc2* (Extended Data Fig. 5k). Gene expression profiling confirmed elevated levels of the EpiHR and coreHRC signatures in Emp1-TOM-high cells, whereas the WNT/Lgr5+ ISC program was downregulated in these cells (Fig. 3c,d and Extended Data Fig. 5l).

Inspection of tissue sections evidenced that cancer cells invading the muscular layer were strongly labeled by the fluorescent reporter (Fig. 3e). In particular, isolated tumor buds and larger clusters in contact with the stroma at the edges on invasion fronts exhibited the highest TOM expression (Inset in Fig. 3e, Extended Data Fig. 6a-d). Emp1-TOM^{high} cell clusters were often found in proximity to peripheral blood vessels in mouse primary CRCs, suggesting a connection with hematogenous or lymphatic dissemination (Extended Data Fig. 6e). CRCs generated by implantation of AKP MTOs in the caecum also exhibited Emp1-TOM^{high} invasion fronts and tumor buds that overexpressed HRC-marker genes (Extended Data Fig. 6f-h).

We also examined livers of mice bearing AKTP tumors at various time points post-orthotopic MTO implantation. In samples collected at early time points, micrometastatic lesions trapped within portal veins and liver sinusoids were populated entirely by Emp1-TOM^{high} cells (Fig. 3f-g and Extended Data Fig. 6i-j). Fitting the scRNAseq analyses, we identified two Emp1-TOM+ subsets; one expressed KRT20 and was located mainly in the tumor cores, whereas the other was positioned at invasion fronts, lacked KRT20 and expressed the highest levels of TOM reporter (Extended Data Fig. 6k). Liver Emp1-TOM^{high} micrometastases were also KRT20 negative (Extended Data Fig. 6l). Of note, Emp1-TOM^{high} tumor buds and micrometastases were labeled with EPCAM and E-CADHERIN, implying that they retained an epithelial organization (Extended Data Fig. 6 and Extended Data Fig. 7a). Analysis of epithelial-to-mesenchymal transition (EMT) master transcription factors showed equivalent expression levels in Emp1-TOM^{high} and Emp1-TOM^{low} cells (Extended Data Fig. 7b). scRNAseq also supported the lack of canonical EMT

markers in human and mouse HRCs (Extended Data Fig. 7c-d). However, we noticed that the coreHRC signature genes *LAMA3, LAMC2, ITGA2* and *PLAUR* belong to a recently characterized partial EMT gene expression module[17]. HRCs in mouse primary CRCs and micrometastases upregulated this partial EMT signature (Extended Data Fig. 4e). Of note, LAMC2 has been previously identified as a specific marker for tumor budding in multiple tumor types, including CRC[18], and we confirmed that its expression correlated with *EMP1* mRNA in CRC patient samples (Extended Data Fig. 7e,g). KRT17, a widely used marker of the basal pancreatic cancer subtype[19], also marked EMP1^{high} invasion fronts and tumor buds (Extended Data Fig. 7f,g). Gene Set Enrichment Analysis (GSEA) also revealed homophilic cell adhesion and apical junctions as central features of HRCs (Fig. 1l). Fittingly, *EMP1* encodes a component of the tight junctions[20,21] and there were multiple other constituents of adherent junction, tight junction and desmosome complexes upregulated in the core HRC program such as *PCDH1, DSC2, CLND4* and *JUP* (Extended Data Fig. 7g and Supplementary Table 2). The latter encodes Plakoglobin which mediates circulation of tumor cells as clusters and confers enhanced metastatic capacity to breast cancer cells [22].

To further explore the relationship between *Emp1* and *Lgr5* expression, we engineered AKTP MTOs bearing both Emp1-iCT and Lgr5-EGFP knock-in reporter cassettes (Extended Data Fig. 8a-f). Confocal imaging of dual labeled MTOs showed a mutually exclusive pattern of expression of the two reporters (Extended Data Fig. 8a) and RT-qPCR analysis confirmed upregulation of *Emp1* and *Lgr5* in sorted TOM+ and EGFP+ cells, respectively (Extended data Fig. 8b). Primary CRCs generated from inoculation of dual labeled MTOs in the caecum also exhibited a mutually exclusive expression pattern of Emp1-TOM and Lgr5-EGFP reporters (Fig. 3h and Extended Data Fig. 8c-d). Emp1-TOM^{high} cells were largely enriched at tumor buds which, in contrast, contained few Lgr5-EGFP+ cells (Fig. 3i). RNA fluorescence in situ hybridization (FISH) analysis on human CRC patient samples also showed that *EMP1* expression was elevated at tumor invasion fronts, whereas *LGR5* marked the tumor cores in most cases (examples in Extended Data Fig. 8g-p).

Finally, we analyzed the livers of mice bearing primary CRCs. Lgr5-EGFP fluorescence was absent in disseminated tumor cells (DTCs) and micrometastases, but was progressively gained during metastatic outgrowth, in a marked antithetic pattern to Emp1-TOM expression (Fig. 3j-k and Extended data Fig. 8e-f). Together with CellRank bioinformatic predictions, these observations suggest that HRCs are endowed with the ability to migrate and disseminate to

foreign organs where they initiate metastatic outgrowth and subsequently give rise to non-HRC populations.

Determinants of the HRC population

The transcription factor YAP opposes the activity of the WNT pathway[23,24] and a YAP-driven gene program has been associated with tumor cell plasticity[25,26], regeneration[27] and metastasis formation in CRC[28]. In addition, YAP promotes the conversion of LGR5+ CRCs cells to a fetal intestine-like progenitor state during chemotherapy[16,25,29,30]. Extended Data Fig. 9a,b shows the expression of the top 50 upregulated fetal intestine progenitor genes from Mustata et al.[31], and the broadly used YAP_22 target gene signature[32]. As a reference, we also show the expression of the Top 50 HRC core genes (Extended Data Fig. 9c). Only 3 out of 22 YAP core signature genes were upregulated in HRCs of CRC patients, whereas most bonafide YAP target genes, including *CTGF* and *CYR61*, were not (Extended Data Fig. 9a). HRCs only upregulated a few canonical markers of the regenerative fetal-like intestinal population, including *ANXA1* or *TACSTD2*, but the majority of them were either not expressed or expressed at low levels by HRCs (Extended Data Fig. 9b). We also found little overlap between the coreHRC and YAP_22 or fetal intestinal progenitor signatures (Extended Data Fig. 9d). Emp1-TOM+ cells isolated from mouse primary CRCs were neither enriched in YAP target genes (Extended Data Fig. 9e-f). Therefore, there are only marginal similarities between YAP+/fetal intestinal progenitors and the HRC state. To obtain further insights into this question, we knocked down YAP levels using shRNAs in MTOs or blocked YAP-driven transcription by over-expressing an inducible dominant-negative TEAD transcription factor that inhibits both YAP and TAZ activity[33] (Extended Data Fig. 9g-m). These genetic manipulations decreased the levels of the canonical YAP-target genes *Ctgf* and *Cyr61*, but did not affect the expression of core HRC genes *Emp1* or *Lamc2* in MTOs, neither the abundance of Emp1-Tom-high cells (Extended Data Fig. 9g-m). As a control for these experiments, we used chemotherapy (Folfiri), which upregulated the YAP_22 signature, including *Ctgf* and *Cyr61*, and the intestinal fetal progenitor program but did not induce the HRC state (Extended Data Fig. 9n,o).

Association analyses in the TGCA COAD cohort revealed a strong correlation between activating KRAS mutations and EpiHR expression levels (Fig. 3l). Using a series of mouse organoids with compound mutations in main CRC driver genes engineered by means of CRISPR/Cas9 (CRISPR Tumor Organoids or CTOs), we confirmed that genotypes containing KRAS G12D mutations exhibited upregulation of coreHRC and EpiHR gene signatures (Fig. 3m and Extended Data Fig.

10a,b). We next searched for associations between HRCs and TME composition using the scRNAseq CRC patient dataset. This analysis exposed a direct correlation between CAFs and HRCs abundance (Fig. 3n). Consistent with this finding, α-SMA+ CAFs surrounded Emp1-TOM^{high} cells in primary AKTP CRCs (Fig. 3o). Co-culture of AKTP MTOs with CAFs augmented 6-fold Emp1-TOM+ cell numbers (Fig. 3p), but YAP knockdown failed to block this effect (Extended Data Fig. 10c). Expression profiling of MTO cells isolated from co-cultures demonstrated upregulation of EpiHR, coreHRC and basal pancreatic cancer signature levels (Fig. 3q-r and Extended Data Fig. 10d). Furthermore, it was evident an arrangement of tumor cell types reminiscent of the *in vivo* organization; Lgr5-EGFP cells were positioned at the organoid center, whereas Emp1-TOM+ cells relocated to the boundaries in contact with the fibroblast population (Fig. 3s and Extended Data Fig. 10e). Fibroblasts surrounding MTOs appeared activated, as shown by the expression of α-SMA (Extended Data Fig. 10f). We also noticed that some organoids gained expression of the pancreatic cancer basal cell marker KRT17 (Extended Data Fig. 10g).

Relapse by residual EMP1+ cells

We next leveraged the inducible Caspase9 cassette inserted in the *Emp1* locus to perform cell ablation experiments in intact tumors (Fig. 4a,b)[2,34]. Inoculation of mice with AP20187 dimerized the chimeric Caspase9 expressed under the *Emp1* locus and specifically killed cells expressing the highest levels of Emp1-TOM reporter (Fig. 4c-e). This effect was reversible, shown by a slow but progressive recovery of the Emp1-TOM-high cell population at invasion fronts upon AP20187 dimerizer (DIM) treatment cessation (Extended Data Fig. 10h-j). DIM treatment was only administered during primary tumor growth but was ceased the day before primary CRC resection (Fig. 4b). Macrometastases were not yet present when DIM treatment finished; therefore, this experimental setting aimed at ablating Emp1^{high} cells in the primary tumor and possibly in incipient metastatic lesions. Remarkably, while DIM treatment did not affect primary tumor growth (Fig. 4f), the majority of mice showed no signs of liver and lung metastatic recurrence and were disease-free at experimental endpoints (Fig. 4g,h and Extended Data Fig. 10k). We made equivalent observations in AKTP CRC growing in nude mice, thus ruling out that genetic cell ablation strategy impaired metastatic progression through activation of the adaptive immune system (Extended Data Fig. 10l-n). When Emp1^{high} cell ablation started one week after primary CRC resection, we observed no changes in metastatic progression and all mice suffered metastatic relapse (Fig. 4i,j). Emp1^{high} cell ablation neither halted metastasis formation when MTOs were directly inoculated in the liver through the spleen (Fig. 4k,l). Reinforcing this observation, we found that intrasplenic

inoculation of $Emp1^{high}$ cells isolated from MTOs generated more and larger liver metastases than either $Lgr5^{high}$ cells or $Emp1^{low}/Lgr5^{low}$ cells but the difference was not substantial, suggesting that all these tumor cell populations hold metastasis initiating capacity in this assay (Extended Data Fig. 10o-r). Moreover, metastases produced by these three cell populations contained the other tumor cell types owing to extensive cell plasticity (Extended Data Fig. 10s). Overall, these data demonstrate that $Emp1^{high}$ HRCs drive metastatic relapse after primary CRC resection, yet they are dispensable after metastatic seeding is completed. This model is further supported by the observation that HRC ablation just after surgery and during metastatic outgrowth decreased the number of small size metastasis very significantly, but did not modify the frequency of large metastases (Extended Data Fig. 10t-w).

Despite the slow-growing metastatic lesions generated by the AKP CRCs, HRC ablation before primary tumor surgery also prevented metastatic recurrence in these triple mutant models (Extended Data Fig. 11a-c). In addition, we demonstrated that HRCs mediated recurrence in CRCs bearing Smad4 mutations (AKPS) (Extended Data Fig. 11d-f). To extend our observations beyond the colon-to-liver metastasis axis, we implanted AKTP MTOs in the rectum, which generated invasive rectal cancers that, as in humans, metastasized preferentially to the lungs (Extended Data Fig. 11g-k). Inspection of lung metastases revealed that, akin to liver metastases, micrometastases were mostly $Emp1^{high}$, whereas larger metastases decreased the percentage of $Emp1^{high}$ cells (Extended Data Fig. 11i). Although we could not surgically resect mouse rectal tumors, ablation of $Emp1^{high}$ cells caused a 20-fold reduction in lung metastasis burden in this model (Extended Data Fig. 11k).

Using a diphtheria toxin receptor (DTR)-based ablation strategy in CRC models, it was previously shown that Lgr5+ CRC cells are necessary for liver metastasis formation[3]. To assess the role of Lgr5+ tumor cells in our relapse models, we knocked-in a DTR cassette into the *Lgr5* locus of AKTP MTOs (Extended Data Fig. 11l-m). Inoculation of this MTO line into the caecum further validated our previous observations that invasion fronts, tumor buds and micrometastases seldom contain Lgr5+ cells (Extended Data Fig. 11n-q). More importantly, treatment with Diphtheria toxin (DT) before surgical removal of the primary CRC effectively eliminated Lgr5+ cells (Fig. 4m-p), yet it did not prevent disease relapse and mice developed overt liver metastatic disease (Fig. 4q-s). Thus, Lgr5+ cells are dispensable for dissemination and metastatic colonization. We validated these results using an independent AKTP MTO line engineered with an iCaspase9-tdTomato (iCT) cassette knocked-in in the *Lgr5* locus (Extended Data Fig. 11r-t). Again, effective ablation

of Lgr5+ cells in the iCT model (Extended Data Fig. 11u-w) neither altered CRC tumor growth (Extended Data Fig. 11x) nor prevented metastatic recurrence (Extended Data Fig. 11y). On the other hand, ablation of Lgr5+ cells after direct inoculation of MTOs in the liver through the portal vein halted metastasis formation (Fig. 4t, u), further supporting a requirement for Lgr5+ cells during metastatic outgrowth[3].

Neoadjuvant immunotherapy avoids relapse

We previously showed that mouse primary AKTP CRCs are models of microsatellite stable (MSS)/mismatch repair proficient CRCs characterized by a relatively low mutational burden, abundant stroma cells and T cell exclusion[12]. Transplantation of AKTP MTOs in caecum also gave rise to T-cell excluded CRCs (Extended Data Fig. 12a). CAFs surrounded *Emp1*-expressing invasion fronts and tumor buds, but T cells did not reach these structures and remained at the tumor periphery (Extended Data Fig. 12b). In contrast, liver micrometastases generated by these primary CRCs exhibited high CD3+ cell density (Fig. 5a). The lack of T cell exclusion was evident and we could visualize T cell-HRC interactions in these early lesions (Fig. 5b). However, metastatic outgrowth was accompanied by a gradual decline of CD3+ cells and the relocalization of T cells to the periphery (Fig. 5c,d). Multiplex immunohistochemistry showed that most infiltrating T cells were CD4+/FOXP3- (Examples in Fig. 5e and quantification in Fig. 5g and Extended Data Fig. 12c). T cell exclusion coincided with progressive CAFs (α-SMA+ and/or POSTN+) and Macrophage (CD68+) recruitment to the metastatic TME (Fig. 5f,g and Extended Data Fig. 12c).

scRNASeq revealed elevated expression of interferon-alpha and -gamma target genes in undifferentiated (Krt20-) HRCs (Fig. 5h and Supplementary Table 4), >95% of which belong to micrometastatic lesions, suggestive of an ongoing inflammatory response as HRCs reach the liver. Indeed, undifferentiated HRCs upregulate multiple interferon response genes compared to the other tumor cell populations in primary CRCs and large metastases (Extended Data Fig. 12d). It was also apparent that HRCs in micrometastases expressed elevated *Cd274* (that encodes PD-L1) and *Ido1* levels, two negative regulators of the immune response (Extended Data Fig. 12d). Flow cytometry confirmed elevated PD-L1 expression at the surface of micrometastatic CRC cells and a progressive decline over subsequent outgrowth (Fig. 5i,j). Based on these findings, we hypothesized that at the onset of organ colonization, lack of a mature TME exposes HRCs to the adaptive immune system, yet disseminated cells bypass immune attack through the expression of immune-modulatory molecules. To test the susceptibility of micrometastatic disease to immunotherapy, we treated mice with anti-PD1 in combination with anti-CTLA4 antibodies in the

neoadjuvant setting, i.e., before the primary tumor was extirpated by surgery (Fig. 5k-p). This approach increased CD8+ cytotoxic T cell numbers in the primary CRC (Fig. 5l,m) but we did not observe alteration of the tumor growth rate (Extended Data Fig. 12e) or curative effects on the primary disease at experimental endpoints (Fig. 5n). Yet, remarkably, most of these mice did not develop metastasis after surgical removal of the primary CRC and remained disease-free at experimental endpoints (Fig. 5o,p). We obtained similar results with neoadjuvant anti-PD1 monotherapy (Extended Data Fig. 12f-i). In contrast, the same regime applied two weeks after surgery (i.e., late anti-PD1/CTLA4 immunotherapy) did not stop metastatic outgrowth (Fig. 5q-s), which is in line with the failure of checkpoint immunotherapy observed in patients with metastatic MSS CRC[35–37]. Hence, during a temporal window after metastatic colonization, immunotherapy is effective in eliminating the residual disease and preventing subsequent metastatic relapse.

DISCUSSION

HRCs represent a defined cell state in a large proportion of patient samples implying a common origin and mechanism of metastatic recurrence across CRC genotypes and molecular subtypes. Pioneering work by de Sauvage and colleagues revealed that Lgr5+ cancer stem cells are dispensable for primary CRC growth yet necessary for metastasis formation in experimental models[3]. Subsequently, Van Rheenen and colleagues proposed that metastases are initiated by disseminated differentiated tumor cells that, through plasticity, produce Lgr5+ cancer stem cells upon reaching the liver[38]. Massagué and colleagues also provided evidence that expression of the adhesion molecule L1CAM in LGR5+ and LGR5- cells is important for the regenerative burst that follows metastatic colonization[39]. We unequivocally show that HRCs are a subset of LGR5 negative cells yet they are neither differentiated nor stem-like but rather co-opt a distinct state that enables migration and colonization of foreign organs. KRAS mutations, cross-talk with stromal fibroblasts and other environmental stimuli such as hypoxia directly instruct HRCs at invasion fronts. The finding that HRCs are enriched in tumor buds strengthens the well-established association of these anatomic structures with poor prognosis[40,41]. In addition, a defining feature of the HRC state is the upregulation of genes encoding cell-to-cell adhesion molecules. We therefore speculate that HRCs may extravasate as cell clusters and colonize foreign organs as oligocellular structures rather than as single cells as previously shown for other cancer types[22,42]. Our data fit with a model whereby HRCs disseminate out of the primary tumor prior to surgical resection and the HRC state is subsequently retained in residual tumor cells lodged in foreign organs. Reacquisition of the Lgr5+ stem cell and proliferation programs occur at a later phase and is necessary for metastatic outgrowth (Fig. 5t). Ablation of HRCs in primary tumors prevents

the vast majority of metastatic relapses. Yet, it is formally possible that other tumor cell populations, including Lgr5+ cells, may generate metastasis if they reach foreign organs, as suggested by experiments of direct inoculation of tumor cells into the blood stream performed herein and elsewhere[3,28,38,39]. Our data also indicate that YAP activity is not required for the specification of HRCs in primary CRCs, although do not rule out a role for YAP during the metastatic cascade as previously proposed[28].

Immune-checkpoint therapy does not exert therapeutic benefits in MSS/mismatch repair-proficient overtly metastatic CRCs[35–37]. Besides the lower neoantigen burden of MSS CRCs, data in experimental models and patient samples indicate that the TME of MSS CRCs excludes and limits the activity of T-cells[12,43]. Our findings reveal that residual metastatic cells lodged in foreign organs lack a mature TME and are susceptible to the attack of the adaptive immune system upon immunotherapy treatment. This window of vulnerability could be exploited to prevent metastatic relapse. Our results back up current efforts to use neoadjuvant immunotherapy in early-stage CRC patients[44]. This and other therapeutic strategies capable of eliminating HRCs may prevent disease relapse if applied before metastatic disease is overt. The CRC relapse mouse model described herein may serve as a powerful pre-clinical platform for testing such therapies.

DATA AVAILABILITY

All data relevant to this study are available from the corresponding author upon reasonable request. Expression arrays and RNA-seq data are available at Gene Expression Omnibus (GEO). The accession number for gene expression sequencing experiments reported in this paper are GEO: GSE190055 (Arrays Emp1-high vs Emp1-low AKTP tumor cells), GSE208139 (Arrays MTOs co-cultured with fibroblasts), GSE207974 (RNAseq chemotherapy) and GSE207668 (RNAseq CTOs). Count matrices for single cell RNAseq experiments were deposited in ArrayExpress under accession number E-MTAB-11284 (10X AKTP primary tumors) E-MTAB-11302 (Smart-seq metastatic progression) and E-MTAB-11981 (Smart-seq AKP micromets). Additional metadata and processed data files, including UMAP embeddings and gene signature scores, are available at Synapse (syn35000645). Source data are provided with this paper.

ACKNOWLEDGEMENTS

We thank all members of the laboratory for their support and discussions. We are grateful for the outstanding assistance by IRB Barcelona core facilities for biostatistics, histopathology, functional genomics and advanced digital microscopy, as well as the flow cytometry, animal facilities of the UB/PCB, and the CRG genomic unit. We are indebted to the HCB-IDIBAPS Biobank for sample and data procurement. Sample collection of this work was also supported by the Xarxa de Bancs de Tumors de Catalunya (XBTC) sponsored by Pla Director d'Oncologia (PDO). AC-S, GT and LJ-G have held FPU fellowships from Spanish Ministry of Universities and AA-V a La Caixa predoctoral fellowship. SC holds a FPI fellowship from Spanish Ministry of Economy and Competitiveness (MINECO). HH is a Miguel Servet (CP14/00229) researcher funded by the Spanish Institute of Health Carlos III (ISCIII) and the Agencia Estatal de Investigación (AEI) and FEDER (SAF2017-89109-P). This work has been supported by ERC advanced grants 884623 (residualCRC to EB) and 883739 to (Epifold to XT); LCF/PR/HR19/52160018 from La Caixa foundation; PID2020-119917RB-I00 from Spanish MICINN; and CRUK Accelerator Award C7932/A26825 (ACRCelerate), in collaboration with AECC (grant GEACC19006BAT_2021). EB, XT and HH are supported by the Fundació La Marató de TV3 (201903-30-31-32). HH received support for the project PID2020-115439GB-I00- funded by MCINN/AEI/10.13039/501100011033. SL was supported by a Wellcome Trust (Senior Clinical Research Fellowship (206314/Z/17/Z)), EJM is funded by the Lee Placito Medical Research Fund (University of Oxford). IRB Barcelona and IBEC are recipients of a Severo Ochoa Award of Excellence from MINECO. Single cell profiling of CRC samples was supported by the Belgian Federation against Cancer grant nos. 2018-127 and 2016-133 and by a grant from Fondation Roi-Baudouin. S.T. is supported by a

Fundamental Clinical Researchers KU Leuven grant and Foundation against Cancer grant for this work. Dedicated to Magdalena Socias Moyà, mother of Adrià Cañellas-Socias, who during her cancer illness hiked 110 km to raise funds for metastasis research at IRB Barcelona.

AUTHOR CONTRIBUTIONS

AC-S designed and performed key experiments including the profiling of residual disease, the analyses of tumor buds and micrometastases, genetic ablation studies and immunotherapy experiments in the CRC relapse model. AC-S, CC, GT, FS and DS generated and characterized MTO knock-in lines. AC-S, XH-M, SP-P and GT developed the CRC relapse model. AC-S, XH-M and SP-P performed all mice work. LM and CS-OA analyzed scRNAseq data. CS-OA and AB-LL analyzed human CRC transcriptomic datasets. AC-M, LM and CS-OA performed statistical analyses. CC and TS performed IF and imaged organoids in vitro. AC-S, CC and OR generated and characterized YAP KD models. AC-S, CC, OR, SC and XT performed in vitro co-cultures. AC-S and AA-V quantified immunofluorescence stainings. AC-S and NF developed the method to purify residual tumor cells in whole livers. MS performed IF and IHC. EM and SL performed multiplex IF. LN performed chemotherapy experiments. LB and JC performed 3D lightsheet imaging. LJ-G, CC, PL and HH provided support with scRNAseq experiments. ST generated single cell RNA sequencing data from CRC patient samples. DVFT and DS generated MTO and CTO biobanks. ES provided strategic support and helped with figures and manuscript writing. EB supervised the study.

COMPETING INTEREST

The authors declare no competing financial interests.

ADDITIONAL INFORMATION

Correspondence and requests for materials should be addressed to Eduard Batlle (eduard.batlle@irbbarcelona.org).

FIGURE LEGENDS.

Fig. 1 | Identification of poor prognosis epithelial CRC cells

a, Identification of TME-HR and EpiHR signatures in a metacohort of 7 pooled human stage I-III CRC datasets (n= 1830 patients, Supplementary Table 1). **b-d,** UMAP layout of whole tumors (stroma + epithelium cells) belonging to the SMC dataset, colored by AllHR **(b)**, TME-HR **(c)** and epithelial-specific HR genes **(d)**. **e,** Kaplan-Meier survival curves indicating relapse-free survival. Two-sided Wald test. Likelihood Ratio Test p-values (LRT-pv) are specified. **f,g**, UMAP layout of 14674 CRC tumor cells (SMC cohort) colored by patient ID **(f)** and expression of the EpiHR signature **(g)**. **h,** Barplot quantifying the sub-population composition of each patient in the SMC (left) and KUL (right) datasets. Patient ID is detailed. Patients with low WNT signature scores are marked with an "*". **i,** Selected Hallmarks, GOSLIM and KEGG gene signatures enriched in HRCs compared to the rest of tumor cells in human and mouse CRC samples. **j,** Boxplot representing the proportion of HRCs in each clinical stage. Box plots have whiskers of maximum 1.5 times the interquartile range. Boxes represent first, second (median) and third quartiles. n= 3, 7, 14, 3, patients from left to right. Two-sided Kruskal-Wallis test. **k,** UMAP of tumor cells colored by expression of the LGR5 signature. **l,** UMAP of tumor cells labelled according to their classification as HRCs, LGR5+, double positive or other cells. **m-o,** Primary tumors were generated in the caecum of c57BL/6J mice by injecting syngeneic AKTP MTOs. UMAPs depicting GFP+ tumor cells dissociated from primary tumors colored by expression levels of (**m**) EpiHR signature, (**n**) Lgr5 signature and (**o**) their classification as HRCs, Lgr5+, double positive or other cells.

Fig. 2 | Spatiotemporal dynamics of CRC metastases resolved by scRNAseq

a, Schematic representation of the mouse model of CRC metastatic relapse developed herein. **b,** Percentage of metastatic recurrence depending on time to primary tumor resection. Number of mice are detailed above the barplot. P-value for generalized linear model. **c,** Intravital bioluminescence imaging (BLI) quantification (photons s^{-1}) of a representative experiment. Grey points and lines represent bioluminescence in the lower thorax of individual mice and purple points in the liver. Representative images of bioluminescence in the same mouse before, after surgery and upon liver metastases formation are shown. **d,** Representative images of whole livers containing GFP-expressing tumor cells obtained using lightsheet microscopy. Scale bars, left image (300 µm on Maximum Intensity Projections (MIP, and selected single plane insets 50 µm); right (100 µm on MIP and single plane insets 50 µm). **e,** Illustration of the longitudinal single cell RNA-expression analysis of tumor cells along the metastatic cascade. **f, g,** UMAP layout of 900 tumor cells isolated from 7 different mice colored by (**f**) metastatic stage and (**g**) Seurat clusters.

h, Barplot showing Seurat cluster composition by sample stage. **i, m, q,** Vector fields representing RNA velocity projected on UMAPs of primary tumors (**i**), micro + small metastases (**m**) and macrometastases (**q**). Colored by the pseudotime estimated for each cell with scVelo. **j, n, r,** UMAPs with cells separated in primary tumors (**j**), micro+small metastases (**n**) and macrometastases (**r**) and colored by gene expression of mKi67, Lgr5 and EpiHR gene signatures. **k, o, s,** Schematics showing distinct hierarchical behavior during the different stages of metastasis formation. **l, p, t,** Smoothed mKi67, Lgr5 and EpiHR gene signature expression trends fitted with Generalized Additive Models as a function of pseudotime in primary tumors (**l**), micro+small (**p**) and large metastases (**t**).

Fig. 3 | Emp1 marks cells enriched in invasion fronts and micrometastases.

a, UMAP depicting EpiHR and *Emp1* levels. **b,** TOM and E-CADH immunostaining in Emp1-iCasp9-tdTomato AKTP MTOs. Scale bar, 50 µm. **c-d,** Expression (normalized intensity) of EpiHR (**c**) and Lgr5 (**d**) signatures in Emp1-high and Emp1-low cells 4 weeks post-implantation. n=4 mice per condition. ROAST-GSA adjusted p-values. **e-h,** Representative images of Emp1-TOM+ and/or Lgr5-GFP+ cells in primary CRCs **(e,h)** and liver metastases **(f,g).** Dashed lines in panels **e,h** encompass tumor buds and invasion fronts or label the portal vein in **g**. NM: Normal Mucosa; ML: Muscle Layer. Scale bars, 500 µm (e,g, h); 100 µm (e, inset); 50 µm (f). **i,** Percentage of Lgr5-GFP^{high} and Emp1-TOM^{high} cells in different CRC regions. Whiskers are maximum and minimum values; n= 855,330 cells from 18 mice. Two-sided Paired Wilcoxon test. **j,** Examples of liver metastases of increasing size. Scale bars, 50 µm (micro and small), 250 µm (medium), 500 µm (macro). **k,** Percentage of Emp1-TOM^{high} and Lgr5-GFP^{high} cells per metastasis size. n= 318276 cells from 137 liver metastases from 17 different mice. LOESS model with a 95% confidence interval. **l,** EpiHR levels versus driver mutations. Difference of medians (x axis) versus p-value of Wilcoxon test (y axis) are shown. **m,** Normalized intensity of EpiHR signature expression in CTOs of different genotypes. n= 6 (WT) and 5 (Kras G12D) CTOs; 2 technical replicates per genotype. P-value for two-sided T-test. **n,** Association between HRC frequency and TME cell populations in SMC patients. Pearson correlation coefficients and p-values are shown. **o,** Representative patterns of TOM, α-SMA and EPCAM in primary CRCs. Dashed lines delimit the caecum. Scale bar: 250 µm; 125 µm (inset). **p,** Percentage of Emp1-TOM^{high} cells (mean ± SD) in the indicated conditions; n=17 (control), 4 (hypoxia) and 8 (fibroblast co-culture). Two-sided Wilcoxon test. **q-r,** Expression (normalized intensity) of EpiHR or basal-like signatures. n=4 biological replicates. ROAST-GSA adjusted p-values. **s,** Representative confocal images of AKTP MTOs co-cultured with colon fibroblasts. Scale bar, 50 µm. Boxes in panels c, d, i, m, q and r indicate first, second

(median) and third quartiles. Whiskers in panels m,q and r indicate maximum of 1.5 times the interquartile range.

Fig. 4 | Emp1-high cells are the origin of metastatic relapse.

a, Emp1-iCaspase9-mediated cell ablation. **b,** Experimental timeline detailing inducible Emp1+ cell ablation and surgery. **c,** Emp1-TOM+ cells in primary tumors after ablation. Lines mark the caecum borders. Scale bars, 500 µm (C), 250 µm (C'). **d,** Representative flow cytometry plot of TOM fluorescence in EPCAM+ cells. **e,** Percentage of Emp1-TOM^{high} tumor cells (mean ± SD) at the time of surgery. Every dot is a mouse, n=4 in both groups. Two-sided Wilcoxon test. **f,** Primary tumor area (mean ± SD) after resection. Each dot is a mouse, n= 33 (control) and 22 (DIM) mice. P-value for linear model after boxcox transformation. **g,** Liver metastases (mean ± SD) at experimental endpoints. Each dot is a mouse, n as in panel **f**. **h,** Percentage of mice that relapse with liver metastases. Two-sided fisher test. **i,** Experimental timeline detailing late ablation of Emp1+ cells and metastatic growth by BLI monitoring. n=9 mice per each group. **j,** Percentage of mice that relapse with liver metastases. Two-sided fisher test. **k,** Metastatic growth by BLI monitoring upon ablation of Emp1 cells 3 days after intrasplenic inoculation of Emp1-iCasp9-Tom organoids, n= 3 mice per each group. **l,** Number of liver metastases in **k**. Mean ± SD. n= 3 mice per group. **m,** Experimental timeline. **n,** Representative stainings of Lgr5-DTR-EGFP+ cells in primary CRCs. Dashed lines outline the serosa. Scale bars, 100 µm. **o,** Representative flow cytometry plot of Lgr5-EGFP fluorescence. **p,** Percentage of Lgr5-GFP^{high} tumor cells (mean ± SD). n=3 (Cont.) and 5 (DT) mice respectively. P-value for generalized linear model**. q,** Primary tumor area (mean ± SD) measured after resection. n=20 mice in control and 16 in DT. Two-sided Wilcoxon test. **r,** Liver metastases (mean ± SD) at experimental endpoints. Each dot is a mouse, n as in (**r**). **s,** Percentage of mice that relapse with liver metastases. Two-sided fisher test. **t,** Liver metastasis growth monitored by BLI after intrasplenic inoculation of MTOs. **u,** Number of liver metastases (Mean ± SD) in (**t**). n=7 mice in control and n=8 in DT treated in (**t,u**). Points and lines of BLI measurements in panels **i, k** and **t,** represent individual mice, trend lines (bold) show a LOESS model and P-values were calculated with mixed effects linear model. P-values comparing in panels **g, r, l** and **u** were calculated using generalized linear model.

Fig. 5 | Neoadjuvant immunotherapy prevents metastatic relapse in CRC.

a-c, Representative images of T cell distribution (CD3+) in liver micrometastasis (**a,b**) and macrometastases (**c**) present in the AKTP CRC relapse model. Scale bars, 100 µm (a), 50 µm (b) and 500 µm (c). **d**, Percentage of CD3+ T Cells (referred to total TME cells) versus metastases

size. n=12 mice, 133 metastases. Linear model with mouse as fixed effect. **e-f,** Representative examples of multiplex immunofluorescence of immune (**e**) and stromal (**f**) markers in metastases. Scale bars, 100 μm (micro and macro), 500 μm (small). **g**, Proportional stacked area graph showing TME cell types in metastasis of different sizes; n= 132 metastasis of 20 mice. **h,** Heatmap showing Hallmark GSEA in cell populations described in Fig. 2g. **i,** Representative flow cytometry contour plot showing PD-L1 expression in tumor cells of micro- and macro-metastases. **j,** Percentage of PD-L1+ tumor cells by flow cytometry. Whiskers encompass the smallest and largest value. Boxes represent first, second (median) and third quartiles. n= 3 (primary), 6 (micro), 4 (small and medium), 2 (macro). Wilcoxon t-test. **k**, Experimental timeline for neoadjuvant immunotherapy treatment. **l-m,** CD3+ (**l**) and GZMB+ (**m**) cell densities in primary CRCs (means ± SD); n= 19 (control), 10 (neoadjuvant). Mixed effects linear model. **n**, Primary CRC area (mean ± SD) after resection. Each dot is a mouse; n= 18 (control), 10 (treated). P-value for linear model. **o**, Liver metastases (mean ± SD) at experimental endpoints. Each dot is a mouse; n= 17 (control), 9 (treated). P-value for generalized linear model. **p**, Percentage of mice that developed liver metastases at experimental endpoints; n as in **o**. P-value for generalized linear model. **q**, Experimental timeline for late immunotherapy. **r**, Liver metastasis growth measured by normalized BLI. Points and lines represent individual mice, trend lines show a LOESS model. n= 4 (control), 5 (late immunotherapy). Analyzed with a linear model. **s,** Liver metastases (mean ± SD) generated by AKTP primary tumors. Dots are an individual mouse. n= 7 (control), 6 (late immunotherapy). P-value for generalized linear model. **t,** Proposed model for metastatic dissemination of CRC and TME co-evolution.

REFERENCES

1. *AJCC Cancer Staging Manual*. *AJCC Cancer Staging Manual* (2017) doi:10.1007/978-3-319-40618-3.
2. Shimokawa, M. *et al.* Visualization and targeting of LGR5 + human colon cancer stem cells. *Nature* **545**, 187–192 (2017).
3. de Sousa e Melo, F. *et al.* A distinct role for Lgr5+ stem cells in primary and metastatic colon cancer. *Nature* **543**, 676–680 (2017).
4. Cortina, C. *et al.* A genome editing approach to study cancer stem cells in human tumors. *EMBO Mol. Med.* **9**, 869–879 (2017).
5. Calon, A. *et al.* Dependency of Colorectal Cancer on a TGF-β-Driven Program in Stromal Cells for Metastasis Initiation. *Cancer Cell* **22**, 571–584 (2012).
6. Calon, A. *et al.* Stromal gene expression defines poor-prognosis subtypes in colorectal cancer. *Nat. Genet.* **47**, 320–329 (2015).
7. Isella, C. *et al.* Stromal contribution to the colorectal cancer transcriptome. *Nat. Genet.* **47**, 312–9 (2015).
8. Lee, H.-O. *et al.* Lineage-dependent gene expression programs influence the immune landscape of colorectal cancer. *Nat. Genet.* **52**, 594–603 (2020).
9. Guinney, J. *et al.* The consensus molecular subtypes of colorectal cancer. *Nat. Med.* **21**, 1350–1356 (2015).
10. Raghavan, S. *et al.* Microenvironment drives cell state, plasticity, and drug response in pancreatic cancer. *Cell* **184**, 6119-6137.e26 (2021).
11. Joanito, I. *et al.* Single-cell and bulk transcriptome sequencing identifies two epithelial tumor cell states and refines the consensus molecular classification of colorectal cancer. *Nat. Genet.* **54**, 963–975 (2022).
12. Tauriello, D. V. F. *et al.* TGFβ drives immune evasion in genetically reconstituted colon cancer metastasis. *Nature* **554**, 538–543 (2018).
13. Massagué, J. & Obenauf, A. C. Metastatic colonization by circulating tumour cells. *Nature* **529**, 298–306 (2016).
14. Barriga, F. M. *et al.* Mex3a Marks a Slowly Dividing Subpopulation of Lgr5+ Intestinal Stem Cells. *Cell Stem Cell* **20**, 801-816.e7 (2017).
15. Lange, M. *et al.* CellRank for directed single-cell fate mapping. *Nat. Methods 2022 192* **19**, 159–170 (2022).
16. Álvarez-Varela, A. *et al.* Mex3a marks drug-tolerant persister colorectal cancer cells that

mediate relapse after chemotherapy. *Nat. Cancer 2022* (2022) doi:10.1038/S43018-022-00402-0.

17. Tyler, M. & Tirosh, I. Decoupling epithelial-mesenchymal transitions from stromal profiles by integrative expression analysis. *Nat. Commun.* **12**, 1–13 (2021).
18. Grigore, A. D., Jolly, M. K., Jia, D., Farach-Carson, M. C. & Levine, H. Tumor budding: The name is EMT. partial EMT. *Journal of Clinical Medicine* vol. 5 51 (2016).
19. Roa-Peña, L. *et al.* Keratin 17 identifies the most lethal molecular subtype of pancreatic cancer. *Sci. Rep.* **9**, 11239 (2019).
20. Durgan, J. *et al.* SOS1 and Ras regulate epithelial tight junction formation in the human airway through EMP1. *EMBO Rep.* **16**, 87–96 (2015).
21. Bangsow, T. *et al.* The epithelial membrane protein 1 is a novel tight junction protein of the blood-brain barrier. *J. Cereb. Blood Flow Metab.* **28**, 1249–1260 (2008).
22. Aceto, N. *et al.* Circulating tumor cell clusters are oligoclonal precursors of breast cancer metastasis. *Cell* **158**, 1110–1122 (2014).
23. Barry, E. R. *et al.* Restriction of intestinal stem cell expansion and the regenerative response by YAP. *Nature* **493**, 106–110 (2013).
24. Cheung, P. *et al.* Regenerative Reprogramming of the Intestinal Stem Cell State via Hippo Signaling Suppresses Metastatic Colorectal Cancer. *Cell Stem Cell* **27**, 590-604.e9 (2020).
25. Vasquez, E. G. *et al.* Dynamic and adaptive cancer stem cell population admixture in colorectal neoplasia. *Cell Stem Cell* **29**, 1213-1228.e8 (2022).
26. Han, T. *et al.* Lineage Reversion Drives WNT Independence in Intestinal Cancer. *Cancer Discov.* **10**, 1590–1609 (2020).
27. Lupo, B. *et al.* Colorectal cancer residual disease at maximal response to EGFR blockade displays a druggable Paneth cell–like phenotype. *Sci. Transl. Med.* **12**, :eaax8313 (2020).
28. Heinz, M. C. *et al.* Liver colonization by colorectal cancer metastases requires YAP-controlled plasticity at the micrometastatic stageCellular determinants of metastatic outgrowth. *Cancer Res.* 1–16 (2022) doi:10.1158/0008-5472.CAN-21-0933.
29. Solé, L. *et al.* p53 wild-type colorectal cancer cells that express a fetal gene signature are associated with metastasis and poor prognosis. *Nat. Commun.* **13**, 2866 (2022).
30. Ohta, Y. *et al.* Cell-matrix interface regulates dormancy in human colon cancer stem cells. *Nature* **680**, 784–794 (2022).
31. Mustata, R. C. *et al.* Identification of Lgr5-Independent Spheroid-Generating Progenitors

of the Mouse Fetal Intestinal Epithelium. *Cell Rep.* **5**, 421–432 (2013).

32. Wang, Y. *et al.* Comprehensive Molecular Characterization of the Hippo Signaling Pathway in Cancer. *Cell Rep.* **25**, 1304-1317.e5 (2018).
33. Yuan, Y. *et al.* YAP1/TAZ-TEAD transcriptional networks maintain skin homeostasis by regulating cell proliferation and limiting KLF4 activity. *Nat. Commun.* **11**, 1472 (2020).
34. Morral, C. *et al.* Zonation of Ribosomal DNA Transcription Defines a Stem Cell Hierarchy in Colorectal Cancer. *Cell Stem Cell* **26**, 845-861.e12 (2020).
35. Brahmer, J. R. *et al.* Safety and Activity of Anti–PD-L1 Antibody in Patients with Advanced Cancer. *N. Engl. J. Med.* **366**, 2455–2465 (2012).
36. Le, D. T. *et al.* PD-1 Blockade in Tumors with Mismatch-Repair Deficiency. *N. Engl. J. Med.* **372**, 2509–2520 (2015).
37. Topalian, S. L. *et al.* Safety, Activity, and Immune Correlates of Anti–PD-1 Antibody in Cancer. *N. Engl. J. Med.* **366**, 2443–2454 (2012).
38. Fumagalli, A. *et al.* Plasticity of Lgr5-Negative Cancer Cells Drives Metastasis in Colorectal Cancer. *Cell Stem Cell* **26**, 569-578.e7 (2020).
39. Ganesh, K. *et al.* L1CAM defines the regenerative origin of metastasis-initiating cells in colorectal cancer. *Nat. cancer* **1**, 28–45 (2020).
40. van Wyk, H. C. *et al.* The Relationship Between Tumor Budding, Tumor Microenvironment, and Survival in Patients with Primary Operable Colorectal Cancer. *Ann. Surg. Oncol.* **26**, 4397–4404 (2019).
41. Lugli, A. *et al.* Recommendations for reporting tumor budding in colorectal cancer based on the International Tumor Budding Consensus Conference (ITBCC) 2016. *Mod. Pathol.* **30**, 1299–1311 (2017).
42. Padmanaban, V. *et al.* E-cadherin is required for metastasis in multiple models of breast cancer. *Nat. 2019 5737774* **573**, 439–444 (2019).
43. Mlecnik, B. *et al.* The tumor microenvironment and Immunoscore are critical determinants of dissemination to distant metastasis. *Sci. Transl. Med.* **8**, 327ra26 (2016).
44. Chalabi, M. *et al.* Neoadjuvant immunotherapy leads to pathological responses in MMR-proficient and MMR-deficient early-stage colon cancers. *Nat. Med.* **26**, 566–576 (2020).

METHODS

MTOs and integration of cassettes using CRISPR

We previously described[12] the establishment of MTOs from primary tumors arising in GEMMs with compound genetic alterations (Apc, K-ras, Tp53, Tgfbr2). MTOs were cultured as detailed by Tauriello et al.[12] and checked bimonthly for mycoplasma contamination. 20 bp small guide RNAs (sgRNA) were designed to cut 9-11 base pairs after the STOP codons using the http://crispr.mit.edu web tool and were cloned into pX330-IRFP hSp-enhanced-Cas9 plasmid[4]. The sgRNA sequence for Emp1 was "AAATAAGCCGAATACGCTCA" and for Lgr5 "GTCTCTAGTGACTATGAGAG". 1kb 5' and 3' homology arms were sequentially cloned into pShuttle vectors containing the inducible Caspase9-tdTomato cassette[34], a kind gift from Toshiro Sato. IRES-DTR-T2A-EGFP-WPRE-BGHpA sequence was cloned between Lgr5 homology arms flanking the gene stop codon. EGFP was cloned after the IRES sequence to generate the LGR5-IRES-EGFP-WPRE-BGHpA donor. CRISPR-Cas9 knock-in editing was carried out as described previously[4]. AKTP-MTO#93 Emp1-iCT was generated from a single tdTomato+ cell (clone#14). MTO#93 Emp1-iCT Lgr5-EGFP was first generated from a single tdTomato+ cell (clone#49), then nucleofected with the Lgr5-EGFP construct and stablished from a pool of sorted EGFP+ cells. MTO#93 Lgr5-iCT was generated by a single tdTomato+ cell (clone#2). MTO#93 Lgr5-DTR-EGFP was generated by sorting a pool of EGFP+ cells. Triple mutant AKP MTO#54[12] Emp1-iCT was generated by sorting a pool of tdTomato+ cells. CRISPR-Cas9 gene editing was subsequently used to introduce a Smad4 mutation in AKP Emp1-iCT to generate AKPS MTOs as previously described[45]. Correct integration of the knockin cassettes was checked by PCR and sequencing of the genomic regions. Correct expression of the reporter cassette was tested by RT-qPCR or by sorting cells from tumors as described in the text. Genotyping primers are detailed in Supplementary Table 5.

Generation of CRISPR-derived Tumor Organoids (CTOs)

To study the effect of gene driver mutations on the HRC phenotype, we sequentially introduced mutations in Apc, Kras, Tp53, Tgfbr2 and/or Smad4 in normal mucosa colonic organoids using CRISPR-Cas9 as described elsewhere[16,45,46]. Compound mutations were introduced in organoids derived from one c57BL/6J mouse, which allowed us to assess the impact of individual mutations on gene expression without other confounding variables. We named these organoids CRISPR-derived Tumor Organoids (CTOs) to distinguish them from Mouse Tumor Organoids (MTOs), that were stablished from GEMMs.

Lentivirus production and MTO infection

For bioluminescent tracking, MTOs were infected with a lentivirus encoding an EGFP-firefly luciferase fusion reporter construct under the control of the PGK promoter. For YAP inhibition experiments, AKTP organoids were infected with shControl (SHC002) or shYAP1 (TRCN0000095864, TRCN0000095865, TRCN0000095866, TRCN0000095867) Mission Sigma-Aldrich lentiviral constructs. We also use a pInducer20 EGFP-TEADi vector, a gift from Ramiro Iglesias-Bartolome (Addgene plasmid # 140145 ; http://n2t.net/addgene:140145 ; RRID:Addgene_140145), that blocks the activity of both YAP and TAZ[33].

Animal experimentation approval and maintenance

Experiments with mice were approved by the Animal Care and Use Committee of Barcelona Science Park under protocol CEEA-PCB-14-000053. Mice were maintained in a specific-pathogen-free (SPF) facility with a 12-h light–dark cycle, under controlled temperature and humidity (18-23°C and 40-60% respectively) and given ad libitum access to standard diet and water. All mice were closely monitored by authors, facility technicians and by an external veterinary scientist responsible for animal welfare. Authors monitored primary tumor and metastasis growth using intravital bioluminescence at least once a week.

Inoculation of MTOs into the caecum and rectum

For all injections, c57BL/6J mice were purchased from Janvier Labs at six weeks of age and injected at 7 to 9 weeks of age. Sex always matched the origin of the tumor. Intra-caecum injections were used for the generation of primary tumors. Organoids were harvested and incubated for 30 minutes with cold HBSS to break down BME (Bio-Techne, 3533-010-02), without disrupting their structure. Cells were then counted and resuspended in 70% BME in HBSS for injection at a concentration of 0.1 X 10^6 cells in 10 µl per mouse. Full organoids were injected with a 30G syringe into the submucosal wall of the distal caecum while looking through binocular lens. We introduced a significant modification to previous protocols[47] by moving the injection site to the apex of the caecum, which allowed posterior surgical resection. For liver colonization studies we used intrasplenic injections of organoids as described before[12]. Rectal injections were performed following a previously described procedure[48]. Maximum tumor volume of 300mm^3 allowed by the animal experimentation committee was never exceeded in these experiments except in experiments shown in Extended Data Fig 11j, where 1 mouse out of 19 developed larger tumor due to very fast tumor growth between the last measurement and the day of sacrifice.

Primary tumor resection

Mice were anesthetized with isoflurane and placed in dorsal recumbency. The abdomen was shaved and sterilized with povidone-iodine surgical solution. A small midline incision - slightly to the left- was performed to open the skin and peritoneum and expose the abdominal area. We placed a sterile surgical drape on top of the abdomen with a circular hole above the incision and sprawled the caecum over the drape using cotton swabs and saline to keep it hydrated. After confirming the presence of a primary tumor, Kelly forceps were used to first knot the surgical suture into the caecum wall, in between the ileocecal junction and the primary tumor. This provided a grip for subsequent caecum ligation. After ligation, the apical caecum containing the primary tumor was excised and any remaining caecal tissue was trimmed. After resection and organ fixation, we measured primary tumor size using a caliper. We provide a video of the surgery, which usually lasted from 5 to 10 minutes (Supplementary video 1). Fitness of mice was monitored weekly throughout the experiment. Mice were euthanized four weeks after resection and metastasis were scored macroscopically.

Pharmacological treatments

For iCasp9-inducible ablation experiments, animals were treated with dimerizer (AP20187, Medchem express, HY-13992) via intraperitoneal injection at 2.5 mg/kg 3 times per week (Emp1-iCasp9) or 5 mg/kg every day (Lgr5-iCasp9). For DTR-inducible ablation mice were treated with 16.7 µg/kg of diphtheria toxin (Sigma-Aldrich, 322326) three times per week. For immunotherapy experiments, 2 shots of 250 µg of anti-mouse CTLA-4 (C2444, Leinco) and/or anti-mouse PD-1 (P372, Leinco) were administered via intraperitoneal injection 5 days apart.

Tumor dissociation for flow cytometry

Primary tumors and micro-dissected liver metastases were chopped with razor blades. Subsequent enzymatic digestion was performed with 200 U/ml collagenase IV (Sigma Aldrich, C5138) in HBSS (Lenovo) for 30 min at 37 °C, in a shaking water bath or a gentleMACS Dissociator (Miltenyi Biotec). Digested tissue fragments were then filtered through 100- and 40-µm meshes, washed, and treated for 5 minutes with ammonium chloride. Single-cell preparations were first blocked with anti-CD16/32 (clone 93; eBioscience) and then stained with APC anti-EPCAM (Biolegend, 118214) and BV605 anti-CD45 (Biolegend, 103155) antibodies at 1:200 concentration. In experiments measuring PD-L1 expression in tumor cells, cells were stained with FITC anti-EPCAM (Santa Cruz, clone G8.8, 53532) and APC anti-PDL1 (BD, 564715, clone MIH5) at 1:200 concentration. Finally, cells were resuspended in HBSS with 0.5% FBS and DAPI (Sigma Aldrich, D9542).

Collection of primary CRC and metastasis samples

Metastatic seeding in the CRC relapse models is not synchronized; as it occurs in patients, tumor cells are shed from the primary tumor continuously during weeks until the day of resection. As a result, metastases expand for different periods in the liver and lung, and exhibit different sizes at experimental timepoints depending on when HRCs colonized the organ. We thus decided to classify according to size instead to time. Four different CRC samples were collected; Primary, Micrometastasis, Small metastasis and Macrometastases. Micrometastases and small metastases were collected from liver of mice 29 to 31 days post primary tumor implantations (i.e. at the time of primary CRC resection). Micrometastases samples were DTCs collected from livers with absent or residual bioluminescence ex vivo in which metastases were not visible. For small metastases, metastatic nodules were visible but small in size (<1.5mm). Macrometastases samples were derived from metastatic nodules larger than 4mm in livers from mice 64 to 66 days after primary tumor implantation. Primary tumor samples were paired with micro, small or macrometastases samples and were collected from both timepoints (29 to 31 and 64 to 66 days post primary tumor implantation).

Isolation of liver residual DTCs

For isolation of low numbers of tumor cells from mice without visible metastases, whole livers were thoroughly minced with razor blades. After an initial 30-minute digestion with 200 U/ml collagenase IV (Sigma-Aldrich, C5138), samples were filtered through 100 µm meshes. Although most cells flowed through, a small fraction of the sample –highly enriched for tumor cells- was retained in the filter (Extended data Fig. 3k,l). Filters were then laid into a 6-well plate and covered with HBSS containing 200 U/ml collagenase IV, 200 µg/ml Dispase II (Sigma-Aldrich, D4693), 40 µg/ml DNase I (Sigma-Aldrich, 10104159001) and 13 µM rock inhibitor (Medchem Express, HY-10583). After re-digestion in a water bath for 30 minutes al 37 °C, most cells now seeped through the filters. The protocol continued with washing, ammonium chloride treatment and antibody staining as described above. DAPI- EGFP+ CD45- cells were gated to sort tumor cells.

Histology and tissue staining

Standard hematoxylin/eosin (HE) and antibody staining were performed on 4-µm tissue sections using standard procedures, as described previously[12]. Details can be found in Supplementary Table 6. The following secondary antibodies were used: donkey anti-goat conjugated to Alexa 488/568/647 (Life Technologies A11055, A11057, A21447), donkey anti-rabbit conjugated to Alexa 488/568/647 (Life Technologies A21206, A10042, A31573) and donkey anti-mouse conjugated to Alexa 488/568/647 (Life Technologies A-21202, A10037, A31571) at RT. Digital

scanned bright-field and fluorescent images were acquired with a NanoZoomer-2.0 HT C9600 scanner (Hamamatsu, Photonics, France). All images were visualized with the NDP.view 2 U123888-01 software (Hamamatsu, Photonics, France) with a gamma correction set at 1.8 in the image control panel.

Co-culture of MTOs and colon fibroblasts

To address the role of fibroblasts in inducing HRCs, we seeded 5000 AKTP Emp1-iCT MTO cells with or without 100k mouse colon fibroblasts in BME (Bio-Techne, 3533-010-02) and supplemented them with MTO cell culture media[12] without noggin nor galunisertib. Co-cultures were analyzed 7 days after by flow cytometry and confocal microscopy. For Fig. 3s and Extended Data Fig. 10e, MTOs and mouse colon fibroblasts were also co-cultured in hanging drops as previously described[49].

In Situ Hybridization on CRC patient samples

Paraffin-embedded tissue sections (2-3 µm in thickness) of human CRC primary tumors were air dried and further dried at 60 ºC over-night prior any staining. To compare *EMP1* and *LGR5* expression, sections were hybridized with RNAscope® Probe Hs-LGR5 (ref: 311021, Bio-Techne R&D Systems) in C1 channel, a custom-made RNAscope® Probe Hs-EMP1 (, Bio-Techne R&D Systems, 895051-C2) in C2 channel and an Alexa568-conjugated antibody against E-CADHERIN. To compare the expression of *EMP1* with other HRC-marker genes, sections were hybridized with RNAscope® Probe Hs-EMP1 mRNA in channel 1 (Bio-Techne R&D Systems, 895051-C1) and stained with the relevant antibodies. FISH probe were detected using the RNAscope® Multiplex Fluorescent Detection Reagents Kit v2 (Bio-Techne R&D Systems, 323110).

Multiplex immunofluorescence

Multiplex immunofluorescence staining was performed on 4-µm-thick formalin-fixed paraffin embedded sections using the OPAL protocol (Akoya Biosciences, Marlborough, MA) on the Leica BOND RXm autostainer (Leica Microsystems, Wetzlar, Germany). Six consecutive staining cycles were performed using the following primary antibody-Opal fluorophore pairings. Stroma panel: CD34 (1:3000, ab81289; Abcam)–Opal 520; CD146 (1:500, ab75769; Abcam)–Opal 570; α-SMA (1:1000, ab5694; Abcam)–Opal 620; PERIOSTIN (1:1000; Abcam, ab227049)–Opal 690; and E-E-CADHERIN (1:500; Cell Signaling, 3195)–Opal 650. Immune panel: (1) LY6G (1:300; BD Pharmingen, 551459)–Opal 540; (2) CD4 (1:500; Abcam, ab183685)–Opal 520; (3) CD8 (1:800; Cell Signaling, 98941)– Opal 570; (4) CD68 (1:1200, Abcam, ab125212)–Opal 620; (5) FOXP3

(1:400; Cell Signaling, 126553)–Opal 650; and (6) E-CADHERIN (1:500; Cell Signaling, 3195)–Opal 690. Primary antibodies were incubated for 60 minutes and detected using the BOND Polymer Refine Detection System (Leica Biosystems, Buffalo Grove, IL, DS9800) according to the manufacturer's instructions, substituting DAB for the Opal fluorophores, with a 10 minute incubation time and without adding hematoxylin. Whole-slide scans and multispectral images (MSI) were obtained on the Akoya Biosciences Vectra Polaris. Batch analysis of the MSIs from each case was performed with the inForm 2.4.8 software provided. Finally, batched analyzed MSIs were fused in HALO (Indica Labs, Albuquerque, NM) to produce a spectrally unmixed reconstructed whole-tissue image, ready for analysis.

Statistical analyses multiplex immunofluorescence

HALO ® IMAGE ANALYSIS PLATFORM software was used for quantification of cell phenotypes within metastases and primary tumors. Two matrices of counts (distinguishing immune and stromal panels) with the total number of cells per metastasis/primary (in rows) assigned to each cell population (in columns) were obtained. Multiple positives were present in both immune and stromal panels. These cases observed in the immune panel only represented the 0.7% of the total assignations and were removed from the analysis. For the stromal panel, aα-SMA and POSTN double positives were kept and labeled in a distinct category aα-SMA/POSTN. The rest of multiple positives, that accumulated the 7% of the total cells, were amalgamated and labeled as "stromal others". For all metastases, information regarding metastasis size (nº of total cells in log2 scale), metastatic burden (defining micro, small and big metastases) and organ site were considered for subsequent analysis.

For the proportional stacked area graph, only the percentages of CD4, FOXP3, CD8, LY6G and CD68 for the immune panel, and the percentages of CD146, CD34, POSTN, α-SMA and α-SMA/POSTN for the stromal panel were considered. The cell composition of all measured metastases (in percentages) was averaged out using the R function aggregate, taking as grouping elements both metastasis size and metastatic burden. Linear mixed effects models were fitted independently for every cell population using the CLR-transformed values as response variable, the metastatic burden and the metastasis size as fixed effects, and the tumor Id (the tumor identifier) as random effect to consider the dependence between metastases for the same tumor.

Quantification of tdTomato and EGFP fluorescence intensity

For the quantification of percentages of Emp1-tdTomato high and Lgr5-EGFP-high in tumor sections we used HALO® IMAGE ANALYSIS PLATFORM. Briefly, the epithelial tumor area was

classified apart from the stroma, background and necrosis using a random forest algorithm. Single cells were detected and tdTomato/EGFP fluorescence intensity was measured for every cell. In primary tumors, Tomato-high and EGFP-high tumor cells were defined as the cells in the 90th percentile of each sample. In liver metastases, Tomato-high and EGFP-high tumors cells were defined as the cells in the 90th percentile for all metastases measured. For Emp1-iCT liver metastases (in Extended Data Fig. 6j) and Lgr5-DTR-EGFP liver metastases (in Extended Data Fig. 11q), tumor cell fluorescence-intensity was analyzed using ImageJ with a custom-made macro. Tumor cells were first detected and isolated using E-CADHERIN to create a mask. TdTomato or EGFP intensity was calculated for every pixel inside the masked area. Then, we plotted the percentage of fluorescence high and low pixels as a function of the area of the metastases (measured in pixels).

Gene expression analysis by RT-qPCR

RT-qPCR and Microarrays were used to compare subpopulations of Emp1-high and -low, Lgr5-high and -low cells dissociated from MTO#93 organoids grown *in vitro* or *in vivo*. Subpopulations were defined in flow cytometry as the top/bottom 10% in fluorescence expression. RNA from 2000 cells was extracted and retrotranscribed to cDNA as described previously[50]. To analyze gene expression changes RT-qPCR was performed using 5 ng of cDNA per each real-time qPCR well. Real-time qPCRs were performed with TaqMan Universal PCR Master Mix (Applied Biosystems, 4369016) or PowerUp SYBR Green Master Mix (Applied Biosystems, 100029284) in triplicates, following manufacturer's instructions. Gene expression levels were normalized using the housekeeping genes *PPIA* or *B2M*. The following TaqMan assays were used: TdTomato-BGHPA (custom made probe; F: GGGCATGGCACCGGCAGCACC, R: CCTACTTGTACAGCTCGTCCATGCC), MmPPIA (Mm002342430_g1), MmB2m (Mm00437762_m1), EGFP (Mr04097229mr), MmEmp1 (Mm00515678_m1), MmLgr5 (Mm0043889_m1), MmSmoc2 (Mm00491553_m1), MmLamC2 (Mm00500494), Ctgf (Mm01192033_g1). The following Sybr primers were used:_MmYAP (F: ACCCTCGTTTTGCCATGAAC, R: TGTGCTGGGATTGATATTCCGTA), MmCyr61 (F: AGGTCTGCGCTAAACAACTCA, R: ATATTCACAGGGTCTGCCTTCT).

Western blotting

Cells were harvested in Cell Recovery Solution (Corning, 354257), pelleted and lysed in RIPA buffer (Tris-HCl pH 7.4, 150 mM NaCl, 0.5% Sodium deoxycolate, 0.1% SDS, 1 mM EDTA, 10 mM NaF, 1 mM PMSF, 1% Triton X-100) supplemented with protease and phosphatase inhibitors (Sigma Aldrich). Primary antibodies we incubated overnight at 4ºC at the following dilutions:

YAP/TAZ (Cell Signalling, CS93622) 1:1000, TAZ (Cell Signalling, 72804), 1:20000 vinculin (Sigma Aldrich, 9264). Anti-Rabbit IgG HRP (NA934V) and anti-mouse IgG HRP (NXA931V) secondary antibodies were diluted 1:5000 and incubated for 1h at RT with the PVDF membranes. Membranes were visualized using Hyper Processor (Amersham Pharmacia Biotech).

RNA sequencing and analysis

We used RNA sequencing to profile CTOs with different genotypes and to profile the effects of chemotherapy treatment on MTOs. For the first dataset, 5,000 cells from CTOs with multiple genotypes (AT, AP, AS, AK, ATP, APS, AKP, AKS, AKPT, AKPS) were seeded in BME (Bio-Techne, 3533-010-02) and cultured in full stem cell medium[4]. Organoids were harvested 7-10 days after seeding. For chemotherapy experiments, MTO54 organoids were dissociated with TryplE express (, Thermo Fisher, 12604039) and single cells were resuspended in BME in 6 well plates in complete stem cell medium. Two days later, the media was removed and fresh media containing Folfiri (5FU at 50 µg/µl (Sigma-Aldrich, F6627) plus SN38 at 100 nM (MedChem, HY-13704)) or control (MTO stem cell media[12]) was added for two days.

RNA was extracted and sequenced as we previously described[16]. RNAseq reads from datasets (CTOs or chemotherapy treatment) were aligned with STAR (v2.5.2)[51] with default parameters to the Mus musculus reference genome built with annotations version GENCODE_mmusculus_vM25. SAM files were converted to BAM and sorted using Sambamba (v0.7.1)[52]. Count matrices were generated using the R (v4.0.5) package Rsubread (v2.4.3)[53] with the GENCODE_mmusculus_vM25 custom annotation. Data from parental versus metastatic MTOs was processed as described in Tauriello et al[12]. Genewise differential expression in the chemotherapy dataset between controls and Folfiri treatment was performed using the R package DESeq2 (v1.30.1)[54]. Normalized values for plots were obtained via the *rlog* function of the same package. Signature scores were defined as the scaled mean of all genes in the signature after scaling the expression matrix. Comparison of signature scores between conditions was assessed using a t-test.

Microarray expression analyses

Microarrays GeneChip Mapping 250K Nsp Assay Kit, Affymetrix) were used to compare Emp1-high and Emp1-low cells from MTO#93 Emp1-iCT primary tumors grown for 4 weeks; and to compare MTO#93 Emp1-iCT MTOs grown in vitro alone or in combination with mouse colon fibroblasts for 7 days. Samples were processed with oligo v1.46.0[55] (fitProbeLevelModel:

background = TRUE, normalize = TRUE, target = "core", method = "plm"). Raw cel files were normalized with RMA method[56] (default paramaters). Probesets were annotated with Clariom_S_Mouse_HT-na36-mm10-transcript Affymetrix databases. Standard quality controls were considered to identify abnormal samples[57]. No samples were excluded due to quality issues.

Differential expression analysis of Emp1-high vs -low data was performed using a linear model with empirical shrinkage (limma R package)[58], taking into account the paired data setting. Differential expression analysis of co-culture data was performed using the same regression method, but considering as adjustment variable the Eklund metric pm.iqr[59] to reduce the influence of technical variation. Benjamini- Hochberg FDR was used for multiple comparisons correction. Gene set analysis was used to explore the enrichment in custom gene sets. The limma's rotation-based approach for enrichment[60] was considered to represent the null distribution. The maxmean enrichment statistic proposed in[61], under restandardization, was used for competitive testing. Gene signatures (Supplementary Table 7) z-scores[62] were used to measure pathway activity. For doing so, normalized expression was adjusted for biological replicate, centered and scaled gene-wise according to the mean and the standard deviation computed across samples. Gene signature z-score was summarized by taking the average of its constituent genes. In addition, a global signature was computed using all genes and used for a priori centering of signature scores. This strategy has been proved to be useful to avoid systematic biases. Only the expression of the most variable probe sets per gene were considered for gene set analyses.

10X mouse single cell RNA sequencing analysis

CellRanger[63] (v4.0.0) was used to align reads to a custom refdata-gex-mm10-2020-A transcriptome including the EGFP and Luciferase genes. Gene expression was analysed with Seurat (v4.0.3)[64–67]. A total of 1,330 cells having <20% mitochondrial content and >3,000 detected genes were considered. Ribosomal reads (17% of the total) were removed. Mitochondrial content was regressed out during SCT normalization[68].

SCT transformed counts were smoothed with MAGIC (v.2.0.3)[69]. Gene signature expression was summarized by taking the average MAGIC score of its constituent genes. HRCs and Lgr5+ cell populations were defined by having a score above the 75th percentile. FindAllMarkers was used to identify differentially expressed genes from raw counts. The HRCs cell population was compared to the rest in order to identify mice HRC markers. Testing was limited to genes detected in >10% of cells and showing >0.25 log-fold difference. Functional enrichment analysis was

performed using the GSEApreranked[70] method ranking genes by the log2 average fold change. The significance threshold was set at 5% Benjamini-Hochberg FDR.

To select for marker genes to track HRCs in mouse tumors, we computed the correlation scores for all genes with the EpiHR signature in the SMC human dataset and the mouse 10X dataset. We used MAGIC score for genes and signature scores as defined previously.

Mouse SMART-seq_v2 single cell RNA sequencing analysis

Smart-seq2 reads were aligned to the UCSC_GRCm38.mm10 genome with zUMIs[71] and analyzed with Seurat[64–67] (v4.0.3). Four technical batches of AKTP at different stages were merged into a single object and two technical batches of AKP micrometastases were merged into another object. A total of 1,057 cells having <20% mitochondrial content and >20,000 UMIs were considered for AKTP, whereas 414 cells with <20% mitochondrial content and >100,000 UMIs were isolated from AKP micrometastases. Ribosomal reads were removed. Mitochondrial content was regressed out during SCT normalization. SCT transformed counts were further imputed and smoothed with MAGIC (v.2.0.3)[69]. Gene signature expression (Supplementary Table 7) was summarized by taking the average MAGIC score of its constituent genes. Non-epithelial cells were removed and normalized again. In order to improve the integration of the four AKTP batches, the IntegrateData function was used with pre-computed anchors based on 3,000 features. The integrated dataset was SCT normalized, smoothed with MAGIC (v.2.0.3)[69], and clustered with FindClusters Seurat function (resolution = 1.2). FindMarkers was used to identify differentially expressed genes from raw counts. CellRank[15] were used to uncover the cell-state dynamics of CRC metastasis from RNA velocity estimates[72,73]. Gene expression or signature expression was represented as a function of latent time with R[74]. Additionally, the integration, normalization, imputation, and trajectory analysis were performed independently for the subset of cells harvested from primary tumors, incipient metastasis, and macrometastases.

Creation of CRC transcriptomic Meta-cohort

Public CRC transcriptomic datasets were downloaded from GEO[75] and NCI GDC commons[76], pre-processed and homogenized into a unique Meta-cohort including 1830 samples from: TCGA[77], GSE38832[78], GSE44076[79], GSE33113[80], GSE14333[81], GSE39582[82] and GSE37892[83] (Supplementary Table 1). The last four datasets include disease-free survival information with a median follow-up of 3.7 years and clinical information across all datasets is gender, age, stage and location of primary tumor (Supplementary Table 1). When not available, MSI status was imputed using the transcriptomic signature reported in[84] through density-based non-parametric

clustering[85,86]. Signature scores were computed as the scaled mean of the genes in the signature after scaling the expression matrix.

Microarray data were processed separately using RMA[57]. Information about sample processing and hybridization was retrieved from the CEL files. For TCGA, the Legacy version was used for expression with clinical annotation from October 2016. Genes were annotated using Ensembl Biomart database (GRCh37)[87,88]. Duplicated samples across platforms were removed from the GA dataset, as well as samples from other locations than colon or rectum. RSEM[89] expressions were already log2-transformed and quantile normalized. Samples TCGA-A6-2679-01A and TCGA-AA-A004-01A were excluded due to an anomalous expression distribution among all samples.

Each microarray series was corrected by Eklund metrics[59], center and scanning date using a mixed-effect linear model[90]. Age, gender, stage, site and MSI were also included in these models. TCGA sets were corrected for occurrences of combinations of center and plate identifier (random effect). For microarrays, probesets were summarized at the gene-level using the first principal component from probesets in that gene. This component was then centered and scaled to the weighted mean of the means and standard deviations of the probesets. The sign of the component was changed to the sign of the probeset contributing the most. All datasets were merged after genewise standardization to the GSE39582 series according to the distribution of gender, age, MSI and stage using undersampling: a sub-sample of the same number of patients and the same distribution according to these clinical variables was selected from the GSE39582 series for each dataset. Expression values were truncated to the maximum and minimum values observed in the reference dataset.

Gene screening for association with relapse

Each gene in the Meta-cohort was evaluated for linear association of its expression with recurrence using a frailty Cox proportional hazards model[91,92], including dataset and technical variables. Statistical significance was assessed by means of a Wald test. Hazard Ratios (HR) and their corresponding 95% confidence intervals were computed as a measure of association. The 2530 genes with $HR>1$ and $p<0.05$ were included in the all_HR signature.

We used the GSE39397 dataset[6] which includes expression profiles of epithelial cancer cells (EPCAM+), CAFs (FAP+), leukocytes (CD45+) and endothelial cells (CD31+) isolated by FACs

from dissociated primary CRCs (n=14), to classify genes according to their expressions in these populations. The EpiHR signature contains genes from the allHR signature which are upregulated (Fold change >1; p<0,05) in EPCAM+ cells compared to the three TME populations (FAP+, CD31+ and CD45+). AllHR genes that did not pass this cut-off comprised the TME-HR signature.

Signature scores were computed as the scaled mean of the genes in the signature after scaling the expression matrix. The association with recurrence of the EpiHR signature in the whole dataset as well as the subclasses of CMS samples was assessed as described above. The likelihood ratio test comparing EpiHR and AllHR p-value was computed with the *drop1* R function. Kaplan Meier plots were generated using the *survfit* and *plot* functions.

Association between clinical variables and the EpiHR signature in the CRC metacohort was assessed by fitting a linear model for each variable of interest independently. Technical factors (dataset and center, as described in extended methods) were included as covariates.

Association between oncogenic alterations and the EpiHR score

Annotations of oncogenic alterations for the TCGA samples were downloaded from [93]. A Wilcoxon test comparing the expression of mutated vs wild-type samples was performed independently for every alteration. The difference of expression medians was used as a measure of the impact of each mutation in the gene expression.

Patient 10X single-cell analysis

Count matrices were downloaded from arrayExpress (E-MTAB-8107 for samples EXT001, EXT002, EXT003, EXT009, EXT010, EXT011, EXT012, EXT013, EXT014, EXT018, EXT019, EXT020, EXT021, EXT022, EXT023, EXT024, EXT025, EXT026, EXT027, EXT028) and GEO (GSE132465 for all SMC..-T samples)[8]. The remaining EXT samples were processed as referred in E-MTAB-8107 and deposited in ArrayExpress under accession number E-MTAB-9934. Cells with mitochondrial content higher than 20%, less than 1000 counts, more than 6000 or less than 200 genes were discarded. Ribosomal genes were also removed from the matrix to avoid technical biases during normalization. Samples with less than 500 cells and not classified as core tumor were discarded from further analyses. The Korea (SMC samples) and Leuven (EXT samples) cohorts were processed independently following the R package Seurat V3 recommendations[67]: samples were combined and normalized using the SCTtransform function regressing mitochondrial percentage, with the method "glmGamPoi", min.cells=1 and return.only.genes=FALSE in order to keep the maximum number of genes. Dimensionality

reduction and visualization were performed using RunPCA and RunUMAP, with 26 principal components. Expression was imputed and smoothened using the MAGIC algorithm[69]. The expression of the EPCAM gene was used to define the connected components corresponding to epithelial cells.

The epithelial component of each cohort was processed as follows: cells with less than 1000/3000 (SMC/KUL) counts and less than 200/1250 genes detected were removed from the dataset. No further normalization was applied. RunPCA, RunUMAP, FindNeighbors and FindClusters were applied, with 5/7 principal components and resolution of 0.7. Expression was imputed and smoothened using the MAGIC algorithm. Signature scores were computed as the mean value of the MAGIC expression per cell for all genes in the signature. The EpiHR and Lgr5 cell populations were defined as cells with the corresponding signature expression above the 75 percentile. Population markers were found using *FindMarkers*. Functional enrichment was computed through the Gene Set Enrichment Analysis implementation in[94] with genes ordered by fold change. Samples in the SMC and KUL datasets were annotated according to their iCMS class[11].

To identify the core gene expression program upregulated in HRCs, we computed the correlation scores for all genes with the EpiHR signature in the SMC and KUL human dataset. The resulting list was ranked by the average correlation across all samples (Supplementary Table 2). The coreHRC signature was defined as the top 100 genes in the SMC dataset.

Association of HRCs and tumor microenvironment populations

We classified clusters according to the expression of known markers of microenvironment components[8]. For each population we estimated the association between its percentage per sample and the percentage of HRCs. Spearman correlation coefficient and p-value were used to assess said association.

Clustering of EpiHR genes according to expression correlation

We computed the Pearson correlation coefficient for all pairwise combinations of genes in the EpiHR signature. We then applied hierarchical clustering (*hclus* in R with method "complete") and defined 6 clusters via the *cutree* function. Upon visual inspection we decided to merge the three clusters with higher correlation, resulting in Cluster 1 (Extended Data Fig. 1h).

Ethics oversight

Samples of primary CRC from patients used for IF and ISH analysis were obtained from the Hospital Clinic de Barcelona-IDIBAPS Biobank (B.0000575), which is integrated in the Spanish National Biobanks Network. Samples were donated by patients under informed consent and they were processed following standard operating procedures with the appropriate approval of the Ethics and Scientific Committee of the Hospital Clinic de Barcelona (register code: HCB/2020/1478) and according to the guidelines of the European Network of Research Ethics Committees, following European, national and local laws.

Statistics

No statistical test was used to determined sample size upfront. Instead sample size was determined empirically according to previous knowledge of the variation in the experimental setup [4,12,34]. A minimum of four mice were quantified in each experiment and each condition. For the majority of in vitro experiments, we used n=>3 according to previous experience with similar experiments. Additional information is detailed in the reporting summary. Automated blind quantifications and blind data analysis were done whenever possible. The sample size typically results in standard error <25% of the mean. No data from in vitro or in vivo experiments were excluded, except for the CRC relapse model, where a small fraction of mice, typically 1 in every 10 in each experiment, were not included in the follow up due to invasion into the ileocaecal junction, which impeded successful surgery. Occasionally, mice bearing CRCs were sacrificed 1-2 days after surgery due to significant weight loss or unhealthy aspect, according to protocols approved by the animal experimental committees. Data distributions were assumed to be normal but this was not formally tested. Transformations were applied whenever needed. For percentage data, normality was assumed for values far form 0 or 100. Statistical analyses were performed using R software package (v.4.0.5) and GraphPad Prism (v.7.03)

Reporting summary

Further information on research design is available in the Nature Research Reporting Summary linked to this article.

ADDITIONAL REFERENCES

45. Matano, M. *et al.* Modeling colorectal cancer using CRISPR-Cas9–mediated engineering of human intestinal organoids. *Nat. Med.* **21**, 256–62 (2015).
46. Drost, J. *et al.* Sequential cancer mutations in cultured human intestinal stem cells. *Nature* **521**, 43–47 (2015).
47. Céspedes, M. V. *et al.* Orthotopic microinjection of human colon cancer cells in nude mice induces tumor foci in all clinically relevant metastatic sites. *Am. J. Pathol.* **170**, 1077–1085 (2007).
48. Chen, Y. C. *et al.* Gut Fecal Microbiota Transplant in a Mouse Model of Orthotopic Rectal Cancer. *Front. Oncol.* **10**, 1–12 (2020).
49. Conti, S. *et al.* CAFs and cancer cells co-migration in 3D spheroid invasion assay. in *Methods in Molecular Biology* vol. 2179 243–256 (2020).
50. Gonzalez-Roca, E. *et al.* Accurate expression profiling of very small cell populations. *PLoS One* **5**, e14418 (2010).
51. Dobin, A. *et al.* STAR: Ultrafast universal RNA-seq aligner. *Bioinformatics* **29**, 15–21 (2013).
52. Tarasov, A., Vilella, A. J., Cuppen, E., Nijman, I. J. & Prins, P. Sambamba: Fast processing of NGS alignment formats. *Bioinformatics* **31**, 2032–2034 (2015).
53. Liao, Y., Smyth, G. K. & Shi, W. The R package Rsubread is easier, faster, cheaper and better for alignment and quantification of RNA sequencing reads. *Nucleic Acids Res.* **47**, e47–e47 (2019).
54. Love, M. I., Huber, W. & Anders, S. Moderated estimation of fold change and dispersion for RNA-seq data with DESeq2. *Genome Biol.* **15**, 550 (2014).
55. Carvalho, B. S. & Irizarry, R. A. A framework for oligonucleotide microarray preprocessing. *Bioinformatics* **26**, 2363–2367 (2010).
56. Bolstad, B. M. *et al.* Quality Assessment of Affymetrix GeneChip Data BT - Bioinformatics and Computational Biology Solutions Using R and Bioconductor. in *Bioinformatics and Computational Biology Solutions Using R and Bioconductor* 33–47 (2005).
57. Irizarry, R. A. *et al.* Exploration, normalization, and summaries of high density oligonucleotide array probe level data. in *Selected Works of Terry Speed* 601–616 (2012). doi:10.1007/978-1-4614-1347-9_15.
58. Ritchie, M. E. *et al.* Limma powers differential expression analyses for RNA-sequencing

and microarray studies. *Nucleic Acids Res.* **43**, e47 (2015).

59. Eklund, A. C. & Szallasi, Z. Correction of technical bias in clinical microarray data improves concordance with known biological information. *Genome Biol.* **9**, R26 (2008).

60. Wu, D. *et al.* ROAST: rotation gene set tests for complex microarray experiments. *Bioinformatics* **26**, 2176–82 (2010).

61. Efron, B. & Tibshirani, R. On testing the significance of sets of genes. *Ann. Appl. Stat.* **1**, 107–129 (2007).

62. Lee, E., Chuang, H. Y., Kim, J. W., Ideker, T. & Lee, D. Inferring pathway activity toward precise disease classification. *PLoS Comput. Biol.* **4**, e1000217 (2008).

63. Zheng, G. X. Y. *et al.* Massively parallel digital transcriptional profiling of single cells. *Nat. Commun.* **8**, 14049 (2017).

64. Hao, Y. *et al.* Integrated analysis of multimodal single-cell data. *Cell* **184**, 3573-3587.e29 (2021).

65. Satija, R., Farrell, J. A., Gennert, D., Schier, A. F. & Regev, A. Spatial reconstruction of single-cell gene expression data. *Nat. Biotechnol.* **33**, 495–502 (2015).

66. Butler, A., Hoffman, P., Smibert, P., Papalexi, E. & Satija, R. Integrating single-cell transcriptomic data across different conditions, technologies, and species. *Nat. Biotechnol.* **36**, 411–420 (2018).

67. Stuart, T. *et al.* Comprehensive Integration of Single-Cell Data. *Cell* **177**, 1888-1902.e21 (2019).

68. Hafemeister, C. & Satija, R. Normalization and variance stabilization of single-cell RNA-seq data using regularized negative binomial regression. *Genome Biol.* **20**, 296 (2019).

69. van Dijk, D. *et al.* Recovering Gene Interactions from Single-Cell Data Using Data Diffusion. *Cell* **174**, 716-729.e27 (2018).

70. Subramanian, A. *et al.* Gene set enrichment analysis: a knowledge-based approach for interpreting genome-wide expression profiles. *Proc Natl Acad Sci U S A* **102**, 15545–15550 (2005).

71. Parekh, S., Ziegenhain, C., Vieth, B., Enard, W. & Hellmann, I. zUMIs - A fast and flexible pipeline to process RNA sequencing data with UMIs. *GigaScience* vol. 7 (2018).

72. La Manno, G. *et al.* RNA velocity of single cells. *Nature* **560**, 494–498 (2018).

73. Bergen, V., Lange, M., Peidli, S., Wolf, F. A. & Theis, F. J. Generalizing RNA velocity to transient cell states through dynamical modeling. *Nat. Biotechnol.* **38**, 1408–1414 (2020).

74. R Core Team. R: A Language and Environment for Statistical Computing. (2020).

75. Barrett, T. & Edgar, R. [19] Gene Expression Omnibus: Microarray Data Storage,

Submission, Retrieval, and Analysis. *Methods in Enzymology* vol. 411 352–369 (2006).

76. Grossman, R. L. *et al.* Toward a Shared Vision for Cancer Genomic Data. *N. Engl. J. Med.* **375**, 1109–1112 (2016).
77. Muzny, D. M. *et al.* Comprehensive molecular characterization of human colon and rectal cancer. *Nature* **487**, 330–337 (2012).
78. Tripathi, M. K. *et al.* Nuclear factor of activated T-cell activity is associated with metastatic capacity in colon cancer. *Cancer Res.* **74**, 6947–6957 (2014).
79. Sanz-Pamplona, R. *et al.* Aberrant gene expression in mucosa adjacent to tumor reveals a molecular crosstalk in colon cancer. *Mol. Cancer* **13**, 46 (2014).
80. Kemper, K. *et al.* Mutations in the Ras-Raf axis underlie the prognostic value of CD133 in colorectal cancer. *Clin. Cancer Res.* **18**, 3132–3141 (2012).
81. Jorissen, R. N. *et al.* Metastasis-Associated Gene Expression Changes Predict Poor Outcomes in Patients with Dukes Stage B and C Colorectal Cancer. *Clin. Cancer Res.* **15**, 7642–7651 (2009).
82. Marisa, L. *et al.* Gene Expression Classification of Colon Cancer into Molecular Subtypes: Characterization, Validation, and Prognostic Value. *PLoS Med.* **10**, e1001453 (2013).
83. Laibe, S. *et al.* A seven-gene signature aggregates a subgroup of stage II colon cancers with stage III. *Omi. A J. Integr. Biol.* **16**, 560–565 (2012).
84. Jorissen, R. N. *et al.* DNA copy-number alterations underlie gene expression differences between microsatellite stable and unstable colorectal cancers. *Clin. Cancer Res.* **14**, 8061–9 (2008).
85. Azzalini, A. & Menardi, G. Clustering via nonparametric density estimation: The R package pdfcluster. *J. Stat. Softw.* **57**, 1–26 (2014).
86. Azzalini, A. & Torelli, N. Clustering via nonparametric density estimation. *Stat. Comput.* **17**, 71–80 (2007).
87. Smedley, D. *et al.* The BioMart community portal: An innovative alternative to large, centralized data repositories. *Nucleic Acids Res.* **43**, W589–W598 (2015).
88. Drost, H. G. & Paszkowski, J. Biomartr: Genomic data retrieval with R. *Bioinformatics* **33**, 1216–1217 (2017).
89. Li, B. & Dewey, C. N. RSEM: Accurate transcript quantification from RNA-Seq data with or without a reference genome. *BMC Bioinformatics* **12**, 323 (2011).
90. Bates, D., Mächler, M., Bolker, B. M. & Walker, S. C. Fitting linear mixed-effects models using lme4. *J. Stat. Softw.* **67**, 1–48 (2015).

91. Therneau, T. M., Grambsch, P. M. & Pankratz, V. S. Penalized survival models and frailty. *J. Comput. Graph. Stat.* **12**, 156–175 (2003).
92. Therneau, T. *coxme: mixed effects Cox models. R package version 2.2-3. www.cran.R-project.org/package=coxme*. *Oikos* (2012).
93. Sanchez-Vega, F. *et al.* Oncogenic Signaling Pathways in The Cancer Genome Atlas. *Cell* **173**, 321-337.e10 (2018).
94. Mootha, V. K. *et al.* PGC-1α-responsive genes involved in oxidative phosphorylation are coordinately downregulated in human diabetes. *Nat. Genet.* **34**, 267–273 (2003).

EXTENDED DATA FIGURE LEGENDS.

Extended Data Fig. 1 | The EpiHR geneset marks a defined tumor cell population across CRCs.

a-c, UMAP layout of whole tumors (stroma + epithelium cells) from 7 CRC patients in the KUL dataset. Colored by **(a)** gene expression of all high hazard ratio genes (AllHR), **(b)** tumor microenvironment-specific HR genes (TME-HR), and (**c**) epithelial-specific HR genes (EpiHR). **d,** Association between clinical variables and the EpiHR signature in the CRC meta cohort was assessed by fitting a linear model for each variable independently. Technical factors (dataset and center, as described in extended methods) were included as covariates**.** Lines show the left and right confidence intervals. n= 1688 patients. **e.** Kaplan-Meier survival curves indicating relapse-free survival according to EpiHR gene signature expression for CRC patients classified by CMS. Two-sided Wald test. **f-g,** UMAP layout of 2718 CRC tumor cells from the KUL cohort colored by **f)** patient ID and **g)** expression of the EpiHR signature. **h,** Heatmap showing Pearson correlation scores in gene expression among EpiHR signature genes in patients from the SMC cohort. Note that most genes belong to one coherent subset (Cluster 1). Gene lists are detailed in Supplementary Table 2. **i,** UMAP layout of human CRC tumor cells colored by the expression of genes belonging to Clusters 1, 2, 3 and 4 identified in (**h**).

Extended Data Fig. 2 | Characterization of HRC features.

a, Heatmap showing scaled expression of the top 50 most correlated genes with the EpiHR signature across SMC patients. Tumor cells are divided as non-HRCs (left, FALSE) and HRCs (right, TRUE). The EpiHR signature score for each individual cell is plotted above the heatmap. **b,** UMAP layout of CRC tumor cells colored by the expression of the coreHRC signature. The coreHRC signature is defined as the top 100 genes with better correlation with the EpiHR signature. **c-d,** Heatmap showing Normalized Enrichment Scores (NES) for Genesets in Gene Ontology Biological Processes (GOBP) in HRCs from different patients in the KUL **(c)** and SMC (**d**) cohorts. Only GOBP genesets with NES scores above 0.5 are shown. Genesets and patients are ordered by hierarchical clustering. **e,** UMAP layout of human CRC tumor cells in the SMC cohort painted with the Basal cell state signature in Pancreatic Ductal Adenocarcinoma (PDAC) by Raghavan et al[10]. **f,** UMAP layout of tumor cells from the KUL cohort showing the expression of the Lgr5 signature. **g,** UMAP of same tumor cells labelled according to their classification into HRCs, Lgr5+, double positive or other cells. **h-j,** UMAP layout of same tumor cells showing gene

expression levels of canonical intestinal stem cell genes *LGR5, OLFM4* and *ASCL2*. **k-o,** UMAPs of tumor cells in the SMC dataset showing gene expression levels of canonical intestinal stem cell genes *LGR5, ASCL2, AXIN2, OLFM4,* and *SMOC2*. **p,q,** Violin plots showing WNT-ON signature expression levels in epithelial tumor cells from patients in the SMC (**p**) and KUL (**q**) cohorts. **r,** Barplot quantifying the HRC composition of each patient (combined SMC and KUL datasets). Patients are classified as iCMS2 or iCMS3 according to Joanito et al[11]. Two-sided Kruskal-Wallis test. **s,t,** UMAPs of mouse CRC AKTP tumor cells colored according to (**s**) the coreHRC signature and (**t**) the Basal cell state signature in Pancreatic cancer by Raghavan et al.[10] **u,** Gene expression levels of EpiHR (left) and coreHRC (right) signatures in MTOs derived from primary tumors or from liver metastases. Boxes represent the first, second (median) and third quartiles. Whiskers indicate maximum and minimum values. Welch two-sided t-test. n= 5 (primary) 10 (metastatic).

Extended Data Fig. 3 | Analysis of the CRC relapse mouse models and purification of DTCs. a, Representative micrograph of hematoxylin- and eosin (HE)-stained adenocarcinoma with subserosal invasion (T4) generated by injection of an AKTP MTO in the mouse caecum. Tumor center (TC), invasive fronts (IF), muscle layer (ML) and normal mucosa (NM) are indicated. Scale bar, 2.5 mm. **b,** Representative image of a different T4 tumor penetrating the muscle layer (ML) and reaching the serosa layer. TB: Tumor buds. Scale bar, 1mm. **c,** Picture of a caecum 21 days after injection and imaged at the time of surgery showing a primary tumor (arrow) in the distal part. **d-e,** Haematoxilin-Eosin (HE) staining of micrometastases and large metastases observed in the liver of orthotopic isografted mouse. Scale bars, 50 µm and 1 mm, respectively. In **e,** tumoral tissue is surrounded by dashed lines. **f-h,** HE staining of lung, lymph node and diaphragm metastases from orthotopic isografted mice. Scale bars, 100 µm (**f** and **h**) 1 mm (**g**). **i,** Graph showing liver longitudinal BLI measurements (photons per second), normalized to the day of primary tumor resection. Points and lines represent individual mice. n= 9 (AKTP), 24 (AKP) mice. **j,** Schematic representation of a novel tissue-dissociation strategy that enables recovery of DTCs from livers. Whole livers are dissected and minced thoroughly. After a mild collagenase IV digestion, samples are filtered through 100 µm meshes. The filter retained sample is highly enriched in tumor cells. Remaining tissue in the filter is re-digested with a stronger enzymatic cocktail to fully digest it, and then re-filtered. **k,** Representative bioluminescent image of a whole liver sample containing luciferase+ tumor cells before enzymatic digestion (B, input), after filtering through 100 µm (B') and 40 µm (B'') meshes (previous protocol), and after recovering and re-digesting tissue retained in the 100 µm filter (B'''). **l,** Image showing the large cell pellet containing

liver cells after 1 mild digestion and the small pellet in the retained and re-digested sample enriched in DTCs. **m,** Percentage of GFP+ cells measured by flow cytometry in samples with 1 round of digestion compared to re-digested samples. Boxes represent the first, second (median) and third quartiles. Whiskers indicate maximum and minimum values. Paired two-sided Wilcoxon test on percentages. n=6 independent paired samples examined in 2 independent experiments. **n,** Representative bioluminescent images, tumor burden and flow-cytometry plots of the 4 different stages analyzed by single-cell Smart-sequencing described in Fig. 2. Micrometastases samples were DTCs collected from livers with absent or low bioluminescence in which metastases were not visible. For small metastases samples, metastatic nodules were visible but small in size (<1.5mm). Macrometastases samples were metastatic nodules larger than 4mm.

Extended Data Fig. 4 | Additional description of residual AKTP and AKP metastatic cells. **a,** UMAPs of colorectal primary tumors and liver metastases at different stages (micro, small and large) colored according to sequencing batch, mouse ID, and sample ID. **b,** UMAPs showing the expression levels of coreHRC, EpiHR, and mKi67 gene signatures and *Lgr5* and *Krt20* genes. **c,** Violin plots showing expression of relevant genes used to define the 6 different Seurat clusters. **d,** Fraction of cells (y axis) from each Seurat cluster (x axis) present in the different sample types: Primary Tumor, micro-, small- and macro- metastases according to the indicated color code. Note the "HRCs Krt20-" are mostly exclusive from micro metastases samples, whereas Lgr5+ cells are highly enriched in small metastases samples. **e,** Smoothed *Krt20* gene and partial EMT gene signature[17] expression trends fitted with Generalized Additive Models as a function of pseudotime in primary tumors, micro+small and large metastases. **f,g,** UMAP of AKP liver micrometastases colored according to timing of profiling and Seurat clusters. **h,** Barplot showing proportion of different Seurat tumor cell types captured in AKP early vs late micrometastases. **i-l,** UMAPs showing the expression levels of the coreHRC, mKi67 and Mex3a gene signatures and *Lgr5* mRNA in AKP metastases. **m,** Barplot showing Seurat cluster distribution across AKP early and late micrometastases. **n.** Violin plots showing expression levels of the Mex3a signature[16] in AKP early and late micrometastases versus AKTP micro and small metastases. **o,** Vector fields representing RNA velocity projected on UMAPs of AKP micrometastases. Colored by the pseudotime estimated for each cell with scVelo. **p,** Smoothed coreHRC, mKi67, and Lgr5 gene signature expression trends in the early and late AKP micrometastasis dataset fitted with Generalized Additive Models as a function of CellRank pseudotime.

Extended Data Fig. 5 | Epithelial membrane protein 1 (EMP1) marks HRCs.

a, Scatter plot showing the correlation value between individual genes in the human SMC cohort (x axis) and in mouse primary tumors (y axis) with the EpiHR signature. Genes with correlation scores higher than 0.8 in both datasets are highlighted. **b,** UMAP of tumor cells from CRC patients in the SMC dataset colored according to the expression of EpiHR signature (left) and of *EMP1* gene (right). **c,** As in **b,** for CRC tumor cells from the KUL datasets. **d,** UMAP representation of Smart-sequencing single cell data of AKTP mouse tumor cells along metastatic relapse sequence colored by the EpiHR signature (left) and *Emp1* gene (right). **e,** Vector fields representing RNA velocity projected on AKTP primary CRC, micro+small and macro metastases UMAPs, colored by the pseudotime estimated for each cell with scVelo. **f,** AKTP tumor cell UMAPs colored by *Emp1* gene expression. **g,** Smoothed *Emp1* gene expression trends fitted with Generalized Additive Models as a function of pseudotime in AKTP primary tumor, micro+small and macro metastases samples. **h,** UMAP representation of AKP micrometastases colored by *Emp1* gene expression. **i,** Smoothed *Emp1* gene expression trends fitted with Generalized Additive Models as a function of pseudotime in AKP micrometastases samples. **j,** Representative flow cytometry plot of TOM expression in *wild-type* and Emp1-iCT AKTP MTOs. **k,** Relative mRNA expression in Emp1-TOM^{high} and Emp1- TOM^{low} sorted cell populations from Emp1-iCT AKTP MTOs *in vitro*. Two-sided t-test after normalizing by *Ppia*. n=3 technical replicates. Mean +/- SD. **l,** Boxplot showing normalized intensity of coreHRC signature expression in Emp1-TOM^{high} and Emp1-TOM^{low} cells dissociated from primary tumors 4 weeks post-implantation. Box plots have whiskers of maximum 1.5 times the interquartile range; boxes represent first, second (median) and third quartiles. n=4 mice per condition. ROAST-GSA adjusted p-values are shown.

Extended Data Fig. 6 | HRCs are enriched in invasion fronts and micrometastases.

a, Primary tumor outlined by cyan line and colored in 4 different regions identified with HALO image analysis classifier (tumor-red, stroma-green, background-yellow, necrosis-blue). Scale bar, 1mm. **b,** TOM cell intensity analysis in the tumor area after segmentation into individual cells. B' and B'' show magnified regions corresponding to tumor core (B') and invasion fronts + tumor buds (B''). Scale bars, 1mm (B), 100 µm (B' and B''). **c,** Representative immunostaining of TOM and E-CADH in the tumor core and in tumor buds of primary tumors derived from Emp1-iCT MTOs 4 weeks post implantation in the caecum. TOM fluorescence is shown with mpl-inferno LUT. The dashed line delimits the caecum edge. Arrows point to tumor buds. Scale bars, 100 µm (tumor core) 50 µm (tumor buds). **d,** Quantification of Emp1-TOM^{high} (defined as cells in percentile 90 for TOM expression) in the tumor core (submucosal area), invasion fronts (inside muscular layer) and isolated glands (over muscular layer). Boxes represent the first, second (median) and third

quartiles. Whiskers indicate maximum and minimum values. Two-sided Wilcoxon test on percentages. n= 8 mice. **e,** Immunofluorescence of TOM, CD31 and DAPI in primary tumors. Amplified insets show the tumor core and invasive glands intermingled in mucosal layers (ML) next to blood vessels. Dashed lines outline healthy intestinal epithelium. Scale bars, 250 µm, 100 µm (tumor core) and 50 µm (tumor buds). **f,** Representative flow cytometry plot of TOM expression in *wild-type* and Emp1-iCT AKP MTOs. **g,** Relative mRNA expression in Emp1-TOM^{high} and Emp1-TOM^{low} sorted cell populations from Emp1-iCT AKP MTOs. Two-sided t-test after normalizing by *Ppia*. n=3 technical replicates. Mean +/- SD. **h,** Representative immunostaining for TOM and E-CADHERIN in Emp1-iCT AKP tumors implanted in the caecum 4 weeks post-implantation. Emp1-TOM fluorescence is shown with an mpl-inferno LUT. Dashed lines delimit the edge of the caecum. Scale bar: 250 µm. **i,** Representative images of TOM and E-CADHERIN staining in micro (left) and medium (right) size metastases. Scale bars: 50 µm and 250 µm. **j,** Percentage of tumor area containing TOM-high and low fluorescent pixels versus metastases size (in pixels). Each dot represents an individual metastasis. **k,** TOM, KRT20 and E- CADHERIN staining in primary tumors generated by Emp1-iCasp9-tdTomato AKTP MTOs. Dashed lines encompass invasion fronts and tumor buds. KRT20 staining is observed in normal mucosa (NM) and to a lesser extent in the tumor core. Tumor cell clusters invading the muscular layer (ML) express high levels of TOM and no KRT20. Amplified insets show an example of tumor core (K') and invasion fronts (K'') with TOM (left) and KRT20 (right) stainings. Scale bars, 500 µm (k) and 100 µm (K' and K''). **l,** Immunofluorescence of TOM and E-CADHERIN (left) and KRT20 and E-CADHERIN (right) in a cluster of tumor cells that enter the liver through a portal vein (PV, delimited with dashed lines). Scale bar, 50 µm.

Extended Data Fig. 7 | HRCs retain an epithelial phenotype.

a, Immunostaining of E-CADHERIN and TOM in Emp1-iCT primary tumors 4 weeks post-implantation of MTOs. Arrows point at examples of E-CADHERIN+ invasion fronts and tumor buds. Dashed lines show the caecum edge. Scale bars, 100 µm. **b,** Boxplot showing normalized expression of genes related to EMT in Emp1-TOM^{high} versus Emp1- TOM^{low} cells. Box plots have whiskers of maximum 1.5 times the interquartile range; Boxes represent first, second (median) and third quartiles. P-value for differential expression with Linear Model for Microarray Analysis (limma). n=4 biological replicates. **c-d,** Violin plots showing expression of selected EMT-related genes in HRCs versus the rest of other cells in mouse epithelial primary tumor cells (**c**) and human tumor cells from the SMC cohort (**d**). Genes present in **b** not shown (Snai1 and Snai2) were undetected in (**c**). **e,** Representative example of *EMP1* mRNA FISH combined with LAMC2

immunofluorescence on human primary CRC tissue section showing an overlapping pattern of expression of *EMP1* and LAMC2 in invasion fronts and tumor buds (arrows). Scale bar, 100 µm. **f,** Representative example of *EMP1* mRNA FISH combined with KRT17 and EPCAM immunofluorescence on human primary CRC tissue sections showing an overlapping pattern of expression of *EMP1* and KRT17 in invading fronts and tumor cell clusters (arrows). Scale bars, 100 µm. **g,** Violin plots showing enrichment of *LAMC2*, *KRT17* and several cell-to-cell adhesion genes in HRCs (SMC cohort).

Extended Data Fig. 8 | Emp1 and Lgr5 mark distinct tumor cell populations.

a, Emp1-iCasp9-tdTomato and Lgr5-EGFP alleles introduced in AKTP MTOs. Confocal imaging of TOM, EGFP and E-CADHERIN immunostaining in edited MTOs. Single z-plane. Scale bar, 10 µm. **b,** Relative mRNA expression in EGFP-high/TOM-low and EGFP-low/TOM-high sorted cells dissociated from subcutaneous AKTP Emp1-iCT Lgr5-EGFP tumors. Two-sided t-test after normalizing by *PPIA*. Mean +/- SD. n= 3 technical replicates. **c,** Immunostaining of TOM, EGFP and E-CADHERIN in Emp1-iCT Lgr5-EGFP primary tumors 4 weeks post-implantation of MTOs in the caecum. Dashed lines encompass tumor buds. Scale bar, 250 µm. **d,** Scatter plot showing normalized Emp1-TOM intensity versus normalized Lgr5-EGFP intensity in 855,330 cells from 18 different primary tumors. Note the absence of double positive cells (TOM and EGFP high). **e,** Representative immunofluorescence staining of TOM, EGFP and E-CADHERIN in liver metastases of increasing size (micro, small, medium) generated from the mouse CRC relapse model. Scale bars, 25 µm (micro) 100 µm (small) 250 µm (medium). **f,** Scatter plot showing TOM intensity versus EGFP intensity in 318,276 cells from 137 different liver metastases. Note the absence of double positive cells (TOM and EGFP high). **g-p,** Examples of dual *EMP1 and LGR5* mRNA ISH combined with E-CADHERIN immunofluorescence on human primary CRC tissue sections demonstrating a mutually exclusive pattern of expression of *EMP1* and *LGR5*. Note that *EMP1* expression is elevated in invasion fronts and tumor cell buds (white arrows). Scale bars, 500 µm (l, p) 250 µm (g, h, i, j, m, n, o) 50 µm (H', k).

Extended Data Fig. 9 | YAP/TAZ signaling is not required for HRC specification.

a-c, Violin plots comparing the expression of genes belonging to the YAP-22 core signature[32] **(a)**, the top 50 genes from the Fetal intestine progenitor signature[31] **(b)** and the top 50 genes of the coreHRC signature **(c)** in HRCs vs other cells in the SMC scRNAseq cohort. **d**, Venn Diagram showing genes that overlap between the coreHRC signature and YAP-22 or Fetal intestine

progenitor signatures. **e.** Boxplot showing normalized intensity of YAP-22 signature expression in Emp1-TOM^{high} and Emp1- TOM^{low} cells dissociated from primary tumors 4 weeks post-implantation. Box plots have whiskers of maximum 1.5 times the interquartile range; boxes represent first, second (median) and third quartiles. n=4 mice per condition. ROAST-GSA adjusted p-values are shown. **f.** Relative mRNA expression measured by RT-qPCR in Emp1-TOM^{high} and Emp1-TOM^{low} sorted cell populations from AKTP Emp1-iCT primary CRCs. Two-sided t-test after normalizing by *PPIA*. n=4 biological replicates. Mean +/- SD. **g,** Western blot for YAP and VINCULIN in non-infected, shControl and shYap infected AKTP organoids. **h,** Western blot quantification of YAP normalized by Vinculin. **i,** Relative mRNA expression (mean ± SD) in MTOs infected with shControl plasmid compared to uninfected MTOs and MTOs infected with three different shYAP plasmids. Analyzed with a mixed effects linear model after normalizing by *PPIA* housekeeping gene. n= 2 biological replicates with 3 technical replicates. **j,** Percentage (mean ± SD) of Emp1-TOM^{high} cells in organoids infected with shControl or shYAP plasmids. Two-sided Wilcoxon t-test. n= 3 (sh67) 7 (all other) measurements examined over 4 independent experiments. **k,** Relative mRNA expression (mean ± SD) in MTOs infected with pInducer GFP-TEADi plasmid treated or untreated with doxycycline (DOX). GFP+ cells were sorted in DOX treated organoids, whereas alive cells were sorted in untreated MTOs. n= 3 technical replicates. Analyzed with a mixed effects linear model after normalizing by *PPIA* housekeeping gene. **l,** Representative flow cytometry plot showing Emp1-TOM fluorescence versus pInducer GFP-TEADi fluorescence in TEADi MTOs untreated or treated with DOX. **m,** Quantification (mean ± SD) of Emp1-TOM^{high} in TEADi MTOs untreated or treated with DOX for 5 days. Two-sided Wilcoxon t-test. n= 2 biological replicates with 3 technical replicates. **n,** Boxplot showing expression levels (normalized intensity) of YAP-22, Fetal and coreHRC signature genes in control MTOs versus MTOs treated with chemotherapy (folfiri) for 4 days. Boxes represent the first, second (median) and third quartiles. Whiskers indicate maximum and minimum values. n=3 biological replicates per condition. Two-sided t-test. **o,** Boxplot showing the expression levels of relevant genes in control MTOs versus MTOs treated with chemotherapy (folfiri) for 4 days. Boxes represent the first, second (median) and third quartiles. Whiskers indicate maximum and minimum values. n=3 biological replicates per condition. Two-sided t-test.

Extended Data Fig. 10 | KRAS mutations and CAFs specify the HRC population.

a-b, Normalized intensity of EpiHR and coreHRC signature expression in CTOs grouped by gain of function mutation in Kras g12d and loss of function mutations in *p53, Smad4 and Tgfbr2*. Box

plots have whiskers of maximum and minimum values; boxes represent first, second (median) and third quartiles. n= 6 (WT) 5 (MUT) CTOs; 2 technical replicates. P-values for two-sided T-tests. **c,** Percentage of Emp1^{high} tumor cells (defined as the top 10% of the TOM population in control MTOs, mean ± SD) in parental (non-infected), control shRNA or shRNAs targeting YAP1. n=5 biological replicates. P-value for two-sided t-test. **d,** Normalized intensity of the coreHRC signature expression in control MTOs versus MTOs co-cultured with colon fibroblasts. Box plots have whiskers of maximum 1.5 times the interquartile range; boxes represent first, second (median) and third quartiles. n= 4 biological replicates. ROAST-GSA adjusted p-value is shown. **e,** Representative images of MTOs Emp1-iCT Lgr5-EGFP co-cultured with colon fibroblasts. Maximum intensity projection of confocal stacks, step 4 µm, z stack 120 µm. Scale bar, 50 µm. **f,** Immunostaining of α-SMA Emp1-iCT Lgr5-EGFP MTOs co-cultured with colon fibroblasts for 2 days. Scale bars, 100 µm. **g,** Immunostaining of KRT17 in 4-days grown MTO Emp1-iCT organoids: fibroblast co-cultures and organoids alone control cultures. Scale bars, 50 µm. **h,** Ablation by dimerizer (DIM) treatment and surgery schedule of mice with AKTP Emp1-iCT primary tumors to assess the recovery of HRCs upon treatment cessation. **i,** Percentage (mean ± SD) of Emp1-TOM^{high} cells (defined as top 10% in control animals) in untreated mice versus mice treated with DIM, with treatment discontinued at various timepoints post-injection. Two-sided T-test. n= 3 (control) 4 (rest) mice. **j,** Representative immunostainings showing effective Emp1-TOM^{high} cell ablation in DIM-treated primary tumors and recovery upon treatment cessation. Dashed lines delimitate the caecum edge. Scale bars, 250 µm. **k,** Lung metastases (mean ± SD) generated by MTO Emp1-iCT up to one month after primary tumor resection, treated with vehicle or DIM as in Fig. 4a. Each dot is a mouse; n= 34 (control) 29 (DIM). P-value for generalized linear model with negative binomial family. **l,** Inducible ablation schedule of nude mice (*nu/nu*) implanted with AKTP Emp1-iCT primary tumors. Resection was not possible due to local spreading of tumors to neighboring tissue. **m,** Primary tumor area (mean ± SD) measured at sacrifice. Each dot is a mouse; n= 4 (control), 5 (DIM) mice. P-value for linear model. **n,** Liver metastases (mean ± SD) generated by MTO Emp1-iCT. Each dot is a mouse; n= 4 (control), 6 (DIM) mice. P-value for generalized linear model. **o,** Schematics of an experiment to analyze the potential of Emp1+, Lgr5+ or double negative (DN) cells to colonize the liver and generate metastases. 25,000 FACS-sorted Emp1-TOM-high, Lgr5-EGFP-high or double negative cells were injected intrasplenically. **p,** Metastatic growth measured by BLI. **q,** Liver metastases (mean ± SD) generated by Emp1-high, Lgr5-high or double negative cells. Each dot is a mouse; n= 5 mice. P-value for generalized linear model. **r,** Distribution of liver metastasis diameters (mean ± SD). n=5 mice per condition. **s,** Percentage (mean ± SD) of Emp1-TOM^{high}, Lgr5-EGFP^{high} and

double negative tumor cells in metastases generated by the injection of Emp1-TOM^{high}, Lgr5-EGFP^{high} and double negative cells, n= 9 (Emp1 and Lgr5) and 10 (DN) mice. Two-sided t-test. **t,** Experimental setup showing inducible HRC ablation after surgery of primary AKPT CRCs. **u,** Liver metastases (mean ± SD) generated by MTO Emp1-iCT in mice treated with vehicle or DIM 1 day after primary tumor resection and until experimental endpoints. Each dot is a mouse; n=30 (control) 12 (DIM) mice P-value for generalized linear model. **v,** Percentage (mean ± SD) of small (diameter equal or smaller than 1 mm^2) and big metastases (bigger than 1 mm^2) in mice treated with vehicle or DIM 1 day after primary tumor resection and until experimental endpoints**.** Mixed effects linear model after boxcox transformation with mouse as random effect, n= 20 (control) and 7 (DIM) mice. **w,** Percentage of mice that developed liver metastases in control and Emp1-ablated mice. Analyzed with a generalized linear model.

Extended Data Fig. 11 | Metastatic relapse in different mouse CRC models arises from HRCs.

a, Inducible ablation and surgery schedule of mice with AKP Emp1-iCT primary tumors. Panels A and A’ show immunostaining of TOM and E-CADHERIN demonstrating effective ablation of Emp1-high cells in primary CRCs. Dashed lines delimitate the caecum edge. Scale bars, 500 µm. **b,** Primary tumor area (mean ± SD) measured after resection. Each dot is a mouse, n= 12 (control) and 6 (DIM) mice. P-value for linear model after boxcox transformation. **c,** Liver metastases (mean ± SD) generated by MTO AKP Emp1-iCT up to one month after primary tumor resection. Each dot is a mouse, n= 12 (control) and 6 (DIM) mice. P-value for generalized linear model with negative binomial family. Bottom panel indicates the percentage of mice that developed liver metastases in the same experiment. Analyzed with a two-sided fisher test. **d,** Inducible ablation and surgery schedule of mice with AKPS Emp1-iCT primary tumors. Panels D and D’ show immunostaining of TOM demonstrating effective ablation of Emp1-TOM^{high} cells in DIM-treated primary tumors. Dashed lines delimitate the caecum edge. Scale bars, 250 µm. **e,** Primary tumor area (mean ± SD) measured after resection. Each dot is a mouse, n= 17 (control) and 19 (DIM) mice. P-value for linear model after boxcox transformation. **f,** Liver metastases (mean ± SD) generated by MTO AKPS Emp1-iCT up to one month after primary tumor resection. Each dot is a mouse, n as in panel **e**. P-value for generalized linear model with negative binomial family. Bottom panel indicate the percentage of mice that developed liver metastases in the same experiment. Analyzed with a two-sided fisher test. **g,** Inducible ablation schedule of mice implanted with AKTP Emp1-iCT MTOs in the rectum. **h,** Longitudinal intravital BLI quantification of AKTP MTOs implanted in the rectum. **i,** Representative TOM and E-CADHERIN

immunostainings of lungs from mice bearing AKTP rectal tumors. Lung metastases of increasing size are shown. Note that TOM expression is higher in micrometastases and progressively reduced. Scale bars, 50 µm. **j,** Primary rectal tumor area (mean ± SD) measured at sacrifice. Each dot is a mouse, n= 9 (control) and 10 (DIM) mice. P-value for linear model after boxcox transformation. **k,** Lung (left panel) and liver (middle panel) metastases (mean ± SD) generated by MTO Emp1-iCT injected in the rectum. Each dot is a mouse, n as in panel **j**. P-value for generalized linear model with Poisson family. Right panel shows the percentage of mice that developed metastases in the same experiment. Analyzed with a two-sided fisher test. **l,** CRISPR-Cas9 targeting strategy to introduce an DTR-GFP cassette into the Lgr5 locus of MTOs. Confocal imaging of immunostaining for EGFP and EPCAM in Lgr5-DTR-EGFP organoids. Scale bar, 30µm. Right panel shows a representative flow cytometry plot of EGFP expression in wild-type and Lgr5-EGFP organoids. **m,** Relative *Lgr5* mRNA expression (mean ± SD) of Lgr5-EGFP^{high} versus -low cells isolated from Lgr5-DTR-EGFP subcutaneous tumors. n=3 biological replicates. Two-sided t-test normalizing to *B2M*. **n,** Immunofluorescence showing EGFP and E-CADHERIN in primary tumors. Insets (N' and N'') correspond to invasion fronts and tumor buds lacking EGFP expression at higher magnification. Scale bars, 500 µm (D) and 100 µm (D' and D''). **o,** Quantification of Lgr5-EGFP^{high} cells (defined as cells in percentile 90 for EGFP expression) in the tumor core, invasion fronts and tumor buds. Boxes represent the first, second (median) and third quartiles. Whiskers indicate maximum and minimum values. Paired two-sided Wilcoxon test on percentages. n= 11 mice. **p,** Representative images of Lgr5-EGFP staining in micro (P) and small (P') metastases. Dashed lines and the yellow arrow surround a micrometastasis. Scale bars: (F) 50 µm; (F') 250 µm. **q,** Percentage of tumor area containing Lgr5-EGFP^{high} and Lgr5-EGFP^{low} cells versus metastases size. Each dot represents an individual metastasis. **r,** CRISPR-Cas9 targeting strategy to introduce an iCaspase-9-TOM cassette into the LGR5 locus of AKTP MTOs. **s,** Representative flow cytometry plot of TOM expression in Lgr5-iCasp9-tdTomato organoids. **t,** Quantification of *Lgr5* mRNA (mean ± SD) by RT-qPCR in Lgr5-TOM^{high} and Lgr5-TOM^{low} cells dissociated from primary tumors grown for 4 weeks. n=3 primary tumors. Analyzed with a mixed effects linear model. **u,** Timing of inducible ablation and surgery in mice implanted with AKTP Lgr5-iCasp9-TOM primary tumors. **v,** Representative flow cytometry plot of Lgr5-TOM fluorescence in controls versus dimerizer-treated mice. DAPI-/EPCAM+ cells are shown. **w,** Percentage (mean ± SD) of Lgr5^{high} tumor cells (defined as the top 10% of the TOM+ population) in control and treated mice. n=4 mice each group. Two-sided Wilcoxon test. **x,** Primary tumor area measured after resection. n= 15 mice each group. Mean with SD, p-value of linear model after boxcox transformation. **y,** Liver metastases counted at experimental endpoints after primary

tumor resection. n= 16 (control) and 21 (Lgr5-ablation) mice. Mean ± SD. Analyzed with a linear model with negative binomial family**.** Left panel show the percentage of mice that developed liver metastases in control and Lgr5-ablated tumors in the same experiment. Two-sided Fisher test.

Extended Data Fig. 12 | Immune checkpoint immunotherapies prevent metastatic relapse. **a**, Representative image of CD3+ cell distribution in primary AKTP CRC showing T cell exclusion. Arrows point to T cells located at the tumor periphery. **b,** Representative immunostaining of Emp1-TOM, CD3 and α-SMA in primary tumors. Scale bars, 100 µm. **c,** Dotplot summarizing regression models applied to multiplex immunofluorescence data. Effects of the total number of cells on the composition of every cell population are represented by different point sizes (defining the magnitude of the effect) and colors (showing both the sign of the effect in blue(-)/red(+) and the statistical significance by color intensity). **d,** Dotplot showing examples of interferon response genes across 6 tumor scRNAseq cell clusters as defined in Fig. 2g. **e,** Bioluminescence monitoring of the effect of the neoadjuvant immunotherapy regime used in Fig. 5k on primary tumor growth. Points and lines represent individual mice, trend lines (bold) show a LOESS model. n= 19 (control) 10 (PD1+CTLA4) mice. Mixed effects linear model with data normalized to time 0 and mouse as random effect. **f,** Schematics of an experiment comparing metastatic relapse in untreated mice and mice treated with neoadjuvant treatment with anti-PD1 monotherapy or anti-PD1+/anti-CTLA4 combined therapy. **g,** Primary tumor area (mean ± SD) measured after resection in the experiment described in **f**. Each dot is an individual mice. n= 10 mice each group. Linear model. **h**, Liver metastases (mean ± SD) generated by AKTP primary tumors up to one month after primary tumor resection in the experiment described in **f**. Each dot is an individual mice. n= 9 (control), 8 (PD1), 10 (PD1/CTLA4). Generalized linear model of Poisson family. **i**, Percentage of mice that developed liver metastases or remained metastases-free at experimental endpoints (4 weeks after resection) in control and immunotherapy-treated tumors. n= 9 (control), 8 (PD1), 10 (PD1/CTLA4). Generalized linear model with beta-binomial distribution.

Supplementary Data 1. Uncropped Western Blot related to Ext.Fig.9g

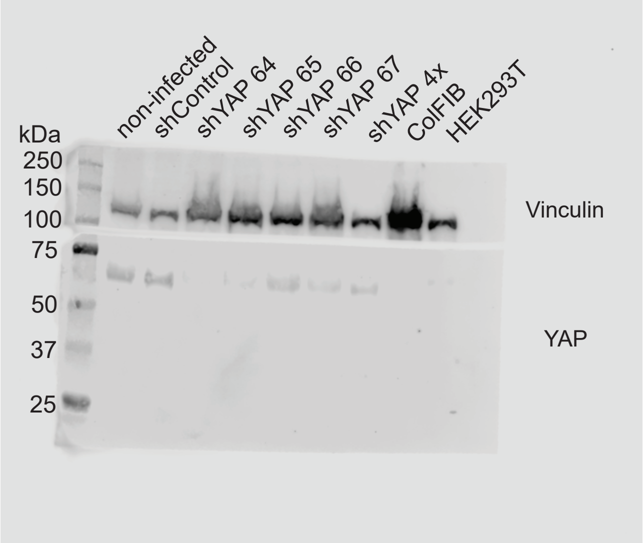

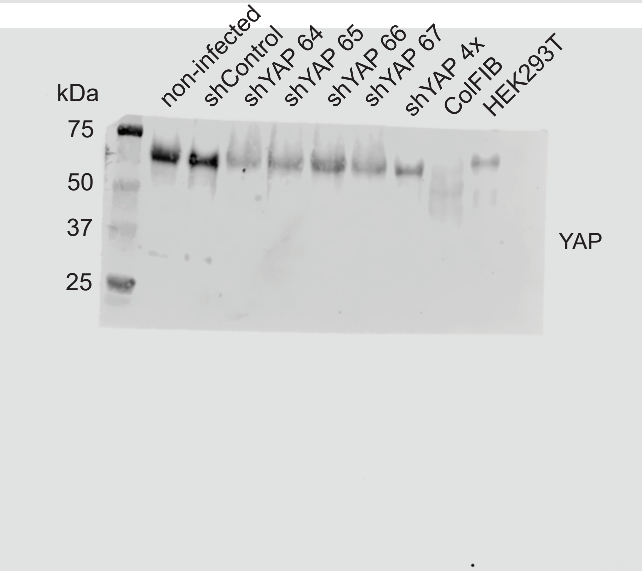

Supplementary Data 2. Example of gating strategy

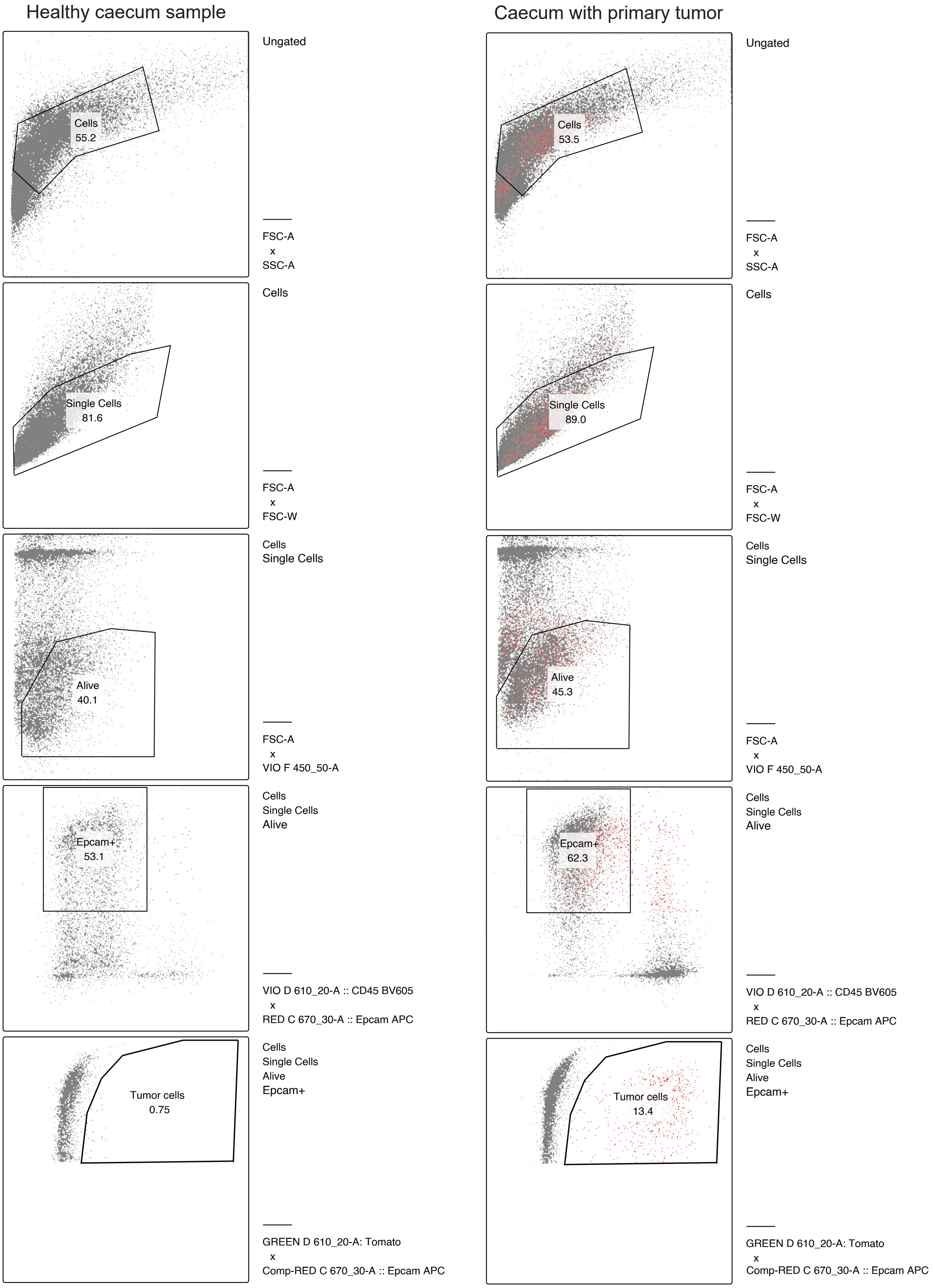

Extended Figures combined

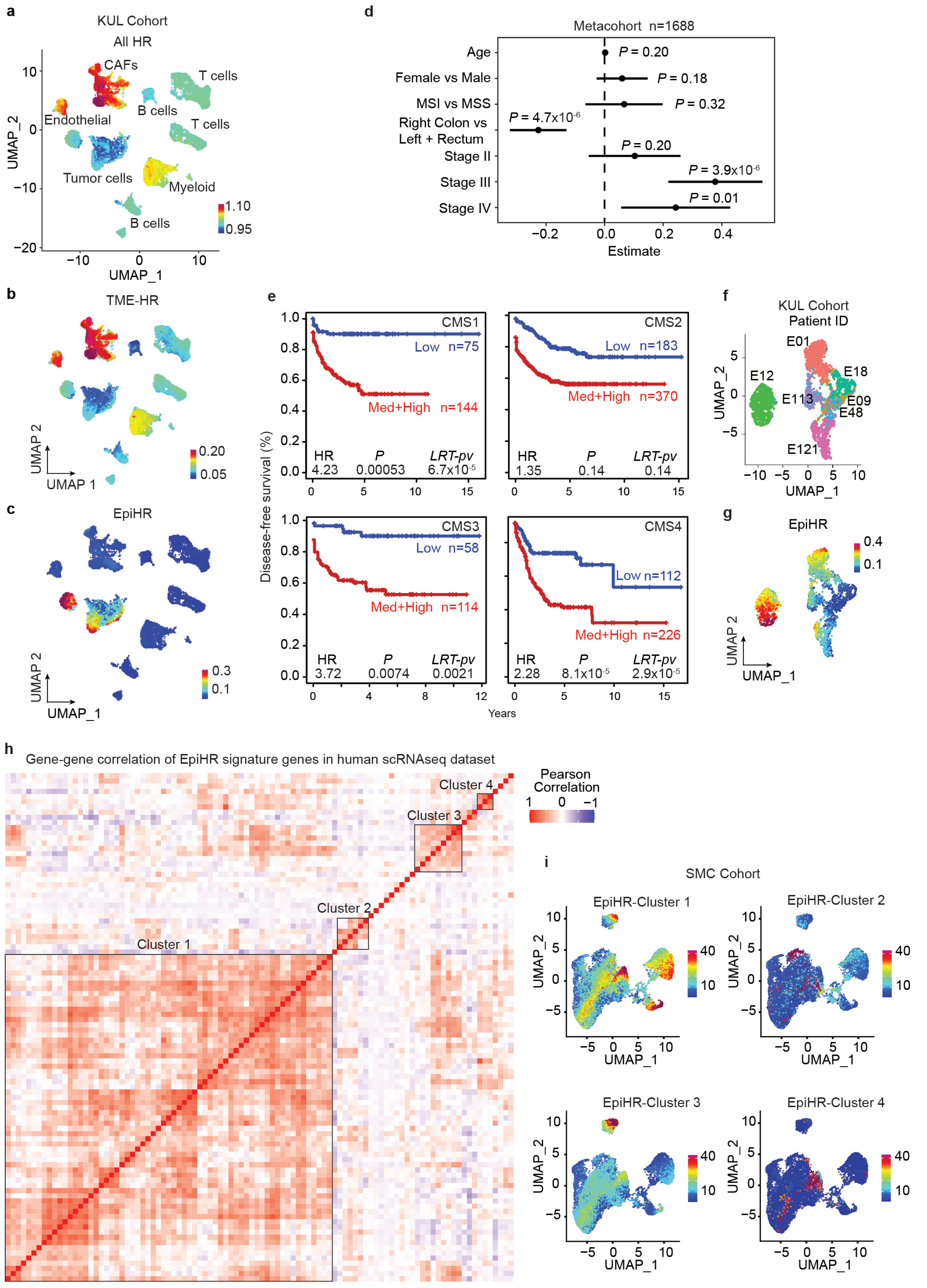
a
KUL Cohort
All HR
CAFs
T cells
B cells
Endothelial
T cells
Tumor cells
Myeloid
B cells
1.10
0.95
UMAP_2
UMAP_1
b
TME-HR
0.20
0.05
UMAP 2
UMAP 1
c
EpiHR
0.3
0.1
UMAP 2
UMAP_1
d
Metacohort n=1688
Age
Female vs Male
MSI vs MSS
Right Colon vs Left + Rectum
Stage II
Stage III
Stage IV
P = 0.20
P = 0.18
P = 0.32
P = 4.7x10-6
P = 0.20
P = 3.9x10-6
P = 0.01
Estimate
e
CMS1
Low n=75
Med+High n=144
HR 4.23
P 0.00053
LRT-pv 6.7x10-5
CMS2
Low n=183
Med+High n=370
HR 1.35
P 0.14
LRT-pv 0.14
CMS3
Low n=58
Med+High n=114
HR 3.72
P 0.0074
LRT-pv 0.0021
CMS4
Low n=112
Med+High n=226
HR 2.28
P 8.1x10-5
LRT-pv 2.9x10-5
Disease-free survival (%)
Years
f
KUL Cohort
Patient ID
E01
E12
E18
E113
E09
E48
E121
UMAP_2
UMAP_1
g
EpiHR
0.4
0.1
UMAP 2
UMAP_1
h
Gene-gene correlation of EpiHR signature genes in human scRNAseq dataset
Cluster 1
Cluster 2
Cluster 3
Cluster 4
Pearson Correlation
1 0 −1
i
SMC Cohort
EpiHR-Cluster 1
EpiHR-Cluster 2
EpiHR-Cluster 3
EpiHR-Cluster 4
40
10
UMAP_2
UMAP_1

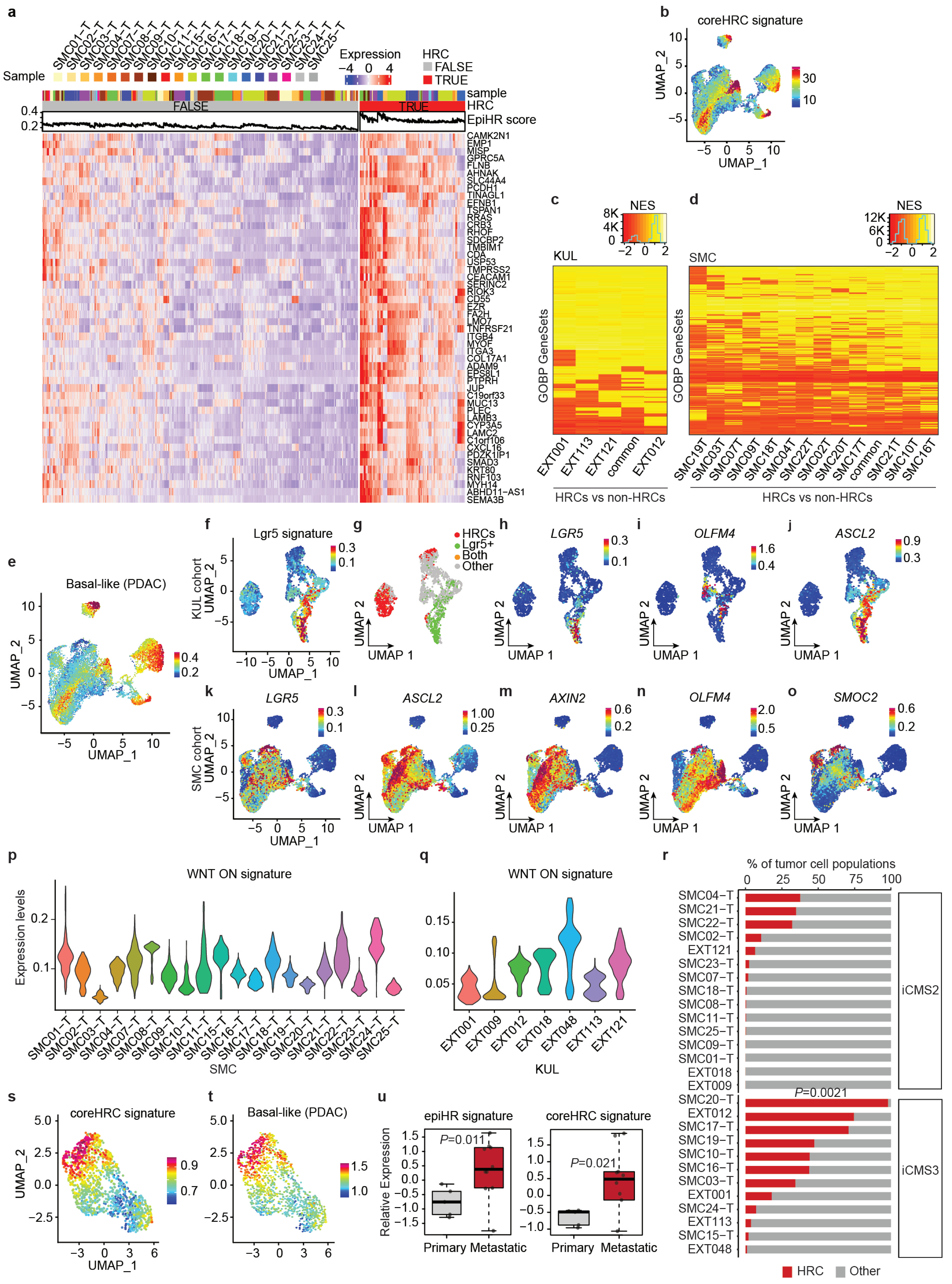
a
Sample
SMC01−T SMC02−T SMC03−T SMC04−T SMC07−T SMC08−T SMC09−T SMC10−T SMC11−T SMC15−T SMC16−T SMC17−T SMC18−T SMC19−T SMC20−T SMC21−T SMC22−T SMC23−T SMC24−T SMC25−T
Expression
−4 0 4
HRC
FALSE
TRUE
sample
HRC
EpiHR score
0.4
0.2
CAMK2N1
EMP1
MISP
GPRC5A
FLNB
AHNAK
SLC44A4
PCDH1
TINAGL1
EFNB1
TSPAN1
RRAS
CRB3
RHOF
SDCBP2
TMBIM1
CDA
USP53
TMPRSS2
CEACAM1
SERINC2
RIOK3
CD55
EZR
FA2H
LMO7
TNFRSF21
ITGB4
MYOF
ITGA3
COL17A1
ADAM9
EPS8L1
PTPRH
JUP
C19orf33
MUC13
PLEC
LAMB3
CYP3A5
LAMC2
C1orf106
CXCL16
PDZK1IP1
SMAD3
KRT80
RNF103
MYH14
ABHD11−AS1
SEMA3B
b
coreHRC signature
UMAP_2
UMAP_1
30
10
c
NES
8K
4K
0
−2 0 2
KUL
GOBP GeneSets
EXT001 EXT113 EXT121 common EXT012
HRCs vs non-HRCs
d
NES
12K
6K
0
−2 0 2
SMC
GOBP GeneSets
SMC19T SMC03T SMC07T SMC09T SMC18T SMC04T SMC22T SMC02T SMC20T SMC17T common SMC21T SMC10T SMC16T
HRCs vs non-HRCs
e
Basal-like (PDAC)
UMAP_2
UMAP_1
0.4
0.2
f
Lgr5 signature
KUL cohort
UMAP_2
UMAP_1
0.3
0.1
g
HRCs
Lgr5+
Both
Other
UMAP 2
UMAP 1
h
LGR5
0.3
0.1
i
OLFM4
1.6
0.4
j
ASCL2
0.9
0.3
k
LGR5
SMC cohort
UMAP_2
UMAP_1
0.3
0.1
l
ASCL2
1.00
0.25
m
AXIN2
0.6
0.2
n
OLFM4
2.0
0.5
o
SMOC2
0.6
0.2
p
WNT ON signature
Expression levels
SMC01−T SMC02−T SMC03−T SMC04−T SMC07−T SMC08−T SMC09−T SMC10−T SMC11−T SMC15−T SMC16−T SMC17−T SMC18−T SMC19−T SMC20−T SMC21−T SMC22−T SMC23−T SMC24−T SMC25−T
SMC
q
WNT ON signature
EXT001 EXT009 EXT012 EXT018 EXT048 EXT113 EXT121
KUL
r
% of tumor cell populations
0 25 50 75 100
SMC04−T SMC21−T SMC22−T SMC02−T EXT121 SMC23−T SMC07−T SMC18−T SMC08−T SMC11−T SMC25−T SMC09−T SMC01−T EXT018 EXT009
iCMS2
P=0.0021
SMC20−T EXT012 SMC17−T SMC19−T SMC10−T SMC16−T SMC03−T EXT001 SMC24−T EXT113 SMC15−T EXT048
iCMS3
HRC
Other
s
coreHRC signature
UMAP_2
UMAP_1
0.9
0.7
t
Basal-like (PDAC)
1.5
1.0
u
epiHR signature
Relative Expression
P=0.011
Primary Metastatic
coreHRC signature
P=0.021
Primary Metastatic

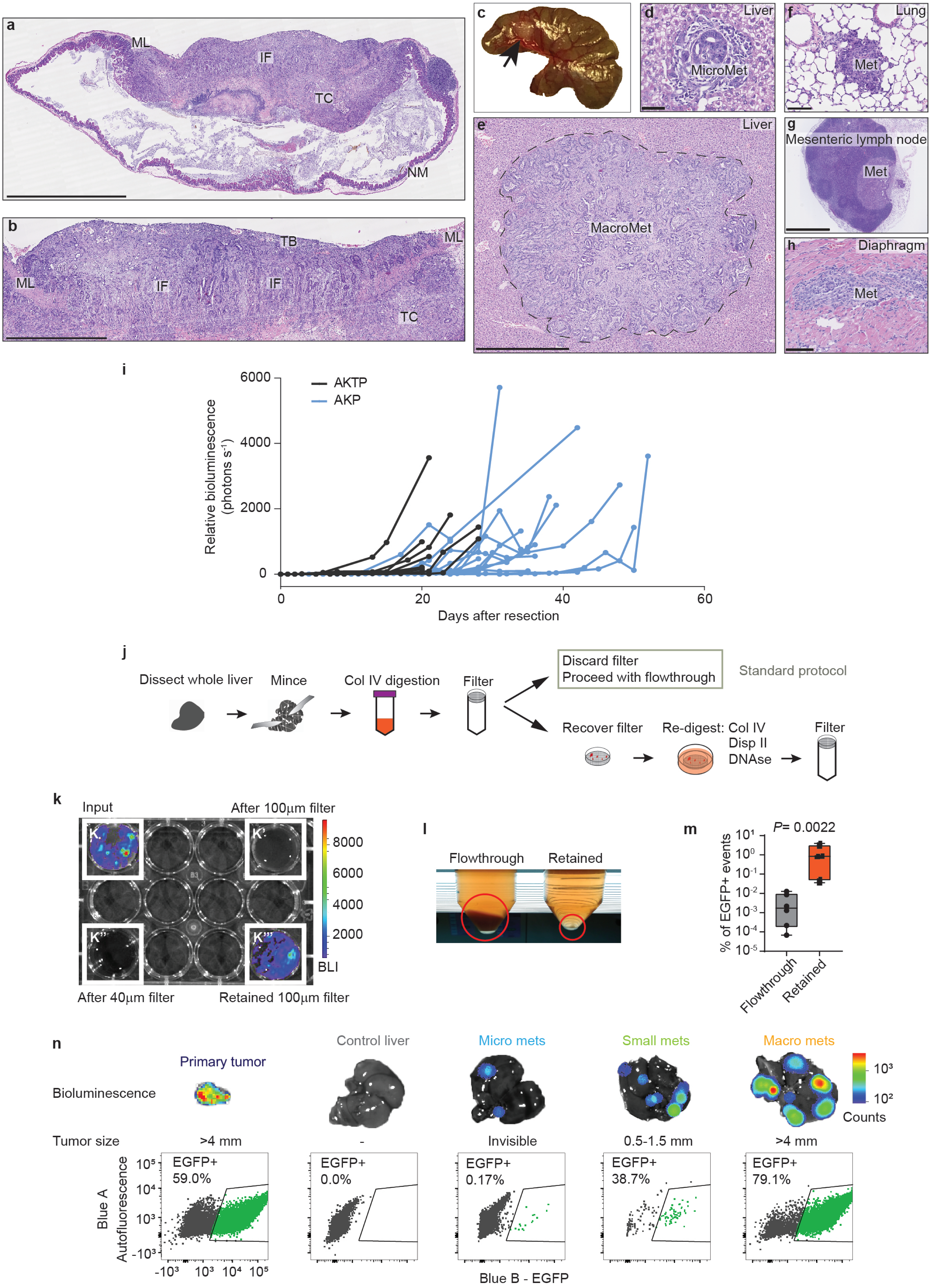

a
ML
IF
TC
NM
b
TB
ML
IF
IF
ML
TC
c
d
Liver
MicroMet
f
Lung
Met
e
Liver
MacroMet
g
Mesenteric lymph node
Met
h
Diaphragm
Met
i
AKTP
AKP
Relative bioluminescence (photons s-1)
6000
4000
2000
0
0
20
40
60
Days after resection
j
Dissect whole liver
Mince
Col IV digestion
Filter
Discard filter
Proceed with flowthrough
Standard protocol
Recover filter
Re-digest: Col IV
Disp II
DNAse
Filter
k
Input
After 100µm filter
K
K'
K''
K'''
8000
6000
4000
2000
BLI
After 40µm filter
Retained 100µm filter
l
Flowthrough
Retained
m
P= 0.0022
% of EGFP+ events
Flowthrough
Retained
n
Primary tumor
Control liver
Micro mets
Small mets
Macro mets
Bioluminescence
Counts
Tumor size
>4 mm
-
Invisible
0.5-1.5 mm
>4 mm
Blue A
Autofluorescence
EGFP+
59.0%
EGFP+
0.0%
EGFP+
0.17%
EGFP+
38.7%
EGFP+
79.1%
Blue B - EGFP

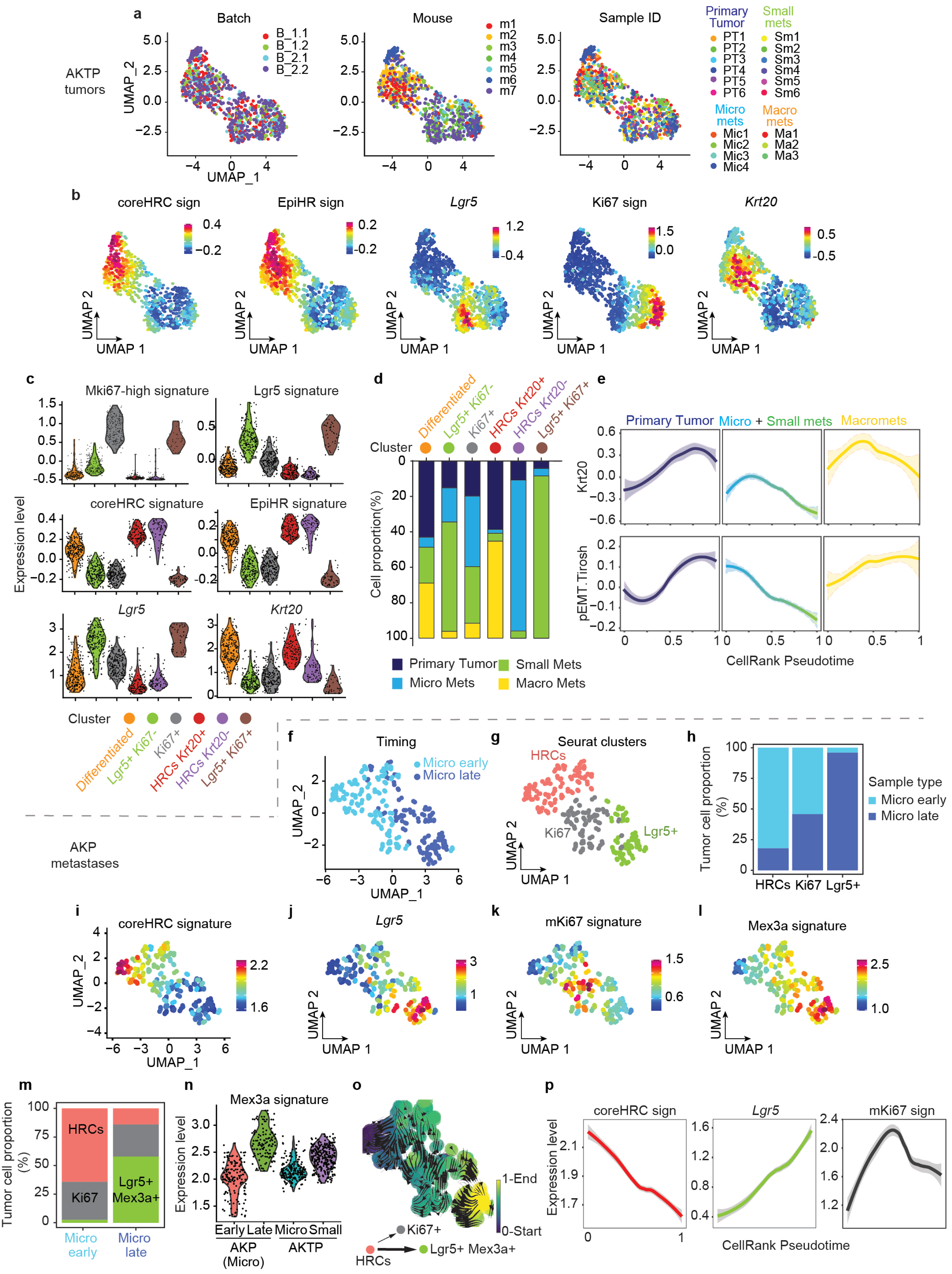

a
AKTP tumors
Batch
B_1.1
B_1.2
B_2.1
B_2.2
Mouse
m1
m2
m3
m4
m5
m6
m7
Sample ID
Primary Tumor
PT1
PT2
PT3
PT4
PT5
PT6
Small mets
Sm1
Sm2
Sm3
Sm4
Sm5
Sm6
Micro mets
Mic1
Mic2
Mic3
Mic4
Macro mets
Ma1
Ma2
Ma3
UMAP_1
UMAP_2
b
coreHRC sign
EpiHR sign
Lgr5
Ki67 sign
Krt20
UMAP 1
UMAP 2
c
Mki67-high signature
Lgr5 signature
coreHRC signature
EpiHR signature
Lgr5
Krt20
Expression level
Cluster
Differentiated
Lgr5+ Ki67-
Ki67+
HRCs Krt20+
HRCs Krt20-
Lgr5+ Ki67+
d
Cluster
Cell proportion(%)
Primary Tumor
Small Mets
Micro Mets
Macro Mets
e
Primary Tumor
Micro + Small mets
Macromets
Krt20
pEMT.Tirosh
CellRank Pseudotime
AKP metastases
f
Timing
Micro early
Micro late
UMAP_1
UMAP_2
g
Seurat clusters
HRCs
Ki67
Lgr5+
UMAP 1
UMAP 2
h
Tumor cell proportion (%)
Sample type
Micro early
Micro late
HRCs
Ki67
Lgr5+
i
coreHRC signature
j
Lgr5
k
mKi67 signature
l
Mex3a signature
m
Tumor cell proportion (%)
HRCs
Ki67
Lgr5+ Mex3a+
Micro early
Micro late
n
Mex3a signature
Expression level
Early
Late
Micro
Small
AKP (Micro)
AKTP
o
1-End
0-Start
Ki67+
HRCs
Lgr5+ Mex3a+
p
coreHRC sign
Lgr5
mKi67 sign
Expression level
CellRank Pseudotime

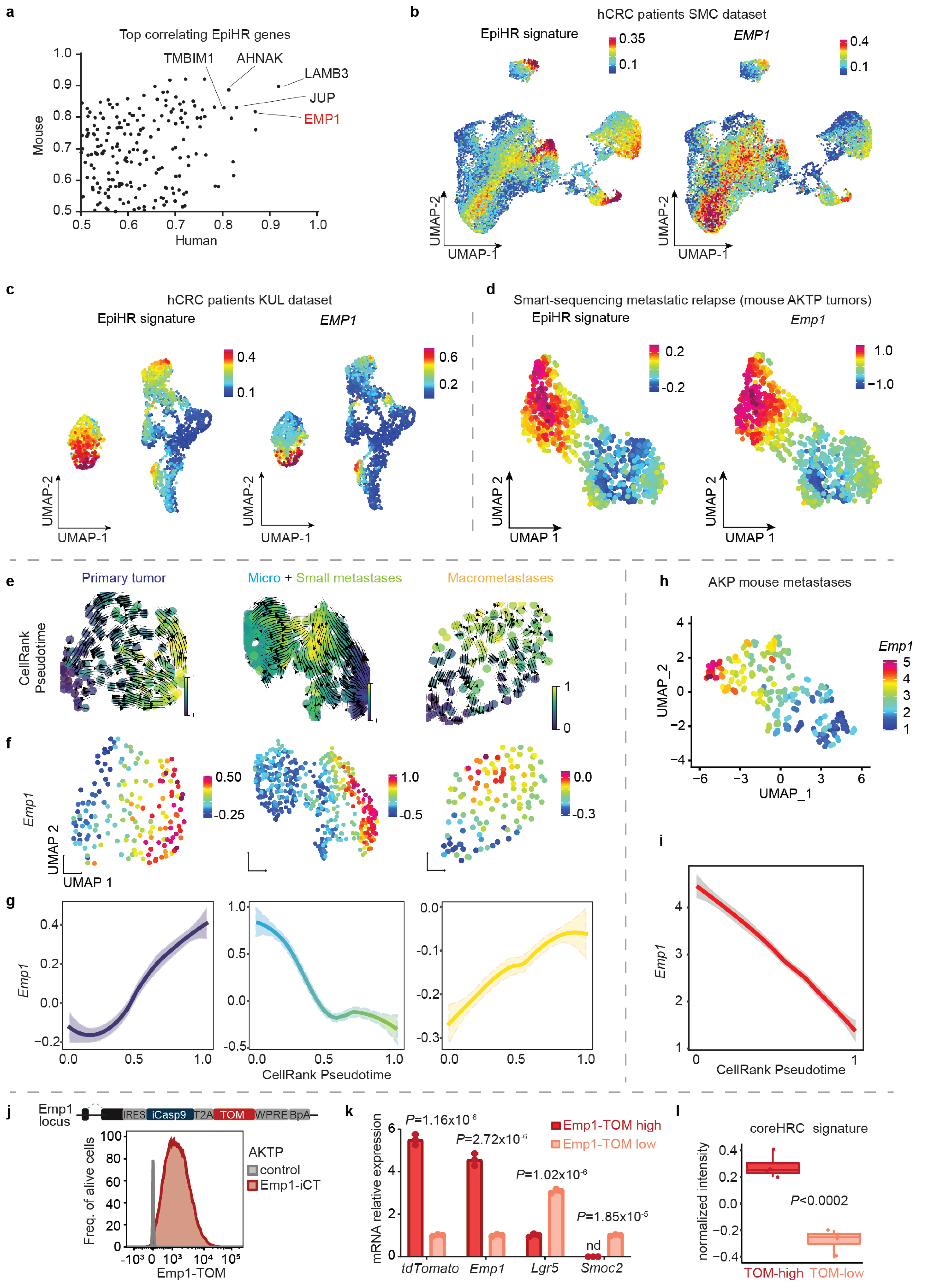
a
Top correlating EpiHR genes
TMBIM1
AHNAK
LAMB3
JUP
EMP1
Mouse
Human
b
hCRC patients SMC dataset
EpiHR signature
EMP1
UMAP-2
UMAP-1
c
hCRC patients KUL dataset
EpiHR signature
EMP1
d
Smart-sequencing metastatic relapse (mouse AKTP tumors)
EpiHR signature
Emp1
UMAP 2
UMAP 1
e
Primary tumor
Micro + Small metastases
Macrometastases
CellRank Pseudotime
f
Emp1
g
Emp1
CellRank Pseudotime
h
AKP mouse metastases
Emp1
UMAP_2
UMAP_1
i
Emp1
CellRank Pseudotime
j
Emp1 locus
IRES
iCasp9
T2A
TOM
WPRE
BpA
AKTP
control
Emp1-iCT
Freq. of alive cells
Emp1-TOM
k
P=1.16x10-6
P=2.72x10-6
P=1.02x10-6
P=1.85x10-5
nd
Emp1-TOM high
Emp1-TOM low
mRNA relative expression
tdTomato
Emp1
Lgr5
Smoc2
l
coreHRC signature
normalized intensity
P<0.0002
TOM-high
TOM-low

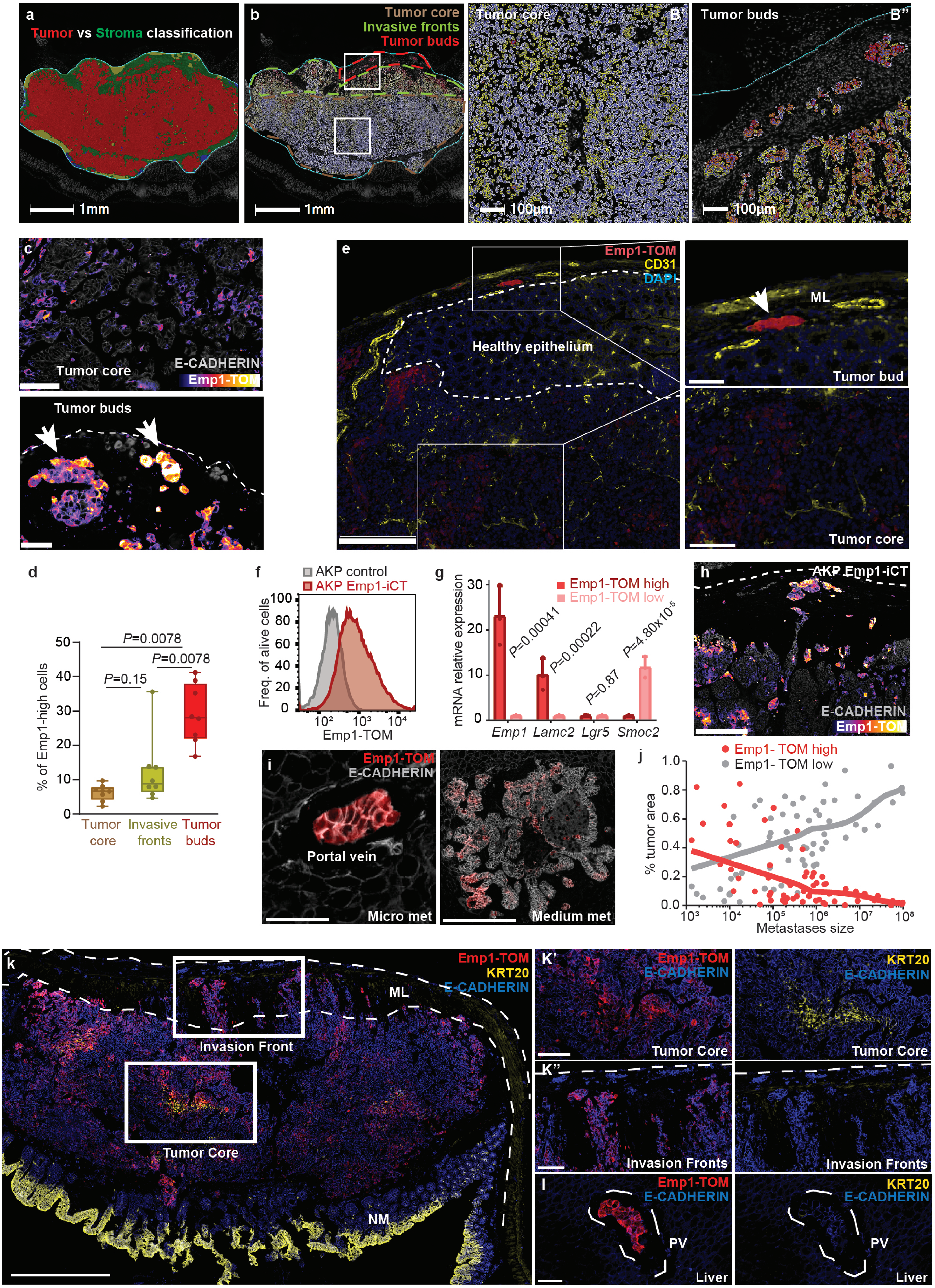
a
Tumor vs Stroma classification
1mm
b
Tumor core
Invasive fronts
Tumor buds
1mm
Tumor core
B'
100µm
Tumor buds
B''
100µm
c
Tumor core
E-CADHERIN
Emp1-TOM
Tumor buds
d
% of Emp1-high cells
P=0.0078
P=0.0078
P=0.15
Tumor core
Invasive fronts
Tumor buds
e
Emp1-TOM
CD31
DAPI
Healthy epithelium
ML
Tumor bud
Tumor core
f
AKP control
AKP Emp1-iCT
Freq. of alive cells
Emp1-TOM
g
mRNA relative expression
Emp1-TOM high
Emp1-TOM low
P=0.00041
P=0.00022
P=0.87
P=4.80x10-5
Emp1
Lamc2
Lgr5
Smoc2
h
AKP Emp1-iCT
E-CADHERIN
Emp1-TOM
i
Emp1-TOM
E-CADHERIN
Portal vein
Micro met
Medium met
j
Emp1- TOM high
Emp1- TOM low
% tumor area
Metastases size
k
Emp1-TOM
KRT20
E-CADHERIN
ML
Invasion Front
Tumor Core
NM
K'
Emp1-TOM
E-CADHERIN
Tumor Core
KRT20
E-CADHERIN
Tumor Core
K''
Invasion Fronts
Invasion Fronts
l
Emp1-TOM
E-CADHERIN
PV
Liver
KRT20
E-CADHERIN
PV
Liver

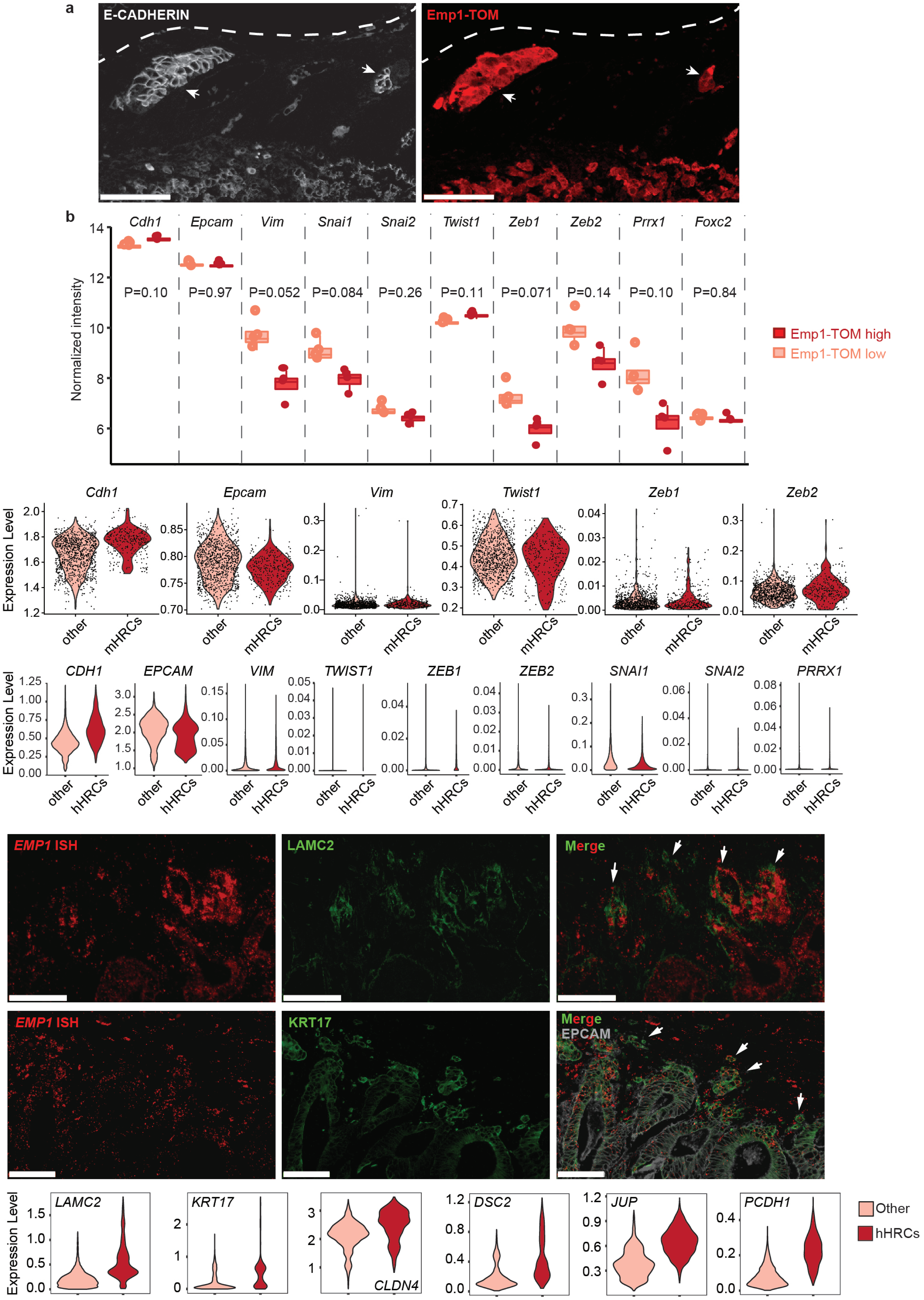
a
E-CADHERIN
Emp1-TOM
b
Cdh1
Epcam
Vim
Snai1
Snai2
Twist1
Zeb1
Zeb2
Prrx1
Foxc2
P=0.10
P=0.97
P=0.052
P=0.084
P=0.26
P=0.11
P=0.071
P=0.14
P=0.10
P=0.84
Normalized intensity
14
12
10
8
6
Emp1-TOM high
Emp1-TOM low
c
Cdh1
Epcam
Vim
Twist1
Zeb1
Zeb2
Expression Level
other
mHRCs
d
CDH1
EPCAM
VIM
TWIST1
ZEB1
ZEB2
SNAI1
SNAI2
PRRX1
Expression Level
other
hHRCs
e
EMP1 ISH
LAMC2
Merge
f
EMP1 ISH
KRT17
Merge
EPCAM
g
LAMC2
KRT17
CLDN4
DSC2
JUP
PCDH1
Expression Level
Other
hHRCs

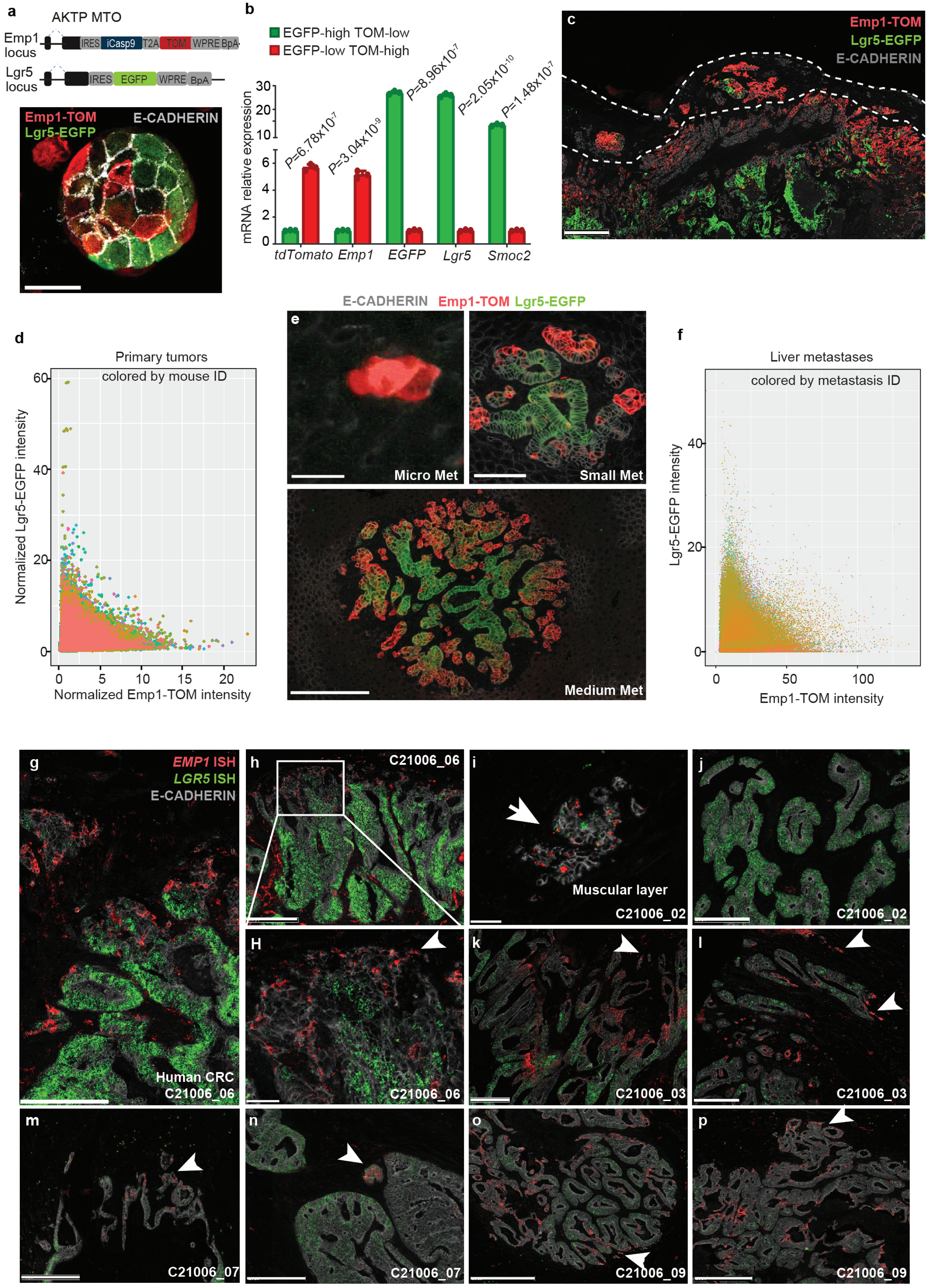

a
AKTP MTO
Emp1 locus
IRES iCasp9 T2A TOM WPRE BpA
Lgr5 locus
IRES EGFP WPRE BpA
Emp1-TOM
Lgr5-EGFP
E-CADHERIN
b
EGFP-high TOM-low
EGFP-low TOM-high
mRNA relative expression
P=6.78x10-7
P=3.04x10-9
P=8.96x10-7
P=2.05x10-10
P=1.48x10-7
tdTomato Emp1 EGFP Lgr5 Smoc2
c
Emp1-TOM
Lgr5-EGFP
E-CADHERIN
d
Primary tumors
colored by mouse ID
Normalized Lgr5-EGFP intensity
Normalized Emp1-TOM intensity
E-CADHERIN Emp1-TOM Lgr5-EGFP
e
Micro Met
Small Met
Medium Met
f
Liver metastases
colored by metastasis ID
Lgr5-EGFP intensity
Emp1-TOM intensity
g
EMP1 ISH
LGR5 ISH
E-CADHERIN
Human CRC
C21006_06
h
C21006_06
i
Muscular layer
C21006_02
j
C21006_02
H
C21006_06
k
C21006_03
l
C21006_03
m
C21006_07
n
C21006_07
o
C21006_09
p
C21006_09

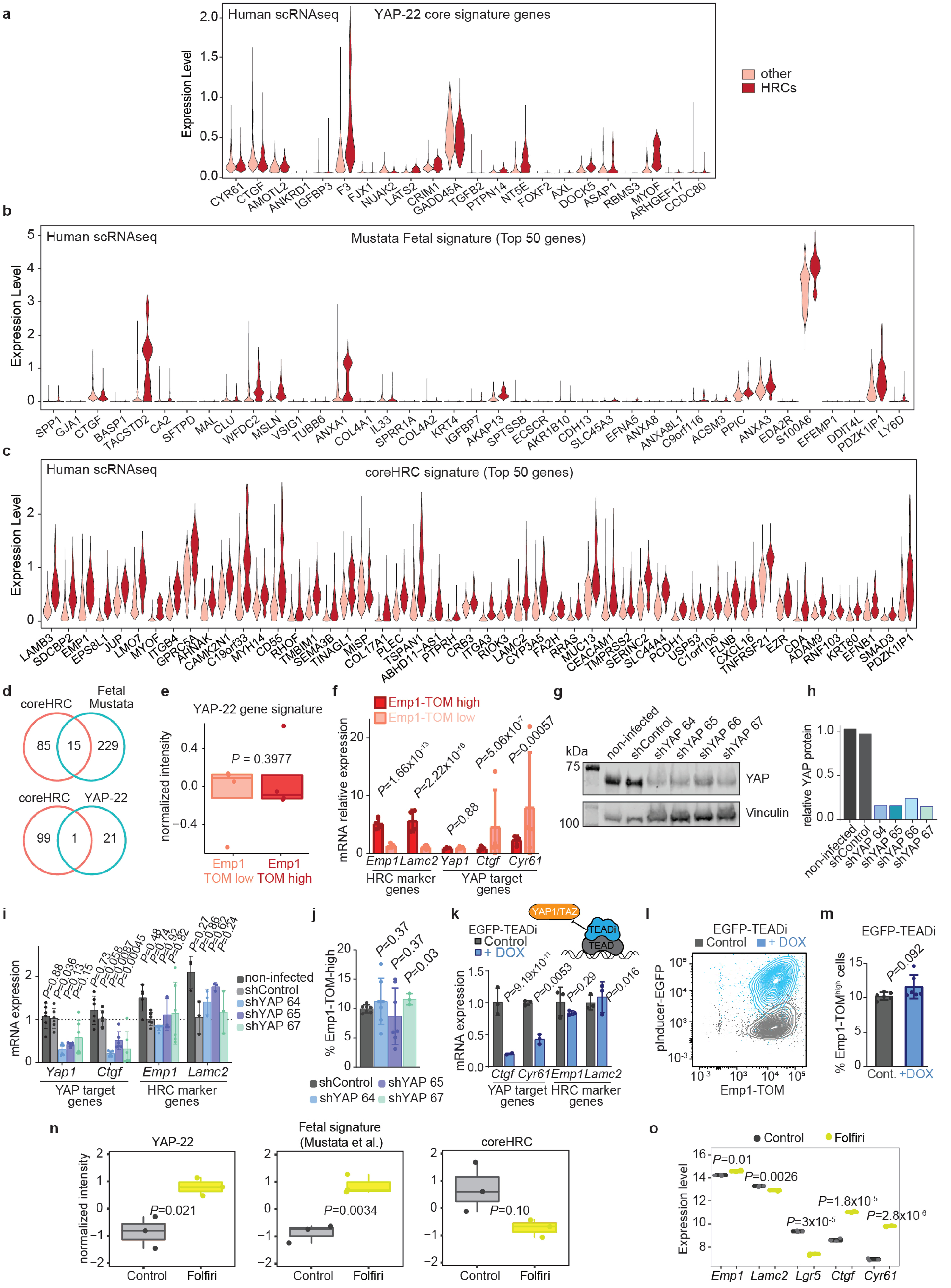
a
Human scRNAseq
YAP-22 core signature genes
Expression Level
other
HRCs
CYR61 CTGF AMOTL2 ANKRD1 IGFBP3 F3 FJX1 NUAK2 LATS2 CRIM1 GADD45A TGFB2 PTPN14 NT5E FOXF2 AXL DOCK5 ASAP1 RBMS3 MYOF ARHGEF17 CCDC80
b
Human scRNAseq
Mustata Fetal signature (Top 50 genes)
Expression Level
SPP1 GJA1 CTGF BASP1 TACSTD2 CA2 SFTPD MAL CLU WFDC2 MSLN VSIG1 TUBB6 ANXA1 COL4A1 IL33 SPRR1A COL4A2 KRT4 IGFBP7 AKAP13 SPTSSB ECSCR AKR1B10 CDH13 SLC45A3 EFNA5 ANXA8 ANXA8L1 C9orf116 ACSM3 PPIC ANXA3 EDA2R S100A6 EFEMP1 DDIT4L PDZK1IP1 LY6D
c
Human scRNAseq
coreHRC signature (Top 50 genes)
Expression Level
LAMB3 SDCBP2 EMP1 EPS8L1 JUP LMO7 MYOF ITGB4 GPRC5A AHNAK CAMK2N1 C19orf33 MYH14 CD55 RHOF TMBIM1 SEMA3B TINAGL1 MISP COL17A1 PLEC TSPAN1 ABHD11-AS1 PTPRH CRB3 ITGA3 RIOK3 LAMC2 CYP3A5 FA2H RRAS MUC13 CEACAM1 TMPRSS2 SERINC2 SLC44A4 PCDH1 USP53 C1orf106 FLNB CXCL16 TNFRSF21 EZR CDA ADAM9 RNF103 KRT80 EFNB1 SMAD3 PDZK1IP1
d
coreHRC
Fetal Mustata
85 15 229
coreHRC
YAP-22
99 1 21
e
YAP-22 gene signature
normalized intensity
P = 0.3977
Emp1 TOM low
Emp1 TOM high
f
Emp1-TOM high
Emp1-TOM low
mRNA relative expression
P=1.66x10^-13
P=2.22x10^-16
P=0.88
P=5.06x10^-7
P=0.00057
Emp1 Lamc2 Yap1 Ctgf Cyr61
HRC marker genes
YAP target genes
g
kDa
75
100
non-infected shControl shYAP 64 shYAP 65 shYAP 66 shYAP 67
YAP
Vinculin
h
relative YAP protein
non-infected shControl shYAP 64 shYAP 65 shYAP 66 shYAP 67
i
mRNA expression
non-infected
shControl
shYAP 64
shYAP 65
shYAP 67
Yap1 Ctgf Emp1 Lamc2
YAP target genes
HRC marker genes
j
% Emp1-TOM-high
P=0.37
P=0.37
P=0.03
shControl shYAP 65
shYAP 64 shYAP 67
k
EGFP-TEADi
Control
+ DOX
YAP1/TAZ
TEADi
TEAD
mRNA expression
P=9.19x10^-11
P=0.0053
P=0.29
P=0.016
Ctgf Cyr61 Emp1 Lamc2
YAP target genes
HRC marker genes
l
EGFP-TEADi
Control
+ DOX
pInducer-EGFP
Emp1-TOM
m
EGFP-TEADi
% Emp1-TOM^high cells
P=0.092
Cont. +DOX
n
YAP-22
normalized intensity
P=0.021
Control Folfiri
Fetal signature (Mustata et al.)
P=0.0034
Control Folfiri
coreHRC
P=0.10
Control Folfiri
o
Control
Folfiri
Expression level
P=0.01
P=0.0026
P=3x10^-5
P=1.8x10^-5
P=2.8x10^-6
Emp1 Lamc2 Lgr5 Ctgf Cyr61

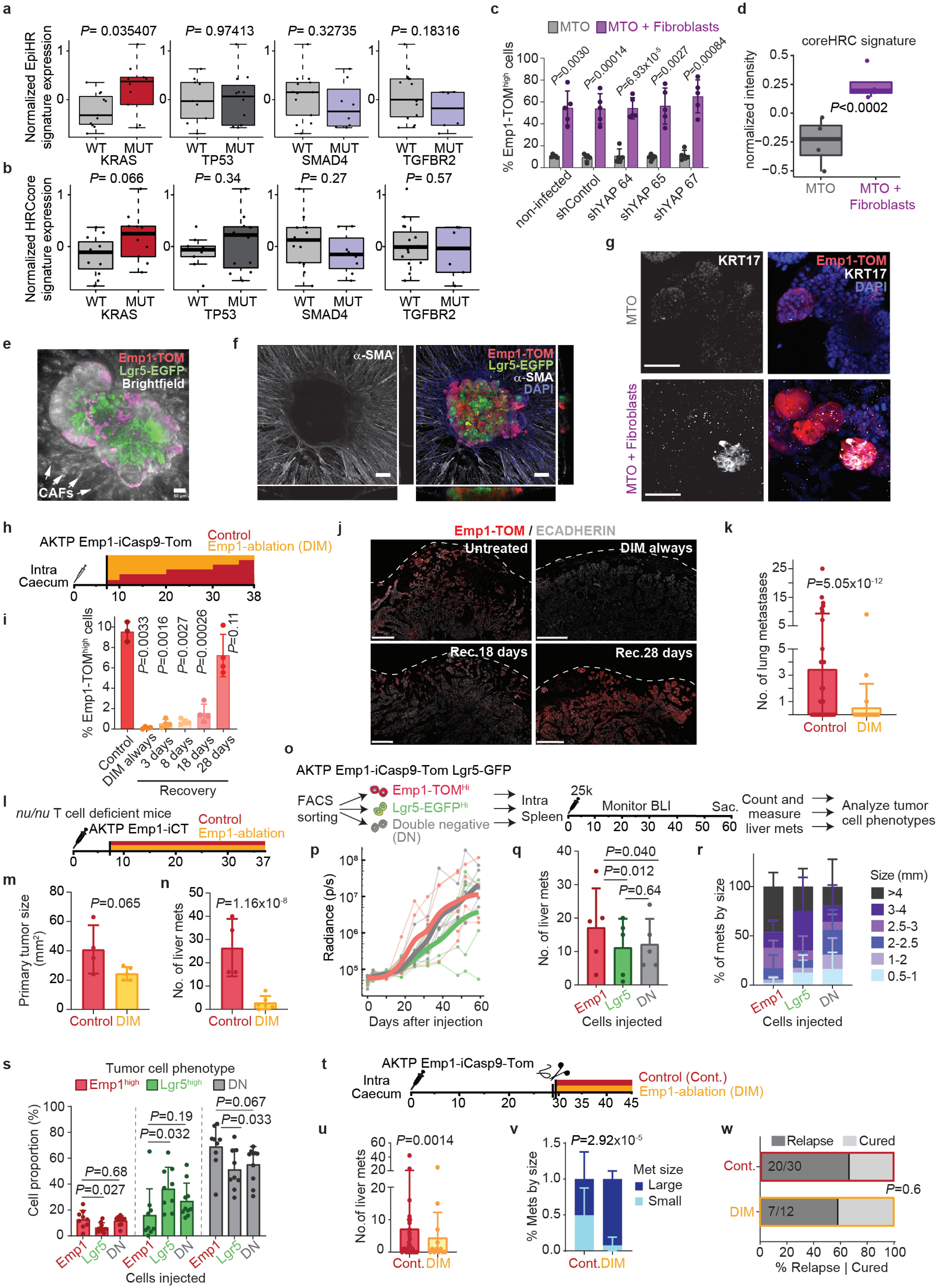

a
Normalized EpiHR signature expression
P= 0.035407
P= 0.97413
P= 0.32735
P= 0.18316
WT MUT KRAS
WT MUT TP53
WT MUT SMAD4
WT MUT TGFBR2
b
Normalized HRCcore signature expression
P= 0.066
P= 0.34
P= 0.27
P= 0.57
WT MUT KRAS
WT MUT TP53
WT MUT SMAD4
WT MUT TGFBR2
c
MTO
MTO + Fibroblasts
% Emp1-TOMhigh cells
P=0.0030
P=0.00014
P=6.93x10-5
P=0.0027
P=0.00084
non-infected
shControl
shYAP 64
shYAP 65
shYAP 67
d
coreHRC signature
normalized intensity
P<0.0002
MTO
MTO + Fibroblasts
e
Emp1-TOM
Lgr5-EGFP
Brightfield
CAFs
50 µm
f
α-SMA
Emp1-TOM
Lgr5-EGFP
α-SMA
DAPI
g
KRT17
Emp1-TOM
KRT17
DAPI
MTO
MTO + Fibroblasts
h
AKTP Emp1-iCasp9-Tom
Control
Emp1-ablation (DIM)
Intra Caecum
0 10 20 30 38
i
% Emp1-TOMhigh cells
P=0.0033
P=0.0016
P=0.0027
P=0.00026
P=0.11
Control
DIM always
3 days
8 days
18 days
28 days
Recovery
j
Emp1-TOM / ECADHERIN
Untreated
DIM always
Rec.18 days
Rec.28 days
k
No. of lung metastases
P=5.05x10-12
Control
DIM
l
nu/nu T cell deficient mice
AKTP Emp1-iCT
Control
Emp1-ablation
0 10 20 30 37
m
Primary tumor size (mm2)
P=0.065
Control DIM
n
No. of liver mets
P=1.16x10-8
Control DIM
o
AKTP Emp1-iCasp9-Tom Lgr5-GFP
FACS sorting
Emp1-TOMHi
Lgr5-EGFPHi
Double negative (DN)
Intra Spleen
25k
Monitor BLI
Sac.
0 10 20 30 40 50 60
Count and measure liver mets
Analyze tumor cell phenotypes
p
Radiance (p/s)
Days after injection
q
No. of liver mets
P=0.040
P=0.012
P=0.64
Emp1 Lgr5 DN
Cells injected
r
% of mets by size
Size (mm)
>4
3-4
2.5-3
2-2.5
1-2
0.5-1
Emp1 Lgr5 DN
Cells injected
s
Tumor cell phenotype
Emp1high
Lgr5high
DN
Cell proportion (%)
P=0.68
P=0.027
P=0.19
P=0.032
P=0.067
P=0.033
Emp1 Lgr5 DN
Cells injected
t
AKTP Emp1-iCasp9-Tom
Intra Caecum
Control (Cont.)
Emp1-ablation (DIM)
0 10 20 30 35 40 45
u
No.of liver mets
P=0.0014
Cont. DIM
v
% Mets by size
P=2.92x10-5
Met size
Large
Small
Cont. DIM
w
Relapse
Cured
Cont.
20/30
DIM
7/12
P=0.6
% Relapse | Cured

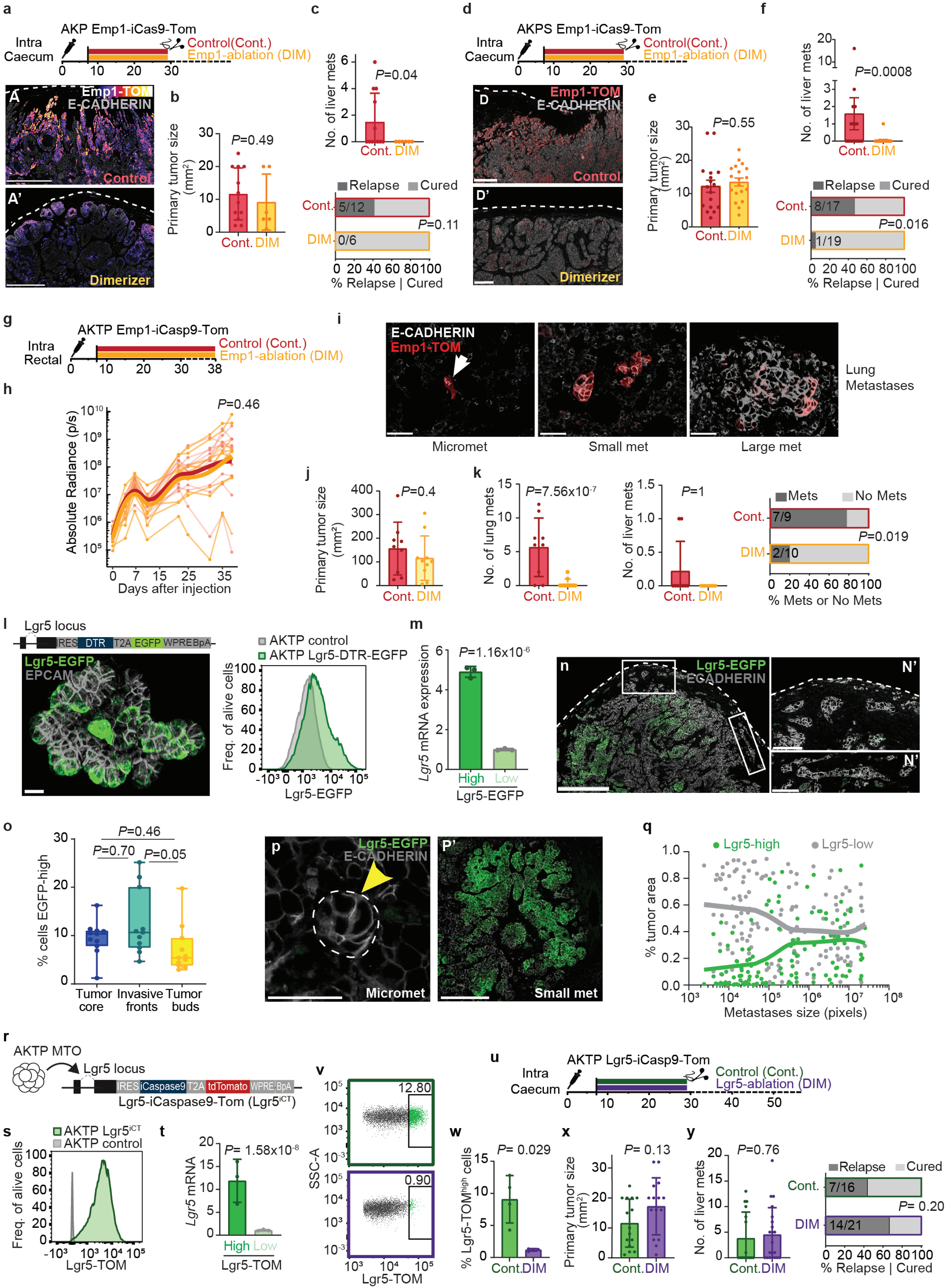
a
AKP Emp1-iCas9-Tom
Intra Caecum
Control(Cont.)
Emp1-ablation (DIM)
0 10 20 30
Emp1-TOM
E-CADHERIN
Control
Dimerizer
b
Primary tumor size (mm²)
P=0.49
Cont. DIM
c
No. of liver mets
P=0.04
Relapse Cured
Cont. 5/12
DIM 0/6
P=0.11
% Relapse | Cured
d
AKPS Emp1-iCas9-Tom
Intra Caecum
Control(Cont.)
Emp1-ablation (DIM)
e
Primary tumor size (mm²)
P=0.55
f
No. of liver mets
P=0.0008
Cont. 8/17
DIM 1/19
P=0.016
% Relapse | Cured
g
AKTP Emp1-iCasp9-Tom
Intra Rectal
Control (Cont.)
Emp1-ablation (DIM)
0 10 20 30 38
h
Absolute Radiance (p/s)
P=0.46
Days after injection
i
E-CADHERIN
Emp1-TOM
Lung Metastases
Micromet
Small met
Large met
j
Primary tumor size (mm²)
P=0.4
k
No. of lung mets
P=7.56x10⁻⁷
No. of liver mets
P=1
Mets No Mets
Cont. 7/9
DIM 2/10
P=0.019
% Mets or No Mets
l
Lgr5 locus
IRES DTR T2A EGFP WPRE BpA
Lgr5-EGFP
EPCAM
AKTP control
AKTP Lgr5-DTR-EGFP
Freq. of alive cells
Lgr5-EGFP
m
Lgr5 mRNA expression
P=1.16x10⁻⁶
High Low
Lgr5-EGFP
n
Lgr5-EGFP
E-CADHERIN
N'
o
% cells EGFP-high
P=0.46
P=0.70
P=0.05
Tumor core
Invasive fronts
Tumor buds
p
Lgr5-EGFP
E-CADHERIN
Micromet
P'
Small met
q
Lgr5-high
Lgr5-low
% tumor area
Metastases size (pixels)
r
AKTP MTO
Lgr5 locus
IRES iCaspase9 T2A tdTomato WPRE BpA
Lgr5-iCaspase9-Tom (Lgr5^iCT)
s
AKTP Lgr5^iCT
AKTP control
Freq. of alive cells
Lgr5-TOM
t
Lgr5 mRNA
P= 1.58x10⁻⁸
High Low
Lgr5-TOM
u
AKTP Lgr5-iCasp9-Tom
Intra Caecum
Control (Cont.)
Lgr5-ablation (DIM)
0 10 20 30 40 50
v
SSC-A
12.80
0.90
Lgr5-TOM
w
% Lgr5-TOM^high cells
P= 0.029
x
Primary tumor size (mm²)
P= 0.13
y
No. of liver mets
P=0.76
Relapse Cured
Cont. 7/16
DIM 14/21
P= 0.20
% Relapse | Cured

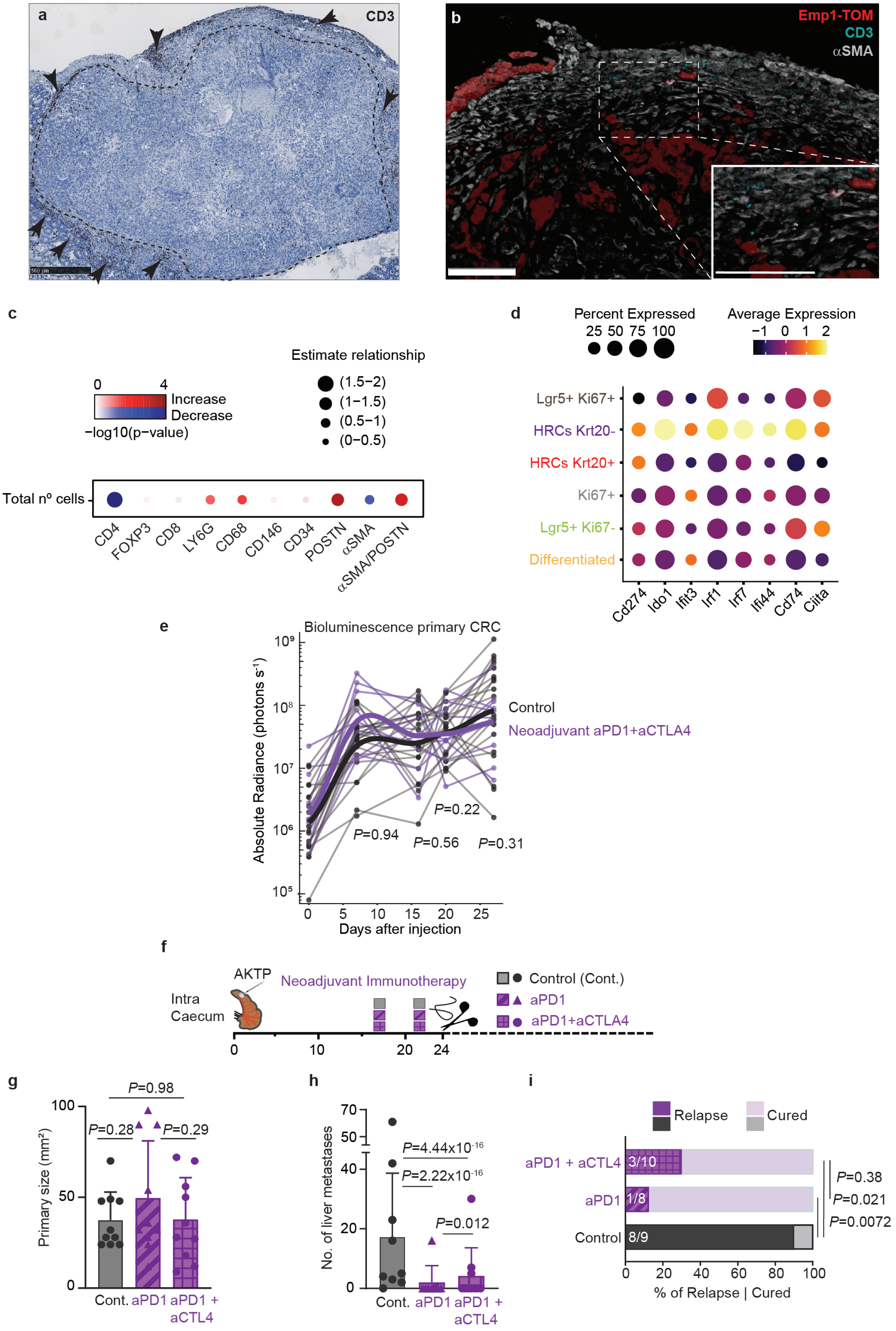
a
CD3
b
Emp1-TOM
CD3
αSMA
c
0
4
Increase
Decrease
−log10(p−value)
Estimate relationship
(1.5−2)
(1−1.5)
(0.5−1)
(0−0.5)
Total nº cells
CD4
FOXP3
CD8
LY6G
CD68
CD146
CD34
POSTN
αSMA
αSMA/POSTN
d
Percent Expressed
25 50 75 100
Average Expression
−1 0 1 2
Lgr5+ Ki67+
HRCs Krt20-
HRCs Krt20+
Ki67+
Lgr5+ Ki67-
Differentiated
Cd274
Ido1
Ifit3
Irf1
Irf7
Ifi44
Cd74
Ciita
e
Bioluminescence primary CRC
Absolute Radiance (photons s⁻¹)
Control
Neoadjuvant aPD1+aCTLA4
P=0.94
P=0.56
P=0.22
P=0.31
Days after injection
f
AKTP
Intra Caecum
Neoadjuvant Immunotherapy
Control (Cont.)
aPD1
aPD1+aCTLA4
0
10
20
24
g
Primary size (mm²)
P=0.98
P=0.28
P=0.29
Cont.
aPD1
aPD1 + aCTL4
h
No. of liver metastases
P=4.44x10⁻¹⁶
P=2.22x10⁻¹⁶
P=0.012
Cont.
aPD1
aPD1 + aCTL4
i
Relapse
Cured
aPD1 + aCTL4
3/10
aPD1
1/8
Control
8/9
P=0.38
P=0.021
P=0.0072
% of Relapse | Cured

Main Figures together

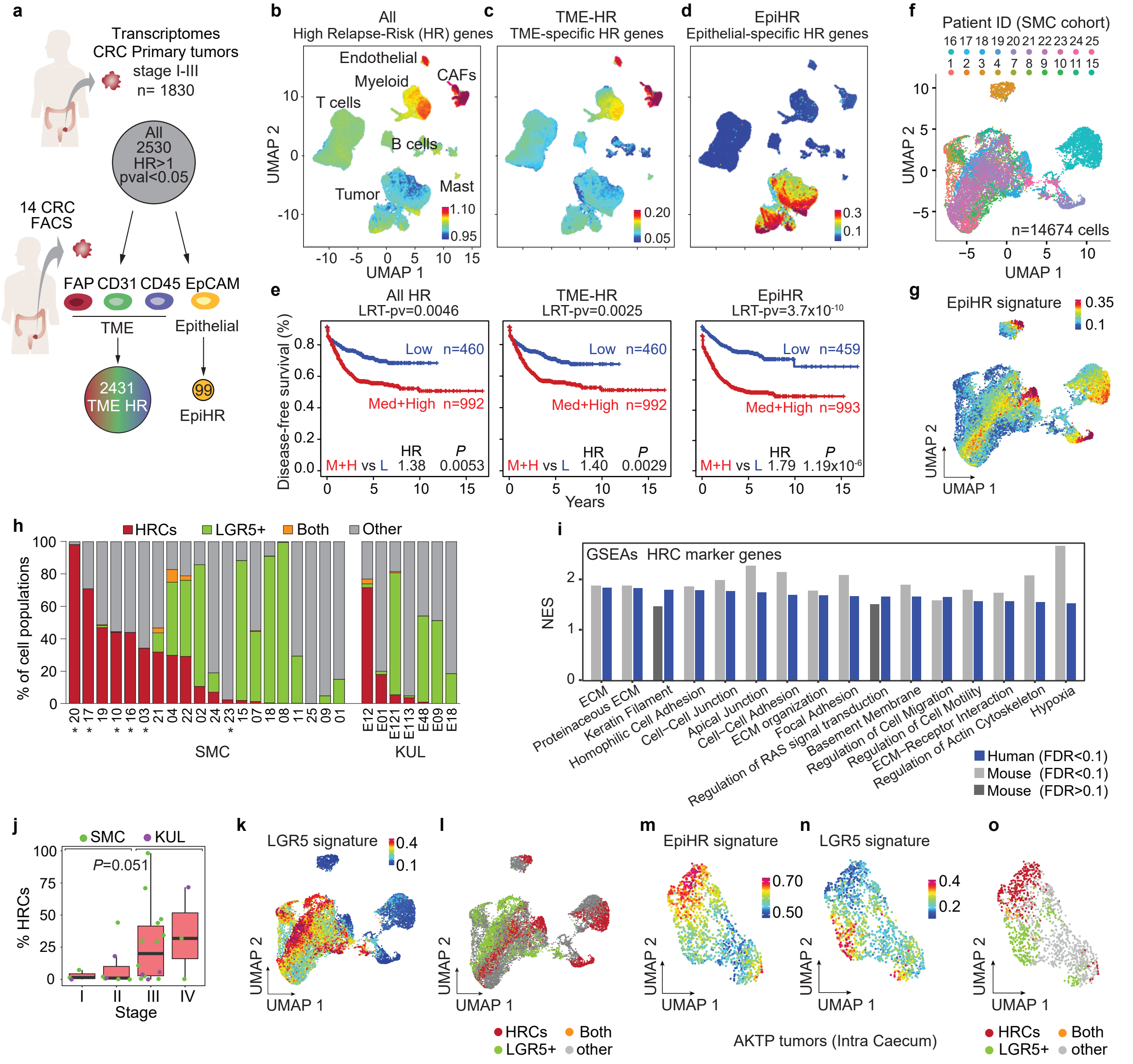
a
Transcriptomes
CRC Primary tumors
stage I-III
n= 1830
All
2530
HR>1
pval<0.05
14 CRC
FACS
FAP CD31 CD45 EpCAM
TME
Epithelial
2431
TME HR
99
EpiHR
b
All
High Relapse-Risk (HR) genes
Endothelial
Myeloid
CAFs
T cells
B cells
Tumor
Mast
1.10
0.95
UMAP 2
UMAP 1
c
TME-HR
TME-specific HR genes
0.20
0.05
d
EpiHR
Epithelial-specific HR genes
0.3
0.1
f
Patient ID (SMC cohort)
16 17 18 19 20 21 22 23 24 25
1 2 3 4 7 8 9 10 11 15
n=14674 cells
UMAP 2
UMAP 1
e
All HR
LRT-pv=0.0046
TME-HR
LRT-pv=0.0025
EpiHR
LRT-pv=3.7x10-10
Disease-free survival (%)
Low n=460
Med+High n=992
Low n=460
Med+High n=992
Low n=459
Med+High n=993
M+H vs L HR 1.38 P 0.0053
M+H vs L HR 1.40 P 0.0029
M+H vs L HR 1.79 P 1.19x10-6
Years
g
EpiHR signature
0.35
0.1
UMAP 2
UMAP 1
h
HRCs
LGR5+
Both
Other
% of cell populations
20 17 19 10 16 03 21 04 22 02 24 23 15 07 18 08 11 25 09 01
E12 E01 E121 E113 E48 E09 E18
SMC
KUL
i
GSEAs HRC marker genes
NES
ECM
Proteinaceous ECM
Keratin Filament
Homophilic Cell Adhesion
Cell−Cell Junction
Apical Junction
Cell−Cell Adhesion
ECM organization
Focal Adhesion
Regulation of RAS signal transduction
Basement Membrane
Regulation of Cell Migration
Regulation of Cell Motility
ECM−Receptor Interaction
Regulation of Actin Cytoskeleton
Hypoxia
Human (FDR<0.1)
Mouse (FDR<0.1)
Mouse (FDR>0.1)
j
SMC
KUL
P=0.051
% HRCs
Stage
k
LGR5 signature
0.4
0.1
UMAP 2
UMAP 1
l
UMAP 2
UMAP 1
HRCs
Both
LGR5+
other
m
EpiHR signature
0.70
0.50
n
LGR5 signature
0.4
0.2
o
UMAP 2
UMAP 1
AKTP tumors (Intra Caecum)
HRCs
Both
LGR5+
other

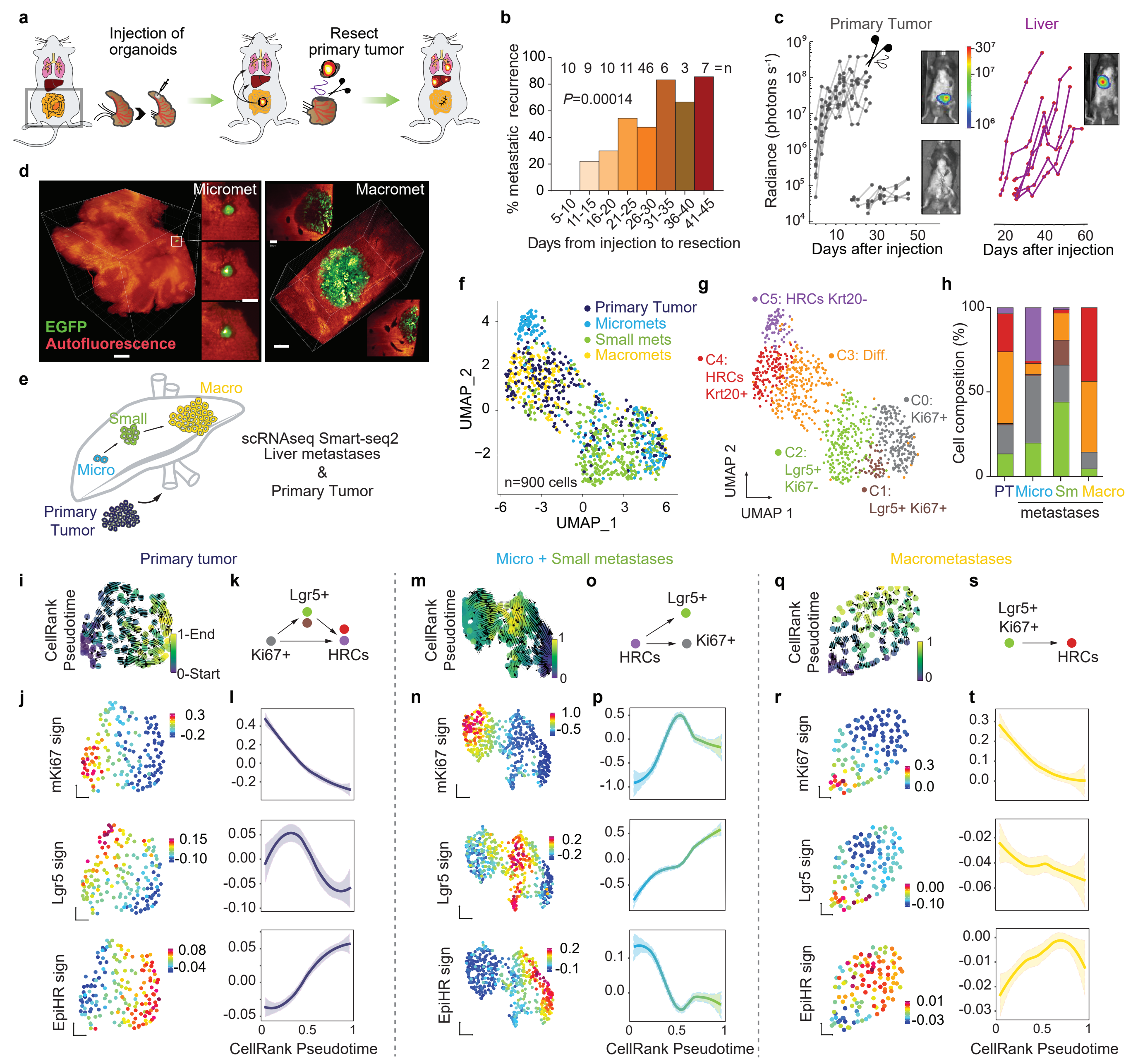

a
Injection of organoids
Resect primary tumor
b
% metastatic recurrence
10 9 10 11 46 6 3 7 =n
P=0.00014
5-10 11-15 16-20 21-25 26-30 31-35 36-40 41-45
Days from injection to resection
c
Primary Tumor
Liver
Radiance (photons s⁻¹)
Days after injection
d
Micromet
Macromet
EGFP
Autofluorescence
e
Macro
Small
Micro
Primary Tumor
scRNAseq Smart-seq2
Liver metastases
&
Primary Tumor
f
Primary Tumor
Micromets
Small mets
Macromets
n=900 cells
UMAP_1
UMAP_2
g
C5: HRCs Krt20-
C4: HRCs Krt20+
C3: Diff.
C0: Ki67+
C2: Lgr5+ Ki67-
C1: Lgr5+ Ki67+
UMAP 1
UMAP 2
h
Cell composition (%)
PT Micro Sm Macro
metastases
Primary tumor
Micro + Small metastases
Macrometastases
i
CellRank Pseudotime
1-End
0-Start
k
Lgr5+
Ki67+
HRCs
m
CellRank Pseudotime
o
Lgr5+
HRCs
Ki67+
q
CellRank Pseudotime
s
Lgr5+
Ki67+
HRCs
j
mKi67 sign
Lgr5 sign
EpiHR sign
l
n
mKi67 sign
Lgr5 sign
EpiHR sign
p
r
mKi67 sign
Lgr5 sign
EpiHR sign
t
CellRank Pseudotime
CellRank Pseudotime
CellRank Pseudotime

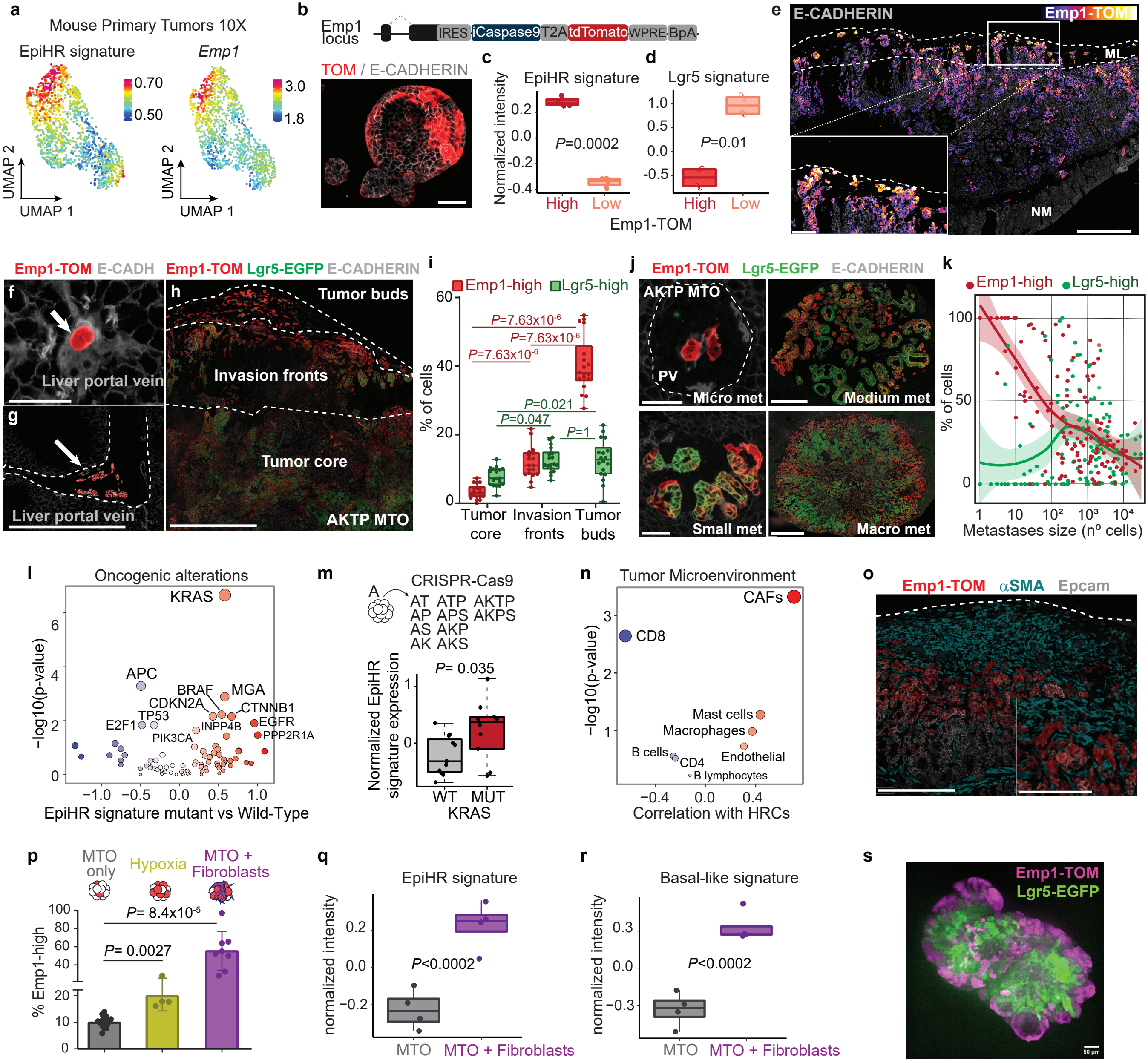

a
Mouse Primary Tumors 10X
EpiHR signature
Emp1
0.70
0.50
3.0
1.8
UMAP 2
UMAP 1
b
Emp1 locus
IRES iCaspase9 T2A tdTomato WPRE BpA
TOM / E-CADHERIN
c
EpiHR signature
Normalized intensity
P=0.0002
High Low
d
Lgr5 signature
P=0.01
High Low
Emp1-TOM
e
E-CADHERIN
Emp1-TOM
ML
NM
f
Emp1-TOM E-CADH
Liver portal vein
g
Liver portal vein
h
Emp1-TOM Lgr5-EGFP E-CADHERIN
Tumor buds
Invasion fronts
Tumor core
AKTP MTO
i
Emp1-high Lgr5-high
% of cells
P=7.63x10-6
P=0.021
P=0.047
P=1
Tumor core
Invasion fronts
Tumor buds
j
Emp1-TOM Lgr5-EGFP E-CADHERIN
AKTP MTO
PV
Micro met
Medium met
Small met
Macro met
k
Emp1-high Lgr5-high
% of cells
Metastases size (nº cells)
l
Oncogenic alterations
KRAS
APC
BRAF
MGA
CDKN2A
CTNNB1
TP53
E2F1
EGFR
INPP4B
PIK3CA
PPP2R1A
-log10(p-value)
EpiHR signature mutant vs Wild-Type
m
CRISPR-Cas9
A
AT ATP AKTP
AP APS AKPS
AS AKP
AK AKS
P= 0.035
Normalized EpiHR signature expression
WT MUT
KRAS
n
Tumor Microenvironment
CAFs
CD8
Mast cells
Macrophages
B cells
CD4
Endothelial
B lymphocytes
-log10(p-value)
Correlation with HRCs
o
Emp1-TOM αSMA Epcam
p
MTO only
Hypoxia
MTO + Fibroblasts
P= 8.4x10-5
P= 0.0027
% Emp1-high
q
EpiHR signature
normalized intensity
P<0.0002
MTO MTO + Fibroblasts
r
Basal-like signature
normalized intensity
P<0.0002
MTO MTO + Fibroblasts
s
Emp1-TOM
Lgr5-EGFP
50 μm

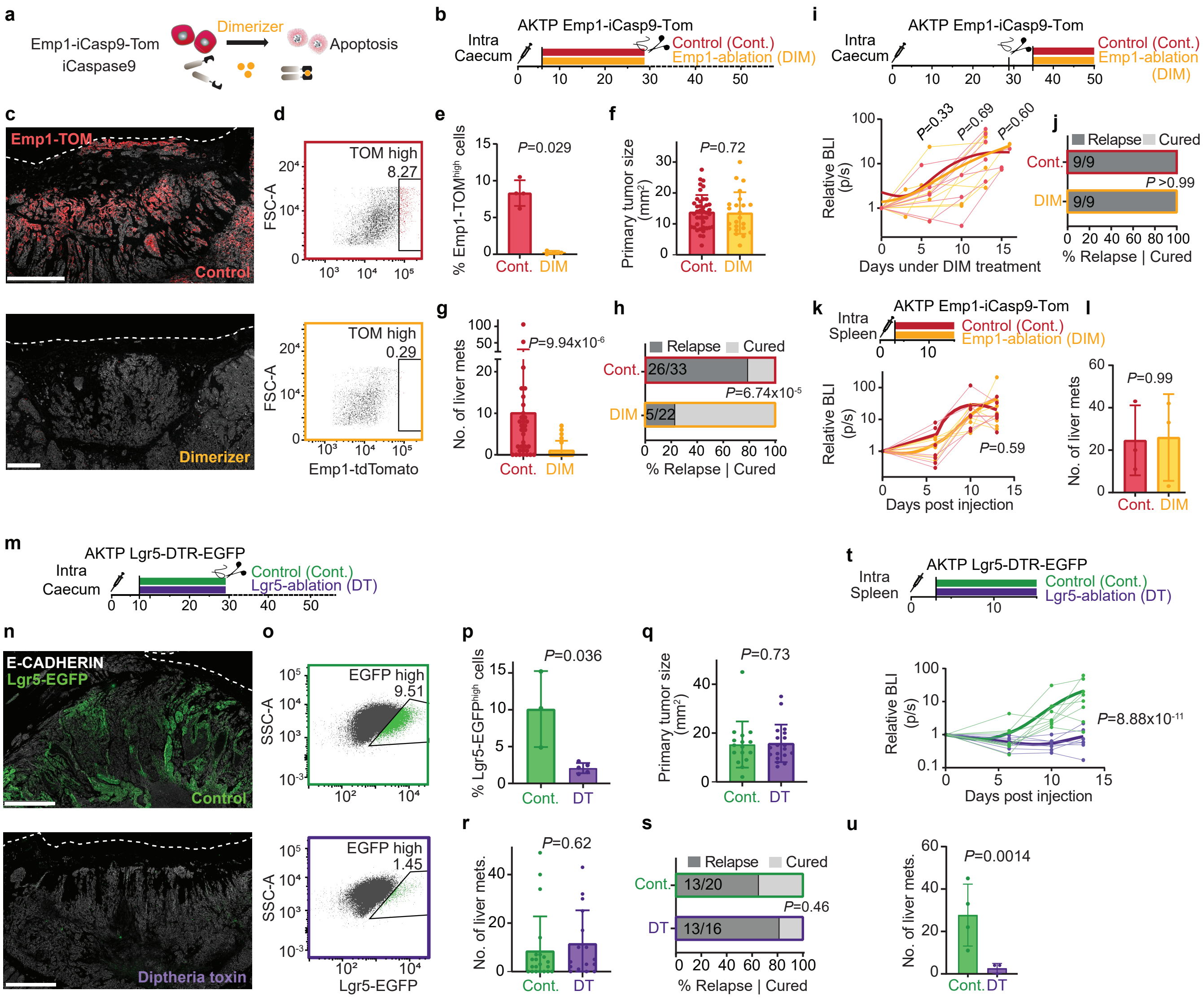
a
Emp1-iCasp9-Tom
iCaspase9
Dimerizer
Apoptosis
b
AKTP Emp1-iCasp9-Tom
Intra Caecum
Control (Cont.)
Emp1-ablation (DIM)
i
AKTP Emp1-iCasp9-Tom
Intra Caecum
Control (Cont.)
Emp1-ablation (DIM)
c
Emp1-TOM
Control
Dimerizer
d
TOM high 8.27
TOM high 0.29
FSC-A
Emp1-tdTomato
e
P=0.029
% Emp1-TOMhigh cells
Cont. DIM
f
P=0.72
Primary tumor size (mm2)
Cont. DIM
P=0.33
P=0.69
P=0.60
Relative BLI (p/s)
Days under DIM treatment
j
Relapse Cured
Cont. 9/9
P >0.99
DIM 9/9
% Relapse | Cured
g
P=9.94x10-6
No. of liver mets
Cont. DIM
h
Relapse Cured
Cont. 26/33
P=6.74x10-5
DIM 5/22
% Relapse | Cured
k
AKTP Emp1-iCasp9-Tom
Intra Spleen
Control (Cont.)
Emp1-ablation (DIM)
P=0.59
Relative BLI (p/s)
Days post injection
l
P=0.99
No. of liver mets
Cont. DIM
m
AKTP Lgr5-DTR-EGFP
Intra Caecum
Control (Cont.)
Lgr5-ablation (DT)
n
E-CADHERIN
Lgr5-EGFP
Control
Diptheria toxin
o
EGFP high 9.51
EGFP high 1.45
SSC-A
Lgr5-EGFP
p
P=0.036
% Lgr5-EGFPhigh cells
Cont. DT
q
P=0.73
Primary tumor size (mm2)
Cont. DT
r
P=0.62
No. of liver mets.
Cont. DT
s
Relapse Cured
Cont. 13/20
P=0.46
DT 13/16
% Relapse | Cured
t
AKTP Lgr5-DTR-EGFP
Intra Spleen
Control (Cont.)
Lgr5-ablation (DT)
P=8.88x10-11
Relative BLI (p/s)
Days post injection
u
P=0.0014
No. of liver mets.
Cont. DT

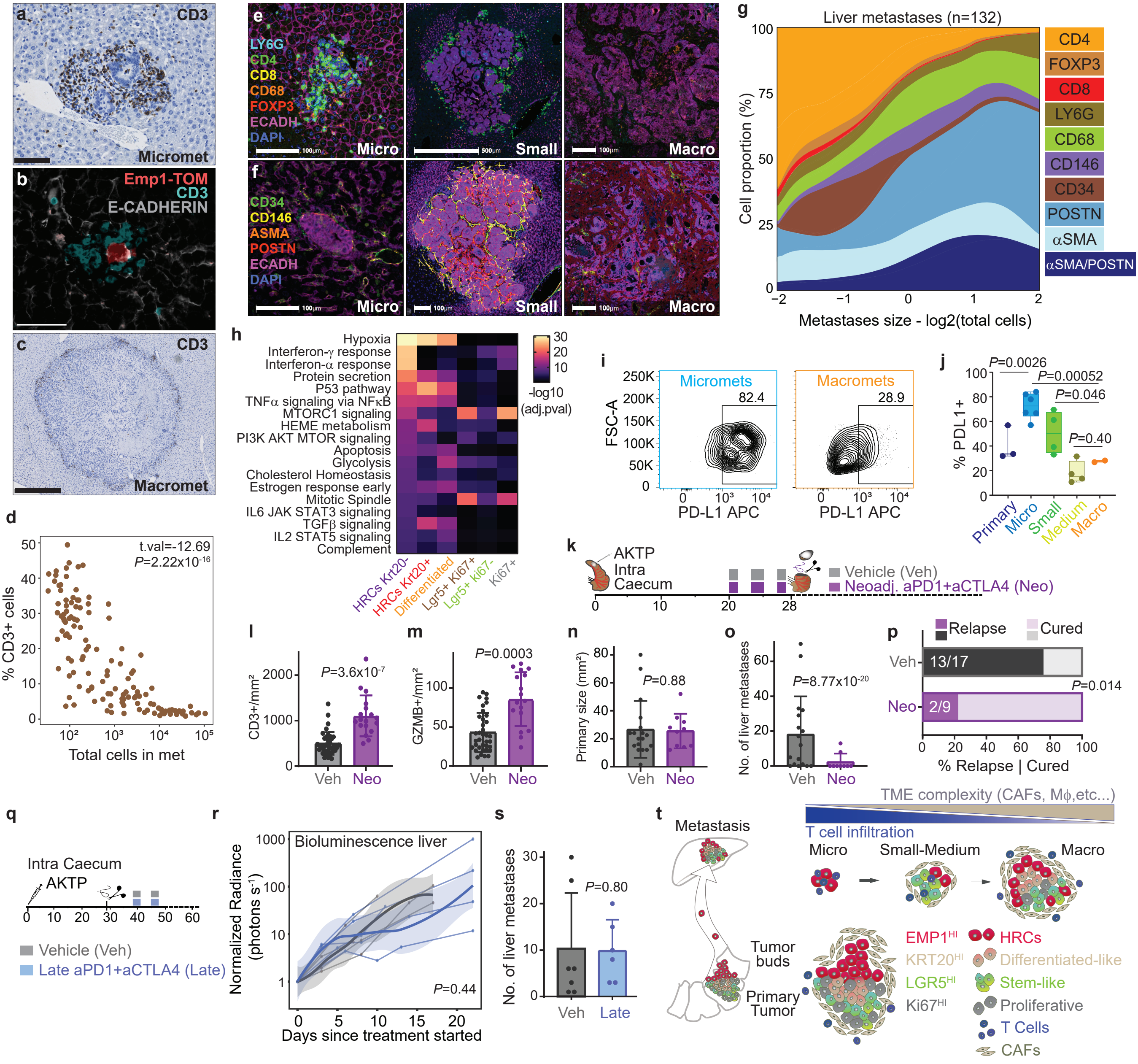

a
CD3
Micromet
b
Emp1-TOM
CD3
E-CADHERIN
c
CD3
Macromet
d
t.val=-12.69
P=2.22x10-16
% CD3+ cells
Total cells in met
e
LY6G
CD4
CD8
CD68
FOXP3
ECADH
DAPI
Micro
Small
Macro
f
CD34
CD146
ASMA
POSTN
ECADH
DAPI
Micro
Small
Macro
g
Liver metastases (n=132)
Cell proportion (%)
Metastases size - log2(total cells)
CD4
FOXP3
CD8
LY6G
CD68
CD146
CD34
POSTN
αSMA
αSMA/POSTN
h
Hypoxia
Interferon-γ response
Interferon-α response
Protein secretion
P53 pathway
TNFα signaling via NFκB
MTORC1 signaling
HEME metabolism
PI3K AKT MTOR signaling
Apoptosis
Glycolysis
Cholesterol Homeostasis
Estrogen response early
Mitotic Spindle
IL6 JAK STAT3 signaling
TGFβ signaling
IL2 STAT5 signaling
Complement
-log10 (adj.pval)
HRCs Krt20-
HRCs Krt20+
Differentiated
Lgr5+ Ki67+
Lgr5+ ki67-
Ki67+
i
FSC-A
Micromets
82.4
Macromets
28.9
PD-L1 APC
j
P=0.0026
P=0.00052
P=0.046
P=0.40
% PDL1+
Primary
Micro
Small
Medium
Macro
k
AKTP Intra Caecum
Vehicle (Veh)
Neoadj. aPD1+aCTLA4 (Neo)
l
CD3+/mm²
P=3.6x10-7
Veh
Neo
m
GZMB+/mm²
P=0.0003
Veh
Neo
n
Primary size (mm²)
P=0.88
Veh
Neo
o
No. of liver metastases
P=8.77x10-20
Veh
Neo
p
Relapse
Cured
Veh
13/17
Neo
2/9
P=0.014
% Relapse | Cured
q
Intra Caecum
AKTP
Vehicle (Veh)
Late aPD1+aCTLA4 (Late)
r
Bioluminescence liver
Normalized Radiance (photons s-1)
P=0.44
Days since treatment started
s
No. of liver metastases
P=0.80
Veh
Late
t
Metastasis
Tumor buds
Primary Tumor
TME complexity (CAFs, Mϕ,etc...)
T cell infiltration
Micro
Small-Medium
Macro
EMP1HI
KRT20HI
LGR5HI
Ki67HI
HRCs
Differentiated-like
Stem-like
Proliferative
T Cells
CAFs

Supplementary Table 2. EpiHR and coreHRC signatures

gene	Correlation with EpiHR<br>mean_correlation_SMC_cohort	mean_correlation_KUL_cohort	epiHR	cluster (Ext data Fig 1h)	core_HRC
LAMB3	0,918653386	0,861439026	1	cl1	1
SDCBP2	0,870061556	0,823929125	0	N.A.	1
EMP1	0,869356026	0,853861678	0	N.A.	1
EPS8L1	0,836992759	0,856944919	1	cl1	1
JUP	0,829972039	0,899397863	0	N.A.	1
LMO7	0,823560828	0,812490924	1	cl1	1
MYOF	0,822877259	0,74822594	0	N.A.	1
ITGB4	0,822413043	0,899083395	0	N.A.	1
GPRC5A	0,818474231	0,946567465	1	cl1	1
AHNAK	0,81310242	0,830276862	0	N.A.	1
CAMK2N1	0,810238117	0,862067215	1	cl1	1
C19orf33	0,8097437	0,767690421	1	cl1	1
MYH14	0,809364727	0,855092786	0	N.A.	1
CD55	0,80690352	0,77916496	0	N.A.	1
RHOF	0,806003022	0,637239125	0	N.A.	1
TMBIM1	0,802892765	0,908537459	0	N.A.	1
SEMA3B	0,800274517	0,887833564	0	N.A.	1
TINAGL1	0,791494665	0,779612514	0	N.A.	1
MISP	0,785808519	0,866383456	0	N.A.	1
COL17A1	0,784847953	0,831061603	1	cl1	1
PLEC	0,783177503	0,845630555	0	N.A.	1
TSPAN1	0,771369807	0,816608635	0	N.A.	1
ABHD11-AS1	0,770624757	0,776251399	0	N.A.	1
PTPRH	0,767578857	0,743807169	0	N.A.	1
CRB3	0,764646664	0,798754211	0	N.A.	1
ITGA3	0,76369857	0,922465825	1	cl1	1
RIOK3	0,762565768	0,756617101	0	N.A.	1
LAMC2	0,761827702	0,905559464	1	cl1	1
CYP3A5	0,760263416	0,819324022	0	N.A.	1

FA2H	0,757944389	0,757118121	0	N.A.	1
RRAS	0,757487095	0,804626606	0	N.A.	1
MUC13	0,755270309	0,791379923	0	N.A.	1
CEACAM1	0,755035049	0,848885407	0	N.A.	1
TMPRSS2	0,754313468	0,753969711	0	N.A.	1
SERINC2	0,754152646	0,817795977	0	N.A.	1
SLC44A4	0,752518672	0,656279333	0	N.A.	1
PCDH1	0,752337117	0,845915809	0	N.A.	1
USP53	0,747713417	0,686489812	0	N.A.	1
C1orf106	0,747662691	0,888007035	0	N.A.	1
FLNB	0,746497922	0,79819095	0	N.A.	1
CXCL16	0,744205325	0,736461602	0	N.A.	1
EZR	0,741632706	0,857506788	0	N.A.	1
TNFRSF21	0,738220623	0,740539549	1	cl1	1
CDA	0,737916406	0,804733531	0	N.A.	1
ADAM9	0,730916623	0,856877216	0	N.A.	1
RNF103	0,728303109	0,486469264	0	N.A.	1
KRT80	0,72743271	0,78670345	1	cl1	1
EFNB1	0,726904417	0,799497428	0	N.A.	1
SMAD3	0,725842808	0,7311038	0	N.A.	1
PDZK1IP1	0,724035065	0,721319521	0	N.A.	1
LRP10	0,723782727	0,806901391	0	N.A.	1
PRSS22	0,721948755	0,898367891	1	cl1	1
AHNAK2	0,720598585	0,794526483	0	N.A.	1
B4GALT1	0,720192625	0,516047798	0	N.A.	1
ANXA11	0,718159193	0,789630652	0	N.A.	1
BAIAP2L1	0,71727505	0,663589727	0	N.A.	1
F11R	0,717251787	0,786237991	0	N.A.	1
DUSP5	0,715463655	0,670896026	0	N.A.	1
DDR1	0,715281511	0,680808704	1	cl1	1
ITGB1	0,712882098	0,711200456	0	N.A.	1
SLC2A1	0,711780311	0,850531314	1	cl1	1
CDKN2B	0,711741628	0,732315104	0	N.A.	1

PLAUR	0,710314441	0,709668478	0	N.A.	1
ITGA2	0,709881919	0,772861781	0	N.A.	1
CDCP1	0,708977938	0,828555373	0	N.A.	1
CD59	0,708669898	0,713700024	0	N.A.	1
DSG2	0,70853198	0,769673499	0	N.A.	1
KLK10	0,708278231	0,863073385	1	cl1	1
ARFGAP3	0,707661508	0,749148046	0	N.A.	1
CD68	0,707248274	0,558115661	0	N.A.	1
CGN	0,70575851	0,822156848	0	N.A.	1
TM4SF1	0,705057034	0,784856608	0	N.A.	1
PPP2CB	0,704963604	0,67509898	0	N.A.	1
HEBP2	0,703978349	0,741815551	0	N.A.	1
UGCG	0,703955227	0,473611497	0	N.A.	1
GBP3	0,703710221	0,662409289	0	N.A.	1
TIMP2	0,701193378	0,683523798	0	N.A.	1
PINK1	0,701133054	0,662597026	0	N.A.	1
CYSTM1	0,700765159	0,535818109	0	N.A.	1
SLC6A8	0,700072171	0,784960057	0	N.A.	1
PPARD	0,699813197	0,421439735	0	N.A.	1
LIPH	0,6987803	0,791527997	0	N.A.	1
CLIC3	0,698171106	0,864836063	0	N.A.	1
SPINT1	0,698021957	0,865446495	1	cls3	1
PTK6	0,697775229	0,744819452	0	N.A.	1
TJP3	0,697641745	0,791192001	0	N.A.	1
STK24	0,696212151	0,815624092	0	N.A.	1
AC009133.12	0,695725517	0,570300342	0	N.A.	1
CEACAM5	0,695434213	0,716394721	0	N.A.	1
MXD1	0,695423592	0,622895742	0	N.A.	1
SNX9	0,694845139	0,703349506	0	N.A.	1
ERO1L	0,694369584	0,81619661	0	N.A.	1
SSFA2	0,693021808	0,670818297	0	N.A.	1
TSPAN3	0,691835542	0,744709847	0	N.A.	1
SLPI	0,691534114	0,822569433	1	cls3	1

BMP2	0,691503031	0,645618766	0	N.A.	1
GJB3	0,690683474	0,803749668	1	cl1	1
LAMA3	0,688601269	0,833874229	1	cl1	1
TMPRSS4	0,687531791	0,770908911	0	N.A.	1
B4GALNT3	0,687261423	0,539818172	0	N.A.	1
TNIP1	0,686091907	0,649657294	0	N.A.	0
CDHR5	0,685947514	0,806702298	0	N.A.	0
POLD4	0,683756701	0,592071689	0	N.A.	0
GDA	0,68126987	0,657050931	0	N.A.	0
MSLN	0,681201908	0,860456436	1	cl1	0
CD2AP	0,681187072	0,570274786	0	N.A.	0
APOL1	0,680629823	0,671955319	0	N.A.	0
GCNT3	0,68050076	0,83639611	0	N.A.	0
PTTG1IP	0,680458919	0,838608132	0	N.A.	0
RHOC	0,680332425	0,661967309	0	N.A.	0
LMTK3	0,679279598	0,7270276	0	N.A.	0
SLC16A3	0,677863333	0,815273476	0	N.A.	0
FAM129B	0,676259772	0,79945779	0	N.A.	0
TRIM31	0,676033911	0,693609344	0	N.A.	0
PVRL2	0,675983023	0,880691491	0	N.A.	0
CDHR2	0,67423523	0,624907184	0	N.A.	0
BCL2L1	0,674174256	0,792951942	0	N.A.	0
ISG20	0,674131165	0,6793177	0	N.A.	0
KCNK1	0,672218063	0,732755152	0	N.A.	0
NEAT1	0,672079523	0,663478681	0	N.A.	0
MYO1E	0,67118211	0,724283764	0	N.A.	0
IFNGR2	0,670728991	0,277270784	0	N.A.	0
MET	0,670354381	0,741349565	0	N.A.	0
CDH1	0,670253265	0,6889997	0	N.A.	0
DST	0,669875081	0,742755296	0	N.A.	0
EPS8	0,669428419	0,572285257	0	N.A.	0
ANGPTL4	0,669316822	0,298592036	0	N.A.	0
PLCD3	0,668227814	0,49029887	0	N.A.	0

PLXNB2	0,667393479	0,82204746	0	N.A.	0
DSC2	0,666774481	0,856581948	0	N.A.	0
ACHE	0,666065257	0,587080845	0	N.A.	0
ARHGAP27	0,665851496	0,752870085	0	N.A.	0
CTNND1	0,665763066	0,761283738	0	N.A.	0
CLIC5	0,665482102	0,547627382	0	N.A.	0
PHLDA3	0,664397509	0,490143529	0	N.A.	0
SDC4	0,664333157	0,69606919	0	N.A.	0
HEPH	0,664084479	0,709072592	0	N.A.	0
MALL	0,664003639	0,828369983	0	N.A.	0
MTMR11	0,663035767	0,601760605	0	N.A.	0
OPTN	0,662401638	0,668640443	0	N.A.	0
KRT19	0,662218606	0,839990568	1	cl1	0
UBE2H	0,661972582	0,630464711	0	N.A.	0
F2RL1	0,660333588	0,698020861	0	N.A.	0
RHBDF1	0,660189233	0,66554889	0	N.A.	0
QSOX1	0,659977425	0,682224629	0	N.A.	0
PSORS1C1	0,659589035	0,226412934	1	cl1	0
LINC01559	0,659380732	0,720203186	0	N.A.	0
CYP4F3	0,658374024	0,760349625	0	N.A.	0
SVIL	0,658118759	0,633618197	0	N.A.	0
PMEPA1	0,656754006	0,681379768	0	N.A.	0
ITGB6	0,65531266	0,701304434	0	N.A.	0
FUT3	0,655088375	0,681646116	0	N.A.	0
ESYT2	0,654707007	0,518821211	0	N.A.	0
EFHD2	0,654263575	0,728375511	0	N.A.	0
PNPLA2	0,65411932	0,73446483	1	cl1	0
ACTN4	0,653177873	0,807135668	0	N.A.	0
FSTL3	0,652951952	0,533152881	0	N.A.	0
ARRDC4	0,651699008	0,508919275	0	N.A.	0
CAB39	0,651213853	0,725662103	0	N.A.	0
CDC42EP2	0,650179896	0,533095512	0	N.A.	0
JOSD1	0,6490135	0,651799563	0	N.A.	0

C1orf116	0,648148997	0,801114373	1	cl1	0
KLF3	0,647910113	0,695737606	0	N.A.	0
HHLA2	0,647113561	0,599462503	0	N.A.	0
GJB4	0,646889036	0,747849462	0	N.A.	0
CHP1	0,64646967	0,67947353	0	N.A.	0
RP11-465N4.	0,64473825	0,501170179	0	N.A.	0
PRSS3	0,64405935	0,736547646	0	N.A.	0
ST14	0,643056902	0,65731654	0	N.A.	0
AGPAT2	0,642233603	0,569678991	0	N.A.	0
TNFRSF12A	0,640872721	0,741069309	0	N.A.	0
OAS1	0,640844028	0,49590937	0	N.A.	0
NPEPPS	0,63941055	0,609905754	0	N.A.	0
CLTB	0,638743348	0,678857202	0	N.A.	0
AKAP12	0,638283854	0,567908723	0	N.A.	0
ARHGAP21	0,638232959	0,772948939	0	N.A.	0
YWHAZ	0,638062873	0,322077845	0	N.A.	0
SGMS2	0,637297923	0,638653748	0	N.A.	0
SPINT2	0,636872608	0,703692876	0	N.A.	0
UNC13D	0,636066951	0,629868986	0	N.A.	0
TOR4A	0,635894494	0,560529488	0	N.A.	0
BHLHE40	0,635184009	0,747613268	0	N.A.	0
TMC5	0,635141373	0,611435314	0	N.A.	0
YOD1	0,634977949	0,320326751	0	N.A.	0
FOSL2	0,634824072	0,724471869	0	N.A.	0
CCDC64B	0,634725481	0,658489397	0	N.A.	0
UPP1	0,634532311	0,519063878	0	N.A.	0
KIAA1522	0,633984971	0,746945582	0	N.A.	0
PLIN3	0,6338953	0,536155184	0	N.A.	0
TGFA	0,633490066	0,676489342	0	N.A.	0
ZFAND3	0,632570504	0,34500798	0	N.A.	0
PDLIM5	0,63257024	0,567099512	0	N.A.	0
BRI3	0,632072257	0,624146898	0	N.A.	0
S100P	0,631007826	0,572528726	0	N.A.	0

OASL	0,629876215	0,364956683	0	N.A.	0
TMEM92	0,629657317	0,600300371	0	N.A.	0
EFNB2	0,628292025	0,792986759	0	N.A.	0
TMCC3	0,628021025	0,628633883	0	N.A.	0
RHPN2	0,627874428	0,784610722	0	N.A.	0
TMC6	0,627766908	0,707636507	0	N.A.	0
VILL	0,627222606	0,635964569	0	N.A.	0
ALS2CL	0,627187461	0,703680616	0	N.A.	0
PTPN12	0,626486202	0,542514084	0	N.A.	0
PIM1	0,626421211	0,38276505	0	N.A.	0
GNG12	0,626032585	0,621903854	0	N.A.	0
ELOVL1	0,625788555	0,855818265	0	N.A.	0
MEF2D	0,625260758	0,45281226	0	N.A.	0
AMN	0,624903602	0,623044229	0	N.A.	0
RHOD	0,6243871	0,647021026	1	cl1	0
CDKN1A	0,624179654	0,599836341	0	N.A.	0
C6orf132	0,624040054	0,734324168	0	N.A.	0
PLXNA3	0,623820621	0,579709748	0	N.A.	0
TICAM1	0,623251155	0,605788204	0	N.A.	0
OCLN	0,623238779	0,719885561	0	N.A.	0
MUC1	0,622964827	0,789950782	0	N.A.	0
LPP	0,622963782	0,541677509	0	N.A.	0
MIR4435-1H(	0,622856919	0,266590261	0	N.A.	0
B3GALT4	0,622657101	0,540556513	0	N.A.	0
TMEM127	0,622303181	0,613895749	0	N.A.	0
EPB41L1	0,621012861	0,680309722	0	N.A.	0
KRT7	0,62052748	0,49344304	0	N.A.	0
MDFI	0,620301263	0,661086417	0	N.A.	0
SPAG9	0,619845124	0,586168813	0	N.A.	0
PAG1	0,619816796	0,477881681	0	N.A.	0
C10orf54	0,619645145	0,543524008	0	N.A.	0
TES	0,618478946	0,316376159	0	N.A.	0
PTPRR	0,618226639	0,669482737	0	N.A.	0

PICALM	0,617280533	0,484381728	0	N.A.	0
AGRN	0,616486518	0,58215378	0	N.A.	0
MIDN	0,61577993	0,798512339	0	N.A.	0
LINC01133	0,615614944	0,769391976	0	N.A.	0
PLS1	0,615008856	0,753358768	0	N.A.	0
BCL9L	0,614942485	0,608557404	0	N.A.	0
BACE2	0,613801702	0,654238358	0	N.A.	0
TMEM63B	0,613252988	0,7653313	0	N.A.	0
LEMD1	0,61288552	0,609721396	1	cl1	0
PRSS8	0,612528405	0,823433129	0	N.A.	0
SLC20A1	0,612300639	0,736429202	1	none	0
DSP	0,611277512	0,58170625	0	N.A.	0
NDRG1	0,61124385	0,592440666	0	N.A.	0
LLGL2	0,609141303	0,671587723	0	N.A.	0
ECM1	0,609050179	0,712743171	0	N.A.	0
MYO7B	0,608384501	0,56631935	0	N.A.	0
LSR	0,607820261	0,773348846	0	N.A.	0
C4BPB	0,607757598	0,500670027	0	N.A.	0
P4HA2	0,607053896	0,707756761	0	N.A.	0
PPP2R5B	0,606800021	0,622240268	0	N.A.	0
EPHA2	0,605771301	0,772237831	1	cl1	0
CTSD	0,605741832	0,639560054	0	N.A.	0
CEACAM6	0,605631848	0,762593525	0	N.A.	0
GTPBP2	0,60518134	0,363238147	0	N.A.	0
XDH	0,605000222	0,460237075	0	N.A.	0
OXSR1	0,604696861	0,504349295	0	N.A.	0
SPTBN1	0,60457059	0,737804414	0	N.A.	0
RASEF	0,60442152	0,630442052	0	N.A.	0
CAST	0,603443421	0,72383654	0	N.A.	0
SUN1	0,603234103	0,655950085	0	N.A.	0
CAPG	0,60309413	0,582150425	0	N.A.	0
MPRIP	0,602824547	0,527812147	0	N.A.	0
FEM1C	0,602800516	0,384174318	0	N.A.	0

MYO5B	0,602217252	0,704987663	0	N.A.	0
DOK4	0,601834665	0,733780704	0	N.A.	0
IRF6	0,60182317	0,402765119	0	N.A.	0
HK2	0,601561406	0,547777613	0	N.A.	0
SLC20A2	0,601519717	0,565440781	0	N.A.	0
PDCD6IP	0,600729891	0,718110341	0	N.A.	0
FHL2	0,600489344	0,642430896	0	N.A.	0
SLC9A1	0,599921304	0,719537139	0	N.A.	0
APOL6	0,59990622	0,617065503	0	N.A.	0
LBH	0,59983539	0,468636646	0	N.A.	0
SQSTM1	0,599776162	0,627569811	0	N.A.	0
CAPN2	0,599062063	0,728344053	0	N.A.	0
MAFK	0,598894823	0,566808049	0	N.A.	0
PCSK7	0,598295284	0,637923341	0	N.A.	0
AIM1L	0,598241789	0,736103053	0	N.A.	0
RAB5B	0,597768119	0,434473134	0	N.A.	0
METRNL	0,597383003	0,508366761	0	N.A.	0
PRRG1	0,597269895	0,227328658	0	N.A.	0
IRF7	0,596574785	0,636158157	0	N.A.	0
SLK	0,596343576	0,677444941	0	N.A.	0
TP53INP2	0,595857843	0,690027993	0	N.A.	0
PRDM1	0,595762086	0,220670586	0	N.A.	0
B4GALT5	0,594179598	0,604368163	0	N.A.	0
PITPNM1	0,593962013	0,386753921	0	N.A.	0
PXN	0,593873884	0,764607087	0	N.A.	0
ATP10B	0,593633322	0,653525959	0	N.A.	0
MICAL2	0,593466951	0,610152643	0	N.A.	0
TAX1BP3	0,592890974	0,404531278	0	N.A.	0
SULT2B1	0,592655786	0,777857874	0	N.A.	0
GPR110	0,592422994	0,536564391	0	N.A.	0
MIR22HG	0,592103443	0,525988408	0	N.A.	0
MMP24-AS1	0,591053287	0,322965846	0	N.A.	0
MYO1C	0,590637505	0,68300652	0	N.A.	0

CHPF	0,590467962	0,758573476	0	N.A.	0
TXNIP	0,59010103	0,470527914	0	N.A.	0
SPATS2L	0,589724313	0,696607344	0	N.A.	0
TMEM120B	0,588389585	0,307042481	0	N.A.	0
ALDH3B1	0,588166694	0,68623845	0	N.A.	0
BCAR1	0,587832855	0,363620563	0	N.A.	0
MUC17	0,587814437	0,658981426	0	N.A.	0
FAM102A	0,587393947	0,703316994	0	N.A.	0
SAT1	0,586795994	0,683531891	0	N.A.	0
SEMA7A	0,58676793	0,502524268	0	N.A.	0
ABHD2	0,586668693	0,613069194	0	N.A.	0
MUC12	0,586558511	0,443192736	0	N.A.	0
SERINC1	0,58609972	0,657141805	0	N.A.	0
CYTH2	0,585553833	0,563737045	0	N.A.	0
SEMA4B	0,584915992	0,762543209	1	cl1	0
EHBP1L1	0,584754102	0,560436145	0	N.A.	0
PARD6B	0,582855132	0,676804677	0	N.A.	0
TNFRSF1A	0,582452133	0,639934025	0	N.A.	0
PVRL4	0,582089718	0,747721641	0	N.A.	0
LYST	0,581996601	0,430169572	0	N.A.	0
LRRFIP1	0,581843537	0,672865929	0	N.A.	0
SLC40A1	0,581811088	0,412711442	0	N.A.	0
ECE1	0,581726471	0,609483818	0	N.A.	0
SH3BGRL3	0,581596503	0,462664717	0	N.A.	0
MYO6	0,581216411	0,751428655	0	N.A.	0
ERBB2IP	0,579901253	0,49312591	0	N.A.	0
MAL2	0,577997954	0,842204837	0	N.A.	0
TJP2	0,577712831	0,68216393	0	N.A.	0
TRIM29	0,577463843	0,742745227	0	N.A.	0
DHRS9	0,577439569	0,62814721	0	N.A.	0
MPZL3	0,577195384	0,6355882	0	N.A.	0
MAPK3	0,577125865	0,782465888	1	cl1	0
ANXA1	0,577071676	0,335263196	0	N.A.	0

LASP1	0,576228279	0,537978942	0	N.A.	0
MBNL2	0,576153825	0,602400123	0	N.A.	0
IL1RN	0,575542521	0,592375338	0	N.A.	0
SCARB2	0,575414644	0,703246074	0	N.A.	0
SAMD9	0,575124093	0,672767509	0	N.A.	0
MYD88	0,575119101	0,803784323	0	N.A.	0
RNF213	0,57502248	0,631612313	0	N.A.	0
IFI27	0,574580484	0,463290844	0	N.A.	0
DNM2	0,574381655	0,740640242	0	N.A.	0
GNAQ	0,573623136	0,49824925	0	N.A.	0
CFLAR	0,57294958	0,388634209	0	N.A.	0
ZNFX1	0,572913108	0,450492653	0	N.A.	0
TMEM106B	0,572717527	0,630433615	0	N.A.	0
HIST1H2BD	0,571256865	0,457540653	0	N.A.	0
KIAA0040	0,570876974	0,568162299	0	N.A.	0
KLK7	0,570581469	0,241721073	1	cl1	0
RMND5A	0,569785467	0,678475656	0	N.A.	0
SNCG	0,569389401	0,237679959	0	N.A.	0
MYO15B	0,56884312	0,55671518	0	N.A.	0
HECA	0,568805436	0,516029238	0	N.A.	0
XIAP	0,568315442	0,552996228	0	N.A.	0
MVP	0,568172016	0,703615963	0	N.A.	0
PPL	0,567934631	0,814860934	1	cl1	0
TIPARP	0,567517604	0,374703379	0	N.A.	0
RILP	0,567428783	0,227041568	0	N.A.	0
EGLN3	0,567022207	0,68044016	0	N.A.	0
NR1D1	0,566645158	0,562459167	0	N.A.	0
SH3RF1	0,566616552	0,75542952	0	N.A.	0
PSAP	0,566254139	0,655268694	0	N.A.	0
TGM2	0,566089515	0,622198396	0	N.A.	0
RAPH1	0,565585409	0,583705668	0	N.A.	0
PLEKHM1	0,564873191	0,456441905	0	N.A.	0
DENND3	0,564778794	0,578733196	0	N.A.	0

APPL2	0,562605216	0,590618622	0	N.A.	0
HSPG2	0,561984935	0,6391399	0	N.A.	0
KLK8	0,561539407	0,363903678	1	cl1	0
SLMAP	0,561211856	0,523053579	0	N.A.	0
GALNT5	0,561047151	0,522366258	0	N.A.	0
GPD2	0,561021634	0,650463758	0	N.A.	0
KIAA1551	0,560660955	0,66909006	0	N.A.	0
RSAD2	0,560404903	0,620705785	0	N.A.	0
WBSCR27	0,560322244	0,094836056	0	N.A.	0
PLEKHG6	0,559630233	0,666133734	0	N.A.	0
EPAS1	0,559391895	0,496289546	0	N.A.	0
SPNS2	0,559234717	0,548990711	0	N.A.	0
BNIP3L	0,559129634	0,405441105	0	N.A.	0
CPEB2	0,5589352	0,286981252	0	N.A.	0
FNDC3B	0,558269587	0,511283394	0	N.A.	0
DOCK5	0,558062762	0,618674855	0	N.A.	0
GK	0,558007479	0,493260459	0	N.A.	0
CDC42BPB	0,55798739	0,659915523	0	N.A.	0
R3HDM4	0,557951089	0,40890371	0	N.A.	0
WSB2	0,557813617	0,605809827	0	N.A.	0
SERTAD2	0,557751454	0,333246501	0	N.A.	0
SESTD1	0,557519222	0,703418541	0	N.A.	0
DNAJC5	0,557433922	0,577148951	0	N.A.	0
GSN	0,556865299	0,64168702	0	N.A.	0
RP1-122P22.	0,556852477	0,38978526	0	N.A.	0
SEZ6L2	0,556225723	0,720758543	1	cl1	0
RELB	0,555617488	0,402676309	0	N.A.	0
ATP1B1	0,555175526	0,610676578	0	N.A.	0
TMC4	0,554667807	0,644891711	0	N.A.	0
INSIG2	0,554665744	0,658567213	0	N.A.	0
SFN	0,554611122	0,551818922	0	N.A.	0
TMOD3	0,553847715	0,545246866	0	N.A.	0
HLA-E	0,553248679	0,382482895	0	N.A.	0

SLC25A23	0,552318375	0,5517143	0	N.A.	0
SLC6A14	0,552105485	0,661982414	0	N.A.	0
SLC22A18	0,551900053	0,700015276	1	cls3	0
GAN	0,551686272	0,714690533	0	N.A.	0
MGLL	0,550879472	0,596622779	0	N.A.	0
P4HA1	0,549434599	0,566128061	0	N.A.	0
MAPK6	0,548929103	0,337889584	0	N.A.	0
S100A6	0,548753375	0,302732441	0	N.A.	0
KCNK6	0,548648435	0,750755502	0	N.A.	0
HIST1H2AC	0,547735816	0,501443854	0	N.A.	0
VEGFA	0,547422954	0,513833707	0	N.A.	0
GUCA2B	0,547276276	0,542000477	0	N.A.	0
GBP2	0,546998145	0,333054057	0	N.A.	0
CTSZ	0,546451054	0,636845143	0	N.A.	0
TMEM30A	0,54624438	0,483354477	0	N.A.	0
RNPEPL1	0,545936292	0,666727445	0	N.A.	0
LPIN2	0,544712767	0,558141076	0	N.A.	0
MMP14	0,54468665	0,526671451	0	N.A.	0
HKDC1	0,544041981	0,482444169	0	N.A.	0
PAM	0,54368229	0,64644885	0	N.A.	0
RALGPS2	0,543203707	0,444302629	0	N.A.	0
ITPR3	0,542836879	0,773585738	0	N.A.	0
NABP1	0,542657977	0,475278312	0	N.A.	0
DUOX2	0,542502694	0,545618168	0	N.A.	0
ARHGAP5	0,542393267	0,629596505	0	N.A.	0
EPS8L2	0,542009996	0,778487279	0	N.A.	0
TOM1L2	0,541920631	0,382769275	0	N.A.	0
SHC1	0,541910935	0,055804737	0	N.A.	0
IFRD1	0,541715616	0,523496982	0	N.A.	0
RP11-268J15	0,541538864	0,468267447	0	N.A.	0
ASAP2	0,541275918	0,704493551	0	N.A.	0
ZNF185	0,5409498	0,581452092	1	cl1	0
SRGAP1	0,540928361	0,6794122	0	N.A.	0

REEP3	0,540829817	0,655452274	0	N.A.	0
SERPINE2	0,540510159	0,716573112	0	N.A.	0
DAPP1	0,540380987	0,074767981	0	N.A.	0
SH2D3A	0,540039139	0,692192395	0	N.A.	0
CYP2S1	0,539831549	0,623360773	0	N.A.	0
LHFPL2	0,539747188	0,339104773	0	N.A.	0
MACF1	0,539078086	0,594828052	0	N.A.	0
SLC6A20	0,538394743	0,560373495	0	N.A.	0
TPM4	0,538152645	0,317697875	0	N.A.	0
FAM126B	0,538118076	0,555756299	0	N.A.	0
ABHD17C	0,538073261	0,528295334	0	N.A.	0
TMEM45B	0,536952795	0,709420926	0	N.A.	0
LRRC1	0,53672514	0,436946117	0	N.A.	0
NPC1	0,536631101	0,494916911	0	N.A.	0
LMBRD1	0,535761014	0,581556939	0	N.A.	0
WIPF2	0,535311376	0,593227771	0	N.A.	0
KIF13B	0,535161488	0,744073304	0	N.A.	0
B2M	0,535074679	0,451429774	0	N.A.	0
MLLT4	0,535053942	0,671285585	0	N.A.	0
ABCG1	0,534957761	0,51487934	0	N.A.	0
B3GNT3	0,534925856	0,681925429	0	N.A.	0
SH3GLB1	0,534815595	0,546805748	0	N.A.	0
SMIM6	0,53448632	0,621405975	0	N.A.	0
SDR16C5	0,534478673	0,655605109	0	N.A.	0
POF1B	0,534011438	0,346195179	0	N.A.	0
CCDC68	0,533060378	0,266232431	0	N.A.	0
MTUS1	0,532486233	0,591622802	0	N.A.	0
DUOXA2	0,532207356	0,428701277	0	N.A.	0
C11orf86	0,531999883	0,43357284	0	N.A.	0
ANTXR2	0,531903293	0,643125823	0	N.A.	0
CALCOCO1	0,531476072	0,562371629	0	N.A.	0
MALAT1	0,530990814	0,586449823	0	N.A.	0
SOWAHC	0,530563435	0,584591039	0	N.A.	0

ITGAV	0,530545578	0,600351449	0	N.A.	0
CLSTN1	0,529495871	0,828427121	0	N.A.	0
CLDN23	0,528974191	0,615857172	0	N.A.	0
TFF1	0,528915348	0,647226068	0	N.A.	0
CDH17	0,528702873	0,594555312	0	N.A.	0
YAP1	0,528695527	0,525456221	0	N.A.	0
TTC9	0,528580309	0,515002113	0	N.A.	0
EGFR	0,528391369	0,528634549	0	N.A.	0
SLC17A5	0,528218334	0,718197926	0	N.A.	0
IST1	0,528217992	0,258895343	0	N.A.	0
KIFC3	0,528172126	0,399174434	0	N.A.	0
LINC00969	0,528154177	0,626349113	0	N.A.	0
HERC4	0,527954779	0,443560945	0	N.A.	0
SMPDL3A	0,52683903	0,496345636	0	N.A.	0
CSTB	0,526817795	0,491606585	0	N.A.	0
NCOR2	0,526647052	0,64606396	0	N.A.	0
SMIM5	0,526501529	0,527835232	0	N.A.	0
RP11-841O2(	0,526173401	0,363847529	0	N.A.	0
PRKCDBP	0,526090265	0,370255103	0	N.A.	0
MKNK2	0,526029577	0,659740039	0	N.A.	0
SIRT7	0,525291676	0,76030063	0	N.A.	0
TRIM15	0,524972485	0,371521586	0	N.A.	0
DUSP3	0,524826289	0,452677634	0	N.A.	0
MYH9	0,524330356	0,566449036	0	N.A.	0
MIER3	0,52353296	0,382813139	0	N.A.	0
SFTA2	0,523169611	0,478116197	1	cl1	0
EHD1	0,522889161	0,406505587	0	N.A.	0
RAB22A	0,522297506	0,566566393	0	N.A.	0
FOXO1	0,522269604	0,570100038	0	N.A.	0
PI3	0,521548376	0,415737391	0	N.A.	0
SMPD1	0,521193519	0,564223115	0	N.A.	0
IFT172	0,520914183	0,421047194	0	N.A.	0
MACC1	0,520251012	0,686492677	0	N.A.	0

RCOR1	0,519853817	0,338821561	0	N.A.	0
PPP1R14D	0,519811413	0,436245164	0	N.A.	0
SIPA1	0,519487776	0,564787471	0	N.A.	0
PKIB	0,519455738	0,540952158	0	N.A.	0
TIMP1	0,519374602	0,703915055	0	N.A.	0
RNF19B	0,519292525	0,211982924	0	N.A.	0
MYEOV	0,518373769	0,625687656	0	N.A.	0
ASS1	0,517886018	0,482273149	0	N.A.	0
APOL2	0,517773218	0,373296174	0	N.A.	0
TFF2	0,517234346	0,595052218	0	N.A.	0
STS	0,517032454	0,578770956	0	N.A.	0
MAPRE3	0,516589344	0,630568273	0	N.A.	0
CTSE	0,516564227	0,496678341	1	cl1	0
ISG15	0,515975933	0,458027125	0	N.A.	0
KLF2	0,515940459	0,409187704	0	N.A.	0
TNK2	0,515217861	0,48688726	0	N.A.	0
SMURF1	0,51519353	0,474034639	0	N.A.	0
PITPNC1	0,514829979	0,297500367	0	N.A.	0
AHCYL2	0,514669335	0,549376413	0	N.A.	0
B3GALT5	0,514584301	0,604185593	0	N.A.	0
ARSA	0,514455347	0,500376996	0	N.A.	0
SMIM22	0,513849173	0,488105573	0	N.A.	0
CAPN5	0,513794724	0,512446457	0	N.A.	0
TRIM25	0,513769892	0,391094682	0	N.A.	0
HOOK3	0,513631841	0,523383397	0	N.A.	0
FXYD5	0,513557066	0,431044247	0	N.A.	0
MIR210HG	0,513395989	0,634678703	0	N.A.	0
GALNT1	0,513085724	0,508535987	0	N.A.	0
KRT16	0,512989641	0,02493821	0	N.A.	0
SLC12A7	0,51282609	0,621665133	0	N.A.	0
TAPBP	0,512457641	0,386607208	0	N.A.	0
NFE2L1	0,511454683	0,491326848	0	N.A.	0
TCF7L2	0,511216342	0,531247499	0	N.A.	0

PIP4K2C	0,511105239	0,44371777	0	N.A.	0
FAM46A	0,510788506	0,605255265	0	N.A.	0
GPRC5C	0,510720307	0,803421506	0	N.A.	0
ARHGEF12	0,50986982	0,703988226	0	N.A.	0
DENND2C	0,509669289	0,289417822	0	N.A.	0
PDP1	0,509247255	0,573277066	0	N.A.	0
CEACAM7	0,509163808	0,323494483	0	N.A.	0
PLSCR1	0,508869533	0,557199247	0	N.A.	0
IDS	0,508761232	0,394868034	0	N.A.	0
GULP1	0,508541279	0,517764254	0	N.A.	0
CDC42EP5	0,508343825	0,276474726	0	N.A.	0
COX6B2	0,508080532	0,690486532	0	N.A.	0
TNFAIP1	0,50751869	0,6622989	0	N.A.	0
DDA1	0,507475299	0,320257968	0	N.A.	0
CIDEC	0,507052585	0,469309823	0	N.A.	0
CASP10	0,507020025	0,363293409	0	N.A.	0
IFNGR1	0,506954584	0,685579317	0	N.A.	0
TUBB2A	0,506835184	0,453434804	0	N.A.	0
IBTK	0,506722728	0,528185611	0	N.A.	0
RNF186	0,506144274	0,290716441	0	N.A.	0
NFKBIE	0,505455712	0,406390707	0	N.A.	0
F3	0,504995258	0,75042491	0	N.A.	0
BCL2L15	0,504745415	0,490890035	0	N.A.	0
GTPBP1	0,504566379	0,30189923	0	N.A.	0
DDX60	0,504454414	0,464337641	0	N.A.	0
KRT15	0,504451152	0,432809164	0	N.A.	0
TAOK3	0,503910208	0,512235929	0	N.A.	0
RP11-783K16	0,503902264	0,358242805	0	N.A.	0
INSIG1	0,503763056	0,483035782	0	N.A.	0
RNF223	0,503669035	0,413703141	0	N.A.	0
EPS8L3	0,50324593	0,640201743	0	N.A.	0
KRT20	0,503234456	0,605278091	0	N.A.	0
GNB1	0,503017401	0,581275723	0	N.A.	0

CYTH1	0,502979981	0,478443489	0	N.A.	0
SEC31A	0,502797254	0,362903321	0	N.A.	0
OAS3	0,502754134	0,590918822	0	N.A.	0
PLAC8	0,502732037	0,60509469	0	N.A.	0
GRN	0,502479665	0,543916298	0	N.A.	0
CLDN12	0,502391613	0,64056347	0	N.A.	0
TYMP	0,502087366	0,330593672	0	N.A.	0
TCIRG1	0,501912479	0,525406864	0	N.A.	0
BIRC3	0,501897109	0,426106018	0	N.A.	0
FAM83H	0,501869103	0,56522893	0	N.A.	0
CYP4F12	0,501107703	0,477744876	0	N.A.	0
ZBTB20	0,501050446	0,433688435	0	N.A.	0
FTH1	0,500937494	0,077613818	0	N.A.	0
PKN2	0,500703011	0,638771842	0	N.A.	0
FOXN2	0,50043951	0,215098665	0	N.A.	0
ATP2B4	0,500407648	0,545573112	0	N.A.	0
RND3	0,499882837	0,753962493	0	N.A.	0
ARF6	0,49968826	0,418154117	0	N.A.	0
RDH13	0,499410619	0,445431841	0	N.A.	0
TMEM139	0,499328615	0,580984647	0	N.A.	0
INF2	0,498978227	0,673122893	0	N.A.	0
C17orf67	0,498773884	-0,031982299	0	N.A.	0
S100A2	0,498329069	0,354499252	1	cl1	0
FUT2	0,498138582	0,505599763	0	N.A.	0
C1GALT1	0,498104563	0,634182714	0	N.A.	0
MMP15	0,497924602	0,531121305	0	N.A.	0
SHOC2	0,497766845	0,400197336	0	N.A.	0
CD164	0,497652451	0,599461963	0	N.A.	0
CHAC1	0,497554911	0,442829993	0	N.A.	0
TRIP10	0,496853055	0,705519786	0	N.A.	0
SECISBP2L	0,496842846	0,601751263	0	N.A.	0
BCL10	0,496635075	0,167956059	0	N.A.	0
IL2RG	0,496488734	0,445616077	0	N.A.	0

CXADR	0,496005348	0,689969582	0	N.A.	0
RNF39	0,495971473	0,626902979	1	cl1	0
FGD6	0,495425108	0,563003656	0	N.A.	0
INPP4B	0,495138133	0,493697665	0	N.A.	0
IL22RA1	0,494882008	0,581940102	0	N.A.	0
MAP1LC3B	0,494777132	0,334574534	0	N.A.	0
TLE1	0,494431019	0,224551059	0	N.A.	0
FXYD3	0,494344471	0,53041723	0	N.A.	0
VASP	0,493850509	0,494231401	0	N.A.	0
TAX1BP1	0,493345765	0,583949406	0	N.A.	0
KRT17	0,493230731	0,568491862	1	cl1	0
FAM114A1	0,493174418	0,578632793	0	N.A.	0
ARHGAP29	0,492981959	0,247018094	0	N.A.	0
BHLHE41	0,492605731	0,504942053	0	N.A.	0
SAV1	0,492593184	0,411934812	0	N.A.	0
PELI1	0,492436493	0,499782198	0	N.A.	0
TMEM87B	0,492243275	0,58137221	0	N.A.	0
CLIP1	0,492073815	0,64004966	0	N.A.	0
CTSB	0,491845214	0,588491146	0	N.A.	0
CTD-2396E7.	0,491165881	0,258571869	0	N.A.	0
ARHGAP12	0,490913923	0,554811684	0	N.A.	0
KLF6	0,490833894	0,635419228	0	N.A.	0
RRBP1	0,490794371	0,55720004	0	N.A.	0
IL32	0,490111497	0,285774635	0	N.A.	0
CCDC186	0,489565885	0,506079752	0	N.A.	0
MYRF	0,489547358	0,676797996	0	N.A.	0
IL18	0,489410974	0,476411983	0	N.A.	0
ARL8B	0,488958805	0,195513781	0	N.A.	0
DPP4	0,488526356	0,49510719	0	N.A.	0
ZBTB7B	0,487845785	0,678609826	0	N.A.	0
ANKLE2	0,48761373	0,48842645	0	N.A.	0
MFI2	0,486947583	0,621157768	0	N.A.	0
HSD17B2	0,485311709	0,456045358	0	N.A.	0

CLOCK	0,485279182	0,60842416	0	N.A.	0
MIR222HG	0,484512181	0,407220891	0	N.A.	0
DLGAP4	0,484491432	0,453873322	0	N.A.	0
STOM	0,484370266	0,515790206	0	N.A.	0
RAP2B	0,483993881	0,614367063	0	N.A.	0
LPCAT4	0,483893751	0,657270824	0	N.A.	0
VNN1	0,483839788	0,488186468	0	N.A.	0
KIAA1217	0,483453653	0,406763782	0	N.A.	0
ODF3B	0,483198729	0,247168187	0	N.A.	0
SERPINB8	0,483041882	0,239628339	0	N.A.	0
BACH1	0,482209954	0,642075416	0	N.A.	0
SUSD6	0,481904296	0,516330409	0	N.A.	0
TMEM30B	0,481899328	0,434025954	0	N.A.	0
CPEB4	0,481783488	0,445159908	0	N.A.	0
C12orf49	0,481749781	0,498171063	0	N.A.	0
TJP1	0,480633155	0,752506656	0	N.A.	0
UBR4	0,479992588	0,417966713	0	N.A.	0
STAT6	0,479938774	0,504697786	0	N.A.	0
C4orf19	0,479527956	0,492244692	0	N.A.	0
LCOR	0,479501431	0,365886174	0	N.A.	0
RAET1L	0,478871197	0,514673208	0	N.A.	0
MXI1	0,477977399	0,454051746	0	N.A.	0
SBNO2	0,477829099	0,381962078	0	N.A.	0
TMEM125	0,477754886	0,570870576	0	N.A.	0
CLDN4	0,477564594	0,639530539	0	N.A.	0
PDE4C	0,476715253	0,242960777	0	N.A.	0
CCDC107	0,475967824	0,464527337	0	N.A.	0
S100A10	0,474964212	0,307863162	0	N.A.	0
PTP4A2	0,474851143	0,186485016	0	N.A.	0
ZBTB43	0,474683471	0,372891029	0	N.A.	0
METRN	0,473971214	0,151582339	0	N.A.	0
KLHL21	0,473557028	0,194069611	0	N.A.	0
SCEL	0,473439109	0,631357019	1	cl1	0

ARRDC1	0,472895616	0,789515828	0	N.A.	0
TLDC1	0,472816182	0,523582457	0	N.A.	0
ULBP2	0,472796254	0,389497959	0	N.A.	0
FAM214B	0,472785113	0,504020053	0	N.A.	0
DCBLD2	0,47273649	0,55493073	0	N.A.	0
CDYL	0,472388062	0,539407839	0	N.A.	0
FAM132A	0,472287498	0,683585704	0	N.A.	0
PNCK	0,472104278	0,198869443	0	N.A.	0
S100A14	0,471955955	0,612393593	0	N.A.	0
PSEN1	0,471218873	0,627015423	0	N.A.	0
HS3ST1	0,471154685	0,321831109	0	N.A.	0
ANKRD13A	0,470803766	0,556057583	0	N.A.	0
LINC00520	0,470792903	0,674467222	0	N.A.	0
DGKA	0,470743017	0,716852008	0	N.A.	0
EIF4E3	0,47074246	0,541641735	0	N.A.	0
RP3-395M20	0,470631316	0,510102477	0	N.A.	0
ANKRD9	0,470452932	0,502401356	0	N.A.	0
AKAP13	0,470292444	0,650705462	0	N.A.	0
ADAM10	0,469911781	0,550447225	0	N.A.	0
HRASLS2	0,469826743	0,264270886	0	N.A.	0
ATP9A	0,469440066	0,512556553	0	N.A.	0
SPRR1B	0,469394784	0,452490938	0	N.A.	0
LENG8	0,469257945	0,595243617	0	N.A.	0
CFDP1	0,469234564	0,623739626	0	N.A.	0
UBL3	0,469220015	0,53159715	0	N.A.	0
PDGFA	0,468798498	0,592745418	0	N.A.	0
SGPP2	0,46857423	0,74798094	0	N.A.	0
GUK1	0,467852191	0,161152908	0	N.A.	0
RRAGC	0,46737164	0,480236821	0	N.A.	0
GAST	0,467020996	0,431646749	0	N.A.	0
S100A11	0,466839356	0,220558293	0	N.A.	0
GRIN2D	0,466616846	0,312873928	0	N.A.	0
PTPRF	0,465854709	0,627760061	0	N.A.	0

DGKH	0,465393259	0,550326659	0	N.A.	0
N4BP1	0,464980447	0,474497295	0	N.A.	0
RARA-AS1	0,464766848	0,291329067	0	N.A.	0
FEZ2	0,464531311	0,643050897	0	N.A.	0
STX7	0,464518318	0,564066605	0	N.A.	0
ANO6	0,464487521	0,404844646	0	N.A.	0
KCMF1	0,46332772	0,633477645	0	N.A.	0
CNKSR1	0,463219669	0,638560889	0	N.A.	0
FOXO3	0,463016663	0,433364255	0	N.A.	0
LIMA1	0,462663913	0,646747399	0	N.A.	0
MST1R	0,462494946	0,630407694	0	N.A.	0
NCOA2	0,462016173	0,209995326	0	N.A.	0
SHROOM3	0,461511016	0,652818032	0	N.A.	0
RASGRP3	0,461492818	0,295816479	0	N.A.	0
SP1	0,461155152	0,417111574	0	N.A.	0
SEPP1	0,460752134	0,350040333	0	N.A.	0
ITPKC	0,460718351	0,512989138	0	N.A.	0
BATF2	0,460535678	0,334919432	0	N.A.	0
PLEKHN1	0,460380278	0,578503372	0	N.A.	0
CTNNA1	0,459813135	0,674472488	0	N.A.	0
TRIOBP	0,459801939	0,585143894	0	N.A.	0
RALGDS	0,459658764	0,18371625	0	N.A.	0
PLOD2	0,459475955	0,178693779	0	N.A.	0
GRHL1	0,459274567	0,355102565	0	N.A.	0
CTSS	0,459143191	0,188715288	0	N.A.	0
TXNRD1	0,459112402	0,275519742	0	N.A.	0
MED15	0,458898599	0,534594051	0	N.A.	0
ARL8A	0,458533821	0,690820279	0	N.A.	0
HES4	0,458366633	0,209981619	0	N.A.	0
SERPINB5	0,458262391	0,618913781	1	cl1	0
LIF	0,458114161	0,608190619	0	N.A.	0
PERP	0,457301149	0,786328142	0	N.A.	0
NFAT5	0,457282755	0,476446163	0	N.A.	0

FOXC1	0,457234003	0,43847209	0	N.A.	0
HELZ2	0,457209329	0,621553747	0	N.A.	0
MMP7	0,456657307	0,3140915	1	cl1	0
LGR4	0,456604827	0,437240219	0	N.A.	0
ABTB1	0,456534889	0,325750164	0	N.A.	0
RREB1	0,456485393	0,451815079	0	N.A.	0
TMEM8A	0,456427524	0,503129743	0	N.A.	0
SLC45A4	0,456152781	0,285824307	0	N.A.	0
PPP1R13L	0,456099364	0,708628779	0	N.A.	0
FAM107B	0,45574357	0,215027966	0	N.A.	0
SCIN	0,455637752	0,463310682	0	N.A.	0
S100A16	0,45529253	0,501712623	0	N.A.	0
RHBDL2	0,455127996	0,664827991	1	cl1	0
LRRC32	0,454871184	0,276599323	0	N.A.	0
SLC35A3	0,453839442	0,597100179	0	N.A.	0
WDR13	0,453506413	0,11829063	0	N.A.	0
AMOTL2	0,453019855	0,714415936	0	N.A.	0
CLMN	0,45290628	0,540275977	0	N.A.	0
FLII	0,452900636	0,600428113	0	N.A.	0
C9orf89	0,452722868	0,293288956	0	N.A.	0
CEMIP	0,452681904	0,364002593	0	N.A.	0
KCTD11	0,452644617	0,585760905	0	N.A.	0
MX2	0,452435636	0,461022367	0	N.A.	0
ATXN1	0,452381668	0,391383607	0	N.A.	0
HSBP1L1	0,45209043	0,29827209	0	N.A.	0
HLA-F	0,451617876	0,295410395	0	N.A.	0
PPIC	0,451539513	0,424293559	0	N.A.	0
PLEKHA1	0,451493956	0,686322379	0	N.A.	0
TGOLN2	0,451477403	0,39653043	0	N.A.	0
VIMP	0,451281461	0,618365387	0	N.A.	0
SEC23A	0,451216674	0,286789128	0	N.A.	0
HDAC9	0,450915134	0,479493715	0	N.A.	0
KHNYN	0,450875171	0,232458655	0	N.A.	0

SECTM1	0,450097054	0,40342675	0	N.A.	0
SORL1	0,450093623	0,565472706	0	N.A.	0
TPT1-AS1	0,450020602	0,219939864	0	N.A.	0
PPP1CB	0,449893309	0,346388162	0	N.A.	0
SLC52A3	0,449841875	0,460601921	0	N.A.	0
JUND	0,449724698	0,538415321	0	N.A.	0
FOSL1	0,449381029	0,287363292	0	N.A.	0
CHMP2B	0,449235148	0,64293261	0	N.A.	0
MYL6	0,449024375	0,217359502	0	N.A.	0
RAB27B	0,448492719	0,506941759	0	N.A.	0
ANXA2	0,448203252	0,452065675	0	N.A.	0
WBP2	0,448144051	0,492323548	0	N.A.	0
TOB1	0,447813213	0,543973435	0	N.A.	0
PDGFB	0,447790755	0,491177421	0	N.A.	0
BDKRB2	0,447704165	0,576405375	0	N.A.	0
DSG3	0,447658098	0,527299579	0	N.A.	0
PFKFB3	0,4470099	0,217617853	0	N.A.	0
NPTN	0,446891064	0,455074206	0	N.A.	0
LRP1	0,446804427	0,450968215	0	N.A.	0
TAF13	0,446683697	0,344284895	0	N.A.	0
DIAPH1	0,44643072	0,724360915	0	N.A.	0
HDHD3	0,446292459	0,596937122	0	N.A.	0
TACSTD2	0,446283628	0,843848373	1	cl1	0
STK39	0,44620458	0,51263925	0	N.A.	0
VAMP5	0,445564147	0,511714335	0	N.A.	0
SLC2A13	0,445195223	0,239693979	0	N.A.	0
CRIM1	0,445089934	0,452904092	0	N.A.	0
ETS1	0,44507917	0,27184605	0	N.A.	0
PMP22	0,444701967	0,459453152	0	N.A.	0
C4orf3	0,444629114	0,066638017	0	N.A.	0
CCL28	0,444506458	0,275713732	0	N.A.	0
GPR37L1	0,444485682	0,519693562	0	N.A.	0
EDEM1	0,444470679	0,080771529	0	N.A.	0

NDEL1	0,444415147	0,358816447	0	N.A.	0
INADL	0,443564561	0,519339903	0	N.A.	0
SMIM2-AS1	0,44345504	0,086623397	0	N.A.	0
TMEM120A	0,443055874	0,525124671	0	N.A.	0
STX4	0,442756969	0,230223669	0	N.A.	0
PTPRK	0,442670377	0,489854403	0	N.A.	0
LYPD3	0,44240349	0,356267241	0	N.A.	0
ZG16B	0,442305057	0,508628147	0	N.A.	0
MUC4	0,442144175	0,606934463	0	N.A.	0
GOLGA2	0,441969544	0,575689637	0	N.A.	0
GNS	0,441374702	0,226293749	0	N.A.	0
LRRC8A	0,441014295	0,69728479	0	N.A.	0
NFKB2	0,441007961	0,090930723	0	N.A.	0
HIVEP2	0,440929582	0,269746085	0	N.A.	0
ZYX	0,440803807	0,157282477	0	N.A.	0
NCSTN	0,440670086	0,381034184	0	N.A.	0
STK40	0,440378372	0,459068596	0	N.A.	0
MAPKAPK2	0,439974688	0,349657073	0	N.A.	0
MUC16	0,439661173	0,345859342	0	N.A.	0
AOC1	0,43956455	0,59106449	0	N.A.	0
TMEM181	0,439557839	0,430286544	0	N.A.	0
PARM1	0,438960053	0,1329815	0	N.A.	0
PLK3	0,438754359	0,550023597	0	N.A.	0
ZNF292	0,438714329	0,44667265	0	N.A.	0
PXDC1	0,438704021	0,470784906	0	N.A.	0
HPGD	0,438662752	0,523530439	0	N.A.	0
GPC1	0,438526147	0,583100944	0	N.A.	0
SPPL2A	0,438461625	0,642625837	0	N.A.	0
FGFBP1	0,438418449	0,577457936	0	N.A.	0
H1F0	0,438376239	0,455693293	0	N.A.	0
GPCPD1	0,437974989	0,355655271	0	N.A.	0
JAG1	0,437951287	0,645908302	0	N.A.	0
ABTB2	0,437947777	0,350341764	0	N.A.	0

RAB4B	0,437932703	0,224358733	0	N.A.	0
BMPR2	0,437531574	0,491399857	0	N.A.	0
GPR115	0,437496403	0,559271226	0	N.A.	0
ARHGAP42	0,437345037	0,425166545	0	N.A.	0
RBMS2	0,437156691	0,303713221	0	N.A.	0
SSUH2	0,437047098	0,427915028	0	N.A.	0
FAM3C	0,436787318	0,605224751	0	N.A.	0
SEMA3C	0,436193003	0,309821044	0	N.A.	0
KCNK5	0,435823105	0,332503853	0	N.A.	0
LAMA5	0,435812991	0,38725887	0	N.A.	0
RHCG	0,435244263	0,356547604	0	N.A.	0
PNPLA8	0,434909192	0,261189419	0	N.A.	0
CTTN	0,434620809	0,693800298	0	N.A.	0
ATL3	0,434274671	0,075600552	0	N.A.	0
GIPC1	0,434145002	0,701816618	0	N.A.	0
NEK7	0,434040188	0,493768125	0	N.A.	0
WASL	0,433796574	0,466637222	0	N.A.	0
PRAP1	0,433669466	0,512621068	0	N.A.	0
NOS2	0,43366594	0,340766327	0	N.A.	0
B3GNT5	0,43303103	0,436989832	0	N.A.	0
GNA13	0,432779642	0,231727654	0	N.A.	0
IFI35	0,43257185	0,430369669	0	N.A.	0
TPT1	0,432444785	-0,220462111	0	N.A.	0
CAPN8	0,432214723	0,628046608	0	N.A.	0
PPP1R11	0,432061301	0,030908349	0	N.A.	0
LATS2	0,431939299	0,575185291	0	N.A.	0
RAB10	0,43189775	0,536134914	0	N.A.	0
PTBP3	0,431583826	0,185976735	0	N.A.	0
TSPAN8	0,431364979	0,448481783	0	N.A.	0
NBL1	0,431111979	0,748993499	0	N.A.	0
MYL9	0,430985202	0,122149176	0	N.A.	0
TRAF4	0,430963786	0,700266539	0	N.A.	0
TNFSF9	0,430813127	0,316493508	0	N.A.	0

TNFSF15	0,430648129	0,425583974	0	N.A.	0
SLC5A3	0,43051672	0,644956872	0	N.A.	0
SLC11A2	0,430215939	0,485396314	0	N.A.	0
ADM	0,430186642	0,518769779	0	N.A.	0
HAS3	0,429994759	0,140450589	0	N.A.	0
CEP170B	0,429628413	0,443229438	0	N.A.	0
RETSAT	0,429370855	0,673616368	0	N.A.	0
ITGB8	0,429110043	0,65520502	0	N.A.	0
CNGA1	0,428977877	0,144473815	0	N.A.	0
ITPK1	0,428892792	0,466259014	0	N.A.	0
CLCF1	0,428889737	0,670003702	0	N.A.	0
ADIPOR2	0,428775512	0,58244489	0	N.A.	0
FAT1	0,42864841	0,464464799	0	N.A.	0
JAK1	0,428524007	0,384058457	0	N.A.	0
FAM160A2	0,428312576	0,523469393	0	N.A.	0
MAP7D1	0,428252513	0,468165144	0	N.A.	0
FRMD3	0,428225471	0,333489864	0	N.A.	0
CD58	0,428141899	0,449441942	0	N.A.	0
FRMD4B	0,427989782	0,225116315	0	N.A.	0
INO80C	0,427693645	0,46067128	0	N.A.	0
INSR	0,427566643	0,575468873	0	N.A.	0
VSIG2	0,427416759	0,613713867	1	cl1	0
THRB	0,427086786	0,481997966	0	N.A.	0
C19orf66	0,427079421	0,605833481	0	N.A.	0
TSPAN14	0,427045594	0,695404928	0	N.A.	0
COBL	0,426840707	0,51281019	0	N.A.	0
UBE2R2	0,426585292	0,668529061	0	N.A.	0
GALNT3	0,42657325	0,553664102	0	N.A.	0
KDM3A	0,426305157	0,219640559	0	N.A.	0
SLC35C1	0,426119078	0,591292305	0	N.A.	0
ARHGEF35	0,426080312	0,57878155	0	N.A.	0
PLA2G16	0,425561174	0,513150736	0	N.A.	0
RHOB	0,425033753	0,340484188	0	N.A.	0

HMOX1	0,424996144	0,452478706	0	N.A.	0
GOLGA3	0,424979332	0,387405408	0	N.A.	0
HAPLN3	0,424847736	0,422879448	0	N.A.	0
FNIP2	0,424746792	0,463261536	0	N.A.	0
SMPD3	0,424380816	0,34214884	0	N.A.	0
EHD4	0,424191346	0,649380587	0	N.A.	0
LDLR	0,423753743	0,627731293	0	N.A.	0
SERPINB9	0,423411376	0,281455679	0	N.A.	0
PRRT2	0,423393079	0,082238666	0	N.A.	0
OAS2	0,422834557	0,463825582	0	N.A.	0
KLK6	0,422497121	0,419212671	1	cl1	0
PML	0,422476221	0,436668321	0	N.A.	0
ARL4C	0,422342349	0,081464628	0	N.A.	0
LPGAT1	0,422160832	0,376452854	0	N.A.	0
C10orf10	0,422119412	0,280303503	0	N.A.	0
RAP1B	0,422023083	-0,024769068	0	N.A.	0
CDC42BPA	0,42179777	0,482431113	0	N.A.	0
ZBTB7A	0,421600082	0,58841703	0	N.A.	0
ERBB2	0,421429711	0,655779783	0	N.A.	0
TRAK1	0,421356011	0,517927634	0	N.A.	0
SH3D21	0,420711455	0,535598645	0	N.A.	0
ZFAND5	0,420701672	0,333641326	0	N.A.	0
BBC3	0,420551618	0,403290012	0	N.A.	0
AHR	0,420531758	0,354327266	0	N.A.	0
FGD4	0,419358869	0,513694955	0	N.A.	0
MAP3K11	0,419080366	0,51997654	0	N.A.	0
MFGE8	0,419051179	0,478632194	0	N.A.	0
PARP14	0,419038835	0,6263058	0	N.A.	0
FAM134C	0,418803075	0,584841288	0	N.A.	0
MEGF6	0,418587599	0,489446523	0	N.A.	0
MINK1	0,418545313	0,467726508	0	N.A.	0
PIK3CB	0,418431793	0,379878434	0	N.A.	0
PTK2B	0,418362603	0,6143092	0	N.A.	0

OSGIN1	0,418279111	0,310211552	0	N.A.	0
TMC7	0,418224755	0,36507954	0	N.A.	0
APLP2	0,418196717	0,740220691	0	N.A.	0
CTDSP1	0,418124973	0,324366469	0	N.A.	0
KTN1	0,41773325	0,614242952	0	N.A.	0
SH3D19	0,41711548	0,651349879	0	N.A.	0
ZBP1	0,416720648	0,497548448	0	N.A.	0
BMP1	0,416554629	0,409127207	0	N.A.	0
AFF4	0,416439729	0,53436696	0	N.A.	0
TNKS1BP1	0,416437098	0,608783955	0	N.A.	0
C4orf48	0,416423474	0,411968135	0	N.A.	0
TPCN1	0,416310953	0,532151156	0	N.A.	0
BCL3	0,416300271	0,434556453	0	N.A.	0
SNRK	0,416287011	0,477751254	0	N.A.	0
TNFAIP2	0,416179957	0,49782015	0	N.A.	0
RYBP	0,415924508	0,296959928	0	N.A.	0
CTAGE5	0,415809191	0,388415072	0	N.A.	0
GABRE	0,415774948	0,463877558	0	N.A.	0
PHF1	0,415447405	0,300905593	0	N.A.	0
DDX17	0,415329888	0,495980969	0	N.A.	0
GRAMD3	0,415249726	0,422170339	0	N.A.	0
ABI1	0,415132482	0,301230124	0	N.A.	0
UBE2B	0,414796914	0,520978412	0	N.A.	0
LAMB2	0,41460186	0,534711051	0	N.A.	0
KIAA2026	0,414264816	0,506858246	0	N.A.	0
ETV6	0,414245172	0,330327927	0	N.A.	0
NSMF	0,413961933	0,559779261	0	N.A.	0
RHOU	0,413823744	0,390752727	0	N.A.	0
TMEM65	0,413708329	0,35997543	0	N.A.	0
SLC35D2	0,413319031	0,298222748	0	N.A.	0
TM4SF5	0,412861614	0,627541122	0	N.A.	0
RP11-395B7.	0,412695544	0,385483206	0	N.A.	0
DHRS3	0,412694601	0,646735433	0	N.A.	0

PPP1R15B	0,41215841	0,435825801	0	N.A.	0
P2RY2	0,411889382	0,098022664	0	N.A.	0
RP11-467L13	0,411626619	0,366878816	0	N.A.	0
RAPGEFL1	0,411510609	0,619878369	0	N.A.	0
SPRR3	0,41106944	0,406330771	0	N.A.	0
CCRL2	0,410962428	0,105648307	0	N.A.	0
TMEM219	0,410908375	0,402315382	0	N.A.	0
ST3GAL1	0,41083801	0,557821133	0	N.A.	0
CEBPG	0,410766742	0,576038751	0	N.A.	0
TMEM164	0,410279336	0,434425124	0	N.A.	0
FAM177A1	0,40998635	0,42205021	0	N.A.	0
PTRF	0,409809698	0,264369911	0	N.A.	0
ST6GALNAC2	0,4097697	0,051795596	0	N.A.	0
NR1H2	0,409238797	0,070180352	0	N.A.	0
TNNC1	0,409214712	0,256556749	0	N.A.	0
TC2N	0,409117399	0,470173587	0	N.A.	0
ICAM1	0,408782055	0,198055474	0	N.A.	0
SLC10A3	0,408762645	0,391086577	0	N.A.	0
CCPG1	0,408744609	0,228674039	0	N.A.	0
SPTAN1	0,408723801	0,648449028	0	N.A.	0
WLS	0,408715653	0,579678269	0	N.A.	0
PPP4R1	0,408457301	0,522048649	0	N.A.	0
HIST1H1C	0,408289639	0,183084036	0	N.A.	0
PACS1	0,408255746	-0,031189424	0	N.A.	0
AP003068.23	0,407565213	0,260744639	0	N.A.	0
WDR45B	0,4075085	0,493252328	0	N.A.	0
AC006262.5	0,407230571	0,595142975	0	N.A.	0
SPAG4	0,40703544	0,049579246	0	N.A.	0
CTBS	0,406547529	0,146184656	0	N.A.	0
LY6D	0,406274892	0,336809208	0	N.A.	0
SHROOM1	0,40623513	0,445694169	0	N.A.	0
CYTH3	0,406051206	0,335488729	0	N.A.	0
NPLOC4	0,405686617	0,368330146	0	N.A.	0

MESDC1	0,405595224	0,292878835	0	N.A.	0
BTG1	0,405220374	0,283507515	0	N.A.	0
LINC00152	0,405171813	-0,043000732	0	N.A.	0
ZNF562	0,405105728	0,38255164	0	N.A.	0
TANC1	0,4046376	0,488983304	0	N.A.	0
MBOAT2	0,404607432	0,644541866	0	N.A.	0
ABCC3	0,404583447	0,650258053	0	N.A.	0
KRAS	0,404545211	0,592043451	0	N.A.	0
DEFB1	0,404501956	0,15194214	0	N.A.	0
SAMD8	0,404328832	-0,019848075	0	N.A.	0
U47924.27	0,403952834	0,226893046	0	N.A.	0
HCFC1R1	0,403881013	-0,151191688	0	N.A.	0
PIK3C2B	0,403844685	0,275358561	0	N.A.	0
PELI2	0,403542436	0,170078916	0	N.A.	0
SNX33	0,403502609	0,193692372	0	N.A.	0
CORO2A	0,403440617	0,616381229	0	N.A.	0
RAP2C	0,403437423	0,098714407	0	N.A.	0
SERPINB1	0,403111339	0,436029522	0	N.A.	0
APOBEC1	0,403040114	0,164361026	0	N.A.	0
UBE2Z	0,402800742	0,363562356	0	N.A.	0
LAIR2	0,402747508	0,033619399	0	N.A.	0
CTGF	0,402730579	0,499546118	0	N.A.	0
GPR35	0,402438716	0,535741598	0	N.A.	0
CDKN2A	0,402390013	0,590180696	0	N.A.	0
YPEL3	0,402377685	0,592304078	0	N.A.	0
GDPD3	0,402145771	0,499391077	1	cl1	0
TPPP	0,40211452	0,236716207	0	N.A.	0
WFDC3	0,402077701	0,394331188	1	cl1	0
CARD10	0,401517686	0,554103442	0	N.A.	0
KLF4	0,401109545	0,379811591	0	N.A.	0
ATP11B	0,400991027	0,427714105	0	N.A.	0
ARHGAP26	0,400894674	0,277516993	0	N.A.	0
ATP6V0E1	0,400816911	0,555835771	0	N.A.	0

TCN2	0,400368573	0,674563413	0	N.A.	0
PQLC3	0,400298467	0,545657329	0	N.A.	0
SYNPO	0,400224147	0,316088648	0	N.A.	0
ACVRL1	0,400194996	0,19052084	0	N.A.	0
PIM3	0,400170537	0,395319187	0	N.A.	0
PACSIN2	0,400114169	0,551987423	0	N.A.	0
OSMR	0,400100965	-0,123883935	0	N.A.	0
SLC12A4	0,400080431	0,343556414	0	N.A.	0
SMG1	0,400029579	0,451917909	0	N.A.	0
IRAK2	0,399945006	0,234569935	0	N.A.	0
WDR45	0,399882159	0,270295567	0	N.A.	0
IL6ST	0,399815181	0,431702068	0	N.A.	0
RIN2	0,399446435	0,344858536	0	N.A.	0
MAFF	0,399436374	0,595028678	0	N.A.	0
EGLN1	0,399250038	0,378696866	0	N.A.	0
MTRNR2L8	0,399213383	0,082871708	0	N.A.	0
CD82	0,3991838	0,634527685	0	N.A.	0
RAMP1	0,399157052	0,396179191	0	N.A.	0
TMEM184A	0,39904649	0,344946433	0	N.A.	0
CTDSP2	0,398700394	0,412854541	0	N.A.	0
RP11-411K7.	0,39869582	0,290296544	0	N.A.	0
FBXW5	0,398237633	0,720820063	0	N.A.	0
ARHGAP8	0,398219962	0,362572895	0	N.A.	0
LY6G6C	0,398194204	0,224129681	0	N.A.	0
CES2	0,397955475	0,283207222	0	N.A.	0
NCKAP1	0,397702736	0,551446027	0	N.A.	0
CAV2	0,397687548	0,695563775	0	N.A.	0
MMP28	0,39742917	0,537540243	0	N.A.	0
CLIC4	0,397391457	-0,015755785	0	N.A.	0
CACNG4	0,397094549	0,270107072	0	N.A.	0
XAF1	0,397032767	0,42445057	0	N.A.	0
TMCC1	0,396882254	0,343745341	0	N.A.	0
IFNLR1	0,396601006	0,300972186	0	N.A.	0

S100A3	0,396555515	0,10173849	0	N.A.	0
TMEM159	0,396518821	0,519754963	0	N.A.	0
HLA-A	0,396451419	0,54591721	0	N.A.	0
AC093673.5	0,396237277	-0,184318944	0	N.A.	0
GNA11	0,396159716	0,548663337	0	N.A.	0
DENND5B	0,39595232	0,439824744	0	N.A.	0
CRIP1	0,395579733	0,290746919	0	N.A.	0
FBLIM1	0,395536946	0,558764981	0	N.A.	0
FAM134B	0,395495948	0,369866796	0	N.A.	0
SPRR2D	0,39540413	0,154213377	0	N.A.	0
PDE9A	0,395308324	0,056260406	0	N.A.	0
KIF16B	0,395154309	0,307465328	0	N.A.	0
ZNF165	0,395099338	0,228907399	0	N.A.	0
ARSD	0,395075662	0,548075094	0	N.A.	0
RSPH3	0,394813692	0,259595603	0	N.A.	0
TOR1AIP2	0,39478607	0,648316249	0	N.A.	0
RASSF7	0,394499297	0,808946434	0	N.A.	0
CLDN7	0,394481228	0,598178509	0	N.A.	0
PRR5L	0,394464928	0,261782413	0	N.A.	0
PDLIM7	0,394063767	0,376188214	0	N.A.	0
DAZAP2	0,394004875	-0,049312921	0	N.A.	0
MGAT4A	0,393857819	0,233458041	0	N.A.	0
TNFRSF14	0,393595103	0,518282096	0	N.A.	0
CCK	0,393528872	0,174019155	0	N.A.	0
SLC37A1	0,392853105	0,661666654	0	N.A.	0
HGSNAT	0,392644349	0,611242624	0	N.A.	0
PTGES	0,392385795	0,347985747	0	N.A.	0
SAMD4A	0,392288904	0,496287279	0	N.A.	0
IL10RB	0,392189049	0,563399541	0	N.A.	0
NPAS2	0,392081203	0,333103052	0	N.A.	0
LPIN3	0,391987382	0,275025166	0	N.A.	0
ITGB5	0,39196922	0,67995202	0	N.A.	0
CH17-360D5.	0,391728322	0,532496733	0	N.A.	0

PPP1R16A	0,39167982	0,43214126	0	N.A.	0
SHH	0,391613537	0,115639647	0	N.A.	0
GDI1	0,391184408	0,50815573	0	N.A.	0
AC005256.1	0,391161987	0,277325721	0	N.A.	0
IKZF2	0,391062234	0,169164614	0	N.A.	0
IGF2BP2	0,391060441	0,499339403	0	N.A.	0
SLC35E1	0,391047334	0,37107892	0	N.A.	0
BCAS1	0,390851101	0,680863411	0	N.A.	0
TNFRSF10B	0,390753374	0,485963949	0	N.A.	0
NT5C2	0,390720609	0,338833375	0	N.A.	0
SLC6A9	0,390478293	0,132492448	0	N.A.	0
RP1-313I6.12	0,390374963	0,042923395	0	N.A.	0
CYP4F11	0,390174411	0,458209329	0	N.A.	0
PFKFB2	0,390143881	0,197355603	0	N.A.	0
CKB	0,389993009	0,539059809	0	N.A.	0
KIAA1671	0,389968439	0,395249433	0	N.A.	0
UPK2	0,389938354	-0,121395299	0	N.A.	0
ONECUT3	0,38993218	0,093391965	0	N.A.	0
ATP8B1	0,389785677	0,318791501	0	N.A.	0
CACFD1	0,389678922	0,663556819	0	N.A.	0
RAB21	0,389515289	0,523332737	0	N.A.	0
RELA	0,389392788	0,247609799	0	N.A.	0
KDM1B	0,389008165	0,210204065	0	N.A.	0
B3GNT8	0,388696709	0,294763489	0	N.A.	0
CREBRF	0,388505638	0,319328738	0	N.A.	0
PTEN	0,388454241	0,080273383	0	N.A.	0
ENTPD2	0,387993856	0,454342133	0	N.A.	0
VPS37B	0,387840107	0,505883875	0	N.A.	0
SEMA6A	0,38728478	0,182451651	0	N.A.	0
VEZT	0,387128323	0,524726648	0	N.A.	0
VCL	0,38690034	0,507285106	0	N.A.	0
RABAC1	0,386666903	0,422805769	0	N.A.	0
TSC22D4	0,386252528	0,315654206	0	N.A.	0

LGALSL	0,386075456	0,281155181	0	N.A.	0
BLCAP	0,385972962	0,561845459	0	N.A.	0
SOCS6	0,385907278	0,228525706	0	N.A.	0
MPPE1	0,385882982	0,365824069	0	N.A.	0
ATP6V1D	0,385713331	0,313813837	0	N.A.	0
FBXO11	0,385530374	0,093785088	0	N.A.	0
LSMEM1	0,385493335	0,379336771	0	N.A.	0
DUSP6	0,385114155	0,388329042	0	N.A.	0
RAPGEF2	0,385092413	0,61702884	0	N.A.	0
GOLM1	0,385068428	0,422647362	0	N.A.	0
PQLC1	0,385036635	0,429074579	0	N.A.	0
DNASE1L1	0,385009281	0,119784509	0	N.A.	0
PHLDA2	0,384940552	0,689888686	0	N.A.	0
DPY19L1	0,384643092	0,490251888	0	N.A.	0
SEL1L	0,384602026	0,382323162	0	N.A.	0
SLC41A2	0,384183815	0,430684841	0	N.A.	0
SAA2	0,384148648	0,521409146	0	N.A.	0
EPPK1	0,384002892	0,3978523	0	N.A.	0
SP140L	0,383960768	0,274721482	0	N.A.	0
LAMA4	0,383538148	0,143773673	0	N.A.	0
MAP3K8	0,383447141	0,152007877	0	N.A.	0
PDLIM2	0,383114478	0,312637301	0	N.A.	0
CCDC120	0,383086192	0,551400598	0	N.A.	0
DDX60L	0,382895526	0,47000923	0	N.A.	0
RNF141	0,382797295	0,06846283	0	N.A.	0
EDN2	0,382696445	0,311776501	0	N.A.	0
CUX1	0,382513767	0,309866049	0	N.A.	0
ERRFI1	0,382344969	0,691817867	0	N.A.	0
CHST4	0,382130137	-0,003320721	0	N.A.	0
SPATA2L	0,382101534	0,090953536	0	N.A.	0
GABARAPL1	0,38204768	0,300027753	0	N.A.	0
LRCH4	0,381849784	0,353079401	0	N.A.	0
NEBL	0,381767483	0,54884866	0	N.A.	0

MFSD6	0,38134486	0,315306735	0	N.A.	0
ELK3	0,381277203	0,219835763	0	N.A.	0
CREB3	0,381251168	0,331831267	0	N.A.	0
PRR15	0,381224014	0,399018617	0	N.A.	0
ARL5B	0,381135302	0,428035878	0	N.A.	0
PRKD2	0,380797909	0,287662827	0	N.A.	0
LRCH1	0,380728288	0,47996353	0	N.A.	0
KIAA0232	0,380650065	0,438498518	0	N.A.	0
ABHD11	0,38055054	0,297479728	0	N.A.	0
SESN2	0,380547721	0,580388968	0	N.A.	0
CNKSR3	0,37972121	0,427811029	0	N.A.	0
ACOX1	0,379682098	0,47654866	0	N.A.	0
KDM4B	0,379554065	0,460376414	0	N.A.	0
CDKN2B-AS1	0,379446639	0,277611142	0	N.A.	0
MICALL2	0,379429642	0,663077698	0	N.A.	0
IGFL1	0,379376902	0,315398309	0	N.A.	0
PJA2	0,379187419	0,011327052	0	N.A.	0
DNAJB2	0,378810446	0,571108142	0	N.A.	0
SAMD9L	0,378751353	0,423061906	0	N.A.	0
ADM2	0,378622965	0,323540454	0	N.A.	0
VAT1	0,378565254	0,360976687	0	N.A.	0
PSD4	0,378318349	0,636749381	0	N.A.	0
UNC5B	0,377957032	0,320655895	0	N.A.	0
FRK	0,377744588	0,605494441	0	N.A.	0
LANCL3	0,377580372	0,272636367	0	N.A.	0
IL1RAP	0,377552662	0,224202773	0	N.A.	0
GBP1	0,377474043	-0,037423043	0	N.A.	0
TAB2	0,377454789	0,241739064	0	N.A.	0
FAM120AOS	0,376995785	0,105403174	0	N.A.	0
IFNAR2	0,376993618	0,393944508	0	N.A.	0
TTYH3	0,376890981	0,424777482	0	N.A.	0
VAMP8	0,376805136	0,538623156	0	N.A.	0
ZFP36L1	0,375942051	0,454816702	0	N.A.	0

PLA2G2F	0,375818157	0,323986409	0	N.A.	0
SPINK1	0,375817808	0,145582601	0	N.A.	0
TM4SF4	0,375786695	0,482756223	1	cls4	0
NPAS1	0,375704718	0,376522557	0	N.A.	0
LGALS3	0,375618402	0,505290735	0	N.A.	0
SH3PXD2A-A	0,375580291	0,141128625	0	N.A.	0
WSB1	0,3752936	0,512318693	0	N.A.	0
C3orf52	0,37460145	0,508031168	0	N.A.	0
TMEM53	0,374414828	0,282155513	0	N.A.	0
PARP8	0,374412746	0,471758673	0	N.A.	0
DYNLT3	0,374183281	0,277436561	0	N.A.	0
DPCR1	0,374177714	-0,041725672	0	N.A.	0
CLDN3	0,374072493	0,405486759	0	N.A.	0
MED13L	0,373965407	0,38717862	0	N.A.	0
MUC20	0,373752849	0,665379709	0	N.A.	0
MOSPD1	0,373670361	0,403417456	0	N.A.	0
MNT	0,373651753	0,154643412	0	N.A.	0
C15orf39	0,373585733	0,392899015	0	N.A.	0
ADIRF	0,373417497	0,174029921	0	N.A.	0
MAP3K2	0,373364086	0,535794414	0	N.A.	0
HLA-C	0,373071937	0,371336494	0	N.A.	0
MARCKS	0,37294437	0,199723881	0	N.A.	0
ACAP2	0,372404509	0,375345443	0	N.A.	0
RP11-462L8.1	0,372317479	0,378153047	0	N.A.	0
CRY2	0,372210931	0,452974444	0	N.A.	0
ZSWIM4	0,372145643	0,320972868	0	N.A.	0
RANBP9	0,371897199	0,353052293	0	N.A.	0
IFNAR1	0,371874756	0,304285341	0	N.A.	0
ATP2B1	0,371821908	0,502983741	0	N.A.	0
PAFAH1B1	0,371500092	0,118779229	0	N.A.	0
TMEM41B	0,371418311	0,582999799	0	N.A.	0
CTTNBP2NL	0,371144112	0,387515865	0	N.A.	0
NTN4	0,37113327	0,144160116	0	N.A.	0

VDR	0,371083984	0,359907501	0	N.A.	0
AGPAT9	0,371065118	0,375147235	0	N.A.	0
CASP7	0,371019665	0,428319756	0	N.A.	0
RP11-285E9.	0,370983281	0,385221768	0	N.A.	0
SERINC3	0,37088684	0,392440611	0	N.A.	0
SMG7	0,370510478	0,614341315	0	N.A.	0
SLC35F5	0,370406307	0,489930825	0	N.A.	0
TMED7	0,370008715	0,426627526	0	N.A.	0
CAP1	0,369989178	0,504539576	0	N.A.	0
PRSS12	0,369908742	0,734141549	0	N.A.	0
WDFY1	0,369908362	0,311382638	0	N.A.	0
PFKP	0,369730202	0,518844298	0	N.A.	0
NRARP	0,36971397	0,07710495	0	N.A.	0
PDXK	0,369638664	0,663466795	0	N.A.	0
TMEM59	0,36953896	0,458886381	0	N.A.	0
SPIRE2	0,369522649	0,226779482	0	N.A.	0
FAM63B	0,369202644	0,525996923	0	N.A.	0
MCU	0,369175001	0,083343191	0	N.A.	0
FBXO2	0,369055872	0,371673907	0	N.A.	0
ALCAM	0,368830234	0,440778196	0	N.A.	0
SP100	0,36881637	0,122541579	0	N.A.	0
KRT8	0,368520789	0,63534071	0	N.A.	0
TPP1	0,368515736	0,269889837	0	N.A.	0
IFIT2	0,368305104	0,403602054	0	N.A.	0
STEAP4	0,367846993	0,476640043	0	N.A.	0
BET1L	0,367701783	0,20551472	0	N.A.	0
ZNF524	0,367687411	0,365789716	0	N.A.	0
NT5C3A	0,367504102	0,303107797	0	N.A.	0
ELF4	0,36749705	-0,044285164	0	N.A.	0
COL16A1	0,367332982	0,56608875	0	N.A.	0
SIRT1	0,367321693	0,101338253	0	N.A.	0
GRB10	0,367240378	0,253900406	0	N.A.	0
MARVELD2	0,36713348	0,396155245	0	N.A.	0

HLA-B	0,366894613	0,476967	0	N.A.	0
AP001610.9	0,366826393	0,326315321	0	N.A.	0
SHB	0,366822495	0,31870048	0	N.A.	0
DENND1B	0,366764331	0,385052952	0	N.A.	0
MTRNR2L12	0,366717721	0,097668043	0	N.A.	0
BTNL3	0,366688249	0,398003783	0	N.A.	0
GIPR	0,366577171	0,441132545	0	N.A.	0
ENO2	0,366570335	0,373291937	0	N.A.	0
ATXN7	0,366491102	0,310875755	0	N.A.	0
PCDH7	0,366381815	0,102648112	0	N.A.	0
CCNL1	0,366264498	0,351483694	0	N.A.	0
KDM5C	0,36625092	0,476211957	0	N.A.	0
NEDD9	0,36616344	0,525286946	0	N.A.	0
PSCA	0,365992817	0,546971186	0	N.A.	0
BIRC2	0,365893314	0,451371462	0	N.A.	0
ITSN1	0,365723902	0,653301177	0	N.A.	0
MFSD4	0,365349714	0,365910307	0	N.A.	0
TFE3	0,365299756	0,359363627	0	N.A.	0
SEC24D	0,365295243	0,326943939	0	N.A.	0
ARHGAP17	0,365242646	0,376224326	0	N.A.	0
GSKIP	0,365063645	0,30133275	0	N.A.	0
VPS4B	0,364978079	0,346748268	0	N.A.	0
ATG9B	0,364780791	0,247893821	0	N.A.	0
FAM83G	0,364735842	0,290728093	0	N.A.	0
STYK1	0,364602185	0,623836804	0	N.A.	0
MAP4K4	0,364241595	0,388178642	0	N.A.	0
ANO8	0,364064366	0,255381814	0	N.A.	0
NRP2	0,363890139	0,455950609	0	N.A.	0
RIMKLA	0,36384034	0,024933116	0	N.A.	0
TMPRSS3	0,363396914	0,387106147	0	N.A.	0
SLC25A25	0,363265421	0,52020148	1	cl1	0
SEMA3B-AS1	0,363264006	0,128632358	0	N.A.	0
KIAA1191	0,363194582	0,364173357	0	N.A.	0

RDH10	0,363191939	0,490418133	0	N.A.	0
AMFR	0,363106836	0,444120966	0	N.A.	0
PHYKPL	0,36309708	0,47860703	0	N.A.	0
SPINK7	0,363054574		0	N.A.	0
AQP12A	0,363047629	-0,005496908	0	N.A.	0
LY75	0,363045325	0,619820304	0	N.A.	0
IRS1	0,362993297	0,465528665	0	N.A.	0
ATG2A	0,362954273	0,280446128	0	N.A.	0
TRAM1	0,362893171	0,698566255	0	N.A.	0
PADI1	0,362341216	0,086935548	0	N.A.	0
ATP13A2	0,362115442	0,684973252	0	N.A.	0
C1orf210	0,361943738	0,299121337	0	N.A.	0
MAF	0,361914951	-0,036257154	0	N.A.	0
TOR1B	0,361816973	0,403886525	0	N.A.	0
HIST1H3E	0,361531687	0,316233546	0	N.A.	0
MUC3A	0,361171689	0,286140782	0	N.A.	0
SLC39A14	0,36101275	0,524462209	0	N.A.	0
VAPA	0,360640314	0,272450021	0	N.A.	0
AQP7	0,360502973	0,276222106	0	N.A.	0
SETD9	0,360433113	0,028995128	0	N.A.	0
DYRK2	0,360229614	0,673033634	0	N.A.	0
PADI2	0,360189268	0,218776286	0	N.A.	0
PPARA	0,360071605	0,241014152	0	N.A.	0
CA13	0,359717834	0,308099904	0	N.A.	0
IFI6	0,359654556	0,593684128	0	N.A.	0
UFM1	0,359653039	0,330639009	0	N.A.	0
STK10	0,359556384	-0,239516452	0	N.A.	0
HLA-G	0,359517577	0,481679269	0	N.A.	0
C6orf223	0,359470623	0,466094206	0	N.A.	0
MAGT1	0,359460091	0,232587644	0	N.A.	0
MARK4	0,35942809	-0,003767225	0	N.A.	0
NCEH1	0,359213638	0,532931418	0	N.A.	0
NR2C2	0,359070621	0,170882912	0	N.A.	0

NFIL3	0,358530023	0,429610309	0	N.A.	0
RP11-7K24.3	0,358425498	0,327701322	0	N.A.	0
VPS18	0,358391933	0,296694896	0	N.A.	0
GLS	0,358385214	0,60715853	0	N.A.	0
WWC1	0,358384653	0,583774956	0	N.A.	0
P2RX4	0,358068087	0,513043631	0	N.A.	0
SBF2	0,357923865	0,15853171	0	N.A.	0
CLIP2	0,357633892	0,389409304	0	N.A.	0
RAB6A	0,357412338	0,308244497	0	N.A.	0
COG3	0,357307203	0,306395344	0	N.A.	0
CTSO	0,357268174	0,183749209	0	N.A.	0
NEURL3	0,357150883	0,378213542	0	N.A.	0
TESK1	0,357099525	0,298403376	0	N.A.	0
TMF1	0,357067921	0,365326119	0	N.A.	0
C1QL1	0,356790286	0,132655245	0	N.A.	0
MMP1	0,356743523	0,34036294	0	N.A.	0
CAMK2D	0,35674338	0,243214964	0	N.A.	0
MOGAT3	0,356651291	0,482585167	0	N.A.	0
C1orf115	0,356601456	0,269701716	0	N.A.	0
MAFG	0,356336811	0,402793971	0	N.A.	0
INAFM1	0,35625219	-0,115238508	0	N.A.	0
MICAL3	0,356158534	0,388814481	0	N.A.	0
RP11-350J20	0,356154514	0,339680368	0	N.A.	0
BAIAP2L2	0,355624247	0,349488827	0	N.A.	0
MROH1	0,35560266	0,415843874	0	N.A.	0
C1orf167	0,35545408	0,381000921	0	N.A.	0
RCAN1	0,355424343	0,229377266	0	N.A.	0
SLCO2B1	0,355282759	0,076448083	0	N.A.	0
PWWP2B	0,355145701	0,181684002	0	N.A.	0
PDZD3	0,355046905	0,232419287	0	N.A.	0
OCEL1	0,354983331	0,2430284	0	N.A.	0
ITPRIPL2	0,354905001	0,397145727	0	N.A.	0
LPAR5	0,354879908	0,612894478	0	N.A.	0

MBD2	0,35477965	0,469956259	0	N.A.	0
TFAP2C	0,35468601	0,459609238	0	N.A.	0
LINC01207	0,354377796	0,424185081	0	N.A.	0
ULK1	0,354337735	0,486752932	0	N.A.	0
SERPINB7	0,353920847	0,127840344	0	N.A.	0
SYTL2	0,353757708	0,437626849	0	N.A.	0
TMSB4X	0,35330644	0,036353208	0	N.A.	0
SLC25A24	0,353285333	0,435102655	0	N.A.	0
MOSPD2	0,353027187	0,303824749	0	N.A.	0
ZHX2	0,353001366	0,362211519	0	N.A.	0
ARHGEF5	0,352965293	0,285845579	0	N.A.	0
CSNK1E	0,35295457	0,473297266	0	N.A.	0
SAMD4B	0,352787242	0,163812324	0	N.A.	0
TM4SF20	0,35270175	0,290484974	0	N.A.	0
USP25	0,352694709	0,330389332	0	N.A.	0
NOSTRIN	0,352674839	0,262916001	0	N.A.	0
TM2D1	0,352654436	0,321200532	0	N.A.	0
SIRT6	0,352570633	0,425684393	0	N.A.	0
AMIGO2	0,35248359	0,655916694	0	N.A.	0
C4orf32	0,352430634	0,335039337	0	N.A.	0
CD97	0,351924459	0,290866845	0	N.A.	0
SULT1C2	0,351822931	0,342154519	0	N.A.	0
MAP4K3	0,351524369	0,316072834	0	N.A.	0
AAK1	0,351436246	0,41935799	0	N.A.	0
TMEM189	0,351398891	0,51952837	0	N.A.	0
URGCP	0,351391641	0,247738736	0	N.A.	0
NUDT4	0,35107542	0,20826162	0	N.A.	0
RNF19A	0,35102743	0,493259884	0	N.A.	0
CPA4	0,35089747	0,058643342	0	N.A.	0
LGALS9	0,350692753	0,26582631	0	N.A.	0
ANXA3	0,350516956	0,561932943	1	none	0
KRT34	0,35043105	0,333407989	0	N.A.	0
B4GALT1-AS1	0,350366423	-0,143701085	0	N.A.	0

CHIC2	0,350140636	0,090931566	0	N.A.	0
ADAM19	0,350091739	0,43762415	0	N.A.	0
SLC22A18AS	0,350022779	0,307473521	0	N.A.	0
KIAA1161	0,349812637	0,175366749	0	N.A.	0
IGFBP3	0,349740177	0,47170004	0	N.A.	0
RASSF6	0,349684063	0,749791798	0	N.A.	0
DPH3	0,349647598	0,157087232	0	N.A.	0
OSBPL3	0,349584447	0,534776966	0	N.A.	0
ACOT11	0,349510405	0,494632478	0	N.A.	0
GLRX	0,349318301	0,345353524	0	N.A.	0
RP11-468E2.4	0,349229533	0,192334686	0	N.A.	0
TTC7A	0,349143573	0,501044555	0	N.A.	0
TFPI	0,349133354	0,577929649	0	N.A.	0
WASF2	0,349074133	0,400862795	0	N.A.	0
FZD5	0,349024231	0,395353206	0	N.A.	0
STK17A	0,348773572	0,174717021	0	N.A.	0
TACC2	0,348679008	0,457485717	0	N.A.	0
CACNA1C	0,348598129	0,284907941	0	N.A.	0
RABIF	0,348529548	0,376068286	0	N.A.	0
AL049794.1	0,348523078	0,05444932	0	N.A.	0
KRT6A	0,348487892	0,538086096	1	cls3	0
FAM13A	0,348451679	0,252953355	0	N.A.	0
ZMYM2	0,348219638	0,457538	0	N.A.	0
AES	0,348123916	0,621200594	0	N.A.	0
HYI	0,348110818	0,4784884	0	N.A.	0
PLXNA1	0,347920283	0,462153662	0	N.A.	0
C15orf52	0,347685557	0,580260434	0	N.A.	0
RALB	0,347673269	0,284165469	0	N.A.	0
VPS9D1	0,347604833	0,380528718	0	N.A.	0
WBP1L	0,34746768	0,147683121	0	N.A.	0
SH3BP2	0,347466101	0,455393009	0	N.A.	0
ABLIM3	0,347263479	0,391458099	0	N.A.	0
DAPK3	0,347215162	0,441081601	0	N.A.	0

AHDC1	0,347019785	0,213749683	0	N.A.	0
WWP1	0,346823878	0,200771875	0	N.A.	0
UBXN6	0,346541249	0,155773374	0	N.A.	0
RAB11FIP1	0,346508927	0,717692479	0	N.A.	0
SLC22A3	0,346488309	0,236615501	0	N.A.	0
NWD2	0,34633786	0,332741647	0	N.A.	0
LINC01186	0,346296259	-0,168920298	0	N.A.	0
RAB9A	0,346149388	0,499068405	0	N.A.	0
BAMBI	0,346038727	0,547467368	0	N.A.	0
EFNA1	0,346011288	0,650839695	0	N.A.	0
TPBG	0,345872042	0,483318033	0	N.A.	0
MSX2	0,345840597	0,406456637	0	N.A.	0
AP001187.9	0,345817102	0,388823795	0	N.A.	0
MGAT4B	0,34575761	0,557264507	0	N.A.	0
ZNF217	0,345686309	0,42099042	0	N.A.	0
VWA1	0,345621949	0,701508787	0	N.A.	0
STX12	0,345608236	0,148723158	0	N.A.	0
CLDN9	0,345466053	0,177740044	0	N.A.	0
RBM47	0,345334337	0,364040069	0	N.A.	0
PPFIA1	0,345322265	0,42127171	0	N.A.	0
GNE	0,345304035	0,354675845	0	N.A.	0
GNA15	0,345246823	0,210515204	0	N.A.	0
TOM1	0,345119195	0,377825461	0	N.A.	0
BTBD6	0,34497048	0,071998034	0	N.A.	0
MAST2	0,344685592	0,396521845	0	N.A.	0
SH3BGRL2	0,344581995	0,405727416	0	N.A.	0
MX1	0,34457932	0,517222636	0	N.A.	0
RAET1G	0,344529744	0,462049016	0	N.A.	0
MTM1	0,344441846	-0,026491928	0	N.A.	0
SMCHD1	0,344323601	0,149650785	0	N.A.	0
TPMT	0,344178908	0,265078899	0	N.A.	0
MTHFR	0,344130219	0,296304469	0	N.A.	0
LEPROT	0,343994043	0,418895458	0	N.A.	0

PLAU	0,343977256	0,449578904	0	N.A.	0
NDFIP2	0,343721296	0,606821907	0	N.A.	0
LAMC1	0,34355493	0,167023879	0	N.A.	0
CNNM4	0,34338014	0,420918656	0	N.A.	0
CARD6	0,343126485	0,464193752	0	N.A.	0
XRN1	0,34308529	0,186917826	0	N.A.	0
CLCN3	0,343027911	0,424136839	0	N.A.	0
SLC38A2	0,34292639	0,291459291	0	N.A.	0
KLF11	0,34291257	-0,043118487	0	N.A.	0
SWAP70	0,342630317	0,389706783	0	N.A.	0
RASA2	0,342474087	0,356204024	0	N.A.	0
TNFRSF10D	0,342415489	0,143889817	0	N.A.	0
PITX1	0,342405234	0,437626145	0	N.A.	0
C11orf68	0,342250282	-0,144749122	0	N.A.	0
EID3	0,342112702	0,030472725	0	N.A.	0
ARRDC2	0,342081601	0,148046341	0	N.A.	0
PODXL	0,34207703	0,492536769	0	N.A.	0
DVL1	0,34199707	0,443206885	0	N.A.	0
ARHGAP1	0,341994241	0,378906752	0	N.A.	0
KPNA4	0,341989712	0,285366475	0	N.A.	0
INSC	0,341872611		0	N.A.	0
CASK	0,341621418	0,184701409	0	N.A.	0
AGFG1	0,341606524	0,48756273	0	N.A.	0
KMT2C	0,341355912	0,495711055	0	N.A.	0
DDAH2	0,341022745	0,323756776	0	N.A.	0
BCAR3	0,340951817	0,209332751	0	N.A.	0
LTBP4	0,340871198	0,442772616	0	N.A.	0
OTUD7B	0,340788475	0,369782931	0	N.A.	0
PAK2	0,340686752	0,189832545	0	N.A.	0
CD47	0,340650376	0,463712138	0	N.A.	0
PLEKHA7	0,340513856	0,548432315	0	N.A.	0
PARP4	0,340494326	0,331605016	0	N.A.	0
ATP11A	0,339895491	0,515019461	0	N.A.	0

TSPAN31	0,339767695	0,361911599	0	N.A.	0
GUCD1	0,339330202	0,364964433	0	N.A.	0
C6orf222	0,339133022	0,296623188	0	N.A.	0
MAP2K2	0,339102455	0,465012973	0	N.A.	0
TOLLIP	0,33883029	0,480037927	0	N.A.	0
C6orf106	0,338492427	0,172732508	0	N.A.	0
SLC9A8	0,338325746	0,128752326	0	N.A.	0
SGK223	0,338311431	0,51135067	0	N.A.	0
STARD4	0,33828559	0,138473154	0	N.A.	0
FAM101A	0,337963709	0,57913217	0	N.A.	0
NR3C2	0,337896825	0,160256089	0	N.A.	0
LYNX1	0,337817828	-0,105868007	0	N.A.	0
KCTD1	0,337762199	0,370965774	0	N.A.	0
DNMBP	0,337305996	0,245675335	0	N.A.	0
C15orf48	0,337236527	0,534193014	0	N.A.	0
ERBB3	0,337226819	0,553613626	0	N.A.	0
TLCD2	0,336937281	0,472097963	0	N.A.	0
ST3GAL4	0,336664809	0,475226666	0	N.A.	0
RP11-553L6.!	0,336656504	0,567639935	0	N.A.	0
NR1D2	0,336641108	0,329791859	0	N.A.	0
DNAJB9	0,336600628	0,348737141	0	N.A.	0
RHOG	0,336436236	0,274038753	0	N.A.	0
OSTM1	0,336223097	0,144837603	0	N.A.	0
KLF13	0,335975247	0,310803512	0	N.A.	0
ARAP2	0,335922924	0,224948242	0	N.A.	0
S100A4	0,335713871	-0,095468774	0	N.A.	0
MRVI1-AS1	0,335485765	-0,078773465	0	N.A.	0
HIPK3	0,335449154	0,073880593	0	N.A.	0
ERLEC1	0,335333245	0,356667766	0	N.A.	0
ACBD3	0,335116758	0,577872231	0	N.A.	0
A4GALT	0,335004228	0,36988541	0	N.A.	0
TRIM69	0,335000765	0,01837874	0	N.A.	0
CYSRT1	0,334803962	0,395091067	0	N.A.	0

PAQR8	0,334770852	0,220388949	0	N.A.	0
TBC1D1	0,33469505	0,164149673	0	N.A.	0
KIF1C	0,33437169	0,263666578	0	N.A.	0
GPR126	0,334127082	0,508106225	0	N.A.	0
SCYL2	0,334044206	0,302461186	0	N.A.	0
STK3	0,333972923	0,256060475	0	N.A.	0
HDAC5	0,333905352	0,363248897	0	N.A.	0
CBLB	0,333668561	0,558071197	0	N.A.	0
TMEM136	0,333591378	0,022539653	0	N.A.	0
ZC3HAV1	0,333525037	0,228341881	0	N.A.	0
PTAFR	0,33344322	0,380188377	0	N.A.	0
TPO	0,333358152	0,205669107	0	N.A.	0
YPEL5	0,333328528	0,374753243	0	N.A.	0
HSPB8	0,333062487	0,226841782	0	N.A.	0
CAV1	0,333017431	0,000136606	0	N.A.	0
IL6R	0,332987649	0,19587787	0	N.A.	0
CLDN1	0,332738895	0,53055288	0	N.A.	0
EXOC7	0,33272556	0,353234379	1	cl1	0
CPNE8	0,332692384	0,426883083	0	N.A.	0
AVL9	0,332518595	0,340337365	0	N.A.	0
ZBTB34	0,332477837	0,251315079	0	N.A.	0
CCNG2	0,332420751	0,512435643	0	N.A.	0
GPR87	0,332369568	0,260654866	0	N.A.	0
ADAM17	0,331861321	0,429865957	0	N.A.	0
UBA6	0,331785897	0,409444946	0	N.A.	0
TPRG1L	0,33158918	0,575609195	0	N.A.	0
TRIP12	0,331556631	0,342480522	0	N.A.	0
AVPI1	0,331534791	0,440564234	0	N.A.	0
SEC61A1	0,331464857	0,530299117	0	N.A.	0
SPSB1	0,331462811	0,189809186	0	N.A.	0
OSBPL2	0,331257362	0,390738928	0	N.A.	0
ACVR2A	0,331116618	0,280503942	0	N.A.	0
CD109	0,33109085	0,521307369	0	N.A.	0

OSBP2	0,331031619	0,238802249	0	N.A.	0
IGSF9	0,330826273	0,328894148	0	N.A.	0
SEMA5A	0,330396595	0,50963557	0	N.A.	0
IGFBP1	0,329853704	0,279647257	0	N.A.	0
ETV3	0,329835936	0,402274055	0	N.A.	0
RNF145	0,329777253	0,538773222	0	N.A.	0
GAREM	0,329710373	0,412864604	0	N.A.	0
GPR108	0,329468556	0,598974353	0	N.A.	0
SERINC5	0,329447205	0,337445016	0	N.A.	0
TSC22D2	0,329422732	0,513943715	0	N.A.	0
LGI4	0,329381279	0,129405168	0	N.A.	0
LURAP1L	0,329246952	0,57480293	0	N.A.	0
DTX3L	0,329224085	0,33985303	0	N.A.	0
TRIM8	0,329223978	0,440074788	0	N.A.	0
SAA1	0,329175334	0,490887892	0	N.A.	0
INPP1	0,329117443	0,500462328	0	N.A.	0
MBOAT7	0,328933141	0,802031279	0	N.A.	0
RNF11	0,328895449	0,033490512	0	N.A.	0
YIPF3	0,328858628	0,215697545	0	N.A.	0
CST6	0,328104392	0,415487183	0	N.A.	0
MIEF2	0,327947874	0,238763979	0	N.A.	0
TMEM54	0,327943472	0,545863423	0	N.A.	0
SLC16A4	0,327929592	0,683112033	0	N.A.	0
RXRA	0,327922095	0,487559415	0	N.A.	0
HIST1H2BC	0,327853711	0,179673902	0	N.A.	0
KIAA0430	0,327799122	0,192935705	0	N.A.	0
COL6A1	0,327704166	0,517562138	0	N.A.	0
TNNT1	0,327626824	0,257522836	0	N.A.	0
PLEKHG1	0,327594619	0,41034591	0	N.A.	0
SLC15A1	0,3275894	0,258149125	0	N.A.	0
HECTD1	0,327519905	0,442362453	0	N.A.	0
LNX2	0,327399727	0,322578013	0	N.A.	0
ALDOB	0,327241719	0,208833523	0	N.A.	0

LMTK2	0,327137101	0,319841019	0	N.A.	0
BACH2	0,327125193	0,081896759	0	N.A.	0
GSDMB	0,327108577	0,526039724	0	N.A.	0
ITM2C	0,32683598	0,142042309	0	N.A.	0
PTPRJ	0,326434108	0,40072298	0	N.A.	0
LCN2	0,326410369	0,556533537	0	N.A.	0
UNC5B-AS1	0,32625761	0,334499941	0	N.A.	0
LTBP1	0,326193043	0,43238806	0	N.A.	0
STAT2	0,326156458	0,296576629	0	N.A.	0
EPB41L4B	0,326077044	0,434349019	0	N.A.	0
PLEKHB2	0,325988691	0,438091325	0	N.A.	0
CLCA4	0,325823454	0,283282281	0	N.A.	0
FAM25A	0,325643427	0,411561251	0	N.A.	0
SLC25A29	0,325347918	0,311982633	0	N.A.	0
SMAD2	0,325324419	0,294497266	0	N.A.	0
MICA	0,325250825	0,485113834	0	N.A.	0
TEP1	0,32522097	0,300160514	0	N.A.	0
MBD6	0,325192206	0,262650532	0	N.A.	0
FNIP1	0,325161817	0,490881442	0	N.A.	0
TCP11L2	0,32507166	0,278456243	0	N.A.	0
RAC1	0,324943801	0,390109318	0	N.A.	0
EVA1B	0,324942219	0,459981239	0	N.A.	0
MYO1D	0,324796376	0,503121782	0	N.A.	0
ACSS2	0,324732976	0,535036508	0	N.A.	0
PPP1R12C	0,324697865	0,258519177	0	N.A.	0
ATG13	0,324368904	0,228250583	0	N.A.	0
PTPN3	0,324089586	0,454820092	0	N.A.	0
MGAT3	0,324010356	0,417698874	0	N.A.	0
ACE2	0,323998851	0,025255828	0	N.A.	0
HGS	0,323981376	0,428940552	0	N.A.	0
CBX4	0,323228853	0,404533652	0	N.A.	0
SGPL1	0,323140309	0,602441832	0	N.A.	0
TNFSF10	0,323127808	0,306189798	0	N.A.	0

GFER	0,322905066	0,231733875	0	N.A.	0
CHMP1B	0,322810654	0,195332819	0	N.A.	0
SLURP1	0,322701906		0	N.A.	0
RP11-465N4.	0,322645867	0,283906071	0	N.A.	0
PIWIL4	0,322565168	0,21814187	0	N.A.	0
CYB5R3	0,322525607	0,502487805	0	N.A.	0
AC005550.3	0,322446788	0,248235821	0	N.A.	0
CH507-42P1	0,322334278	0,396160599	0	N.A.	0
SPRR2A	0,322210615	0,242306563	0	N.A.	0
CMPK2	0,322118896	0,328194118	0	N.A.	0
ABLIM1	0,321808867	0,261441145	0	N.A.	0
DAB2IP	0,321566013	0,276171609	0	N.A.	0
ITSN2	0,321526498	0,369341084	0	N.A.	0
MGRN1	0,321488374	0,586856582	0	N.A.	0
AC093609.1	0,321405457	0,0294406	0	N.A.	0
RP11-446N1	0,32130569	-0,256444694	0	N.A.	0
DIAPH2-AS1	0,321203394	0,326430694	0	N.A.	0
RBCK1	0,321061084	0,467758552	0	N.A.	0
B3GNT2	0,321037665	0,438222022	0	N.A.	0
RP11-50I19.2	0,32049429	0,253866768	0	N.A.	0
MSL1	0,320330785	0,2800661	0	N.A.	0
ZSWIM8	0,320194493	0,361321088	0	N.A.	0
CASP5	0,320191848	0,281992092	0	N.A.	0
ZZEF1	0,319975231	0,113299031	0	N.A.	0
HSH2D	0,319957717	0,213655323	0	N.A.	0
ANKRD12	0,31988124	0,295673729	0	N.A.	0
IL3RA	0,319793997	0,184730571	0	N.A.	0
HERPUD2	0,319629421	0,283852926	0	N.A.	0
MFSD5	0,319530312	0,289886634	0	N.A.	0
STAM2	0,319508385	-0,031765104	0	N.A.	0
AGPAT4	0,319471596	0,024105768	0	N.A.	0
FAM84A	0,319328846	0,191629794	0	N.A.	0
MPP5	0,319315352	0,437393152	0	N.A.	0

RP11-283G6.	0,319283856		0	N.A.	0
DUSP16	0,319262676	-0,008333691	0	N.A.	0
UACA	0,319175487	0,504089766	0	N.A.	0
EP300	0,318915425	0,497094121	0	N.A.	0
RP11-492E3.	0,318864501	0,170420163	0	N.A.	0
RGCC	0,318691119	0,017885332	0	N.A.	0
TMEM176A	0,318409927	0,355691459	0	N.A.	0
RP2	0,318398801	-0,139288471	0	N.A.	0
ELL	0,318377512	0,269897043	0	N.A.	0
TMEM2	0,318369124	0,47012629	0	N.A.	0
ITCH	0,318355207	0,293828596	0	N.A.	0
MARCH2	0,318312039	0,259456368	0	N.A.	0
SFXN3	0,318230301	0,683812123	0	N.A.	0
DOT1L	0,318144146	0,233587335	0	N.A.	0
KRT86	0,318138234	0,246460001	0	N.A.	0
NATD1	0,318039013	0,385407044	0	N.A.	0
KDM6A	0,317933532	0,219829633	0	N.A.	0
ARHGAP23	0,317902673	0,496096778	0	N.A.	0
BEST1	0,317840847	0,070766543	0	N.A.	0
NT5E	0,317761426	0,598441436	0	N.A.	0
RAB5A	0,317711898	0,173606108	0	N.A.	0
TM4SF1-AS1	0,317687729	0,107987945	0	N.A.	0
PNMA2	0,317660563	0,282091343	0	N.A.	0
ZSWIM6	0,317505199	0,314121408	0	N.A.	0
ZNF83	0,317459988	0,65424049	0	N.A.	0
EHD2	0,317180643	0,205392827	0	N.A.	0
CCDC64	0,316948733	0,234897454	0	N.A.	0
MXRA5	0,316804499	-0,04998447	0	N.A.	0
APP	0,316773265	0,523553369	0	N.A.	0
EFCAB14	0,3164789	0,516034431	0	N.A.	0
SERP1	0,316380847	0,030860032	0	N.A.	0
C3	0,316235877	-0,019952396	0	N.A.	0
STXBP2	0,315855432	0,441193901	0	N.A.	0

DDIT3	0,315759883	0,466326477	0	N.A.	0
RP5-1185I7.1	0,315486867	-0,02026259	0	N.A.	0
TFAP2A	0,315458788	0,37813432	0	N.A.	0
UBALD2	0,315283924	0,447347965	0	N.A.	0
ALOX5	0,315013076	0,5240575	0	N.A.	0
STX3	0,314934663	0,424819162	0	N.A.	0
HRCT1	0,31481213	0,340768871	0	N.A.	0
GS1-600G8.5	0,314708262	0,21873662	0	N.A.	0
NPSR1	0,314677736	0,434470902	0	N.A.	0
KLF16	0,314627342	0,215859172	0	N.A.	0
ABHD3	0,314542176	0,614185683	0	N.A.	0
MED13	0,314532357	0,477672903	0	N.A.	0
ZDHHC9	0,314222693	0,2593489	0	N.A.	0
APAF1	0,314022325	0,145245781	0	N.A.	0
GRHL3	0,314009108	0,237742598	0	N.A.	0
SLC7A11	0,313740937	0,461497753	0	N.A.	0
RNF149	0,313622437	0,659934443	0	N.A.	0
SLC26A3	0,313523298	0,315628145	0	N.A.	0
RARA	0,313327451	0,004447435	0	N.A.	0
SIPA1L3	0,313269285	0,290571304	0	N.A.	0
RP1-276N6.2	0,313248899	-0,102446992	0	N.A.	0
RP11-400N13	0,313090245	-0,173508713	0	N.A.	0
GM2A	0,312852199	0,200421445	0	N.A.	0
MAPKBP1	0,312848361	0,154067348	0	N.A.	0
GLG1	0,312500506	0,438646643	0	N.A.	0
STRN3	0,312446548	0,428879887	0	N.A.	0
SGK1	0,312102186	0,1563812	0	N.A.	0
FBXO34	0,31201339	0,42025477	0	N.A.	0
PCSK5	0,311963683	0,478520636	0	N.A.	0
TM9SF3	0,31185771	0,549095644	0	N.A.	0
C20orf195	0,311592637	-0,111584309	0	N.A.	0
EGFL7	0,31156116	-0,049794071	0	N.A.	0
SLC51B	0,311487597	0,244494418	0	N.A.	0

EPN2	0,311375811	0,121660893	0	N.A.	0
OPN3	0,311197936	0,237192689	0	N.A.	0
LGALS1	0,311145883	-0,268913985	0	N.A.	0
MARCH8	0,310947199	0,108535168	0	N.A.	0
MARCH3	0,310879798	0,398856979	0	N.A.	0
CDK19	0,310638275	0,18793621	0	N.A.	0
AREG	0,310580174	0,533149591	0	N.A.	0
ATP6V1G1	0,310491354	0,038166723	0	N.A.	0
NRIP3	0,310461637	0,272122483	0	N.A.	0
SCD	0,310355557	0,443841109	0	N.A.	0
FKBP1B	0,310354217	0,314176456	0	N.A.	0
TMTC2	0,310338864	0,266793142	0	N.A.	0
SRD5A3	0,310153035	0,543849483	0	N.A.	0
SDCBP	0,309994212	-0,052639716	0	N.A.	0
CFB	0,309720732	0,214677026	0	N.A.	0
RP11-10L12.4	0,309684137	-0,082348116	0	N.A.	0
TMPRSS11E	0,309609836	0,366444706	0	N.A.	0
OVOL1	0,309604598	0,246254997	0	N.A.	0
ZBTB38	0,309559107	0,392320585	0	N.A.	0
IGSF3	0,309501771	0,352745745	0	N.A.	0
SPIRE1	0,309406626	0,286466439	0	N.A.	0
AJUBA	0,309369522	0,414455627	0	N.A.	0
ITGA5	0,309340157	0,348476347	0	N.A.	0
RARRES3	0,309334606	0,416800036	0	N.A.	0
RNASET2	0,309099048	0,25093918	0	N.A.	0
SRC	0,309095582	0,232188034	0	N.A.	0
SPOPL	0,308978619	0,013786822	0	N.A.	0
VAMP4	0,308941144	0,297632573	0	N.A.	0
DUSP4	0,308916741	0,575613145	0	N.A.	0
LAMTOR3	0,308904858	0,195830632	0	N.A.	0
PPDPF	0,308855891	0,040487044	0	N.A.	0
SEC14L1	0,308828464	0,387632135	0	N.A.	0
SLCO1B3	0,30873518	0,221540698	0	N.A.	0

RAB8B	0,308633697	0,272658824	0	N.A.	0
KIAA1109	0,308287569	0,224464914	0	N.A.	0
FLCN	0,308216489	0,224510252	0	N.A.	0
ADAMTSL5	0,308122423	0,363235996	0	N.A.	0
CMB9-22P13	0,307997784	0,068560057	0	N.A.	0
SCAMP2	0,307970557	0,451224326	0	N.A.	0
RBFOX2	0,307800904	0,40610377	0	N.A.	0
TRIB3	0,307792713	0,580709377	0	N.A.	0
KATNBL1	0,307760421	0,231049319	0	N.A.	0
SLC7A5	0,307667418	0,267218572	0	N.A.	0
SH3KBP1	0,307513445	0,39254659	0	N.A.	0
TMSB10	0,307407865	-0,009856289	0	N.A.	0
TMEM167B	0,307405304	0,243181409	0	N.A.	0
USP43	0,307380635	0,313286521	0	N.A.	0
TNKS2	0,307351143	0,331756461	0	N.A.	0
MAP2K4	0,307202558	0,029585275	0	N.A.	0
ZCCHC6	0,307144401	0,378845472	0	N.A.	0
JOSD2	0,307143993	0,161844586	0	N.A.	0
IL17RE	0,307075235	0,608755747	0	N.A.	0
PITPNM3	0,307055639	0,287134847	0	N.A.	0
TRANK1	0,307055237	0,152703616	0	N.A.	0
CD24	0,306910912	0,061668488	0	N.A.	0
TRIM22	0,306801301	0,109333675	0	N.A.	0
PTPN22	0,306695405	0,167721575	0	N.A.	0
SREBF1	0,306416383	0,426208533	0	N.A.	0
PCED1B	0,306415662	0,441589152	0	N.A.	0
FAM83E	0,306214463	0,237548037	0	N.A.	0
MYO9B	0,30620893	0,363427921	0	N.A.	0
C16orf72	0,306137071	0,394669242	0	N.A.	0
TRIM33	0,306082783	0,43791206	0	N.A.	0
SLC1A1	0,305993344	0,634985669	0	N.A.	0
CTNNB1	0,305909572	0,234197308	0	N.A.	0
CEBPB	0,305834701	0,209273086	0	N.A.	0

SREK1IP1	0,305730571	0,217452271	0	N.A.	0
CIB1	0,30557232	0,484330293	0	N.A.	0
ZC3H11A	0,30531839	0,31995325	0	N.A.	0
PRKAG2	0,305255432	0,321762226	0	N.A.	0
PRELID2	0,305158398	0,205565133	0	N.A.	0
KCP	0,304857433	0,454200945	0	N.A.	0
SMIM1	0,304762843	0,08565733	0	N.A.	0
DAPK1	0,304691427	0,434760455	0	N.A.	0
RP11-670E13	0,304518451	0,312401666	0	N.A.	0
IL13RA2	0,304437453	0,184696571	0	N.A.	0
CSRNP1	0,304338854	0,001792055	0	N.A.	0
IQSEC2	0,304321041	-0,019249646	0	N.A.	0
FAM134A	0,304108084	0,359469629	0	N.A.	0
GNPTG	0,304107808	0,09896081	0	N.A.	0
CRK	0,303948942	0,221894088	0	N.A.	0
RNF144A	0,303932839	0,043827853	0	N.A.	0
TMEM133	0,303897998	0,232530872	0	N.A.	0
EDN1	0,303869535	0,602691396	0	N.A.	0
TTC17	0,30385419	0,406570972	0	N.A.	0
IL4R	0,303755497	0,310910392	0	N.A.	0
SNX25	0,303725934	-0,120490329	0	N.A.	0
PPP2R2A	0,303535807	0,470861573	0	N.A.	0
NAMPT	0,303502332	0,086805189	0	N.A.	0
TPPP3	0,303498829	0,172839768	0	N.A.	0
RP5-968D22.	0,303458928	0,181322382	0	N.A.	0
PPP1R2	0,303392863	0,075914217	0	N.A.	0
TBC1D8	0,303320028	0,388979704	0	N.A.	0
WNT11	0,303205956	0,519684735	0	N.A.	0
RP11-1398P2	0,303078446	0,365822743	0	N.A.	0
FXYD1	0,302982408	0,204910519	0	N.A.	0
DGCR2	0,302908896	0,678458899	0	N.A.	0
WWP2	0,302860847	0,337977127	0	N.A.	0
GCC2	0,302482344	0,564205664	0	N.A.	0

SLC7A7	0,302464684	0,426305058	0	N.A.	0
PIK3AP1	0,302400657	0,119931839	0	N.A.	0
ABCD1	0,302395294	0,189856984	0	N.A.	0
MIA	0,302159451	-0,26754741	0	N.A.	0
MARK3	0,301999015	0,453734837	0	N.A.	0
WDR26	0,301872245	0,255057336	0	N.A.	0
XKR9	0,30157778	0,237873761	0	N.A.	0
ALPI	0,301431187	0,347915297	0	N.A.	0
DEFB4A	0,301333516	0,306251413	0	N.A.	0
PDE8A	0,301256058	0,181276833	0	N.A.	0
RFNG	0,30123586	0,281329913	0	N.A.	0
AFTPH	0,301149192	0,295422553	0	N.A.	0
VTCN1	0,301006256	0,055322146	0	N.A.	0
LINC00704	0,300970685	0,274085433	0	N.A.	0
OGT	0,300926406	0,271122998	0	N.A.	0
HBP1	0,300805237	0,346502078	0	N.A.	0
NAPG	0,300681127	0,378816066	0	N.A.	0
VPS13D	0,300412253	0,394273918	0	N.A.	0
CAMSAP2	0,300398112	0,503978694	0	N.A.	0
SEPT10	0,300283565	0,477304244	0	N.A.	0
UBR2	0,300119986	0,256945832	0	N.A.	0
EXT1	0,299940544	0,273412297	0	N.A.	0
PHACTR2	0,299738264	0,39494145	0	N.A.	0
C6orf141	0,29964761	0,40355356	0	N.A.	0
MAP3K13	0,299602613	0,137454229	0	N.A.	0
HERC5	0,299451488	0,1086528	0	N.A.	0
NEDD4L	0,299413886	0,344265346	0	N.A.	0
PLCXD1	0,299243146	0,284218817	0	N.A.	0
AC118754.4	0,299003036	0,141078261	0	N.A.	0
ALPPL2	0,298960476	0,216166735	0	N.A.	0
CXCL17	0,298956341	-0,044502029	0	N.A.	0
ARID5A	0,298826007	-0,042927868	0	N.A.	0
HIP1R	0,298807611	0,634492201	0	N.A.	0

CYP2C18	0,298552711	-0,008842563	0	N.A.	0
MGAT2	0,298437126	0,441570076	0	N.A.	0
TSC22D1	0,298377587	0,257860827	0	N.A.	0
TUFT1	0,298134987	0,500444267	0	N.A.	0
TNFRSF1B	0,297881222	-0,06004691	0	N.A.	0
RPTN	0,297568817	0,014573621	0	N.A.	0
RALGAPB	0,297545165	0,295383117	0	N.A.	0
MICALCL	0,297542534	0,19987932	0	N.A.	0
TEC	0,297193791	-0,03612256	0	N.A.	0
YKT6	0,29710141	0,334911404	0	N.A.	0
FAM83A	0,297027805	0,110919615	1	cl1	0
GSK3A	0,296941826	0,250430907	0	N.A.	0
KCNS3	0,296759892	0,090151561	0	N.A.	0
CTD-2260A1	0,296534208	-0,001847417	0	N.A.	0
PYGB	0,296436865	0,455326157	0	N.A.	0
SH3TC1	0,296421205	0,377716281	0	N.A.	0
ARCN1	0,296363929	0,216862234	0	N.A.	0
LINC01088	0,296333556	0,075499548	0	N.A.	0
ADAM8	0,296321185	0,626456614	0	N.A.	0
VAMP3	0,296295133	0,148225778	0	N.A.	0
USP12	0,296055665	0,412170654	0	N.A.	0
PTPRU	0,296036178	0,073343004	0	N.A.	0
TAB3	0,295873349	0,204632021	0	N.A.	0
SLC35F6	0,295597678	0,453909763	0	N.A.	0
ERMP1	0,295501118	0,099275516	0	N.A.	0
DIO3OS	0,295455048	0,220079696	0	N.A.	0
FAM89B	0,295437612	0,280977664	0	N.A.	0
RIPK4	0,295384362	0,569656779	1	cl1	0
PIP5K1B	0,295376466	0,316609085	0	N.A.	0
ZBTB18	0,295025272	0,302565328	0	N.A.	0
NCOA3	0,29487539	0,294449072	0	N.A.	0
LPIN1	0,294811329	0,311788733	0	N.A.	0
SMIM14	0,294157808	0,639454783	0	N.A.	0

ZBTB4	0,294126801	0,070792501	0	N.A.	0
CDK18	0,294100962	0,328828482	0	N.A.	0
C10orf12	0,294026698	0,272307788	0	N.A.	0
PRDM2	0,294002087	0,090576384	0	N.A.	0
WFDC10B	0,293891831	0,155188783	0	N.A.	0
ST6GALNAC6	0,293684684	0,34902863	0	N.A.	0
SERPINB2	0,293478605	0,455033018	0	N.A.	0
MOB3B	0,293476316	0,255007285	0	N.A.	0
HTR1D	0,293421261	0,249719695	0	N.A.	0
CASP4	0,293298845	0,336349312	0	N.A.	0
SAP30BP	0,293143556	0,353341106	0	N.A.	0
TMPRSS11D	0,293140263	0,228223926	0	N.A.	0
ATF4	0,292842366	0,300529231	0	N.A.	0
GGT1	0,292692254	0,331269979	0	N.A.	0
MUC15	0,292529517	0,041286063	0	N.A.	0
LINC01468	0,292455537		0	N.A.	0
AC021218.2	0,292393875	-0,047399678	0	N.A.	0
KB-1615E4.2	0,292313857	-0,104212778	0	N.A.	0
JMJD1C	0,292245867	0,413711503	0	N.A.	0
CCDC6	0,292181073	0,210514231	0	N.A.	0
HNF4A	0,292106879	0,243906282	0	N.A.	0
FAM135A	0,292089973	0,429916001	0	N.A.	0
TRAF3	0,291986808	0,112365166	0	N.A.	0
DBNDD1	0,291880003	0,333645899	0	N.A.	0
ZMIZ1	0,291820294	0,442438933	0	N.A.	0
C6orf15	0,29138091	0,301632129	0	N.A.	0
BRWD3	0,291368553	0,220092757	0	N.A.	0
CTA-384D8.3	0,291363011	0,091562128	0	N.A.	0
RP11-803D5.	0,29122896	0,582474009	0	N.A.	0
AC144449.1	0,291205355	-0,14780323	0	N.A.	0
JAK2	0,291181161	0,190253899	0	N.A.	0
SEMA4G	0,291147511	0,388243285	0	N.A.	0
OGFR	0,291107636	0,447568047	0	N.A.	0

GNG5	0,291010897	-0,059232295	0	N.A.	0
IL1B	0,291005145	0,356522252	0	N.A.	0
SLC35E4	0,290928366	0,29426138	0	N.A.	0
C15orf62	0,290567632	0,457384613	0	N.A.	0
STEAP2	0,29050949	0,533076968	0	N.A.	0
YIPF1	0,290500037	0,269745988	0	N.A.	0
BDKRB1	0,290493219	0,068610545	0	N.A.	0
LITAF	0,290461091	-0,002577244	0	N.A.	0
ARHGAP35	0,290209139	0,275513127	0	N.A.	0
MB21D2	0,29014403	0,195846487	0	N.A.	0
NRIP1	0,290114817	0,041726707	0	N.A.	0
LINC01564	0,290095267	0,431758423	0	N.A.	0
MALT1	0,290073141	0,185910538	0	N.A.	0
RAB11FIP5	0,29002871	0,114173973	0	N.A.	0
GAK	0,28995011	0,341176979	0	N.A.	0
HSPB1	0,289935309	0,49489335	0	N.A.	0
ZNF554	0,289919248	-0,091066385	0	N.A.	0
ATP2A2	0,289815834	0,478427882	0	N.A.	0
ARF4	0,289731091	0,139312085	0	N.A.	0
TMBIM6	0,289680093	0,466836041	0	N.A.	0
C11orf85	0,289646737	0,192080097	0	N.A.	0
WNT2B	0,289585767	0,16828219	0	N.A.	0
NAB1	0,289570431	0,122527297	0	N.A.	0
KLK11	0,289449087	0,638973157	1	cl1	0
SYT7	0,289443467	0,311243901	0	N.A.	0
RP11-53M11	0,289324276	-0,133187711	0	N.A.	0
KAT6A	0,288909908	0,311436169	0	N.A.	0
CSNK1D	0,288709271	0,543620093	0	N.A.	0
FZD7	0,288623331	0,170983943	0	N.A.	0
SH3RF2	0,288551085	0,343317864	0	N.A.	0
RP11-702B10	0,288527063		0	N.A.	0
SUCO	0,288509781	0,525272747	0	N.A.	0
SYVN1	0,288503227	0,388087448	0	N.A.	0

PTK2	0,288292999	0,344182555	0	N.A.	0
MAN2A1	0,288197787	0,334272248	0	N.A.	0
C4BPA	0,288159713	-0,071647204	0	N.A.	0
IFIT1	0,288156474	0,364061671	0	N.A.	0
DUSP10	0,287975028	0,575413495	0	N.A.	0
AP1AR	0,287901628	0,398424802	0	N.A.	0
RTN2	0,287831557	0,103152739	0	N.A.	0
RP11-314N1:	0,287821338	0,332903244	0	N.A.	0
MICAL1	0,287821139	0,338373951	0	N.A.	0
ATP6V1A	0,287809072	0,180330684	0	N.A.	0
FAM219A	0,287708402	0,015452146	0	N.A.	0
ZNRF2	0,287680329	-0,170865083	0	N.A.	0
RP11-63P12.	0,287592988	0,223926891	0	N.A.	0
RP11-817J15	0,287551034	-0,29861886	0	N.A.	0
LINC00342	0,287483485	0,469545547	0	N.A.	0
BTBD7	0,287246665	0,276381833	0	N.A.	0
SP6	0,287201218	0,257484973	0	N.A.	0
NEU1	0,28715232	0,393451978	0	N.A.	0
RICTOR	0,287099142	0,456475946	0	N.A.	0
ENDOD1	0,287079684	0,65834214	0	N.A.	0
MAX	0,286944019	0,423546883	0	N.A.	0
TMEM184B	0,286757774	0,397477127	0	N.A.	0
MAP3K6	0,286739704	0,291226473	0	N.A.	0
RFFL	0,286678542	0,18250647	0	N.A.	0
FAM127A	0,28667677	0,128057111	0	N.A.	0
GLTPD2	0,286664351	0,238525813	0	N.A.	0
HBEGF	0,286595643	0,377658497	0	N.A.	0
PLAT	0,286574784	0,176210729	0	N.A.	0
FLNA	0,2863217	0,281339764	0	N.A.	0
ABL2	0,286308614	0,424169011	0	N.A.	0
VSIG1	0,286288912	-0,127286099	0	N.A.	0
CIDEB	0,286266417	0,306754612	0	N.A.	0
PIP5K1C	0,286060781	0,144106116	0	N.A.	0

SPSB3	0,285925821	0,503083335	0	N.A.	0
BCYRN1	0,285889503	0,161565266	0	N.A.	0
WFDC12	0,285789336	0,259588828	0	N.A.	0
LINC00857	0,28571368	0,08725317	0	N.A.	0
UNC5CL	0,285672837	0,291215674	0	N.A.	0
FBXL3	0,285655956	0,600952791	0	N.A.	0
KNDC1	0,285654644	0,147802838	0	N.A.	0
RP11-290L1.3	0,285545733	-0,228780697	0	N.A.	0
MMP10	0,285532773	0,477251101	0	N.A.	0
SCNN1A	0,285343433	0,574779104	1	cl1	0
ZMYM5	0,285159359	0,262441294	0	N.A.	0
RP11-493L12	0,285086978	-0,155855592	0	N.A.	0
USP6NL	0,285052724	0,28814275	0	N.A.	0
PLEKHA6	0,284909291	0,444508615	0	N.A.	0
TLR3	0,284770874	0,205634504	0	N.A.	0
GOLGB1	0,284590681	0,491958174	0	N.A.	0
FAM84B	0,284587264	0,50544707	0	N.A.	0
BAIAP2	0,284488795	0,580506814	1	cl1	0
FUT11	0,284392407	-0,037002281	0	N.A.	0
PPP1R18	0,284386551	0,108782299	0	N.A.	0
RIPK1	0,284028153	0,447105876	0	N.A.	0
DTX2	0,283802989	0,415859215	0	N.A.	0
KIAA0355	0,283642461	0,281585805	0	N.A.	0
UBAP1	0,283466633	0,075607771	0	N.A.	0
CPTP	0,283411258	-0,03126035	0	N.A.	0
ZDHHC2	0,28336636	0,147004635	0	N.A.	0
OGDH	0,283351641	0,494197704	0	N.A.	0
C7orf43	0,283255498	0,420787165	0	N.A.	0
MORC3	0,283126395	0,396222625	0	N.A.	0
MLPH	0,283124886	0,724688291	0	N.A.	0
MARK2	0,28308024	0,611271664	0	N.A.	0
FBXO33	0,282680897	-0,047642592	0	N.A.	0
ANKRD36C	0,282559723	0,602099678	0	N.A.	0

FLNC	0,282495838	0,494136717	0	N.A.	0
ORMDL2	0,282439271	0,574252506	0	N.A.	0
RTN4R	0,282362915	0,564394141	0	N.A.	0
PARP12	0,282295986	0,422260055	0	N.A.	0
LINC00675	0,282204105	0,230985471	0	N.A.	0
CTC-436P18.	0,282139174	0,484182371	0	N.A.	0
HDAC7	0,281820081	0,346244221	0	N.A.	0
PRSS23	0,281806849	0,424423189	0	N.A.	0
KIAA0930	0,281663097	0,147093514	0	N.A.	0
ACVR1	0,281653666	0,305694947	0	N.A.	0
CCNL2	0,281646859	0,400666395	0	N.A.	0
FAM65A	0,281498788	0,251616663	0	N.A.	0
PCSK6	0,280928095	0,144072082	0	N.A.	0
FAM222B	0,280872325	0,160822432	0	N.A.	0
MPRIP-AS1	0,280628639	0,233182934	0	N.A.	0
RNF38	0,280568897	0,164507006	0	N.A.	0
TMEM43	0,280461078	0,606507708	0	N.A.	0
CA3	0,28040229	0,375511628	0	N.A.	0
KDM5B	0,280372886	0,585450682	0	N.A.	0
C11orf80	0,280363123	0,541624354	0	N.A.	0
OSBPL8	0,28023986	0,0774138	0	N.A.	0
RNF207	0,280045264	0,30942885	0	N.A.	0
BARX2	0,27995322	0,164062845	0	N.A.	0
MAP3K1	0,279784711	0,163517959	0	N.A.	0
ZFYVE27	0,279605665	0,569428398	0	N.A.	0
FRMD5	0,279601085	0,515656895	0	N.A.	0
UBE2J1	0,279551695	0,181589307	0	N.A.	0
TNFAIP3	0,279206873	0,106933513	0	N.A.	0
SLC26A9	0,279072116	0,161678792	0	N.A.	0
DKK4	0,278918404	0,308787876	0	N.A.	0
KDM7A	0,278842655	0,267131934	0	N.A.	0
SLC22A23	0,278779735	0,054682073	0	N.A.	0
RP5-1086K13	0,278769735	-0,08206304	0	N.A.	0

TPK1	0,278462647	-0,000398284	0	N.A.	0
MOB2	0,278384905	0,295191796	0	N.A.	0
HABP4	0,278362818	0,138659461	0	N.A.	0
FAM155A	0,278337576	-0,060954454	1	none	0
NFKBIZ	0,278288703	0,379315598	0	N.A.	0
NCF2	0,278262504	0,519844446	0	N.A.	0
RTN4	0,278192195	0,483267871	0	N.A.	0
PI4K2A	0,278183696	0,007423908	0	N.A.	0
SERPINB4	0,278026351	0,112527022	0	N.A.	0
BTNL8	0,277997104	0,457545044	0	N.A.	0
NUP50-AS1	0,27772756	0,267185129	0	N.A.	0
DHDDS	0,27772229	0,1116221	0	N.A.	0
APOBEC3A	0,277565353	0,034718947	0	N.A.	0
HERPUD1	0,277383918	0,242722054	0	N.A.	0
APOBR	0,27727535	0,083652096	0	N.A.	0
USP32	0,277236072	0,206449771	0	N.A.	0
CCDC71L	0,277212691	-0,024913126	0	N.A.	0
TEF	0,277163564	0,249289352	0	N.A.	0
PLEKHF2	0,276891203	0,286118688	0	N.A.	0
AARS	0,276852982	0,294110051	0	N.A.	0
LARGE	0,276832478	0,237974705	0	N.A.	0
GGT6	0,276740296	0,059130035	0	N.A.	0
RAI2	0,276716799	0,294922349	0	N.A.	0
L1CAM	0,276657699	0,276026057	0	N.A.	0
IRF1	0,276585347	0,335881775	0	N.A.	0
RAB31	0,276575844	-0,11991011	0	N.A.	0
BLVRB	0,27653487	0,26042574	0	N.A.	0
FBRS	0,276488524	0,344520234	0	N.A.	0
CXorf38	0,276488053	0,000272578	0	N.A.	0
WNT7A	0,276351758	0,397296572	0	N.A.	0
IGFBP6	0,276223628	0,109372448	0	N.A.	0
SPATA13	0,276134905	0,156218145	0	N.A.	0
RP11-383J24	0,276081042	0,357206282	0	N.A.	0

PROSER2	0,276029044	0,354413819	0	N.A.	0
VGLL4	0,275851315	0,117211038	0	N.A.	0
COL9A2	0,275809995	0,187207457	0	N.A.	0
IFI44L	0,27553869	0,20813096	0	N.A.	0
RP11-873E20	0,275473124	-0,079960731	0	N.A.	0
GFPT1	0,275299701	0,361859051	0	N.A.	0
C11orf96	0,275275858	0,06843192	0	N.A.	0
B3GAT2	0,27527304	0,157884378	0	N.A.	0
EXOC1	0,275256294	0,484769628	0	N.A.	0
TRADD	0,275227947	0,585178575	0	N.A.	0
MROH6	0,275006238	0,321619797	1	cl1	0
FAM127B	0,274975253	0,174679097	0	N.A.	0
PARP9	0,274735455	0,521940264	0	N.A.	0
IL36G	0,274696676	0,265274454	0	N.A.	0
PEAR1	0,274669136	0,492835233	0	N.A.	0
SLC22A4	0,274566829	0,114524478	0	N.A.	0
ATXN2L	0,274511996	0,337331815	0	N.A.	0
FKBP9	0,274327756	0,347639369	0	N.A.	0
NPC2	0,274205132	0,19535715	0	N.A.	0
ACER3	0,274133657	0,093775366	0	N.A.	0
ASPG	0,27412696	0,129124777	0	N.A.	0
DKK1	0,274113396	0,375653348	0	N.A.	0
BOD1L1	0,274041518	0,245724484	0	N.A.	0
FOCAD	0,273880505	0,121592856	0	N.A.	0
ANKRD11	0,27385401	0,451288235	0	N.A.	0
SLC2A3	0,273825513	0,423898004	0	N.A.	0
FOXJ2	0,273730938	0,105489353	0	N.A.	0
PQLC2	0,273713739	-0,011590455	0	N.A.	0
AC004951.6	0,273627954	0,22211903	0	N.A.	0
ARHGEF4	0,273597077	0,355367088	0	N.A.	0
MKLN1	0,273474346	0,366543222	0	N.A.	0
CX3CL1	0,273430647	0,432081072	0	N.A.	0
CSF1	0,27329472	0,129089859	0	N.A.	0

ST8SIA6	0,273174579	0,288945228	0	N.A.	0
DUSP8	0,273170514	0,480848629	0	N.A.	0
FBN2	0,273155925	0,140003672	0	N.A.	0
UBE2D1	0,273095885	0,393271339	0	N.A.	0
FANK1	0,273083945	0,26927472	0	N.A.	0
ATG14	0,273021692	0,043564604	0	N.A.	0
ADAM12	0,272950188	-0,085550159	0	N.A.	0
FURIN	0,272778374	0,42127299	0	N.A.	0
PIGZ	0,272589721	0,216435415	0	N.A.	0
MGC50722	0,272205379	-0,294732299	0	N.A.	0
NHSL2	0,272175686	-0,139370012	0	N.A.	0
ZMYND15	0,27216159	0,134101882	0	N.A.	0
FCHO2	0,272066139	0,533101614	0	N.A.	0
ENKUR	0,272040342	0,354338834	0	N.A.	0
RP11-96H19.	0,271983814	-0,35784321	0	N.A.	0
SYT8	0,27192882	0,382456574	0	N.A.	0
SPG11	0,271873607	0,339984324	0	N.A.	0
SLC27A1	0,27182209	0,085312171	0	N.A.	0
MDK	0,271771326	0,633781093	0	N.A.	0
CTA-243E7.1	0,271692713	0,204991321	0	N.A.	0
RALBP1	0,271610376	0,355783412	0	N.A.	0
SMOX	0,271537161	0,261237641	0	N.A.	0
AC006273.5	0,271172658	0,413273476	0	N.A.	0
STXBP5	0,27095825	0,294749429	0	N.A.	0
ZFYVE28	0,270779872	0,175631543	0	N.A.	0
TRIM38	0,270732851	-0,075482031	0	N.A.	0
TBC1D20	0,27068803	0,399354272	0	N.A.	0
DGKE	0,270682897	0,285431665	0	N.A.	0
ATF6	0,270682641	0,120033981	0	N.A.	0
CDC42EP1	0,270661392	0,242341405	0	N.A.	0
CHD2	0,270644202	0,309992779	0	N.A.	0
TREML2	0,27050786	0,328826918	0	N.A.	0
LRRC75B	0,270467004	0,280857647	0	N.A.	0

UHMK1	0,270327449	0,452618611	0	N.A.	0
YWHAG	0,27019127	0,407326486	0	N.A.	0
ZNF846	0,270039878	-0,067153836	0	N.A.	0
C1orf226	0,270014984	0,24208776	0	N.A.	0
TRNP1	0,269899577	0,127508758	0	N.A.	0
TMEM131	0,269817798	0,123959751	0	N.A.	0
MSN	0,269802373	-0,128866486	0	N.A.	0
NOXA1	0,269751539	0,279647882	0	N.A.	0
FGFR2	0,269652064	0,384516558	0	N.A.	0
OSBPL10	0,269649558	0,20232705	0	N.A.	0
CDKN1C	0,269638008	0,629799621	0	N.A.	0
PNPLA6	0,26962998	0,464915195	0	N.A.	0
CREB3L2	0,26960469	0,322153606	0	N.A.	0
TMOD1	0,269281878	-0,171903282	0	N.A.	0
SPPL3	0,269142608	0,308363872	0	N.A.	0
PRNP	0,269118955	0,142940176	0	N.A.	0
LEPR	0,268950267	-0,115587741	0	N.A.	0
MGEA5	0,268819649	0,382983675	0	N.A.	0
DGAT1	0,268650831	0,49690751	0	N.A.	0
PPTC7	0,268587225	0,197146214	0	N.A.	0
SBF1	0,268546523	0,405216885	0	N.A.	0
ARFGEF1	0,268494122	0,340321574	0	N.A.	0
ARPC1B	0,268482374	0,165541803	0	N.A.	0
FLJ20021	0,268444388	0,0687964	0	N.A.	0
AP006285.2	0,268108627	0,170833145	0	N.A.	0
CD177	0,267989952	0,192054091	0	N.A.	0
PHACTR3	0,267877339	0,292044842	0	N.A.	0
RNF170	0,267847136	0,396863794	0	N.A.	0
TFEB	0,267777635	0,51529989	0	N.A.	0
ABR	0,267686926	0,378943593	0	N.A.	0
DIAPH2	0,267492985	0,109583381	0	N.A.	0
RP11-1348G1	0,267425467	0,007552628	0	N.A.	0
IL36RN	0,267308731		0	N.A.	0

SYT17	0,267249925	0,384915262	0	N.A.	0
RC3H1	0,267239984	0,29339941	0	N.A.	0
CSNK2A2	0,26708986	0,03237048	0	N.A.	0
SERPINB3	0,26691971	0,068229557	0	N.A.	0
RAB40B	0,266915066	0,247723886	0	N.A.	0
FUT6	0,26689919	0,269986709	0	N.A.	0
DUOXA1	0,266863141	0,251756977	0	N.A.	0
HIST1H2AD	0,26677441		0	N.A.	0
CRABP2	0,266732341	-0,054212486	0	N.A.	0
TRIO	0,266575494	0,211985112	0	N.A.	0
MAPK8IP1	0,26657189	0,18459148	0	N.A.	0
ZNF768	0,266500757	0,369668449	0	N.A.	0
PKP3	0,266294685	0,741235859	0	N.A.	0
SAPCD1-AS1	0,266267042	0,207317654	0	N.A.	0
DOCK1	0,266173423	0,383922686	0	N.A.	0
KPNA7	0,265965629	0,476246785	0	N.A.	0
NDUFA4L2	0,265877457	0,025611072	0	N.A.	0
RP11-449J21	0,265873511	0,045491937	0	N.A.	0
PDK4	0,265861572	-0,051733745	0	N.A.	0
AP1G1	0,265810237	0,430962192	0	N.A.	0
SEMA4D	0,265710213	0,079381958	0	N.A.	0
DTX4	0,265642774	0,416316142	0	N.A.	0
EDAR	0,265600106	0,135589674	0	N.A.	0
ZDHHC18	0,265578127	0,210970885	0	N.A.	0
RALGPS1	0,265547064	0,378544607	0	N.A.	0
CDV3	0,265509995	0,090180979	0	N.A.	0
SETD1B	0,265497204	0,072509219	0	N.A.	0
FNBP4	0,265384545	0,291059084	0	N.A.	0
TPTEP1	0,265184687	0,164126271	0	N.A.	0
TMEM37	0,2651568	0,49216098	0	N.A.	0
FICD	0,265138722	0,152629169	0	N.A.	0
SKIV2L	0,265115661	-0,175731197	0	N.A.	0
CEP350	0,265088706	0,431323136	0	N.A.	0

RAPGEF1	0,265048552	0,445693047	0	N.A.	0
NLRC5	0,265015128	0,216925628	0	N.A.	0
MON2	0,264996243	0,432749059	0	N.A.	0
CAPNS1	0,2649058	0,494801315	0	N.A.	0
GPSM1	0,264821251	0,326959004	0	N.A.	0
MST1	0,26478554	0,232247572	0	N.A.	0
CREB3L1	0,264563703	0,49137497	0	N.A.	0
INPP5K	0,264495971	-0,02351475	0	N.A.	0
TMC1	0,264329582		0	N.A.	0
FABP3	0,264179377	0,07483328	0	N.A.	0
ENTPD8	0,263981979	0,476928772	0	N.A.	0
EGF	0,263960317	0,035903874	0	N.A.	0
IFITM10	0,263935227	0,178578408	0	N.A.	0
TUBB6	0,263926957	0,271713308	0	N.A.	0
ARL14	0,263833485	0,5130588	0	N.A.	0
HIST1H2BG	0,263832336	0,164376079	0	N.A.	0
SULF2	0,263497369	0,372073641	0	N.A.	0
RRAS2	0,263455702	0,137365873	0	N.A.	0
NOS1AP	0,263381397	0,273880926	0	N.A.	0
DEDD2	0,263340493	0,149415708	0	N.A.	0
LAMP3	0,26285524	-0,103403305	0	N.A.	0
MAML2	0,26268484	0,292472585	0	N.A.	0
LDLRAP1	0,262649196	0,324730731	0	N.A.	0
CD63	0,262613725	0,533503476	0	N.A.	0
ANKRD13C	0,262577996	0,218232375	0	N.A.	0
FLOT1	0,262477242	0,123890011	0	N.A.	0
HIST2H2BE	0,262436173	0,288670576	1	cl1	0
PIK3IP1	0,26243424	0,394528554	0	N.A.	0
C8orf58	0,262347475	0,06135126	0	N.A.	0
CUEDC1	0,262130313	0,408817111	0	N.A.	0
MYO1B	0,262057308	0,066135306	0	N.A.	0
NFYA	0,262027635	-0,127082154	0	N.A.	0
SHC2	0,261993839	0,353961991	0	N.A.	0

VGLL1	0,261985683		1	cl1	0
TBL1X	0,26189896	0,065104856	0	N.A.	0
HMCN2	0,261778462	0,256730902	0	N.A.	0
ACTBL2	0,261709053	-0,226075085	0	N.A.	0
RAB3B	0,261585556	0,506821793	0	N.A.	0
CHPF2	0,261556487	0,39222557	0	N.A.	0
HSPA13	0,261466539	0,327022207	0	N.A.	0
IRS2	0,261418195	0,42053152	0	N.A.	0
RP11-104N1(	0,261153926	0,062125261	0	N.A.	0
ANKRD20A3	0,261081094	-0,037457803	0	N.A.	0
ARFGAP1	0,260959423	0,289912149	0	N.A.	0
TBC1D9B	0,260910513	0,361853074	0	N.A.	0
ARPP19	0,260876354	0,287189835	0	N.A.	0
PPARG	0,260847027	0,252504301	0	N.A.	0
ATP8A1	0,260842007	0,482513642	0	N.A.	0
KRT12	0,260745314	0,34927221	0	N.A.	0
SURF4	0,260700314	0,366787151	0	N.A.	0
KIF13A	0,260676223	0,066507303	0	N.A.	0
LOXL4	0,260623288	-0,020110186	0	N.A.	0
AMOT	0,260485128	0,345744055	0	N.A.	0
MAST3	0,260187334	0,015213145	0	N.A.	0
SYT12	0,260146319		0	N.A.	0
CSNK1A1	0,260080472	0,420727459	0	N.A.	0
TEAD1	0,26002196	0,226793092	0	N.A.	0
RP11-758N13	0,259877621	0,085086587	0	N.A.	0
PLEKHG2	0,259819843	0,010709798	0	N.A.	0
TGFBR2	0,259513689	0,367411793	0	N.A.	0
RAB3GAP1	0,259389082	0,299702653	0	N.A.	0
MCOLN1	0,259196686	0,265019959	0	N.A.	0
STRIP2	0,259157109	0,360901934	0	N.A.	0
CTD-2105E13	0,259129436	0,173440517	0	N.A.	0
CYP3A4	0,25892724	0,341472503	0	N.A.	0
CREBBP	0,258781042	0,342361855	0	N.A.	0

AP000679.2	0,258642104	-0,095659278	0	N.A.	0
FAM160A1	0,258503011	0,349554582	0	N.A.	0
DCUN1D3	0,258301857	0,219694535	0	N.A.	0
BCAN	0,258030562	0,240514801	0	N.A.	0
MT-ND4L	0,257916921	0,176785878	0	N.A.	0
KCTD9	0,257829093	0,099936407	0	N.A.	0
FER1L6	0,257820824	0,310543168	0	N.A.	0
MYZAP	0,25780232	0,361121247	0	N.A.	0
SHARPIN	0,257712478	0,079118733	0	N.A.	0
DISP1	0,257616776	-0,043139553	0	N.A.	0
KALRN	0,257556705	0,477160141	0	N.A.	0
CA2	0,257460552	0,217400694	0	N.A.	0
CMIP	0,257453063	0,455258038	0	N.A.	0
CGGBP1	0,257292809	-0,217938224	0	N.A.	0
TNRC18	0,257246759	0,313772487	0	N.A.	0
SYTL4	0,257124033	0,194841914	0	N.A.	0
CPM	0,2570604	0,349714787	0	N.A.	0
NMRK1	0,256987686	0,219192671	0	N.A.	0
TRPV6	0,256981415	0,1622822	0	N.A.	0
NR5A2	0,25689295	0,145039825	0	N.A.	0
MTRNR2L1	0,256769813	0,085571464	0	N.A.	0
DLEU7-AS1	0,256712504	0,045948568	0	N.A.	0
GPT	0,256647771	0,272956088	0	N.A.	0
NUMB	0,256522134	0,519026918	0	N.A.	0
PPFIBP1	0,256383007	0,385850068	0	N.A.	0
RRAD	0,256233998	0,423521914	0	N.A.	0
DLG1	0,256198712	0,612884929	0	N.A.	0
ITFG1	0,256162971	0,480276124	0	N.A.	0
PRRX2	0,256065015	0,210138707	0	N.A.	0
MON1B	0,256014047	0,223306177	0	N.A.	0
PRR13	0,255958367	0,073671475	0	N.A.	0
AFAP1L2	0,255782058	0,154427713	0	N.A.	0
UBTD2	0,255776657	0,286753723	0	N.A.	0

POU5F1	0,255603399	0,07153459	0	N.A.	0
GPRIN2	0,255602882	0,363410158	0	N.A.	0
ZNF587	0,255596617	0,409071338	0	N.A.	0
ACAP1	0,255561046	0,177512546	0	N.A.	0
BTN2A1	0,255550126	0,214121457	0	N.A.	0
ENTPD4	0,255350948	0,512195734	0	N.A.	0
ELK4	0,255347247	0,221048236	0	N.A.	0
SIRT2	0,255291386	0,396203071	0	N.A.	0
FAM91A1	0,254980733	0,178089369	0	N.A.	0
GALNT6	0,254963693	0,326347093	0	N.A.	0
SOS2	0,254906166	0,206206898	0	N.A.	0
PTP4A1	0,254717826	-0,045199029	0	N.A.	0
CTH	0,254676195	0,069811446	0	N.A.	0
CDC42SE2	0,254665728	0,037466918	0	N.A.	0
RP11-35P15.	0,25463493	0,136176334	0	N.A.	0
ARL6IP5	0,254601613	0,456919542	0	N.A.	0
NDP	0,254573401	0,376759479	0	N.A.	0
OSTF1	0,254563484	0,171380194	0	N.A.	0
MICALL1	0,254555553	-0,090065938	0	N.A.	0
PNRC1	0,254437969	-0,090399001	0	N.A.	0
GNB5	0,25439153	0,024164472	0	N.A.	0
LMNTD2	0,254240915	0,306060551	0	N.A.	0
CASZ1	0,254212446	0,393515428	0	N.A.	0
STAT3	0,254172542	0,18367225	0	N.A.	0
POGZ	0,254086103	0,082292776	0	N.A.	0
THSD7A	0,25392127	0,165316632	0	N.A.	0
CYP1A1	0,253850417		0	N.A.	0
CATSPER1	0,253832854	0,402125523	0	N.A.	0
CLDND1	0,25381639	0,342064452	0	N.A.	0
SMAD7	0,253454591	0,078482973	0	N.A.	0
RUNX1	0,25343466	0,072579134	0	N.A.	0
ADAMTS9	0,253284766	0,492987343	0	N.A.	0
TVP23B	0,253217586	0,056294329	0	N.A.	0

OSMR-AS1	0,253215716		0	N.A.	0
XCR1	0,253206587	-0,005945365	0	N.A.	0
HLA-DMA	0,253136844	-0,382304217	0	N.A.	0
PTPN21	0,253110549	0,194401947	0	N.A.	0
HRH1	0,253104538	0,582774322	0	N.A.	0
TPRN	0,253067424	0,316246508	0	N.A.	0
ANKRD37	0,253044652	0,336502373	0	N.A.	0
XK	0,252875226	0,180793939	0	N.A.	0
ADAMTSL4	0,252822263	-0,057622993	0	N.A.	0
NCCRP1	0,252668545	0,36551367	0	N.A.	0
GPR137B	0,252628262	0,513215654	0	N.A.	0
MGAT1	0,25258354	-0,098139582	0	N.A.	0
DCTN4	0,252483082	0,075163013	0	N.A.	0
LMO7DN	0,252441986	0,364156615	0	N.A.	0
NKAIN3	0,252404355	0,405150364	0	N.A.	0
IDO1	0,252329333	0,217949834	0	N.A.	0
AC115522.3	0,25204623		0	N.A.	0
LDHD	0,251942663	0,040534333	0	N.A.	0
RAP1GAP2	0,251851953	0,366150533	0	N.A.	0
RASSF3	0,251831541	0,052060858	0	N.A.	0
FBXW7	0,251668261	0,150992839	0	N.A.	0
IQCD	0,251651656	0,105507616	0	N.A.	0
SSH2	0,251565167	-0,040153018	0	N.A.	0
CARD11	0,251564954	0,295217215	0	N.A.	0
RP11-1399P1	0,251494531	0,280957632	0	N.A.	0
KIAA0226	0,251442743	0,315994361	0	N.A.	0
EAF1	0,25143258	-0,091477865	0	N.A.	0
GATAD1	0,251390042	0,209954886	0	N.A.	0
TPRXL	0,251384483	0,100841154	0	N.A.	0
PIAS1	0,251314229	0,500272087	0	N.A.	0
CASC19	0,251275409	-0,095622261	0	N.A.	0
CTC-444N24.	0,251255843	0,427091883	0	N.A.	0
SLC9A2	0,251224277	0,119757724	0	N.A.	0

ARRDC3	0,251121955	0,337691147	0	N.A.	0
SEC16A	0,251054012	0,386220307	0	N.A.	0
SNX18	0,25101189	-0,11778417	0	N.A.	0
HIF1A	0,250955257	0,14023406	0	N.A.	0
RP11-288L9.1	0,250859213	0,152838775	0	N.A.	0
EIF4G3	0,250829814	0,311722299	0	N.A.	0
GPBP1	0,250735274	0,12755131	0	N.A.	0
TNS1	0,250355651	-0,078054137	0	N.A.	0
PLD2	0,250248548	0,372485545	0	N.A.	0
FNDC3A	0,250207254	0,213650697	0	N.A.	0
RP11-156K13	0,250090079	0,110242104	0	N.A.	0
LINC01226	0,24995983		0	N.A.	0
TAF10	0,24988124	0,252003838	0	N.A.	0
WWTR1	0,249879552	0,285367327	0	N.A.	0
CLU	0,24980257	0,156857428	0	N.A.	0
GLTSCR1	0,24978939	0,232403587	0	N.A.	0
BEAN1	0,249625815	0,543980386	0	N.A.	0
ZNRF1	0,249582146	-0,045233386	0	N.A.	0
DIRC2	0,249545257	0,285755165	0	N.A.	0
DUSP14	0,249510576	0,201917919	0	N.A.	0
UTP14C	0,249456886	0,288856163	0	N.A.	0
CYLD	0,249372102	0,260173064	0	N.A.	0
ABHD4	0,249370032	0,228161495	0	N.A.	0
RP11-267C16	0,24935341	0,306911103	0	N.A.	0
USP38	0,249338323	0,0467155	0	N.A.	0
TFCP2L1	0,249337194	-0,132784458	0	N.A.	0
ZNF160	0,249282434	0,32238561	0	N.A.	0
SUV420H1	0,24926965	0,328185573	0	N.A.	0
CDH3	0,249214371	0,550531908	0	N.A.	0
POU2F2	0,249193205	-0,114375993	0	N.A.	0
GEM	0,249142627	0,333352513	0	N.A.	0
PARP6	0,249116377	0,132440045	0	N.A.	0
PANK3	0,248939096	0,575142735	0	N.A.	0

LGALS9C	0,248920913	0,195581481	0	N.A.	0
BAZ2A	0,248895654	0,532437652	0	N.A.	0
PID1	0,248737903	0,363246944	0	N.A.	0
MBNL1	0,248654306	-0,169595424	0	N.A.	0
OTUD1	0,248629799	0,032094712	0	N.A.	0
PPP1R21	0,248517331	0,514596573	0	N.A.	0
KIF1B	0,248458575	0,222634956	0	N.A.	0
SLC16A5	0,248326495	0,546255915	0	N.A.	0
AC240274.1	0,248304357	-0,089007314	0	N.A.	0
DLG5	0,248066673	0,362329063	0	N.A.	0
CXCL8	0,248052178	0,041565847	0	N.A.	0
MEP1A	0,248000857	0,108468396	0	N.A.	0
CTSA	0,247976911	0,442853414	0	N.A.	0
EFR3A	0,24796825	-0,216633732	0	N.A.	0
PPP3CA	0,247896229	0,471589636	0	N.A.	0
SLC30A1	0,247854664	0,498775429	0	N.A.	0
ALDOC	0,247795302	0,516828988	0	N.A.	0
CCNK	0,24761648	0,090482946	0	N.A.	0
VDAC2	0,247571928	0,272984405	0	N.A.	0
CALCOCO2	0,247523559	0,194391325	0	N.A.	0
SUV420H2	0,247509125	0,312368205	0	N.A.	0
GOLGA7B	0,247496341	0,351929068	0	N.A.	0
ELFN2	0,247492095	-0,029356882	0	N.A.	0
CTA-363E6.2	0,247392196	0,116116359	0	N.A.	0
CD276	0,247292378	0,362179568	0	N.A.	0
AC007246.3	0,247287441	0,367142684	0	N.A.	0
ZDHHC5	0,247286595	0,291724181	0	N.A.	0
PRKCI	0,247269296	0,211933031	0	N.A.	0
DGKQ	0,24722312	0,440050125	0	N.A.	0
MAN2B2	0,247173848	0,138910937	0	N.A.	0
PDLIM1	0,247138727	0,13214068	0	N.A.	0
TMEM57	0,247121937	0,487660205	0	N.A.	0
USP9X	0,247052732	0,449280939	0	N.A.	0

SMCO2	0,246912562	0,097610043	0	N.A.	0
LSMEM2	0,246892862		0	N.A.	0
FEM1B	0,246878702	-0,056374094	0	N.A.	0
SZT2	0,246867898	0,260560469	0	N.A.	0
LUCAT1	0,246854624	0,228053787	0	N.A.	0
PGPEP1	0,246823305	0,288478714	0	N.A.	0
ADD1	0,24664652	0,540135175	0	N.A.	0
DYRK1A	0,246640116	0,219402584	0	N.A.	0
NCK2	0,246353949	0,229621367	0	N.A.	0
TULP4	0,246280358	0,404471593	0	N.A.	0
GBA	0,246184994	0,224274491	0	N.A.	0
ACSL5	0,246182099	0,398827966	0	N.A.	0
STAT5A	0,246119301	0,174902496	0	N.A.	0
EIF4A2	0,246020837	0,2758582	0	N.A.	0
CHMP5	0,245879966	0,253683001	0	N.A.	0
DDX3X	0,245838035	0,418532693	0	N.A.	0
ZNF274	0,245812446	0,140528122	0	N.A.	0
TMEM50B	0,245244572	0,477338218	0	N.A.	0
DENND6B	0,24513408	0,390578124	0	N.A.	0
SLC7A1	0,245044166	0,300572719	0	N.A.	0
GPX4	0,244952953	0,18182995	0	N.A.	0
PPP6R1	0,244922306	0,442493319	0	N.A.	0
YIPF2	0,244903408	0,333087529	0	N.A.	0
EGFR-AS1	0,244793232		0	N.A.	0
VMP1	0,244759848	0,539431743	0	N.A.	0
PRRG4	0,244641205	0,655374634	0	N.A.	0
PIGA	0,244583874	0,07723843	0	N.A.	0
TSC22D3	0,244439502	-0,228406217	0	N.A.	0
INPP5A	0,244429581	0,134122557	0	N.A.	0
ZNF808	0,24437629	0,313870315	0	N.A.	0
RBPMS	0,244328208	0,361963569	0	N.A.	0
RP6-99M1.3	0,244314366	0,162933013	0	N.A.	0
RP11-245G1[illegible]	0,244222841	0,061453543	0	N.A.	0

PLEKHM2	0,244193269	0,10120128	0	N.A.	0
KRT23	0,244133658	0,378158204	0	N.A.	0
KBTBD2	0,244122492	0,083418891	0	N.A.	0
CTB-191K22.	0,2440065	0,389529157	0	N.A.	0
XYLT1	0,243975997	0,406644971	0	N.A.	0
PRSS36	0,243904085	0,101879493	0	N.A.	0
CLDN18	0,243848969	0,323606682	0	N.A.	0
B3GNT7	0,243739068	0,640502485	0	N.A.	0
UNC93B1	0,243699455	0,765831854	0	N.A.	0
RP3-446N13.	0,243633864	0,254531286	0	N.A.	0
RP11-757F18	0,24357369	0,337931685	0	N.A.	0
NIPA1	0,243547035	0,442797007	0	N.A.	0
S100A13	0,243542742	0,313989123	0	N.A.	0
DBP	0,24350292	0,289116405	0	N.A.	0
ABCA5	0,243470575	0,269142412	0	N.A.	0
BCAM	0,243403218	0,204677464	0	N.A.	0
MAMDC4	0,243377631	0,137385742	0	N.A.	0
MAP3K10	0,243308121	0,127998992	0	N.A.	0
PTPRB	0,243198003	0,446886591	0	N.A.	0
PER1	0,243013871	0,365973567	0	N.A.	0
RP11-20I23.8	0,242869453	0,132884882	0	N.A.	0
DLG3	0,242821746	0,399175719	0	N.A.	0
ZNF462	0,242803301	0,023112846	0	N.A.	0
ADAM28	0,242797546	0,329749141	0	N.A.	0
SLC15A3	0,242726065	0,184523796	0	N.A.	0
ZNF264	0,242568193	0,177078817	0	N.A.	0
FBXO46	0,242412885	0,004865038	0	N.A.	0
ARFGEF2	0,242345345	0,125589204	0	N.A.	0
KRT14	0,242237984	0,066390249	0	N.A.	0
GOLPH3	0,242207138	0,145593898	0	N.A.	0
DAAM1	0,242206625	0,562909828	0	N.A.	0
TTYH2	0,24214693	0,267107503	0	N.A.	0
KAT2B	0,242062022	-0,025303201	0	N.A.	0

DAB2	0,242022681	0,126672128	0	N.A.	0
FO538757.2	0,241938561	0,36343757	0	N.A.	0
CDSN	0,241935238	0,368797137	0	N.A.	0
SNX13	0,241903619	0,078359163	0	N.A.	0
SLC51A	0,241841483	0,450046423	0	N.A.	0
TIE1	0,241727796	-0,232642349	0	N.A.	0
C17orf62	0,241688465	0,217826937	0	N.A.	0
TRIB2	0,241652772	0,265707996	0	N.A.	0
PLEKHA8	0,241632069	-0,040476573	0	N.A.	0
KDM5A	0,241459785	0,300537895	0	N.A.	0
FXYD4	0,241354317	0,190993936	1	cls3	0
ZDHHC14	0,241323352	0,356245645	0	N.A.	0
XXYLT1-AS2	0,241291896	0,280214022	0	N.A.	0
TPRG1	0,241244799	0,230724945	0	N.A.	0
ATXN7L3	0,241210807	0,3641474	0	N.A.	0
GRIPAP1	0,241207372	0,314001714	0	N.A.	0
LMBR1L	0,241142806	0,278752158	0	N.A.	0
FNBP1L	0,241126046	0,49224565	0	N.A.	0
MED16	0,241091964	0,266300623	0	N.A.	0
PIK3C2A	0,240963258	0,313648671	0	N.A.	0
TMEM119	0,240932109	-0,101123515	0	N.A.	0
ZNF677	0,240919924	-0,053190423	0	N.A.	0
PSENEN	0,240824	0,116763202	0	N.A.	0
AUTS2	0,240761307	0,320029895	0	N.A.	0
EIF1	0,240688695	0,051760021	0	N.A.	0
REL	0,240640199	0,477656395	0	N.A.	0
IL10RB-AS1	0,240346037	0,285290867	0	N.A.	0
CST3	0,240235699	0,336831224	0	N.A.	0
MFSD12	0,240234306	0,232657348	0	N.A.	0
CA1	0,240164942	0,157282374	0	N.A.	0
HERC6	0,240137332	0,365025974	0	N.A.	0
RAPGEF3	0,240107479	0,089801608	0	N.A.	0
ORAI3	0,240030718	0,247575694	0	N.A.	0

GNAI1	0,239978315	-0,027178627	0	N.A.	0
MYPN	0,239922773		0	N.A.	0
RNF222	0,239754036		0	N.A.	0
AC011718.2	0,239560018		0	N.A.	0
HHLA3	0,239501393	-0,181441874	0	N.A.	0
FOXD1	0,23947893	0,086338425	0	N.A.	0
PAFAH2	0,239159935	0,359182799	0	N.A.	0
ZNF592	0,239010342	0,16603484	0	N.A.	0
GNAI2	0,238965397	0,314509441	0	N.A.	0
TXK	0,238825955	-0,006610798	0	N.A.	0
YTHDF3	0,238811716	0,439850879	0	N.A.	0
CALU	0,238798502	0,341892088	0	N.A.	0
MEF2A	0,238796928	0,325428949	0	N.A.	0
ZNF513	0,238732741	0,291348276	0	N.A.	0
LINC01588	0,238687326	0,17039543	0	N.A.	0
SETX	0,238652296	0,416835036	0	N.A.	0
HECTD4	0,238601708	0,175730693	0	N.A.	0
KCTD7	0,238375129	-0,039038505	0	N.A.	0
SLC8B1	0,23834438	0,249445269	0	N.A.	0
GRIP1	0,238341301	-0,205931065	0	N.A.	0
C2orf54	0,238172277	0,451173563	0	N.A.	0
GABARAP	0,238068298	-0,194901806	0	N.A.	0
PLEKHG3	0,237985322	0,458199272	0	N.A.	0
ZNF543	0,237781854	0,112907887	0	N.A.	0
MEX3C	0,237688262	0,076820616	0	N.A.	0
CYP2C9	0,23766171	0,298616054	0	N.A.	0
WNK1	0,237636887	0,171606582	0	N.A.	0
TRIM56	0,237454485	0,518917574	0	N.A.	0
NRIR	0,237248944	0,103009596	0	N.A.	0
NCK1	0,237204987	-0,233578994	0	N.A.	0
ABHD17A	0,23710265	0,387339631	0	N.A.	0
GPX3	0,237091477	0,265073655	0	N.A.	0
IL1A	0,236748808		0	N.A.	0

RP11-532F12	0,236714766	0,422629449	0	N.A.	0
HIST1H2AE	0,236675302	0,113553563	0	N.A.	0
AFAP1	0,236467932	0,274170678	0	N.A.	0
DYNLRB1	0,23644162	0,275048933	0	N.A.	0
HMGCS1	0,236343408	0,245898499	0	N.A.	0
NRAS	0,236193506	0,005748064	0	N.A.	0
CYP4F35P	0,236129096	0,172809585	0	N.A.	0
TMEM140	0,236102544	0,345599703	0	N.A.	0
ARNTL2	0,235887034	0,413428487	0	N.A.	0
MCFD2	0,235838146	0,562250463	0	N.A.	0
AGAP1	0,235781178	0,261546493	0	N.A.	0
TRIM40	0,235721778	0,258997626	0	N.A.	0
ARHGAP30	0,235680584	0,094947613	0	N.A.	0
CPEB3	0,23557272	0,218890215	0	N.A.	0
TET2	0,235563236	0,280847337	0	N.A.	0
MAOB	0,235546008	0,491561227	0	N.A.	0
CAMSAP3	0,235510327	0,118213607	0	N.A.	0
WDTC1	0,235476195	0,215578712	0	N.A.	0
PLD3	0,235396737	0,10304854	0	N.A.	0
ANO9	0,235353848	0,266250696	0	N.A.	0
WDR37	0,235349484	-0,007626886	0	N.A.	0
PLXNB1	0,235281133	0,358654567	0	N.A.	0
DEGS1	0,235075811	0,318926015	0	N.A.	0
SSBP3	0,235061533	0,163565365	0	N.A.	0
RP11-798K3.	0,235058082	0,118050898	0	N.A.	0
MXRA8	0,234899676	0,094518187	0	N.A.	0
NEURL1B	0,234757685	0,392365893	0	N.A.	0
OSBP	0,23468594	0,409727195	0	N.A.	0
ZFAND2B	0,234626512	0,528879767	0	N.A.	0
TMUB2	0,234617767	0,392094448	0	N.A.	0
TCN1	0,234584318	0,171940035	0	N.A.	0
FAM73A	0,234472459	0,445219368	0	N.A.	0
EVPL	0,23440583	0,552264513	0	N.A.	0

ARHGEF3	0,234346792	0,367108541	0	N.A.	0
DIP2B	0,234311937	0,527732537	0	N.A.	0
MYOM3	0,234292278	-0,071675237	0	N.A.	0
DUSP18	0,234260727	0,196728112	0	N.A.	0
WNT7B	0,234176557	0,124509574	0	N.A.	0
AP1S3	0,234153637	0,128703444	0	N.A.	0
ZDHHC3	0,234108409	0,318366889	0	N.A.	0
EVI5L	0,233941106	0,458093552	0	N.A.	0
UBD	0,233895449	-0,003209073	0	N.A.	0
MAGI1	0,23385088	0,202161069	0	N.A.	0
CNTFR	0,233843335	0,114657619	0	N.A.	0
CTSW	0,233828881	-0,103688821	0	N.A.	0
RNF185	0,233810209	0,049602317	0	N.A.	0
LINC00113	0,233741185	0,443246504	0	N.A.	0
FLJ44511	0,23370222	0,209066946	0	N.A.	0
SLC4A11	0,233666263	0,507368196	0	N.A.	0
AGPAT3	0,233496532	0,263167199	0	N.A.	0
SYNJ2	0,233470272	0,528116429	0	N.A.	0
MAP1LC3B2	0,233439613	0,051522901	0	N.A.	0
RHBDF2	0,233377858	0,337210369	0	N.A.	0
CYB5R1	0,233236375	0,413690345	0	N.A.	0
TMEM44	0,233122446	0,431595226	0	N.A.	0
LRCH3	0,232981268	0,215992556	0	N.A.	0
DENND4C	0,232875719	0,341887607	0	N.A.	0
CCNYL1	0,232716071	0,477284152	0	N.A.	0
RP11-6F2.5	0,232620305	0,005304834	0	N.A.	0
PLA2G4C	0,232578188	0,089468449	0	N.A.	0
ZFYVE1	0,232554327	0,040186994	0	N.A.	0
KIRREL	0,232496981	0,278410624	0	N.A.	0
PITPNA	0,232465427	0,231304754	0	N.A.	0
ANO1	0,232133025	0,335438701	0	N.A.	0
AC099552.4	0,232105556	-0,079831104	0	N.A.	0
RP11-462G2.	0,232017351	0,185612752	0	N.A.	0

CCNT2	0,231984556	0,166173019	0	N.A.	0
EIF4EBP2	0,231920935	0,169562013	0	N.A.	0
FYCO1	0,231881537	0,200900132	0	N.A.	0
NET1	0,231716828	0,586748513	0	N.A.	0
RAB24	0,231693017	0,092419201	0	N.A.	0
DYNC1H1	0,231642444	0,575176798	0	N.A.	0
SLC15A4	0,231384511	0,375004099	0	N.A.	0
PGM2L1	0,231353877	0,411693544	0	N.A.	0
GPR153	0,23131786	0,412763764	0	N.A.	0
APBA3	0,231310275	0,115658947	0	N.A.	0
GATAD2A	0,231294897	0,277820461	0	N.A.	0
HYOU1	0,231200309	0,35735999	0	N.A.	0
TBC1D22B	0,231125493	0,122945732	0	N.A.	0
RUFY3	0,231097104	0,239853347	0	N.A.	0
CTA-243E7.4	0,231095104	-0,031212988	0	N.A.	0
NAB2	0,230818494	0,358059651	0	N.A.	0
AC005262.2	0,230796556	0,234541956	0	N.A.	0
PRMT2	0,23078051	0,041922928	0	N.A.	0
ADCY4	0,230736482	0,291012471	0	N.A.	0
SELK	0,230672787	0,260801934	0	N.A.	0
TMEM40	0,230657486	0,013352182	0	N.A.	0
TMEM27	0,23051526	0,148395918	0	N.A.	0
PPP2R2D	0,230380822	0,243494551	0	N.A.	0
ARID1B	0,230242758	0,140447269	0	N.A.	0
KRT6B	0,230216454	0,287693685	1	cls3	0
GRINA	0,230006191	0,257078043	0	N.A.	0
SGPP1	0,229987778	0,088038413	0	N.A.	0
LRRC3	0,229941183	0,264578202	0	N.A.	0
FRMD8	0,229896235	0,346004817	0	N.A.	0
HIST1H4H	0,229859933	0,252566811	0	N.A.	0
RP11-417L19	0,229778349	-0,111039621	0	N.A.	0
AKTIP	0,229685358	0,254797531	0	N.A.	0
MAP2K3	0,229660445	0,188607403	0	N.A.	0

YIPF6	0,229646146	0,303098447	0	N.A.	0
SLC38A7	0,229558328	0,189856143	0	N.A.	0
REC8	0,229440996	0,174542725	0	N.A.	0
SULT1A2	0,229308626	0,039508385	0	N.A.	0
NPTX2	0,229207878		0	N.A.	0
TMEM56	0,22912982	0,221126929	0	N.A.	0
CTD-3138B18	0,22912341	0,209186371	0	N.A.	0
ELL2	0,229118621	0,472159226	0	N.A.	0
PDE4A	0,229114817	0,046627667	0	N.A.	0
SLFN5	0,229095921	0,416940366	0	N.A.	0
C1QTNF6	0,229043455	-0,149246611	0	N.A.	0
RASA3	0,229040355	0,403606066	0	N.A.	0
GCC1	0,229021858	0,016441892	0	N.A.	0
EXD3	0,22899251	0,460274911	0	N.A.	0
NOTCH2	0,22888027	0,476931819	0	N.A.	0
PEA15	0,228830476	0,050290695	0	N.A.	0
OR2A1-AS1	0,228652386	0,332764823	0	N.A.	0
PSTPIP1	0,228600038	0,078821751	0	N.A.	0
MOV10	0,228380384	0,312605364	0	N.A.	0
RP11-10C24.	0,228351785	-0,163736077	0	N.A.	0
KDELR3	0,228267058	0,404147656	0	N.A.	0
PCMTD1	0,228264355	0,035168539	0	N.A.	0
DNAJC13	0,228134372	0,260907692	0	N.A.	0
STRN4	0,228112248	0,249714904	0	N.A.	0
RORA	0,227845293	0,085926849	0	N.A.	0
LRP11	0,227820277	0,456689107	0	N.A.	0
CTC-573N18.	0,227703396	0,109782588	0	N.A.	0
MMEL1	0,227560277	0,450346124	0	N.A.	0
CYR61	0,22754674	0,091331533	0	N.A.	0
GGTLC2	0,227523653	-0,079787206	0	N.A.	0
ROCK1	0,22743423	0,390484512	0	N.A.	0
IFI27L2	0,22742339	-0,192267381	0	N.A.	0
PHC2	0,227404213	0,417482235	0	N.A.	0

PRKAA1	0,227387843	0,321922312	0	N.A.	0
DOCK9	0,227339513	0,257131059	0	N.A.	0
RNASE1	0,227273004	-0,063213226	0	N.A.	0
ANKRD29	0,227244358	0,024915818	0	N.A.	0
SERAC1	0,22724431	0,153024232	0	N.A.	0
SEC24A	0,227209307	0,363997449	0	N.A.	0
ZNF841	0,227205915	-0,001292181	0	N.A.	0
SERTAD1	0,227176983	0,301629671	0	N.A.	0
CLN8	0,227085459	0,434743679	0	N.A.	0
ANKRD50	0,226975196	0,429682685	0	N.A.	0
CORO1C	0,226782202	0,17598054	0	N.A.	0
IQGAP2	0,22674945	0,232259608	0	N.A.	0
FAM129A	0,226715056	0,077460263	0	N.A.	0
ZNF395	0,22670004	0,209834145	0	N.A.	0
NCOA1	0,22669405	0,403307738	0	N.A.	0
ZNF432	0,226666902	0,213565082	0	N.A.	0
RORC	0,226312464	-0,217909036	0	N.A.	0
BCOR	0,226285151	0,001741317	0	N.A.	0
ZFHX3	0,226281514	0,343145371	0	N.A.	0
ZMYND8	0,226268878	0,56509965	0	N.A.	0
FAM214A	0,226167094	0,422797969	0	N.A.	0
ATG12	0,226026085	0,192086664	0	N.A.	0
PRDM8	0,225907227	0,264312796	0	N.A.	0
MFSD11	0,225863857	0,279924643	0	N.A.	0
NDFIP1	0,225798488	0,441876942	0	N.A.	0
TTC22	0,22568613	0,685348138	0	N.A.	0
CACNB4	0,225586996	0,379112578	0	N.A.	0
RERE	0,225548022	0,236200676	0	N.A.	0
LMO2	0,225458424		0	N.A.	0
ACER2	0,225278914	0,102428975	0	N.A.	0
FAM131A	0,225196422	0,344701719	0	N.A.	0
SLC39A13	0,225023243	0,512478788	0	N.A.	0
LA16c-380H5	0,2249062	0,212701586	0	N.A.	0

FAM45A	0,224889914	0,069215118	0	N.A.	0
RP11-525A16	0,224823045	0,122705054	0	N.A.	0
KCNK15	0,224742948	-0,095646648	0	N.A.	0
TMEM165	0,224698774	0,394602276	0	N.A.	0
EIF2AK3	0,224589412	0,338947099	0	N.A.	0
IL15	0,224525648	0,024601135	0	N.A.	0
RP13-516M1	0,224506348	0,30463566	0	N.A.	0
CELA3B	0,224340151	0,275425336	0	N.A.	0
ALG11	0,224238156	0,049011608	0	N.A.	0
FBXO32	0,224153221	0,313394527	0	N.A.	0
C8G	0,224120504	0,318591498	0	N.A.	0
ENPP1	0,224115904	0,125494737	0	N.A.	0
LINC00482	0,224061092	0,411155192	0	N.A.	0
PPP1R14C	0,223969267	0,204354415	0	N.A.	0
GTF2IRD1	0,223891788	0,426287041	0	N.A.	0
AMOTL1	0,223866774	-0,294683143	0	N.A.	0
VWF	0,223385205	-0,064953835	0	N.A.	0
RND1	0,223375712	0,291814426	0	N.A.	0
TSKU	0,223298387	0,374840968	0	N.A.	0
ATP6V0A1	0,223287571	0,494908793	0	N.A.	0
SAT2	0,223273454	0,267707022	0	N.A.	0
GPR107	0,22324671	0,214224354	0	N.A.	0
RP11-235E17	0,223208711	0,098697749	0	N.A.	0
SMURF2	0,223176346	0,168541077	0	N.A.	0
SCAMP1	0,223149534	0,18010617	0	N.A.	0
CCNI	0,223076231	-0,130612185	0	N.A.	0
MIRLET7BHG	0,223040485	0,158948483	0	N.A.	0
CBLC	0,222740505	0,413438984	0	N.A.	0
NRBP1	0,222733056	0,552223626	0	N.A.	0
REXO1	0,222622228	0,168057635	0	N.A.	0
RP11-79H23.	0,222357717	0,290707829	0	N.A.	0
SLCO2A1	0,222317009	0,366468241	0	N.A.	0
EYS	0,222237414	-0,179185941	0	N.A.	0

SLC12A6	0,222209682	0,296996345	0	N.A.	0
RAB6B	0,222208428	0,137576874	0	N.A.	0
CDYL2	0,222190956	-0,030507184	0	N.A.	0
IFIT3	0,222187845	0,44491209	0	N.A.	0
JHDM1D-AS1	0,222185843	-0,048139811	0	N.A.	0
GCH1	0,222084161	0,24168179	0	N.A.	0
IZUMO4	0,221978759	0,024092852	0	N.A.	0
GBA2	0,221852318	0,409350189	0	N.A.	0
IGHV3-66	0,221826963		0	N.A.	0
TRGV5	0,221800956		0	N.A.	0
ATXN1L	0,221786868	0,310360994	0	N.A.	0
TMEM217	0,221782286	0,026127571	0	N.A.	0
XPR1	0,221778806	0,047182067	0	N.A.	0
CIC	0,221685337	0,334733639	0	N.A.	0
NUP50	0,221677871	0,247669392	0	N.A.	0
TRIB1	0,221601225	0,382074248	0	N.A.	0
SLC25A37	0,221579852	0,325981366	0	N.A.	0
SNTB2	0,221531786	0,017059995	0	N.A.	0
CXCL1	0,221420103	0,486064008	0	N.A.	0
TSPO2	0,221330054	0,091017076	0	N.A.	0
LA16c-380H5	0,221303828	0,084922413	0	N.A.	0
TEX11	0,221228672	-0,006278995	0	N.A.	0
CTA-941F9.9	0,221155572	0,334516783	0	N.A.	0
GCLC	0,2211143	0,203003275	0	N.A.	0
TTC39B	0,221059565	0,193832721	0	N.A.	0
SENP7	0,221042821	0,184126736	0	N.A.	0
NR4A2	0,220887582	0,110930421	0	N.A.	0
HIF1A-AS2	0,220818114	-0,088374596	0	N.A.	0
KB-1460A1.5	0,220808726	0,299730733	0	N.A.	0
MXRA7	0,220736312	0,177800685	0	N.A.	0
PTGS2	0,220716102	0,291319949	0	N.A.	0
TAGLN	0,220697151	-0,153463802	0	N.A.	0
GPBP1L1	0,220671065	0,21350908	0	N.A.	0

VEGFB	0,220665988	0,208320219	0	N.A.	0
TMEM82	0,220630067	0,264743072	0	N.A.	0
TP53INP1	0,220627233	0,229851346	0	N.A.	0
CRCT1	0,22047864	0,330916878	0	N.A.	0
TSPAN12	0,220369186	0,190796903	0	N.A.	0
PIP	0,220367181		0	N.A.	0
KIDINS220	0,220104003	0,506989652	0	N.A.	0
LAMP2	0,219931514	0,228011725	0	N.A.	0
RP1-142L7.8	0,219731638	0,013411814	0	N.A.	0
LINC00628	0,219691294	0,170285162	0	N.A.	0
PPP1R26	0,219678931	0,303374556	0	N.A.	0
SEC24B	0,219585103	0,212453315	0	N.A.	0
GLRA4	0,219557396	0,406689235	0	N.A.	0
ZNF862	0,21949538	-0,101902007	0	N.A.	0
PUM2	0,219336959	0,180446449	0	N.A.	0
CLIP4	0,219301716	0,13191909	0	N.A.	0
PGBD5	0,219266549	0,517985643	0	N.A.	0
CHMP4B	0,219217534	0,328380428	0	N.A.	0
ATF3	0,219132385	0,188487303	0	N.A.	0
B3GALTL	0,219119783	0,043487865	0	N.A.	0
NPIPB15	0,218859856	0,208317191	0	N.A.	0
PLA2G10	0,218841117	0,34979062	1	cl1	0
RP11-963H4.	0,218828439	0,192697081	0	N.A.	0
RAB32	0,218815619	0,018011211	0	N.A.	0
RUNX2	0,218791886	0,197798254	0	N.A.	0
WWC3	0,218791414	0,202556887	0	N.A.	0
MAN1B1	0,218768812	0,262082458	0	N.A.	0
LRIF1	0,218758014	0,461991799	0	N.A.	0
PMAIP1	0,218732867	0,258012232	0	N.A.	0
RP11-1152H1	0,218729733		0	N.A.	0
MTMR3	0,218678176	0,210777767	0	N.A.	0
NR1I2	0,218201169	0,061931358	0	N.A.	0
PPP1R15A	0,218193063	0,356105875	0	N.A.	0

RP11-479G22	0,218060398	-0,007320428	0	N.A.	0
LINC00205	0,217930108	-0,047758021	0	N.A.	0
KLK5	0,217912167		1	cls4	0
CDC14B	0,217911505	-0,004446775	0	N.A.	0
ALDH1A3	0,217884714	0,16976811	0	N.A.	0
NXN	0,217883447	-0,011721806	0	N.A.	0
CTXN1	0,217838619	0,344972544	0	N.A.	0
CDC42BPG	0,217712689	0,373195843	0	N.A.	0
RP11-65L3.2	0,217684838	-0,004993334	0	N.A.	0
ANKIB1	0,217568608	0,165516408	0	N.A.	0
HYAL1	0,217469621	0,318138015	0	N.A.	0
INO80D	0,217413627	0,317438654	0	N.A.	0
RP5-1120P11	0,217351699	0,018027288	0	N.A.	0
IL1R1	0,21734133	0,149774798	0	N.A.	0
CTA-363E19.	0,217298572	0,144259561	0	N.A.	0
THBS1	0,217297038	0,257460645	0	N.A.	0
MAPRE2	0,217292259	0,253735698	0	N.A.	0
AC007292.3	0,217270906	-0,106442308	0	N.A.	0
RP11-164P12	0,217256161	0,338442097	0	N.A.	0
ADHFE1	0,217186643		0	N.A.	0
STX19	0,217063301	0,042020573	0	N.A.	0
GOLT1B	0,217028231	0,262316195	0	N.A.	0
UBXN10-AS1	0,217004834	-0,236690497	0	N.A.	0
FAM160B1	0,216912006	0,296319272	0	N.A.	0
ADAR	0,216800892	0,44225527	0	N.A.	0
ERGIC1	0,216642526	0,213573092	0	N.A.	0
RP11-345F18	0,216558388	0,169833263	0	N.A.	0
EHBP1	0,216445503	0,299338068	0	N.A.	0
WFDC13	0,216411979	-0,264942842	0	N.A.	0
MSMO1	0,216340012	0,49127747	0	N.A.	0
RP11-351J23	0,216023404	0,10037045	0	N.A.	0
NOL4L	0,216011848	0,305254234	0	N.A.	0
USP54	0,215979392	0,346405607	0	N.A.	0

LRRC4	0,215978248	0,109155051	0	N.A.	0
COPA	0,215942573	0,294952481	0	N.A.	0
POLR2J3	0,215937338	0,325172791	0	N.A.	0
KIF7	0,21591437	0,063824988	0	N.A.	0
ENPP4	0,215907312	0,186005792	0	N.A.	0
UPK3BL	0,215876593		0	N.A.	0
TMEM259	0,215729957	0,662760017	0	N.A.	0
RP11-336A1(	0,215725565	0,062420431	0	N.A.	0
SPINK6	0,21565679		0	N.A.	0
PHLPP2	0,215555317	0,258482007	0	N.A.	0
HHATL	0,215438949	0,364486685	0	N.A.	0
SHISA4	0,215415203	0,332381811	0	N.A.	0
RNF128	0,215395533	0,205638174	0	N.A.	0
GBP4	0,21528677	0,086574047	0	N.A.	0
CDS1	0,215248757	0,654756393	0	N.A.	0
CDC42SE1	0,215220819	-0,064605592	0	N.A.	0
CC2D1B	0,215188031	0,198626374	0	N.A.	0
EDEM3	0,215170015	0,427281744	0	N.A.	0
SPRR1A	0,21514281	0,2674194	0	N.A.	0
RP11-64C12.	0,215083308	-0,165121687	0	N.A.	0
DICER1	0,215068186	0,34460176	0	N.A.	0
MOB3C	0,215045403	0,34239912	0	N.A.	0
BTBD19	0,215000909	0,3163065	0	N.A.	0
KDSR	0,214935591	0,151357801	0	N.A.	0
RP5-1158E12	0,21491263	0,129046224	0	N.A.	0
DHX32	0,214888002	0,30249191	0	N.A.	0
PTPN23	0,214840699	0,139543565	0	N.A.	0
C1orf228	0,214745775	0,135167781	0	N.A.	0
MAU2	0,214688643	0,20354822	0	N.A.	0
LINC00657	0,214645431	0,22810502	0	N.A.	0
TMEM150B	0,21439835	0,521939357	0	N.A.	0
DFNA5	0,214319033		0	N.A.	0
FKBP2	0,214272578	0,20560262	0	N.A.	0

MTRNR2L6	0,214208383	0,068157885	0	N.A.	0
ZMYM6	0,21410267	-0,127461063	0	N.A.	0
KISS1	0,21410155	0,45217493	0	N.A.	0
EXOC5	0,214084564	0,080636504	0	N.A.	0
CTD-3252C9.	0,214063514	0,559548004	0	N.A.	0
ATHL1	0,214057577	0,399106571	0	N.A.	0
VAMP2	0,214027769	0,309223821	0	N.A.	0
TGM1	0,213890422	-0,067401755	0	N.A.	0
RAI14	0,213829938	0,334103444	0	N.A.	0
CH507-42P11	0,213778497	0,193368375	0	N.A.	0
FARP1-AS1	0,213585739	0,226100191	0	N.A.	0
EMP3	0,213484603	0,216929312	0	N.A.	0
MID2	0,213462578	0,121094367	0	N.A.	0
TBC1D22A	0,213458377	0,470598322	0	N.A.	0
HLA-DRB1	0,213429895	-0,262061867	0	N.A.	0
AC104654.1	0,213389891	0,120342371	0	N.A.	0
ZFYVE26	0,213342302	0,143430204	0	N.A.	0
KIFC2	0,213202266	0,350916867	0	N.A.	0
AC058791.1	0,213157447	-0,005892036	0	N.A.	0
WWC2	0,213050512	0,2924548	0	N.A.	0
SREBF2	0,213045787	0,454789893	0	N.A.	0
LINC00086	0,213020952	-0,025892689	0	N.A.	0
TMEM191C	0,212979964	0,268577546	0	N.A.	0
CCL8	0,212970068		0	N.A.	0
SLC2A10	0,212967736	0,255096968	0	N.A.	0
DOPEY2	0,212917863	0,34159137	0	N.A.	0
CBFB	0,212915885	0,071939285	0	N.A.	0
TMEM256	0,212669149	-0,142801739	0	N.A.	0
BMS1P14	0,21251583	0,281279608	0	N.A.	0
RRAGD	0,212489307	0,016453635	0	N.A.	0
GBP5	0,212473182		0	N.A.	0
ADCY10	0,212437577	0,129534511	0	N.A.	0
LRRFIP2	0,2122675	0,180399745	0	N.A.	0

RP11-103J8.1	0,212094654	0,183377468	0	N.A.	0
RAD9A	0,211908059	0,415584612	0	N.A.	0
DDX58	0,211904973	0,366493363	0	N.A.	0
ZBTB20-AS2	0,211842643	0,009907413	0	N.A.	0
ZNF365	0,211783054	0,172018135	0	N.A.	0
JAK3	0,211742007	0,076299189	0	N.A.	0
TEAD3	0,211735803	0,367648467	0	N.A.	0
MAST4	0,211656877	0,237294167	0	N.A.	0
SNAP23	0,211645622	0,114303988	0	N.A.	0
SLC46A3	0,211555848	0,339724519	0	N.A.	0
R3HDM2	0,211450031	-0,062961671	0	N.A.	0
ARIH1	0,211173606	0,340941054	0	N.A.	0
ZNF579	0,211158793	-0,167668054	0	N.A.	0
ZBTB46	0,211005116	0,361759291	0	N.A.	0
AXIN1	0,210962126	0,275000021	0	N.A.	0
PPP1R3G	0,210938101	0,440797707	0	N.A.	0
JMJD1C-AS1	0,21079297	0,20107381	0	N.A.	0
SCARF1	0,210744013	0,355839783	0	N.A.	0
KRCC1	0,210552564	0,177530797	0	N.A.	0
GALC	0,210551785	0,403094699	0	N.A.	0
PPP1R3B	0,210520095	0,236848593	0	N.A.	0
AP5Z1	0,21046666	0,386213947	0	N.A.	0
C5orf42	0,21043637	0,181159392	0	N.A.	0
GPA33	0,210429251	0,31912343	0	N.A.	0
AC024560.2	0,210416901	0,255776417	0	N.A.	0
PLEKHD1	0,210270223	-0,0008595	0	N.A.	0
ACSF2	0,210245237	0,17643958	0	N.A.	0
SUN3	0,210185417	0,21770653	0	N.A.	0
TMEM144	0,210160562	0,182920062	0	N.A.	0
KIF9-AS1	0,210107299	0,030574557	0	N.A.	0
HTR3A	0,209976021		0	N.A.	0
LCAT	0,209948711	0,454455692	0	N.A.	0
MTMR10	0,209940546	0,062020788	0	N.A.	0

XIST	0,209883634	0,232539632	0	N.A.	0
DSG4	0,209849383	0,257406828	0	N.A.	0
ADRB1	0,209825505	0,002110921	0	N.A.	0
ACSL3	0,209822547	0,402267853	0	N.A.	0
LEMD3	0,209808987	0,301772299	0	N.A.	0
NF1	0,209592885	0,401551054	0	N.A.	0
KDM6B	0,209577297	0,249318328	0	N.A.	0
CDC37L1	0,209420256	0,093662531	0	N.A.	0
AFF1	0,20941801	0,191105171	0	N.A.	0
FZR1	0,209410616	0,353647477	0	N.A.	0
RP11-164P12	0,209315699	0,045330466	0	N.A.	0
MAP4	0,209313745	0,173130783	0	N.A.	0
LINC01291	0,209294416	0,212642554	0	N.A.	0
MFSD7	0,209285008	0,058773654	0	N.A.	0
DNAJC3	0,209221878	0,473244038	0	N.A.	0
DAG1	0,209175042	0,407149766	0	N.A.	0
ZNF688	0,209049471	-0,012286094	0	N.A.	0
VNN2	0,209009177	-0,060095748	0	N.A.	0
SMAP1	0,208991718	0,356586669	0	N.A.	0
RANBP10	0,208837022	0,485874494	0	N.A.	0
NMUR2	0,208792333	0,161763975	0	N.A.	0
PRKCZ	0,208789595	0,450335618	0	N.A.	0
RLF	0,208741158	0,133737028	0	N.A.	0
SNX30	0,208718625	0,221523634	0	N.A.	0
ACTR2	0,208559911	0,272937728	0	N.A.	0
AK8	0,208469837	0,370542463	0	N.A.	0
SNX19	0,208344752	-0,000286766	0	N.A.	0
CSNK1G3	0,208344456	0,348187786	0	N.A.	0
CLIC6	0,208324296		0	N.A.	0
SLC6A12	0,208246547	0,231129725	0	N.A.	0
PARD3	0,208205522	0,244985983	0	N.A.	0
OSBPL11	0,208195007	0,05339151	0	N.A.	0
RNF152	0,208187718	0,133290073	0	N.A.	0

LRRC19	0,208159154	0,034391504	0	N.A.	0
TMEM86B	0,208075219	0,313059462	0	N.A.	0
ARNTL	0,208044918	0,349787647	0	N.A.	0
PCGF3	0,207721818	0,229744616	0	N.A.	0
LARP4B	0,207587577	0,324875892	0	N.A.	0
IGF2R	0,207484952	0,446030463	0	N.A.	0
DNM3	0,207469176	0,260455235	0	N.A.	0
CTD-2377D24	0,207399034	0,457292755	0	N.A.	0
BTN3A1	0,207385049	0,110983795	0	N.A.	0
ZNF398	0,207315422	-0,016921457	0	N.A.	0
FLRT3	0,207303028	0,298579561	0	N.A.	0
ELF3	0,207290727	0,601706605	0	N.A.	0
ALG2	0,207259505	0,233008873	0	N.A.	0
TAOK1	0,207158794	0,376407995	0	N.A.	0
TFAP2A-AS1	0,20705996	0,019654995	0	N.A.	0
PANX1	0,207046058	-0,234836786	0	N.A.	0
C16orf52	0,206957993	0,056511233	0	N.A.	0
PSORS1C2	0,206945291	0,054770899	0	N.A.	0
PARP10	0,206929118	0,094707388	0	N.A.	0
TBC1D23	0,206902945	0,135073488	0	N.A.	0
RBAK	0,206831029	-0,071212915	0	N.A.	0
ZNF865	0,206783124	0,139436481	0	N.A.	0
PPP1R12B	0,206680515	0,29008159	0	N.A.	0
B3GALT5-AS1	0,206675915	0,221365843	0	N.A.	0
RP11-1017G2	0,206528857	-0,134688817	0	N.A.	0
FBXW11	0,206486715	0,136992047	0	N.A.	0
PTPN14	0,206485258	0,389726924	0	N.A.	0
MUL1	0,206467122	0,126449441	0	N.A.	0
GMIP	0,206462424	0,374654488	0	N.A.	0
SMEK1	0,206228775	0,270316852	0	N.A.	0
TMEM51	0,206154974	0,417223378	0	N.A.	0
SVIL-AS1	0,206140303	0,193245116	0	N.A.	0
RP11-15I11.3	0,206116792	-0,03410022	0	N.A.	0

SSH3	0,206105447	0,465957401	0	N.A.	0
ZNF91	0,206102758	0,330806671	0	N.A.	0
RAB3GAP2	0,20608266	0,263775964	0	N.A.	0
PLSCR4	0,205991977	0,0116747	0	N.A.	0
OBFC1	0,205967648	0,144378001	0	N.A.	0
ERAP1	0,205956102	0,045193175	0	N.A.	0
CLCN7	0,205938016	0,150830824	0	N.A.	0
CLLU1OS	0,205862355		0	N.A.	0
PRKCA	0,205666853	0,282055855	0	N.A.	0
GATAD2B	0,205537939	0,130160118	0	N.A.	0
EVI2B	0,205534431	0,162978854	0	N.A.	0
SRMS	0,205447288	0,239821758	0	N.A.	0
SI	0,20542566	0,338702834	0	N.A.	0
C14orf37	0,205391385	0,188915361	0	N.A.	0
LRP5	0,205354374	0,43749352	0	N.A.	0
DOCK6	0,205339871	0,397124056	0	N.A.	0
ALPK3	0,205314317	0,055205422	0	N.A.	0
AC116366.6	0,2053086		0	N.A.	0
RP11-65L3.4	0,205030998	-0,063223559	0	N.A.	0
AL022341.3	0,204948146	-0,112895106	0	N.A.	0
RP11-783K16	0,204888184	-0,060514202	0	N.A.	0
OSBPL1A	0,20466862	0,451004463	0	N.A.	0
TOX3	0,204660648	0,365558325	0	N.A.	0
KPNA1	0,204650266	0,025388254	0	N.A.	0
RIMS3	0,204468416	0,328899369	0	N.A.	0
CCDC146	0,204396403	0,093261325	0	N.A.	0
ATP7A	0,204349426	0,081950942	0	N.A.	0
WBP5	0,204320233	-0,128005142	0	N.A.	0
HECTD3	0,204184721	0,377655556	0	N.A.	0
LCN12	0,203888741	0,284333662	0	N.A.	0
LYN	0,203832037	0,344475827	0	N.A.	0
RP11-24F11.[illegible]	0,20376793	0,026795062	0	N.A.	0
POFUT2	0,203753326	0,172515817	0	N.A.	0

NAALADL2	0,203581974	0,077431389	0	N.A.	0
SIRPG	0,203508027	-0,128046173	0	N.A.	0
RP11-1017G2	0,203487999	0,079472595	0	N.A.	0
ADNP2	0,203459117	0,180395841	0	N.A.	0
OTUD5	0,203442415	0,154451272	0	N.A.	0
GAL3ST1	0,203435228	0,171839901	0	N.A.	0
BAZ2B	0,203375545	0,446967338	0	N.A.	0
ZBTB7C	0,203334824	0,111640592	0	N.A.	0
SLAMF9	0,203305487	0,196409076	0	N.A.	0
HTT	0,203262379	0,296110191	0	N.A.	0
COL14A1	0,20321869	-0,113799011	0	N.A.	0
BIRC6	0,203172821	0,347800839	0	N.A.	0
BIK	0,203011809	0,273715846	0	N.A.	0
BEND7	0,202935783	0,197884279	0	N.A.	0
PUM1	0,202900607	0,339306292	0	N.A.	0
FAM95B1	0,202827072	-0,008324852	0	N.A.	0
IFFO2	0,202732832	0,260368043	0	N.A.	0
PATL1	0,202732372	0,295770224	0	N.A.	0
ARHGAP32	0,202711565	0,256964294	0	N.A.	0
SLAMF8	0,202587544	-0,062755046	0	N.A.	0
COBLL1	0,202554351	0,151349497	0	N.A.	0
SLC1A3	0,202551164	0,043986681	0	N.A.	0
SC5D	0,202514072	-0,22501179	0	N.A.	0
RAB35	0,20249722	0,203269369	0	N.A.	0
MAGI3	0,202435937	0,504353391	0	N.A.	0
LINC01551	0,202384572	0,083739774	0	N.A.	0
CCDC91	0,202297559	0,327999299	0	N.A.	0
ZNF655	0,202195562	0,183612692	0	N.A.	0
COG6	0,202152913	0,305858461	0	N.A.	0
STK11	0,202112735	0,254455143	0	N.A.	0
CDRT1	0,202022822	0,258236518	0	N.A.	0
SLC7A9	0,201976311	0,134996735	0	N.A.	0
DPP9	0,201937759	0,355219985	0	N.A.	0

KIAA1033	0,201911819	0,34178834	0	N.A.	0
EVA1C	0,201899307	0,326415152	0	N.A.	0
MSR1	0,201888325	-0,017801985	0	N.A.	0
EGLN2	0,20187744	0,132220478	0	N.A.	0
ZNF556	0,201767372	0,099564762	0	N.A.	0
AC023590.1	0,201648026	0,030202259	0	N.A.	0
AQP11	0,20152393	0,219513715	0	N.A.	0
CCRN4L	0,201518894	0,233677728	0	N.A.	0
RP11-503E24	0,201481252	0,264035607	0	N.A.	0
ERICD	0,201453295		0	N.A.	0
ALDOA	0,20135613	0,200430767	0	N.A.	0
GUCY2C	0,201318987	0,203660849	0	N.A.	0
NKAIN1	0,201291734		0	N.A.	0
FOXN3	0,201195736	0,244331656	0	N.A.	0
MGAT5	0,201173113	0,166504847	0	N.A.	0
AC010642.1	0,201115955	-0,128419193	0	N.A.	0
RP11-181E10	0,20106777		0	N.A.	0
IRF2	0,200850815	0,16009975	0	N.A.	0
CHMP4C	0,200810446	0,375504815	0	N.A.	0
KLC1	0,200563631	0,388565427	0	N.A.	0
MPP7	0,200553169	0,182957194	0	N.A.	0
GPR137	0,200532148	0,149455465	0	N.A.	0
LRRC23	0,200438913	-0,323135784	0	N.A.	0
ZC3H6	0,200413494	0,226212961	0	N.A.	0
WARS	0,200368552	-0,055676022	0	N.A.	0
RP11-15A1.7	0,200328008	0,018713396	0	N.A.	0
IL13RA1	0,200310328	0,546469175	0	N.A.	0
KCNQ1OT1	0,200265855	0,468345745	0	N.A.	0
CDC42	0,200194785	-0,050455448	0	N.A.	0
TSC1	0,200179801	0,103330496	0	N.A.	0
ACADVL	0,200156364	0,311694276	0	N.A.	0
IFIH1	0,200018141	0,446531251	0	N.A.	0
JSRP1	0,199908287	0,507189526	0	N.A.	0

BMP2K	0,199836103	0,110333273	0	N.A.	0
NR2F6	0,1998017	0,531703995	0	N.A.	0
RP11-124N14	0,199777362	-0,084786397	0	N.A.	0
SUSD1	0,199770983	0,157708605	0	N.A.	0
VPS41	0,199690577	-0,070734268	0	N.A.	0
SLC50A1	0,199622463	0,328841079	0	N.A.	0
CARHSP1	0,19947022	0,186289641	0	N.A.	0
GNAL	0,199442087	-0,302917571	0	N.A.	0
AC073283.4	0,199432899	-0,083055656	0	N.A.	0
CLASP1	0,199319668	0,152358143	0	N.A.	0
GPR157	0,199203132	0,108904422	0	N.A.	0
NAV2	0,199194872	-0,094800406	0	N.A.	0
RP11-73M7.1	0,199156308	-0,120526179	0	N.A.	0
AC093642.3	0,19912555	0,150675228	0	N.A.	0
MAN1A2	0,199118549	0,417814487	0	N.A.	0
EPN1	0,199116119	0,585877944	0	N.A.	0
AC061992.2	0,199025283	0,383614409	0	N.A.	0
LRIG2	0,19894672	0,203214872	0	N.A.	0
LINC01137	0,198900027	0,0747961	0	N.A.	0
LGR6	0,198853686	0,240046382	0	N.A.	0
ATG101	0,198671575	0,007947001	0	N.A.	0
RP11-399E6.4	0,198656762	0,351906386	0	N.A.	0
NR2E3	0,198642429		0	N.A.	0
PRKAB2	0,198593276	0,241287276	0	N.A.	0
CAMTA2	0,198574814	0,275145005	0	N.A.	0
GPR132	0,198566555	0,126923482	0	N.A.	0
SPECC1	0,19849826	0,106377728	0	N.A.	0
VLDLR-AS1	0,198467059	0,137953179	0	N.A.	0
INPPL1	0,198393024	0,042112104	0	N.A.	0
EGOT	0,198321674	0,237220524	0	N.A.	0
TBC1D17	0,198210049	0,140004588	0	N.A.	0
STK17B	0,198127111	0,08598319	0	N.A.	0
FAM160B2	0,19812555	0,440200946	0	N.A.	0

CYP24A1	0,198111604	0,362305242	0	N.A.	0
SCNN1D	0,197889615	0,303916598	0	N.A.	0
ACE	0,197741595	0,261750087	0	N.A.	0
AC016735.2	0,197659856	0,274318525	0	N.A.	0
RP11-78C3.1	0,197602184	0,124083343	0	N.A.	0
PLA2R1	0,197479783	0,419228531	0	N.A.	0
YPEL2	0,197479574	0,259419682	0	N.A.	0
RP3-508I15.2	0,197456768	0,417695406	0	N.A.	0
EPS15	0,197410074	0,080480888	0	N.A.	0
CLCN6	0,197381575	0,210811406	0	N.A.	0
RP11-432I5.6	0,197357409		0	N.A.	0
TNF	0,197324301	0,397302618	0	N.A.	0
RP11-430H10	0,197249093		0	N.A.	0
QPCT	0,197111542	0,18437455	0	N.A.	0
HOOK2	0,197009617	0,525246441	0	N.A.	0
DENND1A	0,196981689	-0,076577163	0	N.A.	0
EDF1	0,196981205	0,176955434	0	N.A.	0
CBLN3	0,196964816	0,187652652	0	N.A.	0
CPEB1-AS1	0,196916365	-0,11468637	0	N.A.	0
CRELD1	0,196899399	0,167119168	0	N.A.	0
TGFB1	0,196865654	0,086276346	0	N.A.	0
PLEKHO2	0,196834904	-0,032405043	0	N.A.	0
UVSSA	0,196825266	0,185345503	0	N.A.	0
LAD1	0,196817045	0,471367998	0	N.A.	0
SH3BP4	0,19681514	0,393471698	0	N.A.	0
FOXO4	0,196747196	0,01979061	0	N.A.	0
TMEM204	0,196706343	-0,198968058	0	N.A.	0
LATS2-AS1	0,196622198	0,062777842	0	N.A.	0
RP11-701P16	0,196602172		0	N.A.	0
KBTBD11	0,196439168	0,004752443	0	N.A.	0
SKI	0,196436354	0,190789216	0	N.A.	0
RP11-367N14	0,196356047	0,252622771	0	N.A.	0
TMEM185A	0,196267981	0,292010518	0	N.A.	0

PCYT1A	0,195982958	0,23913671	0	N.A.	0
HAVCR2	0,195928416	-0,263036999	0	N.A.	0
C17orf78	0,195794864	0,072791177	0	N.A.	0
DISP2	0,195659959	0,429198275	1	none	0
MIR503HG	0,195644219	0,011080126	0	N.A.	0
HIGD1A	0,195597056	0,252023994	0	N.A.	0
TMCO3	0,195581877	0,445027877	0	N.A.	0
NCKAP5	0,19548832	0,364869234	0	N.A.	0
PANX2	0,195433079	0,0307049	0	N.A.	0
IL17RC	0,195323779	0,429217288	0	N.A.	0
RABGAP1L	0,195320167	0,218300121	0	N.A.	0
ATG9A	0,195297559	0,342519824	0	N.A.	0
RP11-502H18	0,195286096	0,186222427	0	N.A.	0
CCR3	0,195276001	0,249789396	0	N.A.	0
RAET1E	0,195216332	0,196291166	0	N.A.	0
GABRP	0,195161086	-0,041163297	1	cl2	0
BCL6	0,195075721	0,390436028	0	N.A.	0
RP11-1143G9	0,195059927	-0,171425823	0	N.A.	0
SPON2	0,195006517	0,075639516	0	N.A.	0
CCDC13	0,194962887	0,320495717	0	N.A.	0
IGF1R	0,19489092	0,419955449	0	N.A.	0
RP1-140K8.5	0,194840378	0,364878277	0	N.A.	0
CAPN15	0,194732473	0,155685219	0	N.A.	0
SCNN1B	0,194645985	0,076287366	0	N.A.	0
TNC	0,194482763	-0,054453665	0	N.A.	0
STX2	0,194351472	0,319034277	0	N.A.	0
SPTY2D1	0,194350949	0,391450377	0	N.A.	0
FABP1	0,194326221	-0,056783746	0	N.A.	0
RP11-264B17	0,194314341		0	N.A.	0
COL4A3BP	0,194304847	0,144427155	0	N.A.	0
ENC1	0,194301997	0,325770653	0	N.A.	0
PEAK1	0,194124823	0,184590732	0	N.A.	0
MAPK9	0,194084961	0,203252077	0	N.A.	0

C6orf62	0,194051235	0,259824303	0	N.A.	0
ITGB7	0,193886273	0,177788972	0	N.A.	0
PALM	0,193870431		0	N.A.	0
DRAM2	0,193611546	0,097611843	0	N.A.	0
SPECC1L	0,193603098	0,081534164	0	N.A.	0
KMO	0,193543209	0,116964002	0	N.A.	0
ZNF281	0,193531913	0,285399855	0	N.A.	0
TM7SF2	0,193475946	0,225796874	0	N.A.	0
RP11-126O1.	0,1934488	0,027892163	0	N.A.	0
DMRTA1	0,19344216	-0,026863219	0	N.A.	0
FADS6	0,19343427	0,334074417	0	N.A.	0
AGO4	0,193425873	0,187022652	0	N.A.	0
ZNF611	0,193420871	0,059214717	0	N.A.	0
PAQR7	0,19337607	0,201581501	0	N.A.	0
TNFRSF11A	0,193334766	0,249977198	0	N.A.	0
ITCH-AS1	0,193237064		0	N.A.	0
LINC00960	0,193229192	-0,21062368	0	N.A.	0
SLC39A9	0,192978866	0,303772406	0	N.A.	0
ANXA8L1	0,192916433	0,362532567	0	N.A.	0
C1orf177	0,192905338	-0,191221057	0	N.A.	0
TGFBR1	0,192788229	0,1714196	0	N.A.	0
RP11-41O4.2	0,192746735		0	N.A.	0
PCBP4	0,192727512	0,26376695	0	N.A.	0
GOLGA5	0,192724013	0,094303599	0	N.A.	0
SYNGR3	0,192704131	0,41891769	0	N.A.	0
RP11-800A18	0,192653336	-0,070637413	0	N.A.	0
RP11-119D9.	0,192585491	-0,125479217	0	N.A.	0
TFPI2	0,192447078	0,005443537	0	N.A.	0
LUZP1	0,192292473	0,191599818	0	N.A.	0
SLC34A3	0,192236845	0,298638321	0	N.A.	0
CD34	0,192220813	-0,244211821	0	N.A.	0
MYO9A	0,19214202	0,228518167	0	N.A.	0
PEX11B	0,19206265	-0,144699839	0	N.A.	0

SOCS5	0,191883505	0,108003548	0	N.A.	0
TLDC2	0,191858853	0,29590979	0	N.A.	0
SLC11A1	0,191853708	-0,257734828	0	N.A.	0
ZNF430	0,191809338	0,447954317	0	N.A.	0
PAQR3	0,191808088	0,199778966	0	N.A.	0
ZNF224	0,191788131	0,25246198	0	N.A.	0
ACP2	0,191727106	0,32106839	0	N.A.	0
SLC45A2	0,19170343		0	N.A.	0
SLCO4A1	0,191632308	0,292933328	0	N.A.	0
RP11-48B3.3	0,191536307	0,072590542	0	N.A.	0
CLTCL1	0,191521505	0,220769341	0	N.A.	0
HIPK1	0,191362986	0,457826858	0	N.A.	0
EVX1	0,191313892	0,167724411	0	N.A.	0
RP11-212I21	0,191299291	-0,002618707	0	N.A.	0
NWD1	0,191180155	0,041196574	0	N.A.	0
SLC2A1-AS1	0,191154003	0,18718078	0	N.A.	0
MAPK7	0,191152431	-0,153768661	0	N.A.	0
RP11-290M5	0,19114082		0	N.A.	0
NAA60	0,19111669	0,130957518	0	N.A.	0
MAP1LC3A	0,191013754	0,26466483	0	N.A.	0
HHIP-AS1	0,191006984		0	N.A.	0
PSD3	0,190998274	0,166129629	0	N.A.	0
NOMO1	0,190988724	0,262933254	0	N.A.	0
RP11-20I23.7	0,190834126		0	N.A.	0
RAB25	0,190824724	0,296884963	0	N.A.	0
RP11-367G18	0,190798823	0,11477895	0	N.A.	0
SPEN	0,190653004	0,354530345	0	N.A.	0
CARS	0,190619311	0,344191179	0	N.A.	0
SH3PXD2A	0,190605828	0,453452121	0	N.A.	0
C1orf50	0,19054195	0,070039265	0	N.A.	0
GMEB2	0,190388762	0,012639	0	N.A.	0
CHFR	0,190315632	0,066699097	0	N.A.	0
PHLDB2	0,190189822	0,573100138	0	N.A.	0

PRDM6	0,190109636	-0,220600048	0	N.A.	0
FLJ22763	0,189956512	0,071819383	0	N.A.	0
CHRNA10	0,189907827	0,045589773	0	N.A.	0
KIAA1875	0,189810428	0,102898674	0	N.A.	0
WI2-87327B8	0,189773264	-0,04443539	0	N.A.	0
RP11-93B14.	0,189745906	0,169638062	0	N.A.	0
CSF3R	0,189719223	0,224801866	0	N.A.	0
CPT1B	0,18970587	0,166635203	0	N.A.	0
EPHA10	0,189704536	0,346883593	0	N.A.	0
TMEM200B	0,189665668	0,011327148	0	N.A.	0
ILDR1	0,189584578	0,293952873	0	N.A.	0
FSIP2	0,189526075	0,234005825	0	N.A.	0
LA16c-431H6	0,189524782	0,158850859	0	N.A.	0
FAM110C	0,189377154	0,004552687	0	N.A.	0
TMEM214	0,189283039	0,532698634	0	N.A.	0
RALGAPA2	0,189255002	0,185948422	0	N.A.	0
TMEM236	0,189252474	0,268981706	0	N.A.	0
JDP2	0,189215018	-0,024549676	0	N.A.	0
MIB2	0,189212413	0,326017449	0	N.A.	0
GALNT2	0,189204934	0,320055377	0	N.A.	0
MED29	0,189188205	-0,035217126	0	N.A.	0
NDST1	0,18906313	0,074563775	0	N.A.	0
NTAN1	0,189007698	-0,300952846	0	N.A.	0
PPAP2A	0,188858022	0,349381721	0	N.A.	0
POLR2A	0,188782892	0,43808144	0	N.A.	0
PLB1	0,188717404	0,110875331	0	N.A.	0
TFF3	0,188609982	0,265871073	0	N.A.	0
UBA7	0,188548174	0,322660155	0	N.A.	0
RIN3	0,188452127	0,055503366	0	N.A.	0
MYL12A	0,188362497	0,199997631	0	N.A.	0
ARHGEF6	0,18830355	0,042179586	0	N.A.	0
ZNF75D	0,188293372	0,068549096	0	N.A.	0
FGF2	0,188282076	0,349306779	0	N.A.	0

RP11-96D1.1	0,18826604	0,179981841	0	N.A.	0
RP11-496I9.1	0,188145585	0,22857804	0	N.A.	0
ARHGEF28	0,188028215	0,085592065	0	N.A.	0
STARD3	0,187968275	-0,041523282	0	N.A.	0
AC026202.3	0,187800921	0,033679502	0	N.A.	0
UCKL1-AS1	0,187748713	0,148216955	0	N.A.	0
RBM24	0,187606275	0,246690374	0	N.A.	0
GRAMD4	0,18749335	0,486563336	0	N.A.	0
ARHGAP5-AS	0,18732337	0,075456476	0	N.A.	0
KRT81	0,187306408	-0,225765573	0	N.A.	0
NPR2	0,187228539	0,083114843	0	N.A.	0
RILPL1	0,187162762	0,132828607	0	N.A.	0
RP11-77H9.5	0,187158697		0	N.A.	0
TPSB2	0,187113798	-0,090018494	0	N.A.	0
FAM102B	0,187085152	-0,063580061	0	N.A.	0
KMT2E	0,186997121	0,317666103	0	N.A.	0
ADCY6	0,186862384	0,237668062	0	N.A.	0
RNMT	0,186856197	0,41115688	0	N.A.	0
KCNE3	0,186730592	0,042602098	0	N.A.	0
CCDC170	0,186672454	0,191540564	0	N.A.	0
TMPRSS7	0,186527691	0,570917211	0	N.A.	0
ZNF189	0,186320438	-0,094008437	0	N.A.	0
BMF	0,186266357	0,054816433	0	N.A.	0
EPDR1	0,186142921	0,386682662	0	N.A.	0
FOLR2	0,186127629	0,028792976	0	N.A.	0
CDH4	0,186075885		0	N.A.	0
CYBRD1	0,18606719	-0,008287856	0	N.A.	0
AHSA2	0,186064762	0,326869786	0	N.A.	0
NISCH	0,186011872	0,377903147	0	N.A.	0
RNF13	0,185888767	-0,081351202	0	N.A.	0
RP11-420L9.5	0,185862913	0,20014337	0	N.A.	0
ZNF44	0,185839009	0,061385438	0	N.A.	0
TBL1XR1	0,185830706	0,157244983	0	N.A.	0

PIEZO1	0,185802173	0,460296307	0	N.A.	0
ASPH	0,185785124	0,581904323	0	N.A.	0
ABHD17B	0,185768456	0,023572806	0	N.A.	0
METTL15	0,185749766	-0,294136527	0	N.A.	0
LIMCH1	0,185729708	0,303547747	0	N.A.	0
NPR3	0,185680466	-0,130813987	0	N.A.	0
TM6SF1	0,18566874		0	N.A.	0
CTB-178M22	0,185613862		0	N.A.	0
TBX2-AS1	0,185596848	0,03080412	0	N.A.	0
VCPIP1	0,185432188	0,293958862	0	N.A.	0
CCDC9	0,185339932	0,128068274	0	N.A.	0
CLTC	0,185289468	0,326942008	0	N.A.	0
EZH1	0,185245114	0,356694756	0	N.A.	0
ZNF532	0,18523375	0,077699423	0	N.A.	0
ZNF827	0,18519599	0,033131766	0	N.A.	0
PDLIM4	0,185167427	0,094329668	0	N.A.	0
CTDNEP1	0,18515726	0,149387475	0	N.A.	0
CTD-2376I4.2	0,185113635	0,176268525	0	N.A.	0
PPM1A	0,185102975	0,197000334	0	N.A.	0
ZSCAN25	0,185095317	-0,226823777	0	N.A.	0
CD22	0,18509379		0	N.A.	0
CCL2	0,185043502	0,127042537	0	N.A.	0
PHF21A	0,18499873	-0,147248004	0	N.A.	0
CH17-132F21	0,184992003	0,092583329	0	N.A.	0
NBR1	0,184923643	0,333608717	0	N.A.	0
EPB41L5	0,184850495	0,310744807	0	N.A.	0
RNF24	0,184831687	0,159384318	0	N.A.	0
TOB2	0,184797955	0,151945369	0	N.A.	0
SH3TC2	0,184771532	0,289258418	0	N.A.	0
HCN3	0,184741593	-0,04374674	0	N.A.	0
AC083843.1	0,184740414	0,16290718	0	N.A.	0
AL163953.2	0,184724536	-0,062569622	0	N.A.	0
GALNT7	0,184637822	0,421718248	0	N.A.	0

UBL4B	0,184612634		0	N.A.	0
SLC22A14	0,184587024	0,153750222	0	N.A.	0
NRBP2	0,184583355	0,259704316	0	N.A.	0
PER3	0,184573381	0,2158173	0	N.A.	0
C5orf15	0,184402966	0,162090383	0	N.A.	0
TNS3	0,1843745	0,235259241	0	N.A.	0
TMPRSS6	0,184347768	-0,208465827	0	N.A.	0
ELOVL7	0,184343625	0,395864712	0	N.A.	0
XAGE2B	0,184332556	-0,315517422	0	N.A.	0
AMN1	0,184311949	0,199592157	0	N.A.	0
GYS1	0,184305503	0,166788918	0	N.A.	0
OGFRL1	0,184275145	0,434598765	0	N.A.	0
ZNF778	0,184230287	0,031992613	0	N.A.	0
NRBF2	0,184216954	0,150679131	0	N.A.	0
LINC01191	0,184192807	0,14117977	0	N.A.	0
FTX	0,184170688	0,227427751	0	N.A.	0
PLXNA2	0,184131081	0,359166531	0	N.A.	0
CHST5	0,184094291	-0,022545622	0	N.A.	0
RP11-164O23	0,184073511		0	N.A.	0
CLEC14A	0,183999731	-0,149504505	0	N.A.	0
RP11-347P5.	0,183701818	-0,095148846	0	N.A.	0
AL592183.1	0,183558095	0,111833011	0	N.A.	0
PRKCD	0,183491286	0,317377211	0	N.A.	0
CCDC130	0,183364556	0,260202053	0	N.A.	0
LRRC66	0,183333826	0,029757427	0	N.A.	0
STAP2	0,183267313	0,274762631	0	N.A.	0
LINC00887	0,183158553	0,267553522	0	N.A.	0
THAP9-AS1	0,183138838	0,142571561	0	N.A.	0
HERC3	0,183121599	0,132884185	0	N.A.	0
MBP	0,18311903	0,034683329	0	N.A.	0
RHOQ	0,182933911	0,314590838	0	N.A.	0
TNFAIP8	0,182911571	-0,212958274	0	N.A.	0
SPIN1	0,182904899	0,234363579	0	N.A.	0

PPM1N	0,182775274	0,202498515	0	N.A.	0
DGCR5	0,182680056	-0,026340043	0	N.A.	0
TSPAN2	0,182611405	0,216873818	0	N.A.	0
BRD4	0,182586661	0,436012899	0	N.A.	0
KLF10	0,182449126	0,549276544	0	N.A.	0
C2orf88	0,182390378	0,120641286	0	N.A.	0
CAPN3	0,182346156	-0,000309575	0	N.A.	0
CFAP58	0,182290338		0	N.A.	0
DSE	0,182257364	0,004806971	0	N.A.	0
RP11-16E18.	0,182195142	0,349537292	0	N.A.	0
RLIM	0,18217157	0,199153271	0	N.A.	0
TMEM51-AS	0,181976388	0,381338239	0	N.A.	0
HIC2	0,181957984	0,152938817	0	N.A.	0
ZBTB5	0,18194376	0,180906259	0	N.A.	0
CDK13	0,181883678	0,535538917	0	N.A.	0
VPS11	0,181860862	0,14405209	0	N.A.	0
TMEM132A	0,181789639	0,266322787	0	N.A.	0
ALAS1	0,181758453	0,340011447	0	N.A.	0
TRPV3	0,18164193	0,280213113	0	N.A.	0
AMDHD2	0,181620199	0,423874118	0	N.A.	0
KANSL1	0,181502017	0,273253007	0	N.A.	0
ASPHD1	0,181481747	0,005306164	0	N.A.	0
STK4	0,181470451	0,004074473	0	N.A.	0
RP4-651E10.	0,181425687	-0,193282058	0	N.A.	0
LINC00431	0,181384497	0,116525735	0	N.A.	0
FAM118A	0,181304598	0,188783911	0	N.A.	0
RP11-297L17	0,181297054	0,131763734	0	N.A.	0
SLC25A30	0,1811838	-0,064473629	0	N.A.	0
DUSP1	0,18109278	0,203051856	0	N.A.	0
FAM149B1	0,181051688	0,293042436	0	N.A.	0
CTC1	0,181044407	0,063204769	0	N.A.	0
MAP2	0,181004374	0,26945924	0	N.A.	0
MSANTD3	0,180949673	-0,098548804	0	N.A.	0

RP11-175O19	0,180902442	0,345539792	0	N.A.	0
AP3S1	0,180897407	-0,16625635	0	N.A.	0
IQGAP1	0,180832508	0,510673985	0	N.A.	0
NR4A3	0,180830195	0,032291196	0	N.A.	0
BRSK1	0,180775428	0,25908215	0	N.A.	0
TLE4	0,18067326	0,296405127	0	N.A.	0
EML2	0,18057279	0,330799155	0	N.A.	0
C1orf56	0,180464349	0,013016866	0	N.A.	0
RBMS1	0,180436631	-0,178076682	0	N.A.	0
RP4-548D19.	0,180429286	0,114184082	0	N.A.	0
RP11-585P4.	0,180307098	0,259874763	0	N.A.	0
STIM1	0,180293271	0,097459386	0	N.A.	0
TNFRSF19	0,180224396	-0,010757433	0	N.A.	0
RC3H2	0,180223051	0,269719078	0	N.A.	0
RFX1	0,18020421	-0,006117622	0	N.A.	0
NOL3	0,18016763	0,386450203	0	N.A.	0
CADPS	0,180165488	0,123520386	0	N.A.	0
QRICH2	0,180120133	0,125323948	0	N.A.	0
CHD1	0,180100343	0,270773981	0	N.A.	0
CECR2	0,180085069	-0,146695333	0	N.A.	0
FAM71F1	0,180008609		0	N.A.	0
RP11-809O17	0,179991232	0,374586801	0	N.A.	0
NECAP2	0,179909228	0,235911098	0	N.A.	0
TBC1D32	0,179893912	0,145050296	0	N.A.	0
PALLD	0,179879965	0,398740005	0	N.A.	0
TMEM170A	0,17987344	0,210738683	0	N.A.	0
TAOK2	0,179866671	0,401559012	0	N.A.	0
RP3-508I15.2	0,179845518	0,270324964	0	N.A.	0
QKI	0,179836634	-0,117004773	0	N.A.	0
IL22	0,179820086		0	N.A.	0
RP11-106E15	0,179736395		0	N.A.	0
LTB	0,179733897	-0,00814715	0	N.A.	0
PTPRM	0,179719044	-0,117982549	0	N.A.	0

GPATCH8	0,17962985	0,238959319	0	N.A.	0
C12orf75	0,179572514	0,103656727	0	N.A.	0
CCDC85C	0,179565673	0,141912311	0	N.A.	0
MEX3D	0,179432061	0,215079015	0	N.A.	0
NAGA	0,179426845	0,152212493	0	N.A.	0
PSME4	0,179401649	0,070163463	0	N.A.	0
SHPK	0,179383494	-0,08008486	0	N.A.	0
FAM25G	0,179314989	0,458092806	0	N.A.	0
FMO5	0,179291941	0,147772487	0	N.A.	0
NOTCH3	0,179282254	0,104824775	0	N.A.	0
PPP2R5A	0,17926453	0,147931762	0	N.A.	0
CYFIP1	0,179245908	0,685485491	0	N.A.	0
FHOD1	0,17920176	0,15193158	0	N.A.	0
UPK3B	0,179170722	0,146797673	0	N.A.	0
TBC1D10B	0,179168697	0,280728083	0	N.A.	0
RNF44	0,179032493	0,041103715	0	N.A.	0
RIT1	0,178935056	0,253067002	0	N.A.	0
ICAM2	0,178748539	-0,032413554	0	N.A.	0
SRPR	0,178644434	0,247938817	0	N.A.	0
GPR128	0,178636879	0,204548652	0	N.A.	0
MT-ND3	0,178625205	0,311701558	0	N.A.	0
OAF	0,17861749	0,158504685	0	N.A.	0
RB1CC1	0,178607025	0,028165519	0	N.A.	0
ITIH5	0,178485114	0,158163453	0	N.A.	0
UBASH3B	0,178467229	0,064860937	0	N.A.	0
RP11-820I16	0,178466166	0,035670707	0	N.A.	0
COL6A2	0,178413894	-0,316673789	0	N.A.	0
DNER	0,178400618		0	N.A.	0
VWA7	0,178304158	-0,241094895	0	N.A.	0
PCIF1	0,17822911	0,066834212	0	N.A.	0
AMPD3	0,178219897	0,04376821	0	N.A.	0
ANXA2R	0,178207296	0,106430207	0	N.A.	0
PHLPP1	0,178149117	-0,145965086	0	N.A.	0

LINC00294	0,178086672	0,184071605	0	N.A.	0
VIPR1	0,178036162	0,313338238	0	N.A.	0
SULT1B1	0,17800203	-0,028615991	0	N.A.	0
KIF3B	0,177930224	0,206372618	0	N.A.	0
ADIPOR1	0,177925367	0,424191798	0	N.A.	0
COQ10B	0,177898477	0,197972567	0	N.A.	0
CFAP46	0,177841667	-0,195970343	0	N.A.	0
PVR	0,177837651	0,326378704	0	N.A.	0
ZNF319	0,177828147	0,091219404	0	N.A.	0
ALPP	0,177824248	0,281053112	0	N.A.	0
AP5B1	0,177804241	0,136357631	0	N.A.	0
PTGER4	0,177771115	-0,083512926	0	N.A.	0
CSF2	0,177679953	0,273044424	0	N.A.	0
RP11-95M15	0,177636608	0,109851328	0	N.A.	0
S100A5	0,177602715	-0,003278896	0	N.A.	0
HDHD1	0,177540856	0,23009765	0	N.A.	0
C18orf32	0,177540638	-0,012266114	0	N.A.	0
RUNDC1	0,177461254	0,152661273	0	N.A.	0
AC006942.4	0,177388835	0,024977137	0	N.A.	0
ZRANB1	0,17729379	0,132067775	0	N.A.	0
SLC35B3	0,17725024	0,345607661	0	N.A.	0
MLF1	0,177244372	-0,0223275	0	N.A.	0
EPHA1-AS1	0,177233101	-0,067512272	0	N.A.	0
PIP5KL1	0,177188551	-0,034149377	0	N.A.	0
LINC01239	0,177179427	0,193843058	0	N.A.	0
MBD1	0,177125679	-0,003348399	0	N.A.	0
SLC9A3R1	0,176944147	0,306188555	0	N.A.	0
EBLN3	0,176936232	-0,051488773	0	N.A.	0
P3H2	0,176860531	0,430779148	0	N.A.	0
ABCC5	0,176840691	0,417360854	0	N.A.	0
GABRA2	0,176780561	-0,014099954	0	N.A.	0
RP11-491F9.	0,176780163	-0,083427836	0	N.A.	0
PHF8	0,176767828	0,195821287	0	N.A.	0

FAM189A2	0,17662285	0,188770475	0	N.A.	0
EXOC6B	0,176495313	0,240755725	0	N.A.	0
KIAA0513	0,17638953	0,081975868	0	N.A.	0
DNAJC16	0,176347464	0,207372867	0	N.A.	0
GAB2	0,176338358	0,352993307	0	N.A.	0
ZNF226	0,17631959	0,137095943	0	N.A.	0
CD93	0,176238878	-0,108145598	0	N.A.	0
SPINK5	0,17621807	0,324470044	0	N.A.	0
CRYAB	0,176079463	-0,176184419	0	N.A.	0
RP11-281P23	0,176021971	-0,028222144	0	N.A.	0
RP11-314A20	0,176018367	0,162514099	0	N.A.	0
HIST2H4A	0,176015558	-0,027483463	0	N.A.	0
CAPN12	0,176005724	0,307954849	1	none	0
TMEM158	0,175934516	0,378037699	0	N.A.	0
MTF1	0,175564361	0,264513426	0	N.A.	0
RBL2	0,175445864	0,018614452	0	N.A.	0
FOXL1	0,175440057	0,377920556	0	N.A.	0
ARHGEF10	0,175422897	0,195723315	0	N.A.	0
TMEM92-AS1	0,175394529	0,132469089	0	N.A.	0
GDAP2	0,175345734	0,189848328	0	N.A.	0
GAB1	0,175317398	0,213723087	0	N.A.	0
HDLBP	0,17519332	0,114596042	0	N.A.	0
KCNC4	0,175184529	0,400604519	0	N.A.	0
DNAJA4	0,175169664	0,5639123	0	N.A.	0
ZNF800	0,17512954	0,047753288	0	N.A.	0
RGS20	0,1750808	-0,295481462	0	N.A.	0
CFLAR-AS1	0,175021238	-0,117616867	0	N.A.	0
ANKRD13D	0,17501103	0,363845594	0	N.A.	0
CTA-228A9.4	0,17500629	-0,060887173	0	N.A.	0
CXCL6	0,175001263	-0,013508372	0	N.A.	0
SPNS3	0,174996735	-0,145595202	0	N.A.	0
ATP9B	0,17495517	-0,058470887	0	N.A.	0
RP11-434H6.	0,174945316		0	N.A.	0

SCRN1	0,174866201	0,256346264	0	N.A.	0
IP6K1	0,174747283	0,263515321	0	N.A.	0
IL20RB	0,174696154	0,065705881	0	N.A.	0
FMR1	0,174618603	0,416625376	0	N.A.	0
CTD-2105E13	0,174609342	0,40905254	0	N.A.	0
DPP8	0,174608418	0,223812821	0	N.A.	0
SLC35A5	0,174507211	0,002193807	0	N.A.	0
TMEM87A	0,174337298	0,510788997	0	N.A.	0
ZNF12	0,174290225	0,291899519	0	N.A.	0
BRPF3	0,174288424	0,007782576	0	N.A.	0
RP11-20G13.	0,174200001		0	N.A.	0
OPLAH	0,174139643	0,397807155	0	N.A.	0
TLR9	0,174108188		0	N.A.	0
CDNF	0,174043521	0,193239585	0	N.A.	0
NACC2	0,17404027	0,200497908	0	N.A.	0
ECHDC2	0,174002868	0,33194266	0	N.A.	0
RP11-140K17	0,173979428	-0,036125052	0	N.A.	0
SYS1	0,173946045	-0,022001119	0	N.A.	0
RP11-15F12.	0,173828049	0,21855916	0	N.A.	0
RP11-70P17.	0,173820734	0,143225918	0	N.A.	0
SKIL	0,173798224	0,416627853	0	N.A.	0
FMNL2	0,173751261	0,012870366	0	N.A.	0
PAFAH1B2	0,173582394	0,24352668	0	N.A.	0
TNNI2	0,173579496	-0,022543239	0	N.A.	0
B4GALT4	0,173543294	0,402368745	0	N.A.	0
CTC-529I10.2	0,173521842		0	N.A.	0
CORO1B	0,17347209	0,025604041	0	N.A.	0
LRRC42	0,173404699	0,33093323	0	N.A.	0
IKBKB	0,17337161	0,329253822	0	N.A.	0
ZNF654	0,17330869	0,224940903	0	N.A.	0
SOCS3	0,173301128	0,062711337	0	N.A.	0
LILRA5	0,173225286	0,034646191	0	N.A.	0
KCNK7	0,173182735	0,133265307	0	N.A.	0

RP11-567C2.	0,173168395	0,342050578	0	N.A.	0
RARG	0,17316741	-0,037264045	0	N.A.	0
ZNF697	0,173149771	0,247511506	0	N.A.	0
LRG1	0,173128119	0,223963894	0	N.A.	0
TNNT2	0,173122411	0,324259085	0	N.A.	0
RASA1	0,173119312	0,284034148	0	N.A.	0
SLC33A1	0,173072917	0,258776034	0	N.A.	0
RAP1A	0,173062429	-0,159088535	0	N.A.	0
AATK	0,173019529	0,415777387	0	N.A.	0
KLHL24	0,17301688	-0,067651878	0	N.A.	0
KIAA0195	0,172985358	0,348646774	0	N.A.	0
POLN	0,172892462	0,085766164	0	N.A.	0
ZNF607	0,17278927	-0,070629723	0	N.A.	0
LYPD1	0,172772281		0	N.A.	0
USP35	0,172718069	0,276003493	0	N.A.	0
RP1-122P22.	0,172710973	0,259564258	0	N.A.	0
ZNF335	0,172639515	-0,133589439	0	N.A.	0
CCDC92	0,172557067	3,89924E-05	0	N.A.	0
KB-226F1.2	0,172524889		0	N.A.	0
TMEM176B	0,17251652	0,332774106	0	N.A.	0
TNK1	0,172481729	0,330914812	0	N.A.	0
GLIPR1	0,172415235	-0,179172676	0	N.A.	0
MADD	0,172294669	0,070607136	0	N.A.	0
RP11-677M1	0,172128141	0,271381801	0	N.A.	0
LRRC8D	0,172101286	-0,05618999	0	N.A.	0
GGA3	0,171902345	0,209595781	0	N.A.	0
UNC13A	0,171800041	0,3336272	0	N.A.	0
CCDC50	0,171767279	0,218831292	0	N.A.	0
PROM1	0,171765852	0,223733852	0	N.A.	0
ZACN	0,171764771		0	N.A.	0
RP4-541C22.	0,171703691	0,024340575	0	N.A.	0
TBC1D9	0,171662602	0,106293767	0	N.A.	0
MS4A12	0,171652054	0,225197749	0	N.A.	0

TMEM45A	0,171574111	-0,090108248	0	N.A.	0
VTI1A	0,171547213	0,009579905	0	N.A.	0
TMEM229B	0,171524974	-0,039159383	0	N.A.	0
RABGEF1	0,171495343	-0,21269739	0	N.A.	0
GOLGA1	0,171379056	0,010035834	0	N.A.	0
AC026471.6	0,171289263	0,096176814	0	N.A.	0
EAF2	0,171194724	-0,191423152	0	N.A.	0
N4BP2	0,171167211	-0,108954979	0	N.A.	0
BTBD9	0,170989099	0,092730394	0	N.A.	0
RP11-353N14	0,170933173	0,051096686	0	N.A.	0
SLFNL1-AS1	0,170912746		0	N.A.	0
IL15RA	0,170818914	-0,124846456	0	N.A.	0
VSIG10	0,170777186	0,441086422	0	N.A.	0
CDK17	0,17075057	0,409120456	0	N.A.	0
BROX	0,170720564	0,12051811	0	N.A.	0
HIST1H4E	0,170684144	-0,102146105	0	N.A.	0
CELF1	0,170624034	0,175305391	0	N.A.	0
AC010287.1	0,17057594	0,327275221	0	N.A.	0
ELMSAN1	0,170572782	0,545763565	0	N.A.	0
DRC1	0,170506319	-0,080244804	0	N.A.	0
TRAF6	0,170498153	-0,010422107	0	N.A.	0
MANSC1	0,170480561	0,099714062	0	N.A.	0
ALKBH7	0,170450015	0,200433529	0	N.A.	0
SPATA20	0,170418608	0,218756984	0	N.A.	0
TMEM173	0,170414901	0,116208795	0	N.A.	0
CCAT1	0,170362764	0,0698793	0	N.A.	0
COL28A1	0,170314382	0,33153071	0	N.A.	0
RNASEL	0,170306597	0,265253642	0	N.A.	0
AC007228.9	0,170302308	0,196690384	0	N.A.	0
WHAMM	0,170234431	0,130539234	0	N.A.	0
RP11-104L21	0,170193098		0	N.A.	0
RABGAP1	0,170160118	0,256855497	0	N.A.	0
BATF	0,170073801	-0,061441338	0	N.A.	0

RP11-190J1.3	0,17005759	-0,015845183	0	N.A.	0
DIO3	0,170005305	-0,1680564	0	N.A.	0
CCNDBP1	0,170002835	0,378548986	0	N.A.	0
PLCE1	0,169939076	0,04067697	0	N.A.	0
AGR3	0,169918182	0,194936921	0	N.A.	0
RAB18	0,169852824	0,267168889	0	N.A.	0
LINC01569	0,169795569	0,107337101	0	N.A.	0
SPATA6L	0,169470699	-0,097013237	0	N.A.	0
ZBED2	0,169469776	0,21462849	0	N.A.	0
ARID3B	0,169458455	0,030788816	0	N.A.	0
PKN1	0,169442556	0,374862623	0	N.A.	0
PLK2	0,169319581	0,016097467	0	N.A.	0
SLC35E2B	0,169243686	0,412763086	0	N.A.	0
SP3	0,1692295	0,23469021	0	N.A.	0
STXBP3	0,169085071	0,187306975	0	N.A.	0
SCX	0,169084487	0,044466795	0	N.A.	0
TIGIT	0,169081508	-0,213878845	0	N.A.	0
ZSCAN22	0,16907938	-0,003665226	0	N.A.	0
MOGAT2	0,169078738	0,206846633	0	N.A.	0
CTD-2589M5	0,169055669	0,16178968	0	N.A.	0
ZNF449	0,168987826	0,069522606	0	N.A.	0
CTD-2555C10	0,168939729	0,034527961	0	N.A.	0
NGEF	0,168907479	0,533983327	0	N.A.	0
EPHB6	0,168882092	0,25714509	0	N.A.	0
AQP12B	0,168763292	0,317309979	0	N.A.	0
ZFX	0,168706435	0,247591681	0	N.A.	0
WAC	0,168685239	0,253535968	0	N.A.	0
LIN7B	0,168616408	-0,006778506	0	N.A.	0
RP11-338I21	0,168554468	0,162515599	0	N.A.	0
TMCO4	0,168524904	0,303755914	0	N.A.	0
SOS1	0,168499071	0,218368611	0	N.A.	0
CCDC153	0,168495642	-0,245897391	0	N.A.	0
AP000695.4	0,168452321	0,161130092	0	N.A.	0

GABRR2	0,168349383	0,058329981	0	N.A.	0
NRP1	0,168328858	-0,001590519	0	N.A.	0
PBLD	0,168324388	-0,209240086	0	N.A.	0
MAN1C1	0,168248582		0	N.A.	0
BCAP29	0,168209933	0,159074519	0	N.A.	0
TFPT	0,168132902	0,068522197	0	N.A.	0
CCDC28A	0,168123607	-0,263039087	0	N.A.	0
GUCA2A	0,16805049	0,440568392	0	N.A.	0
RP11-184D12	0,16801781		0	N.A.	0
ORMDL3	0,16795923	0,296636886	0	N.A.	0
RP13-726E6.	0,167916309	0,065756466	0	N.A.	0
DNAH1	0,167781048	0,129276159	0	N.A.	0
RP11-65I12.1	0,167748211	0,532604822	0	N.A.	0
RP1-101D8.1	0,167740092	0,19026848	0	N.A.	0
SOD2	0,167685686	-0,080699742	0	N.A.	0
PITPNM2	0,16750551	-0,071193704	0	N.A.	0
LINC00525	0,167502202	0,235083226	0	N.A.	0
DUSP27	0,167478615	0,157194107	1	none	0
ABCB1	0,16740101	0,297906975	0	N.A.	0
ARHGAP18	0,167382017	-0,041389725	0	N.A.	0
PTGIR	0,167336666		0	N.A.	0
LRP4	0,167336003	-0,00969006	0	N.A.	0
ESAM	0,167307219	0,214088778	0	N.A.	0
RNF2	0,167289488	-0,12946271	0	N.A.	0
PTPLB	0,167283438	0,474586263	0	N.A.	0
CREG2	0,167274224	0,386472538	0	N.A.	0
POT1-AS1	0,167229061	0,098399469	0	N.A.	0
CASC4	0,167221051	0,34637322	0	N.A.	0
RP11-278H7.	0,167199759	-0,196300811	0	N.A.	0
FBXL20	0,16715624	0,032625118	0	N.A.	0
ANKRD1	0,167072026	0,407807316	0	N.A.	0
FAM13A-AS1	0,166826029	0,094832754	0	N.A.	0
APOBEC3C	0,166716722	-0,160914768	0	N.A.	0

MAPK8	0,166692819	0,065189756	0	N.A.	0
IL2RA	0,166560385	0,12624485	0	N.A.	0
RP11-358B23	0,166510661		0	N.A.	0
PTGDS	0,1664442	0,029063499	0	N.A.	0
CSF2RA	0,166416139	0,078253757	0	N.A.	0
USP30	0,166383321	-0,086024601	0	N.A.	0
AKT2	0,16637973	0,331413118	0	N.A.	0
KCTD10	0,16633169	0,116212028	0	N.A.	0
ZNF814	0,166304482	-0,065856381	0	N.A.	0
FCHSD1	0,166284512	0,191453767	0	N.A.	0
HSPA5	0,166158499	0,361369926	0	N.A.	0
ANPEP	0,166136081	0,025918116	0	N.A.	0
RP1-102K2.8	0,166128737	0,212960844	0	N.A.	0
AC002117.1	0,16607388	0,094202907	0	N.A.	0
IRF9	0,166016946	0,341945498	0	N.A.	0
AEN	0,165984444	-0,053904813	0	N.A.	0
ARHGEF2	0,165941246	0,311226938	0	N.A.	0
ZBTB1	0,165936035	0,037235071	0	N.A.	0
RP11-147L13	0,165931454	0,150892046	0	N.A.	0
CTD-2026G6.	0,165882316	-0,302480243	0	N.A.	0
PHF23	0,165823707	-0,102035017	0	N.A.	0
HYI-AS1	0,16560372	0,040179849	0	N.A.	0
TMEM50A	0,165595228	0,403623906	0	N.A.	0
PTPN1	0,16551046	-0,081296364	0	N.A.	0
MARVELD3	0,165423277	0,294174153	0	N.A.	0
RP11-626P14	0,165388004	0,218304903	0	N.A.	0
MBTPS1	0,165360068	0,271489942	0	N.A.	0
TMEM81	0,16535411	-0,174607198	0	N.A.	0
TBC1D10A	0,16534681	0,085734238	0	N.A.	0
KIAA1462	0,165065651	-0,036353573	0	N.A.	0
TDP2	0,165014047	0,212863187	0	N.A.	0
SLAIN2	0,164968909	-0,00541151	0	N.A.	0
RP11-71L14.4	0,16490645	-0,068127139	0	N.A.	0

ZER1	0,164797564	0,180719721	0	N.A.	0
FOXJ3	0,164662698	0,139215181	0	N.A.	0
CH17-189H2(	0,164576153	0,323132852	0	N.A.	0
NR4A1	0,164500947	0,157899307	0	N.A.	0
PCDHGA7	0,164419829	0,022309811	0	N.A.	0
RP11-556E13	0,164419763	-0,164323771	0	N.A.	0
PMM1	0,164394525	0,200064199	0	N.A.	0
SYNRG	0,164365769	0,223974338	0	N.A.	0
LL22NC03-86	0,164315139	0,17641444	0	N.A.	0
MTCL1	0,164252404	0,245658862	0	N.A.	0
CHIT1	0,164167458		0	N.A.	0
RP11-349K16	0,164082617	-0,311815668	0	N.A.	0
RGS6	0,164046094	0,193557538	0	N.A.	0
PDK1	0,163926519	0,120196369	0	N.A.	0
ABCA13	0,163834051	-0,104640948	0	N.A.	0
GJB5	0,163833734	0,570084996	0	N.A.	0
GLIS3	0,163736915	0,128646713	0	N.A.	0
KIF21B	0,163728838	-0,116574125	0	N.A.	0
LYZ	0,163715192	-0,055923086	0	N.A.	0
RSPO3	0,163702609	-0,291588186	0	N.A.	0
RP11-304F15	0,163676213	-0,089438812	0	N.A.	0
SSBP2	0,163661851	0,034952091	0	N.A.	0
CH17-118O6	0,163656118	-0,379527016	0	N.A.	0
LINC01504	0,163640108	0,215004635	0	N.A.	0
TMEM117	0,163617509	0,084000461	0	N.A.	0
ALPK1	0,163576983	0,175982627	0	N.A.	0
LHFP	0,16356055	0,041316987	0	N.A.	0
CD274	0,163541413	0,096102586	0	N.A.	0
CPD	0,16339802	0,248701675	0	N.A.	0
MYO18A	0,163393717	0,367637312	0	N.A.	0
MAP3K9	0,163335606	0,426928898	0	N.A.	0
RP11-432J24	0,163324911	-0,012097956	0	N.A.	0
DYNC1LI2	0,163322438	0,31079747	0	N.A.	0

HLA-DQB1	0,16328116	-0,349395019	0	N.A.	0
LIMK1	0,163181722	0,202824944	0	N.A.	0
RP11-10A14.	0,163152977	0,021537286	0	N.A.	0
CERCAM	0,163091791	0,119174547	0	N.A.	0
HIVEP1	0,163069875	0,009177076	0	N.A.	0
RGL2	0,163067713	-0,223118155	0	N.A.	0
CDKL5	0,163052666	0,019952678	0	N.A.	0
HEATR5A	0,162972481	0,362312597	0	N.A.	0
RP11-362F19	0,162907304		0	N.A.	0
RP5-837J1.6	0,162875228	0,097564043	0	N.A.	0
SCAF1	0,162858813	0,420014888	0	N.A.	0
POR	0,162759424	0,430357142	0	N.A.	0
DERL2	0,162734323	0,259789423	0	N.A.	0
RP11-525K10	0,162700006		0	N.A.	0
RP11-598F7.	0,162677417	-0,122385768	0	N.A.	0
AMICA1	0,162658117	-0,290929694	0	N.A.	0
TMEM189-U	0,162632587		0	N.A.	0
RNF181	0,162609043	0,031986017	0	N.A.	0
RAG1	0,162593276	-0,102953856	0	N.A.	0
STX5	0,162577116	0,177463524	0	N.A.	0
FBXL2	0,16257681	0,016674774	0	N.A.	0
GP6	0,162487324	0,134475965	0	N.A.	0
ZFPM2	0,162479869		0	N.A.	0
NUFIP2	0,162479568	0,342287797	0	N.A.	0
CA12	0,162341061	0,39200135	0	N.A.	0
RENBP	0,162324277	0,396502185	0	N.A.	0
MAPK8IP3	0,162303728	0,110062357	0	N.A.	0
MED14	0,16218721	0,206828868	0	N.A.	0
CLEC5A	0,162176963	0,077707051	0	N.A.	0
SLC9A7	0,162112709	0,007952415	0	N.A.	0
ZBED6	0,16209413	0,105237491	0	N.A.	0
MARCH6	0,162093963	0,582466934	0	N.A.	0
BEST4	0,162056436	-0,266248544	0	N.A.	0

RP11-626H12	0,162001285	0,084067172	0	N.A.	0
HN1	0,161980344	0,158195888	0	N.A.	0
LINC01510	0,16193496		0	N.A.	0
FBXL17	0,161873154	-0,003077181	0	N.A.	0
PFKFB4	0,161772536	0,107673368	0	N.A.	0
RP11-755F10	0,16173201	-0,017509888	0	N.A.	0
RP11-390K5.	0,161707313	0,107684654	0	N.A.	0
RP11-219B4.	0,161698545		0	N.A.	0
IL23A	0,161696833	0,569652929	0	N.A.	0
GPATCH2L	0,161644299	0,308265476	0	N.A.	0
SH2B3	0,161598306	-0,164888574	0	N.A.	0
ICAM4	0,161571686	0,16611037	0	N.A.	0
HMBOX1	0,161442347	0,153524581	0	N.A.	0
TP53BP2	0,161391128	0,334808908	0	N.A.	0
RP11-420A23	0,1613892	0,160947588	0	N.A.	0
TRIP4	0,161369759	0,167447181	0	N.A.	0
BIN2	0,161360984	-0,341251827	0	N.A.	0
STAT1	0,161320898	0,15270196	0	N.A.	0
MREG	0,161290009	-0,003863291	0	N.A.	0
RP11-280G9.	0,161238312		0	N.A.	0
VAV2	0,161166586	0,322037759	0	N.A.	0
USH1C	0,161159018	0,471854873	0	N.A.	0
TMEM191B	0,161151889	0,190177434	0	N.A.	0
CBL	0,161149298	0,154269609	0	N.A.	0
RP11-42O15.	0,161051762	0,149746045	0	N.A.	0
MT-CO1	0,160934012	0,378861578	0	N.A.	0
ZNF385D	0,160858989	0,037532155	0	N.A.	0
SPRYD3	0,160841761	-0,09309476	0	N.A.	0
GMFB	0,160829346	0,001153843	0	N.A.	0
GIMAP5	0,160667021		0	N.A.	0
BRD1	0,16060248	0,045428416	0	N.A.	0
FRAS1	0,16058949	0,307303532	0	N.A.	0
SPATA12	0,160576489	0,002807628	0	N.A.	0

PPBP	0,160434173	0,377276014	0	N.A.	0
IL18R1	0,160367221	0,018993421	0	N.A.	0
HRK	0,160359272	-0,025111479	0	N.A.	0
SLC37A3	0,160270133	0,304004026	0	N.A.	0
KSR1	0,160153229	0,130028881	0	N.A.	0
MAN2C1	0,160088188	0,200138649	0	N.A.	0
GGA1	0,160048821	0,392715988	0	N.A.	0
NIPBL	0,159960403	0,368446975	0	N.A.	0
RAB40C	0,159948205	0,270186767	0	N.A.	0
RASA4B	0,159937315	-0,025106121	0	N.A.	0
CHIC1	0,159840645	0,024915453	0	N.A.	0
RP11-134L10	0,159664013	0,006909464	0	N.A.	0
TRPM7	0,159509658	0,136308318	0	N.A.	0
ERCC1	0,15950248	0,001278426	0	N.A.	0
SMCR5	0,159469708	0,018017736	0	N.A.	0
SHROOM4	0,159463915	0,14338689	0	N.A.	0
YTHDF1	0,159343654	0,289845732	0	N.A.	0
RGL1	0,159316948	0,197396939	0	N.A.	0
IHH	0,159286513	0,077011714	0	N.A.	0
ACBD5	0,159253522	0,393875258	0	N.A.	0
CTSL	0,159220125	0,239956502	0	N.A.	0
KDM2A	0,159195364	0,486194667	0	N.A.	0
IGFL2	0,159177818	-0,067799085	0	N.A.	0
RAB3IP	0,158980996	0,226289117	0	N.A.	0
PTPRA	0,158956652	0,241911172	0	N.A.	0
LMF2	0,158945098	0,576152813	0	N.A.	0
GOLGA8B	0,158867003	0,235300453	0	N.A.	0
TRIQK	0,158856925	-0,128245745	0	N.A.	0
EPSTI1	0,158751271	0,23800052	0	N.A.	0
KLF5	0,158733228	0,487084399	0	N.A.	0
CTD-2105E13	0,158655886		0	N.A.	0
CIPC	0,15863439	0,077736795	0	N.A.	0
GRID1	0,158600822		0	N.A.	0

AP2A1	0,158518599	0,234634118	0	N.A.	0
CTIF	0,158499576	0,124026156	0	N.A.	0
STRIP1	0,158249081	0,103437995	0	N.A.	0
COL5A3	0,158224643	0,011654102	0	N.A.	0
WDR11	0,158199158	0,322116077	0	N.A.	0
FGL2	0,158130876	0,158162013	0	N.A.	0
MYADM	0,158098063	0,407622418	0	N.A.	0
NNMT	0,158083761	-0,007824433	0	N.A.	0
MFSD9	0,158010359	0,109350256	0	N.A.	0
PLEKHH2	0,157985693	0,250578443	0	N.A.	0
UBE4A	0,157976563	0,350145476	0	N.A.	0
EEF2K	0,15795797	0,019473447	0	N.A.	0
CCR1	0,157953207	-0,099000061	0	N.A.	0
MKRN2OS	0,157932709	0,127032331	0	N.A.	0
IGHV2-5	0,157851903	-0,24672132	0	N.A.	0
SLC5A9	0,157849575	0,061027552	0	N.A.	0
TNRC6C	0,157844236	0,013435039	0	N.A.	0
UBE2Q1	0,157760666	0,351828224	0	N.A.	0
RP11-696F12	0,15765588	0,067041463	0	N.A.	0
NBPF12	0,157618456	0,323966463	0	N.A.	0
C6orf89	0,157483715	0,211458526	0	N.A.	0
NEDD4	0,157476729	0,187216867	0	N.A.	0
PPCS	0,157466917	-0,009153113	0	N.A.	0
RP11-434B12	0,157410658	0,05072519	0	N.A.	0
BLZF1	0,157388964	0,330813325	0	N.A.	0
OSR2	0,157380374	-0,03082163	0	N.A.	0
TMEM246	0,157353166	0,148944656	0	N.A.	0
VLDLR	0,157249567	0,004189763	0	N.A.	0
SDC1	0,15706344	0,556678269	0	N.A.	0
HSD17B3	0,157026213	0,150570298	0	N.A.	0
LDLRAD1	0,156935934	-0,072914752	0	N.A.	0
RHCE	0,156929112	0,21153329	0	N.A.	0
RP11-805J14	0,156902513	-0,2408747	0	N.A.	0

CILP2	0,15681804		0	N.A.	0
CTA-363E6.7	0,156812751		0	N.A.	0
UBXN10	0,156799231	0,184736979	0	N.A.	0
ATG16L1	0,156592777	0,289468181	0	N.A.	0
CTA-212A2.3	0,156553963	0,223339561	0	N.A.	0
RELL1	0,156537939	0,527431496	0	N.A.	0
BCL2L2	0,156530005	0,282956009	0	N.A.	0
RP3-510D11.	0,156404266	0,186379191	0	N.A.	0
RP11-154J22	0,156378578	0,003213942	0	N.A.	0
VANGL1	0,156327341	0,288362551	0	N.A.	0
RP11-408A13	0,156301752	0,303253642	0	N.A.	0
ZNF664	0,156227428	0,147472679	0	N.A.	0
RP11-98D18.	0,156151583	-0,347142557	0	N.A.	0
ZNF417	0,156111393	0,439053941	0	N.A.	0
CUL7	0,156059371	0,068104355	0	N.A.	0
NCOA4	0,156016397	0,339114449	0	N.A.	0
LIPG	0,155999952	0,116690202	0	N.A.	0
RP11-801F7.	0,155976179	0,120546822	0	N.A.	0
RP11-544A12	0,155911293	-0,04916793	0	N.A.	0
DMXL2	0,15585568	0,141848243	0	N.A.	0
AC010883.5	0,15582673	0,207543888	0	N.A.	0
SYCE1L	0,155800566	0,008414246	0	N.A.	0
TTC9B	0,155798708	-0,05064371	0	N.A.	0
LINC01022	0,155758519	0,116475093	0	N.A.	0
LINC00973	0,155673942	0,015882872	0	N.A.	0
GALR2	0,155638741	-0,001213609	0	N.A.	0
IL1R2	0,15563015	0,217638066	0	N.A.	0
C1orf132	0,155616721	0,028969508	0	N.A.	0
CASKIN2	0,155594866	0,313495974	0	N.A.	0
NCOA6	0,155589918	0,486577066	0	N.A.	0
CDS2	0,155554134	0,072645176	0	N.A.	0
CTB-129P6.4	0,155531291	0,179576521	0	N.A.	0
SERTAD4-AS1	0,155425715	0,101131571	0	N.A.	0

RP11-16E12.	0,155420115	-0,064893133	0	N.A.	0
PKD1	0,155396081	0,275645027	0	N.A.	0
PCNXL3	0,155342257	0,30063558	0	N.A.	0
ZC3H12C	0,155299629	0,016841611	0	N.A.	0
FAM83B	0,15517908	0,178014283	0	N.A.	0
BAHD1	0,15516255	-0,018634258	0	N.A.	0
GPR82	0,155097656	0,062563516	0	N.A.	0
RP11-54C4.3	0,155057681	-0,172935788	0	N.A.	0
FAM174A	0,155046366	-0,090961985	0	N.A.	0
CCDC93	0,154966531	0,387904699	0	N.A.	0
MOB1B	0,154921702	0,337432767	0	N.A.	0
SOD3	0,15489182	0,191119096	0	N.A.	0
PLEKHM3	0,154851915	-0,004042726	0	N.A.	0
EOGT	0,154816397	-0,075980826	0	N.A.	0
OLFM1	0,154752721	-0,036169752	0	N.A.	0
RP5-1059L7.1	0,154730604	0,314152213	0	N.A.	0
PRODH	0,154727233	0,065360439	0	N.A.	0
HEBP1	0,154662021	0,185548239	0	N.A.	0
RP11-138A9.	0,154610817	0,441527335	0	N.A.	0
RP11-355B11	0,154570925	-0,067320855	0	N.A.	0
HOXD11	0,154562089	0,235882336	0	N.A.	0
ANKRD62	0,154516259	-0,110334218	0	N.A.	0
SLCO4A1-AS1	0,154474259	0,094489274	0	N.A.	0
SLC14A1	0,154468795	-0,288384079	0	N.A.	0
KRT13	0,154437221	0,124386989	0	N.A.	0
PRKG2	0,154420116	-0,020241994	0	N.A.	0
DDI2	0,154315914	0,236187531	0	N.A.	0
SNTB1	0,154237694	-0,135673079	0	N.A.	0
PLXNC1	0,154206711	0,006289129	0	N.A.	0
PLEKHH3	0,15417644	0,148222199	0	N.A.	0
TSPAN17	0,154124663	-0,2139024	0	N.A.	0
RP11-338N10	0,154121056		0	N.A.	0
DOC2B	0,154090089	-0,177174059	0	N.A.	0

FUT4	0,154085947	0,318776842	0	N.A.	0
C2orf61	0,154067115	0,154577546	0	N.A.	0
RP11-244F12	0,154037099	0,117620321	0	N.A.	0
C1orf21	0,154023268	0,128821625	0	N.A.	0
CSAD	0,153955122	0,146562104	0	N.A.	0
INPP4A	0,153880221	0,25613314	0	N.A.	0
DDIT4	0,153804183	0,625657038	0	N.A.	0
VPS37C	0,153750672	0,022085114	0	N.A.	0
ARMCX3	0,153739046	0,122531627	0	N.A.	0
NPNT	0,153726863	0,303434738	0	N.A.	0
MMP23B	0,153584375	0,133499161	0	N.A.	0
RNF10	0,153555372	-0,103729872	0	N.A.	0
GCNT4	0,153547269	0,209563104	0	N.A.	0
RP11-146N18	0,153484836	-0,203102141	0	N.A.	0
RP11-1072A3	0,153440474		0	N.A.	0
TMPRSS5	0,153404194	-0,120972249	0	N.A.	0
CTD-2515O10	0,15339906	0,034245969	0	N.A.	0
PCDHGB5	0,15335842		0	N.A.	0
ZNF440	0,153337719	0,264145232	0	N.A.	0
DHX58	0,153330981	0,240867977	0	N.A.	0
RP11-477N3.	0,153327634		0	N.A.	0
PBXIP1	0,153305169	0,293559132	0	N.A.	0
RP11-95P13.	0,153231179	0,196472035	0	N.A.	0
PHYHIP	0,153168766	0,073717546	0	N.A.	0
RP11-20I23.1	0,153139438	0,330927937	0	N.A.	0
LGALS8	0,153086358	0,460762417	0	N.A.	0
PLEK2	0,153056603	0,252814437	0	N.A.	0
SLC30A7	0,153027331	0,370392291	0	N.A.	0
ITGA11	0,15301331	-0,089959021	0	N.A.	0
AC003092.1	0,152990019		0	N.A.	0
RP11-279O9.	0,152985092	0,25141587	0	N.A.	0
FKBP14	0,152970819	-0,055327097	0	N.A.	0
CUL3	0,152888264	0,352572935	0	N.A.	0

MT-ATP8	0,152873397	-0,060050669	0	N.A.	0
SPOCD1	0,152838492	0,326095397	0	N.A.	0
CTD-2260A1	0,152833983	0,380661066	0	N.A.	0
NIPAL2	0,152722606	0,162092638	0	N.A.	0
KRT83	0,152683933	-0,014083432	0	N.A.	0
RP11-794G2	0,152644598	0,177042325	0	N.A.	0
TPST1	0,152552738	-0,201220768	0	N.A.	0
RP5-905G11	0,152464654		0	N.A.	0
PPM1M	0,152371834	-0,131441125	0	N.A.	0
RP11-90D4.3	0,152281721	-0,090979408	0	N.A.	0
PCLO	0,152152946	0,327614521	0	N.A.	0
TOX4	0,152146113	0,184146953	0	N.A.	0
GRID2IP	0,152118468	0,266916874	0	N.A.	0
RP11-799B1	0,152110718	-0,011688522	0	N.A.	0
PNMA1	0,152083938	0,418967138	0	N.A.	0
HCG27	0,152039846	0,369263066	0	N.A.	0
FOXK1	0,152024694	0,029822252	0	N.A.	0
TBC1D3L	0,152008468	0,234777592	0	N.A.	0
CMTM3	0,152006665	-0,085324255	0	N.A.	0
MPEG1	0,151999847	-0,078587418	0	N.A.	0
TAL1	0,151956403		0	N.A.	0
MIER1	0,151939776	0,004695039	0	N.A.	0
SGK2	0,151806398	-0,197206115	0	N.A.	0
ZDHHC20	0,151788372	0,484015114	0	N.A.	0
EXOC8	0,151774197	-0,159119031	0	N.A.	0
ANKRD28	0,151738794	-0,356015661	0	N.A.	0
TRAFD1	0,151676942	0,495108135	0	N.A.	0
LAMP5	0,151620346	-0,299971794	0	N.A.	0
MECP2	0,151495636	0,178379617	0	N.A.	0
CTC-367J11.	0,151441847	-0,089480548	0	N.A.	0
TCTEX1D4	0,151403385	0,20024315	0	N.A.	0
LGALS9B	0,151326619	0,145903563	0	N.A.	0
FAM150A	0,151297609	-0,133998447	0	N.A.	0

GATM	0,151295617	-0,075965624	0	N.A.	0
FBXO6	0,151270407	0,123242759	0	N.A.	0
RP11-479O17	0,151255813		0	N.A.	0
RASGEF1B	0,151233062	-0,144949963	0	N.A.	0
FOXP3	0,151223653	-0,075499705	0	N.A.	0
RP11-254F7.1	0,151182662	0,086920805	0	N.A.	0
RP11-41O4.1	0,151029237	0,058488604	0	N.A.	0
CRIPAK	0,151027574	0,182492172	0	N.A.	0
EFEMP2	0,150991564	0,079854669	0	N.A.	0
COG5	0,150986556	0,460767935	0	N.A.	0
CHMP6	0,150959112	0,139717541	0	N.A.	0
ZNF358	0,150906998	0,132298675	0	N.A.	0
RP11-326C3.	0,150879547		0	N.A.	0
SMAD4	0,150720242	0,167109686	0	N.A.	0
AC090616.2	0,150689296	0,204157	0	N.A.	0
UBQLN2	0,150667543	0,334553312	0	N.A.	0
SPTBN5	0,150650829	0,30431791	0	N.A.	0
C1orf198	0,150631056	-0,088170418	0	N.A.	0
MAPK1	0,15058093	0,249778272	0	N.A.	0
N4BP2L2	0,150558586	0,305142678	0	N.A.	0
LY6G6E	0,150539206	0,169383774	0	N.A.	0
PRR16	0,150486749		0	N.A.	0
CITED4	0,150393651	0,200807295	0	N.A.	0
RP11-214K3.	0,15037398	-0,327510683	0	N.A.	0
BNIP2	0,150316434	0,383225915	0	N.A.	0
ZNF726	0,150308018		0	N.A.	0
PRICKLE1	0,150234451		0	N.A.	0
ZNF213	0,150207458	-0,14308626	0	N.A.	0
ZNF80	0,150196399		0	N.A.	0
GINM1	0,150164436	0,228734134	0	N.A.	0
RP11-894P9.	0,1500181	0,09448708	0	N.A.	0
RP11-385D13	0,150003478	0,305019063	0	N.A.	0
RP11-840I19	0,149914251		0	N.A.	0

DMWD	0,149895871	0,211534555	0	N.A.	0
GPR39	0,149893884	0,256456804	0	N.A.	0
RP4-536B24.	0,149884201	0,351044819	0	N.A.	0
SLC2A12	0,149868551	0,332424835	0	N.A.	0
RNF167	0,149858805	0,194445249	0	N.A.	0
RP5-1142A6.	0,149858329		0	N.A.	0
RP11-536G4.	0,149828031		0	N.A.	0
APC	0,149790473	0,10199824	0	N.A.	0
RP11-378J18	0,14978996	-0,061096937	0	N.A.	0
OPHN1	0,149691382	0,173726849	0	N.A.	0
GCOM1	0,149684916	-0,052745406	0	N.A.	0
NCMAP	0,14967562	-0,171922024	0	N.A.	0
CRYM-AS1	0,149673204	0,063217381	0	N.A.	0
RP13-870H17	0,149631615	0,250255956	0	N.A.	0
OTUD4	0,149574249	0,239351813	0	N.A.	0
WDR44	0,14956773	0,104893032	0	N.A.	0
DHRS7	0,149514552	0,374425933	0	N.A.	0
AMMECR1L	0,149464055	0,072446131	0	N.A.	0
CCNY	0,149372409	-0,012132196	0	N.A.	0
ARL10	0,149309081	-0,141841847	0	N.A.	0
LINC00685	0,149218143	0,332158447	0	N.A.	0
ABHD15	0,149190427	0,272807104	0	N.A.	0
PCAT6	0,149044383	0,279327172	0	N.A.	0
CLK3	0,149023309	0,092031347	0	N.A.	0
RAB1B	0,148862332	0,205626652	0	N.A.	0
RBM33	0,148852219	0,422512125	0	N.A.	0
KIAA1614	0,148830409	-0,005132774	0	N.A.	0
PRKACA	0,148696917	0,051604381	0	N.A.	0
MKL1	0,148667526	0,018387624	0	N.A.	0
ASPRV1	0,148644555	0,216559638	0	N.A.	0
LINC00511	0,148630634	0,319625022	0	N.A.	0
GTDC1	0,148565235	-0,111975787	0	N.A.	0
RP1-117B12.	0,148500687	0,224993816	0	N.A.	0

HYPM	0,148440125		0	N.A.	0
SUPT6H	0,148403784	0,3164283	0	N.A.	0
IER5L	0,148385428	0,503172807	0	N.A.	0
KAZN	0,148351782	0,123820494	0	N.A.	0
KCTD5	0,148330283	0,319593296	0	N.A.	0
CPPED1	0,148318549	0,140705695	0	N.A.	0
FOSB	0,148304706	0,08909217	0	N.A.	0
GS1-124K5.1	0,148283127	-0,108709465	0	N.A.	0
MOB3A	0,148208551	-0,101916218	0	N.A.	0
TWSG1	0,148184025	-0,085597337	0	N.A.	0
LINC00346	0,148178178	0,322301188	0	N.A.	0
EDN3	0,148130642	0,310012812	0	N.A.	0
TBC1D25	0,148118933	-0,193714245	0	N.A.	0
UBR3	0,148083799	0,125844198	0	N.A.	0
LINC00162	0,14799232		0	N.A.	0
CRY1	0,147913542	0,055801528	0	N.A.	0
BRINP1	0,14790957	0,258296158	0	N.A.	0
EPG5	0,147903443	0,20630095	0	N.A.	0
RP11-661A12	0,147884299	-0,349072274	0	N.A.	0
MRVI1	0,147810476	-0,233934221	0	N.A.	0
UBQLNL	0,147790605	0,133991691	0	N.A.	0
SLC44A2	0,147767137	0,268296564	0	N.A.	0
PLCL2	0,147756948	-0,043095251	0	N.A.	0
WISP1	0,147749605	0,095880697	0	N.A.	0
PIK3CD	0,147746253	0,051059644	0	N.A.	0
RP11-402G3.	0,147736147		0	N.A.	0
RP11-329B9.	0,147682387	-0,11442218	0	N.A.	0
MED14OS	0,14762647	-0,043865692	0	N.A.	0
IRGQ	0,14757254	0,147259752	0	N.A.	0
SLC38A10	0,147511143	0,384135483	0	N.A.	0
SNX8	0,147437066	0,279097524	0	N.A.	0
TBX3	0,147349731	0,139295211	0	N.A.	0
ITM2B	0,147335672	0,257276661	0	N.A.	0

AHCYL1	0,147200182	0,305570518	0	N.A.	0
BCO1	0,147164001	0,384773012	0	N.A.	0
MXD4	0,147155791	0,017189651	0	N.A.	0
USP4	0,147152441	0,132395224	0	N.A.	0
WDR91	0,147103602	0,076149639	0	N.A.	0
ARHGEF7	0,147083623	0,364573208	0	N.A.	0
VPS37A	0,147045408	0,120729841	0	N.A.	0
FAM26F	0,14704215	-0,19639393	0	N.A.	0
SPTLC1	0,147031268	0,151740893	0	N.A.	0
ZNF568	0,147018346	0,199893333	0	N.A.	0
LACC1	0,146993113	0,091598638	0	N.A.	0
AP001631.9	0,146887154	0,048700154	0	N.A.	0
ATP6V0D1	0,146883712	0,234131926	0	N.A.	0
IYD	0,146879547	-0,163892806	0	N.A.	0
PDZD7	0,146871302	-0,265452709	0	N.A.	0
EIF1AD	0,146828614	0,277769464	0	N.A.	0
RP11-138A9.	0,146794622	-0,286991853	0	N.A.	0
NCF1	0,146783305	-0,037797695	0	N.A.	0
CASC8	0,146778639	0,029994848	0	N.A.	0
ADTRP	0,146766061	0,197045198	0	N.A.	0
FAM53C	0,146744408	-0,011811903	0	N.A.	0
USP31	0,146722503	0,113712559	0	N.A.	0
UMAD1	0,146719636	-0,113405811	0	N.A.	0
RP11-15H20.	0,146701357	0,077520768	0	N.A.	0
RP4-657D16.	0,146651432	0,481539684	0	N.A.	0
LGMN	0,146614249	0,350104011	0	N.A.	0
AC006273.4	0,146556966	0,063641639	0	N.A.	0
RP11-380J14	0,146546479	7,60501E-05	0	N.A.	0
RP11-473M2	0,146521124	0,168872852	0	N.A.	0
NOVA2	0,14651833	0,399332538	0	N.A.	0
MFSD10	0,146471238	0,326551322	0	N.A.	0
RP11-993B23	0,146463527		0	N.A.	0
ZNF564	0,146379165	0,01594993	0	N.A.	0

NRG4	0,146337686	0,042909635	0	N.A.	0
IKBKE	0,146325511	0,208742873	0	N.A.	0
RNF7	0,146298302	0,226216359	0	N.A.	0
RAB11B	0,146293811	-0,023766428	0	N.A.	0
GPER1	0,146279974	0,059440642	0	N.A.	0
RP11-534L20	0,146265361		0	N.A.	0
TOR1AIP1	0,146263105	0,059330583	0	N.A.	0
CDK9	0,146213762	0,152155899	0	N.A.	0
ROCK2	0,146192599	0,372621469	0	N.A.	0
ATP1A2	0,146180396		0	N.A.	0
MYLK	0,146168283	0,206601968	0	N.A.	0
SND1-IT1	0,146151715	0,34442711	0	N.A.	0
RP11-626G11	0,14614606	0,005572794	0	N.A.	0
MROH7	0,146017138	-0,060072278	0	N.A.	0
RP11-996F15	0,145956047	0,06640897	0	N.A.	0
ADRA2A	0,145942486	0,064752541	0	N.A.	0
RHBDD1	0,145884136	-0,175703211	0	N.A.	0
AC069513.4	0,145863749	-0,06235931	0	N.A.	0
WNT9A	0,145837947	0,072575711	0	N.A.	0
IKBKG	0,145833746	-0,017454992	0	N.A.	0
MSMB	0,145786673	0,145804909	0	N.A.	0
RP11-551L14	0,145752215	-0,203455361	0	N.A.	0
ZDHHC7	0,145674544	0,292216005	0	N.A.	0
OFD1	0,145630467	0,315537126	0	N.A.	0
PLXND1	0,145502689	-0,139563796	0	N.A.	0
EME2	0,14547873	0,422656998	0	N.A.	0
IGLV1-51	0,145368363	-0,018483903	0	N.A.	0
GOLGA4	0,14535089	0,347415121	0	N.A.	0
RP11-195F19	0,145206242	-0,046975342	0	N.A.	0
MYL12B	0,145195379	0,05206527	0	N.A.	0
THSD4	0,145078245	0,359445524	0	N.A.	0
RNF144B	0,145058499	-0,119804757	0	N.A.	0
DTNA	0,14502863	0,007449538	0	N.A.	0

GREB1L	0,145004843	0,157632266	0	N.A.	0
FGD5	0,144957944	0,247167588	0	N.A.	0
NT5DC4	0,144893815	0,441901986	0	N.A.	0
IQSEC1	0,144837593	0,049377672	0	N.A.	0
THEMIS2	0,144760616	0,354915055	0	N.A.	0
TRAF1	0,1447524	0,087575885	0	N.A.	0
TMEM179B	0,144722144	0,315788254	0	N.A.	0
IGF2BP3	0,144707072	0,005585439	0	N.A.	0
CALB1	0,144700643	0,110725561	0	N.A.	0
LINC01420	0,144613339	-0,060750519	0	N.A.	0
MDM4	0,144581875	0,071741252	0	N.A.	0
ATG4A	0,144564981	-0,022080988	0	N.A.	0
USP24	0,144549773	0,303764015	0	N.A.	0
AC129778.2	0,144448528		0	N.A.	0
AC010967.2	0,14441364	0,080185685	0	N.A.	0
RP11-731C17	0,144337476	0,11037976	0	N.A.	0
LYSMD3	0,144324138	-0,170270457	0	N.A.	0
SSH1	0,144324135	9,29206E-05	0	N.A.	0
RP11-573D15	0,144303105	0,113759574	0	N.A.	0
TMEM79	0,144278355	-0,143036626	0	N.A.	0
FRYL	0,144269375	0,171245339	0	N.A.	0
SH3PXD2B	0,14426028	0,126644031	0	N.A.	0
GMEB1	0,144237039	0,226052445	0	N.A.	0
RASA4	0,144144366	0,235545152	0	N.A.	0
RNF139	0,144134128	0,393190455	0	N.A.	0
B3GALT6	0,144079995	0,019989847	0	N.A.	0
PGM3	0,144028392	0,103758028	0	N.A.	0
RP11-400F19	0,144023949	0,223444395	0	N.A.	0
FRMD1	0,14384091	0,242041358	0	N.A.	0
RP11-297D21	0,143765691	-0,278105327	0	N.A.	0
TMEM151A	0,143744918	0,102038827	0	N.A.	0
GTF2B	0,143738039	0,308696242	0	N.A.	0
RP11-90M2.5	0,143730444	0,002287068	0	N.A.	0

GOLPH3L	0,143684366	0,065232602	0	N.A.	0
TMEM9B	0,143662999	0,376884629	0	N.A.	0
LINC00894	0,143564612	0,017831713	0	N.A.	0
C18orf65	0,143526865	-0,17147992	0	N.A.	0
SHBG	0,143525891		0	N.A.	0
ATF7IP	0,143523632	0,077136343	0	N.A.	0
LINC00460	0,143511498	0,119498501	0	N.A.	0
TMEM154	0,143456291	0,221558218	0	N.A.	0
PTPN9	0,143455517	0,105097979	0	N.A.	0
MTRNR2L10	0,143445919	0,362470726	0	N.A.	0
RUNX3	0,143365524	0,247100723	0	N.A.	0
LONRF1	0,143335429	-0,346437984	0	N.A.	0
LURAP1L-AS1	0,143297081	0,109393853	0	N.A.	0
ENTPD5	0,143284332	0,034695902	0	N.A.	0
SDC3	0,143217346	0,231931008	0	N.A.	0
ARL14EPL	0,143214579		0	N.A.	0
CDC14A	0,143176312	0,250457521	0	N.A.	0
TRAPPC10	0,143157551	0,195677305	0	N.A.	0
F2RL3	0,143133307	-0,011522135	0	N.A.	0
HGD	0,143095892	0,064590994	0	N.A.	0
ZBTB22	0,143091339	0,056374562	0	N.A.	0
FAM73B	0,143075078	0,00645156	0	N.A.	0
MMP2	0,14301972	0,068739587	0	N.A.	0
NR6A1	0,142996581	0,201791279	0	N.A.	0
GRAMD1C	0,142992948	-0,133358175	0	N.A.	0
USP30-AS1	0,142948505	0,173614687	0	N.A.	0
BCR	0,142788751	0,183362011	0	N.A.	0
ZNF334	0,142788704	-0,164666014	0	N.A.	0
KIAA1549L	0,142739428	-0,02841611	0	N.A.	0
SRXN1	0,142737709		0	N.A.	0
PPM1B	0,142605741	0,009058832	0	N.A.	0
ZNF225	0,142556624	-0,076553927	0	N.A.	0
PRSS16	0,14252937	-0,202918159	0	N.A.	0

TINCR	0,142415197	0,099497385	0	N.A.	0
MMP25-AS1	0,142317066	-0,120019333	0	N.A.	0
NXF2B	0,142294562	0,028975782	0	N.A.	0
RP5-894A10.	0,14226476	-0,042587486	0	N.A.	0
LILRA2	0,142264408	-0,163852913	0	N.A.	0
BRAF	0,142249958	0,142841091	0	N.A.	0
MANSC4	0,142239477		0	N.A.	0
SLC5A2	0,142211925	-0,264068196	0	N.A.	0
RP11-357P18	0,142205448		0	N.A.	0
GLTSCR1L	0,142157869	0,050759184	0	N.A.	0
TERF2	0,142119251	0,13660766	0	N.A.	0
RP11-207C16	0,142091232	0,126139807	0	N.A.	0
C5orf51	0,142084154	-0,132363213	0	N.A.	0
RAVER1	0,141997928	-0,231658115	0	N.A.	0
FAM3A	0,14191286	0,234647943	0	N.A.	0
KCNJ6	0,141865952		0	N.A.	0
CCDC17	0,141823626	0,076450265	0	N.A.	0
TRIP11	0,141784876	0,261897172	0	N.A.	0
COL1A2	0,14177181	-0,129546195	0	N.A.	0
FEZF1-AS1	0,141672773	0,240569416	0	N.A.	0
AC009950.2	0,141664575		0	N.A.	0
ZNF600	0,141628978	0,509042225	0	N.A.	0
PARK2	0,14162141	-0,011702793	0	N.A.	0
FAM213B	0,141596414	0,480502316	0	N.A.	0
AIM2	0,141584082	0,161711827	0	N.A.	0
CTD-2129N1.	0,141561244	0,124256653	0	N.A.	0
FAM83F	0,141542424	0,315446023	0	N.A.	0
RP11-1149O2	0,141521336		0	N.A.	0
INSL4	0,141482176	0,14805089	0	N.A.	0
CAPN1	0,141476299	0,38732738	0	N.A.	0
RP11-158H5.	0,1414381	0,250651532	0	N.A.	0
ANKRD22	0,141361748	0,049384378	0	N.A.	0
PTPN13	0,141352241	0,129005636	0	N.A.	0

RP11-245J9.6	0,141156059	0,045165502	0	N.A.	0
BACH1-IT2	0,141127398	0,050767408	0	N.A.	0
C8orf4	0,141084543	0,289714432	0	N.A.	0
MUC5AC	0,141080577	0,178713402	0	N.A.	0
RP11-92G12.	0,141026993	0,077317239	0	N.A.	0
FAM188A	0,141021901	0,170971352	0	N.A.	0
RRNAD1	0,140994655	0,199226772	0	N.A.	0
NFATC2IP	0,140823443	0,235752822	0	N.A.	0
CAPS	0,140806754	0,249441688	0	N.A.	0
SIK1	0,140797683	0,131677337	0	N.A.	0
VTN	0,140775567		0	N.A.	0
MYOM1	0,140695304	0,047141735	0	N.A.	0
AMY2B	0,140658052	0,363160013	0	N.A.	0
ZMAT3	0,140644493	-0,090042112	0	N.A.	0
RP3-467K16.	0,140640239	0,225124368	0	N.A.	0
TSHZ1	0,140635197	0,09934727	0	N.A.	0
TMEM200A	0,140619799	0,043804012	0	N.A.	0
LTBR	0,140552368	0,415939663	0	N.A.	0
KIF3C	0,140543808	0,226823887	0	N.A.	0
SOWAHB	0,140528525	0,151627405	0	N.A.	0
HLA-DOA	0,140518594	-0,204561321	0	N.A.	0
PLA2G2A	0,14051548	-0,118463729	0	N.A.	0
CSGALNACT2	0,14050984	0,158145854	0	N.A.	0
ATXN7L1	0,140464855	0,07081366	0	N.A.	0
ANKRD35	0,140460116		0	N.A.	0
PDE6B	0,140432249		0	N.A.	0
TIAF1	0,14042641	-0,228636795	0	N.A.	0
CCND1	0,140416792	0,718624931	0	N.A.	0
C7orf57	0,140350642	-0,247890397	0	N.A.	0
CEACAM3	0,140289911	-0,086957466	0	N.A.	0
AP000350.5	0,140271413	0,036450651	0	N.A.	0
LINC01483	0,140267844	0,112525999	0	N.A.	0
TREM1	0,140257834	-0,180156677	0	N.A.	0

CST7	0,140254283	-0,036331244	0	N.A.	0
LRRN1	0,140244871	0,120019945	0	N.A.	0
EFNA3	0,140229229	0,381438736	0	N.A.	0
RELT	0,140216659	0,280039378	0	N.A.	0
CD163L1	0,140139919	-0,057083427	0	N.A.	0
BSDC1	0,140116548	0,496052548	0	N.A.	0
DENND4A	0,140089614	-0,235533707	0	N.A.	0
HOXB7	0,140012805	0,47871969	0	N.A.	0
DCAF5	0,13997444	0,210479302	0	N.A.	0
SORT1	0,139926853	0,232602282	0	N.A.	0
GAPVD1	0,139926731	0,23805789	0	N.A.	0
CA4	0,139902055	0,212951812	0	N.A.	0
CTC-444N24.	0,13982959	0,198921128	0	N.A.	0
OXR1	0,139813936	-0,099467193	0	N.A.	0
TRIM16	0,139812019	0,144284439	0	N.A.	0
ZNF791	0,139784613	0,241043123	0	N.A.	0
RP11-317L10	0,139766113	-0,023234573	0	N.A.	0
EDA	0,139698225	0,004585049	0	N.A.	0
LINC00467	0,139608262	0,080913589	0	N.A.	0
CRIP2	0,139605536	0,381109679	0	N.A.	0
CHMP2A	0,139585317	0,307613013	0	N.A.	0
ACKR2	0,139526869	-0,121607728	0	N.A.	0
SP2	0,139514516	-0,040097628	0	N.A.	0
CLEC16A	0,139487135	0,25857988	0	N.A.	0
BX470102.3	0,1394349	0,135604836	0	N.A.	0
CLEC7A	0,139434871	-0,314523478	0	N.A.	0
RP5-875H18.	0,139388938	0,39018111	0	N.A.	0
ASCC2	0,13934889	0,063100902	0	N.A.	0
NBAS	0,139321238	0,23481328	0	N.A.	0
ANXA10	0,139317527	0,156525475	0	N.A.	0
ZNF341	0,139292974	0,07264923	0	N.A.	0
AP000704.5	0,139275704	-0,322801588	0	N.A.	0
RP11-195B17	0,139262794	-0,074919772	0	N.A.	0

OLFML2A	0,139248976	0,304134452	0	N.A.	0
GPRIN1	0,139207627	-0,060587586	0	N.A.	0
RP11-305L7.(	0,139193772	-8,40519E-05	0	N.A.	0
PLOD1	0,139136106	0,025620079	0	N.A.	0
EFCAB5	0,139097015	-0,196315865	0	N.A.	0
TTC21A	0,139056564	0,22522595	0	N.A.	0
IGLV1-40	0,13901411	-0,26939949	0	N.A.	0
RP11-211G23	0,138996236	0,136346247	0	N.A.	0
TMEM74	0,138987563		0	N.A.	0
RP11-374M1	0,138964306	-0,150981849	0	N.A.	0
ZC3H3	0,138923022	0,219296276	0	N.A.	0
RP11-642C5.	0,138881051		0	N.A.	0
DCBLD1	0,138870914	0,249179063	0	N.A.	0
ZIC5	0,138863014	0,076839887	0	N.A.	0
SNIP1	0,138839021	0,181824762	0	N.A.	0
LSAMP-AS1	0,138791566	0,270755196	0	N.A.	0
CPE	0,138755358	0,218231869	0	N.A.	0
RAB11FIP4	0,13862949	0,499202024	0	N.A.	0
CREBL2	0,138618981	-0,184977525	0	N.A.	0
ARHGEF10L	0,138605165	0,199825817	0	N.A.	0
PEX26	0,138588726	0,290911538	0	N.A.	0
CD72	0,138573312	0,050443422	0	N.A.	0
ENTPD3	0,138509142	-0,092975943	0	N.A.	0
CDC34	0,138473405	0,354784857	0	N.A.	0
AC069277.2	0,138387298	-0,190999739	0	N.A.	0
MTMR14	0,138381939	-0,141391798	0	N.A.	0
AC009495.2	0,138331982	0,03372306	0	N.A.	0
SLC45A3	0,138316779	0,086680011	0	N.A.	0
AC005281.1	0,138307602	0,210394408	0	N.A.	0
POU2F3	0,138099744	0,169524612	0	N.A.	0
ZNF316	0,138044555	0,255572014	0	N.A.	0
LRRTM1	0,138014825	-0,089184637	0	N.A.	0
RP11-514P8.	0,137932992	-0,075145808	0	N.A.	0

CXCL14	0,137910966	0,022344072	0	N.A.	0
NOMO2	0,137882949	-0,006774848	0	N.A.	0
NEIL1	0,137843568	0,047997832	0	N.A.	0
TNIK	0,137768496	0,099717694	0	N.A.	0
ATG4D	0,137765658	0,092855906	0	N.A.	0
EXOC3L1	0,137731565	0,268503748	0	N.A.	0
HELZ	0,137702693	0,277373598	0	N.A.	0
AC144831.3	0,137660114	0,082951627	0	N.A.	0
RP11-902B17	0,137630377	-0,000563924	0	N.A.	0
SLC25A18	0,137630248	0,140134487	0	N.A.	0
PRKCH	0,137625196	0,015717356	0	N.A.	0
CDK2AP2	0,137618705	0,059734421	0	N.A.	0
RP11-285E9.	0,137609764		0	N.A.	0
CH17-140K24	0,137554342	0,025324605	0	N.A.	0
LINC-PINT	0,137545665	-0,304665662	0	N.A.	0
CDKN2AIP	0,137487078	0,23449144	0	N.A.	0
SAMD12	0,13744879	0,178355206	0	N.A.	0
MARCO	0,137391402	-0,233741249	0	N.A.	0
RP11-326A19	0,137350585	0,160851079	0	N.A.	0
C1RL	0,137341449	0,067182278	0	N.A.	0
AC011298.2	0,137320888	-0,038212489	0	N.A.	0
ARVCF	0,137291096	0,174050597	0	N.A.	0
RWDD2A	0,137273629	0,188309513	0	N.A.	0
GTF2IRD2B	0,137240098	0,081718525	0	N.A.	0
LZTS3	0,137208005	-0,006990862	0	N.A.	0
RBM5	0,137183496	0,222650554	0	N.A.	0
MYRFL	0,13714748	0,270496272	0	N.A.	0
PCSK4	0,137067335	0,037415393	0	N.A.	0
RAI1	0,137048439	0,22389665	0	N.A.	0
AC019117.2	0,137003897	0,087864818	0	N.A.	0
STK35	0,136974612	-0,124287741	0	N.A.	0
CTB-133G6.2	0,136907972	-0,077066879	0	N.A.	0
USP6	0,136785967	-0,322232085	0	N.A.	0

FAM109A	0,136742965	0,413758197	0	N.A.	0
PPP3CC	0,136724796	0,088702146	0	N.A.	0
TMEM175	0,136704978	0,399478751	0	N.A.	0
SAMD12-AS1	0,136661247	0,18698415	0	N.A.	0
AXL	0,136578774	-0,085021484	0	N.A.	0
UBTD1	0,136509569	0,139623732	0	N.A.	0
ANKRD10	0,136461515	0,278009031	0	N.A.	0
GBF1	0,136341892	0,153020702	0	N.A.	0
DNMBP-AS1	0,136338449	-0,336998044	0	N.A.	0
COPG1	0,13632857	0,347084045	0	N.A.	0
EXOC3L4	0,136307925	0,246067581	0	N.A.	0
LNX1	0,136284586	0,008205056	0	N.A.	0
EBLN2	0,136269273	0,303404445	0	N.A.	0
RP11-83B20.	0,136246246	-0,034512808	0	N.A.	0
FBLL1	0,136229933	0,199943991	0	N.A.	0
KRT5	0,136223892		0	N.A.	0
TBC1D5	0,136203676	-0,055897603	0	N.A.	0
AC096772.6	0,136082398	0,173265094	0	N.A.	0
SRSF5	0,135974531	0,370948839	0	N.A.	0
NUPR1L	0,13596963		0	N.A.	0
SYT5	0,135967233	0,053319867	0	N.A.	0
ERN1	0,135934326	0,260373515	0	N.A.	0
GLIS2	0,135907924	-0,020283343	0	N.A.	0
TMEM211	0,135886336	0,059238524	0	N.A.	0
IGHV1-69-2	0,135867112	-0,142838155	0	N.A.	0
ALOX12-AS1	0,135834164	0,209815268	0	N.A.	0
GPR135	0,13577533	0,049116401	0	N.A.	0
DRD1	0,135700182	0,396386422	0	N.A.	0
ATMIN	0,135667712	0,068527879	0	N.A.	0
FSCN2	0,135558051	0,116919587	0	N.A.	0
RP11-524F11	0,135543508		0	N.A.	0
KCTD20	0,135532075	-0,092872334	0	N.A.	0
RP11-81A22.	0,135521414		0	N.A.	0

ADAMTS2	0,135507993	-0,038715077	0	N.A.	0
WIZ	0,135446864	0,358041895	0	N.A.	0
IGKV1-5	0,135421766	-0,318503247	0	N.A.	0
RP11-10J21.3	0,135407357	-0,004032885	0	N.A.	0
RP11-385F5.5	0,135352425	0,006266333	0	N.A.	0
PLGLB2	0,135324902		0	N.A.	0
THY1	0,13530763	-0,238536146	0	N.A.	0
RASL12	0,135259034	-0,074342446	0	N.A.	0
MCM3AP	0,135193728	0,089937739	0	N.A.	0
GLMP	0,135175547	0,262580265	0	N.A.	0
NKIRAS2	0,135074888	0,193291367	0	N.A.	0
ACTB	0,135041094	0,025340914	0	N.A.	0
RP13-516M1	0,134991692	0,042357932	0	N.A.	0
ZNF561	0,134989132	-0,059705837	0	N.A.	0
ELF1	0,134983162	0,121636231	0	N.A.	0
RP11-231E19	0,134956244	0,061844379	0	N.A.	0
CSRNP2	0,134943588	0,389335601	0	N.A.	0
FBXO48	0,134930497	0,016334333	0	N.A.	0
RP11-353N14	0,134889777	0,088023706	0	N.A.	0
BIRC7	0,134869035	0,211508919	0	N.A.	0
XKR4	0,134836978	0,00448682	0	N.A.	0
TNIP3	0,134810225	0,10527796	0	N.A.	0
VCAM1	0,13479066	0,295049547	0	N.A.	0
GALNT10	0,134779818	0,199245731	0	N.A.	0
MCL1	0,134728304	0,13344491	0	N.A.	0
KCNMB1	0,134727686	0,140060441	0	N.A.	0
RP11-563K23	0,134702592	-0,049583366	0	N.A.	0
PHF12	0,134674916	0,300538973	0	N.A.	0
RUFY2	0,134660064	0,157023163	0	N.A.	0
ZDHHC13	0,13464993	0,047278216	0	N.A.	0
ZNF699	0,134619402	0,280552157	0	N.A.	0
FAM189A1	0,134612423	0,19330295	0	N.A.	0
AC004893.11	0,134497405	0,295288887	0	N.A.	0

TLR4	0,134452173	-0,084231432	0	N.A.	0
TM2D2	0,134433414	-0,070106039	0	N.A.	0
HIST2H2AA4	0,134432171	0,285302823	0	N.A.	0
PRSS27	0,134424522	0,093963045	0	N.A.	0
RARRES1	0,134422709	0,056789382	0	N.A.	0
SYNPO2L	0,134383105		0	N.A.	0
TMEM9B-AS1	0,134222832	0,270771547	0	N.A.	0
EBF1	0,134155358	-0,325742572	0	N.A.	0
AC009014.3	0,134149226	0,147354815	0	N.A.	0
ENTPD7	0,134097603	0,134984853	0	N.A.	0
SMPX	0,134077289	-0,057759408	0	N.A.	0
RP11-468E2.	0,13406508	0,099165978	0	N.A.	0
CCDC88B	0,134024173	0,222311659	0	N.A.	0
ITPK1-AS1	0,13401501	-0,089785733	0	N.A.	0
RP11-38M8.1	0,134014593	0,027725809	0	N.A.	0
ABI3BP	0,134009462	-0,074763103	0	N.A.	0
PLVAP	0,134006321	-0,152163018	0	N.A.	0
DHRS2	0,133959303	-0,208308892	0	N.A.	0
BRWD1	0,133914676	0,361120208	0	N.A.	0
RP11-416I2.1	0,133869359	0,003880087	0	N.A.	0
FGF18	0,133839565	-0,034394167	0	N.A.	0
CIITA	0,133746867	-0,104308903	0	N.A.	0
DARS-AS1	0,133744217	-0,041736359	0	N.A.	0
NOD2	0,133738037	0,008985307	0	N.A.	0
AP000487.5	0,133706228	0,1598802	0	N.A.	0
CYP2D6	0,133593597	0,169041272	0	N.A.	0
DNASE2B	0,133566566		0	N.A.	0
PCDHA5	0,133439117		0	N.A.	0
RP11-108K3.	0,133410258	-0,174222381	0	N.A.	0
MPO	0,133341188		0	N.A.	0
ALS2CR12	0,133282841	-0,042477602	0	N.A.	0
SH2D3C	0,133258258	0,066220034	0	N.A.	0
CHRNE	0,133228465		0	N.A.	0

HAP1	0,133224769	0,539579086	0	N.A.	0
PAK3	0,133204367		0	N.A.	0
SLC3A1	0,133134989	-0,152929141	0	N.A.	0
INPP5D	0,133107769	-0,018345513	0	N.A.	0
ERICH6-AS1	0,133087135	-0,237815953	0	N.A.	0
AC007383.4	0,13304422		0	N.A.	0
ANG	0,132979369	0,378665587	0	N.A.	0
HEXDC	0,132888323	0,112701965	0	N.A.	0
RP11-315I20	0,132821021	0,200755112	0	N.A.	0
CAMK4	0,132812497		0	N.A.	0
LINC00494	0,132804388		0	N.A.	0
ZNF394	0,132651028	-0,011661637	0	N.A.	0
PCDHGA10	0,132647767	0,063069984	0	N.A.	0
SAR1A	0,132572324	0,379903471	0	N.A.	0
PTGER1	0,13251141	-0,096636531	0	N.A.	0
RP11-47I22.2	0,132440343	-0,15043413	0	N.A.	0
RP11-640N11	0,132414726	0,071647757	0	N.A.	0
NXPH4	0,132388965	0,121440363	0	N.A.	0
PYROXD1	0,13222681	0,437784059	0	N.A.	0
GAS6-AS1	0,132199469	0,183672562	0	N.A.	0
TRIM62	0,132183351	0,045839524	0	N.A.	0
ARHGEF37	0,132179704	0,015843397	0	N.A.	0
KCTD2	0,132171886	0,222495649	0	N.A.	0
KIAA1211	0,132171293	0,222224056	0	N.A.	0
KREMEN2	0,132158352	-0,076540866	0	N.A.	0
RP11-557C18	0,132139399	-0,131790427	0	N.A.	0
LIG4	0,132089427	0,244150866	0	N.A.	0
CHST1	0,132088421	0,061489082	0	N.A.	0
PPM1J	0,132026531	-0,054016619	0	N.A.	0
CANT1	0,132012465	0,251820395	0	N.A.	0
LINC00865	0,131981629	-0,306129441	0	N.A.	0
SMCR8	0,131874126	0,055344063	0	N.A.	0
RP5-884M6.1	0,131861812	-0,021270112	0	N.A.	0

RP11-838N2.	0,131820679		0	N.A.	0
SEMA6D	0,131745664	-0,06998935	0	N.A.	0
KRT4	0,131537902	0,203434521	0	N.A.	0
ATP7B	0,131393625	0,283908983	0	N.A.	0
LATS1	0,131391578	0,391525085	0	N.A.	0
SERPING1	0,131374223	-0,163483228	0	N.A.	0
TAF5L	0,131323389	0,021080018	0	N.A.	0
RP11-131L23	0,13126615	0,032067586	0	N.A.	0
RP11-147L13	0,131259885	0,209753995	0	N.A.	0
VWA2	0,131210207	-0,168429107	0	N.A.	0
ATAD3C	0,13117172	0,106938649	0	N.A.	0
RP11-317J10	0,131108673	0,11430167	0	N.A.	0
ERVW-1	0,131049641		0	N.A.	0
SPP1	0,131049228	-0,169760509	0	N.A.	0
ERAP2	0,131032866	0,086718126	0	N.A.	0
RP11-304F15	0,13096823		0	N.A.	0
AGAP5	0,130914425	0,150104423	0	N.A.	0
FBXW4	0,130859301	0,215779273	0	N.A.	0
RP11-54H7.4	0,130790924	0,13751943	0	N.A.	0
SLC3A2	0,130784537	0,517783121	0	N.A.	0
CXCL10	0,130718943	0,338199654	0	N.A.	0
NFATC4	0,130702903	0,062499599	0	N.A.	0
NR1I3	0,130680805		0	N.A.	0
LRRC16A	0,130675732	0,003486792	0	N.A.	0
UBE2E2	0,130650757	-0,245575413	0	N.A.	0
WDR27	0,130638352	0,093170918	0	N.A.	0
SPATA18	0,13055557	-0,10664966	0	N.A.	0
DDX3Y	0,130521043	0,113685483	0	N.A.	0
RGL3	0,130520074	0,306602563	0	N.A.	0
RP11-368L12	0,13051267	-0,024836406	0	N.A.	0
CCNT1	0,130511671	0,348541013	0	N.A.	0
ARPC2	0,130423766	-0,019846064	0	N.A.	0
TEKT4	0,130384224		0	N.A.	0

MT-ND1	0,130344995	0,395036326	0	N.A.	0
RP11-433J8.1	0,130287465	0,000855496	0	N.A.	0
SLC22A31	0,130148605		0	N.A.	0
SFT2D3	0,13009135	0,059353931	0	N.A.	0
ZNF431	0,130091272	0,518670663	0	N.A.	0
TRAPPC6B	0,129980492	0,116780811	0	N.A.	0
KRTAP13-2	0,129961282	0,353255453	0	N.A.	0
LINC00365	0,129945025	-0,065505168	0	N.A.	0
RP13-188A5.	0,129912694	-0,002419724	0	N.A.	0
CLEC18C	0,129882856		0	N.A.	0
CRTC3	0,129847874	-0,054786978	0	N.A.	0
CCL22	0,129847506	-0,082172977	0	N.A.	0
TTN-AS1	0,129773806	0,24422011	0	N.A.	0
MEDAG	0,129751675	0,064861234	0	N.A.	0
MT1M	0,129713014	-0,133262254	0	N.A.	0
SLC7A14	0,129672237	0,235985895	0	N.A.	0
KLK1	0,129657124	0,199036017	0	N.A.	0
CTB-127C13.	0,129652033	-0,327425464	0	N.A.	0
BRAP	0,129595521	-0,133632381	0	N.A.	0
CREB5	0,129588752	0,146884549	0	N.A.	0
BVES	0,129578595	0,276828575	0	N.A.	0
MAPK8IP2	0,129537902	0,067204007	0	N.A.	0
KB-1125A3.1	0,129505198	0,194533208	0	N.A.	0
SLIT1	0,129491867	-0,032284543	0	N.A.	0
RP4-591C20.	0,129439712	0,230122786	0	N.A.	0
CD151	0,129426793	0,734132696	0	N.A.	0
LYG1	0,129322456	0,079053687	0	N.A.	0
GK-AS1	0,12930376	0,063444968	0	N.A.	0
RNF217	0,129259333	0,295116297	0	N.A.	0
BAK1	0,129238092	0,086760397	0	N.A.	0
MDM2	0,129221754	0,026212792	0	N.A.	0
SLC2A14	0,12920758		0	N.A.	0
SIRPA	0,129203092	0,11400788	0	N.A.	0

H6PD	0,129121093	0,199970457	0	N.A.	0
TRPM3	0,129120067		0	N.A.	0
MYO1A	0,129091076	0,228632966	0	N.A.	0
OS9	0,129078721	0,336050324	0	N.A.	0
RP4-564F22.5	0,128962076	0,203687781	0	N.A.	0
RP11-333I13	0,128929747	0,015711623	0	N.A.	0
RP11-493L12	0,128913573	0,27481474	0	N.A.	0
SLC22A1	0,128852239	0,018269011	0	N.A.	0
ESRRA	0,128764596	0,174099098	0	N.A.	0
CXCL11	0,128733115	0,105575762	0	N.A.	0
RGPD5	0,128717151	0,164469421	0	N.A.	0
RP11-179A10	0,128663982		0	N.A.	0
TMEM39A	0,128479317	0,409354477	0	N.A.	0
UAP1	0,128471625	0,207192329	0	N.A.	0
ATP6V1C2	0,128438268	-0,291670781	0	N.A.	0
RP1L1	0,128387187	0,371035043	0	N.A.	0
SH3GLB2	0,128382624	0,372020031	0	N.A.	0
RP11-16E12.	0,128380365	-0,210654666	0	N.A.	0
SNAPC3	0,128376998	0,210484873	0	N.A.	0
AC007292.6	0,128352927	-0,131135897	0	N.A.	0
KIAA2018	0,128345575	0,106613191	0	N.A.	0
XXbac-B476C	0,12831851	0,204092222	0	N.A.	0
C9orf85	0,128279822	0,165924165	0	N.A.	0
MCAM	0,128269103	-0,228360122	0	N.A.	0
RP11-317P15	0,128246285		0	N.A.	0
RP11-178C3.	0,128219019	-0,198605563	0	N.A.	0
TNFRSF9	0,128185786	-0,180114793	0	N.A.	0
RP11-818F20	0,128152008	0,40326971	0	N.A.	0
LINC00950	0,12812245	-0,22174149	0	N.A.	0
CADM2	0,128120764	0,114140366	0	N.A.	0
TBC1D8B	0,127987795	0,373109685	0	N.A.	0
CACNA1F	0,127931462	0,163275461	0	N.A.	0
INPP5F	0,127913269	0,303546418	0	N.A.	0

NLGN2	0,127886983	-0,116162606	0	N.A.	0
HS6ST1	0,127885516	0,276682785	0	N.A.	0
SLC9A6	0,127844074	0,116772921	0	N.A.	0
RP11-319G6.	0,127832242	-0,095873982	0	N.A.	0
UBC	0,127827233	0,345844864	0	N.A.	0
SNAPC2	0,127757849	0,200855856	0	N.A.	0
HYAL3	0,127663137	-0,345830041	0	N.A.	0
KIAA0895L	0,127654678	0,116113454	0	N.A.	0
RP1-63M2.7	0,127594504	-0,05864025	0	N.A.	0
ADRA1D	0,127435696		0	N.A.	0
FBXL16	0,127433414	-0,055810927	0	N.A.	0
TMEM55B	0,127426976	0,342546282	0	N.A.	0
KLHL28	0,127420126	0,345413964	0	N.A.	0
RPA4	0,12740835	0,126868993	0	N.A.	0
SAP25	0,127359523	0,278564286	0	N.A.	0
GOLGA6L4	0,12733874	0,411543701	0	N.A.	0
SIK3	0,127326878	0,146595722	0	N.A.	0
CHKA	0,127308024	0,407954302	0	N.A.	0
TMEM86A	0,127188103	0,234345244	0	N.A.	0
RAPSN	0,127163638		0	N.A.	0
XXbac-BPG25	0,127094368	0,198906763	0	N.A.	0
KRTAP3-1	0,127035743	0,027614159	0	N.A.	0
RP13-463N16	0,127019806	-0,095120142	0	N.A.	0
SEPT9	0,126996067	0,035851683	0	N.A.	0
EQTN	0,126985127		0	N.A.	0
CRYBG3	0,126980952	0,229818626	0	N.A.	0
TWF1	0,126960771	0,19486676	0	N.A.	0
SLC28A2	0,126935559	-0,18654286	0	N.A.	0
LETM2	0,126863147	-0,0305306	0	N.A.	0
TANK	0,126819629	0,00713353	0	N.A.	0
ETFDH	0,126819109	0,009146026	0	N.A.	0
FAM50A	0,126811596	0,362167223	0	N.A.	0
CDK14	0,126779173	0,079690545	0	N.A.	0

RP11-747D18	0,12672928	-0,134604386	0	N.A.	0
RP11-428O18	0,126724246	0,332219001	0	N.A.	0
RP11-37B2.1	0,126684165	0,029072251	0	N.A.	0
IFI44	0,126656173	0,410765658	0	N.A.	0
CPAMD8	0,126640904	0,020882469	0	N.A.	0
RNF4	0,126632219	0,095523507	0	N.A.	0
GLI1	0,126601807	-0,318315859	0	N.A.	0
BICD2	0,126581962	0,021494714	0	N.A.	0
CRTC2	0,126530999	0,148624291	0	N.A.	0
BMP8B	0,126524152	-0,076440101	0	N.A.	0
RNF123	0,126522929	-0,049989752	0	N.A.	0
RAB14	0,126503357	0,332229698	0	N.A.	0
KMT2A	0,126480471	0,343311743	0	N.A.	0
CH507-513H4	0,126442219	-0,258288382	0	N.A.	0
ESR2	0,12642656		0	N.A.	0
NID2	0,126425499	-0,083674087	0	N.A.	0
RP11-182J1.3	0,126377185		0	N.A.	0
MCTP2	0,126311612	0,227067126	0	N.A.	0
HLA-DOB	0,126286906	-0,08803447	0	N.A.	0
RP11-800A3.	0,12628083	0,148108862	0	N.A.	0
BTRC	0,126252153	0,228856362	0	N.A.	0
ATAD1	0,126241706	0,050692823	0	N.A.	0
POLM	0,126224219	0,405165533	0	N.A.	0
DOCK7	0,126205366	0,344286375	0	N.A.	0
ABALON	0,126198805	0,098393017	0	N.A.	0
ASB6	0,126150648	0,209078114	0	N.A.	0
RP4-737E23.	0,126129809	0,103561373	0	N.A.	0
ZNF567	0,126082413	0,086103834	0	N.A.	0
AC226118.1	0,126055679	0,205745471	0	N.A.	0
RP11-383H13	0,126018098	0,254870055	0	N.A.	0
RAB2B	0,12601247	-0,083081607	0	N.A.	0
ZNF117	0,125990756	0,113552471	0	N.A.	0
RP11-196G18	0,125966446	-0,200379221	0	N.A.	0

FNBP1	0,125949742	0,119368822	0	N.A.	0
C1S	0,125941357	-0,14182943	0	N.A.	0
RP11-539L10	0,125939054	0,023600781	0	N.A.	0
RP11-223C24	0,125734239	0,313554315	0	N.A.	0
GPRASP1	0,125722575	-0,037611393	0	N.A.	0
HDAC11	0,12565201	0,069225332	0	N.A.	0
ZNF528	0,125613473	0,186320124	0	N.A.	0
RP11-1060J1	0,125574188	-0,111036271	0	N.A.	0
PPP1R1C	0,125570827	0,161399394	0	N.A.	0
SETD5	0,125549067	0,319608582	0	N.A.	0
FAM219B	0,12554279	0,191929239	0	N.A.	0
RP11-583F2.	0,125528464		0	N.A.	0
AGO2	0,125527673	0,338518328	0	N.A.	0
FER	0,125486597	0,267120377	0	N.A.	0
SLC31A2	0,12546297	0,098613251	0	N.A.	0
LPCAT2	0,125459967	0,539025253	0	N.A.	0
FAM153B	0,125444746		0	N.A.	0
ZBTB41	0,125392569	0,031313784	0	N.A.	0
IKZF1	0,125281852	-0,185457386	0	N.A.	0
CERS2	0,125278119	0,430835627	0	N.A.	0
CMTR2	0,125267509	0,282364371	0	N.A.	0
SCARNA2	0,125253253	0,02487679	0	N.A.	0
RP11-250B2.	0,125185693	-0,032800313	0	N.A.	0
CASD1	0,125137731	0,029377787	0	N.A.	0
DNAH12	0,125118253	-0,060333307	0	N.A.	0
CNN1	0,125105706	-0,046738521	0	N.A.	0
CCDC97	0,125069055	0,223683191	0	N.A.	0
IVNS1ABP	0,125032282	0,15172559	0	N.A.	0
ERF	0,124968908	0,193298848	0	N.A.	0
RP11-121E16	0,124967035		0	N.A.	0
RAB17	0,124965854	0,13414002	0	N.A.	0
REV1	0,124849329	0,111071857	0	N.A.	0
IGSF6	0,124835063	-0,29665516	0	N.A.	0

AC017002.1	0,124825972	0,23596513	0	N.A.	0
RP11-150C16	0,124817061	0,00717044	0	N.A.	0
TRIM45	0,124794387	-0,005666508	0	N.A.	0
LINC01138	0,124708992	-0,068095942	0	N.A.	0
VPS13C	0,124610032	0,236373036	0	N.A.	0
RP11-215P8.	0,124566308	-0,014595747	0	N.A.	0
SLC26A1	0,124564676	-0,00392063	0	N.A.	0
RP11-429J17	0,124563572		0	N.A.	0
SUPT5H	0,124549229	0,312891906	0	N.A.	0
GDPD2	0,124523978	-0,183210922	0	N.A.	0
RASAL2	0,124521673	0,258544317	0	N.A.	0
RGP1	0,124500442	0,027271399	0	N.A.	0
TNFRSF18	0,124466299	-0,150188175	0	N.A.	0
LINC00672	0,124384509	0,258606541	0	N.A.	0
NPAS3	0,124362184		0	N.A.	0
HOTAIRM1	0,124356851	0,267212178	0	N.A.	0
SPRY4	0,124326523	0,289258433	0	N.A.	0
PRR34-AS1	0,124254394	0,080529904	0	N.A.	0
RP5-892K4.1	0,12418817	0,085826916	0	N.A.	0
CTNS	0,124113687	0,527603568	0	N.A.	0
AKAP17A	0,124100107	0,365541402	0	N.A.	0
GAS2L1	0,124089577	0,124985218	0	N.A.	0
CTD-2619J13	0,12400874	-0,099201949	0	N.A.	0
RNF14	0,124003171	0,022731461	0	N.A.	0
JARID2	0,124000925	0,078958828	0	N.A.	0
KRTAP2-3	0,123969623	0,004569852	0	N.A.	0
MKL2	0,123934142	0,246519611	0	N.A.	0
CD40	0,123899741	-0,193281346	0	N.A.	0
NPY1R	0,123893178	0,040729624	0	N.A.	0
HPGDS	0,123811294	-0,27099092	0	N.A.	0
BPIFA2	0,123782662	-0,176808884	0	N.A.	0
RP5-1021I20	0,123761167	0,236786835	0	N.A.	0
RPH3AL	0,123695585	0,215600203	0	N.A.	0

KCNJ5	0,12367451		0	N.A.	0
SYTL5	0,123673871	0,233553324	0	N.A.	0
PON2	0,123628044	0,609999907	0	N.A.	0
RP11-486I11	0,123617044	-0,348934795	0	N.A.	0
SELPLG	0,123612298	-0,030651847	0	N.A.	0
TLR6	0,12356753	-0,041584073	0	N.A.	0
CD2BP2	0,123557626	0,2302655	0	N.A.	0
SH3BP5L	0,123549827	0,048701688	0	N.A.	0
FXYD7	0,12353262	-0,078978172	0	N.A.	0
RP3-323A16.	0,123525103	-0,234401285	0	N.A.	0
GNAS	0,123453691	0,062398735	0	N.A.	0
PLEKHA5	0,123449815	0,270206676	0	N.A.	0
RP11-3P17.5	0,123433758		0	N.A.	0
RNF6	0,123415807	0,200105331	0	N.A.	0
C1orf159	0,123391601	0,11017817	0	N.A.	0
SFTPA2	0,123346944	0,271077316	0	N.A.	0
KIAA0368	0,123228929	0,137116838	0	N.A.	0
ADCY9	0,123213755	0,282831807	0	N.A.	0
SEMA4F	0,12314441	0,174802948	0	N.A.	0
GVQW1	0,123139999	0,254293093	0	N.A.	0
CTA-217C2.2	0,123089664		0	N.A.	0
CTC-308K20.	0,123063369	0,173551623	0	N.A.	0
RP3-449M8.9	0,123022511		0	N.A.	0
CNST	0,123018234	0,346588257	0	N.A.	0
FAM193B	0,123000021	0,240168771	0	N.A.	0
PSMD6-AS2	0,122994811	0,304397429	0	N.A.	0
SHC4	0,122981706	0,131683491	0	N.A.	0
ZNF322	0,122962746	-0,141369122	0	N.A.	0
ADAP1	0,12293595	0,477928242	0	N.A.	0
ATP13A4	0,122856646	0,011117001	0	N.A.	0
RP11-432I5.4	0,122793454	0,030721969	0	N.A.	0
RP11-161M6	0,122685163	-0,074673285	0	N.A.	0
SRCAP	0,122683482	0,178090183	0	N.A.	0

OTUB2	0,122651335	0,287347235	0	N.A.	0
RP11-26J3.1	0,122628603	-0,114586195	0	N.A.	0
CTD-2008A1.	0,122623171	0,06197619	0	N.A.	0
RP11-218M2	0,122622976	0,136396663	0	N.A.	0
TTC30B	0,122574903	0,045646225	0	N.A.	0
SPOCK1	0,122570815	-0,167246137	0	N.A.	0
EML6	0,122493107	0,012732346	0	N.A.	0
GSK3B	0,122447357	0,23225856	0	N.A.	0
RP11-475E11	0,122405472	-0,010894152	0	N.A.	0
LINC00106	0,122232096	-0,09270259	0	N.A.	0
LEAP2	0,122182589	0,240618985	0	N.A.	0
FCER1G	0,122160908	-0,326872332	0	N.A.	0
CASC16	0,122148536	0,178847784	0	N.A.	0
DNAJB12	0,122126523	0,23880005	0	N.A.	0
MTMR9	0,122069436	0,192268023	0	N.A.	0
SUFU	0,122067787	0,065490959	0	N.A.	0
LNPEP	0,122064036	0,432974664	0	N.A.	0
SPRED1	0,122038665	0,313339972	0	N.A.	0
CBX7	0,121938553	-0,149422986	0	N.A.	0
LZTR1	0,121936163	-0,041898018	0	N.A.	0
AC010761.10	0,121893139	-0,014868036	0	N.A.	0
DMTN	0,121887034	0,375008395	0	N.A.	0
RP11-295P9.	0,121858487	0,377366587	0	N.A.	0
NPHP3-ACAD	0,121854816	-0,061905805	0	N.A.	0
RP5-965G21.	0,121818954	0,035059985	0	N.A.	0
NOTCH2NL	0,121814538	0,221978511	0	N.A.	0
TMEM88	0,121798199	0,219819342	0	N.A.	0
NKX6-1	0,121775818		0	N.A.	0
TTC7B	0,121766908	-0,119924503	0	N.A.	0
AC074011.2	0,121761971		0	N.A.	0
CTD-2521M2	0,12170165		0	N.A.	0
FARP2	0,121678549	0,362915295	0	N.A.	0
PLA2G2D	0,121616758		0	N.A.	0

THEMIS	0,121595221	0,223357989	0	N.A.	0
SLC9A3R2	0,121581374	0,12181321	0	N.A.	0
AC002467.7	0,121573811	0,318781023	0	N.A.	0
GRAMD1A	0,121507816	0,187826466	0	N.A.	0
AC019181.2	0,121507166		0	N.A.	0
RP11-305L7.3	0,121483171		0	N.A.	0
GGN	0,1213949		0	N.A.	0
EPC2	0,121369116	0,185676597	0	N.A.	0
DOCK9-AS2	0,121256093	0,136937798	0	N.A.	0
RP13-580F15	0,121224527	0,121969524	0	N.A.	0
TNK2-AS1	0,121193838		0	N.A.	0
THNSL2	0,121172314	-0,140780453	0	N.A.	0
SFI1	0,121162977	0,268554668	0	N.A.	0
RP11-514O12	0,12106089	-0,194564342	0	N.A.	0
TMEM38A	0,121043568	0,038191594	0	N.A.	0
MFSD2A	0,121035996	0,335311878	0	N.A.	0
RP11-10C24.	0,121008379	0,115637039	0	N.A.	0
RP11-539L10	0,120995787	-0,233476375	0	N.A.	0
SLC25A36	0,120991745	0,225757311	0	N.A.	0
FLJ31356	0,120988474	0,413818962	0	N.A.	0
RP11-284N8.	0,120987399		0	N.A.	0
RP11-439E19	0,120970863	-0,005131848	0	N.A.	0
SORBS1	0,120956182	-0,117913681	0	N.A.	0
KCNG1	0,120949717	0,107473348	0	N.A.	0
GRB7	0,12088525	0,358611818	0	N.A.	0
P2RX5-TAX1B	0,120845221	-0,116358748	0	N.A.	0
AC009404.2	0,120810154	0,221683516	0	N.A.	0
ARSI	0,120801085	0,097602164	0	N.A.	0
RP11-400N13	0,120766162	-0,092392208	0	N.A.	0
ZCCHC2	0,120759495	0,20633889	0	N.A.	0
GNMT	0,120636661		0	N.A.	0
REN	0,120617288	0,18669205	0	N.A.	0
PACS2	0,120563326	0,335124729	0	N.A.	0

TSPYL2	0,120530323	-0,040603056	0	N.A.	0
GNRH1	0,120453441	0,305488801	0	N.A.	0
CCL13	0,120451802	-0,091786456	0	N.A.	0
FAM63A	0,120388293	-0,140649558	0	N.A.	0
DNAJB6	0,120381556	-0,034853414	0	N.A.	0
AKAP10	0,120367693	0,082631046	0	N.A.	0
XXyac-YX65C	0,120335854		0	N.A.	0
TTBK2	0,120328374	0,325954105	0	N.A.	0
KIAA1107	0,120235794	0,081310606	0	N.A.	0
MLLT4-AS1	0,120201638	0,431987518	0	N.A.	0
UPK3A	0,120173339	-0,034075864	0	N.A.	0
NEK5	0,120137214	-0,005790257	0	N.A.	0
PCAT19	0,120119518	-0,285457525	0	N.A.	0
HOMER3	0,120027917	0,216355684	0	N.A.	0
CAB39L	0,120012203	0,170061081	0	N.A.	0
SNN	0,120002158	0,194095222	0	N.A.	0
IL12RB1	0,119849837	0,357582912	0	N.A.	0
PIGG	0,119848768	0,343092941	0	N.A.	0
VOPP1	0,119828272	0,181799781	0	N.A.	0
DRAM1	0,119826018	-0,03295236	0	N.A.	0
ARHGEF18	0,119668798	0,287501056	0	N.A.	0
AC136616.1	0,119655101	0,344893269	0	N.A.	0
TCF20	0,119590058	0,131950562	0	N.A.	0
CRACR2B	0,119569232	0,306259717	0	N.A.	0
ANKRD33	0,119492576		0	N.A.	0
SEMA6A-AS1	0,119458864	-0,036750101	0	N.A.	0
KANSL3	0,119441724	0,121141679	0	N.A.	0
RP11-227H15	0,119343334	-0,168975944	0	N.A.	0
KIAA0895	0,119329277	0,1926492	0	N.A.	0
RP11-675F6.4	0,119299776	-0,024402574	0	N.A.	0
AC005487.2	0,119295979	0,030721969	0	N.A.	0
PGAP3	0,119294169	0,198879456	0	N.A.	0
HAVCR1	0,119293848	-0,13486379	0	N.A.	0

RP11-297M9	0,119287701	-0,191725167	0	N.A.	0
ALOXE3	0,119208865	0,14723223	0	N.A.	0
ADCY7	0,119177752	0,257732609	0	N.A.	0
TTC14	0,119173503	0,19189215	0	N.A.	0
TLN2	0,119066641	0,189112786	0	N.A.	0
DNHD1	0,119044094	0,208824859	0	N.A.	0
MAPK11	0,118999084	-0,075592913	0	N.A.	0
NRSN2-AS1	0,118952411	-0,23257679	0	N.A.	0
DIP2A	0,118945259	0,142802009	0	N.A.	0
UBE2A	0,118942617	-0,042438243	0	N.A.	0
CXorf65	0,118895228	0,0167993	0	N.A.	0
NXF3	0,118868547	0,124749117	1	cls3	0
UEVLD	0,118855875	-0,121041185	0	N.A.	0
RP1-150O5.3	0,118847543		0	N.A.	0
BTD	0,118803488	0,1167486	0	N.A.	0
STRC	0,11877968	0,105803584	0	N.A.	0
RP11-449D8.	0,118767459	0,117091098	0	N.A.	0
PRR5-ARHGA	0,118645315	-0,00218291	0	N.A.	0
RBMS3	0,118634618	-0,116643251	0	N.A.	0
SQRDL	0,118604755	0,367963659	0	N.A.	0
WFDC5	0,118471159	0,251500084	0	N.A.	0
ILDR2	0,118435359	-0,052286491	0	N.A.	0
WFDC1	0,118417401	0,129229784	0	N.A.	0
DTX1	0,118338736	0,311516488	0	N.A.	0
BEX2	0,11830913	0,070435319	0	N.A.	0
HMGCR	0,118304124	0,242216323	0	N.A.	0
IPMK	0,118236618	-0,03220388	0	N.A.	0
USP34	0,118236133	0,438442831	0	N.A.	0
AP2A2	0,118217619	0,099969369	0	N.A.	0
TEX14	0,118200562		0	N.A.	0
SLA2	0,118113997	-0,036058694	0	N.A.	0
PARP3	0,118113446	0,136271439	0	N.A.	0
RP11-20I23.6	0,118104952	0,391820096	0	N.A.	0

LINC00239	0,118065567	-0,152032562	0	N.A.	0
TUG1	0,11806177	0,123226345	0	N.A.	0
PCDHGA1	0,118056044	-0,070879352	0	N.A.	0
ZDHHC11	0,118017357	0,060494878	0	N.A.	0
FXR2	0,117958612	0,472572466	0	N.A.	0
CD1C	0,117883567	-0,183155979	0	N.A.	0
SPESP1	0,117791169	0,100954369	0	N.A.	0
RP11-563J2.2	0,11776719	0,061010379	0	N.A.	0
GRK5	0,11774256	-0,178409719	0	N.A.	0
TCEB2	0,117684599	0,039783897	0	N.A.	0
ALG10B	0,117660491	-0,113922993	0	N.A.	0
SKAP2	0,117659857	0,340768189	0	N.A.	0
ARHGAP31	0,11764646	-0,211970812	0	N.A.	0
IGSF8	0,117628464	0,554460205	0	N.A.	0
N4BP3	0,117608162	-0,099515066	0	N.A.	0
KIAA0556	0,117603328	0,338071508	0	N.A.	0
TNFSF14	0,117539655		0	N.A.	0
RP3-395M20	0,117480885	0,107204846	0	N.A.	0
AC140912.1	0,117440442	-0,065237288	0	N.A.	0
RP5-1085F17	0,117432588	0,174615781	0	N.A.	0
AHRR	0,117422145	0,158473083	0	N.A.	0
CARD14	0,117388393	0,323569094	0	N.A.	0
ESCO1	0,117358823	0,103463546	0	N.A.	0
THBS2	0,117352915	0,065503794	0	N.A.	0
RP11-528I4.2	0,117327854	0,044466191	0	N.A.	0
LIN37	0,117317937	0,095607139	0	N.A.	0
ITGA6	0,117293771	0,463024178	0	N.A.	0
AC009133.15	0,117255877		0	N.A.	0
TAPT1-AS1	0,11724933	-0,047306366	0	N.A.	0
B3GALT1	0,117217449		0	N.A.	0
EFCAB13	0,117195476	0,188664498	0	N.A.	0
RP3-510D11.	0,117165894	-0,035664096	0	N.A.	0
AQP8	0,117123245	0,201329568	0	N.A.	0

RP11-12J10.3	0,117115588		0	N.A.	0
RP11-88E10.	0,117099926	0,241207263	0	N.A.	0
SNRK-AS1	0,117090659		0	N.A.	0
UNC5A	0,117003391	0,112651955	0	N.A.	0
GTF2I	0,116993404	0,369021915	0	N.A.	0
SNHG23	0,116906958	0,112242532	0	N.A.	0
RP11-54O7.1	0,116899094	0,155998182	0	N.A.	0
PCNX	0,116809242	0,380972065	0	N.A.	0
AC078883.3	0,116789731	0,126465108	0	N.A.	0
RP1-244F24.	0,116765893	-0,10657483	0	N.A.	0
KDM4C	0,116740859	0,19240819	0	N.A.	0
IL24	0,116668392	0,183862267	0	N.A.	0
ZNF704	0,116610606	0,248613125	0	N.A.	0
PLCB3	0,116606306	0,357643385	0	N.A.	0
MPP3	0,116580701	0,297604504	0	N.A.	0
TCAP	0,116563054	-0,068196696	0	N.A.	0
IL17RA	0,116534684	0,084469229	0	N.A.	0
ASTE1	0,116531026	0,065833887	0	N.A.	0
RP5-940J5.6	0,11651642		0	N.A.	0
KCNN2	0,116512463	0,061556441	0	N.A.	0
MSC	0,116497111	-0,118072454	0	N.A.	0
PERM1	0,116486357		0	N.A.	0
ARMCX4	0,116364552	0,171113636	0	N.A.	0
PIK3CA	0,116358135	0,125139631	0	N.A.	0
CD6	0,116194853	-0,034369504	0	N.A.	0
AC144835.1	0,116193973	0,198097503	0	N.A.	0
KCTD13	0,116187513	0,100365952	0	N.A.	0
STK38	0,116186367	0,202851883	0	N.A.	0
PBX4	0,11612047	0,512978895	0	N.A.	0
FBXO42	0,116109037	0,077664173	0	N.A.	0
ANKFN1	0,1160316	0,056881664	0	N.A.	0
UGP2	0,116026135	0,000250981	0	N.A.	0
TRIM58	0,11602081	-0,179790989	0	N.A.	0

G0S2	0,116017407	0,25648654	0	N.A.	0
SOCS1	0,116004194	0,155480992	0	N.A.	0
IL5	0,11599005	0,34381894	0	N.A.	0
DNAJB14	0,115956767	0,260818349	0	N.A.	0
AC018816.3	0,11588417	-0,045349846	0	N.A.	0
DNASE1	0,115882022	0,476169488	0	N.A.	0
KLK13	0,115874932	0,392458114	0	N.A.	0
GDPGP1	0,115831563	0,068062441	0	N.A.	0
TMEM248	0,115801392	0,322966466	0	N.A.	0
TMEM95	0,115782509		0	N.A.	0
INAFM2	0,115762388	0,054831312	0	N.A.	0
CABLES1	0,115636957	-0,021029353	0	N.A.	0
RBM44	0,115572517	0,048814127	0	N.A.	0
DAPK2	0,11556875	-0,040719462	0	N.A.	0
FAM149A	0,115556423	-0,168033853	0	N.A.	0
EPC1	0,115480752	0,105838651	0	N.A.	0
RP11-159G9.	0,115479672	-0,146460509	0	N.A.	0
SYNE2	0,115477236	-0,065141107	0	N.A.	0
NOVA1-AS1	0,115438073	0,010841376	0	N.A.	0
RBM6	0,115419458	0,099992918	0	N.A.	0
CFD	0,115379071	0,065026732	0	N.A.	0
JMY	0,115340321	0,080775373	0	N.A.	0
RALA	0,115331782	0,363961512	0	N.A.	0
HOXA4	0,115330488	-0,119800247	1	cl1	0
RP11-333E1.	0,115329019	0,003332138	0	N.A.	0
HPS5	0,115314973	0,102417793	0	N.A.	0
ST3GAL6	0,11531274	0,271182801	0	N.A.	0
RP1-111C20.	0,11515751	-0,052926382	0	N.A.	0
ZFP57	0,115063117		0	N.A.	0
GATM-AS1	0,115057354	0,11868087	0	N.A.	0
PLS3	0,115048005	0,339545866	0	N.A.	0
LINC00880	0,115034553	0,289314415	0	N.A.	0
ABCC10	0,114986592	0,219458011	0	N.A.	0

KRTAP5-9	0,114954444	0,228939174	0	N.A.	0
ARTN	0,114941894	-0,027717845	0	N.A.	0
PARD3B	0,114934359	0,47044134	0	N.A.	0
UBN2	0,114934199	0,254768964	0	N.A.	0
ACKR3	0,114905291	-0,053639716	0	N.A.	0
LRFN3	0,114880763	0,003634928	0	N.A.	0
OTUD3	0,114850581	0,278949941	0	N.A.	0
MEIS1	0,114759016	-0,245832023	0	N.A.	0
TGFB2	0,114747963	0,113628497	0	N.A.	0
COL5A2	0,114705646	0,029353316	0	N.A.	0
YY1AP1	0,114624276	0,199626059	0	N.A.	0
SNPH	0,114617995	-0,057214835	0	N.A.	0
RP11-11N5.1	0,114555247	-0,22061673	0	N.A.	0
LPAR2	0,114547403	0,293889222	0	N.A.	0
FUCA1	0,114507968	0,271435773	0	N.A.	0
C11orf24	0,114496055	0,035246157	0	N.A.	0
CTC-1337H24	0,114469574	0,035312665	0	N.A.	0
VNN3	0,114334463		0	N.A.	0
XXbac-BPG27	0,114303239	0,007429475	0	N.A.	0
SNX12	0,114220103	0,002404016	0	N.A.	0
EVI5	0,114214218	0,238833072	0	N.A.	0
CHM	0,114208665	-0,1102855	0	N.A.	0
SCN3B	0,114158533	0,283364816	0	N.A.	0
ARHGEF16	0,113983801	0,645972584	0	N.A.	0
FGF11	0,113964475	-0,103147016	0	N.A.	0
LINC00847	0,113944907	-0,085286135	0	N.A.	0
LINC00882	0,113875237	-0,003015134	0	N.A.	0
PTGIS	0,113867559	-0,281175683	0	N.A.	0
ZNF792	0,113865493	-0,070065096	0	N.A.	0
MKNK1	0,113826515	0,085597811	0	N.A.	0
PDE4D	0,113825226	0,179003572	0	N.A.	0
DMXL1	0,113782143	0,282263857	0	N.A.	0
RP1-30M3.5	0,113717308		0	N.A.	0

PTPRE	0,11363518	0,126652546	0	N.A.	0
CTD-2020K17	0,113581896	-0,119365796	0	N.A.	0
HPCA	0,113566474	-0,191386253	0	N.A.	0
ANXA8	0,113553482	0,341638038	0	N.A.	0
OLMALINC	0,113524023	-0,022486773	0	N.A.	0
LYRM9	0,113521742	0,014166108	0	N.A.	0
TRERF1	0,113483124	0,236889081	0	N.A.	0
NUMA1	0,113437067	0,171717852	0	N.A.	0
AC006547.13	0,113412766		0	N.A.	0
TPTE2	0,11331429		0	N.A.	0
BTBD10	0,113311477	0,212605815	0	N.A.	0
CBWD5	0,113301873	0,119626402	0	N.A.	0
IGHV3-7	0,113229422	-0,124752779	0	N.A.	0
RP1-170O19.	0,113165232	0,029670443	0	N.A.	0
CTD-2292M1	0,113114478	-0,167094224	0	N.A.	0
TNKS	0,113104044	0,243841406	0	N.A.	0
CCDC151	0,113098935		0	N.A.	0
HIST1H2BF	0,113020507	-0,073541593	0	N.A.	0
RAB12	0,113018005	0,118023691	0	N.A.	0
EXPH5	0,11299877	0,114100685	0	N.A.	0
SH3BP5	0,112929962	-0,086594223	0	N.A.	0
EFNA5	0,112914502	0,301113144	0	N.A.	0
AC012358.8	0,112909775	0,101189261	0	N.A.	0
CTB-131K11.	0,112907748	-0,103519307	0	N.A.	0
ZNF516	0,112884713	0,068547842	0	N.A.	0
SAA4	0,112880985	0,464724926	0	N.A.	0
SETD7	0,112856225	0,024250488	0	N.A.	0
PRKAA2	0,112851326	0,071147269	0	N.A.	0
RP11-803B1.	0,112789696	0,061866831	0	N.A.	0
RP11-328J2.1	0,112784358		0	N.A.	0
TTBK1	0,112783527	0,007741292	0	N.A.	0
UGGT1	0,112758963	0,341767846	0	N.A.	0
ATP6AP1	0,112745278	0,51329178	0	N.A.	0

SIDT2	0,112685941	0,111872002	0	N.A.	0
LINC01556	0,112624256	0,023252572	0	N.A.	0
CRX	0,112619688	0,09175742	0	N.A.	0
KCND1	0,112569391	0,363750776	0	N.A.	0
RP11-666A2(	0,112565394	0,074632187	0	N.A.	0
IFI16	0,112522131	-0,445403849	0	N.A.	0
EGR3	0,112502592	0,336317759	0	N.A.	0
WDR7	0,112367746	0,246564386	0	N.A.	0
DIP2C	0,11232571	0,164209385	0	N.A.	0
KRBA1	0,112320551		0	N.A.	0
LINC00592	0,112312725	0,227679554	0	N.A.	0
SVOPL	0,112299792		0	N.A.	0
C1R	0,11225928	-0,245126182	0	N.A.	0
C7orf61	0,112231941	-0,050108074	0	N.A.	0
RP11-796E2.4	0,112183072	-0,052185192	0	N.A.	0
SRGAP2C	0,112152412	0,137880184	0	N.A.	0
FBXL18	0,112127691	0,318794737	0	N.A.	0
RP11-442N24	0,11209912	-0,301447231	0	N.A.	0
CTD-2358C21	0,112075123		0	N.A.	0
TBC1D15	0,112014125	0,163186503	0	N.A.	0
AC003991.3	0,112006686	0,147185583	0	N.A.	0
CTF1	0,112003576	-0,157065667	0	N.A.	0
NPB	0,112000781	-0,126787251	0	N.A.	0
TRIM47	0,111880064	0,449409032	0	N.A.	0
EMR2	0,111813169	-0,029552153	0	N.A.	0
ZNF746	0,111741276	-0,049820455	0	N.A.	0
CTC-321K16.	0,111712409		0	N.A.	0
VSTM2L	0,11170118	0,23637657	0	N.A.	0
CXCL5	0,111677832	0,476558003	0	N.A.	0
SNAI3	0,111639126	0,081003244	0	N.A.	0
ARID3A	0,111570648	0,25049956	0	N.A.	0
SLC24A1	0,111564678	0,07560717	0	N.A.	0
MBTPS2	0,111490018	-0,020707435	0	N.A.	0

RP6-109B7.4	0,111488902	0,146631491	0	N.A.	0
GOLGA6L9	0,111420326	0,002096501	0	N.A.	0
FAXDC2	0,111372573	0,534498826	0	N.A.	0
FOLR3	0,111304821	0,170692352	0	N.A.	0
CTC-366B18.	0,111290754	0,091580104	0	N.A.	0
UAP1L1	0,111237081	0,019458526	0	N.A.	0
AF127936.9	0,11120151	0,132390146	0	N.A.	0
NFAM1	0,111198086	0,014887086	0	N.A.	0
CMPK1	0,111170007	0,249218017	0	N.A.	0
CAMSAP1	0,111157464	0,253005447	0	N.A.	0
LY6G5B	0,111079478	-0,037106033	0	N.A.	0
ZNF324	0,111025459	0,093986453	0	N.A.	0
STARD8	0,111010671		0	N.A.	0
RP1-178F10.	0,111009253	0,18479211	0	N.A.	0
TPRA1	0,111003518	0,16445882	0	N.A.	0
VRK3	0,110997996	0,024588384	0	N.A.	0
RP5-999L4.2	0,110934955		0	N.A.	0
LYRM5	0,110923168	-0,225620492	0	N.A.	0
ENTPD3-AS1	0,110917957	-0,219545841	0	N.A.	0
KANK2	0,110894402	0,224410998	0	N.A.	0
RP11-344N10	0,1108702	0,178887874	0	N.A.	0
RP11-138I18	0,110869403	0,176547169	0	N.A.	0
ZNF514	0,11085511	0,242123661	0	N.A.	0
RP11-498C9.	0,110814868	0,240705755	0	N.A.	0
KLC3	0,110808229	0,199779731	0	N.A.	0
FZD1	0,110768076	0,203915975	0	N.A.	0
C1RL-AS1	0,110743706	0,213774826	0	N.A.	0
TTC30A	0,110740258	0,152326596	0	N.A.	0
RHOT1	0,110731031	0,502411025	0	N.A.	0
WISP2	0,110665137	0,28143157	0	N.A.	0
YES1	0,110651273	0,335140435	0	N.A.	0
C1QTNF1-AS	0,110591767	0,034048495	0	N.A.	0
CATSPERB	0,110550509	0,267009826	0	N.A.	0

MT-ND5	0,110546656	0,413780976	0	N.A.	0
LINC01215	0,110522151	-0,225987027	0	N.A.	0
MICU1	0,110467897	0,023146956	0	N.A.	0
PRPH	0,110456963	-0,029024714	1	cls3	0
RP11-147L13	0,11042056	-0,056743171	0	N.A.	0
RP1-67K17.4	0,110317138		0	N.A.	0
SNX24	0,110306916	0,111961084	1	cl1	0
FAM126A	0,110186895	-0,099482543	0	N.A.	0
LONP2	0,11015073	0,242369538	0	N.A.	0
TTC39A	0,110148044	0,134000964	0	N.A.	0
RP11-568A7.	0,11014546	0,150709758	0	N.A.	0
EPHA4	0,110134585	0,29214935	0	N.A.	0
LBX2	0,110098069	0,299018863	0	N.A.	0
RP11-982M1	0,110090428		0	N.A.	0
RP11-186N1	0,110044734	-0,049665458	0	N.A.	0
ZNF784	0,11002972	0,164884005	0	N.A.	0
HES7	0,110026207	-0,190565893	0	N.A.	0
IL17C	0,109902059	0,092848632	0	N.A.	0
RP4-635E18.	0,109873592		0	N.A.	0
FAM122C	0,109819517	-0,145420487	0	N.A.	0
CH17-340M2	0,109781518	0,02421935	0	N.A.	0
SEL1L3	0,109768938	0,376440943	0	N.A.	0
NCK1-AS1	0,109734106	-0,139227533	0	N.A.	0
LARP6	0,109732859	-0,149116019	0	N.A.	0
LLNLR-284B4	0,109732397	0,12667672	0	N.A.	0
TSC2	0,10973198	0,08467362	0	N.A.	0
NLRP2	0,109611522	0,340441781	0	N.A.	0
DSEL	0,109588424	-0,051485765	0	N.A.	0
UTS2	0,10956028	0,000462093	0	N.A.	0
GJC2	0,109546926	-0,063040393	0	N.A.	0
PLD1	0,109500585	0,440396827	0	N.A.	0
BTBD3	0,109466941	0,363047187	0	N.A.	0
GRM3	0,109407347		0	N.A.	0

APBB1	0,109393308	0,109909512	0	N.A.	0
RP11-439M1	0,109390966		0	N.A.	0
PRMT7	0,10938955	0,237990532	0	N.A.	0
DENND2A	0,109388743	-0,203777642	0	N.A.	0
PRRG2	0,109318874	0,405764159	0	N.A.	0
LINC00336	0,109310716	0,200499475	0	N.A.	0
THBD	0,109279629	-0,196812133	0	N.A.	0
RP11-295G2(	0,109268968	-0,131316336	0	N.A.	0
TGFBR3	0,109237459	0,577887011	0	N.A.	0
RP11-334A14	0,109234241	0,107407448	0	N.A.	0
RP11-440I14	0,109181215	0,160934873	0	N.A.	0
ACAD10	0,109125441	-0,003220862	0	N.A.	0
ANKRA2	0,10909963	0,19153614	0	N.A.	0
NIPAL1	0,10909227	0,037413207	0	N.A.	0
HDAC6	0,109077013	0,17152821	0	N.A.	0
SIL1	0,109072497	0,159971902	0	N.A.	0
SOX9	0,109026561	0,35779602	0	N.A.	0
KIAA1715	0,108998383	0,378163821	0	N.A.	0
DNAJB4	0,108985017	0,073203978	0	N.A.	0
KMT2B	0,108971381	0,349075164	0	N.A.	0
TRAPPC8	0,108957657	0,272837283	0	N.A.	0
PLSCR2	0,10893792	0,181206428	0	N.A.	0
CATSPERG	0,108907576	-0,144722632	0	N.A.	0
SBF2-AS1	0,108903773	0,045140777	0	N.A.	0
PER2	0,108888719	0,17943211	0	N.A.	0
ZNF106	0,108875034	0,106019872	0	N.A.	0
C17orf85	0,108854472	0,083048615	0	N.A.	0
RP11-103H7.	0,108806264		0	N.A.	0
IRAK3	0,108800544	-0,344056006	0	N.A.	0
TAPT1	0,108796473	0,107469408	0	N.A.	0
APLP1	0,108782628	0,322221656	0	N.A.	0
RP11-1103G1	0,108771549	-0,299480177	0	N.A.	0
NOXRED1	0,108754143	0,264455404	0	N.A.	0

RP11-269F19	0,108746866	0,072181707	0	N.A.	0
STAT4	0,10868641	-0,037110422	0	N.A.	0
SERF1B	0,108683708		0	N.A.	0
RP11-54A4.2	0,108643778		0	N.A.	0
PCP4L1	0,108587419		0	N.A.	0
SLC39A5	0,108581756	-0,097454087	0	N.A.	0
CTD-2583P5.	0,108569898	-0,027339268	0	N.A.	0
IPO13	0,108528481	0,189627608	0	N.A.	0
ERVH48-1	0,108527971	0,162648886	0	N.A.	0
TRIM23	0,108472873	-0,104032129	0	N.A.	0
CR1	0,108458714		0	N.A.	0
KCND3	0,108442454	0,025019817	0	N.A.	0
ZNF454	0,108390957	-0,079040791	0	N.A.	0
CTD-2035E11	0,108387274	0,199239915	0	N.A.	0
XXyac-YM21(	0,108365837	-0,103122896	0	N.A.	0
ABCA7	0,108310356	0,502199039	0	N.A.	0
RP11-147L13	0,108301517	-0,017779297	0	N.A.	0
AC046143.3	0,10827016	-0,127288378	0	N.A.	0
ATL1	0,108243332	0,177502477	0	N.A.	0
LINC00216	0,108171832		0	N.A.	0
PPP3R1	0,108098078	0,197620609	0	N.A.	0
FBXO31	0,10808609	0,061412217	0	N.A.	0
LRRC8E	0,108029475	0,286511491	0	N.A.	0
CTD-2213F21	0,108018405		0	N.A.	0
ZFAS1	0,10797174	-0,148752577	0	N.A.	0
BCAS4	0,107946716	0,305639977	0	N.A.	0
RP11-380D23	0,107898818	0,240409654	0	N.A.	0
APOOL	0,107886134	0,031246705	0	N.A.	0
SCGB3A2	0,107866793		0	N.A.	0
LINC01473	0,107802817	-0,0185218	0	N.A.	0
LINC01348	0,107754745	0,051258979	0	N.A.	0
TAF4	0,107738279	0,005377915	0	N.A.	0
CTB-25B13.1	0,107661334	0,200394063	0	N.A.	0

CCL5	0,107629186	-0,142211703	0	N.A.	0
PSPN	0,10759682	0,009955173	0	N.A.	0
PVRL3	0,107569189	0,23328223	0	N.A.	0
RP11-108P20	0,107521618	0,284847226	0	N.A.	0
BST2	0,107451971	0,264725103	0	N.A.	0
KDF1	0,107381154	0,460856679	0	N.A.	0
RP3-337H4.9	0,107355137	-0,108311364	0	N.A.	0
PRMT8	0,107327286	-0,174418933	0	N.A.	0
ZNF701	0,107310821	0,345463382	0	N.A.	0
RP11-10C24.	0,107264844	0,112430973	0	N.A.	0
CYB561D1	0,107252458	0,059473266	0	N.A.	0
RP11-168F9.	0,107238906	-0,005169762	0	N.A.	0
LINC00667	0,107237413	-0,306587559	0	N.A.	0
RP5-1112D6.	0,107236928	0,2851716	0	N.A.	0
CEP76	0,107236674	0,052131348	0	N.A.	0
SSTR2	0,107206597		0	N.A.	0
IGLV3-25	0,107168574	-0,103291146	0	N.A.	0
C6orf1	0,107131852	0,326952888	0	N.A.	0
ZNF28	0,107128118	-0,146068514	0	N.A.	0
LA16c-349E1	0,107124881		0	N.A.	0
HIC1	0,10706776	-0,267065536	0	N.A.	0
ATP6V1E1	0,107048092	0,288543986	0	N.A.	0
PIWIL2	0,107032368	-0,081210652	0	N.A.	0
PHF13	0,107018839	0,101404742	0	N.A.	0
FAM131C	0,10697929	0,259673452	0	N.A.	0
RP11-875O1	0,106959852		0	N.A.	0
PCDH19	0,106935524	-0,102648329	0	N.A.	0
TCHH	0,106927726	0,165184646	0	N.A.	0
RP11-745L13	0,106910664	-0,078534737	0	N.A.	0
RP11-798L4.	0,106897941	0,089287259	0	N.A.	0
RP11-505P4.	0,106847331	0,357671948	0	N.A.	0
RAB33A	0,106818619	0,291966829	0	N.A.	0
FAM8A1	0,106808208	0,265230334	0	N.A.	0

CCL3	0,106799455	0,180581727	0	N.A.	0
TXNDC11	0,106777508	0,129412747	0	N.A.	0
PDXP	0,106768683	0,002490779	0	N.A.	0
HPS1	0,106749445	0,108060957	0	N.A.	0
CSF2RB	0,106745676	-0,040083945	0	N.A.	0
EPB42	0,106710994	-0,014272	0	N.A.	0
SNED1	0,106651748	-0,01815087	0	N.A.	0
SGSH	0,106645394	0,207775065	0	N.A.	0
CDR2L	0,106604135	0,39297425	0	N.A.	0
SRGAP3	0,106549744	0,017828297	0	N.A.	0
RP11-3D4.3	0,106539973	0,255234424	0	N.A.	0
ETV7	0,106462095	-0,084921804	0	N.A.	0
ZNF526	0,10641434	0,052858496	0	N.A.	0
PDZK1	0,106368466	0,162523355	0	N.A.	0
COL12A1	0,106349083	-0,276916676	0	N.A.	0
DUSP22	0,106303534	0,132772197	0	N.A.	0
DNAJC1	0,106299614	0,444546175	0	N.A.	0
CLGN	0,106277812	0,159039127	0	N.A.	0
RP11-227G1[illegible]	0,10623965	0,125788388	0	N.A.	0
RP11-379F4.[illegible]	0,106201815		0	N.A.	0
MUCL1	0,106177346	-0,01741135	0	N.A.	0
AC002429.5	0,106167431		0	N.A.	0
RSRP1	0,106112881	0,380620473	0	N.A.	0
POPDC3	0,106104848	0,236762774	0	N.A.	0
TEKT3	0,106084175	0,374153739	0	N.A.	0
ZNF467	0,106062102	0,164359558	0	N.A.	0
CTLA4	0,10604429	0,122569603	0	N.A.	0
ZFYVE16	0,106034511	0,172144725	0	N.A.	0
NBPF15	0,106033983	0,442814913	0	N.A.	0
CARNS1	0,106014595	0,082445827	0	N.A.	0
C18orf8	0,105994537	0,148259817	0	N.A.	0
HSPA12A	0,105988707	0,029434541	0	N.A.	0
VPS8	0,105972604	0,400167804	0	N.A.	0

RAB33B	0,105890273	0,019027055	0	N.A.	0
C2orf72	0,10588963	-0,039264925	0	N.A.	0
GOLGA7	0,105881618	-0,123712052	0	N.A.	0
TBRG1	0,105816379	0,185106347	0	N.A.	0
CD79A	0,105804789	-0,096319916	0	N.A.	0
GANC	0,105723755	0,055172237	0	N.A.	0
RP11-278A23	0,105706824	-0,018479215	0	N.A.	0
RP11-567M1	0,105682756	0,412043719	0	N.A.	0
CYP51A1-AS1	0,105674155	0,029984088	0	N.A.	0
EPCAM	0,105658045	0,393873483	0	N.A.	0
RP11-42I10.1	0,105637167	0,30677839	0	N.A.	0
KLHL38	0,105585409		0	N.A.	0
RP13-131K19	0,105551578	0,010338502	0	N.A.	0
RP5-1024G6.	0,105513127	-0,241054314	0	N.A.	0
CRYBA4	0,105502668	0,049402582	0	N.A.	0
RP11-478J18	0,105499974	0,097631928	0	N.A.	0
CH17-408M7	0,105412559		0	N.A.	0
ZNF609	0,105376797	0,131862342	0	N.A.	0
ZC3H12A	0,105368718	0,377550445	0	N.A.	0
FCHSD2	0,105357338	-0,285740375	0	N.A.	0
KYNU	0,105355274	-0,108589606	0	N.A.	0
WDR11-AS1	0,105332137	-0,012144447	0	N.A.	0
RP11-371F15	0,105331435	-0,004853005	0	N.A.	0
FBXO8	0,105324718	0,056425034	0	N.A.	0
AC004754.3	0,105264613	-0,168946367	0	N.A.	0
ABCA1	0,10520592	0,053755475	0	N.A.	0
MYH11	0,105161678	0,049050603	0	N.A.	0
ANXA6	0,105160003	0,319343648	0	N.A.	0
SLC9C1	0,105155154	-0,083683958	0	N.A.	0
RP11-513M1	0,105117689	0,159985552	0	N.A.	0
ABCA2	0,10510679	0,360450223	0	N.A.	0
AC010761.8	0,105020172	-0,10855882	0	N.A.	0
SRP14-AS1	0,105007509	-0,179572754	0	N.A.	0

RP11-422P24	0,104994425	0,136022448	0	N.A.	0
NBPF10	0,104980488	0,168212943	0	N.A.	0
RP11-329B9.	0,104954715		0	N.A.	0
NAV2-AS3	0,104950172		0	N.A.	0
RP3-393E18.	0,104945507		0	N.A.	0
RP1-79C4.4	0,104935979		0	N.A.	0
RP11-187E13	0,104924355	0,258844189	0	N.A.	0
EPB41L4A	0,104889904	0,11897641	0	N.A.	0
ANKS1A	0,104831964	0,272741137	0	N.A.	0
FPR3	0,104783418	-0,053497345	0	N.A.	0
CCR4	0,104743624	-0,261308659	0	N.A.	0
DCAF10	0,104681655	-0,036660018	0	N.A.	0
PPP1R9A	0,104631482	0,099302475	0	N.A.	0
NLK	0,104622464	-0,049917568	0	N.A.	0
ZNF200	0,104536059	0,026850641	0	N.A.	0
ZBTB33	0,10451804	-0,054563229	0	N.A.	0
LDB1	0,104488334	-0,071186865	0	N.A.	0
TCAIM	0,104484457	-0,276761136	0	N.A.	0
ADRB2	0,104478219	0,089815832	0	N.A.	0
MURC	0,104452562	0,019331958	0	N.A.	0
TTYH1	0,104436407	0,213752203	0	N.A.	0
SIK2	0,104413515	0,030139763	0	N.A.	0
ZNF101	0,104413483	-0,342519098	0	N.A.	0
UPF1	0,104400673	0,230529526	0	N.A.	0
GCNT2	0,104394204	0,016108412	0	N.A.	0
RP11-67L2.2	0,104366974	0,136766487	0	N.A.	0
TP53AIP1	0,104318882	-0,023614322	0	N.A.	0
RTTN	0,104313124	0,206301355	0	N.A.	0
TRIM21	0,104291933	0,109102355	0	N.A.	0
NFKBIL1	0,10426842	-0,183910054	0	N.A.	0
GSE1	0,104257595	0,091125179	0	N.A.	0
FGF3	0,104207925		0	N.A.	0
AC004019.13	0,104204855	0,199857444	0	N.A.	0

NAV1	0,104203754	-0,151490896	0	N.A.	0
SPDYA	0,104167936	-0,201349799	0	N.A.	0
CASC9	0,104166858	-0,079698864	0	N.A.	0
NUAK1	0,104162797	0,008749927	0	N.A.	0
LINC01290	0,104072347	-0,014707639	0	N.A.	0
TMEM134	0,104018757	0,215597376	0	N.A.	0
CTD-2147F2.	0,103969759	0,08213459	0	N.A.	0
CEBPE	0,103966793	0,037257953	0	N.A.	0
CTA-276F8.1	0,103959538	0,013576345	0	N.A.	0
ZNF674	0,103884891	0,356183954	0	N.A.	0
SPI1	0,103865466	-0,215793151	0	N.A.	0
SLC25A25-AS	0,103854054	0,177135393	0	N.A.	0
PSD	0,103777048	0,096668971	0	N.A.	0
TBX15	0,103742835		0	N.A.	0
ZDHHC17	0,103699672	0,019539767	0	N.A.	0
LINC00893	0,10368184	0,115400863	0	N.A.	0
SPON1	0,103669882	0,218852011	0	N.A.	0
NAPRT	0,103598951	0,455787986	0	N.A.	0
PROM2	0,103596406	0,156797057	0	N.A.	0
SMPDL3B	0,103592954	0,215731839	0	N.A.	0
RP11-437B10	0,103568819	-0,045938242	0	N.A.	0
KIAA0391	0,10355025	-0,058218665	0	N.A.	0
RYK	0,103498213	0,225815204	0	N.A.	0
ZNF518A	0,103491009	0,212571563	0	N.A.	0
MID1	0,103468646	0,100409737	0	N.A.	0
RBPMS-AS1	0,103420428	-0,057927653	0	N.A.	0
CCL3L3	0,103401511	0,099120965	0	N.A.	0
SERTAD4	0,103305258	0,03091906	0	N.A.	0
PCYOX1	0,103250714	0,326428391	0	N.A.	0
ADRA2B	0,103238846	0,056556168	0	N.A.	0
RP11-109E12	0,103157831	-0,176578092	0	N.A.	0
WSCD1	0,103130463	-0,001767161	0	N.A.	0
CNTNAP1	0,103095991	0,014959771	0	N.A.	0

SPTB	0,103077316	0,220573294	0	N.A.	0
AKNAD1	0,103075912	-0,06304435	0	N.A.	0
PRKACB	0,103047618	0,070948512	0	N.A.	0
RP11-83A24.	0,10294786	-0,20785138	0	N.A.	0
SLC25A53	0,102947382	0,044678436	0	N.A.	0
WNT10A	0,102937215	0,138092077	0	N.A.	0
RP4-565E6.1	0,102926927		0	N.A.	0
GMDS-AS1	0,102923025	0,051809754	0	N.A.	0
RP11-290D2.	0,102889684	-0,110689557	0	N.A.	0
TRAF3IP2	0,102845946	-0,05741181	0	N.A.	0
CCSER1	0,10275721	0,178419142	0	N.A.	0
CNFN	0,102749593	-0,091186203	0	N.A.	0
RP11-540O1	0,102736225		0	N.A.	0
RP11-378A1	0,10272968	0,005607158	0	N.A.	0
PLEKHG7	0,102657393	0,095375319	0	N.A.	0
ASMT	0,102576009		0	N.A.	0
KIAA2013	0,102560004	0,483610317	0	N.A.	0
CHML	0,102555412	-0,090923991	0	N.A.	0
GRIN3A	0,102548473	0,084818864	0	N.A.	0
CTD-3157E1	0,102484882	0,103576967	0	N.A.	0
CDR2	0,102478123	0,05731986	0	N.A.	0
SEC14L2	0,10245083	0,330908136	0	N.A.	0
TNXB	0,102439496	-0,108850671	0	N.A.	0
MT-ND4	0,102370869	0,367258394	0	N.A.	0
ACRC	0,102361108	0,003027782	0	N.A.	0
RP13-753N3.	0,102201027	-0,113528052	0	N.A.	0
AC004510.3	0,102179979	0,078807592	0	N.A.	0
VPS13A	0,102165966	0,331120563	0	N.A.	0
FAM156B	0,102109552	0,028201655	0	N.A.	0
COL6A3	0,102096207	-0,217730672	0	N.A.	0
PTHLH	0,102093497	0,44211776	0	N.A.	0
PIGQ	0,102086467	0,204304252	0	N.A.	0
C17orf107	0,102006532		0	N.A.	0

DGCR6	0,101983931	-0,20804031	0	N.A.	0
MAP2K7	0,101957186	0,071776967	0	N.A.	0
IGKV6-21	0,101947533		0	N.A.	0
LGALS3BP	0,101947443	0,523328131	0	N.A.	0
RP11-680F20	0,101800986		0	N.A.	0
HORMAD1	0,101791663	0,043898844	0	N.A.	0
RP11-324O2.	0,101767261	0,408074734	0	N.A.	0
ISPD	0,101751297	-0,158006383	0	N.A.	0
RP3-512B11.	0,10174889	0,241185069	0	N.A.	0
KMT2D	0,101647851	0,101033328	0	N.A.	0
QRICH1	0,1016018	0,024219056	0	N.A.	0
CAPZA1	0,101566302	0,215442497	0	N.A.	0
TYROBP	0,101524295	-0,190112153	0	N.A.	0
ZBED9	0,101504717	-0,068146026	0	N.A.	0
CEBPD	0,101485102	0,141084956	0	N.A.	0
RP11-10N16.	0,101478529	0,194761084	0	N.A.	0
LINC00475	0,10146189		0	N.A.	0
MFSD1	0,101457828	0,036182486	0	N.A.	0
SNX11	0,101421648	0,035195576	0	N.A.	0
DCLK1	0,101406524	-0,056663581	0	N.A.	0
DHDH	0,101391521	-0,010879155	0	N.A.	0
SLAMF6	0,101317945		0	N.A.	0
C1orf61	0,101304336		0	N.A.	0
LSINCT5	0,10130374	-0,209743252	0	N.A.	0
PDGFD	0,101278491		0	N.A.	0
TECPR1	0,101278043	0,197114628	0	N.A.	0
ZFP36	0,101232815	0,521803688	0	N.A.	0
DVL3	0,101224929	0,307632169	0	N.A.	0
AC145343.2	0,101143529	0,001093438	0	N.A.	0
TCAF2	0,101131828	0,226908091	0	N.A.	0
TMEM132D	0,101033216		0	N.A.	0
GLIPR1L2	0,101004315	0,243032407	0	N.A.	0
C16orf74	0,100994366	0,010802712	0	N.A.	0

TM9SF1	0,100964918	0,29586853	0	N.A.	0
TADA2B	0,100955954	0,304766347	0	N.A.	0
NBPF19	0,100953038	-0,112686631	0	N.A.	0
CCL7	0,100908249	-0,186745537	0	N.A.	0
BRD2	0,100887086	0,066969911	0	N.A.	0
SH3BP5-AS1	0,100839832	0,223811472	0	N.A.	0
SOX13	0,100826393	0,464157767	0	N.A.	0
RP1-197B17.	0,100605424	0,070345581	0	N.A.	0
PHGR1	0,100563694	-0,088742082	0	N.A.	0
ZDHHC1	0,10055023	0,143477967	0	N.A.	0
AL591893.1	0,100512645	0,146183486	0	N.A.	0
AC114271.2	0,10049914	-0,165164531	0	N.A.	0
GBE1	0,100480962	0,072040049	0	N.A.	0
DYNLL2	0,10047835	0,096817276	0	N.A.	0
RP11-77K12.	0,100463016	0,327646795	0	N.A.	0
RP11-176N1[illegible]	0,100432185		0	N.A.	0
WDR6	0,100426098	0,133608127	0	N.A.	0
RP11-70D24.	0,100419537		0	N.A.	0
CTC-550B14.	0,100409738	0,141985551	0	N.A.	0
HCAR3	0,100324113	0,024274556	0	N.A.	0
PPRC1	0,100315985	0,018858505	0	N.A.	0
AC079466.1	0,100315238	0,20422862	0	N.A.	0
RP11-385F7.	0,100315155	0,074033526	0	N.A.	0
LA16c-60D12	0,100268971	0,034592226	0	N.A.	0
ADPRM	0,100192866	0,281630908	0	N.A.	0
CTC-529P8.1	0,100188333	0,091961746	0	N.A.	0
AC016549.1	0,100171047	0,027793494	0	N.A.	0
GJB2	0,100164661	0,508390304	0	N.A.	0
BAI2	0,100155456	0,02460933	0	N.A.	0
SNAI2	0,100133868	-0,17898299	0	N.A.	0
RP11-78I14.1	0,100106612	0,098657128	0	N.A.	0
FAM47E	0,100089176	0,329047302	0	N.A.	0
AQPEP	0,100007657	0,252979314	0	N.A.	0

SYCE3	0,100003513	0,183762068	0	N.A.	0
MBD5	0,099955032	0,274243651	0	N.A.	0
ATP6V1F	0,099954617	-0,051004103	0	N.A.	0
SCUBE2	0,099912533		0	N.A.	0
WDR47	0,099903183	0,114980313	0	N.A.	0
RP11-356J5.1	0,099866516	-0,138587374	0	N.A.	0
LIX1L	0,099798848	-0,070419472	0	N.A.	0
CTD-2619J13	0,099786703		0	N.A.	0
MED26	0,099769776	0,36579545	0	N.A.	0
ZNF493	0,099755468	-0,076999807	0	N.A.	0
RP11-927P21	0,099692997	0,13498633	0	N.A.	0
RP11-23P13.	0,099663998	-0,053553351	0	N.A.	0
RP11-80H18.	0,099629094		0	N.A.	0
DIRAS2	0,099620944		0	N.A.	0
LRRC3-AS1	0,099617288		0	N.A.	0
ONECUT2	0,099605102	0,394348781	0	N.A.	0
JAZF1	0,09949043	0,285235938	0	N.A.	0
GORASP1	0,09941552	0,06173335	0	N.A.	0
BBOX1-AS1	0,099350032	-0,058138537	0	N.A.	0
CAPN14	0,099341831		0	N.A.	0
WAC-AS1	0,099336195	0,146057373	0	N.A.	0
GPS2	0,099332238	-0,166223811	0	N.A.	0
RP11-56D16.	0,099315068	-0,046155065	0	N.A.	0
LINC00504	0,099281863	0,111036769	0	N.A.	0
ARHGDIA	0,099277615	0,139832435	0	N.A.	0
ARHGAP6	0,099255114	0,171072929	0	N.A.	0
CCL14	0,099246195	0,049523928	0	N.A.	0
NICN1	0,099187096	0,286254452	0	N.A.	0
RP11-61A14.	0,099154871	-0,052196309	0	N.A.	0
RP11-449H3.	0,099142696	-0,233124861	0	N.A.	0
NKPD1	0,099134462		0	N.A.	0
GACAT2	0,099050067	-0,140056111	0	N.A.	0
AC093495.4	0,098967051	0,200950205	0	N.A.	0

FAM209A	0,098880753		0	N.A.	0
LINC00997	0,098875835	0,09466081	0	N.A.	0
LINC01252	0,098866257	-0,133184947	0	N.A.	0
RP11-338N1(	0,0988535		0	N.A.	0
WDR1	0,098807449	0,053342942	0	N.A.	0
ZDHHC21	0,098789681	0,260525782	0	N.A.	0
UBE2F-SCLY	0,098725083	0,035845837	0	N.A.	0
FAM231D	0,098665347	-0,118966107	0	N.A.	0
POPDC2	0,098659467	0,133469637	0	N.A.	0
TMEM47	0,098653169	0,19917539	0	N.A.	0
ABCA10	0,098628635	0,321641699	0	N.A.	0
ZBTB42	0,098603301	0,26902105	0	N.A.	0
CEACAM4	0,098596756		0	N.A.	0
AC079807.2	0,098540133	0,069422794	0	N.A.	0
RP11-96C21.	0,098539317		0	N.A.	0
RP11-631N1(	0,098468138	0,329225934	0	N.A.	0
RP11-23E19.	0,098458075		0	N.A.	0
SPATA41	0,098451969	-0,1894047	0	N.A.	0
NEU4	0,098444539	0,320729923	0	N.A.	0
C10orf113	0,098436851	0,284389163	0	N.A.	0
TTLL1	0,098432316	-0,117567576	0	N.A.	0
RP11-774O3.	0,098377013		0	N.A.	0
RGS3	0,098362146	0,182352064	0	N.A.	0
RP11-302B13	0,098337749	-0,175793959	0	N.A.	0
MKX	0,098280764		0	N.A.	0
SYNJ2BP	0,098250452	0,184035639	0	N.A.	0
KCNQ4	0,09824258	0,15330894	0	N.A.	0
GRAMD1B	0,098238624	0,412969663	0	N.A.	0
CHSY3	0,098161172		0	N.A.	0
SFR1	0,098111482	-0,004154567	0	N.A.	0
RP11-710F7.:	0,097999134	0,062303235	0	N.A.	0
FAM157C	0,09798102	-0,085114177	0	N.A.	0
LRRC8C	0,097976474	0,224777037	0	N.A.	0

HCAR1	0,097960862	-0,01471427	0	N.A.	0
CA14	0,097907207	-0,248965259	0	N.A.	0
C18orf25	0,097902907	0,445192903	0	N.A.	0
RBAK-RBAKD	0,097870489	-0,048946555	0	N.A.	0
KLHL6-AS1	0,097847538		0	N.A.	0
YEATS2	0,097843796	0,341378371	0	N.A.	0
RP11-5C23.1	0,097830147	0,046316698	0	N.A.	0
ATXN2	0,097798928	0,186778429	0	N.A.	0
ZNF155	0,097721422	0,087562981	0	N.A.	0
ZNF320	0,097659645	0,094860825	0	N.A.	0
ZNF441	0,097656516	-0,182796733	0	N.A.	0
ITPKA	0,097654639	0,325971108	0	N.A.	0
RP11-589P10	0,097627639	0,127379221	0	N.A.	0
WWTR1-AS1	0,097623173	0,116479923	0	N.A.	0
RP11-65J21.3	0,097614766	0,159761415	0	N.A.	0
PRRC1	0,097550885	0,136032292	0	N.A.	0
C19orf47	0,097529703	0,052374602	0	N.A.	0
AP4E1	0,097502086	-0,074560097	0	N.A.	0
GIT2	0,097467584	0,043292075	0	N.A.	0
RHOH	0,097452485	-0,045049333	0	N.A.	0
RP11-328C8.	0,097429339		0	N.A.	0
TM6SF2	0,097397416	-0,265612906	0	N.A.	0
TMEM198	0,097369237	0,159735216	0	N.A.	0
LINC00702	0,097335232		0	N.A.	0
RP11-404L6.1	0,097331426	0,247187261	0	N.A.	0
CA9	0,097267992	0,181690418	0	N.A.	0
PPM1L	0,097249685	0,203456785	0	N.A.	0
GGPS1	0,097134777	-0,135707729	0	N.A.	0
MIR181A1HG	0,097117338	-0,193620114	0	N.A.	0
SLC26A11	0,097112169	-0,000382737	0	N.A.	0
PSEN2	0,097100701	0,330571854	0	N.A.	0
CYP3A7	0,097011661	-0,151611442	0	N.A.	0
AKAP5	0,096997136	-0,18002681	0	N.A.	0

TSPAN9	0,096986181	0,083282644	0	N.A.	0
ARHGAP10	0,096923777	-0,100518819	0	N.A.	0
RSG1	0,096922407	0,090955839	0	N.A.	0
RP11-758P17	0,096914762	0,08556891	0	N.A.	0
PRKRIP1	0,096892418	-0,007808882	0	N.A.	0
FOXP2	0,096882034	0,107940279	0	N.A.	0
RP11-318C24	0,096878203	-0,039992241	0	N.A.	0
DNASE1L3	0,096859453		0	N.A.	0
ZFYVE9	0,096805734	-0,104407287	0	N.A.	0
OXCT2	0,096715446	-0,140572428	0	N.A.	0
NFKBID	0,096658995	-0,05341544	0	N.A.	0
C14orf28	0,096588835	-0,019427994	0	N.A.	0
CXorf23	0,096519582	-0,048949466	0	N.A.	0
ADAMTS9-AS	0,096515556		0	N.A.	0
RP11-281P23	0,096415856		0	N.A.	0
ZDHHC11B	0,096406657	0,097595965	0	N.A.	0
SESN3	0,096359903	-0,1153304	0	N.A.	0
IDI2-AS1	0,096338284	0,22108944	0	N.A.	0
PDCD4-AS1	0,0963229	0,164943226	0	N.A.	0
LINC01443	0,096299049	-0,091880549	0	N.A.	0
FOLR1	0,096270084	0,076524243	0	N.A.	0
STON2	0,096239269	0,071861353	0	N.A.	0
ZNF267	0,096118276	0,192451268	0	N.A.	0
BANK1	0,096084965	-0,153705806	0	N.A.	0
RP1-224A6.9	0,096040626	0,050191573	0	N.A.	0
LINC00996	0,096029354	-0,000867714	0	N.A.	0
CLASP2	0,096026023	0,373707242	0	N.A.	0
FZD10	0,095965732		0	N.A.	0
CPNE5	0,095956457	-0,313359938	0	N.A.	0
ETV2	0,095914058	-0,102874635	0	N.A.	0
RP1-278O22.	0,095874448	-0,427920896	0	N.A.	0
C20orf202	0,095826205	-0,045880689	0	N.A.	0
MED23	0,09580757	0,254622418	0	N.A.	0

USP15	0,095794991	0,364141541	0	N.A.	0
NDRG4	0,095789323	0,11320589	0	N.A.	0
RP1-266L20.2	0,095724168		0	N.A.	0
SHF	0,095693445	0,357989945	0	N.A.	0
SAP130	0,095690347	0,141094396	0	N.A.	0
LINC01480	0,095674354	0,003794093	0	N.A.	0
ARG2	0,095578966	0,093566754	0	N.A.	0
CYP26B1	0,09554689	-0,101750622	0	N.A.	0
PATE2	0,095414741		0	N.A.	0
ZNF286B	0,095335943	-0,008335731	0	N.A.	0
ATCAY	0,09532738	0,088828959	0	N.A.	0
RP11-17M16	0,095300686	0,132911638	0	N.A.	0
RP11-225B17	0,095299167	0,002434434	0	N.A.	0
RP11-166P13	0,095298061	-0,029262807	0	N.A.	0
C2orf68	0,095267047	0,09722953	0	N.A.	0
AOAH	0,095258778	0,082724203	0	N.A.	0
C19orf68	0,095221127	0,109020284	0	N.A.	0
NYNRIN	0,095204869	-0,113377563	0	N.A.	0
PIP4K2A	0,095180907	-0,004005936	0	N.A.	0
PBX2	0,095159519	-0,047353632	0	N.A.	0
TMEM106A	0,095153905	0,379087903	0	N.A.	0
PLEKHH1	0,095069615	0,217485323	0	N.A.	0
CTD-2192J16	0,095013305		0	N.A.	0
RP11-367J11	0,094962	0,133054267	0	N.A.	0
RP11-284F21	0,094916458	0,217190039	0	N.A.	0
SHANK3	0,094894863	-0,131522863	0	N.A.	0
RASSF5	0,094883287	-0,12924777	0	N.A.	0
TNS2	0,094877163	-0,024042219	0	N.A.	0
COL21A1	0,094825122		0	N.A.	0
MLC1	0,094807889	0,012971569	0	N.A.	0
RP11-864J10	0,094796731	0,331644082	0	N.A.	0
C1orf54	0,094795593	-0,167333768	0	N.A.	0
ALS2	0,094719234	0,294007372	0	N.A.	0

RP4-657D16.	0,094680891	0,033715252	0	N.A.	0
RP11-510J16	0,094679376	0,224467701	0	N.A.	0
TUBAL3	0,094664836	-0,135137982	0	N.A.	0
DCLRE1C	0,094567401	0,017323961	0	N.A.	0
SPOCK2	0,094557496	-0,167480929	0	N.A.	0
CNOT11	0,09454804	0,229329636	0	N.A.	0
TAF7	0,094524973	0,236458996	0	N.A.	0
AC241377.2	0,094523042	0,165316632	0	N.A.	0
CTD-2350C1	0,094491153	-0,292081791	0	N.A.	0
PHLDA1	0,094485979	0,196077298	0	N.A.	0
MAD1L1	0,094460232	0,431728079	0	N.A.	0
ZNF628	0,094414641	0,387690503	0	N.A.	0
PKNOX1	0,09439508	0,169755917	0	N.A.	0
FCRL2	0,094334264		0	N.A.	0
RP11-563J2.3	0,094323168	0,167018153	0	N.A.	0
IAPP	0,094321195	-0,106595088	0	N.A.	0
LPHN2	0,094296311		0	N.A.	0
GTF2IRD2	0,094290954	0,06130889	0	N.A.	0
RP11-490M8	0,094274477	-0,150022887	0	N.A.	0
PRRX1	0,094260706	-0,260822985	0	N.A.	0
ZKSCAN7	0,094229232		0	N.A.	0
ZNF451	0,094201365	0,194777294	0	N.A.	0
RP11-379F4.	0,094163067		0	N.A.	0
LINC00987	0,094124341	-0,323550475	0	N.A.	0
RIC1	0,093957892	0,242960775	0	N.A.	0
MFN2	0,093941546	0,347961232	0	N.A.	0
RCAN3	0,093926986	0,190225763	0	N.A.	0
TANGO2	0,093908413	-0,06055025	0	N.A.	0
FAIM3	0,093862871	-0,039528233	0	N.A.	0
LRRC29	0,093789817	-0,128870825	0	N.A.	0
RP11-304L19	0,093788338	0,305785978	0	N.A.	0
RP3-453C12.	0,093719579	-0,078590652	0	N.A.	0
RNF31	0,093706532	0,106217832	0	N.A.	0

ITPR1	0,093695241	-0,207155651	0	N.A.	0
SLC39A6	0,09357259	0,378101513	0	N.A.	0
UBR5	0,093543641	0,398916377	0	N.A.	0
TACC1	0,093523286	0,035853563	0	N.A.	0
CCBE1	0,093510222	0,333407989	0	N.A.	0
DPYSL2	0,093492203	0,213784768	0	N.A.	0
KCNN3	0,093309725	0,232658727	0	N.A.	0
PPP4R1-AS1	0,093307819	-0,232991258	0	N.A.	0
PPP6R3	0,093279083	0,131073691	0	N.A.	0
CD81	0,093273554	0,264247267	0	N.A.	0
ATP2A1	0,093263878	0,436874061	0	N.A.	0
LINC00659	0,093256992	0,302646339	0	N.A.	0
TRIM66	0,093219293	0,25682881	0	N.A.	0
APH1B	0,093203765	0,097185842	0	N.A.	0
RP5-884G6.2	0,093193261	-0,121197903	0	N.A.	0
RP11-582E3.	0,093180447	0,149406676	0	N.A.	0
MT-ND2	0,093146633	0,337869925	0	N.A.	0
TP53TG1	0,093126721	-0,263722065	0	N.A.	0
NYAP2	0,093120755	0,063968822	0	N.A.	0
RP11-658F2.8	0,093080771	0,281900007	0	N.A.	0
LILRB1	0,093042375		0	N.A.	0
SRPX2	0,092894913	0,443980867	0	N.A.	0
FAM217B	0,092869333	0,147083089	0	N.A.	0
HLA-DRB5	0,092828417	-0,307095344	0	N.A.	0
PLXDC1	0,092828202	0,263207075	0	N.A.	0
RP11-221J22	0,09280062	-0,27283294	0	N.A.	0
PLLP	0,092787034	0,368969371	0	N.A.	0
IMPA1	0,092741307	0,02489605	0	N.A.	0
RP11-247A12	0,092728383	0,129544719	0	N.A.	0
ATP6V1H	0,092706292	-0,029813322	0	N.A.	0
KIAA1683	0,092693285	0,044507839	0	N.A.	0
AC133644.2	0,092692149	-0,302554371	0	N.A.	0
NDNF	0,092687609	0,006261966	0	N.A.	0

SLCO3A1	0,092669677	-0,073219622	0	N.A.	0
FAM159A	0,092651925	-0,159121197	0	N.A.	0
AKAP11	0,092628071	0,415444161	0	N.A.	0
YIPF5	0,092611992	0,040966738	0	N.A.	0
RP11-362F19	0,092608784	-0,178006667	0	N.A.	0
HNF1B	0,092575012	-0,133120756	0	N.A.	0
POU5F2	0,0925649	-0,081828654	0	N.A.	0
PCBP1	0,092473177	0,273594875	0	N.A.	0
RP11-981P6.	0,092466375	0,202412829	0	N.A.	0
AC093818.1	0,092461122	0,023827408	0	N.A.	0
STAM	0,092406373	0,230585509	0	N.A.	0
RP11-982M1	0,09237926	-0,216440479	0	N.A.	0
LINC01393	0,092370046	0,007207345	0	N.A.	0
SLC8A2	0,092364631	-0,06261757	0	N.A.	0
STX17-AS1	0,09236161	-0,095252123	0	N.A.	0
TP53TG5	0,09235189	-0,147408673	0	N.A.	0
SORBS2	0,092316683	0,199342663	0	N.A.	0
SLC17A4	0,092305531	0,137335578	0	N.A.	0
RP3-414A15.	0,092302902	-0,240831748	0	N.A.	0
SLMO2	0,092284318	-0,078334257	0	N.A.	0
RP11-1008C2	0,092226732	-0,121511642	0	N.A.	0
TRIM26	0,09221793	-0,116870294	0	N.A.	0
RP11-57H14.	0,092214549		0	N.A.	0
NR1H4	0,09221102	0,384339391	0	N.A.	0
ZNF331	0,092197933	0,095520433	0	N.A.	0
IER5	0,092185126	0,330296065	0	N.A.	0
PPP2R3A	0,092176841	-0,092503253	0	N.A.	0
MEG3	0,092136627	-0,001527506	0	N.A.	0
UGT2B17	0,092117317	0,012044961	0	N.A.	0
RP4-773N10.	0,092083325	0,09864648	0	N.A.	0
TSPAN15	0,092081409	0,587690645	0	N.A.	0
LINC01553	0,09202284	0,058641819	0	N.A.	0
SLC25A34	0,091907873	0,050941454	0	N.A.	0

MTCP1	0,09186427	-0,011199851	0	N.A.	0
SYT1	0,091856197	0,274061892	0	N.A.	0
KCTD21	0,09182223	0,184100585	0	N.A.	0
CERK	0,0918042	0,259114926	0	N.A.	0
RP11-555G19	0,091793301		0	N.A.	0
OLFM2	0,091774433		0	N.A.	0
NCS1	0,091695572	-0,210427794	0	N.A.	0
PDE4B	0,09167144	-0,314823815	0	N.A.	0
GRM6	0,091666783		0	N.A.	0
CES3	0,091663966	-0,013236913	0	N.A.	0
RNF122	0,091617335	0,137627635	0	N.A.	0
SNHG12	0,09161424	0,216524425	0	N.A.	0
ENPP5	0,091591143	0,028023958	0	N.A.	0
NAGLU	0,091535388	0,144587869	0	N.A.	0
SUN2	0,09153475	0,410729056	0	N.A.	0
NPHP3	0,091479064	0,122265586	0	N.A.	0
F8A1	0,091457137	0,214059959	0	N.A.	0
RP11-334C17	0,091423993	0,110393714	0	N.A.	0
LINC00444	0,09140303	0,255715234	0	N.A.	0
TMCC2	0,091343687	-0,03839173	0	N.A.	0
INO80B	0,091307876	-0,079394219	0	N.A.	0
PCDHB14	0,091286966	0,247491895	0	N.A.	0
RP11-326G21	0,091286378	0,19221128	0	N.A.	0
AC114803.3	0,09125004		0	N.A.	0
AC007009.1	0,091195155	-0,06573197	0	N.A.	0
RP11-1246C1	0,091181141	-0,028084954	0	N.A.	0
RP11-49I11.1	0,091167109	0,132922428	0	N.A.	0
MMAA	0,091117547	0,140648296	0	N.A.	0
TRPM6	0,091059036	0,072102782	0	N.A.	0
TMEM263	0,091052638	0,073181028	0	N.A.	0
IGJ	0,090968857	-0,286501116	0	N.A.	0
CREB1	0,09095761	0,195860033	0	N.A.	0
C5orf66-AS1	0,090906458	-0,004947558	0	N.A.	0

GYPC	0,090900089	-0,151108494	0	N.A.	0
RP11-415F23	0,090876365		0	N.A.	0
CELSR1	0,090859852	0,235325036	0	N.A.	0
CDIPT	0,090764154	0,452454082	0	N.A.	0
RP11-353N14	0,090729842	0,158756198	0	N.A.	0
HCN2	0,090666966		0	N.A.	0
RP11-622C24	0,090632752		0	N.A.	0
GRK4	0,090574333	-0,177698761	0	N.A.	0
KLC4	0,090557696	0,361984321	0	N.A.	0
ACTR3	0,090535852	0,135100112	0	N.A.	0
TM9SF4	0,090469818	0,127423872	0	N.A.	0
BNIP3	0,090422541	-0,074134763	0	N.A.	0
RMDN3	0,090400946	0,171023048	0	N.A.	0
DRD4	0,090396594	-0,093314446	0	N.A.	0
CYB561D2	0,090356831	-0,142256804	0	N.A.	0
DR1	0,090311515	0,152410479	0	N.A.	0
JAG2	0,090294239	0,088875696	0	N.A.	0
RP11-388M2	0,09028593	0,339071703	0	N.A.	0
C15orf26	0,090278159	0,203086643	0	N.A.	0
SMG6	0,090270198	0,255444861	0	N.A.	0
AC137932.4	0,090267351	-0,032560216	0	N.A.	0
XXbac-BPG15	0,090236902	0,091442305	0	N.A.	0
COG7	0,090222627	0,156114703	0	N.A.	0
PWP2	0,090159188	-0,129471633	0	N.A.	0
CTB-22K21.2	0,090125411		0	N.A.	0
AADAC	0,090106061	0,041975513	0	N.A.	0
RP11-519G16	0,090102713	0,109736402	0	N.A.	0
HAS2	0,090097224	-0,213535198	0	N.A.	0
AC114730.3	0,090084522	0,068286904	0	N.A.	0
NHLRC4	0,090082802	-0,079825225	0	N.A.	0
VSTM5	0,090069198	0,098578308	0	N.A.	0
TP53I13	0,089989998	0,17748129	0	N.A.	0
PRRT4	0,0899541		0	N.A.	0

ZNF222	0,08994147	-0,102355808	0	N.A.	0
OCLM	0,089883821	0,234508776	0	N.A.	0
SRF	0,089865633	-0,112289117	0	N.A.	0
AC093382.1	0,08983884		0	N.A.	0
LAIR1	0,089833645	0,108927063	0	N.A.	0
SLC30A2	0,089814449	-0,108537858	0	N.A.	0
LTBP3	0,089775457	0,221361315	0	N.A.	0
ARHGAP15	0,089746846	-0,28195753	0	N.A.	0
RP11-126K1.	0,089731182	-0,260793314	0	N.A.	0
SLC52A1	0,0896419		0	N.A.	0
RP11-277P12	0,089607352	-0,139010475	0	N.A.	0
DQX1	0,089572976	0,2801829	0	N.A.	0
ZNF790	0,089557201	0,079031292	0	N.A.	0
RP11-809C18	0,089553325		0	N.A.	0
RP11-775D22	0,089533862	0,084769121	0	N.A.	0
CLIC2	0,089525863	0,084211603	0	N.A.	0
CDH16	0,089453078	0,266840285	0	N.A.	0
RP11-1223D1	0,089421378		0	N.A.	0
PLEKHA2	0,089367909	-0,013894759	0	N.A.	0
AC108004.3	0,089331081	-0,101297865	0	N.A.	0
LINC00924	0,08932868		0	N.A.	0
C16orf58	0,089283784	0,248690876	0	N.A.	0
ZNF384	0,089282522	0,147047184	0	N.A.	0
TNRC6B	0,089232156	0,129936857	0	N.A.	0
MMP25	0,089197352		0	N.A.	0
VGLL3	0,089184768	0,100220591	0	N.A.	0
CUL4B	0,089169526	0,087257516	0	N.A.	0
RP11-385F5.4	0,089163501	0,04927704	0	N.A.	0
MMGT1	0,089138812	0,089222033	0	N.A.	0
BHLHB9	0,089082447	-0,309512185	0	N.A.	0
RP11-571M6	0,089065569		0	N.A.	0
SLA	0,089031785	-0,104098465	0	N.A.	0
TMEM102	0,089028551	0,035358284	0	N.A.	0

SLC25A45	0,089013399	0,307488062	0	N.A.	0
RP11-456H18	0,089003947		0	N.A.	0
HIST1H4J	0,089002825	0,036672908	0	N.A.	0
RP11-398H6.	0,088994362	0,044665184	0	N.A.	0
SLC16A2	0,088989724	0,082492167	0	N.A.	0
FBXO3	0,088977043	0,241377787	0	N.A.	0
ECSCR	0,088954393	-0,288492126	0	N.A.	0
RHBDD2	0,088951271	0,42630429	0	N.A.	0
RP4-794H19.	0,088941838	0,231154443	0	N.A.	0
PPP1R3D	0,088908303	0,202205844	0	N.A.	0
SIN3B	0,088897233	0,127841982	0	N.A.	0
RP11-1000B6	0,088866006		0	N.A.	0
RP11-105N13	0,08885184	-0,066011203	0	N.A.	0
GHET1	0,088831991	-0,027739004	0	N.A.	0
EFHC1	0,088819883	0,316296376	0	N.A.	0
RP11-480C16	0,088810376	-0,012294932	0	N.A.	0
DEDD	0,088692136	0,079650793	0	N.A.	0
MUM1	0,088690015	0,175234838	0	N.A.	0
SSPN	0,08868403	-0,031419176	0	N.A.	0
AKT1	0,088676467	0,084205075	0	N.A.	0
ZBTB17	0,088602199	0,317538908	0	N.A.	0
CNNM1	0,088601726		0	N.A.	0
FAM193A	0,088601654	0,203367393	0	N.A.	0
AC074117.10	0,088599194	0,154739032	0	N.A.	0
RP11-303E16	0,088447703	0,099307093	0	N.A.	0
LINC00562	0,088325191	0,023833766	0	N.A.	0
BAIAP3	0,088256585	0,100371118	0	N.A.	0
RP4-809F18.	0,088190748		0	N.A.	0
AC083843.2	0,088141624	0,081092198	0	N.A.	0
ZNF585A	0,088133176	0,052110376	0	N.A.	0
RP13-726E6.	0,088056167		0	N.A.	0
HEXIM1	0,08804971	0,314326913	0	N.A.	0
AC007182.6	0,088038401	0,05803202	0	N.A.	0

ELMO3	0,087990285	0,368942283	0	N.A.	0
PKIA	0,087981869	0,306456693	0	N.A.	0
AC004076.7	0,087940914		0	N.A.	0
AC074212.6	0,087924297	-0,071509212	0	N.A.	0
RNASEK	0,087911407	0,112694045	0	N.A.	0
STAB1	0,087910502	-0,104537075	0	N.A.	0
RP13-317D12	0,087873357	-0,257883807	0	N.A.	0
RP11-574K11	0,08784388	0,047572172	0	N.A.	0
TP53I3	0,087838846	0,489616165	0	N.A.	0
EHD3	0,087834006		0	N.A.	0
PAEP	0,08782431	0,266155687	1	cls4	0
IGKC	0,087756727	-0,229284947	0	N.A.	0
RP4-718J7.4	0,087683166	0,335806186	0	N.A.	0
ZNF821	0,087642118	0,241980927	0	N.A.	0
SWT1	0,087633672	0,28331828	0	N.A.	0
CD27	0,087617158	-0,331685637	0	N.A.	0
PDLIM3	0,087607511	-0,211126415	0	N.A.	0
CELSR2	0,087607051	0,520955162	0	N.A.	0
KCNIP4	0,087605333	0,055128546	0	N.A.	0
ATG2B	0,087599586	0,039155076	0	N.A.	0
CTD-2541M1	0,087545645	0,023444472	0	N.A.	0
ABCB9	0,087538216	-0,070573641	0	N.A.	0
PPP6R2	0,087520376	0,315861931	0	N.A.	0
NAPB	0,087517745	0,239089134	0	N.A.	0
RALGAPA1	0,087497526	0,214652851	0	N.A.	0
RNASE4	0,087486524	0,240299846	0	N.A.	0
ZNF17	0,087349027	-0,153636912	0	N.A.	0
KCNJ2-AS1	0,087301841	0,058749871	0	N.A.	0
MFNG	0,08729431	-0,02713245	0	N.A.	0
CTC-428G20.	0,087256767	0,009285644	0	N.A.	0
RP11-793H13	0,087227167	-0,044359349	0	N.A.	0
JKAMP	0,087224189	0,310026064	0	N.A.	0
APOC4-APOC	0,087210391		0	N.A.	0

LRRC37A	0,087114111	0,176832752	0	N.A.	0
RIPK3	0,087109221	0,071925527	0	N.A.	0
RP11-43F13.4	0,087075087		0	N.A.	0
RP11-77H9.6	0,087069017	0,17837461	0	N.A.	0
GPR146	0,087032524	-0,138177156	0	N.A.	0
CNPPD1	0,087031509	0,402370189	0	N.A.	0
DCTN1	0,087023301	0,243200971	0	N.A.	0
MDGA1	0,086992922		0	N.A.	0
PCP2	0,086856177	0,173254234	0	N.A.	0
ZNF18	0,086800946	0,054377916	0	N.A.	0
FBXO28	0,08676413	0,259105126	0	N.A.	0
FLJ31104	0,086742888	0,089641957	0	N.A.	0
ANKLE1	0,086712962	-0,099094523	0	N.A.	0
RP11-996F15	0,086694627	-0,159945608	0	N.A.	0
CTD-2308N2	0,086605418		0	N.A.	0
LUM	0,086602789	-0,057157046	0	N.A.	0
TRIM7	0,086576763	0,20168667	0	N.A.	0
AC073283.7	0,086412135	0,030190843	0	N.A.	0
FAM229A	0,086404603	0,087221858	0	N.A.	0
BCL2L11	0,086342823	0,056426942	0	N.A.	0
KLF8	0,086337415	0,192542596	0	N.A.	0
XXbac-BPGBP	0,086330619	0,243403246	0	N.A.	0
CYP4F2	0,086310821	0,052404517	0	N.A.	0
NAGS	0,086306243	0,046984453	0	N.A.	0
TK2	0,086295457	0,004985084	0	N.A.	0
SOSTDC1	0,086254077	-0,152449679	0	N.A.	0
RP11-278C7.	0,086246797	-0,134214434	0	N.A.	0
ZNF8	0,086229024	0,096768225	0	N.A.	0
SHANK2-AS3	0,086223626		0	N.A.	0
AP006748.1	0,086222388	0,092188858	0	N.A.	0
ITGA1	0,086219375	0,201001707	0	N.A.	0
KRT36	0,086213169	-0,271760522	0	N.A.	0
TBC1D14	0,086150678	-0,031861891	0	N.A.	0

RP11-575F12	0,086150248	-0,141017923	0	N.A.	0
TUBB3	0,08614024	0,402414906	0	N.A.	0
PAOX	0,08610199	0,259974732	0	N.A.	0
CTC-487M23	0,08608258	0,231171092	0	N.A.	0
RNF216	0,086080626	0,202057175	0	N.A.	0
RP11-1094H2	0,086073739	0,172148608	0	N.A.	0
ZNF311	0,086072881	0,01032232	0	N.A.	0
CTD-2540B15	0,086061359		0	N.A.	0
ASH1L	0,086043946	0,345666657	0	N.A.	0
RNASEH2B-A	0,08602207	-0,376666657	0	N.A.	0
HECTD2	0,085981912	-0,061618154	0	N.A.	0
GJC1	0,085981784		0	N.A.	0
SCGB2A1	0,085964721	0,133748097	0	N.A.	0
RP11-325L7.1	0,085933292	0,114254319	0	N.A.	0
TOP3B	0,085931288	0,135340296	0	N.A.	0
TM4SF18	0,08588832	-0,216322752	0	N.A.	0
ICOSLG	0,085888253	0,015435786	0	N.A.	0
SLC26A4	0,085879933	-0,088653378	0	N.A.	0
ALPK2	0,085874158	0,086666922	0	N.A.	0
CTD-2249K22	0,085872467	0,192819235	0	N.A.	0
PGBD4	0,085862485	-0,114643385	0	N.A.	0
C10orf11	0,085833499	-0,074580671	0	N.A.	0
ZMAT1	0,085832746	0,128903924	0	N.A.	0
RP11-53O19.	0,085815343	0,224544324	0	N.A.	0
AC005534.8	0,085811837		0	N.A.	0
CYGB	0,085784092	0,171875059	0	N.A.	0
RP4-583P15.	0,085724312	0,102780014	0	N.A.	0
RP11-136L23	0,085649847	0,018393901	0	N.A.	0
SGK494	0,085628542	-0,133739965	0	N.A.	0
HUS1B	0,085623133	0,021619	0	N.A.	0
NTN1	0,085611912	0,073406207	0	N.A.	0
IGKV3-15	0,085591888	-0,253438035	0	N.A.	0
SERTAD3	0,085580349	0,14383737	0	N.A.	0

RP11-21L23.3	0,085548137	-0,009004897	0	N.A.	0
RP11-677M1	0,085437124	0,056646305	0	N.A.	0
IGKV1D-17	0,085433589		0	N.A.	0
MR1	0,085410867	0,027741278	0	N.A.	0
RP11-483I13	0,085360892	-0,218615962	0	N.A.	0
AC092171.4	0,085357725		0	N.A.	0
HNF4G	0,085347578	0,484226872	0	N.A.	0
LINC00649	0,085312429	-0,019115412	0	N.A.	0
SEMA3A	0,085263306	-0,018861888	0	N.A.	0
ADAP2	0,085172092	0,216119897	0	N.A.	0
RP4-612B15.	0,085157987	-0,146381739	0	N.A.	0
ADRBK2	0,085139226	0,009532343	0	N.A.	0
RP11-656D10	0,085132586	0,00946484	0	N.A.	0
GOLGA6L2	0,085131092		0	N.A.	0
DAP	0,08512551	0,196880124	0	N.A.	0
ABLIM2	0,08511824	0,100625555	0	N.A.	0
RP11-472M1	0,085109535	0,232150643	0	N.A.	0
CCDC88A	0,085063212	-0,236470151	0	N.A.	0
ASCC1	0,085053679	0,144748572	0	N.A.	0
AC009501.4	0,085031017	-0,015543179	0	N.A.	0
ZNF761	0,084910588	0,117808252	0	N.A.	0
SLC44A1	0,084894422	0,340949623	0	N.A.	0
MIOX	0,084872061	-0,003754033	0	N.A.	0
CENPT	0,084862135	-0,063495482	0	N.A.	0
RAF1	0,084836914	-0,193799463	0	N.A.	0
FAM69A	0,084816413	0,02500404	0	N.A.	0
CTC-236F12.4	0,08475431	0,04208877	0	N.A.	0
ABHD12	0,084739414	0,374167363	0	N.A.	0
RP11-129M1	0,084730579	-0,0239405	0	N.A.	0
CDKL4	0,084659403	0,094095731	0	N.A.	0
TRIM36	0,084648403	-0,208283371	0	N.A.	0
C5AR1	0,084611032	0,129341679	0	N.A.	0
TSPAN5	0,084533283	0,171629463	0	N.A.	0

LCA5L	0,084494466	-0,242515056	0	N.A.	0
ASMTL-AS1	0,084480805	-0,108988347	0	N.A.	0
PPAPDC1B	0,084472333	0,312652617	0	N.A.	0
RP1-224A6.3	0,084446952	-0,006310575	0	N.A.	0
KLHL36	0,084406599	-0,001305725	0	N.A.	0
DSG2-AS1	0,084382		0	N.A.	0
CYBB	0,084349576	0,105690331	0	N.A.	0
RP11-254F7.	0,08433119		0	N.A.	0
KDM3B	0,084280157	0,203477206	0	N.A.	0
LHFPL4	0,084230916	0,072007702	0	N.A.	0
SLC35D1	0,084212503	0,247883691	0	N.A.	0
KLHDC1	0,084211273	-0,201751219	0	N.A.	0
FAM220A	0,084158607	0,183299842	0	N.A.	0
TSNARE1	0,084156357	0,081487051	0	N.A.	0
AAED1	0,084154094	-0,282381533	0	N.A.	0
TMEM135	0,0841447	-0,011560341	0	N.A.	0
PEX14	0,084111333	0,07545512	0	N.A.	0
AC009948.5	0,084072652	0,053585046	0	N.A.	0
LINC00937	0,084051596	0,173980479	0	N.A.	0
RP11-804H8.	0,084040697	0,111115967	0	N.A.	0
USP36	0,084024499	0,144257629	0	N.A.	0
AF165138.7	0,084024011		0	N.A.	0
ZNF468	0,083964865	-0,126790773	0	N.A.	0
FRMPD3	0,083927411	0,131816706	0	N.A.	0
CBLL1	0,083925012	-0,129087686	0	N.A.	0
CYP4F8	0,083897181	0,024369932	0	N.A.	0
SGK3	0,083876006	0,095892118	0	N.A.	0
FBXL14	0,083718272	0,001588989	1	cl1	0
EFNA2	0,083693679	0,069418558	0	N.A.	0
SGSM2	0,083623186	0,460727056	0	N.A.	0
RP11-498C9.	0,083587786	0,003977519	0	N.A.	0
RP11-167N5.	0,083555499		0	N.A.	0
FAM46C	0,083543969	-0,272383098	0	N.A.	0

ARG1	0,083532897		0	N.A.	0
SMIM24	0,083386457	0,107022808	0	N.A.	0
LRP5L	0,083369437	-0,012519727	0	N.A.	0
KAT6B	0,08335566	0,192951058	0	N.A.	0
MT1F	0,083313479	0,033114832	0	N.A.	0
EPHA1	0,083284288	0,095800413	0	N.A.	0
NPHS1	0,083279395	-0,14373812	0	N.A.	0
MTMR12	0,08326004	0,066473341	0	N.A.	0
FCN1	0,083225887		0	N.A.	0
KIAA0907	0,083052224	0,380054763	0	N.A.	0
ZMIZ2	0,083047965	0,191170118	0	N.A.	0
CTD-2540F13	0,083004825	-0,01946257	0	N.A.	0
SLC37A2	0,082994551	0,210026679	0	N.A.	0
RP11-770J1.5	0,082951694	-0,052423544	0	N.A.	0
THAP9	0,08291929	-0,05609181	0	N.A.	0
PRMT9	0,082883427	0,162952192	0	N.A.	0
GZMH	0,08286067	0,238289351	0	N.A.	0
RP11-763B22	0,082826754	-0,012677131	0	N.A.	0
RP5-1063M2	0,082821785	-0,062145088	0	N.A.	0
SPICE1	0,082804187	0,106427949	0	N.A.	0
C10orf107	0,082774979	-0,112121688	0	N.A.	0
ERV3-1	0,08277181	0,06979752	0	N.A.	0
ACCS	0,082767138	-0,028692557	0	N.A.	0
SUMO4	0,082753056	0,315016032	0	N.A.	0
APOBEC3G	0,082751446	-0,21829735	0	N.A.	0
F8A3	0,082741911	-0,204794572	0	N.A.	0
ME1	0,08271852	0,205012172	0	N.A.	0
CTC-241N9.1	0,082629358	-0,066555697	0	N.A.	0
PIGR	0,082615021	0,243199195	0	N.A.	0
MECOM	0,082604036	0,163910319	0	N.A.	0
ARID2	0,082594565	0,30538808	0	N.A.	0
ZNF883	0,082578153		0	N.A.	0
EIF3C	0,082544808	0,040330485	0	N.A.	0

C1orf229	0,082499952	0,160826467	0	N.A.	0
RP4-539M6.2	0,082451728		0	N.A.	0
TRPM4	0,082442907	0,382177206	0	N.A.	0
CCDC24	0,082433278	0,062466859	0	N.A.	0
MAMLD1	0,082382315	0,175462831	0	N.A.	0
TMPPE	0,082368584	-0,083765037	0	N.A.	0
SULF1	0,082303875	-0,327320886	0	N.A.	0
UBAP2	0,082292251	-0,005297463	0	N.A.	0
NUMBL	0,082274583	0,329379861	0	N.A.	0
GREM1	0,082254642	0,183140084	0	N.A.	0
FBXL19	0,082232467	0,073091796	0	N.A.	0
PRRC2B	0,082231242	0,300920472	0	N.A.	0
XXbac-BPG15	0,082204262		0	N.A.	0
BRWD1-AS2	0,082084373	0,073023609	0	N.A.	0
MAP3K14	0,082072135	0,177662745	0	N.A.	0
AL354822.1	0,082066929	-0,198109187	0	N.A.	0
CTD-2310F14	0,08205474		0	N.A.	0
EN1	0,082039852	-0,082610553	0	N.A.	0
FAM171B	0,082038828	0,081015879	0	N.A.	0
NEO1	0,082030486	0,166005146	0	N.A.	0
AREL1	0,081975973	0,235999148	0	N.A.	0
HMCN1	0,081937504		0	N.A.	0
MTMR1	0,081926806	-0,127240135	0	N.A.	0
LIPA	0,081888052	0,040125526	0	N.A.	0
CTD-2288F12	0,081883644	-0,102240773	0	N.A.	0
CTB-1144G6.	0,081758068	0,035879194	0	N.A.	0
LYSMD4	0,081756669	0,040638406	0	N.A.	0
C19orf67	0,081695336		0	N.A.	0
ACKR1	0,081695225	-0,206125357	0	N.A.	0
DMTF1	0,0816769	-0,094993257	0	N.A.	0
TECPR2	0,081674763	-0,018300775	0	N.A.	0
CEMP1	0,081620651		0	N.A.	0
RIC3	0,08161786		0	N.A.	0

ZNF529-AS1	0,081607131	-0,39361017	0	N.A.	0
LPL	0,081604933	-0,149616931	0	N.A.	0
SENP1	0,081586715	-0,207341495	0	N.A.	0
TNFRSF10A	0,081582017	0,363210452	0	N.A.	0
ARHGDIG	0,08153265	-0,08797508	0	N.A.	0
FLVCR2	0,081472326	0,070996339	0	N.A.	0
RP11-256P1.	0,081462965		0	N.A.	0
SEPT5	0,081460591	-0,107859976	0	N.A.	0
KLHL34	0,081458891	0,213184417	0	N.A.	0
TGFBR3L	0,081422848	-0,323679882	0	N.A.	0
PBX1	0,081281591	-0,075112481	0	N.A.	0
RP4-758J18.1	0,081270279	0,332584038	0	N.A.	0
ITFG3	0,08124436	0,45074135	0	N.A.	0
ABHD5	0,081238799	-0,093997384	0	N.A.	0
BTN3A2	0,081235579	-0,153423285	0	N.A.	0
RAC2	0,081228784	-0,253415883	0	N.A.	0
RP11-171I2.2	0,081206207	0,069089612	0	N.A.	0
C2CD2L	0,081202099	0,171594142	0	N.A.	0
LONRF3	0,081194017	0,227781061	0	N.A.	0
AC002306.1	0,081161142		0	N.A.	0
ABCB4	0,081158071	0,294155694	0	N.A.	0
NPPA-AS1	0,081151794	0,055071606	0	N.A.	0
FCER2	0,081143534		0	N.A.	0
UHRF1BP1	0,081142814	0,155080159	0	N.A.	0
SLC13A4	0,081131128		0	N.A.	0
NDOR1	0,081083192	0,047096268	0	N.A.	0
RP11-483L5.1	0,081071705		0	N.A.	0
C5orf22	0,081044158	0,227907815	0	N.A.	0
LYSMD1	0,080988002	0,208506463	0	N.A.	0
CYP1B1	0,080963763	0,003315409	0	N.A.	0
RP11-488L18	0,080923393	0,205871955	0	N.A.	0
LCA5	0,080917292	0,272121214	0	N.A.	0
RP11-3D4.2	0,080890265		0	N.A.	0

RP11-131L12	0,080877937		0	N.A.	0
RP11-499P20	0,08082403	-0,046437056	0	N.A.	0
USP18	0,080735918	0,356693183	0	N.A.	0
RASAL1	0,080721278	0,143401361	1	cl1	0
F2R	0,080661094	0,097888135	0	N.A.	0
RP3-412A9.1	0,080653041	-0,163487585	0	N.A.	0
DNAH9	0,080633039	0,135660183	0	N.A.	0
BTN3A3	0,080606381	0,313744043	0	N.A.	0
C5orf46	0,080521202	0,125606625	0	N.A.	0
FLYWCH1	0,080506516	0,163976108	0	N.A.	0
IGHV3-15	0,080480091	-0,267392012	0	N.A.	0
BLK	0,080474045	0,084516815	0	N.A.	0
ACRV1	0,080441942		0	N.A.	0
RGPD8	0,080418658	0,179519812	0	N.A.	0
RP11-434H6.	0,080388738	-0,209143272	0	N.A.	0
SIGLEC7	0,080384985		0	N.A.	0
ANK3	0,080369998	0,036225686	0	N.A.	0
RP3-406A7.7	0,080369437	-0,243747016	0	N.A.	0
LMCD1	0,080340008	-0,233135213	0	N.A.	0
MAN2B1	0,080326018	0,104628161	0	N.A.	0
LINC00958	0,080285185	0,047323845	0	N.A.	0
LA16c-380H5	0,080282931	0,240897385	0	N.A.	0
ARHGAP39	0,08027672	0,013449849	0	N.A.	0
MBNL1-AS1	0,080256932	-0,140769172	0	N.A.	0
RP11-240G22	0,08018012	0,082587823	0	N.A.	0
IL23R	0,080153267	-0,059431967	0	N.A.	0
PNPLA7	0,080145225	0,359645891	0	N.A.	0
PPIP5K1	0,08014298	0,077906275	0	N.A.	0
RP11-426L16	0,08011092	-0,025234084	0	N.A.	0
PRMT5-AS1	0,080043387	-0,047435869	0	N.A.	0
AKNA	0,080030549	-0,251717732	0	N.A.	0
AC104534.2	0,079992273	0,258798933	0	N.A.	0
RP11-473M2	0,079947249	0,037963205	0	N.A.	0

LINC01426	0,0799203	-0,239379181	0	N.A.	0
SPAG8	0,079869739	0,303081599	0	N.A.	0
SMARCA5-AS	0,07986238	-0,150224493	0	N.A.	0
RP11-87C12.	0,079838163		0	N.A.	0
RP11-286N2	0,079816457	0,11770957	0	N.A.	0
GPR17	0,079767536	-0,007468906	0	N.A.	0
ATP6AP1L	0,079747006	0,314303695	0	N.A.	0
RASL10A	0,07969214	0,062645199	0	N.A.	0
IL34	0,07961424	-0,002938884	0	N.A.	0
RTEL1-TNFRS	0,079566191	0,092807145	0	N.A.	0
NLRC3	0,079557627	0,110160615	0	N.A.	0
ZNF461	0,079554272	-0,074648563	0	N.A.	0
KIAA1211L	0,079515954	0,279016142	0	N.A.	0
ZC3H7B	0,079507526	0,021041982	0	N.A.	0
HCST	0,079494257	-0,101106529	0	N.A.	0
GLTP	0,079443889	0,223697557	0	N.A.	0
RP11-184I16	0,079435884		0	N.A.	0
ZNF518B	0,079346612	0,115653208	0	N.A.	0
PWAR6	0,079336503	-0,105271171	0	N.A.	0
WDR59	0,079313791	0,193661056	0	N.A.	0
PDZD2	0,079257882		0	N.A.	0
WIPI1	0,079190747	-0,055035504	0	N.A.	0
APOBEC3F	0,079172938	0,184929415	0	N.A.	0
SLC6A6	0,079165856	0,125947793	0	N.A.	0
RP11-776H1	0,079140021	0,274507336	0	N.A.	0
TRIM5	0,079099491	-0,125444921	0	N.A.	0
RP11-431K24	0,079075259	0,187324456	0	N.A.	0
ALAD	0,079067063	0,357224843	0	N.A.	0
C15orf65	0,079042941	-0,067272168	0	N.A.	0
DNAJC24	0,079026276	0,060380866	0	N.A.	0
RP11-244H3.	0,07901968	0,035147225	0	N.A.	0
SPCS3	0,07900911	0,053075732	0	N.A.	0
ZNF646	0,07900832	-0,034932928	0	N.A.	0

CFAP54	0,078961499	0,105393635	0	N.A.	0
EXOC3	0,078961032	0,389950413	0	N.A.	0
IGHV3-23	0,078906474	-0,209145521	0	N.A.	0
ABCA9-AS1	0,078902529	0,121360167	0	N.A.	0
CCL4	0,078900096	0,0273175	0	N.A.	0
PHKB	0,078739285	0,3512254	0	N.A.	0
RAB40AL	0,078680042	-0,153867779	0	N.A.	0
CTD-2033C11	0,078653482	0,059109505	0	N.A.	0
RP11-626G11	0,078645818	0,075400901	0	N.A.	0
FAM177B	0,078618962	-0,051121598	0	N.A.	0
RP11-386I8.6	0,078589779		0	N.A.	0
SLC25A43	0,078581189	0,138193889	0	N.A.	0
PURB	0,078571089	0,030100197	0	N.A.	0
H3F3B	0,078570808	0,128408181	0	N.A.	0
CEBPA	0,078553147	0,142383672	0	N.A.	0
SLC25A44	0,078402303	-0,19012057	0	N.A.	0
FOXD3-AS1	0,078400133	-0,037349203	0	N.A.	0
NAIP	0,078397068	-0,160691354	0	N.A.	0
CD9	0,078382981	0,438280124	0	N.A.	0
OPA3	0,078322245	0,109036065	0	N.A.	0
RP3-388N13.	0,078265549		0	N.A.	0
AC006277.2	0,078214747	-0,071428842	0	N.A.	0
IRF5	0,078189612	-0,086339485	0	N.A.	0
RP11-107N15	0,078179036	-0,027293944	0	N.A.	0
LRRC37A3	0,078175306	-0,166305245	0	N.A.	0
TMEM132B	0,07814432	0,190835136	0	N.A.	0
CFAP74	0,078066652	-0,075673826	0	N.A.	0
CLC	0,078042343	-0,256404596	0	N.A.	0
LINC00694	0,07803847		0	N.A.	0
AC007620.3	0,078013557	0,059084412	0	N.A.	0
RP11-505K9.	0,078011698		0	N.A.	0
ERBB4	0,078006141		0	N.A.	0
BMI1	0,077935375	-0,229887596	0	N.A.	0

LAT2	0,077933173	-0,100121264	0	N.A.	0
ZNF488	0,077916803	0,300551948	0	N.A.	0
CDC42EP4	0,077913645	0,146890792	0	N.A.	0
TMEM187	0,077904618	0,20226239	0	N.A.	0
DAB1	0,077887743	-0,05771186	0	N.A.	0
USP8	0,077814578	0,310648236	0	N.A.	0
RP11-45P15.	0,077804996	0,006800455	0	N.A.	0
CD200R1	0,077802843	-0,159696913	0	N.A.	0
RP11-385D1	0,077784181	-0,029673612	0	N.A.	0
RP11-276H7.	0,077749853	0,204968422	0	N.A.	0
RP11-44N21.	0,077748072	-0,12222089	0	N.A.	0
PHF2	0,077684004	0,199113895	0	N.A.	0
MYSM1	0,077682546	0,073878254	0	N.A.	0
ASB12	0,077635282		0	N.A.	0
PAGE5	0,077634423	-0,178047786	0	N.A.	0
CPLX1	0,077617141	-0,135781526	0	N.A.	0
KIAA1407	0,07760987	0,230604411	0	N.A.	0
RNF215	0,077579989	0,281859609	0	N.A.	0
PHC3	0,07751532	0,116835437	0	N.A.	0
RP11-116D1	0,077513395	0,089765202	0	N.A.	0
C19orf80	0,077467495		0	N.A.	0
RP11-297C4.	0,077442519	0,102796747	0	N.A.	0
ATL2	0,077439385	0,350588413	0	N.A.	0
TCEA2	0,077439324	0,233550523	0	N.A.	0
RP11-407N8.	0,077432563		0	N.A.	0
PPFIA4	0,077430361	0,256812644	0	N.A.	0
HCP5B	0,077382559	0,343198712	0	N.A.	0
FAM53B	0,077372543	0,160066198	0	N.A.	0
C9orf147	0,077372204	0,284735731	0	N.A.	0
RHEB	0,077370948	-0,074428534	0	N.A.	0
NLN	0,077348659	-0,09123337	0	N.A.	0
NRDE2	0,077314132	-0,005645325	0	N.A.	0
CCM2	0,077276497	0,171380341	0	N.A.	0

MN1	0,077252824		0	N.A.	0
FABP2	0,077242208	-0,172179646	0	N.A.	0
RP11-434D12	0,077223284		0	N.A.	0
NFATC1	0,077203945	-0,116715081	0	N.A.	0
HRASLS5	0,077186901	-0,084860744	0	N.A.	0
BAALC-AS1	0,077178583	0,100954369	0	N.A.	0
MGARP	0,077143037	0,020502171	0	N.A.	0
AC133785.1	0,077039573	0,324467132	0	N.A.	0
MAS1	0,076984559	0,186979434	0	N.A.	0
NAPEPLD	0,076982766	0,168469494	0	N.A.	0
TRPC7-AS1	0,076919598	-0,247219715	0	N.A.	0
NUDT14	0,076898687	0,101780916	0	N.A.	0
SLC46A1	0,076857626	0,000422703	0	N.A.	0
VPS53	0,076838336	0,20256546	0	N.A.	0
CYP17A1-AS1	0,076833286	0,22907883	0	N.A.	0
ERO1LB	0,076814414	-0,107727916	0	N.A.	0
RNF183	0,076792305	0,346959204	0	N.A.	0
ZNF570	0,076730278	0,240316701	0	N.A.	0
SEC22B	0,07671193	0,033053106	0	N.A.	0
ITPKB	0,076710433	-0,009932543	0	N.A.	0
BET1	0,076641397	0,043251009	0	N.A.	0
COPZ2	0,076589745	0,079958285	0	N.A.	0
RPRM	0,07654126		0	N.A.	0
SERF1A	0,076529716	-0,172128885	0	N.A.	0
MAFA	0,076440541	0,179732487	0	N.A.	0
ZNF750	0,076397623	0,115842729	0	N.A.	0
RP11-596D21	0,076387269	0,017660603	0	N.A.	0
DAGLB	0,076332473	0,050761907	0	N.A.	0
EPB41L3	0,076312908	-0,084663518	0	N.A.	0
TCF4	0,076282344	-0,293747388	0	N.A.	0
AGFG2	0,076260728	0,056944972	0	N.A.	0
ANTXR1	0,076257197	-0,076368387	0	N.A.	0
AC010226.4	0,07620584	0,240812673	0	N.A.	0

RBM27	0,076182646	0,018672808	0	N.A.	0
RP11-572B2.	0,076177861	-0,03847024	0	N.A.	0
MAFB	0,076146192	0,209351069	0	N.A.	0
CTD-3195I5.3	0,076101088		0	N.A.	0
NBPF14	0,076082638	0,355626044	0	N.A.	0
RP11-268G12	0,076080105		0	N.A.	0
ZBTB47	0,076042425	-0,010611322	0	N.A.	0
NAGK	0,07602614	0,090437545	0	N.A.	0
NFX1	0,076022483	0,24917067	0	N.A.	0
TMEM150A	0,076017073	0,292696534	0	N.A.	0
GS1-393G12.	0,075924715		0	N.A.	0
OSBPL7	0,075919493	0,256541175	0	N.A.	0
PISD	0,075917551	-0,057932049	0	N.A.	0
HIST2H2BF	0,075914558	0,002520217	0	N.A.	0
CELSR3	0,075882455	0,041810444	0	N.A.	0
YJEFN3	0,075858084	-0,114874708	0	N.A.	0
TNFSF8	0,07578584	-0,225987027	0	N.A.	0
CCDC30	0,075776781	0,303148854	0	N.A.	0
MYO18B	0,075748189		0	N.A.	0
SLC26A7	0,075719304	0,384058979	0	N.A.	0
SLC36A1	0,075703579	0,248864197	0	N.A.	0
RAB3IL1	0,075697982	0,006860894	0	N.A.	0
LINC01547	0,075695261	0,018619211	0	N.A.	0
XPNPEP2	0,075683453	0,068190864	0	N.A.	0
RP11-483F11	0,075682606	-0,181814791	0	N.A.	0
ZMIZ1-AS1	0,075642479	-0,162231019	0	N.A.	0
NMT2	0,075570113	0,066800272	0	N.A.	0
ABHD13	0,075564261	0,523815065	0	N.A.	0
RP5-1011O1.	0,075548937	0,009567155	0	N.A.	0
TSHZ2	0,075544079	0,151584027	0	N.A.	0
FRMPD1	0,075531748	0,467787713	0	N.A.	0
DDX26B	0,075511877	0,253001611	0	N.A.	0
CHD9	0,075498559	0,118952556	0	N.A.	0

SLC35G2	0,075477733	0,118432464	0	N.A.	0
KISS1R	0,075468803		0	N.A.	0
RHOXF1	0,075430477	0,038842384	0	N.A.	0
LAMA1	0,075404093	0,123167657	0	N.A.	0
SPHK1	0,075377583	0,215590888	0	N.A.	0
ATP8B3	0,075347734	0,058065477	0	N.A.	0
PORCN	0,075340399	0,379189609	0	N.A.	0
IGLL5	0,075303	0,008142199	0	N.A.	0
PPIAL4G	0,075297453	0,128776045	0	N.A.	0
PACRG	0,075246745	0,021921613	0	N.A.	0
LINC00276	0,075222492		0	N.A.	0
RP11-5C23.2	0,075216361	-0,017094967	0	N.A.	0
SIGLEC15	0,075164185	-0,234032755	0	N.A.	0
GNB4	0,075145314	-0,203430869	0	N.A.	0
TRIM31-AS1	0,075134509	0,123240753	0	N.A.	0
NPIPA3	0,075112181	0,206831419	0	N.A.	0
CNTN3	0,075110047	-0,005346951	0	N.A.	0
RP3-394A18.	0,075040811	-0,025998902	0	N.A.	0
NFE2L3	0,075031108	0,161907061	0	N.A.	0
AC062017.1	0,074967069	0,005289217	0	N.A.	0
FRAT1	0,074960963	0,056206519	0	N.A.	0
RP5-1125A11	0,074938133	0,360823818	0	N.A.	0
ASIP	0,074932647	0,004418455	0	N.A.	0
CBWD3	0,074897392	0,062842876	0	N.A.	0
PF4	0,074889544	0,12123127	0	N.A.	0
COL4A2	0,07482664	-0,114964081	0	N.A.	0
PDE8B	0,074778997		0	N.A.	0
GOLT1A	0,074738219	-0,174083824	0	N.A.	0
ZNF852	0,074735772	0,044473391	0	N.A.	0
KCNH2	0,074731724	0,023057619	0	N.A.	0
NACC1	0,07471732	-0,021840265	0	N.A.	0
NIPAL3	0,074681492	0,29728355	0	N.A.	0
ELF2	0,074611055	0,184456847	0	N.A.	0

HMGN4	0,074581253	-0,237362249	0	N.A.	0
RP3-500L14.2	0,074564729	-0,252964371	0	N.A.	0
P2RX7	0,074555752	0,016442452	0	N.A.	0
ZNF730	0,074545694	-0,240987153	0	N.A.	0
KIAA1841	0,074525418	0,303219891	0	N.A.	0
RP4-742C19.	0,074525279	0,019869434	0	N.A.	0
RP11-723O4.	0,074519985	0,264381868	0	N.A.	0
PHLDB3	0,07447712	0,074201441	0	N.A.	0
FAM89A	0,074471404	-0,005071667	0	N.A.	0
FAM3D	0,074451279	0,204792151	0	N.A.	0
MZF1	0,074435674	0,321453759	0	N.A.	0
AC004854.4	0,074341454	0,185387383	0	N.A.	0
IGKV1-8	0,074280677		0	N.A.	0
MAP1S	0,0742351	0,308542646	0	N.A.	0
SH2D5	0,074218674	-0,229621299	0	N.A.	0
CADM1	0,074203159	-0,012935664	0	N.A.	0
ZNF678	0,074178538	-0,133590711	0	N.A.	0
MEIS2	0,074113291	0,224887314	0	N.A.	0
RP11-568A7.	0,074078154	0,349881932	0	N.A.	0
SARM1	0,074057844	-0,034847562	0	N.A.	0
ZNF621	0,074055954	0,342808913	0	N.A.	0
SMUG1	0,074007339	0,117958322	0	N.A.	0
SPHK2	0,073976325	0,172421696	0	N.A.	0
TMEM167A	0,073949495	-0,085617605	0	N.A.	0
ZFP41	0,073944509	0,14890546	0	N.A.	0
LINC01376	0,073943523	0,084621612	0	N.A.	0
PPIP5K2	0,073926625	-0,141598391	0	N.A.	0
ZNF687	0,073919042	-0,040748609	0	N.A.	0
RP11-214K3.	0,073825159	0,052117311	0	N.A.	0
RP11-341G23	0,073817842	0,000518573	0	N.A.	0
C3orf58	0,073704741	0,124851438	0	N.A.	0
TRIM4	0,073704528	-0,057821915	0	N.A.	0
IFIT5	0,07369705	0,017287948	0	N.A.	0

RP11-115C10	0,07368556		0	N.A.	0
RNF220	0,073662376	0,262801037	0	N.A.	0
RP11-130L8.1	0,073646324	0,003424039	0	N.A.	0
HLA-DRA	0,073641048	-0,473620746	0	N.A.	0
ELOVL3	0,073586891	-0,069730469	0	N.A.	0
NOMO3	0,073583621	0,247849928	0	N.A.	0
AC011288.2	0,073574479	-0,099420258	0	N.A.	0
LGALS2	0,073563712	0,167915751	0	N.A.	0
CCDC90B	0,073536456	-0,354636851	0	N.A.	0
MOSPD3	0,073519257	-0,142567445	0	N.A.	0
CTC-543D15.	0,073434337	-0,050075603	0	N.A.	0
CD8A	0,073355292	0,053885187	0	N.A.	0
DNLZ	0,073326679	-0,106661849	0	N.A.	0
C6orf99	0,073319868	-0,054733693	0	N.A.	0
RFX7	0,073310032	-0,164498856	0	N.A.	0
MOG	0,073298717		0	N.A.	0
FBXL5	0,073253748	0,026755219	0	N.A.	0
THAP6	0,073241559	-0,091976096	0	N.A.	0
ZNF19	0,073227275	-0,226466843	0	N.A.	0
PNPLA1	0,073151226		0	N.A.	0
RP4-550H1.7	0,073145279	-0,149220003	0	N.A.	0
PLAG1	0,073139828	0,153748449	0	N.A.	0
AC026188.1	0,073022107	0,221653306	0	N.A.	0
RP11-298J20	0,072998379	0,084472399	0	N.A.	0
S100A8	0,072987094	-0,085862165	0	N.A.	0
ZNF26	0,072984582	0,178904303	0	N.A.	0
RP11-307E17	0,07295188	0,293802749	0	N.A.	0
STX1A	0,072939916	0,452460119	0	N.A.	0
GP1BA	0,072935994	-0,005610392	0	N.A.	0
AC015987.1	0,072932325	0,36909902	0	N.A.	0
RP11-2H3.6	0,072923289	0,258695592	0	N.A.	0
ZNF552	0,072893378	0,114098352	0	N.A.	0
ARAP1	0,072888326	0,157301156	0	N.A.	0

NHEJ1	0,072849697	-0,283952602	0	N.A.	0
BAG3	0,072839731	0,400157152	0	N.A.	0
SCNM1	0,072839341	0,193012902	0	N.A.	0
RP11-1220K2	0,072827739	0,232810494	0	N.A.	0
ZNF483	0,072801092	-0,096315385	0	N.A.	0
RP11-72I8.1	0,072761401	0,043313585	0	N.A.	0
IGHV4-59	0,07271938	-0,210439137	0	N.A.	0
ENDOV	0,072711901	0,01810276	0	N.A.	0
C1QTNF2	0,072689773	-0,239290773	0	N.A.	0
RP11-637O1	0,072684794	0,171832858	0	N.A.	0
FAM20C	0,072684064	0,321482037	0	N.A.	0
PPP1R36	0,072570763	0,011678702	0	N.A.	0
RP5-837J1.4	0,072570578	-0,016235705	0	N.A.	0
NPTX1	0,072522628		0	N.A.	0
AP1G2	0,072520064	0,277812666	0	N.A.	0
TAP1	0,072517128	0,282144155	0	N.A.	0
CTD-2589H1	0,072492424	0,22133668	0	N.A.	0
LINC01285	0,072417356	-0,067673977	0	N.A.	0
ELMO2	0,072382047	0,03142639	0	N.A.	0
TAGAP	0,072358157	-0,003492776	0	N.A.	0
RP4-669L17.	0,072336941	0,066871942	0	N.A.	0
HHAT	0,072277866	0,016861483	0	N.A.	0
S1PR4	0,072276727	-0,212772214	0	N.A.	0
AP003068.9	0,072261145	-0,031998442	0	N.A.	0
IGLC7	0,07225918	0,232464176	0	N.A.	0
MAPK1IP1L	0,072253237	0,12007669	0	N.A.	0
ZNF860	0,072209688	-0,094169264	0	N.A.	0
CTD-2017F17	0,072184629	0,084205757	0	N.A.	0
ZNF548	0,072167904	-0,184127325	0	N.A.	0
CARKD	0,072161523	0,39517598	0	N.A.	0
C20orf24	0,072157107	0,143301379	0	N.A.	0
PI15	0,072089844	-0,033794926	0	N.A.	0
PCDHB15	0,07208601	0,021693875	0	N.A.	0

RP11-631N1(	0,072083299	0,211239837	0	N.A.	0
ZC3H12D	0,072067587	0,241227934	0	N.A.	0
C9orf66	0,072066143	-0,143335435	0	N.A.	0
CNN2	0,072040971	-0,102320618	0	N.A.	0
FILIP1L	0,072036345	0,068263421	0	N.A.	0
ANKRD6	0,072033342	-0,007354161	0	N.A.	0
VWA5A	0,071985961	0,212946959	0	N.A.	0
FAM167B	0,071981799	0,076062312	0	N.A.	0
KIAA1468	0,071962883	0,337201995	0	N.A.	0
HMGA1P4	0,071891264	0,472254829	0	N.A.	0
METTL25	0,071874301	-0,098562755	0	N.A.	0
FAM47E-STB	0,071861366	0,460513542	0	N.A.	0
ASXL2	0,071844962	0,104873569	0	N.A.	0
RP11-946L16	0,071842327		0	N.A.	0
SERPINE1	0,071838524	0,161054976	0	N.A.	0
CERS6	0,071802388	0,417365719	0	N.A.	0
SLC26A6	0,071785903	0,230314302	0	N.A.	0
RP11-884K1(	0,071775839	-0,066064479	0	N.A.	0
SPTLC3	0,071761568	0,025165542	0	N.A.	0
ABCB6	0,071732731	0,157868486	0	N.A.	0
RP11-276H7.	0,071696301	0,179801128	0	N.A.	0
TTLL6	0,071694711	-0,113429854	0	N.A.	0
BNIPL	0,071674272	0,047134007	0	N.A.	0
RNF121	0,071670116	0,2215187	0	N.A.	0
HIST2H2AB	0,071663795	-0,223511591	0	N.A.	0
KANSL1L	0,071649995	0,024870932	0	N.A.	0
STAU2	0,071633739	0,156940432	0	N.A.	0
ATP8B2	0,071604067		0	N.A.	0
ZNF236	0,071599907	-0,25513769	0	N.A.	0
AC116366.5	0,071556343	-0,088758096	0	N.A.	0
SPRED3	0,07154318	0,335242931	0	N.A.	0
NEURL2	0,07151734	0,249683091	0	N.A.	0
POLDIP3	0,071507073	0,370438808	0	N.A.	0

SLC6A7	0,07150604	0,238793933	0	N.A.	0
LY86	0,071490521	-0,116428664	0	N.A.	0
FAM21A	0,071453336	0,235286531	0	N.A.	0
TTC34	0,071444649	0,168422275	0	N.A.	0
TRIM3	0,071442096	0,507148594	0	N.A.	0
CTD-2139B15	0,071419795	-0,26790159	0	N.A.	0
ANKRD24	0,07137634	-0,020792332	0	N.A.	0
DENND5A	0,071327823	-0,043238967	0	N.A.	0
DLEC1	0,071305589	-0,057846169	0	N.A.	0
AC131056.3	0,071275406		0	N.A.	0
RP11-283G6.	0,071238366	-0,039800382	0	N.A.	0
FBXO10	0,071209935	0,030158628	0	N.A.	0
KDM5D	0,071148045	0,030017958	0	N.A.	0
AEBP1	0,071145223	-0,076373348	0	N.A.	0
IGKV1-16	0,071117355		0	N.A.	0
NFKBIB	0,07111701	0,053660805	0	N.A.	0
HLA-DMB	0,071102392	-0,041301473	0	N.A.	0
TMEM178A	0,071101005	0,119278854	0	N.A.	0
ODF3L1	0,071043179	-0,038008442	0	N.A.	0
RP11-348N5.	0,071034926	0,256509466	0	N.A.	0
ASIC3	0,071020267	-0,02982322	0	N.A.	0
SLC4A3	0,070994415	-0,085731317	0	N.A.	0
RP11-866E20	0,07098558		0	N.A.	0
RP11-707G14	0,070879109	-0,08579064	0	N.A.	0
RP11-356C4.	0,070874764	0,106437278	0	N.A.	0
LOX	0,070868829	0,059717182	0	N.A.	0
RP11-14N7.2	0,070841602	-0,091504421	0	N.A.	0
RP11-160O5.	0,070839359	-0,243766387	0	N.A.	0
AP000295.10	0,070825347	0,003684149	0	N.A.	0
STAT5B	0,070787323	0,436927371	0	N.A.	0
HAL	0,070760297	0,051948529	0	N.A.	0
CLIC1	0,070747517	-0,16227017	0	N.A.	0
TCL1A	0,070726392	-0,377631941	0	N.A.	0

CTD-3051D2:	0,070704754	0,350789002	0	N.A.	0
RP11-90E5.1	0,070679568		0	N.A.	0
SEPT4	0,070622595		0	N.A.	0
TNNI1	0,070621391	0,038967506	0	N.A.	0
PROC	0,070594705	-0,04451116	0	N.A.	0
HTR4	0,070540986	-0,100037818	0	N.A.	0
NIN	0,070500783	0,012598566	0	N.A.	0
RBM41	0,070498692	0,329729064	0	N.A.	0
RP11-130C1!	0,070418931		0	N.A.	0
FBXL19-AS1	0,07041051	-0,026755867	0	N.A.	0
SLC4A8	0,070386122	-0,040010255	0	N.A.	0
NOP14-AS1	0,070372984	0,160882221	0	N.A.	0
GGT7	0,070363618	0,131830357	0	N.A.	0
RING1	0,070308182	0,043802826	0	N.A.	0
CYP4A22-AS1	0,070307149	-0,1001894	0	N.A.	0
CACNA2D3	0,070303693		0	N.A.	0
RP5-965G21.	0,070296205	-0,062711259	0	N.A.	0
CDKL3	0,070285465	0,030428954	0	N.A.	0
SH3GL1	0,070266194	0,316568468	0	N.A.	0
SIM2	0,070092846	-0,07301073	0	N.A.	0
ODF2L	0,070090046	0,285084491	0	N.A.	0
AL356585.2	0,070060348	0,087419937	0	N.A.	0
SIGLEC12	0,070042905		0	N.A.	0
AC005264.2	0,070040932		0	N.A.	0
FRMPD2	0,070038107	-0,052088583	0	N.A.	0
CCDC84	0,070011833	0,308075611	0	N.A.	0
PDF	0,070001926	-0,18269102	0	N.A.	0
KLHL3	0,069915294	-0,220481209	0	N.A.	0
KCND2	0,069890037		0	N.A.	0
IGSF1	0,069870841	-0,274724751	0	N.A.	0
WDPCP	0,069860306	-0,00836446	0	N.A.	0
MIA3	0,069821558	0,431299939	0	N.A.	0
MCC	0,069806372	0,006447309	0	N.A.	0

FOXA1	0,069798817	0,113735	0	N.A.	0
SGMS1	0,069795843	0,240112736	0	N.A.	0
IMPAD1	0,069793909	0,273498217	0	N.A.	0
PTPLA	0,069667149	0,039317366	0	N.A.	0
RP11-299H22	0,069647584		0	N.A.	0
MED8	0,069612608	-0,079994461	0	N.A.	0
U2AF1L4	0,069592316	-0,061727452	0	N.A.	0
PHRF1	0,069526671	0,027276976	0	N.A.	0
NFKBIA	0,069504887	0,193770927	0	N.A.	0
LRAT	0,06948199	0,149188972	0	N.A.	0
STC1	0,069434662	0,109863961	0	N.A.	0
KAZALD1	0,069402553	0,128783795	0	N.A.	0
KLHL20	0,069392439	-0,010733512	0	N.A.	0
SKAP1	0,069376474	-0,195778971	0	N.A.	0
RP11-219D15	0,069330429	0,365574284	0	N.A.	0
C1QTNF9	0,069284704		0	N.A.	0
KRT18	0,069275559	0,359484335	0	N.A.	0
JMJD7-PLA2G	0,069254501	-0,026486086	0	N.A.	0
MPDZ	0,069210134	0,114902595	0	N.A.	0
RP11-306G20	0,069188963	0,428055581	0	N.A.	0
AC144652.1	0,069167261	-0,036665656	0	N.A.	0
AKIRIN2	0,069127681	-0,03884025	0	N.A.	0
FZD8	0,069101239	-0,074865994	0	N.A.	0
RP5-1180E21	0,06904536	-0,137152695	0	N.A.	0
ARNT	0,068983352	0,047263614	0	N.A.	0
RP11-439C15	0,068983305		0	N.A.	0
ADORA2A-AS	0,068961003	0,06615666	0	N.A.	0
AMBRA1	0,068939288	0,005827227	0	N.A.	0
GPR125	0,068935813	0,269976463	0	N.A.	0
C5orf30	0,068904508	0,069688685	0	N.A.	0
ACAN	0,06889784		0	N.A.	0
CDHR4	0,068897453	0,033299464	0	N.A.	0
RAVER2	0,06889662	0,046255476	0	N.A.	0

SOCS7	0,06882344	-0,139330797	0	N.A.	0
CFAP70	0,068771513	-0,031379665	0	N.A.	0
C3orf18	0,068769448	-0,022044493	0	N.A.	0
ZFP91	0,068765867	0,075382245	0	N.A.	0
AC097724.3	0,068744136	0,424751393	0	N.A.	0
FKRP	0,068693725	-0,031145446	0	N.A.	0
AC241585.2	0,068690244	0,092853715	0	N.A.	0
HEPHL1	0,068671009	0,057779679	0	N.A.	0
ZEB2-AS1	0,068665969		0	N.A.	0
RP11-785D18	0,0686147	0,025987602	0	N.A.	0
RP11-802E16	0,068576013	0,201207919	0	N.A.	0
RP11-680F8.1	0,068534526	-0,230960244	0	N.A.	0
RP11-334J6.6	0,068507791	0,14117977	0	N.A.	0
RP11-872D17	0,068494039		0	N.A.	0
RP11-45M22	0,068444652	0,358898778	0	N.A.	0
GHRL	0,068390496		0	N.A.	0
XXyac-YX65C	0,068377981	0,302337829	0	N.A.	0
NFATC3	0,068359592	0,127222884	0	N.A.	0
SOGA1	0,068357945	-0,150867366	0	N.A.	0
RP11-511B23	0,068328069	-0,262843891	0	N.A.	0
RP11-701H16	0,068318259		0	N.A.	0
RP11-179K3.	0,068154859	0,028752686	0	N.A.	0
ZNF641	0,068126616	0,126440313	0	N.A.	0
ATN1	0,068090193	0,399112748	0	N.A.	0
RP11-531A24	0,068065514		0	N.A.	0
TKTL1	0,068062193		0	N.A.	0
ERICH2	0,068057817	0,222382019	0	N.A.	0
RP3-324O17.	0,068035315	0,031621702	0	N.A.	0
FAM76A	0,068033524	0,041783571	0	N.A.	0
RP11-496H1.	0,068009659	-0,051662823	0	N.A.	0
AZI2	0,067995716	-0,400032081	0	N.A.	0
STMN3	0,067987071	0,276913998	0	N.A.	0
RP11-11N5.3	0,067982373	-0,117858358	0	N.A.	0

EXOC3L2	0,067981138	0,136627972	0	N.A.	0
GPR183	0,067954838	0,312274306	0	N.A.	0
KCNH8	0,067949426	0,209311646	0	N.A.	0
PSKH1	0,067902288	-0,042883622	0	N.A.	0
CAPNS2	0,067898062		0	N.A.	0
ZNF558	0,067867006	0,07238043	0	N.A.	0
LRRC56	0,067815721	-0,105475779	0	N.A.	0
VPS37D	0,067782675	0,074802422	0	N.A.	0
FGF13	0,067757002	-0,121235483	0	N.A.	0
SLFNL1	0,067752372		0	N.A.	0
REST	0,067746501	0,241312822	0	N.A.	0
ORAI2	0,067715921	0,083938904	0	N.A.	0
KLF7	0,067693322	0,143988714	0	N.A.	0
TTC31	0,067682195	0,273794221	0	N.A.	0
RP11-7F18.2	0,067593054	-0,084673042	0	N.A.	0
LIN7C	0,067572354	0,270363036	0	N.A.	0
EXOC4	0,067570641	-0,034149896	0	N.A.	0
SLC39A11	0,067560553	0,165890997	0	N.A.	0
ACTN1-AS1	0,067525181	-0,041517328	0	N.A.	0
CTB-51J22.1	0,067469122	-0,140944943	0	N.A.	0
MTRNR2L3	0,067462127	0,23918097	0	N.A.	0
SIX4	0,067462018	0,098418771	0	N.A.	0
GS1-124K5.3	0,067413437	0,088383428	0	N.A.	0
U47924.31	0,067395122	-0,126925023	0	N.A.	0
MAP4K1	0,06739268	-0,322186582	0	N.A.	0
KBTBD11-OT	0,067334124	0,064872324	0	N.A.	0
PYHIN1	0,067295027	0,064720984	0	N.A.	0
EPHX3	0,067266019	0,047943544	0	N.A.	0
RAB30	0,067260464	0,06222274	0	N.A.	0
CHST3	0,067259645	0,445034787	0	N.A.	0
DNM3OS	0,067241077	-0,029552105	0	N.A.	0
SERPINB9P1	0,067231882	0,482810453	0	N.A.	0
RP11-663N2	0,067221366	0,048427928	0	N.A.	0

PRDM10	0,067215095	0,207124506	0	N.A.	0
BHLHE40-AS1	0,067169963	-0,240967093	0	N.A.	0
HCG17	0,067168021	0,052935631	0	N.A.	0
NEB	0,06715681	0,049687658	0	N.A.	0
MED22	0,067154383	0,081020549	0	N.A.	0
RP11-84A19.	0,067152497		0	N.A.	0
DYNC2H1	0,067137723	-0,058641008	0	N.A.	0
NPHP4	0,06708406	-0,12237398	0	N.A.	0
FAM71A	0,067063933	0,383258909	0	N.A.	0
C2orf42	0,067053526	0,170019662	0	N.A.	0
LINC01254	0,067006815		0	N.A.	0
NHS	0,066985485	0,214559943	0	N.A.	0
ASB15	0,066972705		0	N.A.	0
C8orf37-AS1	0,066842567	0,128767606	0	N.A.	0
HECW1	0,066831502	-0,291836401	0	N.A.	0
SPAG7	0,066828581	-0,00427233	0	N.A.	0
CTSG	0,06681465	-0,15803553	0	N.A.	0
CSGALNACT1	0,066799899	0,114598185	0	N.A.	0
DNM1P35	0,066766398	0,240488401	0	N.A.	0
CTC-534A2.2	0,066752396	-0,355488821	0	N.A.	0
ZNF541	0,066744182	-0,145976741	0	N.A.	0
FBXO43	0,066740337	0,297885367	0	N.A.	0
ANKRD36B	0,06672513	-0,029415241	0	N.A.	0
IMPG2	0,06672394	0,181331739	0	N.A.	0
KRT10	0,066706991	0,031270327	0	N.A.	0
MPZL2	0,06668836	0,274691473	0	N.A.	0
LIMD1	0,066680287	-0,280977588	0	N.A.	0
AC100830.3	0,066662629		0	N.A.	0
ATE1	0,066661535	0,116523462	0	N.A.	0
RGS5	0,066621168	-0,297550782	0	N.A.	0
A2ML1	0,06660675	0,071140188	0	N.A.	0
RP1-56K13.2	0,066587705	0,331175731	0	N.A.	0
AKT3	0,066581854		0	N.A.	0

PHKA2	0,06657212	-0,151513842	0	N.A.	0
BACH1-AS1	0,066522053	-0,110432137	0	N.A.	0
ZNF525	0,066503738	-0,043749148	0	N.A.	0
LTBP2	0,06645254	0,112033693	0	N.A.	0
LRRN2	0,066447847	-0,102148919	0	N.A.	0
NT5DC3	0,066382001	0,241835225	0	N.A.	0
RP11-286E11	0,066372137	-0,207182157	0	N.A.	0
RP11-506K6.	0,06627562		0	N.A.	0
RP11-34P13.	0,066180262	0,040149845	0	N.A.	0
ZNF706	0,06616267	0,129349212	0	N.A.	0
TNIP2	0,066130814	0,018389691	0	N.A.	0
CD248	0,066108777	-0,114691369	0	N.A.	0
RP11-196G11	0,066095659	0,048671283	0	N.A.	0
RP11-83N9.5	0,066060683	0,201287138	0	N.A.	0
RBSN	0,066012514	0,185018308	0	N.A.	0
BCL2L14	0,066003302	0,026381127	0	N.A.	0
TAPBPL	0,066001647	0,231337076	0	N.A.	0
IGSF9B	0,065943652	-0,040986441	0	N.A.	0
RP11-158K1.	0,06593172		0	N.A.	0
SLC14A2	0,0659288	0,059496054	0	N.A.	0
VMO1	0,065896502	0,337021075	0	N.A.	0
EDIL3	0,065896044	-0,174906786	0	N.A.	0
EMX1	0,065869422	0,023736374	0	N.A.	0
SLC9A5	0,065861229	-0,009519765	0	N.A.	0
RP11-686O6.	0,065851272	0,129800096	0	N.A.	0
RP11-251M1	0,065849173		0	N.A.	0
IL2RB	0,065841344	-0,340559857	0	N.A.	0
KITLG	0,065771739	0,233459119	0	N.A.	0
TRIM60	0,065728464	0,208515838	0	N.A.	0
GET4	0,065723017	0,143817409	0	N.A.	0
HOXC11	0,065709091	0,146662553	0	N.A.	0
CTD-3126B10	0,06569413	-0,104490748	0	N.A.	0
SARAF	0,065685175	0,179472945	0	N.A.	0

BFAR	0,06568209	0,087199444	0	N.A.	0
KANSL1-AS1	0,065669548	-0,266397117	0	N.A.	0
RP11-714G18	0,065662451		0	N.A.	0
EREG	0,06565167	0,385393707	0	N.A.	0
PSMD5-AS1	0,065637065	0,36697219	0	N.A.	0
VSIG4	0,065634146	-0,273115128	0	N.A.	0
HIATL2	0,06559947	0,042528257	0	N.A.	0
RP5-1061H20	0,065591374	-0,031975079	0	N.A.	0
RP11-536K7.	0,065536562	-0,338316193	0	N.A.	0
SLC16A9	0,065503043	-0,106302693	0	N.A.	0
HHIPL1	0,065500671		0	N.A.	0
FNDC5	0,065494955	-0,008670373	0	N.A.	0
SUSD2	0,065488598	0,175777827	0	N.A.	0
ENO3	0,065473549	-0,026069172	0	N.A.	0
PAN2	0,06547158	0,015372844	0	N.A.	0
XRRA1	0,065426761	0,047397408	0	N.A.	0
CNOT8	0,065335316	0,148790191	0	N.A.	0
SBSN	0,065324503		0	N.A.	0
RP3-460G2.2	0,065312908	-0,232745866	0	N.A.	0
ANKS4B	0,065286467	0,056531795	0	N.A.	0
SLX4	0,065267925	0,146792817	0	N.A.	0
RP11-217B1.	0,065258696	0,087689766	0	N.A.	0
CHRNB4	0,065246306	-0,006304325	0	N.A.	0
RP11-322E11	0,065236005	0,175349885	0	N.A.	0
RP11-415F23	0,065231826		0	N.A.	0
ORAI1	0,06521598	-0,19990619	0	N.A.	0
U47924.32	0,065207225		0	N.A.	0
WTIP	0,065166327	-0,083631679	0	N.A.	0
MMRN2	0,065128477	-0,291133764	0	N.A.	0
ASAP1	0,065122509	0,081685784	0	N.A.	0
SNX14	0,065082191	0,527422879	0	N.A.	0
SENP5	0,065050311	0,039927988	0	N.A.	0
KIAA0319L	0,065029782	0,208895252	0	N.A.	0

RP11-989E6.	0,064962405	-0,004815863	0	N.A.	0
MAP4K5	0,064958212	0,109960394	0	N.A.	0
RP11-260M2	0,064907969	0,115780489	0	N.A.	0
MT-CO2	0,064896057	0,287923986	0	N.A.	0
CTD-2319I12	0,064884037	0,006902172	0	N.A.	0
FLT1	0,064856718	-0,149155506	0	N.A.	0
RP4-761J14.1	0,064850647	-0,006556062	0	N.A.	0
RP11-48B3.4	0,0648044	-0,091115448	0	N.A.	0
RP11-278L15	0,064798677	0,428641447	0	N.A.	0
ADAT3	0,064791992	-0,032831006	0	N.A.	0
CTD-2526A2.	0,064719864	0,044112527	0	N.A.	0
STK24-AS1	0,064666272	0,019019831	0	N.A.	0
RP11-414H1	0,064658581		0	N.A.	0
LINC00885	0,064653714		0	N.A.	0
SEPT7-AS1	0,064632104	-0,013291952	0	N.A.	0
ADO	0,064620557	0,303220984	0	N.A.	0
CYP46A1	0,064620442	0,138783187	0	N.A.	0
ZNF34	0,064613047	-0,294831665	0	N.A.	0
AC016831.7	0,064596538	-0,116030328	0	N.A.	0
NRN1	0,064536842	-0,23391102	0	N.A.	0
RP11-706O15	0,064531365	-0,292624494	0	N.A.	0
RP11-10K16.	0,064500386	0,044365387	0	N.A.	0
RP11-457M1	0,064452364	0,069441296	0	N.A.	0
VPS28	0,064430316	-0,040265892	0	N.A.	0
INTS1	0,064428036	0,36671861	0	N.A.	0
NLRX1	0,064423393	-0,136771522	0	N.A.	0
ISLR	0,064411276	-0,10746874	0	N.A.	0
XXbac-BPG15	0,064372577		0	N.A.	0
ZBED4	0,0643723	0,144435803	0	N.A.	0
RP11-79O8.1	0,064371151	-0,009969889	0	N.A.	0
TBC1D13	0,064270518	-0,172040431	0	N.A.	0
RP13-890H12	0,064259914	-0,149926563	0	N.A.	0
ANGPT2	0,064178929	0,045729649	0	N.A.	0

PRDM16	0,064128923	0,187465879	0	N.A.	0
OSBPL10-AS1	0,064098359	0,151112512	0	N.A.	0
PLAGL2	0,064097343	-0,187438623	0	N.A.	0
RP11-584P21	0,064079922		0	N.A.	0
TSG101	0,064066841	0,371589516	0	N.A.	0
CTC-459F4.1	0,064039149	-0,142064896	0	N.A.	0
ZNF606	0,06403716	0,155768779	0	N.A.	0
LINC00589	0,064011829	0,205659038	0	N.A.	0
WDFY3	0,064009249	0,280889224	0	N.A.	0
POU6F2	0,064005304	0,018768785	0	N.A.	0
TNFRSF10C	0,064004398	-0,261236724	0	N.A.	0
ZNF517	0,063993827	-0,037279489	0	N.A.	0
TTC6	0,063919622	-0,087482772	0	N.A.	0
ZNF707	0,063866296	0,098149878	0	N.A.	0
RP11-92K15.	0,063863049	0,110406996	0	N.A.	0
MLXIP	0,063803828	0,203370241	0	N.A.	0
XXbac-BPG18	0,063799313	-0,202542673	0	N.A.	0
ALDH1L1	0,063781394	0,08840037	0	N.A.	0
CTD-2235C13	0,063777892	0,228732558	0	N.A.	0
IGF2BP2-AS1	0,063773651	-0,049080404	0	N.A.	0
RP11-761E20	0,063711793	0,174409573	0	N.A.	0
C19orf26	0,063708285		0	N.A.	0
FAM71F2	0,063701476	0,314445042	0	N.A.	0
FAM9C	0,063654007		0	N.A.	0
GDE1	0,063653483	-0,090613193	0	N.A.	0
GPATCH1	0,06353249	-0,036094713	0	N.A.	0
RP11-474P2.	0,063482712	0,385104678	0	N.A.	0
STX16	0,063415913	0,038050149	0	N.A.	0
ACAP3	0,063385518	0,367629617	0	N.A.	0
CCL4L1	0,063382998	-0,017336411	0	N.A.	0
COX17	0,063356191	-0,168613348	0	N.A.	0
KCNJ2	0,063348939	0,068895749	0	N.A.	0
ZNF140	0,063344673	-0,183178226	0	N.A.	0

RP11-32B5.7	0,063285006		0	N.A.	0
RP11-47J17.2	0,063259456		0	N.A.	0
CD4	0,063250093	-0,039516773	0	N.A.	0
C11orf45	0,063201346		0	N.A.	0
RP11-410D17	0,063161337	-0,107892026	0	N.A.	0
YTHDC1	0,063130737	0,427429121	0	N.A.	0
LGSN	0,063080262	0,107276586	0	N.A.	0
NPIPB5	0,063062883	0,180935372	0	N.A.	0
MIB1	0,063053781	0,213360786	0	N.A.	0
ZNF282	0,063046831	0,302396147	0	N.A.	0
MAGIX	0,062997261	-0,103316758	0	N.A.	0
AMELX	0,062991525		0	N.A.	0
RP11-33O4.1	0,062968267	0,184974106	0	N.A.	0
RAB37	0,062964674	0,221139509	0	N.A.	0
HSPA1L	0,062960118	-0,188863296	0	N.A.	0
ZNF266	0,062957158	0,120133932	0	N.A.	0
CTBP1-AS	0,06294884	-0,028052569	0	N.A.	0
REPS2	0,062935386	0,162031607	0	N.A.	0
LRFN4	0,062912837	0,196719111	0	N.A.	0
NFATC2	0,062881002	0,281063334	0	N.A.	0
SNHG18	0,062879263	0,202404279	0	N.A.	0
IGHV1-2	0,062815633	-0,08162501	0	N.A.	0
DNAH3	0,062773295	-0,075870093	0	N.A.	0
BSCL2	0,062766742	-0,319817245	0	N.A.	0
ISM2	0,062738545	0,159337532	0	N.A.	0
NFASC	0,062717345	-0,101117178	0	N.A.	0
MIR155HG	0,062705164	0,037174111	0	N.A.	0
FAM168A	0,062684543	-0,060174296	0	N.A.	0
PATL2	0,062681537	0,170880189	0	N.A.	0
USP2	0,062679588	-0,061922604	0	N.A.	0
GUCY2D	0,062662954	0,084390405	0	N.A.	0
RP11-141O11	0,062619777		0	N.A.	0
UTY	0,062608808	-0,053543498	0	N.A.	0

IGKV1-12	0,062596612	0,202197214	0	N.A.	0
RP4-665J23.1	0,062594534	-0,22812405	0	N.A.	0
RP11-473I1.5	0,062563514	-0,200577659	0	N.A.	0
CNOT6L	0,062551328	-0,110331414	0	N.A.	0
RP11-274B21	0,062494857	0,242187566	0	N.A.	0
ZNF571	0,062489217	0,164308601	0	N.A.	0
RP11-299H21	0,062477655	0,15528828	0	N.A.	0
SYT15	0,062456439	-0,124510033	0	N.A.	0
MYLPF	0,062442108	-0,298097696	0	N.A.	0
LINC01125	0,062438857	-0,045667918	0	N.A.	0
SPRY2	0,062432484	0,306413247	0	N.A.	0
AC104667.3	0,062427145	0,075830175	0	N.A.	0
PRX	0,062410451	-0,131366599	0	N.A.	0
TMEM249	0,062406868	-0,157337632	0	N.A.	0
LINC01213	0,062405816	-0,264458048	0	N.A.	0
BEX5	0,06237728	-0,079833697	0	N.A.	0
LA16c-444G7	0,062335832	-0,137017836	0	N.A.	0
GATA6	0,062326562	0,168116253	0	N.A.	0
KLRD1	0,062316658	-0,124470766	0	N.A.	0
CTD-2325P2.	0,062290838		0	N.A.	0
MT1H	0,062252897	-0,111342941	0	N.A.	0
STAP1	0,062237017	-0,088758096	0	N.A.	0
KCNN4	0,062231401	0,478891023	1	none	0
WNT5A	0,062167216	0,029874313	0	N.A.	0
WBP2NL	0,062158071	0,020743421	0	N.A.	0
RP11-57A1.1	0,062117505		0	N.A.	0
ING3	0,062096313	-0,243593299	0	N.A.	0
RP11-35G9.3	0,062055908	0,111788104	0	N.A.	0
TINF2	0,062047358	-0,052833381	0	N.A.	0
ZSCAN20	0,062030093	0,078943372	0	N.A.	0
MARCH1	0,062023631	-0,358556114	0	N.A.	0
RP11-365H22	0,061999977	0,356095881	0	N.A.	0
ABHD16A	0,061932705	-0,227006042	0	N.A.	0

STMN4	0,061919115	0,034357583	0	N.A.	0
LINC00964	0,061914709		0	N.A.	0
ACSM1	0,061892725	-0,131419833	0	N.A.	0
ZNF624	0,061891547	-0,115200358	0	N.A.	0
ANKHD1-EIF4	0,061882532	0,029022788	0	N.A.	0
ATP2A3	0,061844909	0,037390073	0	N.A.	0
DDR2	0,061841249	0,00644587	0	N.A.	0
RP11-635N19	0,061816847	0,173555269	0	N.A.	0
ZNF471	0,061814568	0,185056462	0	N.A.	0
SMG5	0,061805784	0,227659347	0	N.A.	0
GRIK2	0,061789108		0	N.A.	0
RP11-38L15.2	0,061762397	-0,06289385	0	N.A.	0
ASPHD2	0,061660393	0,366113771	0	N.A.	0
PCDHB13	0,061589052	0,315446797	0	N.A.	0
HOXD10	0,061586054	0,123909979	0	N.A.	0
ITGAL	0,061584003	0,060026507	0	N.A.	0
RP11-709A23	0,061566543		0	N.A.	0
TCP11L1	0,061561292	0,009911015	0	N.A.	0
RP11-20G13.	0,061546421	0,315932116	0	N.A.	0
RP11-16K12.	0,061441381		0	N.A.	0
RP13-192B19	0,061441026	-0,094570511	0	N.A.	0
RBM14-RBM	0,061434003		0	N.A.	0
FAM206A	0,061424018	-0,069640854	0	N.A.	0
C6orf47	0,061387835	0,223534153	0	N.A.	0
PTPN7	0,061366151	-0,230766425	0	N.A.	0
GRM7	0,061363625	-0,149720762	0	N.A.	0
AC245100.1	0,061346573	0,016123831	0	N.A.	0
TRPV2	0,061316249	0,168655484	0	N.A.	0
CH507-9B2.9	0,061302585	-0,027301095	0	N.A.	0
NBEA	0,061268247	0,098859539	0	N.A.	0
HIVEP3	0,061240811	0,048256535	0	N.A.	0
ARID1A	0,061225119	0,404492992	0	N.A.	0
MYBPC3	0,061143878	-0,006546293	0	N.A.	0

POSTN	0,061080539	-0,123856803	0	N.A.	0
OSBPL5	0,061076728	0,25722085	0	N.A.	0
RP11-51J9.6	0,061056543	0,255524516	0	N.A.	0
DNAJC27-AS1	0,061048366	0,006827928	0	N.A.	0
PLEKHS1	0,061041776	-0,046459506	0	N.A.	0
TRIM11	0,061027299	0,146635881	0	N.A.	0
RAB38	0,061017223	-0,053305005	0	N.A.	0
LINC00282	0,060970086	0,142284428	0	N.A.	0
ZNF776	0,060938877	0,297211665	0	N.A.	0
PWWP2A	0,060873513	-0,030551037	0	N.A.	0
RP1-45I4.3	0,060795804	0,145193926	0	N.A.	0
CDIP1	0,06076203	-0,043738562	0	N.A.	0
CCNJ	0,060761256	0,137320512	0	N.A.	0
RP11-661P17	0,060759143	0,024211582	0	N.A.	0
IGLV1-44	0,060749142	-0,195763393	0	N.A.	0
RP11-455F5.4	0,060739631	-0,060307131	0	N.A.	0
SYNE3	0,060675368	-0,033547877	0	N.A.	0
TMC8	0,06066222	0,321393478	0	N.A.	0
ZNF148	0,060625059	0,140966863	0	N.A.	0
USH1G	0,060596875		0	N.A.	0
LINC01063	0,06053195	-0,10699011	0	N.A.	0
CTD-3222D19	0,060527157	0,157195292	0	N.A.	0
H2AFJ	0,060512132	0,034082634	0	N.A.	0
AC113617.1	0,060501733	0,450226896	0	N.A.	0
CAMKK2	0,060417284	0,158353858	0	N.A.	0
OGFR-AS1	0,060407738		0	N.A.	0
ZBTB40	0,06039031	0,140432253	0	N.A.	0
C10orf25	0,06037542	-0,014702695	0	N.A.	0
ARSH	0,060343625	0,137780765	0	N.A.	0
GNA12	0,060335884	-0,25994647	0	N.A.	0
ARF3	0,060246394	0,562077251	0	N.A.	0
C7	0,060227643	-0,113860331	0	N.A.	0
ANKRD23	0,060225666	-0,033125607	0	N.A.	0

KCNK17	0,060216872		0	N.A.	0
RP11-28H5.2	0,060214439	-0,062544707	0	N.A.	0
TNFRSF4	0,060211558	-0,151567731	0	N.A.	0
FNDC1	0,060205199		0	N.A.	0
RP11-2B6.3	0,060203111	-0,001263896	0	N.A.	0
RP11-83M16	0,060163117		0	N.A.	0
RP1-261D10.	0,060140859	-0,290855772	0	N.A.	0
RP11-524O1.	0,060044388		0	N.A.	0
DLG4	0,060022562	-0,214676653	0	N.A.	0
FEZ1	0,06000996		0	N.A.	0
ST3GAL2	0,059994895	0,192625549	0	N.A.	0
PEX12	0,059986585	-0,054099605	0	N.A.	0
MTRNR2L11	0,059947108	-0,026219658	0	N.A.	0
RP11-90B9.3	0,059916344		0	N.A.	0
NPFFR1	0,05987262	-0,097793087	0	N.A.	0
RP11-439E19	0,059860564	0,201430451	0	N.A.	0
COG1	0,059839837	0,233633	0	N.A.	0
RP3-522J7.7	0,059835117		0	N.A.	0
RP11-156K23	0,059831239	0,038234268	0	N.A.	0
RP11-157P1.	0,05982569	0,163636511	0	N.A.	0
ZNF785	0,059723129	0,039254974	0	N.A.	0
RP1-317E23.	0,059682497	-0,043563554	0	N.A.	0
OSGIN2	0,059663979	0,091758648	0	N.A.	0
METTL4	0,059659572	-0,013266688	0	N.A.	0
PIK3R4	0,05965518	0,218802735	0	N.A.	0
CTB-147N14.	0,059610248	-0,188103969	0	N.A.	0
CTB-25B13.5	0,05955417	-0,220272986	0	N.A.	0
TET3	0,059547023	0,124131417	0	N.A.	0
PLBD2	0,059517994	0,097671364	0	N.A.	0
ZNF469	0,059473255	0,143826072	0	N.A.	0
FAM120A	0,059470548	0,218283266	0	N.A.	0
XBP1	0,05944021	0,051046832	0	N.A.	0
RTP4	0,059407989	0,047399063	0	N.A.	0

RP11-69E11.	0,0593884	-0,123448445	0	N.A.	0
IKZF5	0,059357927	0,013669278	0	N.A.	0
OCIAD2	0,059246424	0,240959564	0	N.A.	0
B3GALNT2	0,059246287	-0,08658514	0	N.A.	0
DOCK8	0,059181476	-0,122414467	0	N.A.	0
RP1-151F17.	0,05915892	0,040993305	0	N.A.	0
SYT13	0,059138419	0,127298534	0	N.A.	0
AP001055.6	0,059070952		0	N.A.	0
WDR93	0,059057871	-0,071614951	0	N.A.	0
VWDE	0,059044367	-0,189198108	0	N.A.	0
CTC-526N19.	0,059018471		0	N.A.	0
PRKAR2A-AS	0,059015736	0,471308692	0	N.A.	0
ZMYND10	0,058948742	-0,004950152	0	N.A.	0
CCDC159	0,058881096	0,12705033	0	N.A.	0
A1BG	0,058868861	-0,061307593	0	N.A.	0
AC002331.1	0,058845279		0	N.A.	0
HRC	0,058806804	0,069958372	0	N.A.	0
PIP5K1A	0,058754875	0,289304475	0	N.A.	0
EGR2	0,058746668	-0,13582997	0	N.A.	0
CAMK2G	0,058745211	0,505029918	0	N.A.	0
GSDMA	0,05870715	0,014019132	0	N.A.	0
NBPF9	0,058699641	0,244270781	0	N.A.	0
RP5-1112D6.	0,058696195	0,16016409	0	N.A.	0
RP3-402G11.	0,058667288		0	N.A.	0
LINC00852	0,058586242	-0,195954362	0	N.A.	0
ZBTB6	0,058504874	0,333306414	0	N.A.	0
DNAAF1	0,058470942	0,269266402	0	N.A.	0
SEMA3D	0,058451865	0,055840399	0	N.A.	0
PAX8	0,058444862	0,523013926	0	N.A.	0
RAB6C	0,058429619	0,064918745	0	N.A.	0
CD96	0,05840909	-0,164773143	0	N.A.	0
FAM103A1	0,058402966	0,096539663	0	N.A.	0
PLEKHG5	0,058392641	0,04296927	0	N.A.	0

RP11-173D3.	0,058364362	-0,106308259	0	N.A.	0
RP11-73E17.	0,058336777	0,019929879	0	N.A.	0
NFIB	0,058309382	0,092129757	0	N.A.	0
XXyac-YRM2(	0,058285145	0,069114157	0	N.A.	0
AP000569.9	0,058274965	-0,229081613	0	N.A.	0
PSAPL1	0,058177018	0,317188132	0	N.A.	0
SUPT7L	0,058157564	0,016937256	0	N.A.	0
RP11-138I1.4	0,058156385	0,07203622	0	N.A.	0
TSNAXIP1	0,058123865	-0,036318479	0	N.A.	0
AC022201.5	0,058113799	0,088917406	0	N.A.	0
LA16c-358B7	0,058100614	0,304325948	0	N.A.	0
LINC00936	0,058090657	0,089142067	0	N.A.	0
AK4	0,058080656	-0,081284662	0	N.A.	0
ZEB2	0,058078722	-0,099308663	0	N.A.	0
CTD-3203P2.	0,05807545	0,05849693	0	N.A.	0
RP11-59H7.4	0,058068476	0,104248432	0	N.A.	0
LST1	0,058067758	-0,269286494	0	N.A.	0
PPM1K	0,05805389	0,126959213	0	N.A.	0
SLX1B	0,058023598	0,188714191	0	N.A.	0
LINC01235	0,058020034	-0,08448119	0	N.A.	0
R3HCC1L	0,057967822	0,16924788	0	N.A.	0
CITED2	0,057935916	-0,115376996	0	N.A.	0
TBC1D2	0,05789192	0,356636267	0	N.A.	0
MUC5B	0,057881941	0,302503591	0	N.A.	0
FCER1A	0,05787754	0,04508128	0	N.A.	0
KCNQ3	0,057820085	0,126171716	0	N.A.	0
SYN1	0,057782807	0,030285053	0	N.A.	0
FUT1	0,057773396	0,393055431	0	N.A.	0
CCL20	0,057716686	0,152124846	0	N.A.	0
RP11-410E4.	0,057686963	0,013597035	0	N.A.	0
LINC00174	0,057679021	0,076910274	0	N.A.	0
KCNAB2	0,05766529	-0,052724913	0	N.A.	0
RP11-347C12	0,057662467	-0,113143582	0	N.A.	0

PRR36	0,057548142	-0,234250767	0	N.A.	0
RP11-588G21	0,057524458		0	N.A.	0
SACS	0,057441377	-0,094894146	0	N.A.	0
HSPB9	0,057430473	0,199574479	0	N.A.	0
H2BFS	0,057420936	0,224579517	0	N.A.	0
SLC2A9	0,057407251	-0,095707156	0	N.A.	0
FUOM	0,057395452	0,258651408	0	N.A.	0
ALPL	0,057378213		0	N.A.	0
SIAH2	0,057366604	0,433657568	0	N.A.	0
TM9SF2	0,057311406	0,452211306	0	N.A.	0
ZCWPW1	0,057271049	0,165848882	0	N.A.	0
ZNF669	0,057256819	-0,172033363	0	N.A.	0
SFRP5	0,057253947	-0,038892696	0	N.A.	0
BTBD16	0,057230733	-0,176593091	0	N.A.	0
CSNK2A3	0,057226657	-0,167126952	0	N.A.	0
TMEM238	0,057200305	0,083214473	0	N.A.	0
RP11-247A12	0,057182308		0	N.A.	0
FABP4	0,057160086	-0,200089191	0	N.A.	0
LINC01389	0,057142949	-0,134553708	0	N.A.	0
DEPDC5	0,057056336	-0,2669948	0	N.A.	0
RP11-66D17.	0,057041458		0	N.A.	0
TMBIM4	0,057040333	0,159848669	0	N.A.	0
RP11-148B18	0,057029998		0	N.A.	0
MYH15	0,057021601	0,021272307	0	N.A.	0
HS3ST5	0,056993214		0	N.A.	0
RAPGEF5	0,056973692	0,131620772	0	N.A.	0
RP11-532F6.	0,056941291	-0,346059454	0	N.A.	0
ALDH3B2	0,0569377	-0,14684882	0	N.A.	0
AGAP3	0,056899558	0,523352536	0	N.A.	0
C19orf12	0,056886313	-0,073663808	0	N.A.	0
CTC-505O3.3	0,056884769		0	N.A.	0
GPR114	0,05686655	0,04753496	0	N.A.	0
KAAG1	0,056864259	0,182492503	0	N.A.	0

CTD-2357A8.	0,056829259	0,313936128	0	N.A.	0
LINC00957	0,056811579	-0,067158873	0	N.A.	0
TBC1D3B	0,056804543	0,006800455	0	N.A.	0
TBC1D24	0,056715188	-0,130044864	0	N.A.	0
RAD54L2	0,056684473	0,349179503	0	N.A.	0
SLC35A1	0,056674927	0,616231714	0	N.A.	0
RP11-452L6.5	0,056663898	0,181055189	0	N.A.	0
YPEL1	0,056659739	-0,067353104	0	N.A.	0
SIPA1L1	0,056647654	0,307885366	0	N.A.	0
SYCP3	0,056615393	-0,140262974	0	N.A.	0
S100A7	0,056589742		0	N.A.	0
CTB-171A8.1	0,056563108	0,076257672	0	N.A.	0
RPGR	0,056518107	-0,009950017	0	N.A.	0
PROCA1	0,056505879	-0,144039735	0	N.A.	0
RP11-368I7.4	0,056491381	0,026984199	0	N.A.	0
GIMAP1	0,056440552	0,049897452	0	N.A.	0
HLX	0,056429423	-0,131554054	0	N.A.	0
THBS3	0,056418946	0,262870953	0	N.A.	0
ANKFY1	0,056346063	0,247962044	0	N.A.	0
AVEN	0,056345352	0,213997541	0	N.A.	0
RP11-582J16	0,056344422	-0,047778209	0	N.A.	0
RAMP2-AS1	0,056317458	0,027449584	0	N.A.	0
SH2D4B	0,056219031		0	N.A.	0
TRIM10	0,056198029	0,15027476	0	N.A.	0
MMP24	0,056181624	-0,099716427	0	N.A.	0
LINC00921	0,056178029	0,015935306	0	N.A.	0
PAXIP1-AS2	0,056177176	0,196083372	0	N.A.	0
WNT5B	0,056161696	-0,272199332	0	N.A.	0
ZNF503-AS2	0,056150796	0,001613086	0	N.A.	0
GLI2	0,056143163	0,155621685	0	N.A.	0
SP110	0,056133897	0,457404707	0	N.A.	0
LINC01220	0,056101035	0,295231285	0	N.A.	0
NLGN3	0,056087306	0,005447437	0	N.A.	0

ATAD2B	0,056046267	0,069166828	0	N.A.	0
RP11-429J17	0,056036835	-0,118645652	0	N.A.	0
AFF3	0,056012808	-0,085913398	0	N.A.	0
FBXO27	0,055979302	-0,02882133	0	N.A.	0
TIRAP	0,055927209	-0,077405548	0	N.A.	0
PRORY	0,05592205	-0,012712	0	N.A.	0
BDNF-AS	0,055910764	-0,266262945	0	N.A.	0
MYDGF	0,055858287	0,42362438	0	N.A.	0
ESRP1	0,055843703	0,218650748	0	N.A.	0
CD19	0,055829094	-0,13135768	0	N.A.	0
CASQ2	0,055783142		0	N.A.	0
GLCCI1	0,055719921	0,121505787	0	N.A.	0
KLHL26	0,055662458	0,200361868	0	N.A.	0
WDR66	0,055650393	0,036794947	0	N.A.	0
MMP19	0,055622335	0,152405915	0	N.A.	0
PRR12	0,055619588	0,123060671	0	N.A.	0
ZFP36L2	0,055615794	0,286906475	0	N.A.	0
BTLA	0,055601236	-0,121317344	0	N.A.	0
XX-C2158C12	0,055551499	-0,035386973	0	N.A.	0
AC093627.10	0,055533285	0,212485704	0	N.A.	0
RP11-87C12.	0,055508262	0,260988349	0	N.A.	0
SRRM2-AS1	0,05547091	-0,091927807	0	N.A.	0
ZSCAN29	0,05546387	0,058023495	0	N.A.	0
TTR	0,055445989	0,106137431	0	N.A.	0
PLIN5	0,05538915		0	N.A.	0
GPR98	0,055353096	0,057652132	0	N.A.	0
INSRR	0,055301405		0	N.A.	0
LMNA	0,055268802	0,496755795	0	N.A.	0
DOCK10	0,055246217	-0,061738955	0	N.A.	0
PTPN18	0,055243042	0,315995013	0	N.A.	0
C16orf45	0,055207324		0	N.A.	0
ZNF317	0,055163499	-0,381844194	0	N.A.	0
RBM7	0,055138314	0,109570808	0	N.A.	0

TMEM245	0,055127871	0,422943208	0	N.A.	0
UBE2W	0,055115246	0,411669274	0	N.A.	0
S1PR1	0,055095173	0,117471666	0	N.A.	0
SPRED2	0,055043854	0,512954025	0	N.A.	0
SLC23A3	0,055041265	0,416128947	0	N.A.	0
AC124944.3	0,054999241	-0,009338369	0	N.A.	0
RP11-312B8.	0,054967686	0,172194348	0	N.A.	0
PTPLAD2	0,054885667	-0,303523254	0	N.A.	0
RP11-659E9.	0,054879872		0	N.A.	0
SLC1A7	0,054874144	-0,01435015	0	N.A.	0
MKRN1	0,054851411	0,21285081	0	N.A.	0
CAPRIN2	0,054849574	0,250878105	0	N.A.	0
C17orf59	0,054835063	0,188164785	0	N.A.	0
DCX	0,054831845		0	N.A.	0
TRPT1	0,054822439	0,12782185	0	N.A.	0
ANKK1	0,054638547	0,361544813	0	N.A.	0
CABP4	0,054600421	-0,004145188	0	N.A.	0
CSTF3-AS1	0,054563419		0	N.A.	0
RP11-347I19	0,054547775	-0,094817802	0	N.A.	0
COL4A1	0,054545084	-0,118203212	0	N.A.	0
LTB4R2	0,054538389	0,084125848	0	N.A.	0
RP11-298I3.4	0,054483168	-0,118301073	0	N.A.	0
SEC24C	0,054473239	0,431397919	0	N.A.	0
RBM4	0,054439278	0,188671852	0	N.A.	0
VPS13B	0,054392514	0,056797349	0	N.A.	0
NAT8	0,054370548		0	N.A.	0
C8orf34	0,054351938	-0,072499956	0	N.A.	0
C5orf56	0,054323162	0,349026393	0	N.A.	0
XX-C2158C6.	0,054292218	-0,076256617	0	N.A.	0
RP11-327P2.	0,054267564	0,166842721	0	N.A.	0
PIGV	0,05423965	0,22135539	0	N.A.	0
KIAA0319	0,054232596	0,258453833	0	N.A.	0
ACYP2	0,054188781	-0,004842868	0	N.A.	0

BLOC1S3	0,054164754	-0,121566478	0	N.A.	0
NBPF8	0,054140784	0,124635856	0	N.A.	0
FLNB-AS1	0,054131625		0	N.A.	0
IGHA2	0,054125387	0,001583657	0	N.A.	0
LA16c-OS12.1	0,054089108	0,030822837	0	N.A.	0
RP11-308D13	0,054072907	0,32078436	0	N.A.	0
HSD11B1L	0,054060414	-0,06188656	0	N.A.	0
ASB7	0,054038504	0,039054211	0	N.A.	0
CTD-2270P14	0,054038131	-0,112796844	0	N.A.	0
RHEBL1	0,054037006	-0,079803907	0	N.A.	0
AC023481.1	0,054002825	0,068922956	0	N.A.	0
GPR176	0,053995705		0	N.A.	0
ST6GALNAC4	0,053985456	-0,130532428	0	N.A.	0
PCDHGA5	0,053920268	0,101905316	0	N.A.	0
BBS10	0,053917681	0,190163373	0	N.A.	0
TVP23C	0,053915147	0,136793578	0	N.A.	0
MPND	0,05389663	0,159631755	0	N.A.	0
FOXH1	0,053896469	-0,204226797	0	N.A.	0
CTD-2588E21	0,053874781		0	N.A.	0
LOXL2	0,053855136	-0,117483588	0	N.A.	0
RP11-231E6.	0,053854238	-0,022830712	0	N.A.	0
ABCC8	0,053807539	0,003951785	0	N.A.	0
TEKT2	0,053786247		0	N.A.	0
RP11-1379J2	0,053715037	0,475524155	0	N.A.	0
CRAMP1L	0,053663984	0,179419545	0	N.A.	0
LRRC75A	0,053636553	-0,123167596	0	N.A.	0
UPK1B	0,053619188		0	N.A.	0
FAS	0,053618987	0,126328965	0	N.A.	0
GAA	0,053600685	0,325170163	0	N.A.	0
STC2	0,053583948	0,17162841	0	N.A.	0
TBCEL	0,053582354	-0,069462898	0	N.A.	0
NTRK2	0,053522129	-0,018725601	0	N.A.	0
ELAVL3	0,05349791		0	N.A.	0

LINC01118	0,053487224		0	N.A.	0
C7orf65	0,053486439		0	N.A.	0
ACBD4	0,053481941	0,009453385	0	N.A.	0
FKBP6	0,053478963		0	N.A.	0
FOXD4	0,05347139	0,072225576	0	N.A.	0
PLEKHA3	0,05346464	-0,148337041	0	N.A.	0
COL4A4	0,053456725		0	N.A.	0
RP3-455J7.4	0,053452134		0	N.A.	0
ADCY5	0,0534451		0	N.A.	0
MAPK14	0,053427292	0,038599098	0	N.A.	0
MAMDC2-AS	0,053420887		0	N.A.	0
CACNG8	0,053395641	0,259038566	0	N.A.	0
RP5-1198O2(	0,053369229	-0,212217861	0	N.A.	0
ZNF644	0,053354763	0,316510425	0	N.A.	0
SASH3	0,053348246	-0,200949039	0	N.A.	0
CEP85	0,053340089	-0,016619984	0	N.A.	0
RP11-143E21	0,053301622		0	N.A.	0
SNAP29	0,053282697	0,034901295	0	N.A.	0
CFAP69	0,053279162	0,003379281	0	N.A.	0
SYDE1	0,053256978		0	N.A.	0
CHST6	0,053231674	0,327092491	0	N.A.	0
RP6-201G10.	0,053201084	0,097576683	0	N.A.	0
MBOAT1	0,053197511	0,372071341	0	N.A.	0
C1orf220	0,053195622	0,014512399	0	N.A.	0
RP11-404F10	0,053185431	-0,122946027	0	N.A.	0
RP11-575I8.1	0,053178535	0,017552871	0	N.A.	0
XXyac-YX155	0,053164719	-0,0073352	0	N.A.	0
CYB5D2	0,053162415	0,298959405	0	N.A.	0
CTD-2047H1(	0,053147725	0,085881369	0	N.A.	0
MUT	0,053138372	0,002886729	0	N.A.	0
RP11-556N4.	0,053121414	0,31086417	0	N.A.	0
ZNF69	0,05311995	-0,235693268	0	N.A.	0
RP5-903G2.2	0,053119158	0,004647869	0	N.A.	0

RP11-65N13.	0,053096632	0,023901053	0	N.A.	0
ZFP82	0,053080655	0,171215731	0	N.A.	0
MYO7A	0,053044739	0,156607009	0	N.A.	0
FAM85B	0,053037487	0,080849133	0	N.A.	0
RP3-399L15.3	0,052975639	0,271115107	0	N.A.	0
RP11-863P13	0,052970658		0	N.A.	0
RP11-531F16	0,052969608	-0,065782525	0	N.A.	0
CD86	0,052956957	0,056952241	0	N.A.	0
CALB2	0,052946964	-0,390554056	0	N.A.	0
RP11-159F24	0,05292402	-0,160912935	0	N.A.	0
ZNF134	0,052908822	-0,249024777	0	N.A.	0
PRR14L	0,052893519	0,235371643	0	N.A.	0
TNRC6A	0,052886448	0,242630878	0	N.A.	0
GOLGA8A	0,052876793	0,319802461	0	N.A.	0
RP11-15A1.3	0,052854698	0,004895027	0	N.A.	0
ADAMTS13	0,052852238	0,266278829	0	N.A.	0
MYLIP	0,052821441	0,26828488	0	N.A.	0
RP11-1379J2	0,052818277	-0,199085312	0	N.A.	0
IL1RL2	0,052770291	-0,034769699	0	N.A.	0
SKOR1	0,052736903	0,019448887	0	N.A.	0
RIMS2	0,052718404	0,056769571	0	N.A.	0
WDR48	0,05270767	-0,158348142	0	N.A.	0
TMED5	0,052694979	-0,109481548	0	N.A.	0
SPG20	0,052664519	0,03628003	0	N.A.	0
MGP	0,052662295	-0,138174718	0	N.A.	0
RASAL2-AS1	0,052658868	0,097348812	0	N.A.	0
TMTC3	0,052643714	0,088632185	0	N.A.	0
MYO1G	0,052632736	-0,032198832	0	N.A.	0
LMOD3	0,052614923	-0,162865462	0	N.A.	0
JAM3	0,052611801	-0,124306453	0	N.A.	0
PDE5A	0,052601065	0,034162315	0	N.A.	0
POM121C	0,052542817	0,010441099	0	N.A.	0
TNFRSF11B	0,052541285	0,55215939	0	N.A.	0

FGFR1OP2	0,052523179	0,174231306	0	N.A.	0
LINC00501	0,052508042	0,276896306	0	N.A.	0
SNX29	0,052491276	-0,018784248	0	N.A.	0
CFAP53	0,052487283	0,003175331	0	N.A.	0
SNX16	0,052447115	0,088466647	0	N.A.	0
MT-CYB	0,05243993	0,198508073	0	N.A.	0
COL8A1	0,05243667	-0,247059457	0	N.A.	0
RP4-533D7.4	0,052388084	0,014722007	0	N.A.	0
FOXC2	0,052377901	0,01591779	0	N.A.	0
PTN	0,052308305	0,119815681	0	N.A.	0
CD300A	0,052273371	0,050833414	0	N.A.	0
PHLDB1	0,05227003	0,116656562	0	N.A.	0
DIDO1	0,052268937	0,279669366	0	N.A.	0
TMEM61	0,052253014	0,279852812	0	N.A.	0
PCED1A	0,05221884	-0,112021808	0	N.A.	0
FAM83H-AS1	0,05218003	0,346068209	0	N.A.	0
LY9	0,05213001		0	N.A.	0
DNAJC4	0,052108737	-0,108832014	0	N.A.	0
ADD3	0,052080583	0,245076826	0	N.A.	0
HESX1	0,052071238	-0,207396838	0	N.A.	0
NR2F2-AS1	0,052065855		0	N.A.	0
RNASEK-C17	0,052049296	-0,089545863	0	N.A.	0
RP11-169E6.	0,052011088		0	N.A.	0
CPQ	0,051996138	0,100332119	0	N.A.	0
RP11-414C23	0,051989548		0	N.A.	0
RP11-434I12	0,051982893	0,137741728	0	N.A.	0
LBHD1	0,051801632	-0,070129029	0	N.A.	0
ZFAND6	0,051796682	0,180894063	0	N.A.	0
RP11-77H9.2	0,051756145	0,0460884	0	N.A.	0
ZMYM1	0,051715164	-0,103713643	0	N.A.	0
CES4A	0,051693686	0,179131561	0	N.A.	0
RP5-1157M2	0,051670032	0,169482246	0	N.A.	0
SNX22	0,051616664	-0,053120764	0	N.A.	0

HABP2	0,051603689	0,021094837	0	N.A.	0
DNAH5	0,051578431	0,22125538	0	N.A.	0
RP11-466P24	0,051569768	-0,174910009	0	N.A.	0
CYP4V2	0,051544222	-0,135014748	0	N.A.	0
CD80	0,051542516		0	N.A.	0
SSTR1	0,051509133	-0,269045249	0	N.A.	0
TMED6	0,051497385	-0,144975674	0	N.A.	0
LINC01023	0,051479095	-0,220306736	0	N.A.	0
GATS	0,051460699	0,244150168	0	N.A.	0
TTC28	0,051399081	0,121460623	0	N.A.	0
GK5	0,051382487	0,085931252	0	N.A.	0
ZNF275	0,051376785	-0,315534395	0	N.A.	0
RP11-258F22	0,051335256		0	N.A.	0
CTD-2349P21	0,051308119	0,410990166	0	N.A.	0
FAM95C	0,051302885	0,040675499	0	N.A.	0
WBSCR17	0,051297903		0	N.A.	0
CLIP3	0,051291159	-0,271699566	0	N.A.	0
RP11-506M1	0,051250088	-0,24916852	0	N.A.	0
C5AR2	0,051232882		0	N.A.	0
RGS9BP	0,05113003		0	N.A.	0
PDE7A	0,051119478	0,12674696	0	N.A.	0
DNTTIP1	0,051104747	0,418044824	0	N.A.	0
LINC00629	0,051103118		0	N.A.	0
ADPGK-AS1	0,051052716		0	N.A.	0
RP11-379B8.	0,051041506	-0,09865832	0	N.A.	0
RFPL4A	0,051008938		0	N.A.	0
PLEKHA4	0,051003821	-0,079502911	0	N.A.	0
ZNF555	0,050997771	-0,093589913	0	N.A.	0
THRSP	0,050981654	0,120578431	0	N.A.	0
SNTA1	0,050969544	0,361935813	0	N.A.	0
FAM98C	0,050931318	0,367202593	0	N.A.	0
NPIPA5	0,050927034	0,029406476	0	N.A.	0
BBS12	0,050906862	0,08159955	0	N.A.	0

DOCK4	0,050881114	0,031134692	0	N.A.	0
ZNF114	0,050878195	0,256719882	0	N.A.	0
CAND1	0,050822586	0,115848935	0	N.A.	0
MMP9	0,050804855	-0,201143661	0	N.A.	0
DNM1	0,050770312	0,176200672	0	N.A.	0
NSUN3	0,05074568	0,137184994	0	N.A.	0
SDF2	0,050695374	0,357892694	0	N.A.	0
PIAS3	0,050688729	0,315484494	0	N.A.	0
MUS81	0,05062401	0,182442355	0	N.A.	0
LINC01176	0,050615447	0,16300979	0	N.A.	0
STAU1	0,050607163	0,138214267	0	N.A.	0
ACKR4	0,050600394	0,049017457	0	N.A.	0
KLHL9	0,050579182	0,127287826	0	N.A.	0
RGS11	0,050572932	0,363343783	0	N.A.	0
SYDE2	0,050570332	0,042288421	0	N.A.	0
AF131215.8	0,050555802		0	N.A.	0
FUT8-AS1	0,050554933	0,00504572	0	N.A.	0
FFAR2	0,050541671	-0,126370138	0	N.A.	0
DESI2	0,050541657	-0,046592194	0	N.A.	0
RP11-480C22	0,050521444		0	N.A.	0
MAML1	0,050478487	-0,110483789	0	N.A.	0
RP11-112J3.1	0,050449391	-0,107022849	0	N.A.	0
ZXDB	0,050438081	-0,118275161	0	N.A.	0
PXDNL	0,050428158	0,044112527	0	N.A.	0
RP11-214C8.	0,050416041	0,042058105	0	N.A.	0
ITGB2-AS1	0,050398735	0,102037571	0	N.A.	0
ARHGEF1	0,050391434	0,379433561	0	N.A.	0
RP1-100J12.1	0,050382413	-0,143244633	0	N.A.	0
RPRD1B	0,05036742	0,160628957	0	N.A.	0
TSPEAR-AS1	0,050294795	0,103672351	0	N.A.	0
AC006262.4	0,05025925	0,097182502	0	N.A.	0
ITGB2	0,050255864	-0,195952856	0	N.A.	0
FAF2	0,050200979	0,45567315	0	N.A.	0

HS3ST6	0,05020019	0,184611213	0	N.A.	0
USP42	0,050191957	0,089342396	0	N.A.	0
CCDC69	0,050185646	0,15297011	0	N.A.	0
DLG3-AS1	0,050126999	0,204939986	0	N.A.	0
HIPK1-AS1	0,050097978	-0,272561628	0	N.A.	0
HCG25	0,05006476		0	N.A.	0
ASAP3	0,050062499	-0,092679384	0	N.A.	0
SNX29P2	0,050052102	-0,125730185	0	N.A.	0
KCNC3	0,049985161	0,015707655	0	N.A.	0
SLC25A28	0,049982841	0,167021691	0	N.A.	0
GPNMB	0,049981817	-0,181537603	0	N.A.	0
CXXC5	0,049974695	0,319849329	0	N.A.	0
RP11-736K20	0,049923723	-0,143240827	0	N.A.	0
GCNT1	0,049902422	0,156420034	0	N.A.	0
HCRT	0,049891546	0,122606838	0	N.A.	0
RP11-430G17	0,049855166	-0,160742264	0	N.A.	0
SEMA4A	0,04981845	0,110953743	0	N.A.	0
CD69	0,049799245	-0,114086412	0	N.A.	0
RP11-803P9.	0,049794963	0,150519898	0	N.A.	0
NACAD	0,04978692		0	N.A.	0
CDH5	0,049739574	0,075400901	0	N.A.	0
RP11-356J5.1	0,049719782	0,048699824	0	N.A.	0
TESK2	0,049703725	0,089995934	0	N.A.	0
PEX13	0,049682596	0,089138882	0	N.A.	0
SMG8	0,049556673	0,338727161	0	N.A.	0
AP3S2	0,049510004	-0,115215468	0	N.A.	0
KLF12	0,04949839	0,008900291	0	N.A.	0
ZBTB20-AS4	0,049492812	0,151478709	0	N.A.	0
GPR75	0,049491644		0	N.A.	0
RP11-60L3.1	0,049447709		0	N.A.	0
RP13-379L11	0,049428128		0	N.A.	0
LINC00923	0,049419649	0,038805576	0	N.A.	0
ARMC5	0,049417341	-0,179288116	0	N.A.	0

GRTP1-AS1	0,049398113	-0,159453705	0	N.A.	0
AC012499.1	0,049349347		0	N.A.	0
PDPN	0,049345931	0,008626501	0	N.A.	0
CXCL9	0,049340754	0,011390384	0	N.A.	0
BTAF1	0,049319217	0,023676951	0	N.A.	0
RP11-413H22	0,049305309	0,11767033	0	N.A.	0
AC106801.1	0,049303796		0	N.A.	0
THAP8	0,049241918	0,045735284	0	N.A.	0
LNX1-AS1	0,049225336		0	N.A.	0
CCDC78	0,049173907	0,307963073	0	N.A.	0
UBQLN1	0,049142493	0,471190002	0	N.A.	0
VEZF1	0,049096729	0,04231526	0	N.A.	0
AC012363.4	0,049059931	0,002037195	0	N.A.	0
UVRAG	0,049046117	0,050481228	0	N.A.	0
TMIE	0,04900005	0,053002083	0	N.A.	0
RP5-858B6.1	0,048899575	0,252675539	0	N.A.	0
RNF208	0,048893553	-0,042375798	0	N.A.	0
AC009133.21	0,048851666	0,110537444	0	N.A.	0
RP4-550H1.5	0,048845759		0	N.A.	0
AGO3	0,048829988	0,109975669	0	N.A.	0
CITF22-49E9.	0,048828039		0	N.A.	0
RIPK2	0,04880363	0,056794154	0	N.A.	0
RP13-494C23	0,048800495		0	N.A.	0
RP11-326C3.	0,048794964		0	N.A.	0
TJAP1	0,048786019	-0,017319153	0	N.A.	0
BACE1-AS	0,048776334	-0,084533812	0	N.A.	0
CTC-479C5.1	0,048751304	-0,076093092	0	N.A.	0
OBSL1	0,048720838	0,055848945	0	N.A.	0
CTD-2012K14	0,048695566		0	N.A.	0
COL18A1	0,048691356	-0,218483021	0	N.A.	0
KIAA1919	0,048634788	-0,032671132	0	N.A.	0
SOCS4	0,048628499	-0,151785648	0	N.A.	0
BAIAP2-AS1	0,048591575	0,173457359	0	N.A.	0

CTD-3105H1	0,048576205	0,136840482	0	N.A.	0
MEIS3	0,048559634	0,026399884	0	N.A.	0
RP11-474B16	0,048547241		0	N.A.	0
SCN9A	0,048547144	0,225489394	0	N.A.	0
USP49	0,048528622	0,286230248	0	N.A.	0
RP11-219G1	0,048464835	0,068985489	0	N.A.	0
ZNF212	0,048462557	0,057205177	0	N.A.	0
RP11-706O1	0,048437283	0,229031835	0	N.A.	0
CGRRF1	0,048433095	0,11337265	0	N.A.	0
TMEM115	0,048427845	0,321598458	0	N.A.	0
RP11-535A1	0,048404876	-0,237626893	0	N.A.	0
RP11-798M1	0,04839222	0,089312083	0	N.A.	0
PAQR5	0,048384938	-0,002301308	0	N.A.	0
RP11-192H2	0,048367101	-0,126792599	0	N.A.	0
SPDYE5	0,048353925	-0,016388647	0	N.A.	0
PAPD5	0,048331025	-0,161403972	0	N.A.	0
PHACTR1	0,048327388	-0,04637346	0	N.A.	0
G3BP2	0,048324508	-0,015351512	0	N.A.	0
NGRN	0,048317289	-0,007548952	1	cl2	0
RP11-234K2	0,048306752	0,078987935	0	N.A.	0
AGPAT6	0,04830409	0,453457863	0	N.A.	0
TMPRSS13	0,048299707	0,053874984	0	N.A.	0
DUSP7	0,048276315	0,022140054	0	N.A.	0
TCF21	0,048268686	0,165624403	0	N.A.	0
CXCR1	0,048257543	0,36239337	0	N.A.	0
DZANK1	0,048241651	0,087025672	0	N.A.	0
GNG11	0,048232593	-0,246459901	0	N.A.	0
RP4-671O14.	0,048225268	-0,108358869	0	N.A.	0
LRRC41	0,048203356	-0,146730258	0	N.A.	0
FOXD2	0,048182402	-0,205520019	0	N.A.	0
RP11-18C24.	0,048169957		0	N.A.	0
CTC-498J12.	0,048167533	-0,131138633	0	N.A.	0
FAM198B	0,048160255	0,222539645	0	N.A.	0

ID4	0,048116421	-0,12793195	0	N.A.	0
GOSR2	0,048052964	0,120996404	0	N.A.	0
ZNF683	0,048041623	0,10364874	0	N.A.	0
HS1BP3	0,047973901	0,259092918	0	N.A.	0
RP11-345P4.	0,04796596	0,082019753	0	N.A.	0
SUGCT	0,047955844	-0,15830066	0	N.A.	0
AC002398.11	0,047934356		0	N.A.	0
POTEE	0,047924352		0	N.A.	0
C16orf89	0,0479105	-0,299354089	0	N.A.	0
PNRC2	0,047908309	-0,204115442	0	N.A.	0
CCDC152	0,047882978	-0,117167484	0	N.A.	0
PKD2L2	0,047857872		0	N.A.	0
CD300E	0,047855995	0,214355301	0	N.A.	0
FILIP1	0,047826908	-0,23916611	0	N.A.	0
POGK	0,047800506	0,127320714	0	N.A.	0
RLTPR	0,047778674	-0,088758096	0	N.A.	0
MGC45922	0,047778341	-0,035489937	0	N.A.	0
RP5-1119A7.	0,047736061	-0,108386536	0	N.A.	0
OST4	0,047730629	-0,162071999	0	N.A.	0
AP004550.1	0,047729986	-0,055394544	0	N.A.	0
SLC2A6	0,047709557	0,277731536	0	N.A.	0
LCORL	0,047689578	0,070586974	0	N.A.	0
MED12	0,047662505	0,082174958	0	N.A.	0
DET1	0,04766173	-0,256113572	0	N.A.	0
CLEC4E	0,047659903		0	N.A.	0
RP11-977B10	0,047590343	-0,088471364	0	N.A.	0
TRAPPC2B	0,047586524	0,167281457	0	N.A.	0
LRRK1	0,047577026	-0,032980152	0	N.A.	0
SPEG	0,047551089	0,043152163	0	N.A.	0
C17orf82	0,047530317	0,116162047	0	N.A.	0
REG1B	0,047520207	-0,004820972	0	N.A.	0
RNF111	0,047518478	0,163531187	0	N.A.	0
RP11-756H6.	0,047499689	0,156115603	0	N.A.	0

SALL4	0,047481882	-0,027586718	0	N.A.	0
RP11-162G1(	0,047455857	-0,069089437	0	N.A.	0
CLDN19	0,04743795		0	N.A.	0
CHRNB1	0,047426473	-0,14374634	0	N.A.	0
CTD-2521M2	0,047408016		0	N.A.	0
BCORL1	0,047401418	0,038188888	0	N.A.	0
APBA1	0,047374662	-0,107388139	0	N.A.	0
NAT6	0,047345761	0,227248174	0	N.A.	0
TMEM220	0,04722122	-0,054421894	0	N.A.	0
FAM117A	0,047217981	0,320449867	0	N.A.	0
CTRL	0,0471544	0,104954283	0	N.A.	0
KAL1	0,047141637	0,026832826	0	N.A.	0
RP11-89K21.	0,047106928	-0,325356653	0	N.A.	0
PCNXL4	0,047086437	0,098264323	0	N.A.	0
PLS3-AS1	0,047077267	0,359387648	0	N.A.	0
UBE2V1	0,047057744	-0,028286598	0	N.A.	0
WNT10B	0,047042482	0,014722007	0	N.A.	0
PLEKHO1	0,046980403	-0,242803379	0	N.A.	0
CNTNAP3	0,046928541	-0,283539552	0	N.A.	0
KLB	0,046886298	0,312195027	0	N.A.	0
MARK1	0,046884473	0,155289704	0	N.A.	0
RP4-635E18.	0,046869215	0,108572936	0	N.A.	0
OCRL	0,046859052	-0,162902993	0	N.A.	0
MEI1	0,046846843	-0,237476911	0	N.A.	0
ZFP28	0,046762586	-0,275314242	0	N.A.	0
SLC41A3	0,04673978	-0,251053483	0	N.A.	0
MAP3K7CL	0,046662872	0,101413076	0	N.A.	0
ZCCHC10	0,046660741	0,2471738	0	N.A.	0
DUOX1	0,046626559	0,178974486	0	N.A.	0
RP11-667M1	0,046609778		0	N.A.	0
TRBV25-1	0,046546979	-0,387867461	0	N.A.	0
RP11-74E22.	0,046526956	0,059563247	0	N.A.	0
DRGX	0,046428188	0,191455494	0	N.A.	0

C7orf60	0,046422938	-0,148597256	0	N.A.	0
LAMA2	0,046381307	-0,042532112	0	N.A.	0
AC140725.4	0,046371268		0	N.A.	0
RP11-2E11.6	0,046358543	0,006800455	0	N.A.	0
RHBDL1	0,046309427	0,114528273	0	N.A.	0
AC097662.2	0,046295706		0	N.A.	0
TIAM2	0,046290494	0,362599531	0	N.A.	0
GOLGA6L10	0,046290004	0,286770952	0	N.A.	0
ZXDC	0,046280121	0,087803194	0	N.A.	0
EPN2-AS1	0,046261849		0	N.A.	0
RP11-961A15	0,046238074	0,014005086	0	N.A.	0
ARHGEF9	0,04621707	0,015633473	0	N.A.	0
LINC01465	0,046203451	0,215531435	0	N.A.	0
PAPPA	0,046180412	-0,046407798	0	N.A.	0
AFAP1-AS1	0,046170143	0,076118312	0	N.A.	0
CTB-55O6.12	0,046160082	0,20516228	0	N.A.	0
RNASE7	0,046104065	-0,068589757	0	N.A.	0
RP11-130F10	0,046103899		0	N.A.	0
CTD-2366F13	0,046088381	0,020401274	0	N.A.	0
EAF1-AS1	0,046077692	0,044033493	0	N.A.	0
SLC25A42	0,046043244	-0,130699932	0	N.A.	0
BMP4	0,046034478	0,553632062	0	N.A.	0
FBLN5	0,045994855	0,06068011	0	N.A.	0
MT-ATP6	0,045974797	0,224152915	0	N.A.	0
RP11-626K17	0,045951906	0,097793156	0	N.A.	0
RP11-932O9.	0,045941246		0	N.A.	0
AGAP10	0,045940983	0,350153207	0	N.A.	0
CTD-2026K11	0,045917097	0,054727096	0	N.A.	0
SLC35E2	0,045915115	0,014751671	0	N.A.	0
MATN3	0,045889797		0	N.A.	0
RP1-187B23.	0,045878716	-0,146865157	0	N.A.	0
MTOR	0,045799267	-0,051957261	0	N.A.	0
NRXN1	0,045775699		0	N.A.	0

APBB2	0,045774773	-0,023529035	0	N.A.	0
AC011523.2	0,045773287	-0,138122281	0	N.A.	0
VPS39	0,045753448	0,139840726	0	N.A.	0
CTD-2530H1:	0,045738359	-0,076230895	0	N.A.	0
PAPD7	0,045726307	-0,00295587	0	N.A.	0
JRK	0,045692271	0,109681586	0	N.A.	0
MTURN	0,045681701	0,055609615	0	N.A.	0
RP4-647C14.	0,045679771	0,367954129	0	N.A.	0
ZNF41	0,04564781	-0,078700274	0	N.A.	0
EN2	0,045636875	0,34290262	0	N.A.	0
SEPHS2	0,045633849	-0,165149341	0	N.A.	0
ZNF250	0,045630471	-0,316324725	0	N.A.	0
LINC01004	0,045612608	0,215837594	0	N.A.	0
AKAP7	0,045608736	0,088140394	0	N.A.	0
CLK1	0,04555249	0,277589115	0	N.A.	0
CSF1R	0,04551551	-0,157700284	0	N.A.	0
PRSS35	0,045508862		0	N.A.	0
ATP6V1C1	0,045462146	0,064037407	0	N.A.	0
AC007966.1	0,045445119	0,015193087	0	N.A.	0
GON4L	0,045421079	0,118386504	0	N.A.	0
RP11-500B12	0,045396387	-0,071564116	0	N.A.	0
HCAR2	0,045389671	-0,023476861	0	N.A.	0
RP4-533D7.5	0,045370077	-0,279407261	0	N.A.	0
EFHC2	0,045343223	-0,229383603	0	N.A.	0
PALM3	0,045310723	0,081824251	0	N.A.	0
UBE3A	0,04530659	0,294997648	0	N.A.	0
ABCG5	0,045288704	0,351441326	0	N.A.	0
PDZD8	0,045279271	0,197388693	0	N.A.	0
CDK16	0,045261588	-0,03318628	0	N.A.	0
XXbac-BPG3(	0,045254418	-0,126918645	0	N.A.	0
RAB43	0,045224662	-0,073392519	0	N.A.	0
PGBD3	0,04511853		0	N.A.	0
NPIPB4	0,04511465	0,429773919	0	N.A.	0

RP1-228P16.	0,0450965	-0,08959094	0	N.A.	0
AIP	0,045074824	-0,059081654	0	N.A.	0
FAM218A	0,045067858	-0,055489061	0	N.A.	0
LRRC46	0,045056905	-0,182731883	0	N.A.	0
PRDM15	0,045056504	0,087419712	0	N.A.	0
ANKDD1A	0,045042681	-0,042445014	0	N.A.	0
GPR64	0,045022358		0	N.A.	0
GS1-259H13.	0,045021243	0,116251739	0	N.A.	0
RP11-66H6.3	0,044998201	-0,02875174	0	N.A.	0
FAM83C-AS1	0,044965405	0,057497946	0	N.A.	0
RP4-568C11.	0,044926928	0,147932666	0	N.A.	0
MYCBP2-AS1	0,044906681	0,044670365	0	N.A.	0
LMOD1	0,044901763	-0,013183274	0	N.A.	0
ZNF57	0,044894225	-0,142551348	0	N.A.	0
RHOBTB1	0,044880471	0,247834363	0	N.A.	0
ERCC6	0,044876323	0,027734285	0	N.A.	0
RP11-465B22	0,044875635	0,295667888	0	N.A.	0
KCNQ1-AS1	0,044861212		0	N.A.	0
PCGF2	0,044834172	-0,008914137	0	N.A.	0
CCZ1	0,044811842	0,256132575	0	N.A.	0
C20orf194	0,04479884	0,095547482	0	N.A.	0
SERPINA1	0,04475969	0,364724474	0	N.A.	0
CECR3	0,044742997	0,256814726	0	N.A.	0
EXTL3-AS1	0,044708254	0,173659146	0	N.A.	0
HDC	0,044690147	-0,014083432	0	N.A.	0
NMNAT2	0,044688337	-0,277677768	0	N.A.	0
CYYR1	0,044665459	-0,027616885	0	N.A.	0
RUNDC3A	0,044650824	0,396583104	0	N.A.	0
TBX21	0,044630583		0	N.A.	0
ZCCHC14	0,044612196	0,252013721	0	N.A.	0
RP11-573D15	0,044537761	-0,058778855	0	N.A.	0
HBA1	0,044520262	0,098678502	0	N.A.	0
MROH8	0,044494081	0,001428075	0	N.A.	0

ZNF878	0,044481112	-0,122405616	0	N.A.	0
LY96	0,044456296	-0,34819614	0	N.A.	0
RP11-930O11	0,044433285	0,25951483	0	N.A.	0
CBWD7	0,044335314	0,090970348	0	N.A.	0
MYBPC1	0,044247328	0,076576503	1	none	0
ZNF605	0,044210474	-0,046725244	0	N.A.	0
PDE4DIP	0,044198754	0,079932151	0	N.A.	0
LIPC	0,04415697		0	N.A.	0
CEBPB-AS1	0,044147061	0,105664491	0	N.A.	0
HIST1H4D	0,044115374	-0,20443618	0	N.A.	0
RP11-253E3.	0,044091322	0,015264439	0	N.A.	0
ZFPL1	0,044082219	0,17322485	0	N.A.	0
CPT1A	0,044066672	-0,163421634	0	N.A.	0
RP11-350J20	0,04400565	-0,091322052	0	N.A.	0
RP11-439E19	0,043973642	0,008196029	0	N.A.	0
PRR15L	0,043969751	0,487238169	0	N.A.	0
RP11-324I22	0,043956666	0,172083994	0	N.A.	0
XXbac-BPG17	0,0439515		0	N.A.	0
EXTL3	0,043904814	0,282504384	0	N.A.	0
UFL1	0,043885675	0,387888575	0	N.A.	0
RP11-160H22	0,043885125	-0,073419476	0	N.A.	0
SHISA3	0,04388122		0	N.A.	0
ACP5	0,043880703	-0,099747008	0	N.A.	0
LACTB	0,043871281	0,154255976	0	N.A.	0
KLRF1	0,043866546	-0,062707975	0	N.A.	0
WAS	0,04380448	-0,141531745	0	N.A.	0
C17orf100	0,043736876	0,088388901	0	N.A.	0
NXPE4	0,043729795	-0,169390388	0	N.A.	0
AC069363.1	0,04372106	-0,083490092	0	N.A.	0
FCRLB	0,043718599	-0,108122656	0	N.A.	0
KLF9	0,043688267	-0,066485012	0	N.A.	0
FXYD2	0,043687853	0,3872307	0	N.A.	0
CTB-46B19.2	0,043687794	-0,005625819	0	N.A.	0

KB-208E9.1	0,043668099		0	N.A.	0
MAVS	0,043639026	0,151444041	0	N.A.	0
GPR162	0,043630236	-0,066151017	0	N.A.	0
CTD-2036P10	0,043585702	0,021219307	0	N.A.	0
RP1-78B3.1	0,04356139	0,138377444	0	N.A.	0
ANKRD18A	0,043508201	0,191057514	0	N.A.	0
SLC25A48	0,043503802	-0,204795424	0	N.A.	0
HIGD2B	0,043495743	0,039657391	0	N.A.	0
WEE2-AS1	0,043482111	-0,000771911	0	N.A.	0
ZNF345	0,043451196	0,075589736	0	N.A.	0
RP11-894P9.	0,043445909		0	N.A.	0
C9orf41-AS1	0,043369259		0	N.A.	0
TIMP3	0,043362636	0,006595219	0	N.A.	0
C3orf62	0,043360378	0,139731116	0	N.A.	0
RP11-191G24	0,043358188	-0,187634925	0	N.A.	0
LRRC70	0,043337133		0	N.A.	0
TDRD7	0,04332077	0,280050518	0	N.A.	0
RP5-940J5.9	0,043310045	-0,331409816	0	N.A.	0
CCDC144A	0,043309694	0,133542941	0	N.A.	0
SCAP	0,043303183	0,264826114	0	N.A.	0
HSF4	0,043251321	0,044217075	0	N.A.	0
TMEM130	0,043236462	0,043253808	0	N.A.	0
KLHL25	0,043226748	0,046901231	0	N.A.	0
CNTNAP3B	0,043218158	-0,170065372	0	N.A.	0
RP11-539G18	0,043170669	0,288944564	0	N.A.	0
MFAP5	0,043132448	-0,230865292	0	N.A.	0
ZNF277	0,0431297	-0,224008081	0	N.A.	0
KLHL29	0,043128943	0,048009435	0	N.A.	0
TYK2	0,043054379	0,067370799	0	N.A.	0
DPYD	0,043042178	0,087867565	0	N.A.	0
RP11-159D12	0,042972057	0,123501503	0	N.A.	0
LPP-AS2	0,042964463	0,20511476	0	N.A.	0
AC093901.1	0,04296345	0,14723576	0	N.A.	0

BTN2A2	0,042957365	0,243389141	0	N.A.	0
RP4-569M23	0,042932719	-0,02011289	0	N.A.	0
CHURC1-FNT	0,042926953	-0,111795667	0	N.A.	0
C4orf33	0,042897119	-0,189173263	0	N.A.	0
UNC13B	0,042896098	0,152574554	0	N.A.	0
RP11-650P15	0,04289102	0,254791821	0	N.A.	0
FOXK2	0,042876154	0,161545994	0	N.A.	0
HUNK	0,042843008	-0,026644147	0	N.A.	0
ERC1	0,042833446	0,085516475	0	N.A.	0
UNKL	0,042818709	-0,06592303	0	N.A.	0
C1QTNF4	0,042807114	-0,281482155	0	N.A.	0
SHANK2-AS1	0,0427725		0	N.A.	0
ARL2BP	0,042761229	-0,049754773	0	N.A.	0
CLEC4A	0,042720739	0,131436092	0	N.A.	0
RP11-474D1.	0,042670383		0	N.A.	0
RP5-882C2.2	0,042658178	0,208889875	0	N.A.	0
ZNF799	0,042585926	0,148131892	0	N.A.	0
NOXO1	0,042533272	0,452413124	0	N.A.	0
MEGF8	0,042512124	0,247092237	0	N.A.	0
EEPD1	0,042488379	0,01608789	0	N.A.	0
POLK	0,042469132	-0,100760808	0	N.A.	0
LRRC57	0,042468094	0,087806434	0	N.A.	0
STON1	0,042452109	0,238043401	0	N.A.	0
AC005329.7	0,042409747	0,181393091	0	N.A.	0
HLA-DQA2	0,042392307		0	N.A.	0
PPAPDC1A	0,042385058	0,01190635	0	N.A.	0
TXLNA	0,042330106	-0,005733291	0	N.A.	0
SYNJ1	0,042313983	-0,275413305	0	N.A.	0
RANBP17	0,042288726	0,128215052	0	N.A.	0
AGBL1	0,042271157		0	N.A.	0
GRHL2	0,042212584	-0,134049067	0	N.A.	0
RP11-575F12	0,042202296	-0,057643264	0	N.A.	0
RP11-597D13	0,042174042	-0,10186899	0	N.A.	0

CH507-254M	0,042172985	-0,0387709	0	N.A.	0
APC2	0,042151748	0,01704278	0	N.A.	0
ABCC1	0,042127656	0,154320861	0	N.A.	0
IL17D	0,042127349	-0,106755127	0	N.A.	0
ZNF235	0,042116122	-0,057047275	0	N.A.	0
ZNF407	0,042105113	0,232260888	0	N.A.	0
GLIS3-AS1	0,042075995	0,07780627	0	N.A.	0
INHBB	0,042068772	0,30078859	0	N.A.	0
LINC00393	0,042057238	0,075345339	0	N.A.	0
TRPV1	0,042021637	0,300319725	0	N.A.	0
EMILIN2	0,041989957	0,145702711	0	N.A.	0
MEGF11	0,041966835		0	N.A.	0
RP11-1148L6	0,041956442	0,028987544	0	N.A.	0
RP11-568N6.	0,041947508	0,085060539	0	N.A.	0
PANK4	0,041926359	0,054116159	0	N.A.	0
RP11-428K3.	0,041894599	0,051216649	0	N.A.	0
RP11-89H19.	0,041893114	-0,066350673	0	N.A.	0
LINC01433	0,041883243		0	N.A.	0
RP11-202A13	0,041881434	0,148451194	0	N.A.	0
RP11-545M1	0,041878341	0,031287697	0	N.A.	0
C21orf91-OT	0,041847832		0	N.A.	0
CH17-125A1(	0,041839456		0	N.A.	0
PHF11	0,041832721	-0,038793282	0	N.A.	0
A2M	0,041821649	-0,041130682	0	N.A.	0
CTA-384D8.3	0,041821628		0	N.A.	0
NR3C1	0,041815996	0,108801153	0	N.A.	0
RP11-797H7.	0,041812071	-0,020632409	0	N.A.	0
C11orf63	0,041800267		0	N.A.	0
PARP15	0,041753203	-0,123907018	0	N.A.	0
ZNF404	0,04174971	0,011913904	0	N.A.	0
ZNF121	0,041749018	-0,002925703	0	N.A.	0
CITF22-1A6.3	0,04173679	0,093783972	0	N.A.	0
RP3-508I15.1	0,041728327	0,213740669	0	N.A.	0

LINC00941	0,041711223	-0,181454237	0	N.A.	0
AP001258.4	0,04168587	0,068203048	0	N.A.	0
TAGLN3	0,041664209		0	N.A.	0
TLK2	0,041663246	0,136891096	0	N.A.	0
PHYHD1	0,041637277	0,090867301	0	N.A.	0
LGALS7B	0,041548114	0,199666532	0	N.A.	0
RP11-384C4.	0,041521431		0	N.A.	0
ZC4H2	0,041487757	-0,047964412	0	N.A.	0
SEMG1	0,041486502	-0,147421079	0	N.A.	0
RDH16	0,041447906	0,160242887	0	N.A.	0
DMBX1	0,041413974	-0,025905335	0	N.A.	0
RAB44	0,041410284		0	N.A.	0
RP11-138C9.	0,041398843	-0,060611892	0	N.A.	0
TP53I11	0,041392296	0,05013641	0	N.A.	0
PCDHGA8	0,041390811	0,308170505	0	N.A.	0
RETN	0,041384703		0	N.A.	0
AC005757.6	0,041377301		0	N.A.	0
IL4I1	0,04136965	-0,143080254	0	N.A.	0
USP9Y	0,041363903	0,089057697	0	N.A.	0
AC073046.25	0,041363526	-0,086997116	0	N.A.	0
COL7A1	0,041286735	0,277151037	0	N.A.	0
GPR158	0,041281121	0,098711071	0	N.A.	0
RP11-981G7.	0,041273923		0	N.A.	0
FSTL1	0,041226182	-0,085561952	0	N.A.	0
RP11-46H11.	0,041223013		0	N.A.	0
FHL1	0,041192203	0,094616759	0	N.A.	0
RP11-432J22	0,041156245	-0,319852255	0	N.A.	0
RECQL5	0,041144251	0,327322628	0	N.A.	0
LRRC16B	0,041138675	0,008583826	0	N.A.	0
NFKB1	0,041104574	0,092124742	0	N.A.	0
LINC01449	0,041099389		0	N.A.	0
RP11-522I20	0,041093798	-0,086231948	0	N.A.	0
C12orf50	0,040992858	0,146165371	0	N.A.	0

B4GALNT2	0,040989392	0,181718014	0	N.A.	0
ZNF23	0,040971992	-0,216682979	0	N.A.	0
MYH3	0,040924954	0,098984241	0	N.A.	0
BTC	0,040858178	0,064028921	0	N.A.	0
SLC5A12	0,040844535	-0,109482806	0	N.A.	0
IQUB	0,040835587	0,038277041	0	N.A.	0
AC017002.2	0,040833071	0,231105303	0	N.A.	0
ZNF438	0,040802529	0,210007216	0	N.A.	0
LDLRAD2	0,040802202		0	N.A.	0
PIK3C3	0,040764159	0,280673968	0	N.A.	0
PIKFYVE	0,040748325	0,101141312	0	N.A.	0
SEPT8	0,04074777	-0,147713716	0	N.A.	0
C16orf62	0,040740621	-0,281001767	0	N.A.	0
GADD45B	0,040722244	0,262520977	0	N.A.	0
FIGF	0,04071229		0	N.A.	0
SLC25A21	0,040688672	0,101302822	0	N.A.	0
CLLU1	0,040651216		0	N.A.	0
SEMG2	0,040649679	0,326574771	0	N.A.	0
OTOGL	0,040612564	-0,094501136	0	N.A.	0
GAMT	0,040611171		0	N.A.	0
CSNK1G2	0,040565455	0,2625935	0	N.A.	0
CATSPER2	0,040535029	0,348397924	0	N.A.	0
LYL1	0,040494572	-0,00169474	0	N.A.	0
RP11-74E22.[illegible]	0,040472199	0,256439795	0	N.A.	0
CTD-2600O9[illegible]	0,040471594	0,272708353	0	N.A.	0
DLGAP1	0,040423867	0,104602294	0	N.A.	0
CTC-308K20.[illegible]	0,040372147		0	N.A.	0
RP11-90L1.8	0,040370311	0,081482611	0	N.A.	0
ARSD-AS1	0,040326694	0,006634189	0	N.A.	0
RP11-230G5.[illegible]	0,040317492		0	N.A.	0
EHMT1	0,040312302	0,238516804	0	N.A.	0
TRPC1	0,040269476	0,289041591	0	N.A.	0
CTD-2017D1[illegible]	0,040226696	0,201586703	0	N.A.	0

PRSS53	0,040199309	0,329562827	0	N.A.	0
UGT2B7	0,040193769	-0,178527338	0	N.A.	0
RP11-396F22	0,040160837		0	N.A.	0
SYNPR-AS1	0,040156495	-0,032669774	0	N.A.	0
RGS17	0,040147755	0,061478769	0	N.A.	0
CADPS2	0,04009683	0,015920258	0	N.A.	0
FAM65C	0,040075502	0,104995292	0	N.A.	0
HFE	0,040062186	0,316546573	0	N.A.	0
EFNB3	0,040038128		0	N.A.	0
RP11-363D14	0,039961112		0	N.A.	0
OOEP	0,039905881	0,108658533	0	N.A.	0
COLEC10	0,039789051		1	cl1	0
ANKRD20A4	0,039709891		0	N.A.	0
POC1B	0,03968789	-0,167949328	0	N.A.	0
ZNF341-AS1	0,039666998	0,277622564	0	N.A.	0
ITM2A	0,039658114	-0,248596922	0	N.A.	0
FES	0,03964605	-0,097113884	0	N.A.	0
FCGR3A	0,039633436	0,098592647	0	N.A.	0
BEX4	0,039584265	-0,18737712	0	N.A.	0
RGMA	0,039525215	-0,174090892	0	N.A.	0
ZSWIM1	0,039478251	-0,123350617	0	N.A.	0
LINC00524	0,039445413	-0,244473285	0	N.A.	0
RP11-348P10	0,039371729	0,301979141	0	N.A.	0
ZFYVE19	0,039344785	0,116403119	0	N.A.	0
MNX1-AS2	0,039243422	0,134118591	0	N.A.	0
LL22NC03-2H	0,039232608	0,275875513	0	N.A.	0
IKZF4	0,039203336	-0,24110859	0	N.A.	0
C9orf69	0,03916048	0,225989971	0	N.A.	0
CEACAM8	0,039137951	0,241452567	0	N.A.	0
C9orf172	0,039063907	0,238465444	0	N.A.	0
WFS1	0,039054743	0,303481508	0	N.A.	0
STK32A	0,039043867		0	N.A.	0
RP11-440G5.	0,038995153		0	N.A.	0

TYW5	0,038978321	0,126074638	0	N.A.	0
ANKRD27	0,038934895	-0,066924774	0	N.A.	0
FAM209B	0,038907498	0,019295354	0	N.A.	0
RP5-875H18.	0,038900638	0,090280148	0	N.A.	0
PALM2	0,038877772	-0,05980259	0	N.A.	0
EXTL1	0,03878709		0	N.A.	0
MVB12B	0,038784629	-0,194495094	0	N.A.	0
MOGAT1	0,038783414	0,020852719	0	N.A.	0
SLC22A15	0,038782876	0,126703797	0	N.A.	0
CCS	0,03878097	0,324791664	0	N.A.	0
ZNF321P	0,038765798	-0,152063689	0	N.A.	0
CAP2	0,038763875	-0,076709858	0	N.A.	0
FGA	0,038761358		0	N.A.	0
DGKB	0,038749583		0	N.A.	0
KCNAB3	0,03873005	0,038686074	0	N.A.	0
TLN1	0,038699025	-0,09484452	0	N.A.	0
FAM101B	0,038679134	-0,021752713	0	N.A.	0
MIER2	0,038646172	0,155307373	0	N.A.	0
LMBR1	0,038632278	-0,018398878	0	N.A.	0
SLC1A5	0,03862023	0,438556979	0	N.A.	0
CDK11A	0,038610297	0,106970034	0	N.A.	0
CTD-3060P21	0,038608102	0,258865363	0	N.A.	0
SMARCA2	0,038591616	0,1502535	0	N.A.	0
TIMP4	0,038509031	-0,083951572	0	N.A.	0
NAA30	0,038505093	0,060016251	0	N.A.	0
TMEM74B	0,038491516	0,162574107	0	N.A.	0
LINC00235	0,038450691	0,02008516	0	N.A.	0
RP11-227G15	0,038420659	0,063922833	0	N.A.	0
RP11-321G12	0,038415327	-0,054569319	0	N.A.	0
EFCAB6	0,038396404	-0,0742273	0	N.A.	0
CCR6	0,038389486	-0,11997447	0	N.A.	0
CERS1	0,038381573	0,031578778	0	N.A.	0
AIRE	0,038371144		0	N.A.	0

NECAP1	0,038278684	0,197427007	0	N.A.	0
XKR8	0,038221189	-0,020789891	0	N.A.	0
NKIRAS1	0,038201121	-0,004075897	0	N.A.	0
TMEM161B	0,038185959	0,104487494	0	N.A.	0
AP000708.1	0,038137177		0	N.A.	0
RP11-60A8.1	0,038135456	-0,118168831	0	N.A.	0
HAND2-AS1	0,038094637	-0,044303254	0	N.A.	0
GLIS2-AS1	0,038092184	0,032314066	0	N.A.	0
CTB-102L5.4	0,03808773	0,179476454	0	N.A.	0
ANKRD44	0,038087342	-0,205794266	0	N.A.	0
RP11-436K8.	0,038083973		0	N.A.	0
PPP1R9B	0,038037077	-0,033962089	0	N.A.	0
DGCR8	0,038009675	0,227950471	0	N.A.	0
CSNK1G1	0,038003768	0,157277227	0	N.A.	0
RFWD2	0,037962745	0,096064302	0	N.A.	0
CTDP1	0,03795697	-0,065985681	0	N.A.	0
RFX2	0,037938137	-0,022706746	0	N.A.	0
HIST1H2AK	0,037898213	-0,144539615	0	N.A.	0
AC008079.10	0,037846697	0,166232703	0	N.A.	0
RP4-800J21.3	0,037800478	0,035901515	0	N.A.	0
CERS5	0,0377783	0,043315763	0	N.A.	0
LINC01068	0,037716157	0,077239807	0	N.A.	0
FOXP4	0,037672404	0,291836476	0	N.A.	0
SSC5D	0,037667555	0,324906067	0	N.A.	0
WIPF3	0,037666298	-0,093868671	0	N.A.	0
HPS6	0,037662217	-0,072622107	0	N.A.	0
LPO	0,037651978	-0,049496528	0	N.A.	0
BAG5	0,037596514	0,151977779	0	N.A.	0
CSNK1A1L	0,037581598	0,125373515	0	N.A.	0
LURAP1	0,037574514		0	N.A.	0
GATA2-AS1	0,037564118	0,070513871	0	N.A.	0
LINC01170	0,037559824	0,047932409	0	N.A.	0
RP11-1293J1	0,037507578		0	N.A.	0

AC007879.2	0,037496231	-0,017338627	0	N.A.	0
SNAI3-AS1	0,037495811	0,17378084	0	N.A.	0
SHOX2	0,037455983	0,071856897	0	N.A.	0
RP3-467L1.4	0,037441224	-0,091790434	0	N.A.	0
AKR1B10	0,037427914	0,087597245	0	N.A.	0
DEAF1	0,037395062	0,064738716	0	N.A.	0
RBFOX1	0,037386148		0	N.A.	0
ZNF343	0,03735565	-0,051440229	0	N.A.	0
GNGT2	0,037324331	-0,007592587	0	N.A.	0
AC015971.2	0,037270536	0,312683834	0	N.A.	0
ZNF557	0,037209835	-0,006056809	0	N.A.	0
CFP	0,037200108		0	N.A.	0
RP11-345P4.	0,037162084	0,418581038	0	N.A.	0
DIO2	0,037126073	0,035098787	0	N.A.	0
TMEM63C	0,037067699	-0,243295173	0	N.A.	0
HOXD1	0,037059568	0,306498973	0	N.A.	0
CLCN5	0,037042791	0,126876549	0	N.A.	0
FMN1	0,036989059	0,180008794	0	N.A.	0
PURA	0,036988003	0,329242879	0	N.A.	0
RP11-363E6.	0,036987009	0,132799983	0	N.A.	0
FGF12	0,036956283	-0,020015494	0	N.A.	0
CTC-548K16.	0,036941075	-0,050748133	0	N.A.	0
RNF112	0,036836938		0	N.A.	0
DPH1	0,036773076	-0,099507844	0	N.A.	0
CTD-3128G1(	0,036765727		0	N.A.	0
VASN	0,036758073	0,404038044	0	N.A.	0
FZD6	0,036714107	0,296114931	0	N.A.	0
AC104532.4	0,036711877	-0,059755392	0	N.A.	0
CAMK2N2	0,036647152	-0,05245725	0	N.A.	0
AC097721.2	0,036642244	-0,021075312	0	N.A.	0
C9orf57	0,036602437	-0,071792181	0	N.A.	0
AC093375.1	0,036570871	-0,04349788	0	N.A.	0
MMP13	0,036569557	-0,013200768	0	N.A.	0

ULBP3	0,036559101	-0,097726589	0	N.A.	0
CCDC125	0,036556583	-0,102565351	0	N.A.	0
JMJD6	0,036515656	-0,229097426	0	N.A.	0
PP7080	0,036503267	0,130951051	0	N.A.	0
RP11-334J6.7	0,03649828	0,055827894	0	N.A.	0
APOBEC3B	0,036497777	-0,000748349	0	N.A.	0
RP11-430C7.	0,0364681		0	N.A.	0
ZNF70	0,036429942	-0,116108545	0	N.A.	0
MAPT	0,036262408	0,128272396	0	N.A.	0
FOXA3	0,036237074	0,219283086	0	N.A.	0
RP11-284M1	0,036234126	0,126684386	0	N.A.	0
PDGFRB	0,036206884	-0,124410898	0	N.A.	0
DIS3	0,036199679	0,155463649	0	N.A.	0
CTC-455F18.	0,036194154	-0,180450256	0	N.A.	0
LINC00324	0,036171922	0,048551784	0	N.A.	0
SNX7	0,036167544	0,035854418	0	N.A.	0
RP13-270P17	0,036080953	-0,111131125	0	N.A.	0
TRMT44	0,036079035	-0,21151506	0	N.A.	0
RARRES2	0,036059461	-0,378867159	0	N.A.	0
PLEKHF1	0,036037836	-0,374114097	0	N.A.	0
RAP2C-AS1	0,036022323	0,098584561	0	N.A.	0
MIS18A-AS1	0,036012855	-0,077614022	0	N.A.	0
PPP1R13B	0,035984839	0,242905749	0	N.A.	0
STAG3	0,035957669	-0,041610503	0	N.A.	0
MORN5	0,035943059	-0,267387159	0	N.A.	0
RP11-46D6.1	0,035937846	0,432723375	0	N.A.	0
WBSCR28	0,035913168	0,113888514	0	N.A.	0
KBTBD4	0,035878176	-0,066227353	0	N.A.	0
RBM23	0,035791526	0,141981671	0	N.A.	0
AC091801.1	0,035720899	-0,083490092	0	N.A.	0
APLNR	0,035714563	0,200766131	0	N.A.	0
CLECL1	0,03571018	-0,225163244	0	N.A.	0
TUSC1	0,035694503	-0,243471677	0	N.A.	0

RP11-768F21	0,035681403		0	N.A.	0
FBXO44	0,035661101	0,102133767	0	N.A.	0
CELA2A	0,035641671		0	N.A.	0
AC007038.7	0,035622411	0,075157046	0	N.A.	0
TEX22	0,035617245	-0,045461917	0	N.A.	0
RP11-203H2.	0,035611694	0,017171152	0	N.A.	0
NBEAL2	0,035549707	0,229335069	0	N.A.	0
GLRA2	0,035501836		0	N.A.	0
HID1	0,035472851	0,251025491	0	N.A.	0
RP4-564F22.7	0,035457148	-0,044999068	0	N.A.	0
LINC00540	0,035452516	-0,120465437	0	N.A.	0
RP11-800A3.	0,035426709	0,078373631	0	N.A.	0
CTC-209H22.	0,035351626	0,158747665	0	N.A.	0
RNF146	0,03531338	-0,068002168	0	N.A.	0
TSSK6	0,035298464	-0,000555223	0	N.A.	0
RP5-1186P10	0,035261572	-0,045506036	0	N.A.	0
SNX21	0,035253116	-0,013936918	0	N.A.	0
CCDC157	0,035181189	0,035821098	0	N.A.	0
CYP19A1	0,035154855		0	N.A.	0
LINC00869	0,035113126	-0,11185571	0	N.A.	0
TTLL3	0,035049329	0,118433842	0	N.A.	0
RP11-667K14	0,034974843		0	N.A.	0
RP11-1006G1	0,034974062	0,227578377	0	N.A.	0
C7orf31	0,034964307	-0,029500026	0	N.A.	0
CD5	0,034944693	0,047244184	0	N.A.	0
SH2D4A	0,034889979	0,393203825	0	N.A.	0
SENP2	0,034878804	0,096874019	0	N.A.	0
CTD-2013N24	0,034867952	-0,045397069	0	N.A.	0
RP13-131K19	0,034853601	-0,172731386	0	N.A.	0
RP3-475N16.	0,034799441	0,106916349	0	N.A.	0
RP4-625H18.	0,034782534	0,055038331	0	N.A.	0
ZNF616	0,034772698	-0,020485192	0	N.A.	0
C3orf35	0,034772338	0,153977909	0	N.A.	0

ATP6V0C	0,034764988	0,162595999	0	N.A.	0
MAML3	0,034708882	0,208254101	0	N.A.	0
CYP27C1	0,034705789	0,128064066	0	N.A.	0
CPNE9	0,034686648	-0,14963029	0	N.A.	0
DLC1	0,034682989	-0,068632741	0	N.A.	0
DLGAP1-AS2	0,034637548	-0,11406037	0	N.A.	0
LA16c-358B7	0,034604688	-0,125267546	0	N.A.	0
AC007128.1	0,03458968	-0,075643239	0	N.A.	0
NBPF11	0,034588914	0,118123663	0	N.A.	0
ZNF396	0,034571642	-0,110415606	0	N.A.	0
MYO5A	0,034545285	0,335769017	0	N.A.	0
FAM186B	0,034535673	0,133904438	0	N.A.	0
CD38	0,034532029	-0,101049601	0	N.A.	0
AC159540.1	0,034521675	0,247601754	0	N.A.	0
HLA-DQA1	0,034494405	-0,352226755	0	N.A.	0
INPP5E	0,03449089	0,018468558	0	N.A.	0
TNFSF12	0,034478616	-0,187933243	0	N.A.	0
RP11-394O4.	0,034463045	-0,088010483	0	N.A.	0
RP11-48G14.	0,034456938	-0,13423057	0	N.A.	0
RP11-347C12	0,034429097	-0,100251144	0	N.A.	0
ACTN2	0,034385738	-0,055617827	0	N.A.	0
ANKHD1	0,034384058	0,076932784	0	N.A.	0
TMEM171	0,034383008	0,059417876	0	N.A.	0
RP13-216E22	0,034360823	0,00531646	0	N.A.	0
RP11-533E19	0,034359944	-0,015861564	0	N.A.	0
MLX	0,034344467	0,399360365	0	N.A.	0
DAAM2	0,034322631	-0,049688007	0	N.A.	0
RFTN1	0,034317152	-0,040387737	0	N.A.	0
CHST11	0,034316268	-0,004747702	0	N.A.	0
PAPD4	0,034303207	-0,025017563	0	N.A.	0
RP11-393I2.4	0,034298583	0,179779969	0	N.A.	0
RP11-1277A3	0,034258555	0,414226354	0	N.A.	0
NAIF1	0,034220725	-0,087630272	0	N.A.	0

TRAPPC12-AS	0,034201003	-0,026235528	0	N.A.	0
IREB2	0,034176421	0,242271321	0	N.A.	0
DHRSX	0,034172015	0,000870085	0	N.A.	0
RP11-259K5.	0,034111565	0,363817778	0	N.A.	0
UCN3	0,03411091	-0,217825741	0	N.A.	0
RP3-508I15.1	0,034108193		0	N.A.	0
LINC01001	0,034106899	0,276440761	0	N.A.	0
RP11-195C7.	0,034076431	-0,069623726	0	N.A.	0
MMP3	0,034047977	-0,26624711	0	N.A.	0
ZCCHC24	0,03398995	-0,026405847	0	N.A.	0
MERTK	0,033969441		0	N.A.	0
CTD-2005H7.	0,033950682		0	N.A.	0
NPIPA1	0,033879006	-0,030400658	0	N.A.	0
LOXL1-AS1	0,03386964	-0,030574007	0	N.A.	0
ARHGEF17	0,033869532	0,19653849	0	N.A.	0
EIF4EBP3	0,033848376	-0,123154004	0	N.A.	0
LINC00662	0,03381338	-0,0066826	0	N.A.	0
ZNF529	0,033696604	-0,030912272	0	N.A.	0
RP11-452L6.	0,033693236	0,039821545	0	N.A.	0
RAB11B-AS1	0,033685151	-0,132519208	0	N.A.	0
RP11-1114A5	0,03367688	0,09746014	0	N.A.	0
CTA-228A9.3	0,033676636	0,082206614	0	N.A.	0
ZNF653	0,033653505	-0,044565843	0	N.A.	0
LDB3	0,03365312		0	N.A.	0
CDRT4	0,033627777	0,169065021	0	N.A.	0
KIF26B	0,033577882	-0,042949763	0	N.A.	0
RBM12B	0,033444514	0,001070996	0	N.A.	0
ESYT3	0,033444154	-0,187990804	0	N.A.	0
LMCD1-AS1	0,033441218	0,117758558	0	N.A.	0
ZBTB25	0,033362235	0,195111495	0	N.A.	0
AQP6	0,033362058		0	N.A.	0
REG4	0,033343171	0,199883373	0	N.A.	0
USO1	0,033339699	0,331154702	0	N.A.	0

RP11-712B9.	0,033316817	-0,029281239	0	N.A.	0
RP11-168O1(	0,033277488		0	N.A.	0
AC006004.1	0,033270045	0,194067119	0	N.A.	0
CELSR3-AS1	0,033266695		0	N.A.	0
ATP13A3	0,03325695	0,220886616	0	N.A.	0
HNRNPU-AS1	0,033241351	0,242756508	0	N.A.	0
WDR31	0,033206214	-0,174134281	0	N.A.	0
TSC22D1-AS1	0,033186693	-0,109515401	0	N.A.	0
PLD5	0,033181963	-0,308079787	0	N.A.	0
BMP3	0,033148066		0	N.A.	0
KCNMB3	0,033105136	0,199432364	0	N.A.	0
VMAC	0,033088284	0,089663581	0	N.A.	0
ERN2	0,033068475	0,19652859	0	N.A.	0
IGHV3-74	0,033047145		0	N.A.	0
XCL2	0,033043419		0	N.A.	0
TSLP	0,033019117	-0,17332353	0	N.A.	0
VSIG10L	0,033016533	0,352434293	0	N.A.	0
CATSPER3	0,033015699	0,024350113	0	N.A.	0
VKORC1L1	0,033008015	0,256007595	0	N.A.	0
SMC5-AS1	0,032998633	-0,301288013	0	N.A.	0
YY2	0,032983393	0,165834708	0	N.A.	0
ZNF680	0,032978275	0,183249441	0	N.A.	0
HES5	0,032969159	0,067041463	0	N.A.	0
TMEM234	0,032945104	0,106919868	0	N.A.	0
SLC31A1	0,032935257	0,151530645	0	N.A.	0
RP13-638C3.	0,032892615	0,057006712	0	N.A.	0
ZNF233	0,032848566	-0,047350925	0	N.A.	0
RP11-66B24.	0,032824212	-0,190501991	0	N.A.	0
RP1-65J11.1	0,032812411	-0,138379721	0	N.A.	0
FOXN1	0,032811615	0,021956489	0	N.A.	0
HSD17B14	0,032794859	-0,222119056	0	N.A.	0
AC005253.2	0,032793118	-0,083841005	0	N.A.	0
PIPOX	0,032761303	-0,00060493	0	N.A.	0

FRRS1	0,032737354	0,005332148	0	N.A.	0
TMEM163	0,032715336	-0,106069038	0	N.A.	0
KB-1125A3.1	0,032709055	0,028044813	0	N.A.	0
IGKV1-17	0,03268993	-0,287041343	0	N.A.	0
GRAPL	0,032686469		0	N.A.	0
RP3-508I15.9	0,032676303	-0,072871766	0	N.A.	0
MSANTD1	0,03264931	0,070347577	0	N.A.	0
ARL4A	0,032634725	0,177483568	0	N.A.	0
HSPB3	0,03263022	0,372622198	0	N.A.	0
CBX6	0,032622413	0,097995572	0	N.A.	0
POM121	0,032622143	0,141437137	0	N.A.	0
MAP2K5	0,032565664	-0,119666722	0	N.A.	0
RP11-354E11	0,032557299	-0,105425003	0	N.A.	0
TMEM145	0,032554555	-0,189144087	0	N.A.	0
GLDN	0,032503781	0,024631035	0	N.A.	0
AC106900.6	0,03250007		0	N.A.	0
PPP1R3F	0,032495054	-0,118995483	0	N.A.	0
CPXM1	0,032489395	-0,063026766	0	N.A.	0
CD160	0,03248939	0,05059413	0	N.A.	0
PYGM	0,032455546	0,122334311	0	N.A.	0
RP11-700J17	0,032453084	0,125389303	0	N.A.	0
ABO	0,03237394	0,24379813	0	N.A.	0
FOXD2-AS1	0,032364858	-0,126031586	0	N.A.	0
C16orf71	0,032364174	0,182077649	0	N.A.	0
TFEC	0,032351136	-0,28915815	0	N.A.	0
SPTLC2	0,032296846	0,625414331	0	N.A.	0
PLEK	0,03228301	0,010147516	0	N.A.	0
RP11-98D18.	0,032201602	-0,060508802	0	N.A.	0
LINC01212	0,032192541		0	N.A.	0
NAPSA	0,032183418	0,210495439	0	N.A.	0
M1AP	0,03218159	0,145114787	0	N.A.	0
HOPX	0,032133594	0,037870233	0	N.A.	0
AL365181.2	0,032095575	0,218981406	0	N.A.	0

IGLV3-10	0,032088785		0	N.A.	0
TANC2	0,032060602	0,243189096	0	N.A.	0
C7orf25	0,032032614	-0,365175704	0	N.A.	0
PRF1	0,032026427	0,059842022	0	N.A.	0
ANKMY2	0,032025602	0,001228128	0	N.A.	0
DMBT1	0,032005571	0,019676303	0	N.A.	0
EBI3	0,031986219		0	N.A.	0
KRTAP4-1	0,031966392	0,237079807	0	N.A.	0
HOXB-AS1	0,031953344	-0,033821428	0	N.A.	0
TTC37	0,031939326	0,043732825	0	N.A.	0
TTC39C-AS1	0,031938385		0	N.A.	0
SNX4	0,031925987	-0,104739942	0	N.A.	0
FALEC	0,031915502	0,013548433	0	N.A.	0
ZNF793	0,031887644	-0,068599816	0	N.A.	0
DPF3	0,031879569	0,003500256	0	N.A.	0
CDON	0,031769929	0,031659846	0	N.A.	0
PRKCG	0,031738302	-0,049562048	0	N.A.	0
HLA-DQB2	0,031710523		0	N.A.	0
FAM208A	0,031673349	0,206359704	0	N.A.	0
KIAA1958	0,0316504	-0,188572032	0	N.A.	0
KCTD21-AS1	0,031617477	0,154575741	0	N.A.	0
P2RY1	0,031588861	-0,101777537	0	N.A.	0
LTN1	0,03158172	0,197590589	0	N.A.	0
PJA1	0,031550878	-0,055959137	0	N.A.	0
EDA2R	0,031498439		0	N.A.	0
VASH1	0,031456703	-0,195356412	0	N.A.	0
ZNF578	0,031435959	0,171092318	0	N.A.	0
RP11-30K9.6	0,031423965	-0,266886029	0	N.A.	0
DEC1	0,03141442		0	N.A.	0
CCDC23	0,031409054	0,12036085	0	N.A.	0
RP11-165J3.6	0,031374465	-0,041761932	0	N.A.	0
AC004980.11	0,031364825	0,072097382	0	N.A.	0
OSER1-AS1	0,031348649	-0,085416664	0	N.A.	0

CTD-2574D2:	0,031283297		0	N.A.	0
PTOV1-AS1	0,03125529	0,078767149	0	N.A.	0
FAM21C	0,031245676	0,274110851	0	N.A.	0
CPSF7	0,031239659	0,042989274	0	N.A.	0
AC092669.3	0,031208998	0,196424055	0	N.A.	0
CFAP44	0,03120622	0,066035794	0	N.A.	0
RP11-1007O:	0,03120391	-0,011373925	0	N.A.	0
TSPAN10	0,031158261	-0,092357933	0	N.A.	0
IPCEF1	0,031157876	0,00402868	0	N.A.	0
RP13-977J11	0,031155454	0,260875845	0	N.A.	0
SCMH1	0,031137137	-0,253419664	0	N.A.	0
FN3K	0,031115696	0,253417551	0	N.A.	0
STRN	0,031049615	-0,022840331	0	N.A.	0
TBC1D12	0,031047469	-0,028090992	0	N.A.	0
GALNT4	0,031010832	0,002584294	0	N.A.	0
RP11-796E10	0,030994161	-0,107687351	0	N.A.	0
RP11-378J18	0,030986783		0	N.A.	0
AC004076.5	0,030950568	0,337447321	0	N.A.	0
RP3-416H24.	0,030943583	0,026821521	0	N.A.	0
RP1-50J22.4	0,030940343		0	N.A.	0
HIST1H1E	0,030881944	-0,194981737	0	N.A.	0
RTN4RL2	0,030864448	0,003634315	0	N.A.	0
ITPKB-AS1	0,030849689		0	N.A.	0
C8orf89	0,030842718		0	N.A.	0
F13A1	0,030839778	-0,005883238	0	N.A.	0
RHPN1-AS1	0,030770754	0,063766285	0	N.A.	0
SLC6A4	0,030732634	0,070460679	0	N.A.	0
CTD-3075F15	0,030727358	-0,11239796	0	N.A.	0
THUMPD3-A:	0,030713721	0,103716752	0	N.A.	0
EDC3	0,030713557	0,07395209	0	N.A.	0
RP11-809N8.	0,03070481	0,02386129	0	N.A.	0
AKIRIN1	0,030666676	-0,066540116	0	N.A.	0
CRKL	0,030650114	-0,070303186	0	N.A.	0

GSN-AS1	0,030610551		0	N.A.	0
RP11-692D12	0,030582393	0,073543388	0	N.A.	0
CLEC10A	0,030570036		0	N.A.	0
RNPC3	0,03056425	0,306771019	0	N.A.	0
BPI	0,030550019		0	N.A.	0
AGBL2	0,030529629	-0,071504395	0	N.A.	0
ZNF385C	0,030505947	-0,230695572	0	N.A.	0
TNNC2	0,030496798	0,044276669	0	N.A.	0
DCUN1D1	0,030495638	-0,080429184	0	N.A.	0
GABARAPL2	0,030464385	0,109325987	0	N.A.	0
RP11-481J2.3	0,030439625	-0,095545779	0	N.A.	0
GPR160	0,030431651	-0,003219314	0	N.A.	0
CTD-2568A17	0,030406812		0	N.A.	0
ITPRIP	0,030336217	0,142524257	0	N.A.	0
AE000662.92	0,030312701		0	N.A.	0
ARL4D	0,030308766	-0,15489023	0	N.A.	0
CHRNA7	0,030297603	0,052589963	0	N.A.	0
IL11	0,030294214	0,395717096	0	N.A.	0
C9orf16	0,030240576	-0,077736597	0	N.A.	0
CDH26	0,030219672		0	N.A.	0
AC010127.3	0,030216431		0	N.A.	0
UGT2A3	0,030189982	-0,092970468	0	N.A.	0
LAT	0,030174994	0,133629092	0	N.A.	0
LARP1B	0,030133957	0,173890085	0	N.A.	0
TMED1	0,030117029	0,230803144	0	N.A.	0
AVIL	0,03011546	0,247407849	0	N.A.	0
TOPORS-AS1	0,030112346	-0,000694392	0	N.A.	0
C19orf38	0,030112271	0,08346109	0	N.A.	0
AZGP1	0,030096457	0,388419231	0	N.A.	0
UBN1	0,030064998	0,313526279	0	N.A.	0
DYNLRB2	0,030047043	-0,162277345	0	N.A.	0
TFG	0,030028974	0,393305136	0	N.A.	0
AC068499.10	0,02991921		0	N.A.	0

RP11-160H22	0,029907314		0	N.A.	0
RP11-307B6.	0,029873641	0,06195142	0	N.A.	0
MYLK3	0,029870193	0,116979116	0	N.A.	0
ANKRD49	0,029828277	0,054652953	0	N.A.	0
ZBTB37	0,029816482	-0,036719595	0	N.A.	0
TFRC	0,029815164	0,31886016	0	N.A.	0
CBFA2T2	0,029764207	0,079607937	0	N.A.	0
SLC24A3	0,029717931	0,004776541	0	N.A.	0
RP11-547D24	0,029680023	-0,237828663	0	N.A.	0
RP11-299G20	0,029667797	0,234296469	0	N.A.	0
TNMD	0,029642248	-0,273236942	0	N.A.	0
RP11-706O15	0,02961384		0	N.A.	0
FCN3	0,029600138	0,037528515	0	N.A.	0
FAM222A	0,029594664	-0,153539444	0	N.A.	0
GS1-166A23.	0,029588722		0	N.A.	0
IL21R	0,029549612		0	N.A.	0
NRAP	0,029533909	0,026283336	0	N.A.	0
AIF1	0,029496218	-0,168610509	0	N.A.	0
HPSE	0,029465557	0,258224001	0	N.A.	0
RP11-649G15	0,029459928	0,129858852	0	N.A.	0
CTD-2528L19	0,029368292	-0,187377169	0	N.A.	0
NCALD	0,029346334	-0,045236562	0	N.A.	0
RP11-1060G2	0,02930772		0	N.A.	0
RAB11A	0,029275456	0,000871081	0	N.A.	0
UBXN11	0,029262881	-0,066297183	0	N.A.	0
INTS6	0,029261148	0,314287018	0	N.A.	0
NEBL-AS1	0,029247766	0,085140004	0	N.A.	0
SLC47A1	0,029242937		0	N.A.	0
GPR144	0,029238936		0	N.A.	0
RARB	0,029237442		0	N.A.	0
FCAR	0,029236422		0	N.A.	0
SLC16A14	0,029235787	0,274705285	0	N.A.	0
MCTP1	0,029201779	-0,207913076	0	N.A.	0

LMBRD2	0,029168642	0,209935062	0	N.A.	0
CTD-2587M2	0,029155622	0,009735997	0	N.A.	0
RP3-355L5.5	0,029098912	0,230597364	0	N.A.	0
CNOT4	0,029089024	0,108151623	0	N.A.	0
BCL2L13	0,029060884	-0,008516194	0	N.A.	0
LINC00304	0,029033925		0	N.A.	0
CLINT1	0,028978798	0,33204669	0	N.A.	0
BATF3	0,028972945	-0,017414213	0	N.A.	0
ZNF337-AS1	0,028928322	-0,275863394	0	N.A.	0
ZBTB11	0,028924213	0,064667759	0	N.A.	0
TLR10	0,028920835	-0,144327963	0	N.A.	0
WDR81	0,028897908	0,264516422	0	N.A.	0
SLFN13	0,028893723	-0,31590888	0	N.A.	0
MAP3K3	0,028882959	0,091798041	0	N.A.	0
STX18-AS1	0,02886798	-0,111571663	0	N.A.	0
RP11-1348G1	0,028865333	0,267838221	0	N.A.	0
RP11-347C12	0,028829971	0,254539502	0	N.A.	0
RP11-396B14	0,028810355	0,206136542	0	N.A.	0
CYP3A43	0,028709329		0	N.A.	0
DKKL1	0,028693877		0	N.A.	0
LEMD2	0,028689942	0,21628229	0	N.A.	0
SOWAHD	0,02867372	0,311981595	0	N.A.	0
CEP85L	0,028650071	-0,083961277	0	N.A.	0
ZBTB26	0,028621525	-0,113562143	0	N.A.	0
FGFR1	0,028611007	-0,174505937	0	N.A.	0
CLDN16	0,028610875	-0,273837676	0	N.A.	0
CNOT2	0,028610575	0,288907075	0	N.A.	0
LRP12	0,028608849	0,149071524	0	N.A.	0
FAM171A2	0,028586858		0	N.A.	0
SLC19A2	0,028581168	0,134895337	0	N.A.	0
RP11-715J22	0,028580941	0,008493465	0	N.A.	0
FOXP1	0,028554014	0,175417251	0	N.A.	0
AC137934.1	0,028528126	-0,040160303	0	N.A.	0

CCDC183	0,028490755	0,04372345	0	N.A.	0
GNG10	0,028461479	-0,102433154	0	N.A.	0
SCAND1	0,028419443	0,057957501	0	N.A.	0
DMGDH	0,02840658	0,054574867	0	N.A.	0
AC016738.4	0,028405063		0	N.A.	0
LENG9	0,028388608	0,25125698	0	N.A.	0
IL12A	0,028382567	0,038020906	0	N.A.	0
RP4-545C24.	0,02831924		0	N.A.	0
MEMO1	0,028315497	-0,024533386	0	N.A.	0
PLA2G2C	0,028308754		0	N.A.	0
SEC16B	0,028287838	0,132073476	0	N.A.	0
RP11-45A17.	0,028262067	-0,044767076	0	N.A.	0
RP11-219B4.	0,028249752	0,030385504	0	N.A.	0
CLEC18A	0,028244773		0	N.A.	0
CTC-480C2.1	0,028131281	-0,062955222	0	N.A.	0
RP11-264B17	0,028125557	0,120159069	0	N.A.	0
CARF	0,028117126	-0,144878978	0	N.A.	0
ZNF33B	0,02811259	-0,057012579	0	N.A.	0
FGF7	0,02807005	-0,219156997	0	N.A.	0
TMEM232	0,028044576	0,114691793	0	N.A.	0
MAFIP	0,028043685	-0,092286758	0	N.A.	0
CTNNA2	0,028004192	-0,143738911	0	N.A.	0
RP11-817O1	0,027972838	-0,159902328	0	N.A.	0
RP11-486O1	0,027967873	-0,154751846	0	N.A.	0
CHI3L1	0,027957438	0,131294413	0	N.A.	0
AC093159.1	0,027953759	-0,085065704	0	N.A.	0
SLC7A10	0,027935151		0	N.A.	0
KIR2DL4	0,027884044		0	N.A.	0
KLHL41	0,027872124		0	N.A.	0
SLC25A16	0,027827013	-0,004011337	0	N.A.	0
SYK	0,027822072	0,055407412	0	N.A.	0
PARD3-AS1	0,02781246	0,129907836	0	N.A.	0
RP11-21L1.1	0,027804349	-2,57483E-05	0	N.A.	0

GNPTAB	0,027802342	-0,073716894	0	N.A.	0
FAM83C	0,027797318	-0,276747189	0	N.A.	0
TAS2R4	0,027795772	0,227161836	0	N.A.	0
TBXA2R	0,027788805	-0,044601153	0	N.A.	0
ZNF333	0,027787277	0,047865707	0	N.A.	0
TBC1D2B	0,027764028	0,042497337	0	N.A.	0
NELL2	0,027762968		0	N.A.	0
NTF3	0,027754083		0	N.A.	0
RP11-294N21	0,027731019		0	N.A.	0
CLK2	0,027689324	0,022414832	0	N.A.	0
CTB-13F3.1	0,02766379	-0,004504226	0	N.A.	0
ZNF671	0,027614146	-0,080170565	0	N.A.	0
RP11-509J21	0,027582042	-0,106571248	0	N.A.	0
RP11-158I9.8	0,027566005	-0,082138822	0	N.A.	0
L3MBTL1	0,027561404	0,030337701	0	N.A.	0
BTBD1	0,027539409	-0,117348953	0	N.A.	0
NLRP6	0,027528056	-0,039078951	0	N.A.	0
RP11-420A6.	0,027501718	0,134312687	0	N.A.	0
MSH5	0,02749669	0,118258524	0	N.A.	0
AP001062.7	0,027492153	0,117498368	0	N.A.	0
ZDBF2	0,027489236	0,168522863	0	N.A.	0
RP11-440D17	0,027480033		0	N.A.	0
RRM2B	0,027478002	-0,259837563	0	N.A.	0
DTHD1	0,027472517		0	N.A.	0
ADCY2	0,027457214		0	N.A.	0
FAM179A	0,027432729		0	N.A.	0
SPINK9	0,027412393		0	N.A.	0
RP11-689P11	0,027355897	0,06288085	0	N.A.	0
C4orf47	0,027348967	0,086023954	0	N.A.	0
RP11-61A14.	0,027334925	-0,188269342	0	N.A.	0
BLACAT1	0,027306206	0,079103064	0	N.A.	0
CTD-2666L21	0,027305407		0	N.A.	0
LINC00862	0,027304727	0,212849304	0	N.A.	0

TCHP	0,027293836	-0,24407448	0	N.A.	0
LINC00926	0,027292173	0,120111742	0	N.A.	0
RP11-489E7.	0,027235054	-0,049930244	0	N.A.	0
LIN54	0,027230887	-0,120905703	0	N.A.	0
RP11-105N1	0,027218323	0,101183741	0	N.A.	0
PLIN4	0,027204665		0	N.A.	0
RP11-381O7.	0,027200365		0	N.A.	0
HIST1H2BE	0,027160169	-0,249600668	0	N.A.	0
IL18BP	0,027139534	-0,071573965	0	N.A.	0
RP11-805I24	0,027137241	0,277984902	0	N.A.	0
FIZ1	0,027132091	0,075270207	0	N.A.	0
PCDH9	0,027096004	-0,013181315	0	N.A.	0
HEY1	0,027031612	-0,002473551	0	N.A.	0
CCDC80	0,026998956	0,216957704	0	N.A.	0
PHF3	0,02699825	0,050483435	0	N.A.	0
DISC1	0,026989803	-0,119384794	0	N.A.	0
ZNF844	0,026981224	0,085574686	0	N.A.	0
IGHG2	0,026884887	-0,011212937	0	N.A.	0
AP000253.1	0,026884736	0,050323856	0	N.A.	0
ERICH4	0,02686825		0	N.A.	0
VPS45	0,026837292	0,342479107	0	N.A.	0
AC005480.1	0,026817677	-0,222691337	0	N.A.	0
SAP30L	0,026812826	0,100787797	0	N.A.	0
GLUL	0,026752403	-0,056153451	0	N.A.	0
SHANK2	0,026744726	0,145563324	0	N.A.	0
GORAB	0,026743347	-0,076244757	0	N.A.	0
DLG1-AS1	0,026733763	-0,209481109	0	N.A.	0
CTC-250I14.6	0,026732091	0,180776676	0	N.A.	0
B3GNT9	0,026730512	0,12672677	0	N.A.	0
AC092301.3	0,026668963	0,128076149	0	N.A.	0
RP11-449P15	0,026667132	0,006599937	0	N.A.	0
RP11-686G8.	0,026640156	0,324373981	0	N.A.	0
HOXC9	0,026640033	0,118433903	0	N.A.	0

TUSC3	0,026521778	-0,04531655	0	N.A.	0
RNF148	0,026500324	0,16648459	0	N.A.	0
KIAA1467	0,026409537	0,038402074	0	N.A.	0
AC012368.1	0,026405603		0	N.A.	0
RP11-2C24.7	0,026385979	-0,085544105	0	N.A.	0
RP11-967K21	0,026299331	0,32272411	0	N.A.	0
UBR1	0,026257328	0,132734576	0	N.A.	0
STYXL1	0,026250572	0,027970871	0	N.A.	0
BZW1	0,026234066	-0,023847269	0	N.A.	0
CABP1	0,026187633	-0,324763779	0	N.A.	0
RBBP6	0,026179242	0,260155601	0	N.A.	0
NPIPB11	0,026163188	0,195192797	0	N.A.	0
RP11-22N19.	0,026158242	-0,001210997	0	N.A.	0
DCST2	0,026109956	0,028831242	0	N.A.	0
ZNF66	0,026078159	0,330609263	0	N.A.	0
LDOC1L	0,026069661	-0,326576895	0	N.A.	0
CPA3	0,026061464	0,015286399	0	N.A.	0
PAQR6	0,02601806	0,190125066	0	N.A.	0
C16orf70	0,026010173	0,085812139	0	N.A.	0
RP11-793H13	0,025987348	-0,197819517	0	N.A.	0
RASSF9	0,025967682	-0,061474091	0	N.A.	0
AXDND1	0,025950139	-0,061583315	0	N.A.	0
HOXB3	0,025924754	0,228291741	0	N.A.	0
FOXQ1	0,025913282	0,04852395	0	N.A.	0
HINFP	0,025898628	0,138758502	0	N.A.	0
NHLH1	0,025842951	0,102555456	0	N.A.	0
PLA2G12B	0,025827417	-0,109873238	0	N.A.	0
CD300LB	0,025825026		0	N.A.	0
RSPO2	0,025682659		0	N.A.	0
GS1-114I9.1	0,025605738		0	N.A.	0
MLXIPL	0,025604609	0,225450208	0	N.A.	0
RP1-267L14.(	0,025591779	0,173855662	0	N.A.	0
RP11-734K2.	0,025508548	-0,116369949	0	N.A.	0

FBXL8	0,02548894	0,291067639	0	N.A.	0
AP001437.1	0,025445222	0,024348573	0	N.A.	0
SOX30	0,025423111	0,052604178	0	N.A.	0
RP11-268G13	0,02538864	0,08058074	0	N.A.	0
FMO4	0,025371253	0,069207256	0	N.A.	0
HERC1	0,025363802	0,014949416	0	N.A.	0
LINC01503	0,025345765	-0,046721046	0	N.A.	0
SIGLEC1	0,025320619	-0,123886016	0	N.A.	0
IFI27L1	0,025320382	0,114686004	0	N.A.	0
SNX3	0,025311768	-0,149351484	0	N.A.	0
PAPOLA	0,025296449	0,090718518	0	N.A.	0
FAM78A	0,025295564		0	N.A.	0
PDE6A	0,025281057	0,092809015	0	N.A.	0
LINC00571	0,025258437	0,354816296	0	N.A.	0
TRIM54	0,02525769	-0,222529797	0	N.A.	0
PKP1	0,025231937	0,305685037	0	N.A.	0
P4HA3	0,025216966	0,067777467	0	N.A.	0
TMEM254	0,025202586	-0,263625611	0	N.A.	0
MSI2	0,025185315	0,211356854	0	N.A.	0
CTD-3001H11	0,02514629	0,133348763	0	N.A.	0
AIM1	0,025116434	0,087260994	0	N.A.	0
TLR5	0,025097177	-0,005995137	0	N.A.	0
CTD-2506P8.	0,02509391	-0,059414992	0	N.A.	0
LHX1	0,025073064	0,028339947	0	N.A.	0
RP11-46H11.	0,025070999	-0,053834883	0	N.A.	0
SMAP2	0,02506997	-0,032260756	0	N.A.	0
NOD1	0,02506821	-0,100746908	0	N.A.	0
STX16-NPEPL	0,025057165	-0,01079708	0	N.A.	0
ATF5	0,025028912	-0,022229334	0	N.A.	0
PRR22	0,024972965	0,162745928	0	N.A.	0
USP3	0,024950273	0,210153031	0	N.A.	0
ZBTB21	0,024944385	0,131508488	0	N.A.	0
AC005786.5	0,024868589		0	N.A.	0

ATXN7L2	0,024847046	0,075161518	0	N.A.	0
IL18RAP	0,024841861		0	N.A.	0
TLL2	0,024813544	-0,135481786	0	N.A.	0
RP5-1139B12	0,024808418		0	N.A.	0
STAG2	0,024750956	0,250377057	0	N.A.	0
ZMYND12	0,024677452	0,072130825	0	N.A.	0
TMED10	0,024664005	0,387031995	0	N.A.	0
LINC01460	0,024647138		0	N.A.	0
RP11-111F5.:	0,024633763	0,011097543	0	N.A.	0
ZNF304	0,024574754	0,199421163	0	N.A.	0
CHD3	0,024517355	-0,07021015	0	N.A.	0
ZFP62	0,024502299	-0,330368321	0	N.A.	0
EDNRB	0,024453906	0,197336201	0	N.A.	0
ACTR10	0,024443734	-0,050090263	0	N.A.	0
CH507-513H4	0,024413049		0	N.A.	0
INE2	0,024405267	0,249031159	0	N.A.	0
RP11-655M1	0,024382084	-0,088489805	0	N.A.	0
GRAMD2	0,02436071	0,132118527	0	N.A.	0
IZUMO1	0,024357122	-0,113980706	0	N.A.	0
DCP2	0,024325144	0,299854657	0	N.A.	0
CEP120	0,024309887	0,213971632	0	N.A.	0
RP11-55K13.	0,024303364	0,089717599	0	N.A.	0
ZBTB48	0,024302126	0,320567545	0	N.A.	0
GHRLOS	0,024297099	0,103643378	0	N.A.	0
DICER1-AS1	0,024282396	0,210915126	0	N.A.	0
RANBP6	0,0242736	0,033936972	0	N.A.	0
ARL17B	0,024250862	-0,060805432	0	N.A.	0
MAMSTR	0,024236016	-0,096589363	0	N.A.	0
PDIA2	0,024232982	-0,048807036	0	N.A.	0
AC145110.1	0,024226158		0	N.A.	0
ADPRHL1	0,024211028	-0,315166114	0	N.A.	0
RP11-345M2	0,024209302	0,245443889	0	N.A.	0
RP1-249I4.2	0,024203146		0	N.A.	0

LLGL1	0,024199152	0,399485192	0	N.A.	0
FAM212B	0,024118204	0,164138676	0	N.A.	0
RP11-21M24	0,02410303	0,056194818	0	N.A.	0
RP11-884K10	0,024088099		0	N.A.	0
RP11-53O19.	0,024033022	0,276669062	0	N.A.	0
RP11-309L24	0,023998369	0,012098758	0	N.A.	0
LRRC28	0,023943429	-0,119181833	0	N.A.	0
RP5-1056H1.	0,023878364	-0,008175608	0	N.A.	0
FYTTD1	0,023863834	-0,094382663	0	N.A.	0
AC074183.4	0,023847287	-0,151547463	0	N.A.	0
CNTD2	0,023797887	0,028845418	0	N.A.	0
ABCG2	0,023731726	0,263408527	0	N.A.	0
RP11-458D21	0,023707038	0,299253252	0	N.A.	0
POLR2J2	0,023646798	0,027182653	0	N.A.	0
RP11-296O14	0,023633386		0	N.A.	0
SDS	0,02359128	-0,164500728	0	N.A.	0
RP11-734K21	0,023566718		0	N.A.	0
KLHL5	0,023564531	-0,109498848	0	N.A.	0
RP11-1057B6	0,023561814		0	N.A.	0
AC004009.3	0,023535111		0	N.A.	0
FUT8	0,023526641	0,100808982	0	N.A.	0
RP4-789D17.	0,023524735	0,058329981	0	N.A.	0
CCR7	0,023503337	-0,297317956	0	N.A.	0
FAM210B	0,023472007	0,297613347	0	N.A.	0
RP4-616B8.4	0,023468832	-0,018644341	0	N.A.	0
SPARCL1	0,023426301	0,134782418	0	N.A.	0
PACSIN3	0,02333304	0,189156556	0	N.A.	0
RP11-22P6.2	0,02333197	0,138511921	0	N.A.	0
CTD-2527I21	0,023329776		0	N.A.	0
AC010096.1	0,023306896		0	N.A.	0
PCDHB10	0,023281551	0,020816288	0	N.A.	0
RP4-669H2.1	0,023276762	0,181011341	0	N.A.	0
RP11-789C1.	0,023264939	-0,263532863	0	N.A.	0

MZB1	0,023256441	-0,190954411	0	N.A.	0
WNT4	0,023229901	-0,078086419	0	N.A.	0
USP26	0,023193043		0	N.A.	0
LIPE-AS1	0,023180778	-0,062078352	0	N.A.	0
FRMD6	0,023146377	0,143794203	0	N.A.	0
ANKMY1	0,023137686	0,098491062	0	N.A.	0
TBX6	0,023096535	0,207455973	0	N.A.	0
PAPLN	0,023083348		0	N.A.	0
RP11-767C1.	0,023077797	0,126027941	0	N.A.	0
RP5-1050D4.	0,023064127	-0,043732013	0	N.A.	0
DBN1	0,023018273	0,433082133	0	N.A.	0
PCDHB7	0,022977499	0,140322187	0	N.A.	0
RP1-28H20.3	0,022975429	0,137231506	0	N.A.	0
BPNT1	0,022969042	-0,014284758	0	N.A.	0
SURF1	0,022953772	0,135375206	0	N.A.	0
MFAP3	0,022904099	0,066269511	0	N.A.	0
HOTTIP	0,022893887	0,12631727	0	N.A.	0
CBLN4	0,022880589		0	N.A.	0
RP1-206D15.	0,022847377	0,008577696	0	N.A.	0
CAPN10	0,022835264	0,345362771	0	N.A.	0
RASGEF1A	0,02281017	0,138231596	0	N.A.	0
IRF2BP2	0,022731426	0,355407216	0	N.A.	0
CTD-2034I4.2	0,022714248		0	N.A.	0
SCNN1G	0,022713731	0,301828534	0	N.A.	0
EXT2	0,022705835	0,426075701	0	N.A.	0
RP11-21B23.	0,022695806	0,398737047	0	N.A.	0
SEZ6	0,02268774		0	N.A.	0
TTC9C	0,022665924	-0,096607509	0	N.A.	0
CCDC81	0,022644283	0,053915578	0	N.A.	0
RP11-206L10	0,022611314	-0,026579847	0	N.A.	0
RP11-576N17	0,022594436		0	N.A.	0
RP11-315O6.	0,022591827	-0,288471993	0	N.A.	0
GPR25	0,022570145		0	N.A.	0

AC068831.10	0,022548068	-0,009560142	0	N.A.	0
DLGAP3	0,02251011	0,289747232	0	N.A.	0
TNFRSF17	0,022483725		0	N.A.	0
C10orf111	0,022460708	0,306805328	0	N.A.	0
DYNC1I2	0,022393594	0,535466344	0	N.A.	0
MCF2L	0,022387835	-0,007908949	0	N.A.	0
CELF5	0,022369853	-0,135844488	0	N.A.	0
CTD-2201I18	0,022359758	-0,066647338	0	N.A.	0
HR	0,022279582	0,109615047	0	N.A.	0
RP11-527J8.1	0,022243568	0,333407989	0	N.A.	0
RAB11FIP2	0,022229378	-0,032269321	0	N.A.	0
CCDC132	0,022229116	0,009363971	0	N.A.	0
PCDHB9	0,02221311	0,26628246	0	N.A.	0
CROCC	0,022209201	0,098867907	0	N.A.	0
LRRC37B	0,022191268	2,1722E-05	0	N.A.	0
ARMC8	0,02216878	0,08973756	0	N.A.	0
ABCG8	0,022126011	0,116464762	0	N.A.	0
A1BG-AS1	0,022124171	0,016859792	0	N.A.	0
RP11-266J6.2	0,022123347	0,363819122	0	N.A.	0
PPP1R14A	0,022103627	-0,174071687	0	N.A.	0
SELM	0,022099079	0,092591378	0	N.A.	0
ID1	0,022083607	0,365187487	0	N.A.	0
DNAAF3	0,022083217	0,02343854	0	N.A.	0
ASCL5	0,022056511	0,08550543	0	N.A.	0
PCNXL2	0,022024389	-0,163892682	0	N.A.	0
OAZ3	0,02201324	0,361421909	0	N.A.	0
WNT6	0,021951567	-0,101294844	0	N.A.	0
SLX1A	0,021936254	-0,010969473	0	N.A.	0
DPH6-AS1	0,021900992	-0,076169819	0	N.A.	0
HACE1	0,021819313	0,011093338	0	N.A.	0
KRTAP5-5	0,021792315	0,172998656	0	N.A.	0
ZNF718	0,021787847	0,109322445	0	N.A.	0
CCDC148	0,02178318	0,041437294	0	N.A.	0

ZBTB39	0,021772884	0,003497712	0	N.A.	0
LDHA	0,021693637	0,087391507	0	N.A.	0
APBB3	0,021681185	0,079928488	0	N.A.	0
BRPF1	0,021667011	0,058411344	0	N.A.	0
GABBR1	0,021659566	-0,051145806	0	N.A.	0
ANKRD40	0,02165655	0,07880301	0	N.A.	0
ZNF205	0,021642858	0,155856367	0	N.A.	0
EPB41L4A-AS	0,021633877	-0,030628015	0	N.A.	0
TRAPPC9	0,021554013	0,057248374	0	N.A.	0
CD226	0,02152544	0,043246612	0	N.A.	0
SMARCA1	0,021514906	-0,067232661	0	N.A.	0
CTC-260F20.:	0,021511425	0,145281785	0	N.A.	0
RP11-182J23	0,021487213	0,307363391	0	N.A.	0
ZNF703	0,021427276	0,254267299	0	N.A.	0
NHLH2	0,021386979	-0,014083432	0	N.A.	0
ANKRD42	0,021385564	0,088059869	0	N.A.	0
DLGAP1-AS1	0,021362764	-0,035606163	0	N.A.	0
SIDT1-AS1	0,021360537	0,146960934	0	N.A.	0
GLI4	0,021358916	-0,013653594	0	N.A.	0
LMO4	0,021350912	0,253160004	0	N.A.	0
FCGR2A	0,021318658	-0,292500016	0	N.A.	0
NADK	0,021305907	0,32091539	0	N.A.	0
AFP	0,021295323	0,022881801	0	N.A.	0
EDNRA	0,02126684	-0,029020141	0	N.A.	0
IGLC3	0,021261093	-0,263295013	0	N.A.	0
RUSC1-AS1	0,021223908	0,188563311	0	N.A.	0
RP11-676J12	0,021143993		0	N.A.	0
CEP170	0,021095327	0,248497034	0	N.A.	0
C5orf52	0,021072378	-0,216749821	0	N.A.	0
NCR3	0,021048139	-0,068626731	0	N.A.	0
RP1-199J3.7	0,021032366	0,180181168	0	N.A.	0
RP11-1105G2	0,021023988	0,031304688	0	N.A.	0
GNG12-AS1	0,020976905	0,097322008	0	N.A.	0

HS2ST1	0,020930241	0,35263999	0	N.A.	0
RP11-255M2	0,020925176	0,04951962	0	N.A.	0
TMEM41A	0,02092473	0,317539865	0	N.A.	0
RP11-792A8.	0,02088856	0,148897646	0	N.A.	0
CCND2-AS1	0,020882527	0,043216492	0	N.A.	0
RP11-88I18.2	0,020829637	-0,177079386	0	N.A.	0
TDRD6	0,020808824	0,09696501	0	N.A.	0
FOXF1	0,020747649	-0,057810304	0	N.A.	0
CTC-425F1.4	0,020739638	0,101167804	0	N.A.	0
FAM53A	0,020734643	0,016185478	0	N.A.	0
SLC7A2	0,020698272	0,203486814	0	N.A.	0
GOLGA8N	0,020654627	-0,013438024	0	N.A.	0
ZHX1	0,020621575	0,27843924	0	N.A.	0
HIAT1	0,020611243	0,075909952	0	N.A.	0
IRX5	0,020587531	-0,086800757	0	N.A.	0
TBX1	0,020586485	-0,130979215	0	N.A.	0
ZNF597	0,020584514	0,113279992	0	N.A.	0
RP11-359K18	0,020574955	0,258117171	0	N.A.	0
CDC42EP3	0,020528386	-0,126155092	0	N.A.	0
KB-1410C5.5	0,020481195		0	N.A.	0
ZNF660	0,020457922		0	N.A.	0
SHD	0,020445808	0,224296311	0	N.A.	0
MAP1B	0,020406632	0,391411038	0	N.A.	0
SPDYE3	0,020383473	-0,108326879	0	N.A.	0
PSMB8-AS1	0,020373695	-0,204914825	0	N.A.	0
VWCE	0,020346848	0,051018109	0	N.A.	0
RP11-514D23	0,020346027		0	N.A.	0
RP11-448A19	0,020343149	0,174098535	0	N.A.	0
RNASE6	0,020341993	-0,45622833	0	N.A.	0
SELE	0,020328942		0	N.A.	0
CFI	0,020328612	-0,294400065	0	N.A.	0
CAPZA2	0,020275895	-0,201896477	0	N.A.	0
TMEM184C	0,020252266	-0,076897422	0	N.A.	0

RP11-526A4.	0,020220266	0,016801615	0	N.A.	0
ARMCX2	0,0202154	0,174912714	0	N.A.	0
SECISBP2	0,020204314	0,26605116	0	N.A.	0
RP11-188P17	0,020198174	-0,0276325	0	N.A.	0
C1QTNF1	0,020184202	-0,143760942	0	N.A.	0
RP3-402G11.	0,020166191		0	N.A.	0
LCP1	0,020158597	-0,218010676	0	N.A.	0
DCUN1D4	0,020114655	-0,263838795	0	N.A.	0
MGC16275	0,020113502		0	N.A.	0
RP13-270P17	0,020085438	0,075615488	0	N.A.	0
SEPT12	0,020019455		0	N.A.	0
RP11-388C12	0,020014641	-0,170164771	0	N.A.	0
SPDEF	0,020014265	0,119362654	0	N.A.	0
RP11-407N17	0,020000103	0,26841577	0	N.A.	0
ZNF619	0,019992842	0,160038964	0	N.A.	0
CHRD	0,019988507	-0,233312825	0	N.A.	0
KLKB1	0,019957573	-0,058210917	0	N.A.	0
CTD-2283N19	0,019942055	-0,13034914	0	N.A.	0
AC073257.2	0,019894819		0	N.A.	0
RP11-27M24	0,019882366	-0,073668986	0	N.A.	0
CTD-2015G9.	0,019852423		0	N.A.	0
LSS	0,019811461	0,369780551	0	N.A.	0
TRIM39	0,019766984	-0,25472267	0	N.A.	0
FBN1	0,019758237	-0,065190364	0	N.A.	0
PCDH11Y	0,019711706	0,000597634	0	N.A.	0
LINC00240	0,019688535	0,022219601	0	N.A.	0
MBLAC1	0,019658237	-0,046040182	0	N.A.	0
ZNF787	0,019657692	0,139167741	0	N.A.	0
RP11-134K13	0,01965008		0	N.A.	0
ZYG11B	0,019635115	0,276352235	0	N.A.	0
PCOLCE2	0,019609363	0,236737887	0	N.A.	0
ADSSL1	0,019595298	-0,080977787	0	N.A.	0
KCNE2	0,019559681		0	N.A.	0

IGHV3-73	0,019535924		0	N.A.	0
CTC-329D1.2	0,019458307	0,121945616	0	N.A.	0
NFIX	0,019445259	0,160667427	0	N.A.	0
ENTPD6	0,019438881	0,24179159	0	N.A.	0
CHST12	0,019329211	-0,067934106	0	N.A.	0
NLRP3	0,01930939	-0,193252775	0	N.A.	0
CNNM2	0,019307372	0,113703878	0	N.A.	0
ZNF837	0,0192572	0,041651646	0	N.A.	0
MOB1A	0,019250373	-0,156713137	0	N.A.	0
PDGFC	0,019237299	-0,171787657	0	N.A.	0
FOXG1-AS1	0,019237282		0	N.A.	0
SPG7	0,019235258	0,453399053	0	N.A.	0
FN1	0,019211367	-0,232173241	0	N.A.	0
PPP4R4	0,019207014		0	N.A.	0
LINGO3	0,019178518		0	N.A.	0
PVRL3-AS1	0,019172957	-0,09730381	0	N.A.	0
ZNF774	0,019162824	0,041515945	0	N.A.	0
RP11-407N17	0,019155356	0,233649812	0	N.A.	0
GIGYF2	0,019150533	0,065500072	0	N.A.	0
FBXO38	0,019120168	0,196698942	0	N.A.	0
UBASH3A	0,019109794	-0,337384868	0	N.A.	0
ALDH1A2	0,019088104	-0,145935694	0	N.A.	0
TTC16	0,019058863	0,075359264	0	N.A.	0
FAM122A	0,019043724	-0,186713595	0	N.A.	0
SLC16A13	0,019042311	0,180446205	0	N.A.	0
CTD-2135D7.	0,018949981	-0,090321491	0	N.A.	0
SIX3	0,01893859	-0,051117089	0	N.A.	0
CPT1C	0,018928853	-0,175489915	0	N.A.	0
BASP1	0,018911937	-0,027610065	0	N.A.	0
RP5-1159O4.	0,018879396	0,094905337	0	N.A.	0
RP11-890B15	0,018861741	0,118317783	0	N.A.	0
JADE3	0,018806231	-0,089767606	0	N.A.	0
TBX20	0,018794121	0,135434664	0	N.A.	0

GXYLT1	0,018733874	0,093415348	0	N.A.	0
NLRP1	0,018732406	-0,023471512	0	N.A.	0
CBS	0,018727503		0	N.A.	0
COTL1	0,018719165	-0,413192902	0	N.A.	0
SLC29A4	0,018714807	-0,017779896	0	N.A.	0
HOXB2	0,018630629	0,138138447	0	N.A.	0
ST5	0,018615554	0,214006616	0	N.A.	0
XXbac-BPGBI	0,018614635		0	N.A.	0
RP1-43E13.2	0,018598432	0,064127639	0	N.A.	0
DNAH7	0,018596074	0,016586672	0	N.A.	0
PPP1R27	0,018582215	-0,030309957	0	N.A.	0
HEXA	0,018556738	-0,061008486	0	N.A.	0
PRDM12	0,018546331	0,03158512	0	N.A.	0
RP11-321N4.	0,018541732	0,000965671	0	N.A.	0
KLHL11	0,018486501	0,218176424	0	N.A.	0
DDX39B	0,018445696	0,227782719	0	N.A.	0
PLA2G3	0,018445325	0,0964197	0	N.A.	0
CDH11	0,018433153	0,120286511	0	N.A.	0
RP11-524H1	0,018406735	0,301985414	0	N.A.	0
RP11-707M3	0,018396445		0	N.A.	0
NHSL1	0,01838286	0,115739747	0	N.A.	0
ARSJ	0,018356718	0,105661516	0	N.A.	0
GATSL3	0,018269665	0,005455703	0	N.A.	0
ZNF777	0,018236021	0,028864563	0	N.A.	0
FBXL12	0,018230009	0,241371332	0	N.A.	0
CTXN2	0,01822207		0	N.A.	0
RP5-1061H2	0,018199973		0	N.A.	0
CTD-2026K1	0,018159127	-0,087502804	0	N.A.	0
PRKAR1A	0,018126948	0,006660487	0	N.A.	0
ZNF347	0,018126052	-0,184269838	0	N.A.	0
AC007383.3	0,018123026	-0,016743779	0	N.A.	0
HEATR4	0,018111434	-0,02753249	0	N.A.	0
RP11-382J12	0,018098142	-0,060701065	0	N.A.	0

TCF25	0,018083622	-0,137066749	0	N.A.	0
MAB21L3	0,018039576	0,241332414	0	N.A.	0
ABCA8	0,018012515		0	N.A.	0
LINC01524	0,01799716		0	N.A.	0
KRT78	0,017968558		0	N.A.	0
ZNF35	0,017932821	-0,356215324	0	N.A.	0
CASP9	0,017923033	0,079501327	0	N.A.	0
XG	0,017914041		0	N.A.	0
ST7-AS1	0,017892507	-0,13032166	0	N.A.	0
RP11-274B18	0,017883051		0	N.A.	0
HDAC11-AS1	0,017869994	0,083690062	0	N.A.	0
ALOX12B	0,017845025	-0,06093526	0	N.A.	0
ZNF780A	0,017823982	-0,005054383	0	N.A.	0
LINC00412	0,017795405		0	N.A.	0
RNF40	0,017775346	0,244267789	0	N.A.	0
TRIM68	0,017747559	-0,235204362	0	N.A.	0
XXbac-BPG29	0,017743303	-0,047645422	0	N.A.	0
RP11-322E11	0,017723755		0	N.A.	0
CTRC	0,017710712	0,021916774	0	N.A.	0
RP11-390N6.	0,017702079	-0,342120796	0	N.A.	0
CD84	0,017694391	-0,022644435	0	N.A.	0
LPAR1	0,017602426	0,246951018	0	N.A.	0
TCP11	0,017598079		0	N.A.	0
TULP3	0,017537113	0,028250063	0	N.A.	0
AC068831.16	0,017491158		0	N.A.	0
NRCAM	0,017481981	0,022886645	0	N.A.	0
PCAT7	0,017469378	-0,125600239	0	N.A.	0
PAX2	0,01746073		0	N.A.	0
RP11-81K13.	0,017456322		0	N.A.	0
MEFV	0,01745421		0	N.A.	0
RP11-351I21	0,017439502		0	N.A.	0
CTD-2240J17	0,017406426		0	N.A.	0
IRF4	0,01734081	0,044112527	0	N.A.	0

STX11	0,017340201	0,326751815	0	N.A.	0
RP11-6J21.2	0,017315357	-0,023947029	0	N.A.	0
RP4-781K5.4	0,017311995	0,109799545	0	N.A.	0
RAB7B	0,017277932	0,058509672	0	N.A.	0
NIPAL4	0,017206477	0,017818375	0	N.A.	0
RUNX1T1	0,017192008	-0,04779246	0	N.A.	0
GRIN2B	0,01718398	0,044942427	0	N.A.	0
ANKRD52	0,017168698	0,147664085	0	N.A.	0
ERCC5	0,017167722	0,011987908	0	N.A.	0
CTD-2306A12	0,01715411		0	N.A.	0
CMB9-22P13	0,017152032	-0,004566147	0	N.A.	0
KLHDC7A	0,017123952	0,51982487	0	N.A.	0
PCYOX1L	0,017096616	-0,043745053	0	N.A.	0
PLCH2	0,017091911	0,208142486	0	N.A.	0
CCDC183-AS1	0,017056586	0,250451096	0	N.A.	0
LINC01347	0,017035854	-0,097864271	0	N.A.	0
UXT-AS1	0,016959391	-0,053006857	0	N.A.	0
FLJ22447	0,016947394	-0,14073666	0	N.A.	0
ZNF598	0,016884574	0,047768658	0	N.A.	0
PLA2G6	0,016870486	0,208633324	0	N.A.	0
RP11-760H22	0,016867264	-0,171884852	0	N.A.	0
LRRC17	0,016855946	-0,310048891	0	N.A.	0
LRSAM1	0,016824577	0,033493952	0	N.A.	0
IGLON5	0,016798917		0	N.A.	0
NAAA	0,016745359	0,249723808	0	N.A.	0
CTD-2308L22	0,016694698	-0,247166968	0	N.A.	0
RP5-1148A21	0,016692384	0,158534337	0	N.A.	0
LHB	0,016692173	0,293291584	0	N.A.	0
KLHL17	0,016677217	0,286346812	0	N.A.	0
RP11-458F8.4	0,016626534	-0,05289435	0	N.A.	0
FBLN7	0,016609166	-0,001657104	0	N.A.	0
BCDIN3D-AS1	0,01660713	0,038124893	0	N.A.	0
ENG	0,016595365	-0,038688448	0	N.A.	0

DOK2	0,016553807	-0,014140406	0	N.A.	0
IGIP	0,016551982	0,153451135	0	N.A.	0
ZNF749	0,016537204	0,077310118	0	N.A.	0
CNTD1	0,016523973	-0,069817109	0	N.A.	0
DNAJC6	0,01649824	-0,083282194	0	N.A.	0
RP11-76I23.7	0,01648112		0	N.A.	0
TMIGD2	0,0164288		0	N.A.	0
RP11-305L7.1	0,016395058	0,080731941	0	N.A.	0
ATE1-AS1	0,016390371	0,10369958	0	N.A.	0
RP11-573D15	0,016351377	0,023781577	0	N.A.	0
RP11-395G23	0,016340069	0,263816808	0	N.A.	0
VCAN	0,016338051	-0,11739687	0	N.A.	0
HIST1H2BH	0,016315475	0,081917865	0	N.A.	0
KCNF1	0,016281025		0	N.A.	0
HNRNPCL2	0,016253828		0	N.A.	0
RP4-697K14.	0,01620959	0,030502774	0	N.A.	0
RP11-53B2.3	0,016103623		0	N.A.	0
PRKCE	0,0161	0,252813244	0	N.A.	0
HDAC4	0,016095498	0,044349778	0	N.A.	0
POU4F1	0,01606383	0,157779873	0	N.A.	0
ZSWIM3	0,016047517	0,094694753	0	N.A.	0
CCSAP	0,016036309	-0,06229572	0	N.A.	0
RP11-111K18	0,016034622		0	N.A.	0
RP4-591N18.	0,016033361		0	N.A.	0
RP5-857K21.	0,016011932	0,094111568	0	N.A.	0
LTC4S	0,015999684	0,068103326	0	N.A.	0
RP11-17E13.	0,015991972	0,111944823	0	N.A.	0
RP11-286E11	0,015976852	-0,206861277	0	N.A.	0
IFT20	0,015972184	0,153457272	0	N.A.	0
HIST1H2AB	0,015901328	-0,193494465	0	N.A.	0
CECR6	0,015896253	0,010504664	0	N.A.	0
SAMSN1	0,015861823	-0,294122924	0	N.A.	0
CTD-2561B21	0,01586037	0,110280984	0	N.A.	0

MALRD1	0,015852165	0,252993871	0	N.A.	0
BOK	0,015819946	0,078514527	0	N.A.	0
CHRFAM7A	0,015787441	0,179715805	0	N.A.	0
TUBA8	0,015776162	-0,032479978	0	N.A.	0
IGLV3-21	0,015769631	-0,167006878	0	N.A.	0
ATP1B2	0,015766885	0,005977146	0	N.A.	0
HCLS1	0,015753427	-0,038759253	0	N.A.	0
ZC2HC1A	0,015736471	0,061242284	0	N.A.	0
RP11-461L13	0,015727726		0	N.A.	0
GREB1	0,015726336		0	N.A.	0
ZC3H4	0,015703004	0,046034922	0	N.A.	0
MTMR6	0,015687286	0,082944078	0	N.A.	0
RP11-571I18	0,015686807	0,04833765	0	N.A.	0
HLA-DPA1	0,015616488	-0,239922844	0	N.A.	0
PCDH17	0,015583011	-0,045576545	0	N.A.	0
COLGALT2	0,015569139	0,055162417	0	N.A.	0
TRIM9	0,015541252	-0,032564436	0	N.A.	0
RP11-59D5--	0,015514172	0,251570446	0	N.A.	0
RNF165	0,015510375	-0,075142175	0	N.A.	0
RP13-131K19	0,01548839		0	N.A.	0
PIK3CG	0,015469518		0	N.A.	0
GJA4	0,015457547	-0,046864356	0	N.A.	0
IGLC2	0,015457342	0,018431234	0	N.A.	0
ZNF362	0,015423663	-0,028254825	0	N.A.	0
GABRB1	0,015414271	0,262579425	0	N.A.	0
CXCR3	0,015407354	-0,196274474	0	N.A.	0
HIF3A	0,015368672	0,062035367	0	N.A.	0
BTBD2	0,015363989	0,212517609	0	N.A.	0
SHISA8	0,015355907		0	N.A.	0
SOX4	0,015329291	0,293999358	0	N.A.	0
TAC3	0,015328633	0,192367556	0	N.A.	0
SNAPC4	0,015328039	-0,271739982	0	N.A.	0
RP11-90K6.1	0,015295279	0,098584884	0	N.A.	0

RP11-637A17	0,015294354	0,130680211	0	N.A.	0
FAM184B	0,015278623	0,193574889	0	N.A.	0
RP11-321A17	0,015244357	0,301280996	0	N.A.	0
CLCN1	0,015235354	-0,100511134	0	N.A.	0
PTGS1	0,015232062	-0,275013289	0	N.A.	0
ZNF845	0,015198252	-0,123209441	0	N.A.	0
PLOD3	0,015184394	0,400246136	0	N.A.	0
RP11-492E3.	0,015141916	-0,023397754	0	N.A.	0
AGTPBP1	0,015121331	0,098985779	0	N.A.	0
GBGT1	0,015035678	-0,037655801	0	N.A.	0
CITF22-92A6	0,014939917	-0,034314129	0	N.A.	0
ZNF765	0,014912955	0,227323316	0	N.A.	0
CYP2R1	0,014905296	0,168401539	0	N.A.	0
IL31RA	0,014892253		0	N.A.	0
RP11-15J22.8	0,014889936	-0,135476377	0	N.A.	0
RRP12	0,014877034	-0,016558899	0	N.A.	0
UBXN2A	0,014856344	-0,027582775	0	N.A.	0
AC010524.2	0,014839306		0	N.A.	0
PDE10A	0,014829912	0,06563373	0	N.A.	0
SLITRK6	0,014825454	0,02057934	0	N.A.	0
TSIX	0,014822073	0,010235304	0	N.A.	0
CLCN4	0,014740351	-0,039510793	0	N.A.	0
PLCD1	0,014701881	-0,116394452	0	N.A.	0
AF186192.1	0,014665406		0	N.A.	0
KB-1183D5.1	0,014635573	0,099327318	0	N.A.	0
DLGAP4-AS1	0,014633696	-0,083702087	0	N.A.	0
ZNF782	0,014621919	-0,177627261	0	N.A.	0
LINC01183	0,014616003	0,063289713	0	N.A.	0
AC007405.6	0,014583021	-0,016545825	0	N.A.	0
RP5-1074L1.4	0,014577703	0,164427717	0	N.A.	0
RP11-98D18.	0,014545505		0	N.A.	0
PODNL1	0,014542172	-0,058611856	0	N.A.	0
RP11-309M2	0,014533693		0	N.A.	0

PHKG1	0,014527077	-0,085501079	0	N.A.	0
RP11-158M2	0,014505649		0	N.A.	0
C1orf204	0,0144873	0,127795785	0	N.A.	0
AC078883.4	0,014478583	-0,029043239	0	N.A.	0
METTL20	0,014438788	0,214434305	0	N.A.	0
RP11-343L5.2	0,014431709		0	N.A.	0
BAD	0,0143881	0,317016731	0	N.A.	0
STOML1	0,014347189	0,306637255	0	N.A.	0
KIAA1244	0,014308222	0,242690038	0	N.A.	0
SYNGR1	0,014289548	-0,164102079	0	N.A.	0
RP11-171I2.5	0,014288176	0,138724784	0	N.A.	0
RP11-689K5.	0,014281166	-0,011135635	0	N.A.	0
RP11-725P16	0,014279585	-0,132146375	0	N.A.	0
RP11-305L7.1	0,014245455		0	N.A.	0
SLCO1B1	0,014238986	-0,075643647	0	N.A.	0
OIT3	0,014169728	0,101741538	0	N.A.	0
PCDH10	0,014132156		0	N.A.	0
SPATA13-AS1	0,014132156		0	N.A.	0
ENTPD1	0,014131207	-0,279897627	0	N.A.	0
RP11-51J9.5	0,014130029	-0,130600901	0	N.A.	0
ZNF284	0,014110474	0,259073012	0	N.A.	0
RP13-616I3.1	0,01408304	0,184691455	0	N.A.	0
TOPORS	0,014072613	-0,009772795	0	N.A.	0
CREB3L3	0,014062801	0,030797786	0	N.A.	0
SP8	0,014037666	-0,151996142	0	N.A.	0
EDRF1	0,014032273	0,12759243	0	N.A.	0
POLB	0,014018589	0,074447448	0	N.A.	0
TGFB1I1	0,013966364	0,02349361	0	N.A.	0
DCTN5	0,013929671	0,199480242	0	N.A.	0
UBE2G1	0,013895772	0,180358149	0	N.A.	0
AC009480.3	0,013867764		0	N.A.	0
S100B	0,013841046	0,260396865	0	N.A.	0
PITRM1	0,013791781	0,028620176	0	N.A.	0

NPTXR	0,01376941		0	N.A.	0
RP11-245J9.4	0,013757297		0	N.A.	0
KCNK9	0,013753013	0,241535926	0	N.A.	0
MATK	0,013738503	-0,093256732	0	N.A.	0
ZEB1	0,013713098	0,039704742	0	N.A.	0
CASC1	0,013694969	-0,098480882	0	N.A.	0
AC010745.1	0,013675569	0,204180681	0	N.A.	0
NMB	0,013660976	0,137317153	0	N.A.	0
AC007040.11	0,013568747	0,186817608	0	N.A.	0
SEPT14	0,013565918		0	N.A.	0
PPP1R16B	0,013517802	-0,338316193	0	N.A.	0
SLC7A8	0,013502587	0,008702714	0	N.A.	0
AP001372.2	0,013489016	-0,167142506	0	N.A.	0
ESRP2	0,013477591	0,214427376	0	N.A.	0
RP1-140A9.1	0,013426885	0,051650342	0	N.A.	0
IGHV3-53	0,01341184		0	N.A.	0
RP11-522B15	0,013406779	0,151291396	0	N.A.	0
IGLV1-47	0,013376586	-0,024260374	0	N.A.	0
SLC13A5	0,013349674	0,075192094	0	N.A.	0
ZNF540	0,013348174	-0,010528766	0	N.A.	0
ZFPM1	0,013309411	0,106039464	0	N.A.	0
RP11-284F21	0,013250643	0,017920904	0	N.A.	0
ARHGEF11	0,013249739	0,167295808	0	N.A.	0
RP11-580I16	0,013242409	-0,088447378	0	N.A.	0
UGDH-AS1	0,013240095	-0,158797989	0	N.A.	0
IGHV4-34	0,013162254	-0,077037333	0	N.A.	0
RP11-212P7.	0,013156498	0,16964377	0	N.A.	0
RP11-284F21	0,01314967	0,209723953	0	N.A.	0
RP11-353K11	0,013135093	0,040750523	0	N.A.	0
RP11-475O23	0,013130052	0,00111828	0	N.A.	0
RP11-138E2.	0,013048591		0	N.A.	0
KIZ	0,01298554	0,180154443	0	N.A.	0
AVPR1A	0,012878779		0	N.A.	0

KB-68A7.1	0,012870464		0	N.A.	0
AC079305.10	0,012838573	0,053001534	0	N.A.	0
BCAS3	0,012829918	0,01513724	0	N.A.	0
ERCC6L2	0,01282112	-0,128615191	0	N.A.	0
PHKG2	0,012791522	-0,228167371	0	N.A.	0
FAM199X	0,012786222	0,207870592	0	N.A.	0
FST	0,012780733	-0,104526445	0	N.A.	0
USP33	0,012778527	0,37184199	0	N.A.	0
HOXA10-AS	0,012768558	-0,034612227	0	N.A.	0
RP11-304L19	0,012710513	-0,083393705	0	N.A.	0
HOXD-AS2	0,012690561	0,203940161	0	N.A.	0
AP2B1	0,012681917	0,183189905	0	N.A.	0
LL21NC02-21	0,012670415	-0,331793594	0	N.A.	0
RP11-1055B8	0,012667512		0	N.A.	0
C9orf3	0,012649735	0,17494365	0	N.A.	0
SULT1E1	0,01262246	0,005243415	0	N.A.	0
AC004257.1	0,012601098		0	N.A.	0
CTD-3074O7	0,012596327	-0,21537207	0	N.A.	0
RP11-849H4.	0,012460248	-0,208876646	0	N.A.	0
DNASE1L2	0,012456807	0,171268238	0	N.A.	0
RP11-274B21	0,012455198	0,215590907	0	N.A.	0
CTD-3185P2.	0,012441875	0,066209146	0	N.A.	0
RP11-503N18	0,012407833	0,11725184	0	N.A.	0
SNX32	0,0123523	-0,168095269	0	N.A.	0
BIN3	0,012348305	0,048437149	0	N.A.	0
CTD-2302E22	0,012307791		0	N.A.	0
PHEX	0,01230205	-0,065761911	0	N.A.	0
APTR	0,012289274	-0,113293494	0	N.A.	0
NPDC1	0,012274867	0,172238698	0	N.A.	0
MGAT4C	0,0122697		0	N.A.	0
RP5-1050D4.	0,012261336	-0,1266003	0	N.A.	0
RP11-134G8.	0,012254513	0,030848325	0	N.A.	0
IGHG3	0,012254126	-0,092326291	0	N.A.	0

SNCA	0,012242964	-0,022041608	0	N.A.	0
RP11-486A14	0,012220932	0,113564193	0	N.A.	0
C21orf91	0,012208099	0,055474691	0	N.A.	0
TM4SF19-AS	0,012176847	0,027284596	0	N.A.	0
ZNF71	0,012165714	0,00611082	0	N.A.	0
TBC1D3D	0,012119885	0,169372117	0	N.A.	0
GALNTL6	0,01210239		0	N.A.	0
MYH4	0,012021104	-0,249782363	0	N.A.	0
SELL	0,011984851	-0,322179136	0	N.A.	0
AC005339.2	0,011954995	-0,20469643	0	N.A.	0
GCN1L1	0,011930239	0,127077569	0	N.A.	0
FSIP2-AS1	0,011916806	-0,113896976	0	N.A.	0
RP1-167O22.	0,011915138		0	N.A.	0
RP11-346C20	0,011895289	0,053748625	0	N.A.	0
XKR6	0,011846514	-0,08156105	0	N.A.	0
INSL6	0,01180588		0	N.A.	0
ZNF112	0,011776206	0,366354014	0	N.A.	0
DCUN1D2	0,011672526	-0,105465287	0	N.A.	0
SMARCD3	0,011659297		0	N.A.	0
POLR2M	0,011612536	-0,033623578	0	N.A.	0
SEPSECS	0,011606042	0,161570471	0	N.A.	0
ARHGAP31-A	0,011515465		0	N.A.	0
ADAM20P1	0,011509074	0,487261893	0	N.A.	0
HAR1A	0,01150701	-0,176540049	0	N.A.	0
KCNJ12	0,011450235		0	N.A.	0
ZNF793-AS1	0,011443069	-0,022717094	0	N.A.	0
AC092171.2	0,011402143	0,362548775	0	N.A.	0
GS1-279B7.2	0,01139815	0,083283446	0	N.A.	0
EPHB1	0,011387351	0,086953632	0	N.A.	0
AP000442.1	0,011319965	0,321760411	0	N.A.	0
TTC39A-AS1	0,011307737	-0,1201726	0	N.A.	0
HIST3H2BB	0,011280659	-0,004357548	0	N.A.	0
CEP104	0,011265108	0,118850368	0	N.A.	0

ZNF480	0,011261503	0,105281514	0	N.A.	0
ST3GAL5	0,011242133	-0,059303871	0	N.A.	0
RGS12	0,011157629	0,224056537	0	N.A.	0
SHISA5	0,011133096	0,184828793	0	N.A.	0
AC000111.6	0,011120775	-0,23682514	0	N.A.	0
PICK1	0,011082086	0,025691093	0	N.A.	0
SLAMF7	0,01108063	0,049348267	0	N.A.	0
WASH1	0,011060494	0,396932261	0	N.A.	0
AC005481.5	0,011059089	0,05600904	0	N.A.	0
UNC79	0,011014473	-0,220132295	0	N.A.	0
UGT1A1	0,011010143	0,065330048	0	N.A.	0
CGNL1	0,010979761	0,292578166	0	N.A.	0
CCDC154	0,010968793	0,11531601	0	N.A.	0
CLSTN3	0,010955933	0,11495415	0	N.A.	0
IL1RL1	0,01095541		0	N.A.	0
RB1	0,010890783	0,386007006	0	N.A.	0
ITLN2	0,010879782		0	N.A.	0
CH507-338C2	0,010798988	0,189290882	0	N.A.	0
MRAP2	0,010762006	-0,186203265	0	N.A.	0
RP11-96K19.	0,010746741	-0,05815157	0	N.A.	0
PCDHB11	0,01073712	0,07659815	0	N.A.	0
RP1-170O19.	0,010736781	0,019406168	0	N.A.	0
RP11-74J13.8	0,010686975	-0,171599889	0	N.A.	0
UNQ6494	0,010681638	0,002158595	0	N.A.	0
LINC00479	0,010652527	0,192650865	0	N.A.	0
RP11-431J24	0,010621061	-0,065317963	0	N.A.	0
KIF17	0,010617988		0	N.A.	0
PMEL	0,010591933	-0,285257133	0	N.A.	0
FBXW10	0,010591721	0,017910863	0	N.A.	0
HNF4A-AS1	0,010558575		0	N.A.	0
RP4-761J14.8	0,010540736	-0,303905067	0	N.A.	0
TBX19	0,010484145	0,114821256	0	N.A.	0
HIATL1	0,0104784	0,181151249	0	N.A.	0

KB-1208A12.	0,010427502	0,02969958	0	N.A.	0
AIRN	0,010413689	-0,03847024	0	N.A.	0
C1QA	0,010393478	0,059618895	0	N.A.	0
C19orf18	0,010393292	-0,059431967	0	N.A.	0
PAX5	0,010344091	-0,230112138	0	N.A.	0
MYO5C	0,010340658	0,284014835	0	N.A.	0
RP13-766D2(	0,010335309		0	N.A.	0
RP11-398C13	0,010330222	0,098620049	0	N.A.	0
PKD1L1	0,010260477	-0,286209907	0	N.A.	0
CYTH4	0,010250896	-0,006856285	0	N.A.	0
ZNF169	0,010160722	-0,121279676	0	N.A.	0
SRGAP2B	0,010155399	-0,004508314	0	N.A.	0
WDR82	0,010104691	0,004117868	0	N.A.	0
AC009120.10	0,010060099	0,207888509	0	N.A.	0
RP11-502I4.3	0,010029397	-0,010661658	0	N.A.	0
GGT2	0,010020214	0,271918044	0	N.A.	0
RP11-770G2.	0,010004946	0,211323737	0	N.A.	0
MEP1B	0,009995738	-0,112715575	0	N.A.	0
C9orf9	0,009967354	-0,175785605	0	N.A.	0
FGF22	0,009954124	0,008201033	0	N.A.	0
LINC01146	0,009918056	0,055701115	0	N.A.	0
RNGTT	0,009914108	0,097848788	0	N.A.	0
IGLV6-57	0,009891736		0	N.A.	0
ILK	0,009864885	0,028776254	0	N.A.	0
C9orf41	0,009852633	-0,003637598	0	N.A.	0
LINC01511	0,009849652	0,246224317	0	N.A.	0
DCP1A	0,009805261	0,21708499	0	N.A.	0
RP11-297D21	0,009758794	0,1678942	0	N.A.	0
ASAH1	0,009719663	0,283999523	0	N.A.	0
ASIC4	0,009705271	0,010416942	0	N.A.	0
PTPN6	0,009695425	0,109972971	0	N.A.	0
AP000254.8	0,009680688	0,286009167	0	N.A.	0
AC004878.2	0,009672294	0,195175317	0	N.A.	0

CTC-508F8.1	0,00965378	-0,361757006	0	N.A.	0
ZNF503	0,009645037	0,102937879	0	N.A.	0
TRHDE	0,009635338	0,028202894	0	N.A.	0
CHST7	0,009631173	0,167935084	0	N.A.	0
MIEF1	0,009596576	0,002886596	0	N.A.	0
MPPED2	0,009569591	-0,014686827	0	N.A.	0
ITGAM	0,009507061	-0,249805844	0	N.A.	0
IKZF3	0,009505926	-0,243005947	0	N.A.	0
RP11-137H2.	0,009496324	-0,158499496	0	N.A.	0
CWC25	0,009490952	0,101562095	0	N.A.	0
COLEC12	0,009481734	-0,000821791	0	N.A.	0
ARHGEF25	0,009463369		0	N.A.	0
RP11-287D1.	0,009430683	-0,093248938	0	N.A.	0
ADH4	0,009426894	0,153965648	0	N.A.	0
FBLN2	0,009375466	-0,258175263	0	N.A.	0
P2RY10	0,009342602	-0,238373313	0	N.A.	0
PILRA	0,00934057	0,205060088	0	N.A.	0
PTER	0,009327534	0,10233176	0	N.A.	0
DNAJC25-GN	0,009319921	-0,187276248	0	N.A.	0
ABCD4	0,009318275	0,19736139	0	N.A.	0
RP5-1042K1(	0,009234607	0,172425522	0	N.A.	0
KIR3DX1	0,009201362		0	N.A.	0
C6orf48	0,009189172	-0,252912459	0	N.A.	0
KRTAP5-10	0,009186473	0,165799013	0	N.A.	0
PLCXD2	0,009141488	0,020165779	0	N.A.	0
ATP10A	0,00910606	-0,190195551	0	N.A.	0
STAC3	0,009069993	-0,004403648	0	N.A.	0
MAGED2	0,009064102	-0,033336711	0	N.A.	0
ADGB	0,009045223	-0,269612078	0	N.A.	0
IGFBP7	0,009014504	-0,258883584	0	N.A.	0
RDH5	0,009006124	0,233608029	0	N.A.	0
C5orf45	0,008972964	-0,163895786	0	N.A.	0
CHI3L2	0,008968881	-0,173878502	0	N.A.	0

ABAT	0,008942404	0,072599412	0	N.A.	0
POU3F1	0,008930824		0	N.A.	0
RP11-454P21	0,008892895	-0,009577256	0	N.A.	0
TMCC1-AS1	0,008888866	0,107468745	0	N.A.	0
STX8	0,0088866	-0,091576248	0	N.A.	0
USP47	0,008872051	0,240057323	0	N.A.	0
RP11-455F5.!	0,008847232	-0,221307029	0	N.A.	0
RP11-692C24	0,008843929	-0,071888049	0	N.A.	0
GHR	0,008843444	-0,21381232	0	N.A.	0
CD101	0,008785539		0	N.A.	0
SLC4A9	0,008785237	0,138580493	0	N.A.	0
RP11-676J12	0,008775095		0	N.A.	0
SENP3	0,008755606	-0,10540165	0	N.A.	0
PRH1	0,008741564	-0,021791371	0	N.A.	0
PROSER3	0,008686192	0,029857066	0	N.A.	0
SOX18	0,00866678	0,075400901	0	N.A.	0
KIT	0,008655018	-0,000464747	0	N.A.	0
REPS1	0,008651054	0,165385235	0	N.A.	0
LINC00954	0,008595725	-0,098601	0	N.A.	0
RP11-265E18	0,008577713	0,440500658	0	N.A.	0
NAPA-AS1	0,008574469	0,043255277	0	N.A.	0
CA5B	0,008536037	-0,211235759	0	N.A.	0
ZNF486	0,008527535	0,050434886	0	N.A.	0
PDE6D	0,008525274	-0,051165677	0	N.A.	0
ACVR1B	0,008472775	0,115713929	0	N.A.	0
TWF2	0,00845973	0,153079867	0	N.A.	0
CHN2	0,008451217	-0,190475252	0	N.A.	0
RP11-76P2.4	0,008416106		0	N.A.	0
RP11-388M2	0,008407288	0,127527232	0	N.A.	0
C11orf72	0,008341587		0	N.A.	0
ZSWIM5	0,008331478	0,037122877	0	N.A.	0
NDUFV2-AS1	0,008278631	0,08991084	0	N.A.	0
RP5-1091N2.	0,008274908		0	N.A.	0

C11orf70	0,008250968	-0,281734626	0	N.A.	0
ZNF843	0,008231479	-0,184548716	0	N.A.	0
BGLAP	0,008201898	0,040021711	0	N.A.	0
RP11-1260E1	0,008180288	-0,345025285	0	N.A.	0
CACNA2D4	0,008135818	0,195163289	0	N.A.	0
USP11	0,008135538	0,043418952	0	N.A.	0
NAPA	0,008082317	0,241447716	0	N.A.	0
ACOX3	0,008077469	0,001289673	0	N.A.	0
RP11-265O1	0,008076252		0	N.A.	0
SDF4	0,008070054	0,448886056	0	N.A.	0
RP11-262A1	0,008060967		0	N.A.	0
RP11-613D1	0,008060125	-0,215829369	0	N.A.	0
NOV	0,008041282	0,030806489	0	N.A.	0
ZNF425	0,008039447	0,005376476	0	N.A.	0
LINC00535	0,00803724	-0,044460358	0	N.A.	0
AF127936.3	0,008022262	-0,072224274	0	N.A.	0
PPARGC1A	0,008017293	0,186535657	0	N.A.	0
ATF2	0,008014328	-0,08369258	0	N.A.	0
FP325317.1	0,007954884		0	N.A.	0
ZNF20	0,007915973	-0,288785629	0	N.A.	0
SFRP4	0,007911607	0,064395603	0	N.A.	0
IDUA	0,007908183	0,252029074	0	N.A.	0
KRTAP5-AS1	0,007879769	0,161093979	0	N.A.	0
PGC	0,007861623	-0,299984731	0	N.A.	0
SNX10	0,007851018	-0,377532316	0	N.A.	0
TMED8	0,007847178	-0,227579931	0	N.A.	0
ZNF737	0,00783796	0,162514107	0	N.A.	0
NKTR	0,007768903	0,261652463	0	N.A.	0
C21orf62	0,0077522	-0,185610101	0	N.A.	0
IL17RD	0,007737884	0,053349881	0	N.A.	0
COL5A1	0,00772114	0,028378084	0	N.A.	0
FBXO25	0,007676862	-0,294608394	0	N.A.	0
PGF	0,007667724	0,065058571	0	N.A.	0

MFAP4	0,007665348	0,230948298	0	N.A.	0
WDR89	0,007614371	0,072348983	0	N.A.	0
DCAF8	0,007613293	0,090363341	0	N.A.	0
KRTAP5-8	0,007567259		0	N.A.	0
LINC00299	0,00755043	-0,076753258	0	N.A.	0
MORC2-AS1	0,007525007	-0,205543702	0	N.A.	0
RP4-781K5.2	0,007478426	0,022607379	0	N.A.	0
NOL4	0,007460003	-0,080980724	0	N.A.	0
ZNF682	0,007449529	-0,204164911	0	N.A.	0
HMGXB3	0,007436494	0,087480341	0	N.A.	0
ZNF713	0,007392744	-0,320397194	0	N.A.	0
ZCWPW2	0,007333599	-0,163055471	0	N.A.	0
ALG1L	0,007320969	-0,062434136	0	N.A.	0
ETNK2	0,007310789	0,270026257	0	N.A.	0
C12orf76	0,007303279	-0,015034689	0	N.A.	0
APOBEC3D	0,007301355	-0,151380459	0	N.A.	0
LINC00266-1	0,007298308		0	N.A.	0
IFNG	0,007272554		0	N.A.	0
TPD52	0,007248916	0,336238485	0	N.A.	0
RP4-734G22.	0,007226121	-0,118158327	0	N.A.	0
USP40	0,007219481	0,193539969	0	N.A.	0
PTCHD2	0,007187239	0,047755693	0	N.A.	0
QSER1	0,007151171	0,056193826	0	N.A.	0
GABRB2	0,007150215	-0,119583812	0	N.A.	0
AARSD1	0,007148008	0,067364514	0	N.A.	0
UCN2	0,007136976	0,333407989	0	N.A.	0
ABHD8	0,007130244	0,057359224	0	N.A.	0
DFNB31	0,007129726	0,141754576	0	N.A.	0
SMNDC1	0,007103774	0,071854995	0	N.A.	0
RP11-346D14	0,007075319	0,01159259	0	N.A.	0
EP400	0,006991811	0,262233674	0	N.A.	0
RP11-180M1	0,006977107	-0,164110214	0	N.A.	0
RP5-933K21.	0,006960014		0	N.A.	0

AC018737.3	0,006952013	0,024083864	0	N.A.	0
LDB2	0,006928066		0	N.A.	0
ARL15	0,006847575	0,197464252	0	N.A.	0
RP1-125I3.2	0,006829888		0	N.A.	0
CDH15	0,006810918	-0,132231705	0	N.A.	0
RP11-522M2	0,006799199	0,227663316	0	N.A.	0
RP4-583P15.	0,006766271	0,023235001	0	N.A.	0
RP11-1143G	0,006747704	0,076614116	0	N.A.	0
RP11-2B6.2	0,006734405	-0,020686283	0	N.A.	0
SPDYC	0,006734134	0,196513167	0	N.A.	0
RP5-965G21.	0,00671285	0,425851595	0	N.A.	0
RP11-365P13	0,00671161	0,187909291	0	N.A.	0
C17orf50	0,006705681		0	N.A.	0
CTD-2047H1	0,006688275		0	N.A.	0
KLHL31	0,006672294	-0,009243008	0	N.A.	0
CD40LG	0,006649492		0	N.A.	0
CTC-529I10.1	0,006645456	0,283399912	0	N.A.	0
RBM38	0,00664339	0,133521548	0	N.A.	0
RP11-131L23	0,0066331	0,010107048	0	N.A.	0
TNFRSF13C	0,006613061	-0,103448968	0	N.A.	0
CEP83-AS1	0,006605863	0,005495921	0	N.A.	0
RP11-282O1	0,006580274		0	N.A.	0
LAPTM5	0,006556087	-0,408581301	0	N.A.	0
LINC00886	0,006538706	0,10506728	0	N.A.	0
FAM227B	0,006537426	0,117367782	0	N.A.	0
DDX59	0,006510667	0,038445631	0	N.A.	0
ZMYM4	0,006505834	0,111166698	0	N.A.	0
UNC93A	0,006434155	-0,076022853	0	N.A.	0
CTC-518P12.	0,006432579	0,033951211	0	N.A.	0
RP11-108M9	0,006426791	-0,157105555	0	N.A.	0
EML5	0,006391619	0,168112135	0	N.A.	0
SYNE1	0,006367049	-0,038330236	0	N.A.	0
FAM156A	0,006346908	0,137582472	0	N.A.	0

HOXB-AS4	0,006338125		0	N.A.	0
PGBD2	0,006294314	-0,147773125	0	N.A.	0
RP11-57H12.	0,006292054	-0,007230728	0	N.A.	0
UTRN	0,00625038	0,320925841	0	N.A.	0
LSP1	0,006243874	-0,249820617	0	N.A.	0
NEU3	0,006229041	-0,172537274	0	N.A.	0
CEP44	0,006226459	-0,126515085	0	N.A.	0
BBS4	0,006209236	0,101435366	0	N.A.	0
FBXO30	0,006187835	-0,180773948	0	N.A.	0
AP5S1	0,006163547	0,205242417	0	N.A.	0
CYP26A1	0,006144631		0	N.A.	0
RP11-25H12.	0,006133145	-0,170618441	0	N.A.	0
TMEM59L	0,00612096		0	N.A.	0
PECAM1	0,006119541	-0,168147867	0	N.A.	0
ZBTB11-AS1	0,006063857	0,302861399	0	N.A.	0
PRKD1	0,006061884		0	N.A.	0
RELL2	0,006042674	-0,113751026	0	N.A.	0
POLG	0,006016644	0,251869177	0	N.A.	0
RP11-528A4.	0,00599948	0,176566722	0	N.A.	0
RP11-195F19	0,005994624	-0,153555235	0	N.A.	0
ZBED5	0,005961686	0,069194177	0	N.A.	0
SS18L1	0,005933872	0,022735128	0	N.A.	0
IGLV3-1	0,005915702	-0,151516345	0	N.A.	0
TUBB4A	0,005892298	-0,068856584	0	N.A.	0
TMEM253	0,005859817	0,262941072	0	N.A.	0
AC003956.1	0,005843978	0,182120548	0	N.A.	0
WDR60	0,005841765	0,504408436	0	N.A.	0
SLC35G5	0,00583793	0,046906907	0	N.A.	0
USF1	0,005831608	-0,023695807	0	N.A.	0
BOLL	0,005826463		0	N.A.	0
AC011513.4	0,005820343	0,258528713	0	N.A.	0
ZBTB45	0,005809598	-0,095154164	0	N.A.	0
RP11-1018N1	0,005800222		0	N.A.	0

RP11-295M3	0,005778527	0,085646688	0	N.A.	0
RP11-388M2	0,005770262	0,068441322	0	N.A.	0
MPST	0,005769023	0,042989139	0	N.A.	0
FCRLA	0,00576534	-0,344267135	0	N.A.	0
AC092835.2	0,005735988		0	N.A.	0
LCMT1-AS1	0,00572145		0	N.A.	0
LINC01558	0,005698264	0,144509535	0	N.A.	0
SLC28A3	0,005686497	0,140385789	0	N.A.	0
ZNF625	0,005624978		0	N.A.	0
EXD2	0,0056228	0,179866634	0	N.A.	0
TRIM52	0,005620914	0,087129879	0	N.A.	0
BBS2	0,005618693	-0,027101231	0	N.A.	0
LINC01012	0,005590537	-0,072749064	0	N.A.	0
FBXO24	0,00557733	0,328314763	0	N.A.	0
CXCR5	0,005555634	0,031310603	0	N.A.	0
RP11-213H15	0,005544963		0	N.A.	0
NRF1	0,005465061	-0,065875802	0	N.A.	0
MS4A6A	0,005455721	-0,235904769	0	N.A.	0
RP11-575L7.8	0,005392077		0	N.A.	0
HLA-DPB1	0,00538956	-0,289030382	0	N.A.	0
FRS2	0,005380075	0,285383945	0	N.A.	0
ZNF549	0,005354949	-0,338316193	0	N.A.	0
CTD-3126B10	0,005349859		0	N.A.	0
LPXN	0,005339709	-0,328901827	0	N.A.	0
SPDYE1	0,005302568		0	N.A.	0
CD163	0,005263568	0,036040585	0	N.A.	0
MORN4	0,005247813	0,333147481	0	N.A.	0
TCP10L2	0,005203802	-0,135219761	0	N.A.	0
RP11-1391J7	0,005200983	0,021205342	0	N.A.	0
CCDC126	0,00519505	-0,03214097	0	N.A.	0
GAS1	0,005171267	-0,086200802	0	N.A.	0
RP11-373D23	0,005170477	0,211707031	0	N.A.	0
EPM2AIP1	0,005145873	-0,128841344	0	N.A.	0

RP11-314A20	0,005122658	-0,306535125	0	N.A.	0
CHST13	0,005072623	-0,037703948	0	N.A.	0
TAP2	0,005061533	0,227367085	0	N.A.	0
RP11-213G2.	0,005054746	0,171701687	0	N.A.	0
KANK3	0,00502491		0	N.A.	0
SPRY3	0,00501511	-0,055489061	0	N.A.	0
RP11-1149O2	0,004987994	-0,128208862	0	N.A.	0
SPATA9	0,004971959		0	N.A.	0
PHKA1	0,004968061	0,023073625	0	N.A.	0
LA16c-390H2	0,004960868	-0,133325802	0	N.A.	0
GIGYF1	0,004917623	0,150902374	0	N.A.	0
TBC1D10C	0,004831121	-0,223461233	0	N.A.	0
RP11-53I6.3	0,004739574	0,360961433	0	N.A.	0
TMEM99	0,004720789	-0,14325946	0	N.A.	0
PIK3R3	0,004710108	0,031024648	0	N.A.	0
CTB-58E17.5	0,004694367	0,153967938	0	N.A.	0
AKAP2	0,004643777	0,253991034	0	N.A.	0
SPARC	0,004593037	-0,173135807	0	N.A.	0
BBX	0,004577543	-0,013882739	0	N.A.	0
GPR161	0,004498233	-0,062068426	0	N.A.	0
WEE1	0,004493898	0,099239088	0	N.A.	0
MICB	0,004464225	0,098767997	0	N.A.	0
RP13-554M1	0,004393657	0,371611845	0	N.A.	0
TIPARP-AS1	0,004386357	-0,021409995	0	N.A.	0
RP11-977G19	0,004379875	-0,000419396	0	N.A.	0
RP11-165E7.	0,004348581	0,024309003	0	N.A.	0
LINC01272	0,004348112	-0,257712527	0	N.A.	0
CTD-2336O2	0,004313855	0,199814752	0	N.A.	0
PRKX	0,004278441	-0,012852237	0	N.A.	0
AC108488.4	0,004241347	0,082795885	0	N.A.	0
STKLD1	0,004204032	0,20128348	0	N.A.	0
EPB41L4A-AS	0,004185617	-0,1009596	0	N.A.	0
TRGC1	0,004060796		0	N.A.	0

GNLY	0,004053536	-0,140150798	0	N.A.	0
ZNF763	0,004042334	0,175889801	0	N.A.	0
USF2	0,004038964	0,314850413	0	N.A.	0
RP11-143E21	0,00397764	0,264192172	0	N.A.	0
FGB	0,003966215	-0,013244507	0	N.A.	0
RP11-6I2.3	0,003960372		0	N.A.	0
MAP3K5	0,00390856	0,074959058	0	N.A.	0
ZNF285	0,003908276		0	N.A.	0
RP3-402G11.	0,00380383	0,150238573	0	N.A.	0
DGCR14	0,003787947	0,11981248	0	N.A.	0
ICAM5	0,003776036	0,129601357	0	N.A.	0
LINC01579	0,003748611	-0,101633528	0	N.A.	0
DNAJC9-AS1	0,003728864	-0,239634558	0	N.A.	0
MAGEE1	0,003718933		0	N.A.	0
LINC00907	0,003718843		0	N.A.	0
RP11-253M7	0,003710046		0	N.A.	0
RP11-158H5.	0,003697088	-0,261509598	0	N.A.	0
ITGB1BP2	0,003694426	-0,078166364	0	N.A.	0
NUTM2B-AS1	0,003651364	0,231649454	0	N.A.	0
DOC2A	0,003643877	0,097707393	0	N.A.	0
CTD-2349P21	0,003612965		0	N.A.	0
PC	0,003606625	0,07244716	0	N.A.	0
PPM1H	0,003603322	-0,20070649	0	N.A.	0
PPP5D1	0,003591071	0,029994093	0	N.A.	0
FERMT3	0,003534409	-0,183298168	0	N.A.	0
SH2D1A	0,003507208	-0,273498348	0	N.A.	0
ZNF510	0,003503376	-0,372582147	0	N.A.	0
SCN4A	0,003477434		0	N.A.	0
ZNF429	0,003469535	-0,190325736	0	N.A.	0
TSPAN4	0,003452209	-0,295126367	0	N.A.	0
ACOT9	0,003443792	0,275252461	0	N.A.	0
DPEP2	0,003425977		0	N.A.	0
DPY19L3	0,003403253	-0,091429925	0	N.A.	0

ZNF610	0,003398437	-0,143229782	0	N.A.	0
ZNF75A	0,003345588	-0,248813864	0	N.A.	0
TTLL4	0,003345579	0,250058292	0	N.A.	0
MFSD2B	0,003304088	-0,080918458	0	N.A.	0
MOGS	0,003251999	0,249884603	0	N.A.	0
FCAMR	0,003246702		0	N.A.	0
PRICKLE2	0,00323105	0,061237235	0	N.A.	0
AR	0,003195736		0	N.A.	0
APITD1-CORT	0,003190822	-0,284650984	0	N.A.	0
CHST15	0,00317554	-0,070935261	0	N.A.	0
RP11-624L4.1	0,003171552	-0,022192046	0	N.A.	0
JARID2-AS1	0,003103718	-0,225884463	0	N.A.	0
KCNIP2	0,003101712	-0,094451458	0	N.A.	0
MFSD8	0,003095719	0,101064904	0	N.A.	0
PPAPDC2	0,00309053	0,071561487	0	N.A.	0
NUDCD3	0,003029906	0,229499742	0	N.A.	0
TSPAN33	0,003022142	0,267784604	0	N.A.	0
ZNF547	0,003008948	0,34603388	0	N.A.	0
RFK	0,002994017	0,160986054	0	N.A.	0
FAM225B	0,002924933	0,19026848	0	N.A.	0
RP11-351J23	0,002903055	0,376015322	0	N.A.	0
NOX4	0,002811591	-0,04093386	0	N.A.	0
DHTKD1	0,002801473	0,110428259	0	N.A.	0
RASGRP2	0,002780291	-0,049160732	0	N.A.	0
RSPRY1	0,002765441	0,186066652	0	N.A.	0
FRY	0,002740196	-0,060541364	0	N.A.	0
RP11-1082L8	0,00273347		0	N.A.	0
LINC00379	0,002726317		0	N.A.	0
LINC01508	0,002711309	0,051521097	0	N.A.	0
RP11-101E13	0,002703249	-0,066043289	0	N.A.	0
CASKIN1	0,0026991	-0,06324418	0	N.A.	0
TMEM185B	0,002692847	0,038311516	0	N.A.	0
ZNF638	0,002598649	0,016278837	0	N.A.	0

HEATR5B	0,002587614	0,05732514	0	N.A.	0
CTD-2562J15	0,002578846		0	N.A.	0
SIPA1L2	0,002536517	-0,141341453	0	N.A.	0
SGMS1-AS1	0,00245903	0,125931419	0	N.A.	0
HDX	0,002439528	0,406637574	0	N.A.	0
GPRC5D	0,002436998	0,152009444	0	N.A.	0
CNOT3	0,002431754	0,072674985	0	N.A.	0
LAYN	0,002425438	-0,037717878	0	N.A.	0
KIAA0100	0,002424478	-0,010410518	0	N.A.	0
RP11-400N9.	0,002342979	-0,036480823	0	N.A.	0
HTRA4	0,002341745		0	N.A.	0
ANKRD34A	0,002308773	-0,040737619	0	N.A.	0
SAR1B	0,002272021	0,380344794	0	N.A.	0
KLK3	0,002269399	0,022974679	0	N.A.	0
CH507-9B2.3	0,002269227	-0,040462911	0	N.A.	0
MPZL1	0,002250574	0,116424528	0	N.A.	0
CTC-250I14.3	0,002224133		0	N.A.	0
RP11-195L15	0,00218783	0,125995268	0	N.A.	0
CTD-2231E14	0,002180621	0,042900913	0	N.A.	0
CHADL	0,002172459	-0,033564295	0	N.A.	0
SLC5A1	0,002158019	0,210751805	0	N.A.	0
RP11-83C7.1	0,002073775		0	N.A.	0
STPG1	0,002034766	-0,186459693	0	N.A.	0
LINC01554	0,001990467	0,038179167	0	N.A.	0
CASC2	0,00197397	-0,327879764	0	N.A.	0
TSPAN18	0,00193931	-0,025496438	0	N.A.	0
MIR143HG	0,001929822	-0,04779246	0	N.A.	0
SEPT1	0,00188915	-0,073993609	0	N.A.	0
TSSK3	0,001870187	-0,109524295	0	N.A.	0
RP11-432I5.2	0,00186788	0,054697443	0	N.A.	0
AL133245.2	0,00185419		0	N.A.	0
RP11-377D9.	0,00182814	-0,015994341	0	N.A.	0
ZADH2	0,001811672	0,000588547	0	N.A.	0

DHRS1	0,001731587	0,110356421	0	N.A.	0
AGAP4	0,001725798	0,24607293	0	N.A.	0
CTA-212A2.4	0,001723038	-0,134998157	0	N.A.	0
HSDL1	0,001719781	0,074572981	0	N.A.	0
RP11-575H3.	0,001692477	0,055017381	0	N.A.	0
MTUS2	0,001687515	-0,109552447	0	N.A.	0
MARCH5	0,001656663	-0,312985475	0	N.A.	0
LILRB2	0,001607317		0	N.A.	0
RP11-514P8.	0,001603989	-0,078970823	0	N.A.	0
MAP2K1	0,001568028	-0,020159773	0	N.A.	0
AP003419.16	0,001541107	-0,001487935	0	N.A.	0
TIGD7	0,001540259	-0,324960897	0	N.A.	0
RP11-467C18	0,001523878		0	N.A.	0
EFNA4	0,001506799	0,227813777	0	N.A.	0
RP1-80N2.3	0,001478779	-0,044480214	0	N.A.	0
PIK3R2	0,001476183	-0,093322999	0	N.A.	0
RP11-258F1.	0,001436367	0,044112527	0	N.A.	0
NYAP1	0,001432633	0,2595287	0	N.A.	0
LINC01353	0,001427856	0,029078615	0	N.A.	0
RP11-435J9.2	0,001425347		0	N.A.	0
COLEC11	0,001418365	-0,314671989	0	N.A.	0
CTB-113I20.2	0,001412588	-0,158227899	0	N.A.	0
RGS4	0,001388947		0	N.A.	0
RP11-823E8.	0,001368753		0	N.A.	0
ADARB1	0,001301031	-0,006349643	0	N.A.	0
OBP2B	0,001294242		0	N.A.	0
NKD2	0,001244678	-0,068972074	0	N.A.	0
ZNF839	0,001234381	-0,043249664	0	N.A.	0
SLC9A3	0,001197248		0	N.A.	0
RAD21-AS1	0,001178563	-0,089026479	0	N.A.	0
AGPAT1	0,001173443	-0,244561255	0	N.A.	0
NXPE1	0,001157224	-0,069933483	0	N.A.	0
PRR26	0,001152009	-0,197205517	0	N.A.	0

SLC38A1	0,00112001	0,07036791	0	N.A.	0
RP11-29H23.	0,001115725	-0,213918434	0	N.A.	0
SRRM3	0,001081051	0,487939231	0	N.A.	0
RP11-616M2	0,001033315	0,404457809	0	N.A.	0
DNAJC28	0,001008828	0,294466495	0	N.A.	0
RASSF2	0,000997187	-0,164192624	0	N.A.	0
PTPRG	0,000966533	0,05079868	0	N.A.	0
KLLN	0,000928846	-0,090888159	0	N.A.	0
RP11-350N1	0,000918695	0,076226398	0	N.A.	0
CDC73	0,000916074	0,470251908	0	N.A.	0
MED1	0,000884477	0,08036883	0	N.A.	0
BFSP1	0,0008716	0,284681288	0	N.A.	0
LDHC	0,000856587	-0,420094802	0	N.A.	0
C2orf50	0,000844259	-0,085713322	0	N.A.	0
C14orf159	0,000836887	-0,113098712	0	N.A.	0
TRAK2	0,000830186	0,208706764	0	N.A.	0
MIF-AS1	0,000807731	-0,19388195	0	N.A.	0
HOXC5	0,000789237	0,128533111	0	N.A.	0
RP11-273G1	0,000777213		0	N.A.	0
CALM2	0,000753471	0,012538765	0	N.A.	0
LCLAT1	0,000737942	-0,027172525	0	N.A.	0
GAS8	0,000735143	0,016790504	0	N.A.	0
HYDIN	0,000726372	-0,270187	0	N.A.	0
HES2	0,000722535	0,060864651	0	N.A.	0
CTD-2026K1	0,000711271		0	N.A.	0
RP11-488L18	0,000697424	0,010531615	0	N.A.	0
ZDHHC8	0,000693215	0,179969931	0	N.A.	0
RP11-73M18	0,000672172	-0,149130166	0	N.A.	0
RP13-20L14.	0,000620361	-0,10579413	0	N.A.	0
RP11-497H1	0,000600977	-0,002081159	0	N.A.	0
KBTBD8	0,000575125	-0,201179426	0	N.A.	0
IGSF22	0,000555108	-0,227136239	0	N.A.	0
FAM167A	0,000459048	-0,082520373	0	N.A.	0

IGKV1-39	0,000396291	-0,102583013	0	N.A.	0
RP11-818O24	0,000322593		0	N.A.	0
FAM168B	0,000313224	0,137320137	0	N.A.	0
RP11-118K6.	0,000266728	0,192266869	0	N.A.	0
TMTC1	0,000239849		0	N.A.	0
RP11-196G11	0,000228244	-0,114466462	0	N.A.	0
RP11-473M2	0,000215724		0	N.A.	0
ASB1	0,000198562	-0,025148805	0	N.A.	0
FAM20B	0,000176894	0,119392836	0	N.A.	0
DENND6A	0,000117635	-0,163273559	0	N.A.	0
SRGN	0,000112492	-0,233222827	0	N.A.	0
SIAH1	0,00010757	0,391683744	0	N.A.	0
ZNF608	0,000105183	-0,095780847	0	N.A.	0
RP11-367G6.	9,93893E-05	-0,088758096	0	N.A.	0
SLC7A6	9,86744E-05	0,319447272	0	N.A.	0
RP11-505P4.	9,57138E-05		0	N.A.	0
APLN	7,49573E-05	0,246445651	0	N.A.	0
TMEM91	6,10941E-05	-0,129042387	0	N.A.	0
CEACAM19	6,07599E-05	-0,14715335	0	N.A.	0
LINC00942	3,9778E-05	0,05472756	0	N.A.	0
ZNF823	-1,18282E-05	-0,093802578	0	N.A.	0
LEFTY2	-1,27631E-05	-0,076351601	0	N.A.	0
PPP1R42	-4,13001E-05		0	N.A.	0
RBBP8	-4,84071E-05	-0,018495689	0	N.A.	0
EMILIN1	-6,48376E-05	0,364320607	0	N.A.	0
IGKV2-24	-7,16739E-05		0	N.A.	0
RP1-292L20.3	-0,000125522	0,105659761	0	N.A.	0
PAK1	-0,000135019	0,19775324	0	N.A.	0
RP11-540K16	-0,00014929		0	N.A.	0
AC019186.1	-0,00020136	0,115877089	0	N.A.	0
CDC37L1-AS1	-0,000207821	-0,156322784	0	N.A.	0
RP11-862L9.3	-0,000213991		0	N.A.	0
MED17	-0,000219569	0,08856868	0	N.A.	0

RP4-621N11.	-0,000232981	0,107125664	0	N.A.	0
BRINP2	-0,000236368	0,14117977	0	N.A.	0
OXTR	-0,000267719	-0,032485965	0	N.A.	0
RYR2	-0,00028113	-0,077078527	0	N.A.	0
PKDCC	-0,000285059	0,197974545	0	N.A.	0
IL10RA	-0,000407829	-0,349158047	0	N.A.	0
NOVA1	-0,000421355		0	N.A.	0
ZBTB14	-0,000443069	0,086900272	0	N.A.	0
CITED1	-0,000455359	0,129947062	0	N.A.	0
ANKS1B	-0,000465931		0	N.A.	0
KLHL22	-0,000482164	-0,001049344	0	N.A.	0
KIF1A	-0,000488677		0	N.A.	0
EFHB	-0,000494049	-0,001523976	0	N.A.	0
GALT	-0,000507957	-0,100397897	0	N.A.	0
CTC-490G23.	-0,000583652	-0,150208323	0	N.A.	0
PRKCB	-0,000642745	-0,138899593	0	N.A.	0
C1orf162	-0,000667075	-0,049253213	0	N.A.	0
SLC23A2	-0,000674981	0,143172793	0	N.A.	0
ZNF33A	-0,000685175	-0,083414601	0	N.A.	0
SLC35A2	-0,000690407	0,29328723	0	N.A.	0
IGLV2-14	-0,000721391	-0,199421529	0	N.A.	0
RP11-425A6.	-0,000723251		0	N.A.	0
ZNF672	-0,000725812	-0,148908908	0	N.A.	0
SLC2A11	-0,00072629	-0,086101041	0	N.A.	0
LINC00518	-0,000744129	0,253107376	0	N.A.	0
EMCN	-0,00076009	-0,026564488	0	N.A.	0
CELA3A	-0,000777627	0,270033864	0	N.A.	0
ARNT2	-0,000790779	0,008947265	0	N.A.	0
CACNA1E	-0,000827268		0	N.A.	0
AP000344.3	-0,0008285	-0,174765926	0	N.A.	0
LINC00982	-0,000840059		0	N.A.	0
TRPM5	-0,000847412	-0,040087865	0	N.A.	0
HIST1H3A	-0,000863342	-0,265803453	0	N.A.	0

RP11-727A23	-0,000901965	0,160492765	0	N.A.	0
ANKRD65	-0,00095859	-0,245514616	0	N.A.	0
HIST1H2AI	-0,000965092	0,01461122	0	N.A.	0
RP11-500G22	-0,000978012		0	N.A.	0
RP11-297P16	-0,000982686	0,185361819	0	N.A.	0
RP3-467N11.	-0,001005566	-0,073813227	0	N.A.	0
AZIN2	-0,001063722	0,005408607	0	N.A.	0
TRPV4	-0,001073034	-0,307490807	0	N.A.	0
RP11-357H14	-0,00110483	0,274977046	0	N.A.	0
RDH11	-0,001134149	0,261140752	0	N.A.	0
RP11-85L21.4	-0,001144722		0	N.A.	0
LINC00539	-0,001167292	-0,348839689	0	N.A.	0
RP11-44N11.	-0,00118973	-0,113623148	0	N.A.	0
GJB6	-0,001215846	0,019455818	0	N.A.	0
CARD8-AS1	-0,001288898	0,091832706	0	N.A.	0
CCDC114	-0,001299867	0,171071584	0	N.A.	0
RP11-849H4.	-0,00133476	-0,307430028	0	N.A.	0
RP11-16P6.1	-0,001335214	0,19751104	0	N.A.	0
CTA-14H9.5	-0,001335988	0,070976508	0	N.A.	0
UBE3C	-0,001364711	0,349757353	0	N.A.	0
RP11-150O12	-0,001364715	-0,056315218	0	N.A.	0
CTD-2270L9.	-0,001375499	-0,140455172	0	N.A.	0
VHL	-0,001384913	-0,143153679	0	N.A.	0
AP000230.1	-0,001433405		0	N.A.	0
RP11-362K14	-0,001491079	-0,056878355	0	N.A.	0
CTD-2302E22	-0,001496235	-0,013016838	0	N.A.	0
EBF4	-0,001525371	0,201976515	0	N.A.	0
ZNF268	-0,001534001	-0,000418775	0	N.A.	0
STAG1	-0,001548919	-0,079629171	0	N.A.	0
U73166.2	-0,001562007	0,44192734	0	N.A.	0
NTN5	-0,001609926	0,050110687	0	N.A.	0
ACTA2	-0,001630833	-0,191074124	0	N.A.	0
SLC13A2	-0,001638989	0,154265089	0	N.A.	0

AC108868.6	-0,001658009	0,061598112	0	N.A.	0
DNAJC12	-0,001687548	-0,050749619	0	N.A.	0
GRASP	-0,001718175	-0,269354945	0	N.A.	0
TMEM104	-0,001737535	0,245341076	0	N.A.	0
GDF11	-0,001737617	0,030832664	0	N.A.	0
GUCY1A3	-0,001754108		0	N.A.	0
FYN	-0,00176843	0,103350423	0	N.A.	0
PDXDC1	-0,001807479	0,385874646	0	N.A.	0
NCOR1	-0,001886311	0,290208771	0	N.A.	0
ZFAND2A	-0,001926552	0,421680573	0	N.A.	0
FGFR3	-0,001947256	0,167975818	0	N.A.	0
RP11-110I1.1	-0,001948819	-0,031994112	0	N.A.	0
EFCAB8	-0,001991606		0	N.A.	0
AASS	-0,002013216	-0,12237008	0	N.A.	0
RP1-202O8.3	-0,002068411	0,055927098	0	N.A.	0
ZNF575	-0,002108556	0,00434307	0	N.A.	0
PLA2G1B	-0,002116661	-0,058220557	0	N.A.	0
PNPLA3	-0,002117823	0,123895513	0	N.A.	0
CTA-384D8.3	-0,002124348	-0,069028052	0	N.A.	0
FERMT2	-0,002134641	-0,171581455	0	N.A.	0
RASGRF2	-0,002188659	0,333969289	0	N.A.	0
SASH1	-0,002214377	-0,011885974	0	N.A.	0
LINC00330	-0,002224281	-0,03613409	0	N.A.	0
CASP8	-0,002230129	0,088896994	0	N.A.	0
NKX2-5	-0,002256852		0	N.A.	0
ATP6V0A2	-0,002270096	0,259472008	0	N.A.	0
ARL11	-0,002275525	-0,100946746	0	N.A.	0
RP11-471M2	-0,002315766	0,071982769	0	N.A.	0
ZBTB49	-0,00234106	0,170391142	0	N.A.	0
SYN2	-0,002384488		0	N.A.	0
CSRP1	-0,002440147	-0,182817673	0	N.A.	0
RP11-164H13	-0,00244114		0	N.A.	0
FUK	-0,002445519	-0,08740401	0	N.A.	0

RP11-443B7.	-0,002460653	0,072565575	0	N.A.	0
PILRB	-0,002539244	0,08731291	0	N.A.	0
RP5-1112D6.	-0,002567588	0,113225045	0	N.A.	0
SDE2	-0,002593247	0,24811854	0	N.A.	0
MATN4	-0,002602389	0,014816338	0	N.A.	0
COL11A2	-0,002604702	0,07148131	0	N.A.	0
AC113189.5	-0,002610945	0,03712464	0	N.A.	0
ST6GALNAC3	-0,002611368		0	N.A.	0
PLD4	-0,002628623		0	N.A.	0
PDE11A	-0,002637046	-0,098018139	0	N.A.	0
AKAP8L	-0,002666373	-0,099198852	0	N.A.	0
TTN	-0,002680346	0,008412637	0	N.A.	0
GLB1L	-0,002707008	0,082214765	0	N.A.	0
COL1A1	-0,002711534	-0,0475467	0	N.A.	0
FAM155B	-0,002739973		0	N.A.	0
AC131097.4	-0,002741081		0	N.A.	0
RP1-92O14.3	-0,002764043	0,115039497	0	N.A.	0
FGF19	-0,002766895	0,24318832	0	N.A.	0
AC091814.2	-0,00276971		0	N.A.	0
UXS1	-0,002778632	0,285131134	0	N.A.	0
GDF15	-0,002804339	0,351009069	0	N.A.	0
CRYGS	-0,002823626	0,016399497	0	N.A.	0
CTD-2650P22	-0,002830746		0	N.A.	0
LRRC48	-0,002867729	0,050243583	0	N.A.	0
RP11-474O21	-0,002875539	-0,291562418	0	N.A.	0
CLEC2B	-0,002876586	0,081369784	0	N.A.	0
OSER1	-0,002883606	-0,087005655	0	N.A.	0
GGTLC3	-0,002887321		0	N.A.	0
CTD-2007H1	-0,002894494	-0,144327239	0	N.A.	0
AC005954.4	-0,00291938	0,042551129	0	N.A.	0
TMEM64	-0,002934432	-0,002279519	0	N.A.	0
TTLL10	-0,002944437	0,025681908	0	N.A.	0
RP11-219B17	-0,00296015		0	N.A.	0

LIPJ	-0,002965884		0	N.A.	0
SEC61G	-0,003002582	-0,22147065	0	N.A.	0
AP1B1	-0,003012221	0,09038991	0	N.A.	0
ST20-AS1	-0,003020829	-0,041673645	0	N.A.	0
BMP7	-0,003029546	0,27762239	0	N.A.	0
TRAM1L1	-0,003031833	-0,09000857	0	N.A.	0
GATA6-AS1	-0,003068309	-0,0903477	0	N.A.	0
MTPN	-0,00307007	0,01799233	0	N.A.	0
HSD11B2	-0,003070502	0,145918974	0	N.A.	0
BCL2	-0,003070618	-0,260082404	0	N.A.	0
LINC01003	-0,003113282	0,22072602	0	N.A.	0
RP11-135L13	-0,003166081	0,034658328	0	N.A.	0
LINC01152	-0,003244698	0,086571502	0	N.A.	0
RP11-534C12	-0,003276921		0	N.A.	0
TARSL2	-0,003299803	0,262977383	0	N.A.	0
FEM1A	-0,003353498	0,293869269	0	N.A.	0
HIST1H2BB	-0,00338003	-0,219575576	0	N.A.	0
RRS1-AS1	-0,003409194		0	N.A.	0
RP5-1068E13	-0,003424729	0,190665358	0	N.A.	0
RP4-756G23.	-0,003430445		0	N.A.	0
MS4A1	-0,003496297	-0,180666953	0	N.A.	0
RAB3C	-0,003521902		0	N.A.	0
GDAP1	-0,003555575	-0,175345557	0	N.A.	0
ERICH1	-0,003559633	0,313429373	0	N.A.	0
COPB1	-0,003637523	0,291079995	0	N.A.	0
GIMAP2	-0,003637567	-0,214244257	0	N.A.	0
WBP4	-0,003669645	0,295368124	0	N.A.	0
CACNB2	-0,003716888	-0,316215629	0	N.A.	0
RP11-783K16	-0,003718466	0,210110781	0	N.A.	0
ZNF136	-0,003755125	0,117116214	0	N.A.	0
CTB-75G16.3	-0,003771122		0	N.A.	0
AC005355.2	-0,003778064	-0,248966268	0	N.A.	0
FBXL22	-0,003833921	-0,073756802	0	N.A.	0

DYRK3	-0,003906957	0,026802236	0	N.A.	0
MSMP	-0,003917975		0	N.A.	0
IGKV2D-24	-0,003930151		0	N.A.	0
SYNGAP1	-0,003947014	0,14741977	0	N.A.	0
DYRK1B	-0,003998949	0,037387235	0	N.A.	0
TMEM240	-0,00408853	0,331466477	0	N.A.	0
CTA-221G9.7	-0,004109488	0,255234424	0	N.A.	0
SUMF1	-0,004122922	-0,097789621	0	N.A.	0
CTD-3222D1	-0,004137326	0,080417911	0	N.A.	0
RP11-615I2.6	-0,0041481		0	N.A.	0
EFCAB12	-0,004202309	-0,004337111	0	N.A.	0
DLGAP2	-0,004206771		0	N.A.	0
GLUD1	-0,004249489	0,17464499	0	N.A.	0
ZNF506	-0,004259924	-0,020651486	0	N.A.	0
ARMC4	-0,004302979		0	N.A.	0
RP1-86C11.7	-0,004306514		0	N.A.	0
RP3-406P24.	-0,004342538		0	N.A.	0
AC022007.5	-0,004354628	0,106502794	0	N.A.	0
RP11-67L3.4	-0,0043964	-0,009403856	0	N.A.	0
LINC00523	-0,004422758	0,310615491	0	N.A.	0
RP11-302F12	-0,004426572		0	N.A.	0
ZNF585B	-0,004429297	0,091145368	0	N.A.	0
F7	-0,004452107	-0,002953204	0	N.A.	0
LINC00545	-0,004456956	-0,021263805	0	N.A.	0
SEMA6B	-0,004469817	0,15477685	0	N.A.	0
PRDM4	-0,004520897	-0,052889352	0	N.A.	0
CTD-2619J13	-0,004526957	-0,049338628	0	N.A.	0
RP11-2E11.9	-0,004531828	0,144270463	0	N.A.	0
TSNAX-DISC1	-0,004563645	-0,007866093	0	N.A.	0
RP1-163G9.2	-0,004609912		0	N.A.	0
CTC-273B12.	-0,004609924	0,116237638	0	N.A.	0
RP11-611O2.	-0,004610701	0,072565575	0	N.A.	0
VIM	-0,004631678	-0,407737952	0	N.A.	0

BMP6	-0,004634869		0	N.A.	0
ALDH1L2	-0,004645664	-0,080839488	0	N.A.	0
AC093732.1	-0,004684892	-0,05262621	0	N.A.	0
RP11-61E11.	-0,004749517		0	N.A.	0
RP5-1096D14	-0,004768304		0	N.A.	0
TNNI3	-0,00478155	-0,025783709	0	N.A.	0
AC005786.7	-0,004787845	0,099005435	0	N.A.	0
RP11-285F7.	-0,004808533	0,028976771	0	N.A.	0
NADSYN1	-0,004876257	-0,04960793	0	N.A.	0
RP11-430B1.	-0,004894913	0,202513281	0	N.A.	0
C9orf91	-0,004928249	-0,078732383	0	N.A.	0
RP11-408H1.	-0,00493146	-0,027784039	0	N.A.	0
RP4-798A10.	-0,004945466	0,323215644	0	N.A.	0
SEMA3F	-0,00496652	0,150125602	0	N.A.	0
ICA1	-0,00499971	0,312274725	0	N.A.	0
RP11-103J8.2	-0,00504362	-0,096469944	0	N.A.	0
BCL2A1	-0,005072868	0,260192494	0	N.A.	0
LEKR1	-0,005075121	0,051899838	0	N.A.	0
ZNF276	-0,005096144	0,23206218	0	N.A.	0
FCRL5	-0,005119478		0	N.A.	0
TTLL5	-0,005130751	-0,230614112	0	N.A.	0
NBPF26	-0,00513743	-0,024894597	0	N.A.	0
CLMP	-0,005180867	-0,164379635	0	N.A.	0
MTRNR2L13	-0,005233393		0	N.A.	0
LAG3	-0,005250605	-0,034171971	0	N.A.	0
AC145124.2	-0,005261392	0,164968005	0	N.A.	0
GPR3	-0,005263499	0,080892612	0	N.A.	0
ETV1	-0,005312247	0,143615319	0	N.A.	0
ARHGAP26-A	-0,005317914	0,018496156	0	N.A.	0
GNA14-AS1	-0,005346205		0	N.A.	0
CXorf40B	-0,005352932	-0,19269301	0	N.A.	0
DIO1	-0,005368117		0	N.A.	0
S100Z	-0,005368488		0	N.A.	0

RP13-580B18	-0,005382776	0,291809992	0	N.A.	0
CTC-251D13.	-0,005396622	0,165360447	0	N.A.	0
GTF2A1	-0,005411595	0,133276219	0	N.A.	0
CTD-2035E11	-0,00541527	0,263876912	0	N.A.	0
ITGA4	-0,005495249	-0,216897317	0	N.A.	0
AQP3	-0,005496947	0,03482334	0	N.A.	0
IGHV3-43	-0,005589551		0	N.A.	0
ZKSCAN1	-0,005604719	0,27488372	0	N.A.	0
ATG7	-0,005607076	-0,06585885	0	N.A.	0
RP5-1009E24	-0,00565221	0,157882403	0	N.A.	0
SPRY4-IT1	-0,005672715	0,005876191	0	N.A.	0
RP11-138I1.2	-0,005712732		0	N.A.	0
RP4-622L5.7	-0,005791015	0,268280626	0	N.A.	0
C3AR1	-0,005803026	0,307833848	0	N.A.	0
NFYC-AS1	-0,005817312	0,033154942	0	N.A.	0
RP11-177J6.1	-0,005820506		0	N.A.	0
POMT1	-0,005820809	0,052369059	0	N.A.	0
KCNK10	-0,005842817	0,134280488	0	N.A.	0
DDX47	-0,00590422	-0,149156411	0	N.A.	0
CTD-2503I6.1	-0,005951266		0	N.A.	0
AC004895.4	-0,00599623	0,100530125	0	N.A.	0
LTB4R	-0,006045311	0,126824499	0	N.A.	0
CREM	-0,006091284	-0,299074462	0	N.A.	0
PRH2	-0,006113561		0	N.A.	0
CCR5	-0,006118123	0,122834765	0	N.A.	0
RP13-650J16	-0,00613096	0,011657518	0	N.A.	0
WWC2-AS2	-0,006169886	0,118705979	0	N.A.	0
MED18	-0,006218954	-0,090861905	0	N.A.	0
RP11-552D8.	-0,006271095	0,327317507	0	N.A.	0
RP11-89F3.2	-0,006290406	-0,062627744	0	N.A.	0
RP13-467H17	-0,006301123	-0,176306557	0	N.A.	0
RP11-413M3	-0,006324827		0	N.A.	0
RP11-787I22	-0,006365226		0	N.A.	0

S1PR3	-0,006378301	0,13353991	0	N.A.	0
CDKL2	-0,006425285	0,178802143	0	N.A.	0
PRSS1	-0,006451188	0,215332874	0	N.A.	0
ZNF25	-0,006460706	0,271915599	0	N.A.	0
KRTAP1-1	-0,006482609		0	N.A.	0
AIFM2	-0,006484292	-0,016888806	0	N.A.	0
SNURF	-0,006551507	-0,338316193	0	N.A.	0
RP11-552F3.9	-0,006610857	0,209574766	0	N.A.	0
KATNAL1	-0,006631992	0,069629519	0	N.A.	0
DLL4	-0,006654568	0,046706508	0	N.A.	0
RP11-354P11	-0,006665311	0,306815492	0	N.A.	0
RP11-247A12	-0,00666542	-0,04965632	0	N.A.	0
TRPC4AP	-0,006691479	0,410120763	0	N.A.	0
RP4-613B23.	-0,006692633	0,090175989	0	N.A.	0
MTMR8	-0,006698364	0,231985449	0	N.A.	0
AOX1	-0,006717788	-0,187263351	0	N.A.	0
LINC01436	-0,006719173	-0,328536498	0	N.A.	0
RBM15	-0,006723229	-0,270030537	0	N.A.	0
PABPC1L	-0,006732305	-0,083289627	0	N.A.	0
MYO1F	-0,006740878	0,080403452	0	N.A.	0
SCAANT1	-0,006784549		0	N.A.	0
AC142293.3	-0,006790392	-0,121880195	0	N.A.	0
ARHGAP24	-0,006807397	-0,061623998	0	N.A.	0
MID1IP1-AS1	-0,006810239	0,164993103	0	N.A.	0
CTD-3064M3	-0,006881046		0	N.A.	0
MT-CO3	-0,006884731	0,21473431	0	N.A.	0
ST6GAL2	-0,006892521	-0,042481034	0	N.A.	0
WDR78	-0,006907768	0,307065894	0	N.A.	0
LINC00309	-0,006920412		0	N.A.	0
PINK1-AS	-0,00693188	0,222999212	0	N.A.	0
RBM48	-0,006935283	-0,393535355	0	N.A.	0
RP11-799D4.	-0,006940687	0,013574364	0	N.A.	0
AC064834.1	-0,006944846	-0,334615788	0	N.A.	0

LRRC39	-0,006976329	0,034246956	0	N.A.	0
DOCK2	-0,006979	0,119668595	0	N.A.	0
RP11-485G7.	-0,007070119	-0,194949289	0	N.A.	0
BCL9	-0,007102961	-0,067640586	0	N.A.	0
NDN	-0,007151575	0,022272456	0	N.A.	0
STRADA	-0,00715445	0,012045647	0	N.A.	0
TAF1	-0,00716797	0,324914006	0	N.A.	0
MAT2A	-0,00718615	0,299783252	0	N.A.	0
ABCA3	-0,007200605	-0,042844987	0	N.A.	0
ZFP2	-0,007203369	-0,059914316	0	N.A.	0
FAM212B-AS	-0,007235431		0	N.A.	0
LINC00202-1	-0,007241608	0,208006948	0	N.A.	0
RSPH1	-0,00724788	-0,052841051	0	N.A.	0
IFIT1B	-0,00727443		0	N.A.	0
AP001347.6	-0,007277985	-0,128302674	0	N.A.	0
CCDC144NL	-0,007362448	-0,108389047	0	N.A.	0
RP11-351M1	-0,007367472	0,237388584	0	N.A.	0
TEN1-CDK3	-0,007389444	0,329727718	0	N.A.	0
GPR171	-0,007402753	-0,025274358	0	N.A.	0
TMEM56-RW	-0,007420414	-0,156191949	0	N.A.	0
FBXO21	-0,007434276	0,248234649	0	N.A.	0
HIST2H4B	-0,007434434		0	N.A.	0
FOXG1	-0,007435624	-0,076788981	0	N.A.	0
CCDC168	-0,007457051	-0,029872433	0	N.A.	0
AC108142.1	-0,007458279	-0,179113103	0	N.A.	0
DOK3	-0,007469513	-0,102312234	0	N.A.	0
BEGAIN	-0,007476241	0,004629465	0	N.A.	0
PLIN2	-0,007476241	-0,20296877	0	N.A.	0
RGS2	-0,007486747	0,036194047	0	N.A.	0
RP11-546K22	-0,007504204	0,290468764	0	N.A.	0
AZU1	-0,007512187		0	N.A.	0
KCNE1	-0,007547804		0	N.A.	0
KCNJ14	-0,007555149	-0,10562524	0	N.A.	0

TLR2	-0,007616433	0,131292385	0	N.A.	0
TMPRSS9	-0,007620663		0	N.A.	0
RP11-93G5.1	-0,007637384	0,145841722	0	N.A.	0
RNFT1	-0,007655787	-0,045325082	0	N.A.	0
ZBTB44	-0,007661311	-0,197774535	0	N.A.	0
ADPGK	-0,007721511	-0,062484242	0	N.A.	0
JADE2	-0,00773143	0,232948148	0	N.A.	0
ADCYAP1	-0,007782008	0,027322808	0	N.A.	0
ABCB11	-0,007795698		0	N.A.	0
C9orf72	-0,007825664	-0,039982681	0	N.A.	0
TATDN3	-0,007849896	0,054237472	0	N.A.	0
ZNF500	-0,00785876	-0,056487976	0	N.A.	0
RTBDN	-0,007928789		0	N.A.	0
RP5-851M4.1	-0,007934454		0	N.A.	0
C15orf41	-0,007986042	-0,059879744	0	N.A.	0
PPP1R32	-0,007992681	0,043437039	0	N.A.	0
LY6K	-0,008052476	0,018592192	0	N.A.	0
AKAP9	-0,00807229	0,349116586	0	N.A.	0
ZNF630	-0,008089438	-0,080883264	0	N.A.	0
RP11-345P4.	-0,008142771	-0,116062048	0	N.A.	0
NR2F1-AS1	-0,008184238	-0,104725616	0	N.A.	0
INCA1	-0,008205757	-0,039890644	0	N.A.	0
ZNF764	-0,008213995	-0,184539563	0	N.A.	0
AC005224.2	-0,008216825	-0,039865291	0	N.A.	0
LINC00630	-0,00823427	0,284049898	0	N.A.	0
BRD9	-0,008294584	0,103368531	0	N.A.	0
RP11-627G18	-0,008299344	0,029070831	0	N.A.	0
RP11-339B21	-0,008303798	-0,045211831	0	N.A.	0
RP11-553A21	-0,008315948	-0,153881635	0	N.A.	0
GZMM	-0,008316828	-0,19861074	0	N.A.	0
OVOL1-AS1	-0,008326799	-0,094812583	0	N.A.	0
RAPGEF6	-0,008366837	-0,336446018	0	N.A.	0
SPRR2E	-0,008379291		0	N.A.	0

CD46	-0,008447767	0,286160403	0	N.A.	0
SUPT4H1	-0,008451591	-0,020633336	0	N.A.	0
FAM110B	-0,008452455	-0,2577597	0	N.A.	0
HEXA-AS1	-0,00846479	-0,088554534	0	N.A.	0
PRTN3	-0,008501893		0	N.A.	0
RP11-276E17	-0,008503822	-0,310658695	0	N.A.	0
AC107081.5	-0,008509574		0	N.A.	0
TFAP2E	-0,008520554		0	N.A.	0
LINC01169	-0,008542178		0	N.A.	0
SULT1A1	-0,008550949	-0,021353172	0	N.A.	0
IFT140	-0,008574243	0,069241894	0	N.A.	0
ROR1-AS1	-0,008601896		0	N.A.	0
BAI1	-0,008637287	0,127322382	0	N.A.	0
CTD-2515A14	-0,008658345		0	N.A.	0
ZNF618	-0,008706559	0,033868007	0	N.A.	0
RP11-391J2.3	-0,008774245	0,05933785	0	N.A.	0
RP11-390P24	-0,008786363	-0,005195317	0	N.A.	0
CDH19	-0,008790237		0	N.A.	0
CDH2	-0,008790237		0	N.A.	0
HSPB7	-0,008790237	-0,11147247	0	N.A.	0
MYOT	-0,008790237		0	N.A.	0
PABPC5	-0,008790237		0	N.A.	0
PLP1	-0,008790237		0	N.A.	0
RADIL	-0,008790237		0	N.A.	0
RP11-557H15	-0,008790237		0	N.A.	0
RP11-92C4.6	-0,008790237	-0,338316193	0	N.A.	0
SLC26A10	-0,008790237	-0,120597876	0	N.A.	0
SLITRK2	-0,008790237		0	N.A.	0
ST3GAL6-AS1	-0,008790237	-0,268040146	0	N.A.	0
ST6GALNAC5	-0,008790237	0,179906687	0	N.A.	0
TRPC4	-0,008790237		0	N.A.	0
RFX3-AS1	-0,00879325	-0,316218562	0	N.A.	0
CCL18	-0,008805053	0,028922551	0	N.A.	0

C15orf27	-0,008837193	-0,045211831	0	N.A.	0
FOXD4L1	-0,008846114		0	N.A.	0
CCDC85A	-0,008859598	-0,066982504	0	N.A.	0
OSR1	-0,008874784	-0,082059541	0	N.A.	0
MAP3K7	-0,008912742	-0,126759715	0	N.A.	0
PEX2	-0,009001321	-0,074481851	0	N.A.	0
RP11-107M1	-0,009068283	-0,118487322	0	N.A.	0
RP11-108K3.	-0,009079511	-0,045182377	0	N.A.	0
RP5-1028K7.	-0,009083872	-0,239605536	0	N.A.	0
CRLF3	-0,009086271	0,079535178	0	N.A.	0
ANKS3	-0,009120728	0,282609465	0	N.A.	0
GRM4	-0,009120923		0	N.A.	0
RP11-383M4	-0,00919415	-0,047646538	0	N.A.	0
RP4-534N18.	-0,009194193		0	N.A.	0
RBM15B	-0,009195862	0,144198685	0	N.A.	0
PTDSS2	-0,009218772	-0,06064974	0	N.A.	0
RUNDC3B	-0,00922216	0,07023245	0	N.A.	0
RP11-259N1	-0,009282776	0,191982634	0	N.A.	0
PARVG	-0,009296748	-0,159331328	0	N.A.	0
PROZ	-0,009300723	0,117826795	0	N.A.	0
CHKB-CPT1B	-0,00938451	0,073785746	0	N.A.	0
INHBA	-0,009402737	-0,065442293	0	N.A.	0
POLH	-0,009426173	-0,065135267	0	N.A.	0
MFAP3L	-0,009438069	-0,093326318	0	N.A.	0
BANP	-0,009475504	-0,073817776	0	N.A.	0
RP11-1100L3	-0,009503131	0,285769448	0	N.A.	0
RP11-504A18	-0,009515828	0,141658286	0	N.A.	0
CTD-2006H1	-0,009534942	0,040554777	0	N.A.	0
LINC01372	-0,009569818	0,164633872	0	N.A.	0
AC008781.7	-0,009584584	-0,113815587	0	N.A.	0
NCKIPSD	-0,009604358	0,048435344	0	N.A.	0
RP11-403P17	-0,009610729		0	N.A.	0
NSUN4	-0,009624781	0,089403254	0	N.A.	0

SNRPN	-0,009626504	-0,093921772	0	N.A.	0
HYAL2	-0,009630671	0,469625062	0	N.A.	0
CEP97	-0,009658451	-0,040600523	0	N.A.	0
AGER	-0,009668131	0,083632317	0	N.A.	0
PRDM11	-0,009668744	-0,11930917	0	N.A.	0
SLC4A4	-0,009710955	0,074488381	0	N.A.	0
TUBA1A	-0,009711616	0,14732137	0	N.A.	0
CTC-350I8.1	-0,009712128	0,092101135	0	N.A.	0
RP11-449P15	-0,009716912	0,262317464	0	N.A.	0
SOX10	-0,009723454		0	N.A.	0
ZNF248	-0,009739168	-0,2013252	0	N.A.	0
CYP51A1	-0,009761337	0,177093404	0	N.A.	0
RP11-162A12	-0,00979096	-0,264693937	0	N.A.	0
RDX	-0,009794953	-0,218493636	0	N.A.	0
RP11-1275H2	-0,009805265	-0,018265408	0	N.A.	0
RP11-395B7.	-0,009806028	0,157294363	0	N.A.	0
CTD-2165H1(	-0,009807522	0,072544781	0	N.A.	0
ACACB	-0,009816756	-0,057412396	0	N.A.	0
RP11-95O2.5	-0,009832938		0	N.A.	0
ZGPAT	-0,009862504	-0,084367225	0	N.A.	0
UHRF1BP1L	-0,009866583	-0,031175651	0	N.A.	0
AC005592.2	-0,009871639	0,140115259	0	N.A.	0
ZNF10	-0,009919352	-0,234561615	0	N.A.	0
GPR133	-0,009924953		0	N.A.	0
RP11-421L21	-0,009953673	-0,068864438	0	N.A.	0
ANXA9	-0,009956806	-0,235529911	0	N.A.	0
MIATNB	-0,009963577	0,000780525	0	N.A.	0
CELA1	-0,009998462		0	N.A.	0
CTD-2245F17	-0,010003193	-0,123764265	0	N.A.	0
CLDN5	-0,01000392	0,130647893	0	N.A.	0
TCF24	-0,010013381	0,417736727	0	N.A.	0
IPP	-0,01001621	0,077169025	0	N.A.	0
RP11-392P7.	-0,010016698	-0,23161075	0	N.A.	0

RP11-248M1	-0,010023616		0	N.A.	0
KB-1507C5.2	-0,010027554	-0,315902064	0	N.A.	0
CTD-2201E18	-0,010031635	0,011644009	0	N.A.	0
RP4-594I10.3	-0,01003406	-0,184137806	0	N.A.	0
NBPF1	-0,010052999	-0,060608877	0	N.A.	0
MC1R	-0,010105212	-0,059511062	0	N.A.	0
CHGB	-0,010116178	-0,003314536	0	N.A.	0
ZNF708	-0,010125769	0,096791004	0	N.A.	0
CCDC180	-0,010155732	0,027225882	0	N.A.	0
OTUD7A	-0,010158871	0,101526288	0	N.A.	0
PRSS33	-0,010208216	0,281880059	0	N.A.	0
RMND5B	-0,010218734	-0,015094025	0	N.A.	0
LYPD6B	-0,010226773	0,3820784	0	N.A.	0
PDCD1	-0,010231253	-0,25107506	0	N.A.	0
ZFAT	-0,010245334	-0,04146337	0	N.A.	0
RN7SL832P	-0,010247107	0,072109535	0	N.A.	0
AC133528.2	-0,010275334	0,174016899	0	N.A.	0
RP11-366L5.1	-0,010279761	0,399700867	0	N.A.	0
HLCS	-0,010330639	0,097367578	0	N.A.	0
RP11-277A4.	-0,010331275	-0,02742486	0	N.A.	0
ZAR1L	-0,010334321		0	N.A.	0
HIST1H3F	-0,010353834	-0,13213753	0	N.A.	0
ALKBH5	-0,010359432	0,262286617	0	N.A.	0
SDR42E2	-0,010362618	-0,116051518	0	N.A.	0
HIST1H3H	-0,010382391	-0,089324489	0	N.A.	0
CACNB3	-0,010399543	0,105304892	0	N.A.	0
IGLL1	-0,010405096		0	N.A.	0
RP11-319E16	-0,010417406	-0,068696423	0	N.A.	0
TMEM213	-0,010419962	-0,002060108	0	N.A.	0
C10orf76	-0,010441191	-0,087672869	0	N.A.	0
ELDR	-0,010468577		0	N.A.	0
ROBO4	-0,010507209	-0,124040505	0	N.A.	0
RAB36	-0,01053143	-0,20067844	0	N.A.	0

CLEC2D	-0,010546571	-0,180587793	0	N.A.	0
FADS3	-0,010565217	0,102440892	0	N.A.	0
RP13-1039J1	-0,010574395	0,023151751	0	N.A.	0
HIST1H3J	-0,010593949	-0,181190358	0	N.A.	0
RTCA	-0,010613428	-0,136591355	0	N.A.	0
ZNF350	-0,010697314	0,007859879	0	N.A.	0
PTPN2	-0,010697338	0,03196784	0	N.A.	0
LYPD5	-0,010704591	-0,280619257	0	N.A.	0
KANTR	-0,010723051	-0,017664637	0	N.A.	0
IGFALS	-0,010754881	0,011265832	0	N.A.	0
TPCN2	-0,010755262	0,084579858	0	N.A.	0
RP11-1E4.1	-0,010762323	0,239370682	0	N.A.	0
RP1-41C23.4	-0,01077061	0,041734934	0	N.A.	0
C2orf81	-0,010808219	-0,102120877	0	N.A.	0
HNRNPUL2	-0,010823636	0,247361505	0	N.A.	0
SPSB2	-0,010835803	0,399308381	0	N.A.	0
TCEANC	-0,010884426	0,284306348	0	N.A.	0
RP11-90D11.	-0,010886185		0	N.A.	0
MDFIC	-0,010898383	0,147626329	0	N.A.	0
ANO7	-0,010925556	-0,131015586	0	N.A.	0
PAX9	-0,010960446	0,118345058	0	N.A.	0
RP11-290F5.	-0,011029312	-0,063696316	0	N.A.	0
C8orf82	-0,011032611	0,309157076	0	N.A.	0
CDKL1	-0,011093836	-0,382331553	0	N.A.	0
CTA-204B4.2	-0,011169527	0,125217132	0	N.A.	0
WASF3	-0,011202785	-0,239822341	0	N.A.	0
RP11-70C1.1	-0,011217419		0	N.A.	0
RP11-874J12	-0,011231561	-0,118937287	0	N.A.	0
YIPF4	-0,011275706	0,450461419	0	N.A.	0
KIAA1549	-0,011339414	0,223483996	0	N.A.	0
KRTDAP	-0,011358559	0,184781697	0	N.A.	0
AP000936.4	-0,011395764		0	N.A.	0
RNF133	-0,011438348		0	N.A.	0

RHOBTB2	-0,011466221	0,251803457	0	N.A.	0
OBSCN	-0,011468574	-0,103102682	0	N.A.	0
RTKN	-0,011476635	0,458531052	0	N.A.	0
DGKI	-0,011478828		0	N.A.	0
GS1-293C5.1	-0,01148677	0,347170563	0	N.A.	0
GAS2L3	-0,011507548	-0,294655716	0	N.A.	0
RP11-527L4.2	-0,011508299		0	N.A.	0
MTX3	-0,011514373	-0,260452817	0	N.A.	0
ZNF215	-0,011531824	-0,367794363	0	N.A.	0
DLX2	-0,011562391	0,19195881	0	N.A.	0
GPR75-ASB3	-0,011582231	0,113554045	0	N.A.	0
CTD-2562J15	-0,01162828	0,008897243	0	N.A.	0
PCSK1N	-0,011628409	0,195582835	0	N.A.	0
CTD-2196E14	-0,011680138	0,262282366	0	N.A.	0
CENPB	-0,01170001	0,333880576	0	N.A.	0
SIX5	-0,011710592	-0,102931928	0	N.A.	0
RGS9	-0,011779699		0	N.A.	0
DDX6	-0,011781804	0,298535709	0	N.A.	0
MAP1A	-0,011882965		0	N.A.	0
RP11-384O8.1	-0,01190482	0,051905023	0	N.A.	0
CEP95	-0,011961375	0,012830043	0	N.A.	0
CTB-50L17.14	-0,011967277		0	N.A.	0
RP11-290L1.2	-0,012003752	-0,058784395	0	N.A.	0
FAM24B	-0,01215422	-0,32514249	0	N.A.	0
EFHD1	-0,012206028	-0,100425843	0	N.A.	0
MANEAL	-0,012218435	0,129466042	0	N.A.	0
ZFC3H1	-0,012223768	0,247874988	0	N.A.	0
PIK3CD-AS2	-0,012262319	-0,338665365	0	N.A.	0
HCFC2	-0,012317077	0,334825467	0	N.A.	0
DDX19B	-0,012375107	-0,087632615	0	N.A.	0
RP11-96L14.7	-0,01240293	0,231663617	0	N.A.	0
IL6	-0,012426373	-0,023286072	0	N.A.	0
ERG	-0,012440294	-0,115927138	0	N.A.	0

KBTBD3	-0,012482426	-0,066219669	0	N.A.	0
HOXA2	-0,012486423	-0,09497712	0	N.A.	0
LINC00948	-0,012498945	-0,30603189	0	N.A.	0
CLDN14	-0,012526329	0,034165711	0	N.A.	0
ADORA2B	-0,012534908	0,140837072	0	N.A.	0
PLAC8L1	-0,012565801	0,140795559	0	N.A.	0
RFPL3S	-0,012575195	0,256599365	0	N.A.	0
DHX8	-0,012591574	0,054518353	0	N.A.	0
XYLT2	-0,012593944	0,183837572	0	N.A.	0
GLIDR	-0,012605086	-0,270323656	0	N.A.	0
PDE6G	-0,012622499		0	N.A.	0
SLC39A1	-0,012700241	0,62323366	0	N.A.	0
BAG4	-0,012705851	0,136642692	0	N.A.	0
KLHL2	-0,012725607	0,166921291	0	N.A.	0
LRRC8B	-0,012773723	0,032361044	0	N.A.	0
AC142528.1	-0,012799331	-0,007839244	0	N.A.	0
UFSP1	-0,012840639	-0,053358552	0	N.A.	0
RP11-1000B6	-0,012856361	-0,064836799	0	N.A.	0
RP11-95I16.4	-0,012877034		0	N.A.	0
CCDC122	-0,012897458	0,057886901	0	N.A.	0
RP11-379K17	-0,012901483	-0,189247249	0	N.A.	0
PNISR	-0,012905935	0,170138917	0	N.A.	0
MCPH1	-0,012917944	0,269664901	0	N.A.	0
TUBB2B	-0,012949681	0,259573147	0	N.A.	0
FAM106A	-0,012960301		0	N.A.	0
NCR3LG1	-0,012964575	-0,051221269	0	N.A.	0
CAMP	-0,012967761		0	N.A.	0
RP11-130L8.1	-0,012982585	0,092024492	0	N.A.	0
RP11-368I7.6	-0,012991278	0,02407142	0	N.A.	0
PLGLB1	-0,013052144	0,235675194	0	N.A.	0
RP11-98G7.1	-0,013052632		0	N.A.	0
COX4I2	-0,01305495	-0,031344298	0	N.A.	0
MIA2	-0,013061249	0,228708934	0	N.A.	0

POMT2	-0,013063745	0,101964194	0	N.A.	0
TTC33	-0,013079096	0,011181945	0	N.A.	0
MSX1	-0,013161941	-0,016535301	0	N.A.	0
FAM66D	-0,013176295		0	N.A.	0
BLNK	-0,013183261	-0,106803249	0	N.A.	0
HOXA13	-0,013205428	0,114298196	0	N.A.	0
CATSPERD	-0,013207233	0,286869231	0	N.A.	0
OLAH	-0,013242796		0	N.A.	0
ADAMDEC1	-0,01324398	0,066408498	0	N.A.	0
GFOD1	-0,01324401	0,126180092	0	N.A.	0
SIKE1	-0,013249307	0,071517199	0	N.A.	0
RP11-582J16	-0,013262561	0,245688367	0	N.A.	0
GMFG	-0,013274873	-0,382461965	0	N.A.	0
MDS2	-0,013290321	-0,393250958	0	N.A.	0
C1orf122	-0,013321203	0,343144331	0	N.A.	0
LINC00337	-0,01333244	-0,075620245	0	N.A.	0
TRGV10	-0,013387123		0	N.A.	0
HM13-AS1	-0,013393099	-0,245049769	0	N.A.	0
RP11-426C22	-0,013402065		0	N.A.	0
NDUFB2-AS1	-0,013428256	-0,047887619	0	N.A.	0
NUDT11	-0,01345463	-0,041666698	0	N.A.	0
FUZ	-0,013455878	-0,133661271	0	N.A.	0
WRN	-0,013519615	0,119098256	0	N.A.	0
REEP1	-0,01353088	0,292183342	0	N.A.	0
USP19	-0,013549726	0,301081384	0	N.A.	0
RIMKLB	-0,013580899	-0,079956686	0	N.A.	0
HSD17B1	-0,013590822	-0,143101507	0	N.A.	0
PCAT29	-0,01359919		0	N.A.	0
RP11-54O7.1	-0,013607789		0	N.A.	0
CRYZ	-0,013615144	0,166246396	0	N.A.	0
ZNF565	-0,013635209	-0,095565005	0	N.A.	0
PHACTR4	-0,013677806	0,136965101	0	N.A.	0
HMSD	-0,0136903	-0,092254307	0	N.A.	0

CHD8	-0,013716643	0,086861464	0	N.A.	0
ST8SIA4	-0,013722324	-0,250169038	0	N.A.	0
AC011747.3	-0,013791876	0,285224053	0	N.A.	0
NGFR	-0,013797723		0	N.A.	0
SLC1A2	-0,013810864	0,13567291	0	N.A.	0
PARD6G	-0,013813396	-0,153348924	0	N.A.	0
AC093620.5	-0,013839696	0,440359039	0	N.A.	0
PCDHGA3	-0,013858614	-0,075534426	0	N.A.	0
SP9	-0,013859595		0	N.A.	0
RP11-15E18.	-0,013930352	0,053262862	0	N.A.	0
TRAM2	-0,013947126	0,212449181	0	N.A.	0
KIAA0753	-0,013966238	0,185154636	0	N.A.	0
RP1-10C16.1	-0,0139796	-0,04044213	0	N.A.	0
RP11-572O1	-0,013988201	-0,119254904	0	N.A.	0
LILRB4	-0,013992193	-0,151109869	0	N.A.	0
ZNF318	-0,014023403	0,12430483	0	N.A.	0
C14orf105	-0,014028338	-0,079010884	0	N.A.	0
IPPK	-0,014057881	-0,015778478	0	N.A.	0
RP11-603J24	-0,014070546		0	N.A.	0
LINC00265	-0,014079853	-0,203081768	0	N.A.	0
SHISA2	-0,014139298	0,136511235	0	N.A.	0
B4GALT6	-0,014170111	0,03996466	0	N.A.	0
ANKZF1	-0,014170851	0,239373116	0	N.A.	0
TIGD3	-0,01418543	-0,048894039	0	N.A.	0
C5orf66	-0,014188289	0,168974432	0	N.A.	0
ZNF577	-0,014214132	-0,04125553	0	N.A.	0
MS4A8	-0,014249468	0,103104331	0	N.A.	0
ULBP1	-0,014260184	0,168436081	0	N.A.	0
RP11-944C7.	-0,0142681	-0,264657465	0	N.A.	0
NDST2	-0,014292421	0,138068062	0	N.A.	0
CTD-3010D2	-0,014304878	0,043583768	0	N.A.	0
RTN4RL1	-0,014330657		0	N.A.	0
FAM120B	-0,014335747	0,101774674	0	N.A.	0

RGAG4	-0,014365901		0	N.A.	0
ANAPC2	-0,014379681	-0,006704193	0	N.A.	0
RP11-219A15	-0,014381279	0,211863958	0	N.A.	0
AC000403.4	-0,014466482		0	N.A.	0
FLT3LG	-0,014480794	0,067836486	0	N.A.	0
AC007228.11	-0,01448936	0,126983831	0	N.A.	0
CDH7	-0,014490365	0,045875056	0	N.A.	0
KIFAP3	-0,014535978	0,073742233	0	N.A.	0
RP11-58C22.	-0,014572901		0	N.A.	0
RP11-408A13	-0,014589465	-0,007613536	0	N.A.	0
USP44	-0,014643276	0,049494608	0	N.A.	0
TMEM221	-0,014644624		0	N.A.	0
AC004156.3	-0,014683031	-0,063655608	0	N.A.	0
ZNF252P-AS1	-0,014691973	0,082689298	0	N.A.	0
FSIP1	-0,014692062	0,097348129	0	N.A.	0
CPNE2	-0,014695249	-0,051434003	0	N.A.	0
SGTB	-0,014732348	-0,060451361	0	N.A.	0
XXbac-BPG55	-0,014736727		0	N.A.	0
BZRAP1	-0,014743108	0,023740738	0	N.A.	0
NEURL4	-0,014757335	0,260761605	0	N.A.	0
ABL1	-0,014760063	-0,145049754	0	N.A.	0
NPIPB6	-0,014773709	0,101400219	0	N.A.	0
RP11-296K13	-0,014789036		0	N.A.	0
AC006547.15	-0,014803765		0	N.A.	0
HOXA10-HOX	-0,0148139	0,055215796	0	N.A.	0
RP4-535B20.	-0,014819476	0,191323033	0	N.A.	0
PRODH2	-0,014857846	-0,01298123	0	N.A.	0
FAM124A	-0,014862011	-0,00208848	0	N.A.	0
SPZ1	-0,014866226	-0,034769699	0	N.A.	0
ZCCHC11	-0,014873549	-0,038916626	0	N.A.	0
CACNB1	-0,014905924	-0,030190372	0	N.A.	0
RP11-379F4.9	-0,014933234	-0,13247871	0	N.A.	0
HIST1H3I	-0,014940579	-0,071900501	0	N.A.	0

TLE2	-0,014964741	-0,260159342	0	N.A.	0
DHH	-0,014975367		0	N.A.	0
RP11-115D19	-0,014976646	0,01594531	0	N.A.	0
ANXA5	-0,014990046	-0,03773587	0	N.A.	0
RP11-506H20	-0,015009251	0,009333675	0	N.A.	0
KIAA0196	-0,015013815	-0,011165215	0	N.A.	0
KLHDC2	-0,015039459	0,047951774	0	N.A.	0
ENDOU	-0,015051618	0,012990927	0	N.A.	0
C17orf99	-0,015077191	0,001666266	0	N.A.	0
ST20-MTHFS	-0,015081866	0,079012914	0	N.A.	0
RP11-188P20	-0,015090873		0	N.A.	0
RP11-342K6.	-0,015106966	0,264693109	0	N.A.	0
KCNMB4	-0,015115866	0,350334465	0	N.A.	0
LMAN2L	-0,015160505	0,271012124	0	N.A.	0
RP11-285F16	-0,015165307		0	N.A.	0
WFDC2	-0,015170769	-0,1458914	0	N.A.	0
ACAD11	-0,015200159	0,235802851	0	N.A.	0
PCDHA4	-0,015217468	0,117438775	0	N.A.	0
KAT7	-0,015231485	-0,214069517	0	N.A.	0
CTD-2331H1	-0,015240381	0,08921264	0	N.A.	0
RP11-196G18	-0,015242011	0,039636734	0	N.A.	0
MYCBPAP	-0,015282426		0	N.A.	0
HINT3	-0,015282895	0,203549064	0	N.A.	0
ZEB1-AS1	-0,015298978	0,187122775	0	N.A.	0
SLC25A41	-0,015311842	0,169157984	0	N.A.	0
AGAP2	-0,015323374	-0,330554979	0	N.A.	0
CH17-264L24	-0,015332308		0	N.A.	0
RPTOR	-0,015356456	-0,091280471	0	N.A.	0
FHAD1	-0,015384014	0,061784818	0	N.A.	0
ADSS	-0,01546394	0,208012492	0	N.A.	0
NANOG	-0,015473246	0,01477552	0	N.A.	0
RP11-192H23	-0,015532586	-0,032667423	0	N.A.	0
OLFML1	-0,015537842		0	N.A.	0

MYCL	-0,015628666	-0,296621151	0	N.A.	0
FIG4	-0,015650314	-0,056198593	0	N.A.	0
ZNF426	-0,015781058	-0,228099489	0	N.A.	0
BRSK2	-0,015792228	0,186107575	0	N.A.	0
HNMT	-0,015826769	-0,070636572	0	N.A.	0
SPTBN4	-0,015838257	0,223428194	0	N.A.	0
CAPN6	-0,015871971	-0,078286463	0	N.A.	0
AC011242.6	-0,015878906	-0,034419692	0	N.A.	0
DACT1	-0,015882382	0,023023374	0	N.A.	0
TREM2	-0,015900474	0,093796351	0	N.A.	0
FGFRL1	-0,015950692	0,147690767	0	N.A.	0
WISP3	-0,016054118	0,076621917	0	N.A.	0
MVD	-0,016074261	0,294487388	0	N.A.	0
RP11-342I1.2	-0,016078918	-0,025234084	0	N.A.	0
CASC3	-0,016105185	0,18553316	0	N.A.	0
HAGLR	-0,016130149	-0,040461275	0	N.A.	0
SSC4D	-0,016131297	-0,122357479	0	N.A.	0
RSBN1	-0,016131735	0,095370631	0	N.A.	0
CLDN6	-0,016145751		0	N.A.	0
TRIM41	-0,016169081	-0,205026752	0	N.A.	0
PYCARD-AS1	-0,016170589	0,120221397	0	N.A.	0
GAREML	-0,016181363	-0,212427927	0	N.A.	0
CCDC181	-0,016211156	0,200300704	0	N.A.	0
STYX	-0,016261907	-0,230692133	0	N.A.	0
C12orf4	-0,016265435	-0,021193278	0	N.A.	0
AKR1C1	-0,016271306	0,052657582	0	N.A.	0
GPBAR1	-0,016293391	0,055879383	0	N.A.	0
AK9	-0,016325385	-0,227182585	0	N.A.	0
RP11-66N24.	-0,016333002	0,036499921	0	N.A.	0
MTRNR2L4	-0,016333333	-0,004703053	0	N.A.	0
RP11-22A3.2	-0,016404947	0,250259409	0	N.A.	0
COL3A1	-0,016420334	-0,110831554	0	N.A.	0
MTMR4	-0,016450373	-0,050645869	0	N.A.	0

RUNDC3A-AS	-0,016483712	0,140101286	0	N.A.	0
HEG1	-0,0164928	0,136120671	0	N.A.	0
FLT4	-0,016513594	0,022898799	0	N.A.	0
PPP1R3E	-0,016530629	0,301008054	0	N.A.	0
NEGR1	-0,016543959		0	N.A.	0
RP11-669C19	-0,016580326		0	N.A.	0
RP3-395M20	-0,016585481	0,190838049	0	N.A.	0
ZFP3	-0,016592471	-0,081027798	0	N.A.	0
PIGB	-0,016608122	-0,054419438	0	N.A.	0
USP37	-0,01661476	-0,148405307	0	N.A.	0
RP11-173C1.	-0,016621534	-0,243706005	0	N.A.	0
RUSC2	-0,016648978	-0,003833361	0	N.A.	0
FBXL13	-0,016707322	-0,00863128	1	cl1	0
EPB41	-0,016712537	-0,000447986	0	N.A.	0
LGALS8-AS1	-0,016726654	-0,059534745	0	N.A.	0
RP11-778D9.	-0,016743223	-0,19313276	0	N.A.	0
MCF2L2	-0,016766302	0,265810237	0	N.A.	0
RP11-94I2.4	-0,016766435	0,032182556	0	N.A.	0
RP11-154H23	-0,016788355	0,310933182	0	N.A.	0
SHC3	-0,016808668	-0,029454426	0	N.A.	0
RP11-262H14	-0,016810356	-0,178673096	0	N.A.	0
MMP12	-0,016849332	0,034788371	0	N.A.	0
ZFP90	-0,016876224	0,064490943	0	N.A.	0
ZBTB32	-0,016895268		0	N.A.	0
CTD-2049O4	-0,016897774	-0,207129777	0	N.A.	0
SCN11A	-0,016904454		0	N.A.	0
CMYA5	-0,01691461	0,054335651	0	N.A.	0
KCNE5	-0,016917058		0	N.A.	0
LRRC49	-0,016918274	0,009621985	0	N.A.	0
LINS	-0,016950385	-0,050818542	0	N.A.	0
RP11-96O20.	-0,016975259	-0,07075999	0	N.A.	0
RP11-452F19	-0,016975916	0,060281435	0	N.A.	0
DDIT4L	-0,017011138		0	N.A.	0

MSTN	-0,017036838	0,160872973	0	N.A.	0
RBP1	-0,017128945	0,142729967	0	N.A.	0
SOX7	-0,017149019	-0,023010259	0	N.A.	0
ZNF879	-0,017157075	-0,11781442	0	N.A.	0
SARS	-0,017176207	0,255837968	0	N.A.	0
RP11-363J20	-0,017200389	0,044852133	0	N.A.	0
PHF20L1	-0,017232489	0,257767907	0	N.A.	0
CYS1	-0,017266487	-0,146865157	0	N.A.	0
RAD51AP2	-0,017267693	-0,052465036	0	N.A.	0
AC012074.2	-0,017282724		0	N.A.	0
TBCK	-0,017311759	0,028925807	0	N.A.	0
RP11-848P1.	-0,017341586	0,098115869	0	N.A.	0
MAN1A1	-0,017381446	0,191796092	0	N.A.	0
RP11-588H23	-0,017401895		0	N.A.	0
RP11-293P20	-0,01740567	-0,053724054	0	N.A.	0
RP11-190C22	-0,017423247		0	N.A.	0
SPIN2A	-0,017452541	-0,110512158	0	N.A.	0
CLHC1	-0,017452736	0,229403909	0	N.A.	0
RP11-343H5.	-0,017463449	-0,027102454	0	N.A.	0
ACTG2	-0,017488239	-0,318045556	0	N.A.	0
KLRC1	-0,01750193	0,127658525	0	N.A.	0
SRRM2	-0,017512918	0,455498019	0	N.A.	0
MANEA	-0,017514268	0,14298134	0	N.A.	0
RP11-646I6.5	-0,017524046	-0,085047183	0	N.A.	0
FCGRT	-0,017542348	-0,004624826	0	N.A.	0
MAP6	-0,017588426		0	N.A.	0
AQP1	-0,017630832	-0,190338493	0	N.A.	0
INPP5J	-0,017658294	-0,133118406	0	N.A.	0
GALNT8	-0,01767946	-0,046449132	0	N.A.	0
SCARF2	-0,017693701	0,024403105	0	N.A.	0
RSF1	-0,017706664	0,372753559	0	N.A.	0
RP11-672L10	-0,017712808	0,025484908	0	N.A.	0
TPM3	-0,017735043	-0,067755132	0	N.A.	0

PPP3CB	-0,017745904	-0,121050054	0	N.A.	0
WDR20	-0,017766205	0,015332099	0	N.A.	0
CTBP1	-0,017775184	0,353095232	0	N.A.	0
CTD-2145A24	-0,017812333	-0,007319528	0	N.A.	0
ELL3	-0,017840937	-0,112419225	0	N.A.	0
BOLA2B	-0,017846914	-0,188128897	0	N.A.	0
MYO3B	-0,017852197	0,093824842	0	N.A.	0
SLC22A5	-0,017889042	0,152871611	0	N.A.	0
AC012360.6	-0,017890124		0	N.A.	0
RP11-83J21.3	-0,017904654	0,114495005	0	N.A.	0
CXorf36	-0,017912261	0,026816542	0	N.A.	0
RP11-470P21	-0,017938923	-0,021461349	0	N.A.	0
LIMS2	-0,017962156	-0,172556295	0	N.A.	0
MANBA	-0,017970081	0,262447905	0	N.A.	0
CRAT	-0,018007252	0,040659971	0	N.A.	0
RP11-574F21	-0,018031181	-0,000801007	0	N.A.	0
NAT1	-0,018033322	0,162865783	0	N.A.	0
KCNRG	-0,018039306	0,09203234	0	N.A.	0
SLC4A1	-0,01808999	0,06358389	0	N.A.	0
IGHV4-39	-0,018095107	-0,259427332	0	N.A.	0
RP11-297A16	-0,018118705		0	N.A.	0
C10orf55	-0,018124112		0	N.A.	0
PMCH	-0,018204762		0	N.A.	0
HSPBAP1	-0,018232288	0,215454315	0	N.A.	0
CTD-2001C12	-0,018251785	-0,019183416	0	N.A.	0
OXER1	-0,018252099	-0,026660926	0	N.A.	0
ZNF615	-0,018262618	-0,107807376	0	N.A.	0
ZNF470	-0,018381206	-0,101817164	0	N.A.	0
CD164L2	-0,01840907	-0,123629058	0	N.A.	0
CTC-203F4.2	-0,018469162	-0,145993611	0	N.A.	0
LPPR2	-0,0184807	0,136112554	0	N.A.	0
PNLDC1	-0,018525854	0,264190829	0	N.A.	0
SLC29A3	-0,018542427	0,24833999	0	N.A.	0

PRB1	-0,018576574	0,126120144	0	N.A.	0
RP11-806H1(	-0,018581254	0,053121578	0	N.A.	0
UBE2O	-0,018616796	0,038648533	0	N.A.	0
RP11-435O5.	-0,018622866	-0,033974411	0	N.A.	0
PLA2G15	-0,018638148	0,122499403	0	N.A.	0
SQLE	-0,01864123	0,434911666	0	N.A.	0
PCDHB16	-0,018658317	-0,229137408	0	N.A.	0
LSM12	-0,018693936	-0,132499117	0	N.A.	0
ESPN	-0,018707759	-0,049096005	0	N.A.	0
XCL1	-0,018709122	0,085978502	0	N.A.	0
FLJ13224	-0,018715963	0,120057473	0	N.A.	0
FAM129C	-0,018742592	-0,136012552	0	N.A.	0
ZNF354B	-0,018760523	-0,108757768	0	N.A.	0
ZNF223	-0,018805145	0,014014629	0	N.A.	0
BBIP1	-0,01882407	-0,056029118	0	N.A.	0
LRRC25	-0,018830417		0	N.A.	0
ZNF582	-0,018835234		0	N.A.	0
RSPH9	-0,018898409	0,033154308	0	N.A.	0
CD74	-0,01894483	-0,40316035	0	N.A.	0
DCDC2	-0,018951692	-0,153913468	0	N.A.	0
KHDRBS3	-0,018968951	0,03991442	0	N.A.	0
VCPKMT	-0,018973613	-0,11528089	0	N.A.	0
AFAP1L1	-0,018975417		0	N.A.	0
RP11-730K11	-0,019016274	0,195489296	0	N.A.	0
RP11-403P17	-0,019025337	-0,16655498	0	N.A.	0
CDCP2	-0,019032815		0	N.A.	0
PIGO	-0,019098927	0,27564957	0	N.A.	0
FAM46B	-0,019110102	0,134176093	0	N.A.	0
CTD-3088G3.	-0,019121566	0,045973901	0	N.A.	0
PRLR	-0,019174586	0,033390903	0	N.A.	0
TERC	-0,019243934	-0,07773891	0	N.A.	0
ZNF581	-0,019268758	-0,0048543	0	N.A.	0
LGALS4	-0,019361682	0,046451313	0	N.A.	0

NANOS1	-0,01936374	0,034351128	0	N.A.	0
PIAS2	-0,019365209	-0,088363442	0	N.A.	0
LIFR	-0,019382341	0,194611799	0	N.A.	0
ANO10	-0,019396071	-0,055436748	0	N.A.	0
HIST2H3D	-0,01942091	0,058940154	0	N.A.	0
C10orf128	-0,019426945	-0,283534921	0	N.A.	0
LMF1	-0,019466664	0,338039731	0	N.A.	0
HCG15	-0,019471802	-0,043726642	0	N.A.	0
RP11-727F15	-0,019489491	0,119432594	0	N.A.	0
SCG3	-0,019546729	-0,120898451	0	N.A.	0
FRMD4A	-0,019555342	-0,097179567	0	N.A.	0
DNAJC27	-0,019581008	-0,032699337	0	N.A.	0
ACY3	-0,01961139	-0,178557825	0	N.A.	0
RP5-967N21.	-0,019611764	-0,072448303	0	N.A.	0
RP11-47L3.1	-0,019624297	-0,06844733	0	N.A.	0
RP5-1073O3.	-0,019624297		0	N.A.	0
LINC00944	-0,019665235	0,203144604	0	N.A.	0
RP11-380D23	-0,019705385	-0,144466459	0	N.A.	0
KCNG3	-0,019706793		0	N.A.	0
RP11-1112C1	-0,019728693	0,2878873	0	N.A.	0
ZNF410	-0,019736915	-0,000625238	0	N.A.	0
GPM6B	-0,01974735	0,022757886	0	N.A.	0
ADAMTS8	-0,019770469		0	N.A.	0
RP11-336K24	-0,019773836	0,068779859	0	N.A.	0
MESTIT1	-0,019793659	-0,027692683	0	N.A.	0
CARD16	-0,019845961	-0,168130814	0	N.A.	0
CHMP3	-0,019847276	0,256324943	0	N.A.	0
HPCAL1	-0,019848857	0,178608308	0	N.A.	0
NEK9	-0,019867001	0,265812177	0	N.A.	0
REEP6	-0,019881051	0,264980114	0	N.A.	0
AHCTF1	-0,019881716	0,035876296	0	N.A.	0
PNKP	-0,019888757	0,041053024	0	N.A.	0
LRP2BP	-0,019959472	-0,003086988	0	N.A.	0

RP11-410N8.	-0,019981348		0	N.A.	0
IGHM	-0,019982646	-0,073395501	0	N.A.	0
CLK4	-0,020000291	0,185937843	0	N.A.	0
AC138969.4	-0,02002092	0,080666553	0	N.A.	0
TIGD6	-0,020029111	-0,02694023	0	N.A.	0
HP1BP3	-0,020038605	0,070376143	0	N.A.	0
SNAP25	-0,020045532		0	N.A.	0
HCG9	-0,020056955	0,187576631	0	N.A.	0
STXBP5-AS1	-0,020078332	0,353446737	0	N.A.	0
RP11-7O11.3	-0,02009732	-0,087726259	0	N.A.	0
RAET1E-AS1	-0,020148011	0,204475242	0	N.A.	0
MLLT1	-0,020186559	0,024067954	0	N.A.	0
ABCC2	-0,020193371	0,129388485	0	N.A.	0
ROR2	-0,020224147	0,052518082	0	N.A.	0
PARVA	-0,020229496	-0,113057803	0	N.A.	0
C1QL4	-0,020235447		0	N.A.	0
FSTL4	-0,020271574	-0,072075395	0	N.A.	0
TLCD1	-0,020272425	0,182780266	0	N.A.	0
ACVR2B	-0,020289942	0,124135564	0	N.A.	0
ZNF484	-0,020293068	-0,106633046	0	N.A.	0
TMEM42	-0,020313623	-0,0511508	0	N.A.	0
RP11-649A18	-0,020328042		0	N.A.	0
KLRC2	-0,020344816		0	N.A.	0
RP1-153G14.	-0,020351838	0,096287134	0	N.A.	0
ZNF175	-0,020450897	0,176194511	0	N.A.	0
RP11-98D18.	-0,020507537		0	N.A.	0
RP11-296I10	-0,020533085	0,141217245	0	N.A.	0
AC005618.6	-0,020551909	0,284080932	0	N.A.	0
AC006116.17	-0,020593985	0,001788808	0	N.A.	0
NRXN2	-0,020598718	0,4106549	0	N.A.	0
FMNL1	-0,020611672	-0,165045921	0	N.A.	0
FAM114A2	-0,020612312	0,083583631	0	N.A.	0
TBX2	-0,020617053		0	N.A.	0

RP11-506O24	-0,020673901		0	N.A.	0
NLGN1	-0,020680171		0	N.A.	0
C9orf171	-0,020736386		0	N.A.	0
CCDC136	-0,020739688	-0,051933323	0	N.A.	0
RP11-930P14	-0,020745573	-0,052485943	0	N.A.	0
CCDC113	-0,020778123	-0,178320359	0	N.A.	0
TPST2	-0,020820778	0,235091629	0	N.A.	0
TPSAB1	-0,020851591	-0,128487982	0	N.A.	0
NUDT17	-0,020879186	-0,066522265	0	N.A.	0
LINC01144	-0,020882559	-0,049720071	0	N.A.	0
TNFRSF25	-0,020886345	0,31669777	0	N.A.	0
UBE2D3	-0,020905792	-0,182992082	0	N.A.	0
SOX8	-0,020919625	0,033167698	0	N.A.	0
STOML3	-0,020922579	-0,001748178	0	N.A.	0
C9orf50	-0,020960748		0	N.A.	0
SNTN	-0,020975559	-0,307683085	0	N.A.	0
URB2	-0,020978806	-0,167222377	0	N.A.	0
CTD-2516F10	-0,021008552		0	N.A.	0
SLC4A2	-0,021086659	0,227748797	0	N.A.	0
DNAL1	-0,021093637	-0,011932199	0	N.A.	0
RP6-91H8.3	-0,021129515		0	N.A.	0
MFHAS1	-0,021160374	0,253047096	0	N.A.	0
COL11A1	-0,021191227	-0,161964078	0	N.A.	0
TMEM8B	-0,021238559	0,071539122	0	N.A.	0
AGA	-0,021238879	-0,234942465	0	N.A.	0
RP4-655J12.4	-0,021239763	0,187531127	0	N.A.	0
PARP11	-0,021243959	0,098883014	0	N.A.	0
EIF3J-AS1	-0,021268788	-0,053289307	0	N.A.	0
LINC00870	-0,021304464		0	N.A.	0
C10orf91	-0,021331841	0,113246448	0	N.A.	0
KCNQ1	-0,021357218	0,114539956	0	N.A.	0
ZNF629	-0,02137676	-0,033280102	0	N.A.	0
NSD1	-0,0214061	0,171555842	0	N.A.	0

SDSL	-0,021414209	-0,208053063	0	N.A.	0
NIPSNAP3B	-0,021429998		0	N.A.	0
TTL	-0,021450398	0,376027831	0	N.A.	0
CRTC3-AS1	-0,021455903	-0,148377162	0	N.A.	0
RP5-821D11.	-0,021472554	0,006939964	0	N.A.	0
RP11-415C15	-0,021475156		0	N.A.	0
AC074289.1	-0,021495003	0,230315758	0	N.A.	0
CCDC12	-0,021496371	-0,129034003	0	N.A.	0
ADRA2C	-0,021509575	0,002983667	0	N.A.	0
COLQ	-0,021535752		0	N.A.	0
CLIP1-AS1	-0,021542462	0,196959151	0	N.A.	0
FAAH2	-0,021547588	0,187561736	0	N.A.	0
ASB16	-0,021595497	-0,067390666	0	N.A.	0
NOP9	-0,021598501	-0,059990169	0	N.A.	0
ZNF433	-0,021602548	0,135902587	0	N.A.	0
RP11-1020A1	-0,021613733		0	N.A.	0
UBE2F	-0,021620894	0,219356414	0	N.A.	0
UBAP1L	-0,021665012	0,078352409	0	N.A.	0
SIGLEC10	-0,021692481	-0,07410037	0	N.A.	0
E4F1	-0,021733114	0,181634798	0	N.A.	0
AC020571.3	-0,021737964	0,140347743	0	N.A.	0
RP5-864K19.	-0,02176749	0,08138658	0	N.A.	0
LINC01344	-0,021816895		0	N.A.	0
GPR20	-0,021827973	0,14117977	0	N.A.	0
CECR1	-0,021848261	-0,145637746	0	N.A.	0
RP11-131N1	-0,021876505		0	N.A.	0
RP11-84G21.	-0,021878109	-0,045234224	0	N.A.	0
PIAS4	-0,021891209	-0,076006125	0	N.A.	0
MOCS1	-0,021929554	0,081196452	0	N.A.	0
PI16	-0,021947993		0	N.A.	0
YPEL4	-0,02196756	0,063246976	0	N.A.	0
CTC-523E23.	-0,022071132	-0,240439153	0	N.A.	0
AC079354.5	-0,022085664	0,297961466	0	N.A.	0

RP11-358N2.	-0,022127143	-0,012781477	0	N.A.	0
PBX3	-0,022175019	-0,020448921	0	N.A.	0
ZNF408	-0,022181023	-0,139555898	0	N.A.	0
GZMK	-0,022190555	-0,157934345	0	N.A.	0
ZMYND11	-0,022228534	-0,166314097	0	N.A.	0
C22orf29	-0,022286259	-0,096880023	0	N.A.	0
AC006129.2	-0,022301654	-0,248912471	0	N.A.	0
EEF1A2	-0,022307787	-0,02164579	0	N.A.	0
AP4B1	-0,022317128	-0,230844965	0	N.A.	0
HEYL	-0,02233345	0,152098045	0	N.A.	0
SPACA4	-0,022340554	0,135199055	0	N.A.	0
CNEP1R1	-0,022404763	-0,233271027	0	N.A.	0
NUTM2A-AS1	-0,022438837	-0,140696697	0	N.A.	0
ZUFSP	-0,022484245	-0,124553152	0	N.A.	0
LL0XNC01-23	-0,022499498	0,063027311	0	N.A.	0
FAM166A	-0,022509461		0	N.A.	0
ZNF700	-0,022523737	-0,05197372	0	N.A.	0
ARAF	-0,022557364	0,126480903	0	N.A.	0
RP11-44N11.	-0,022557661	0,03964064	0	N.A.	0
SLC10A7	-0,022581339	-0,122843258	0	N.A.	0
SLC12A5	-0,022613535	-0,019793728	0	N.A.	0
AF213884.2	-0,022615544	-0,036603453	0	N.A.	0
KB-1732A1.1	-0,022650636	0,016848726	0	N.A.	0
AIDA	-0,022663289	-0,056936466	0	N.A.	0
ZBED3-AS1	-0,022689644	0,050472017	0	N.A.	0
RP5-1098D14	-0,022818172		0	N.A.	0
RP11-338K17	-0,022832825		0	N.A.	0
DBF4B	-0,02284441	-0,021937617	0	N.A.	0
RP11-737O24	-0,022888927	0,330064619	0	N.A.	0
LCE1E	-0,022907259	-0,226429061	0	N.A.	0
RGS7	-0,022918143		0	N.A.	0
RP5-1116H23	-0,022953987		0	N.A.	0
RP11-27N21.	-0,022959654	0,25474024	0	N.A.	0

SCAF4	-0,02297243	0,149217704	0	N.A.	0
FEV	-0,022976915		0	N.A.	0
RP11-131L12	-0,022982623	0,071215763	0	N.A.	0
DRP2	-0,023017563		0	N.A.	0
CTD-2292P10	-0,023045665		0	N.A.	0
C17orf70	-0,023045979	0,092046027	0	N.A.	0
CYP2U1	-0,023047824	0,034117293	0	N.A.	0
RP11-554E23	-0,023059022		0	N.A.	0
PLXNB3	-0,023077481	0,353294547	0	N.A.	0
RP1-193H18.	-0,023087028	0,102926754	0	N.A.	0
DMRTA2	-0,023093185	-0,076140422	0	N.A.	0
P4HTM	-0,023099134	0,314106653	0	N.A.	0
MEF2C	-0,023134908	-0,258890732	0	N.A.	0
IGHV1-24	-0,023169673		0	N.A.	0
CDH13	-0,023179884	-0,157402119	0	N.A.	0
RP11-575A19	-0,023179887	0,084424151	0	N.A.	0
RP11-297P16	-0,023190766	0,174723608	0	N.A.	0
ITGA2B	-0,023200541		0	N.A.	0
EMC7	-0,023204316	0,432082585	0	N.A.	0
BST1	-0,023248972	-0,019183488	0	N.A.	0
SLC25A27	-0,023250778	-0,113827971	0	N.A.	0
PGM1	-0,023253142	0,054239785	0	N.A.	0
RP11-550A5.	-0,023258244	0,143776263	0	N.A.	0
PTPDC1	-0,023284888	-0,090738898	0	N.A.	0
COL9A3	-0,023293803	-0,00817089	0	N.A.	0
CTB-113P19.	-0,023328701	-0,298732054	0	N.A.	0
RP11-713M1	-0,023341515	0,173358712	0	N.A.	0
ST18	-0,023358918	0,06564083	0	N.A.	0
LINC00471	-0,023402218	-0,060015842	0	N.A.	0
L3MBTL3	-0,023407801	0,039275309	0	N.A.	0
TSPAN32	-0,023458139	-0,135697672	0	N.A.	0
SCN1B	-0,023478098	0,027783657	0	N.A.	0
PDPR	-0,023480974	0,14553157	0	N.A.	0

RP11-603J24	-0,023483722		0	N.A.	0
RP11-1C8.5	-0,023486643	-0,122068253	0	N.A.	0
RNU11	-0,023520745	0,006507364	0	N.A.	0
AF001548.5	-0,023524882	-0,283736995	0	N.A.	0
BTBD11	-0,023559097	0,229652892	0	N.A.	0
TOMM20L	-0,023563385	0,051190873	0	N.A.	0
AC084809.3	-0,023572059	0,06358727	0	N.A.	0
TRMT1L	-0,023589554	-0,11121703	0	N.A.	0
GARNL3	-0,02362409	0,112929154	0	N.A.	0
CCSER2	-0,023627417	0,075832064	0	N.A.	0
INVS	-0,023634	-0,134521238	0	N.A.	0
CHRNA3	-0,023646662		0	N.A.	0
MIR497HG	-0,023657267	-0,010023148	0	N.A.	0
LBP	-0,023673596	-0,148035039	0	N.A.	0
COPB2	-0,023762062	0,104036837	0	N.A.	0
LA16c-395F1	-0,023767259	-0,097889543	0	N.A.	0
RP11-276E15	-0,02378788	0,108529325	0	N.A.	0
ZNF256	-0,023788864	-0,246323688	0	N.A.	0
ALG10	-0,02379486	-0,029342102	0	N.A.	0
SLC17A7	-0,023808169		0	N.A.	0
PAN3	-0,023808687	0,069075249	0	N.A.	0
CPXM2	-0,023857819	-0,214156296	0	N.A.	0
GALNT12	-0,023881045	0,350391305	0	N.A.	0
OR51E1	-0,023891468	-0,085298554	0	N.A.	0
CDR1	-0,023945387	-0,020098892	0	N.A.	0
GOLGA6L22	-0,02396402	-0,148035039	0	N.A.	0
RP11-615I2.2	-0,024019937	0,116701133	0	N.A.	0
RP3-402G11.	-0,024029305	-0,118430005	0	N.A.	0
AC000068.5	-0,024078525	0,137404292	0	N.A.	0
VIM-AS1	-0,024091018	0,129621917	0	N.A.	0
CTD-2012K14	-0,024101231	-0,168122293	0	N.A.	0
AC092580.4	-0,024112537	0,077863219	0	N.A.	0
AC007163.3	-0,02412489		0	N.A.	0

RP11-65J3.14	-0,02413179	-0,085823028	0	N.A.	0
PCMTD2	-0,024145653	-0,135333992	0	N.A.	0
MSS51	-0,024149571	0,144702638	0	N.A.	0
GBX2	-0,024177515		0	N.A.	0
AC025171.1	-0,024177866	-0,191851707	0	N.A.	0
HOXC8	-0,024186485	0,079515914	0	N.A.	0
TTC36	-0,024202669	-0,132076887	0	N.A.	0
RBM43	-0,024205151	-0,357306748	0	N.A.	0
SCAMP1-AS1	-0,024225752	-0,056927793	0	N.A.	0
SFRP1	-0,024232851		0	N.A.	0
NHLRC1	-0,02427018	-0,358526753	0	N.A.	0
ACAA1	-0,024275904	0,30647259	0	N.A.	0
CFAP57	-0,024290199		0	N.A.	0
DESI1	-0,024304393	0,154277453	0	N.A.	0
PLD6	-0,024339285	-0,096726806	0	N.A.	0
KIAA1024	-0,024411334	0,289251024	0	N.A.	0
MATN1-AS1	-0,024424852	-0,070121354	0	N.A.	0
AKAP3	-0,024426893	0,091524878	0	N.A.	0
CDAN1	-0,024430558	-0,202869309	0	N.A.	0
ZNF503-AS1	-0,02443237	-0,060913302	0	N.A.	0
CTB-129P6.1	-0,024458027		0	N.A.	0
VAMP1	-0,024461166	-0,060867231	0	N.A.	0
RP11-105C19	-0,024486464		0	N.A.	0
PIGS	-0,024497888	0,383726797	0	N.A.	0
RP11-867G23	-0,024498514	-0,348136208	0	N.A.	0
GALNS	-0,024544196	0,18242678	0	N.A.	0
NEUROD1	-0,024555112		0	N.A.	0
TERT	-0,024595511	0,062096426	0	N.A.	0
RP3-337D23.	-0,024622549		0	N.A.	0
RASSF4	-0,024637126	-0,155113751	0	N.A.	0
CCP110	-0,024657032	0,354787677	0	N.A.	0
SEC61A2	-0,024677139	-0,017497888	0	N.A.	0
UCN	-0,024689897	-0,165973761	0	N.A.	0

RP11-110I1.1	-0,024711289	0,256972614	0	N.A.	0
TUBE1	-0,024719317	-0,002811535	0	N.A.	0
FAM127C	-0,024725526	-0,111863147	0	N.A.	0
RP11-705O1.	-0,024738089	0,1625939	0	N.A.	0
TOP3A	-0,024744816	0,118319558	0	N.A.	0
PCDHGB6	-0,024754483	0,042909635	0	N.A.	0
MSL2	-0,02479404	-0,058487332	0	N.A.	0
ARID4B	-0,024836941	0,38760809	0	N.A.	0
CTD-2095E4.	-0,024850668	0,296302236	0	N.A.	0
PEX11A	-0,02485308	-0,177565298	0	N.A.	0
LINC00243	-0,024860521		0	N.A.	0
PPP1R12A	-0,024866908	0,337137842	0	N.A.	0
MAK	-0,024870963	-0,18152977	0	N.A.	0
LMAN1	-0,024894197	-0,272354403	0	N.A.	0
RNF169	-0,024909341	0,007429394	0	N.A.	0
DSTYK	-0,024928146	0,134536691	0	N.A.	0
TAT-AS1	-0,024939585	-0,24592771	0	N.A.	0
ZNF7	-0,02494522	0,068504499	0	N.A.	0
LINC01431	-0,024954823	0,112691577	0	N.A.	0
IGKV2-30	-0,024975599	-0,238373313	0	N.A.	0
MEIG1	-0,024978593	-0,139582944	0	N.A.	0
AC068282.3	-0,025020344	0,068566601	0	N.A.	0
TNNI3K	-0,025044346	-0,010422763	0	N.A.	0
CD14	-0,025049127	-0,298264478	0	N.A.	0
RASGRP1	-0,025050338	0,032673208	0	N.A.	0
AGBL5-AS1	-0,025070786	-0,268890694	0	N.A.	0
SCAI	-0,025086783	-0,263343524	0	N.A.	0
GPR4	-0,025095185	0,061371692	0	N.A.	0
CHST8	-0,025129938		0	N.A.	0
RP11-476H16	-0,025168978	-0,02411316	0	N.A.	0
ANKRD31	-0,025247275	-0,076259698	0	N.A.	0
IGHG1	-0,025299338	-0,18157295	0	N.A.	0
GSTM2	-0,025363583	-0,058781245	0	N.A.	0

AL133493.2	-0,025382491	0,1474486	0	N.A.	0
DES	-0,02538675	-0,265310667	0	N.A.	0
LINC01060	-0,025391633		0	N.A.	0
RP11-121M2	-0,025400535	0,001841037	0	N.A.	0
RNF41	-0,025436122	-0,141241993	0	N.A.	0
NFE2L2	-0,02543865	0,264604082	0	N.A.	0
HK1	-0,025482295	0,030321832	0	N.A.	0
CELF2	-0,025483297	-0,21117593	0	N.A.	0
CD300LF	-0,025498306		0	N.A.	0
TIMM23B	-0,025506152	0,156067377	0	N.A.	0
CTD-2033D1!	-0,025561296		0	N.A.	0
LINC00654	-0,025564981	-0,205104534	0	N.A.	0
HDAC10	-0,025597296	0,282538747	0	N.A.	0
PAPOLG	-0,025636114	-0,195984854	0	N.A.	0
RP11-571M6	-0,025665829	0,122998174	0	N.A.	0
LRBA	-0,025681033	0,028146006	0	N.A.	0
MTSS1	-0,02568104	-0,052508472	0	N.A.	0
TRAPPC11	-0,025681417	-0,143006643	0	N.A.	0
RP11-692C24	-0,025683669	0,061163592	0	N.A.	0
ALK	-0,025702868		0	N.A.	0
C5orf17	-0,025736415	-0,198635496	0	N.A.	0
ZNF423	-0,025739365	0,137249692	0	N.A.	0
ITK	-0,025760488	-0,145309899	0	N.A.	0
ADCK5	-0,025808823	0,271069598	0	N.A.	0
KNG1	-0,025819345	0,306565026	0	N.A.	0
SCAPER	-0,02582364	0,065821442	0	N.A.	0
RP1-191J18.6	-0,025826553	0,075045364	0	N.A.	0
TCF12	-0,025832859	0,075004095	0	N.A.	0
GAL3ST4	-0,025876774	0,042455087	0	N.A.	0
RP5-1024G6.	-0,025896316	-0,02786001	0	N.A.	0
SNX15	-0,025928905	0,311795886	0	N.A.	0
CD48	-0,025979728	0,004590085	0	N.A.	0
RP11-317N8.	-0,025991887	-0,050086333	0	N.A.	0

SCAF8	-0,025993179	0,235611726	0	N.A.	0
FAM71D	-0,026009037	0,117173021	0	N.A.	0
USP51	-0,026027411		0	N.A.	0
SLC25A21-AS	-0,026035449	-0,149852494	0	N.A.	0
SRR	-0,026051814	0,085369869	0	N.A.	0
LRTM1	-0,026052079	-0,060508802	0	N.A.	0
IGKV3-20	-0,02605883	0,057074542	0	N.A.	0
PALMD	-0,026070182	-0,138529922	0	N.A.	0
POLR3A	-0,026099358	-0,051788991	0	N.A.	0
FBXW8	-0,026111161	-0,142298157	0	N.A.	0
CTD-2571L23	-0,026129488	0,063615991	0	N.A.	0
NTSR1	-0,026216086	0,129331383	0	N.A.	0
ANKAR	-0,026248817	0,125805999	0	N.A.	0
RP11-401P9.	-0,02625441	-0,036169022	0	N.A.	0
FAM13B	-0,026305647	-0,064341649	0	N.A.	0
GFI1	-0,02631271	-0,11547557	0	N.A.	0
SDC2	-0,026317088	-0,048366343	0	N.A.	0
ZHX1-C8orf7	-0,026344911	0,115801102	0	N.A.	0
CIRBP-AS1	-0,02636523	0,168469674	0	N.A.	0
IGHV5-51	-0,026380012	-0,010054131	0	N.A.	0
MCM3AP-AS	-0,026386255	0,033090773	0	N.A.	0
ISX	-0,026397466	0,235639339	0	N.A.	0
NCKAP5L	-0,026415508	0,188237591	0	N.A.	0
UBALD1	-0,026420397	-0,212060153	0	N.A.	0
NMD3	-0,02644252	0,048114925	0	N.A.	0
C20orf85	-0,026466353		0	N.A.	0
CP	-0,026495007	0,063069044	0	N.A.	0
SGSM1	-0,026501002	0,003796614	0	N.A.	0
TTPAL	-0,026547265	0,132559657	0	N.A.	0
ELFN1	-0,026556389	0,12877596	0	N.A.	0
PTPRT	-0,026579465		0	N.A.	0
LINGO1	-0,02658331		1	cls3	0
AC005740.6	-0,02660954		0	N.A.	0

SRBD1	-0,026675381	0,057580929	0	N.A.	0
ARHGAP4	-0,026676888	-0,197684298	0	N.A.	0
ST3GAL4-AS1	-0,026679007	-0,19968602	0	N.A.	0
SLC26A2	-0,026746473	-0,102871538	0	N.A.	0
RAMP3	-0,026766279	-0,196619632	0	N.A.	0
RP4-694B14.	-0,026784349	0,227959848	0	N.A.	0
ZNF571-AS1	-0,026796431	-0,081774836	0	N.A.	0
CTD-3065J16	-0,026815775	-0,134603734	0	N.A.	0
TMEM33	-0,026829884	0,272703052	0	N.A.	0
TMOD2	-0,02684273	0,222155848	0	N.A.	0
ARHGAP44	-0,026846744	-0,203443004	0	N.A.	0
ZSCAN30	-0,026860193	-0,126311546	0	N.A.	0
ZNF614	-0,026876493	-0,073928711	0	N.A.	0
RP11-538D16	-0,026885746	0,230498637	0	N.A.	0
KDM4D	-0,026908249	0,08096787	0	N.A.	0
RP11-417E7.	-0,026936088		0	N.A.	0
SIN3A	-0,026960122	0,145421843	0	N.A.	0
CTU1	-0,027002712	-0,06728702	0	N.A.	0
RP11-110I1.6	-0,027073536	0,078946464	0	N.A.	0
SAG	-0,027079086	-0,06999344	0	N.A.	0
TREH	-0,02708597	-0,029818564	0	N.A.	0
AKAP6	-0,027090562	0,129928422	0	N.A.	0
CACUL1	-0,02710578	0,367538534	0	N.A.	0
SLAIN1	-0,027106976	-0,203754428	0	N.A.	0
CLCNKA	-0,027120579		0	N.A.	0
RCOR2	-0,027218813	0,13038712	0	N.A.	0
SLC22A11	-0,027251111	0,148585237	0	N.A.	0
CDK5R2	-0,027290207		0	N.A.	0
GNAI3	-0,027308826	-0,130649777	0	N.A.	0
OBP2A	-0,027314795	-0,117176989	0	N.A.	0
OXT	-0,02734124		0	N.A.	0
PNMT	-0,027363758	-0,201096232	0	N.A.	0
ZNF836	-0,0273672	-0,077476125	0	N.A.	0

RP11-375I20	-0,027381285	0,213011784	0	N.A.	0
SCARB1	-0,027398484	0,250734721	0	N.A.	0
LINC00645	-0,027416088		0	N.A.	0
FPR1	-0,027419823	0,262926395	0	N.A.	0
RP11-413H22	-0,027425745	0,014722007	0	N.A.	0
RP11-166O4.	-0,027436729	0,175520919	0	N.A.	0
RMRP	-0,027469797	0,205666483	0	N.A.	0
RP11-283G6.	-0,027489202	0,001047933	0	N.A.	0
CTD-2154B17	-0,027496992		0	N.A.	0
C21orf62-AS1	-0,027511308	-0,127848327	0	N.A.	0
SV2C	-0,027513909	0,17181073	0	N.A.	0
ZNF783	-0,02751958	0,027012846	0	N.A.	0
OPRL1	-0,027529047		0	N.A.	0
C2CD4B	-0,027539048	0,12056533	0	N.A.	0
TMEM260	-0,027588135	-0,136582444	0	N.A.	0
RP11-53I6.4	-0,027630292		0	N.A.	0
HBB	-0,027652302	-0,065937247	0	N.A.	0
SSR4	-0,027712436	-0,011650976	0	N.A.	0
ABHD12B	-0,027719931	-0,19626522	0	N.A.	0
RP11-420N3.	-0,027744052	0,066264963	0	N.A.	0
CFAP43	-0,027767664	-0,151354849	0	N.A.	0
BEST2	-0,027806756	-0,13187	0	N.A.	0
CYB5B	-0,027808269	-0,142576759	0	N.A.	0
GATA2	-0,027826605	0,089695589	0	N.A.	0
IRAK4	-0,027842105	0,005955524	0	N.A.	0
ATP2C1	-0,02786704	0,449784855	0	N.A.	0
CAMK2B	-0,027902703	0,071152823	0	N.A.	0
RP5-855D21.	-0,027941848	0,174951697	0	N.A.	0
ZNF721	-0,027980744	0,028479871	0	N.A.	0
SCRG1	-0,027985289	0,009959805	0	N.A.	0
BICC1	-0,027990791	-0,206640195	0	N.A.	0
RNU12	-0,028002848	0,161507238	0	N.A.	0
NKG7	-0,02804625	-0,166293439	0	N.A.	0

ATP6V1G2	-0,028064295		0	N.A.	0
CUL9	-0,028074414	-0,081913241	0	N.A.	0
RP13-314C10	-0,028084154		0	N.A.	0
CCDC176	-0,028131363	0,005789893	0	N.A.	0
IGHG4	-0,028151511	-0,082987637	0	N.A.	0
AP5M1	-0,028157842	-0,281738375	0	N.A.	0
RP11-182J1.5	-0,028166854		0	N.A.	0
MLLT3	-0,028172421	-0,02482421	0	N.A.	0
GABPA	-0,028212264	-0,1212866	0	N.A.	0
RP11-20I20.4	-0,028249784		0	N.A.	0
SYTL1	-0,028336515	0,158314419	0	N.A.	0
CARM1	-0,028373782	0,2452412	0	N.A.	0
RP11-63L7.5	-0,028418778	-0,185261353	0	N.A.	0
FXYD6	-0,028433904	0,16374623	0	N.A.	0
EML3	-0,028520585	0,071339502	0	N.A.	0
CCDC108	-0,028541334	-0,090161199	0	N.A.	0
BGN	-0,028543424	-0,201953494	0	N.A.	0
MIR4458HG	-0,028567147	-0,131641616	0	N.A.	0
FAM227A	-0,028586073	0,127960743	0	N.A.	0
SOX6	-0,028606159	0,210957144	0	N.A.	0
UPP2	-0,028650259		0	N.A.	0
RP11-850A17	-0,028758862	0,063108067	0	N.A.	0
KLF15	-0,028783014		0	N.A.	0
LA16c-390E6	-0,028783772	0,155216815	0	N.A.	0
AL023806.1	-0,028794125		0	N.A.	0
SPAST	-0,028795824	0,159661775	0	N.A.	0
DNAJC25	-0,028824287	-0,059489339	0	N.A.	0
C2CD4C	-0,028831313	0,029278737	0	N.A.	0
CCDC88C	-0,028895671	0,013735676	0	N.A.	0
HOXC6	-0,02890672	-0,10985658	0	N.A.	0
PI4KA	-0,028925023	0,174163199	0	N.A.	0
TAC1	-0,028981283	-0,086037874	0	N.A.	0
RP4-673M15	-0,028990742	-0,002876792	0	N.A.	0

VANGL2	-0,029004472	0,12622593	0	N.A.	0
CDK8	-0,029004928	0,321397933	0	N.A.	0
CTB-179K24.	-0,029070651	-0,299152488	0	N.A.	0
TMEM252	-0,029108716	-0,163544892	0	N.A.	0
RP11-325F22	-0,029141053		0	N.A.	0
RP11-275I14	-0,029141164	-0,1317812	0	N.A.	0
RP4-710M16	-0,029148778	0,354619229	0	N.A.	0
SLC6A19	-0,02916133		0	N.A.	0
SATB1	-0,029180554	0,226652458	0	N.A.	0
LINC00992	-0,029256893	-0,11280199	0	N.A.	0
RP11-18H21.	-0,029320287	0,035018591	0	N.A.	0
CORO7	-0,029320553	0,145059158	0	N.A.	0
RP11-154H23	-0,02938877	0,298409036	0	N.A.	0
RP4-610C12.	-0,029420231	-0,019425044	0	N.A.	0
CPB2-AS1	-0,029421552		0	N.A.	0
BSN	-0,029443279		0	N.A.	0
ZNF559	-0,029487008	-0,229412006	0	N.A.	0
DDHD1	-0,029487296	0,110919089	0	N.A.	0
P3H1	-0,029495127	0,107657672	0	N.A.	0
FAM183A	-0,029496717		0	N.A.	0
TRMT13	-0,029551948	-0,1194257	0	N.A.	0
GZMA	-0,029555661	-0,190813515	0	N.A.	0
RP11-696N14	-0,029561808	-0,105374064	0	N.A.	0
CTD-2574D22	-0,029563227	0,12790772	0	N.A.	0
AP001439.2	-0,029611655	0,296893427	0	N.A.	0
SLFN11	-0,029624927	0,064906472	0	N.A.	0
TRIM35	-0,029626192	-0,028067315	0	N.A.	0
AC005775.2	-0,02969926	0,040569641	0	N.A.	0
CRYBB3	-0,029713558	0,234390152	0	N.A.	0
RP11-223C24	-0,029716112	-0,325996825	0	N.A.	0
INO80	-0,029746847	0,070186545	0	N.A.	0
RP11-793H13	-0,029797248	0,047884273	0	N.A.	0
RP11-217B7.	-0,029848204		0	N.A.	0

DSCR9	-0,029857483	-0,1029559	0	N.A.	0
CCL26	-0,029864177	0,229687808	0	N.A.	0
RP5-1033H22	-0,029911673	-0,15210927	0	N.A.	0
LBX2-AS1	-0,029926361	-0,002818782	0	N.A.	0
AC011747.4	-0,02993136	-0,044233708	0	N.A.	0
ARPC5	-0,029957742	-0,123067312	0	N.A.	0
C12orf42	-0,029986838	0,210756405	0	N.A.	0
TGIF1	-0,030012851	0,271302817	0	N.A.	0
CYP17A1	-0,030017357	-0,027339268	0	N.A.	0
ADAMTS1	-0,030039133	0,135114787	0	N.A.	0
AGO1	-0,030051057	-0,160222671	0	N.A.	0
SEC31B	-0,030076705	0,154917478	0	N.A.	0
AK1	-0,030084826	0,134931066	0	N.A.	0
RP11-700H6.	-0,030136317	0,396794056	0	N.A.	0
ZNF436	-0,030155065	0,059286956	0	N.A.	0
PLAC9	-0,030162772	-0,022353255	0	N.A.	0
CTC-246B18.	-0,030181402	-0,105459018	0	N.A.	0
CTD-2184C24	-0,030236626	0,227685785	0	N.A.	0
RP11-328K4.	-0,0302407	-0,170066307	0	N.A.	0
RP11-337C18	-0,03027589	-0,100814835	0	N.A.	0
RP11-375N15	-0,030278319		0	N.A.	0
ALG1L2	-0,030298677	-0,080504426	0	N.A.	0
GXYLT2	-0,030332812	-0,02926139	0	N.A.	0
TSPYL5	-0,030384738		0	N.A.	0
FAM65B	-0,030430464	-0,074821232	0	N.A.	0
ZNF563	-0,030480008	-0,151639225	0	N.A.	0
FFAR4	-0,030506781	0,217262544	0	N.A.	0
PBRM1	-0,030517926	-0,173098142	0	N.A.	0
IER3	-0,030535342	0,087456085	0	N.A.	0
RP11-983P16	-0,030535621	-0,036929545	0	N.A.	0
RP11-677M1	-0,030563225	-0,014579664	0	N.A.	0
TGFB3	-0,03057733	0,020213711	0	N.A.	0
RP11-314N13	-0,030595812	-0,055779656	0	N.A.	0

RAB39A	-0,030614859		0	N.A.	0
RP11-255E6.6	-0,030638347	0,017476409	0	N.A.	0
RP11-252A24	-0,030674807	0,040523549	0	N.A.	0
C2CD3	-0,030678886	0,224549115	0	N.A.	0
RNF217-AS1	-0,030748809		0	N.A.	0
ADAMTS7	-0,030787465	-0,254212092	0	N.A.	0
RP11-109J4.1	-0,030810671	-0,126682947	0	N.A.	0
NUDT18	-0,030824867	-0,144821064	0	N.A.	0
CCER2	-0,030829726	0,142040118	0	N.A.	0
BRF1	-0,03083	0,239680066	0	N.A.	0
TESPA1	-0,030840871	-0,231364012	0	N.A.	0
C22orf42	-0,030877555		0	N.A.	0
RP11-788H18	-0,030877555		0	N.A.	0
HOMEZ	-0,03090413	0,200548137	0	N.A.	0
RABL2B	-0,031052851	-0,056900557	0	N.A.	0
RP11-380O24	-0,031075496	-0,150552393	0	N.A.	0
ZNF492	-0,031077672	-0,003484453	0	N.A.	0
RP3-465N24.	-0,031082374	-0,161225295	0	N.A.	0
RET	-0,031097416	0,072916664	0	N.A.	0
TNFRSF13B	-0,031101757	0,00498682	0	N.A.	0
RP11-630C16	-0,031116996		0	N.A.	0
RP11-359E3.	-0,031118915	0,024807426	0	N.A.	0
B3GNT6	-0,031124217	-0,088969101	0	N.A.	0
CTB-61M7.2	-0,031133121		0	N.A.	0
RP11-295I5.3	-0,031139045	0,207161779	0	N.A.	0
HIST1H4I	-0,03116762	-0,172389987	0	N.A.	0
RP11-61L19.2	-0,031182576	0,296229747	0	N.A.	0
RP11-727A23	-0,031198985	0,252252069	0	N.A.	0
TIGD2	-0,031220518	-0,046468517	0	N.A.	0
DAND5	-0,031223893	-0,13863491	0	N.A.	0
RP11-78A19.	-0,031246201	0,256972614	0	N.A.	0
XPO7	-0,031265806	-0,015644314	0	N.A.	0
SCG2	-0,031273537	0,179240031	0	N.A.	0

ZNF131	-0,031278583	-0,13401467	0	N.A.	0
CLEC2A	-0,031283087		0	N.A.	0
SCN3A	-0,031302199		0	N.A.	0
ENPP3	-0,03131119	-0,141060938	0	N.A.	0
DHX38	-0,031398478	0,104292954	0	N.A.	0
TTLL11	-0,031460565	0,037478814	0	N.A.	0
ANGPTL6	-0,031469363	0,326280281	0	N.A.	0
AC005037.3	-0,031487109	0,014902765	0	N.A.	0
FAM181B	-0,03150576	-0,049490728	0	N.A.	0
RP11-1151B1	-0,031507917		0	N.A.	0
SENP6	-0,031515702	0,249750152	0	N.A.	0
CTD-2033A1(	-0,031525731	-0,114487542	0	N.A.	0
C16orf46	-0,031540723	-0,375313348	0	N.A.	0
SLC44A3	-0,031594639	0,220956569	0	N.A.	0
C6orf25	-0,031601375	-0,201758205	0	N.A.	0
MSRA	-0,031615869	0,187679787	0	N.A.	0
NAT16	-0,031636993		0	N.A.	0
NUS1	-0,0316445	-0,192569561	0	N.A.	0
PIBF1	-0,031651337	0,220382348	0	N.A.	0
TMEM110-M	-0,031660124	0,194611799	0	N.A.	0
RP11-307C12	-0,031671325	0,209251114	0	N.A.	0
AC025335.1	-0,031701845	-0,041224654	0	N.A.	0
VEPH1	-0,031733722	-0,087798575	0	N.A.	0
TRAPPC12	-0,031735219	0,12088637	0	N.A.	0
CCDC110	-0,031757748	-0,133270621	0	N.A.	0
PIGN	-0,031774625	-0,107881997	0	N.A.	0
RPRML	-0,031784288		0	N.A.	0
ATP6V1B1	-0,031794578	0,000920475	0	N.A.	0
RP1-12G14.7	-0,031808325	0,111156675	0	N.A.	0
RP11-156E8.	-0,031812703	0,124985517	0	N.A.	0
EYA3	-0,03184054	0,095971975	0	N.A.	0
C1orf101	-0,031841988	-0,2034256	0	N.A.	0
LINC00449	-0,031873957	-0,063682449	0	N.A.	0

RNF125	-0,031895286	0,031367123	0	N.A.	0
GPR65	-0,031905234	-0,242489795	0	N.A.	0
LINC01136	-0,031906846		0	N.A.	0
ENGASE	-0,031930547	0,058579132	0	N.A.	0
RWDD3	-0,0319868	-0,025853661	0	N.A.	0
FAM66C	-0,031996407	-0,017298946	0	N.A.	0
SEC63	-0,032008378	0,258279176	0	N.A.	0
RP11-21M24	-0,032023568		0	N.A.	0
TRMT10B	-0,032023777	-0,070524802	0	N.A.	0
GABPB2	-0,032048205	0,273088127	0	N.A.	0
RP11-370A5.	-0,03206686	0,070154621	0	N.A.	0
ANK1	-0,032089567	0,118676684	0	N.A.	0
RP11-650K20	-0,032157426	0,139423592	0	N.A.	0
FAM78B	-0,032178125	-0,137633309	0	N.A.	0
AL589743.1	-0,032182524	0,023470822	0	N.A.	0
SETD8	-0,032191363	0,014077575	0	N.A.	0
FAXC	-0,032192918		0	N.A.	0
TPD52L2	-0,032193324	0,11842099	0	N.A.	0
VPS16	-0,032196127	-0,119797754	0	N.A.	0
AKR1C4	-0,032218437	-0,015117364	0	N.A.	0
EFTUD1	-0,032219463	0,067594289	0	N.A.	0
ATRIP	-0,032251905	-0,035789732	0	N.A.	0
CTA-221G9.1	-0,032259405	0,032302575	0	N.A.	0
RP11-72M17	-0,032292407	-0,011169117	0	N.A.	0
CD247	-0,032304722	-0,113100556	0	N.A.	0
GNGT1	-0,032349651	-0,251812192	0	N.A.	0
RP11-830F9.	-0,032392307	-0,068388127	0	N.A.	0
ARMC7	-0,032402961	0,193060929	0	N.A.	0
SCD5	-0,0324067	-0,285225418	0	N.A.	0
SP4	-0,032447125	-0,066116191	0	N.A.	0
ADAMTS6	-0,032452284	-0,005591738	0	N.A.	0
TP53BP1	-0,032491527	-0,008043801	0	N.A.	0
NXPE3	-0,032499217	0,120959959	0	N.A.	0

GIF	-0,032538033	-0,172627553	0	N.A.	0
SOX5	-0,032559654	-0,031996085	0	N.A.	0
RP1-120G22.	-0,032572867	-0,13381446	0	N.A.	0
CLNK	-0,032583584	-0,06342262	0	N.A.	0
D2HGDH	-0,032584686	0,174980403	0	N.A.	0
TMEM170B	-0,032585476	-0,255171671	0	N.A.	0
C9orf156	-0,032593065	0,011908956	0	N.A.	0
LINC01355	-0,032594429	0,129631618	0	N.A.	0
RP11-161M6	-0,032623018	-0,134272379	0	N.A.	0
RP11-127L20	-0,032741954		0	N.A.	0
DRAXIN	-0,032822747	0,092621571	0	N.A.	0
BACE1	-0,032847178	0,090356121	0	N.A.	0
DLK2	-0,032847481		0	N.A.	0
STIM2	-0,032878263	0,29285862	0	N.A.	0
RP11-778D9.	-0,032883264		0	N.A.	0
ULK3	-0,032886637	0,206030633	0	N.A.	0
ETAA1	-0,032889659	0,086498187	0	N.A.	0
TLE6	-0,032973765	-0,135488531	0	N.A.	0
MT1HL1	-0,032974859	0,048455864	0	N.A.	0
ZNF710	-0,032977303	-0,107012365	0	N.A.	0
IRX3	-0,032978698	-0,117408321	0	N.A.	0
MIIP	-0,03300311	-0,046728422	0	N.A.	0
PADI3	-0,033020356	-0,123786359	0	N.A.	0
CPNE4	-0,033029172		0	N.A.	0
VPS33B	-0,033049912	0,169692692	0	N.A.	0
H3F3C	-0,033062825	-0,052976047	0	N.A.	0
LEPROTL1	-0,033066664	0,028789218	0	N.A.	0
DDX43	-0,033067981	-0,050262379	0	N.A.	0
FGD2	-0,033072512	-0,027667342	0	N.A.	0
GLCE	-0,033073698	0,052130717	0	N.A.	0
FAM19A2	-0,033086745	-0,011162959	0	N.A.	0
PLA1A	-0,033111743	0,178447336	0	N.A.	0
MAGEH1	-0,033119475	-0,112021906	0	N.A.	0

GGT5	-0,033121325	0,137370961	0	N.A.	0
CTD-2325M2	-0,03314583	0,123269884	0	N.A.	0
RP11-173P15	-0,033157761		0	N.A.	0
RP11-159H10	-0,033177504	-0,098072801	0	N.A.	0
AP000769.7	-0,033178981		0	N.A.	0
SLC16A1	-0,03318932	0,236088743	0	N.A.	0
GLYR1	-0,033224181	0,010316345	0	N.A.	0
AC019330.1	-0,033232126	-0,145996287	0	N.A.	0
NXPE2	-0,033237102	-0,217621141	0	N.A.	0
MYLK4	-0,033240422	-0,055790914	0	N.A.	0
MYBL1	-0,033290183	-0,097754217	0	N.A.	0
N4BP2L1	-0,033330589	0,081311998	0	N.A.	0
HECW2	-0,033420038	0,129289636	0	N.A.	0
PLCG2	-0,033444445	0,157132706	0	N.A.	0
FAM49B	-0,033449116	-0,137806954	0	N.A.	0
RP11-46A10.	-0,033462413	0,13657281	0	N.A.	0
ZNF652	-0,033487399	0,126225433	0	N.A.	0
RNF180	-0,033496014	-0,33128097	0	N.A.	0
SCAMP4	-0,033582825	0,433340421	0	N.A.	0
ADAM32	-0,033586887	0,234773514	0	N.A.	0
LINC00867	-0,033623271	-0,126319003	0	N.A.	0
RP11-186B7.	-0,033638775	0,07765737	0	N.A.	0
BMX	-0,033666329	-0,180362209	0	N.A.	0
CRLF1	-0,033682931	-0,251637519	0	N.A.	0
TSTD2	-0,033741341	-0,226108982	0	N.A.	0
ZC3HAV1L	-0,033763693	-0,033992501	0	N.A.	0
ZNF354A	-0,033779253	-0,067324243	0	N.A.	0
HIST1H2AM	-0,033818426	-0,129124918	0	N.A.	0
CREBZF	-0,033852986	0,280385487	0	N.A.	0
RP11-169E6.	-0,033856211	0,087978673	0	N.A.	0
SULT1C4	-0,03385832		0	N.A.	0
SHROOM2	-0,033868461	0,451012069	0	N.A.	0
FAM215B	-0,033900751	0,396011709	0	N.A.	0

CTC-338M12	-0,033903665	-0,247794304	0	N.A.	0
ZNF234	-0,033908335	-0,020281958	0	N.A.	0
PLXDC2	-0,033970385	-0,035262481	0	N.A.	0
RP11-10A14.	-0,034021648	-0,03820528	0	N.A.	0
RP11-669E14	-0,034022668		0	N.A.	0
ABI3	-0,034079185	-0,288342468	0	N.A.	0
ZNF720	-0,034149089	0,032703528	0	N.A.	0
TAF3	-0,034163313	0,312553433	0	N.A.	0
SZRD1	-0,034179224	-0,109544369	0	N.A.	0
SPAG1	-0,034194363	0,111764096	0	N.A.	0
RP11-293A21	-0,034217299	-0,178670769	0	N.A.	0
C2CD2	-0,034234776	0,370202116	0	N.A.	0
SEPW1	-0,034252814	-0,157276416	0	N.A.	0
DLX4	-0,034272505	0,025481153	0	N.A.	0
REM1	-0,034305156	-0,045242009	0	N.A.	0
PCDHGA11	-0,034324213	0,237196145	0	N.A.	0
EID2B	-0,034362444	0,023693665	0	N.A.	0
USP27X	-0,034370872	-0,00691661	0	N.A.	0
RBBP9	-0,034392917	-0,483909576	0	N.A.	0
IGLV2-11	-0,034400158		0	N.A.	0
DLG2	-0,034419423	0,040089478	0	N.A.	0
GAB3	-0,034425542		0	N.A.	0
A1CF	-0,034431362	-0,139740669	0	N.A.	0
MIR3179-3	-0,034476784		0	N.A.	0
IGHV1-18	-0,034485617	-0,076928157	0	N.A.	0
AC034243.1	-0,034492811	-0,129702665	0	N.A.	0
SEC62	-0,034541694	0,392462283	0	N.A.	0
RP11-517P14	-0,034566899	-0,060567842	0	N.A.	0
COL10A1	-0,034593433	-0,122924012	0	N.A.	0
RP11-293M1	-0,034620049	0,339619263	0	N.A.	0
ZNF574	-0,034625882	-0,198189743	0	N.A.	0
RP11-661A12	-0,034649783		0	N.A.	0
NR1H3	-0,03465978	-0,039986296	0	N.A.	0

RP11-66N24.	-0,034666826	-0,022372618	0	N.A.	0
LCTL	-0,034671349		0	N.A.	0
CTD-2547L16	-0,03468335	-0,174104521	0	N.A.	0
CTD-2192J16	-0,034707268	-0,04116748	0	N.A.	0
SALRNA2	-0,03474665		0	N.A.	0
PARD6G-AS1	-0,034758566	0,112279953	0	N.A.	0
CTC-537E7.3	-0,034779739	-0,112197979	0	N.A.	0
RP11-44F14.	-0,034801874	-0,169553923	0	N.A.	0
APOBEC3H	-0,034823077		0	N.A.	0
BMPER	-0,034829041	-0,025639342	0	N.A.	0
PRSS57	-0,034843526	0,301119163	0	N.A.	0
PRSS21	-0,03487604		0	N.A.	0
ZBED6CL	-0,034900243	-0,12947942	0	N.A.	0
PHF7	-0,034900819	-0,041691252	0	N.A.	0
PAK4	-0,035013959	0,417341471	0	N.A.	0
GJC3	-0,035036892	-0,044795229	0	N.A.	0
GPR173	-0,035049071		0	N.A.	0
RP11-521B24	-0,035051029	-0,083106326	0	N.A.	0
ZFHX4	-0,035071099	-0,049736412	0	N.A.	0
CTC-1337H24	-0,035082156	0,195925627	0	N.A.	0
ZNF623	-0,035085169	0,272041827	0	N.A.	0
KRT75	-0,035104725		0	N.A.	0
PREX1	-0,035114901	-0,059091081	0	N.A.	0
RP11-11N7.4	-0,035169267		0	N.A.	0
GAS6-AS2	-0,035212196		0	N.A.	0
KIAA1377	-0,035234543	-0,187945161	0	N.A.	0
MPZ	-0,035289237	-0,016120961	0	N.A.	0
ARHGAP25	-0,035319746	0,131102131	0	N.A.	0
BMPR1A	-0,035381221	-0,018799736	0	N.A.	0
CARD8	-0,035435488	-0,169502689	0	N.A.	0
AP001059.6	-0,035452274	0,124845311	0	N.A.	0
PNOC	-0,035472004	0,026852192	0	N.A.	0
KRT40	-0,035472367	0,239466157	0	N.A.	0

SERPINF1	-0,035491977	0,10769203	0	N.A.	0
CCDC149	-0,035494957	0,067889491	0	N.A.	0
RP11-549B18	-0,035504957	0,121615522	0	N.A.	0
SLC15A2	-0,035563458	0,116598576	0	N.A.	0
C16orf93	-0,035572201	-0,222145471	0	N.A.	0
LINC01270	-0,035590958		0	N.A.	0
RASSF8	-0,035641302		0	N.A.	0
TUSC8	-0,035663639	-0,152233575	0	N.A.	0
SLC25A35	-0,035677879	-0,060096638	0	N.A.	0
MTTP	-0,035751949	0,041233447	0	N.A.	0
DNAH17	-0,035775658		0	N.A.	0
MS4A2	-0,035836291	-0,015278661	0	N.A.	0
RP11-22A3.1	-0,035880547		0	N.A.	0
SLC44A5	-0,035951617	0,096370189	0	N.A.	0
RIBC1	-0,03600111	0,023563368	0	N.A.	0
AGAP6	-0,036071887	-0,032636962	0	N.A.	0
TMEM156	-0,036081654	-0,159771983	0	N.A.	0
TUBGCP6	-0,036114621	0,248349736	0	N.A.	0
CD52	-0,036116699	-0,320876369	0	N.A.	0
FAM69B	-0,036126088	0,131522796	0	N.A.	0
RP11-326C3.	-0,036136581	0,102239856	0	N.A.	0
Z69720.2	-0,036162061		0	N.A.	0
NECAB3	-0,036172956	0,028204857	0	N.A.	0
AC008753.6	-0,036198234		0	N.A.	0
LINC01187	-0,036220104		0	N.A.	0
GTF2E1	-0,036279488	-0,074996316	0	N.A.	0
FAM188B	-0,03634504	-0,122864119	0	N.A.	0
NPM2	-0,036354885	-0,229464824	0	N.A.	0
C9orf153	-0,036362077		0	N.A.	0
LINC01526	-0,036377561	0,087249701	0	N.A.	0
RP11-280O1.	-0,036387318		0	N.A.	0
RP11-802D6.	-0,036405315		0	N.A.	0
TPSG1	-0,036421992	0,221390333	0	N.A.	0

HIST1H2BO	-0,03643152	-0,155626886	0	N.A.	0
ARMCX6	-0,036440696	0,035304632	0	N.A.	0
KLC2	-0,036489495	0,118823695	0	N.A.	0
ZNF487	-0,036511998	0,010614545	0	N.A.	0
TP73	-0,036538998	-0,261471904	0	N.A.	0
CTD-2313J17	-0,036541188	-0,130425705	0	N.A.	0
CCDC82	-0,036550542	0,281931961	0	N.A.	0
HSPA6	-0,036552846	0,22474659	0	N.A.	0
OGN	-0,036564732	-0,157365624	0	N.A.	0
RP11-266L9.!	-0,036567853		0	N.A.	0
RP11-69I8.3	-0,036579973	-0,084866524	0	N.A.	0
RP11-273B2(	-0,036585477	-0,13644575	0	N.A.	0
JUN	-0,036602083	0,123079747	0	N.A.	0
THAP2	-0,036602837	-0,105673692	0	N.A.	0
GOSR1	-0,036638574	0,006924987	0	N.A.	0
RP11-4B16.4	-0,036681151		0	N.A.	0
CH507-9B2.5	-0,036682725	-0,001687268	0	N.A.	0
RIN1	-0,036692804	0,269007313	1	cl2	0
C19orf54	-0,036705168	-0,11989964	0	N.A.	0
TGM4	-0,036727236		0	N.A.	0
CPA2	-0,036738678	0,185207027	0	N.A.	0
IBA57	-0,036739572	-0,214685097	0	N.A.	0
CTB-119C2.1	-0,036749854	0,121358327	0	N.A.	0
ROBO3	-0,036761042	-0,064683189	0	N.A.	0
SLC25A30-AS	-0,036778415	-0,067260036	0	N.A.	0
ZNF14	-0,036800068	0,126402915	0	N.A.	0
ZNF445	-0,036818779	0,172184132	0	N.A.	0
GUCY1A2	-0,036830162		0	N.A.	0
ANKRD33B	-0,036885421	0,082367129	0	N.A.	0
RP11-817O1	-0,036906946	0,068071323	0	N.A.	0
KRBOX4	-0,036910602	0,070033418	0	N.A.	0
ZG16	-0,036925711	0,098333165	0	N.A.	0
FAM107A	-0,036956381	0,050472017	0	N.A.	0

SCOC-AS1	-0,037057638	-0,256608625	0	N.A.	0
BARX1	-0,037080914		0	N.A.	0
CTC-527H23.	-0,037085298	0,122021729	0	N.A.	0
RP11-70C1.3	-0,037142972	0,228428406	0	N.A.	0
CLSTN2	-0,037148877		1	none	0
FBXL4	-0,037154389	0,298373082	0	N.A.	0
IGHA1	-0,037208498	-0,001243119	0	N.A.	0
ENPEP	-0,037217897	0,120111753	0	N.A.	0
RGS19	-0,037218938	-0,122541554	0	N.A.	0
NUDT13	-0,037311832	-0,031610427	0	N.A.	0
PSMA3-AS1	-0,037400585	-0,167740916	0	N.A.	0
LINC01410	-0,03743969		0	N.A.	0
RABL3	-0,037459997	0,110714353	0	N.A.	0
RP11-499E18	-0,037479373	-0,258654466	0	N.A.	0
SEMA4C	-0,037517008	-0,001918035	0	N.A.	0
AC009410.1	-0,037526489	-0,172157542	0	N.A.	0
ATRN	-0,037572743	0,064379492	0	N.A.	0
RCOR3	-0,037586258	0,055511145	0	N.A.	0
RP11-540O1	-0,037608466	-0,128244842	0	N.A.	0
SLC17A9	-0,037634982	0,010171974	0	N.A.	0
RP11-54G14.	-0,037659553	0,237816232	0	N.A.	0
METTL16	-0,037689009	-0,249205571	0	N.A.	0
MORF4L2-AS	-0,037700524	-0,204359881	0	N.A.	0
CAPN10-AS1	-0,037717177	0,022131458	0	N.A.	0
LPCAT1	-0,037724858	0,227025097	0	N.A.	0
DPF2	-0,037739192	0,298522673	0	N.A.	0
TMEM68	-0,037744018	-0,134122552	0	N.A.	0
CSDE1	-0,037757949	-0,329016626	0	N.A.	0
PON3	-0,037834069	-0,049115677	0	N.A.	0
CD36	-0,037838023	-0,116589318	0	N.A.	0
C11orf95	-0,037843043	0,071370476	0	N.A.	0
UBE3B	-0,037870136	0,046133238	0	N.A.	0
APOL3	-0,037895956	-0,121342527	0	N.A.	0

CTDSPL2	-0,037914494	-0,061967489	0	N.A.	0
CRMP1	-0,037966103		0	N.A.	0
CTD-3193O1	-0,037985256		0	N.A.	0
PGM5P2	-0,037986284	0,273402094	0	N.A.	0
RP11-849F2.!	-0,038022545	0,173639896	0	N.A.	0
STK36	-0,038032321	-0,011729472	0	N.A.	0
MADCAM1	-0,038074562	0,277051101	0	N.A.	0
SLC38A9	-0,038080588	0,057242335	0	N.A.	0
ALMS1	-0,038104265	0,285381743	0	N.A.	0
C2orf27B	-0,03814521	-0,050374402	0	N.A.	0
NARFL	-0,038251409	-0,0710757	0	N.A.	0
ESM1	-0,038318187	0,119907963	0	N.A.	0
PDPK1	-0,038319835	0,353686686	0	N.A.	0
MORN3	-0,03833648	0,061023144	0	N.A.	0
DUSP15	-0,038411102	-0,066709359	0	N.A.	0
ZNF649	-0,038419145	-0,333079693	0	N.A.	0
RP11-1348G1	-0,038444632	0,108382866	0	N.A.	0
TRIM14	-0,038463799	0,137699486	0	N.A.	0
CDK2AP1	-0,038474396	-0,110579974	0	N.A.	0
DAGLA	-0,038491313	0,012189855	0	N.A.	0
ROBO2	-0,038495451	-0,137498824	0	N.A.	0
PIGM	-0,038529719	0,253975971	0	N.A.	0
BAALC	-0,038562936	-0,257169163	0	N.A.	0
CTD-2314G2	-0,038570078	0,185801021	0	N.A.	0
LINC01089	-0,038619078	-0,047701182	0	N.A.	0
SLC12A9	-0,038637541	0,203128517	0	N.A.	0
NRROS	-0,038663205	0,144148133	0	N.A.	0
SLC22A17	-0,03876921		0	N.A.	0
CTB-58E17.3	-0,038770583	-0,036187082	0	N.A.	0
DHRS12	-0,038776982	0,175228379	0	N.A.	0
AF129408.17	-0,03878329		0	N.A.	0
GNG2	-0,038793617	-0,021051064	0	N.A.	0
WAPAL	-0,038840407	-0,030161309	0	N.A.	0

APBA2	-0,038856314	0,18617038	0	N.A.	0
SERPINB6	-0,038870642	0,323236594	0	N.A.	0
TSPEAR-AS2	-0,038870925	0,023588336	0	N.A.	0
RP11-89K11.	-0,038953729	-0,080170565	0	N.A.	0
C11orf54	-0,038976778	0,085907016	0	N.A.	0
GFRA1	-0,038988729		0	N.A.	0
GCK	-0,039013578		0	N.A.	0
RP11-936I5.1	-0,039057162		0	N.A.	0
AF196972.9	-0,039140917	0,010295143	0	N.A.	0
CMKLR1	-0,039203944		0	N.A.	0
CTC-241F20.4	-0,03921092		0	N.A.	0
PCDHB2	-0,039222253	0,000891557	0	N.A.	0
PSMD10	-0,039294902	-0,108238813	0	N.A.	0
C17orf98	-0,039295607		0	N.A.	0
CEND1	-0,039307439	0,007950892	0	N.A.	0
PPP2R3C	-0,039383402	-0,114188927	0	N.A.	0
SNRNP27	-0,039454125	0,017319061	0	N.A.	0
ATG16L2	-0,039457297	-0,090984816	0	N.A.	0
RP3-333H23.	-0,039472063	0,021417558	0	N.A.	0
GGACT	-0,03950469	0,136470456	0	N.A.	0
RP11-356I2.4	-0,039505013	-0,028856414	0	N.A.	0
ZMYM3	-0,039538147	-0,225018507	0	N.A.	0
SH3YL1	-0,039553145	0,182203103	0	N.A.	0
RP11-755B10	-0,039563441		0	N.A.	0
RP11-234B24	-0,039563496	-0,040230383	0	N.A.	0
FTO	-0,03957966	-0,128941536	0	N.A.	0
DZIP1	-0,03958281	-0,058854415	0	N.A.	0
COMP	-0,039631816	0,209377737	0	N.A.	0
SEC24B-AS1	-0,039636109	-0,151298781	0	N.A.	0
F5	-0,039643105	-0,132761015	0	N.A.	0
IGHMBP2	-0,039643578	0,115831316	0	N.A.	0
CTB-50L17.10	-0,039670581	-0,087859525	0	N.A.	0
FCGBP	-0,039672563	0,260177058	0	N.A.	0

INTS5	-0,039691993	-0,222347084	0	N.A.	0
WBP1	-0,039698354	-0,07673184	0	N.A.	0
OSM	-0,039717989	0,031803448	0	N.A.	0
KLHL42	-0,039724689	0,118305933	0	N.A.	0
RP3-337O18.	-0,039737696	-0,264570892	0	N.A.	0
SMN2	-0,039750349	0,090537373	0	N.A.	0
CARD9	-0,039754455	-0,141802955	0	N.A.	0
GNG13	-0,039763025	-0,174411512	0	N.A.	0
BCL11B	-0,039791645	-0,133517117	0	N.A.	0
SERPINA4	-0,03984342	0,113323091	0	N.A.	0
CTB-152G17.	-0,039848653	0,134639429	0	N.A.	0
CTD-2116N2(	-0,039852448	-0,104266961	0	N.A.	0
ZNF263	-0,039873001	0,066764741	0	N.A.	0
MAPK15	-0,03992487	-0,202051282	0	N.A.	0
RP11-518L10	-0,039950742	0,035440467	0	N.A.	0
ZNF586	-0,040006139	-0,205897811	0	N.A.	0
SOX17	-0,040013284	-0,188389274	0	N.A.	0
TG	-0,040053129	-0,00646542	0	N.A.	0
CTSK	-0,040065677	0,055848739	0	N.A.	0
POLR3GL	-0,040070573	0,079528311	0	N.A.	0
PRKG1	-0,040182557	-0,210214325	0	N.A.	0
FBP2	-0,040248038	0,158153434	0	N.A.	0
C10orf67	-0,040272977	-0,085145186	0	N.A.	0
CTB-25B13.9	-0,040301014	0,083283446	0	N.A.	0
TEKT5	-0,040309393	-0,052744057	0	N.A.	0
GTF3C1	-0,040310942	-0,028741867	0	N.A.	0
CCL15	-0,040378883	0,024833251	0	N.A.	0
XPOT	-0,040407365	-0,009119347	0	N.A.	0
KLHDC7B	-0,040453394	-0,170903855	0	N.A.	0
KDM4A	-0,040472919	-0,027997915	0	N.A.	0
RP11-350G8.	-0,040497759		0	N.A.	0
RP11-381K2(	-0,040499734	-0,386517281	0	N.A.	0
EPHB4	-0,0405345	0,147080456	0	N.A.	0

PKP4	-0,040591637	0,122129934	0	N.A.	0
AC090587.5	-0,040598556	-0,07366167	0	N.A.	0
CXCL3	-0,040601603	0,426846184	0	N.A.	0
CHKB	-0,040615579	0,255012127	0	N.A.	0
RP11-561B11	-0,040626784		0	N.A.	0
CHN1	-0,040639739	-0,04122818	0	N.A.	0
CTDSPL	-0,040689681	0,089174765	0	N.A.	0
KCNV1	-0,04071207	-0,11338108	0	N.A.	0
AC007950.2	-0,040715944	-0,033561886	0	N.A.	0
SYCP2	-0,04072902	0,005819786	0	N.A.	0
VIPAS39	-0,040735384	0,018809661	0	N.A.	0
AC005363.11	-0,040769297	-0,02981579	0	N.A.	0
RP11-250B2.	-0,040786377	0,027362727	0	N.A.	0
ZRSR1	-0,040877974		0	N.A.	0
PLA2G4F	-0,040888221	0,015422932	0	N.A.	0
GRAP	-0,040948783	-0,037041979	0	N.A.	0
RP11-620J15	-0,040983617		0	N.A.	0
AC093585.6	-0,040995185	0,105843724	0	N.A.	0
ACSL1	-0,041002915	0,038864887	0	N.A.	0
CALY	-0,041027374		0	N.A.	0
C1orf95	-0,041055339	0,282532256	0	N.A.	0
HOXD13	-0,041064225	0,061089958	0	N.A.	0
AC005944.2	-0,041079594	-0,12364569	0	N.A.	0
FYB	-0,041090656	-0,18955687	0	N.A.	0
ZNF182	-0,041125881	0,029526252	0	N.A.	0
PRSS45	-0,041137903		0	N.A.	0
RP11-250B2.	-0,041195648	0,329926764	0	N.A.	0
PTAR1	-0,04128093	0,262156712	0	N.A.	0
SH3GL2	-0,041294533		0	N.A.	0
GPR83	-0,041294606	0,119833141	0	N.A.	0
CIB4	-0,041341198	0,063261305	0	N.A.	0
SERPINF2	-0,041343759	-0,242579506	0	N.A.	0
VAV1	-0,041344731	-0,093191595	0	N.A.	0

PCAT2	-0,041359528	0,042314878	0	N.A.	0
DACT3-AS1	-0,041375595		0	N.A.	0
POU6F2-AS1	-0,04138256	0,026113746	0	N.A.	0
ALDH6A1	-0,041387925	-0,071134563	0	N.A.	0
FIGN	-0,041402439	0,264023925	0	N.A.	0
FGF14-AS2	-0,041423418	-0,05245725	0	N.A.	0
AC009473.1	-0,041485524		0	N.A.	0
RP11-111M2	-0,0414903	-0,124771925	0	N.A.	0
GLOD5	-0,041511624	-0,127452649	0	N.A.	0
CLCN2	-0,041555336	0,224291375	0	N.A.	0
CTD-2012K14	-0,041615078		0	N.A.	0
MB	-0,041618568	0,162958145	0	N.A.	0
HCG11	-0,041629758	-0,143361879	0	N.A.	0
CTD-2081C10	-0,041679241		0	N.A.	0
UBLCP1	-0,041708719	-0,312549122	0	N.A.	0
PCDHB12	-0,041754766		0	N.A.	0
THSD1	-0,041770196	0,020835158	0	N.A.	0
GPAM	-0,041796534	0,009976026	0	N.A.	0
CAPN9	-0,041796555	0,176828944	0	N.A.	0
MEF2BNB-M	-0,041846803	0,252094388	0	N.A.	0
UCP3	-0,041859054		0	N.A.	0
RP11-27M9.1	-0,041870645		0	N.A.	0
ERLIN2	-0,041874137	0,269653655	0	N.A.	0
TMX3	-0,041919442	0,380721363	0	N.A.	0
FBXO7	-0,041967853	-0,041029548	0	N.A.	0
C17orf51	-0,042004355	0,047135114	0	N.A.	0
RP11-3P17.4	-0,042005812	-0,073445965	0	N.A.	0
ZFYVE21	-0,042065263	0,203822235	0	N.A.	0
CABP7	-0,042099676		0	N.A.	0
AF127936.7	-0,0421494	0,296578473	0	N.A.	0
PCAT1	-0,042171382	-0,139981951	0	N.A.	0
SLCO5A1	-0,042212525	0,266395567	0	N.A.	0
ARSG	-0,042259962	-0,269174012	0	N.A.	0

RP11-211N8.	-0,042330976	-0,024863376	0	N.A.	0
EIF3CL	-0,042336575	-0,217712692	0	N.A.	0
RP11-876N24	-0,042381265		0	N.A.	0
TAF4B	-0,042392584	-0,204272232	0	N.A.	0
SLC16A12	-0,042428665		0	N.A.	0
COX7A1	-0,042434386	-0,173003597	0	N.A.	0
GAS5-AS1	-0,042459308	0,131606674	0	N.A.	0
ERMAP	-0,042521307	-0,094771722	0	N.A.	0
RIC8A	-0,042535926	0,023699533	0	N.A.	0
CTB-96E2.7	-0,042564066		0	N.A.	0
PAF1	-0,042601915	0,466593977	0	N.A.	0
PON1	-0,042607553	0,077998511	0	N.A.	0
RNF182	-0,042610024		0	N.A.	0
HUWE1	-0,042610658	0,271321114	0	N.A.	0
CEP135	-0,042627728	0,010739199	0	N.A.	0
RP11-481J2.4	-0,042641345	-0,137651986	0	N.A.	0
C1QTNF3	-0,042645742	-0,060066912	0	N.A.	0
FENDRR	-0,04267516		0	N.A.	0
DKK3	-0,042686387	0,070799463	0	N.A.	0
CTC-503J8.4	-0,042709814	6,58404E-05	0	N.A.	0
KLHL6	-0,042725607	-0,128633565	0	N.A.	0
ULK2	-0,04277806	-0,248914052	0	N.A.	0
GATA3-AS1	-0,042791425	-0,048143372	0	N.A.	0
PXDN	-0,042798236	0,009348588	0	N.A.	0
DCN	-0,042804901	-0,249066929	0	N.A.	0
TMEM31	-0,042836682	0,051670952	0	N.A.	0
BTN1A1	-0,042849259		0	N.A.	0
CALHM3	-0,042850389	-0,13353802	0	N.A.	0
HOMER2	-0,042867991	0,027486578	0	N.A.	0
AMIGO1	-0,042924458	0,011561906	0	N.A.	0
TMEM25	-0,042929711	0,048767104	0	N.A.	0
AP3D1	-0,042945496	0,432333353	0	N.A.	0
PPAP2B	-0,042974904	-0,219677466	0	N.A.	0

RP13-942N8.	-0,042978965	-0,151358364	0	N.A.	0
RASSF1-AS1	-0,043009438	-0,080320716	0	N.A.	0
ESRRG	-0,043014272	-0,238102892	0	N.A.	0
PEX5L	-0,043061769		0	N.A.	0
PPP2R3B	-0,043086925	0,091769558	0	N.A.	0
RP11-455O6.	-0,0431164	0,136163744	0	N.A.	0
JRKL	-0,043150874	-0,00852321	0	N.A.	0
RP11-307L14	-0,043180078	0,04281172	0	N.A.	0
PDP2	-0,043194238	0,161672346	0	N.A.	0
RP11-475O6.	-0,043196039		0	N.A.	0
RBM12	-0,043199481	0,208593328	0	N.A.	0
GPR124	-0,043215111	-0,106358887	0	N.A.	0
C4orf29	-0,043236297	0,066552751	0	N.A.	0
ERICH3	-0,043243223	0,236229665	0	N.A.	0
RP11-305K5.	-0,043250088	-0,073795557	0	N.A.	0
PANK1	-0,043309088	-0,05519915	0	N.A.	0
ZNF460	-0,043317194	0,10189102	0	N.A.	0
GPLD1	-0,04332427		0	N.A.	0
GPC4	-0,043336534	-0,090327386	0	N.A.	0
GPX8	-0,043380384	-0,269072203	0	N.A.	0
RP11-467P9.	-0,043412106	0,101459042	0	N.A.	0
SMIM12	-0,043425886	0,141691762	0	N.A.	0
CTD-2555A7.	-0,043429936	-0,126832881	0	N.A.	0
ARAP3	-0,043506039	0,242113588	0	N.A.	0
GALE	-0,043510127	0,442664017	0	N.A.	0
CTD-2547H1	-0,043533603	-0,047673282	0	N.A.	0
AP1M1	-0,043538177	-0,047665545	0	N.A.	0
SAP30L-AS1	-0,04354808	-0,058741363	0	N.A.	0
IKBIP	-0,043568	-0,053862461	0	N.A.	0
RP11-1017G	-0,043573964	0,136456352	0	N.A.	0
ACOXL	-0,043595995	-0,035054796	0	N.A.	0
CTD-2547G2	-0,043764222	0,169744904	0	N.A.	0
RP11-526I2.5	-0,043799201	0,005617409	0	N.A.	0

NBPF3	-0,043886135	-0,140772266	0	N.A.	0
HAPLN1	-0,043906953		0	N.A.	0
IPO7	-0,043960674	0,024736226	0	N.A.	0
FGR	-0,043963996	-0,153020154	0	N.A.	0
CENPC	-0,043967892	0,02346936	0	N.A.	0
HOXD4	-0,044041527	0,147716069	0	N.A.	0
FGF20	-0,044059917	0,357053307	0	N.A.	0
ANGPTL5	-0,044111234	-0,159945608	0	N.A.	0
LIMS1	-0,044215788	0,127923799	0	N.A.	0
ASCL1	-0,044227113		0	N.A.	0
SVIP	-0,044237216	0,156555359	0	N.A.	0
PPP1R10	-0,044264777	0,066767856	0	N.A.	0
ZSCAN9	-0,044307451	0,107601717	0	N.A.	0
SIRPB1	-0,04432774	0,234783152	0	N.A.	0
ARFRP1	-0,044335566	0,080647163	0	N.A.	0
SOX12	-0,044371962	0,00880912	0	N.A.	0
RP11-1055B8	-0,044374068	0,249645432	0	N.A.	0
RP11-863P13	-0,044480325	0,021669065	0	N.A.	0
QPCTL	-0,044491913	-0,096218639	0	N.A.	0
KIZ-AS1	-0,044492213	-0,100890727	0	N.A.	0
IFNL1	-0,044503231		0	N.A.	0
UBXN1	-0,044515132	0,088579857	0	N.A.	0
RP11-443B7.	-0,044538268	0,101837471	0	N.A.	0
UGT2B10	-0,044553175		0	N.A.	0
FAM161B	-0,044563088	0,056936545	0	N.A.	0
CACNA1A	-0,044573036	0,07738115	0	N.A.	0
ACTR1A	-0,044604212	-0,043313217	0	N.A.	0
ZNF788	-0,044605596		0	N.A.	0
AC005523.2	-0,044632521	-0,060822989	0	N.A.	0
CTA-398F10.	-0,044761623	-0,314767257	0	N.A.	0
HRAS	-0,044771774	0,198351362	0	N.A.	0
SLC25A40	-0,044778308	-0,1121261	0	N.A.	0
RP11-440L14	-0,044786792	-0,101726119	0	N.A.	0

YTHDC2	-0,044787412	-0,042238932	0	N.A.	0
ITGBL1	-0,044854029	-0,043578553	0	N.A.	0
PGAM2	-0,044900734	-0,080590171	0	N.A.	0
MSANTD2	-0,044922879	-0,071210906	0	N.A.	0
RINL	-0,044953498	-0,079562403	0	N.A.	0
TOM1L1	-0,044973205	-0,159136411	0	N.A.	0
KLRG1	-0,045013565	-0,03181014	0	N.A.	0
DNMT3A	-0,045014197	-0,159472088	0	N.A.	0
AC253572.1	-0,045041255	-0,0857215	0	N.A.	0
SLC36A4	-0,045043948	-0,051136704	0	N.A.	0
CACNA1D	-0,045045704	0,173080548	0	N.A.	0
RP11-23F23.2	-0,045075779	-0,04741053	0	N.A.	0
RP11-166B2.	-0,045079747	-0,109979754	0	N.A.	0
NCOA7	-0,045090484	0,042348124	0	N.A.	0
AP001059.7	-0,045100996		0	N.A.	0
RP11-571L19	-0,045131271	-0,269912478	0	N.A.	0
RP11-551L14	-0,045154186	-0,153153926	0	N.A.	0
MMRN1	-0,045161201		0	N.A.	0
ING1	-0,045179468	0,221979533	0	N.A.	0
TNFSF13	-0,045215246	0,307483151	0	N.A.	0
KLRB1	-0,045250703	-0,026688319	0	N.A.	0
GADD45A	-0,045251651	0,252406472	0	N.A.	0
ZNF658	-0,045268786	0,118450246	0	N.A.	0
F8	-0,045322847	-0,229476418	0	N.A.	0
FAM195B	-0,045333398	-0,134785701	0	N.A.	0
TMEM110	-0,045337591	-0,17424793	0	N.A.	0
LENG8-AS1	-0,045345568	0,082688073	0	N.A.	0
RP11-25K19.	-0,045362914	-0,09744263	0	N.A.	0
RP11-127B20	-0,045394548	0,014254553	0	N.A.	0
IL16	-0,045394737	-0,311758011	0	N.A.	0
TMEM132C	-0,045405497		0	N.A.	0
GNAO1	-0,045415037		0	N.A.	0
SETD1A	-0,045429024	-0,087321312	0	N.A.	0

Z83844.1	-0,045501486	-0,026125815	0	N.A.	0
RP11-722E23	-0,045509393	0,113213266	0	N.A.	0
WFIKKN1	-0,045510239	0,23574981	0	N.A.	0
DERL1	-0,04553059	0,376727791	0	N.A.	0
RP4-671G15.	-0,045573647	0,243625159	0	N.A.	0
MANEA-AS1	-0,04558479	0,119322343	0	N.A.	0
GRK7	-0,045628804		0	N.A.	0
FGF9	-0,045663834	0,101136562	0	N.A.	0
AUH	-0,045686745	0,188661198	0	N.A.	0
RP11-538P18	-0,04570499	-0,199242293	0	N.A.	0
ATP6V1E2	-0,045717229	-0,134483027	0	N.A.	0
TBC1D19	-0,045720402	0,190894929	0	N.A.	0
BRICD5	-0,045746606	0,152785669	0	N.A.	0
LINC00881	-0,045765846	0,368355053	0	N.A.	0
FAM174B	-0,045768582	0,305886867	0	N.A.	0
FZD9	-0,045771285		0	N.A.	0
SMEK2	-0,045809242	-0,013464049	0	N.A.	0
CCDC129	-0,045812491	-0,076385861	0	N.A.	0
RP11-436D10	-0,045815734		0	N.A.	0
SDK2	-0,045817895		0	N.A.	0
IL10	-0,045827347		0	N.A.	0
SRCIN1	-0,045828828	0,42894884	0	N.A.	0
RCE1	-0,045910532	0,481607532	0	N.A.	0
MEGF9	-0,045936596	0,151800515	0	N.A.	0
LINC01134	-0,045972966	0,066194142	0	N.A.	0
TLE3	-0,045974587	0,19951805	0	N.A.	0
PELI3	-0,045989562	0,044581665	0	N.A.	0
DHRS11	-0,046015032	0,08745402	0	N.A.	0
AZIN1	-0,046041161	-0,209446338	0	N.A.	0
ENO1-AS1	-0,046070374	-0,023369795	0	N.A.	0
USP22	-0,046084492	0,133035975	0	N.A.	0
RP11-474G23	-0,046159725	-0,110193146	0	N.A.	0
IGDCC3	-0,046228329		0	N.A.	0

MIXL1	-0,046245521	0,304041626	0	N.A.	0
KCNJ8	-0,046259954		0	N.A.	0
NCDN	-0,046264206	0,120457164	0	N.A.	0
RP11-434D9.	-0,046336885	0,071269667	0	N.A.	0
LINC01268	-0,046339353	0,033241368	0	N.A.	0
FAM228B	-0,046346636	-0,275743576	0	N.A.	0
LINC00884	-0,046348718	-0,135227939	0	N.A.	0
CLASRP	-0,046357411	0,072327286	0	N.A.	0
RP11-890B15	-0,046412367	0,311516488	0	N.A.	0
RGL4	-0,046432357	0,312823144	0	N.A.	0
TCTA	-0,046437072	-0,354284928	0	N.A.	0
STK11IP	-0,046456654	0,148287735	0	N.A.	0
KCNN1	-0,04646125		0	N.A.	0
PCOLCE	-0,04648895	-0,219343561	0	N.A.	0
RP11-867G2.	-0,046503906	0,09469126	0	N.A.	0
CTA-384D8.3	-0,046528323		0	N.A.	0
CTD-2588C8.	-0,0465536	0,031177597	0	N.A.	0
RP11-428J1.5	-0,046569395		0	N.A.	0
ASNS	-0,046591902	-0,080616532	0	N.A.	0
ESYT1	-0,046619652	0,333899526	0	N.A.	0
AC009506.1	-0,04662611	-0,176831191	0	N.A.	0
ESPNL	-0,046627574	-0,190211508	0	N.A.	0
CRADD	-0,046627846	-0,308831006	0	N.A.	0
IWS1	-0,046646069	0,141209855	0	N.A.	0
RP11-52J3.2	-0,046647037		0	N.A.	0
LRIT3	-0,046663751	-0,164400728	0	N.A.	0
RP11-435O5.	-0,046684706	-0,038096577	0	N.A.	0
RP11-303E16	-0,04669231	-0,051750319	0	N.A.	0
KIAA0125	-0,046694943	-0,183041667	0	N.A.	0
DTX3	-0,046711141	-0,263475201	0	N.A.	0
PMF1-BGLAP	-0,046714776	-0,284599019	0	N.A.	0
BBS9	-0,046718268	0,047596837	0	N.A.	0
VPREB3	-0,04671859	-0,312336614	0	N.A.	0

GPATCH3	-0,046748964	-0,014658091	0	N.A.	0
EPS15L1	-0,046764038	-0,148448709	0	N.A.	0
MED25	-0,046844094	0,215633228	0	N.A.	0
AC129492.6	-0,046857525	0,036608357	0	N.A.	0
SLC43A2	-0,046882655	-0,04618811	0	N.A.	0
FKBP15	-0,046909412	0,241071128	0	N.A.	0
RP11-791G15	-0,046914605	0,112794675	0	N.A.	0
SPEF1	-0,046925733	-0,095606033	0	N.A.	0
ZNF772	-0,046980625		0	N.A.	0
PCBP3	-0,046984006	-0,193402557	0	N.A.	0
TTC39C	-0,046990334	-0,264461449	0	N.A.	0
HCG18	-0,047027006	-0,207074995	0	N.A.	0
LRRC27	-0,047030808	-0,128122612	0	N.A.	0
IFI30	-0,04703806	0,215438738	0	N.A.	0
ZNF850	-0,047049335	-0,169490963	0	N.A.	0
CCT6B	-0,047120687	-0,002376547	0	N.A.	0
RP11-496D24	-0,047164543	0,123119172	0	N.A.	0
HELB	-0,04719223	-0,188332762	0	N.A.	0
TBKBP1	-0,047204945	0,201500479	0	N.A.	0
PDCD10	-0,047211545	-0,069697441	0	N.A.	0
LYPD6	-0,047237508	-0,176898129	0	N.A.	0
POTEC	-0,047241512	-0,14565648	0	N.A.	0
ZZZ3	-0,047255174	-0,190715395	0	N.A.	0
GPANK1	-0,047304409	-0,294229116	0	N.A.	0
PIGH	-0,047310847	0,159588484	0	N.A.	0
TTTY15	-0,047327584	0,113046798	0	N.A.	0
MLH3	-0,047328458	0,122362993	0	N.A.	0
LMO7-AS1	-0,047328857	0,010753234	0	N.A.	0
JPH4	-0,047424695	0,031578778	0	N.A.	0
SLC2A2	-0,047428309	0,058008352	0	N.A.	0
WDR72	-0,047468324	-0,044336035	0	N.A.	0
RALY-AS1	-0,047472308	-0,037092355	0	N.A.	0
RP11-73K9.2	-0,047489127	-0,001802442	0	N.A.	0

RP11-306I1.2	-0,047517168	0,185957301	0	N.A.	0
LILRB3	-0,047520156	0,061965224	0	N.A.	0
RP4-790G17.	-0,047562577	0,140134487	0	N.A.	0
CCL19	-0,047619486	-0,349805407	0	N.A.	0
PRR5	-0,047627889	0,028459764	0	N.A.	0
CCDC173	-0,047636448	-0,199909019	0	N.A.	0
RP11-316O14	-0,047654008	-0,016571673	0	N.A.	0
TMEM116	-0,047658422	-0,146954698	0	N.A.	0
CSTA	-0,047702527	-0,245026463	0	N.A.	0
HMG20A	-0,047702832	0,013193969	0	N.A.	0
SMYD1	-0,047708138		0	N.A.	0
GLB1L3	-0,047768041		0	N.A.	0
GNA14	-0,047781383	-0,292269477	0	N.A.	0
PRICKLE2-AS	-0,047791624	-0,144003339	0	N.A.	0
RP11-77P16.	-0,047821159	-0,084956434	0	N.A.	0
MYADML2	-0,047848402	0,17481047	0	N.A.	0
GSTCD	-0,047848719	-0,06432798	0	N.A.	0
SRP54	-0,047887805	0,176452224	0	N.A.	0
MATN2	-0,047913081	0,043975735	0	N.A.	0
TRAPPC13	-0,047916479	-0,158018812	0	N.A.	0
KPNA6	-0,047967761	0,489395546	0	N.A.	0
CXCL2	-0,047968731	0,136399595	0	N.A.	0
SMC5	-0,047974873	0,073679458	0	N.A.	0
TMEM35	-0,047977936		0	N.A.	0
RAB28	-0,048024565	-0,092295418	0	N.A.	0
PEX16	-0,048068539	-0,039588759	0	N.A.	0
RP11-109M1	-0,048075798	-0,036985729	0	N.A.	0
BCO2	-0,048104308	0,003634402	0	N.A.	0
MMP11	-0,048121365	-0,196590279	0	N.A.	0
CTD-2020K17	-0,048183568	-0,162787489	0	N.A.	0
MTRR	-0,048213241	-0,112182575	0	N.A.	0
ADPRH	-0,048214209	-0,108096831	0	N.A.	0
LYPLA2	-0,048284588	0,42892016	0	N.A.	0

EDARADD	-0,048292353	-0,243922322	0	N.A.	0
RP11-216B9.	-0,048376731	0,24311684	0	N.A.	0
CGA	-0,048380653		0	N.A.	0
SLITRK5	-0,048440625		0	N.A.	0
PLIN1	-0,048488109		0	N.A.	0
CTD-2027I19	-0,048494664	-0,258148003	0	N.A.	0
RP11-80H5.7	-0,048507546		0	N.A.	0
UBE4B	-0,048513334	-0,198336407	0	N.A.	0
RP11-166B2.	-0,048539796	-0,010739222	0	N.A.	0
ZNF546	-0,048544376	-0,058638398	0	N.A.	0
RP1-20C7.6	-0,04855963		0	N.A.	0
RP11-6B4.1	-0,04863581	-0,012476491	0	N.A.	0
CSF3	-0,048643833	-0,063707002	0	N.A.	0
LCN8	-0,048726746		0	N.A.	0
DMPK	-0,048805525	-0,000949514	0	N.A.	0
SRRD	-0,048816496	0,10867541	0	N.A.	0
AP4S1	-0,048820701	0,062018009	0	N.A.	0
ZNF283	-0,04883519	0,12747402	0	N.A.	0
GDPD5	-0,048856693	0,065194909	0	N.A.	0
AP000654.4	-0,048914142		0	N.A.	0
ASIC2	-0,04891875	-0,200807866	0	N.A.	0
RP1-234P15.	-0,0489377	-0,076779856	0	N.A.	0
VPS54	-0,049038006	-0,001183246	0	N.A.	0
LINC00323	-0,049065017	-0,055509452	0	N.A.	0
CTD-2517O1(	-0,049100165	0,077333607	0	N.A.	0
XXbac-BPG29	-0,049104493	-0,021477036	0	N.A.	0
AP1S2	-0,049167517	-0,199596488	0	N.A.	0
IL37	-0,049190401	0,196850394	0	N.A.	0
ZNF251	-0,049314083	0,332342267	0	N.A.	0
TAF12	-0,04931888	-0,044614394	0	N.A.	0
RP11-263K19	-0,04933975	-0,117878812	0	N.A.	0
ZCCHC8	-0,04936743	0,02973925	0	N.A.	0
COL8A2	-0,049373244	-0,046439839	0	N.A.	0

BNC2	-0,049376426	0,312427088	0	N.A.	0
ZHX3	-0,049432279	0,212676148	0	N.A.	0
EGFL8	-0,049432794	0,324399721	0	N.A.	0
C6orf3	-0,049479113	0,013067842	0	N.A.	0
MTUS2-AS1	-0,049482744	0,068385814	0	N.A.	0
PPP1R26-AS1	-0,049577042	-0,072312856	0	N.A.	0
HAUS3	-0,049579322	0,177554018	0	N.A.	0
RTN3	-0,049588482	0,279117313	0	N.A.	0
GPSM3	-0,0495936	-0,360770009	0	N.A.	0
SYMPK	-0,04963171	0,02414813	0	N.A.	0
RAP2A	-0,049636096	0,394877244	0	N.A.	0
LA16c-321D4	-0,04965697	0,016523739	0	N.A.	0
ALG12	-0,049680725	0,024705227	0	N.A.	0
RP11-160E2.	-0,049732808	0,153884489	0	N.A.	0
RAPGEF4	-0,049769636	-0,244763223	0	N.A.	0
GRPEL2	-0,049813976	0,026843309	0	N.A.	0
KATNAL2	-0,049836591		0	N.A.	0
IL7	-0,049847656	-0,009556579	0	N.A.	0
RP11-480A16	-0,049851571	0,024635937	0	N.A.	0
RP11-95D17.	-0,049858178	0,036701147	0	N.A.	0
TRAF7	-0,049898811	-0,149924398	0	N.A.	0
HIST1H2AL	-0,049935132	-0,259500543	0	N.A.	0
RP11-304L19	-0,049970712	0,452944244	0	N.A.	0
LGALS7	-0,049984577		0	N.A.	0
CD53	-0,049996245	-0,177027414	0	N.A.	0
PUS10	-0,049997706	0,180340187	0	N.A.	0
EYA2	-0,049998793		0	N.A.	0
RP4-597N16.	-0,050007939	0,105174518	0	N.A.	0
RBM11	-0,05001316	-0,118187351	0	N.A.	0
PI4KB	-0,050036096	0,104616359	0	N.A.	0
IGKV1-6	-0,050117362	-0,331221377	0	N.A.	0
F10	-0,050138139	0,348228445	0	N.A.	0
RP3-492J12.2	-0,050185713		0	N.A.	0

PCK1	-0,050256912	0,060901812	0	N.A.	0
CTB-161C1.1	-0,050282766	0,163064082	0	N.A.	0
FDX1	-0,050336044	0,108916937	0	N.A.	0
LINC00680	-0,050406502	-0,111718553	0	N.A.	0
SFT2D2	-0,050439762	0,159832012	0	N.A.	0
AC002076.10	-0,050481142	0,067930874	0	N.A.	0
CHKB-AS1	-0,050483881	-0,289381084	0	N.A.	0
CEP112	-0,050515709	-0,105599012	0	N.A.	0
AC072062.1	-0,050522995		0	N.A.	0
RP11-706P11	-0,050561663		0	N.A.	0
RP11-360L9.7	-0,050589495	0,193759509	0	N.A.	0
ARL6IP1	-0,050609387	0,01759371	0	N.A.	0
C1orf168	-0,050730731	-0,107458933	0	N.A.	0
KLHL35	-0,05074402	-0,074524947	0	N.A.	0
RP11-359E10	-0,050744312	-0,027498033	0	N.A.	0
ZNF773	-0,050765154	-0,103679539	0	N.A.	0
CD3D	-0,050784523	-0,217976137	0	N.A.	0
RP11-494H4.	-0,050802578		0	N.A.	0
MS4A7	-0,050812842	-0,169393736	0	N.A.	0
RP11-21A7A.	-0,050823327	-0,164490617	0	N.A.	0
AOC2	-0,050847106	0,028201298	0	N.A.	0
ING2	-0,050851844	0,330577984	0	N.A.	0
PTGDR2	-0,050856361	-0,088195983	0	N.A.	0
BLOC1S6	-0,050860507	0,173026678	0	N.A.	0
CCDC142	-0,050867604	-0,012122295	0	N.A.	0
ALOX5AP	-0,050870081	-0,288064017	0	N.A.	0
RP11-464F9.1	-0,050885019	0,012174677	0	N.A.	0
PAXBP1-AS1	-0,050911576	-0,085932738	0	N.A.	0
ETNK1	-0,050951565	0,243885893	0	N.A.	0
KLHL18	-0,050962931	-0,480125416	0	N.A.	0
MYH10	-0,051008338	-0,202601377	0	N.A.	0
C17orf104	-0,051025226	0,035589043	0	N.A.	0
SBDS	-0,051040884	0,107478606	0	N.A.	0

SS18	-0,051044532	-0,046264424	0	N.A.	0
U47924.29	-0,051057996	0,362149408	0	N.A.	0
VTI1B	-0,051108239	-0,126739416	0	N.A.	0
RP11-432B6.	-0,051133266		0	N.A.	0
FGD3	-0,051150055	-0,319767363	0	N.A.	0
LINC01540	-0,051213856	-0,238987647	0	N.A.	0
RP11-138P22	-0,05121807		0	N.A.	0
CALHM2	-0,05123232	0,061735315	0	N.A.	0
AP001469.9	-0,051247636	-0,045368074	0	N.A.	0
AGR2	-0,05125736	0,091406596	0	N.A.	0
EMB	-0,05127871	-0,327400913	0	N.A.	0
LINC01209	-0,051299554	0,144252029	0	N.A.	0
HAR1B	-0,05133877	-0,086267063	0	N.A.	0
GFPT2	-0,051349776	-0,026267043	0	N.A.	0
BMP5	-0,051378374		0	N.A.	0
NR2F1	-0,051381958	-0,026806831	0	N.A.	0
ARPIN	-0,051388252	0,184727462	0	N.A.	0
RP11-706O15	-0,051392063	0,129551449	0	N.A.	0
SLAMF1	-0,051394488	-0,105215345	0	N.A.	0
SLC25A1	-0,051405192	0,163822569	0	N.A.	0
RRAGB	-0,051421286	-0,140774167	0	N.A.	0
RP13-39P12.	-0,051433533	-0,140473646	0	N.A.	0
LYPD8	-0,051463002	-0,055881299	0	N.A.	0
RP11-314C16	-0,051478008		0	N.A.	0
C14orf132	-0,051485326	0,09590838	0	N.A.	0
MRC1	-0,051520778	0,036920301	0	N.A.	0
FAM50B	-0,051521633	-0,036679502	0	N.A.	0
HIST1H2AG	-0,051608226	-0,170089653	0	N.A.	0
PCDHGA6	-0,051634608	-0,023957696	0	N.A.	0
CEP128	-0,051670299	-0,315749351	0	N.A.	0
ATP1A3	-0,051699258	-0,115218195	0	N.A.	0
HIST1H2BI	-0,051727561	-0,237225992	0	N.A.	0
CCNJL	-0,05181178	-0,120623354	0	N.A.	0

PDE3B	-0,051818394	-0,11142058	0	N.A.	0
UNC119	-0,051827257	-0,19127062	0	N.A.	0
EPOR	-0,051898316	-0,01863027	0	N.A.	0
OVOL2	-0,051904102	0,133994086	0	N.A.	0
ATP8A2	-0,051910538	-0,271446322	0	N.A.	0
CHST10	-0,051921328	0,042545689	0	N.A.	0
CDH24	-0,051947542	0,072625996	0	N.A.	0
C9orf163	-0,051957938		0	N.A.	0
RP11-102N12	-0,051984329	0,015060511	0	N.A.	0
CDK10	-0,051996401	0,32720462	0	N.A.	0
CTC-378H22.	-0,052044397		0	N.A.	0
RP11-415C15	-0,052044397		0	N.A.	0
C9orf135	-0,052086404		0	N.A.	0
ZNF383	-0,052111232	-0,052737623	0	N.A.	0
CYP2E1	-0,052199676	0,142587601	0	N.A.	0
SERP2	-0,052250991	-0,141735129	0	N.A.	0
CTA-212A2.2	-0,052252331	-0,227052952	0	N.A.	0
LRRN3	-0,052304777		0	N.A.	0
HAGH	-0,052336996	0,229491681	0	N.A.	0
RP11-114F3.4	-0,052337662		0	N.A.	0
YBEY	-0,052346937	-0,045743016	0	N.A.	0
LINC00184	-0,052350255		0	N.A.	0
MAPK4	-0,052404953		0	N.A.	0
TRAF3IP2-AS	-0,0524165	-0,039498739	0	N.A.	0
MAPKAP1	-0,05243233	-0,177671213	0	N.A.	0
RP11-1275H2	-0,052436133	-0,047499468	0	N.A.	0
FAM210A	-0,052446949	0,164107327	0	N.A.	0
LIPE	-0,052539454	0,015887685	0	N.A.	0
RP11-644F5.	-0,052546041	0,076686535	0	N.A.	0
PRKAB1	-0,052570351	0,348114482	0	N.A.	0
CTD-2568A17	-0,052574413	0,237110685	0	N.A.	0
ZNF280D	-0,052586767	0,056909563	0	N.A.	0
RP11-231I16	-0,052603558		0	N.A.	0

TWIST1	-0,052609942		0	N.A.	0
CYB5D1	-0,052639299	-0,033786742	0	N.A.	0
RP11-46C24.	-0,052655108	-0,0315157	0	N.A.	0
CNIH3	-0,052677931	0,004787804	0	N.A.	0
AF131216.1	-0,052701706	0,144528093	0	N.A.	0
CRYBB2	-0,052741579	-0,001506573	0	N.A.	0
TBX18	-0,052742099	0,050032358	0	N.A.	0
XX-FW83563	-0,052754558	0,042961784	0	N.A.	0
ACRBP	-0,052758362	-0,19551824	0	N.A.	0
CTD-2371O3	-0,052774945	-0,098114163	0	N.A.	0
KIAA1598	-0,052794586	-0,01119953	0	N.A.	0
RP11-440L14	-0,052837968	0,012906369	0	N.A.	0
SLC23A1	-0,052881703	0,14614218	0	N.A.	0
RP11-31F15.	-0,052945372	-0,025234084	0	N.A.	0
CDC20B	-0,052967646		0	N.A.	0
INTS3	-0,052978249	0,071732364	0	N.A.	0
TLR1	-0,053029409	-0,092816783	0	N.A.	0
ZNF648	-0,05305447		0	N.A.	0
PITPNB	-0,0531	-0,127993274	0	N.A.	0
RP5-858B6.3	-0,053100297		0	N.A.	0
RP1-198K11.	-0,053110793	-0,171039764	0	N.A.	0
FNTB	-0,053110832	0,003201672	0	N.A.	0
NXPH3	-0,053115675		0	N.A.	0
RP11-352G9.	-0,053149434	0,235492783	0	N.A.	0
HSD17B13	-0,053185742	0,101157682	0	N.A.	0
TSACC	-0,053195757	-0,074693833	0	N.A.	0
SIRT4	-0,053205627	0,023879631	0	N.A.	0
HIST1H2BN	-0,053214329	-0,237035746	0	N.A.	0
HTR2A	-0,053247829		0	N.A.	0
WDR63	-0,053248568	0,058828896	0	N.A.	0
LINC00167	-0,053249641	-0,018107382	0	N.A.	0
ZNF551	-0,053257351	0,11822054	0	N.A.	0
LOH12CR1	-0,053308969	-0,349240794	0	N.A.	0

SAMD15	-0,053461123	-0,284507096	0	N.A.	0
KCNMB2	-0,053480646	0,257326007	0	N.A.	0
RP4-561L24.3	-0,053499022	0,038322605	0	N.A.	0
DACT3	-0,053518473	0,26841577	0	N.A.	0
CST4	-0,053533705	-0,268799474	0	N.A.	0
CTD-2529O2	-0,05360665		0	N.A.	0
RP11-540A21	-0,053632845	0,156800498	0	N.A.	0
AC079922.3	-0,053634419	-0,033983838	0	N.A.	0
RP11-284F21	-0,05364762	-0,06958774	0	N.A.	0
ZNF833P	-0,053658982	-0,161099524	0	N.A.	0
CRB2	-0,053691648	-0,032085972	0	N.A.	0
HCK	-0,053708966	-0,136742005	0	N.A.	0
RP13-516M1	-0,053713105	0,177790994	0	N.A.	0
EFR3B	-0,053780341	0,039664825	0	N.A.	0
FMOD	-0,053786909	-0,237699629	0	N.A.	0
CYB5R4	-0,053794269	-0,080938997	0	N.A.	0
TFIP11	-0,053803143	0,031827166	0	N.A.	0
CTC-231O11.	-0,053810594	0,177757624	0	N.A.	0
ROGDI	-0,053829747	0,266123671	0	N.A.	0
HOXA5	-0,053840343	-0,031094038	0	N.A.	0
SLC22A2	-0,053844672	-0,036837214	0	N.A.	0
PQLC2L	-0,053850222	0,107473348	0	N.A.	0
ZCCHC3	-0,05388996	-0,007026983	0	N.A.	0
RP11-17J14.2	-0,053913259	0,039328624	0	N.A.	0
RP11-560J1.2	-0,053917121	-0,288367417	0	N.A.	0
RP5-1057J7.7	-0,054006226	-0,147290646	0	N.A.	0
JUNB	-0,054024101	0,511385	0	N.A.	0
GAS7	-0,054051508	-0,238141215	0	N.A.	0
CPA5	-0,054058422	-0,088758096	0	N.A.	0
AC100830.4	-0,054090546	0,099005264	0	N.A.	0
UHRF2	-0,054135853	-0,050209354	0	N.A.	0
SOX15	-0,054146854		0	N.A.	0
SPATA6	-0,054161572	0,053370096	0	N.A.	0

C12orf56	-0,054169066	-0,095693475	0	N.A.	0
STT3B	-0,054289042	0,242887855	0	N.A.	0
C1orf87	-0,054290021		0	N.A.	0
AHSG	-0,054293336	0,181892388	0	N.A.	0
RP1-302G2.5	-0,05436174		0	N.A.	0
MYO10	-0,054379293	0,17381888	0	N.A.	0
GSR	-0,05438176	-0,227841021	0	N.A.	0
GRIK4	-0,054431882		0	N.A.	0
ZNF84	-0,054443199	0,111994127	0	N.A.	0
C9orf117	-0,054474418	0,052950807	0	N.A.	0
SLC38A4	-0,054479132	0,016839355	0	N.A.	0
PRDM13	-0,054479413	-0,037059929	0	N.A.	0
GTSE1-AS1	-0,054495389	-0,188654855	0	N.A.	0
VAC14	-0,054518406	0,046532414	0	N.A.	0
ZNF416	-0,054526758	0,175477211	0	N.A.	0
ARMCX5	-0,054583493	-0,203832726	0	N.A.	0
CAPN13	-0,05458759	-0,132279904	0	N.A.	0
VPS36	-0,05459097	0,097804756	0	N.A.	0
SLC29A2	-0,054633001	0,185723271	0	N.A.	0
DUSP26	-0,054644497		0	N.A.	0
KIF26A	-0,054645543	-0,107871212	0	N.A.	0
RP11-689C9.	-0,054673668		0	N.A.	0
CTD-2013N1	-0,054724715	0,161314459	0	N.A.	0
ALG9	-0,054729575	-0,010382703	0	N.A.	0
RAB7A	-0,054787225	-0,069295385	0	N.A.	0
RCAN2	-0,054835163	-0,096532152	0	N.A.	0
EGR1	-0,054843695	0,038204002	0	N.A.	0
PLEKHG4	-0,054849216	0,025390469	0	N.A.	0
RP11-631F7.	-0,054926402	0,137817428	0	N.A.	0
FGL1	-0,054942593		0	N.A.	0
SLC35E3	-0,054951691	0,046420409	0	N.A.	0
LA16c-385E7	-0,054953894	-0,027339268	0	N.A.	0
LINC01127	-0,054956631	-0,096260115	0	N.A.	0

LINC00959	-0,054957502	-0,020352169	0	N.A.	0
HPS3	-0,054963418	-0,089347774	0	N.A.	0
UROC1	-0,054992461	-0,068824098	0	N.A.	0
WDFY2	-0,054994214	0,048210906	0	N.A.	0
EXOC2	-0,054995307	0,120830924	0	N.A.	0
RP11-519G16	-0,054997962	-0,228257868	0	N.A.	0
AC005152.3	-0,05503529		0	N.A.	0
TRIM24	-0,055049575	-0,258299377	0	N.A.	0
LINC00920	-0,055071781	-0,007993878	0	N.A.	0
RP11-342K6.	-0,055103593		0	N.A.	0
RP1-16A9.1	-0,055119683		0	N.A.	0
RP11-123K3.	-0,055148517		0	N.A.	0
C11orf98	-0,055149209	-0,372285865	0	N.A.	0
BRD3	-0,055165448	-0,065970552	0	N.A.	0
AC007277.3	-0,055219685	0,291989283	0	N.A.	0
IGKV4-1	-0,055230169	-0,013093232	0	N.A.	0
HPN	-0,055245541	0,002320423	0	N.A.	0
RRAGA	-0,05526322	-0,012724881	0	N.A.	0
PPP1R37	-0,055266566	0,171769244	0	N.A.	0
GJA3	-0,05530682		0	N.A.	0
AP4B1-AS1	-0,055309738		0	N.A.	0
IL7R	-0,055370093	-0,004918953	0	N.A.	0
NHLRC3	-0,055434938	0,075018928	0	N.A.	0
GSAP	-0,055456047	0,278117089	0	N.A.	0
GNB3	-0,055516641	-0,064338933	0	N.A.	0
MAST4-AS1	-0,055521244	-0,333434642	0	N.A.	0
RBBP5	-0,055538876	-0,082314329	0	N.A.	0
POU6F1	-0,055542211	0,191269711	0	N.A.	0
RP11-484K9.	-0,055589132	0,008433379	0	N.A.	0
SIDT1	-0,055643169	-0,001421861	0	N.A.	0
RP4-593H12.	-0,055666097	0,145018128	0	N.A.	0
CADM4	-0,05566681	0,136123822	0	N.A.	0
SKIDA1	-0,055683188	-0,083490092	0	N.A.	0

SFRP2	-0,0556978	-0,331701956	0	N.A.	0
MAT2B	-0,055719752	-0,320999653	0	N.A.	0
UGDH	-0,055741101	0,00442211	0	N.A.	0
ZSCAN32	-0,055741554	0,009132173	0	N.A.	0
PRRC2C	-0,055773182	0,457866253	0	N.A.	0
ZC3H10	-0,055791377	-0,128565743	0	N.A.	0
BECN1	-0,055822433	0,097804638	0	N.A.	0
RETNLB	-0,05584091	-0,296866511	0	N.A.	0
TNS4	-0,055846977	0,372542954	0	N.A.	0
ZBTB10	-0,055856366	0,08028584	0	N.A.	0
LRRC36	-0,055861242	-0,169919632	0	N.A.	0
RP11-515O1	-0,055868344		0	N.A.	0
CRISPLD2	-0,055939059	-0,175060846	0	N.A.	0
FZD4	-0,055956887	0,05007691	0	N.A.	0
MRI1	-0,055963563	-0,204183203	0	N.A.	0
ZNF880	-0,05597452	0,034701938	0	N.A.	0
ZNF133	-0,055980087	-0,113176718	0	N.A.	0
RP11-203B9.	-0,056010874		0	N.A.	0
RP11-148B1	-0,056022435	0,014959771	0	N.A.	0
RBM18	-0,056051462	0,042273476	0	N.A.	0
AC017074.2	-0,056058629	-0,367764653	0	N.A.	0
FPGT	-0,056073415	-0,043725764	0	N.A.	0
RERG	-0,056133418		0	N.A.	0
RAB39B	-0,056162972	-0,058476831	0	N.A.	0
MORF4L1	-0,056170462	0,122729821	0	N.A.	0
ABCA12	-0,056188373	0,116458314	0	N.A.	0
CLPS	-0,056203547		0	N.A.	0
GNG7	-0,056208112	-0,02028811	0	N.A.	0
ARRB1	-0,05622425	0,122001278	0	N.A.	0
TCEAL3	-0,056236473	-0,070917217	0	N.A.	0
NSF	-0,056255406	-0,167757874	0	N.A.	0
PRUNE2	-0,056291893	0,03172791	0	N.A.	0
PRR4	-0,056339254	0,058536235	0	N.A.	0

UBOX5	-0,056345448	-0,055082273	0	N.A.	0
PTH2	-0,056358758		0	N.A.	0
MYNN	-0,056365222	0,084346077	0	N.A.	0
KDELR2	-0,056381673	-0,082469797	0	N.A.	0
ACR	-0,056433099	0,065952873	0	N.A.	0
CHP2	-0,05647226	0,017664334	0	N.A.	0
MIPOL1	-0,056545175	-0,22630541	0	N.A.	0
THAP7-AS1	-0,056566286	-0,272191343	0	N.A.	0
LINC00543	-0,056595611	0,408195887	0	N.A.	0
TRPM2-AS	-0,056607289	0,01536188	0	N.A.	0
RFWD3	-0,056614042	-0,081296126	0	N.A.	0
AATBC	-0,05664461	-0,18412617	0	N.A.	0
TMEM17	-0,056645295	0,041219853	0	N.A.	0
PARS2	-0,056703071	0,087228877	0	N.A.	0
AC004549.6	-0,056707623		0	N.A.	0
PPIL6	-0,056708528	-0,285669511	0	N.A.	0
RP11-297N6.	-0,05672518	-0,007376395	0	N.A.	0
IGKV1-27	-0,056796079	-0,003684285	0	N.A.	0
CTD-2152M2	-0,056823616		0	N.A.	0
AC002451.3	-0,056847282	0,042360174	0	N.A.	0
AP001058.3	-0,056869644	-0,177913346	0	N.A.	0
AP006216.10	-0,056873801	0,18479211	0	N.A.	0
RP11-441O1!	-0,05687969	0,088231487	0	N.A.	0
DYNLT1	-0,056893289	-0,096940485	0	N.A.	0
STX6	-0,056920466	0,055880589	0	N.A.	0
AQP9	-0,056945053	-0,192574988	0	N.A.	0
XXbac-BPG30	-0,056950432	-0,224154321	0	N.A.	0
RP11-353N14	-0,056954696	0,096846785	0	N.A.	0
PYGO2	-0,056962036	-0,100522169	0	N.A.	0
PLCXD3	-0,056971035		0	N.A.	0
RP1-267D11.	-0,057012036	-0,239592204	0	N.A.	0
KIF5C	-0,057067765	0,068318156	0	N.A.	0
UIMC1	-0,057095801	0,149473342	0	N.A.	0

CTD-2659N1!	-0,057130261	-0,085408847	0	N.A.	0
LZIC	-0,057152785	-0,297968686	0	N.A.	0
ACTR3C	-0,057168426	0,010233264	0	N.A.	0
PYGL	-0,057173983	-0,110706409	0	N.A.	0
DCHS1	-0,057180553	-0,031109234	0	N.A.	0
RP11-802O2:	-0,057197598	0,211362429	0	N.A.	0
WASF1	-0,057198858	-0,059028237	0	N.A.	0
RP11-147L13	-0,057199467	0,075012447	0	N.A.	0
AMDHD1	-0,057234293	-0,046665637	0	N.A.	0
GJA1	-0,057255938	0,104395225	0	N.A.	0
RP5-991G20.	-0,05727468	-0,072322052	0	N.A.	0
KLHL15	-0,057297763	-0,212183568	0	N.A.	0
RP11-680F20	-0,057321361		0	N.A.	0
NUP214	-0,057323857	0,231826577	0	N.A.	0
BTNL9	-0,057331861	0,241205851	0	N.A.	0
NKX3-1	-0,057344332	-0,051972085	0	N.A.	0
NDNL2	-0,057352766	0,17841579	0	N.A.	0
ZNF766	-0,057398785	0,109725187	0	N.A.	0
RFX3	-0,057440703	0,00654733	0	N.A.	0
FDXACB1	-0,057450472	0,031253347	0	N.A.	0
PIK3IP1-AS1	-0,057452212	0,032596962	0	N.A.	0
CDKN2D	-0,057472789	-0,068463716	0	N.A.	0
USHBP1	-0,057492097	0,041146927	0	N.A.	0
ACTRT3	-0,057526709	0,080264317	0	N.A.	0
PRICKLE4	-0,057527139	-0,284084576	0	N.A.	0
ARSB	-0,057528211	0,0120299	0	N.A.	0
DBNDD2	-0,057543049	-0,071442857	0	N.A.	0
SETSIP	-0,05756028	0,119311545	0	N.A.	0
DZIP1L	-0,057561409		0	N.A.	0
AP001412.1	-0,05760031	0,029191109	0	N.A.	0
RP11-680A1:	-0,057614357		0	N.A.	0
E2F5	-0,057653324	-0,108060004	0	N.A.	0
ZNF566	-0,057667937	0,093611762	0	N.A.	0

RP11-147L13	-0,057713542	0,021888662	0	N.A.	0
TNFSF4	-0,05773346	-0,080474895	0	N.A.	0
TMUB1	-0,057753241	0,428331381	0	N.A.	0
IRX2	-0,057817932	0,188023686	0	N.A.	0
NAA35	-0,05785235	0,173148531	0	N.A.	0
AC001226.7	-0,057853908	-0,103179656	0	N.A.	0
USPL1	-0,057868137	0,250385184	0	N.A.	0
LINC01479	-0,057883163		0	N.A.	0
CTD-2516F10	-0,057932415	0,155530994	0	N.A.	0
AC012360.4	-0,057933795	-0,185227956	0	N.A.	0
TTC25	-0,057973409	0,210774811	0	N.A.	0
DDB2	-0,058023149	-0,170313248	0	N.A.	0
ELF5	-0,058108808	-0,02002941	0	N.A.	0
LPAR6	-0,058126493	-0,064401788	0	N.A.	0
RP11-109N23	-0,058145441	0,234225929	0	N.A.	0
C6orf226	-0,058180016	0,0340957	0	N.A.	0
RP11-265N7.	-0,058190741	-0,145952334	0	N.A.	0
ACSF3	-0,058217643	0,295745639	0	N.A.	0
NIT1	-0,058225221	0,027238449	0	N.A.	0
CHRM3-AS2	-0,05825673		0	N.A.	0
PDE12	-0,058263161	-0,039800441	0	N.A.	0
STARD5	-0,058309135	-0,031477375	0	N.A.	0
RP11-302L19	-0,058318156	0,025527421	0	N.A.	0
ABHD6	-0,058362338	0,127645848	0	N.A.	0
CD300C	-0,05840817		0	N.A.	0
VWA8	-0,058447039	-0,112099709	0	N.A.	0
VSTM4	-0,058521277	-0,227200158	0	N.A.	0
RP11-142A22	-0,058613262		0	N.A.	0
RP4-666F24.	-0,058654034	-0,043231967	0	N.A.	0
P2RY11	-0,058669737	-0,003317283	0	N.A.	0
RP1-257A7.5	-0,058769888	-0,00421056	0	N.A.	0
RIPPLY3	-0,058801656	-0,029099781	0	N.A.	0
PTCH1	-0,058804772	0,281962542	0	N.A.	0

TRIM16L	-0,058817148	0,015278367	0	N.A.	0
HAGLROS	-0,05885896	-0,160118528	0	N.A.	0
KIF2A	-0,058992243	-0,008080477	0	N.A.	0
LRRC37A2	-0,059046492	0,098616893	0	N.A.	0
RP11-210M1	-0,059096059		0	N.A.	0
KCNH6	-0,059125161	-0,185155523	0	N.A.	0
ICE1	-0,059159679	0,282639968	0	N.A.	0
PTCHD1	-0,059168474		0	N.A.	0
ITFG1-AS1	-0,059170114		0	N.A.	0
CTB-47B11.3	-0,059240412	-0,019819473	0	N.A.	0
CTD-3214H1	-0,0592507	0,068795652	0	N.A.	0
ZNF202	-0,059324103	0,24249071	0	N.A.	0
DEF6	-0,059348621	-0,058222577	0	N.A.	0
LRRC31	-0,059363639	-0,220516039	0	N.A.	0
HIST1H2AH	-0,059499213	-0,117781952	0	N.A.	0
PRKCQ	-0,059512229		0	N.A.	0
FBXO18	-0,059550812	-0,028029463	0	N.A.	0
RP11-490O6.	-0,059562324	-0,053008559	0	N.A.	0
RGMB-AS1	-0,059583042	0,087998085	0	N.A.	0
RP11-91K11.	-0,059607154	-0,184047734	0	N.A.	0
GPRC5B	-0,059662895	0,121190957	0	N.A.	0
CALML4	-0,059687332	0,274928611	0	N.A.	0
CACTIN	-0,059690787	0,06427103	0	N.A.	0
CKLF-CMTM1	-0,059696329	-0,125052138	0	N.A.	0
AOC3	-0,059711877	-0,009499703	0	N.A.	0
MATR3	-0,059739005	-0,482396136	0	N.A.	0
MCM9	-0,059740704	-0,164328533	0	N.A.	0
RP11-44F14.	-0,059762492	-0,042494363	0	N.A.	0
SEMA6C	-0,059779823	0,11884159	0	N.A.	0
RP11-379B18	-0,059796496	-0,220997912	0	N.A.	0
GSC	-0,059819947		0	N.A.	0
CFAP45	-0,059872201	0,031314541	0	N.A.	0
KLHDC10	-0,059885221	0,010332506	0	N.A.	0

ITLN1	-0,059912195	-0,072496641	0	N.A.	0
PNMA6A	-0,059914169		0	N.A.	0
EEF1G	-0,059955434	-0,423405205	0	N.A.	0
PRPF18	-0,059991043	0,17989123	0	N.A.	0
RDH12	-0,060005027	-0,148885632	0	N.A.	0
EHHADH	-0,060010848	-0,118201459	0	N.A.	0
PDE1B	-0,060042404	0,073655637	0	N.A.	0
GATC	-0,060060963	-0,131518685	0	N.A.	0
HIST1H3C	-0,06007589	-0,344237954	0	N.A.	0
SAMD11	-0,060087986		0	N.A.	0
EML1	-0,060101142	0,163110124	0	N.A.	0
FAM49A	-0,060132519	-0,245162956	0	N.A.	0
AC011997.1	-0,060156422	0,058176961	0	N.A.	0
S1PR2	-0,060231646	-0,170218197	0	N.A.	0
SLC30A4	-0,06024771	-0,157466989	0	N.A.	0
SH3BP1	-0,060282425	0,083217623	0	N.A.	0
FAN1	-0,06029694	-0,047225284	0	N.A.	0
RP11-455F5.[illegible]	-0,060328701	-0,236325568	0	N.A.	0
RP11-225H2[illegible]	-0,060354314		0	N.A.	0
SHISA6	-0,060391311		0	N.A.	0
DLX6	-0,060396249	-0,073719042	0	N.A.	0
FLOT2	-0,060409792	0,034087315	0	N.A.	0
HERC2	-0,060459167	0,229367573	0	N.A.	0
ZNF717	-0,060492801	-0,054427313	0	N.A.	0
RP11-102M1[illegible]	-0,060522664	-0,050044944	0	N.A.	0
KCNIP2-AS1	-0,060570762	0,153243867	0	N.A.	0
CSPG5	-0,060585749	0,155773757	0	N.A.	0
TBCCD1	-0,060616491	-0,233034425	0	N.A.	0
SYN3	-0,060654346	0,13399773	0	N.A.	0
RP11-231D2[illegible]	-0,060659679		0	N.A.	0
RP11-798G7.[illegible]	-0,060685058	-0,008386951	0	N.A.	0
FSCN3	-0,06069284	0,015181645	0	N.A.	0
SLC30A3	-0,060707584		0	N.A.	0

FKBP1C	-0,060710225	-0,332572451	0	N.A.	0
RP11-458J1.1	-0,060731125	-0,067599182	0	N.A.	0
RP11-152N13	-0,060754254	0,027413861	0	N.A.	0
CECR5-AS1	-0,060758977	0,101756188	0	N.A.	0
TCAF1	-0,060783546	0,299397532	0	N.A.	0
SULT1A4	-0,060804879	0,006836738	0	N.A.	0
MT-ND6	-0,060810381	0,181918519	0	N.A.	0
ELTD1	-0,060953415	-0,236142469	0	N.A.	0
LINC01024	-0,060991529	0,100797039	0	N.A.	0
C1orf74	-0,06106425	-0,010328996	0	N.A.	0
SUCLA2-AS1	-0,061147254	-0,053834883	0	N.A.	0
BCAT1	-0,061169093	0,107322253	0	N.A.	0
C1QB	-0,06120996	0,204305524	0	N.A.	0
ROM1	-0,061221507	-0,323747085	0	N.A.	0
RP11-474G23	-0,061234551	-0,127170368	0	N.A.	0
LINC00339	-0,061237076	-0,266793237	0	N.A.	0
ADCY3	-0,061292898	-0,036720194	0	N.A.	0
LZTS2	-0,061313349	0,052609946	0	N.A.	0
RP11-390F4.1	-0,061341892	0,08501013	0	N.A.	0
MAEL	-0,061348765	0,055669387	0	N.A.	0
VSIG8	-0,06136538	-0,020506955	0	N.A.	0
CCDC106	-0,061373957	-0,432627335	0	N.A.	0
TMOD4	-0,061383538	0,098905859	0	N.A.	0
CTD-3193K9.	-0,061403454	0,11052362	0	N.A.	0
NR2C1	-0,061418545	-0,107486386	0	N.A.	0
ZSCAN26	-0,06143099	-0,153785128	0	N.A.	0
CAMK1D	-0,061448211	-0,022912417	0	N.A.	0
RP11-230C9.	-0,061489801	-0,042337005	0	N.A.	0
SLC4A10	-0,061640791		0	N.A.	0
FAM92B	-0,061676047		0	N.A.	0
EIF4ENIF1	-0,061681357	0,148509867	0	N.A.	0
RP11-752G15	-0,061707892	-0,018390052	0	N.A.	0
MBTD1	-0,061710685	-0,022386954	0	N.A.	0

SPTSSB	-0,061760363	0,019651876	0	N.A.	0
CCDC42B	-0,061777894	0,078814971	0	N.A.	0
MME	-0,061822205	0,025240324	0	N.A.	0
TVP23C-CDR1	-0,061839333		0	N.A.	0
RABEP1	-0,06185438	-0,184549223	0	N.A.	0
ZBTB9	-0,061903945	-0,076173449	0	N.A.	0
TCF7	-0,061945808	-0,048912726	0	N.A.	0
CTB-113D17.	-0,061945876	0,045421041	0	N.A.	0
CNIH2	-0,061968828	0,313114854	0	N.A.	0
HSF2BP	-0,061983231	0,131613059	0	N.A.	0
MAP10	-0,062075716	-0,114830133	0	N.A.	0
RP11-426L16	-0,062092352	-0,047438404	0	N.A.	0
CDK20	-0,062139591	-0,070160256	0	N.A.	0
TTTY14	-0,062148221	-0,145599626	0	N.A.	0
HTR3E	-0,062168219		0	N.A.	0
TAS2R14	-0,062195034	0,148293643	0	N.A.	0
YME1L1	-0,062247999	0,345138072	0	N.A.	0
LINC00665	-0,062260853	-0,226667525	0	N.A.	0
PRKD3	-0,062266012	-0,137709341	0	N.A.	0
APOD	-0,062269006	0,163664621	0	N.A.	0
RP1-151F17.	-0,062302807	0,165509622	0	N.A.	0
RP3-473L9.4	-0,062348467	0,018698658	0	N.A.	0
RP11-546K22	-0,062349081	-0,226617383	0	N.A.	0
TRGC2	-0,06236756		0	N.A.	0
ODF3L2	-0,062404989	0,081823509	0	N.A.	0
NTNG2	-0,062430372		0	N.A.	0
URB1-AS1	-0,062446309	0,090135274	0	N.A.	0
ELMOD3	-0,062484118	0,15679553	0	N.A.	0
LRRIQ4	-0,062502039	-0,026597964	0	N.A.	0
NEK11	-0,062561274	-0,072152816	0	N.A.	0
RASL11B	-0,062608699	-0,055051731	0	N.A.	0
ZNF100	-0,062633415	0,003334399	0	N.A.	0
FUBP3	-0,062651149	0,063257735	0	N.A.	0

TCF7L1	-0,062706639	0,044870892	0	N.A.	0
JADE1	-0,062783756	-0,187959029	0	N.A.	0
HOXD3	-0,062784737	-0,06384095	0	N.A.	0
GHRH	-0,062797495		0	N.A.	0
RP11-221N13	-0,062812265	0,309301802	0	N.A.	0
RP11-700H6.	-0,062839827	0,21559004	0	N.A.	0
RP11-775C24	-0,062895496	0,131612282	0	N.A.	0
KB-1562D12.	-0,06300918	0,033741599	0	N.A.	0
RP11-819C21	-0,06303153	0,349393098	0	N.A.	0
INMT	-0,063089244		0	N.A.	0
TGIF2	-0,063107355	-0,029304782	0	N.A.	0
MAN1B1-AS1	-0,063138246	-0,052598022	0	N.A.	0
RP11-524C21	-0,063147862	0,031623484	0	N.A.	0
PIGP	-0,063154117	-0,215078422	0	N.A.	0
FBRSL1	-0,063175664	0,181246649	0	N.A.	0
RP11-443B7.	-0,06319708	-0,092997671	0	N.A.	0
TMEM150C	-0,063231305	-0,167955219	0	N.A.	0
LINC00638	-0,06324301		0	N.A.	0
ZNF439	-0,063250907	-0,248960866	0	N.A.	0
THADA	-0,06329443	0,005966486	0	N.A.	0
SUSD4	-0,06332797	-0,029105341	0	N.A.	0
BOLA2	-0,063339449	-0,105348791	0	N.A.	0
RP11-755B10	-0,063349027	-0,054624309	0	N.A.	0
SGSM3	-0,063427379	0,197618728	0	N.A.	0
SPTSSA	-0,063432183	-0,225046205	0	N.A.	0
RP11-351D16	-0,06345625	0,011093667	0	N.A.	0
KB-1568E2.1	-0,063460916		0	N.A.	0
HIST1H2BM	-0,063470688	-0,315213904	0	N.A.	0
XPO4	-0,06349785	0,284196231	0	N.A.	0
ZC3H7A	-0,063504331	0,111907093	0	N.A.	0
UGT2B4	-0,063533803		0	N.A.	0
FOXRED2	-0,063601164	-0,13268748	0	N.A.	0
RP4-816N1.6	-0,063609355	-0,163647856	0	N.A.	0

CRCP	-0,06363702	-0,19031109	0	N.A.	0
DNAJC14	-0,063672859	-0,079804889	0	N.A.	0
RP11-297K8.	-0,063679073	-0,168518739	0	N.A.	0
RP4-569M23	-0,063701695		0	N.A.	0
MITF	-0,063766491	-0,068903286	0	N.A.	0
PTGDR	-0,063786069	-0,250688294	0	N.A.	0
RP11-307N1(	-0,063804978	-0,038719654	0	N.A.	0
ZNF30	-0,063815594	-0,201604163	0	N.A.	0
PDCD4	-0,063819452	-0,197474292	0	N.A.	0
FAM19A3	-0,063849681		0	N.A.	0
RP11-495P1(	-0,063865809	-0,073878153	0	N.A.	0
TCEB3-AS1	-0,063881754	-0,131104863	0	N.A.	0
IGHV3-48	-0,063899021		0	N.A.	0
UCK1	-0,063915224	-0,237985052	0	N.A.	0
CHRM3	-0,063951802	0,165142083	0	N.A.	0
MMP17	-0,063963879		0	N.A.	0
DNAJB1	-0,063992464	0,25459915	0	N.A.	0
UPRT	-0,064006172	-0,076897223	0	N.A.	0
RP11-798G7.	-0,064094111	-0,039702897	0	N.A.	0
RP11-80H5.2	-0,064134154	0,202295038	0	N.A.	0
DYNC1I1	-0,064175347	-0,273961893	0	N.A.	0
KIF3A	-0,064225226	0,034839539	0	N.A.	0
REP15	-0,064228455	0,113494955	0	N.A.	0
DENND4B	-0,06422987	0,00204405	0	N.A.	0
RP11-475I24	-0,064306423	-0,140071981	0	N.A.	0
EPHX1	-0,064310936	-0,046234633	0	N.A.	0
CTD-2538C1.	-0,06433764	0,051795379	0	N.A.	0
SPACA3	-0,064342222	-0,045454661	0	N.A.	0
KRBOX1	-0,064359254		0	N.A.	0
DNAL4	-0,064415002	0,111852446	0	N.A.	0
NUB1	-0,064453354	0,265621554	0	N.A.	0
ZNF16	-0,064549734	-0,00252391	0	N.A.	0
PIK3R6	-0,064565093	-0,05245725	0	N.A.	0

TRPA1	-0,064600908		0	N.A.	0
RNF25	-0,064629005	-0,017741952	0	N.A.	0
RP11-59C5.3	-0,064674441	0,206061514	0	N.A.	0
ASB16-AS1	-0,064744866	-0,023145923	0	N.A.	0
S100A12	-0,064767064	0,038428745	0	N.A.	0
STAC2	-0,064775002		0	N.A.	0
ADAMTS4	-0,064822416	-0,076359246	0	N.A.	0
ETS2	-0,064847909	-0,297194233	0	N.A.	0
ZNF736	-0,064857653	-0,183730286	0	N.A.	0
CTD-2349P21	-0,064886576	-0,089691438	0	N.A.	0
APOC2	-0,064933764	-0,262194917	0	N.A.	0
SCARA3	-0,064933906	-0,051033008	0	N.A.	0
ID2-AS1	-0,064976336	-0,056427219	0	N.A.	0
ART5	-0,065043317		0	N.A.	0
LINC01451	-0,065048293	-0,093583792	0	N.A.	0
FAR1	-0,065058573	0,102693566	0	N.A.	0
RIC8B	-0,06506207	0,380066612	0	N.A.	0
FRRS1L	-0,065164409	0,081667818	0	N.A.	0
RHOJ	-0,065167964	0,093476156	0	N.A.	0
PELO	-0,065189762	0,023895827	0	N.A.	0
RP11-227G15	-0,065243172		0	N.A.	0
LINC00668	-0,06526147	0,035365412	0	N.A.	0
SCN8A	-0,065312956	0,018425249	0	N.A.	0
ADORA3	-0,065341609	0,368015909	0	N.A.	0
RP11-93B14.	-0,065365048		0	N.A.	0
EDC4	-0,065375909	0,007842031	0	N.A.	0
RP11-416N2.	-0,065381996	0,054631779	0	N.A.	0
CRYBA2	-0,065415698	-0,30629508	0	N.A.	0
ASB2	-0,065418018	-0,080387704	0	N.A.	0
RP11-57G10.	-0,065424323	0,000133142	0	N.A.	0
RP11-78O7.2	-0,06542991	0,284252388	0	N.A.	0
CTA-292E10.	-0,065477598	0,117029871	0	N.A.	0
RP11-63M22	-0,065494137	-0,075850054	0	N.A.	0

RP11-77K12.	-0,065495733	0,02330289	0	N.A.	0
RP5-827C21.	-0,065568177	0,122171214	0	N.A.	0
ALKBH8	-0,065604518	-0,00121979	0	N.A.	0
PREPL	-0,06565096	0,160491084	0	N.A.	0
LINC00607	-0,065658338		0	N.A.	0
SMO	-0,065698181	-0,141801331	0	N.A.	0
FAM178A	-0,065718299	-0,008377577	0	N.A.	0
ADAMTS12	-0,065722044	0,108982463	0	N.A.	0
GLT8D2	-0,065724685	-0,013834002	0	N.A.	0
LINC00861	-0,065791838	-0,093103196	0	N.A.	0
RP11-468E2.	-0,065811223	0,027712185	0	N.A.	0
LY6G5C	-0,065812268	-0,152407729	0	N.A.	0
DOLPP1	-0,065882341	0,076322394	0	N.A.	0
B4GALT2	-0,06592689	0,039238345	0	N.A.	0
GPR113	-0,065969757	0,015493462	0	N.A.	0
CCDC171	-0,066030266	0,010628606	0	N.A.	0
RP11-442H21	-0,066059915	0,201675755	0	N.A.	0
MAP2K6	-0,066077392	-0,151998674	0	N.A.	0
POMGNT1	-0,066094556	-0,12864716	0	N.A.	0
TSHZ3	-0,066156863	0,017673947	0	N.A.	0
MT2A	-0,066201053	-0,20710425	0	N.A.	0
MZF1-AS1	-0,066211011	-0,062189984	0	N.A.	0
MORC2	-0,066248474	0,124487877	0	N.A.	0
HUS1	-0,066281582	-0,048616734	0	N.A.	0
MIS12	-0,066298342	-0,109475749	0	N.A.	0
ZIC2	-0,06632227	0,231206515	0	N.A.	0
AC012487.2	-0,06635955	-0,06566422	0	N.A.	0
TMEM105	-0,066366508	0,133613129	0	N.A.	0
KB-1471A8.1	-0,066369142		0	N.A.	0
RP1-80N2.2	-0,066460683	0,210576184	0	N.A.	0
HBE1	-0,066499295	-0,002060108	0	N.A.	0
AC009961.3	-0,066556939	0,043232849	0	N.A.	0
EIF4G2	-0,066650678	0,235169887	0	N.A.	0

SRGAP2	-0,066664579	-0,142417773	0	N.A.	0
KIF27	-0,066725479	-0,187257413	0	N.A.	0
EML4	-0,066742557	-0,039860277	0	N.A.	0
RP11-390P2.	-0,066782375	-0,144633678	0	N.A.	0
TIGD4	-0,066800512	-0,243751944	0	N.A.	0
CABLES2	-0,06684259	0,031045339	0	N.A.	0
CNOT1	-0,06687526	0,05329049	0	N.A.	0
RP11-434D9.	-0,06691319	-0,071166322	0	N.A.	0
MT1E	-0,066938956	-0,250480088	0	N.A.	0
LRRIQ1	-0,066950432	-0,125188191	0	N.A.	0
SDCBP2-AS1	-0,066977174	-0,26495607	0	N.A.	0
PCDHGC3	-0,067026462	-0,09015563	0	N.A.	0
POMZP3	-0,067033185	0,029298048	0	N.A.	0
INTS6-AS1	-0,067046104	0,07866582	0	N.A.	0
NRL	-0,067053319	-0,237903765	0	N.A.	0
DGKD	-0,067055688	0,011466644	0	N.A.	0
CTC-336P14.	-0,067055743	0,083295607	0	N.A.	0
LINC00909	-0,067064868	-0,030115209	0	N.A.	0
AC084809.2	-0,067081043	-0,023164402	0	N.A.	0
NEUROD2	-0,067119456		0	N.A.	0
RP5-1136G1	-0,067124587	0,245645608	0	N.A.	0
TNFSF11	-0,067176022	0,189727643	0	N.A.	0
RP11-121C2.	-0,067231663	0,083339666	0	N.A.	0
C4A	-0,067304322		0	N.A.	0
LINC00242	-0,067319803		0	N.A.	0
TMEM121	-0,067324411		0	N.A.	0
PTGES2-AS1	-0,067339539	-0,012191854	0	N.A.	0
TRAF3IP3	-0,06735487	-0,336667694	0	N.A.	0
PROS1	-0,06736558	0,175843829	0	N.A.	0
TM2D3	-0,067399146	0,11851765	0	N.A.	0
DYSF	-0,067471977	-0,125701726	0	N.A.	0
TATDN2	-0,067473838	0,072080248	0	N.A.	0
ZNF696	-0,06751757	0,306986384	0	N.A.	0

GATA3	-0,067535263	-0,078787933	0	N.A.	0
CD244	-0,06754995		0	N.A.	0
LINC01214	-0,067559488	-0,189163077	0	N.A.	0
FLJ27354	-0,067580231	-0,010996257	0	N.A.	0
RAB34	-0,067582651	-0,103074586	0	N.A.	0
ARX	-0,067589623	-0,208800747	0	N.A.	0
CRYZL1	-0,067592699	0,194972905	0	N.A.	0
OTOA	-0,067639048	-0,096333518	0	N.A.	0
ZNF337	-0,06765066	0,264442678	0	N.A.	0
HPS4	-0,067665399	0,359712824	0	N.A.	0
RP11-243A14	-0,067690186	-0,042731732	0	N.A.	0
ELMOD2	-0,067734416	0,401380551	0	N.A.	0
SCUBE3	-0,067786549	-0,196508902	0	N.A.	0
C4orf36	-0,067810939	0,014621754	0	N.A.	0
TMEM178B	-0,067873995	-0,047913844	0	N.A.	0
SYNGR2	-0,067940338	0,616256387	0	N.A.	0
LINC01411	-0,067945341	-0,024390664	0	N.A.	0
RP11-323J4.1	-0,067950105		0	N.A.	0
OVCH2	-0,067960637		0	N.A.	0
ZNF544	-0,067970187	-0,017253745	0	N.A.	0
CTD-2619J13	-0,067991442		0	N.A.	0
ZNF300	-0,068002118	-0,028148727	0	N.A.	0
RP11-47A8.5	-0,068011175	-0,025559173	0	N.A.	0
LINC00910	-0,068014653	0,010148851	0	N.A.	0
AGBL3	-0,068050748	-0,086510107	0	N.A.	0
FCGR2B	-0,068085056	0,041265871	0	N.A.	0
AQP5	-0,068105523	0,074387905	0	N.A.	0
RP6-65G23.5	-0,068111276	0,245015841	0	N.A.	0
RP11-598F7.1	-0,06811745		0	N.A.	0
DEFA5	-0,068142524	0,146135279	0	N.A.	0
SCG5	-0,068167823	-0,009851096	0	N.A.	0
RP11-387M2	-0,068168086		0	N.A.	0
ZNF397	-0,068199265	0,147247852	0	N.A.	0

PPM1F	-0,06821213	-0,167710144	0	N.A.	0
USP28	-0,068228203	-0,204893914	0	N.A.	0
ADAMTS17	-0,068259954	-0,269061212	0	N.A.	0
RP11-1157N2	-0,068276735		0	N.A.	0
RP11-13K12.	-0,06831028	0,19710109	0	N.A.	0
TBK1	-0,068350081	0,216864255	0	N.A.	0
PLA2G4A	-0,068385345	0,156718927	0	N.A.	0
RP11-60L3.6	-0,06840006		0	N.A.	0
SETDB1	-0,068420672	0,039187685	0	N.A.	0
HSD3B7	-0,068439101	0,171629509	0	N.A.	0
GPR84	-0,068444181	0,315932116	0	N.A.	0
IGLV8-61	-0,068446503		0	N.A.	0
CRELD2	-0,068451722	0,245129349	0	N.A.	0
B9D2	-0,068472191	-0,175211372	0	N.A.	0
RP1-60O19.1	-0,068483784	0,00561079	0	N.A.	0
RP11-404O13	-0,068507328		0	N.A.	0
C2orf44	-0,068525415	-0,09498567	0	N.A.	0
FAM109B	-0,06853026	-0,248117467	0	N.A.	0
RP11-166P13	-0,068546249	0,074165534	0	N.A.	0
LINC00574	-0,068595833	0,007088511	0	N.A.	0
CDIPT-AS1	-0,06860906	-0,200048854	0	N.A.	0
PRELP	-0,068684202	0,016833699	0	N.A.	0
AF015262.2	-0,068714102		0	N.A.	0
RHBDL3	-0,068721352		0	N.A.	0
LA16c-325D7	-0,068731002	-0,005388244	0	N.A.	0
MARVELD1	-0,068737857	-0,174905016	0	N.A.	0
CYB5R2	-0,068739787	-0,051684531	0	N.A.	0
SMC2-AS1	-0,068758949	-0,092637339	0	N.A.	0
SLC19A3	-0,068760756	-0,012946206	0	N.A.	0
IGLV3-19	-0,068789035		0	N.A.	0
STX17	-0,068916955	0,157502001	0	N.A.	0
GOLGA8R	-0,068924033		0	N.A.	0
ZAP70	-0,068936254	-0,189240177	0	N.A.	0

LINC01094	-0,068990318	-0,189300122	0	N.A.	0
RP11-739B23	-0,069009853	-0,091787878	0	N.A.	0
GPR18	-0,069018745	-0,330161772	0	N.A.	0
RASGRF1	-0,069042583		0	N.A.	0
SLC39A4	-0,069082743	0,525253436	0	N.A.	0
RNF139-AS1	-0,06908994	-0,204560304	0	N.A.	0
NPC1L1	-0,069100636	0,190006753	0	N.A.	0
ZNF142	-0,069102454	-0,024244431	0	N.A.	0
TRAPPC5	-0,069163821	-0,106242928	0	N.A.	0
RP11-77H9.8	-0,069175416	-0,02437652	0	N.A.	0
RAD52	-0,06918514	-0,002269954	0	N.A.	0
PAGR1	-0,069276641	-0,053220347	0	N.A.	0
KB-1980E6.3	-0,069278973		0	N.A.	0
ROR1	-0,069353856	-0,023334666	0	N.A.	0
GPR37	-0,069384322	-0,116827459	0	N.A.	0
STRA6	-0,06938845	0,192893036	0	N.A.	0
STK4-AS1	-0,069389124	0,128747696	0	N.A.	0
ERC2	-0,069413452		0	N.A.	0
LIMD2	-0,069426673	-0,371316076	0	N.A.	0
FAM66B	-0,069454924	-0,197971532	0	N.A.	0
GPR55	-0,069485381	0,09044238	0	N.A.	0
CRTAM	-0,0695108		0	N.A.	0
RP11-128A17	-0,069527936	-0,056337412	0	N.A.	0
IL11RA	-0,069550264	0,22452391	0	N.A.	0
ZKSCAN3	-0,069581128	0,099223502	0	N.A.	0
RP11-398K22	-0,069596112	-0,091198677	0	N.A.	0
PCDH11X	-0,069606012	-0,105842508	0	N.A.	0
HIST1H2BJ	-0,069625672	-0,169129074	0	N.A.	0
ZFHX2	-0,069637907	0,176948001	0	N.A.	0
TGFBRAP1	-0,069644562	-0,24672245	0	N.A.	0
NDE1	-0,069666314	0,145265599	0	N.A.	0
HIPK2	-0,069686055	0,329837181	0	N.A.	0
KCNMA1	-0,069697132	0,105332385	0	N.A.	0

ARHGEF26	-0,069738043	-0,009475041	0	N.A.	0
SCAMP5	-0,069757686	0,275868975	0	N.A.	0
RP11-367N14	-0,069769998	-0,069217584	0	N.A.	0
PPP2R1B	-0,069778808	0,096479324	0	N.A.	0
SLC4A7	-0,069826928	-0,133635059	0	N.A.	0
ASAH2B	-0,06988405	-0,048908976	0	N.A.	0
RP11-1100L3	-0,069893561		0	N.A.	0
SFMBT1	-0,069928381	0,160714568	0	N.A.	0
MDP1	-0,069952253	-0,017805128	0	N.A.	0
DDAH1	-0,069986115	-0,029917516	0	N.A.	0
C15orf40	-0,0700072	-0,095446286	0	N.A.	0
CTD-2008P7.	-0,070024227	-0,005579261	0	N.A.	0
MEPCE	-0,070031593	-0,005269333	0	N.A.	0
ABCB10	-0,070112877	0,179645934	0	N.A.	0
UCHL1	-0,070197604	-0,203818603	0	N.A.	0
MMD	-0,070226882	0,299792755	0	N.A.	0
RP11-266K4.	-0,070234414	0,018914752	0	N.A.	0
RP5-963E22.	-0,070263858	0,126471359	0	N.A.	0
GPR19	-0,070276474		0	N.A.	0
AFF2	-0,070308135	0,053384142	0	N.A.	0
SETD2	-0,070314622	-0,029978377	0	N.A.	0
ZNF257	-0,070335622	-0,148798317	0	N.A.	0
CCDC61	-0,070339894	0,036323854	0	N.A.	0
FAM32A	-0,070363745	-0,069539463	0	N.A.	0
RP4-794I6.4	-0,070389434	0,092470827	0	N.A.	0
RP11-157F20	-0,07039389	0,077041406	0	N.A.	0
CTD-2012J19	-0,070414874	0,086548768	0	N.A.	0
TMED4	-0,070439353	0,352816923	0	N.A.	0
FRZB	-0,070440386	-0,116588146	0	N.A.	0
KIF19	-0,07046124	-0,107667982	0	N.A.	0
RP3-461P17.	-0,070547418	0,054542662	0	N.A.	0
PPP2R5E	-0,070608097	-0,053220388	0	N.A.	0
DCAF17	-0,070636613	-0,21232739	0	N.A.	0

MIOS	-0,070662196	-0,16404035	0	N.A.	0
CXXC4	-0,070708603	-0,105668567	0	N.A.	0
MNDA	-0,070733033	-0,175417695	0	N.A.	0
NUDT12	-0,070743584	0,028097851	0	N.A.	0
PYROXD2	-0,070763794	0,129644021	0	N.A.	0
MYO15A	-0,070810014	0,175472236	0	N.A.	0
SETBP1	-0,070817533	-0,136274535	0	N.A.	0
RP11-35O15.[illegible]	-0,070818465	-0,104922996	0	N.A.	0
GMPPB	-0,070841223	-0,010192159	0	N.A.	0
LMNTD1	-0,07087248	-0,191295133	0	N.A.	0
RP11-795F19[illegible]	-0,070872569		0	N.A.	0
CLUL1	-0,070885074		0	N.A.	0
RP5-1125A11[illegible]	-0,070941709		0	N.A.	0
RP11-190A12[illegible]	-0,070961508	0,106418957	0	N.A.	0
RP11-354K1.[illegible]	-0,070993315	-0,098315121	0	N.A.	0
RP11-490B18[illegible]	-0,071008178	-0,069730469	0	N.A.	0
KRBA2	-0,071012554	0,10738948	0	N.A.	0
RP11-697M1[illegible]	-0,071014388	-0,102040132	0	N.A.	0
RP3-428L16.[illegible]	-0,071018698	-0,087374832	0	N.A.	0
SYNC	-0,071034732	-0,337063402	0	N.A.	0
KIAA1755	-0,07108057		0	N.A.	0
SLC16A7	-0,071087779	-0,000838944	0	N.A.	0
LINC00618	-0,071116086	-0,023347373	0	N.A.	0
PABPC3	-0,071123067	-0,131292143	0	N.A.	0
COL15A1	-0,071158435	-0,044492777	0	N.A.	0
ITGAX	-0,07117415	-0,001229569	0	N.A.	0
RP11-1055B8[illegible]	-0,071177217	-0,049348157	0	N.A.	0
MYT1	-0,071187286	0,07279475	0	N.A.	0
GFAP	-0,071220594		0	N.A.	0
RP3-388E23.[illegible]	-0,071246528	-0,047551926	0	N.A.	0
METTL6	-0,071271609	-0,280648225	0	N.A.	0
HPX	-0,071284866	0,124421366	0	N.A.	0
YLPM1	-0,071298075	0,043508257	0	N.A.	0

GLB1	-0,071329173	0,146092885	0	N.A.	0
DRD2	-0,071356893	-0,088094985	0	N.A.	0
SH2B2	-0,071414112	-0,163455706	0	N.A.	0
RP11-66B24.	-0,071425272	-0,083194829	0	N.A.	0
PHTF1	-0,071431248	-0,038176059	0	N.A.	0
PAPSS2	-0,071448995	0,003223309	0	N.A.	0
RP11-546J1.1	-0,071549869	-0,026258741	0	N.A.	0
RP3-461F17.:	-0,071585903	0,201033621	0	N.A.	0
KCTD18	-0,071602598	0,21879132	0	N.A.	0
DPT	-0,071650787	-0,217688248	0	N.A.	0
EMR1	-0,071675986		0	N.A.	0
ANGEL2	-0,071741492	0,113988554	0	N.A.	0
APLF	-0,071764699	0,068946334	0	N.A.	0
CELF3	-0,071844532	0,11029525	0	N.A.	0
IGHV3-49	-0,071847654		0	N.A.	0
CLN3	-0,071882561	0,057009478	0	N.A.	0
AP000866.1	-0,071953523	0,146033991	0	N.A.	0
RP11-220C2.	-0,071987699	0,042422839	0	N.A.	0
AC005537.2	-0,0720002	0,007564695	0	N.A.	0
SARNP	-0,072006021	0,013222454	0	N.A.	0
PINLYP	-0,072053038	0,021709898	0	N.A.	0
SF3A1	-0,072075995	0,127804604	0	N.A.	0
RP11-159D12	-0,072079043	-0,094996421	0	N.A.	0
RP11-48B3.5	-0,07212934	0,053915578	0	N.A.	0
GRIN1	-0,072141972	-0,018943097	0	N.A.	0
RP11-5A19.5	-0,072145602	-0,085413198	0	N.A.	0
MOCOS	-0,072156998	-0,104400035	0	N.A.	0
CTD-2090I13	-0,07219448	-0,178879452	0	N.A.	0
KDR	-0,072309677	0,038607337	0	N.A.	0
ADM5	-0,07232182	0,034566296	0	N.A.	0
DDO	-0,072344562	-0,116547894	0	N.A.	0
U91319.1	-0,072353432		0	N.A.	0
CKMT2-AS1	-0,072375671	0,140018676	0	N.A.	0

SIX3-AS1	-0,072445284	-0,014266278	0	N.A.	0
RP11-111M2	-0,072445336	-0,172693223	0	N.A.	0
TRAC	-0,072471286	-0,328787373	0	N.A.	0
TMEM63A	-0,07247272	0,228336065	0	N.A.	0
SP2-AS1	-0,072482109	0,199784109	0	N.A.	0
EIF5A2	-0,07250959	0,075463432	0	N.A.	0
TPH1	-0,072537995	0,315065298	0	N.A.	0
DHFRL1	-0,072557519	0,069032335	0	N.A.	0
RP11-402D21	-0,072577986	0,075663292	0	N.A.	0
ZNF385A	-0,07259363	0,08345945	0	N.A.	0
PCYT1B	-0,072612645		0	N.A.	0
C6orf163	-0,072649401	0,009955539	0	N.A.	0
HMGN3-AS1	-0,072756297	0,14847044	0	N.A.	0
CCDC121	-0,072790773	-0,170730757	0	N.A.	0
SLC9B2	-0,072840537	-0,089057694	0	N.A.	0
PPT2-EGFL8	-0,072870135	0,29362402	0	N.A.	0
CCDC102B	-0,072879817	-0,364500236	0	N.A.	0
UNK	-0,072942375	-0,07540146	0	N.A.	0
MAGI2	-0,072944584	-0,032420592	1	none	0
RP3-329E20.	-0,072977798	-0,037127264	0	N.A.	0
IGBP1	-0,072990039	-0,106721116	0	N.A.	0
TCTEX1D1	-0,073004904		0	N.A.	0
ZNF146	-0,073062715	-0,253797888	0	N.A.	0
MYT1L	-0,073098466	0,088950574	0	N.A.	0
RP11-770J1.4	-0,07319783	0,042216629	0	N.A.	0
PTX3	-0,073244664		0	N.A.	0
HELQ	-0,073289506	-0,122602503	0	N.A.	0
RP11-739L10	-0,073291367	0,256092663	0	N.A.	0
B3GNT4	-0,073296671	0,103354797	0	N.A.	0
PIP4K2B	-0,073308962	-0,053819736	0	N.A.	0
RP11-386G11	-0,073337666	-0,453467558	0	N.A.	0
SERHL2	-0,073390439	0,084378465	0	N.A.	0
PCDHGB4	-0,073394849		0	N.A.	0

URAD	-0,073412673	-0,20142858	0	N.A.	0
ZCCHC18	-0,073450056		0	N.A.	0
RP11-1007O2	-0,073467895	0,112810864	0	N.A.	0
CCDC3	-0,07348062	0,014286807	0	N.A.	0
RP11-474P2.	-0,073505545		0	N.A.	0
BCL7B	-0,073516972	0,014285088	0	N.A.	0
RP11-617F23	-0,07353893	0,107015588	0	N.A.	0
MTFR1L	-0,073568527	-0,239713486	0	N.A.	0
ANXA4	-0,073582682	0,318504789	0	N.A.	0
RP11-548H3.	-0,07358618	-0,158297991	0	N.A.	0
SRPK3	-0,073594092	-0,31874739	0	N.A.	0
SHISA9	-0,073597173		0	N.A.	0
TAS1R3	-0,07361148	-0,277628999	0	N.A.	0
SIGIRR	-0,073628847	0,403903815	0	N.A.	0
CSRNP3	-0,073678605	-0,199527178	0	N.A.	0
DCTN6	-0,073738266	0,17217697	0	N.A.	0
AC009299.3	-0,073785241	-0,255510009	0	N.A.	0
GIT1	-0,073809256	-0,022718097	0	N.A.	0
INTU	-0,073900569	-0,062585368	0	N.A.	0
RBM26-AS1	-0,073932684	-0,195609325	0	N.A.	0
CYP20A1	-0,073978385	-0,128157079	0	N.A.	0
LDLRAD4	-0,074011207	0,007365814	0	N.A.	0
FJX1	-0,074024967	-0,095048112	0	N.A.	0
ZNF180	-0,074105439	-0,191815744	0	N.A.	0
C8orf86	-0,074157997		0	N.A.	0
FITM1	-0,07416179	0,195904803	0	N.A.	0
ME3	-0,074162786	-0,089602244	0	N.A.	0
MYH7B	-0,074166317	-0,111250404	0	N.A.	0
ALOX15	-0,07419706	0,244426477	0	N.A.	0
C10orf99	-0,074211728	-0,155140015	0	N.A.	0
UBXN7	-0,074245645	0,094948623	0	N.A.	0
HOXB13	-0,074246277	-0,21103639	0	N.A.	0
RP11-620J15	-0,074292853		0	N.A.	0

CTC-492K19.	-0,074318104		0	N.A.	0
RP5-943J3.2	-0,074342771	-0,133593448	0	N.A.	0
LINC01346	-0,074356697		0	N.A.	0
TIA1	-0,0744035	0,153013595	0	N.A.	0
TVP23A	-0,07440672		0	N.A.	0
LRP6	-0,074414071	0,015721081	0	N.A.	0
CHST14	-0,074417637	0,163237707	0	N.A.	0
RP11-301O1!	-0,074419614	0,141939188	0	N.A.	0
TIGD5	-0,07443913	-0,136505443	0	N.A.	0
PALB2	-0,074473754	0,140399514	0	N.A.	0
FLYWCH2	-0,074529139	0,099423615	0	N.A.	0
SLN	-0,07453057		0	N.A.	0
PIM2	-0,074537729	-0,059169223	0	N.A.	0
SDR42E1	-0,07457167	-0,22030575	0	N.A.	0
WDR19	-0,074592395	-0,169606126	0	N.A.	0
STX1B	-0,074594659	0,278529367	0	N.A.	0
AL928768.3	-0,074602864	-0,262194917	0	N.A.	0
OTULIN	-0,074698001	-0,023649826	0	N.A.	0
RP4-549L20.:	-0,074725231	-0,175037586	0	N.A.	0
RP11-867G2:	-0,074740115	-0,116539298	0	N.A.	0
RBPJ	-0,074744822	0,048886415	0	N.A.	0
RP11-629O1.	-0,074781652	0,05172196	0	N.A.	0
ATP8B4	-0,074824945	0,165513294	0	N.A.	0
RCBTB1	-0,07483886	0,038331241	0	N.A.	0
SMIM19	-0,074840207	0,000425303	0	N.A.	0
TAF1A-AS1	-0,074842579	-0,194105209	0	N.A.	0
TAF1C	-0,074872383	0,144753343	0	N.A.	0
SPN	-0,074879016	-0,331355535	0	N.A.	0
RP11-480I12	-0,074895893	0,094004252	0	N.A.	0
MLLT6	-0,074907384	0,299683268	0	N.A.	0
AP3B1	-0,074907784	0,145703347	0	N.A.	0
PLAGL1	-0,074950484	-0,32242933	0	N.A.	0
CTD-2078B5.	-0,074980383	0,042972404	0	N.A.	0

RFPL4B	-0,075002368		0	N.A.	0
MYPOP	-0,075028853	-0,206328013	0	N.A.	0
CTD-2501B8.	-0,075175449		0	N.A.	0
RP11-599B13	-0,075180649	0,002540124	0	N.A.	0
METTL7A	-0,075182789	-0,136991666	0	N.A.	0
RP3-380B8.4	-0,07518625	-0,022818435	0	N.A.	0
HMGCS2	-0,075196695	-0,149373348	0	N.A.	0
TSPYL4	-0,075205546	0,128701957	0	N.A.	0
LRRC7	-0,07528409	-0,062447338	0	N.A.	0
ZNF442	-0,075304602	0,032226038	0	N.A.	0
WNK4	-0,075312589	-0,226511634	0	N.A.	0
ISG20L2	-0,075335828	-0,079976737	0	N.A.	0
TMEM44-AS1	-0,075350669	-0,100842906	0	N.A.	0
TCFL5	-0,07535216	0,12130093	0	N.A.	0
FAM131B	-0,075362085	0,090525168	0	N.A.	0
RP11-392O17	-0,075402072	-0,012266413	0	N.A.	0
ZNF260	-0,075404756	-0,109299034	0	N.A.	0
CCL24	-0,075405267	-0,203873742	0	N.A.	0
LINC00843	-0,075422855		0	N.A.	0
PXK	-0,075437088	-0,135856038	0	N.A.	0
APOM	-0,075449637	-0,343589502	0	N.A.	0
OPRD1	-0,075497635	-0,120459399	0	N.A.	0
CTD-2186M1	-0,07552265	-0,19010029	0	N.A.	0
AC144831.1	-0,075523412	0,072350564	0	N.A.	0
AL358852.1	-0,075569493	-0,225416747	0	N.A.	0
AGMO	-0,075615033	0,142078007	0	N.A.	0
NSMAF	-0,075620373	0,143466811	0	N.A.	0
PCNT	-0,075636237	-0,171463268	0	N.A.	0
C16orf54	-0,075636792	-0,158570881	0	N.A.	0
SOAT1	-0,075673231	-0,026886619	0	N.A.	0
CTA-126B4.7	-0,075702733	0,205365573	0	N.A.	0
SP140	-0,075743047	-0,19366105	0	N.A.	0
DCAF6	-0,075766014	-0,062187221	0	N.A.	0

HSF1	-0,075776812	0,060019936	0	N.A.	0
ZNF587B	-0,075814895	-0,101296354	0	N.A.	0
RP11-276H19	-0,075816594	-0,113290383	0	N.A.	0
SIMC1	-0,0758271	-0,320975416	0	N.A.	0
ZSCAN31	-0,075858625	-0,340230776	0	N.A.	0
SLC30A6	-0,075891788	0,130273293	0	N.A.	0
RP11-771K4.	-0,075938	0,097577297	0	N.A.	0
AL162759.1	-0,075948441		0	N.A.	0
HTRA1	-0,075961572	0,146538296	0	N.A.	0
AURKC	-0,076014694	0,052051677	0	N.A.	0
CSTL1	-0,076016534	0,241789426	0	N.A.	0
AQR	-0,076071683	0,104401525	0	N.A.	0
MILR1	-0,076076082	0,110926456	0	N.A.	0
AC007255.8	-0,076081489	0,131468165	0	N.A.	0
CTD-2260A17	-0,076090108	-0,121415304	0	N.A.	0
FAM111A	-0,076132505	-0,135755458	0	N.A.	0
FAM151B	-0,076155441	-0,124038275	0	N.A.	0
CHGA	-0,076156245		0	N.A.	0
MYEF2	-0,076158606	-0,226850329	0	N.A.	0
RP1-179N16.	-0,076168762	-0,09892291	0	N.A.	0
CYSLTR1	-0,076194069	-0,296068138	0	N.A.	0
OVGP1	-0,076195223	-0,296036411	0	N.A.	0
KLK4	-0,076220722	-0,068636712	0	N.A.	0
RP11-708J19	-0,076244661	0,156577419	0	N.A.	0
TMEM72	-0,076245224	-0,021519532	0	N.A.	0
TMEM60	-0,076294369	-0,188395705	0	N.A.	0
RP13-20L14.1	-0,076297189	0,090824085	0	N.A.	0
ZNF79	-0,076305609	-0,071221979	0	N.A.	0
ZGLP1	-0,076331127	-0,177194538	0	N.A.	0
TMEM123	-0,076375389	0,022455387	0	N.A.	0
OCA2	-0,076375704	-0,070546698	0	N.A.	0
LETMD1	-0,076406103	-0,119142328	0	N.A.	0
L3MBTL4	-0,076435751	0,09157474	0	N.A.	0

DFNB59	-0,076442257	0,001628323	0	N.A.	0
PRKAR2B	-0,076445233	0,224436225	0	N.A.	0
RP11-203M5	-0,076448307	-0,092980845	0	N.A.	0
NPPC	-0,076555552		0	N.A.	0
SLC18A1	-0,076617378	0,003449588	0	N.A.	0
CTD-2035E11	-0,076671291	0,128199061	0	N.A.	0
AC097468.4	-0,076699435	0,282039722	0	N.A.	0
RP5-1186N24	-0,076726582	0,23644011	0	N.A.	0
NCOA5	-0,076787025	-0,203742069	0	N.A.	0
C11orf65	-0,076811089	0,050940944	0	N.A.	0
ARSK	-0,076843879	0,002446472	0	N.A.	0
RP11-403P17	-0,076862063		0	N.A.	0
ZNF530	-0,07686548	-0,157929648	0	N.A.	0
SYNM	-0,076874506	-0,109728548	0	N.A.	0
MARCH7	-0,076906031	0,170918698	0	N.A.	0
SH3RF3	-0,076914537	-0,169230686	0	N.A.	0
P2RX6	-0,077008417	0,109900792	0	N.A.	0
LAPTM4A	-0,077065932	0,243197515	0	N.A.	0
SLC38A11	-0,07707227	0,009656847	0	N.A.	0
NEFL	-0,077075524		0	N.A.	0
RP11-111F5.4	-0,077137191	-0,458546214	0	N.A.	0
RP11-532M2	-0,077138065	0,219805737	0	N.A.	0
ZNF521	-0,077180393	-0,042883996	0	N.A.	0
LINC00642	-0,077225734	0,218662624	0	N.A.	0
ZXDA	-0,077261212	0,05476131	0	N.A.	0
CBFA2T3	-0,07727357	-0,015604291	0	N.A.	0
DDX31	-0,077350072	-0,10562969	0	N.A.	0
PAX6	-0,077413896	-0,189520429	0	N.A.	0
BTG2	-0,077448658	-0,129270722	0	N.A.	0
PEBP4	-0,077451687		0	N.A.	0
PGRMC2	-0,077458633	0,270432697	0	N.A.	0
CBLN1	-0,077480065	-0,028853954	0	N.A.	0
MEX3B	-0,077499689	0,037939702	0	N.A.	0

RP11-307C12	-0,077503371	-0,01697961	0	N.A.	0
ISL2	-0,077525474	-0,001750179	0	N.A.	0
DCAF4L1	-0,07754807	0,132984116	0	N.A.	0
ITGB3	-0,077570331	0,333407989	0	N.A.	0
ATXN3	-0,077673746	0,230833211	0	N.A.	0
LRP4-AS1	-0,07767473	-0,087280508	0	N.A.	0
RP11-1055B8	-0,077702299	0,06403341	0	N.A.	0
H1FX	-0,077706124	0,19545181	0	N.A.	0
ASAH2	-0,077707402	-0,07282511	0	N.A.	0
SIX1	-0,077711153	-0,065866323	0	N.A.	0
LINC01198	-0,077755801		0	N.A.	0
RAB26	-0,077816642	-0,024587471	0	N.A.	0
PRAF2	-0,077830127	-0,123889023	0	N.A.	0
NOTUM	-0,07785051	0,099901943	0	N.A.	0
COL24A1	-0,077860984		0	N.A.	0
AP001626.2	-0,077862891	0,149894727	0	N.A.	0
WDR24	-0,077891891	-0,290830375	0	N.A.	0
CAMKV	-0,077897472		0	N.A.	0
KLHDC8B	-0,077958201	0,047668832	0	N.A.	0
SIAE	-0,077960524	-0,028579742	0	N.A.	0
AC062029.1	-0,077985356	0,126215811	0	N.A.	0
TSR3	-0,077991273	-0,11166232	0	N.A.	0
SSR1	-0,078013077	0,042491551	0	N.A.	0
DEPDC7	-0,078025834	0,158884089	0	N.A.	0
RP11-626G11	-0,078037044	-0,212790825	0	N.A.	0
RP11-425D10	-0,078080289		0	N.A.	0
SUGP2	-0,078116363	0,098080554	0	N.A.	0
ZNF177	-0,078158049		0	N.A.	0
SLC45A1	-0,078242158	0,006092096	0	N.A.	0
ARL6	-0,07824235	-0,158220918	0	N.A.	0
HOXA6	-0,07828337	0,012503699	0	N.A.	0
IGLV2-8	-0,078410789	-0,075683943	0	N.A.	0
TXLNB	-0,078429427		0	N.A.	0

RP11-173P15	-0,0784651	-0,055358158	0	N.A.	0
RBBP8NL	-0,078486439	0,078257054	0	N.A.	0
RP11-99A1.2	-0,078501758	0,226985603	0	N.A.	0
PDGFRL	-0,078571843	-0,132144746	0	N.A.	0
BLOC1S1	-0,078594688	-0,229317707	0	N.A.	0
RP11-996F15	-0,078600963	-0,040607495	0	N.A.	0
ZNF665	-0,078615902	-0,121407539	0	N.A.	0
GID4	-0,078618932	-0,297542579	0	N.A.	0
SLC16A10	-0,07861918	-0,156962053	0	N.A.	0
CALD1	-0,078640752	-0,347420536	0	N.A.	0
CTD-2566J3.1	-0,078676356	-0,100877275	0	N.A.	0
RBM39	-0,07872818	0,245464564	0	N.A.	0
NUP98	-0,078772595	0,042331099	0	N.A.	0
IQCH	-0,078829684	0,116980169	0	N.A.	0
BX004987.4	-0,078905337	-0,09034469	0	N.A.	0
P3H3	-0,078907867	-0,263975222	0	N.A.	0
C2CD4A	-0,078911243	-0,119795027	0	N.A.	0
CCDC174	-0,078956109	0,217420155	0	N.A.	0
ZNF254	-0,078977196	-0,081465218	0	N.A.	0
RP11-498B4.	-0,078989633	-0,111383691	0	N.A.	0
RASD2	-0,078997544	0,056162911	0	N.A.	0
CA7	-0,079051399	-0,010242668	0	N.A.	0
NGLY1	-0,079058485	0,085175148	0	N.A.	0
CHERP	-0,079069214	0,050569124	0	N.A.	0
RP5-894D12.	-0,079077468	-0,058066948	0	N.A.	0
RP11-16N11.	-0,079112941	-0,188458201	0	N.A.	0
SLC35D3	-0,07913595	0,039119998	0	N.A.	0
RP11-482D24	-0,079350476		0	N.A.	0
DIABLO	-0,079382595	-0,049158618	0	N.A.	0
KIAA1328	-0,079412253	0,15521023	0	N.A.	0
FGF17	-0,079427816	-0,019223599	0	N.A.	0
OTUD6A	-0,079432767		0	N.A.	0
ZBTB8A	-0,079439037	0,045455915	0	N.A.	0

SGCE	-0,079440247	-0,124401043	0	N.A.	0
C16orf86	-0,079460857	0,119390294	0	N.A.	0
TLX1	-0,079482972	0,307727797	0	N.A.	0
TOX2	-0,07954673	0,230240568	0	N.A.	0
INHBE	-0,079554389		0	N.A.	0
LL09NC01-25	-0,07958705	-0,283727266	0	N.A.	0
CHMP1A	-0,079604208	0,189276184	0	N.A.	0
ICA1L	-0,079616384	-0,049311705	0	N.A.	0
ANKH	-0,079623653	-0,092644052	0	N.A.	0
CTD-2342J14	-0,079687192	0,030063743	0	N.A.	0
HVCN1	-0,079735421	-0,087172191	0	N.A.	0
FBXO41	-0,079775217	-0,050246671	0	N.A.	0
STK31	-0,07984843	-0,230228206	0	N.A.	0
IQCE	-0,079916825	-0,078596214	0	N.A.	0
WDR55	-0,079928163	0,115325975	0	N.A.	0
RP1-102E24.	-0,080015866	0,036387657	0	N.A.	0
MRC2	-0,080029706	-0,031814698	0	N.A.	0
SERINC4	-0,080046286	-0,008219694	0	N.A.	0
SCRIB	-0,080053303	0,163880366	0	N.A.	0
KCTD12	-0,080130626	-0,065540534	0	N.A.	0
OGDHL	-0,080200006	-0,387182386	0	N.A.	0
RP11-345J4.5	-0,080203602	-0,316731107	0	N.A.	0
USP16	-0,080222636	0,03599624	0	N.A.	0
RP11-396C23	-0,080331929	-0,106178766	0	N.A.	0
AC084219.4	-0,080335506	-0,048499301	0	N.A.	0
C3orf17	-0,080337959	0,168623844	0	N.A.	0
SYTL3	-0,080361892	-0,158539195	0	N.A.	0
SULT4A1	-0,080362709		0	N.A.	0
VWA3A	-0,080377511	-0,035099317	0	N.A.	0
ETV5	-0,080388488	-0,035334117	0	N.A.	0
FLJ21408	-0,080426714	0,037032811	0	N.A.	0
INHBA-AS1	-0,080469721		0	N.A.	0
OSCAR	-0,080502312	0,079546603	0	N.A.	0

RP11-126O1.	-0,080547866	0,219902451	0	N.A.	0
CTD-2587H24	-0,0806234	0,092441981	0	N.A.	0
AC008746.12	-0,080625659	0,129375088	0	N.A.	0
REG3G	-0,080658851		0	N.A.	0
RP11-680F8.1	-0,080659392	0,035761081	0	N.A.	0
KLK2	-0,080669678	0,367082782	0	N.A.	0
SMARCC2	-0,080679299	0,362066603	0	N.A.	0
RP11-848P1.	-0,080729626	-0,044199663	0	N.A.	0
NEK8	-0,080776469	0,27771907	0	N.A.	0
COLCA2	-0,080839572	-0,024720173	0	N.A.	0
ADAT1	-0,08086212	-0,214952222	0	N.A.	0
GRIP2	-0,080908698		0	N.A.	0
CTB-40H15.4	-0,080928106		0	N.A.	0
ZFP14	-0,080943333	0,160124112	0	N.A.	0
RP1-102E24.	-0,08107939	-0,028424768	0	N.A.	0
ZNF443	-0,081132478	0,129143336	0	N.A.	0
RP11-469H8.	-0,081134881	0,005302043	0	N.A.	0
RP13-714J12	-0,081146234	0,248981415	0	N.A.	0
CTSF	-0,081155153	-0,009406808	0	N.A.	0
PGGT1B	-0,081177011	0,049279277	0	N.A.	0
TOX	-0,08121501	-0,153470947	0	N.A.	0
CTB-5E10.3	-0,081219372	0,374153739	0	N.A.	0
RP11-120K24	-0,081228234	-0,18568356	0	N.A.	0
DOPEY1	-0,081299497	0,109664121	0	N.A.	0
P2RX5	-0,081432715	-0,318485275	0	N.A.	0
EXOC3-AS1	-0,081451291	0,273966537	0	N.A.	0
CTD-2626G1	-0,081481039	-0,189420204	0	N.A.	0
C1orf127	-0,081493348		0	N.A.	0
RP11-20E24.	-0,081513852	-0,261463662	0	N.A.	0
THBS4	-0,081521671	0,01535409	0	N.A.	0
RP11-443B20	-0,081541261	0,38450048	0	N.A.	0
RP11-535A5.	-0,081571181		0	N.A.	0
FNDC4	-0,08157293	-0,205518724	0	N.A.	0

STARD9	-0,081645947	0,006551566	0	N.A.	0
RAB11FIP3	-0,081698783	0,130984601	0	N.A.	0
ANKRD17	-0,081702965	0,209109634	0	N.A.	0
ZNRF3-AS1	-0,081711048	-0,056694524	0	N.A.	0
ETHE1	-0,081750138	0,120229077	0	N.A.	0
AC004791.2	-0,081757591		0	N.A.	0
RP11-290H9.	-0,081845903	0,010004181	0	N.A.	0
ZNF211	-0,081848686	-0,010757137	0	N.A.	0
CNPY1	-0,081861221	0,197301238	0	N.A.	0
ENPP2	-0,081897086	0,225457976	0	N.A.	0
RP11-849F2.	-0,081907184		0	N.A.	0
RP1-261G23.	-0,081928043	0,294435373	0	N.A.	0
ASL	-0,081934772	0,247536611	0	N.A.	0
RP11-620J15	-0,082018833	-0,306253156	0	N.A.	0
EPM2A	-0,082036233	0,002456836	0	N.A.	0
SLC25A20	-0,082050709	-0,085775496	0	N.A.	0
HIST1H2BK	-0,082051358	0,01828579	0	N.A.	0
SNHG22	-0,082068348	0,091861569	0	N.A.	0
SLC38A3	-0,082106898		0	N.A.	0
CLN5	-0,082116847	0,268511468	0	N.A.	0
GTF2F1	-0,082124894	0,116650018	0	N.A.	0
NTS	-0,082125246	-0,239189159	0	N.A.	0
RP5-855F16.	-0,082156382	-0,250029806	0	N.A.	0
DNAJC18	-0,082195311	0,063450875	0	N.A.	0
COLCA1	-0,082214753	-0,232602615	0	N.A.	0
CTD-2020K1	-0,082237514	-0,08998221	0	N.A.	0
RP11-290F24	-0,08223955	0,030374935	0	N.A.	0
AC145212.2	-0,082247651	-0,084022609	0	N.A.	0
ZNF184	-0,082279722	-0,261972132	0	N.A.	0
CTC-332L22.	-0,082309795	0,025198908	0	N.A.	0
SAMD14	-0,08234466	0,220191874	0	N.A.	0
TUBB1	-0,08234524		0	N.A.	0
FHOD3	-0,082347248	-0,127413632	0	N.A.	0

SACM1L	-0,082362109	-0,02804	0	N.A.	0
CYHR1	-0,082376295	0,432621282	0	N.A.	0
RP11-181C3.	-0,082450959	-0,17897879	0	N.A.	0
PRR19	-0,082455943	0,155119466	0	N.A.	0
MS4A4A	-0,082456709	-0,341802228	0	N.A.	0
C19orf44	-0,082490924	0,092665894	0	N.A.	0
CCDC71	-0,082493349	-0,168216671	0	N.A.	0
NPAT	-0,082573304	0,353257204	0	N.A.	0
CMC4	-0,082578146	-0,156512036	0	N.A.	0
COQ10A	-0,082597971	0,211000446	0	N.A.	0
ELOVL5	-0,082676873	-0,123920004	0	N.A.	0
TANGO6	-0,082700691	-0,038555276	0	N.A.	0
LENG1	-0,082734159	0,166278992	0	N.A.	0
XX-FW80269	-0,082749537		0	N.A.	0
PAM16	-0,082802758	-0,377088242	0	N.A.	0
BICD1	-0,082814109	0,098431789	0	N.A.	0
PLCG1-AS1	-0,08284868	-0,101723004	0	N.A.	0
IRF2BPL	-0,082981069	0,114306832	0	N.A.	0
DNALI1	-0,083002996	-0,250483245	0	N.A.	0
ZNF709	-0,083054116	-0,122045274	0	N.A.	0
GALNT16	-0,083059168		0	N.A.	0
STXBP4	-0,083166647	0,027022783	0	N.A.	0
ATP5S	-0,083183868	-0,022815431	0	N.A.	0
LSM11	-0,083297735	-0,114382405	0	N.A.	0
PPP2R4	-0,083321335	-0,077933064	0	N.A.	0
SLC18A2	-0,083321952	0,086408698	0	N.A.	0
SMYD4	-0,083404761	0,10295633	0	N.A.	0
RP11-380N8.	-0,083411359	0,127693305	0	N.A.	0
APCDD1	-0,083442263	-0,220557307	0	N.A.	0
IFITM1	-0,083451997	-0,156882253	0	N.A.	0
ARIH2OS	-0,083481896	-0,000715627	0	N.A.	0
CTD-3131K8.	-0,083492142	-0,080340317	0	N.A.	0
RP11-123B3.	-0,08349236		0	N.A.	0

DEPDC4	-0,083497622	-0,084115929	0	N.A.	0
HIST1H4A	-0,083519445	0,022160425	0	N.A.	0
SCGB1B2P	-0,083522058	-0,077829431	0	N.A.	0
IDNK	-0,083528257	-0,012454216	0	N.A.	0
TRAPPC2	-0,083549119	-0,080404263	0	N.A.	0
IFT80	-0,083551589	-0,19694137	0	N.A.	0
FAM154B	-0,083557691	-0,236954426	0	N.A.	0
INPP5B	-0,083566919	0,006962911	0	N.A.	0
SEC22A	-0,083609945	-0,158410102	0	N.A.	0
CD99L2	-0,083612767	-0,176375733	0	N.A.	0
NME5	-0,083623145	0,172824626	0	N.A.	0
RP11-360L9.4	-0,083654191	-0,07191456	0	N.A.	0
LINC00639	-0,083674593	-0,15384164	0	N.A.	0
ULK4	-0,083682569	-0,021690546	0	N.A.	0
TEX12	-0,083699787	-0,23114849	0	N.A.	0
RP11-111J6.2	-0,083785252	-0,080757973	0	N.A.	0
RP11-231G3.	-0,083787123	0,132641239	0	N.A.	0
FOS	-0,083795064	0,317466015	0	N.A.	0
GRIN2C	-0,083806549	-0,07201275	0	N.A.	0
KPNA5	-0,083815924	0,097521519	0	N.A.	0
ZNF853	-0,083833633		0	N.A.	0
NOS3	-0,083859594	0,042510795	0	N.A.	0
STXBP1	-0,083870195	0,338675437	0	N.A.	0
PGK1	-0,083871559	0,083877009	0	N.A.	0
RP11-136K7.	-0,083873978		0	N.A.	0
RP4-605O3.4	-0,08392366	0,233049543	0	N.A.	0
CRTC1	-0,083939982	-0,131797904	0	N.A.	0
PRRT3	-0,084042152	0,187761518	0	N.A.	0
ZBED1	-0,084042395	-0,123034247	0	N.A.	0
RP3-337H4.1	-0,084059592		0	N.A.	0
ABHD1	-0,084129488	-0,135586257	0	N.A.	0
MAN2A2	-0,0841435	0,045847167	0	N.A.	0
CRACR2A	-0,084144106	-0,189594616	0	N.A.	0

RP11-320M2	-0,084163251	-0,108555264	0	N.A.	0
RP6-65G23.3	-0,084202384	-0,153352813	0	N.A.	0
RP4-755D9.1	-0,084240644		0	N.A.	0
BBS1	-0,084259614	-0,246536634	0	N.A.	0
CIART	-0,084289687	0,186392179	0	N.A.	0
RP11-294J22	-0,08434395	-0,088856695	0	N.A.	0
NLRP11	-0,084373349		0	N.A.	0
PCSK9	-0,084378695	0,218669368	0	N.A.	0
SYT11	-0,084387163	-0,132479793	0	N.A.	0
LINC01117	-0,084429531		0	N.A.	0
MATN1	-0,084442501		0	N.A.	0
ZNF613	-0,084455975	-0,151834212	0	N.A.	0
HIST1H2AJ	-0,084485601	-0,104304701	0	N.A.	0
AP006621.5	-0,084520432	-0,025764563	0	N.A.	0
MYRIP	-0,084537558	-0,158282958	0	N.A.	0
MED7	-0,084596292	-0,246778567	0	N.A.	0
PTGER2	-0,084598654	-0,187914196	0	N.A.	0
SBK1	-0,084607223	-0,196139358	0	N.A.	0
CTC-471J1.11	-0,084649165	-0,324481836	0	N.A.	0
ZKSCAN2	-0,084752874	0,055171802	0	N.A.	0
CTD-2256P15	-0,084767661	-0,046153732	0	N.A.	0
RP11-664D1.	-0,084773706		0	N.A.	0
SAMD10	-0,084808122	-0,092651279	0	N.A.	0
SPTBN2	-0,084863225	0,348709407	0	N.A.	0
RP11-738E22	-0,084900947		0	N.A.	0
GRB14	-0,084908567	-0,003215956	0	N.A.	0
C5orf28	-0,084916203	-0,162967056	0	N.A.	0
CCNT2-AS1	-0,084967641	0,063292504	0	N.A.	0
MSH4	-0,084970389	0,109800919	0	N.A.	0
PDX1	-0,084977427	-0,103715096	0	N.A.	0
RP3-466P17.	-0,084982846		0	N.A.	0
CTA-293F17.	-0,08500499	-0,26042629	0	N.A.	0
C10orf82	-0,085041143	0,074439041	0	N.A.	0

RASSF10	-0,085049209	-0,191546227	0	N.A.	0
ZNF519	-0,085063752	-0,102773211	0	N.A.	0
RP4-545L17.1	-0,085100701	-0,058588263	0	N.A.	0
CLDND2	-0,085133781	-0,081690088	0	N.A.	0
CTD-2553L13	-0,085135643	-0,083929656	0	N.A.	0
DMRT2	-0,085137423	-0,155224171	0	N.A.	0
CTD-2410N18	-0,085141092	-0,010759682	0	N.A.	0
CYP2B6	-0,085144469	-0,109585727	0	N.A.	0
SPATA7	-0,08518219	-0,127737138	0	N.A.	0
ZNF490	-0,08519297	0,025899702	0	N.A.	0
RAB3D	-0,085285524	-0,143711551	0	N.A.	0
TMEM141	-0,085357809	0,170333793	0	N.A.	0
RP11-30P6.6	-0,085389569	-0,025971063	0	N.A.	0
HSPA1B	-0,085390883	0,12233952	0	N.A.	0
RP5-1121E10	-0,085499861	0,015084526	0	N.A.	0
CD70	-0,085509635	-0,09652867	0	N.A.	0
RP11-736K20	-0,085526566	-0,140443697	0	N.A.	0
ROBO1	-0,085543726	0,248785216	0	N.A.	0
CTB-32O4.2	-0,085564464	0,146388921	0	N.A.	0
RP11-1275H2	-0,085601129	0,176265522	0	N.A.	0
RP11-6N17.6	-0,08560942	0,17238512	0	N.A.	0
ICK	-0,085618132	-0,08393627	0	N.A.	0
WRNIP1	-0,085671084	-0,118901725	0	N.A.	0
FKTN	-0,085686301	-0,026460005	0	N.A.	0
NXF1	-0,085723239	0,088612286	0	N.A.	0
RP11-497G19	-0,085731048		0	N.A.	0
RP11-539I5.1	-0,085748609	-0,120880055	0	N.A.	0
FBXO36	-0,085766687	-0,006054332	0	N.A.	0
C11orf71	-0,085775395	0,018299704	0	N.A.	0
LRMP	-0,08577941	-0,091601248	0	N.A.	0
ZNF415	-0,085802163	-0,189563646	0	N.A.	0
CCL11	-0,08582552	-0,13256801	0	N.A.	0
MED9	-0,085854073	-0,21636372	0	N.A.	0

SLC7A4	-0,085871696	0,226919637	0	N.A.	0
RBM34	-0,085879733	0,015735675	0	N.A.	0
MPP2	-0,085883569		0	N.A.	0
RP11-247L20	-0,085981868	-0,172151894	0	N.A.	0
TMEM62	-0,085984306	0,105291659	0	N.A.	0
AC099850.1	-0,086082703	-0,097802457	0	N.A.	0
MYOM2	-0,086130886	0,060368206	0	N.A.	0
NELFB	-0,086139353	0,149917597	0	N.A.	0
HIST4H4	-0,086219457	-0,158649099	0	N.A.	0
EXOG	-0,086223401	0,268637706	0	N.A.	0
RP11-499F3.:	-0,08627345		0	N.A.	0
TRBC2	-0,08632265	-0,185996289	0	N.A.	0
FIRRE	-0,086323155	0,123064926	0	N.A.	0
NSUN7	-0,086350782	-0,342739996	0	N.A.	0
KIAA1456	-0,086355955	0,032983962	0	N.A.	0
SERF2	-0,086400331	-0,280849697	0	N.A.	0
ARHGAP19	-0,086475982	-0,173068971	0	N.A.	0
ZNF674-AS1	-0,086476394	-0,050814576	0	N.A.	0
ADAM15	-0,086503465	0,366128756	0	N.A.	0
ZNF747	-0,086531794	0,000269079	0	N.A.	0
CFL2	-0,086569005	-0,065853851	0	N.A.	0
GLDC	-0,086584787	0,176638557	0	N.A.	0
RP11-545D19	-0,08658657	0,272800237	0	N.A.	0
DMD	-0,086640645	0,000225264	0	N.A.	0
RP5-899E9.1	-0,08664591		0	N.A.	0
HIST1H4B	-0,08664962	-0,110069828	0	N.A.	0
CCDC74B	-0,086660798		0	N.A.	0
C16orf95	-0,086716329	-0,049714542	0	N.A.	0
GPC2	-0,086812369	0,008056528	0	N.A.	0
LINC00526	-0,086831558	-0,396341226	0	N.A.	0
UGT2B15	-0,086892071	0,247310454	0	N.A.	0
TGM3	-0,086975169	-0,160197039	0	N.A.	0
PCCA	-0,086980618	-0,217287988	0	N.A.	0

LINC00114	-0,087055058	-0,185022828	0	N.A.	0
FAM76B	-0,087073782	0,113120464	0	N.A.	0
LONP1	-0,087102925	0,153273782	0	N.A.	0
ABCF3	-0,08714412	0,150516111	0	N.A.	0
FRS3	-0,087151415	-0,234274284	0	N.A.	0
STEAP1	-0,087165936	0,273039413	0	N.A.	0
DDX5	-0,087198552	0,262997613	0	N.A.	0
NMUR1	-0,087210183	0,063444968	0	N.A.	0
DKFZP434I07	-0,087238179	0,122186356	0	N.A.	0
ZNF77	-0,087309762	0,027286103	0	N.A.	0
ATP6V0B	-0,087410077	0,277228372	0	N.A.	0
TMEM203	-0,087435601	0,1679572	0	N.A.	0
SRD5A1	-0,087499142	-0,044541817	0	N.A.	0
SPPL2B	-0,087519068	0,321854849	0	N.A.	0
ZNF711	-0,087543413	-0,064433391	0	N.A.	0
TRIM46	-0,0875528	0,010463906	0	N.A.	0
USB1	-0,087565278	-0,152041229	0	N.A.	0
SMIM10	-0,087614139	-0,367901167	0	N.A.	0
CDHR3	-0,087623331	0,064470843	0	N.A.	0
GRTP1	-0,087625194	0,215716055	0	N.A.	0
RP11-422P24	-0,087647664	-0,193740329	0	N.A.	0
ZNF124	-0,087723528	-0,210621279	0	N.A.	0
RP11-195B3.	-0,087736087		0	N.A.	0
BCL7A	-0,087778964	-0,21339539	0	N.A.	0
RND2	-0,087831284	-0,060731603	0	N.A.	0
SCGN	-0,088011126		1	none	0
ZNF589	-0,08811788	-0,022584752	0	N.A.	0
CENPBD1	-0,088184919	-0,104587799	0	N.A.	0
SPATA24	-0,088203942	-0,132470475	0	N.A.	0
ANKRD36	-0,08822171	-0,101631327	0	N.A.	0
APOE	-0,088238094	-0,264054525	0	N.A.	0
DCAF15	-0,088272361	-0,153945892	0	N.A.	0
RP5-1159O4.	-0,088327054	0,198470035	0	N.A.	0

TSPO	-0,088335913	0,176268154	0	N.A.	0
POMC	-0,088341874	0,333902142	0	N.A.	0
KCNE4	-0,088354052	0,214203606	0	N.A.	0
RP11-181G12	-0,088384689	0,022793764	0	N.A.	0
OVOL3	-0,088474618	0,240972955	0	N.A.	0
DDHD2	-0,088531901	0,046174011	0	N.A.	0
PLBD1-AS1	-0,088540399	0,200859244	0	N.A.	0
BTBD17	-0,088574646	0,239363182	0	N.A.	0
FAM179B	-0,08860971	0,100403235	0	N.A.	0
C22orf46	-0,088677898	0,05348106	0	N.A.	0
NEXN	-0,088708269	-0,12072352	0	N.A.	0
AC003075.4	-0,088766406	-0,153419294	0	N.A.	0
RP11-79P5.9	-0,088881409	0,098077411	0	N.A.	0
CNNM3	-0,088895478	0,137980749	0	N.A.	0
TMEM67	-0,088905223	0,249297265	0	N.A.	0
DDX39B-AS1	-0,088934875	0,049031941	0	N.A.	0
CTBP1-AS2	-0,088960788	-1,44841E-05	0	N.A.	0
FGF8	-0,088973946	0,264041094	0	N.A.	0
C17orf77	-0,089012048	-0,179729665	0	N.A.	0
RP11-307P5.	-0,089057682	0,089170759	0	N.A.	0
NXT2	-0,089073133	-0,465973636	0	N.A.	0
HSPA1A	-0,089087998	0,180886641	0	N.A.	0
GTF2H4	-0,089118201	0,063735614	0	N.A.	0
PHC1	-0,089145412	0,114677892	0	N.A.	0
CEP290	-0,089173031	0,192073362	0	N.A.	0
ZFY	-0,089206577	-0,167121357	0	N.A.	0
GPR137C	-0,08925139		0	N.A.	0
ACTL10	-0,089266079	0,040127302	0	N.A.	0
KCNMB2-AS1	-0,089266302	0,048183	0	N.A.	0
XXbac-B444P	-0,089373394	0,134571427	0	N.A.	0
TMEM194B	-0,089373671	-0,323108887	0	N.A.	0
AC013463.2	-0,089441444		0	N.A.	0
TTC4	-0,089442192	-0,031542878	0	N.A.	0

CDPF1	-0,089521213	0,030755021	0	N.A.	0
ICOS	-0,089662713	0,028333334	0	N.A.	0
RP11-547D24	-0,089663275		0	N.A.	0
VWA5B2	-0,089708096	0,004537914	0	N.A.	0
KCTD15	-0,089759492	-0,1596563	0	N.A.	0
GPT2	-0,089765251	-0,123159751	0	N.A.	0
MED6	-0,089815034	-0,020991083	0	N.A.	0
ADAM22	-0,089831016	-0,174301094	0	N.A.	0
NUPL1	-0,089836004	0,19045615	0	N.A.	0
PDIK1L	-0,089836633	-0,182568994	0	N.A.	0
CEP68	-0,089873048	-0,217644382	0	N.A.	0
TNN	-0,089930812	0,010348121	0	N.A.	0
FAM13C	-0,089954331		0	N.A.	0
PHIP	-0,089980635	0,192461171	0	N.A.	0
SV2A	-0,090005876		0	N.A.	0
SNX20	-0,090028239	-0,222420462	0	N.A.	0
AC142472.6	-0,090055845	-0,1951159	0	N.A.	0
ANGEL1	-0,090133585	0,03593714	0	N.A.	0
INTS9	-0,090162628	-0,022122262	0	N.A.	0
APPBP2	-0,090163111	-0,041255384	0	N.A.	0
DTWD2	-0,090189846	-0,081824882	0	N.A.	0
RP11-403I13	-0,090197163	0,094935395	0	N.A.	0
RABGGTA	-0,090212173	0,022378331	0	N.A.	0
FAM122B	-0,090214369	-0,024233713	0	N.A.	0
PLA2G7	-0,09027453	0,277770782	0	N.A.	0
ATR	-0,090275726	-0,141352796	0	N.A.	0
RP11-527D7.	-0,090367621		0	N.A.	0
IFT27	-0,09039797	0,02067136	0	N.A.	0
POMGNT2	-0,090415316	-0,103212002	0	N.A.	0
AP001462.6	-0,090441684	0,332962447	0	N.A.	0
PAN3-AS1	-0,090446905	-0,03973758	0	N.A.	0
RPRD2	-0,090483902	0,261660332	0	N.A.	0
TSGA10	-0,090507974	-0,221299505	0	N.A.	0

C6orf52	-0,090514244	-0,043994944	0	N.A.	0
ACOT8	-0,090519862	-0,221774728	0	N.A.	0
NUDT16	-0,090533627	-0,042804398	0	N.A.	0
JAKMIP1	-0,090624919		0	N.A.	0
JAM2	-0,09064259	-0,045016488	0	N.A.	0
C1orf27	-0,090710669	-0,120222216	0	N.A.	0
UBE2L6	-0,090760196	-0,330342727	0	N.A.	0
AK7	-0,09079246	0,063139261	0	N.A.	0
TMEM208	-0,090793837	0,430283966	0	N.A.	0
PAPSS1	-0,090810573	-0,064657918	0	N.A.	0
RP11-379H18	-0,090866091	0,14292389	0	N.A.	0
PGAP1	-0,090900179	-0,024568745	0	N.A.	0
ARFIP1	-0,090920343	0,065743945	0	N.A.	0
RP11-545I5.3	-0,090926504	0,075534833	0	N.A.	0
ZNF287	-0,09094435	0,250017843	0	N.A.	0
BOC	-0,09099058	0,156137809	0	N.A.	0
TRAT1	-0,091061361	-0,376712579	0	N.A.	0
SLC48A1	-0,091073114	0,05959144	0	N.A.	0
RCN3	-0,091176057	-0,014006017	0	N.A.	0
MAP3K12	-0,091177863	-0,077725421	0	N.A.	0
KIAA1324L	-0,09126593	-0,09386671	0	N.A.	0
GTF3C2	-0,091276805	0,126744125	0	N.A.	0
KIAA1147	-0,091313974	0,025869258	0	N.A.	0
RHPN1	-0,091318296	-0,156646761	0	N.A.	0
SRSF12	-0,09153488	-0,125461221	0	N.A.	0
ISL1	-0,091603399	-0,008689685	0	N.A.	0
SEPT3	-0,091630238	0,064409311	0	N.A.	0
ZNF436-AS1	-0,091654051	0,037132358	0	N.A.	0
RP11-386I14	-0,091693437	0,091767191	0	N.A.	0
TLK1	-0,091718181	0,253788817	0	N.A.	0
HARS2	-0,091724727	0,251271186	0	N.A.	0
CCDC60	-0,091724972	-0,492175277	0	N.A.	0
C6orf120	-0,091757197	0,002793876	0	N.A.	0

FAM213A	-0,091757936	0,094182877	0	N.A.	0
DDX26B-AS1	-0,091804484	0,265846655	0	N.A.	0
ZNF329	-0,091812313	0,053840267	0	N.A.	0
FEZF1	-0,091886483	-0,13325094	0	N.A.	0
RP4-777L9.2	-0,091911495	-0,287894547	0	N.A.	0
TROVE2	-0,091911999	0,028283081	0	N.A.	0
MIAT	-0,091935294	-0,163818747	0	N.A.	0
MIR646HG	-0,091940028		0	N.A.	0
CTD-2587H1!	-0,091982707	0,226985603	0	N.A.	0
C11orf21	-0,091990716		0	N.A.	0
ZBTB24	-0,092032457	-0,111282825	0	N.A.	0
CD81-AS1	-0,092049844		0	N.A.	0
HDGFRP3	-0,092067206	-0,12508846	0	N.A.	0
PTCHD4	-0,092117222	-0,338316193	0	N.A.	0
TRIM73	-0,09211814	-0,199114253	0	N.A.	0
LYG2	-0,092147491		0	N.A.	0
RP1-27K12.2	-0,092193956		0	N.A.	0
AC012506.4	-0,092228438		0	N.A.	0
ZNF594	-0,092242589	0,009804659	0	N.A.	0
SYAP1	-0,092276658	0,102929545	0	N.A.	0
RP11-713M1	-0,092424389	-0,20612478	0	N.A.	0
ATP10D	-0,092439941	0,079965368	0	N.A.	0
TMEM210	-0,092445181	-0,010114837	0	N.A.	0
PEX1	-0,09249512	-0,005720342	0	N.A.	0
FRMD6-AS1	-0,092496234	-0,00597368	0	N.A.	0
LINC01534	-0,092498039	-0,307479721	0	N.A.	0
RP11-798M1	-0,092522539	-0,127857404	0	N.A.	0
RSPH14	-0,092551399	-0,291603812	0	N.A.	0
ZNF595	-0,092562715	0,280586706	0	N.A.	0
GIMAP7	-0,092569518	-0,286637273	0	N.A.	0
LARP4	-0,092578644	0,011654971	0	N.A.	0
RP4-680D5.2	-0,092601983	0,010096112	0	N.A.	0
ZNF583	-0,092658927	-0,203013925	0	N.A.	0

AC104820.2	-0,092659693	-0,088758096	0	N.A.	0
RTN1	-0,092795296	-0,274394986	0	N.A.	0
CASS4	-0,092813314		0	N.A.	0
GP2	-0,092832367	0,001005435	0	N.A.	0
EYA1	-0,092844764	-0,293304492	0	N.A.	0
LINC01572	-0,092927124	-0,06489314	0	N.A.	0
MLLT11	-0,092927297	-0,223846627	0	N.A.	0
CHD7	-0,092962146	0,141826738	0	N.A.	0
KSR2	-0,092988312	-0,087119737	0	N.A.	0
TRIM72	-0,092995471	-0,048029085	0	N.A.	0
HAMP	-0,093037469		0	N.A.	0
LINC01545	-0,093092728		0	N.A.	0
LY6H	-0,093153322	-0,149601982	0	N.A.	0
HGFAC	-0,093204522	-0,085075356	0	N.A.	0
RAB1A	-0,093226047	0,235365112	0	N.A.	0
RP13-497K6.	-0,093259605	-0,18769134	0	N.A.	0
KDM4A-AS1	-0,093287251	-0,01153483	0	N.A.	0
CDK12	-0,09331287	-0,038117395	0	N.A.	0
CPLX2	-0,093412216	0,171081515	0	N.A.	0
RP11-626G1	-0,093415244		0	N.A.	0
RP11-395N3.	-0,093418918	-0,077098527	0	N.A.	0
VPS52	-0,093427551	-0,073673484	0	N.A.	0
SCT	-0,093439719	0,326702557	0	N.A.	0
RABEP2	-0,093478283	0,190770934	0	N.A.	0
AC024592.9	-0,093520495	0,020781782	0	N.A.	0
ERP44	-0,093554709	0,069891697	0	N.A.	0
TBPL1	-0,093560481	-0,000197845	0	N.A.	0
MFI2-AS1	-0,093579666	0,04631249	0	N.A.	0
FGFBP2	-0,093651423		0	N.A.	0
B3GALNT1	-0,093660529	0,084916981	0	N.A.	0
RP11-268P4.	-0,093661739	0,153481055	0	N.A.	0
PRR14	-0,093688244	0,129930272	0	N.A.	0
AKR1C2	-0,093693641	0,046166811	0	N.A.	0

UBQLN4	-0,09379069	-0,123751508	0	N.A.	0
AMER1	-0,093881956	-0,042617804	0	N.A.	0
RP11-432J24	-0,093960795	0,072916664	0	N.A.	0
IQCK	-0,094023023	-0,179621122	0	N.A.	0
SYCE2	-0,094033183	-0,148428596	0	N.A.	0
ADH1A	-0,094044225		0	N.A.	0
CHUK	-0,094060991	-0,005436715	0	N.A.	0
ST8SIA1	-0,094062561		0	N.A.	0
POU2AF1	-0,094080933	0,029133301	0	N.A.	0
CTC-428H11.	-0,094149402	-0,228600847	0	N.A.	0
ZSWIM7	-0,09416933	-0,224714852	0	N.A.	0
FAM110A	-0,094197432	0,069484975	0	N.A.	0
PDIA5	-0,094210776	0,108817557	0	N.A.	0
COL13A1	-0,094216673	0,311407799	0	N.A.	0
EMX2OS	-0,094278077	-0,038726245	0	N.A.	0
COPS2	-0,09427826	-0,114101809	0	N.A.	0
RCBTB2	-0,094305058	-0,259106434	0	N.A.	0
FANCM	-0,094310678	-0,013637918	0	N.A.	0
RP4-798A10.	-0,094323307	0,020394221	0	N.A.	0
TMEM168	-0,094327128	0,186938966	0	N.A.	0
MYCNOS	-0,094332921	0,031784317	0	N.A.	0
RP1-239B22.	-0,094382364	-0,066864238	0	N.A.	0
TADA1	-0,094393163	-0,122537995	0	N.A.	0
RP11-452L6.7	-0,094394484	0,003377415	0	N.A.	0
CRIP3	-0,094418934	-0,133315397	0	N.A.	0
TRIL	-0,094425894	0,212849304	0	N.A.	0
CTD-2256P15	-0,094427458	-0,323038516	0	N.A.	0
KLK15	-0,094484189	-0,086131314	0	N.A.	0
RP11-258C19	-0,094494874	0,128301732	0	N.A.	0
SMAD5-AS1	-0,094516765	0,063950191	0	N.A.	0
LL22NC03-32	-0,09456622	-0,056461212	0	N.A.	0
ASGR1	-0,094605722	0,019409477	0	N.A.	0
RBL1	-0,094640375	-0,10155607	0	N.A.	0

RP11-717F1.1	-0,094681691	-0,168747899	0	N.A.	0
KLHL7-AS1	-0,094728569		0	N.A.	0
GMPPA	-0,094736563	0,079619406	0	N.A.	0
ZDHHC23	-0,094762645	0,029765142	0	N.A.	0
LINC00864	-0,094774703		0	N.A.	0
ING4	-0,094776071	-0,016461942	0	N.A.	0
RP11-713P17	-0,094793459	0,208226539	0	N.A.	0
RP11-180I4.4	-0,094797308		0	N.A.	0
TRMT10A	-0,094809082	0,034894464	0	N.A.	0
SF1	-0,094835021	0,101872838	0	N.A.	0
ZNF596	-0,094899899	-0,17767253	0	N.A.	0
PCF11	-0,094940575	0,07529317	0	N.A.	0
KIAA2012	-0,095022907	0,058570601	0	N.A.	0
ZNF786	-0,095053198	-0,178709596	0	N.A.	0
CPS1	-0,095132508	0,228849894	0	N.A.	0
SERPIND1	-0,09516892		0	N.A.	0
RP11-22L13.1	-0,095172213	0,087693578	0	N.A.	0
MBLAC2	-0,095193404	-0,111937901	0	N.A.	0
RP5-858L17.1	-0,095301197		0	N.A.	0
MAOA	-0,095324111	0,024527556	0	N.A.	0
RP11-178L8.8	-0,095343124		0	N.A.	0
PRSS56	-0,095345952	-0,08449875	0	N.A.	0
OLFML3	-0,095361274	0,157530017	0	N.A.	0
GZF1	-0,095364273	0,3190686	0	N.A.	0
KCNC2	-0,095372255		0	N.A.	0
RP11-341N2.1	-0,095401147	0,276901645	0	N.A.	0
UBXN2B	-0,095405975	-0,187339756	0	N.A.	0
PECR	-0,095422452	0,256918039	0	N.A.	0
CMTM4	-0,095489669	0,188333311	0	N.A.	0
ZSCAN18	-0,095493133	0,146233309	0	N.A.	0
ZNF497	-0,095497214	0,136847018	0	N.A.	0
MOAP1	-0,095614214	-0,001624645	0	N.A.	0
RP11-18H7.1	-0,095616602	-0,039262665	0	N.A.	0

CACNA2D2	-0,095640838	-0,091555266	0	N.A.	0
HAGHL	-0,095660691	0,208875621	0	N.A.	0
AKR1C3	-0,095708753	-0,066050092	0	N.A.	0
RP4-758J18.1	-0,09574373	0,06310452	0	N.A.	0
CD83	-0,095767342	-0,413216823	0	N.A.	0
ATG5	-0,095794659	-0,046064308	0	N.A.	0
DPP10	-0,095840837	-0,303838559	0	N.A.	0
RP11-477J21	-0,095857806	0,438954365	0	N.A.	0
CDK5R1	-0,0958624	0,123920615	0	N.A.	0
C1orf105	-0,095979097	0,299583942	0	N.A.	0
RP11-126K1.	-0,096018784		0	N.A.	0
AC004012.1	-0,096025461	0,237200699	0	N.A.	0
ZNF324B	-0,096052231	0,083518572	0	N.A.	0
B3GALT2	-0,096056503	-0,363310443	0	N.A.	0
RP11-423H2.	-0,096058502	-0,044621181	0	N.A.	0
ARHGEF40	-0,096107574	0,110769862	0	N.A.	0
RRN3	-0,096126807	-0,00532189	0	N.A.	0
NEK10	-0,09616087		0	N.A.	0
HARBI1	-0,096222202	0,091007748	0	N.A.	0
AAMDC	-0,096242572	-0,161866568	0	N.A.	0
REV3L	-0,096310726	-0,205206208	0	N.A.	0
TRABD2B	-0,09633059	0,012605694	0	N.A.	0
CTD-3032J10	-0,096331999		0	N.A.	0
TMEM182	-0,096333653	-0,059074925	0	N.A.	0
CAPZB	-0,096509929	0,017909598	0	N.A.	0
CTC-559E9.1	-0,096562595	0,083839784	0	N.A.	0
SH2D6	-0,096600197	-0,003914008	0	N.A.	0
HOXA1	-0,096662515	0,057125436	0	N.A.	0
ANKS6	-0,0966796	0,209224904	0	N.A.	0
ADAMTSL2	-0,096737929	-0,124949143	0	N.A.	0
LINC01555	-0,096741168		0	N.A.	0
ADRBK1	-0,096741382	0,30031348	0	N.A.	0
ACVR2B-AS1	-0,096777697		0	N.A.	0

IGF1	-0,096904117	-0,066111086	0	N.A.	0
TSPEAR	-0,096940994	0,126391947	0	N.A.	0
FLVCR1-AS1	-0,096996608	-0,275718588	0	N.A.	0
DYM	-0,097120391	-0,068697298	0	N.A.	0
FUT7	-0,097138757	0,3986027	0	N.A.	0
RAB9B	-0,097157271	0,010269904	0	N.A.	0
C2orf82	-0,097161182	-0,123563814	0	N.A.	0
GLTSCR2	-0,097192735	-0,40106412	0	N.A.	0
KIF24	-0,097210525	-0,114073627	0	N.A.	0
LRRC43	-0,097222261	-0,110984828	0	N.A.	0
TAF2	-0,097248017	0,169897935	0	N.A.	0
AC008074.3	-0,097337111	0,219484518	0	N.A.	0
FKBP8	-0,09738907	0,053736854	0	N.A.	0
SH2B1	-0,097391037	0,057653775	0	N.A.	0
COL27A1	-0,097421325	-0,042343559	0	N.A.	0
MINPP1	-0,097491231	0,197587493	0	N.A.	0
NKD1	-0,097498323	0,136124513	0	N.A.	0
TDRD5	-0,097508778	-0,126735818	0	N.A.	0
RP11-111A21	-0,097560352	0,033797437	0	N.A.	0
CUL5	-0,097628139	-0,020164179	0	N.A.	0
LRRC10B	-0,097693923	0,034703185	0	N.A.	0
PVRL1	-0,0977537	-0,071148221	0	N.A.	0
RFX5	-0,097898123	-0,315572541	0	N.A.	0
TBL2	-0,097932804	0,100082693	0	N.A.	0
AC092484.1	-0,097938846	-0,28915815	0	N.A.	0
TWIST2	-0,09793928	-0,103620519	0	N.A.	0
ISYNA1	-0,098013925	-0,04520666	0	N.A.	0
ACPP	-0,098028062	0,355991959	0	N.A.	0
MBIP	-0,098041094	0,093644417	0	N.A.	0
GNB2	-0,098086818	0,267464225	0	N.A.	0
RP11-486G15	-0,09810038		0	N.A.	0
SDCCAG8	-0,098129756	0,10871316	0	N.A.	0
SYNJ2BP-COX	-0,098140371	-0,093778277	0	N.A.	0

RP11-467D6.	-0,098157581	-0,092879605	0	N.A.	0
C2-AS1	-0,098170104		0	N.A.	0
CALCA	-0,098170563	-0,142340488	0	N.A.	0
APOA1	-0,098219573	0,021297925	0	N.A.	0
HOMER1	-0,098256713	0,170081594	0	N.A.	0
CYP39A1	-0,098285979	-0,105421393	0	N.A.	0
CYP27B1	-0,098301699	-0,159723997	0	N.A.	0
AF127577.8	-0,098314839	0,362920869	0	N.A.	0
STARD13	-0,098361025	-0,030485703	0	N.A.	0
ENKD1	-0,098380795	-0,27010261	0	N.A.	0
CH25H	-0,098401954		0	N.A.	0
IDI1	-0,098449706	-0,011546476	0	N.A.	0
ZGRF1	-0,09849706	-0,001527404	0	N.A.	0
BCKDK	-0,098577346	0,245768984	0	N.A.	0
RP6-74O6.6	-0,098635878	0,293145725	0	N.A.	0
FREM2	-0,098772576	-0,168869207	0	N.A.	0
KANSL2	-0,098794902	-0,093158452	0	N.A.	0
LRRC73	-0,098815124	-0,011940884	0	N.A.	0
LRIG1	-0,098953464	-0,057933647	0	N.A.	0
RP11-595B24	-0,098993183	-0,302301509	0	N.A.	0
GPR89B	-0,0990201	0,02297035	0	N.A.	0
AKAP8	-0,099038569	0,058270691	0	N.A.	0
SFSWAP	-0,099088657	0,151419374	0	N.A.	0
EID2	-0,099100738	-0,085146158	0	N.A.	0
AP4M1	-0,099136144	0,169209603	0	N.A.	0
WDSUB1	-0,099136448	0,02437838	0	N.A.	0
SOCS2	-0,099146037	0,102743202	0	N.A.	0
DND1	-0,099183699	-0,112180923	0	N.A.	0
RP11-307C12	-0,099192138	-0,023782458	0	N.A.	0
ZNF419	-0,099281266	0,278466209	0	N.A.	0
DOLK	-0,099290929	0,062013596	0	N.A.	0
AC005614.3	-0,099298411		0	N.A.	0
FCRL3	-0,099339186	-0,057605904	0	N.A.	0

S100A9	-0,099368376	-0,25558352	0	N.A.	0
PCTP	-0,099413501	-0,117367523	0	N.A.	0
DIEXF	-0,099429942	0,025995275	0	N.A.	0
SCYL1	-0,099440107	-0,03540769	0	N.A.	0
FAM207A	-0,099536748	-0,140961133	0	N.A.	0
SLC25A47	-0,099611297	0,082703476	0	N.A.	0
ENO4	-0,099659819	-0,113707677	0	N.A.	0
PTK7	-0,099661634	-0,378691547	0	N.A.	0
LINC00896	-0,09972482	0,07845501	0	N.A.	0
THAP3	-0,09974764	-0,274227383	0	N.A.	0
RP11-73K9.3	-0,099798147	0,180261303	0	N.A.	0
ELK1	-0,099805746	-0,28514731	0	N.A.	0
FAM57A	-0,0998464	-0,143928931	0	N.A.	0
RP11-326K13	-0,099858466	-0,289934344	0	N.A.	0
GNG8	-0,099894251		0	N.A.	0
TIAM1	-0,099911574	0,075724846	0	N.A.	0
RP11-151A6.	-0,099921761		0	N.A.	0
ADH1C	-0,099924957	-0,288581169	0	N.A.	0
EP400NL	-0,099948965	0,032478919	0	N.A.	0
C14orf79	-0,099964341	-0,116306062	0	N.A.	0
RP11-260M1	-0,099991401	-0,347408515	0	N.A.	0
C7orf26	-0,10003992	0,237039135	0	N.A.	0
ZNF197	-0,100048307	0,100816254	0	N.A.	0
C18orf21	-0,100048523	-0,042027077	0	N.A.	0
COL9A1	-0,10007544		0	N.A.	0
CD79B	-0,100150246	0,075560032	0	N.A.	0
C3orf38	-0,100155865	-0,25120054	0	N.A.	0
RP11-120K19	-0,100157277	0,046965638	0	N.A.	0
TH	-0,100164261	-0,150115642	0	N.A.	0
URB1	-0,100167969	-0,012053478	0	N.A.	0
AGAP2-AS1	-0,100221402	0,089739214	0	N.A.	0
ZNF213-AS1	-0,100242662	-0,080197573	0	N.A.	0
ANKEF1	-0,100260511	-0,325058317	0	N.A.	0

NRXN3	-0,100262828	-0,238138368	0	N.A.	0
PGBD1	-0,100267357	-0,304506918	0	N.A.	0
P4HB	-0,10028723	0,504162775	0	N.A.	0
CCND3	-0,100349824	-0,023366645	0	N.A.	0
HTRA3	-0,100449223	-0,058634599	0	N.A.	0
ZNF740	-0,100450337	0,011831972	0	N.A.	0
C19orf81	-0,100482763	-0,135572822	0	N.A.	0
FZD3	-0,100511365	-0,201721752	0	N.A.	0
TXNDC15	-0,10053638	-0,157271767	0	N.A.	0
RP11-804A23	-0,100552827		0	N.A.	0
GID8	-0,100571961	-0,012474522	0	N.A.	0
MSH3	-0,100652135	-0,072998514	0	N.A.	0
KCNA7	-0,100704491	0,006410315	0	N.A.	0
EIF1B	-0,100728726	0,019379856	0	N.A.	0
TFCP2	-0,100750155	0,192514343	0	N.A.	0
IGHGP	-0,100767092	0,006845336	0	N.A.	0
WHSC1L1	-0,100809129	0,220275084	0	N.A.	0
FUT10	-0,100900151	-0,235866458	0	N.A.	0
MLYCD	-0,100984434	0,364788087	0	N.A.	0
FPR2	-0,101010532	0,206731428	0	N.A.	0
NETO2	-0,101021593	-0,138997897	0	N.A.	0
ARRDC3-AS1	-0,101026639	-0,133601317	0	N.A.	0
STK19	-0,101064009	0,22247413	0	N.A.	0
CYTIP	-0,101092657	-0,279127701	0	N.A.	0
C11orf57	-0,101139156	-0,197775635	0	N.A.	0
IFITM2	-0,101159685	-0,255035883	0	N.A.	0
CTBP2	-0,101240689	0,142711796	0	N.A.	0
LINC01301	-0,101247026	-0,097082921	0	N.A.	0
SERPINA10	-0,101258989	0,109954994	0	N.A.	0
LINC01273	-0,101285436	-0,141587924	0	N.A.	0
RP11-707G18	-0,101304651	0,046358276	0	N.A.	0
MGA	-0,101337312	0,009342067	0	N.A.	0
RNF114	-0,101404048	0,080428604	0	N.A.	0

SMIM13	-0,101415949	-0,291589323	0	N.A.	0
ZNF626	-0,101442697	-0,238373313	0	N.A.	0
MARC2	-0,10149251	0,141705476	0	N.A.	0
RP11-7F17.8	-0,101503378	-0,005148025	0	N.A.	0
BCKDHB	-0,101571412	-0,32531394	0	N.A.	0
PTPRC	-0,101580947	-0,120542329	0	N.A.	0
RP11-167B3.	-0,101614992		0	N.A.	0
CTD-2377O1	-0,101618417		0	N.A.	0
IGKV3-11	-0,101629772	0,123795494	0	N.A.	0
TYRP1	-0,101656018	-0,011009798	0	N.A.	0
BEST3	-0,101679825	0,29072189	0	N.A.	0
BAZ1A	-0,101688134	0,018262801	0	N.A.	0
RP11-505K9.	-0,101693715	-0,118510105	0	N.A.	0
SLU7	-0,101734899	0,099898394	0	N.A.	0
RP11-643M1	-0,101776044	-0,079646101	0	N.A.	0
RP11-141M1	-0,101861794		0	N.A.	0
NAALADL1	-0,101884974	-0,063791137	0	N.A.	0
UBE2QL1	-0,101920281		0	N.A.	0
NAGPA	-0,101933672	0,027547968	0	N.A.	0
RAP1GAP	-0,10211207	-0,053426218	0	N.A.	0
RP11-89M20	-0,102120913	-0,002060108	0	N.A.	0
PDE1A	-0,10214535		0	N.A.	0
LMLN	-0,102235799	0,151129371	0	N.A.	0
SIX2	-0,102254118		0	N.A.	0
RP11-126K1.	-0,102291864		0	N.A.	0
SIRT3	-0,102318306	-0,202007682	0	N.A.	0
KL	-0,102374113		0	N.A.	0
WDR25	-0,102390637	-0,149872785	0	N.A.	0
RP11-412D9.	-0,102398608		0	N.A.	0
TMEM256-PI	-0,102400817	0,25432021	0	N.A.	0
RP11-600F24	-0,102437401	0,080089931	0	N.A.	0
SVOP	-0,102453882		0	N.A.	0
CCL17	-0,102479765		0	N.A.	0

IDE	-0,102513387	-0,12681979	0	N.A.	0
SMAD5	-0,102626694	0,115604142	0	N.A.	0
LINC00570	-0,1026357	-0,000951186	0	N.A.	0
CTD-3064M3	-0,102681307	-0,057449403	0	N.A.	0
DGKZ	-0,102685593	0,175433396	0	N.A.	0
PRPF39	-0,102795678	-0,026363459	0	N.A.	0
HBA2	-0,102820944	-0,079255454	0	N.A.	0
NEUROG3	-0,102829081	0,161012613	0	N.A.	0
AC012507.3	-0,10283616	0,191692544	0	N.A.	0
MFSD6L	-0,10287309	0,087552076	0	N.A.	0
GPR56	-0,102915861	0,239790363	0	N.A.	0
DIRAS3	-0,102967212	-0,004461398	0	N.A.	0
DACT2	-0,102979236		0	N.A.	0
ZNRD1-AS1	-0,102982625	0,004268879	0	N.A.	0
RP11-1299A1	-0,103001904	-0,086027407	0	N.A.	0
TEX29	-0,103004816	0,004537914	0	N.A.	0
SH3BGR	-0,103067496	0,100240107	0	N.A.	0
METTL21B	-0,103074501	-0,261160495	0	N.A.	0
GFOD2	-0,103084514	-0,076496513	0	N.A.	0
CTR9	-0,103117572	0,082957325	0	N.A.	0
SMDT1	-0,103152178	-0,266717636	0	N.A.	0
ZFP1	-0,10317397	-0,044370474	0	N.A.	0
FLJ37035	-0,103290832	-0,136031016	0	N.A.	0
PIGC	-0,103314577	0,331706541	0	N.A.	0
RP1-159A19.	-0,103369893	-0,233481515	0	N.A.	0
GATA4	-0,103381297	-0,014398417	0	N.A.	0
ENTPD1-AS1	-0,10338361	-0,14033927	0	N.A.	0
DDX42	-0,103409364	0,381091892	0	N.A.	0
ZNF420	-0,10341656	-0,098261093	0	N.A.	0
CCDC102A	-0,103435949	-0,126602754	0	N.A.	0
DLX3	-0,103500514	-0,12174515	0	N.A.	0
OTUD6B-AS1	-0,103528151	-0,129981641	0	N.A.	0
AC144450.2	-0,103578642	0,014074104	0	N.A.	0

METTL14	-0,103599952	0,034307295	0	N.A.	0
PDZRN3	-0,103601417	0,292083455	0	N.A.	0
MACROD2	-0,103625164	0,071724158	0	N.A.	0
LDLRAD3	-0,103697343	0,008469085	0	N.A.	0
ZFAND4	-0,103736174	0,075308812	0	N.A.	0
PTGES3L	-0,10377222	0,089049782	0	N.A.	0
EMID1	-0,103879242		0	N.A.	0
ACSL4	-0,1039042	-0,158099399	0	N.A.	0
KLHL7	-0,104014461	0,002496369	0	N.A.	0
CTTNBP2	-0,104059448	-0,207662906	0	N.A.	0
VRK2	-0,104060589	0,008389391	0	N.A.	0
RP11-610P16	-0,104068666	0,127507841	0	N.A.	0
CWF19L2	-0,104106739	0,112108056	0	N.A.	0
SLC38A6	-0,104180158	0,129111566	0	N.A.	0
BCL6B	-0,104204052		0	N.A.	0
VPS33A	-0,104220424	-0,315176595	0	N.A.	0
SMARCAL1	-0,104235681	-0,164907709	0	N.A.	0
KLHDC9	-0,104260503	-0,233139984	0	N.A.	0
XAB2	-0,104313614	0,019026597	0	N.A.	0
CTA-363E6.6	-0,104346404	-0,061070442	0	N.A.	0
BCKDHA	-0,104375596	-0,137450365	0	N.A.	0
RP11-394I13	-0,104386931	0,055759948	0	N.A.	0
LAMP1	-0,104406823	0,420677615	0	N.A.	0
ATOH8	-0,104489813	0,210030113	0	N.A.	0
AUP1	-0,104493481	0,281423833	0	N.A.	0
SPATA5	-0,104539275	0,071630738	0	N.A.	0
RBP7	-0,104544454	-0,075832205	0	N.A.	0
RP4-613A2.1	-0,1045598	0,159364284	0	N.A.	0
HNF1A	-0,104573266	-0,17862486	0	N.A.	0
HOXB6	-0,104618695	0,553463278	0	N.A.	0
FGD5-AS1	-0,10462558	0,053044615	0	N.A.	0
ZNF891	-0,104642603	0,035149634	0	N.A.	0
AC006547.14	-0,104665012		0	N.A.	0

NQO2	-0,104692627	-0,049455517	0	N.A.	0
IGHV3-21	-0,10471682	-0,22982107	0	N.A.	0
SMAD6	-0,104735214	-0,0908964	0	N.A.	0
RP11-295H24	-0,104738202	-0,063061895	0	N.A.	0
RP11-399K21	-0,104750521	0,200574126	0	N.A.	0
CNDP1	-0,104880469	-0,257034054	0	N.A.	0
AC002454.1	-0,104910074		0	N.A.	0
IGLV2-23	-0,104929478	-0,010615561	0	N.A.	0
TRMT12	-0,104929754	-0,014906814	0	N.A.	0
WIPF1	-0,105096616	-0,165690673	0	N.A.	0
NAT9	-0,105106197	-0,076730304	0	N.A.	0
ADAMTS14	-0,105111043	0,122100689	0	N.A.	0
RP11-318E3.9	-0,10513481	0,153620059	0	N.A.	0
C8orf37	-0,105167325	0,056628728	0	N.A.	0
ACOT4	-0,105215556	-0,22789281	0	N.A.	0
DSCAML1	-0,105227627	-0,10941462	0	N.A.	0
HEATR6	-0,105259755	0,087919587	0	N.A.	0
RP3-496C20.	-0,105279692	-0,048458403	0	N.A.	0
CCDC40	-0,105329463	-0,047012716	0	N.A.	0
SPTY2D1-AS1	-0,105342334	-0,286409127	0	N.A.	0
EIF2S3L	-0,105367772	-0,109371915	0	N.A.	0
IGLV3-9	-0,105414192	-0,225163244	0	N.A.	0
RP11-1060J1	-0,105449156	0,038617062	0	N.A.	0
MARCH9	-0,105599435	0,238026417	0	N.A.	0
RP11-770J1.3	-0,10567813	-0,273272437	0	N.A.	0
CTC-523E23.	-0,105688262		0	N.A.	0
HTR1F	-0,105691726		0	N.A.	0
APOL4	-0,10572908	-0,27043428	0	N.A.	0
NUPR1	-0,105751466	-0,158948836	0	N.A.	0
ZNF107	-0,105873922	-0,067471439	0	N.A.	0
CDK7	-0,105955696	-0,117552708	0	N.A.	0
IDH1-AS1	-0,106003562	-0,313956692	0	N.A.	0
ENHO	-0,106038815		0	N.A.	0

NAA25	-0,106047147	0,087142541	0	N.A.	0
CPOX	-0,106052091	0,178460568	0	N.A.	0
CTD-2006K23	-0,106056648		0	N.A.	0
ARGLU1	-0,106081597	0,316991036	0	N.A.	0
ZNRF3	-0,106101363	-0,055489145	0	N.A.	0
PCDH8	-0,106109673		0	N.A.	0
C5orf38	-0,106120734	0,011075135	0	N.A.	0
RPPH1	-0,106145862	0,156187504	0	N.A.	0
ZBTB3	-0,106163699	0,013506531	0	N.A.	0
CXorf57	-0,1063147	-0,102242785	0	N.A.	0
RP11-109D9.	-0,106531911		0	N.A.	0
C8orf44	-0,106562402	0,085142113	0	N.A.	0
LPHN1	-0,106728961	0,27418562	0	N.A.	0
NEURL1	-0,106799582	-0,058934997	0	N.A.	0
PCSK1	-0,106807878	-0,145162845	0	N.A.	0
CLEC11A	-0,106835224	-0,202142424	0	N.A.	0
NOP10	-0,106903827	-0,152679161	0	N.A.	0
NUCB1	-0,106905402	-0,082672471	0	N.A.	0
PRUNE	-0,106959114	-0,010046027	0	N.A.	0
LRRC2	-0,106975088	-0,210263912	0	N.A.	0
MB21D1	-0,107011134	-0,175177261	0	N.A.	0
CLDN10	-0,107024191		0	N.A.	0
CEP164	-0,107056473	0,153024242	0	N.A.	0
CHL1	-0,107131507	-0,261509598	0	N.A.	0
ST7	-0,107149985	-0,305114772	0	N.A.	0
ALDH5A1	-0,107150592	-0,178649279	0	N.A.	0
PIGBOS1	-0,107153788	-0,230491707	0	N.A.	0
KCNJ11	-0,107163035	-0,157411152	0	N.A.	0
RP11-326I11	-0,107179106	0,249854086	0	N.A.	0
KLHL32	-0,10720533	-0,134179101	0	N.A.	0
FBXO15	-0,107271005	-0,034291472	0	N.A.	0
KIRREL3	-0,107294358	-0,105869318	0	N.A.	0
FAM175B	-0,107390869	0,091923901	0	N.A.	0

ACTG1	-0,107458172	-0,109889457	0	N.A.	0
ANK2	-0,107725027	0,109422504	0	N.A.	0
REM2	-0,107796012	0,082293678	0	N.A.	0
ATG10	-0,107862714	-0,130804654	0	N.A.	0
RP4-753F5.1	-0,107864344	-0,032580665	0	N.A.	0
ZNF391	-0,107962896	-0,322543549	0	N.A.	0
RP11-30L15.(	-0,108194546		0	N.A.	0
CYBA	-0,108223047	0,049032427	0	N.A.	0
FBLN1	-0,108272173	0,073349198	0	N.A.	0
DDTL	-0,108319111	-0,126155523	0	N.A.	0
TEAD2	-0,108319254	-0,334679431	0	N.A.	0
ARHGAP9	-0,108411913	-0,221607244	0	N.A.	0
IFT74-AS1	-0,108432202	0,012410485	0	N.A.	0
ENTHD2	-0,108446454	0,114765219	0	N.A.	0
NUP153	-0,108486734	0,163077924	0	N.A.	0
THEG	-0,108494276		0	N.A.	0
PPFIBP2	-0,108537025	-0,048376125	0	N.A.	0
LOXL1	-0,108552726	-0,245661826	0	N.A.	0
TMEM220-A!	-0,108552808	-0,297882311	0	N.A.	0
CEPT1	-0,108608591	0,081036051	0	N.A.	0
GDPD1	-0,10861582	0,169694306	0	N.A.	0
SLC2A4	-0,108619478	-0,142932716	0	N.A.	0
DNAJB5	-0,108662845	-0,073931139	0	N.A.	0
C8orf44-SGK	-0,108688201	-0,050984075	0	N.A.	0
DACH1	-0,108704563	-0,112822557	0	N.A.	0
OSCP1	-0,108756325	0,056037221	0	N.A.	0
ZFP69	-0,108756892	-0,12594194	0	N.A.	0
RP11-517B11	-0,108761231		0	N.A.	0
SMOC1	-0,108808929	0,315499611	0	N.A.	0
ZNF473	-0,108850487	-0,057694967	0	N.A.	0
RP11-343J3.2	-0,108876046		0	N.A.	0
ARID4A	-0,10888691	0,22134394	0	N.A.	0
TMEM80	-0,108990492	-0,220996899	0	N.A.	0

WNT3	-0,109006305	-0,049935548	0	N.A.	0
FBF1	-0,109048863	-0,06054638	0	N.A.	0
CEP19	-0,109052792	-0,308529427	0	N.A.	0
AGBL4	-0,109066696	0,140080386	0	N.A.	0
RP11-462G2.	-0,109113093	0,052859364	0	N.A.	0
TSSC4	-0,109160003	0,053607769	0	N.A.	0
PTBP2	-0,109160419	-0,156319925	0	N.A.	0
ERICH5	-0,109161169	-0,008448679	0	N.A.	0
AEBP2	-0,109166303	0,089029615	0	N.A.	0
RP11-220I1.5	-0,109172365	-0,077020045	0	N.A.	0
PRICKLE3	-0,109179427	-0,177493714	0	N.A.	0
IER2	-0,109300501	0,289497968	0	N.A.	0
PRR9	-0,109321568		0	N.A.	0
SLC52A2	-0,109402772	0,437838145	0	N.A.	0
SYF2	-0,109448489	0,072773888	0	N.A.	0
AC053503.4	-0,109461223	0,238888793	0	N.A.	0
FOCAD-AS1	-0,109461431	-0,246292209	0	N.A.	0
CTD-2240J17	-0,109467868	-0,125461221	0	N.A.	0
FLJ37453	-0,10951954	0,084642919	0	N.A.	0
MTA3	-0,109607209	0,200550066	0	N.A.	0
ARMCX1	-0,109609494	0,284196837	0	N.A.	0
IRF2BP1	-0,109614244	-0,055021841	0	N.A.	0
RP11-134G8.	-0,109623639	-0,252360046	0	N.A.	0
LINC00853	-0,10963684	-0,064628577	0	N.A.	0
GTPBP10	-0,109693127	0,079023949	0	N.A.	0
JPH1	-0,109833587	-0,098243851	0	N.A.	0
PANO1	-0,109837306	0,133124872	0	N.A.	0
AGL	-0,109879535	0,130813627	0	N.A.	0
GTF3C4	-0,109955944	-0,107153282	0	N.A.	0
ACO2	-0,11003348	0,162477202	0	N.A.	0
SLC27A4	-0,110042166	0,332263237	0	N.A.	0
GEMIN4	-0,110082969	0,09755786	0	N.A.	0
CDADC1	-0,110106649	-0,098497669	0	N.A.	0

DENND1C	-0,110110691	-0,182877612	0	N.A.	0
RP11-651L5.3	-0,110116534	-0,036748019	0	N.A.	0
CA5A	-0,110130242	0,02521042	0	N.A.	0
RGS1	-0,110152042	-0,216940953	0	N.A.	0
ZNF141	-0,110154244	-0,104273857	0	N.A.	0
RP11-93B14.	-0,110163	-0,039315617	0	N.A.	0
UBE2M	-0,110165235	0,236261261	0	N.A.	0
RP11-401P9.	-0,110173645	0,005232605	0	N.A.	0
AC022182.1	-0,110206131	-0,234758617	0	N.A.	0
PCDHA10	-0,110214184		0	N.A.	0
DUSP28	-0,110234086	0,022569078	0	N.A.	0
AC009403.2	-0,110347326	-0,424231886	0	N.A.	0
MAP6D1	-0,110383236	-0,120105156	0	N.A.	0
SATB2-AS1	-0,110436762	-0,096037228	0	N.A.	0
LAMTOR5	-0,110451537	0,082537631	0	N.A.	0
GLIPR2	-0,110502418	-0,362750899	0	N.A.	0
RP13-131K19	-0,110509174	0,253107376	0	N.A.	0
ZNF37A	-0,110547797	0,0981182	0	N.A.	0
RP3-323P24.	-0,110560935	-0,08856288	0	N.A.	0
RASSF1	-0,110587121	-0,346018252	0	N.A.	0
TMLHE	-0,1106978	-0,484268643	0	N.A.	0
EFEMP1	-0,11071842	0,243981052	0	N.A.	0
IKBKAP	-0,110745811	-0,097750054	0	N.A.	0
RP11-390E23	-0,110755852	-0,127106197	0	N.A.	0
RP11-451G4.	-0,110764231	0,311745518	0	N.A.	0
C11orf30	-0,110772224	0,067228005	0	N.A.	0
ST6GALNAC1	-0,110849344	-0,090739901	0	N.A.	0
ENAH	-0,110856415	-0,092631234	0	N.A.	0
LINC01126	-0,110884504	0,053416652	0	N.A.	0
SEP15	-0,110940069	0,147570083	0	N.A.	0
PLA2G4D	-0,110953674	0,144779829	0	N.A.	0
LRRIQ3	-0,110966403	-0,373767387	0	N.A.	0
ZNF662	-0,111027037	0,116138296	0	N.A.	0

NME3	-0,111081684	0,38181588	0	N.A.	0
CHD6	-0,111118311	0,045350091	0	N.A.	0
ZFP30	-0,111231401	-0,100726691	0	N.A.	0
ATOH7	-0,111251358	-0,003609781	0	N.A.	0
RP11-162A12	-0,111256187		0	N.A.	0
OAZ1	-0,11157278	-0,03743802	0	N.A.	0
LL0XNC01-7P	-0,111619773	0,101495955	0	N.A.	0
ZNF45	-0,111640435	-0,12462053	0	N.A.	0
CCDC117	-0,111672984	-0,010140459	0	N.A.	0
TBP	-0,111707561	0,04693403	0	N.A.	0
RP11-332J15	-0,111712188		0	N.A.	0
GCLM	-0,111738482	-0,265372973	0	N.A.	0
TCTN3	-0,11174299	-0,046589237	0	N.A.	0
CNOT6	-0,111774965	-0,226543121	0	N.A.	0
C12orf5	-0,111853215	-0,162107997	0	N.A.	0
ZNF491	-0,11195204	-0,080106893	0	N.A.	0
TARS	-0,11197219	0,240885643	0	N.A.	0
TRDC	-0,111996837		0	N.A.	0
CACNA1H	-0,112006417	0,174007787	0	N.A.	0
AKAP14	-0,112069517	-0,270585702	0	N.A.	0
FHDC1	-0,112109276	0,143792	0	N.A.	0
AC007773.2	-0,112176191	-0,015340367	0	N.A.	0
ZNF668	-0,112201221	-0,277081791	0	N.A.	0
RP11-996F15	-0,112404991	0,196171612	0	N.A.	0
RGS14	-0,112432338	-0,042472291	0	N.A.	0
RAB42	-0,112452173	0,156087843	0	N.A.	0
RP11-645C24	-0,112471412		0	N.A.	0
PTPRN	-0,112471415	-0,065828982	0	N.A.	0
RP11-1109F1	-0,11254889		0	N.A.	0
PTPRN2	-0,112630288	0,147744732	0	N.A.	0
ZNF280B	-0,112638826	-0,179859287	0	N.A.	0
RP11-262H14	-0,112641612	-0,368168617	0	N.A.	0
FOXN3-AS1	-0,112690759	0,044668025	0	N.A.	0

SLC10A5	-0,112713812	-0,06793121	0	N.A.	0
IGHD	-0,112727692	-0,238373313	0	N.A.	0
EXOSC6	-0,112764212	0,108063033	0	N.A.	0
MARS	-0,112824564	0,228257854	0	N.A.	0
USP27X-AS1	-0,112885383	-0,137027608	0	N.A.	0
WTAP	-0,112897065	-0,026770097	0	N.A.	0
CDK6	-0,112991916	-0,041002462	0	N.A.	0
AC005523.3	-0,113049171	0,166370016	0	N.A.	0
RP11-697E2.	-0,11310505	0,027602856	0	N.A.	0
TEX101	-0,113106823	0,026283336	0	N.A.	0
UBE2K	-0,113198673	-0,138355371	0	N.A.	0
STAM-AS1	-0,113229015	0,109955723	0	N.A.	0
LINC01260	-0,113257764	0,089350064	0	N.A.	0
MCOLN3	-0,113267815	0,019589737	0	N.A.	0
DUSP19	-0,113312542	0,096574261	0	N.A.	0
RP11-242D8.	-0,113352404	0,028329898	0	N.A.	0
CDH23	-0,11340557	-0,188708907	0	N.A.	0
SYNGR4	-0,113407346	-0,338998546	0	N.A.	0
SULT2A1	-0,113436609	-0,230806035	0	N.A.	0
RP11-424N2	-0,113446979	0,122441668	0	N.A.	0
IL2	-0,1134999		0	N.A.	0
C11orf53	-0,113603879	-0,190270429	0	N.A.	0
FAM72A	-0,113690956	-0,2095444	0	N.A.	0
ATP5EP2	-0,113779594	-0,292403541	0	N.A.	0
PPCDC	-0,113785974	-0,064276913	0	N.A.	0
CD8B	-0,113811146	0,065286158	0	N.A.	0
FHL3	-0,113871903	-0,304204612	0	N.A.	0
BPGM	-0,113909388	-0,131792207	0	N.A.	0
ATP13A1	-0,113926854	0,254842907	0	N.A.	0
RASAL3	-0,113933718	-0,1195951	0	N.A.	0
RP11-669N7.	-0,113937619	-0,197452857	0	N.A.	0
POLG2	-0,113954121	0,000347476	0	N.A.	0
RP11-513M1	-0,113990309	-0,184971372	0	N.A.	0

SNCAIP	-0,114037249	-0,377956934	0	N.A.	0
CD302	-0,114058883	-0,27470936	0	N.A.	0
DBNL	-0,114160039	0,153253405	0	N.A.	0
ADNP	-0,114231128	0,062981824	0	N.A.	0
TBX10	-0,11431913	-0,149572202	0	N.A.	0
FSD1	-0,114396425	0,050160716	0	N.A.	0
NAT2	-0,114396805	0,158489531	0	N.A.	0
ZCCHC12	-0,114407843		0	N.A.	0
AC142119.1	-0,11446917	-0,119725798	0	N.A.	0
DCLK2	-0,114470652		0	N.A.	0
SFTA1P	-0,114492982	-0,063752043	0	N.A.	0
CASC15	-0,114529039	0,112950896	0	N.A.	0
HIGD2A	-0,114535404	-0,386619348	0	N.A.	0
NR0B2	-0,114575458	0,094683595	0	N.A.	0
RP11-421M1	-0,114624394	-0,205659702	0	N.A.	0
ATAT1	-0,114652987	-0,346642016	0	N.A.	0
UBE2D4	-0,114660585	-0,18890042	0	N.A.	0
KBTBD12	-0,114685261	0,172851274	0	N.A.	0
EXO5	-0,114718163	0,169405082	0	N.A.	0
LSM14B	-0,114813335	-0,161594946	0	N.A.	0
YAF2	-0,114866012	-0,195161058	0	N.A.	0
HOXD8	-0,11490721	0,059358965	0	N.A.	0
NT5M	-0,114931331	-0,107798453	0	N.A.	0
SLC35C2	-0,114972168	0,158037546	0	N.A.	0
LINC00863	-0,115001744	0,05421278	0	N.A.	0
CKM	-0,115023075	0,060742791	0	N.A.	0
RP11-97C16.	-0,115023215	-0,130900294	0	N.A.	0
ZNF280C	-0,115179611	-0,092926337	0	N.A.	0
ZCCHC7	-0,115289591	0,011859579	0	N.A.	0
RP4-563E14.	-0,115292742	-0,043940074	0	N.A.	0
TCEB3	-0,115394862	0,182650523	0	N.A.	0
OXNAD1	-0,115542305	-0,240095859	0	N.A.	0
RP11-127B2(	-0,115612469	-0,006251181	0	N.A.	0

RP11-98I9.4	-0,115676947	-0,23338751	0	N.A.	0
SLC8A1	-0,115688745	-0,273604502	0	N.A.	0
AMH	-0,115748846	0,103461051	0	N.A.	0
NELFA	-0,115841971	-0,051302741	0	N.A.	0
HOXA-AS3	-0,115851736	-0,026905873	0	N.A.	0
KLF1	-0,115913045		0	N.A.	0
KCNK12	-0,115928078		0	N.A.	0
ANKRD30B	-0,115971968	0,013922958	0	N.A.	0
TNPO1	-0,116024391	0,057025064	0	N.A.	0
ACTA1	-0,116040694	0,014341183	0	N.A.	0
IZUMO2	-0,116070475	-0,349851694	0	N.A.	0
SCAF11	-0,116071991	0,265098941	0	N.A.	0
RP11-265N6.	-0,116076527	0,40978219	0	N.A.	0
SLC27A3	-0,116078258	0,25585006	0	N.A.	0
ZNF214	-0,116106619	-0,271489728	0	N.A.	0
STAMBP	-0,116171559	0,219074088	0	N.A.	0
FUCA2	-0,116172479	0,14713647	0	N.A.	0
BBS5	-0,116197209	0,356975999	0	N.A.	0
HMHA1	-0,116197548	0,085499582	0	N.A.	0
C2orf69	-0,11625453	-0,171607132	0	N.A.	0
XXbac-B135H	-0,116259566	0,007739107	0	N.A.	0
ZMYND19	-0,116284513	-0,031817712	0	N.A.	0
RP3-467K16.	-0,116364575		0	N.A.	0
CYP27A1	-0,116372862	-0,068072483	0	N.A.	0
ACSM3	-0,1163746	-0,328382403	0	N.A.	0
RP11-112J3.1	-0,116382494	-0,091638561	0	N.A.	0
AC004231.2	-0,116385016	0,202300158	0	N.A.	0
BTK	-0,116408219	-0,106748793	0	N.A.	0
SUOX	-0,116416825	-0,044670584	0	N.A.	0
THRA	-0,11644365	-0,178694904	0	N.A.	0
ZNF805	-0,11652738	0,209285367	0	N.A.	0
PIFO	-0,116589876	-0,341612058	0	N.A.	0
RP5-1057I20	-0,116598555	0,055082955	0	N.A.	0

LRRC14	-0,116633308	0,143190583	0	N.A.	0
NCF4	-0,11665215	-0,304065347	0	N.A.	0
GHDC	-0,116680966	0,103695653	0	N.A.	0
CFTR	-0,116710349	-0,185027059	0	N.A.	0
KBTBD7	-0,11676993	-0,20958342	0	N.A.	0
IFFO1	-0,116780264	-0,206197022	0	N.A.	0
DALRD3	-0,116850865	0,120793276	0	N.A.	0
PTPN4	-0,116864197	-0,132581187	0	N.A.	0
ITGA10	-0,116966636	0,03465005	0	N.A.	0
GNPDA1	-0,117036716	-0,161200979	0	N.A.	0
RP11-406H23	-0,117075187	0,335806186	0	N.A.	0
TTLL7	-0,11714213	-0,110790222	0	N.A.	0
FAM178B	-0,117218644	-0,22160522	0	N.A.	0
CWH43	-0,117219943	-0,354228384	0	N.A.	0
RP11-145M4	-0,117234972		0	N.A.	0
ADCK4	-0,117299778	-0,022410356	0	N.A.	0
SETD4	-0,117326766	-0,040003411	0	N.A.	0
FOXS1	-0,117344346		0	N.A.	0
BOLA3-AS1	-0,117424683		0	N.A.	0
SETDB2	-0,117448814	-0,31912173	0	N.A.	0
C5orf63	-0,117457069	-0,118289687	0	N.A.	0
DPY19L4	-0,117472902	-0,005603849	0	N.A.	0
LINC01515	-0,117476118	-0,11907776	0	N.A.	0
OSBPL6	-0,117537906		0	N.A.	0
RP11-314O13	-0,117538924	-0,246778365	0	N.A.	0
SCFD1	-0,117562674	0,201257691	0	N.A.	0
CCNO	-0,117610458	-0,006004973	0	N.A.	0
PTCH2	-0,117659441	0,102868808	0	N.A.	0
XPNPEP3	-0,117762007	-0,358259212	0	N.A.	0
ZNF280A	-0,117914451	-0,135946787	0	N.A.	0
GAD1	-0,117924359	-0,193741945	0	N.A.	0
SLC39A10	-0,118126854	0,364013747	0	N.A.	0
PTPRD-AS1	-0,118173117	-0,070770311	0	N.A.	0

FANCE	-0,118217137	0,063700865	0	N.A.	0
U2AF1	-0,118334886	-0,079472813	0	N.A.	0
MAP4K2	-0,118344121	-0,094756372	0	N.A.	0
ATG4B	-0,118388979	0,427648058	0	N.A.	0
HLF	-0,118484753	0,018448765	0	N.A.	0
SPIB	-0,118580508	-0,191701602	0	N.A.	0
ZNF684	-0,1187658	-0,066585783	0	N.A.	0
RAB20	-0,118804543	0,188438983	0	N.A.	0
C3orf67	-0,118810406	0,277930652	0	N.A.	0
CHMP7	-0,118886418	-0,125125377	0	N.A.	0
KCTD17	-0,118903736	-0,357734061	0	N.A.	0
LIN52	-0,118911418	0,061889243	0	N.A.	0
SLC41A1	-0,118937953	0,01401526	0	N.A.	0
MYCBP2	-0,11900177	0,270661594	0	N.A.	0
HOXC10	-0,119141956		0	N.A.	0
RIMBP2	-0,119147054	0,059594188	1	cl2	0
VTA1	-0,119151734	-0,111919382	0	N.A.	0
RNF32	-0,119329524	-0,184305319	0	N.A.	0
TPM1	-0,119383786	0,151900973	0	N.A.	0
RILPL2	-0,119385023	-0,276884557	0	N.A.	0
ZNF501	-0,119424942	-0,196731398	0	N.A.	0
CTD-2383M3	-0,119477803	-0,161172714	0	N.A.	0
FARP1	-0,119498263	0,061081009	0	N.A.	0
FAM208B	-0,11952095	0,254636228	0	N.A.	0
CEP162	-0,119591287	-0,306158107	0	N.A.	0
MTSS1L	-0,119613374	0,019826057	0	N.A.	0
ITGA7	-0,119617952	0,007256277	0	N.A.	0
ATP11C	-0,119623632	0,077624162	0	N.A.	0
RP11-1277A3	-0,11965529	0,289443986	0	N.A.	0
LCP2	-0,11975052	0,051246601	0	N.A.	0
G2E3	-0,119789343	0,103309677	0	N.A.	0
DNAJC5B	-0,119795948	-0,119156043	0	N.A.	0
RP1-257A7.4	-0,119797853		0	N.A.	0

CLCNKB	-0,119816993		0	N.A.	0
RP5-994D16.	-0,119826642	-0,281038577	0	N.A.	0
C17orf49	-0,119845078	-0,075050085	0	N.A.	0
SHKBP1	-0,119930038	-0,207221397	0	N.A.	0
DIS3L2	-0,119934977	0,059262901	0	N.A.	0
BAG1	-0,12004945	0,11031176	0	N.A.	0
RP11-299J3.8	-0,120049662	-0,030718704	0	N.A.	0
GSTA1	-0,120106338	-0,139214776	1	none	0
PKN3	-0,1201596	-0,08426498	0	N.A.	0
TTC19	-0,120253706	-0,025359081	0	N.A.	0
CXCR4	-0,120332514	-0,219140151	0	N.A.	0
ARPC1A	-0,120339703	0,096353903	0	N.A.	0
CIB2	-0,120425435	0,033418575	0	N.A.	0
SLC43A3	-0,120483556	-0,102198714	0	N.A.	0
HOXB8	-0,120500663	-0,107560259	1	cl2	0
EVX1-AS	-0,120517198	0,176984319	0	N.A.	0
DNAJC22	-0,120519977	-0,062894144	0	N.A.	0
PPP4R2	-0,120538862	0,124989554	0	N.A.	0
PCK2	-0,120606439	0,227955364	0	N.A.	0
RP11-115C21	-0,120744254	-0,051701871	0	N.A.	0
MFN1	-0,120764638	0,008191744	0	N.A.	0
BDH1	-0,120777846	-0,003751202	0	N.A.	0
C2orf70	-0,120811148	-0,164809025	0	N.A.	0
PSTK	-0,120817136	-0,204245395	0	N.A.	0
ATXN7L3B	-0,120932582	-0,145932267	0	N.A.	0
ATF7	-0,121048704	0,18984603	0	N.A.	0
UBA3	-0,121079937	-0,164196561	0	N.A.	0
RP11-359B12	-0,121081783	-0,080380747	0	N.A.	0
STAMBPL1	-0,1211415	-0,01146746	0	N.A.	0
RP11-11N9.4	-0,121162977	-0,270063207	0	N.A.	0
TNFSF13B	-0,121195298	0,024930469	0	N.A.	0
ECD	-0,121221255	-0,311123525	0	N.A.	0
AGGF1	-0,121278832	-0,198621408	0	N.A.	0

BPTF	-0,121297918	0,246088066	0	N.A.	0
PROX1	-0,121404408	-0,199984008	0	N.A.	0
SIGLEC9	-0,121418332	0,087948413	0	N.A.	0
NINL	-0,121445909	0,104201373	0	N.A.	0
SFXN5	-0,121447408	-0,044271562	0	N.A.	0
LINC00858	-0,121483283	0,009901924	0	N.A.	0
PPIL2	-0,121642261	0,347996511	0	N.A.	0
TMEM89	-0,121672522		0	N.A.	0
ZNF813	-0,121755277	-0,112933301	0	N.A.	0
TNFAIP8L3	-0,121837399		0	N.A.	0
FAM212A	-0,121844623	0,075400901	0	N.A.	0
LKAAEAR1	-0,121855596		0	N.A.	0
ECHDC3	-0,12189369	-0,317818774	0	N.A.	0
DHCR24	-0,121925648	0,310707335	0	N.A.	0
PKIG	-0,121997615	-0,096309882	0	N.A.	0
PCYT2	-0,122038217	0,475257412	0	N.A.	0
ZMAT5	-0,122051744	-0,145377904	0	N.A.	0
E2F6	-0,122095267	-0,22535916	0	N.A.	0
RP11-27K13.	-0,122100017	-0,169408637	0	N.A.	0
RP11-93K22.	-0,122104462	0,026280281	0	N.A.	0
RP11-323F24	-0,122160088	0,036713347	0	N.A.	0
ZBTB2	-0,122190515	-0,184410913	0	N.A.	0
LL22NC03-75	-0,122322409	0,060324184	0	N.A.	0
CLPX	-0,122353753	0,008185609	0	N.A.	0
PTPN20A	-0,122517483		0	N.A.	0
HIST3H2A	-0,122575236	-0,004433302	0	N.A.	0
EVL	-0,122589772	-0,341864222	0	N.A.	0
H1FX-AS1	-0,122592174		0	N.A.	0
C19orf73	-0,122607086	-0,087969493	0	N.A.	0
FAM221A	-0,122705129	-0,278782706	0	N.A.	0
CASC11	-0,122764689	-0,160817471	0	N.A.	0
KIF25	-0,122775668		0	N.A.	0
FLI1	-0,122821388	-0,018377443	0	N.A.	0

DDN	-0,122838506	-0,169149517	0	N.A.	0
MTIF3	-0,122925796	-0,041441706	0	N.A.	0
MED10	-0,122928427	0,153881971	0	N.A.	0
ALDH18A1	-0,122930996	-0,04363162	0	N.A.	0
DEFA6	-0,122932339	0,0542539	0	N.A.	0
HEPACAM2	-0,122933633	-0,048456419	0	N.A.	0
AIF1L	-0,123063828	-0,069615846	0	N.A.	0
RP13-1032I1	-0,123066564	-0,132807336	0	N.A.	0
RP3-477O4.1	-0,123077852		0	N.A.	0
RAB23	-0,123123251	-0,278477398	0	N.A.	0
ANXA13	-0,123161746	0,067140886	0	N.A.	0
CHSY1	-0,123163245	0,167747104	0	N.A.	0
BTBD8	-0,123165711	0,082351401	0	N.A.	0
PTPN11	-0,123167832	-0,057774159	0	N.A.	0
RP4-728D4.2	-0,12318552		0	N.A.	0
TNFAIP6	-0,123281792	0,027928222	0	N.A.	0
SETD3	-0,123302499	-0,060221928	0	N.A.	0
P3H4	-0,123316872	0,136863205	0	N.A.	0
LAMA5-AS1	-0,123356063	0,051503027	0	N.A.	0
CTD-2293H3.	-0,123375573		0	N.A.	0
TYW1B	-0,123388708	-0,171885665	0	N.A.	0
RP11-89G4.1	-0,123392056	0,154518784	0	N.A.	0
RP11-498P14	-0,123407633	0,024356232	0	N.A.	0
RP11-488C13	-0,123491093	-0,095687091	0	N.A.	0
SP5	-0,123539792	0,122998334	0	N.A.	0
DLST	-0,123582312	0,258294388	0	N.A.	0
LZTS1	-0,12365308		0	N.A.	0
LACE1	-0,123656538	-0,137177096	0	N.A.	0
GNL1	-0,123700957	-0,171500551	0	N.A.	0
SPRTN	-0,123830886	-0,087852838	0	N.A.	0
SPIN4	-0,123841432	-0,086066074	0	N.A.	0
C1D	-0,123931247	-0,05455789	0	N.A.	0
DNAJC21	-0,123997273	0,180910978	0	N.A.	0

RAB27A	-0,124041373	-0,270757388	0	N.A.	0
ASCC3	-0,124050235	-0,015338249	0	N.A.	0
SMCO4	-0,12415018	0,184587721	0	N.A.	0
APBB1IP	-0,124152107	-0,185527777	0	N.A.	0
LRRC24	-0,124238865	-0,097919663	0	N.A.	0
RP11-426C22	-0,124257741	-0,064951498	0	N.A.	0
SEMA3G	-0,124354516		0	N.A.	0
C5orf49	-0,124563502		0	N.A.	0
GSTK1	-0,12462875	-0,265726472	0	N.A.	0
ABCC6	-0,124701361	-0,205360777	0	N.A.	0
RP1-178F15.4	-0,124724906	0,087168243	0	N.A.	0
TRAM2-AS1	-0,124783902	-0,189785447	0	N.A.	0
CMAS	-0,124828697	-0,032592404	0	N.A.	0
PAWR	-0,124869777	0,406498871	0	N.A.	0
AMACR	-0,124882279	-0,154985659	0	N.A.	0
PEX6	-0,12502386	-0,304123976	0	N.A.	0
ASPN	-0,125064579	-0,043266766	0	N.A.	0
KCNIP3	-0,125065907	0,116095858	0	N.A.	0
SLC9B1	-0,125122619	-0,01567862	0	N.A.	0
SLC2A5	-0,125157317	-0,23691223	0	N.A.	0
HRASLS	-0,125165829	0,258992819	0	N.A.	0
HS3ST3B1	-0,12522815	0,102942049	0	N.A.	0
ATP6V1B2	-0,125273638	0,079667524	0	N.A.	0
RP5-1007H16	-0,125304298	0,26839848	0	N.A.	0
MOXD1	-0,125367013	-0,208805464	0	N.A.	0
MYCN	-0,125367953	0,038206712	0	N.A.	0
SLC25A46	-0,125372347	0,017090273	0	N.A.	0
ZNF527	-0,125397276	-0,152970958	0	N.A.	0
PSMD5	-0,125444043	-0,145831875	0	N.A.	0
DOK7	-0,125447297	-0,326800046	0	N.A.	0
CCDC134	-0,12552337	-0,115694312	0	N.A.	0
RASSF8-AS1	-0,125544217	-0,047438404	0	N.A.	0
LIPT2	-0,125559888	-0,259929209	0	N.A.	0

CABYR	-0,12560785	-0,348743052	0	N.A.	0
C21orf33	-0,125631298	-0,045686448	0	N.A.	0
CACHD1	-0,125658989	-0,002184318	0	N.A.	0
PPP1R35	-0,125693635	0,019985914	0	N.A.	0
TMEM241	-0,125702374	-0,148711396	0	N.A.	0
RANBP2	-0,125728111	0,290041307	0	N.A.	0
ZNF507	-0,125780307	0,035098376	0	N.A.	0
IFT88	-0,125780829	-0,014895383	0	N.A.	0
TRAF3IP1	-0,125870482	0,019910416	0	N.A.	0
CTSH	-0,125977905	0,259115464	0	N.A.	0
DEXI	-0,125999549	0,115211667	0	N.A.	0
NRTN	-0,126008413	-0,059103407	0	N.A.	0
RP11-533E19	-0,126021845	-0,217552571	0	N.A.	0
CTD-2203K17	-0,126044438	-0,123915896	0	N.A.	0
MRAS	-0,126142611	-0,188850404	0	N.A.	0
PPP2R5C	-0,126197765	-0,175875556	0	N.A.	0
AP3M1	-0,126221862	0,214540555	0	N.A.	0
ADCY1	-0,126236642		0	N.A.	0
C20orf144	-0,126270883	0,222289822	0	N.A.	0
RP11-418J17	-0,126290062	0,016079699	0	N.A.	0
PMM2	-0,126304939	-0,219199373	0	N.A.	0
PDS5A	-0,126491201	0,180750142	0	N.A.	0
SERPINA11	-0,126511251		0	N.A.	0
KCNJ3	-0,126565207	0,078749144	0	N.A.	0
HOXB5	-0,126612833	0,13633162	0	N.A.	0
PTPRS	-0,126677354	-0,277010935	0	N.A.	0
RP11-93H24.	-0,126732872	-0,103224128	0	N.A.	0
TYRO3	-0,12675732	0,364233744	0	N.A.	0
ATM	-0,126764006	-0,038545592	0	N.A.	0
FBXO45	-0,126765469	-0,081034976	0	N.A.	0
C6orf57	-0,126791876	-0,302905269	0	N.A.	0
CXCR6	-0,126800934	-0,255908773	0	N.A.	0
ZKSCAN8	-0,126928739	0,009141305	0	N.A.	0

RP11-835E18	-0,126956383	-0,267219395	0	N.A.	0
LINC01311	-0,127006237	-0,132484527	0	N.A.	0
RP11-1069G1	-0,127052415	0,119090677	0	N.A.	0
CH507-154B1	-0,127062429	-0,159118561	0	N.A.	0
RPN1	-0,127066913	0,047552707	0	N.A.	0
TDG	-0,127116722	0,32276043	0	N.A.	0
CD28	-0,127118127	-0,088758096	0	N.A.	0
RP13-401N8.	-0,127141007	-0,316515634	0	N.A.	0
RP11-589C21	-0,127166016	0,036932738	0	N.A.	0
ITGA9	-0,127180791	-0,011294976	0	N.A.	0
HEXIM2	-0,127208399	-0,304651005	0	N.A.	0
FADD	-0,127212633	-0,030063941	0	N.A.	0
USP45	-0,127214915	0,314932644	0	N.A.	0
VPS26B	-0,127334644	0,159119139	0	N.A.	0
UBA1	-0,12734213	0,418028593	0	N.A.	0
BLVRA	-0,127414306	-0,20023982	0	N.A.	0
CSTF2T	-0,127495195	-0,083694516	0	N.A.	0
ORM2	-0,127504298	-0,125740945	0	N.A.	0
GRAP2	-0,127509724	-0,224931514	0	N.A.	0
FAM86C1	-0,127546176	-0,082168892	0	N.A.	0
HCG14	-0,127546488	-0,111644555	0	N.A.	0
ATOH1	-0,127567028	-0,068849792	0	N.A.	0
PART1	-0,127745104	-0,170974327	0	N.A.	0
RP11-703H8.	-0,127751322	-0,040043206	0	N.A.	0
TRMT2B	-0,127789793	-0,086424507	0	N.A.	0
GPKOW	-0,127791204	0,021165169	0	N.A.	0
RP5-1057I20	-0,127901099		0	N.A.	0
CXorf56	-0,127958753	-0,353384326	0	N.A.	0
EIF2AK2	-0,127978404	0,092692098	0	N.A.	0
RP5-1171I10	-0,127997965	-0,048966773	0	N.A.	0
IQCC	-0,128045414	-0,084771638	0	N.A.	0
PRRC2A	-0,128093899	-0,004007738	0	N.A.	0
SUPT3H	-0,128120477	-0,348639053	0	N.A.	0

CTD-3025N2(	-0,128140054	-0,046661525	0	N.A.	0
RP11-37C7.3	-0,128194097	0,023149295	0	N.A.	0
AGXT	-0,128266303	-0,246739678	0	N.A.	0
C18orf54	-0,128275413	-0,300336428	0	N.A.	0
KIAA0922	-0,128426448	-0,21792434	0	N.A.	0
CTD-2287O1(	-0,128536954	-0,100754631	0	N.A.	0
ZNF219	-0,12857292	-0,134934072	0	N.A.	0
CNTROB	-0,128599029	-0,098753978	0	N.A.	0
HSPA2	-0,128603486	-0,1044291	0	N.A.	0
CCAT2	-0,128629145		0	N.A.	0
TSPYL1	-0,128708696	0,015326698	0	N.A.	0
AF064858.7	-0,12871355		0	N.A.	0
PDCL	-0,128789718	0,113525431	0	N.A.	0
LYRM1	-0,128809899	-0,213170258	0	N.A.	0
RP11-370I10	-0,128891739	0,086637852	0	N.A.	0
CTB-55O6.13	-0,128918433		0	N.A.	0
CD37	-0,129031465	-0,285975368	0	N.A.	0
S100A1	-0,129072074	-0,191979227	0	N.A.	0
UBL7-AS1	-0,129157451	0,084535491	0	N.A.	0
C1QC	-0,129162919	-0,250483387	0	N.A.	0
DSCR3	-0,129248502	-0,042324734	0	N.A.	0
CCL25	-0,129250658	0,11182054	0	N.A.	0
TPGS1	-0,129272543	0,209981235	0	N.A.	0
PSMA6	-0,129296595	-0,006447601	0	N.A.	0
MED31	-0,129426798	-0,176654288	0	N.A.	0
LCK	-0,12945266	-0,252067351	0	N.A.	0
CTD-2540B1!	-0,129455264	0,06294544	0	N.A.	0
RP11-544I20	-0,12945993	-0,029425528	0	N.A.	0
TMEM194A	-0,129466723	-0,320561115	0	N.A.	0
OSBPL9	-0,129533871	-0,041201257	0	N.A.	0
CTD-2235C1:	-0,129603519	0,161341183	0	N.A.	0
RP11-732A2:	-0,129659847	0,175008673	0	N.A.	0
HIP1	-0,129668254	-0,388807235	0	N.A.	0

PRKAG2-AS1	-0,129682114	-0,106726176	0	N.A.	0
TBC1D16	-0,12972826	-0,328181027	0	N.A.	0
DYX1C1	-0,129739813	-0,250003902	0	N.A.	0
RP11-298I3.1	-0,129768742	0,017838827	0	N.A.	0
RP11-490B18	-0,129781685	-0,065385718	0	N.A.	0
ECEL1	-0,129790772		0	N.A.	0
ANKRD18B	-0,129804539	0,328980266	0	N.A.	0
ZNF584	-0,129804708	0,011452902	0	N.A.	0
CD2	-0,129824354	-0,205776604	0	N.A.	0
CPSF4L	-0,129946158	0,402736728	0	N.A.	0
IPO8	-0,129948696	-0,066637921	0	N.A.	0
CEP250	-0,129966307	-0,146442515	0	N.A.	0
ARC	-0,130013566	0,189990657	0	N.A.	0
NOTCH4	-0,130023192	-0,10588439	0	N.A.	0
CIZ1	-0,130056964	0,247501966	0	N.A.	0
CTC-338M12	-0,130075892	-0,15041267	0	N.A.	0
DVL2	-0,130101979	-0,016754898	0	N.A.	0
CERS4	-0,130132588		0	N.A.	0
TUBG2	-0,130177955	0,10416771	0	N.A.	0
ERCC3	-0,130179656	0,157326227	0	N.A.	0
GLYCTK	-0,130204301	0,262325978	0	N.A.	0
SULT1A3	-0,130274516	-0,09081712	0	N.A.	0
LSAMP	-0,130349057	-0,097481409	0	N.A.	0
NCAM1	-0,130411794		0	N.A.	0
DHX16	-0,130416538	-0,075695354	0	N.A.	0
RP11-536C5.	-0,130515144	0,087942304	0	N.A.	0
PFN4	-0,130587557		0	N.A.	0
MED19	-0,130680235	0,091659113	0	N.A.	0
IL26	-0,130681575	0,081138471	0	N.A.	0
AC105760.2	-0,130858726	-0,271075883	0	N.A.	0
CEP89	-0,130936163	-0,189406766	0	N.A.	0
SLC16A11	-0,130977462	-0,055280489	0	N.A.	0
GSG2	-0,130989555	-0,139972754	0	N.A.	0

RP11-20D14.	-0,131013339	-0,064815792	0	N.A.	0
AF064858.6	-0,131035692	-0,016912494	0	N.A.	0
CNPY4	-0,131082004	0,181988131	0	N.A.	0
EWSAT1	-0,131083408	0,096295576	0	N.A.	0
XPNPEP1	-0,131119026	0,102833573	0	N.A.	0
TMED9	-0,131131212	0,430478728	0	N.A.	0
CTA-246H3.1	-0,131205422		0	N.A.	0
CCBL1	-0,131222519	-0,021433175	0	N.A.	0
ZDHHC12	-0,131249147	0,211003127	0	N.A.	0
USP20	-0,131270434	0,097808371	0	N.A.	0
RP11-326C3.	-0,131366435	-0,069346899	0	N.A.	0
CLP1	-0,131454986	-0,041134123	0	N.A.	0
RAD17	-0,131577377	-0,181912107	0	N.A.	0
SMN1	-0,131587936	-0,142736407	0	N.A.	0
CMTR1	-0,131724454	0,234266697	0	N.A.	0
CH507-39O4	-0,131785753	-0,149520799	0	N.A.	0
TCEAL7	-0,131817138	0,003529533	0	N.A.	0
KIAA0825	-0,131872726	-0,072647039	0	N.A.	0
LCMT2	-0,13192071	-0,026356688	0	N.A.	0
ZNF346	-0,131973308	-0,216341636	0	N.A.	0
ZNF569	-0,131999401		0	N.A.	0
PLAC4	-0,132153964	0,026674497	0	N.A.	0
TCEANC2	-0,132191881	0,029512398	0	N.A.	0
XPC	-0,132194079	0,323991381	0	N.A.	0
RP11-96C23.	-0,132223862		0	N.A.	0
MGST3	-0,132251024	0,178260197	0	N.A.	0
NTMT1	-0,132340821	0,107078145	0	N.A.	0
RP11-471B22	-0,132475314		0	N.A.	0
WASIR2	-0,132551375	0,182659064	0	N.A.	0
CTD-2366F13	-0,132552136	-0,139422953	0	N.A.	0
RNF166	-0,132634377	-0,343704774	0	N.A.	0
C5orf24	-0,132650425	0,031294879	0	N.A.	0
LFNG	-0,132656175	0,087188253	0	N.A.	0

FGFBP3	-0,132657848	-0,114367287	0	N.A.	0
TOR1A	-0,132791857	0,112786934	0	N.A.	0
TSTD3	-0,132879222	-0,358656207	0	N.A.	0
B4GALNT4	-0,132918126	0,128721318	0	N.A.	0
LRRC69	-0,132976487	0,025930234	0	N.A.	0
ELMO1	-0,133012006	-0,016995884	0	N.A.	0
NR2F2	-0,133059191	0,066512644	0	N.A.	0
HOXD9	-0,133155273	-0,044643211	0	N.A.	0
ADAD2	-0,13322187		0	N.A.	0
CTD-2033A1(	-0,133222584	0,191237451	0	N.A.	0
HIST1H1D	-0,133255462	-0,403279524	0	N.A.	0
PIH1D2	-0,133263356	-0,150896005	0	N.A.	0
TNPO3	-0,133453715	0,00107704	0	N.A.	0
ABRACL	-0,133465133	-0,052291439	0	N.A.	0
DUSP9	-0,133473952	-0,173726784	0	N.A.	0
RP11-500C11	-0,133476428	-0,114267837	0	N.A.	0
TRIP6	-0,13348733	0,135827289	0	N.A.	0
RP11-342M1	-0,133495823		0	N.A.	0
RP11-434E6.4	-0,133616553	-0,084834724	0	N.A.	0
FZD2	-0,133821814		0	N.A.	0
LINC01356	-0,133911382	-0,132342084	0	N.A.	0
NFRKB	-0,133922629	0,284833445	0	N.A.	0
RP11-7I15.4	-0,133936952		0	N.A.	0
TSPAN13	-0,134049095	0,178770632	0	N.A.	0
BLOC1S5	-0,134207527	-0,18951983	0	N.A.	0
EEF1A1	-0,134261805	-0,385434411	0	N.A.	0
CTD-2033D15	-0,134300917		0	N.A.	0
LINC01485	-0,134365697	0,02632041	0	N.A.	0
VASH2	-0,134388267	-0,085947647	0	N.A.	0
DCLRE1B	-0,134406298	-0,431335544	0	N.A.	0
COCH	-0,134517901	-0,33805875	0	N.A.	0
BEND3	-0,134528603	-0,089155029	0	N.A.	0
C1QTNF9B-A	-0,134567281	-0,244910196	0	N.A.	0

bP-21264C1.	-0,134735181	-0,067597786	0	N.A.	0
PPM1D	-0,134738445	-0,021552027	0	N.A.	0
GGCX	-0,134761554	-0,188111859	0	N.A.	0
EXOC6	-0,134817656	0,052138986	0	N.A.	0
PEX11G	-0,134844892	-0,089333499	0	N.A.	0
SERPINH1	-0,134856373	0,127891726	0	N.A.	0
FASTKD1	-0,134857425	-0,189250206	0	N.A.	0
MPG	-0,134859883	-0,226530707	0	N.A.	0
ICAM3	-0,134873184	-0,387607061	0	N.A.	0
ZNF681	-0,134960373	-0,21272167	0	N.A.	0
GGNBP2	-0,135020921	-0,038359209	0	N.A.	0
FLVCR1	-0,13506181	0,04405803	0	N.A.	0
TMEM143	-0,135136071	0,045012914	0	N.A.	0
C10orf88	-0,13515141	-0,295834461	0	N.A.	0
CST1	-0,135301169	-0,016696397	0	N.A.	0
ZFP69B	-0,135308551	0,024353275	0	N.A.	0
CC2D1A	-0,135348464	0,202788239	0	N.A.	0
GDF9	-0,135356238	-0,002512435	0	N.A.	0
PGLS	-0,135368896	0,007216815	0	N.A.	0
C17orf96	-0,135559703	-0,194610841	0	N.A.	0
RP5-956O18.	-0,135883502	-0,000800248	0	N.A.	0
AP000251.3	-0,135911559	-0,034333598	0	N.A.	0
PCM1	-0,135926486	0,075130768	0	N.A.	0
XPO6	-0,135983697	0,166634683	0	N.A.	0
SPEF2	-0,135984755	-0,116752426	0	N.A.	0
PYCARD	-0,135985664	0,083952708	0	N.A.	0
RECK	-0,136079019	0,114534034	0	N.A.	0
ZNF780B	-0,13610918	-0,326078819	0	N.A.	0
HSPB11	-0,136133987	0,006915578	0	N.A.	0
TAL2	-0,136154511	-0,039912307	0	N.A.	0
RP11-419J16	-0,136177113	-0,31653683	0	N.A.	0
PHF10	-0,136186232	-0,119504328	0	N.A.	0
EFCAB7	-0,136197185	-0,310012695	0	N.A.	0

GPRASP2	-0,136283407	0,290859869	0	N.A.	0
RP11-187E13	-0,136285046	-0,14157872	0	N.A.	0
AC009120.11	-0,136395825	0,267148671	0	N.A.	0
MLK7-AS1	-0,136400974		0	N.A.	0
RP11-84C10.	-0,136413071		0	N.A.	0
RP11-563N4.	-0,136413209	-0,147874781	0	N.A.	0
RP11-35N6.1	-0,136511454	0,192867929	0	N.A.	0
DMRT1	-0,13655764	-0,006323414	0	N.A.	0
KCNK15-AS1	-0,13655947	-0,200641934	0	N.A.	0
SGCB	-0,136564902	-0,048599036	0	N.A.	0
RP11-395A13	-0,136588284	0,024577161	0	N.A.	0
FMNL3	-0,136630797	-0,194401236	0	N.A.	0
CSMD2	-0,136700794		0	N.A.	0
ANKRD54	-0,13677546	-0,222062889	0	N.A.	0
POLR3D	-0,136836195	-0,060679132	0	N.A.	0
METTL22	-0,136857623	-0,164612726	0	N.A.	0
PSMG3-AS1	-0,136952445	-0,229510103	0	N.A.	0
ZNF639	-0,137024427	-0,106228827	0	N.A.	0
RP4-639F20.	-0,137034624	0,018205846	0	N.A.	0
LHPP	-0,137085762	-0,184024996	0	N.A.	0
RP11-109G23	-0,137106446	0,052596177	0	N.A.	0
LINC01021	-0,13717694	-0,056663581	0	N.A.	0
RP11-372K14	-0,137241191	0,008590929	0	N.A.	0
ITPR2	-0,137279071	-0,023026427	0	N.A.	0
FOXO6	-0,137315838	0,314314213	0	N.A.	0
VENTX	-0,137321789		0	N.A.	0
RP11-179B2.	-0,137327212	-0,126953179	0	N.A.	0
RASIP1	-0,13738827	-0,034166164	0	N.A.	0
STMND1	-0,13738985	-0,273987565	0	N.A.	0
RP13-1032I1	-0,137403633	-0,048189899	0	N.A.	0
SNAI1	-0,137439242	-0,058855003	0	N.A.	0
FAM225A	-0,137452892		0	N.A.	0
MAPK12	-0,137458506	-0,049100827	0	N.A.	0

RP11-452K12	-0,137558238	0,166813562	0	N.A.	0
MT1A	-0,137563741	-0,313164801	0	N.A.	0
MTG1	-0,137566131	0,040070229	0	N.A.	0
RP11-320N7.	-0,137593776		0	N.A.	0
TRAF2	-0,137619316	0,025764982	0	N.A.	0
HOXA-AS2	-0,137669844	0,067631886	0	N.A.	0
CNP	-0,137802557	0,113425538	0	N.A.	0
GDF10	-0,137889552		0	N.A.	0
RP11-382D12	-0,137932297	-0,049602633	0	N.A.	0
RP11-1000B6	-0,13793751	0,155398628	0	N.A.	0
SNAP47	-0,137973484	-0,186010889	0	N.A.	0
SYP	-0,137973977	0,071944616	0	N.A.	0
DTNB	-0,138012541	-0,047481048	0	N.A.	0
AC087294.2	-0,138039997	-0,006718913	0	N.A.	0
RP11-22C11.	-0,13808396	-0,119473985	0	N.A.	0
FBXO17	-0,138108233	-0,016697065	0	N.A.	0
ACSS3	-0,138198031		0	N.A.	0
MFSD3	-0,138206621	0,048952625	0	N.A.	0
PARP16	-0,138208386	-0,004688209	0	N.A.	0
TMEM222	-0,138402088	-0,025623388	0	N.A.	0
LINC00493	-0,138425977	-0,170950314	0	N.A.	0
U91328.19	-0,138442482	0,075459411	0	N.A.	0
ALDH8A1	-0,138470348	-0,053885272	0	N.A.	0
TDRD3	-0,138573033	0,085027582	0	N.A.	0
SAFB2	-0,138573404	0,126894659	0	N.A.	0
LOH12CR2	-0,138826647	0,185449086	0	N.A.	0
PPP1R1A	-0,138861432	-0,059431967	0	N.A.	0
NMNAT1	-0,138867274	-0,317343704	0	N.A.	0
MAEA	-0,138885939	0,113238285	0	N.A.	0
ADNP-AS1	-0,138898703	0,109635043	0	N.A.	0
XXbac-BPG25	-0,13895918	-0,288234772	0	N.A.	0
PPT2	-0,138998103	0,047361281	0	N.A.	0
RP11-307L14	-0,139007494	-0,0999624	0	N.A.	0

RNF115	-0,139008027	-0,195898979	0	N.A.	0
PCGF5	-0,139045695	-0,043224429	0	N.A.	0
ADAL	-0,139048561	-0,204194805	0	N.A.	0
DNAJC10	-0,139081422	-0,02215739	0	N.A.	0
AC010894.3	-0,139110193	0,27457982	0	N.A.	0
LIN7A	-0,139125638	-0,088778038	0	N.A.	0
TDRP	-0,139253178		0	N.A.	0
SRRM5	-0,139281679	0,020249443	0	N.A.	0
C2orf48	-0,139412885	-0,081654182	0	N.A.	0
CAPN7	-0,139415048	0,408833412	0	N.A.	0
LINC01573	-0,139426136		0	N.A.	0
ACTR5	-0,13942825	0,21407844	0	N.A.	0
RP11-332H14	-0,139721086	-0,049788484	0	N.A.	0
ANKRD34B	-0,139767788		0	N.A.	0
CCZ1B	-0,139801714	0,171445265	0	N.A.	0
RLN1	-0,13981044		0	N.A.	0
ARHGDIB	-0,139820713	-0,480614547	0	N.A.	0
ZNF143	-0,13982809	-0,043477659	0	N.A.	0
USP46-AS1	-0,139843409	-0,31179807	0	N.A.	0
TAS2R30	-0,139900366	-0,196281515	0	N.A.	0
IGFL4	-0,13999486	0,349206214	0	N.A.	0
OLFM4	-0,140044751	-0,024729785	0	N.A.	0
CTC-461F20.1	-0,140090483	0,009955539	0	N.A.	0
DPP7	-0,140123305	-0,089275862	0	N.A.	0
TCTE3	-0,140167617	0,005604715	0	N.A.	0
CTC-444N24.	-0,140199683		0	N.A.	0
LANCL1	-0,140229095	-0,098900507	0	N.A.	0
RRP8	-0,140253568	-0,108713443	0	N.A.	0
NPW	-0,14025712	-0,119820643	0	N.A.	0
BMS1	-0,140354712	0,296289597	0	N.A.	0
RP11-313P13	-0,140428505	-0,277699064	0	N.A.	0
HMGCL	-0,140467289	0,176111126	0	N.A.	0
RP11-646I6.6	-0,140525614	0,007385164	0	N.A.	0

TMEM218	-0,140574329	-0,257153826	0	N.A.	0
GRM8	-0,140577286	-0,19163009	0	N.A.	0
FAM133B	-0,140589717	0,04220408	0	N.A.	0
BSPRY	-0,140598948	0,091369821	0	N.A.	0
ASB8	-0,140738101	-0,203507816	0	N.A.	0
IGFLR1	-0,140891151	-0,052935518	0	N.A.	0
C1orf186	-0,140936857	-0,119191746	0	N.A.	0
LRRC40	-0,140948958	-0,090943776	0	N.A.	0
CTD-2256P15	-0,141072621	-0,162905158	0	N.A.	0
RP11-141B14	-0,141077655	0,07469367	0	N.A.	0
GABPB1-AS1	-0,141084157	0,017494566	0	N.A.	0
CCDC175	-0,141104024	0,117781459	0	N.A.	0
Z83851.4	-0,141210586	-0,123529564	0	N.A.	0
CEP72	-0,141248008	-0,146617013	0	N.A.	0
LRP8	-0,141323194	0,061033319	0	N.A.	0
EPHX4	-0,141331726	-0,024268374	0	N.A.	0
PLBD1	-0,141468115	0,07419372	0	N.A.	0
ZNF502	-0,141509972	-0,298002816	0	N.A.	0
ILVBL	-0,141519478	0,093136052	0	N.A.	0
PKP2	-0,14152061	0,11301884	0	N.A.	0
RP11-120D5.	-0,141571379	-0,121622286	0	N.A.	0
RABL2A	-0,141669901	0,057211809	0	N.A.	0
RP11-596C23	-0,141680456	0,01477941	0	N.A.	0
AMPD2	-0,141687107	-0,137590325	0	N.A.	0
NRSN2	-0,141728961	0,057039477	0	N.A.	0
DYNLL1	-0,141776545	-0,132775522	0	N.A.	0
ZNF512	-0,14181815	-0,149343279	0	N.A.	0
GPR63	-0,141967039		0	N.A.	0
HEXB	-0,142053771	-0,040790393	0	N.A.	0
RHOV	-0,142105729	0,186266782	0	N.A.	0
ANAPC16	-0,142131393	-0,13973098	0	N.A.	0
C5	-0,142216562	-0,156181568	0	N.A.	0
TMEM101	-0,142357104	0,07752224	0	N.A.	0

FSCN1	-0,142466984	0,071219736	0	N.A.	0
RP11-435O5.	-0,142480544	-0,243674385	0	N.A.	0
TOMM40L	-0,142581244	-0,338869692	0	N.A.	0
TMCO6	-0,142698472	0,034419512	0	N.A.	0
CTD-3193O1	-0,142717658	0,069994528	0	N.A.	0
ZNF573	-0,142779037	0,124386626	0	N.A.	0
AP000439.3	-0,142958248	0,149313008	0	N.A.	0
GRK1	-0,143017413	-0,046240502	0	N.A.	0
RP11-138J23	-0,143057382	0,018460532	0	N.A.	0
MCMDC2	-0,143069345	-0,269320065	0	N.A.	0
RBM45	-0,143180148	-0,183555989	0	N.A.	0
TMSB15B	-0,143210833	-0,036706573	0	N.A.	0
DMKN	-0,143234406	-0,101025631	0	N.A.	0
NMU	-0,143259244	-0,216599143	0	N.A.	0
ARFGAP2	-0,143314503	0,309638255	0	N.A.	0
RP11-191L17	-0,143355512	0,061668585	0	N.A.	0
S100PBP	-0,143360553	-0,243479818	0	N.A.	0
HOTAIR	-0,143641863	0,070210936	0	N.A.	0
FAM57B	-0,143696765		0	N.A.	0
MBD3	-0,14369686	-0,053266332	0	N.A.	0
AC002310.7	-0,143699778	-0,222457682	0	N.A.	0
C1orf52	-0,143784415	-0,00047364	0	N.A.	0
TAF1B	-0,143885019	-0,011383163	0	N.A.	0
CTB-193M12	-0,143894463	-0,04010113	0	N.A.	0
STK38L	-0,14394845	-0,16446727	0	N.A.	0
AXIN2	-0,143981026	0,091604158	0	N.A.	0
CNIH4	-0,144036938	0,02589252	0	N.A.	0
RNF130	-0,144061684	-0,38285293	0	N.A.	0
DCAF12	-0,144093255	-0,151580117	0	N.A.	0
KLHL13	-0,144146307		0	N.A.	0
POC1B-GALN	-0,144215015	0,002572478	0	N.A.	0
HKR1	-0,144242704	0,042025748	0	N.A.	0
CH507-154B1	-0,144268294	-0,020326036	0	N.A.	0

PIWIL1	-0,144300545	0,208534114	0	N.A.	0
C14orf93	-0,144306036	-0,186565907	0	N.A.	0
HIST1H2BL	-0,144340262	-0,27040894	0	N.A.	0
ALG13	-0,144350785	-0,244877447	0	N.A.	0
SUCLG2-AS1	-0,1443517	-0,067778331	0	N.A.	0
ZNF181	-0,144385389	-0,038850952	0	N.A.	0
CCHCR1	-0,144402761	-0,243669259	0	N.A.	0
NEUROG2	-0,144442101	-0,019387735	0	N.A.	0
IMPACT	-0,14446441	-0,313327625	0	N.A.	0
FERMT1	-0,144585571	0,163322298	0	N.A.	0
IFT46	-0,144624096	0,164087858	0	N.A.	0
RP11-290D2.	-0,144768176	-0,040637056	0	N.A.	0
DLL3	-0,144832871	-0,053393967	0	N.A.	0
ROMO1	-0,144857042	-0,240793653	0	N.A.	0
POLE	-0,144867139	0,108756827	0	N.A.	0
PEX19	-0,144874731	0,072751224	0	N.A.	0
BAAT	-0,144920426	-0,171392249	0	N.A.	0
CTCF	-0,14496145	-0,022594471	0	N.A.	0
RP13-895J2.3	-0,145034104	0,028987564	0	N.A.	0
RP13-582O9.	-0,145057523	0,059945963	0	N.A.	0
AC022182.3	-0,145078137	-0,023315451	0	N.A.	0
KIF12	-0,145084453	-0,000723961	0	N.A.	0
RP11-326C3.	-0,145093834	-0,355539428	0	N.A.	0
EIF2B5-AS1	-0,145137607	0,022452701	0	N.A.	0
ZNF85	-0,145268984	-0,235068607	0	N.A.	0
POLRMT	-0,145349449	-0,070095175	0	N.A.	0
MED11	-0,145386717	-0,312667696	0	N.A.	0
SMPD2	-0,145437254	-0,008153443	0	N.A.	0
GTF2H2C	-0,145458263	0,161315407	0	N.A.	0
MTERF4	-0,145461842	0,038672986	0	N.A.	0
RP11-110I1.1	-0,145471302	0,086521979	0	N.A.	0
LEFTY1	-0,145474824	-0,186553648	0	N.A.	0
ZC3H12B	-0,145514071	-0,108954124	0	N.A.	0

LINC01550	-0,145540162	-0,149476725	0	N.A.	0
LINC01132	-0,145543604		0	N.A.	0
PRR34	-0,145586134	-0,074355979	0	N.A.	0
FHIT	-0,145669461	-0,226471784	0	N.A.	0
ITFG2	-0,145694809	-0,108428377	0	N.A.	0
SEC23IP	-0,145820825	0,022766411	0	N.A.	0
TUT1	-0,145839674	0,120058579	0	N.A.	0
POLI	-0,145847126	0,176811642	0	N.A.	0
IGF2-AS	-0,145865021		0	N.A.	0
RP11-324E6.	-0,145925358	-0,009499833	0	N.A.	0
DPYSL3	-0,145940321	-0,078798167	0	N.A.	0
SMAD1	-0,145943401	0,025037254	0	N.A.	0
RP11-573G6.	-0,146052776		0	N.A.	0
ZKSCAN5	-0,146055887	-0,398196739	0	N.A.	0
CCDC96	-0,146065649	0,052723974	0	N.A.	0
RORA-AS1	-0,146070021	-0,051848828	0	N.A.	0
SLC16A1-AS1	-0,146071503	0,009824849	0	N.A.	0
C10orf95	-0,146334476	-0,05230672	0	N.A.	0
RP11-61K9.3	-0,146442569	-0,047961659	0	N.A.	0
ARHGAP33	-0,14645237	0,10132612	0	N.A.	0
KDM2B	-0,14645552	-0,08176085	0	N.A.	0
DHX37	-0,146593112	-0,144099056	0	N.A.	0
ZNF446	-0,146605303	0,086338072	0	N.A.	0
IRAK1	-0,146660422	0,345203981	0	N.A.	0
GPN2	-0,146756543	-0,080518339	0	N.A.	0
KCTD14	-0,146872015	-0,231757449	0	N.A.	0
MYH13	-0,146898027		0	N.A.	0
RP11-872J21	-0,14691904	0,076348545	0	N.A.	0
KANK1	-0,146985397	-0,27241551	0	N.A.	0
MAFG-AS1	-0,147014378	-0,119581467	0	N.A.	0
CCND2	-0,147025185	0,041400324	0	N.A.	0
GPR85	-0,147031063	0,309070572	0	N.A.	0
C1orf110	-0,147040842		0	N.A.	0

TAF7L	-0,147050436	0,013501112	0	N.A.	0
UBP1	-0,147056224	0,046256992	0	N.A.	0
SLC26A8	-0,147146101	-0,097738443	0	N.A.	0
DENND2D	-0,147151317	-0,124702878	0	N.A.	0
AC005307.1	-0,14717877	-0,248705759	0	N.A.	0
NIFK-AS1	-0,147185188	0,125458365	0	N.A.	0
ZNF43	-0,147206554	0,157971074	0	N.A.	0
NINJ2	-0,147247133	-0,105196017	0	N.A.	0
MT3	-0,14725281	-0,27431172	0	N.A.	0
DNAH10	-0,147256313	0,084979714	0	N.A.	0
NSUN6	-0,147269091	-0,037261512	0	N.A.	0
MED20	-0,147368545	0,123805587	0	N.A.	0
POU5F1B	-0,147404742	-0,2525191	0	N.A.	0
SAMD3	-0,147469319	-0,111201167	0	N.A.	0
BAHCC1	-0,147507242	-0,183081643	0	N.A.	0
DYNLL1-AS1	-0,147612552	0,020588446	0	N.A.	0
COL2A1	-0,147639312	0,057332223	0	N.A.	0
LINC01116	-0,147712172	-0,08574458	0	N.A.	0
TEX2	-0,147712826	0,007786672	0	N.A.	0
TOR2A	-0,147768193	-0,085709736	0	N.A.	0
AC018890.6	-0,147808207		0	N.A.	0
RP11-394B2.	-0,147917785	0,011912371	0	N.A.	0
C19orf25	-0,147942398	0,212213892	0	N.A.	0
CFH	-0,147972723	0,135239042	0	N.A.	0
IFT122	-0,147975143	-0,08714326	0	N.A.	0
TMEM19	-0,147994814	-0,1423572	0	N.A.	0
SIT1	-0,148104663	-0,054592829	0	N.A.	0
SPATA2	-0,148306228	0,01399784	0	N.A.	0
C2CD4D	-0,148346082	-0,008720564	0	N.A.	0
CXCL13	-0,148393054	0,119791062	0	N.A.	0
RP11-178L8.	-0,148417726	0,070474217	0	N.A.	0
VAV3	-0,148555634	-0,412408916	0	N.A.	0
RP11-545E17	-0,148584848	-0,0316898	0	N.A.	0

HIST1H3B	-0,148630737	-0,407449669	0	N.A.	0
ZNF414	-0,148711663	-0,074233031	0	N.A.	0
CSRP2	-0,148782696	-0,11895873	0	N.A.	0
TMEM161B-/	-0,14886932	0,021709174	0	N.A.	0
CCDC101	-0,148880181	-0,085183613	0	N.A.	0
ANKRD13B	-0,148929175	-0,132337873	0	N.A.	0
SMIM7	-0,148933706	0,047857484	0	N.A.	0
NANOS3	-0,148953977	-0,144160983	0	N.A.	0
CLCC1	-0,148961244	-0,066710106	0	N.A.	0
PDIA4	-0,149048748	0,055459361	0	N.A.	0
KLK12	-0,149084295	-0,01600082	0	N.A.	0
CDHR1	-0,149216928	-0,085605109	0	N.A.	0
DHX40	-0,14922583	-0,063740566	0	N.A.	0
SUZ12	-0,149264392	0,023016818	0	N.A.	0
OLFML2B	-0,149328294	0,018482093	0	N.A.	0
VIPR2	-0,149385366	0,141741995	0	N.A.	0
C17orf97	-0,149500006	-0,158914512	0	N.A.	0
INTS2	-0,149516961	-0,05753274	0	N.A.	0
PTPRD	-0,14952643	-0,161325648	0	N.A.	0
UGGT2	-0,149544063	0,111340129	0	N.A.	0
MCF2L-AS1	-0,149554304	-0,319111117	0	N.A.	0
LINC01124	-0,149576978	-0,125064762	0	N.A.	0
RP11-89K10.	-0,149613879	0,007366529	0	N.A.	0
SPRYD7	-0,149623289	0,028099135	0	N.A.	0
CD7	-0,149740198	-0,071420764	0	N.A.	0
APOC1	-0,149746485	-0,33809682	0	N.A.	0
QSOX2	-0,149810498	0,101303508	0	N.A.	0
CCDC7	-0,149817595	0,033231954	0	N.A.	0
LRRC74B	-0,149826309		0	N.A.	0
GPX7	-0,149892875	-0,152112107	0	N.A.	0
PLCG1	-0,149901428	-0,088850941	0	N.A.	0
IL17A	-0,149903605		0	N.A.	0
FAM169A	-0,149980057	-0,124246407	0	N.A.	0

RP11-440D17	-0,149995763	-0,326303357	0	N.A.	0
PEG10	-0,150014146	0,056864792	0	N.A.	0
RP11-579D7.	-0,150038588	-0,359293634	0	N.A.	0
UBAC2-AS1	-0,150091484	-0,104989322	0	N.A.	0
DHX35	-0,150127686	-0,253027447	0	N.A.	0
OR2B6	-0,150212555	0,047964544	0	N.A.	0
VKORC1	-0,150279287	-0,07037885	0	N.A.	0
ZNF444	-0,150754011	-0,159775842	0	N.A.	0
SERPINI1	-0,150759004	-0,037380662	0	N.A.	0
CCL21	-0,150799287	-0,193853282	0	N.A.	0
PTP4A3	-0,150812558	-0,338014875	0	N.A.	0
UBIAD1	-0,150900887	-0,092730181	0	N.A.	0
MLK4	-0,150908287	-0,071705107	0	N.A.	0
ANKDD1B	-0,150977014	-0,054222712	0	N.A.	0
DCAF16	-0,151009587	-0,273279202	0	N.A.	0
CSPG4	-0,151053541	-0,168310646	0	N.A.	0
MFAP1	-0,151054942	0,070654919	0	N.A.	0
RP11-318A15	-0,15107905	-0,153034203	0	N.A.	0
RP1-118J21.2	-0,151107997	0,039543115	0	N.A.	0
KREMEN1	-0,151122983	0,206062712	0	N.A.	0
CLYBL	-0,151198986	-0,165730228	0	N.A.	0
IGFBP5	-0,151304922	-0,120675869	0	N.A.	0
NINJ1	-0,151356395	-0,001611206	0	N.A.	0
FAM43A	-0,151397375	-0,207106339	0	N.A.	0
ERVK3-1	-0,15142923	0,025453977	0	N.A.	0
AP3B2	-0,151473534		0	N.A.	0
MAST1	-0,151618081	0,106123649	0	N.A.	0
WDR83	-0,151697862	0,025421628	0	N.A.	0
WDR53	-0,151706539	-0,299298366	0	N.A.	0
C9orf43	-0,151715225	-0,058076329	0	N.A.	0
FAM132B	-0,151739795	0,002685007	0	N.A.	0
POLR2B	-0,151799142	0,167347778	0	N.A.	0
C9orf116	-0,151803328	-0,476348095	0	N.A.	0

MTMR2	-0,151823758	-0,276085904	0	N.A.	0
SEPN1	-0,151859661	0,142259117	0	N.A.	0
PKD2	-0,152025685	-0,038010388	0	N.A.	0
CTHRC1	-0,152090113	-0,070426379	0	N.A.	0
VAMP7	-0,152108462	0,023602321	0	N.A.	0
RP4-803J11.2	-0,152116281	0,043667932	0	N.A.	0
HNRNPL	-0,15214054	-0,022610537	0	N.A.	0
PSTPIP2	-0,152154901	-0,172400647	0	N.A.	0
EMC1	-0,152155532	-0,020666985	0	N.A.	0
CTC-260E6.6	-0,152187957	-0,057716874	0	N.A.	0
ZNF230	-0,152285215	-0,029920885	0	N.A.	0
REEP2	-0,152492444		0	N.A.	0
PAXBP1	-0,152521132	-0,046906644	0	N.A.	0
ZNF534	-0,15254687	0,0117763	0	N.A.	0
MUTYH	-0,152583986	-0,183918634	0	N.A.	0
XKRX	-0,152634387	-0,087660232	0	N.A.	0
ACY1	-0,152636674	0,109262572	0	N.A.	0
RP11-795F19	-0,152716635		0	N.A.	0
RPP14	-0,152723841	-0,151004627	0	N.A.	0
DDX41	-0,152772092	-0,287715962	0	N.A.	0
LOXL3	-0,152854025	-0,110000663	0	N.A.	0
TRIM44	-0,152862253	0,126326613	0	N.A.	0
RER1	-0,152954873	0,347742211	0	N.A.	0
HEY2	-0,153000341		0	N.A.	0
NRG2	-0,153043734		0	N.A.	0
HOXC4	-0,153051238		0	N.A.	0
SH2D2A	-0,153064975	-0,328739523	0	N.A.	0
RAB29	-0,153252889	-0,283829946	0	N.A.	0
ZDHHC6	-0,153282751	-0,140119527	0	N.A.	0
KIAA1429	-0,153331519	0,25177224	0	N.A.	0
SCPEP1	-0,153442808	0,057069365	0	N.A.	0
GAS2	-0,153528089	0,047875156	0	N.A.	0
IL33	-0,153602953	-0,21443964	0	N.A.	0

GALNT9	-0,15370802	-0,316585055	0	N.A.	0
PDS5B	-0,153732157	0,191865234	0	N.A.	0
CBX8	-0,153777099	-0,319435118	0	N.A.	0
BRF2	-0,153832505	-0,057236486	0	N.A.	0
LINC01424	-0,153844709		0	N.A.	0
RP11-140I16	-0,153914754	0,089930239	0	N.A.	0
RP11-66N11.	-0,15411292	0,076524851	0	N.A.	0
RP5-1065J22	-0,154113305	-0,057131479	0	N.A.	0
EID1	-0,154272211	-0,234714751	0	N.A.	0
FNTA	-0,154394923	-0,163709545	0	N.A.	0
TRIM13	-0,154420242	-0,053748136	0	N.A.	0
SEC11C	-0,154451332	-0,064688862	0	N.A.	0
C10orf126	-0,154573881		0	N.A.	0
RP5-968P14.	-0,154576259	0,025527421	0	N.A.	0
RITA1	-0,154600348	-0,207216766	0	N.A.	0
TCEA3	-0,154601253	-0,127444528	0	N.A.	0
AGK	-0,154767367	-0,350395871	0	N.A.	0
AC093627.9	-0,154770417	0,17785496	0	N.A.	0
CTD-2369P2.	-0,154781572	0,330854972	0	N.A.	0
SPINK4	-0,154906991	-0,175864494	0	N.A.	0
ZRSR2	-0,154992337	-0,143310079	0	N.A.	0
CDK11B	-0,155047489	0,329786732	0	N.A.	0
GFI1B	-0,155100696	-0,202862785	0	N.A.	0
FTSJ1	-0,155117218	-0,013473547	0	N.A.	0
DNAJC17	-0,155120827	0,307233122	0	N.A.	0
HS6ST2	-0,155181382	-0,050171265	0	N.A.	0
CPN1	-0,155225612		0	N.A.	0
ZNF3	-0,155229467	0,002233075	0	N.A.	0
VPRBP	-0,155232974	-0,153540721	0	N.A.	0
CALM3	-0,155331388	-0,219091465	0	N.A.	0
FKBP1A	-0,155425924	-0,402058575	0	N.A.	0
CPSF1	-0,155465053	0,004274577	0	N.A.	0
CTB-129O4.1	-0,155514095	-0,271008574	0	N.A.	0

CTC-537E7.2	-0,155516312	-0,126723606	0	N.A.	0
RP11-390F4.(	-0,155516675		0	N.A.	0
BZRAP1-AS1	-0,155518858	0,033785924	0	N.A.	0
AC007386.4	-0,15557468	-0,147354978	0	N.A.	0
ACOT2	-0,155577878	-0,071295483	0	N.A.	0
RP11-727F15	-0,155854647	0,105352709	0	N.A.	0
RP11-61J19.5	-0,155929428	-0,121887754	0	N.A.	0
FAM27C	-0,155953351	-0,053333811	0	N.A.	0
OLR1	-0,156071752	-0,310446633	0	N.A.	0
APH1A	-0,156077201	-0,015158689	0	N.A.	0
FITM2	-0,15608421	-0,18150842	0	N.A.	0
IGHV3-30	-0,156191029		0	N.A.	0
GPR180	-0,156206646	0,121529837	0	N.A.	0
C1orf216	-0,156259914	-0,285367123	0	N.A.	0
MTHFSD	-0,156271071	-0,146600594	0	N.A.	0
PARG	-0,156301083	0,233225558	0	N.A.	0
RAB8A	-0,156317548	0,013125021	0	N.A.	0
POLL	-0,156433067	-0,233516777	0	N.A.	0
KAT5	-0,15646768	-0,023231163	0	N.A.	0
CTB-55O6.8	-0,156474038	0,011276509	0	N.A.	0
GRM1	-0,156519619		0	N.A.	0
ALKBH1	-0,156705392	-0,20739138	0	N.A.	0
IQCG	-0,156815926	0,124625819	0	N.A.	0
PKN2-AS1	-0,156886388	0,070821212	0	N.A.	0
RGS13	-0,15688914	-0,214203215	0	N.A.	0
SF3B1	-0,157011458	0,025029085	0	N.A.	0
PSAT1	-0,157088855	-0,1166236	0	N.A.	0
ZNF689	-0,157089703	-0,098165958	0	N.A.	0
SLC25A51	-0,157230735	-0,312855653	0	N.A.	0
AKR1B1	-0,157231718	-0,163545187	0	N.A.	0
RP11-95P2.3	-0,157400825	0,003265002	0	N.A.	0
RFESD	-0,15746815	-0,111827546	0	N.A.	0
TMEM255B	-0,157482187	-0,301326991	0	N.A.	0

GNAS-AS1	-0,157485316		0	N.A.	0
CRYL1	-0,157549998	0,0620927	0	N.A.	0
ZDHHC24	-0,157556424	0,074372867	0	N.A.	0
PIK3R1	-0,157595041	-0,047128879	0	N.A.	0
CMTM7	-0,157663227	-0,064011834	0	N.A.	0
IGFBPL1	-0,157670305		0	N.A.	0
POFUT1	-0,157674692	-0,153322188	0	N.A.	0
CXXC1	-0,157702614	-0,032334271	0	N.A.	0
TRDMT1	-0,157723395	0,014670624	0	N.A.	0
APOPT1	-0,157744831	-0,014076507	0	N.A.	0
TAF1D	-0,157798001	-0,153605901	0	N.A.	0
ZCCHC9	-0,157845007	-0,133113229	0	N.A.	0
KRTAP5-2	-0,157900275	0,43059688	0	N.A.	0
TMEM147-A!	-0,158088341	-0,125310508	0	N.A.	0
TMEM254-A!	-0,158196952	-0,229615583	0	N.A.	0
C1orf233	-0,158245979	-0,073572011	0	N.A.	0
RAB2A	-0,158321365	0,070918468	0	N.A.	0
ARHGAP22	-0,158333133	-0,038055842	0	N.A.	0
ACOX2	-0,158379121	-0,293593474	0	N.A.	0
HYLS1	-0,158414106	-0,44206608	0	N.A.	0
PRRT1	-0,158420337	-0,068080845	0	N.A.	0
CEBPA-AS1	-0,1584409	-0,260637944	0	N.A.	0
TAF6L	-0,158454514	0,064426459	0	N.A.	0
CTD-2576D5.	-0,158465165	-0,079572355	0	N.A.	0
ASB13	-0,158581966	0,291966051	0	N.A.	0
AC004540.5	-0,158631255	0,45824634	0	N.A.	0
NOL9	-0,158651748	0,095111262	0	N.A.	0
IL20RA	-0,158671698	0,166091707	0	N.A.	0
LY6G6D	-0,158697519	-0,144267912	0	N.A.	0
SLC6A16	-0,158841626	0,002401309	0	N.A.	0
CTD-2385L22	-0,158985747	0,10286675	0	N.A.	0
RP11-290F5.	-0,159056152		0	N.A.	0
CD3G	-0,15909308	-0,136066519	0	N.A.	0

AKAP1	-0,159096477	0,187655779	0	N.A.	0
PMFBP1	-0,159101866	-0,205736979	0	N.A.	0
TUBGCP5	-0,159106028	-0,166227594	0	N.A.	0
PROSER1	-0,159172784	-0,033990961	0	N.A.	0
SCYL3	-0,159263781	0,12086445	0	N.A.	0
MNX1	-0,159380234	-0,221044057	0	N.A.	0
RLBP1	-0,159410606	0,091445652	0	N.A.	0
RP11-108M1	-0,159507292	0,070519134	0	N.A.	0
RP11-85A1.3	-0,159569016	-0,022825176	0	N.A.	0
KIAA0141	-0,159659156	-0,156759187	0	N.A.	0
NANP	-0,159698111	-0,112541043	0	N.A.	0
TFR2	-0,159732436	0,120861773	0	N.A.	0
PARN	-0,159767897	0,062544736	0	N.A.	0
RP11-712L6.5	-0,159806707	-0,019659996	0	N.A.	0
ZBED5-AS1	-0,159917933	-0,290304835	0	N.A.	0
RP11-977G19	-0,159987694	-0,221545561	0	N.A.	0
AKR1E2	-0,160046112	-0,300796705	0	N.A.	0
COMMD1	-0,160066393	-0,005282946	0	N.A.	0
ACOT13	-0,160118882	-0,012541348	0	N.A.	0
RP11-46J23.1	-0,160143404	0,15299895	0	N.A.	0
PKI55	-0,160151686	0,349315519	0	N.A.	0
ZFP37	-0,160167198	-0,349190209	0	N.A.	0
ZFP64	-0,16017253	-0,090260331	0	N.A.	0
ZC2HC1C	-0,160206466	-0,077647582	0	N.A.	0
RP11-334G22	-0,160440378		0	N.A.	0
PSMB10	-0,160493055	-0,215475393	0	N.A.	0
ACADS	-0,160515213	0,107433464	0	N.A.	0
NOL6	-0,160580397	0,015530469	0	N.A.	0
CLRN3	-0,160596687	0,136582046	0	N.A.	0
RP11-126L15	-0,160626345	-0,173459336	0	N.A.	0
TAB1	-0,160783041	-0,06132588	0	N.A.	0
RP4-635E18.	-0,16085916	0,305975354	0	N.A.	0
C10orf35	-0,160873417	0,0744596	0	N.A.	0

FBXL15	-0,161000921	0,051998597	0	N.A.	0
APOA2	-0,161167532		0	N.A.	0
TCP10L	-0,161263176	-0,171763086	0	N.A.	0
COMMD6	-0,161356358	-0,352441488	0	N.A.	0
FOXJ1	-0,161358871	-0,206668942	0	N.A.	0
RP11-629B11	-0,161399425	-0,078181016	0	N.A.	0
AC002550.6	-0,161473407		0	N.A.	0
RAD9B	-0,161474065	-0,148035039	0	N.A.	0
ZNF675	-0,161531116	0,18344006	0	N.A.	0
CAMLG	-0,161551533	-0,271364062	0	N.A.	0
MTHFD2	-0,16157931	-0,314805289	0	N.A.	0
ARMC9	-0,161618893	-0,095747518	0	N.A.	0
CTSV	-0,16167352	0,290233004	0	N.A.	0
FAM3B	-0,161746144	-0,198674055	0	N.A.	0
DGKG	-0,161779456	-0,178083699	0	N.A.	0
SIRT5	-0,161807259	0,005638187	0	N.A.	0
ZNF24	-0,161808259	0,097152205	0	N.A.	0
RP11-196G18	-0,161876123	0,075626721	0	N.A.	0
GALK2	-0,162007264	0,125223643	0	N.A.	0
ART3	-0,162110116	0,025682937	0	N.A.	0
ERMARD	-0,162146791	-0,216118169	0	N.A.	0
LPCAT3	-0,162221943	0,078125682	0	N.A.	0
FPGS	-0,162242824	0,275381849	0	N.A.	0
MT1X	-0,16224831	-0,069330437	0	N.A.	0
L3HYPDH	-0,162367142	-0,092912688	0	N.A.	0
C21orf2	-0,162387088	-0,177291446	0	N.A.	0
KIN	-0,162416118	0,141385851	0	N.A.	0
CAPS2	-0,162450447	-0,038191838	0	N.A.	0
ST6GAL1	-0,162550934	0,071336743	0	N.A.	0
CPT2	-0,162764442	-0,343631397	0	N.A.	0
NPL	-0,162852761	0,045296935	0	N.A.	0
MFAP2	-0,162895651	0,261512846	0	N.A.	0
CXorf40A	-0,162938351	-0,230870592	0	N.A.	0

STARD4-AS1	-0,163046315	-0,117727341	0	N.A.	0
RABL6	-0,163051467	0,1475489	0	N.A.	0
DHX34	-0,163083451	-0,082907017	0	N.A.	0
RP1-309F20.:	-0,163111686	-0,253052051	0	N.A.	0
FTL	-0,163142406	-0,436121532	0	N.A.	0
FANCC	-0,163204868	-0,195081118	0	N.A.	0
CTD-2369P2.	-0,163330684	-0,020127534	0	N.A.	0
HCG20	-0,163353937	0,257466207	0	N.A.	0
RP5-827C21.	-0,163385382	-0,021142174	0	N.A.	0
ALKBH6	-0,163438803	-0,094071871	0	N.A.	0
FAM118B	-0,163449055	-0,162603143	0	N.A.	0
ARIH2	-0,163520641	-0,100858657	0	N.A.	0
RP5-1092A3.	-0,163522588	0,043490561	0	N.A.	0
ANKRD16	-0,163643244	0,17610524	0	N.A.	0
NBEAL1	-0,163650367	-0,481196969	0	N.A.	0
SRPX	-0,163810277	-0,016939361	0	N.A.	0
PRKRIR	-0,163989624	-0,127368191	0	N.A.	0
SRPK2	-0,164116955	-0,096630927	0	N.A.	0
DHCR7	-0,164218487	0,179671493	0	N.A.	0
RAMP2	-0,164242577	-0,190722494	0	N.A.	0
CDKAL1	-0,164369382	-0,156172744	0	N.A.	0
C10orf131	-0,164455406		0	N.A.	0
AC021224.1	-0,164480433	-0,046556194	0	N.A.	0
CLPTM1	-0,164493208	0,370967654	0	N.A.	0
ZP3	-0,164503226	-0,232392597	0	N.A.	0
C3orf14	-0,1645082	-0,468160382	0	N.A.	0
C2CD5	-0,164621766	-0,196743453	0	N.A.	0
KTN1-AS1	-0,164642819	-0,291001964	0	N.A.	0
DBR1	-0,164685926	-0,221655549	0	N.A.	0
HEMK1	-0,164689643	-0,097320126	0	N.A.	0
PTGR2	-0,164734475	-0,051551543	0	N.A.	0
RP11-410L14	-0,164883952	-0,294565276	0	N.A.	0
EPB41L2	-0,164900555	0,2187165	0	N.A.	0

ZCCHC4	-0,164931704	0,080005157	0	N.A.	0
DNAH11	-0,164939594	-0,254885052	0	N.A.	0
RXFP4	-0,164977464	-0,116084944	0	N.A.	0
RBM26	-0,164978022	0,289963465	0	N.A.	0
NCKAP1L	-0,164987392	-0,227373497	0	N.A.	0
FIBCD1	-0,165000125	-0,040591595	0	N.A.	0
NEK1	-0,16504524	-0,384606959	0	N.A.	0
ZNF81	-0,165106829	0,092577293	0	N.A.	0
RP11-57A19.	-0,165112363		0	N.A.	0
SMARCD1	-0,165140769	-0,165312	0	N.A.	0
ASTN2	-0,165213096	-0,249459477	0	N.A.	0
HIF1AN	-0,165219974	-0,062114771	0	N.A.	0
VSTM2B	-0,165220554	-0,234592937	0	N.A.	0
DKFZp434P2	-0,165247364	-0,111262731	0	N.A.	0
TTI2	-0,165350718	-0,254565078	0	N.A.	0
ELAVL2	-0,165371572	-0,032119701	0	N.A.	0
CYP2J2	-0,165377077	0,114717603	0	N.A.	0
CANX	-0,165419933	0,069288956	0	N.A.	0
ITPRIPL1	-0,165466784		0	N.A.	0
FAM175A	-0,165665091	-0,304761312	0	N.A.	0
AL450992.2	-0,165690745		0	N.A.	0
LINC00115	-0,16573394	0,009126935	0	N.A.	0
APOH	-0,165770895	-0,168270417	0	N.A.	0
RBP4	-0,165779207	0,110134487	0	N.A.	0
DLL1	-0,165791851	0,072100903	0	N.A.	0
FAM117B	-0,165855818	-0,247902789	0	N.A.	0
CUL2	-0,165980774	0,142533534	0	N.A.	0
EGFL6	-0,166003421		0	N.A.	0
CCDC127	-0,16614903	0,200873204	0	N.A.	0
CXCL12	-0,166213568	-0,145520751	0	N.A.	0
CCAR2	-0,166262034	-0,270866848	0	N.A.	0
RP1-223E5.4	-0,166286441	-0,057118166	0	N.A.	0
CD3E	-0,166313101	-0,252677645	0	N.A.	0

PDGFRA	-0,166314231	0,260807931	0	N.A.	0
USP7	-0,166405114	0,147132485	0	N.A.	0
SLC13A3	-0,166449256	-0,150716598	0	N.A.	0
RP11-277B15	-0,166450654	0,193007467	0	N.A.	0
CPA6	-0,166608197	-0,348071273	0	N.A.	0
TMEM107	-0,166770759	-0,184364848	0	N.A.	0
MSANTD4	-0,166801278	0,004092459	0	N.A.	0
TET1	-0,1669617	-0,261673463	0	N.A.	0
PLCB4	-0,166964035	-0,096281468	0	N.A.	0
ZNF830	-0,167041024	0,062691792	0	N.A.	0
CCDC14	-0,167106701	0,155474549	0	N.A.	0
EPN3	-0,167107235	0,193736829	0	N.A.	0
RP6-109B7.5	-0,167190558	-0,256575846	0	N.A.	0
RP5-1074L1.1	-0,167236185	-0,13281901	0	N.A.	0
RGS10	-0,167258876	-0,414201044	0	N.A.	0
PKM	-0,167281583	0,281063891	0	N.A.	0
AHI1	-0,167440576	0,118614132	0	N.A.	0
RANBP3	-0,167476134	-0,172844924	0	N.A.	0
TAZ	-0,167484295	0,218449597	0	N.A.	0
CCDC15	-0,167496444	-0,02229236	0	N.A.	0
FBXW2	-0,167506415	0,187609712	0	N.A.	0
STX18	-0,167536053	0,226426759	0	N.A.	0
NOTCH1	-0,167537271	-0,095955594	0	N.A.	0
TDO2	-0,167559308	-0,214650853	0	N.A.	0
ATRX	-0,167563103	0,214164149	0	N.A.	0
LINC00094	-0,167577603	-0,325401748	0	N.A.	0
GPC3	-0,16767624	-0,12670222	0	N.A.	0
N6AMT1	-0,16767837	-0,205737745	0	N.A.	0
ALKBH3	-0,167740475	-0,211449156	0	N.A.	0
RBM22	-0,167829429	-0,02797178	0	N.A.	0
PDE3A	-0,167870497	-0,039803172	0	N.A.	0
TRNAU1AP	-0,167918339	0,253210509	0	N.A.	0
RP11-498C9.	-0,168005066		0	N.A.	0

SPIDR	-0,168032506	0,066253722	0	N.A.	0
MORN1	-0,168088001	-0,207530037	0	N.A.	0
TTPA	-0,168147835	-0,279907446	0	N.A.	0
CNDP2	-0,168204089	-0,147549147	0	N.A.	0
TERF2IP	-0,168255327	0,107601866	0	N.A.	0
KHDC1	-0,168305343	-0,145402254	0	N.A.	0
ZNF90	-0,168385804	-0,361244699	0	N.A.	0
RP11-245J9.5	-0,168438475		0	N.A.	0
AC104654.2	-0,168496537		0	N.A.	0
FAM172A	-0,168539363	-0,067540045	0	N.A.	0
SPATA17	-0,168574885	-0,216543549	0	N.A.	0
FGD1	-0,168577852		0	N.A.	0
TOE1	-0,168596751	0,085994881	0	N.A.	0
COX10	-0,168683675	-0,346631706	0	N.A.	0
GTF2E2	-0,168703124	-0,191476221	0	N.A.	0
FAM35A	-0,168784151	-0,336853146	0	N.A.	0
ZSCAN21	-0,168834828	0,092760389	0	N.A.	0
LINC00176	-0,168836588	0,00394554	0	N.A.	0
RBM4B	-0,168875775	-0,23493069	0	N.A.	0
HSD17B11	-0,168887686	0,103285773	0	N.A.	0
RP4-680D5.8	-0,168945649	-0,329601956	0	N.A.	0
NUAK2	-0,168961355	-0,099860705	0	N.A.	0
ECM2	-0,169049674	-0,303702421	0	N.A.	0
RP11-498C9.	-0,169084215	-0,26392906	0	N.A.	0
RP11-67M9.1	-0,169102479	0,070361615	0	N.A.	0
RP11-68L18.	-0,169254883		0	N.A.	0
WIBG	-0,169517518	0,208929507	0	N.A.	0
MIP	-0,169543519	0,310619148	0	N.A.	0
LNP1	-0,169597083	0,018716566	0	N.A.	0
PCGF1	-0,169701182	-0,070540012	0	N.A.	0
RP11-559M2	-0,169705089	0,076755745	0	N.A.	0
KLHL8	-0,169708672	-0,340595865	0	N.A.	0
GPR155	-0,169761159	0,052816684	0	N.A.	0

IGSF23	-0,169775723	0,133332274	0	N.A.	0
RP11-251G23	-0,169786357	-0,227374614	0	N.A.	0
RP11-1069G1	-0,170016496		0	N.A.	0
NAA40	-0,170027989	0,185830276	0	N.A.	0
LINC00402	-0,170097707		0	N.A.	0
RNF212	-0,170102624	0,104291894	0	N.A.	0
TUSC2	-0,170179974	-0,29551344	0	N.A.	0
HOXB4	-0,170238452	0,302804466	0	N.A.	0
FAM200A	-0,170282769	-0,041228068	0	N.A.	0
ZNF227	-0,170319089	-0,226444772	0	N.A.	0
CAMKK1	-0,170368352	0,16455218	0	N.A.	0
WIF1	-0,17044255		0	N.A.	0
RP1-168L15.5	-0,170694499	-0,02983824	0	N.A.	0
RP11-182L21	-0,170775974	-0,101788994	0	N.A.	0
TRAF5	-0,170794123	-0,004374033	0	N.A.	0
CIRBP	-0,170939467	0,119038253	0	N.A.	0
ISCA2	-0,170968096	0,019498054	0	N.A.	0
TCTN2	-0,171029801	0,085109125	0	N.A.	0
PRAME	-0,171073772	-0,152261826	0	N.A.	0
ADCK2	-0,17110539	-0,000366649	0	N.A.	0
AP001505.10	-0,1711192	-0,124547571	0	N.A.	0
C3orf33	-0,171194395	-0,095712358	0	N.A.	0
LINC01234	-0,171261064	-0,190150932	0	N.A.	0
ZNF512B	-0,171307809	0,000427825	0	N.A.	0
RASL10B	-0,17133499		0	N.A.	0
SLC30A9	-0,171340839	-0,20876916	0	N.A.	0
SLC4A1AP	-0,171372325	-0,004907781	0	N.A.	0
AP000692.10	-0,171508802	0,329611396	0	N.A.	0
ANGPTL2	-0,171510863	-0,201476516	0	N.A.	0
SEC14L4	-0,171550735		0	N.A.	0
TMEM71	-0,17156215	-0,072898669	0	N.A.	0
WT1	-0,171698432	-0,314246103	0	N.A.	0
FAM41C	-0,171720978	0,145183678	0	N.A.	0

CLDN15	-0,171818825	-0,052353323	0	N.A.	0
RNF43	-0,171833911	0,082788978	0	N.A.	0
INSM1	-0,171939692		0	N.A.	0
TTC21B	-0,172173062	-0,045053964	0	N.A.	0
ATP6AP2	-0,17225939	-0,082931665	0	N.A.	0
PLCB1	-0,1722696	-0,003099779	0	N.A.	0
ARHGEF19	-0,172275944	0,060101798	0	N.A.	0
LXN	-0,172396928	-0,010207922	0	N.A.	0
SOX14	-0,172575418	0,105004034	0	N.A.	0
ORM1	-0,172652479	-0,055316807	0	N.A.	0
AC018359.1	-0,172661219	-0,211610483	0	N.A.	0
ZNF286A	-0,172699449	-0,121439581	0	N.A.	0
KMT2E-AS1	-0,172752304	0,087619722	0	N.A.	0
SPIN3	-0,172773223	-0,112211294	0	N.A.	0
GIN1	-0,172792458	-0,115161184	0	N.A.	0
FSD1L	-0,172899512	-0,310359887	0	N.A.	0
PRCP	-0,172999141	-0,207187229	0	N.A.	0
OXLD1	-0,173045333	-0,086282178	0	N.A.	0
LANCL2	-0,173151232	-0,338838148	0	N.A.	0
PACRGL	-0,173369704	-0,320648207	0	N.A.	0
RP11-863K10	-0,17344652		0	N.A.	0
THAP1	-0,173499901	0,051458355	0	N.A.	0
BTG3	-0,173619285	-0,125839908	0	N.A.	0
DDX28	-0,173661556	-0,160222538	0	N.A.	0
BAP1	-0,173664491	0,075141534	0	N.A.	0
ZC3H14	-0,173678229	-0,286186101	0	N.A.	0
DIXDC1	-0,173734718	0,081159554	0	N.A.	0
ZNF138	-0,17374495	-0,162190905	0	N.A.	0
SELO	-0,173822953	0,068979396	0	N.A.	0
FASN	-0,17395102	0,234612821	0	N.A.	0
CTD-2024I7.1	-0,174023097		0	N.A.	0
NCLN	-0,174107067	0,20255686	0	N.A.	0
TUBA3E	-0,174175897		0	N.A.	0

SEC61B	-0,174180724	-0,206159724	0	N.A.	0
PRR7-AS1	-0,174202124	-0,227703498	0	N.A.	0
EXOSC10	-0,174289049	-0,133198214	0	N.A.	0
CDK5RAP3	-0,174371939	0,2704897	0	N.A.	0
MIEN1	-0,174416005	-0,197994378	0	N.A.	0
RP13-753N3.	-0,174437358	-0,178861536	0	N.A.	0
MYLK-AS1	-0,174579129	-0,287806399	0	N.A.	0
RP11-91G21.	-0,174735811	-0,087497985	0	N.A.	0
CTC-232P5.1	-0,17475107		0	N.A.	0
RP11-758H9.	-0,174763762	-0,068044115	0	N.A.	0
UBE2Q2	-0,174805209	-0,165687739	0	N.A.	0
MSRB2	-0,17481591	0,156900461	0	N.A.	0
LINC01521	-0,174850456	-0,020105997	0	N.A.	0
ASIC1	-0,174982623	-0,09086839	0	N.A.	0
RNF219	-0,175100782	0,005775544	0	N.A.	0
CCDC77	-0,175138173	-0,044507868	0	N.A.	0
AACS	-0,17523426	-0,147532684	0	N.A.	0
CMTM6	-0,175246348	0,06721708	0	N.A.	0
EPT1	-0,175415848	-0,040511844	0	N.A.	0
NADK2	-0,175493082	0,046104497	0	N.A.	0
PLN	-0,175681738	-0,039887399	0	N.A.	0
TEX9	-0,175871786	-0,238380316	0	N.A.	0
ACTR8	-0,175889266	0,114007696	0	N.A.	0
RNF135	-0,175959667	-0,108903044	0	N.A.	0
TMEM201	-0,176048813	-0,088696233	0	N.A.	0
GTF2H1	-0,176049319	-0,195676547	0	N.A.	0
RP11-1079K1	-0,176142336	-0,025954211	0	N.A.	0
TTC3	-0,17629085	-0,009932801	0	N.A.	0
ACLY	-0,176339072	-0,079326815	0	N.A.	0
FAM184A	-0,176402834		0	N.A.	0
CSNK2A1	-0,176523685	0,021220872	0	N.A.	0
RP11-197K6.	-0,176525209	-0,343782048	0	N.A.	0
TRRAP	-0,176539597	0,1112403	0	N.A.	0

C2orf43	-0,176540434	-0,265083017	0	N.A.	0
LTK	-0,176562534	0,097106267	0	N.A.	0
PARD6A	-0,176567488	-0,289642279	0	N.A.	0
NUDT7	-0,176630833	-0,368882655	0	N.A.	0
AC024592.12	-0,176659978	-0,033478156	0	N.A.	0
AMT	-0,176744573	-0,020413617	0	N.A.	0
CWC22	-0,176754581	0,10040566	0	N.A.	0
SOWAHA	-0,176757479	0,140447372	0	N.A.	0
RBKS	-0,176792127	-0,234309526	0	N.A.	0
ZNF692	-0,176840839	-0,024812754	0	N.A.	0
DGUOK-AS1	-0,177077664	-0,29487939	0	N.A.	0
GCG	-0,177078628	0,103100628	0	N.A.	0
DSTN	-0,17712704	-0,131787524	0	N.A.	0
CRBN	-0,177191576	-0,11313233	0	N.A.	0
AMD1	-0,177199897	0,157160641	0	N.A.	0
SMAD9	-0,177207729	-0,146001764	0	N.A.	0
TBCC	-0,177220628	0,009258682	0	N.A.	0
POLR3C	-0,177231928	0,070151967	0	N.A.	0
RP11-177H2.	-0,177404694	0,347944736	0	N.A.	0
TMEM251	-0,177478888	-0,250787011	0	N.A.	0
RAB3A	-0,177537582	0,106303003	0	N.A.	0
SRI	-0,177553752	-0,129624567	0	N.A.	0
CYB5RL	-0,177672615	-0,130202069	0	N.A.	0
WDR5B	-0,17777885	-0,161096024	0	N.A.	0
PRIMPOL	-0,177844745	-0,179057837	0	N.A.	0
SEPT6	-0,177856257	-0,249837917	0	N.A.	0
RP11-477D19	-0,178064638	-0,007295929	0	N.A.	0
ZNF195	-0,178099905	0,023251689	0	N.A.	0
RP11-218F10	-0,178196804	-0,0171944	0	N.A.	0
NAP1L5	-0,178203342	-0,21827989	0	N.A.	0
PGM5	-0,178267394		0	N.A.	0
RAB30-AS1	-0,178277078	-0,242831747	0	N.A.	0
RP11-634H22	-0,178286511	0,188416378	0	N.A.	0

FCHO1	-0,178325748	0,016768041	0	N.A.	0
AARS2	-0,178441932	0,186721817	0	N.A.	0
SAMD1	-0,178465152	0,146870474	0	N.A.	0
RP11-165A2(	-0,178475166	-0,012176048	0	N.A.	0
SOBP	-0,178531523	-0,260271253	0	N.A.	0
HILPDA	-0,178695937	-0,071592884	0	N.A.	0
NSFL1C	-0,178727688	0,025550588	0	N.A.	0
CEP192	-0,17880771	0,036109461	0	N.A.	0
MBNL3	-0,178879442	-0,031819303	0	N.A.	0
SMIM15	-0,178903773	-0,124630006	0	N.A.	0
C15orf57	-0,178923654	-0,032508544	0	N.A.	0
C1orf194	-0,178946534	-0,139393162	0	N.A.	0
RP11-146F11	-0,178999194	-0,173943794	0	N.A.	0
NODAL	-0,179006781		0	N.A.	0
ADPRHL2	-0,179032058	0,034093929	0	N.A.	0
L3MBTL2	-0,179086302	-0,085748426	0	N.A.	0
AASDH	-0,179125129	-0,049613999	0	N.A.	0
OXSM	-0,17916735	-0,253053246	0	N.A.	0
CENPL	-0,179186857	-0,363022161	0	N.A.	0
ARHGEF38	-0,179212263	0,018450187	0	N.A.	0
GNAZ	-0,179352105	-0,028197087	0	N.A.	0
CCDC22	-0,179368191	-0,256562388	0	N.A.	0
COMMD2	-0,179422283	-0,251493133	0	N.A.	0
RP11-254F7.4	-0,17950312	-0,127817188	0	N.A.	0
ORAOV1	-0,179533694	-0,052269122	0	N.A.	0
BDP1	-0,179577926	0,081680331	0	N.A.	0
SYBU	-0,179579593	-0,305875388	0	N.A.	0
RTKN2	-0,179631965	-0,004179514	0	N.A.	0
DCLRE1A	-0,179694488	-0,236893404	0	N.A.	0
ZNF816	-0,179704208	-0,069206036	0	N.A.	0
DBH-AS1	-0,179801577		0	N.A.	0
ZNF576	-0,179943677	-0,017146235	0	N.A.	0
CTD-2015H6.	-0,179945591	-0,213155821	0	N.A.	0

OPA1	-0,179993531	0,16314904	0	N.A.	0
RP3-431P23.	-0,180000524	-0,033130301	0	N.A.	0
AKAP4	-0,18005936	-0,050511744	0	N.A.	0
NGFRAP1	-0,180071777	-0,408968625	0	N.A.	0
RECQL	-0,180098001	-0,192974515	0	N.A.	0
MTR	-0,180214079	0,078909642	0	N.A.	0
EGR4	-0,180237543	-0,004588041	0	N.A.	0
FAM166B	-0,180245824	-0,03613772	0	N.A.	0
TRIM37	-0,180282631	-0,0166567	0	N.A.	0
LMAN2	-0,180335564	0,150421205	0	N.A.	0
DDX23	-0,180427999	-0,057732068	0	N.A.	0
LRRC6	-0,180467986	-0,017869738	0	N.A.	0
TMED3	-0,180540374	0,061151885	0	N.A.	0
FECH	-0,180573007	-0,177541774	0	N.A.	0
TMSB15A	-0,180612364		0	N.A.	0
PFKL	-0,180716633	0,117454316	0	N.A.	0
USP46	-0,180755552	-0,02235429	0	N.A.	0
SUDS3	-0,180786379	0,227589598	0	N.A.	0
SPERT	-0,181059052	-0,06775217	0	N.A.	0
FAM110D	-0,181095939		0	N.A.	0
B3GAT3	-0,181179766	0,168984505	0	N.A.	0
AC007099.1	-0,181211623	0,005036517	0	N.A.	0
SLX4IP	-0,181362935	-0,080896105	0	N.A.	0
LINC01006	-0,181408026	-0,187427329	0	N.A.	0
ACVR1C	-0,181425021	-0,204801803	0	N.A.	0
PCP4	-0,18144509	-0,310842525	0	N.A.	0
FAM173A	-0,181468788	0,140188763	0	N.A.	0
DERL3	-0,181480829	-0,114179613	0	N.A.	0
CNBD2	-0,1815428	-0,025190315	0	N.A.	0
RP11-322E11	-0,181759484	0,075694812	0	N.A.	0
LINC01560	-0,181882355	0,223848769	0	N.A.	0
PKLR	-0,181958385		0	N.A.	0
ZNRD1	-0,182054702	-0,103988862	0	N.A.	0

PSPH	-0,182060877	-0,419854597	0	N.A.	0
TARBP1	-0,182193825	-0,017030557	0	N.A.	0
LCN15	-0,18236251	-0,166494353	0	N.A.	0
NID1	-0,18239984	-0,126594329	0	N.A.	0
CRIPT	-0,182418048	-0,250247534	0	N.A.	0
GTF2H2	-0,182563054	-0,239117124	0	N.A.	0
YRDC	-0,182643603	0,162484007	0	N.A.	0
ZNF485	-0,182718448	-0,122969486	0	N.A.	0
RP11-602N24	-0,182845097	-0,233870089	0	N.A.	0
ZNF550	-0,182967495	0,074774034	0	N.A.	0
ERVMER34-1	-0,18299991	-0,0615689	0	N.A.	0
CYFIP2	-0,183087227	-0,052246263	0	N.A.	0
NES	-0,183136071	0,058875824	0	N.A.	0
MEIOB	-0,183141817	-0,235849003	0	N.A.	0
FAM171A1	-0,183182534	-0,103415122	0	N.A.	0
CYP4X1	-0,183330939	-0,173826072	0	N.A.	0
CTD-2555O16	-0,183401748	0,073660981	0	N.A.	0
FBXL6	-0,183497901	0,18641871	0	N.A.	0
RP1-40E16.1	-0,183504253	-0,070101534	0	N.A.	0
TTC5	-0,183548394	-0,0727722	0	N.A.	0
NREP	-0,183796242	-0,308096492	0	N.A.	0
CA11	-0,183852853	-0,017825211	0	N.A.	0
CTD-2227E11	-0,18399778	0,025205877	0	N.A.	0
RP11-401F2.3	-0,183998662	-0,089765176	0	N.A.	0
YBX3	-0,184074128	-0,025807553	0	N.A.	0
DCP1B	-0,184083978	-0,057435739	0	N.A.	0
PLCL1	-0,184166171	-0,288731556	0	N.A.	0
YTHDF2	-0,184171197	-0,009895337	0	N.A.	0
REEP5	-0,184184491	0,460405387	0	N.A.	0
RP5-881L22.5	-0,184361335	-0,277031702	0	N.A.	0
RP11-110G21	-0,184376389	-0,198810084	0	N.A.	0
COG4	-0,184410562	-0,093346476	0	N.A.	0
CKAP4	-0,184681801	-0,009282683	0	N.A.	0

SLC39A2	-0,18468393		0	N.A.	0
ASB4	-0,184695072	0,216294223	0	N.A.	0
ZNF76	-0,184729572	-0,237167442	0	N.A.	0
RUSC1	-0,184734018	0,168210871	0	N.A.	0
DNAJC3-AS1	-0,184786434	0,105210951	0	N.A.	0
RP11-141J13	-0,184865168	-0,298882191	0	N.A.	0
CLPSL2	-0,185091504	-0,137805119	0	N.A.	0
RP11-10A14.	-0,185149827	-0,016202958	0	N.A.	0
CD44	-0,18524605	-0,321441415	0	N.A.	0
CTPS2	-0,185452808	-0,147992431	0	N.A.	0
HSCB	-0,185475666	-0,282794753	0	N.A.	0
GGA2	-0,185661163	-0,226868995	0	N.A.	0
ST3GAL3	-0,18567805	0,048056706	0	N.A.	0
JMJD4	-0,185700469	0,014691378	0	N.A.	0
HAUS4	-0,185786798	-0,367998943	0	N.A.	0
FBP1	-0,185802611	-0,090541081	0	N.A.	0
RHBG	-0,185959707	0,006800455	0	N.A.	0
PRKCSH	-0,186016721	0,241409565	0	N.A.	0
DAPL1	-0,186091673	-0,155132335	0	N.A.	0
PET100	-0,186137014	-0,219377534	0	N.A.	0
RANGAP1	-0,186149046	-0,266476732	0	N.A.	0
SNX6	-0,18632647	-0,318976142	0	N.A.	0
DNMT3B	-0,186450451	-0,102833117	0	N.A.	0
LA16c-352F7	-0,186535918		0	N.A.	0
GPATCH2	-0,186617015	0,218346752	0	N.A.	0
SELT	-0,186656437	0,162536357	0	N.A.	0
CASP2	-0,186657392	-0,153139374	0	N.A.	0
CEP57L1	-0,186787981	-0,306485012	0	N.A.	0
MCOLN2	-0,186830218	-0,122334029	0	N.A.	0
FKBP7	-0,186888065	0,045022913	0	N.A.	0
CD99	-0,186901768	0,074155455	0	N.A.	0
PEX10	-0,186904987	-0,030612617	0	N.A.	0
RP11-644N4.	-0,186922807	0,042750086	0	N.A.	0

PPP1R8	-0,186951837	-0,319148696	0	N.A.	0
CD200	-0,186971267	-0,36906331	0	N.A.	0
RAD51B	-0,187050673	-0,210865181	0	N.A.	0
MAP7D3	-0,18707892	-0,121277983	0	N.A.	0
DNAH2	-0,187122528	0,12574651	0	N.A.	0
AIG1	-0,187191833	-0,027616431	0	N.A.	0
C11orf49	-0,187226569	-0,100344748	0	N.A.	0
PFDN1	-0,187357299	0,221805375	0	N.A.	0
GALNT11	-0,187391492	0,064913313	0	N.A.	0
SMIM11	-0,187448128	0,035973061	0	N.A.	0
SAMD13	-0,187470018	-0,232113249	0	N.A.	0
FAM173B	-0,187476986	-0,104043236	0	N.A.	0
THOP1	-0,187584868	0,227491534	0	N.A.	0
WDR35	-0,187705603	-0,084623463	0	N.A.	0
AFMID	-0,187839808	0,036715129	0	N.A.	0
CASP3	-0,187897557	0,187553239	0	N.A.	0
XXbac-BPG32	-0,187983506	-0,167490089	0	N.A.	0
DDX11-AS1	-0,188067293	-0,376152085	0	N.A.	0
UFSP2	-0,188077129	-0,245014518	0	N.A.	0
HMGXB4	-0,188138696	0,014857682	0	N.A.	0
IGF2BP1	-0,188379307		0	N.A.	0
AC006994.2	-0,188380243	0,104816432	0	N.A.	0
FXR1	-0,188498375	-0,144636799	0	N.A.	0
SEPSECS-AS1	-0,18850505	-0,303899811	0	N.A.	0
PAXIP1	-0,188543478	-0,081017953	0	N.A.	0
MAP3K4	-0,18857445	0,082534789	0	N.A.	0
RBM19	-0,188724676	-0,145042528	0	N.A.	0
TOP2B	-0,188776104	-0,027559631	0	N.A.	0
MTF2	-0,188863167	-0,155379975	0	N.A.	0
SPIN2B	-0,188874531	0,05720167	0	N.A.	0
SOX1	-0,188941056	0,02055077	0	N.A.	0
OMA1	-0,188970334	-0,18798335	0	N.A.	0
ZNF2	-0,188975942	-0,095077584	0	N.A.	0

RP1-8B1.4	-0,188984613	-0,273897335	0	N.A.	0
CCDC57	-0,188996325	-0,27589991	0	N.A.	0
CYP4B1	-0,189089888	0,350333322	0	N.A.	0
RP11-697E2.	-0,189219976	0,004213313	0	N.A.	0
NEMF	-0,18923393	0,155191934	0	N.A.	0
RPAP1	-0,189260384	0,023288144	0	N.A.	0
RP5-832C2.5	-0,189275392	0,039732787	0	N.A.	0
RP11-190C22	-0,189277361	0,207351522	0	N.A.	0
ESPL1	-0,189299275	-0,203264621	0	N.A.	0
RP11-285A1.	-0,189319568		0	N.A.	0
DSC3	-0,189351722	0,222355715	0	N.A.	0
ECI1	-0,189353689	0,189032199	0	N.A.	0
RP11-152N13	-0,189386687	-0,203066472	0	N.A.	0
RP11-402J6.1	-0,189391838	-0,246427291	0	N.A.	0
CES1	-0,189489576	-0,06863756	0	N.A.	0
WDYHV1	-0,189501761	-0,247990056	0	N.A.	0
SHPRH	-0,189953815	0,056433414	0	N.A.	0
LRRC20	-0,190075256	-0,135143163	0	N.A.	0
DDRGK1	-0,190141174	0,180314007	0	N.A.	0
PPWD1	-0,19016021	-0,158672152	0	N.A.	0
SDF2L1	-0,190231349	-0,272512129	0	N.A.	0
MARS2	-0,190343303	-0,192369036	0	N.A.	0
DLX6-AS1	-0,190350502	-0,222594664	0	N.A.	0
RP11-44F21.	-0,190351572	-0,144407674	0	N.A.	0
ZC3H18	-0,190535798	0,065419894	0	N.A.	0
B3GNTL1	-0,190611288	-0,17301083	0	N.A.	0
BDH2	-0,190678806	-0,334358338	0	N.A.	0
RNF168	-0,190753324	0,048143351	0	N.A.	0
RP11-666O2.	-0,190928961	-0,217823306	0	N.A.	0
CEP131	-0,190938116	-0,173896792	0	N.A.	0
CLUH	-0,190952083	0,09178946	0	N.A.	0
B4GAT1	-0,191249917	0,24299602	0	N.A.	0
MSTO1	-0,191254791	-0,155901052	0	N.A.	0

VARS2	-0,191337948	-0,18035798	0	N.A.	0
LRPAP1	-0,191426197	0,098727046	0	N.A.	0
PLTP	-0,191510749	-0,057078584	0	N.A.	0
TEX40	-0,191521038	-0,249040396	0	N.A.	0
UNC45A	-0,191603699	0,003806611	0	N.A.	0
TIMM10B	-0,191656984	-0,271130549	0	N.A.	0
ORC4	-0,191721454	-0,006788373	0	N.A.	0
KRIT1	-0,191768637	-0,022357781	0	N.A.	0
RP11-42O4.2	-0,191781774	0,085273201	0	N.A.	0
ZPR1	-0,191785104	-0,25745012	0	N.A.	0
AF064858.8	-0,191797905	-0,067340679	0	N.A.	0
P2RY8	-0,19181173	-0,088758096	0	N.A.	0
TXNDC16	-0,191828019	0,019092986	0	N.A.	0
ERCC2	-0,191862721	-0,090492242	0	N.A.	0
GZMB	-0,191867157	-0,373351738	0	N.A.	0
C2orf49	-0,191907108	0,122035614	0	N.A.	0
RP11-352M1	-0,19190885	-0,109510683	0	N.A.	0
ASH2L	-0,192010602	-0,207827244	0	N.A.	0
DUS4L	-0,192238314	-0,220971372	0	N.A.	0
PRR3	-0,192247451	-0,191986394	0	N.A.	0
MVB12A	-0,192266699	-0,272276792	0	N.A.	0
TIFA	-0,192272938	-0,404274265	0	N.A.	0
TNKS2-AS1	-0,192358846	-0,093358627	0	N.A.	0
TDRKH	-0,192435602	-0,043311381	0	N.A.	0
CEACAM21	-0,192454927		0	N.A.	0
RP11-396C23	-0,192463474	-0,12767819	0	N.A.	0
GADD45G	-0,192549419	-0,187284368	0	N.A.	0
NFE2	-0,192615108	-0,026320883	0	N.A.	0
HYKK	-0,192616424	-0,429532279	0	N.A.	0
MICU2	-0,19271376	-0,150238313	0	N.A.	0
NPEPL1	-0,193185439	0,124096631	0	N.A.	0
PRMT6	-0,193191792	-0,390751512	0	N.A.	0
GPD1L	-0,193196782	-0,344438255	0	N.A.	0

PROCR	-0,193259628	-0,24268127	0	N.A.	0
PLCH1	-0,193278766	-0,062755822	0	N.A.	0
CTNNBIP1	-0,193281052	0,327262624	0	N.A.	0
SWSAP1	-0,193368147	0,012517988	0	N.A.	0
BCDIN3D	-0,193376503	-0,15057182	0	N.A.	0
RNF138	-0,193463773	-0,37521444	0	N.A.	0
THAP7	-0,193559119	-0,266618147	0	N.A.	0
SPRYD4	-0,193572151	-0,211047226	0	N.A.	0
PASK	-0,193579366	-0,179810973	0	N.A.	0
E2F7	-0,193609192	-0,324494213	0	N.A.	0
COG8	-0,193747026	-0,199126284	0	N.A.	0
C20orf196	-0,193773698	-0,339231412	0	N.A.	0
VGF	-0,193890839	-0,221285225	0	N.A.	0
IPO11	-0,194024035	-0,425067036	0	N.A.	0
HHEX	-0,194063542	-0,263719565	0	N.A.	0
RP11-511B23	-0,194241015	0,068107124	0	N.A.	0
FKBPL	-0,194354404	-0,123551436	0	N.A.	0
C7orf49	-0,194410634	-0,019301651	0	N.A.	0
SLC25A22	-0,194550023	-0,195172142	0	N.A.	0
LRRC74A	-0,19464743		0	N.A.	0
ARID5B	-0,194661185	-0,107650326	0	N.A.	0
LUC7L	-0,194761228	0,132978745	0	N.A.	0
REG1A	-0,194810018	-0,125996421	0	N.A.	0
PHTF2	-0,194862029	0,056978373	0	N.A.	0
BRMS1L	-0,194882026	-0,230556102	0	N.A.	0
ATP5L2	-0,195003928	-0,332518464	0	N.A.	0
UBE3D	-0,1950205	-0,247976295	0	N.A.	0
ACTR1B	-0,195053116	0,18402398	0	N.A.	0
THTPA	-0,195098934	-0,367761142	0	N.A.	0
FBXO16	-0,195307612	-0,257191722	0	N.A.	0
FRAT2	-0,195443133	-0,125894976	0	N.A.	0
GALNT14	-0,195521083		0	N.A.	0
EHF	-0,195538187	0,172720665	0	N.A.	0

EDEM2	-0,195572936	0,168369493	0	N.A.	0
LRTOMT	-0,195649052	-0,12434242	0	N.A.	0
KTI12	-0,195703133	-0,201780389	0	N.A.	0
DCAF7	-0,195762256	0,149541879	0	N.A.	0
NNT	-0,195843771	0,062312265	0	N.A.	0
PHOSPHO2	-0,196011533	-0,157894893	0	N.A.	0
RWDD2B	-0,196107828	-0,212436354	0	N.A.	0
RMND1	-0,196124448	-0,410169232	0	N.A.	0
C19orf45	-0,196158227	-0,274171647	0	N.A.	0
TRIM32	-0,196296086	-0,103034574	0	N.A.	0
LINC00441	-0,1963604	-0,16651788	0	N.A.	0
PRPF40B	-0,19636244	0,096651345	0	N.A.	0
LACTB2	-0,196372599	0,041945997	0	N.A.	0
SUGP1	-0,196386329	-0,162736555	0	N.A.	0
TTF1	-0,196528596	-0,12739841	0	N.A.	0
CWF19L1	-0,196594085	0,013819505	0	N.A.	0
GMPR	-0,196623755	-0,21628111	0	N.A.	0
DDX51	-0,196631028	0,0057349	0	N.A.	0
HOGA1	-0,196755731	0,163493037	0	N.A.	0
PRSS3P2	-0,19678304	0,166285241	0	N.A.	0
CERKL	-0,196993816	-0,103781465	0	N.A.	0
ADCK1	-0,197059526	-0,096857606	0	N.A.	0
SH3BGRL	-0,197133106	-0,591848227	0	N.A.	0
KIAA1614-AS	-0,197180309	-0,199291127	0	N.A.	0
PDSS2	-0,197228972	-0,374494287	0	N.A.	0
HSD17B6	-0,197243502	-0,302632711	0	N.A.	0
INTS4	-0,197260115	-0,331468601	0	N.A.	0
HAS2-AS1	-0,197427122	-0,369286547	0	N.A.	0
SLC2A4RG	-0,197453031	0,024834825	0	N.A.	0
CISH	-0,19745439	-0,375573918	0	N.A.	0
ZNF620	-0,19747414	-0,353046675	0	N.A.	0
GORASP2	-0,197665193	0,307374778	0	N.A.	0
SNAPC1	-0,197675448	-0,284657975	0	N.A.	0

UBFD1	-0,19771222	-0,037318675	0	N.A.	0
SNRNP200	-0,197762142	0,174958107	0	N.A.	0
ERP27	-0,19777743	-0,186172273	0	N.A.	0
RP11-267M2	-0,197859802	0,083241293	0	N.A.	0
TMEM242	-0,197903666	0,089876913	0	N.A.	0
SENP8	-0,19790404	-0,076753634	0	N.A.	0
SLMO1	-0,19790684	-0,002091146	0	N.A.	0
METTL18	-0,198044727	-0,230711795	0	N.A.	0
RGN	-0,198121942	0,016520309	0	N.A.	0
MSI1	-0,198128705	-0,32709653	0	N.A.	0
CRABP1	-0,198142611	-0,099420258	0	N.A.	0
HSPA4L	-0,198151506	-0,158446697	0	N.A.	0
ICMT	-0,198195477	0,14876996	0	N.A.	0
BLOC1S2	-0,198203605	-0,370026046	0	N.A.	0
MIR181A2HG	-0,198214789	-0,111355745	0	N.A.	0
WDFY4	-0,198280468	-0,247591382	0	N.A.	0
NMI	-0,198501202	-0,084654697	0	N.A.	0
RP11-135A1.	-0,198554536	0,00701776	0	N.A.	0
RP11-119B16	-0,198652321	0,232661398	0	N.A.	0
NIPA2	-0,198683179	-0,161816732	0	N.A.	0
RP1-39G22.7	-0,198732973	-0,001650266	0	N.A.	0
COPG2	-0,198868572	-0,267509541	0	N.A.	0
DPM3	-0,199011415	0,240524787	0	N.A.	0
ENDOG	-0,199061374	0,129618395	0	N.A.	0
C17orf53	-0,199082187	-0,39178644	0	N.A.	0
TRMT5	-0,199116177	-0,320860322	0	N.A.	0
TSTD1	-0,199147603	-0,074802152	0	N.A.	0
CHRAC1	-0,199255539	-0,234962704	0	N.A.	0
THUMPD1	-0,199348429	0,063199556	0	N.A.	0
ARSE	-0,199380524	-0,261512814	0	N.A.	0
THAP5	-0,199475495	-0,377571489	0	N.A.	0
OGFOD1	-0,199673008	-0,276465684	0	N.A.	0
ZMPSTE24	-0,199681031	-0,023851151	0	N.A.	0

ADH6	-0,199793135	-0,254448158	0	N.A.	0
GIMAP4	-0,199895134	-0,245220464	0	N.A.	0
EMC10	-0,199921813	0,288985857	0	N.A.	0
NKAP	-0,200138521	0,186279041	0	N.A.	0
COX15	-0,200198826	-0,32194841	0	N.A.	0
SLC35A4	-0,200203105	-0,276011862	0	N.A.	0
DDX20	-0,200249816	0,041198608	0	N.A.	0
DNAJC15	-0,200386342	-0,030878277	0	N.A.	0
GPRIN3	-0,200401442	-0,251865151	0	N.A.	0
HNRNPCL1	-0,200570452	0,048387275	0	N.A.	0
SEC22C	-0,200794041	-0,315591127	0	N.A.	0
LTF	-0,200946651		0	N.A.	0
SLC35F2	-0,201094875	0,073813891	0	N.A.	0
KIAA1324	-0,201167673	-0,161910479	0	N.A.	0
ATP2C2	-0,201212311	-0,045617061	0	N.A.	0
NFYC	-0,201421098	-0,170188397	0	N.A.	0
ELN	-0,201424868	-0,119282601	0	N.A.	0
CHCHD5	-0,201607186	-0,165975654	0	N.A.	0
LIPT1	-0,20162404	-0,097276386	0	N.A.	0
MAD2L1BP	-0,201689649	0,04750919	0	N.A.	0
MORC4	-0,20169067	0,008401922	0	N.A.	0
ADAMTS15	-0,201760113	-0,068093341	0	N.A.	0
DHRS13	-0,201817982	-0,262811188	0	N.A.	0
RBP2	-0,201920088	-0,378756067	0	N.A.	0
CCR10	-0,202287607	-0,04830035	0	N.A.	0
TXNDC5	-0,202293694	0,050284112	0	N.A.	0
THNSL1	-0,202429738	-0,067558276	0	N.A.	0
LRIG3	-0,202477041	0,075488971	0	N.A.	0
SPATC1L	-0,20263455	-0,218007493	0	N.A.	0
RP11-435O5.	-0,202702239	-0,024385799	0	N.A.	0
CC2D2A	-0,202731662	-0,01997541	0	N.A.	0
LIMK2	-0,202756362	-0,058605956	0	N.A.	0
SERGEF	-0,20276378	-0,159372085	0	N.A.	0

MPP6	-0,202778379	-0,162927134	0	N.A.	0
ZNF253	-0,202796122	-0,255527781	0	N.A.	0
NFXL1	-0,202821035	-0,166119477	0	N.A.	0
DROSHA	-0,202962896	-0,057958545	0	N.A.	0
C6orf136	-0,203019144	0,072946234	0	N.A.	0
DUSP11	-0,203037148	-0,106960208	0	N.A.	0
RP11-554J4.1	-0,203244069	0,01586197	0	N.A.	0
TIAL1	-0,203297557	0,089054699	0	N.A.	0
ACIN1	-0,203302023	0,168923372	0	N.A.	0
RP11-452H21	-0,203628827		0	N.A.	0
ACTN1	-0,203718761	0,218246475	0	N.A.	0
RP1-40E16.1	-0,203893922		0	N.A.	0
SETMAR	-0,2040138	-0,223678287	0	N.A.	0
TMSB4Y	-0,204078788	0,095117678	0	N.A.	0
TOLLIP-AS1	-0,204182184	0,129829616	0	N.A.	0
FGFR1OP	-0,204428794	0,091567217	0	N.A.	0
DTD2	-0,204524891	-0,310539922	0	N.A.	0
ZNHIT1	-0,204546179	-0,284159503	0	N.A.	0
BAG6	-0,204553642	0,006624446	0	N.A.	0
RP1-315G1.3	-0,204632273	-0,194314711	0	N.A.	0
ARL1	-0,204655254	-0,013335131	0	N.A.	0
ZFR	-0,20465898	-0,040905385	0	N.A.	0
UBL5	-0,204720679	-0,253187555	0	N.A.	0
ELP2	-0,204832473	-0,316567973	0	N.A.	0
RAP1GDS1	-0,204920437	-0,240671954	0	N.A.	0
CH17-373J23	-0,204935164	0,222818468	0	N.A.	0
ATP6V0E2-AS	-0,205096201	0,126963724	0	N.A.	0
WARS2	-0,205177824	-0,227223602	0	N.A.	0
SRP68	-0,205258578	0,086415381	0	N.A.	0
C2orf15	-0,205270779	-0,226510172	0	N.A.	0
HMGA1	-0,205533787	-0,081281524	0	N.A.	0
RAC3	-0,205565321	-0,042341834	0	N.A.	0
IQCH-AS1	-0,205652869	-0,13259006	0	N.A.	0

DAD1	-0,205664114	0,037353732	0	N.A.	0
ZNF32	-0,205717836	-0,055887732	0	N.A.	0
DMAP1	-0,205813619	-0,227023324	0	N.A.	0
LETM1	-0,205921587	0,045885429	0	N.A.	0
LINC01123	-0,206077733	-0,167841674	0	N.A.	0
NEK3	-0,20607887	-0,099894223	0	N.A.	0
EMC3	-0,206123505	0,021133513	0	N.A.	0
M6PR	-0,206215074	-0,081490258	0	N.A.	0
GPHN	-0,206440481	-0,041243728	0	N.A.	0
ASH1L-AS1	-0,20648977	-0,033271807	0	N.A.	0
CYB561	-0,206562272	0,225170062	0	N.A.	0
MDM1	-0,206564078	-0,082929153	0	N.A.	0
OTX1	-0,206690941	0,093202383	0	N.A.	0
ZSCAN16	-0,206816754	-0,247653657	0	N.A.	0
MRFAP1L1	-0,20693204	0,013901128	0	N.A.	0
SPAG16	-0,206949377	-0,255578226	0	N.A.	0
FADS2	-0,207156084	0,037302627	0	N.A.	0
SCP2	-0,207290327	-0,173031591	0	N.A.	0
CALR	-0,207446917	0,208746589	0	N.A.	0
HIST2H2AC	-0,207476409	-0,306606155	0	N.A.	0
ARFIP2	-0,207521207	0,01253073	0	N.A.	0
CBX2	-0,207537571	-0,161860894	0	N.A.	0
SLC35B4	-0,207615813	-0,150215976	0	N.A.	0
PUS3	-0,207730628	-0,132633495	0	N.A.	0
TSPAN7	-0,207751097	0,024290205	0	N.A.	0
GSTM4	-0,207974984	-0,234210693	0	N.A.	0
GNPDA2	-0,207981707	0,001639673	0	N.A.	0
AC004540.4	-0,208045535	-0,119028173	0	N.A.	0
COX7A2	-0,208315924	-0,086761354	0	N.A.	0
SAMD5	-0,208339363	-0,050851455	0	N.A.	0
ODAM	-0,208432868	0,015658323	0	N.A.	0
AMMECR1	-0,20843353	0,164911915	0	N.A.	0
LRRC26	-0,208544499	-0,16008841	0	N.A.	0

ABCC4	-0,208609279	-0,297633728	0	N.A.	0
MPI	-0,208610639	-0,273478542	0	N.A.	0
WDR73	-0,208737227	-0,129011033	0	N.A.	0
ERGIC2	-0,208764639	0,034181402	0	N.A.	0
AC007036.6	-0,208914875		0	N.A.	0
HOXA11-AS	-0,209068456	0,086572494	0	N.A.	0
WIPI2	-0,209225826	0,065212316	0	N.A.	0
GPATCH11	-0,209362466	-0,108981967	0	N.A.	0
TMEM128	-0,209526551	-0,208713452	0	N.A.	0
RP11-544A12	-0,20961658	-0,026937458	0	N.A.	0
XXbac-B135H	-0,209761744	-0,066776714	0	N.A.	0
ZNHIT2	-0,209774214	-0,277098394	0	N.A.	0
PXN-AS1	-0,209857046	-0,025664792	0	N.A.	0
THAP4	-0,209897952	-0,229032786	0	N.A.	0
SCRN3	-0,210411515	-0,190559986	0	N.A.	0
ZNF775	-0,210412623	-0,139410524	0	N.A.	0
IQCB1	-0,210674616	0,122322867	0	N.A.	0
RP11-421L21	-0,210722153	-0,256306046	0	N.A.	0
RAB5C	-0,21078436	-0,142206675	0	N.A.	0
DNAH14	-0,210790101	-0,385346471	0	N.A.	0
GABRA4	-0,210791976	-0,045904614	0	N.A.	0
COX19	-0,210817975	-0,051520323	0	N.A.	0
MLKL	-0,210827245	-0,084889357	0	N.A.	0
SLC7A6OS	-0,210841198	0,156240003	0	N.A.	0
NDUFA3	-0,210909233	-0,426168873	0	N.A.	0
FAAH	-0,211044582	-0,095122552	0	N.A.	0
GRB2	-0,211066598	-0,316380235	0	N.A.	0
MOK	-0,211150457	0,039514438	0	N.A.	0
DAXX	-0,211159448	-0,206017933	0	N.A.	0
TMEM192	-0,211217296	-0,134093716	0	N.A.	0
R3HCC1	-0,21135444	-0,120649212	0	N.A.	0
NMT1	-0,211398681	-0,128497058	0	N.A.	0
MDH1B	-0,211430599		0	N.A.	0

RP11-77P6.2	-0,211522894	-0,232363206	0	N.A.	0
DFFB	-0,211616076	-0,091441247	0	N.A.	0
UGT8	-0,2118027	0,332305784	0	N.A.	0
WDR41	-0,21184838	-0,427391405	0	N.A.	0
MCEE	-0,21185167	0,174206649	0	N.A.	0
GMPR2	-0,211863297	-0,132045252	0	N.A.	0
KCTD6	-0,211948933	-0,406656928	0	N.A.	0
PFN1	-0,212003836	-0,426135519	0	N.A.	0
PXYLP1	-0,212087053	-0,391633974	0	N.A.	0
C17orf58	-0,212115844	-0,12216312	0	N.A.	0
VMA21	-0,212128429	-0,267094459	0	N.A.	0
SNAPC5	-0,212199522	-0,339137013	0	N.A.	0
BPHL	-0,21225554	0,078525479	0	N.A.	0
STARD3NL	-0,212438116	-0,432511072	0	N.A.	0
CCDC51	-0,212512315	-0,140886359	0	N.A.	0
CYP2W1	-0,212527006	-0,043506893	0	N.A.	0
HM13	-0,212822785	0,293720895	0	N.A.	0
POGLUT1	-0,212839094	-0,14597082	0	N.A.	0
CAMK1	-0,212884	-0,205558146	0	N.A.	0
RPP21	-0,212943633	-0,434131881	0	N.A.	0
C5orf34	-0,213142677	-0,06935674	0	N.A.	0
BOK-AS1	-0,213157167	-0,161312401	0	N.A.	0
TRA2A	-0,213165433	-0,030686546	0	N.A.	0
YY1	-0,213182912	0,153566067	0	N.A.	0
METTL12	-0,213188996	-0,222289698	0	N.A.	0
C3orf70	-0,213368409	-0,20223635	0	N.A.	0
OTUB1	-0,213377393	0,37442668	0	N.A.	0
SMG9	-0,213490224	-0,344244002	0	N.A.	0
MPDU1	-0,213677374	0,03784698	0	N.A.	0
LRRC58	-0,213790523	0,286523232	0	N.A.	0
XPA	-0,214077555	0,079187972	0	N.A.	0
MTRF1	-0,214094313	-0,442622827	0	N.A.	0
AC004381.6	-0,214132945	-0,098511226	0	N.A.	0

RP11-81H14.	-0,214192889	-0,012595286	0	N.A.	0
KCTD3	-0,214194232	0,28284269	0	N.A.	0
ZNF627	-0,214222797	-0,32049758	0	N.A.	0
DPF1	-0,214237477		0	N.A.	0
RUFY1	-0,21424023	0,046835484	0	N.A.	0
ZNF670	-0,214261961	-0,235608211	0	N.A.	0
RNLS	-0,214282202	-0,126631266	0	N.A.	0
PGS1	-0,214325544	-0,172048817	0	N.A.	0
CUL4A	-0,214556546	0,132651919	0	N.A.	0
SMIM3	-0,21456797	-0,261163945	0	N.A.	0
MAATS1	-0,214634401	-0,253510281	0	N.A.	0
RHBDD3	-0,21467908	0,178968735	0	N.A.	0
RP11-108M9	-0,214692012	0,078084904	0	N.A.	0
DECR2	-0,214717483	0,341208566	0	N.A.	0
ZNF770	-0,214730009	-0,285866534	0	N.A.	0
TTC32	-0,214788209	-0,150620091	0	N.A.	0
IRF3	-0,215074783	0,23517795	0	N.A.	0
REG3A	-0,215084986	0,006584699	0	N.A.	0
CAGE1	-0,215108837		0	N.A.	0
CCDC150	-0,215233992	-0,204814212	0	N.A.	0
DXO	-0,215238164	-0,341564685	0	N.A.	0
MTFR1	-0,215372236	-0,304401821	0	N.A.	0
KIAA1279	-0,215378023	-0,08270767	0	N.A.	0
PPP6C	-0,21539232	-0,349489369	0	N.A.	0
WDR90	-0,215440987	0,02035704	0	N.A.	0
STK26	-0,215517491	-0,160984017	0	N.A.	0
DNAJC30	-0,215743887	-0,296624209	0	N.A.	0
ZNF691	-0,215805137	-0,204616242	0	N.A.	0
C11orf74	-0,215895418	-0,315652156	0	N.A.	0
ZSCAN12	-0,215975236	0,141973735	0	N.A.	0
SAYSD1	-0,216431765	-0,144985795	0	N.A.	0
NF2	-0,216434148	-0,086879406	0	N.A.	0
ERGIC3	-0,216459815	-0,219455508	0	N.A.	0

UPF2	-0,216465339	0,152614056	0	N.A.	0
TRMT2A	-0,216561753	0,081442194	0	N.A.	0
GUF1	-0,216614062	-0,269974765	0	N.A.	0
STK33	-0,216626021		0	N.A.	0
TEX261	-0,216713041	-0,055459307	0	N.A.	0
COLGALT1	-0,216814434	0,164129435	0	N.A.	0
AE000662.93	-0,216927108	0,111157156	0	N.A.	0
PODXL2	-0,216990102	-0,046518477	0	N.A.	0
NSDHL	-0,217093424	0,157459579	0	N.A.	0
SLC12A8	-0,217157264	-0,149460061	0	N.A.	0
ZNF330	-0,217233071	-0,24804951	0	N.A.	0
MCUR1	-0,21723756	0,054355072	0	N.A.	0
FREM1	-0,217318526	-0,071928338	0	N.A.	0
SPRY1	-0,217376446	0,226837555	0	N.A.	0
SRA1	-0,217479641	-0,283530558	0	N.A.	0
DHX33	-0,217688242	-0,056869112	0	N.A.	0
OXGR1	-0,217714173	-0,173812636	0	N.A.	0
RP11-660L16	-0,217750606	-0,19262926	0	N.A.	0
NHLRC2	-0,217809368	0,245485365	0	N.A.	0
SCO2	-0,217823249	0,291504062	0	N.A.	0
DHX36	-0,217892695	0,067642583	0	N.A.	0
RNF34	-0,217983646	-0,094856845	0	N.A.	0
KCTD16	-0,217997317	-0,078052946	0	N.A.	0
URI1	-0,218069881	0,101028827	0	N.A.	0
SPINK2	-0,218455928	-0,276857145	0	N.A.	0
SORBS3	-0,218507509	-0,041840263	0	N.A.	0
ABI2	-0,218563817	-0,394244745	0	N.A.	0
FDPS	-0,218613961	0,080274163	0	N.A.	0
PTS	-0,218678465	-0,038355404	0	N.A.	0
PPIL4	-0,218715476	0,16514939	0	N.A.	0
DNM1L	-0,218729675	0,012165405	0	N.A.	0
AMBP	-0,218748452	-0,207504048	0	N.A.	0
HEATR3	-0,218820549	0,120480041	0	N.A.	0

USP3-AS1	-0,218845841	0,125243342	0	N.A.	0
OTC	-0,218941729	0,073284118	0	N.A.	0
CABIN1	-0,218983227	0,042911232	0	N.A.	0
CPSF3L	-0,21899111	-0,056630714	0	N.A.	0
CEL	-0,219001204	-0,25570179	0	N.A.	0
AMZ2	-0,219029055	-0,294344119	0	N.A.	0
AP000275.65	-0,219154286		0	N.A.	0
CEP152	-0,219203562	0,073441203	0	N.A.	0
C9orf24	-0,219216492	-0,192354139	0	N.A.	0
CROT	-0,219229821	0,106932922	0	N.A.	0
MEAF6	-0,219241677	-0,082982877	0	N.A.	0
ID3	-0,219243004	-0,373603274	0	N.A.	0
TNFAIP8L1	-0,219393149	-0,380499445	0	N.A.	0
LAMB1	-0,219563333	-0,011000069	0	N.A.	0
MPV17	-0,219653394	-0,255694853	0	N.A.	0
SLC25A38	-0,219671997	-0,267259418	0	N.A.	0
AC016700.5	-0,219745586	-0,152416213	0	N.A.	0
GARS	-0,219810808	-0,038500567	0	N.A.	0
RIBC2	-0,219874027	-0,16392197	0	N.A.	0
SAMHD1	-0,219927217	-0,31573247	0	N.A.	0
GALNT18	-0,219991378	0,071031556	0	N.A.	0
AC004069.2	-0,220077502	-0,286545251	0	N.A.	0
KDM8	-0,220828462	0,006164755	0	N.A.	0
ZP2	-0,221026306	-0,049038601	0	N.A.	0
COX18	-0,221032953	-0,240978086	0	N.A.	0
TBXAS1	-0,221087974	-0,020882836	0	N.A.	0
NVL	-0,221155624	-0,124745114	0	N.A.	0
ZNF622	-0,221198819	0,099431127	0	N.A.	0
STK16	-0,221277848	-0,091662695	0	N.A.	0
COMMD3	-0,221280992	0,036680346	0	N.A.	0
CTD-2532N2(	-0,221310061		0	N.A.	0
ZNF738	-0,221522993	-0,366464637	0	N.A.	0
MMACHC	-0,221626957	-0,367178396	0	N.A.	0

NRGN	-0,22165919	-0,210035124	0	N.A.	0
RHOT2	-0,221721912	0,222714658	0	N.A.	0
FAM201A	-0,221798141	-0,253705942	0	N.A.	0
GIPC2	-0,221849059	-0,301572782	0	N.A.	0
NAT14	-0,221867396	0,339481608	0	N.A.	0
EVI2A	-0,221972534	-0,321712878	0	N.A.	0
PDDC1	-0,221983076	-0,112790409	0	N.A.	0
ELP3	-0,221985888	-0,166824626	0	N.A.	0
MAGEA11	-0,221989542	-0,283407726	0	N.A.	0
CHURC1	-0,222027029	-0,271776409	0	N.A.	0
SEC23B	-0,222122904	-0,103637216	0	N.A.	0
IFT57	-0,222154662	-0,236608527	0	N.A.	0
ZNF724P	-0,222156945	0,007284973	0	N.A.	0
TTC23	-0,222219266	-0,110076071	0	N.A.	0
EMC6	-0,222273011	-0,098826135	0	N.A.	0
AP006222.2	-0,222313601	-0,248979881	0	N.A.	0
PIF1	-0,222428315	-0,053647963	0	N.A.	0
DBT	-0,222602408	-0,099273609	0	N.A.	0
AC093388.3	-0,222631867	-0,176848757	0	N.A.	0
RP11-438L19	-0,222644074	0,327847792	0	N.A.	0
SLC35B1	-0,22265838	-0,058178175	0	N.A.	0
LINC01011	-0,222798418	-0,116835401	0	N.A.	0
KB-431C1.4	-0,222989388	-0,097599328	0	N.A.	0
MOCS3	-0,223001387	-0,159018277	0	N.A.	0
RP3-509I19.1	-0,223136712	0,021350318	0	N.A.	0
FAM229B	-0,223206456	-0,21183508	0	N.A.	0
SLC1A4	-0,223362632	-0,193356738	0	N.A.	0
GPR143	-0,223461148	0,056922246	0	N.A.	0
LINC01106	-0,223464002	-0,019347268	0	N.A.	0
GSTT2B	-0,223510675	-0,219977837	0	N.A.	0
RNF214	-0,223702471	-0,090698007	0	N.A.	0
YARS	-0,223936057	-0,19463655	0	N.A.	0
NUP160	-0,224010464	-0,077743988	0	N.A.	0

GPR150	-0,224064608	-0,117489213	0	N.A.	0
PIGL	-0,224084312	-0,204590762	0	N.A.	0
POLR1A	-0,224107164	-0,15047648	0	N.A.	0
PROB1	-0,224113592	0,140690359	0	N.A.	0
TMPO-AS1	-0,224156899	-0,321511634	0	N.A.	0
PTCD1	-0,224273269	-0,10572009	0	N.A.	0
CPNE6	-0,224288291	-0,160372014	0	N.A.	0
EEA1	-0,224563775	0,052824639	0	N.A.	0
SELENBP1	-0,224592217	-0,096225786	0	N.A.	0
TEX10	-0,224645578	-0,270105467	0	N.A.	0
CRYM	-0,224671027	0,001774965	0	N.A.	0
PAXIP1-AS1	-0,224741036	-0,028852185	0	N.A.	0
SLC2A8	-0,224844525	-0,031599852	0	N.A.	0
B4GALT7	-0,224929052	0,195532299	0	N.A.	0
GOPC	-0,225154698	0,018733775	0	N.A.	0
RPRD1A	-0,225412529	0,126829336	0	N.A.	0
SCRN2	-0,225455102	0,212165852	0	N.A.	0
PAIP1	-0,225719347	-0,029876274	0	N.A.	0
CTB-58E17.1	-0,225919164	0,150499259	0	N.A.	0
ZNF714	-0,225969318	-0,302381165	0	N.A.	0
C19orf57	-0,226056212	0,061196569	0	N.A.	0
SRSF8	-0,226204776	-0,119882264	0	N.A.	0
SLC25A14	-0,226309002	-0,411575052	0	N.A.	0
MEX3A	-0,226580668	-0,196365671	0	N.A.	0
MPHOSPH9	-0,227014133	-0,072030896	0	N.A.	0
C2	-0,227057444	-0,15487401	0	N.A.	0
KRI1	-0,227103091	0,203316231	0	N.A.	0
ERI1	-0,227373664	-0,088303027	0	N.A.	0
BLOC1S4	-0,227395372	-0,009477692	0	N.A.	0
RHOA	-0,227442388	-0,294505273	0	N.A.	0
TMEM206	-0,227451158	-0,162204378	0	N.A.	0
ZKSCAN4	-0,227520121	-0,031718199	0	N.A.	0
MTERF1	-0,227572399	-0,321701215	0	N.A.	0

C11orf1	-0,227658113	-0,369213563	0	N.A.	0
ETF1	-0,227717326	-0,184587346	0	N.A.	0
LINC01091	-0,227791156	-0,314857644	0	N.A.	0
ACO1	-0,227907227	-0,050091045	0	N.A.	0
TMTC4	-0,228085903	0,302582174	0	N.A.	0
CTD-2371O3	-0,228105464	0,083301078	0	N.A.	0
ISCA1	-0,228190653	-0,234774457	0	N.A.	0
POT1	-0,228484438	-0,287658386	0	N.A.	0
E2F3	-0,228547118	-0,167324984	0	N.A.	0
ERCC4	-0,228594857	-0,062031108	0	N.A.	0
LINC00261	-0,228620319	-0,101518177	0	N.A.	0
RAD51D	-0,228625356	0,092257599	0	N.A.	0
PREP	-0,228789477	0,174552324	0	N.A.	0
GNL3L	-0,228860764	-0,315102642	0	N.A.	0
SERPINA6	-0,228922512	0,060687742	0	N.A.	0
ABCD3	-0,229168057	0,375428557	0	N.A.	0
SOX2	-0,229225194	-0,139275941	0	N.A.	0
PATZ1	-0,229343148	-0,317562219	0	N.A.	0
AC002310.12	-0,229369262	0,036190273	0	N.A.	0
LSM1	-0,229549303	0,081035592	0	N.A.	0
MRGBP	-0,229563398	0,17906085	0	N.A.	0
ZMAT2	-0,229591643	-0,471432629	0	N.A.	0
SLC25A26	-0,229619688	-0,154388088	0	N.A.	0
PRPF8	-0,229662342	0,119133609	0	N.A.	0
SETD6	-0,229703041	-0,065700922	0	N.A.	0
SRSF4	-0,229801682	0,112361021	0	N.A.	0
RSAD1	-0,229867363	-0,023263296	0	N.A.	0
MMS19	-0,229901781	0,04734095	0	N.A.	0
SCFD2	-0,230094105	-0,24704693	0	N.A.	0
SHQ1	-0,230106463	-0,413572609	0	N.A.	0
GFM2	-0,230171123	-0,129898128	0	N.A.	0
CYTL1	-0,230176819		0	N.A.	0
LCMT1	-0,230185803	-0,199014673	0	N.A.	0

RAD54B	-0,23023778	-0,258654815	0	N.A.	0
CNTNAP2	-0,23024422	-0,06745628	0	N.A.	0
UBA6-AS1	-0,230346621	-0,339562172	0	N.A.	0
CCDC163P	-0,230356114	-0,235708423	0	N.A.	0
FASTKD5	-0,230378405	0,038296906	0	N.A.	0
DHX30	-0,230461197	-0,136188035	0	N.A.	0
B4GALT3	-0,23066153	-0,179430141	0	N.A.	0
SPATA5L1	-0,230789295	-0,061956339	0	N.A.	0
LEF1	-0,230814264	-0,179247869	0	N.A.	0
GSTP1	-0,230976545	-0,144501782	0	N.A.	0
GSTZ1	-0,230995674	-0,258434396	0	N.A.	0
TMEM138	-0,231018164	-0,045050739	0	N.A.	0
FAM120C	-0,231066025	-0,088145362	0	N.A.	0
MYL5	-0,231158387	-0,258096201	0	N.A.	0
RARS2	-0,231272064	-0,068311958	0	N.A.	0
USE1	-0,23134058	-0,515269125	0	N.A.	0
SNRNP35	-0,231345794	-0,157632581	0	N.A.	0
CEP63	-0,231547054	-0,097284437	0	N.A.	0
GTPBP6	-0,231653028	-0,14113289	0	N.A.	0
PALD1	-0,231684151	-0,145077243	0	N.A.	0
FASTKD3	-0,23171409	-0,129738322	0	N.A.	0
RSRC2	-0,231788768	-0,205912354	0	N.A.	0
HMGA2	-0,231903257	-0,059043457	0	N.A.	0
EFCC1	-0,231966647	-0,06380488	0	N.A.	0
GTF3C3	-0,232051142	0,127107028	0	N.A.	0
DDX11	-0,232098275	0,012922572	0	N.A.	0
BCL2L10	-0,232223994	-0,426269762	0	N.A.	0
ICE2	-0,23230479	-0,243561053	0	N.A.	0
METTL2B	-0,232368485	-0,326784414	0	N.A.	0
RP11-727F15	-0,232536746	0,0095814	0	N.A.	0
NEK4	-0,232671458	-0,283523082	0	N.A.	0
IFT81	-0,232791984	-0,075901372	0	N.A.	0
RP11-176H8.	-0,232860784	-0,352370734	0	N.A.	0

TIMM22	-0,233105814	-0,078135211	0	N.A.	0
DDX19A	-0,233146349	0,265832947	0	N.A.	0
MDN1	-0,233162631	-0,02830413	0	N.A.	0
CTD-2501E16	-0,233162838	-0,096918252	0	N.A.	0
LRRC61	-0,233166632	-0,302098627	0	N.A.	0
USP21	-0,233224681	-0,058624626	0	N.A.	0
CMTM2	-0,23326535	0,134851525	0	N.A.	0
HAUS2	-0,233311657	-0,166136102	0	N.A.	0
C12orf43	-0,233338788	0,019820914	0	N.A.	0
ZSCAN5A	-0,233375199	-0,070380282	0	N.A.	0
ZNF93	-0,233412212	-0,322629907	0	N.A.	0
CMBL	-0,233433905	-0,041573649	0	N.A.	0
ZBED8	-0,233510477	0,053985294	0	N.A.	0
ST8SIA6-AS1	-0,233597244		0	N.A.	0
LIG3	-0,233646721	-0,124364928	0	N.A.	0
IMMP2L	-0,233706802	-0,430365832	0	N.A.	0
VPS26A	-0,233803356	-0,234952328	0	N.A.	0
CPNE7	-0,233805331	-0,197058403	0	N.A.	0
FAM71E1	-0,233917251	-0,302996993	0	N.A.	0
MAT1A	-0,233996396	-0,072800147	0	N.A.	0
SNHG9	-0,234145611	-0,126932463	0	N.A.	0
WDR83OS	-0,234279318	-0,231571037	0	N.A.	0
STUB1	-0,2343621	0,009003248	0	N.A.	0
CDC27	-0,234418434	-0,168266383	0	N.A.	0
PRDM5	-0,234517266	0,106157554	0	N.A.	0
TTC13	-0,234551312	0,043967738	0	N.A.	0
C8orf76	-0,234562222	0,174004376	0	N.A.	0
TMEM180	-0,234565945	-0,108409858	0	N.A.	0
ST20	-0,234741046	-0,341140316	0	N.A.	0
ZNF48	-0,234831956	-0,080331918	0	N.A.	0
ASNSD1	-0,234990956	-0,429039886	0	N.A.	0
SFT2D1	-0,235003601	0,081321093	0	N.A.	0
LRP3	-0,235143327	-0,208998675	0	N.A.	0

NIPSNAP3A	-0,235166559	-0,24045707	0	N.A.	0
TMX4	-0,235188299	-0,009323192	0	N.A.	0
CEP41	-0,235234325	-0,259885758	0	N.A.	0
RPAP2	-0,235259348	-0,13653263	0	N.A.	0
NUP62	-0,235386903	-0,199718663	0	N.A.	0
FASTK	-0,235440067	0,078349656	0	N.A.	0
FAM92A1	-0,23549009	-0,422887443	0	N.A.	0
CPSF2	-0,235557056	-0,035810178	0	N.A.	0
RSU1	-0,235701273	-0,293344997	0	N.A.	0
NUDT22	-0,235726469	-0,065829065	0	N.A.	0
AC093323.3	-0,235837134	-0,033966431	0	N.A.	0
PSMB9	-0,236055445	-0,407226064	0	N.A.	0
E2F2	-0,236077845	-0,24095714	0	N.A.	0
PRAC1	-0,236155236	-0,378627583	0	N.A.	0
KRTCAP2	-0,236162693	0,108469508	0	N.A.	0
MTRF1L	-0,23618611	-0,095758773	0	N.A.	0
GPR89A	-0,236362233	0,112299018	0	N.A.	0
TCF3	-0,236499705	0,300010136	0	N.A.	0
TPM2	-0,236588386	-0,280144908	0	N.A.	0
TAF8	-0,236591986	0,016318747	0	N.A.	0
RASD1	-0,236615704	-0,15640324	0	N.A.	0
XPO5	-0,236852927	-0,046962977	0	N.A.	0
CCDC115	-0,236870372	-0,190460123	0	N.A.	0
GPI	-0,236972252	-0,178334225	0	N.A.	0
STRADB	-0,237074145	-0,291987422	0	N.A.	0
CCDC138	-0,237096419	-0,196447741	0	N.A.	0
HIST1H1B	-0,237146239	-0,368797211	0	N.A.	0
ZSCAN16-AS1	-0,237467546	-0,320219961	0	N.A.	0
HYPK	-0,237506457	-0,029583725	0	N.A.	0
SPNS1	-0,237558592	0,201063575	0	N.A.	0
NUCB2	-0,237581725	0,031405332	0	N.A.	0
SDR39U1	-0,237590797	-0,132428926	0	N.A.	0
XRCC4	-0,237607508	-0,14449852	0	N.A.	0

DEPTOR	-0,237612144	-0,009315114	0	N.A.	0
AAR2	-0,237670859	0,036050461	0	N.A.	0
RCC2	-0,237689539	0,010125641	0	N.A.	0
SNHG8	-0,237788408	-0,278231036	0	N.A.	0
TTC12	-0,237851593	-0,166365568	0	N.A.	0
RP11-728G15	-0,237890848	0,008933511	0	N.A.	0
SART1	-0,237994242	-0,026959674	0	N.A.	0
SMS	-0,238031288	-0,508674266	0	N.A.	0
ARRDC1-AS1	-0,23847582	-0,0288482	0	N.A.	0
RPAIN	-0,238505847	-0,240073472	0	N.A.	0
FAM72C	-0,238533249	-0,252833844	0	N.A.	0
LGR5	-0,238671402	-0,047819679	0	N.A.	0
ZNF174	-0,238857933	-0,08373917	0	N.A.	0
KIAA1257	-0,238906987	-0,206613932	0	N.A.	0
SNRNP48	-0,238983349	-0,078336246	0	N.A.	0
CASC21	-0,239035732	-0,043024243	0	N.A.	0
ABCB8	-0,239129948	0,106410733	0	N.A.	0
TUBD1	-0,239191288	-0,206904741	0	N.A.	0
SMARCAD1	-0,239265253	-0,319411908	0	N.A.	0
GMCL1	-0,239392235	-0,234536643	0	N.A.	0
C2orf76	-0,239469593	-0,234757378	0	N.A.	0
ARL13B	-0,239482919	-0,234528632	0	N.A.	0
TMEM216	-0,239589718	-0,010761562	0	N.A.	0
TMC3-AS1	-0,239976027	-0,313384893	0	N.A.	0
MGC32805	-0,240120796	-0,153535754	0	N.A.	0
MORF4L2	-0,240233644	-0,285612179	0	N.A.	0
UBXN8	-0,240260499	0,104398351	0	N.A.	0
TMEM55A	-0,240321309	-0,230363442	0	N.A.	0
ACAD8	-0,240434927	-0,173226989	0	N.A.	0
ABHD14A	-0,240437248	-0,394654328	0	N.A.	0
ATPAF2	-0,240460648	-0,051646413	0	N.A.	0
SMTNL2	-0,240524448	-0,000122524	0	N.A.	0
IP6K2	-0,240589254	0,096591588	0	N.A.	0

SDHAF2	-0,240639166	-0,196113078	0	N.A.	0
NUDT19	-0,240821559	-0,196754216	0	N.A.	0
MKS1	-0,241151986	-0,173210143	0	N.A.	0
TOMM70A	-0,241238517	-0,42780928	0	N.A.	0
UTP15	-0,24126629	-0,372639406	0	N.A.	0
C17orf80	-0,241483034	0,099360868	0	N.A.	0
FAM200B	-0,24177109	-0,3217477	0	N.A.	0
PITPNA-AS1	-0,241830433	-0,202715108	0	N.A.	0
COG2	-0,241855019	0,028912873	0	N.A.	0
SREK1	-0,241869134	0,144489638	0	N.A.	0
ZNF599	-0,24193627	-0,266963303	0	N.A.	0
RP11-38C17.	-0,242133204	-0,054214261	0	N.A.	0
TXNL4B	-0,242410813	-0,157144498	0	N.A.	0
APOLD1	-0,242462431	-0,259686756	0	N.A.	0
CALML3	-0,242465986	0,063931845	0	N.A.	0
CCDC109B	-0,242545495	-0,212095014	0	N.A.	0
ZNF74	-0,242565361	-0,223694482	0	N.A.	0
SCOC	-0,242621324	-0,126095384	0	N.A.	0
IL27RA	-0,242711041	-0,349593154	0	N.A.	0
TUBGCP2	-0,242758648	0,120964545	0	N.A.	0
ANAPC4	-0,242814202	0,072376533	0	N.A.	0
SCML2	-0,242834927	0,063247496	0	N.A.	0
DNAJB11	-0,242906167	0,311320617	0	N.A.	0
IRAK1BP1	-0,243156744	-0,474845595	0	N.A.	0
LARP1	-0,243265365	0,201659183	0	N.A.	0
HAAO	-0,243268117	-0,287683876	0	N.A.	0
HLTF	-0,243300526	-0,44902164	0	N.A.	0
KIAA1586	-0,24358768	0,030292315	0	N.A.	0
ST7L	-0,243622616	-0,176418557	0	N.A.	0
CUTC	-0,24367434	-0,169929812	0	N.A.	0
SUPT20H	-0,243694572	0,10548445	0	N.A.	0
MTA2	-0,243863931	-0,194021512	0	N.A.	0
C12orf57	-0,243972426	-0,423614027	0	N.A.	0

TNPO2	-0,244177846	-0,132036507	0	N.A.	0
TMED2	-0,244213661	-0,106836205	0	N.A.	0
PEX5	-0,244546866	0,061893147	0	N.A.	0
PHYHIPL	-0,244765523	-0,10920217	0	N.A.	0
RNFT2	-0,244777309	-0,163952205	0	N.A.	0
C14orf80	-0,244897928	-0,311455471	0	N.A.	0
ASXL1	-0,244959933	-0,192184157	0	N.A.	0
NARF	-0,245019011	0,048654876	0	N.A.	0
ASGR2	-0,245030377	-0,087969201	0	N.A.	0
FAHD1	-0,245074897	-0,117225745	0	N.A.	0
SYNE4	-0,245101159	0,089926191	0	N.A.	0
LARS2	-0,245341361	-0,087948449	0	N.A.	0
HOXA3	-0,245545798	-0,230227324	0	N.A.	0
PPP4C	-0,24566022	-0,064982131	0	N.A.	0
SMIM8	-0,245677435	-0,007545665	0	N.A.	0
C20orf96	-0,245749956	-0,193607616	0	N.A.	0
FOXA2	-0,245854319	0,123484088	0	N.A.	0
ZNF789	-0,245875569	-0,172621567	0	N.A.	0
NRD1	-0,245909446	0,350119903	0	N.A.	0
NME7	-0,246316572	-0,117110679	0	N.A.	0
PPP2R2C	-0,246345472	-0,292917887	0	N.A.	0
PAK6	-0,246348594	0,014994264	0	N.A.	0
CASP1	-0,246378675	-0,41134328	0	N.A.	0
EPRS	-0,246467213	0,274515705	0	N.A.	0
CSRP2BP	-0,246514886	0,035764432	0	N.A.	0
ALDH3A1	-0,246616089	-0,316672322	0	N.A.	0
C12orf66	-0,246654777	-0,226320944	0	N.A.	0
CHTF8	-0,246662904	0,050487283	0	N.A.	0
CTB-31O20.2	-0,246798475	-0,033377671	0	N.A.	0
EMC2	-0,246988129	-0,21382386	0	N.A.	0
SATB2	-0,247036678	-0,07748086	0	N.A.	0
STOX1	-0,247087446	-0,19281333	0	N.A.	0
ORC2	-0,247122258	0,047256755	0	N.A.	0

RP3-522D1.1	-0,247132412	-0,465475982	0	N.A.	0
SYNPR	-0,247205906		0	N.A.	0
CEP295	-0,247269675	-0,146699652	0	N.A.	0
FAM189B	-0,24731632	-0,206728848	0	N.A.	0
ZNF207	-0,247371076	0,325308865	0	N.A.	0
RRP36	-0,247486717	0,10293363	0	N.A.	0
IGF2	-0,247682908	0,086477932	0	N.A.	0
DTNBP1	-0,247688531	0,013679149	0	N.A.	0
TACR2	-0,247693186	0,140077371	0	N.A.	0
ZNF496	-0,247707497	-0,018078565	0	N.A.	0
MLLT10	-0,247739777	0,052263169	0	N.A.	0
DHX57	-0,24781448	0,113472846	0	N.A.	0
MAP7	-0,248130778	0,02918114	0	N.A.	0
C9orf64	-0,248219771	-0,34696533	0	N.A.	0
NACA2	-0,248468978	-0,430831095	0	N.A.	0
POLD3	-0,248533169	-0,071212167	0	N.A.	0
TXNRD3	-0,248739624	-0,017798132	0	N.A.	0
MKRN2	-0,248742947	-0,248967471	0	N.A.	0
TMEM231	-0,248836663	0,009729617	0	N.A.	0
EVA1A	-0,248911462	-0,082091863	0	N.A.	0
GLYATL1	-0,248911673	-0,360889148	0	N.A.	0
RBM10	-0,249512326	0,007050914	0	N.A.	0
MTBP	-0,249621508	-0,004948634	0	N.A.	0
ARL9	-0,249634459	-0,068818324	0	N.A.	0
GEMIN5	-0,250073388	-0,315665754	0	N.A.	0
ALG14	-0,250078339	0,054633015	0	N.A.	0
ZNF239	-0,250277327	-0,172669425	0	N.A.	0
SMU1	-0,250291673	-0,238323999	0	N.A.	0
DCK	-0,250294556	-0,375166552	0	N.A.	0
MEF2BNB	-0,250676962	-0,122368771	0	N.A.	0
CLPTM1L	-0,250800914	0,312209604	0	N.A.	0
FKBP10	-0,250838906	0,093517076	0	N.A.	0
BRK1	-0,250863833	-0,30356669	0	N.A.	0

MTHFD1L	-0,250886142	-0,243288013	0	N.A.	0
NMNAT3	-0,250965125	-0,23534995	0	N.A.	0
ATAD3B	-0,250970189	-0,091359317	0	N.A.	0
POLR3E	-0,250982006	-0,038643945	0	N.A.	0
MPC1	-0,2510257	-0,209674567	0	N.A.	0
THAP11	-0,251092134	-0,162809976	0	N.A.	0
ELOF1	-0,251170453	-0,021245414	0	N.A.	0
UROD	-0,251231937	-0,121218526	0	N.A.	0
HIRA	-0,251284344	0,012564225	0	N.A.	0
RP11-611E13	-0,251409431	0,012555527	0	N.A.	0
C14orf119	-0,251497681	-0,11851574	0	N.A.	0
SFXN1	-0,251510524	-0,223126641	0	N.A.	0
CIR1	-0,251514124	0,211098736	0	N.A.	0
TAF11	-0,251789463	-0,155727224	0	N.A.	0
SSX2IP	-0,251832758	-0,286931068	0	N.A.	0
UNC119B	-0,252144001	-0,137180128	0	N.A.	0
BCAP31	-0,252311824	0,135836291	0	N.A.	0
BSG	-0,252657979	0,400199387	0	N.A.	0
C12orf60	-0,252770088	-0,142599063	0	N.A.	0
SART3	-0,25282873	0,080039858	0	N.A.	0
DUS2	-0,252829267	-0,13721538	0	N.A.	0
NAA16	-0,252869953	-0,139440163	0	N.A.	0
FTSJ3	-0,253096787	-0,120426961	0	N.A.	0
CTC-459F4.3	-0,253166652	0,000472663	0	N.A.	0
ZSCAN2	-0,25317806	-0,025104141	0	N.A.	0
GKAP1	-0,253203667	0,064933892	0	N.A.	0
TOPBP1	-0,25331061	-0,222809801	0	N.A.	0
YIF1A	-0,25334123	0,174948034	0	N.A.	0
FAM185A	-0,253424909	-0,171364476	0	N.A.	0
CTA-445C9.1	-0,253535698	-0,342305675	0	N.A.	0
POU2F1	-0,253553969	0,180854749	0	N.A.	0
RP11-279F6.1	-0,253693021	0,056291145	0	N.A.	0
PAQR4	-0,253795062	0,456718689	0	N.A.	0

MAPKAPK5-A	-0,253855769	0,053400701	0	N.A.	0
ALDH3A2	-0,254132483	0,026778205	0	N.A.	0
MTAP	-0,254169913	-0,07548164	0	N.A.	0
CA8	-0,254177296	-0,055844661	0	N.A.	0
C14orf169	-0,254519194	0,100570247	0	N.A.	0
TASP1	-0,254699585	-0,072002545	0	N.A.	0
YTHDF3-AS1	-0,254721281	-0,229655506	0	N.A.	0
ZNF92	-0,254762367	-0,131862125	0	N.A.	0
TRIM2	-0,25486887	0,042641438	0	N.A.	0
AC005076.5	-0,255107677	0,099815365	0	N.A.	0
FTCDNL1	-0,255592668	-0,191121839	0	N.A.	0
ATP2A1-AS1	-0,255648088	-0,321903597	0	N.A.	0
RQCD1	-0,25574372	-0,284811656	0	N.A.	0
SLC39A7	-0,255900747	0,09353133	0	N.A.	0
BBS7	-0,255985343	-0,077655386	0	N.A.	0
CTD-3184A7.	-0,256165555	-0,143233975	0	N.A.	0
PMS2	-0,256433489	-0,195817184	0	N.A.	0
RLN2	-0,256440523	-0,186169219	0	N.A.	0
TP53RK	-0,256501478	-0,217335438	0	N.A.	0
AC011043.1	-0,256550324	-0,06829865	0	N.A.	0
SLC25A33	-0,256553243	-0,299069991	0	N.A.	0
TONSL	-0,25660545	-0,101264968	0	N.A.	0
GSPT2	-0,256657487	-0,118409126	0	N.A.	0
CRTAP	-0,256689987	-0,377880629	0	N.A.	0
NEDD1	-0,256914953	-0,336874899	0	N.A.	0
LRRC34	-0,256968395	-0,173814514	0	N.A.	0
ARPC3	-0,256997779	-0,293316689	0	N.A.	0
BNIP1	-0,257014185	-0,121704062	0	N.A.	0
UBE2G2	-0,257074488	-0,184698763	0	N.A.	0
PPARGC1B	-0,257113109	-0,187389858	0	N.A.	0
PANK2	-0,257374308	-0,168091704	0	N.A.	0
PI4K2B	-0,257393342	-0,146664179	0	N.A.	0
GLB1L2	-0,2574013	-0,042522454	0	N.A.	0

DOK1	-0,257437404	-0,039043008	0	N.A.	0
TAGLN2	-0,25748256	-0,12966322	0	N.A.	0
XPO1	-0,257529788	0,052153666	0	N.A.	0
RCHY1	-0,257718478	0,038709247	0	N.A.	0
AGBL5	-0,257809756	-0,387809341	0	N.A.	0
NEIL3	-0,257814414	-0,412965719	0	N.A.	0
CISD2	-0,257835848	-0,10748043	0	N.A.	0
PHYH	-0,257842739	0,034807203	0	N.A.	0
DHRS7B	-0,257961398	0,018050422	0	N.A.	0
SLTM	-0,258129049	0,022989264	0	N.A.	0
TTLL12	-0,258204413	0,21104503	0	N.A.	0
KNTC1	-0,258252797	0,022651544	0	N.A.	0
PPP2CA	-0,258529368	-0,058792089	0	N.A.	0
RXRB	-0,258544313	-0,165076905	0	N.A.	0
RMI1	-0,258706413	-0,158156526	0	N.A.	0
CASP8AP2	-0,258713772	-0,179744633	0	N.A.	0
MTFP1	-0,258773055	-0,09396486	0	N.A.	0
STRBP	-0,258954735	-0,264046872	0	N.A.	0
ANAPC1	-0,259010316	-0,164630512	0	N.A.	0
NFIA	-0,259049187	-0,17781819	0	N.A.	0
THEM4	-0,259091771	-0,31809007	0	N.A.	0
DCTN2	-0,25915196	0,074290893	0	N.A.	0
DEF8	-0,259177664	-0,036698541	0	N.A.	0
ERLIN1	-0,259187654	0,054578356	0	N.A.	0
RINT1	-0,259232809	0,062277696	0	N.A.	0
TST	-0,2594804	0,059565863	0	N.A.	0
HOXB-AS3	-0,25951099	0,236855075	0	N.A.	0
RMDN2	-0,259596377	-0,15056322	0	N.A.	0
POLR1B	-0,259711752	-0,13421615	0	N.A.	0
DNASE2	-0,259724939	0,172715263	0	N.A.	0
FDFT1	-0,259766722	0,049282594	0	N.A.	0
IGFBP4	-0,25982091	0,134439982	0	N.A.	0
TUBGCP4	-0,259884345	-0,032994504	0	N.A.	0

CCDC112	-0,260278908	0,147518537	0	N.A.	0
FCF1	-0,260456001	-0,330531791	0	N.A.	0
CCDC18	-0,260597322	-0,239844562	0	N.A.	0
AK3	-0,26074102	-0,092853612	0	N.A.	0
GSTM3	-0,261024098	-0,357214358	0	N.A.	0
RAB15	-0,261211246	-0,321789084	0	N.A.	0
EAPP	-0,261262631	-0,098608328	0	N.A.	0
PLAA	-0,261293482	-0,375005295	0	N.A.	0
IARS	-0,2613094	-0,045955364	0	N.A.	0
NUP43	-0,261490358	-0,197032443	0	N.A.	0
STEAP3	-0,261566184	0,157853533	0	N.A.	0
ALDH1A1	-0,261614676	-0,141998245	0	N.A.	0
UMODL1	-0,261723261	-0,227479349	0	N.A.	0
SLC18B1	-0,261792943	0,096239954	0	N.A.	0
RP11-172H24	-0,261949989	-0,30180611	0	N.A.	0
NGDN	-0,262039723	-0,055882366	0	N.A.	0
UBL4A	-0,26207433	0,083053331	0	N.A.	0
ZNF273	-0,262166149	-0,38917745	0	N.A.	0
CHTOP	-0,262259712	-0,063255205	0	N.A.	0
ELOVL6	-0,262318458	-0,046816964	0	N.A.	0
CMTM8	-0,262589386	0,083960276	0	N.A.	0
COX16	-0,26263428	-0,265318518	0	N.A.	0
EIF4E2	-0,262643077	0,029513769	0	N.A.	0
CCDC43	-0,262714195	-0,158491531	0	N.A.	0
PVT1	-0,262892948	0,041044783	0	N.A.	0
MTPAP	-0,262915454	0,127158774	0	N.A.	0
CSPP1	-0,263120996	-0,096026888	0	N.A.	0
PRAC2	-0,263160146	-0,264459857	0	N.A.	0
C16orf87	-0,263210842	-0,212338722	0	N.A.	0
MID1IP1	-0,263223981	-0,124914279	1	none	0
PIGK	-0,263268109	0,059684041	0	N.A.	0
PTGFRN	-0,263304216	-0,0539295	0	N.A.	0
SNX1	-0,263310288	-0,107859047	0	N.A.	0

THAP10	-0,263388159	-0,235655393	0	N.A.	0
ABHD10	-0,263388948	-0,341305451	0	N.A.	0
TSEN54	-0,263754793	0,176334147	0	N.A.	0
MOB4	-0,263774725	-0,020001208	0	N.A.	0
CNTLN	-0,263775477	-0,22938519	0	N.A.	0
FAM60A	-0,263797818	0,109369619	0	N.A.	0
UCP2	-0,263864441	-0,55611679	0	N.A.	0
PTGR1	-0,26396483	-0,039683748	0	N.A.	0
OGG1	-0,264024037	0,107646107	0	N.A.	0
HDGF	-0,264079884	0,226285576	0	N.A.	0
ZDHHC16	-0,264177236	-0,153830162	0	N.A.	0
CDC25B	-0,264359051	-0,021864267	0	N.A.	0
MON1A	-0,264380612	-0,01512962	0	N.A.	0
CLUAP1	-0,264450097	-0,208943868	0	N.A.	0
EIF4G1	-0,264668552	0,236105872	0	N.A.	0
ENOX2	-0,264729559	-0,379384826	0	N.A.	0
DEGS2	-0,26477538	0,275234203	0	N.A.	0
ELP5	-0,264891538	-0,452701072	0	N.A.	0
ID2	-0,264910221	-0,21297408	0	N.A.	0
ATF7IP2	-0,26495243	-0,157057718	0	N.A.	0
PITX2	-0,265008257	-0,074339476	0	N.A.	0
TMEM160	-0,265035905	-0,30457893	0	N.A.	0
TADA2A	-0,265176978	-0,020194256	0	N.A.	0
SON	-0,265190697	0,516339633	0	N.A.	0
GS1-124K5.4	-0,265227703	-0,179333117	0	N.A.	0
RNH1	-0,265272352	0,164601437	0	N.A.	0
FADS1	-0,265281318	0,03073853	0	N.A.	0
DUS1L	-0,265293618	-0,008765695	0	N.A.	0
COX20	-0,265622952	-0,346313356	0	N.A.	0
KPNA3	-0,265710032	-0,153262081	0	N.A.	0
SNHG7	-0,265711973	-0,069693831	0	N.A.	0
IPO9-AS1	-0,265775295	-0,083252888	0	N.A.	0
PAIP2B	-0,265940965	-0,189797064	0	N.A.	0

ING5	-0,265992897	-0,082643185	0	N.A.	0
CPNE3	-0,266069896	-0,186611648	0	N.A.	0
RP11-108L7.1	-0,266134328	-0,25311047	0	N.A.	0
DPP10-AS1	-0,266254679	-0,259971526	0	N.A.	0
PSMA2	-0,266286259	-0,102990134	0	N.A.	0
KPTN	-0,266293341	-0,187257704	0	N.A.	0
PRDX5	-0,266365598	-0,215274104	0	N.A.	0
EFCAB2	-0,266495269	-0,397309825	0	N.A.	0
UBAC2	-0,266552093	0,180665578	0	N.A.	0
IFT74	-0,266626572	-0,520959259	0	N.A.	0
IRF8	-0,266636493	-0,162365565	0	N.A.	0
TMEM69	-0,266743459	-0,259231561	0	N.A.	0
FAM86B1	-0,266848407	0,18783233	0	N.A.	0
CBR3	-0,266855377	-0,11588124	0	N.A.	0
CENPQ	-0,267016724	-0,21116527	0	N.A.	0
MSRB1	-0,26706627	0,024699365	0	N.A.	0
SKIV2L2	-0,267146535	-0,116669066	0	N.A.	0
ZNF302	-0,26721471	0,045661288	0	N.A.	0
NSRP1	-0,26728939	0,06981736	0	N.A.	0
GABPB1	-0,267325507	-0,176996412	0	N.A.	0
PAH	-0,267443943	-0,162073398	0	N.A.	0
UNC50	-0,267560692	-0,120247204	0	N.A.	0
CBWD1	-0,267788427	-0,021081784	0	N.A.	0
INTS7	-0,26781059	-0,237823778	0	N.A.	0
SRSF6	-0,267819195	-0,088774464	0	N.A.	0
GTF3C5	-0,267860319	-0,134607569	0	N.A.	0
AC123023.1	-0,267983895	-0,263317966	0	N.A.	0
ANKRD26	-0,268041381	-0,052162538	0	N.A.	0
FANCL	-0,268069306	-0,134414578	0	N.A.	0
INTS12	-0,268277944	-0,028637039	0	N.A.	0
PEPD	-0,268394455	0,030464676	0	N.A.	0
PLP2	-0,268412913	0,023020836	0	N.A.	0
FAR2	-0,268453319	-0,211859478	0	N.A.	0

AC091729.9	-0,268793773	-0,333218744	0	N.A.	0
TPP2	-0,269086399	-0,061603206	0	N.A.	0
IGFBP2	-0,269166179	0,081722008	0	N.A.	0
DOCK11	-0,269366445	-0,138215426	0	N.A.	0
BRIP1	-0,269489096	-0,36135503	0	N.A.	0
QPRT	-0,269681697	-0,330250087	0	N.A.	0
CAMTA1	-0,269776799	-0,457763729	0	N.A.	0
CCDC53	-0,269945158	-0,239267246	0	N.A.	0
CNTRL	-0,270201012	-0,198844942	0	N.A.	0
CKMT1A	-0,270232615	-0,035266322	0	N.A.	0
TADA3	-0,270374671	-0,388481415	0	N.A.	0
FAIM2	-0,270607203	-0,251268578	0	N.A.	0
HDHD2	-0,270637827	-0,246296634	0	N.A.	0
STT3A	-0,270674715	-0,187467075	0	N.A.	0
CNIH1	-0,270721675	-0,37437684	0	N.A.	0
CKMT1B	-0,270778752	0,084688132	0	N.A.	0
SMTN	-0,27082721	-0,195350094	0	N.A.	0
TBC1D4	-0,270908009	-0,071855157	0	N.A.	0
MTL5	-0,270925438	-0,321585442	0	N.A.	0
TIGD1	-0,271494424	-0,0160793	0	N.A.	0
MNX1-AS1	-0,271532117	-0,315966509	0	N.A.	0
SCML4	-0,271535	-0,377998206	0	N.A.	0
CCBL2	-0,271667046	-0,418664247	0	N.A.	0
DCAF11	-0,271690164	0,010977809	0	N.A.	0
TMEM199	-0,271796707	-0,086431087	0	N.A.	0
C21orf58	-0,271821522	-0,106087403	0	N.A.	0
GYLTL1B	-0,272056764	-0,172332453	0	N.A.	0
KEAP1	-0,272198711	0,029638772	0	N.A.	0
UCKL1	-0,272207921	0,26836747	0	N.A.	0
COIL	-0,272479322	-0,060253047	0	N.A.	0
GEMIN8	-0,272509978	-0,034927568	0	N.A.	0
GSTA4	-0,272532134	-0,254336068	0	N.A.	0
RNF20	-0,272572454	-0,305379696	0	N.A.	0

BRD8	-0,272618996	-0,145162428	0	N.A.	0
PPAP2C	-0,272631426	0,485032051	0	N.A.	0
CCNF	-0,272700547	-0,251678474	0	N.A.	0
TUBGCP3	-0,272772778	-0,232834328	0	N.A.	0
UBR7	-0,272877285	-0,168704104	0	N.A.	0
ABT1	-0,273107054	-0,432452536	0	N.A.	0
ANAPC10	-0,273144987	-0,210614999	0	N.A.	0
ZNF296	-0,273145553	-0,370521983	0	N.A.	0
TRPM2	-0,273161653	-0,186752513	0	N.A.	0
AGT	-0,273191594	-0,050430096	0	N.A.	0
PTCD2	-0,273203053	-0,195646548	0	N.A.	0
PPP1R14B	-0,27322485	0,092500644	0	N.A.	0
ACTR6	-0,273424771	-0,302395365	0	N.A.	0
MTERF2	-0,273427944	-0,481900624	0	N.A.	0
AGTRAP	-0,273573355	0,196858418	0	N.A.	0
TRAPPC3	-0,273736817	-0,094995076	0	N.A.	0
ZC3HC1	-0,273758729	-0,394458243	0	N.A.	0
KBTBD6	-0,273923354	-0,205197451	0	N.A.	0
NUDT3	-0,273978897	-0,114220526	0	N.A.	0
PGM2	-0,274181228	-0,278127046	0	N.A.	0
PNPLA4	-0,274324131	-0,188550047	0	N.A.	0
STK25	-0,274449463	0,407774995	0	N.A.	0
PRPF3	-0,274456949	0,050220068	0	N.A.	0
RASL11A	-0,274529507	-0,304066215	0	N.A.	0
MED27	-0,274565639	-0,285074215	0	N.A.	0
BOLA1	-0,274815759	-0,33279745	0	N.A.	0
TGFBI	-0,274847439	-0,065243806	0	N.A.	0
GJB1	-0,274918071	-0,081273015	0	N.A.	0
HSPB6	-0,275008101	-0,169330139	0	N.A.	0
PFAS	-0,275096881	-0,252802702	0	N.A.	0
PIDD1	-0,275210386	0,066454897	0	N.A.	0
POLR3B	-0,275253788	-0,227447828	0	N.A.	0
PTBP1	-0,27531332	-0,141240361	0	N.A.	0

AAGAB	-0,275364523	0,112410965	0	N.A.	0
HNRNPLL	-0,275461034	-0,142701894	0	N.A.	0
NME6	-0,275546696	-0,231045039	0	N.A.	0
EPHX2	-0,275562378	-0,042415245	0	N.A.	0
DRAP1	-0,275703327	-0,240816441	0	N.A.	0
SRP19	-0,275711313	0,192348476	0	N.A.	0
PRKAR1B	-0,275723263	-0,19098657	0	N.A.	0
ZCCHC17	-0,275746916	-0,097023177	0	N.A.	0
C12orf73	-0,275754607	-0,154772033	0	N.A.	0
KLHDC4	-0,275755577	-0,161793141	0	N.A.	0
ARL6IP6	-0,27583644	0,112151941	0	N.A.	0
WRB	-0,275885329	-0,1727494	0	N.A.	0
C19orf40	-0,275886979	-0,177842395	0	N.A.	0
TP53	-0,27600886	-0,303219409	0	N.A.	0
TMEM147	-0,276046908	-0,16643131	0	N.A.	0
EIF3B	-0,276110679	0,236402173	0	N.A.	0
TRIM59	-0,276480071	-0,415899697	0	N.A.	0
HSD17B7	-0,276738962	0,180700895	0	N.A.	0
IER3IP1	-0,276809952	-0,107229597	0	N.A.	0
ERCC8	-0,276841227	-0,557881001	0	N.A.	0
NUP188	-0,276964725	0,111875045	0	N.A.	0
FAP	-0,277087413	-0,130659582	0	N.A.	0
C11orf84	-0,277473156	-0,064647564	0	N.A.	0
ARL17A	-0,277512133	0,044910221	0	N.A.	0
COQ6	-0,277789215	-0,322384352	0	N.A.	0
GLRX2	-0,277889456	0,191840925	0	N.A.	0
WNK2	-0,27790676	-0,266810557	0	N.A.	0
GTPBP3	-0,278010425	-0,043306031	0	N.A.	0
ELP4	-0,278113014	-0,131268705	0	N.A.	0
ZFAND1	-0,278277194	-0,216376708	0	N.A.	0
THOC5	-0,278429288	-0,462863272	0	N.A.	0
IMPA2	-0,278472331	-0,044109229	0	N.A.	0
SBSPON	-0,27857778	-0,087432911	0	N.A.	0

OGFOD3	-0,278605558	0,013651838	0	N.A.	0
KLHL12	-0,278608884	0,001585882	0	N.A.	0
SMYD3	-0,278748188	-0,299546703	0	N.A.	0
TTC8	-0,278859836	0,038552197	0	N.A.	0
GNPAT	-0,278929909	-3,01248E-05	0	N.A.	0
CFAP97	-0,278932647	-0,22488743	0	N.A.	0
ADAT2	-0,279177959	-0,128036746	0	N.A.	0
FGGY	-0,279223845	-0,244551759	0	N.A.	0
ATF6B	-0,279371161	0,010442034	0	N.A.	0
MPP1	-0,279456469	-0,07034938	0	N.A.	0
C14orf1	-0,279467735	0,138103409	0	N.A.	0
ARMC2	-0,279610658	-0,180062555	0	N.A.	0
TYW1	-0,279655992	0,2302509	0	N.A.	0
SUMF2	-0,279698458	-0,148520307	0	N.A.	0
METAP1D	-0,27987654	0,002240667	0	N.A.	0
CHMP4A	-0,279949223	0,169564725	0	N.A.	0
OAZ2	-0,279954181	-0,104808711	0	N.A.	0
DERA	-0,280042372	-0,147861546	0	N.A.	0
TAF5	-0,280132303	-0,135777321	0	N.A.	0
MTO1	-0,280219845	-0,263543063	0	N.A.	0
APEX2	-0,280222442	0,024193345	0	N.A.	0
GPX1	-0,280373562	-0,24469828	0	N.A.	0
HOXA11	-0,280457823	-0,091636142	0	N.A.	0
SLC19A1	-0,280480892	0,041388027	0	N.A.	0
COMMD5	-0,280594498	-0,066215921	0	N.A.	0
MT1G	-0,280702303	-0,254695544	0	N.A.	0
DZIP3	-0,280791945	-0,189170499	0	N.A.	0
CCNE2	-0,280819077	-0,125303097	0	N.A.	0
MASTL	-0,281004795	0,074426955	0	N.A.	0
DMC1	-0,281007416	-0,20921984	0	N.A.	0
HSPH1	-0,281083215	0,140922463	0	N.A.	0
RNF113A	-0,281259842	-0,219133795	0	N.A.	0
RRP7A	-0,2813448	-0,2417115	0	N.A.	0

NCAPH2	-0,281353172	-0,170936466	0	N.A.	0
DHPS	-0,281758033	0,234757978	0	N.A.	0
NOSIP	-0,281777871	-0,202824447	0	N.A.	0
PRRT3-AS1	-0,281781369	-0,308440162	0	N.A.	0
LSM14A	-0,281811288	-0,36354345	0	N.A.	0
COASY	-0,281852805	0,170694155	0	N.A.	0
NOM1	-0,281890498	-0,022190037	0	N.A.	0
PRPF6	-0,282193583	0,189253537	0	N.A.	0
NUP155	-0,282254538	-0,159294521	0	N.A.	0
ZBTB8OS	-0,282289043	-0,182091346	0	N.A.	0
NDRG3	-0,282349588	-0,126553718	0	N.A.	0
TPD52L1	-0,282504982	-0,168319132	0	N.A.	0
KIF21A	-0,282511952	0,428828466	0	N.A.	0
SMAGP	-0,2826688	0,285733732	0	N.A.	0
POLE4	-0,282932226	-0,325406927	0	N.A.	0
PCGF6	-0,283096056	-0,289649816	0	N.A.	0
CNOT7	-0,283175816	-0,247089906	0	N.A.	0
ACADSB	-0,283402906	0,062061808	0	N.A.	0
QTRTD1	-0,283508259	-0,158241692	0	N.A.	0
KDELR1	-0,283520217	0,09572767	0	N.A.	0
TICRR	-0,283556448	-0,184284341	0	N.A.	0
TFDP2	-0,283751821	-0,256568458	0	N.A.	0
HCFC1	-0,283934704	0,035623904	0	N.A.	0
PPOX	-0,284090659	-0,184196716	0	N.A.	0
PREB	-0,284924907	-0,015973577	0	N.A.	0
NSMCE2	-0,285214719	-0,182683402	0	N.A.	0
ANKRD46	-0,285251378	-0,102707929	0	N.A.	0
ASMTL	-0,285291829	0,181084038	0	N.A.	0
WDR36	-0,285325933	0,069809142	0	N.A.	0
CSK	-0,28554845	-0,285589508	0	N.A.	0
LZTFL1	-0,285630535	-0,24283842	0	N.A.	0
LSM10	-0,285657561	-0,215596479	0	N.A.	0
C8orf33	-0,285685387	-0,081627037	0	N.A.	0

MED24	-0,285788032	-0,049089948	0	N.A.	0
ZNF572	-0,285855448	-0,01834351	0	N.A.	0
PIGW	-0,285897768	-0,392820619	0	N.A.	0
RBMXL1	-0,285909149	-0,117507712	0	N.A.	0
VWA9	-0,286198304	-0,145256884	0	N.A.	0
CCDC94	-0,286301932	-0,067728984	0	N.A.	0
SPATA33	-0,286375006	-0,057022847	0	N.A.	0
FAM81A	-0,286437753	-0,215780337	0	N.A.	0
TMEM209	-0,286465152	-0,038696638	0	N.A.	0
GALM	-0,286804437	-0,027349931	0	N.A.	0
HIST1H3G	-0,286932334	-0,217148938	0	N.A.	0
ACSL6	-0,286948274	-0,26861901	0	N.A.	0
DNAAF2	-0,287033957	-0,213826278	0	N.A.	0
CCDC47	-0,287259517	0,089061526	0	N.A.	0
ARPC4	-0,287413184	-0,069879525	0	N.A.	0
POC5	-0,287433223	0,092416627	0	N.A.	0
UTP20	-0,287598976	-0,245620016	0	N.A.	0
GNB1L	-0,28767843	-0,212796685	0	N.A.	0
RP11-214N9.	-0,287691716	0,031535547	0	N.A.	0
SWI5	-0,287745789	-0,096286638	0	N.A.	0
RP13-582O9.	-0,287832265	-0,145530548	0	N.A.	0
STIL	-0,288131916	-0,261735747	0	N.A.	0
DHRS4-AS1	-0,288168056	-0,404273363	0	N.A.	0
BRE	-0,288171189	-0,049399793	0	N.A.	0
UBAP2L	-0,288729297	-0,068857295	0	N.A.	0
DNAJC11	-0,288771851	-0,02978871	0	N.A.	0
ZNF232	-0,288875152	-0,291955537	0	N.A.	0
PDK3	-0,288877689	-0,138606873	0	N.A.	0
HSF2	-0,288908265	-0,324237148	0	N.A.	0
TMX1	-0,288963971	-0,180296051	0	N.A.	0
RP11-132A1.	-0,28896821	-0,287150002	0	N.A.	0
HSBP1	-0,288987988	-0,351136759	0	N.A.	0
CDK5	-0,289010676	-0,163686971	0	N.A.	0

UQCR11	-0,289059287	-0,285167284	0	N.A.	0
NUBP1	-0,289193155	-0,015638405	0	N.A.	0
EIF2AK4	-0,28920345	0,200098084	0	N.A.	0
ACACA	-0,289265704	0,070915265	0	N.A.	0
SGTA	-0,289437038	-0,252611882	0	N.A.	0
THOC1	-0,28946002	-0,065135382	0	N.A.	0
TRIM65	-0,289609222	0,219680572	0	N.A.	0
NUDT8	-0,289853763	-0,021900342	0	N.A.	0
RP5-908M14	-0,289975221	-0,028582828	0	N.A.	0
PEF1	-0,290040505	-0,08600896	0	N.A.	0
ACAA2	-0,290455766	-0,091246348	0	N.A.	0
PIGT	-0,290461499	0,063928097	0	N.A.	0
RP11-785H5.	-0,290498679	0,022970506	0	N.A.	0
AGPS	-0,290539183	-0,263086323	0	N.A.	0
FBXO9	-0,290583544	-0,218957706	0	N.A.	0
RP11-386G1	-0,290605295	-0,223135863	0	N.A.	0
GPALPP1	-0,29080869	-0,182943953	0	N.A.	0
ME2	-0,290858507	-0,150395577	0	N.A.	0
KDELC1	-0,291165673	-0,175505095	0	N.A.	0
GLS2	-0,291474247	-0,292537143	0	N.A.	0
TRAPPC4	-0,291682459	-0,16382925	0	N.A.	0
C9orf40	-0,291745033	-0,089217991	0	N.A.	0
TXNDC17	-0,292142507	-0,15196816	0	N.A.	0
ACADM	-0,292159784	-0,333990117	0	N.A.	0
USP10	-0,292164075	-0,120171261	0	N.A.	0
RSBN1L	-0,292408864	-0,012796865	0	N.A.	0
TBCE	-0,29244944	0,21151809	0	N.A.	0
ZDHHC4	-0,292604509	-0,222780075	0	N.A.	0
COPS7A	-0,292644492	-0,070394814	0	N.A.	0
TCEAL1	-0,29291911	-0,361997896	0	N.A.	0
TRUB1	-0,293094091	-0,412186577	0	N.A.	0
PHAX	-0,293196547	0,013829896	0	N.A.	0
NSUN2	-0,293335048	-0,102897855	0	N.A.	0

CDC23	-0,293400328	-0,133106423	0	N.A.	0
GTPBP8	-0,293690945	-0,167074214	0	N.A.	0
HN1L	-0,29374424	0,07499602	0	N.A.	0
RAD23B	-0,294004112	0,047939762	0	N.A.	0
CDK5RAP2	-0,294325389	-0,077162629	0	N.A.	0
PRPF4B	-0,294403769	0,03612494	0	N.A.	0
THUMPD2	-0,294441502	0,081997036	0	N.A.	0
PLCB2	-0,294739037	-0,228399727	0	N.A.	0
AC009005.2	-0,294768049	-0,362827659	0	N.A.	0
FAM104A	-0,295163897	-0,036349777	0	N.A.	0
XYLB	-0,295219418	-0,211331449	0	N.A.	0
MANBAL	-0,295439505	0,009552565	0	N.A.	0
ZW10	-0,295525576	-0,224822665	0	N.A.	0
KIAA1143	-0,295589044	-0,168022453	0	N.A.	0
TARBP2	-0,296052245	-0,430467614	0	N.A.	0
GSDMD	-0,296070969	0,155039962	0	N.A.	0
TRAPPC6A	-0,296122809	-0,417512247	0	N.A.	0
SDHAF1	-0,296279967	-0,001242344	0	N.A.	0
MCCC1	-0,296343027	-0,297815996	0	N.A.	0
CSTF1	-0,296511007	-0,017007043	0	N.A.	0
MTA1	-0,296936081	0,146377485	0	N.A.	0
NAF1	-0,297067714	-0,385042676	0	N.A.	0
SND1	-0,29709998	-0,003317337	0	N.A.	0
DNAJA1	-0,297145712	-0,202014433	0	N.A.	0
EIF2AK1	-0,297236332	-0,09672077	0	N.A.	0
TINAG	-0,297463329	-0,359905496	0	N.A.	0
HTATIP2	-0,297502049	0,126945463	0	N.A.	0
CBR4	-0,297668426	-0,049703123	0	N.A.	0
SSU72	-0,297726875	-0,089215217	0	N.A.	0
PGAM1	-0,297956637	-0,183926169	0	N.A.	0
YWHAH	-0,298028989	-0,068337262	0	N.A.	0
GAS6	-0,298221401	0,138258868	1	none	0
ATG4C	-0,298415017	-0,233277907	0	N.A.	0

UBE2D2	-0,298456353	-0,270917148	0	N.A.	0
RAB4A	-0,298508179	-0,349668999	0	N.A.	0
CDKN2C	-0,29870276	-0,358164334	0	N.A.	0
MLH1	-0,29896247	-0,289359433	0	N.A.	0
AP3M2	-0,298967647	0,105554609	0	N.A.	0
TMEM52	-0,298983071	-0,003908733	0	N.A.	0
DYNC2LI1	-0,299082423	-0,15401563	0	N.A.	0
NNT-AS1	-0,299103711	-0,17335325	0	N.A.	0
LARS	-0,299114067	-0,328651501	0	N.A.	0
HNRNPH2	-0,299165854	-0,126264382	0	N.A.	0
ZNHIT6	-0,29918888	-0,158098809	0	N.A.	0
MIF	-0,299307491	-0,336589981	0	N.A.	0
OSGEPL1	-0,29941393	-0,064212388	0	N.A.	0
NUP88	-0,299447087	-0,199931128	0	N.A.	0
DAK	-0,29973919	0,023947574	0	N.A.	0
METTL23	-0,299780876	-0,298823183	0	N.A.	0
CCNG1	-0,299793085	-0,590163168	0	N.A.	0
COPS7B	-0,300019604	-0,174335923	0	N.A.	0
HULC	-0,300106118		0	N.A.	0
BIVM	-0,300153788	-0,121746517	0	N.A.	0
PTRH1	-0,300205004	0,222839682	0	N.A.	0
PUSL1	-0,300331635	0,041384906	0	N.A.	0
GPD1	-0,300352735	-0,137733041	0	N.A.	0
HSD17B12	-0,300666349	0,057762364	0	N.A.	0
TTI1	-0,300904733	-0,23519989	0	N.A.	0
INIP	-0,301267651	-0,220848192	0	N.A.	0
POP1	-0,30151846	-0,100620872	0	N.A.	0
C1orf86	-0,3018158	-0,376203809	0	N.A.	0
C1orf53	-0,302129619	-0,256359442	0	N.A.	0
AFG3L2	-0,30220946	0,000601204	0	N.A.	0
NUP133	-0,302226074	-0,101417248	0	N.A.	0
TTC38	-0,302293336	0,114942199	0	N.A.	0
SURF2	-0,302369054	-0,35618328	0	N.A.	0

C1orf109	-0,302430825	-0,374190096	0	N.A.	0
GEMIN7	-0,302501442	0,078869701	0	N.A.	0
NDUFAF3	-0,302546472	-0,369199093	0	N.A.	0
PCBD2	-0,302562241	-0,139751814	0	N.A.	0
PRPF38A	-0,302691768	-0,26689335	0	N.A.	0
VAPB	-0,302728679	-0,353973253	0	N.A.	0
TMEM38B	-0,302764642	-0,085130965	0	N.A.	0
DGAT2	-0,302788011	0,031415481	0	N.A.	0
ARL14EP	-0,302804431	-0,092809485	0	N.A.	0
MANF	-0,302893084	0,067864596	0	N.A.	0
METTL7B	-0,302901343	0,158692174	0	N.A.	0
FANCF	-0,302918208	-0,109259079	0	N.A.	0
MPV17L	-0,303156418	-0,075303976	0	N.A.	0
HEATR1	-0,303442805	-0,088576623	0	N.A.	0
RP11-474D1.	-0,303601908	-0,220312438	0	N.A.	0
TCTN1	-0,303688248	-0,306731389	0	N.A.	0
GEN1	-0,303749184	-0,108149239	0	N.A.	0
METTL17	-0,303760638	0,107539597	0	N.A.	0
RPUSD1	-0,30380614	-0,157811843	0	N.A.	0
AGPAT5	-0,303865053	-0,11711006	0	N.A.	0
RAB13	-0,304041889	-0,277227685	0	N.A.	0
COQ7	-0,304221661	0,063449238	0	N.A.	0
PNP	-0,304373722	0,116855691	0	N.A.	0
RFC1	-0,304539601	-0,106440491	0	N.A.	0
POLR3F	-0,304740491	-0,165263516	0	N.A.	0
VPS72	-0,304863157	0,004506557	0	N.A.	0
EIF2B2	-0,305057733	-0,087750398	0	N.A.	0
RAD50	-0,305113266	0,027879258	0	N.A.	0
FAHD2B	-0,305243601	-0,305040498	0	N.A.	0
C19orf52	-0,305270343	0,006283853	0	N.A.	0
INO80E	-0,305389847	-0,140847158	0	N.A.	0
HDGFRP2	-0,305397793	-0,025641038	0	N.A.	0
LYRM2	-0,305544895	-0,142734488	0	N.A.	0

CBWD2	-0,305714222	-0,077839182	0	N.A.	0
PTOV1	-0,306073267	0,357797003	0	N.A.	0
MPHOSPH8	-0,306409618	0,286702225	0	N.A.	0
C6orf211	-0,306478972	-0,125450965	0	N.A.	0
CRNDE	-0,306515031	-0,188617163	0	N.A.	0
AIFM3	-0,306659693	-0,218459744	0	N.A.	0
UTP3	-0,306764896	0,016190646	0	N.A.	0
PROSC	-0,30689463	-0,111888123	0	N.A.	0
CNOT10	-0,30716217	0,085107019	0	N.A.	0
CBY1	-0,307522982	-0,28036989	0	N.A.	0
EME1	-0,307537826	-0,20394589	0	N.A.	0
TRABD	-0,308621229	0,235581063	0	N.A.	0
TXNRD2	-0,308848476	-0,229186069	0	N.A.	0
EEFSEC	-0,309307506	-0,148719254	0	N.A.	0
RP5-908M14	-0,309307825	-0,210787403	0	N.A.	0
EIF2A	-0,309513944	-0,434945235	0	N.A.	0
FKBP5	-0,309660679	-0,519229729	0	N.A.	0
NOP2	-0,309910161	-0,117943711	0	N.A.	0
ZRANB2	-0,309926326	0,082472026	0	N.A.	0
SMKR1	-0,309965435	-0,344514066	0	N.A.	0
MAPRE1	-0,310408389	-0,203255546	0	N.A.	0
AKR7A3	-0,310414747	0,172018119	0	N.A.	0
PPIL3	-0,310429116	-0,213788995	0	N.A.	0
PEX3	-0,310449225	-0,279696194	0	N.A.	0
LY6E	-0,31056515	-0,144140877	0	N.A.	0
RP11-244M2	-0,310699349	-0,369171949	0	N.A.	0
SLC27A2	-0,310817186	-0,129536345	0	N.A.	0
CS	-0,310845174	0,087885876	0	N.A.	0
TBL3	-0,310921247	-0,231807553	0	N.A.	0
CTNNAL1	-0,311019312	-0,173549868	0	N.A.	0
SPA17	-0,311205811	-0,361807763	0	N.A.	0
HCCS	-0,311215571	-0,283099528	0	N.A.	0
CPVL	-0,311387946	-0,07817999	0	N.A.	0

PDZD11	-0,311393043	-0,189025101	0	N.A.	0
PHF6	-0,31155809	-0,223065316	0	N.A.	0
SF3A2	-0,311735618	0,142318067	0	N.A.	0
CHPT1	-0,311859483	0,168711669	0	N.A.	0
SMOC2	-0,312127845	-0,190956572	0	N.A.	0
FGFR4	-0,312245847	0,07127615	0	N.A.	0
BLM	-0,312297689	-0,311260877	0	N.A.	0
RNMTL1	-0,312570349	-0,058225998	0	N.A.	0
L2HGDH	-0,312825034	-0,271577179	0	N.A.	0
DNAH6	-0,312876675	-0,296580906	0	N.A.	0
HAUS8	-0,312973086	-0,175937921	0	N.A.	0
SLC25A17	-0,313003655	-0,113113508	0	N.A.	0
CFL1	-0,313130453	-0,09426089	0	N.A.	0
HMG20B	-0,313294276	-0,217693196	0	N.A.	0
C19orf43	-0,31361905	-0,12982909	0	N.A.	0
FDXR	-0,313744753	-0,207903963	0	N.A.	0
NPRL2	-0,314138596	-0,098095208	0	N.A.	0
KATNA1	-0,314303136	-0,154365144	0	N.A.	0
CAAP1	-0,314356148	-0,259187655	0	N.A.	0
NDUFAF1	-0,314548209	-0,171396521	0	N.A.	0
RPN2	-0,314715049	0,013144097	0	N.A.	0
BCL11A	-0,314761655	-0,290300075	0	N.A.	0
MYBBP1A	-0,314792738	-0,167518494	0	N.A.	0
C22orf39	-0,314834558	-0,252039374	0	N.A.	0
MITD1	-0,31486711	-0,029498691	0	N.A.	0
VPS4A	-0,314899352	0,090018219	0	N.A.	0
NDUFAF7	-0,314914632	-0,04091618	0	N.A.	0
FIGNL1	-0,315006402	-0,303012355	0	N.A.	0
STK32C	-0,315130183	-0,289150843	0	N.A.	0
PHPT1	-0,315572589	-0,262080854	0	N.A.	0
EIF6	-0,315622914	-0,050906353	0	N.A.	0
DNA2	-0,315845076	-0,044794185	0	N.A.	0
KHSRP	-0,315917512	0,049693038	0	N.A.	0

EBAG9	-0,316123122	0,019310698	0	N.A.	0
GLMN	-0,316164102	-0,15159539	0	N.A.	0
EIF1AY	-0,316183656	-0,274764159	0	N.A.	0
MAPKAPK5	-0,316681213	-0,185226189	0	N.A.	0
PPP2R5D	-0,316699151	-0,264144551	0	N.A.	0
STARD10	-0,316702164	-0,165268866	0	N.A.	0
LYPLAL1	-0,316792562	-0,241144907	0	N.A.	0
TIMM21	-0,316885742	-0,249521726	0	N.A.	0
CLPB	-0,317054362	-0,28224407	0	N.A.	0
TBC1D31	-0,317101603	-0,271453324	0	N.A.	0
ERCC6L	-0,317302977	-0,14552061	0	N.A.	0
GLE1	-0,317403609	-0,324930851	0	N.A.	0
PLEKHB1	-0,317706317	-0,414237899	0	N.A.	0
NOL12	-0,317720852	-0,141406041	0	N.A.	0
TDP1	-0,317831725	-0,161856841	0	N.A.	0
C7orf73	-0,317951486	-0,38608991	0	N.A.	0
C19orf60	-0,318003008	-0,167500223	0	N.A.	0
IAH1	-0,31802104	-0,169768225	0	N.A.	0
TRMT1	-0,318103054	-0,065702462	0	N.A.	0
CCDC66	-0,318327249	0,163453608	0	N.A.	0
ATOX1	-0,318330968	-0,511057992	0	N.A.	0
KAT8	-0,318746977	-0,198328389	0	N.A.	0
FBXO4	-0,318752543	-0,186118189	0	N.A.	0
SDCCAG3	-0,318800417	-0,02619933	0	N.A.	0
ACTL8	-0,318837346		0	N.A.	0
DCAF4	-0,318896974	-0,12140132	0	N.A.	0
BTF3L4	-0,319050418	-0,25616149	0	N.A.	0
TPR	-0,319275005	0,068905027	0	N.A.	0
UBL7	-0,319369509	-0,141814768	0	N.A.	0
MESDC2	-0,319382048	0,099510618	0	N.A.	0
TCEAL8	-0,319412248	-0,302262024	0	N.A.	0
MAK16	-0,319437896	-0,26837112	0	N.A.	0
PFDN5	-0,319438919	-0,400679685	0	N.A.	0

OSGEPL1-AS1	-0,319471939	0,098468111	0	N.A.	0
ZC3H13	-0,319585014	0,033518588	0	N.A.	0
HIBADH	-0,319592681	-0,163256234	0	N.A.	0
PSME3	-0,320297661	0,00387381	0	N.A.	0
CENPJ	-0,320312341	-0,253717135	0	N.A.	0
SPATS2	-0,321510194	-0,13714803	0	N.A.	0
DIS3L	-0,321715516	-0,252421101	0	N.A.	0
ZNF428	-0,321914036	-0,249540489	0	N.A.	0
TMEM11	-0,321960172	-0,215485153	0	N.A.	0
NUP205	-0,322056847	-0,015898555	0	N.A.	0
TAF1A	-0,322081777	-0,219918497	0	N.A.	0
GLA	-0,322239271	-0,3020371	0	N.A.	0
SNHG15	-0,322425669	-0,0991778	0	N.A.	0
GCAT	-0,322471453	-0,434208594	0	N.A.	0
PSMC6	-0,322474538	-0,20118069	0	N.A.	0
MNAT1	-0,322643343	-0,148059024	0	N.A.	0
RPP25	-0,322655763	-0,004696713	0	N.A.	0
MED4	-0,322733832	-0,361053711	0	N.A.	0
BUD13	-0,322870741	-0,170456129	0	N.A.	0
FAM96B	-0,322963506	-0,351464798	0	N.A.	0
HSD17B8	-0,323410365	-0,217922484	0	N.A.	0
SLC39A3	-0,323701576	-0,084508074	0	N.A.	0
EIF4B	-0,323822288	-0,459796537	0	N.A.	0
SLC35G1	-0,323823142	-0,153718019	0	N.A.	0
PPME1	-0,323953804	0,087041739	0	N.A.	0
YAE1D1	-0,324015586	-0,192709811	0	N.A.	0
WDR33	-0,324034901	-0,148459498	0	N.A.	0
CDKN1B	-0,324058134	-0,228660401	0	N.A.	0
COMMD10	-0,324088351	-0,558463087	0	N.A.	0
GAPDH	-0,324296261	-0,275150248	0	N.A.	0
VPS25	-0,324403137	0,074967395	0	N.A.	0
ISY1	-0,324818877	0,022426729	0	N.A.	0
TCEB1	-0,324849511	-0,153509221	0	N.A.	0

RPUSD2	-0,324883746	-0,084524018	0	N.A.	0
TTF2	-0,324900503	-0,201373467	0	N.A.	0
TAF9B	-0,325274382	-0,166985003	0	N.A.	0
FARS2	-0,325324205	-0,231937611	0	N.A.	0
CGREF1	-0,325367682	0,00101514	0	N.A.	0
WDR54	-0,325465704	-0,092064396	0	N.A.	0
DPEP1	-0,325698565	-0,036760743	0	N.A.	0
RP11-128M1	-0,325737088	-0,26934535	0	N.A.	0
SBNO1	-0,325752727	0,058545616	0	N.A.	0
TUBB4B	-0,325899143	-0,095706492	0	N.A.	0
PCBP2	-0,325980398	-0,410054763	0	N.A.	0
XRCC3	-0,32599917	-0,19344166	0	N.A.	0
DUSP23	-0,326062068	-0,127231134	0	N.A.	0
UBTF	-0,326079069	0,060111125	0	N.A.	0
TMEM129	-0,326200413	-0,060711923	0	N.A.	0
SLC25A32	-0,326293433	0,109442706	0	N.A.	0
ANKRD32	-0,326359154	-0,190826428	0	N.A.	0
SNX27	-0,326617741	0,390558847	0	N.A.	0
PDCD11	-0,327084703	-0,12133456	0	N.A.	0
CEP83	-0,327140702	-0,112415382	0	N.A.	0
NFIC	-0,32734616	-0,369785467	0	N.A.	0
CRNKL1	-0,327563019	-0,063951312	0	N.A.	0
DDB1	-0,327626551	0,109818562	0	N.A.	0
PCNP	-0,327639316	-0,45544379	0	N.A.	0
HOXA10	-0,327671541	-0,068487543	0	N.A.	0
VPS9D1-AS1	-0,327757475	-0,174962996	0	N.A.	0
ADA	-0,32778306	-0,176140555	0	N.A.	0
TSEN2	-0,327903976	0,091609445	0	N.A.	0
IL17RB	-0,328032131	-0,043385581	0	N.A.	0
RIIAD1	-0,328333558	-0,338518868	0	N.A.	0
VPS51	-0,328370176	-0,14466301	0	N.A.	0
NUP62CL	-0,328460182	-0,293915588	0	N.A.	0
PGAM5	-0,328628491	0,151874878	0	N.A.	0

CDK2	-0,328636416	-0,309989407	0	N.A.	0
COQ5	-0,328643061	-0,216573462	0	N.A.	0
PRCC	-0,328724624	0,0187828	0	N.A.	0
C16orf91	-0,328783008	-0,170483802	0	N.A.	0
WDHD1	-0,328885443	-0,26542232	0	N.A.	0
RPP38	-0,329004401	0,02616632	0	N.A.	0
GFM1	-0,329201541	0,100552458	0	N.A.	0
HOXA7	-0,329215839	-0,264135171	0	N.A.	0
CORO1A	-0,329255535	-0,356766051	0	N.A.	0
ASB9	-0,329474552	-0,31819975	0	N.A.	0
RNF8	-0,32980028	-0,175924829	0	N.A.	0
PAAF1	-0,329932718	-0,060004846	0	N.A.	0
ATP1A1	-0,330055647	0,104580216	0	N.A.	0
SCAMP3	-0,330147332	-0,21083792	0	N.A.	0
MAP9	-0,33017088	-0,211370938	0	N.A.	0
PAIP2	-0,330383454	-0,322190967	0	N.A.	0
SLC30A5	-0,330385675	-0,365782214	0	N.A.	0
FLAD1	-0,330758071	-0,101735387	0	N.A.	0
FAM192A	-0,330780805	-0,04413221	0	N.A.	0
HOXA9	-0,330786808	0,092803262	0	N.A.	0
ANKRD39	-0,330927091	-0,110276375	0	N.A.	0
SCLY	-0,330999713	-0,478884892	0	N.A.	0
CDC25A	-0,331099552	-0,21504103	0	N.A.	0
ARMC1	-0,33127368	-0,248292957	0	N.A.	0
H2AFY2	-0,3314067	-0,484130125	0	N.A.	0
WDR92	-0,33142313	-0,308440105	0	N.A.	0
HAX1	-0,331692468	-0,114551234	0	N.A.	0
NDUFV2	-0,332215428	-0,225452411	0	N.A.	0
TSR1	-0,33225984	-0,088745542	0	N.A.	0
THOC2	-0,33252432	0,159101816	0	N.A.	0
APTX	-0,332547968	-0,245433172	0	N.A.	0
FUNDC1	-0,332649011	-0,217949532	0	N.A.	0
SMARCA5	-0,333129172	-0,088430227	0	N.A.	0

SUCLA2	-0,333317218	-0,157972388	0	N.A.	0
RNF157	-0,333483128	-0,104609701	0	N.A.	0
ATP1B3	-0,333598801	0,210537267	0	N.A.	0
ANXA7	-0,333728921	-0,066192295	0	N.A.	0
RFXAP	-0,334067929	-0,366480259	0	N.A.	0
ATF1	-0,33410652	-0,284581606	0	N.A.	0
CYB561A3	-0,334503724	-0,051913422	0	N.A.	0
LINC00998	-0,334588933	-0,463938591	0	N.A.	0
LRWD1	-0,334882054	0,012396068	0	N.A.	0
XRCC1	-0,334921191	-0,121197414	0	N.A.	0
HSP90B1	-0,335115168	0,068844813	0	N.A.	0
XXYLT1	-0,335205075	0,017788598	0	N.A.	0
KIAA0586	-0,335358495	-0,082396215	0	N.A.	0
NARS	-0,335388894	-0,082902102	0	N.A.	0
LIN9	-0,335463248	-0,358440475	0	N.A.	0
RAD18	-0,335518498	-0,082912873	0	N.A.	0
PXMP4	-0,335723692	-0,087830151	0	N.A.	0
LSG1	-0,335948099	0,04454454	0	N.A.	0
SRFBP1	-0,336110039	-0,116349253	0	N.A.	0
SMPD4	-0,336180504	-0,165508581	0	N.A.	0
LINC00116	-0,336248859	-0,376336036	0	N.A.	0
GOT1	-0,336266134	-0,285803922	0	N.A.	0
TBC1D30	-0,336406605	-0,241748664	0	N.A.	0
SEH1L	-0,336478311	-0,364336046	0	N.A.	0
CHDH	-0,336535542	-0,028301907	0	N.A.	0
MYO19	-0,336668877	-0,085970603	0	N.A.	0
GPSM2	-0,336777759	-0,176551321	0	N.A.	0
AC002116.7	-0,337040188	-0,139469602	0	N.A.	0
PPFIA3	-0,337750402	-0,18502116	0	N.A.	0
SEPT11	-0,337763276	-0,075724465	0	N.A.	0
NME1-NME2	-0,338042672	-0,388817104	0	N.A.	0
PPAN	-0,338067933	-0,155368424	0	N.A.	0
SNX2	-0,338170209	-0,277115033	0	N.A.	0

CASC5	-0,338413274	-0,303847293	0	N.A.	0
SUPV3L1	-0,338862611	-0,187948356	0	N.A.	0
METTL9	-0,339265255	-0,128750203	0	N.A.	0
NDUFAF5	-0,339321113	-0,160104177	0	N.A.	0
SLC25A13	-0,339486827	-0,037458571	0	N.A.	0
MGMT	-0,339818778	-0,369183028	0	N.A.	0
NUDT9	-0,340110263	0,059872358	0	N.A.	0
USP14	-0,340130461	-0,300157739	0	N.A.	0
ELAC2	-0,340132566	-0,27397545	0	N.A.	0
ZNF593	-0,340148567	-0,240134678	0	N.A.	0
EIF5AL1	-0,340621936	-0,479891392	0	N.A.	0
TRIM27	-0,340629661	-0,117544902	0	N.A.	0
NUDT6	-0,340782378	-0,358716785	0	N.A.	0
MXD3	-0,341036176	-0,209609761	0	N.A.	0
CHD4	-0,341167103	0,090422262	0	N.A.	0
RP9	-0,341337217	-0,159614761	0	N.A.	0
MBD4	-0,34139971	-0,206468712	0	N.A.	0
INTS8	-0,341524323	0,027341513	0	N.A.	0
PDK2	-0,341755509	-0,41554706	0	N.A.	0
GYG2	-0,342046498	-0,143244606	0	N.A.	0
PEMT	-0,342235573	-0,26592387	0	N.A.	0
DDX10	-0,342442765	-0,159227292	0	N.A.	0
PET117	-0,3425081	-0,015854499	0	N.A.	0
RCCD1	-0,342616154	-0,108247104	0	N.A.	0
ALDH4A1	-0,342696338	-0,18564118	0	N.A.	0
COMMD9	-0,342731964	-0,409130252	0	N.A.	0
RP3-325F22.!	-0,342772776	-0,190554719	0	N.A.	0
CTA-29F11.1	-0,342939146	-0,103588619	0	N.A.	0
CCNI2	-0,342971981	-0,254639573	0	N.A.	0
SNAPIN	-0,343855673	-0,214235161	0	N.A.	0
TBC1D7	-0,343991843	-0,092128066	0	N.A.	0
TMEM39B	-0,343996189	0,070549878	0	N.A.	0
PQBP1	-0,344643835	0,06596604	0	N.A.	0

COA7	-0,344743552	-0,122073224	0	N.A.	0
GNG4	-0,344826508	-0,362682726	0	N.A.	0
TRMT61B	-0,344899197	-0,188113285	0	N.A.	0
ZRANB3	-0,344939683	-0,105154062	0	N.A.	0
SSSCA1	-0,345140244	-0,155399333	0	N.A.	0
MTG2	-0,345173086	-0,134197409	0	N.A.	0
WDR70	-0,345204992	-0,36840458	0	N.A.	0
HAUS6	-0,345548378	-0,182235755	0	N.A.	0
PFN2	-0,34560651	-0,309484118	0	N.A.	0
CLDN2	-0,34563956	-0,032630748	0	N.A.	0
BARD1	-0,345650207	-0,385429273	0	N.A.	0
SKP1	-0,345688495	-0,226679389	0	N.A.	0
MAD2L2	-0,345752026	-0,266602805	0	N.A.	0
CKMT2	-0,345892539	-0,426264306	0	N.A.	0
TOMM5	-0,346072858	-0,395241212	0	N.A.	0
CREB3L4	-0,346135667	-0,362273573	0	N.A.	0
MIF4GD	-0,346173943	-0,165568696	0	N.A.	0
C10orf2	-0,346558534	-0,375110376	0	N.A.	0
CDC16	-0,346849776	0,245858117	0	N.A.	0
ASPSCR1	-0,346880575	-0,271932737	0	N.A.	0
NENF	-0,346884404	-0,182133078	0	N.A.	0
PDCD7	-0,346922351	0,202037713	0	N.A.	0
KLHL23	-0,347090581	-0,090178367	0	N.A.	0
HOXB9	-0,347225732	-0,165627486	0	N.A.	0
PLA2G12A	-0,347405344	-0,285313714	0	N.A.	0
NEIL2	-0,347452593	-0,370400739	0	N.A.	0
DDX24	-0,348038736	-0,1720633	0	N.A.	0
HOOK1	-0,348043341	0,079029181	0	N.A.	0
IFT52	-0,348165976	-0,41095806	0	N.A.	0
ORC5	-0,34842203	0,054856226	0	N.A.	0
NPHP1	-0,348458406	-0,095914735	0	N.A.	0
EFCAB11	-0,348855301	-0,429646845	0	N.A.	0
SURF6	-0,348955923	0,047770523	0	N.A.	0

RTN4IP1	-0,34897148	-0,245817252	0	N.A.	0
ACD	-0,349046226	-0,128381048	0	N.A.	0
KIF18B	-0,349140468	-0,20374881	0	N.A.	0
METTL8	-0,349143403	-0,318280396	0	N.A.	0
TSNAX	-0,34924605	-0,097508274	0	N.A.	0
MAP7D2	-0,349410854	0,059257918	0	N.A.	0
RFXANK	-0,349413792	-0,24354718	0	N.A.	0
BCAS2	-0,349431256	-0,152156216	0	N.A.	0
NT5C	-0,349540826	0,181198946	0	N.A.	0
MTFR2	-0,349689099	-0,407720676	0	N.A.	0
SLC25A12	-0,349726729	0,091376919	0	N.A.	0
LEO1	-0,349821456	-0,109145884	0	N.A.	0
RNF126	-0,349838999	0,24580081	0	N.A.	0
POLQ	-0,349851597	-0,362198319	0	N.A.	0
ZNF580	-0,349939091	-0,073519408	0	N.A.	0
PABPN1	-0,350065622	0,202202458	0	N.A.	0
EHMT2	-0,350100384	-0,238347923	0	N.A.	0
RDH14	-0,350166227	-0,203911843	0	N.A.	0
MAPKAPK3	-0,350179797	-0,50763545	0	N.A.	0
SAPCD2	-0,350410827	-0,280615126	0	N.A.	0
PRKAR2A	-0,350491515	-0,109750979	0	N.A.	0
CISD3	-0,350498142	-0,053395291	0	N.A.	0
FAM161A	-0,350506912	-0,110863241	0	N.A.	0
TXNDC9	-0,350564505	-0,308071991	0	N.A.	0
CHID1	-0,350934562	-0,269727071	0	N.A.	0
SCML1	-0,350954897	-0,339396561	0	N.A.	0
CEP70	-0,351137059	-0,125326334	0	N.A.	0
DNAAF5	-0,35114029	-0,156888908	0	N.A.	0
CHAMP1	-0,351212501	-0,189122133	0	N.A.	0
GANAB	-0,351413635	0,062047532	0	N.A.	0
RPAP3	-0,351504406	-0,335490339	0	N.A.	0
CCDC167	-0,351512026	-0,283051094	0	N.A.	0
SPOP	-0,351636992	0,061817668	0	N.A.	0

TARS2	-0,351707514	0,128188732	0	N.A.	0
RAD1	-0,351783757	-0,370755762	0	N.A.	0
C10orf32	-0,351884201	-0,22447334	0	N.A.	0
JMJD8	-0,351907758	0,239052604	0	N.A.	0
FRA10AC1	-0,351925416	-0,146124066	0	N.A.	0
RMDN1	-0,352001867	-0,0761864	0	N.A.	0
MEN1	-0,352060185	0,02413296	0	N.A.	0
PSMD9	-0,352164136	-0,261105977	0	N.A.	0
TMEM161A	-0,352206716	-0,039308783	0	N.A.	0
TMCO1	-0,352408323	0,491894354	0	N.A.	0
TSPAN6	-0,352410197	-0,079102593	0	N.A.	0
HDAC8	-0,352535269	-0,103972509	0	N.A.	0
EXTL2	-0,352684492	-0,330832446	0	N.A.	0
AP003774.1	-0,35288468	-0,411552678	0	N.A.	0
UBE2E1	-0,35299209	-0,388786241	0	N.A.	0
LAMTOR1	-0,353145082	-0,149748742	0	N.A.	0
OGFOD2	-0,35342473	-0,041477022	0	N.A.	0
CHD1L	-0,353645191	-0,162708636	0	N.A.	0
FBXO5	-0,353664246	-0,278953144	0	N.A.	0
MTFMT	-0,353728412	-0,226668767	0	N.A.	0
USMG5	-0,353827089	-0,369452289	0	N.A.	0
NCBP1	-0,353919044	-0,078299559	0	N.A.	0
CUL1	-0,353965976	0,029610758	0	N.A.	0
NUDCD1	-0,354105213	-0,241244337	0	N.A.	0
CKLF	-0,354129576	-0,255617715	0	N.A.	0
DYRK4	-0,354247446	-0,389401421	0	N.A.	0
EIF4EBP1	-0,354432012	-0,296638917	0	N.A.	0
C19orf53	-0,354519984	-0,089460631	0	N.A.	0
UBE2J2	-0,35455966	0,06358021	0	N.A.	0
UTP23	-0,354813524	-0,092089958	0	N.A.	0
TRAIP	-0,354883072	-0,415604963	0	N.A.	0
ENOSF1	-0,354973069	-0,139417709	0	N.A.	0
PSMC4	-0,355299111	-0,402996618	0	N.A.	0

CCNH	-0,355544998	-0,189571363	0	N.A.	0
FAM162A	-0,35565808	-0,225376425	0	N.A.	0
C17orf75	-0,355672894	-0,151364216	0	N.A.	0
DDX50	-0,355874135	-0,257380628	0	N.A.	0
CIT	-0,355954944	-0,37018975	0	N.A.	0
ELAC1	-0,356125466	0,141681769	0	N.A.	0
ALKBH4	-0,356180195	-0,181499539	0	N.A.	0
EIF2S3	-0,356193174	-0,42620431	0	N.A.	0
EIF5	-0,356546522	-0,185008188	0	N.A.	0
MSL3	-0,35664386	-0,257898093	0	N.A.	0
TELO2	-0,356657853	-0,114290524	0	N.A.	0
SSBP4	-0,356912461	-0,016602941	0	N.A.	0
PABPC1	-0,357087297	-0,503439155	0	N.A.	0
RSL24D1	-0,357561832	-0,435562889	0	N.A.	0
MAGED1	-0,357629847	-0,13109627	0	N.A.	0
API5	-0,357739766	-0,381260807	0	N.A.	0
DCAKD	-0,358011433	-0,211330083	0	N.A.	0
CDX1	-0,358064569	0,094652007	0	N.A.	0
EEF2	-0,358229309	-0,440714491	0	N.A.	0
LINC01184	-0,358234082	-0,369864674	0	N.A.	0
CHCHD10	-0,358250823	-0,181160371	0	N.A.	0
MIR762HG	-0,358341257	-0,159433788	0	N.A.	0
CAT	-0,358559399	-0,192958883	0	N.A.	0
DCTD	-0,358648867	-0,338889963	0	N.A.	0
C4orf46	-0,358705232	-0,381135375	0	N.A.	0
PEX7	-0,358791777	-0,01955122	0	N.A.	0
MTDH	-0,35899315	0,041195972	0	N.A.	0
EIF2B4	-0,359010482	-0,018061161	0	N.A.	0
ABCB7	-0,359255156	-0,123796067	0	N.A.	0
DDX52	-0,359360843	-0,163778506	0	N.A.	0
LRPPRC	-0,359440927	-0,192810788	0	N.A.	0
TIPIN	-0,359499943	-0,512155818	0	N.A.	0
IPO9	-0,359528349	-0,22572992	0	N.A.	0

NPRL3	-0,359546724	-0,286615446	0	N.A.	0
NUDT2	-0,35972551	-0,224761376	0	N.A.	0
RNASEH1-AS	-0,359878955	-0,40843413	0	N.A.	0
L1TD1	-0,360037162	-0,28188791	0	N.A.	0
PLK4	-0,360140889	-0,337563635	0	N.A.	0
CHRNA5	-0,360280283	-0,044713636	0	N.A.	0
CDC40	-0,360360793	-0,225376976	0	N.A.	0
MDC1	-0,360951639	0,013110157	0	N.A.	0
RP11-12G12.	-0,36095879	-0,402124391	0	N.A.	0
HAUS5	-0,360983083	-0,089732126	0	N.A.	0
CAD	-0,361008339	0,020043107	0	N.A.	0
EFTUD2	-0,361051128	0,102760807	0	N.A.	0
MESP1	-0,361453377	-0,171528484	0	N.A.	0
EIF3A	-0,36151622	0,089061161	0	N.A.	0
BIN1	-0,361849579	-0,177015401	0	N.A.	0
RBFA	-0,362532802	-0,062468242	0	N.A.	0
C19orf24	-0,362676822	-0,064320789	0	N.A.	0
NDUFS1	-0,362809519	0,086347056	0	N.A.	0
ZNF326	-0,362870832	0,238961255	0	N.A.	0
FANCD2	-0,362937632	-0,241020019	0	N.A.	0
METTL10	-0,362959097	-0,216729214	0	N.A.	0
PELP1	-0,363111341	-0,091132853	0	N.A.	0
NT5C3B	-0,363161793	-0,585115214	0	N.A.	0
SNHG21	-0,363598499	-0,150336934	0	N.A.	0
TOMM7	-0,36363494	-0,529353941	0	N.A.	0
SF3B4	-0,363791692	-0,112953143	0	N.A.	0
RP11-245D16	-0,36392907	-0,354135193	0	N.A.	0
TMEM18	-0,363975534	-0,00954634	0	N.A.	0
IPO4	-0,36398088	0,134245993	0	N.A.	0
COMTD1	-0,364033788	0,083432695	0	N.A.	0
METAP1	-0,364159526	-0,185741337	0	N.A.	0
TOP1	-0,364206066	-0,013791272	0	N.A.	0
PLGRKT	-0,364559088	-0,04311471	0	N.A.	0

OXA1L	-0,364588609	-0,137859244	0	N.A.	0
RDM1	-0,364727973	-0,36991658	0	N.A.	0
DUSP12	-0,364956783	-0,194298613	0	N.A.	0
PRIM2	-0,365010375	-0,322100489	0	N.A.	0
NBN	-0,365483305	-0,292635039	0	N.A.	0
VCP	-0,365522103	-0,175729237	0	N.A.	0
EMC4	-0,365888583	-0,110335435	0	N.A.	0
TRMT61A	-0,365991069	-0,202631254	0	N.A.	0
HNRNPH1	-0,36599569	-0,152824744	0	N.A.	0
RCL1	-0,3663186	-0,089327719	0	N.A.	0
C1orf174	-0,366337158	-0,313707628	0	N.A.	0
FN3KRP	-0,366420961	-0,251701798	0	N.A.	0
C1GALT1C1	-0,36648107	-0,022529784	0	N.A.	0
BORA	-0,366737107	-0,387005202	0	N.A.	0
NDUFA7	-0,366766931	-0,046191678	0	N.A.	0
SLC29A1	-0,36677071	0,035470602	0	N.A.	0
DHODH	-0,367567631	-0,178628868	0	N.A.	0
POMP	-0,367581413	-0,311962977	0	N.A.	0
E2F8	-0,367730865	-0,444224382	0	N.A.	0
MMS22L	-0,367938604	-0,170991042	0	N.A.	0
JPX	-0,367939602	-0,277099069	0	N.A.	0
PIGU	-0,367964309	0,029862551	0	N.A.	0
SLC25A19	-0,367968502	-0,420128173	0	N.A.	0
CMC2	-0,367995107	-0,352677829	0	N.A.	0
FAM98B	-0,368059915	-0,02576156	0	N.A.	0
RP11-329L6.1	-0,368174451	-0,182749819	0	N.A.	0
KIF5B	-0,36824294	0,091308789	0	N.A.	0
ZNF771	-0,36828663	-0,209589301	0	N.A.	0
SYPL1	-0,3684598	-0,056907371	0	N.A.	0
PNKD	-0,368460626	-0,030396767	0	N.A.	0
CAMKMT	-0,368622543	-0,373066794	0	N.A.	0
SUV39H1	-0,36863017	-0,279013859	0	N.A.	0
TMEM230	-0,368720525	-0,534195762	0	N.A.	0

DPH7	-0,368794003	-0,329792882	0	N.A.	0
DONSON	-0,369052935	0,012137867	0	N.A.	0
CCNE1	-0,36906618	-0,173608377	0	N.A.	0
COPS8	-0,369121174	-0,256194683	0	N.A.	0
EXOSC1	-0,369138423	-0,318602411	0	N.A.	0
POLA1	-0,369189503	-0,125980665	0	N.A.	0
C12orf29	-0,369247492	-0,376188725	0	N.A.	0
GCFC2	-0,369498749	-0,34020338	0	N.A.	0
SFXN2	-0,369631397	-0,237932639	0	N.A.	0
ERI2	-0,369761287	-0,191906848	0	N.A.	0
G6PD	-0,36988704	-0,153049472	0	N.A.	0
PGRMC1	-0,370010272	0,153275376	0	N.A.	0
FASTKD2	-0,370043933	-0,154965186	0	N.A.	0
MARCKSL1	-0,370187811	-0,112239436	0	N.A.	0
OTUD6B	-0,370248595	-0,348237007	0	N.A.	0
IFT43	-0,370373185	-0,13153741	0	N.A.	0
TMEM205	-0,370683432	0,089284749	0	N.A.	0
MCM8	-0,370813666	-0,112320173	0	N.A.	0
YBX2	-0,370987101	-0,196549518	0	N.A.	0
DOHH	-0,371058912	-0,149077934	0	N.A.	0
TRIM28	-0,371398103	-0,079488395	0	N.A.	0
GUSB	-0,371912567	-0,228875356	0	N.A.	0
MTHFS	-0,371913699	-0,293061201	0	N.A.	0
RPUSD4	-0,372083539	-0,209229461	0	N.A.	0
ALG6	-0,372226733	-0,141263162	0	N.A.	0
PTTG2	-0,372267094	-0,042788703	0	N.A.	0
ARHGAP11B	-0,372477367	-0,063093044	0	N.A.	0
DPH6	-0,37247861	-0,023877555	0	N.A.	0
DUSP2	-0,372635225	-0,230732109	0	N.A.	0
FAHD2A	-0,372877407	-0,334748188	0	N.A.	0
LRRC47	-0,372949622	-0,041761023	0	N.A.	0
IFITM3	-0,373032341	-0,443631783	0	N.A.	0
EIF2S2	-0,373419188	0,004581725	0	N.A.	0

DPP3	-0,373511724	0,086837108	0	N.A.	0
TEAD4	-0,373548547	-0,2486619	0	N.A.	0
PRPF4	-0,373589785	-0,026052761	0	N.A.	0
UTP14A	-0,373809753	0,059275117	0	N.A.	0
SCLT1	-0,373842261	-0,31798122	0	N.A.	0
PHGDH	-0,374060849	-0,326382055	0	N.A.	0
SMYD5	-0,374074955	0,120735559	0	N.A.	0
CEP78	-0,374402877	-0,190388492	0	N.A.	0
SDHA	-0,374429511	-0,041602421	0	N.A.	0
SUSD3	-0,374492285	-0,402721198	0	N.A.	0
TSSC1	-0,374516308	-0,360479159	0	N.A.	0
RHNO1	-0,374639835	-0,198929248	0	N.A.	0
MRM1	-0,374724502	-0,064689935	0	N.A.	0
IFT22	-0,37472844	-0,412439431	0	N.A.	0
CDC7	-0,374844882	-0,294610245	0	N.A.	0
MEA1	-0,375006326	-0,283745685	0	N.A.	0
POLR1E	-0,375052762	-0,188554669	0	N.A.	0
ACP6	-0,375186554	-0,257100878	0	N.A.	0
NDRG2	-0,37541008	-0,002468778	0	N.A.	0
SSR3	-0,375441565	-0,276747125	0	N.A.	0
ILF3-AS1	-0,375462458	-0,001876008	0	N.A.	0
GEMIN2	-0,375507973	-0,407063641	0	N.A.	0
WRAP53	-0,37552731	-0,081824043	0	N.A.	0
MECR	-0,375567102	-0,348508469	0	N.A.	0
ORMDL1	-0,375711129	-0,270488473	0	N.A.	0
NELFE	-0,376082886	-0,228310576	0	N.A.	0
ACSS1	-0,376188741	-0,043143931	0	N.A.	0
INCENP	-0,376492231	-0,287886248	0	N.A.	0
CEP57	-0,376622801	-0,063920832	0	N.A.	0
ARPC5L	-0,376731062	-0,039527372	0	N.A.	0
TRMT11	-0,376789221	-0,308084051	0	N.A.	0
QTRT1	-0,376909204	-0,171920975	0	N.A.	0
UFD1L	-0,377598428	-0,276694099	0	N.A.	0

AADAT	-0,377636048	-0,36220033	0	N.A.	0
GRK6	-0,377647754	-0,281997588	0	N.A.	0
CEBPZOS	-0,377713148	-0,134597038	0	N.A.	0
RIF1	-0,377811691	0,007570086	0	N.A.	0
PDIA3	-0,377823694	0,016955702	0	N.A.	0
PITHD1	-0,377848889	-0,314185774	0	N.A.	0
SARS2	-0,377879708	-0,204066149	0	N.A.	0
C12orf65	-0,377962143	-0,233046287	0	N.A.	0
SEC13	-0,37826902	-0,20935466	0	N.A.	0
NEK6	-0,378379652	-0,231854733	0	N.A.	0
ACAT2	-0,378406961	-0,091440343	0	N.A.	0
CENPI	-0,378600222	-0,002171143	0	N.A.	0
CWC15	-0,3789084	-0,27069274	0	N.A.	0
WWOX	-0,378916515	-0,263742463	0	N.A.	0
KIF14	-0,378934845	-0,203588055	0	N.A.	0
TRIT1	-0,378940029	-0,191683387	0	N.A.	0
PSMC3IP	-0,379156305	-0,393885898	0	N.A.	0
FAM98A	-0,379206852	-0,253466463	0	N.A.	0
MOCS2	-0,379221203	-0,392849244	0	N.A.	0
ALG1	-0,379275782	-0,067467702	0	N.A.	0
ORC3	-0,379714614	-0,359773667	0	N.A.	0
CKAP2L	-0,379971296	-0,214944016	0	N.A.	0
EEF2KMT	-0,380000336	-0,191511656	0	N.A.	0
RTCB	-0,380031602	0,074601522	0	N.A.	0
HTRA2	-0,380138896	-0,299076732	0	N.A.	0
METTL13	-0,380308863	0,005339365	0	N.A.	0
PNPO	-0,380317545	-0,378533464	0	N.A.	0
MMADHC	-0,380779345	-0,156108076	0	N.A.	0
PARP2	-0,380985599	-0,189268345	0	N.A.	0
NFYB	-0,381015139	-0,40389208	0	N.A.	0
PRADC1	-0,381096731	-0,051419138	0	N.A.	0
DGCR6L	-0,381100129	-0,353470849	0	N.A.	0
EIF2B1	-0,381232902	-0,267610137	0	N.A.	0

DNTTIP2	-0,381284165	-0,150161419	0	N.A.	0
GINS4	-0,381424341	-0,266176942	0	N.A.	0
NUDT16L1	-0,381481243	-0,115068344	0	N.A.	0
FAM72D	-0,381716325	-0,056354487	0	N.A.	0
FAU	-0,381835601	-0,430771275	0	N.A.	0
SLC25A4	-0,38194649	-0,104957893	0	N.A.	0
FAF1	-0,382010505	-0,291145645	0	N.A.	0
PXMP2	-0,382205538	-0,13643861	0	N.A.	0
PIN4	-0,382250961	-0,322582591	0	N.A.	0
PSMA1	-0,382270846	-0,452928397	0	N.A.	0
CPNE1	-0,382503393	-0,01715805	0	N.A.	0
TOMM20	-0,382721164	-0,375956637	0	N.A.	0
WDR62	-0,382865336	-0,045397148	0	N.A.	0
PNN	-0,382914777	0,068189919	0	N.A.	0
HMCES	-0,382953451	-0,150445665	0	N.A.	0
SLC25A6	-0,383100665	-0,550344732	0	N.A.	0
KNOP1	-0,383134964	0,018363501	0	N.A.	0
TRNT1	-0,383136577	-0,376402843	0	N.A.	0
DRG2	-0,383331756	-0,11805917	0	N.A.	0
POLR1C	-0,38349292	-0,215027716	0	N.A.	0
TACO1	-0,383574997	-0,063080916	0	N.A.	0
PYCRL	-0,383602978	-0,367761394	0	N.A.	0
LRRC45	-0,383624714	-0,326019983	0	N.A.	0
WBP11	-0,383641703	-0,145313817	0	N.A.	0
MED21	-0,38377492	-0,402776513	0	N.A.	0
PIGX	-0,384125796	0,142068041	0	N.A.	0
PSMC1	-0,384188315	-0,308013073	0	N.A.	0
KDELC2	-0,384500164	-0,185672197	0	N.A.	0
FOPNL	-0,384587595	-0,320050258	0	N.A.	0
CREG1	-0,384610567	-0,251552173	0	N.A.	0
HBS1L	-0,384625071	-0,149527457	0	N.A.	0
LIAS	-0,385073363	-0,507778447	0	N.A.	0
DFFA	-0,385140509	-0,46828916	0	N.A.	0

FIS1	-0,385469253	-0,248875768	0	N.A.	0
IDH1	-0,385943247	-0,194275861	0	N.A.	0
COMMD7	-0,385959177	-0,367566727	0	N.A.	0
RWDD4	-0,386161317	-0,191189922	0	N.A.	0
C1orf43	-0,3862	0,135592007	0	N.A.	0
AKT1S1	-0,386206119	-0,240565954	0	N.A.	0
XRCC6BP1	-0,386230385	-0,214098782	0	N.A.	0
ATP5SL	-0,386264339	-0,020497886	0	N.A.	0
SORD	-0,386572525	-0,215623402	0	N.A.	0
USP48	-0,386674501	-0,236012453	0	N.A.	0
MED28	-0,386838662	-0,380057563	0	N.A.	0
ARF1	-0,386898891	0,057542999	0	N.A.	0
EIF2B5	-0,387023402	-0,197866851	0	N.A.	0
VPS35	-0,387107148	0,104227157	0	N.A.	0
PRKAG1	-0,387129075	-0,233482492	0	N.A.	0
CALM1	-0,387207949	-0,147346085	0	N.A.	0
RIOK2	-0,38727924	-0,107585007	0	N.A.	0
MAF1	-0,387597459	-0,404916638	0	N.A.	0
SPR	-0,387664817	0,216458479	0	N.A.	0
WDR3	-0,387777894	-0,20764397	0	N.A.	0
DYNC1LI1	-0,387813632	-0,150685438	0	N.A.	0
DHRS4L2	-0,38781819	-0,064022771	0	N.A.	0
SLC25A15	-0,388089065	-0,239713283	0	N.A.	0
CDCA2	-0,388291881	-0,272090712	0	N.A.	0
DPH5	-0,388328268	-0,520767381	0	N.A.	0
PRMT5	-0,388653692	-0,176094693	0	N.A.	0
C9orf152	-0,38865769	-0,251795739	0	N.A.	0
THEM6	-0,388767756	0,194339212	0	N.A.	0
ACTR3B	-0,388992079	-0,205312207	0	N.A.	0
WRAP73	-0,38899422	-0,081679637	0	N.A.	0
ARF5	-0,389216869	-0,230678001	0	N.A.	0
TIPRL	-0,389266706	-0,491674549	0	N.A.	0
TESC	-0,389395861	0,04329681	0	N.A.	0

PLRG1	-0,389554694	-0,372539294	0	N.A.	0
ARL16	-0,38986024	-0,231868556	0	N.A.	0
NUBPL	-0,389939634	-0,247472156	0	N.A.	0
ANAPC13	-0,390017568	-0,425737257	0	N.A.	0
KDM1A	-0,390027027	0,015686902	0	N.A.	0
ACAD9	-0,390208466	-0,008754904	0	N.A.	0
DNAJA3	-0,390219619	-0,16180095	0	N.A.	0
THG1L	-0,390333671	-0,43610823	0	N.A.	0
HMOX2	-0,390342519	0,005655056	0	N.A.	0
PRR7	-0,390425733	-0,207118529	0	N.A.	0
SGOL2	-0,390534183	-0,26131201	0	N.A.	0
TXNL1	-0,390548155	-0,327798105	0	N.A.	0
ZBED3	-0,390769212	-0,264469015	0	N.A.	0
PDHX	-0,391156243	-0,188420903	0	N.A.	0
C9orf114	-0,391165933	-0,338984894	0	N.A.	0
OXCT1	-0,391525427	-0,137970831	0	N.A.	0
NUP107	-0,391577998	0,010902049	0	N.A.	0
NCBP2	-0,391584639	-0,354090382	0	N.A.	0
NDUFV3	-0,391615005	-0,265641213	0	N.A.	0
MTHFD2L	-0,391627937	-0,336917351	0	N.A.	0
SAP18	-0,391747703	-0,244078907	0	N.A.	0
TGDS	-0,391941002	-0,043142264	0	N.A.	0
DPM2	-0,39208704	-0,178174933	0	N.A.	0
UQCRHL	-0,392476949	-0,315276518	0	N.A.	0
GRHPR	-0,392838765	-0,1143684	0	N.A.	0
RTFDC1	-0,392851383	-0,375942795	0	N.A.	0
MACROD1	-0,392877136	-0,325960564	0	N.A.	0
CENPO	-0,393020876	-0,131237258	0	N.A.	0
POLR2J	-0,393034508	-0,213377373	0	N.A.	0
RRP1	-0,393049723	0,035593998	0	N.A.	0
NOC4L	-0,393095155	-0,33962704	0	N.A.	0
TMEM183A	-0,393096528	-0,245436717	0	N.A.	0
METTL21A	-0,393102844	-0,217515721	0	N.A.	0

PBDC1	-0,393159998	0,193896649	0	N.A.	0
NUP54	-0,393288602	-0,214622537	0	N.A.	0
RNASEH1	-0,393488686	-0,072331427	0	N.A.	0
RALY	-0,393673245	-0,169637238	0	N.A.	0
TMEM223	-0,393763312	0,208123801	0	N.A.	0
TCEA1	-0,394085969	-0,513525004	0	N.A.	0
SNW1	-0,394140037	-0,186368872	0	N.A.	0
USP2-AS1	-0,39417088	-0,300183753	0	N.A.	0
POLA2	-0,394234105	-0,100119572	0	N.A.	0
NOA1	-0,394253651	-0,131131779	0	N.A.	0
SMYD2	-0,394354329	-0,505586968	0	N.A.	0
PGAP2	-0,394445329	-0,300585402	0	N.A.	0
FDX1L	-0,394683186	-0,193738545	0	N.A.	0
GCA	-0,395007641	-0,309197051	0	N.A.	0
MVK	-0,395345959	-0,037696	0	N.A.	0
GCHFR	-0,395759625	-0,094552509	0	N.A.	0
TCF19	-0,396257555	-0,187675915	0	N.A.	0
STXBP6	-0,396344326	-0,116169708	0	N.A.	0
NT5DC1	-0,396603089	-0,207992182	0	N.A.	0
UTP6	-0,396690706	-0,189250646	0	N.A.	0
GLT8D1	-0,396760977	-0,270153193	0	N.A.	0
VSNL1	-0,396872885	-0,36148693	0	N.A.	0
RRP1B	-0,396874075	-0,190654517	0	N.A.	0
EXOSC4	-0,397066539	-0,071629299	0	N.A.	0
ANLN	-0,397118491	-0,271996736	0	N.A.	0
SCCPDH	-0,397780407	-0,145211575	0	N.A.	0
BRAT1	-0,398006062	0,095131928	0	N.A.	0
CIAO1	-0,398193048	0,047593155	0	N.A.	0
TBCD	-0,398510802	-0,064986	0	N.A.	0
FOXRED1	-0,398768571	-0,383480142	0	N.A.	0
IK	-0,398887247	-0,215678374	0	N.A.	0
EXO1	-0,399073904	-0,307936644	0	N.A.	0
OSTC	-0,39955043	-0,272661796	0	N.A.	0

TIMM9	-0,399578587	-0,458791835	0	N.A.	0
HDDC3	-0,399725191	-0,166277272	0	N.A.	0
MGME1	-0,399947539	-0,097194883	0	N.A.	0
MYC	-0,40001019	-0,194765038	0	N.A.	0
KXD1	-0,400029065	-0,377021031	0	N.A.	0
SDAD1	-0,400086795	0,056219446	0	N.A.	0
NOB1	-0,400098776	-0,276576872	0	N.A.	0
FAM204A	-0,400108359	-0,176413258	0	N.A.	0
TAMM41	-0,400230295	-0,283463591	0	N.A.	0
ORC1	-0,400238415	-0,365178745	0	N.A.	0
TMEM177	-0,400288201	-0,13201622	0	N.A.	0
FRG1	-0,400338188	0,033267338	0	N.A.	0
FUS	-0,400388117	0,040965407	0	N.A.	0
HADHB	-0,400414039	-0,204105449	0	N.A.	0
OCIAD1	-0,400801474	-0,178842823	0	N.A.	0
MRE11A	-0,400844975	-0,371338777	0	N.A.	0
RBX1	-0,401191909	-0,309465059	0	N.A.	0
FANCA	-0,401230898	-0,116481678	0	N.A.	0
NKRF	-0,401261919	-0,110660089	0	N.A.	0
ALDH16A1	-0,401409809	0,013489885	0	N.A.	0
MCRS1	-0,401495503	-0,356242382	0	N.A.	0
H2AFX	-0,401508736	-0,38719063	0	N.A.	0
LRRC59	-0,401560592	-0,216376338	0	N.A.	0
ATRAID	-0,401785076	-0,149003057	0	N.A.	0
SAP30	-0,40192065	-0,015827517	0	N.A.	0
PSPC1	-0,401995116	-0,04217888	0	N.A.	0
ASRGL1	-0,402038209	-0,359804607	0	N.A.	0
BRI3BP	-0,402515571	0,088163922	0	N.A.	0
THOC6	-0,402822826	-0,396484963	0	N.A.	0
SLC12A2	-0,4028841	-0,08540291	0	N.A.	0
KATNB1	-0,402932977	-0,085288784	0	N.A.	0
SLC38A5	-0,403300014	0,318906968	0	N.A.	0
ATP6V0E2	-0,403578499	-0,1973323	0	N.A.	0

FANCG	-0,403840306	-0,18299477	0	N.A.	0
UBAC1	-0,403948688	-0,223120882	0	N.A.	0
GAL	-0,404118176	-0,252584196	0	N.A.	0
NDC80	-0,40441674	-0,470246481	0	N.A.	0
FAM216A	-0,404719327	-0,316205899	0	N.A.	0
MCMBP	-0,40474383	-0,20606412	0	N.A.	0
YARS2	-0,404826202	-0,253107157	0	N.A.	0
RECQL4	-0,404878032	-0,311947753	0	N.A.	0
CCDC28B	-0,405253335	-0,267628675	0	N.A.	0
TMEM186	-0,405407759	0,005526581	0	N.A.	0
ETV4	-0,405663542	-0,207312748	0	N.A.	0
MYL6B	-0,405689642	-0,43578862	0	N.A.	0
CFAP36	-0,405849843	-0,244179167	0	N.A.	0
CCDC137	-0,40588343	0,164157316	0	N.A.	0
PM20D2	-0,405894902	-0,245664455	0	N.A.	0
FAM58A	-0,405935662	-0,18798205	0	N.A.	0
PTPRO	-0,406056121	-0,33727652	0	N.A.	0
TGS1	-0,406532577	0,169769667	0	N.A.	0
RFT1	-0,406746979	-0,074063529	0	N.A.	0
KIF18A	-0,40694898	-0,097822966	0	N.A.	0
UBE2I	-0,406983791	-0,216834532	0	N.A.	0
BAG2	-0,40705039	-0,516387794	0	N.A.	0
TMEM109	-0,407058639	-0,046193931	0	N.A.	0
CHORDC1	-0,407086252	-0,428487408	0	N.A.	0
GRWD1	-0,407268862	-0,202544176	0	N.A.	0
EIF4E	-0,407498459	-0,4729904	0	N.A.	0
SMC6	-0,407530774	-0,02476793	0	N.A.	0
MCAT	-0,407850014	-0,313416069	0	N.A.	0
CSTF2	-0,407897925	-0,285075267	0	N.A.	0
TXLNG	-0,407932686	-0,129811372	0	N.A.	0
RPGRIP1L	-0,408105513	-0,376079586	0	N.A.	0
MINA	-0,40817836	-0,169587615	0	N.A.	0
GTF2F2	-0,408559382	-0,041218819	0	N.A.	0

C1orf112	-0,408574091	-0,332729263	0	N.A.	0
TOP1MT	-0,408974672	-0,219100635	0	N.A.	0
MSH2	-0,409170709	-0,312439059	0	N.A.	0
FAIM	-0,409188483	-0,078699368	0	N.A.	0
TAF6	-0,409237431	-0,225479203	0	N.A.	0
PPIE	-0,409464861	-0,234328119	0	N.A.	0
TMEM98	-0,409639627	-0,037783993	0	N.A.	0
MPHOSPH6	-0,409873641	-0,487798122	0	N.A.	0
ZNF695	-0,409882027	-0,441323859	0	N.A.	0
SLC5A6	-0,409902649	-0,110222995	0	N.A.	0
SLC35B2	-0,410109026	-0,089139359	0	N.A.	0
MIS18BP1	-0,410163031	-0,307187958	0	N.A.	0
RGMB	-0,410328093	-0,20216373	0	N.A.	0
TTC26	-0,410341067	-0,179321067	0	N.A.	0
EARS2	-0,41045316	-0,207941083	0	N.A.	0
VIL1	-0,410606353	0,155132596	0	N.A.	0
TRMU	-0,410769409	-0,323887469	0	N.A.	0
METTL3	-0,410796877	-0,218785326	0	N.A.	0
UQCC1	-0,410851595	0,056676816	0	N.A.	0
TEX264	-0,410962184	0,118616983	0	N.A.	0
SNHG19	-0,41103041	-0,417978447	0	N.A.	0
TTC27	-0,411159382	-0,255263905	0	N.A.	0
GLOD4	-0,411160255	-0,319814212	0	N.A.	0
NXT1	-0,411206871	-0,325126467	0	N.A.	0
FUBP1	-0,411560032	-0,006263276	0	N.A.	0
PPP5C	-0,411759055	-0,167743036	0	N.A.	0
PPP2R1A	-0,412114213	0,049981234	0	N.A.	0
TRAPPC1	-0,412181688	-0,380097818	0	N.A.	0
LINC01315	-0,412278255	-0,34324849	0	N.A.	0
LAPTM4B	-0,412362633	0,007664337	0	N.A.	0
TMEM9	-0,412621597	0,32005787	0	N.A.	0
MGST2	-0,412653459	0,093286918	0	N.A.	0
ABCF1	-0,412865387	-0,018482848	0	N.A.	0

IDH3G	-0,413033851	-0,137846415	0	N.A.	0
GNPNAT1	-0,413231064	-0,488485479	0	N.A.	0
APPL1	-0,413272608	-0,10524491	0	N.A.	0
FAH	-0,413362244	-0,445251184	0	N.A.	0
INTS10	-0,413386261	-0,161492207	0	N.A.	0
DTWD1	-0,413427159	-0,435632425	0	N.A.	0
JAGN1	-0,413567027	0,075173944	0	N.A.	0
SPG21	-0,4136229	-0,383937336	0	N.A.	0
ISCU	-0,413738438	-0,418265822	0	N.A.	0
BRCC3	-0,413857228	-0,060141696	0	N.A.	0
R3HDM1	-0,414110381	-0,365317956	0	N.A.	0
CHAF1B	-0,414297754	-0,234177841	0	N.A.	0
FUNDC2	-0,414307226	-0,414075078	0	N.A.	0
RPP25L	-0,414519024	-0,187507306	0	N.A.	0
EPHB2	-0,414602488	-0,035147719	0	N.A.	0
SESN1	-0,414631309	-0,261297393	0	N.A.	0
LARP7	-0,414752945	0,024281485	0	N.A.	0
COX7A2L	-0,415028796	-0,526451659	0	N.A.	0
TMEM243	-0,415708379	-0,587271815	0	N.A.	0
KIF20A	-0,415892655	-0,278100416	0	N.A.	0
POLR3H	-0,415912153	-0,266124529	0	N.A.	0
WDR76	-0,41629645	-0,308592227	0	N.A.	0
PGD	-0,416334549	-0,448432844	0	N.A.	0
EED	-0,416703052	-0,20573988	0	N.A.	0
BUB1B	-0,416877341	-0,242720684	0	N.A.	0
C14orf142	-0,4169821	-0,38776891	0	N.A.	0
TYSND1	-0,417036965	-0,122343062	0	N.A.	0
CTU2	-0,417054462	-0,225101956	0	N.A.	0
CTNNBL1	-0,417542349	-0,220879487	0	N.A.	0
SPCS2	-0,417810781	-0,136368335	0	N.A.	0
FAM195A	-0,417815736	-0,141666071	0	N.A.	0
DDIAS	-0,418080238	-0,100704468	0	N.A.	0
DLEU2	-0,41826322	-0,384353658	0	N.A.	0

RNF187	-0,418265278	0,241303822	0	N.A.	0
NQO1	-0,418448309	-0,022710656	0	N.A.	0
PUS7	-0,418577256	-0,407776139	0	N.A.	0
HSPA4	-0,41859691	-0,172064907	0	N.A.	0
REEP4	-0,419239247	-0,052339821	0	N.A.	0
TSEN34	-0,419345791	0,193683195	0	N.A.	0
EMC8	-0,419581345	-0,220407279	0	N.A.	0
WHSC1	-0,419592311	-0,115932524	0	N.A.	0
AURKAIP1	-0,419675909	-0,203919257	0	N.A.	0
ZCRB1	-0,419880396	-0,376439412	0	N.A.	0
MARC1	-0,419968231	-0,220030858	0	N.A.	0
UFC1	-0,420004051	-0,467208424	0	N.A.	0
DUS3L	-0,420061747	-0,344485414	0	N.A.	0
MCTS1	-0,420101831	-0,452189672	0	N.A.	0
SUCLG2	-0,420195724	-0,106767542	0	N.A.	0
NOX1	-0,420218788	-0,212651958	0	N.A.	0
POLD1	-0,420845591	-0,316789602	0	N.A.	0
WDR75	-0,42112979	-0,313916176	0	N.A.	0
TMEM5	-0,421240228	-0,00078713	0	N.A.	0
CNPY3	-0,421443669	-0,309074199	0	N.A.	0
COPZ1	-0,421613117	-0,32220033	0	N.A.	0
RGS16	-0,421627992	-0,083485034	0	N.A.	0
MCCC2	-0,421868761	-0,339743566	0	N.A.	0
KIF11	-0,421908476	-0,375787953	0	N.A.	0
RP11-111M2	-0,421926626	-0,427548703	0	N.A.	0
LINC01224	-0,422396612	-0,139322659	0	N.A.	0
LTA4H	-0,42248682	-0,348359286	0	N.A.	0
SHCBP1	-0,422488329	-0,254017731	0	N.A.	0
CCNB1IP1	-0,422523521	-0,416033276	0	N.A.	0
RAD54L	-0,422701531	-0,291082202	0	N.A.	0
COX6B1	-0,42304192	-0,329312929	0	N.A.	0
EMP2	-0,423170936	0,122212381	0	N.A.	0
DLAT	-0,423337956	-0,411538611	0	N.A.	0

ODF2	-0,423373964	-0,370621106	0	N.A.	0
METTL1	-0,423490205	-0,350834754	0	N.A.	0
CDX2	-0,423556482	-0,042671768	0	N.A.	0
YIF1B	-0,423602549	-0,255654334	0	N.A.	0
NTHL1	-0,42373864	0,366423698	0	N.A.	0
MTIF2	-0,423878883	-0,32856495	0	N.A.	0
CDCA4	-0,42413477	-0,325004061	0	N.A.	0
USP13	-0,424313627	0,152071349	0	N.A.	0
COA5	-0,42435433	-0,304259263	0	N.A.	0
TYW3	-0,424405306	-0,376918119	0	N.A.	0
SAFB	-0,424657244	-0,164862099	0	N.A.	0
PRKRA	-0,424860665	-0,066106389	0	N.A.	0
C15orf61	-0,425357825	-0,29925017	0	N.A.	0
DHX29	-0,425544189	-0,325685969	0	N.A.	0
SUV39H2	-0,42600776	-0,355245428	0	N.A.	0
CKAP5	-0,426179131	-0,33175502	0	N.A.	0
IQGAP3	-0,426192264	-0,090402094	0	N.A.	0
NFU1	-0,426295216	-0,360099974	0	N.A.	0
RBM42	-0,426491453	-0,204252861	0	N.A.	0
PWP1	-0,426792336	-0,163438704	0	N.A.	0
SCO1	-0,426868273	-0,269841911	0	N.A.	0
PMS1	-0,427242782	-0,151019586	0	N.A.	0
NOL8	-0,427369822	-0,149858276	0	N.A.	0
KIAA0226L	-0,427485307	-0,534045082	0	N.A.	0
ZNF22	-0,427546152	-0,198836285	0	N.A.	0
TBCB	-0,427781936	-0,284179258	0	N.A.	0
RPF1	-0,427831749	0,052279573	0	N.A.	0
DTL	-0,427968848	-0,203989767	0	N.A.	0
NAT10	-0,428031939	-0,257028973	0	N.A.	0
SLC43A1	-0,428045053	-0,312115837	0	N.A.	0
EXOSC9	-0,428096464	-0,232676214	0	N.A.	0
POLR1D	-0,428139787	-0,233693236	0	N.A.	0
TTC1	-0,428175175	-0,18688923	0	N.A.	0

RNF26	-0,428382917	-0,047592282	0	N.A.	0
PPAT	-0,428390839	-0,424652506	0	N.A.	0
LYSMD2	-0,428411685	-0,45111841	0	N.A.	0
BYSL	-0,428478292	-0,229796655	0	N.A.	0
NRM	-0,429261513	-0,21749515	0	N.A.	0
DDX27	-0,42947469	-0,107428686	0	N.A.	0
UBA2	-0,429723306	-0,129854193	0	N.A.	0
TAF9	-0,429824457	-0,54599064	0	N.A.	0
MYB	-0,430055429	-0,269217103	0	N.A.	0
PGP	-0,430283839	-0,165692861	0	N.A.	0
RNASEH2C	-0,430521819	-0,323155037	0	N.A.	0
CHCHD7	-0,431181703	-0,324871582	0	N.A.	0
NSMCE4A	-0,431366031	-0,19589047	0	N.A.	0
TRMT6	-0,431410749	-0,121890106	0	N.A.	0
HADHA	-0,431553723	-0,300057855	0	N.A.	0
NOP14	-0,432009847	-0,300504983	0	N.A.	0
TMEM14C	-0,432266656	-0,423194747	0	N.A.	0
DPAGT1	-0,432347912	-0,146793529	0	N.A.	0
GATB	-0,43236627	-0,027115761	0	N.A.	0
EMD	-0,432677421	-0,421790625	0	N.A.	0
ARRB2	-0,432755628	-0,404531443	0	N.A.	0
PRELID1	-0,432970942	-0,283463174	0	N.A.	0
PABPC4	-0,432981802	-0,169766338	0	N.A.	0
LAP3	-0,433330757	-0,392995695	0	N.A.	0
PMVK	-0,433502874	0,043564659	0	N.A.	0
UBE2E3	-0,433663719	-0,020429123	0	N.A.	0
FTSJ2	-0,433784824	-0,508985371	0	N.A.	0
TSTA3	-0,433806663	0,006664698	0	N.A.	0
ARHGEF39	-0,433899711	-0,228934039	0	N.A.	0
CINP	-0,434118741	-0,410475124	0	N.A.	0
GTF3C6	-0,434170947	-0,25594399	0	N.A.	0
RANGRF	-0,434402181	-0,291382264	0	N.A.	0
COX6A1	-0,434472523	-0,049386003	0	N.A.	0

ITGB1BP1	-0,434525923	-0,387902898	0	N.A.	0
SMC3	-0,434534002	-0,250245994	0	N.A.	0
PUS1	-0,434918713	-0,233827046	0	N.A.	0
ETFA	-0,435710144	-0,293351554	0	N.A.	0
GPN1	-0,435719511	-0,214631448	0	N.A.	0
SLC39A8	-0,435782328	-0,391517075	0	N.A.	0
NDUFS4	-0,435783976	-0,307255476	0	N.A.	0
AP1M2	-0,435805091	-0,000203715	0	N.A.	0
GSTO2	-0,435900606	-0,348162686	0	N.A.	0
SFPQ	-0,436060591	-0,347726007	0	N.A.	0
NACA	-0,436308094	-0,497402896	0	N.A.	0
FKBP11	-0,436314948	-0,467398504	0	N.A.	0
SKA1	-0,436402554	-0,391279588	0	N.A.	0
TTK	-0,436688633	-0,356000655	0	N.A.	0
SNUPN	-0,437011446	-0,167346767	0	N.A.	0
TIMM44	-0,437097501	-0,111575882	0	N.A.	0
USP5	-0,437115635	-0,076500235	0	N.A.	0
PTRHD1	-0,437182134	-0,438210925	0	N.A.	0
NDUFA6	-0,437182998	-0,276817712	0	N.A.	0
SMARCA4	-0,437372546	0,195444981	0	N.A.	0
C1orf35	-0,437498729	-0,158784554	0	N.A.	0
BCAT2	-0,437599611	-0,132926778	0	N.A.	0
ACYP1	-0,437670663	0,035672182	0	N.A.	0
NDUFA13	-0,437688587	-0,249805144	0	N.A.	0
CCDC85B	-0,437692464	-0,426965431	0	N.A.	0
RAD51	-0,437866531	-0,317907727	0	N.A.	0
ATAD5	-0,438142296	-0,247065335	0	N.A.	0
SHMT2	-0,438148844	-0,357074618	0	N.A.	0
UBE2S	-0,438296913	-0,252580451	0	N.A.	0
TUBA4A	-0,43842943	-0,052479362	0	N.A.	0
TUBA1C	-0,438599455	-0,16981024	0	N.A.	0
BUD31	-0,4387382	-0,43343123	0	N.A.	0
TMEM126A	-0,438787692	-0,248457877	0	N.A.	0

TMEM70	-0,439000812	-0,256026119	0	N.A.	0
TRABD2A	-0,4391816	-0,089105069	0	N.A.	0
CHCHD4	-0,439274193	-0,422596046	0	N.A.	0
DHX15	-0,439393012	0,033405379	0	N.A.	0
MED30	-0,43950245	-0,432138944	0	N.A.	0
TCERG1	-0,439529805	-0,168820041	0	N.A.	0
SSNA1	-0,43997553	-0,031608418	0	N.A.	0
GPS1	-0,440256332	0,155846265	0	N.A.	0
ANAPC7	-0,440679884	-0,172387781	0	N.A.	0
RNF5	-0,441697683	-0,189151143	0	N.A.	0
TMEM237	-0,441716042	-0,260135044	0	N.A.	0
PSME1	-0,441920275	-0,238447597	0	N.A.	0
NOC3L	-0,442464161	-0,106541301	0	N.A.	0
ECI2	-0,442617812	-0,208612649	0	N.A.	0
DPCD	-0,442627731	-0,310052969	0	N.A.	0
GNB2L1	-0,443424584	-0,565404051	0	N.A.	0
THUMPD3	-0,443428442	-0,427632079	0	N.A.	0
TSN	-0,443664506	-0,24162892	0	N.A.	0
CEBPZ	-0,443761719	0,079803199	0	N.A.	0
CDC25C	-0,443881906	-0,232068941	0	N.A.	0
EPHB3	-0,444469632	-0,163868041	0	N.A.	0
PSMD12	-0,444506687	-0,065506896	0	N.A.	0
HIRIP3	-0,444630971	-0,148777728	0	N.A.	0
LYPLA1	-0,44476186	-0,401079406	0	N.A.	0
APOO	-0,445341211	-0,243240055	0	N.A.	0
PPP1R7	-0,445697085	-0,037769803	0	N.A.	0
SUMO2	-0,445866769	-0,259866996	0	N.A.	0
KIF15	-0,446116425	-0,327706489	0	N.A.	0
EIF3D	-0,446160301	-0,434001953	0	N.A.	0
KIF2C	-0,446450017	-0,271975839	0	N.A.	0
GINS3	-0,44672852	-0,385505685	0	N.A.	0
CDK5RAP1	-0,446731848	-0,401765007	0	N.A.	0
CHTF18	-0,447114689	-0,262353455	0	N.A.	0

PIR	-0,447355166	-0,463984273	0	N.A.	0
CHAC2	-0,447592911	-0,268741907	0	N.A.	0
COPS4	-0,447748128	-0,234178597	0	N.A.	0
EMG1	-0,447762154	-0,204138164	0	N.A.	0
EIF3L	-0,44779888	-0,583785916	0	N.A.	0
GCDH	-0,447898524	-0,484802136	0	N.A.	0
FANCB	-0,448109006	-0,213922381	0	N.A.	0
NAA50	-0,448135882	-0,313784954	0	N.A.	0
TEX30	-0,448398476	-0,468335099	0	N.A.	0
NSL1	-0,448420956	-0,376889581	0	N.A.	0
EIF3E	-0,448479794	-0,537032669	0	N.A.	0
HDAC3	-0,448627206	-0,164758534	0	N.A.	0
UCK2	-0,448742873	-0,249919767	0	N.A.	0
ESCO2	-0,448758591	-0,395428471	0	N.A.	0
ISOC1	-0,448850017	-0,322736207	0	N.A.	0
APMAP	-0,448877427	-0,064646247	0	N.A.	0
NIF3L1	-0,448904731	-0,271323918	0	N.A.	0
PPA1	-0,44915664	-0,251394392	0	N.A.	0
EBP	-0,449263392	0,14248364	0	N.A.	0
ARL5A	-0,449263884	-0,558481305	0	N.A.	0
DCPS	-0,449328865	-0,259623934	0	N.A.	0
GOLIM4	-0,44937279	-0,144548293	0	N.A.	0
TEFM	-0,449557078	0,070325625	0	N.A.	0
COMT	-0,449644538	0,039002322	0	N.A.	0
RHOBTB3	-0,450188959	-0,100754036	0	N.A.	0
PSRC1	-0,450448174	-0,371328755	0	N.A.	0
FAM111B	-0,450475532	-0,31510781	0	N.A.	0
ZNF367	-0,451596981	-0,319619632	0	N.A.	0
SF3B3	-0,451737185	0,067750562	0	N.A.	0
ZC3H8	-0,451755951	-0,157124731	0	N.A.	0
SUMO3	-0,452004361	-0,335491234	0	N.A.	0
NUP85	-0,452563706	-0,139417523	0	N.A.	0
PNPT1	-0,452986314	-0,191513533	0	N.A.	0

QRSL1	-0,453099647	-0,45351858	0	N.A.	0
ASPM	-0,453216314	-0,227046889	0	N.A.	0
UBA5	-0,453372058	-0,254587057	0	N.A.	0
FIP1L1	-0,453394387	0,127811666	0	N.A.	0
MORN2	-0,453568366	-0,320429639	0	N.A.	0
C12orf10	-0,453657369	-0,292013928	0	N.A.	0
HNRNPUL1	-0,453694214	-0,266842682	0	N.A.	0
NUP210	-0,454078268	-0,054713036	0	N.A.	0
RSRC1	-0,454239577	-0,334212675	0	N.A.	0
DNPEP	-0,454413749	0,029538139	0	N.A.	0
GYG1	-0,454422474	-0,284049742	0	N.A.	0
PPP1R1B	-0,454757515	-0,265433399	0	N.A.	0
TPI1	-0,454810997	-0,302596011	0	N.A.	0
KHK	-0,454832206	-0,05808086	0	N.A.	0
USP39	-0,454970214	-0,347432796	0	N.A.	0
C1orf123	-0,455289705	-0,420714503	0	N.A.	0
TSR2	-0,455633775	-0,440186336	0	N.A.	0
METTL2A	-0,455692876	-0,397535497	0	N.A.	0
GPAA1	-0,455704259	-0,161719854	0	N.A.	0
AUNIP	-0,455939145	-0,438332652	0	N.A.	0
AASDHPPT	-0,456137172	-0,405472314	0	N.A.	0
SRRM1	-0,456508025	0,059027012	0	N.A.	0
TIMM17A	-0,456649041	-0,201263998	0	N.A.	0
UBE2L3	-0,456875419	-0,359265927	0	N.A.	0
MTX2	-0,457315239	-0,319594282	0	N.A.	0
MSH6	-0,457358616	-0,250999891	0	N.A.	0
UBA52	-0,457378973	-0,517388118	0	N.A.	0
DDC	-0,457390156	-0,229608462	0	N.A.	0
MIPEP	-0,457402772	-0,283334363	0	N.A.	0
JTB	-0,457759788	-0,295502751	0	N.A.	0
DDX49	-0,457873881	-0,376226907	0	N.A.	0
RTF1	-0,458252469	-0,178772828	0	N.A.	0
QARS	-0,458537811	-0,340277038	0	N.A.	0

NCAPD3	-0,458628066	-0,283548297	0	N.A.	0
AKIP1	-0,459034357	-0,155758341	0	N.A.	0
TMEM258	-0,459176157	-0,388955195	0	N.A.	0
BRCA2	-0,459295139	-0,16845711	0	N.A.	0
ATP5E	-0,459340258	-0,340679556	0	N.A.	0
NOL10	-0,459406732	-0,275466388	0	N.A.	0
DIMT1	-0,459426131	-0,554273894	0	N.A.	0
MRRF	-0,459480163	-0,095310088	0	N.A.	0
PINX1	-0,459490888	-0,429754929	0	N.A.	0
EIF2D	-0,459592332	-0,362449865	0	N.A.	0
EIF3H	-0,459813243	-0,468782899	0	N.A.	0
RWDD1	-0,460145946	-0,465480394	0	N.A.	0
PFDN2	-0,460737966	-0,194319793	0	N.A.	0
RPA1	-0,460963952	-0,382225475	0	N.A.	0
PRMT3	-0,46101615	-0,237933225	0	N.A.	0
SLC37A4	-0,46103375	-0,160383866	0	N.A.	0
NMRAL1	-0,46105879	0,075033098	0	N.A.	0
PPP1CA	-0,461264804	-0,234418501	0	N.A.	0
UTP11L	-0,461369797	-0,515481894	0	N.A.	0
PTRH2	-0,461471489	-0,284833404	0	N.A.	0
CUEDC2	-0,461715235	-0,583114638	0	N.A.	0
HJURP	-0,46172705	-0,213950941	0	N.A.	0
IVD	-0,461738352	-0,344931494	0	N.A.	0
RPE	-0,462126186	-0,555029337	0	N.A.	0
SPAG5	-0,46216859	-0,326432426	0	N.A.	0
ELP6	-0,462420248	-0,472101159	0	N.A.	0
POLE2	-0,462587791	-0,38102172	0	N.A.	0
TIMMDC1	-0,462686716	-0,189872065	0	N.A.	0
CPSF4	-0,462714677	-0,360026298	0	N.A.	0
CDCA8	-0,463088924	-0,391594446	0	N.A.	0
SMIM20	-0,463291608	-0,317845761	0	N.A.	0
DHX9	-0,463433876	-0,144793506	0	N.A.	0
RAD23A	-0,463440091	-0,356710459	0	N.A.	0

CWC27	-0,4637461	-0,21568495	0	N.A.	0
MPHOSPH10	-0,463899818	-0,239563255	0	N.A.	0
BRMS1	-0,464449346	-0,272470998	0	N.A.	0
STX10	-0,464505824	-0,052894982	0	N.A.	0
FAM105A	-0,464707766	-0,150393205	0	N.A.	0
ALKBH2	-0,465197756	-0,359672244	0	N.A.	0
NCAPD2	-0,465374907	-0,251888891	0	N.A.	0
ODC1	-0,465385217	-0,496871191	0	N.A.	0
PARVB	-0,46550751	-0,371287122	0	N.A.	0
FBXW9	-0,465570473	-0,267379246	0	N.A.	0
CARS2	-0,465573445	-0,102732436	0	N.A.	0
NAP1L4	-0,465679415	-0,095523677	0	N.A.	0
COX5B	-0,465679831	-0,283907647	0	N.A.	0
TDGF1	-0,466294421	-0,453523104	0	N.A.	0
OARD1	-0,466348027	-0,327800137	0	N.A.	0
CCDC25	-0,466364425	-0,274429767	0	N.A.	0
AP1S1	-0,466572921	-0,185358064	0	N.A.	0
HES1	-0,466724191	0,000341786	0	N.A.	0
HSDL2	-0,466757495	-0,385439316	0	N.A.	0
PPHLN1	-0,466985959	-0,337133453	0	N.A.	0
CLTA	-0,467224189	-0,261803082	0	N.A.	0
MTX1	-0,467244626	-0,401301864	0	N.A.	0
WDR4	-0,467420657	-0,153252439	0	N.A.	0
CRLS1	-0,467556331	-0,232677247	0	N.A.	0
LYRM7	-0,467750479	-0,214103344	0	N.A.	0
TM7SF3	-0,467858294	-0,264262123	0	N.A.	0
ARL3	-0,467859878	-0,232112054	0	N.A.	0
SEPT2	-0,467914707	-0,29168911	0	N.A.	0
NCAPH	-0,468184433	-0,302124859	0	N.A.	0
MLF2	-0,46832918	-0,373486497	0	N.A.	0
POLR2C	-0,468377313	-0,007032208	0	N.A.	0
U2AF2	-0,468532503	0,042469171	0	N.A.	0
EIF3J	-0,468584687	-0,321698609	0	N.A.	0

REXO4	-0,468594258	-0,265391428	0	N.A.	0
QDPR	-0,468844687	-0,47382863	0	N.A.	0
TPGS2	-0,468921004	-0,454958601	0	N.A.	0
KRR1	-0,469035253	-0,235970814	0	N.A.	0
CCNC	-0,469105464	-0,260158386	0	N.A.	0
PIGF	-0,46921046	0,234770299	0	N.A.	0
NT5DC2	-0,469410238	-0,097230743	0	N.A.	0
UMPS	-0,469632978	-0,103992149	0	N.A.	0
NOC2L	-0,469809683	-0,215264767	0	N.A.	0
DNAJA2	-0,469817346	0,015002787	0	N.A.	0
F12	-0,470196113	0,090154766	0	N.A.	0
ASF1A	-0,470330966	-0,327540993	0	N.A.	0
EXOSC2	-0,47036197	-0,325175315	0	N.A.	0
BRD7	-0,470609908	0,024497553	0	N.A.	0
AURKA	-0,470879097	-0,406437142	0	N.A.	0
KIF23	-0,471068757	-0,335821258	0	N.A.	0
RABGGTB	-0,471293462	-0,377017451	0	N.A.	0
VDAC1	-0,471585136	-0,297949445	0	N.A.	0
DDX55	-0,471937477	-0,051369538	0	N.A.	0
TIMM17B	-0,472215459	0,079823389	0	N.A.	0
NANS	-0,472266396	-0,14051971	0	N.A.	0
GHITM	-0,472680689	-0,204247267	0	N.A.	0
ERAL1	-0,473251377	-0,288073987	0	N.A.	0
GINS1	-0,47334568	-0,201504429	0	N.A.	0
YEATS4	-0,473550592	-0,376858156	0	N.A.	0
SMARCD2	-0,473704468	-0,001474487	0	N.A.	0
ATP5G2	-0,47389774	-0,458696591	0	N.A.	0
POP4	-0,473955541	-0,132943274	0	N.A.	0
NUP35	-0,474136833	-0,258149336	0	N.A.	0
PRPF38B	-0,474351342	-0,001221347	0	N.A.	0
GTF2H3	-0,474448894	-0,298202142	0	N.A.	0
DLEU1	-0,474488336	-0,562612656	0	N.A.	0
NDUFAF6	-0,474862731	-0,399770292	0	N.A.	0

ESF1	-0,475126406	-0,027568484	0	N.A.	0
MCM10	-0,475270135	-0,295154815	0	N.A.	0
SS18L2	-0,475277298	-0,354524422	0	N.A.	0
LRR1	-0,47542732	-0,469149364	0	N.A.	0
ALG8	-0,476089956	-0,13565657	0	N.A.	0
CETN3	-0,476207102	-0,449048964	0	N.A.	0
SF3B6	-0,476341593	-0,409441599	0	N.A.	0
POLR3G	-0,476626575	-0,338008448	0	N.A.	0
PDCD2L	-0,476809894	-0,308752801	0	N.A.	0
NUFIP1	-0,476828653	-0,267794595	0	N.A.	0
ABCF2	-0,476836265	-0,397061365	0	N.A.	0
TCTEX1D2	-0,476849767	-0,444304559	0	N.A.	0
RAD21	-0,476993771	-0,203666321	0	N.A.	0
SMIM4	-0,477697158	-0,607198631	0	N.A.	0
CDC45	-0,477809822	-0,395659302	0	N.A.	0
HPDL	-0,477930047	-0,228245124	0	N.A.	0
SASS6	-0,478111276	-0,183337126	0	N.A.	0
NUPL2	-0,478346932	-0,361469386	0	N.A.	0
PKMYT1	-0,478614881	-0,27653584	0	N.A.	0
CDKN2AIPNL	-0,478662806	-0,500832303	0	N.A.	0
ARV1	-0,478807626	-0,121088159	0	N.A.	0
BRCA1	-0,478963072	0,049081438	0	N.A.	0
RBM8A	-0,479051231	-0,352002088	0	N.A.	0
NFS1	-0,479223463	-0,523140202	0	N.A.	0
WDR5	-0,479332831	-0,248491354	0	N.A.	0
ST13	-0,479361537	-0,500806503	0	N.A.	0
OSGEP	-0,479622736	-0,190016216	0	N.A.	0
COQ4	-0,479653848	-0,22662107	0	N.A.	0
SLC27A5	-0,479764621	-0,416672965	0	N.A.	0
CLN6	-0,479799158	0,012587346	0	N.A.	0
KIFC1	-0,479799506	-0,414240631	0	N.A.	0
NCBP2-AS2	-0,479847787	-0,02043099	0	N.A.	0
UQCC3	-0,480106985	-0,005204889	0	N.A.	0

PMPCB	-0,480542379	-0,277373739	0	N.A.	0
EIF4A3	-0,480639443	-0,236216791	0	N.A.	0
PDRG1	-0,480707521	-0,34467189	0	N.A.	0
TATDN1	-0,480720198	-0,385751028	0	N.A.	0
CDK1	-0,48088552	-0,398669705	0	N.A.	0
SNHG10	-0,481298479	-0,037180574	0	N.A.	0
DEPDC1	-0,481468018	-0,373633736	0	N.A.	0
IMPDH1	-0,482649106	-0,252253303	0	N.A.	0
KIAA1524	-0,482717241	-0,398804799	0	N.A.	0
MRS2	-0,482905761	-0,301454198	0	N.A.	0
ADCK3	-0,483226477	-0,209080321	0	N.A.	0
YWHAB	-0,483229788	-0,154444184	0	N.A.	0
TFB1M	-0,483318255	-0,182694802	0	N.A.	0
pk	-0,483498445	-0,354268242	0	N.A.	0
TXNL4A	-0,483559308	-0,012427573	0	N.A.	0
FBXO22	-0,483679271	-0,404762233	0	N.A.	0
WDR34	-0,483690597	-0,048127013	0	N.A.	0
UBE2V2	-0,484647609	-0,480308922	0	N.A.	0
UXT	-0,485087109	-0,540965074	0	N.A.	0
KNSTRN	-0,485215458	-0,47008035	0	N.A.	0
DARS	-0,485500013	-0,221531869	0	N.A.	0
APIP	-0,485734087	-0,530373089	0	N.A.	0
CDC26	-0,485840406	-0,169272183	0	N.A.	0
LLPH	-0,485986633	-0,230764352	0	N.A.	0
NDC1	-0,48600044	-0,304935891	0	N.A.	0
CAPRIN1	-0,486045404	-0,287359454	0	N.A.	0
HNRNPA0	-0,486049175	-0,557308524	0	N.A.	0
PPT1	-0,486131712	-0,015056706	0	N.A.	0
NDUFB4	-0,48622682	-0,179720621	0	N.A.	0
GART	-0,486353154	-0,378412561	0	N.A.	0
KIF4A	-0,486675533	-0,324951701	0	N.A.	0
URM1	-0,486760849	-0,160207507	0	N.A.	0
HENMT1	-0,486997838	-0,481112781	0	N.A.	0

MYEOV2	-0,487040345	-0,414528459	0	N.A.	0
ECT2	-0,48730621	-0,143113942	0	N.A.	0
IARS2	-0,487421694	0,169039661	0	N.A.	0
WDR12	-0,487575709	-0,350875915	0	N.A.	0
ARHGAP11A	-0,488303124	-0,342105837	0	N.A.	0
DIAPH3	-0,48854286	-0,377484367	0	N.A.	0
HACL1	-0,488717042	-0,42847465	0	N.A.	0
DDT	-0,488963912	-0,312393185	0	N.A.	0
CHAF1A	-0,48902735	-0,093758787	0	N.A.	0
BUB1	-0,489166108	-0,33551227	0	N.A.	0
C7orf55	-0,489362828	-0,392832324	0	N.A.	0
DSN1	-0,489561205	-0,177940594	0	N.A.	0
HGH1	-0,489572193	-0,35164033	0	N.A.	0
NDUFB5	-0,489900445	-0,372894054	0	N.A.	0
UBXN4	-0,49026453	0,098752409	0	N.A.	0
TACC3	-0,490421597	-0,335369745	0	N.A.	0
BCLAF1	-0,490714955	-0,154661034	0	N.A.	0
NDUFA1	-0,491028734	-0,410639263	0	N.A.	0
PMPCA	-0,491146324	-0,175077403	0	N.A.	0
ALYREF	-0,491264643	-0,102320142	0	N.A.	0
DARS2	-0,49159326	-0,103057411	0	N.A.	0
ACOT7	-0,491886909	-0,098112498	0	N.A.	0
SSR2	-0,492247668	-0,3267787	0	N.A.	0
FOXP4-AS1	-0,492460022	-0,517340393	0	N.A.	0
PDIA6	-0,492500091	0,007771181	0	N.A.	0
SUGT1	-0,492519795	-0,217322697	0	N.A.	0
RPP30	-0,492910238	-0,416574327	0	N.A.	0
LMNB2	-0,492959517	-0,250448589	0	N.A.	0
PTPMT1	-0,493025592	-0,289149458	0	N.A.	0
TIMELESS	-0,493395668	-0,352786193	0	N.A.	0
TCEAL4	-0,493646858	-0,47187105	0	N.A.	0
MPV17L2	-0,493674799	-0,057571008	0	N.A.	0
CENPP	-0,494027833	-0,389749244	0	N.A.	0

E2F1	-0,49404464	-0,250429938	0	N.A.	0
GMDS	-0,494943148	-0,251994548	0	N.A.	0
PFDN4	-0,494993339	-0,445196717	0	N.A.	0
ICT1	-0,495144817	-0,273868383	0	N.A.	0
CCDC59	-0,495313893	-0,064533076	0	N.A.	0
RBM25	-0,495314185	0,246921786	0	N.A.	0
FAM104B	-0,4959078	-0,433276704	0	N.A.	0
NUP93	-0,495974281	-0,450492811	0	N.A.	0
NEK2	-0,496352925	-0,332224218	0	N.A.	0
UCHL5	-0,496407008	-0,51736257	0	N.A.	0
IMP3	-0,496447534	-0,067682259	0	N.A.	0
BCL2L12	-0,496675529	-0,276033252	0	N.A.	0
DDOST	-0,497214753	-0,152287411	0	N.A.	0
ARMC6	-0,497217367	-0,091559162	0	N.A.	0
CNN3	-0,497399861	-0,459163252	0	N.A.	0
HTATSF1	-0,497447955	-0,059062891	0	N.A.	0
DDX21	-0,497592971	-0,090933503	0	N.A.	0
FKBP4	-0,49767179	-0,281534049	0	N.A.	0
LAMTOR2	-0,497779702	-0,21883515	0	N.A.	0
MELK	-0,497794565	-0,324606948	0	N.A.	0
C16orf59	-0,498172353	-0,275817519	0	N.A.	0
SUMO1	-0,498200761	-0,450863045	0	N.A.	0
E2F4	-0,499013826	0,001252715	0	N.A.	0
NAA15	-0,49931842	-0,080110591	0	N.A.	0
PYCR2	-0,499321897	-0,11436949	0	N.A.	0
WDR43	-0,49944479	-0,22565387	0	N.A.	0
WDR18	-0,499638346	-0,235336666	0	N.A.	0
PIH1D1	-0,499741282	-0,27551764	0	N.A.	0
CCAR1	-0,499778388	-0,092113575	0	N.A.	0
GNL2	-0,499786304	-0,402950491	0	N.A.	0
DECR1	-0,499979267	-0,471985091	0	N.A.	0
CLSPN	-0,500174448	-0,356550348	0	N.A.	0
CMC1	-0,500252529	-0,281651907	0	N.A.	0

N6AMT2	-0,500485724	-0,129019631	0	N.A.	0
ASUN	-0,500648878	-0,12872671	0	N.A.	0
CENPA	-0,500680299	-0,434983693	0	N.A.	0
STARD7	-0,500792875	-0,251048406	0	N.A.	0
ADRM1	-0,500881953	-0,18933095	0	N.A.	0
CDC5L	-0,501001105	-0,269875706	0	N.A.	0
DNAJC7	-0,501335188	-0,135992341	0	N.A.	0
TRA2B	-0,501348887	-0,332545464	0	N.A.	0
HIST1H4C	-0,501462658	-0,510237832	0	N.A.	0
THRAP3	-0,501883579	-0,031911977	0	N.A.	0
NUDT21	-0,501923804	-0,418315527	0	N.A.	0
BAZ1B	-0,502125749	-0,169560239	0	N.A.	0
PRR11	-0,502205455	-0,363978927	0	N.A.	0
RACGAP1	-0,502350665	-0,400459596	0	N.A.	0
CPSF6	-0,502490319	-0,389535933	0	N.A.	0
G3BP1	-0,502830973	-0,190504223	0	N.A.	0
APOA1BP	-0,503076956	-0,139069513	0	N.A.	0
PSMC3	-0,503572673	-0,453109732	0	N.A.	0
PHF14	-0,503734656	0,054945858	0	N.A.	0
FAM83D	-0,503995922	-0,351667593	0	N.A.	0
WBSCR16	-0,504356493	-0,106205222	0	N.A.	0
GTPBP4	-0,504596016	-0,274972351	0	N.A.	0
PSMD1	-0,504654709	0,078736826	0	N.A.	0
AAMP	-0,504721442	-0,095715253	0	N.A.	0
EMC9	-0,504923964	-0,393259479	0	N.A.	0
RPF2	-0,504981512	-0,356044956	0	N.A.	0
CENPV	-0,504995922	0,220847101	0	N.A.	0
ENO1	-0,505045695	-0,36616126	0	N.A.	0
SLC25A11	-0,505664081	-0,182318585	0	N.A.	0
SEC11A	-0,505681201	-0,383372835	0	N.A.	0
NCAPG	-0,505703375	-0,334084048	0	N.A.	0
RNPS1	-0,505778602	-0,232492063	0	N.A.	0
MAPK13	-0,505959855	-0,219610643	0	N.A.	0

TMEM126B	-0,50598124	-0,27682972	0	N.A.	0
DLGAP5	-0,506029802	-0,317144407	0	N.A.	0
ALG5	-0,506168317	0,025617853	0	N.A.	0
ALDH2	-0,50618307	-0,199481186	0	N.A.	0
PSMD7	-0,506487605	-0,0385167	0	N.A.	0
GTSE1	-0,507024836	-0,343780698	0	N.A.	0
BABAM1	-0,507176039	-0,331399935	0	N.A.	0
PRTFDC1	-0,507272107	-0,537072214	0	N.A.	0
HARS	-0,507333707	-0,431343587	0	N.A.	0
CNPY2	-0,50764082	-0,060302456	0	N.A.	0
ETFB	-0,507714138	-0,164412113	0	N.A.	0
NIP7	-0,507740988	-0,456991168	0	N.A.	0
PFKM	-0,50821738	-0,330760924	0	N.A.	0
GTF2A2	-0,508246566	-0,243984053	0	N.A.	0
SRP14	-0,508371195	-0,274728657	0	N.A.	0
SMARCE1	-0,508420653	-0,349945081	0	N.A.	0
CSTF3	-0,508432004	-0,221454365	0	N.A.	0
WDR46	-0,508564962	-0,289616392	0	N.A.	0
SNX17	-0,508645535	-0,30781931	0	N.A.	0
USP1	-0,508662691	-0,288852866	0	N.A.	0
ABHD14B	-0,508727188	-0,342021838	0	N.A.	0
UHRF1	-0,508870254	-0,370405539	0	N.A.	0
UPF3A	-0,509759641	-0,165709397	0	N.A.	0
C7orf55-LUC	-0,51005428	-0,198004467	0	N.A.	0
UQCRQ	-0,510846459	-0,453269224	0	N.A.	0
ELFN1-AS1	-0,510919528	-0,442102366	0	N.A.	0
SPDL1	-0,511015167	-0,276202341	0	N.A.	0
RBM28	-0,511161113	-0,079880732	0	N.A.	0
RP11-1094M	-0,511286608	-0,371780457	0	N.A.	0
KIAA0020	-0,511637121	-0,22611107	0	N.A.	0
EIF2B3	-0,511659494	-0,126561955	0	N.A.	0
SPC24	-0,511678867	-0,426711419	0	N.A.	0
ECHDC1	-0,511793584	-0,54782397	0	N.A.	0

MZT2B	-0,511812823	-0,20366131	0	N.A.	0
PDCL3	-0,511860734	-0,485577673	0	N.A.	0
PSMD8	-0,511893843	-0,380895014	0	N.A.	0
NCAPG2	-0,511896924	-0,30748664	0	N.A.	0
MTERF3	-0,511963552	-0,160941476	0	N.A.	0
BCL7C	-0,512007542	-0,251247899	0	N.A.	0
NARS2	-0,512196552	-0,512184245	0	N.A.	0
PCCB	-0,512276728	-0,326829714	0	N.A.	0
CENPM	-0,512361432	-0,308369029	0	N.A.	0
DEPDC1B	-0,512634573	-0,449265588	0	N.A.	0
SUPT16H	-0,512660838	-0,131004483	0	N.A.	0
ECH1	-0,512925732	-0,263233875	0	N.A.	0
ILKAP	-0,513211489	-0,353120368	0	N.A.	0
PSMG4	-0,513422482	-0,244749429	0	N.A.	0
NR2C2AP	-0,513847714	-0,391216181	0	N.A.	0
SRPRB	-0,514356769	-0,218322812	0	N.A.	0
GPN3	-0,51445329	-0,401599878	0	N.A.	0
AATF	-0,514523277	-0,315225904	0	N.A.	0
CPSF3	-0,514581305	-0,285492432	0	N.A.	0
ZNHIT3	-0,514751666	-0,312230423	0	N.A.	0
TBRG4	-0,514920998	-0,239667395	0	N.A.	0
DRG1	-0,515032237	-0,339773476	0	N.A.	0
HELLS	-0,515370221	-0,278311755	0	N.A.	0
PMF1	-0,515503363	-0,194647029	0	N.A.	0
COA3	-0,515784376	-0,344657296	0	N.A.	0
C1orf131	-0,516291763	-0,045463571	0	N.A.	0
BAX	-0,516390232	-0,62822489	0	N.A.	0
MNS1	-0,516406705	-0,322024394	0	N.A.	0
HMMR	-0,516422838	-0,404568974	0	N.A.	0
HMGN5	-0,516771984	-0,522923793	0	N.A.	0
POLR2D	-0,516846227	-0,329160168	0	N.A.	0
GLRX5	-0,516955736	0,003111326	0	N.A.	0
SF3B2	-0,517095452	-0,227721762	0	N.A.	0

DTD1	-0,517174478	-0,255783039	0	N.A.	0
GPX2	-0,517191561	-0,344712896	0	N.A.	0
C2orf47	-0,51736622	-0,410372627	0	N.A.	0
ALG3	-0,517398981	-0,067393315	0	N.A.	0
TFAP4	-0,517544353	-0,41433957	0	N.A.	0
FAM64A	-0,51762708	-0,447874482	0	N.A.	0
LAMTOR4	-0,517686238	-0,270263622	0	N.A.	0
DBF4	-0,517761709	-0,415333842	0	N.A.	0
XRCC2	-0,518177637	-0,356764508	0	N.A.	0
RIOK1	-0,518538566	-0,400905642	0	N.A.	0
YDJC	-0,518653039	-0,087354964	0	N.A.	0
PRIM1	-0,518725369	-0,453729643	0	N.A.	0
AKR7A2	-0,519239797	-0,2784853	0	N.A.	0
CDCA3	-0,519302526	-0,333166098	0	N.A.	0
DCTN3	-0,519379462	-0,466493957	0	N.A.	0
PSME2	-0,519578054	-0,380261822	0	N.A.	0
GALK1	-0,519616571	0,21056616	0	N.A.	0
PPIF	-0,519735445	-0,345589644	0	N.A.	0
CETN2	-0,520387697	-0,478883244	0	N.A.	0
CDC6	-0,520439743	-0,380841738	0	N.A.	0
PRC1	-0,52046809	-0,295068334	0	N.A.	0
MYBL2	-0,52051025	-0,340120902	0	N.A.	0
B9D1	-0,520600232	-0,418698132	0	N.A.	0
DLD	-0,520603408	-0,267925784	0	N.A.	0
APEH	-0,520674736	-0,106421144	0	N.A.	0
COQ2	-0,521047645	-0,270975621	0	N.A.	0
EEF1B2	-0,521171838	-0,678420843	0	N.A.	0
EIF3F	-0,521177749	-0,549483433	0	N.A.	0
AURKB	-0,521206043	-0,402458318	0	N.A.	0
MTCH1	-0,521293716	-0,264568062	0	N.A.	0
NDUFA12	-0,521311048	-0,365080343	0	N.A.	0
PLK1	-0,521360725	-0,382698039	0	N.A.	0
COQ3	-0,521662432	-0,382225223	0	N.A.	0

UBE2C	-0,522199427	-0,455368214	0	N.A.	0
IDH3A	-0,522229873	-0,373690908	0	N.A.	0
C6orf203	-0,52249882	-0,066787902	0	N.A.	0
NOL11	-0,522900675	-0,196290092	0	N.A.	0
RBM14	-0,522909141	0,063606312	0	N.A.	0
KRTCAP3	-0,522930211	0,010848185	0	N.A.	0
CHEK2	-0,523111782	-0,52905332	0	N.A.	0
TARDBP	-0,523340955	-2,6077E-05	0	N.A.	0
EEF1D	-0,5234451	-0,629231565	0	N.A.	0
HAUS7	-0,523534838	-0,277320148	0	N.A.	0
NAA38	-0,523670186	-0,482262771	0	N.A.	0
ECSIT	-0,523704749	-0,363877566	0	N.A.	0
G6PC3	-0,523821016	-0,229091845	0	N.A.	0
NUSAP1	-0,524092014	-0,366976386	0	N.A.	0
RABEPK	-0,524275113	-0,316128929	0	N.A.	0
PRDX1	-0,524344971	-0,439193922	0	N.A.	0
CHCHD6	-0,524372288	-0,282857081	0	N.A.	0
CEP55	-0,524397772	-0,37545891	0	N.A.	0
GGH	-0,524621669	-0,206944421	0	N.A.	0
DPH2	-0,525138798	-0,225268627	0	N.A.	0
IFRD2	-0,525265545	-0,290648893	0	N.A.	0
CD3EAP	-0,525282777	-0,391540854	0	N.A.	0
DDX54	-0,525288253	-0,392597839	0	N.A.	0
RRP9	-0,525486696	-0,239870019	0	N.A.	0
TUBB	-0,525535937	-0,466722586	0	N.A.	0
ALDH1B1	-0,525553419	-0,297309454	0	N.A.	0
MTHFD1	-0,525634336	-0,368726945	0	N.A.	0
TSEN15	-0,526387579	-0,348719674	0	N.A.	0
NLE1	-0,526423832	-0,486259349	0	N.A.	0
C4orf27	-0,526702369	-0,504815401	0	N.A.	0
POLDIP2	-0,526871825	-0,261622504	0	N.A.	0
RPA2	-0,527201869	-0,447992842	0	N.A.	0
LIG1	-0,527254703	-0,355599872	0	N.A.	0

SEPHS1	-0,527304242	-0,381024591	0	N.A.	0
CCDC74A	-0,527374492	-0,16634889	0	N.A.	0
COX7C	-0,527423221	-0,522851784	0	N.A.	0
TRMT10C	-0,52758896	-0,365286519	0	N.A.	0
BOP1	-0,527656899	-0,242159384	0	N.A.	0
RRM2	-0,527887571	-0,444541885	0	N.A.	0
ARMC10	-0,52801594	-0,102445998	0	N.A.	0
CENPE	-0,528051356	-0,250430957	0	N.A.	0
BID	-0,528902391	-0,154290946	0	N.A.	0
TUBG1	-0,529057353	-0,236834378	0	N.A.	0
COPE	-0,529284223	-0,280581227	0	N.A.	0
IMMT	-0,52941132	-0,13292697	0	N.A.	0
RBMX2	-0,529653958	-0,268739598	0	N.A.	0
SRSF11	-0,529747849	0,215328961	0	N.A.	0
OIP5	-0,530162026	-0,355720186	0	N.A.	0
MPLKIP	-0,530716259	-0,190398812	0	N.A.	0
GSPT1	-0,530759802	-0,212659134	0	N.A.	0
ASNA1	-0,530785846	-0,21946481	0	N.A.	0
NIPSNAP1	-0,530802627	-0,151442675	0	N.A.	0
FABP6	-0,530998423	-0,441395065	0	N.A.	0
SGOL1	-0,53103347	-0,363065571	0	N.A.	0
VARS	-0,531055874	-0,271244972	0	N.A.	0
SPC25	-0,531116963	-0,426497969	0	N.A.	0
PSMC2	-0,531380348	-0,16342488	0	N.A.	0
DENR	-0,531522902	-0,365054668	0	N.A.	0
PCMT1	-0,531657302	-0,122209047	0	N.A.	0
TMEM106C	-0,531803276	-0,105353975	0	N.A.	0
SMC1A	-0,532014594	-0,143300983	0	N.A.	0
POP5	-0,532101292	-0,365835245	0	N.A.	0
NSUN5	-0,532230489	0,098735637	0	N.A.	0
NEDD8	-0,532319053	-0,4046746	0	N.A.	0
MLST8	-0,532448551	-0,273439832	0	N.A.	0
UQCR10	-0,53252828	-0,392222259	0	N.A.	0

PARL	-0,533102357	-0,225126722	0	N.A.	0
PUS7L	-0,533260704	-0,221283322	0	N.A.	0
PCID2	-0,533986239	-0,217521614	0	N.A.	0
PSMF1	-0,534020465	-0,231389444	0	N.A.	0
PSMD2	-0,534064121	-0,127789223	0	N.A.	0
RPUSD3	-0,534656767	0,029379963	0	N.A.	0
UQCRB	-0,534737079	-0,580190524	0	N.A.	0
ARL6IP4	-0,534956887	-0,280869938	0	N.A.	0
HNRNPAB	-0,535044262	-0,192711911	0	N.A.	0
SAMM50	-0,535083307	-0,255234115	0	N.A.	0
RPP40	-0,535347083	-0,472783034	0	N.A.	0
GRPEL1	-0,535891979	-0,342914173	0	N.A.	0
EZH2	-0,53644133	-0,357164716	0	N.A.	0
UPF3B	-0,536524433	-0,1277548	0	N.A.	0
NDUFB1	-0,536596867	-0,406557811	0	N.A.	0
CTSC	-0,537103226	-0,509498249	0	N.A.	0
ADH5	-0,537221814	-0,548696464	0	N.A.	0
RFC4	-0,537248175	-0,470074638	0	N.A.	0
ENOPH1	-0,537438265	-0,441008728	0	N.A.	0
CCDC86	-0,53792965	-0,489042295	0	N.A.	0
UBB	-0,538280717	-0,371298092	0	N.A.	0
FANCI	-0,538493358	-0,424225767	0	N.A.	0
AKR1A1	-0,538692903	-0,082843957	0	N.A.	0
GTF2H5	-0,538826817	-0,36089869	0	N.A.	0
DHFR	-0,539091596	-0,393661551	0	N.A.	0
RFC5	-0,539274877	-0,388184163	0	N.A.	0
GLO1	-0,539587924	-0,539964319	0	N.A.	0
ASF1B	-0,539963266	-0,387607831	0	N.A.	0
GEMIN6	-0,540041804	-0,348175595	0	N.A.	0
MKI67	-0,540062031	-0,205144352	0	N.A.	0
COQ9	-0,540076782	-0,238049642	0	N.A.	0
POLR2L	-0,540396872	-0,456960444	0	N.A.	0
C9orf78	-0,540487117	-0,234292788	0	N.A.	0

TOMM34	-0,540524313	-0,407981472	0	N.A.	0
PYURF	-0,540783992	-0,520999511	0	N.A.	0
AIMP1	-0,541417769	-0,145993684	0	N.A.	0
CHEK1	-0,541588705	-0,456809638	0	N.A.	0
CIRH1A	-0,541908389	-0,280376778	0	N.A.	0
CKS1B	-0,541953421	-0,412170075	0	N.A.	0
CD320	-0,541987811	-0,184442291	0	N.A.	0
ERP29	-0,542010797	-0,224661163	0	N.A.	0
SNX5	-0,542052044	-0,361072255	0	N.A.	0
ATAD2	-0,54235006	-0,178776337	0	N.A.	0
CHCHD1	-0,542357964	-0,216847988	0	N.A.	0
NME4	-0,542431453	-0,364226062	0	N.A.	0
KAT2A	-0,5427193	0,004500844	0	N.A.	0
CCDC124	-0,542782468	-0,210032037	0	N.A.	0
HSD17B4	-0,542800161	-0,404739828	0	N.A.	0
PSMD11	-0,543014618	-0,239230367	0	N.A.	0
LRRCC1	-0,543154536	-0,335381577	0	N.A.	0
PTMS	-0,543215302	-0,306966805	0	N.A.	0
AP2S1	-0,543384413	-0,52909414	0	N.A.	0
RRS1	-0,544346877	-0,060526151	0	N.A.	0
COX8A	-0,544377635	-0,411307657	0	N.A.	0
TXN2	-0,544384544	-0,366175963	0	N.A.	0
HIBCH	-0,544428962	-0,492573105	0	N.A.	0
MZT2A	-0,544575676	-0,397683448	0	N.A.	0
SEPT7	-0,544869197	-0,41318731	0	N.A.	0
CFAP20	-0,54552417	-0,259510689	0	N.A.	0
GRSF1	-0,545591972	-0,278802604	0	N.A.	0
GLRX3	-0,545631382	-0,348877386	0	N.A.	0
BCS1L	-0,545818098	-0,339477398	0	N.A.	0
ZWILCH	-0,545845379	-0,480246454	0	N.A.	0
TMEM14A	-0,545947553	-0,037444352	0	N.A.	0
CSNK2B	-0,545954506	-0,212626334	0	N.A.	0
CDC37	-0,545977958	-0,189700174	0	N.A.	0

TOR3A	-0,546104067	-0,371656862	0	N.A.	0
CTPS1	-0,546230014	-0,287704996	0	N.A.	0
TXN	-0,546268949	-0,429700017	0	N.A.	0
GDI2	-0,546651221	-0,443650077	0	N.A.	0
H3F3A	-0,546768101	-0,386940398	0	N.A.	0
NSA2	-0,546779494	-0,663856596	0	N.A.	0
EIF2S1	-0,546842626	-0,374205092	0	N.A.	0
BTF3	-0,547114992	-0,554594735	0	N.A.	0
DCXR	-0,547155529	-0,236080582	0	N.A.	0
ITPA	-0,54755335	-0,213328016	0	N.A.	0
C19orf70	-0,547606128	-0,454912822	0	N.A.	0
PES1	-0,547813858	-0,103180055	0	N.A.	0
YWHAE	-0,547823543	-0,1994243	0	N.A.	0
MRFAP1	-0,548185394	-0,192771109	0	N.A.	0
CENPU	-0,548282151	-0,430001795	0	N.A.	0
DGUOK	-0,54833289	-0,257696703	0	N.A.	0
DNAJC9	-0,548600368	-0,382185534	0	N.A.	0
FOXM1	-0,548619509	-0,324903207	0	N.A.	0
EIF3G	-0,548854486	-0,507499677	0	N.A.	0
PBK	-0,549297082	-0,448354926	0	N.A.	0
PPIB	-0,549571679	-0,315160902	0	N.A.	0
C21orf59	-0,549813797	-0,033034087	0	N.A.	0
AP2M1	-0,549984886	-0,140620704	0	N.A.	0
FXN	-0,550154651	-0,544117453	0	N.A.	0
TMX2	-0,550284564	0,120375619	0	N.A.	0
BOD1	-0,55053633	-0,515083925	0	N.A.	0
TOP2A	-0,550845935	-0,266507102	0	N.A.	0
RBBP4	-0,550949234	-0,478633798	0	N.A.	0
HRSP12	-0,551122394	-0,452096242	0	N.A.	0
CCNA2	-0,55143715	-0,401821708	0	N.A.	0
MCM6	-0,551746066	-0,164207611	0	N.A.	0
C11orf58	-0,552040935	-0,547254098	0	N.A.	0
MYCBP	-0,552072363	-0,357082213	0	N.A.	0

PTCD3	-0,552268729	-0,16252884	0	N.A.	0
ATXN10	-0,552320514	-0,45847077	0	N.A.	0
PRDX6	-0,552344224	-0,53168157	0	N.A.	0
TK1	-0,552793909	-0,3916313	0	N.A.	0
EXOSC7	-0,552882775	-0,348378753	0	N.A.	0
SRRT	-0,553103847	-0,138485284	0	N.A.	0
BLMH	-0,553779194	-0,589851897	0	N.A.	0
TIMM23	-0,553782603	-0,408244301	0	N.A.	0
KIF22	-0,553895167	-0,275133932	0	N.A.	0
EIF1AX	-0,554258486	-0,359730987	0	N.A.	0
FEN1	-0,554429744	-0,355093685	0	N.A.	0
SLBP	-0,554477835	-0,326430135	0	N.A.	0
HNRNPA1L2	-0,554510204	-0,36202127	0	N.A.	0
AAAS	-0,554884639	-0,391196736	0	N.A.	0
NUDT15	-0,555075165	-0,474222041	0	N.A.	0
TFB2M	-0,555172209	-0,415634119	0	N.A.	0
LTV1	-0,55527731	-0,512860318	0	N.A.	0
PSMG2	-0,555497949	-0,383048666	0	N.A.	0
SNRPA1	-0,555503642	-0,262748549	0	N.A.	0
NDUFS2	-0,555871115	-0,082100617	0	N.A.	0
CKAP2	-0,55594037	-0,458007609	0	N.A.	0
TIMM8B	-0,5560712	-0,466387833	0	N.A.	0
MAZ	-0,556568169	-0,198845223	0	N.A.	0
NTPCR	-0,556642202	-0,437718939	0	N.A.	0
LSM8	-0,557134653	-0,474041417	0	N.A.	0
TECR	-0,557192257	0,113049129	0	N.A.	0
COX11	-0,557424771	-0,426020228	0	N.A.	0
KHDRBS1	-0,557494523	-0,369936706	0	N.A.	0
TWISTNB	-0,557602016	-0,198703743	0	N.A.	0
CDT1	-0,557633662	-0,362163395	0	N.A.	0
CDCA5	-0,557681475	-0,379908919	0	N.A.	0
IDH3B	-0,557969598	-0,380264225	0	N.A.	0
MGST1	-0,557987067	-0,225208257	0	N.A.	0

CCNB2	-0,558065388	-0,460629163	0	N.A.	0
SLC25A3	-0,558126991	-0,377390053	0	N.A.	0
PHF19	-0,558347508	-0,417369768	0	N.A.	0
HNRNPH3	-0,558427659	-0,458765039	0	N.A.	0
DDX56	-0,558474228	-0,41502213	0	N.A.	0
UQCRC1	-0,558542249	-0,212261203	0	N.A.	0
NUTF2	-0,558610599	-0,285317828	0	N.A.	0
YWHAQ	-0,558735118	-0,241908074	0	N.A.	0
TERF1	-0,558887176	-0,151681734	0	N.A.	0
MFF	-0,559158057	-0,297423403	0	N.A.	0
POC1A	-0,559327339	-0,375445479	0	N.A.	0
COX7B	-0,559505395	-0,461526705	0	N.A.	0
PSMB6	-0,559605166	-0,454704504	0	N.A.	0
DSCC1	-0,559782538	-0,453560972	0	N.A.	0
MND1	-0,559892065	-0,459225271	0	N.A.	0
DCUN1D5	-0,560006501	-0,446163483	0	N.A.	0
CCDC58	-0,560111517	-0,395737893	0	N.A.	0
DAP3	-0,560800615	-0,424396977	0	N.A.	0
MAGEF1	-0,561996592	-0,378185698	0	N.A.	0
NHP2L1	-0,562112869	-0,530069123	0	N.A.	0
UQCRFS1	-0,562242895	-0,430756354	0	N.A.	0
KLHDC3	-0,562303181	-0,394069396	0	N.A.	0
KPNA2	-0,562323235	-0,435522435	0	N.A.	0
UQCRC2	-0,562330902	-0,413118923	0	N.A.	0
NUF2	-0,562395859	-0,302205385	0	N.A.	0
STIP1	-0,5625488	-0,26503112	0	N.A.	0
SKA3	-0,562653277	-0,490423521	0	N.A.	0
C14orf2	-0,562939624	-0,542143851	0	N.A.	0
CCNB1	-0,562961892	-0,444945962	0	N.A.	0
RCN1	-0,563238906	-0,24275832	0	N.A.	0
TIMM8A	-0,56325358	-0,339778846	0	N.A.	0
TIMM50	-0,5633055	-0,441276664	0	N.A.	0
SLC25A10	-0,56376413	-0,127365614	0	N.A.	0

EXOSC3	-0,564303129	-0,385968768	0	N.A.	0
SDHC	-0,564532375	-0,540035665	0	N.A.	0
SMARCC1	-0,564571457	-0,200751399	0	N.A.	0
TYMS	-0,56512514	-0,526957044	0	N.A.	0
COX4I1	-0,565353008	-0,392393658	0	N.A.	0
MALSU1	-0,565375049	-0,386908712	0	N.A.	0
IMMP1L	-0,565394871	-0,367295872	0	N.A.	0
HDAC1	-0,565725449	-0,077091589	0	N.A.	0
COMMD8	-0,566195813	-0,316370011	0	N.A.	0
RAD51AP1	-0,566444118	-0,434315864	0	N.A.	0
NOP56	-0,567166624	-0,251894733	0	N.A.	0
NOL7	-0,567663091	-0,354402339	0	N.A.	0
RCC1	-0,567811446	-0,309540101	0	N.A.	0
MZT1	-0,568106496	-0,450485902	0	N.A.	0
POP7	-0,568203645	-0,440608603	0	N.A.	0
HINT2	-0,568318893	-0,264925613	0	N.A.	0
HMBS	-0,568515501	-0,390988458	0	N.A.	0
UQCC2	-0,568625144	-0,583595748	0	N.A.	0
CDC20	-0,568744347	-0,370327009	0	N.A.	0
GBAS	-0,568972623	-0,535523067	0	N.A.	0
UCHL3	-0,569129098	-0,136998777	0	N.A.	0
RAD51C	-0,569533534	-0,369318464	0	N.A.	0
BCCIP	-0,569746822	-0,35067299	0	N.A.	0
HAT1	-0,569871232	-0,289079099	0	N.A.	0
MKKS	-0,570035848	-0,2210748	0	N.A.	0
POLR2F	-0,570102883	-0,503406543	0	N.A.	0
SMC2	-0,570197481	-0,191810558	0	N.A.	0
PSMD13	-0,570288975	-0,197487273	0	N.A.	0
HES6	-0,570559183	-0,378840231	0	N.A.	0
CDCA7L	-0,570595269	-0,570703564	0	N.A.	0
PRKDC	-0,570706615	-0,083534411	0	N.A.	0
UTP18	-0,570718991	-0,336426448	0	N.A.	0
PNO1	-0,570962226	-0,213750887	0	N.A.	0

NDUFA11	-0,571111271	-0,413748658	0	N.A.	0
PPIG	-0,571126409	-0,0446967	0	N.A.	0
SF3A3	-0,571273173	-0,474705312	0	N.A.	0
TMA16	-0,571324436	-0,25210684	0	N.A.	0
CBX5	-0,571372337	-0,291057233	0	N.A.	0
SAC3D1	-0,571712111	-0,228878288	0	N.A.	0
MCM2	-0,571914923	-0,26043133	0	N.A.	0
TBCA	-0,572144953	-0,41580612	0	N.A.	0
KIAA0101	-0,572311867	-0,512659026	0	N.A.	0
COPS3	-0,572572384	-0,405969658	0	N.A.	0
EIF5A	-0,572871552	-0,557385437	0	N.A.	0
GSS	-0,572899011	-0,087298525	0	N.A.	0
RARS	-0,572980429	-0,032478301	0	N.A.	0
PSMG3	-0,573464952	-0,272933343	0	N.A.	0
LUC7L3	-0,573464984	0,035908126	0	N.A.	0
PYCR1	-0,574045202	-0,315248965	0	N.A.	0
ANAPC11	-0,57433739	-0,329527347	0	N.A.	0
TCOF1	-0,574473311	-0,414194558	0	N.A.	0
GPATCH4	-0,574478828	-0,265016863	0	N.A.	0
RFC2	-0,57489471	-0,444056861	0	N.A.	0
DEK	-0,574904962	-0,159328397	0	N.A.	0
TOMM22	-0,575009278	-0,513785852	0	N.A.	0
PHF20	-0,575615242	-0,245975063	0	N.A.	0
PSMB7	-0,576014663	-0,420150854	0	N.A.	0
ABCE1	-0,576118141	-0,311755321	0	N.A.	0
TPX2	-0,576247122	-0,448924223	0	N.A.	0
SRP72	-0,576777091	-0,285217524	0	N.A.	0
CHCHD2	-0,577060753	-0,446890084	0	N.A.	0
KIF20B	-0,577066361	-0,31142375	0	N.A.	0
DHRS4	-0,577234142	-0,241715237	0	N.A.	0
C8orf59	-0,577514875	-0,311796788	0	N.A.	0
VPS29	-0,578848517	-0,374011556	0	N.A.	0
PSMD6	-0,578944365	-0,365183224	0	N.A.	0

EIF3M	-0,578957581	-0,533774593	0	N.A.	0
RSL1D1	-0,579454951	-0,490302294	0	N.A.	0
TKT	-0,579459882	-0,431575571	0	N.A.	0
NONO	-0,579565164	-0,567410597	0	N.A.	0
DPM1	-0,57968125	-0,310004318	0	N.A.	0
CENPF	-0,580365678	-0,189082737	0	N.A.	0
SKP2	-0,580452338	-0,433635104	0	N.A.	0
RAE1	-0,580456835	-0,384876026	0	N.A.	0
UQCRH	-0,580492462	-0,602488632	0	N.A.	0
CENPK	-0,580763749	-0,479634539	0	N.A.	0
WDR74	-0,580813166	-0,243904889	0	N.A.	0
RRP15	-0,581055034	-0,017347943	0	N.A.	0
TXNDC12	-0,581278033	-0,234447433	0	N.A.	0
COMMD4	-0,581741035	-0,514335147	0	N.A.	0
CBR1	-0,58188535	-0,128748442	0	N.A.	0
LHX4-AS1	-0,582024925	-0,382213108	0	N.A.	0
RFC3	-0,582332538	-0,479122245	0	N.A.	0
GOT2	-0,582369813	-0,364451977	0	N.A.	0
NIFK	-0,582619647	-0,384636643	0	N.A.	0
OAT	-0,58287906	-0,214129922	0	N.A.	0
VRK1	-0,582982886	-0,412951608	0	N.A.	0
NSMCE1	-0,583065554	-0,499485046	0	N.A.	0
COX6C	-0,583162046	-0,441248701	0	N.A.	0
PSMA7	-0,583172226	-0,422020847	0	N.A.	0
SHMT1	-0,583314607	-0,271705394	0	N.A.	0
SNRPB2	-0,583336533	-0,35161918	0	N.A.	0
PDCD2	-0,583346607	-0,381360462	0	N.A.	0
PRDX4	-0,583692514	-0,225090357	0	N.A.	0
PUF60	-0,583744737	-0,362623022	0	N.A.	0
ATAD3A	-0,583787973	-0,343892376	0	N.A.	0
PARPBP	-0,584142903	-0,423285468	0	N.A.	0
NDUFC1	-0,584658575	-0,41026447	0	N.A.	0
FARSB	-0,584896892	-0,336339906	0	N.A.	0

UBE2N	-0,585162608	-0,520374519	0	N.A.	0
ENY2	-0,585167617	-0,453408738	0	N.A.	0
PHF5A	-0,586019046	-0,385588642	0	N.A.	0
LMNB1	-0,586111682	-0,492625788	0	N.A.	0
PSMB2	-0,586451461	-0,475043822	0	N.A.	0
PIN1	-0,586642327	-0,214471239	0	N.A.	0
ORC6	-0,586822499	-0,32751364	0	N.A.	0
KPNB1	-0,586885711	-0,073465177	0	N.A.	0
RNPEP	-0,586996835	0,047713235	0	N.A.	0
CTC-524C5.2	-0,587210933	-0,181126803	0	N.A.	0
ITGAE	-0,587510097	-0,573022707	0	N.A.	0
UROS	-0,587664737	-0,307241728	0	N.A.	0
ATP5I	-0,5877946	-0,545888805	0	N.A.	0
LAS1L	-0,587823013	-0,228422577	0	N.A.	0
RMI2	-0,587952647	-0,335900448	0	N.A.	0
C7orf50	-0,588092125	-0,531356106	0	N.A.	0
SIGMAR1	-0,588381588	-0,151357244	0	N.A.	0
ATP5H	-0,588556484	-0,422703992	0	N.A.	0
POLR2I	-0,588584556	-0,297750715	0	N.A.	0
NDUFB3	-0,588887354	-0,457456623	0	N.A.	0
RRM1	-0,589709846	-0,421318248	0	N.A.	0
NAP1L1	-0,590025181	-0,636504802	0	N.A.	0
ATP5D	-0,591197387	-0,505164043	0	N.A.	0
FARSA	-0,59135205	-0,251363129	0	N.A.	0
NDUFB7	-0,591539557	-0,281942518	0	N.A.	0
PTGES2	-0,591717646	-0,28609583	0	N.A.	0
TROAP	-0,591929821	-0,323079802	0	N.A.	0
RBM3	-0,591932	-0,526168627	0	N.A.	0
TRAPPC2L	-0,59296084	-0,338821212	0	N.A.	0
DPY30	-0,59351708	-0,362706256	0	N.A.	0
DBI	-0,593550503	-0,526266715	0	N.A.	0
SMC4	-0,593800477	-0,300982137	0	N.A.	0
PSMB4	-0,594433885	-0,373385793	0	N.A.	0

SUB1	-0,594560944	-0,424285559	0	N.A.	0
DNAJC2	-0,594848074	-0,364881558	0	N.A.	0
C12orf45	-0,594954539	-0,396891446	0	N.A.	0
HSPBP1	-0,595626252	-0,285015818	0	N.A.	0
SDHB	-0,595772901	-0,264336318	0	N.A.	0
LBR	-0,595929248	-0,111349759	0	N.A.	0
PSMB8	-0,5961774	-0,434722644	0	N.A.	0
ANP32E	-0,596302293	-0,41090371	0	N.A.	0
CENPH	-0,596710814	-0,554032846	0	N.A.	0
PSMD4	-0,596771064	-0,34121233	0	N.A.	0
NUP37	-0,597068118	-0,243015563	0	N.A.	0
COPRS	-0,597351278	-0,347074209	0	N.A.	0
ITGB3BP	-0,597574315	-0,421776233	0	N.A.	0
SNF8	-0,5977223	-0,294463749	0	N.A.	0
MCM5	-0,598092214	-0,281953311	0	N.A.	0
ECE2	-0,598282799	-0,426948995	0	N.A.	0
ALDH7A1	-0,598498144	-0,433336906	0	N.A.	0
ZWINT	-0,598625182	-0,411307759	0	N.A.	0
PSMB5	-0,598826609	-0,413113891	0	N.A.	0
POLR2K	-0,598854706	-0,550783895	0	N.A.	0
OLA1	-0,599281227	-0,467128678	0	N.A.	0
TSFM	-0,599426322	-0,461761566	0	N.A.	0
DAZAP1	-0,599548193	-0,192602621	0	N.A.	0
ADSL	-0,599968871	-0,475402666	0	N.A.	0
C16orf13	-0,600027873	-0,3873336	0	N.A.	0
MMAB	-0,600445518	-0,311867601	0	N.A.	0
TRUB2	-0,600493338	-0,391772596	0	N.A.	0
PPIL1	-0,600571422	-0,376916581	0	N.A.	0
NDUFA5	-0,600578712	-0,571684975	0	N.A.	0
FAM136A	-0,60069117	-0,176789783	0	N.A.	0
NOLC1	-0,602136448	-0,286053734	0	N.A.	0
TIMM10	-0,602158856	-0,497096446	0	N.A.	0
CENPW	-0,60224508	-0,423164262	0	N.A.	0

EIF4H	-0,602372411	-0,362008853	0	N.A.	0
SKA2	-0,602490689	-0,476444965	0	N.A.	0
HSPA14	-0,602502032	-0,200802453	0	N.A.	0
HSPA9	-0,602660094	-0,150657087	0	N.A.	0
CLPP	-0,603345748	-0,448386414	0	N.A.	0
CIAPIN1	-0,603418598	-0,299697918	0	N.A.	0
GINS2	-0,603443045	-0,420337819	0	N.A.	0
MLEC	-0,603926679	-0,125982536	0	N.A.	0
MIS18A	-0,604242702	-0,409401412	0	N.A.	0
NAA10	-0,604651797	-0,361636778	0	N.A.	0
CYCS	-0,604688465	-0,513721261	0	N.A.	0
NABP2	-0,604917475	-0,48826828	0	N.A.	0
PSMA5	-0,605119504	-0,299420231	0	N.A.	0
HPRT1	-0,605153076	-0,578645698	0	N.A.	0
COA4	-0,605349739	-0,209107845	0	N.A.	0
TRIP13	-0,605392752	-0,442983423	0	N.A.	0
ATPAF1	-0,606010013	-0,337684722	0	N.A.	0
ATP5O	-0,60653683	-0,35725773	0	N.A.	0
PSMB3	-0,607138251	-0,485546432	0	N.A.	0
CYB5A	-0,60725058	-0,427527983	0	N.A.	0
PDHB	-0,607571492	-0,32293686	0	N.A.	0
PAK1IP1	-0,60774656	-0,360639831	0	N.A.	0
UBE2T	-0,607890494	-0,405466733	0	N.A.	0
LYRM4	-0,60868857	-0,483241528	0	N.A.	0
PTDSS1	-0,608693048	-0,477489115	0	N.A.	0
DNAJC8	-0,608999955	-0,524179758	0	N.A.	0
CDKN3	-0,610186344	-0,440976136	0	N.A.	0
ATPIF1	-0,610225873	-0,437846472	0	N.A.	0
EIF5B	-0,610341798	-0,018365285	0	N.A.	0
ATG3	-0,611111758	-0,392023124	0	N.A.	0
SDHD	-0,611263298	-0,544403492	0	N.A.	0
TRIAP1	-0,611458804	-0,487593249	0	N.A.	0
WDR61	-0,612209299	-0,35975196	0	N.A.	0

REPIN1	-0,613263817	-0,17759325	0	N.A.	0
GMPS	-0,613401726	-0,313596209	0	N.A.	0
ECHS1	-0,613584398	-0,300386074	0	N.A.	0
SLC25A39	-0,613811325	-0,433705651	0	N.A.	0
MAD2L1	-0,614092088	-0,466486826	0	N.A.	0
PRPF40A	-0,614358923	-0,123050021	0	N.A.	0
SMARCB1	-0,614455614	-0,407442147	0	N.A.	0
SHFM1	-0,615320663	-0,498676846	0	N.A.	0
NOP16	-0,615414491	-0,278060073	0	N.A.	0
SAE1	-0,615604947	-0,511903176	0	N.A.	0
HDDC2	-0,615791602	-0,457091509	0	N.A.	0
HSP90AA1	-0,615830604	-0,088997784	0	N.A.	0
PSMD14	-0,615905788	-0,260335072	0	N.A.	0
ACN9	-0,616245375	-0,359235433	0	N.A.	0
APITD1	-0,616454275	-0,420366807	0	N.A.	0
AIMP2	-0,616587507	-0,335766912	0	N.A.	0
CECR5	-0,616613399	-0,2984741	0	N.A.	0
ARL2	-0,616669679	-0,387836491	0	N.A.	0
PDCD6	-0,616746175	-0,329202196	0	N.A.	0
METTL5	-0,617420486	-0,203508318	0	N.A.	0
COPS5	-0,617894496	-0,1348393	0	N.A.	0
AK2	-0,618000933	-0,411805642	0	N.A.	0
ATP5J	-0,618076799	-0,439213674	0	N.A.	0
GADD45GIP1	-0,618185994	-0,371152564	0	N.A.	0
PRPF31	-0,618602732	-0,362601622	0	N.A.	0
BIRC5	-0,618886916	-0,495814881	0	N.A.	0
FIBP	-0,618952783	-0,331892861	0	N.A.	0
DDX39A	-0,619009212	-0,377339302	0	N.A.	0
C11orf73	-0,619029874	-0,324461969	0	N.A.	0
MEST	-0,619118647	-0,313921638	0	N.A.	0
SRSF9	-0,619283321	-0,146193095	0	N.A.	0
GNL3	-0,619449225	-0,304184045	0	N.A.	0
PDSS1	-0,619924214	-0,368079243	0	N.A.	0

SRSF10	-0,620137609	-0,008823748	0	N.A.	0
NAA20	-0,62016852	-0,34840941	0	N.A.	0
TMPO	-0,620521711	-0,32623003	0	N.A.	0
NUBP2	-0,620601326	-0,378655259	0	N.A.	0
PPID	-0,620624968	-0,30901899	0	N.A.	0
ADI1	-0,620647973	-0,322773518	0	N.A.	0
ALDH9A1	-0,621461388	-0,392444978	0	N.A.	0
EIF4A1	-0,622787346	-0,450745054	0	N.A.	0
EXOSC8	-0,62284358	-0,472672006	0	N.A.	0
PSMA4	-0,622854646	-0,530035252	0	N.A.	0
POLE3	-0,622940609	-0,485842019	0	N.A.	0
CENPN	-0,623231839	-0,521818115	0	N.A.	0
C14orf166	-0,623273089	-0,581386497	0	N.A.	0
GSTO1	-0,623734759	-0,506326732	0	N.A.	0
U2SURP	-0,623847113	0,101926659	0	N.A.	0
NDUFS7	-0,623971598	-0,579728686	0	N.A.	0
CNBP	-0,624230983	-0,291173436	0	N.A.	0
LYAR	-0,624532461	-0,444646882	0	N.A.	0
COX14	-0,624840522	-0,391370779	0	N.A.	0
ATIC	-0,625720911	-0,396112774	0	N.A.	0
SFXN4	-0,626117146	-0,286973392	0	N.A.	0
TMEM14B	-0,626382242	-0,41714112	0	N.A.	0
KARS	-0,626522751	-0,29029759	0	N.A.	0
HINT1	-0,626717883	-0,489620527	0	N.A.	0
DNMT1	-0,627282906	-0,269792505	0	N.A.	0
FH	-0,62738921	-0,546856943	0	N.A.	0
C19orf48	-0,62743633	-0,505988206	0	N.A.	0
DTYMK	-0,627483601	-0,408510958	0	N.A.	0
PPP1CC	-0,627499813	-0,419053232	0	N.A.	0
AIFM1	-0,628275967	-0,483096492	0	N.A.	0
NDUFA4	-0,628455204	-0,506979857	0	N.A.	0
UNG	-0,628516366	-0,367041598	0	N.A.	0
PAFAH1B3	-0,628759775	-0,275993254	0	N.A.	0

NIT2	-0,628909957	-0,350212327	0	N.A.	0
SLC25A5	-0,628962111	-0,472663295	0	N.A.	0
MRTO4	-0,630083357	-0,504904137	0	N.A.	0
RNASEH2A	-0,630434382	-0,39493378	0	N.A.	0
PPA2	-0,630502037	-0,329561826	0	N.A.	0
STRAP	-0,630792739	-0,51133529	0	N.A.	0
ZNF511	-0,630869022	-0,296815606	0	N.A.	0
NDUFB2	-0,630985201	-0,502565335	0	N.A.	0
PSMB1	-0,631191237	-0,398134929	0	N.A.	0
THYN1	-0,631353302	-0,385826854	0	N.A.	0
MAGOHB	-0,631499215	-0,481746131	0	N.A.	0
GTF3A	-0,631508015	-0,502654565	0	N.A.	0
DDX1	-0,631647462	-0,246651224	0	N.A.	0
ADK	-0,632145434	-0,544460501	0	N.A.	0
CASP6	-0,63303567	-0,335173281	0	N.A.	0
NELFCD	-0,633252067	-0,212781171	0	N.A.	0
APRT	-0,633338787	-0,500104015	0	N.A.	0
MCM3	-0,633782782	-0,33781701	0	N.A.	0
PSMA3	-0,633793864	-0,478767616	0	N.A.	0
BUB3	-0,634126307	-0,510046648	0	N.A.	0
GAR1	-0,634824179	-0,319418072	0	N.A.	0
PTTG1	-0,636242242	-0,458141076	0	N.A.	0
KIF9	-0,63657288	-0,360804111	0	N.A.	0
TALDO1	-0,636794125	-0,491639619	0	N.A.	0
EWSR1	-0,636876501	-0,138469696	0	N.A.	0
XRN2	-0,637029377	-0,453145832	0	N.A.	0
PRPF19	-0,637077944	-0,398202428	0	N.A.	0
PCNA	-0,637117017	-0,383244955	0	N.A.	0
HMGB2	-0,637460893	-0,483893487	0	N.A.	0
TOMM40	-0,637485783	-0,279783	0	N.A.	0
SAAL1	-0,637532201	-0,459442982	0	N.A.	0
TPRKB	-0,637565361	-0,462442845	0	N.A.	0
REXO2	-0,637621512	-0,102109372	0	N.A.	0

PSMC5	-0,637983272	-0,415277317	0	N.A.	0
DCAF13	-0,6383553	-0,318690924	0	N.A.	0
CSE1L	-0,638550228	-0,391228916	0	N.A.	0
ANP32A	-0,638940273	-0,432291716	0	N.A.	0
TFAM	-0,639670535	-0,35416402	0	N.A.	0
RBM17	-0,639837136	-0,331531928	0	N.A.	0
NDUFA9	-0,639901522	-0,124572723	0	N.A.	0
TFDP1	-0,640045995	-0,342792334	0	N.A.	0
VDAC3	-0,640336252	-0,321622913	0	N.A.	0
EXOSC5	-0,641043026	-0,377335314	0	N.A.	0
NDUFC2	-0,641738799	-0,177775656	0	N.A.	0
FKBP3	-0,642227687	-0,345968351	0	N.A.	0
SPCS1	-0,642248004	-0,351781642	0	N.A.	0
POLR2H	-0,642331725	-0,36976403	0	N.A.	0
SNRNP40	-0,642772579	-0,505803058	0	N.A.	0
WDR77	-0,644105523	-0,512089473	0	N.A.	0
TAF15	-0,64421135	-0,257833027	0	N.A.	0
TRAP1	-0,644578866	-0,350054922	0	N.A.	0
HADH	-0,644933434	-0,395344763	0	N.A.	0
SET	-0,644946507	-0,266015512	0	N.A.	0
RUVBL2	-0,645712762	-0,318325824	0	N.A.	0
MCM4	-0,645915976	-0,429444592	0	N.A.	0
STRA13	-0,646277442	-0,419206149	0	N.A.	0
XRCC5	-0,646288535	-0,314939614	0	N.A.	0
PSMD3	-0,646407113	-0,309241526	0	N.A.	0
SRSF2	-0,646687796	-0,425808795	0	N.A.	0
GMNN	-0,64722473	-0,341056071	0	N.A.	0
APEX1	-0,647326555	-0,449293391	0	N.A.	0
COA1	-0,647737812	-0,43362902	0	N.A.	0
RPIA	-0,647814729	-0,465254854	0	N.A.	0
SNRPG	-0,647925177	-0,422372882	0	N.A.	0
HNRNPC	-0,648478881	-0,433866949	0	N.A.	0
PLEKHJ1	-0,648654204	-0,066586011	0	N.A.	0

SRM	-0,648714077	-0,425513683	0	N.A.	0
PDCD5	-0,648976171	-0,383112678	0	N.A.	0
CUTA	-0,649051499	-0,620841088	0	N.A.	0
PFDN6	-0,649531729	-0,407953228	0	N.A.	0
POLR3K	-0,64982835	-0,401985974	0	N.A.	0
C9orf142	-0,649909511	-0,219196542	0	N.A.	0
LSM6	-0,650104166	-0,532796398	0	N.A.	0
TUBA1B	-0,650178047	-0,488461564	0	N.A.	0
PDHA1	-0,650423091	-0,428165882	0	N.A.	0
CHCHD3	-0,650590764	-0,404790127	0	N.A.	0
SNRNP70	-0,65130684	-0,361153338	0	N.A.	0
TCP1	-0,651349089	-0,459461695	0	N.A.	0
MPC2	-0,651473498	-0,486844095	0	N.A.	0
EBNA1BP2	-0,651652407	-0,371949723	0	N.A.	0
BOLA3	-0,652081138	-0,386437229	0	N.A.	0
ASCL2	-0,652281383	-0,466991125	0	N.A.	0
BRIX1	-0,652986383	-0,397738544	0	N.A.	0
COA6	-0,653951072	-0,399376932	0	N.A.	0
IPO5	-0,654531384	-0,303532151	0	N.A.	0
ACTL6A	-0,654633054	-0,283551543	0	N.A.	0
NDUFA8	-0,654825406	-0,338696034	0	N.A.	0
IMPDH2	-0,655036031	-0,532065601	0	N.A.	0
AGMAT	-0,655724173	-0,399302229	0	N.A.	0
ATP5C1	-0,65589197	-0,422948903	0	N.A.	0
HNRNPU	-0,656076517	-0,239011205	0	N.A.	0
NPM1	-0,656080288	-0,677038746	0	N.A.	0
EIF3I	-0,657229122	-0,482444919	0	N.A.	0
NASP	-0,657413214	-0,455329561	0	N.A.	0
IDH2	-0,658178119	-0,366157632	0	N.A.	0
DUT	-0,658824029	-0,533299663	0	N.A.	0
IMP4	-0,659104363	-0,470558968	0	N.A.	0
CDCA7	-0,659266625	-0,326599282	0	N.A.	0
ANAPC5	-0,659408161	-0,136060656	0	N.A.	0

SNRPD3	-0,659455057	-0,549111027	0	N.A.	0
ZC3H15	-0,659521284	-0,164450073	0	N.A.	0
ILF3	-0,659878771	-0,294727578	0	N.A.	0
CBX1	-0,661116676	-0,369742985	0	N.A.	0
LAGE3	-0,661914685	-0,475185599	0	N.A.	0
TMEM97	-0,66220468	-0,239486895	0	N.A.	0
GCSH	-0,662345096	-0,53327441	0	N.A.	0
NAE1	-0,66247114	-0,338033111	0	N.A.	0
POLR2E	-0,662642977	-0,386014668	0	N.A.	0
METAP2	-0,662954666	-0,405590915	0	N.A.	0
EIF3K	-0,663849562	-0,621569455	0	N.A.	0
ERI3	-0,66433271	-0,457250708	0	N.A.	0
NUDT5	-0,66457155	-0,402253979	0	N.A.	0
AHSA1	-0,664799055	-0,527446408	0	N.A.	0
NUDT1	-0,664816032	-0,485425865	0	N.A.	0
RPA3	-0,665588327	-0,533388631	0	N.A.	0
FABP5	-0,665659848	-0,646019737	0	N.A.	0
CLNS1A	-0,666586039	-0,511157942	0	N.A.	0
NDUFAF4	-0,666669329	-0,340667229	0	N.A.	0
SYNCRIP	-0,667812511	-0,213362002	0	N.A.	0
YBX1	-0,667865612	-0,621774441	0	N.A.	0
COX5A	-0,667884606	-0,489945104	0	N.A.	0
C11orf31	-0,668459981	-0,614994097	0	N.A.	0
RUVBL1	-0,668539758	-0,478378865	0	N.A.	0
FAM96A	-0,669551738	-0,491818192	0	N.A.	0
PPM1G	-0,669922263	-0,421631643	0	N.A.	0
PPIA	-0,669969946	-0,566409175	0	N.A.	0
FBL	-0,670171548	-0,546297738	0	N.A.	0
DNPH1	-0,670277347	-0,530071358	0	N.A.	0
XRCC6	-0,670713963	-0,483696913	0	N.A.	0
HAUS1	-0,67078066	-0,334331579	0	N.A.	0
COPS6	-0,671193317	-0,210716032	0	N.A.	0
CDC123	-0,671396528	-0,501029859	0	N.A.	0

HNRNPDL	-0,671619441	-0,34753731	0	N.A.	0
ATP5J2	-0,671911118	-0,521405964	0	N.A.	0
NDUFA2	-0,673168534	-0,680913661	0	N.A.	0
NDUFA10	-0,673327892	-0,542198881	0	N.A.	0
LSM3	-0,673593497	-0,517180688	0	N.A.	0
SIVA1	-0,673639546	-0,355374479	0	N.A.	0
TMA7	-0,673757463	-0,56800456	0	N.A.	0
MCM7	-0,675058215	-0,379486691	0	N.A.	0
MINOS1	-0,67525984	-0,431532667	0	N.A.	0
C1QBP	-0,675335758	-0,587997036	0	N.A.	0
HNRNPK	-0,675920496	-0,447323835	0	N.A.	0
HMGN1	-0,676205093	-0,547158807	0	N.A.	0
CKS2	-0,676717282	-0,495257721	0	N.A.	0
ATP5B	-0,677261872	-0,411453993	0	N.A.	0
NDUFB8	-0,677274015	-0,313383143	0	N.A.	0
SNRPA	-0,677560837	-0,354711992	0	N.A.	0
EI24	-0,677659043	-0,295113569	0	N.A.	0
NOP58	-0,677750661	-0,245747506	0	N.A.	0
HNRNPF	-0,678836678	-0,355555329	0	N.A.	0
VBP1	-0,678863969	-0,457495148	0	N.A.	0
ATP5G3	-0,679409361	-0,502543008	0	N.A.	0
CACYBP	-0,679521317	-0,42832188	0	N.A.	0
CMSS1	-0,679558927	-0,435713654	0	N.A.	0
ATP5F1	-0,680025925	-0,478913289	0	N.A.	0
ACP1	-0,680394805	-0,360938369	0	N.A.	0
EBPL	-0,680985444	-0,361664097	0	N.A.	0
SUCLG1	-0,681360557	-0,503844247	0	N.A.	0
MDH2	-0,682251138	-0,526607062	0	N.A.	0
NDUFB9	-0,682318654	-0,245032618	0	N.A.	0
NDUFB10	-0,68269175	-0,384037016	0	N.A.	0
NDUFAF2	-0,683262619	-0,596352133	0	N.A.	0
ANP32B	-0,683439988	-0,45081593	0	N.A.	0
HDAC2	-0,683524627	-0,260198113	0	N.A.	0

PRMT1	-0,683852932	-0,405039398	0	N.A.	0
SNRNP25	-0,684277953	-0,451615454	0	N.A.	0
THOC3	-0,684587048	-0,392852407	0	N.A.	0
ESD	-0,684773451	-0,453636613	0	N.A.	0
PTPLAD1	-0,684843813	-0,186087455	0	N.A.	0
WBSCR22	-0,684874503	-0,338926149	0	N.A.	0
NDUFS3	-0,685056624	-0,398363569	0	N.A.	0
ELAVL1	-0,686410782	-0,510209233	0	N.A.	0
C20orf27	-0,686467594	-0,485618151	0	N.A.	0
HNRNPD	-0,686536952	-0,179879731	0	N.A.	0
PTGES3	-0,687505539	-0,555746009	0	N.A.	0
ENSA	-0,688266206	-0,467266032	0	N.A.	0
ANAPC15	-0,688683268	-0,490107426	0	N.A.	0
RNASEH2B	-0,68896221	-0,575017056	0	N.A.	0
H2AFY	-0,689840735	-0,152192502	0	N.A.	0
MAGOH	-0,689936922	-0,554521416	0	N.A.	0
LSM4	-0,690112303	-0,462978092	0	N.A.	0
SNRPB	-0,690463551	-0,4303832	0	N.A.	0
NDUFS8	-0,690719651	-0,555640508	0	N.A.	0
NDUFB11	-0,69097842	-0,550007696	0	N.A.	0
SF3B5	-0,692210525	-0,562111015	0	N.A.	0
HNRNPA1	-0,692238248	-0,6240058	0	N.A.	0
ATP5A1	-0,69245139	-0,575153885	0	N.A.	0
CCT5	-0,693781179	-0,291821417	0	N.A.	0
C17orf89	-0,694014271	-0,561667695	0	N.A.	0
BZW2	-0,69423564	-0,537479587	0	N.A.	0
THOC7	-0,69453769	-0,37650952	0	N.A.	0
CCDC34	-0,69511074	-0,511367224	0	N.A.	0
SNRPD2	-0,695882722	-0,670715135	0	N.A.	0
CCT4	-0,696371783	-0,337749758	0	N.A.	0
TRMT112	-0,696901926	-0,568890193	0	N.A.	0
DDX46	-0,697246686	-0,496881644	0	N.A.	0
MDH1	-0,697335989	-0,461170479	0	N.A.	0

ISOC2	-0,697947417	-0,534785282	0	N.A.	0
SRSF1	-0,698108973	-0,197032304	0	N.A.	0
SSBP1	-0,698274156	-0,572901593	0	N.A.	0
NDUFB6	-0,698823688	-0,420737471	0	N.A.	0
PTMA	-0,700294005	-0,511898009	0	N.A.	0
SOD1	-0,700801408	-0,464789814	0	N.A.	0
PPIH	-0,70128904	-0,588414933	0	N.A.	0
DDX18	-0,701419924	-0,294705374	0	N.A.	0
CCT8	-0,701759478	-0,480122194	0	N.A.	0
LDHB	-0,702396149	-0,6815864	0	N.A.	0
NDUFV1	-0,702473042	-0,502291658	0	N.A.	0
RBMX	-0,704932969	-0,674892923	0	N.A.	0
ATP5L	-0,705372526	-0,52046835	0	N.A.	0
AHCY	-0,70571762	-0,502971861	0	N.A.	0
RCN2	-0,70658727	-0,482858335	0	N.A.	0
CCT7	-0,708423623	-0,336827834	0	N.A.	0
RBBP7	-0,708834991	-0,282784095	0	N.A.	0
TMEM261	-0,70956767	-0,480737589	0	N.A.	0
HMGB3	-0,710508712	-0,442247246	0	N.A.	0
STOML2	-0,710912409	-0,522437581	0	N.A.	0
H2AFZ	-0,711457312	-0,530999243	0	N.A.	0
CISD1	-0,712783468	-0,295279421	0	N.A.	0
POLD2	-0,712985478	-0,524656486	0	N.A.	0
NUDCD2	-0,713130589	-0,511819623	0	N.A.	0
TUFM	-0,713247164	-0,438516443	0	N.A.	0
PAICS	-0,714012401	-0,517631976	0	N.A.	0
PSIP1	-0,714283043	-0,496017667	0	N.A.	0
HMGN3	-0,715404767	-0,613213572	0	N.A.	0
NUCKS1	-0,715423635	-0,398502653	0	N.A.	0
POLR2G	-0,716310572	-0,635633589	0	N.A.	0
SLIRP	-0,716478884	-0,486812725	0	N.A.	0
H2AFV	-0,716950973	-0,480837955	0	N.A.	0
SRPK1	-0,717008058	-0,376271868	0	N.A.	0

PHB2	-0,719112057	-0,5261422	0	N.A.	0
CYC1	-0,71940016	-0,343475399	0	N.A.	0
SERBP1	-0,719445194	-0,436230606	0	N.A.	0
MTCH2	-0,721467818	-0,32397239	0	N.A.	0
SSRP1	-0,724587591	-0,475084634	0	N.A.	0
PARP1	-0,725636857	-0,489602254	0	N.A.	0
HNRNPR	-0,726185925	-0,359638808	0	N.A.	0
PCBD1	-0,726620512	-0,354690647	0	N.A.	0
STMN1	-0,72686478	-0,538304684	0	N.A.	0
NUDC	-0,727268589	-0,480843497	0	N.A.	0
CCT6A	-0,727542843	-0,269900584	0	N.A.	0
DNAJC19	-0,727904454	-0,415475888	0	N.A.	0
HNRNPM	-0,728251999	-0,454080733	0	N.A.	0
RAN	-0,72856265	-0,464172519	0	N.A.	0
ACAT1	-0,731056131	-0,581421214	0	N.A.	0
LSM5	-0,731410284	-0,522978933	0	N.A.	0
ILF2	-0,732429701	-0,473157886	0	N.A.	0
CDK4	-0,733404973	-0,440097002	0	N.A.	0
NDUFS5	-0,73362223	-0,570470259	0	N.A.	0
PA2G4	-0,733903117	-0,528423289	0	N.A.	0
CBX3	-0,734817191	-0,48935375	0	N.A.	0
SRSF7	-0,734872213	-0,458426197	0	N.A.	0
PRDX2	-0,736972108	-0,594971393	0	N.A.	0
NDUFAB1	-0,737282519	-0,482269214	0	N.A.	0
NME1	-0,737305142	-0,536112394	0	N.A.	0
NPM3	-0,73807517	-0,552054545	0	N.A.	0
RANBP1	-0,739583333	-0,564822966	0	N.A.	0
PARK7	-0,740271766	-0,571958046	0	N.A.	0
SNRPC	-0,741143947	-0,641059499	0	N.A.	0
SNRPD1	-0,741273328	-0,592349311	0	N.A.	0
PDAP1	-0,741715154	-0,545779847	0	N.A.	0
SSB	-0,742206445	-0,469790438	0	N.A.	0
PRDX3	-0,743405349	-0,613746647	0	N.A.	0

ATP5G1	-0,745580714	-0,635405251	0	N.A.	0
PHB	-0,745808122	-0,532654323	0	N.A.	0
CCT3	-0,745871543	-0,473839278	0	N.A.	0
HMGN2	-0,747239046	-0,624312408	0	N.A.	0
HSPA8	-0,747369292	-0,485164269	0	N.A.	0
SRP9	-0,749590407	-0,598496473	0	N.A.	0
ERH	-0,74971316	-0,493705847	0	N.A.	0
DKC1	-0,750377121	-0,381445786	0	N.A.	0
CCT2	-0,75067246	-0,373368388	0	N.A.	0
PSMG1	-0,751312062	-0,474856575	0	N.A.	0
HSP90AB1	-0,75157825	-0,501965769	0	N.A.	0
HSPD1	-0,754075008	-0,579130767	0	N.A.	0
HNRNPA2B1	-0,755410177	-0,48512672	0	N.A.	0
HSD17B10	-0,756854631	-0,44479559	0	N.A.	0
NDUFS6	-0,757718302	-0,415012291	0	N.A.	0
EEF1E1	-0,760389823	-0,487870701	0	N.A.	0
DCTPP1	-0,761896286	-0,36212896	0	N.A.	0
BANF1	-0,762570869	-0,629397181	0	N.A.	0
NHP2	-0,76739723	-0,618069919	0	N.A.	0
HNRNPA3	-0,768296729	-0,559737389	0	N.A.	0
LSM2	-0,771096667	-0,638277336	0	N.A.	0
NCL	-0,771770742	-0,529410481	0	N.A.	0
SRSF3	-0,772824956	-0,538410593	0	N.A.	0
HMGB1	-0,780648561	-0,502585688	0	N.A.	0
GGCT	-0,7817603	-0,506587063	0	N.A.	0
PEBP1	-0,784250936	-0,69111521	0	N.A.	0
SNRPE	-0,789133493	-0,625695256	0	N.A.	0
SNRPF	-0,794191165	-0,597826167	0	N.A.	0
LSM7	-0,797421998	-0,567592616	0	N.A.	0
HSPE1	-0,801245001	-0,624661373	0	N.A.	0
TIMM13	-0,80305769	-0,567037898	0	N.A.	0
A2M-AS1		0,158513394	0	N.A.	0
A4GNT			0	N.A.	0

AADACL2			0	N.A.	0
AADACL2-AS1			0	N.A.	0
AANAT			0	N.A.	0
AARD			0	N.A.	0
ABC12-49244600F4.4			0	N.A.	0
ABCA4			0	N.A.	0
ABCA6		0,122251418	0	N.A.	0
ABCA9		0,159834853	0	N.A.	0
ABCB5			0	N.A.	0
ABCC11		0,248761503	0	N.A.	0
ABCC12			0	N.A.	0
ABCC9			0	N.A.	0
ABCD2		-0,088758096	0	N.A.	0
ABCG4			0	N.A.	0
ABHD14A-ACY1		-0,333224209	0	N.A.	0
ABHD15-AS1			0	N.A.	0
ABHD16B			0	N.A.	0
ABRA			0	N.A.	0
AC000032.2			0	N.A.	0
AC000067.1			0	N.A.	0
AC000068.10		0,067349196	0	N.A.	0
AC000068.9		0,162763704	0	N.A.	0
AC002064.5			0	N.A.	0
AC002066.1			0	N.A.	0
AC002365.5			0	N.A.	0
AC002398.12		0,008985307	0	N.A.	0
AC002398.13			0	N.A.	0
AC002456.2			0	N.A.	0
AC002463.3			0	N.A.	0
AC002480.2			0	N.A.	0
AC002480.3		0,044112527	0	N.A.	0
AC002480.4			0	N.A.	0
AC002511.2			0	N.A.	0

AC002511.3			0	N.A.	0
AC002550.5		-0,115437591	0	N.A.	0
AC003005.2			0	N.A.	0
AC003006.7			0	N.A.	0
AC003088.1			0	N.A.	0
AC003090.1			0	N.A.	0
AC003101.1			0	N.A.	0
AC003986.6			0	N.A.	0
AC003986.7			0	N.A.	0
AC004066.3			0	N.A.	0
AC004067.5			0	N.A.	0
AC004158.1			0	N.A.	0
AC004160.4			0	N.A.	0
AC004221.2			0	N.A.	0
AC004237.1			0	N.A.	0
AC004381.7		-0,112500357	0	N.A.	0
AC004448.5			0	N.A.	0
AC004471.10			0	N.A.	0
AC004490.1		0,220585316	0	N.A.	0
AC004593.3		0,06761821	0	N.A.	0
AC004691.5			0	N.A.	0
AC004775.5			0	N.A.	0
AC004824.2			0	N.A.	0
AC004837.5		0,207835211	0	N.A.	0
AC004878.8			0	N.A.	0
AC004906.3			0	N.A.	0
AC004941.3			0	N.A.	0
AC004947.2			0	N.A.	0
AC004988.1			0	N.A.	0
AC005009.1			0	N.A.	0
AC005009.2		0,115490784	0	N.A.	0
AC005013.5			0	N.A.	0
AC005019.3			0	N.A.	0

AC005042.4			0	N.A.	0
AC005082.12			0	N.A.	0
AC005197.2			0	N.A.	0
AC005220.3			0	N.A.	0
AC005237.4		-0,037118778	0	N.A.	0
AC005253.4			0	N.A.	0
AC005276.1		-0,012598741	0	N.A.	0
AC005281.2		0,061598112	0	N.A.	0
AC005304.1			0	N.A.	0
AC005307.3		-0,247619467	0	N.A.	0
AC005324.6		0,298278559	0	N.A.	0
AC005355.1			0	N.A.	0
AC005477.1		0,142827328	0	N.A.	0
AC005498.3			0	N.A.	0
AC005498.4			0	N.A.	0
AC005522.7		0,162512936	0	N.A.	0
AC005540.3		0,385453666	0	N.A.	0
AC005546.2			0	N.A.	0
AC005592.1			0	N.A.	0
AC005592.3			0	N.A.	0
AC005606.14			0	N.A.	0
AC005606.15			0	N.A.	0
AC005609.16			0	N.A.	0
AC005616.2			0	N.A.	0
AC005624.2		-0,091396266	0	N.A.	0
AC005625.1			0	N.A.	0
AC005703.3			0	N.A.	0
AC005754.8			0	N.A.	0
AC005932.1			0	N.A.	0
AC006007.1			0	N.A.	0
AC006042.6			0	N.A.	0
AC006126.3		-0,002001467	0	N.A.	0
AC006126.4			0	N.A.	0

AC006129.1			0	N.A.	0
AC006159.4		0,387970496	0	N.A.	0
AC006160.5		-0,027206994	0	N.A.	0
AC006369.2		0,133790052	0	N.A.	0
AC006372.4			0	N.A.	0
AC006372.5			0	N.A.	0
AC006449.2			0	N.A.	0
AC006460.2		0,312475542	0	N.A.	0
AC006482.1			0	N.A.	0
AC006547.8		-0,03847024	0	N.A.	0
AC006946.15			0	N.A.	0
AC006946.16			0	N.A.	0
AC007040.6			0	N.A.	0
AC007040.8			0	N.A.	0
AC007050.17			0	N.A.	0
AC007064.24		-0,097864271	0	N.A.	0
AC007126.1		0,04418319	0	N.A.	0
AC007161.5			0	N.A.	0
AC007163.6			0	N.A.	0
AC007192.6			0	N.A.	0
AC007193.8			0	N.A.	0
AC007193.9			0	N.A.	0
AC007285.6		-0,146684907	0	N.A.	0
AC007292.4			0	N.A.	0
AC007319.1		-0,13826328	0	N.A.	0
AC007325.1			0	N.A.	0
AC007349.5			0	N.A.	0
AC007349.7			0	N.A.	0
AC007362.3			0	N.A.	0
AC007364.1			0	N.A.	0
AC007365.1		-0,072964582	0	N.A.	0
AC007365.3			0	N.A.	0
AC007381.2			0	N.A.	0

AC007381.3			0	N.A.	0
AC007386.2			0	N.A.	0
AC007389.3			0	N.A.	0
AC007391.2			0	N.A.	0
AC007403.2			0	N.A.	0
AC007405.4		-0,007659978	0	N.A.	0
AC007405.8			0	N.A.	0
AC007422.1		0,034357583	0	N.A.	0
AC007556.3		0,306251413	0	N.A.	0
AC007563.5			0	N.A.	0
AC007566.10			0	N.A.	0
AC007743.1			0	N.A.	0
AC007750.5			0	N.A.	0
AC007879.4			0	N.A.	0
AC007879.5			0	N.A.	0
AC007879.7			0	N.A.	0
AC007880.1			0	N.A.	0
AC007906.1			0	N.A.	0
AC007950.1		0,256570298	0	N.A.	0
AC008063.2			0	N.A.	0
AC008063.3		0,263327843	0	N.A.	0
AC008067.2			0	N.A.	0
AC008073.9			0	N.A.	0
AC008074.1		0,124445685	0	N.A.	0
AC008088.4			0	N.A.	0
AC008268.1			0	N.A.	0
AC008269.2			0	N.A.	0
AC008277.1			0	N.A.	0
AC008440.5			0	N.A.	0
AC008592.8		0,266713809	0	N.A.	0
AC008697.1			0	N.A.	0
AC008746.3			0	N.A.	0
AC008937.2			0	N.A.	0

AC008937.3			0	N.A.	0
AC009065.4		0,092205615	0	N.A.	0
AC009120.4			0	N.A.	0
AC009120.5		0,154617352	0	N.A.	0
AC009133.17		0,250858065	0	N.A.	0
AC009227.2			0	N.A.	0
AC009228.1		0,014959771	0	N.A.	0
AC009229.5			0	N.A.	0
AC009262.2			0	N.A.	0
AC009276.4			0	N.A.	0
AC009299.2		0,074697101	0	N.A.	0
AC009303.1			0	N.A.	0
AC009336.19			0	N.A.	0
AC009495.4		-0,225163244	0	N.A.	0
AC009502.4		0,081790301	0	N.A.	0
AC009505.2			0	N.A.	0
AC009518.4			0	N.A.	0
AC009542.2			0	N.A.	0
AC009784.3			0	N.A.	0
AC010084.1			0	N.A.	0
AC010136.2			0	N.A.	0
AC010504.2		0,261199982	0	N.A.	0
AC010524.4			0	N.A.	0
AC010547.9			0	N.A.	0
AC010649.1			0	N.A.	0
AC010746.3			0	N.A.	0
AC010761.13			0	N.A.	0
AC010761.6			0	N.A.	0
AC010761.9			0	N.A.	0
AC010884.1			0	N.A.	0
AC010890.1			0	N.A.	0
AC010894.5			0	N.A.	0
AC010976.2			0	N.A.	0

AC010987.6			0	N.A.	0
AC011247.3			0	N.A.	0
AC011290.4			0	N.A.	0
AC011294.3			0	N.A.	0
AC011738.4			0	N.A.	0
AC011747.6			0	N.A.	0
AC011893.3			0	N.A.	0
AC011899.9			0	N.A.	0
AC012123.1			0	N.A.	0
AC012314.8		0,099828457	0	N.A.	0
AC012358.7			0	N.A.	0
AC012442.6			0	N.A.	0
AC012462.2			0	N.A.	0
AC012494.1			0	N.A.	0
AC012531.25			0	N.A.	0
AC012593.1			0	N.A.	0
AC012613.2		0,228744416	0	N.A.	0
AC012668.2			0	N.A.	0
AC013264.2			0	N.A.	0
AC013275.2		-0,261803509	0	N.A.	0
AC015688.3			0	N.A.	0
AC015691.13		0,250250155	0	N.A.	0
AC015849.12			0	N.A.	0
AC015849.19			0	N.A.	0
AC015849.2		0,053083561	0	N.A.	0
AC015922.6			0	N.A.	0
AC015933.2			0	N.A.	0
AC015936.3			0	N.A.	0
AC015969.3		0,18184655	0	N.A.	0
AC016629.3			0	N.A.	0
AC016683.5			0	N.A.	0
AC016700.2			0	N.A.	0
AC016722.3		0,193210016	0	N.A.	0

AC016722.4			0	N.A.	0
AC016723.4			0	N.A.	0
AC016735.1		-0,065957726	0	N.A.	0
AC016738.3			0	N.A.	0
AC016745.3		-0,146865157	0	N.A.	0
AC016768.1			0	N.A.	0
AC016907.3			0	N.A.	0
AC016910.1			0	N.A.	0
AC016912.3			0	N.A.	0
AC016995.3			0	N.A.	0
AC016999.2		0,213993898	0	N.A.	0
AC017028.10			0	N.A.	0
AC017060.1		0,373902596	0	N.A.	0
AC017101.10			0	N.A.	0
AC017104.2			0	N.A.	0
AC018462.2		0,361909903	0	N.A.	0
AC018464.3			0	N.A.	0
AC018647.3			0	N.A.	0
AC018766.4			0	N.A.	0
AC018766.6			0	N.A.	0
AC018878.3			0	N.A.	0
AC019100.3		0,317484497	0	N.A.	0
AC019172.2			0	N.A.	0
AC019206.1			0	N.A.	0
AC020594.5		0,075163742	0	N.A.	0
AC020743.2			0	N.A.	0
AC020956.3			0	N.A.	0
AC021188.4			0	N.A.	0
AC022154.7		-0,146865157	0	N.A.	0
AC022431.2			0	N.A.	0
AC022816.2			0	N.A.	0
AC023115.2			0	N.A.	0
AC023347.1		0,035187887	0	N.A.	0

AC024084.1			0	N.A.	0
AC024704.2			0	N.A.	0
AC026150.8			0	N.A.	0
AC026167.1			0	N.A.	0
AC026904.1			0	N.A.	0
AC027119.1			0	N.A.	0
AC027601.1		-0,030411141	0	N.A.	0
AC034187.2			0	N.A.	0
AC055764.1			0	N.A.	0
AC061961.2		0,174463626	0	N.A.	0
AC062028.1		0,183547058	0	N.A.	0
AC064834.2			0	N.A.	0
AC064834.3			0	N.A.	0
AC067945.2			0	N.A.	0
AC067959.1		0,185622821	0	N.A.	0
AC068196.1			0	N.A.	0
AC068491.2			0	N.A.	0
AC068492.1		0,343588128	0	N.A.	0
AC068533.7			0	N.A.	0
AC068580.6			0	N.A.	0
AC068580.7		0,268151694	0	N.A.	0
AC068831.15			0	N.A.	0
AC068831.3		-0,099218049	0	N.A.	0
AC068831.6			0	N.A.	0
AC069368.3			0	N.A.	0
AC073072.5			0	N.A.	0
AC073107.1		-0,07971405	0	N.A.	0
AC073115.6			0	N.A.	0
AC073115.7			0	N.A.	0
AC073130.3			0	N.A.	0
AC073133.1		-0,055489061	0	N.A.	0
AC073133.2		-0,078135637	0	N.A.	0
AC073254.1		0,203166009	0	N.A.	0

AC073316.1			0	N.A.	0
AC073316.2			0	N.A.	0
AC073333.8			0	N.A.	0
AC073342.12			0	N.A.	0
AC073343.13			0	N.A.	0
AC073641.2			0	N.A.	0
AC073657.1		-0,060508802	0	N.A.	0
AC073834.3		-0,057629436	0	N.A.	0
AC073842.19		0,099674316	0	N.A.	0
AC073934.6		0,17457869	0	N.A.	0
AC073957.15			0	N.A.	0
AC074363.1		0,267566087	0	N.A.	0
AC074366.3			0	N.A.	0
AC074391.1		0,178139772	0	N.A.	0
AC078941.1			0	N.A.	0
AC079135.1			0	N.A.	0
AC079145.4			0	N.A.	0
AC079354.3			0	N.A.	0
AC079610.2			0	N.A.	0
AC079630.4			0	N.A.	0
AC079742.4			0	N.A.	0
AC079767.4			0	N.A.	0
AC079776.3			0	N.A.	0
AC079988.3			0	N.A.	0
AC083949.1		-0,246901544	0	N.A.	0
AC084082.3			0	N.A.	0
AC087430.1			0	N.A.	0
AC087491.2		0,096295576	0	N.A.	0
AC087499.10			0	N.A.	0
AC090154.1		0,051086021	0	N.A.	0
AC090505.4			0	N.A.	0
AC090617.1			0	N.A.	0
AC090627.1		0,027024748	0	N.A.	0

AC090952.5			0	N.A.	0
AC091132.1			0	N.A.	0
AC091133.1			0	N.A.	0
AC091153.4			0	N.A.	0
AC091199.1			0	N.A.	0
AC091493.2			0	N.A.	0
AC091633.3			0	N.A.	0
AC091814.3			0	N.A.	0
AC091878.1			0	N.A.	0
AC092159.2		-0,073585913	0	N.A.	0
AC092162.1			0	N.A.	0
AC092198.1			0	N.A.	0
AC092535.3			0	N.A.	0
AC092580.3			0	N.A.	0
AC092614.2		0,047567521	0	N.A.	0
AC092620.3		0,05933785	0	N.A.	0
AC092652.1			0	N.A.	0
AC092667.2			0	N.A.	0
AC092675.3			0	N.A.	0
AC092839.3			0	N.A.	0
AC092839.4			0	N.A.	0
AC092881.1		0,078294522	0	N.A.	0
AC093110.3		-0,264570892	0	N.A.	0
AC093390.1			0	N.A.	0
AC093627.7			0	N.A.	0
AC093690.1			0	N.A.	0
AC093702.1			0	N.A.	0
AC093802.1		0,011169857	0	N.A.	0
AC093850.2			0	N.A.	0
AC096558.1			0	N.A.	0
AC096559.1		0,10156583	0	N.A.	0
AC096579.13		0,291319028	0	N.A.	0
AC096649.2			0	N.A.	0

AC097381.1			0	N.A.	0
AC097467.2			0	N.A.	0
AC097495.2			0	N.A.	0
AC097713.4			0	N.A.	0
AC098617.1			0	N.A.	0
AC098820.2			0	N.A.	0
AC098820.4		0,210001999	0	N.A.	0
AC098823.3			0	N.A.	0
AC098828.2		-0,1084766	0	N.A.	0
AC098973.2		-0,110654708	0	N.A.	0
AC099342.1		0,38822783	0	N.A.	0
AC099344.3		-0,024594426	0	N.A.	0
AC100830.5		0,078294522	0	N.A.	0
AC102953.4			0	N.A.	0
AC103563.8			0	N.A.	0
AC104024.1			0	N.A.	0
AC104024.2			0	N.A.	0
AC104076.3			0	N.A.	0
AC104088.1		0,110177933	0	N.A.	0
AC104134.2		0,015859071	0	N.A.	0
AC104135.2			0	N.A.	0
AC104653.1		0,0768443	0	N.A.	0
AC104655.3		-0,148988748	0	N.A.	0
AC104699.1			0	N.A.	0
AC104809.3			0	N.A.	0
AC105339.1		0,010413208	0	N.A.	0
AC105402.4			0	N.A.	0
AC105760.3		-0,031131404	0	N.A.	0
AC106053.1			0	N.A.	0
AC106786.1			0	N.A.	0
AC106869.2		-0,06119256	0	N.A.	0
AC106873.4		-0,087091051	0	N.A.	0
AC107218.3			0	N.A.	0

AC108004.2		-0,025234084	0	N.A.	0
AC108868.5			0	N.A.	0
AC108938.5		0,057929071	0	N.A.	0
AC109333.10			0	N.A.	0
AC109642.1			0	N.A.	0
AC109826.1		0,253520133	0	N.A.	0
AC109829.1			0	N.A.	0
AC110615.1			0	N.A.	0
AC110769.3			0	N.A.	0
AC110781.3		-0,015294075	0	N.A.	0
AC112715.2			0	N.A.	0
AC112721.1			0	N.A.	0
AC112721.2			0	N.A.	0
AC114730.2			0	N.A.	0
AC114730.5			0	N.A.	0
AC114730.7		-0,030121414	0	N.A.	0
AC114730.8			0	N.A.	0
AC116035.1			0	N.A.	0
AC116614.1			0	N.A.	0
AC124944.5			0	N.A.	0
AC128709.4		0,175318394	0	N.A.	0
AC129492.1			0	N.A.	0
AC130469.2		-0,054183333	0	N.A.	0
AC131056.5		0,269706999	0	N.A.	0
AC131263.1		0,257589351	0	N.A.	0
AC133106.2			0	N.A.	0
AC135178.7			0	N.A.	0
AC136352.5		0,182242331	0	N.A.	0
AC137723.5		0,040030622	0	N.A.	0
AC137932.5			0	N.A.	0
AC137932.6			0	N.A.	0
AC138035.2		-0,060819826	0	N.A.	0
AC139099.4			0	N.A.	0

AC139099.5			0	N.A.	0
AC139100.3			0	N.A.	0
AC139887.4		-0,156149809	0	N.A.	0
AC141272.1			0	N.A.	0
AC141928.1		-0,026153694	0	N.A.	0
AC144836.1			0	N.A.	0
AC147651.4		0,065744066	0	N.A.	0
AC147651.5			0	N.A.	0
AC159540.2			0	N.A.	0
AC195454.1			0	N.A.	0
AC233755.1			0	N.A.	0
AC233755.2			0	N.A.	0
AC244230.1		0,228287442	0	N.A.	0
ACADL			0	N.A.	0
ACBD7		-0,140249552	0	N.A.	0
ACER1			0	N.A.	0
ACMSD			0	N.A.	0
ACOT1		0,221215571	0	N.A.	0
ACOT12		-0,093160418	0	N.A.	0
ACSBG1			0	N.A.	0
ACSBG2			0	N.A.	0
ACSM2A			0	N.A.	0
ACSM2B			0	N.A.	0
ACSM5		0,085086587	0	N.A.	0
ACSM6			0	N.A.	0
ACTA2-AS1		-0,012360889	0	N.A.	0
ACTC1		-0,016227897	0	N.A.	0
ACTL6B			0	N.A.	0
ACTL7A		-0,014083432	0	N.A.	0
ACTL7B			0	N.A.	0
ACTN3			0	N.A.	0
AD000091.2			0	N.A.	0
AD000671.6			0	N.A.	0

AD001527.7			0	N.A.	0
ADAM11			0	N.A.	0
ADAM20		0,09629621	0	N.A.	0
ADAM21			0	N.A.	0
ADAM23			0	N.A.	0
ADAM29			0	N.A.	0
ADAM33		-0,280866607	0	N.A.	0
ADAM7			0	N.A.	0
ADAMTS10			0	N.A.	0
ADAMTS16		-0,014054397	0	N.A.	0
ADAMTS18			0	N.A.	0
ADAMTS19		-0,091410105	0	N.A.	0
ADAMTS20			0	N.A.	0
ADAMTS3			0	N.A.	0
ADAMTS5		-0,237817163	0	N.A.	0
ADAMTS9-AS1			0	N.A.	0
ADAMTSL1		-0,171577683	0	N.A.	0
ADAMTSL3		-0,006737522	0	N.A.	0
ADARB2			0	N.A.	0
ADARB2-AS1			0	N.A.	0
ADCYAP1R1			0	N.A.	0
ADD2		0,103478018	0	N.A.	0
ADH1B			0	N.A.	0
ADORA1		0,101505268	0	N.A.	0
ADORA2A			0	N.A.	0
ADRA1A			0	N.A.	0
ADRB3			0	N.A.	0
AF011889.2		0,210032705	0	N.A.	0
AF038458.5			0	N.A.	0
AF064858.11			0	N.A.	0
AF064860.7		0,222726693	0	N.A.	0
AF121898.3			0	N.A.	0
AF124730.4		0,148686451	0	N.A.	0

AF127577.10			0	N.A.	0
AF127936.5			0	N.A.	0
AF131215.6			0	N.A.	0
AF131216.5			0	N.A.	0
AF131216.6		0,143789501	0	N.A.	0
AF131217.1			0	N.A.	0
AF146191.4		-0,052154202	0	N.A.	0
AF178030.2			0	N.A.	0
AF196970.3			0	N.A.	0
AF230666.2			0	N.A.	0
AGRP			0	N.A.	0
AGTR1			0	N.A.	0
AHSP		-0,025234084	0	N.A.	0
AICDA			0	N.A.	0
AIPL1		-0,041843372	0	N.A.	0
AJ003147.9		0,026283336	0	N.A.	0
AJ006998.2		0,010504664	0	N.A.	0
AJ011932.1			0	N.A.	0
AJ239322.1			0	N.A.	0
AJAP1			0	N.A.	0
AK5		-0,072540193	0	N.A.	0
AKR1B15			0	N.A.	0
AKR1D1			0	N.A.	0
AL022344.5		0,035082506	0	N.A.	0
AL022344.7			0	N.A.	0
AL022476.2			0	N.A.	0
AL022578.1			0	N.A.	0
AL035610.2			0	N.A.	0
AL109761.5			0	N.A.	0
AL109763.2			0	N.A.	0
AL109767.1			0	N.A.	0
AL122127.25			0	N.A.	0
AL132709.8			0	N.A.	0

AL157902.3			0	N.A.	0
AL161668.5		-0,036612563	0	N.A.	0
AL365202.1			0	N.A.	0
AL513523.2		-0,262987561	0	N.A.	0
AL592528.1			0	N.A.	0
AL645728.1			0	N.A.	0
AL672294.1			0	N.A.	0
AL928742.12			0	N.A.	0
ALAS2			0	N.A.	0
ALB			0	N.A.	0
ALDH1L1-AS2			0	N.A.	0
ALG13-AS1			0	N.A.	0
ALKBH3-AS1		-0,149338531	0	N.A.	0
ALLC			0	N.A.	0
ALOX12			0	N.A.	0
ALOX15B		-0,271408439	0	N.A.	0
ALS2CR11		0,005755801	0	N.A.	0
ALX3			0	N.A.	0
AMER2			0	N.A.	0
AMHR2			0	N.A.	0
AMIGO3		0,191144241	0	N.A.	0
AMPD1			0	N.A.	0
AMPH			0	N.A.	0
AMZ1		0,010336041	0	N.A.	0
ANGPT1		-0,23751237	0	N.A.	0
ANGPT4			0	N.A.	0
ANGPTL1			0	N.A.	0
ANGPTL7			0	N.A.	0
ANKRD2			0	N.A.	0
ANKRD20A2		0,050211548	0	N.A.	0
ANKRD30A			0	N.A.	0
ANKRD30BL			0	N.A.	0
ANKRD34C			0	N.A.	0

ANKRD45		0,269034692	0	N.A.	0
ANKRD53			0	N.A.	0
ANKRD55			0	N.A.	0
ANKRD61		-0,044682659	0	N.A.	0
ANKRD66			0	N.A.	0
ANKRD7		0,035909195	0	N.A.	0
ANKUB1			0	N.A.	0
ANO1-AS1			0	N.A.	0
ANO1-AS2			0	N.A.	0
ANO2			0	N.A.	0
ANO3		0,28564292	0	N.A.	0
ANO4			0	N.A.	0
ANO5			0	N.A.	0
ANP32D			0	N.A.	0
AP000223.42			0	N.A.	0
AP000233.4			0	N.A.	0
AP000255.6			0	N.A.	0
AP000265.1		-0,100007591	0	N.A.	0
AP000266.7			0	N.A.	0
AP000289.6		-0,061463922	0	N.A.	0
AP000304.12		-0,047864984	0	N.A.	0
AP000318.2		0,435854968	0	N.A.	0
AP000320.6		0,152710136	0	N.A.	0
AP000343.2		0,014722007	0	N.A.	0
AP000345.1			0	N.A.	0
AP000349.2		-0,026702031	0	N.A.	0
AP000350.10		0,042750086	0	N.A.	0
AP000355.2			0	N.A.	0
AP000438.2		0,061989084	0	N.A.	0
AP000439.1			0	N.A.	0
AP000442.4			0	N.A.	0
AP000473.5			0	N.A.	0
AP000473.8			0	N.A.	0

AP000476.1			0	N.A.	0
AP000593.7			0	N.A.	0
AP000640.10		0,287127054	0	N.A.	0
AP000688.29			0	N.A.	0
AP000695.6			0	N.A.	0
AP000696.2		0,23933568	0	N.A.	0
AP000697.6		0,008936393	0	N.A.	0
AP000705.7			0	N.A.	0
AP000721.4			0	N.A.	0
AP000797.4			0	N.A.	0
AP000892.4			0	N.A.	0
AP001046.5		0,143789501	0	N.A.	0
AP001046.6			0	N.A.	0
AP001056.1			0	N.A.	0
AP001057.1			0	N.A.	0
AP001059.5			0	N.A.	0
AP001063.1			0	N.A.	0
AP001257.1		0,195904803	0	N.A.	0
AP001432.14			0	N.A.	0
AP001434.2			0	N.A.	0
AP001468.58			0	N.A.	0
AP001469.7			0	N.A.	0
AP001471.1			0	N.A.	0
AP001476.2			0	N.A.	0
AP001476.3			0	N.A.	0
AP001596.6			0	N.A.	0
AP001604.3		-0,338316193	0	N.A.	0
AP001610.5		-0,046122416	0	N.A.	0
AP001626.1			0	N.A.	0
AP001627.1		-0,10805899	0	N.A.	0
AP002884.2		0,044869346	0	N.A.	0
AP002954.3			0	N.A.	0
AP002954.4			0	N.A.	0

AP003025.2			0	N.A.	0
AP003774.4			0	N.A.	0
AP003774.6			0	N.A.	0
AP004372.1			0	N.A.	0
AP005530.2			0	N.A.	0
AP006621.8		-0,013811343	0	N.A.	0
AP006621.9		0,372198168	0	N.A.	0
APCDD1L		-0,005973519	0	N.A.	0
APELA			0	N.A.	0
APOA1-AS			0	N.A.	0
APOB			0	N.A.	0
APOBEC2		0,062658061	0	N.A.	0
APOBEC3B-AS1		-0,344885361	0	N.A.	0
APOBEC4		-0,058329868	0	N.A.	0
APOC3		0,026283336	0	N.A.	0
APOF			0	N.A.	0
AQP10			0	N.A.	0
AQP2			0	N.A.	0
AQP4-AS1			0	N.A.	0
ARAP1-AS1			0	N.A.	0
ARAP1-AS2			0	N.A.	0
ARHGAP20		-0,080387704	0	N.A.	0
ARHGAP28			0	N.A.	0
ARHGEF15			0	N.A.	0
ARHGEF33			0	N.A.	0
ARL5C			0	N.A.	0
ARMC12		-0,233465888	0	N.A.	0
ARMC3		0,104895788	0	N.A.	0
ARMCX3-AS1			0	N.A.	0
ARMS2			0	N.A.	0
ARPC4-TTLL3		-0,138065558	0	N.A.	0
ARPP21			0	N.A.	0
ARR3			0	N.A.	0

ARRDC5			0	N.A.	0
ART4			0	N.A.	0
ASB10			0	N.A.	0
ASB11		0,239831307	0	N.A.	0
ASB14		0,058001787	0	N.A.	0
ASCL4			0	N.A.	0
ASIC5			0	N.A.	0
ASPA		0,012990927	0	N.A.	0
ASPDH			0	N.A.	0
ASTL			0	N.A.	0
ASTN1			0	N.A.	0
ASXL3			0	N.A.	0
ASZ1			0	N.A.	0
ATP11A-AS1			0	N.A.	0
ATP12A		0,203646366	0	N.A.	0
ATP13A5			0	N.A.	0
ATP1A4			0	N.A.	0
ATP1B3-AS1			0	N.A.	0
ATP1B4			0	N.A.	0
ATP2B2			0	N.A.	0
ATP2B3			0	N.A.	0
ATP4B			0	N.A.	0
ATP5J2-PTCD1			0	N.A.	0
ATP6V0D2			0	N.A.	0
ATP6V1B1-AS1			0	N.A.	0
ATRNL1			0	N.A.	0
AVPR2			0	N.A.	0
AWAT2			0	N.A.	0
B3GAT1			0	N.A.	0
B4GALNT1			0	N.A.	0
BAALC-AS2			0	N.A.	0
BACE2-IT1			0	N.A.	0
BAI3			0	N.A.	0

BANCR			0	N.A.	0
BANF2		-0,111496295	0	N.A.	0
BARX1-AS1			0	N.A.	0
BBOX1			0	N.A.	0
BCHE		0,247187261	0	N.A.	0
BDNF			0	N.A.	0
BEAN1-AS1			0	N.A.	0
BEND2			0	N.A.	0
BEND4		-0,119409565	0	N.A.	0
BEND5		-0,085913398	0	N.A.	0
BEND6			0	N.A.	0
BEX1			0	N.A.	0
BFSP2		-0,130425705	0	N.A.	0
BFSP2-AS1			0	N.A.	0
BHLHA15			0	N.A.	0
BHLHA9		0,086912565	0	N.A.	0
BHLHE22			0	N.A.	0
BHMT		-0,057605904	0	N.A.	0
BHMT2			0	N.A.	0
BIRC6-AS2		0,231852562	0	N.A.	0
BLID			0	N.A.	0
BMP8A			0	N.A.	0
BMPR1B		0,163781425	0	N.A.	0
BMPR1B-AS1			0	N.A.	0
BNC1			0	N.A.	0
bP-2189O9.3			0	N.A.	0
BPIFB1			0	N.A.	0
BPIFB4			0	N.A.	0
BRDT			0	N.A.	0
BSN-AS2			0	N.A.	0
BSND			0	N.A.	0
BTG4			0	N.A.	0
BVES-AS1			0	N.A.	0

BX255923.3			0	N.A.	0
BX322559.3		-0,213918434	0	N.A.	0
C10orf105			0	N.A.	0
C10orf90			0	N.A.	0
C11orf16			0	N.A.	0
C11orf44		-0,018498013	0	N.A.	0
C11orf52			0	N.A.	0
C11orf87			0	N.A.	0
C11orf88			0	N.A.	0
C11orf91		0,486274108	0	N.A.	0
C11orf94		-0,000164059	0	N.A.	0
C11orf97		0,177802537	0	N.A.	0
C12orf40			0	N.A.	0
C12orf54		0,288804646	0	N.A.	0
C12orf71		0,024083864	0	N.A.	0
C12orf74			0	N.A.	0
C12orf77			0	N.A.	0
C12orf79			0	N.A.	0
C12orf80			0	N.A.	0
C14orf178			0	N.A.	0
C14orf39			0	N.A.	0
C15orf38-AP3S2			0	N.A.	0
C15orf53			0	N.A.	0
C15orf54			0	N.A.	0
C15orf59			0	N.A.	0
C15orf59-AS1			0	N.A.	0
C16orf47			0	N.A.	0
C16orf90			0	N.A.	0
C16orf96			0	N.A.	0
C17orf105			0	N.A.	0
C17orf61-PLSCR3		-0,151367356	0	N.A.	0
C17orf64		-0,025234084	0	N.A.	0
C18orf42		0,055796637	0	N.A.	0

C19orf35		0,171832858	0	N.A.	0
C19orf71			0	N.A.	0
C19orf84			0	N.A.	0
C1orf100			0	N.A.	0
C1orf140			0	N.A.	0
C1orf141		0,068107124	0	N.A.	0
C1orf143			0	N.A.	0
C1orf145			0	N.A.	0
C1orf147		0,032898196	0	N.A.	0
C1orf158			0	N.A.	0
C1orf189			0	N.A.	0
C1orf234		0,211474217	0	N.A.	0
C1orf94			0	N.A.	0
C1QL3			0	N.A.	0
C1QTNF3-AMACR			0	N.A.	0
C1QTNF5			0	N.A.	0
C1QTNF7		-0,209238787	0	N.A.	0
C1QTNF8			0	N.A.	0
C1QTNF9B			0	N.A.	0
C20orf141			0	N.A.	0
C20orf166-AS1			0	N.A.	0
C20orf197			0	N.A.	0
C20orf203			0	N.A.	0
C22orf15			0	N.A.	0
C22orf23		0,049680117	0	N.A.	0
C22orf31			0	N.A.	0
C22orf34			0	N.A.	0
C2orf40			0	N.A.	0
C2orf66			0	N.A.	0
C2orf73		0,060210134	0	N.A.	0
C2orf74		0,012560558	0	N.A.	0
C2orf80			0	N.A.	0
C2orf83			0	N.A.	0

C2orf91		0,059085364	0	N.A.	0
C3orf20			0	N.A.	0
C3orf36			0	N.A.	0
C3orf49			0	N.A.	0
C3orf67-AS1		0,218441627	0	N.A.	0
C3orf80			0	N.A.	0
C4B			0	N.A.	0
C4orf22			0	N.A.	0
C4orf26			0	N.A.	0
C4orf50			0	N.A.	0
C4orf51			0	N.A.	0
C5orf47		0,034357583	0	N.A.	0
C6			0	N.A.	0
C6orf10			0	N.A.	0
C6orf118			0	N.A.	0
C6orf165		-0,252894512	0	N.A.	0
C6orf195			0	N.A.	0
C6orf47-AS1		0,021619	0	N.A.	0
C6orf58		-0,106231977	0	N.A.	0
C7orf33			0	N.A.	0
C7orf34			0	N.A.	0
C7orf69			0	N.A.	0
C7orf72			0	N.A.	0
C8A			0	N.A.	0
C8B			0	N.A.	0
C8orf46		0,042534969	0	N.A.	0
C8orf48			0	N.A.	0
C8orf49		0,107732166	0	N.A.	0
C8orf87			0	N.A.	0
C8orf88		-0,202447485	0	N.A.	0
C9			0	N.A.	0
C9orf106		0,10353456	0	N.A.	0
C9orf129			0	N.A.	0

C9orf131			0	N.A.	0
C9orf139			0	N.A.	0
C9orf170			0	N.A.	0
C9orf173		-0,261848912	0	N.A.	0
C9orf173-AS1		0,173973547	0	N.A.	0
C9orf47			0	N.A.	0
C9orf84		0,013370774	0	N.A.	0
C9orf92			0	N.A.	0
CA10			0	N.A.	0
CA6		0,073451438	0	N.A.	0
CACNA1B			0	N.A.	0
CACNA1C-AS1		-0,080035384	0	N.A.	0
CACNA1C-AS2		-0,139939874	0	N.A.	0
CACNA1G			0	N.A.	0
CACNA1G-AS1			0	N.A.	0
CACNA1I			0	N.A.	0
CACNA1S		0,056783282	0	N.A.	0
CACNA2D1		0,329897066	0	N.A.	0
CACNG3			0	N.A.	0
CACTIN-AS1			0	N.A.	0
CADM3			0	N.A.	0
CADM3-AS1			0	N.A.	0
CAHM			0	N.A.	0
CALCB			0	N.A.	0
CALCR		0,024736465	0	N.A.	0
CALCRL		-0,044453462	0	N.A.	0
CALHM1			0	N.A.	0
CALML3-AS1		0,184641497	0	N.A.	0
CALML6			0	N.A.	0
CALN1			0	N.A.	0
CAMK1G			0	N.A.	0
CAMK2A			0	N.A.	0
CAND2			0	N.A.	0

CAPN11		0,037778938	0	N.A.	0
CAPSL			0	N.A.	0
CARD17			0	N.A.	0
CARTPT			0	N.A.	0
CASC10			0	N.A.	0
CASC18			0	N.A.	0
CASK-AS1			0	N.A.	0
CASQ1			0	N.A.	0
CASR			0	N.A.	0
CATIP		-0,113073	0	N.A.	0
CATSPER4		0,273978834	0	N.A.	0
CAV3			0	N.A.	0
CBLN2			0	N.A.	0
CBY3		0,346059433	0	N.A.	0
CC2D2B			0	N.A.	0
CCDC103			0	N.A.	0
CCDC116		0,102925216	0	N.A.	0
CCDC141			0	N.A.	0
CCDC144NL-AS1		0,047142793	0	N.A.	0
CCDC147-AS1			0	N.A.	0
CCDC155			0	N.A.	0
CCDC158		-0,057605904	0	N.A.	0
CCDC160			0	N.A.	0
CCDC166		0,169844441	0	N.A.	0
CCDC169			0	N.A.	0
CCDC178			0	N.A.	0
CCDC179			0	N.A.	0
CCDC184		-0,054776406	0	N.A.	0
CCDC26			0	N.A.	0
CCDC33		0,033564399	0	N.A.	0
CCDC36			0	N.A.	0
CCDC38		0,039558427	0	N.A.	0
CCDC39		-0,111509386	0	N.A.	0

CCDC42			0	N.A.	0
CCDC62		0,100404708	0	N.A.	0
CCDC63			0	N.A.	0
CCDC65		-0,103696705	0	N.A.	0
CCDC67		-0,13558316	0	N.A.	0
CCDC70			0	N.A.	0
CCDC73		-0,033236238	0	N.A.	0
CCDC79		0,186222427	0	N.A.	0
CCDC8			0	N.A.	0
CCDC83			0	N.A.	0
CCDC87		0,059103248	0	N.A.	0
CCDC89		-0,056663581	0	N.A.	0
CCIN			0	N.A.	0
CCL1			0	N.A.	0
CCL15-CCL14		0,272992736	0	N.A.	0
CCL16		0,118036944	0	N.A.	0
CCL23			0	N.A.	0
CCL27		0,000705319	0	N.A.	0
CCM2L			0	N.A.	0
CCNA1			0	N.A.	0
CCNB3		-0,04776802	0	N.A.	0
CCR2			0	N.A.	0
CCR8			0	N.A.	0
CCR9		0,047550175	0	N.A.	0
CD180			0	N.A.	0
CD1A			0	N.A.	0
CD1B			0	N.A.	0
CD1D		-0,146847718	0	N.A.	0
CD1E		-0,308079787	0	N.A.	0
CD200R1L			0	N.A.	0
CD207			0	N.A.	0
CD209			0	N.A.	0
CD300LD			0	N.A.	0

CD300LG			0	N.A.	0
CD33		0,22885242	0	N.A.	0
CD5L			0	N.A.	0
CDH12			0	N.A.	0
CDH18			0	N.A.	0
CDH20			0	N.A.	0
CDH22			0	N.A.	0
CDH6		-0,077890875	0	N.A.	0
CDH8		0,169294948	0	N.A.	0
CDK15			0	N.A.	0
CDKN2A-AS1			0	N.A.	0
CDO1		-0,05312472	0	N.A.	0
CDR1-AS		-0,059303777	0	N.A.	0
CDRT15L2			0	N.A.	0
CEACAM16			0	N.A.	0
CEACAM18		0,390905335	0	N.A.	0
CECR7			0	N.A.	0
CELF2-AS1			0	N.A.	0
CELF4			0	N.A.	0
CELF6			0	N.A.	0
CER1		0,19358288	0	N.A.	0
CERS3			0	N.A.	0
CES5A			0	N.A.	0
CETP		-0,19208203	0	N.A.	0
CFAP126		-0,229634179	0	N.A.	0
CFAP221			0	N.A.	0
CFAP52			0	N.A.	0
CFAP61		-0,254079073	0	N.A.	0
CFC1			0	N.A.	0
CFHR1			0	N.A.	0
CFHR3		0,166819163	0	N.A.	0
CGB2		0,352993988	0	N.A.	0
CGB7		-0,065629575	0	N.A.	0

CH17-118O6.3		0	N.A.	0
CH17-270A2.2		0	N.A.	0
CH17-302M23.1		0	N.A.	0
CH17-353B19.1	0,212313578	0	N.A.	0
CH17-360D5.3	0,481977331	0	N.A.	0
CH17-437K3.1		0	N.A.	0
CH17-80A12.1	0,151026597	0	N.A.	0
CH507-145C22.1	-0,159945608	0	N.A.	0
CH507-210P18.1	0,278573823	0	N.A.	0
CH507-236L23.1		0	N.A.	0
CH507-24F1.1	-0,06119256	0	N.A.	0
CH507-254M2.1	-0,086214174	0	N.A.	0
CH507-42P11.6	0,364379078	0	N.A.	0
CH507-513H4.1		0	N.A.	0
CH507-513H4.3	-0,115718355	0	N.A.	0
CH507-513H4.5		0	N.A.	0
CH507-528H12.1	0,271778021	0	N.A.	0
CH507-9B2.8		0	N.A.	0
CHAD	0,372381846	0	N.A.	0
CHD5	0,339428679	0	N.A.	0
CHODL		0	N.A.	0
CHRDL1	-0,341721309	0	N.A.	0
CHRDL2	0,018592192	0	N.A.	0
CHRM1	-0,074178516	0	N.A.	0
CHRM2		0	N.A.	0
CHRM4		0	N.A.	0
CHRM5		0	N.A.	0
CHRNA1		0	N.A.	0
CHRNA6		0	N.A.	0
CHRNA9		0	N.A.	0
CHRNB2		0	N.A.	0
CHST2		0	N.A.	0
CHST9		0	N.A.	0

CIB3			0	N.A.	0
CIDEA			0	N.A.	0
CILP			0	N.A.	0
CLDN10-AS1		0,234160984	0	N.A.	0
CLDN11		-0,295132472	0	N.A.	0
CLDN22		0,081138471	0	N.A.	0
CLDN8		0,225817442	0	N.A.	0
CLEC12A		0,054728317	0	N.A.	0
CLEC12B			0	N.A.	0
CLEC17A			0	N.A.	0
CLEC18B			0	N.A.	0
CLEC19A			0	N.A.	0
CLEC1A			0	N.A.	0
CLEC1B			0	N.A.	0
CLEC2L			0	N.A.	0
CLEC3B			0	N.A.	0
CLEC4C			0	N.A.	0
CLEC4D			0	N.A.	0
CLEC4F			0	N.A.	0
CLEC4G			0	N.A.	0
CLEC4M			0	N.A.	0
CLEC6A			0	N.A.	0
CLEC9A		-0,080951034	0	N.A.	0
CLPSL1			0	N.A.	0
CLVS1		0,024083864	0	N.A.	0
CLVS2			0	N.A.	0
CMA1			0	N.A.	0
CMB9-55A18.1			0	N.A.	0
CMP21-97G8.1			0	N.A.	0
CMP21-97G8.2		-0,021182102	0	N.A.	0
CMTM1		0,053293719	0	N.A.	0
CMTM5			0	N.A.	0
CNGA4			0	N.A.	0

CNGB1		0,236951663	0	N.A.	0
CNGB3		-0,350742472	0	N.A.	0
CNKSR2			0	N.A.	0
CNR1			0	N.A.	0
CNR2			0	N.A.	0
CNRIP1		0,002381383	0	N.A.	0
CNTF			0	N.A.	0
CNTN1		-0,12642022	0	N.A.	0
CNTN2			0	N.A.	0
CNTN4			0	N.A.	0
CNTN5		-0,30847373	0	N.A.	0
CNTN6			0	N.A.	0
CNTNAP5			0	N.A.	0
COL18A1-AS1		0,232150643	0	N.A.	0
COL19A1			0	N.A.	0
COL20A1		0,002722114	0	N.A.	0
COL22A1		0,30937273	0	N.A.	0
COL23A1			0	N.A.	0
COL25A1		0,175796347	0	N.A.	0
COL26A1		-0,387758915	0	N.A.	0
COL4A2-AS1			0	N.A.	0
COL4A2-AS2			0	N.A.	0
COL4A3			0	N.A.	0
COL4A5		-0,147290646	0	N.A.	0
COL4A6			0	N.A.	0
COL6A5		0,137208041	0	N.A.	0
COL6A6			0	N.A.	0
CORIN		0,044525659	0	N.A.	0
CORO2B			0	N.A.	0
CORO6		0,045280893	0	N.A.	0
CORO7-PAM16			0	N.A.	0
CORT		-0,115691727	0	N.A.	0
COX6A2			0	N.A.	0

COX8C			0	N.A.	0
CPA1			0	N.A.	0
CPB1			0	N.A.	0
CPB2			0	N.A.	0
CPEB1			0	N.A.	0
CPEB2-AS1			0	N.A.	0
CPED1		-0,273111738	0	N.A.	0
CPLX3		0,143373481	0	N.A.	0
CPN2			0	N.A.	0
CPO			0	N.A.	0
CPZ			0	N.A.	0
CR1L			0	N.A.	0
CR2		-0,294603043	0	N.A.	0
CRB1		-0,068684195	0	N.A.	0
CRHBP		-0,142060103	0	N.A.	0
CRHR2			0	N.A.	0
CRISPLD1			0	N.A.	0
CRLF2			0	N.A.	0
CRP			0	N.A.	0
CRTAC1			0	N.A.	0
CRYBA1			0	N.A.	0
CRYBB1			0	N.A.	0
CRYGD			0	N.A.	0
CRYGN			0	N.A.	0
CSAG1			0	N.A.	0
CSDC2			0	N.A.	0
CSE1L-AS1			0	N.A.	0
CSMD1			0	N.A.	0
CSMD3		0,04468533	0	N.A.	0
CSNK1G2-AS1			0	N.A.	0
CSNK2B-LY6G5B-562		-0,187972307	0	N.A.	0
CSRP3			0	N.A.	0
CST11			0	N.A.	0

CST2		-0,212592089	0	N.A.	0
CST5			0	N.A.	0
CT45A10			0	N.A.	0
CT55			0	N.A.	0
CT62			0	N.A.	0
CTA-150C2.13		0,398138741	0	N.A.	0
CTA-212D2.2		0,059340545	0	N.A.	0
CTA-221G9.11		0,304673468	0	N.A.	0
CTA-223H9.9			0	N.A.	0
CTA-243E7.2			0	N.A.	0
CTA-268H5.14			0	N.A.	0
CTA-305I2.1			0	N.A.	0
CTA-31J9.2			0	N.A.	0
CTA-331P3.1			0	N.A.	0
CTA-339C12.1			0	N.A.	0
CTA-342B11.1			0	N.A.	0
CTA-342B11.2			0	N.A.	0
CTA-363E6.5		0,053870431	0	N.A.	0
CTA-373H7.7			0	N.A.	0
CTA-390C10.9			0	N.A.	0
CTA-392E5.1			0	N.A.	0
CTA-397H3.3		9,04937E-06	0	N.A.	0
CTA-407F11.8			0	N.A.	0
CTA-481E9.4		0,093178046	0	N.A.	0
CTA-503F6.2			0	N.A.	0
CTA-796E4.4			0	N.A.	0
CTA-797E19.2			0	N.A.	0
CTA-833B7.2			0	N.A.	0
CTA-984G1.5			0	N.A.	0
CTA-989H11.1		0,002812394	0	N.A.	0
CTA-992D9.10			0	N.A.	0
CTA-992D9.11			0	N.A.	0
CTAG2			0	N.A.	0

CTAGE1			0	N.A.	0
CTAGE15		-0,079735045	0	N.A.	0
CTAGE4		0,217808664	0	N.A.	0
CTAGE6		-0,08211732	0	N.A.	0
CTAGE8			0	N.A.	0
CTB-107G13.1			0	N.A.	0
CTB-108O6.2			0	N.A.	0
CTB-113P19.1			0	N.A.	0
CTB-113P19.4			0	N.A.	0
CTB-114C7.4			0	N.A.	0
CTB-118P15.2			0	N.A.	0
CTB-11I22.1		0,011035405	0	N.A.	0
CTB-11I22.2			0	N.A.	0
CTB-120L21.1			0	N.A.	0
CTB-12A17.3			0	N.A.	0
CTB-12O2.1		-0,027339268	0	N.A.	0
CTB-133G6.1		-0,225163244	0	N.A.	0
CTB-138E5.1			0	N.A.	0
CTB-147C13.1		0,128160971	0	N.A.	0
CTB-147C22.8		0,322187757	0	N.A.	0
CTB-147C22.9		0,474446859	0	N.A.	0
CTB-151G24.1			0	N.A.	0
CTB-158D10.3			0	N.A.	0
CTB-161M19.4		0,010450598	0	N.A.	0
CTB-167B5.1			0	N.A.	0
CTB-174D11.1			0	N.A.	0
CTB-175E5.7		0,163464693	0	N.A.	0
CTB-176F20.3		-0,033059873	0	N.A.	0
CTB-17P3.4			0	N.A.	0
CTB-180A7.3			0	N.A.	0
CTB-184G21.3			0	N.A.	0
CTB-186G2.1			0	N.A.	0
CTB-186G2.4		0,065040643	0	N.A.	0

CTB-186H2.3		0	N.A.	0
CTB-189B5.3		0	N.A.	0
CTB-191K22.6	0,058828896	0	N.A.	0
CTB-193M12.1		0	N.A.	0
CTB-30L5.1		0	N.A.	0
CTB-31N19.5	0,032892105	0	N.A.	0
CTB-31O20.3	-0,045242429	0	N.A.	0
CTB-31O20.8		0	N.A.	0
CTB-31O20.9		0	N.A.	0
CTB-33G10.11		0	N.A.	0
CTB-35F21.1		0	N.A.	0
CTB-39G8.2	0,059168623	0	N.A.	0
CTB-41I6.1		0	N.A.	0
CTB-41I6.2		0	N.A.	0
CTB-43P18.1	-0,267832545	0	N.A.	0
CTB-49A3.4	-0,178475295	0	N.A.	0
CTB-49A3.5		0	N.A.	0
CTB-4E7.1		0	N.A.	0
CTB-50L17.7		0	N.A.	0
CTB-50L17.9	0,103604969	0	N.A.	0
CTB-55O6.10	0,283046259	0	N.A.	0
CTB-55O6.4		0	N.A.	0
CTB-58E17.9		0	N.A.	0
CTB-60B18.10		0	N.A.	0
CTB-78F1.1		0	N.A.	0
CTB-79E8.2		0	N.A.	0
CTB-83J4.1	0,035865989	0	N.A.	0
CTB-91J4.1		0	N.A.	0
CTB-92J24.3		0	N.A.	0
CTC-228N24.2	-0,261848912	0	N.A.	0
CTC-232P5.3		0	N.A.	0
CTC-242N15.1		0	N.A.	0
CTC-265F19.3		0	N.A.	0

CTC-273B12.5	0,34389655	0	N.A.	0
CTC-276P9.4		0	N.A.	0
CTC-281F24.1		0	N.A.	0
CTC-296K1.3		0	N.A.	0
CTC-297N7.1		0	N.A.	0
CTC-297N7.9		0	N.A.	0
CTC-301O7.4		0	N.A.	0
CTC-325H20.4		0	N.A.	0
CTC-325J23.2		0	N.A.	0
CTC-327F10.5		0	N.A.	0
CTC-329D1.3		0	N.A.	0
CTC-338M12.1	0,343684064	0	N.A.	0
CTC-338M12.2	0,104962059	0	N.A.	0
CTC-339O9.1		0	N.A.	0
CTC-340A15.2		0	N.A.	0
CTC-340I23.2		0	N.A.	0
CTC-343N3.1	-0,028308157	0	N.A.	0
CTC-344H19.4	0,19457346	0	N.A.	0
CTC-360G5.6		0	N.A.	0
CTC-360G5.9		0	N.A.	0
CTC-366B18.2		0	N.A.	0
CTC-384G19.1		0	N.A.	0
CTC-420A11.2		0	N.A.	0
CTC-429P9.1	0,225225324	0	N.A.	0
CTC-429P9.2	-0,119409565	0	N.A.	0
CTC-429P9.4		0	N.A.	0
CTC-431G16.2	-0,110976234	0	N.A.	0
CTC-435M10.3		0	N.A.	0
CTC-435M10.6		0	N.A.	0
CTC-436K13.1		0	N.A.	0
CTC-436K13.2		0	N.A.	0
CTC-436P18.1	-0,099739463	0	N.A.	0
CTC-441N14.1		0	N.A.	0

CTC-441N14.2		0	N.A.	0
CTC-444N24.13	-0,030166881	0	N.A.	0
CTC-448D22.1		0	N.A.	0
CTC-453G23.5		0	N.A.	0
CTC-453G23.8	-0,17055876	0	N.A.	0
CTC-454I21.3	-0,099099305	0	N.A.	0
CTC-457L16.2		0	N.A.	0
CTC-458I2.2	-0,08092989	0	N.A.	0
CTC-459M5.2		0	N.A.	0
CTC-467M3.1		0	N.A.	0
CTC-479C5.10		0	N.A.	0
CTC-482H14.5		0	N.A.	0
CTC-487M23.5	0,044112527	0	N.A.	0
CTC-487M23.7		0	N.A.	0
CTC-492K19.4		0	N.A.	0
CTC-493L21.1		0	N.A.	0
CTC-497E21.3	0,096068791	0	N.A.	0
CTC-498J12.3	0,029615827	0	N.A.	0
CTC-499B15.7		0	N.A.	0
CTC-499J9.1		0	N.A.	0
CTC-506B8.1		0	N.A.	0
CTC-510F12.2		0	N.A.	0
CTC-510F12.7		0	N.A.	0
CTC-518B2.12		0	N.A.	0
CTC-523E23.4		0	N.A.	0
CTC-523E23.5		0	N.A.	0
CTC-529G1.1		0	N.A.	0
CTC-542B22.2	-0,099739463	0	N.A.	0
CTC-546K23.1		0	N.A.	0
CTC-548K16.2		0	N.A.	0
CTC-548K16.5		0	N.A.	0
CTC-558O2.1		0	N.A.	0
CTC-558O2.2	0,09102857	0	N.A.	0

CTC-559E9.4		0	N.A.	0
CTC-575N7.1		0	N.A.	0
CTC-806A22.1	0,214361305	0	N.A.	0
CTCFL		0	N.A.	0
CTD-2002J20.1		0	N.A.	0
CTD-2003C8.2		0	N.A.	0
CTD-2006C1.13		0	N.A.	0
CTD-2006K23.2		0	N.A.	0
CTD-2008L17.1		0	N.A.	0
CTD-2008L17.2		0	N.A.	0
CTD-2012K14.3	-0,327681265	0	N.A.	0
CTD-2012K14.4	-0,314214783	0	N.A.	0
CTD-2013N17.4		0	N.A.	0
CTD-2014B16.3		0	N.A.	0
CTD-2017C7.1		0	N.A.	0
CTD-2017C7.2		0	N.A.	0
CTD-2017F17.1	0,230731372	0	N.A.	0
CTD-2021H9.2	0,402560851	0	N.A.	0
CTD-2021H9.3	0,175370843	0	N.A.	0
CTD-2023M8.1		0	N.A.	0
CTD-2024P10.1		0	N.A.	0
CTD-2024P10.2		0	N.A.	0
CTD-2026D20.2		0	N.A.	0
CTD-2026K11.3		0	N.A.	0
CTD-2026K11.6	0,264498552	0	N.A.	0
CTD-2031P19.5	0,081138471	0	N.A.	0
CTD-2034I21.2	-0,146159673	0	N.A.	0
CTD-2036P10.5	-0,136193838	0	N.A.	0
CTD-2036P10.6		0	N.A.	0
CTD-2044J15.1		0	N.A.	0
CTD-2054N24.2		0	N.A.	0
CTD-2058B24.2		0	N.A.	0
CTD-2061E19.6		0	N.A.	0

CTD-2066L21.3	-0,047231321	0	N.A.	0
CTD-2072I24.1		0	N.A.	0
CTD-2083E4.6		0	N.A.	0
CTD-2089N3.2		0	N.A.	0
CTD-2091N23.1		0	N.A.	0
CTD-2105E13.14	0,023538198	0	N.A.	0
CTD-2113L7.1		0	N.A.	0
CTD-2116N17.1	-0,041233565	0	N.A.	0
CTD-2117L12.1		0	N.A.	0
CTD-2118P12.1	0,073084264	0	N.A.	0
CTD-2126E3.3		0	N.A.	0
CTD-2126E3.4		0	N.A.	0
CTD-2130O13.1	0,179732487	0	N.A.	0
CTD-2134A5.4	0,045817691	0	N.A.	0
CTD-2135D7.5		0	N.A.	0
CTD-2135J3.3		0	N.A.	0
CTD-2140B24.6	-0,097864271	0	N.A.	0
CTD-2142D14.1	0,483024011	0	N.A.	0
CTD-2151A2.1		0	N.A.	0
CTD-2154I11.2	-0,018967617	0	N.A.	0
CTD-2162K18.3		0	N.A.	0
CTD-2162K18.4		0	N.A.	0
CTD-2168K21.2		0	N.A.	0
CTD-2171N6.1	-0,140394041	0	N.A.	0
CTD-2184D3.3		0	N.A.	0
CTD-2184D3.5	0,206870995	0	N.A.	0
CTD-2184D3.6		0	N.A.	0
CTD-2184D3.7		0	N.A.	0
CTD-2192J16.20	0,097114575	0	N.A.	0
CTD-2192J16.22	-0,209886889	0	N.A.	0
CTD-2193P3.2		0	N.A.	0
CTD-2195B23.3		0	N.A.	0
CTD-2196E14.3		0	N.A.	0

CTD-2199O4.1	0,105819084	0	N.A.	0
CTD-2199O4.6		0	N.A.	0
CTD-2201E9.4		0	N.A.	0
CTD-2201G3.1		0	N.A.	0
CTD-2207P18.2		0	N.A.	0
CTD-2213F21.3		0	N.A.	0
CTD-2215E18.1	0,319623583	0	N.A.	0
CTD-2223O18.1	-0,230162589	0	N.A.	0
CTD-2227I18.1	0,179732487	0	N.A.	0
CTD-2231E14.4		0	N.A.	0
CTD-2236F14.1		0	N.A.	0
CTD-2240E14.4		0	N.A.	0
CTD-2240J17.1		0	N.A.	0
CTD-2240J17.3		0	N.A.	0
CTD-2245F17.6		0	N.A.	0
CTD-2246P4.1		0	N.A.	0
CTD-2256P15.5		0	N.A.	0
CTD-2263F21.1	0,146140569	0	N.A.	0
CTD-2265O21.3		0	N.A.	0
CTD-2265O21.7		0	N.A.	0
CTD-2267D19.3		0	N.A.	0
CTD-2269F5.1		0	N.A.	0
CTD-2270F17.1		0	N.A.	0
CTD-2280E9.1		0	N.A.	0
CTD-2281M20.1		0	N.A.	0
CTD-2287O16.4	0,047989625	0	N.A.	0
CTD-2288O8.1		0	N.A.	0
CTD-2291D10.1		0	N.A.	0
CTD-2292M14.1	-0,13275093	0	N.A.	0
CTD-2292P10.2		0	N.A.	0
CTD-2296D1.5		0	N.A.	0
CTD-2298J14.2		0	N.A.	0
CTD-2306M5.1		0	N.A.	0

CTD-2311M21.3		0	N.A.	0
CTD-2313J17.7		0	N.A.	0
CTD-2314B22.1		0	N.A.	0
CTD-2315E11.1		0	N.A.	0
CTD-2320G14.2		0	N.A.	0
CTD-2325A15.5	-0,095581944	0	N.A.	0
CTD-2325P2.3		0	N.A.	0
CTD-2329K10.1		0	N.A.	0
CTD-2330J20.2		0	N.A.	0
CTD-2330K9.3		0	N.A.	0
CTD-2334D19.1		0	N.A.	0
CTD-2337A12.1		0	N.A.	0
CTD-2339F6.1		0	N.A.	0
CTD-2340E1.2		0	N.A.	0
CTD-2341M24.1	0,109736688	0	N.A.	0
CTD-2342N23.3		0	N.A.	0
CTD-2349P21.11		0	N.A.	0
CTD-2349P21.6		0	N.A.	0
CTD-2353F22.1		0	N.A.	0
CTD-2358C21.3	0,376494317	0	N.A.	0
CTD-2358C21.4		0	N.A.	0
CTD-2369P2.5		0	N.A.	0
CTD-2369P2.8	0,088213979	0	N.A.	0
CTD-2373H9.5		0	N.A.	0
CTD-2373N4.5	0,422594587	0	N.A.	0
CTD-2376I20.1		0	N.A.	0
CTD-2376I4.1	-8,40519E-05	0	N.A.	0
CTD-2377D24.4	-0,107397537	0	N.A.	0
CTD-2378E12.1	0,162917966	0	N.A.	0
CTD-2378E21.1		0	N.A.	0
CTD-2380F24.1		0	N.A.	0
CTD-2384B11.2	0,236229665	0	N.A.	0
CTD-2410N18.5		0	N.A.	0

CTD-2501M5.1	0,204747135	0	N.A.	0
CTD-2506J14.1		0	N.A.	0
CTD-2507G9.1		0	N.A.	0
CTD-2509G16.2		0	N.A.	0
CTD-2509G16.5		0	N.A.	0
CTD-2515H24.2		0	N.A.	0
CTD-2517M22.16		0	N.A.	0
CTD-2521M24.6	0,055869398	0	N.A.	0
CTD-2523D13.2	0,266399379	0	N.A.	0
CTD-2524L6.3		0	N.A.	0
CTD-2525I3.2		0	N.A.	0
CTD-2525I3.3		0	N.A.	0
CTD-2525I3.5	0,18479211	0	N.A.	0
CTD-2528A14.5		0	N.A.	0
CTD-2531D15.4		0	N.A.	0
CTD-2531D15.5	0,026295128	0	N.A.	0
CTD-2532K18.2	0,378777871	0	N.A.	0
CTD-2534I21.8		0	N.A.	0
CTD-2535I10.1		0	N.A.	0
CTD-2535L24.2	-0,109404982	0	N.A.	0
CTD-2535L24.3	-0,027339268	0	N.A.	0
CTD-2536I1.3		0	N.A.	0
CTD-2537I9.12	-0,07517481	0	N.A.	0
CTD-2537I9.16		0	N.A.	0
CTD-2537I9.18	0,172741018	0	N.A.	0
CTD-2540L5.6	0,320539817	0	N.A.	0
CTD-2540M10.1		0	N.A.	0
CTD-2541J13.1		0	N.A.	0
CTD-2541M15.3	-0,111333376	0	N.A.	0
CTD-2542L18.1		0	N.A.	0
CTD-2544N14.3	-0,238373313	0	N.A.	0
CTD-2545M3.2		0	N.A.	0
CTD-2545M3.8		0	N.A.	0

CTD-2547L24.4		0	N.A.	0
CTD-2553C6.1		0	N.A.	0
CTD-2554C21.2	-0,07201275	0	N.A.	0
CTD-2554C21.3		0	N.A.	0
CTD-2555A7.3		0	N.A.	0
CTD-2555O16.1		0	N.A.	0
CTD-2555O16.2	0,209176475	0	N.A.	0
CTD-2561B21.5		0	N.A.	0
CTD-2561B21.7		0	N.A.	0
CTD-2561F5.1		0	N.A.	0
CTD-2562J17.4		0	N.A.	0
CTD-2562J17.7		0	N.A.	0
CTD-2583A14.10	-0,108933045	0	N.A.	0
CTD-2583P5.1		0	N.A.	0
CTD-2587H24.5		0	N.A.	0
CTD-2589M5.5		0	N.A.	0
CTD-2600O9.2	0,28525821	0	N.A.	0
CTD-2616J11.11	0,233884123	0	N.A.	0
CTD-2616J11.14	-0,295575684	0	N.A.	0
CTD-2616J11.2		0	N.A.	0
CTD-2616J11.9	-0,144708927	0	N.A.	0
CTD-2619J13.19	-0,013798843	0	N.A.	0
CTD-2619J13.23		0	N.A.	0
CTD-2619J13.3		0	N.A.	0
CTD-2619J13.9		0	N.A.	0
CTD-2620I22.3		0	N.A.	0
CTD-2623N2.11		0	N.A.	0
CTD-2630F21.1	0,045724086	0	N.A.	0
CTD-2631K10.1		0	N.A.	0
CTD-2636A23.2	-0,037820226	0	N.A.	0
CTD-2639E6.9	0,203757399	0	N.A.	0
CTD-2647E9.3		0	N.A.	0
CTD-2647L4.5		0	N.A.	0

CTD-2649C14.3		0	N.A.	0
CTD-2651B20.3		0	N.A.	0
CTD-2653D5.1		0	N.A.	0
CTD-2653M23.3		0	N.A.	0
CTD-2655K5.1	0,138355364	0	N.A.	0
CTD-2659N19.2		0	N.A.	0
CTD-2659N19.9		0	N.A.	0
CTD-3020H12.4		0	N.A.	0
CTD-3028N15.1		0	N.A.	0
CTD-3028N15.3		0	N.A.	0
CTD-3032J10.2		0	N.A.	0
CTD-3035D6.2		0	N.A.	0
CTD-3035K23.6		0	N.A.	0
CTD-3035K23.7	-0,087969201	0	N.A.	0
CTD-3037G24.5	0,132375788	0	N.A.	0
CTD-3051D23.1		0	N.A.	0
CTD-3064H18.2		0	N.A.	0
CTD-3064H18.4		0	N.A.	0
CTD-3065B20.2		0	N.A.	0
CTD-3065B20.3	0,219641412	0	N.A.	0
CTD-3065J16.6		0	N.A.	0
CTD-3073N11.9		0	N.A.	0
CTD-3076O17.1		0	N.A.	0
CTD-3076O17.2		0	N.A.	0
CTD-3080P12.3		0	N.A.	0
CTD-3099C6.7	-0,040171218	0	N.A.	0
CTD-3105H18.18		0	N.A.	0
CTD-3105H18.7	0,079582923	0	N.A.	0
CTD-3110H11.1	-0,242612079	0	N.A.	0
CTD-3113P16.5	0,007353581	0	N.A.	0
CTD-3116E22.7	0,092101135	0	N.A.	0
CTD-3116E22.8		0	N.A.	0
CTD-3118D11.2		0	N.A.	0

CTD-3137H5.1			0	N.A.	0
CTD-3154N5.1		-0,174148215	0	N.A.	0
CTD-3154N5.2			0	N.A.	0
CTD-3162L10.1		0,001047933	0	N.A.	0
CTD-3179P9.1			0	N.A.	0
CTD-3179P9.2			0	N.A.	0
CTD-3187F8.14		0,140134487	0	N.A.	0
CTD-3193K9.11			0	N.A.	0
CTD-3193O13.11			0	N.A.	0
CTD-3193O13.13			0	N.A.	0
CTD-3193O13.14			0	N.A.	0
CTD-3194G12.1			0	N.A.	0
CTD-3194G12.2			0	N.A.	0
CTD-3203P2.1		0,125886042	0	N.A.	0
CTD-3214H19.16			0	N.A.	0
CTD-3214H19.6		0,094967225	0	N.A.	0
CTD-3220F14.1		0,034920916	0	N.A.	0
CTD-3220F14.3			0	N.A.	0
CTD-3224I3.3			0	N.A.	0
CTD-3224K15.2		0,392132009	0	N.A.	0
CTD-3224K15.3		-0,189787438	0	N.A.	0
CTD-3234P18.6		0,318719311	0	N.A.	0
CTD-3239E11.2			0	N.A.	0
CTD-3252C9.2		-0,087515972	0	N.A.	0
CTNNA3		0,050167029	0	N.A.	0
CTNND2			0	N.A.	0
CUBN		0,221072751	0	N.A.	0
CUX2			0	N.A.	0
CUZD1			0	N.A.	0
CX3CR1		-0,062156001	0	N.A.	0
CXCR2			0	N.A.	0
CXorf21			0	N.A.	0
CXorf22		-0,069489142	0	N.A.	0

CXorf58		-0,193214118	0	N.A.	0
CYP11A1			0	N.A.	0
CYP1A2		0,043898844	0	N.A.	0
CYP1B1-AS1		-0,061043867	0	N.A.	0
CYP21A2			0	N.A.	0
CYP26C1			0	N.A.	0
CYP2A6		0,018592192	0	N.A.	0
CYP2C19		0,086940096	0	N.A.	0
CYP2C8			0	N.A.	0
CYP2F1			0	N.A.	0
CYP4A11		0,123950764	0	N.A.	0
CYP4A22			0	N.A.	0
CYP4F22			0	N.A.	0
CYP4F26P			0	N.A.	0
CYP7A1		0,057294996	0	N.A.	0
CYP7B1			0	N.A.	0
CYSLTR2			0	N.A.	0
D4S234E		-0,248711153	0	N.A.	0
DACH2			0	N.A.	0
DAO			0	N.A.	0
DAW1			0	N.A.	0
DAZL			0	N.A.	0
DBET		-0,207959664	0	N.A.	0
DBH			0	N.A.	0
DBX2			0	N.A.	0
DCANP1			0	N.A.	0
DCC		-0,274153029	0	N.A.	0
DCDC1		0,075324254	0	N.A.	0
DCDC2B		0,322550317	0	N.A.	0
DCDC2C			0	N.A.	0
DCHS2			0	N.A.	0
DCLK3			0	N.A.	0
DCSTAMP			0	N.A.	0

DCT			0	N.A.	0
DDR1-AS1			0	N.A.	0
DDX25		0,001035877	0	N.A.	0
DDX4			0	N.A.	0
DEFA3			0	N.A.	0
DEFB119			0	N.A.	0
DEFB124			0	N.A.	0
DENND5B-AS1			0	N.A.	0
DEPDC1-AS1		0,143789501	0	N.A.	0
DGAT2L6			0	N.A.	0
DGCR9			0	N.A.	0
DHRS7C			0	N.A.	0
DIRAS1			0	N.A.	0
DIRC1			0	N.A.	0
DIRC3		-0,017052547	0	N.A.	0
DIRC3-AS1			0	N.A.	0
DKFZP434A062			0	N.A.	0
DKFZP434K028		0,3562943	0	N.A.	0
DKFZP434L187			0	N.A.	0
DKFZp779M0652			0	N.A.	0
DKK2		-0,025234084	0	N.A.	0
DLEU7		0,210572473	0	N.A.	0
DLGAP1-AS5		-0,084834724	0	N.A.	0
DLX1			0	N.A.	0
DLX5			0	N.A.	0
DMP1			0	N.A.	0
DMRT3			0	N.A.	0
DNAH10OS			0	N.A.	0
DNAH8			0	N.A.	0
DNAI1			0	N.A.	0
DNAI2			0	N.A.	0
DNAJB13			0	N.A.	0
DNAJB5-AS1			0	N.A.	0

DNAJB7		-0,009238789	0	N.A.	0
DNAJC5G			0	N.A.	0
DNTT			0	N.A.	0
DOCK3		0,322520926	0	N.A.	0
DOCK4-AS1			0	N.A.	0
DOK5			0	N.A.	0
DOK6			0	N.A.	0
DPEP3			0	N.A.	0
DPP6		0,357671948	0	N.A.	0
DPP9-AS1			0	N.A.	0
DPPA4			0	N.A.	0
DPY19L2		0,109987327	0	N.A.	0
DPYD-AS1			0	N.A.	0
DPYS			0	N.A.	0
DPYSL4		0,008375974	0	N.A.	0
DPYSL5			0	N.A.	0
DRC7		0,068678829	0	N.A.	0
DRD5		0,301828534	0	N.A.	0
DRICH1		0,370964876	0	N.A.	0
DSC1			0	N.A.	0
DSCAM			0	N.A.	0
DSCAS		0,07973725	0	N.A.	0
DSG1		0,051240824	0	N.A.	0
DSG1-AS1		0,06262993	0	N.A.	0
DUSP13			0	N.A.	0
DYDC2			0	N.A.	0
EBF2		0,235984239	0	N.A.	0
EBF3			0	N.A.	0
ECT2L			0	N.A.	0
EDDM3A			0	N.A.	0
EDNRB-AS1			0	N.A.	0
EDRF1-AS1			0	N.A.	0
EFCAB1			0	N.A.	0

EFCAB10		0,005744592	0	N.A.	0
EFCAB14-AS1			0	N.A.	0
EFCAB3		-0,224426966	0	N.A.	0
EFCAB9			0	N.A.	0
EFS			0	N.A.	0
EGFLAM		0,081230251	0	N.A.	0
EGFLAM-AS4			0	N.A.	0
EHMT2-AS1		0,044279686	0	N.A.	0
ELANE			0	N.A.	0
ELAVL4		0,063444968	0	N.A.	0
ELMOD1			0	N.A.	0
ELOVL2			0	N.A.	0
ELOVL2-AS1			0	N.A.	0
ELOVL4		-0,235290495	0	N.A.	0
ELSPBP1			0	N.A.	0
EMILIN3			0	N.A.	0
EMR3			0	N.A.	0
EMX2			0	N.A.	0
ENAM			0	N.A.	0
ENOX1		0,087504759	0	N.A.	0
ENPP6			0	N.A.	0
ENTHD1			0	N.A.	0
EOMES		-0,104937672	0	N.A.	0
EP300-AS1		-0,141782174	0	N.A.	0
EPGN			0	N.A.	0
EPHA3			0	N.A.	0
EPHA5			0	N.A.	0
EPHA6			0	N.A.	0
EPHA7			0	N.A.	0
EPHA8		0,155969132	0	N.A.	0
EPO			0	N.A.	0
EPPIN		-0,084642708	0	N.A.	0
EPYC		0,212739267	0	N.A.	0

ERAS		0,09048175	0	N.A.	0
ERCC6-PGBD3			0	N.A.	0
ERICH1-AS1		0,092101135	0	N.A.	0
ERICH6			0	N.A.	0
ERMN		0,191537343	0	N.A.	0
ERVFRD-1			0	N.A.	0
ERVH-1			0	N.A.	0
ERVMER61-1			0	N.A.	0
ESR1			0	N.A.	0
ESRRB		0,027369755	0	N.A.	0
ESX1			0	N.A.	0
ETV3L			0	N.A.	0
EVC			0	N.A.	0
EVC2			0	N.A.	0
EVPLL		0,101221679	0	N.A.	0
EVX2		-0,133570526	0	N.A.	0
EXD1			0	N.A.	0
EYA4		-0,04779246	0	N.A.	0
EZR-AS1			0	N.A.	0
F10-AS1			0	N.A.	0
F11			0	N.A.	0
F2			0	N.A.	0
F2RL2			0	N.A.	0
F8A2			0	N.A.	0
FABP12		0,189663764	0	N.A.	0
FABP7			0	N.A.	0
FAM124B			0	N.A.	0
FAM133A			0	N.A.	0
FAM135B		-0,001750179	0	N.A.	0
FAM138B			0	N.A.	0
FAM150B		0,21870844	0	N.A.	0
FAM151A			0	N.A.	0
FAM153A			0	N.A.	0

FAM153C			0	N.A.	0
FAM154A		-0,271897679	0	N.A.	0
FAM157B		0,190510123	0	N.A.	0
FAM159B		0,13144423	0	N.A.	0
FAM162B			0	N.A.	0
FAM163A			0	N.A.	0
FAM163B		0,287127054	0	N.A.	0
FAM169B		0,164300885	0	N.A.	0
FAM170A			0	N.A.	0
FAM170B			0	N.A.	0
FAM170B-AS1			0	N.A.	0
FAM180A			0	N.A.	0
FAM180B			0	N.A.	0
FAM182B		0,061048125	0	N.A.	0
FAM196A			0	N.A.	0
FAM198A			0	N.A.	0
FAM19A1			0	N.A.	0
FAM19A4			0	N.A.	0
FAM19A5			0	N.A.	0
FAM20A		-0,356598221	0	N.A.	0
FAM216B			0	N.A.	0
FAM221B			0	N.A.	0
FAM222A-AS1		-0,256395722	0	N.A.	0
FAM228A		0,004537914	0	N.A.	0
FAM26E			0	N.A.	0
FAM43B			0	N.A.	0
FAM53B-AS1			0	N.A.	0
FAM66A			0	N.A.	0
FAM69C			0	N.A.	0
FAM71B			0	N.A.	0
FAM72B		-0,067803276	0	N.A.	0
FAM74A6			0	N.A.	0
FAM81B		-0,225859288	0	N.A.	0

FAM83A-AS1			0	N.A.	0
FAM86B2		0,114512939	0	N.A.	0
FAM87A		-0,076351601	0	N.A.	0
FAM87B			0	N.A.	0
FAM90A1		0,243282181	0	N.A.	0
FAM90A26			0	N.A.	0
FAM9B			0	N.A.	0
FANCD2OS			0	N.A.	0
FARSA-AS1			0	N.A.	0
FASLG		0,122973633	0	N.A.	0
FAT2			0	N.A.	0
FAT3			0	N.A.	0
FAT4		-0,290807184	0	N.A.	0
FATE1			0	N.A.	0
FBN3			0	N.A.	0
FBXL7			0	N.A.	0
FBXO3-AS1		0,273384089	0	N.A.	0
FBXO39		0,159654992	0	N.A.	0
FBXO40			0	N.A.	0
FBXO47			0	N.A.	0
FCGR1A		-0,13150946	0	N.A.	0
FCGR1B		0,010504664	0	N.A.	0
FCGR3B		-0,215772573	0	N.A.	0
FCN2			0	N.A.	0
FCRL1			0	N.A.	0
FCRL4			0	N.A.	0
FCRL6		-0,149803229	0	N.A.	0
FDCSP		-0,281355771	0	N.A.	0
FER1L5			0	N.A.	0
FFAR1			0	N.A.	0
FFAR3			0	N.A.	0
FGF1			0	N.A.	0
FGF10			0	N.A.	0

FGF10-AS1			0	N.A.	0
FGF12-AS3			0	N.A.	0
FGF13-AS1			0	N.A.	0
FGF14			0	N.A.	0
FGF16			0	N.A.	0
FGF23			0	N.A.	0
FGF4			0	N.A.	0
FGF5			0	N.A.	0
FGG		0,29175436	0	N.A.	0
FHL5			0	N.A.	0
FIBIN		-0,058138904	0	N.A.	0
FIGLA			0	N.A.	0
FKSG59			0	N.A.	0
FKSG63		0,186222427	0	N.A.	0
FLG-AS1			0	N.A.	0
FLG2		0,009743993	0	N.A.	0
FLJ16779			0	N.A.	0
FLJ33360		0,037909559	0	N.A.	0
FLJ35934			0	N.A.	0
FLJ38122			0	N.A.	0
FLJ39080			0	N.A.	0
FLJ40288			0	N.A.	0
FLJ41941			0	N.A.	0
FLJ42969			0	N.A.	0
FLJ43879		0,257172813	0	N.A.	0
FLJ45079			0	N.A.	0
FLRT1			1	none	0
FLRT2			0	N.A.	0
FLT3		-0,088935629	0	N.A.	0
FMN2		-0,099509523	0	N.A.	0
FMO1		-0,189553819	0	N.A.	0
FMO2			0	N.A.	0
FMO3		0,352553131	0	N.A.	0

FNDC7		-0,02543432	0	N.A.	0
FNDC8		-0,095396813	0	N.A.	0
FNDC9			0	N.A.	0
FO538757.3			0	N.A.	0
FOLH1			0	N.A.	0
FOXB1		0,057484978	0	N.A.	0
FOXC2-AS1		-0,126463422	0	N.A.	0
FOXD3		-0,029286771	0	N.A.	0
FOXD4L3			0	N.A.	0
FOXD4L5			0	N.A.	0
FOXE3		-0,289123872	0	N.A.	0
FOXF2			0	N.A.	0
FOXI2			0	N.A.	0
FOXI3			0	N.A.	0
FOXL2			0	N.A.	0
FPGT-TNNI3K		-0,040000788	0	N.A.	0
FREM3		-0,148445782	0	N.A.	0
FRMD6-AS2			0	N.A.	0
FRMD7			0	N.A.	0
FRMPD4			0	N.A.	0
FRY-AS1			0	N.A.	0
FSBP		-0,181421577	0	N.A.	0
FSD2		0,269966323	0	N.A.	0
FTCD			0	N.A.	0
FUT5			0	N.A.	0
FXYD6-FXYD2			0	N.A.	0
G6PC2			0	N.A.	0
GABBR2			0	N.A.	0
GABRA1			0	N.A.	0
GABRA3			0	N.A.	0
GABRA5			0	N.A.	0
GABRB3		0,089439317	0	N.A.	0
GABRD		-0,080218077	0	N.A.	0

GABRG3			0	N.A.	0
GABRQ			0	N.A.	0
GABRR1		-0,018307101	0	N.A.	0
GADL1			0	N.A.	0
GAFA3		0,2607463	0	N.A.	0
GAGE10			0	N.A.	0
GAL3ST2		-0,11173399	0	N.A.	0
GAL3ST3			0	N.A.	0
GALNT13			0	N.A.	0
GALNT15		0,001802683	0	N.A.	0
GALR1			0	N.A.	0
GAP43			0	N.A.	0
GAPDHS			0	N.A.	0
GAPT		-0,15936099	0	N.A.	0
GAS8-AS1		-0,036120294	0	N.A.	0
GATA1			0	N.A.	0
GATA5			0	N.A.	0
GATSL2		0,259189226	0	N.A.	0
GBP6		0,244280816	0	N.A.	0
GBP7		-0,042226885	0	N.A.	0
GC		0,18726914	0	N.A.	0
GCGR		0,030074965	0	N.A.	0
GCKR			0	N.A.	0
GCM1			0	N.A.	0
GCSAM			0	N.A.	0
GCSAML		-0,086047992	0	N.A.	0
GCSAML-AS1			0	N.A.	0
GDAP1L1			0	N.A.	0
GDF3			0	N.A.	0
GDF5			0	N.A.	0
GDF5OS			0	N.A.	0
GDF6			0	N.A.	0
GDF7			0	N.A.	0

GDNF			0	N.A.	0
GDNF-AS1			0	N.A.	0
GDPD4		0,062658061	0	N.A.	0
GFRA2			0	N.A.	0
GFRA3			0	N.A.	0
GGTLC1		0,284137373	0	N.A.	0
GH1			0	N.A.	0
GHRHR		0,014871809	0	N.A.	0
GIMAP6		0,003154696	0	N.A.	0
GIMAP8			0	N.A.	0
GIP		0,237871634	0	N.A.	0
GIPC3			0	N.A.	0
GJA5			0	N.A.	0
GJB7		0,134236857	0	N.A.	0
GJD3			0	N.A.	0
GJD4			0	N.A.	0
GKN2			0	N.A.	0
GLI3		-0,04228731	0	N.A.	0
GLIPR1L1			0	N.A.	0
GLIS1			0	N.A.	0
GLP2R			0	N.A.	0
GLRA1			0	N.A.	0
GLRA3			0	N.A.	0
GLRB			0	N.A.	0
GLT1D1		0,374153739	0	N.A.	0
GLUD2		-0,107421204	0	N.A.	0
GLYAT			0	N.A.	0
GLYATL2			0	N.A.	0
GML			0	N.A.	0
GNAT1			0	N.A.	0
GNAT2		-0,295524546	0	N.A.	0
GNG3			0	N.A.	0
GNRH2			0	N.A.	0

GOLGA6L1			0	N.A.	0
GOLGA6L6			0	N.A.	0
GOLGA8G			0	N.A.	0
GOLGA8H			0	N.A.	0
GOLGA8M			0	N.A.	0
GOLGA8Q		-0,032138145	0	N.A.	0
GOLGA8S		0,34389655	0	N.A.	0
GOT1L1			0	N.A.	0
GP5			0	N.A.	0
GP9			0	N.A.	0
GPAT2		-0,198857481	0	N.A.	0
GPC5			0	N.A.	0
GPC6		0,057820363	0	N.A.	0
GPHA2			0	N.A.	0
GPIHBP1			0	N.A.	0
GPM6A			0	N.A.	0
GPR1			0	N.A.	0
GPR111			0	N.A.	0
GPR116		0,169820266	0	N.A.	0
GPR12			0	N.A.	0
GPR141		0,089715331	0	N.A.	0
GPR142		-0,195763393	0	N.A.	0
GPR15			0	N.A.	0
GPR156			0	N.A.	0
GPR174			0	N.A.	0
GPR179			0	N.A.	0
GPR182			0	N.A.	0
GPR21			0	N.A.	0
GPR22		-0,082307229	0	N.A.	0
GPR27			0	N.A.	0
GPR31		-0,094053427	0	N.A.	0
GPR34		-0,368580876	0	N.A.	0
GPR42			0	N.A.	0

GPR52			0	N.A.	0
GPR61		0,044211482	0	N.A.	0
GPR62			0	N.A.	0
GPR68		0,075301832	0	N.A.	0
GPR78			0	N.A.	0
GPR88		-0,143942637	0	N.A.	0
GPR97			0	N.A.	0
GREM2		-0,04779246	0	N.A.	0
GRIA3			0	N.A.	0
GRIA4			0	N.A.	0
GRID2		-0,025234084	0	N.A.	0
GRIK1-AS1			0	N.A.	0
GRIK3			0	N.A.	0
GRIK5		0,068107124	0	N.A.	0
GRIN2A			0	N.A.	0
GRIN3B			0	N.A.	0
GRM2			0	N.A.	0
GRM5			0	N.A.	0
GRM7-AS1			0	N.A.	0
GRP			0	N.A.	0
GRPEL2-AS1			0	N.A.	0
GRPR			0	N.A.	0
GS1-114I9.3			0	N.A.	0
GS1-120K12.4		0,082061365	0	N.A.	0
GS1-166A23.1		0,014443976	0	N.A.	0
GS1-204I12.4		0,000133094	0	N.A.	0
GS1-24F4.2			0	N.A.	0
GS1-304P7.3			0	N.A.	0
GS1-57L11.1		0,151424471	0	N.A.	0
GS1-72M22.1		-0,05245725	0	N.A.	0
GSDMC		0,156228396	0	N.A.	0
GSG1			0	N.A.	0
GSG1L			0	N.A.	0

GSTA2			1	none	0
GSTM1		-0,269249097	0	N.A.	0
GSTM5		-0,0247584	0	N.A.	0
GTF2A1L			0	N.A.	0
GTF3C2-AS1		-0,00166296	0	N.A.	0
GTSCR1			0	N.A.	0
GTSF1		-0,005704487	0	N.A.	0
GTSF1L			0	N.A.	0
GUCA1A		-0,223969799	0	N.A.	0
GUCA1B		0,050472017	0	N.A.	0
GUCY1B3			0	N.A.	0
GUCY2F			0	N.A.	0
GYPB			0	N.A.	0
GYPE			0	N.A.	0
H2AFB1			0	N.A.	0
H2BFM			0	N.A.	0
HAND1			0	N.A.	0
HAND2		-0,109964216	0	N.A.	0
HAO1		0,074120493	0	N.A.	0
HAO2			0	N.A.	0
HAPLN2		0,048683863	0	N.A.	0
HAPLN4		0,161501152	0	N.A.	0
HAS1		-0,077078527	0	N.A.	0
HBD			0	N.A.	0
HBG2		0,048148047	0	N.A.	0
HBM			0	N.A.	0
HBQ1		-0,315595948	0	N.A.	0
HBZ			0	N.A.	0
HCCAT5			0	N.A.	0
HCFC1-AS1			0	N.A.	0
HCG16			0	N.A.	0
HCG21			0	N.A.	0
HCG22			0	N.A.	0

HCG23			0	N.A.	0
HCN1		0,059381636	0	N.A.	0
HCN4		0,269706999	0	N.A.	0
HCRTR1			0	N.A.	0
HCRTR2			0	N.A.	0
HEATR9			0	N.A.	0
HEMGN			0	N.A.	0
HEPACAM			0	N.A.	0
HEPN1			0	N.A.	0
HFE2			0	N.A.	0
HFM1		-0,000928656	0	N.A.	0
HGF			0	N.A.	0
HHIP			0	N.A.	0
HHIPL2			0	N.A.	0
HHLA1		0,352229869	0	N.A.	0
HID1-AS1			0	N.A.	0
HIF1A-AS1			0	N.A.	0
HIGD1B		-0,101869378	0	N.A.	0
HIPK4			0	N.A.	0
HIST1H1A			0	N.A.	0
HIST1H1T			0	N.A.	0
HIST1H4F			0	N.A.	0
HIST1H4L		-0,075746223	0	N.A.	0
HIST2H3PS2			0	N.A.	0
HK3		-0,075287494	0	N.A.	0
HLA-DQB1-AS1			0	N.A.	0
HLX-AS1			0	N.A.	0
HMGCLL1			0	N.A.	0
HMHB1			0	N.A.	0
HMP19			0	N.A.	0
HMX2			0	N.A.	0
HNRNPUL2-BSCL2		0,014071285	0	N.A.	0
HORMAD2			0	N.A.	0

HORMAD2-AS1			0	N.A.	0
HOXB-AS2			0	N.A.	0
HOXB1		0,039305884	0	N.A.	0
HOXC-AS1			0	N.A.	0
HOXC12			0	N.A.	0
HOXC13		0,340769968	0	N.A.	0
HP			0	N.A.	0
HP09025			0	N.A.	0
HPCAL4		-0,068586	0	N.A.	0
HPD			0	N.A.	0
HPN-AS1			0	N.A.	0
HPR			0	N.A.	0
HPSE2			0	N.A.	0
HRG		0,076765674	0	N.A.	0
HRH2			0	N.A.	0
HRH4		0,047156587	0	N.A.	0
HRNR		0,335806186	0	N.A.	0
HS3ST2		0,460177339	0	N.A.	0
HS3ST3A1			0	N.A.	0
HSD11B1		0,231837217	0	N.A.	0
HSD3B1		0,068526457	0	N.A.	0
HSD3B2			0	N.A.	0
HSF5			0	N.A.	0
HSPA12B		0,084962858	0	N.A.	0
HSPB2			0	N.A.	0
HSPE1-MOB4		0,117225074	0	N.A.	0
HTN1			0	N.A.	0
HTR2B		-0,047203356	0	N.A.	0
HTR3C			0	N.A.	0
HTR6		0,136022464	0	N.A.	0
HTR7			0	N.A.	0
HYAL4		0,275571916	0	N.A.	0
HZGJ			0	N.A.	0

IBA57-AS1		0,078120771	0	N.A.	0
IBSP		-0,239893348	0	N.A.	0
IDI2		0,047932409	0	N.A.	0
IDO2			0	N.A.	0
IFNB1			0	N.A.	0
IFNE		0,430719971	0	N.A.	0
IFNG-AS1		-0,225163244	0	N.A.	0
IGDCC4			0	N.A.	0
IGFBP7-AS1			0	N.A.	0
IGFL3			0	N.A.	0
IGFN1		0,092740075	0	N.A.	0
IGHE			0	N.A.	0
IGHEP1			0	N.A.	0
IGHEP2			0	N.A.	0
IGHV1-12			0	N.A.	0
IGHV1-14			0	N.A.	0
IGHV1-17			0	N.A.	0
IGHV1-3			0	N.A.	0
IGHV1-45			0	N.A.	0
IGHV1-46			0	N.A.	0
IGHV1-58			0	N.A.	0
IGHV1-67			0	N.A.	0
IGHV1-69			0	N.A.	0
IGHV1OR15-2			0	N.A.	0
IGHV1OR15-9			0	N.A.	0
IGHV2-26			0	N.A.	0
IGHV2-70		-0,078534737	0	N.A.	0
IGHV2OR16-5			0	N.A.	0
IGHV3-11		-0,261928123	0	N.A.	0
IGHV3-13			0	N.A.	0
IGHV3-19			0	N.A.	0
IGHV3-20			0	N.A.	0
IGHV3-22			0	N.A.	0

IGHV3-33			0	N.A.	0
IGHV3-35			0	N.A.	0
IGHV3-41			0	N.A.	0
IGHV3-42			0	N.A.	0
IGHV3-52			0	N.A.	0
IGHV3-62			0	N.A.	0
IGHV3-64			0	N.A.	0
IGHV3-71			0	N.A.	0
IGHV3-72			0	N.A.	0
IGHV3OR15-7			0	N.A.	0
IGHV3OR16-13			0	N.A.	0
IGHV3OR16-8			0	N.A.	0
IGHV3OR16-9			0	N.A.	0
IGHV4-28			0	N.A.	0
IGHV4-31			0	N.A.	0
IGHV4-4			0	N.A.	0
IGHV4-55			0	N.A.	0
IGHV4-61		-0,225163244	0	N.A.	0
IGHV5-78		-0,000597916	0	N.A.	0
IGHV6-1			0	N.A.	0
IGHV7-34-1			0	N.A.	0
IGKV1-13			0	N.A.	0
IGKV1-9			0	N.A.	0
IGKV1D-12			0	N.A.	0
IGKV1D-13			0	N.A.	0
IGKV1D-16			0	N.A.	0
IGKV1D-33			0	N.A.	0
IGKV1D-37			0	N.A.	0
IGKV1D-39			0	N.A.	0
IGKV1D-42			0	N.A.	0
IGKV1D-43			0	N.A.	0
IGKV1D-8		-0,291545473	0	N.A.	0
IGKV1OR-2			0	N.A.	0

IGKV1OR2-108			0	N.A.	0
IGKV2-18			0	N.A.	0
IGKV2-26			0	N.A.	0
IGKV2-28			0	N.A.	0
IGKV2-29			0	N.A.	0
IGKV2D-28			0	N.A.	0
IGKV2D-29			0	N.A.	0
IGKV2D-30			0	N.A.	0
IGKV2D-40			0	N.A.	0
IGKV2OR22-3			0	N.A.	0
IGKV2OR22-4			0	N.A.	0
IGKV3-7			0	N.A.	0
IGKV3D-11			0	N.A.	0
IGKV3D-15			0	N.A.	0
IGKV3D-20			0	N.A.	0
IGKV3OR2-268			0	N.A.	0
IGKV5-2			0	N.A.	0
IGKV6D-21			0	N.A.	0
IGKV6D-41			0	N.A.	0
IGKV7-3			0	N.A.	0
IGLC4			0	N.A.	0
IGLC5			0	N.A.	0
IGLC6			0	N.A.	0
IGLJCOR18			0	N.A.	0
IGLV1-36			0	N.A.	0
IGLV1-41		-0,035408727	0	N.A.	0
IGLV1-50			0	N.A.	0
IGLV10-54			0	N.A.	0
IGLV11-55			0	N.A.	0
IGLV2-18			0	N.A.	0
IGLV2-28			0	N.A.	0
IGLV2-34			0	N.A.	0
IGLV2-5			0	N.A.	0

IGLV3-12			0	N.A.	0
IGLV3-16			0	N.A.	0
IGLV3-27			0	N.A.	0
IGLV4-3			0	N.A.	0
IGLV4-60			0	N.A.	0
IGLV4-69			0	N.A.	0
IGLV5-37			0	N.A.	0
IGLV5-45			0	N.A.	0
IGLV5-48			0	N.A.	0
IGLV5-52		0,373897891	0	N.A.	0
IGLV7-35			0	N.A.	0
IGLV7-43		-0,013291443	0	N.A.	0
IGLV7-46		-0,111727881	0	N.A.	0
IGLV9-49			0	N.A.	0
IGLVI-70			0	N.A.	0
IGLVIV-66-1			0	N.A.	0
IGSF10			0	N.A.	0
IGSF11		-0,05648196	0	N.A.	0
IGSF21			0	N.A.	0
IGSF5		0,376867434	0	N.A.	0
IL12A-AS1			0	N.A.	0
IL12B			0	N.A.	0
IL12RB2		-0,057605904	0	N.A.	0
IL13			0	N.A.	0
IL17B		-0,075670816	0	N.A.	0
IL17F			0	N.A.	0
IL19			0	N.A.	0
IL1RAPL1			0	N.A.	0
IL1RAPL2			0	N.A.	0
IL20			0	N.A.	0
IL21			0	N.A.	0
IL21-AS1			0	N.A.	0
IL21R-AS1		0,03408571	0	N.A.	0

IL22RA2			0	N.A.	0
IL25			0	N.A.	0
IL27			0	N.A.	0
IL3			0	N.A.	0
IL36B			0	N.A.	0
IL4			0	N.A.	0
IL5RA			0	N.A.	0
IL9R			0	N.A.	0
IMPG1			0	N.A.	0
INA			0	N.A.	0
INHA			0	N.A.	0
INMT-FAM188B			0	N.A.	0
INSL3			0	N.A.	0
INSM2			0	N.A.	0
IP6K3			0	N.A.	0
IQCA1			0	N.A.	0
IQCA1-AS1			0	N.A.	0
IQCF1		0,356104625	0	N.A.	0
IQCJ-SCHIP1		-0,036843357	0	N.A.	0
IQCJ-SCHIP1-AS1			0	N.A.	0
IQSEC3			0	N.A.	0
IRG1			0	N.A.	0
IRGC			0	N.A.	0
IRGM			0	N.A.	0
IRX1			0	N.A.	0
IRX6			0	N.A.	0
ISLR2			0	N.A.	0
ISM1			0	N.A.	0
ISM1-AS1			0	N.A.	0
ITGA8			0	N.A.	0
ITGAD			0	N.A.	0
ITGB5-AS1			0	N.A.	0
ITIH1			0	N.A.	0

ITIH2			0	N.A.	0
ITIH3			0	N.A.	0
ITIH4			0	N.A.	0
ITIH6		-0,082069374	0	N.A.	0
ITPR1-AS1			0	N.A.	0
IVL		-0,015981438	0	N.A.	0
JADRR			0	N.A.	0
JAKMIP2		0,142471726	0	N.A.	0
JAKMIP3			0	N.A.	0
JAZF1-AS1			0	N.A.	0
JMJD7			0	N.A.	0
JPH2			0	N.A.	0
JPH3			0	N.A.	0
KANK4			0	N.A.	0
KB-1043D8.6			0	N.A.	0
KB-1107E3.1			0	N.A.	0
KB-1184D12.1		0,197054287	0	N.A.	0
KB-1254G8.1		-0,047274872	0	N.A.	0
KB-1410C5.3		0,214531827	0	N.A.	0
KB-1440D3.14			0	N.A.	0
KB-1448A5.1			0	N.A.	0
KB-1458E12.1			0	N.A.	0
KB-1466C5.1			0	N.A.	0
KB-1507C5.3			0	N.A.	0
KB-1507C5.4		0,101741538	0	N.A.	0
KB-1592A4.15			0	N.A.	0
KB-1615E4.3			0	N.A.	0
KB-173C10.1			0	N.A.	0
KB-173C10.2			0	N.A.	0
KB-1836B5.4			0	N.A.	0
KB-431C1.5			0	N.A.	0
KC6			0	N.A.	0
KCNA1			0	N.A.	0

KCNA2			0	N.A.	0
KCNA3		-0,288993621	0	N.A.	0
KCNA5			0	N.A.	0
KCNAB1		0,177532633	0	N.A.	0
KCNB1			0	N.A.	0
KCNB2			0	N.A.	0
KCNC1			0	N.A.	0
KCNC4-AS1			0	N.A.	0
KCNG2			0	N.A.	0
KCNH1			0	N.A.	0
KCNH3		0,303038183	0	N.A.	0
KCNH4		-0,078184381	0	N.A.	0
KCNIP1			0	N.A.	0
KCNIP4-IT1			0	N.A.	0
KCNJ1			0	N.A.	0
KCNJ10			0	N.A.	0
KCNJ13		-0,083695745	0	N.A.	0
KCNJ15			0	N.A.	0
KCNJ18			0	N.A.	0
KCNK13			0	N.A.	0
KCNK2			0	N.A.	0
KCNK3		-0,060508802	1	none	0
KCNK4		0,054655736	0	N.A.	0
KCNMA1-AS1			0	N.A.	0
KCNMA1-AS3			0	N.A.	0
KCNQ2		-0,177297527	0	N.A.	0
KCNQ5			0	N.A.	0
KCNS1			0	N.A.	0
KCNS2			0	N.A.	0
KCNT1			0	N.A.	0
KCNT2			0	N.A.	0
KCTD19		-0,017987591	0	N.A.	0
KCTD4			0	N.A.	0

KCTD8			0	N.A.	0
KEL			0	N.A.	0
KERA			0	N.A.	0
KHDC1L		0,008318853	0	N.A.	0
KHDRBS2			0	N.A.	0
KIAA0408			0	N.A.	0
KIAA1024L			0	N.A.	0
KIAA1045			0	N.A.	0
KIAA1210			0	N.A.	0
KIAA1644			0	N.A.	0
KIAA1731NL		0,268866575	0	N.A.	0
KIAA2022			0	N.A.	0
KIF25-AS1			0	N.A.	0
KIF4B		0,275485585	0	N.A.	0
KIF5A		0,360802572	0	N.A.	0
KIF6			0	N.A.	0
KIR2DL1			0	N.A.	0
KIR2DL3			0	N.A.	0
KIR3DL1			0	N.A.	0
KIR3DL2			0	N.A.	0
KIR3DL3			0	N.A.	0
KIRREL2			0	N.A.	0
KLF14			0	N.A.	0
KLF17			0	N.A.	0
KLHDC8A		0,206122488	0	N.A.	0
KLHL1			0	N.A.	0
KLHL10			0	N.A.	0
KLHL14			0	N.A.	0
KLHL30		0,40511623	0	N.A.	0
KLHL4			0	N.A.	0
KLHL40			0	N.A.	0
KLK9			0	N.A.	0
KLRC3			0	N.A.	0

KLRC4			0	N.A.	0
KLRC4-KLRK1			0	N.A.	0
KLRF2			0	N.A.	0
KLRG2		-0,355803839	0	N.A.	0
KLRK1			0	N.A.	0
KNCN			0	N.A.	0
KRT1			0	N.A.	0
KRT2			0	N.A.	0
KRT222		-0,200298407	0	N.A.	0
KRT24			0	N.A.	0
KRT27			0	N.A.	0
KRT3			0	N.A.	0
KRT31			0	N.A.	0
KRT32			0	N.A.	0
KRT33B			0	N.A.	0
KRT39		-0,057605904	0	N.A.	0
KRT6C			0	N.A.	0
KRT72			0	N.A.	0
KRT73			0	N.A.	0
KRT73-AS1			0	N.A.	0
KRT74			0	N.A.	0
KRT79			0	N.A.	0
KRT84			0	N.A.	0
KRT85			0	N.A.	0
KRT9		-0,202411877	0	N.A.	0
KRTAP1-5			0	N.A.	0
KRTAP10-10			0	N.A.	0
KRTAP10-2		0,033784266	0	N.A.	0
KRTAP12-1			0	N.A.	0
KRTAP12-3			0	N.A.	0
KRTAP17-1			0	N.A.	0
KRTAP2-2			0	N.A.	0
KRTAP21-2			0	N.A.	0

KRTAP4-12			0	N.A.	0
KRTAP4-8			0	N.A.	0
KRTAP5-11			0	N.A.	0
KRTAP5-4			0	N.A.	0
KRTAP5-6			0	N.A.	0
KRTAP5-7			0	N.A.	0
KY		0,419462918	0	N.A.	0
L29074.3		-0,122608884	0	N.A.	0
L34079.4			0	N.A.	0
L3MBTL4-AS1			0	N.A.	0
LA16c-306E5.2		0,198561538	0	N.A.	0
LA16c-313D11.9			0	N.A.	0
LA16c-316G12.2			0	N.A.	0
LA16c-329F2.1			0	N.A.	0
LA16c-359F1.1			0	N.A.	0
LA16c-360A4.1			0	N.A.	0
LA16c-360H6.3			0	N.A.	0
LA16c-366D1.3			0	N.A.	0
LA16c-444G7.1			0	N.A.	0
LA16c-83F12.6			0	N.A.	0
LAMB4			0	N.A.	0
LAMC3			0	N.A.	0
LAMP5-AS1			0	N.A.	0
LANCL1-AS1			0	N.A.	0
LAX1			0	N.A.	0
LBX1			0	N.A.	0
LCE1B			0	N.A.	0
LCE1D		0,019465074	0	N.A.	0
LCMT1-AS2		0,15670665	0	N.A.	0
LCN1			0	N.A.	0
LCN10			0	N.A.	0
LCN6		0,144607419	0	N.A.	0
LCNL1			0	N.A.	0

LCT		-0,045242429	0	N.A.	0
LDHAL6A			0	N.A.	0
LDHAL6B			0	N.A.	0
LDLRAD4-AS1			0	N.A.	0
LDOC1		0,038696391	0	N.A.	0
LECT1			0	N.A.	0
LENEP			0	N.A.	0
LEP			0	N.A.	0
LGALS12			0	N.A.	0
LGALS14			0	N.A.	0
LGI1			0	N.A.	0
LGI2		0,313656259	0	N.A.	0
LHFPL1			0	N.A.	0
LHFPL5		-0,19365632	0	N.A.	0
LHX2			0	N.A.	0
LHX4			0	N.A.	0
LHX5		0,072490566	0	N.A.	0
LHX6			0	N.A.	0
LHX8			0	N.A.	0
LHX9			0	N.A.	0
LIFR-AS1			0	N.A.	0
LILRA1			0	N.A.	0
LILRA4			0	N.A.	0
LILRA6		0,089146849	0	N.A.	0
LILRB5		0,068107124	0	N.A.	0
LIM2			0	N.A.	0
LIMD1-AS1		-0,059431967	0	N.A.	0
LIME1		-0,181121351	0	N.A.	0
LIMS3			0	N.A.	0
LIMS3L			0	N.A.	0
LIN28A			0	N.A.	0
LINC-ROR		-0,225575871	0	N.A.	0
LINC00028			0	N.A.	0

LINC00032			0	N.A.	0
LINC00087			0	N.A.	0
LINC00092			0	N.A.	0
LINC00102			0	N.A.	0
LINC00111		0,200186235	0	N.A.	0
LINC00158			0	N.A.	0
LINC00159			0	N.A.	0
LINC00163			0	N.A.	0
LINC00165			0	N.A.	0
LINC00200			0	N.A.	0
LINC00211			0	N.A.	0
LINC00221			0	N.A.	0
LINC00222			0	N.A.	0
LINC00237			0	N.A.	0
LINC00264			0	N.A.	0
LINC00266-4P		0,010348121	0	N.A.	0
LINC00271		0,025817047	0	N.A.	0
LINC00278		0,130361925	0	N.A.	0
LINC00298			0	N.A.	0
LINC00303			0	N.A.	0
LINC00305			0	N.A.	0
LINC00310			0	N.A.	0
LINC00311			0	N.A.	0
LINC00313			0	N.A.	0
LINC00316		0,085710832	0	N.A.	0
LINC00319			0	N.A.	0
LINC00322			0	N.A.	0
LINC00327			0	N.A.	0
LINC00332			0	N.A.	0
LINC00345			0	N.A.	0
LINC00352		0,166949078	0	N.A.	0
LINC00355		0,070218576	0	N.A.	0
LINC00359		-0,007987439	0	N.A.	0

LINC00381		0,164924072	0	N.A.	0
LINC00384			0	N.A.	0
LINC00390		-0,016890837	0	N.A.	0
LINC00397			0	N.A.	0
LINC00398			0	N.A.	0
LINC00403			0	N.A.	0
LINC00421			0	N.A.	0
LINC00423			0	N.A.	0
LINC00426		-0,058646116	0	N.A.	0
LINC00427		0,180616052	0	N.A.	0
LINC00434		0,164924072	0	N.A.	0
LINC00443		0,256120872	0	N.A.	0
LINC00454			0	N.A.	0
LINC00458		-0,265103939	0	N.A.	0
LINC00461		-0,177926203	0	N.A.	0
LINC00462		-0,299782322	0	N.A.	0
LINC00463		0,082367129	0	N.A.	0
LINC00470			0	N.A.	0
LINC00472			0	N.A.	0
LINC00473			0	N.A.	0
LINC00484		-0,094053427	0	N.A.	0
LINC00487		0,309969438	0	N.A.	0
LINC00488			0	N.A.	0
LINC00491			0	N.A.	0
LINC00500			0	N.A.	0
LINC00506			0	N.A.	0
LINC00507			0	N.A.	0
LINC00517			0	N.A.	0
LINC00519			0	N.A.	0
LINC00521			0	N.A.	0
LINC00528		-0,027339268	0	N.A.	0
LINC00534			0	N.A.	0
LINC00536		0,081138471	0	N.A.	0

LINC00538		0,178566987	0	N.A.	0
LINC00544			0	N.A.	0
LINC00548			0	N.A.	0
LINC00551			0	N.A.	0
LINC00563			0	N.A.	0
LINC00565			0	N.A.	0
LINC00566		0,096295576	0	N.A.	0
LINC00572		-0,057571338	0	N.A.	0
LINC00578			0	N.A.	0
LINC00581			0	N.A.	0
LINC00582			0	N.A.	0
LINC00593			0	N.A.	0
LINC00598			0	N.A.	0
LINC00601			0	N.A.	0
LINC00602			0	N.A.	0
LINC00604			0	N.A.	0
LINC00605			0	N.A.	0
LINC00619		-0,050812401	0	N.A.	0
LINC00620			0	N.A.	0
LINC00621			0	N.A.	0
LINC00624		-0,053529758	0	N.A.	0
LINC00626			0	N.A.	0
LINC00632			0	N.A.	0
LINC00636			0	N.A.	0
LINC00637			0	N.A.	0
LINC00640			0	N.A.	0
LINC00643			0	N.A.	0
LINC00648			0	N.A.	0
LINC00652		-0,130701951	0	N.A.	0
LINC00656			0	N.A.	0
LINC00664			0	N.A.	0
LINC00670			0	N.A.	0
LINC00677			0	N.A.	0

LINC00678		0,190988487	0	N.A.	0
LINC00682		-0,025234084	0	N.A.	0
LINC00683		0,010504664	0	N.A.	0
LINC00686		0,014722007	0	N.A.	0
LINC00691		0,06615666	0	N.A.	0
LINC00693			0	N.A.	0
LINC00698		0,272093854	0	N.A.	0
LINC00706			0	N.A.	0
LINC00707			0	N.A.	0
LINC00839			0	N.A.	0
LINC00840			0	N.A.	0
LINC00844			0	N.A.	0
LINC00856			0	N.A.	0
LINC00866			0	N.A.	0
LINC00877			0	N.A.	0
LINC00883		-0,029487836	0	N.A.	0
LINC00890			0	N.A.	0
LINC00891			0	N.A.	0
LINC00895		0,327133367	0	N.A.	0
LINC00900			0	N.A.	0
LINC00906			0	N.A.	0
LINC00908			0	N.A.	0
LINC00922			0	N.A.	0
LINC00925			0	N.A.	0
LINC00927			0	N.A.	0
LINC00929			0	N.A.	0
LINC00930		-0,051126337	0	N.A.	0
LINC00935			0	N.A.	0
LINC00939		0,24281335	0	N.A.	0
LINC00940		-0,108803223	0	N.A.	0
LINC00961			0	N.A.	0
LINC00968			0	N.A.	0
LINC00970		0,09432797	0	N.A.	0

LINC00971			0	N.A.	0
LINC00974		-0,125188335	0	N.A.	0
LINC00976			0	N.A.	0
LINC00977			0	N.A.	0
LINC00989			0	N.A.	0
LINC01010			0	N.A.	0
LINC01013			0	N.A.	0
LINC01018			0	N.A.	0
LINC01030			0	N.A.	0
LINC01031			0	N.A.	0
LINC01032		0,016520309	0	N.A.	0
LINC01033			0	N.A.	0
LINC01034		0,218882564	0	N.A.	0
LINC01036			0	N.A.	0
LINC01037			0	N.A.	0
LINC01046			0	N.A.	0
LINC01048		0,024736465	0	N.A.	0
LINC01050		0,046009322	0	N.A.	0
LINC01055			0	N.A.	0
LINC01058			0	N.A.	0
LINC01072			0	N.A.	0
LINC01080		0,234225929	0	N.A.	0
LINC01082			0	N.A.	0
LINC01085			0	N.A.	0
LINC01093			0	N.A.	0
LINC01095			0	N.A.	0
LINC01096		0,034637901	0	N.A.	0
LINC01097			0	N.A.	0
LINC01098		0,113655057	0	N.A.	0
LINC01102		0,056783282	0	N.A.	0
LINC01104			0	N.A.	0
LINC01111			0	N.A.	0
LINC01114			0	N.A.	0

LINC01119			0	N.A.	0
LINC01120		-0,091076015	0	N.A.	0
LINC01122			0	N.A.	0
LINC01135			0	N.A.	0
LINC01139			0	N.A.	0
LINC01140		0,101741538	0	N.A.	0
LINC01142			0	N.A.	0
LINC01150			0	N.A.	0
LINC01154			0	N.A.	0
LINC01160		-0,045506036	0	N.A.	0
LINC01163			0	N.A.	0
LINC01166		0,011255996	0	N.A.	0
LINC01168			0	N.A.	0
LINC01180			0	N.A.	0
LINC01185			0	N.A.	0
LINC01197			0	N.A.	0
LINC01204			0	N.A.	0
LINC01205			0	N.A.	0
LINC01206			0	N.A.	0
LINC01208			0	N.A.	0
LINC01210		0,144443728	0	N.A.	0
LINC01219		0,138748622	0	N.A.	0
LINC01227		-0,213758722	0	N.A.	0
LINC01228		0,06615666	0	N.A.	0
LINC01229			0	N.A.	0
LINC01230			0	N.A.	0
LINC01233			0	N.A.	0
LINC01250			0	N.A.	0
LINC01251			0	N.A.	0
LINC01257			0	N.A.	0
LINC01262			0	N.A.	0
LINC01264			0	N.A.	0
LINC01265			0	N.A.	0

LINC01266			0	N.A.	0
LINC01267			0	N.A.	0
LINC01269		0,071552306	0	N.A.	0
LINC01271		-0,005094042	0	N.A.	0
LINC01276		0,056783282	0	N.A.	0
LINC01277		0,192379733	0	N.A.	0
LINC01281			0	N.A.	0
LINC01282			0	N.A.	0
LINC01289			0	N.A.	0
LINC01293			0	N.A.	0
LINC01297			0	N.A.	0
LINC01299			0	N.A.	0
LINC01300		0,129434834	0	N.A.	0
LINC01305			0	N.A.	0
LINC01314			0	N.A.	0
LINC01317			0	N.A.	0
LINC01323			0	N.A.	0
LINC01324			0	N.A.	0
LINC01330			0	N.A.	0
LINC01336			0	N.A.	0
LINC01337			0	N.A.	0
LINC01340		0,019117864	0	N.A.	0
LINC01342		0,096390144	0	N.A.	0
LINC01354			0	N.A.	0
LINC01361		-0,048429862	0	N.A.	0
LINC01362		0,057294996	0	N.A.	0
LINC01366			0	N.A.	0
LINC01374			0	N.A.	0
LINC01381			0	N.A.	0
LINC01388		0,15005184	0	N.A.	0
LINC01394			0	N.A.	0
LINC01395			0	N.A.	0
LINC01396			0	N.A.	0

LINC01399		-0,109835195	0	N.A.	0
LINC01402			0	N.A.	0
LINC01412			0	N.A.	0
LINC01413		-0,259397978	0	N.A.	0
LINC01415			0	N.A.	0
LINC01419		-0,043967284	0	N.A.	0
LINC01422			0	N.A.	0
LINC01423			0	N.A.	0
LINC01429			0	N.A.	0
LINC01435		0,062479862	0	N.A.	0
LINC01441		0,02712786	0	N.A.	0
LINC01446			0	N.A.	0
LINC01447			0	N.A.	0
LINC01452		-0,08885435	0	N.A.	0
LINC01470			0	N.A.	0
LINC01471			0	N.A.	0
LINC01474		0,172322709	0	N.A.	0
LINC01475			0	N.A.	0
LINC01481		-0,068540659	0	N.A.	0
LINC01482			0	N.A.	0
LINC01484			0	N.A.	0
LINC01489			0	N.A.	0
LINC01492			0	N.A.	0
LINC01497		0,018448765	0	N.A.	0
LINC01501			0	N.A.	0
LINC01502			0	N.A.	0
LINC01506			0	N.A.	0
LINC01507			0	N.A.	0
LINC01509			0	N.A.	0
LINC01513		-0,015820271	0	N.A.	0
LINC01516			0	N.A.	0
LINC01522			0	N.A.	0
LINC01527			0	N.A.	0

LINC01529			0	N.A.	0
LINC01531			0	N.A.	0
LINC01535		0,205487926	0	N.A.	0
LINC01537			0	N.A.	0
LINC01539			0	N.A.	0
LINC01543			0	N.A.	0
LINC01546			0	N.A.	0
LINC01548		-0,174508826	0	N.A.	0
LINC01561			0	N.A.	0
LINC01562			0	N.A.	0
LINC01563			0	N.A.	0
LINC01565		0,107473348	0	N.A.	0
LINC01567			0	N.A.	0
LINC01568			0	N.A.	0
LINC01571			0	N.A.	0
LINC01574		0,193940469	0	N.A.	0
LINC01583			0	N.A.	0
LINGO2			0	N.A.	0
LINGO4			0	N.A.	0
LIPN			0	N.A.	0
LIX1			0	N.A.	0
LL09NC01-139C3.1			0	N.A.	0
LL0XNC01-116E7.2		-0,242234701	0	N.A.	0
LL0XNC01-16G2.1			0	N.A.	0
LL21NC02-1C16.2			0	N.A.	0
LL22NC03-102D1.18		0,279454759	0	N.A.	0
LL22NC03-104C7.1			0	N.A.	0
LL22NC03-63E9.3			0	N.A.	0
LLNLF-187D8.1			0	N.A.	0
LLNLR-246C6.1			0	N.A.	0
LLNLR-249E10.1			0	N.A.	0
LLNLR-268E12.1		0,047523644	0	N.A.	0
LLNLR-284B4.1			0	N.A.	0

LLNLR-304G9.1			0	N.A.	0
LLNLR-307A6.1			0	N.A.	0
LLPH-AS1			0	N.A.	0
LMF1-AS1			0	N.A.	0
LMLN-AS1			0	N.A.	0
LMO3			0	N.A.	0
LMX1B		0,226239247	0	N.A.	0
LONRF2			0	N.A.	0
LOR			0	N.A.	0
LOXHD1			0	N.A.	0
LPA		0,3285535	0	N.A.	0
LPAR3		0,081138471	0	N.A.	0
LPAR4		0,005139866	0	N.A.	0
LPHN3			0	N.A.	0
LPP-AS1			0	N.A.	0
LPPR3		-0,067340679	0	N.A.	0
LPPR4			0	N.A.	0
LPPR5		0,021253186	0	N.A.	0
LRCH2			0	N.A.	0
LRCOL1		-0,297073071	0	N.A.	0
LRFN1			0	N.A.	0
LRFN2		-0,338527874	0	N.A.	0
LRFN5			0	N.A.	0
LRGUK		0,26594567	0	N.A.	0
LRP1B			0	N.A.	0
LRP2			0	N.A.	0
LRRC15			0	N.A.	0
LRRC18			0	N.A.	0
LRRC2-AS1			0	N.A.	0
LRRC38			0	N.A.	0
LRRC3B			0	N.A.	0
LRRC4B			0	N.A.	0
LRRC4C		-0,056663581	0	N.A.	0

LRRC52			0	N.A.	0
LRRC55			0	N.A.	0
LRRC63			0	N.A.	0
LRRC71			0	N.A.	0
LRRC9		0,237980208	0	N.A.	0
LRRD1		0,236848587	0	N.A.	0
LRRK2			0	N.A.	0
LRRN4		-0,240700833	0	N.A.	0
LRRN4CL		-0,017730457	0	N.A.	0
LRRTM2		-0,215514438	0	N.A.	0
LRRTM4			0	N.A.	0
LRTM2			0	N.A.	0
LTA			0	N.A.	0
LUC7L2		-0,070650632	0	N.A.	0
LUZP2			0	N.A.	0
LY6G6F			0	N.A.	0
LY86-AS1		-0,218431062	0	N.A.	0
LYPD2			0	N.A.	0
LYPLAL1-AS1		0,035909195	0	N.A.	0
LYVE1		0,06900012	0	N.A.	0
LYZL2			0	N.A.	0
LYZL4		0,172954168	0	N.A.	0
MAB21L1			0	N.A.	0
MAB21L2			0	N.A.	0
MACC1-AS1			0	N.A.	0
MAFA-AS1		0,091477809	0	N.A.	0
MAG		-0,080170565	0	N.A.	0
MAGEA10			0	N.A.	0
MAGEB10			0	N.A.	0
MAGED4			0	N.A.	0
MAGEE2			0	N.A.	0
MAGEL2		-0,133570526	0	N.A.	0
MAGI1-AS1		0,228744416	0	N.A.	0

MAL			0	N.A.	0
MAMDC2		-0,018957241	0	N.A.	0
MAP1LC3C			0	N.A.	0
MAP3K14-AS1		-0,013510018	0	N.A.	0
MAP3K15			0	N.A.	0
MAPK10		0,126265388	0	N.A.	0
MARCH10			0	N.A.	0
MARCH4			0	N.A.	0
MASP1		0,117760074	0	N.A.	0
MASP2		0,150728695	0	N.A.	0
MBD3L1			0	N.A.	0
MBL2			0	N.A.	0
MBOAT4			0	N.A.	0
MC4R			0	N.A.	0
MCEMP1		0,065498977	0	N.A.	0
MCF2			0	N.A.	0
MCHR1			0	N.A.	0
MCHR2			0	N.A.	0
MCIDAS		0,032213848	0	N.A.	0
MCM8-AS1			0	N.A.	0
MDC1-AS1			0	N.A.	0
MDGA2			0	N.A.	0
MED12L			0	N.A.	0
MED4-AS1		-0,388190246	0	N.A.	0
MEF2B			0	N.A.	0
MEF2C-AS1		-0,034769699	0	N.A.	0
MEG8			0	N.A.	0
MEG9			0	N.A.	0
MEGF10			0	N.A.	0
MEIKIN		0,132496596	0	N.A.	0
MEIS1-AS2		0,150941019	0	N.A.	0
MEOX1			0	N.A.	0
MEOX2		0,058015713	0	N.A.	0

MEPE			0	N.A.	0
MESP2		0,05643183	0	N.A.	0
METTL11B			0	N.A.	0
METTL24			0	N.A.	0
MGAM		0,093180811	0	N.A.	0
MGAT5B		0,002652887	0	N.A.	0
MICU3		0,09354134	0	N.A.	0
MIR100HG		-0,056663581	0	N.A.	0
MIR133A1HG			0	N.A.	0
MIR1539			0	N.A.	0
MIR202HG			0	N.A.	0
MIR3179-1			0	N.A.	0
MIR3180-1			0	N.A.	0
MIR3180-4		0,075183656	0	N.A.	0
MIR325HG			0	N.A.	0
MIR99AHG			0	N.A.	0
MKNK1-AS1			0	N.A.	0
MKRN3		-0,017334247	0	N.A.	0
MKX-AS1			0	N.A.	0
MLIP		-0,046146087	0	N.A.	0
MLNR			0	N.A.	0
MMP16			0	N.A.	0
MMP20		-0,187980581	0	N.A.	0
MMP21			0	N.A.	0
MMP27			0	N.A.	0
MMP8			0	N.A.	0
MOBP			0	N.A.	0
MORC1			0	N.A.	0
MOV10L1		-0,035638133	0	N.A.	0
MPL		-0,042036555	0	N.A.	0
MPP4		0,269992716	0	N.A.	0
MPPED1			0	N.A.	0
MRAP			0	N.A.	0

MRGPRF		-0,106566266	0	N.A.	0
MRGPRF-AS1			0	N.A.	0
MRGPRX2			0	N.A.	0
MRGPRX3			0	N.A.	0
MRO		0,243460549	0	N.A.	0
MROH9			0	N.A.	0
MS4A14			0	N.A.	0
MS4A15		0,06881033	0	N.A.	0
MS4A3			0	N.A.	0
MS4A4E			0	N.A.	0
MS4A5			0	N.A.	0
MS4A6E			0	N.A.	0
MSH5-SAPCD1			0	N.A.	0
MSRB3		-0,02340041	0	N.A.	0
MT1B		0,119661661	0	N.A.	0
MT4		-0,049692811	0	N.A.	0
MTMR7		-0,082307229	0	N.A.	0
MTNR1A			0	N.A.	0
MUM1L1			0	N.A.	0
MUSK			0	N.A.	0
MYBPC2			0	N.A.	0
MYBPH			0	N.A.	0
MYBPHL		0,246723535	0	N.A.	0
MYCT1		-0,19774898	0	N.A.	0
MYF6			0	N.A.	0
MYH6			0	N.A.	0
MYH7			0	N.A.	0
MYHAS			0	N.A.	0
MYL10			0	N.A.	0
MYL2			0	N.A.	0
MYL3		0,037114613	0	N.A.	0
MYL4		-0,141801331	0	N.A.	0
MYL7			0	N.A.	0

MYLK-AS2			0	N.A.	0
MYLK2			0	N.A.	0
MYO16		-0,109266923	0	N.A.	0
MYO16-AS1		0,124035059	0	N.A.	0
MYO1H			0	N.A.	0
MYO3A			0	N.A.	0
MYOC			0	N.A.	0
MYOCD			0	N.A.	0
MYOZ1			0	N.A.	0
MYOZ2			0	N.A.	0
MYOZ3			0	N.A.	0
NAALAD2			0	N.A.	0
NAALADL2-AS2			0	N.A.	0
NADK2-AS1			0	N.A.	0
NALCN			0	N.A.	0
NALCN-AS1			0	N.A.	0
NAMA		0,249183174	0	N.A.	0
NANOGNB			0	N.A.	0
NAP1L2			0	N.A.	0
NAP1L3			0	N.A.	0
NAP1L6			0	N.A.	0
NAT8L		0,077626177	0	N.A.	0
NAV2-AS2			0	N.A.	0
NAV3		-0,03392888	0	N.A.	0
NBAT1			0	N.A.	0
NBPF4			0	N.A.	0
NCAM2			0	N.A.	0
NCAN			0	N.A.	0
NCBP2-AS1			0	N.A.	0
NCBP2L			0	N.A.	0
NCR1			0	N.A.	0
NCR2			0	N.A.	0
NCRNA00250		0,162514099	0	N.A.	0

NDST3		-0,089246263	0	N.A.	0
NDUFC2-KCTD14			0	N.A.	0
NECAB1			0	N.A.	0
NECAB2			0	N.A.	0
NEDD8-MDP1		0,035909195	0	N.A.	0
NEFH		-0,234497338	0	N.A.	0
NEFM			0	N.A.	0
NELL1			0	N.A.	0
NETO1			0	N.A.	0
NEXN-AS1			0	N.A.	0
NFE4			0	N.A.	0
NFIA-AS2			0	N.A.	0
NGF			0	N.A.	0
NHS-AS1			0	N.A.	0
NIM1K		-0,056663581	0	N.A.	0
NKAIN2		-0,038637595	0	N.A.	0
NKAIN4			0	N.A.	0
NKAPL			0	N.A.	0
NKX2-1			0	N.A.	0
NKX2-1-AS1			0	N.A.	0
NKX2-3			0	N.A.	0
NKX3-2			0	N.A.	0
NKX6-2			0	N.A.	0
NKX6-3			0	N.A.	0
NLGN4X			0	N.A.	0
NLGN4Y		-0,087091051	0	N.A.	0
NLRC4			0	N.A.	0
NLRP12		-0,025417325	0	N.A.	0
NLRP14			0	N.A.	0
NLRP4			0	N.A.	0
NLRP7			0	N.A.	0
NLRP8			0	N.A.	0
NMBR			0	N.A.	0

NME2		-0,030121414	0	N.A.	0
NME8			0	N.A.	0
NME9		0,129800483	0	N.A.	0
NMRK2			0	N.A.	0
NMS			0	N.A.	0
NNAT		0,067065763	0	N.A.	0
NOG			0	N.A.	0
NOS1			0	N.A.	0
NOX3			0	N.A.	0
NOX5			0	N.A.	0
NPAS4			0	N.A.	0
NPFFR2			0	N.A.	0
NPHP3-AS1		-0,338316193	0	N.A.	0
NPIPA7			0	N.A.	0
NPIPA8			0	N.A.	0
NPIPB3		0,330183392	0	N.A.	0
NPIPB7			0	N.A.	0
NPIPB8		0,001544495	0	N.A.	0
NPIPB9			0	N.A.	0
NPPA		0,072729628	0	N.A.	0
NPR1			0	N.A.	0
NPY			0	N.A.	0
NPY4R			0	N.A.	0
NPY5R			0	N.A.	0
NR0B1		-0,025234084	0	N.A.	0
NR2E1			0	N.A.	0
NR5A1			0	N.A.	0
NRG1		0,055374917	0	N.A.	0
NRG3			0	N.A.	0
NRIP2		0,083283446	0	N.A.	0
NRK			0	N.A.	0
NRN1L			0	N.A.	0
NRSN1			0	N.A.	0

NTF4			0	N.A.	0
NTM		0,056882882	0	N.A.	0
NTNG1			0	N.A.	0
NTRK1			0	N.A.	0
NTRK3			0	N.A.	0
NUCB1-AS1			0	N.A.	0
NUDT10		-0,013567514	0	N.A.	0
NUGGC			0	N.A.	0
NUP210L			0	N.A.	0
NUTM2A		-0,072506321	0	N.A.	0
NUTM2B			0	N.A.	0
NUTM2D			0	N.A.	0
NUTM2E		0,008710349	0	N.A.	0
NUTM2G			0	N.A.	0
NXNL2			0	N.A.	0
NXPH1		0,153433482	0	N.A.	0
OCIAD1-AS1			0	N.A.	0
OCM		-0,03596857	0	N.A.	0
OCSTAMP			0	N.A.	0
ODF1			0	N.A.	0
OFCC1		-0,153389838	0	N.A.	0
OGFRP1		-0,375276435	0	N.A.	0
OLIG1			0	N.A.	0
OLIG2			0	N.A.	0
OMD		-0,221404202	0	N.A.	0
OMG			0	N.A.	0
OPA1-AS1			0	N.A.	0
OPCML			0	N.A.	0
OPN1SW			0	N.A.	0
OPN4			0	N.A.	0
OPN5			0	N.A.	0
OPRM1			0	N.A.	0
OR10A4		0,266713809	0	N.A.	0

OR10A5			0	N.A.	0
OR10P1			0	N.A.	0
OR13A1			0	N.A.	0
OR13C5			0	N.A.	0
OR1J1			0	N.A.	0
OR2A7			0	N.A.	0
OR2C3			0	N.A.	0
OR2D2			0	N.A.	0
OR2L13			0	N.A.	0
OR4C6			0	N.A.	0
OR51E2			0	N.A.	0
OR52K2			0	N.A.	0
OR52N4			0	N.A.	0
OR5M11			0	N.A.	0
OR7A5			0	N.A.	0
OR7D2			0	N.A.	0
OR8D4			0	N.A.	0
OR8G1			0	N.A.	0
OR8S1			0	N.A.	0
OSTN			0	N.A.	0
OSTN-AS1			0	N.A.	0
OTOF			0	N.A.	0
OTOG			0	N.A.	0
OTOP2			0	N.A.	0
OTOR			0	N.A.	0
OTOS			0	N.A.	0
OVCA2		-0,208540971	0	N.A.	0
OVCH1		0,019552565	0	N.A.	0
OVCH1-AS1			0	N.A.	0
P2RX1			0	N.A.	0
P2RX2			0	N.A.	0
P2RX3			0	N.A.	0
P2RY12			0	N.A.	0

P2RY13		0,21061856	0	N.A.	0
P2RY14		-0,080170565	0	N.A.	0
P2RY6		-0,244866579	0	N.A.	0
P3H2-AS1			0	N.A.	0
P4HA2-AS1			0	N.A.	0
PABPC4L			0	N.A.	0
PABPN1L			0	N.A.	0
PACERR			0	N.A.	0
PACSIN1			0	N.A.	0
PADI4		-0,049490728	0	N.A.	0
PADI6			0	N.A.	0
PAGE2			0	N.A.	0
PAGE2B			0	N.A.	0
PAGE3			0	N.A.	0
PAK7		-0,067340679	0	N.A.	0
PALM2-AKAP2			0	N.A.	0
PAMR1		0,112524993	0	N.A.	0
PANDAR			0	N.A.	0
PAPL		0,092101135	0	N.A.	0
PAPOLB		0,074764166	0	N.A.	0
PAPPA-AS1			0	N.A.	0
PAPPA-AS2		-0,04779246	0	N.A.	0
PAPPA2			0	N.A.	0
PAQR9-AS1			0	N.A.	0
PATE1		0,34389655	0	N.A.	0
PAX1			0	N.A.	0
PAX3			0	N.A.	0
PBOV1		0,011689245	0	N.A.	0
PCA3			0	N.A.	0
PCAT14			0	N.A.	0
PCAT18		0,119613478	0	N.A.	0
PCDH12		0,049988878	0	N.A.	0
PCDH18			0	N.A.	0

PCDH20			0	N.A.	0
PCDHA1			0	N.A.	0
PCDHA11			0	N.A.	0
PCDHA12			0	N.A.	0
PCDHA13			0	N.A.	0
PCDHA2			0	N.A.	0
PCDHA3			0	N.A.	0
PCDHA6			0	N.A.	0
PCDHA7			0	N.A.	0
PCDHA9		0,012735721	0	N.A.	0
PCDHAC1			0	N.A.	0
PCDHAC2		-0,103762047	0	N.A.	0
PCDHB1			0	N.A.	0
PCDHB3		-0,087091051	0	N.A.	0
PCDHB4		0,026570911	0	N.A.	0
PCDHB5			0	N.A.	0
PCDHB6			0	N.A.	0
PCDHB8			0	N.A.	0
PCDHGA12		0,111203572	0	N.A.	0
PCDHGA2		-0,023750482	0	N.A.	0
PCDHGA4		0,391339458	0	N.A.	0
PCDHGA9		0,059888376	0	N.A.	0
PCDHGB1		-0,020876396	0	N.A.	0
PCDHGB2		-0,127001163	0	N.A.	0
PCDHGB3		0,21817745	0	N.A.	0
PCDHGB7			0	N.A.	0
PCDHGC4			0	N.A.	0
PCDHGC5			0	N.A.	0
PCOLCE-AS1		-0,051365804	0	N.A.	0
PCSK2		0,322029999	0	N.A.	0
PCSK6-AS1			0	N.A.	0
PDC		0,255234424	0	N.A.	0
PDCD1LG2			0	N.A.	0

PDE1C			0	N.A.	0
PDE2A			0	N.A.	0
PDE6H			0	N.A.	0
PDE7B		0,069225607	0	N.A.	0
PDX1-AS1		-0,033632365	0	N.A.	0
PDZD4			0	N.A.	0
PDZD9			0	N.A.	0
PDZRN3-AS1		0,128899395	0	N.A.	0
PDZRN4			0	N.A.	0
PEG3			0	N.A.	0
PENK			0	N.A.	0
PEX5L-AS1			0	N.A.	0
PF4V1			0	N.A.	0
PFKFB1			0	N.A.	0
PFN3			0	N.A.	0
PGA3			0	N.A.	0
PGA5			0	N.A.	0
PGLYRP1		0,03116295	0	N.A.	0
PGLYRP2			0	N.A.	0
PGLYRP4		-0,087163387	0	N.A.	0
PGM5-AS1			0	N.A.	0
PGM5P3-AS1			0	N.A.	0
PGM5P4-AS1			0	N.A.	0
PGPEP1L			0	N.A.	0
PGR			0	N.A.	0
PHF21B			0	N.A.	0
PHKA2-AS1			0	N.A.	0
PHOSPHO1			0	N.A.	0
PIANP			0	N.A.	0
PIEZO2		-0,033730152	0	N.A.	0
PIH1D3			0	N.A.	0
PIK3C2G		0,154952171	0	N.A.	0
PIK3CD-AS1			0	N.A.	0

PIK3R5		-0,370437798	0	N.A.	0
PISRT1		0,001075161	0	N.A.	0
PITRM1-AS1		0,555664003	0	N.A.	0
PITX3			0	N.A.	0
PKD1L2		-0,14073666	0	N.A.	0
PKD1L3			0	N.A.	0
PKD2L1			0	N.A.	0
PKDREJ			0	N.A.	0
PKHD1		0,035909195	0	N.A.	0
PKHD1L1			0	N.A.	0
PKNOX2			0	N.A.	0
PLA2G4B			0	N.A.	0
PLA2G4E		0,087042122	0	N.A.	0
PLA2G4E-AS1			0	N.A.	0
PLA2G5			0	N.A.	0
PLAC1		-0,131997209	0	N.A.	0
PLCD4		0,17446872	0	N.A.	0
PLCE1-AS1			0	N.A.	0
PLCL2-AS1			0	N.A.	0
PLEKHG4B			0	N.A.	0
PLG		-0,2168217	0	N.A.	0
PLK5			0	N.A.	0
PLXNA4			0	N.A.	0
PM20D1			0	N.A.	0
PMP2			0	N.A.	0
PNLIPRP1			0	N.A.	0
PNLIPRP3			0	N.A.	0
PNMA3			0	N.A.	0
PNMA5			0	N.A.	0
PNMAL1			0	N.A.	0
PNMAL2		0,312425052	0	N.A.	0
PODN		0,054826697	0	N.A.	0
POMK		0,131702667	0	N.A.	0

POTEF		0,242678817	0	N.A.	0
POTEI		0,09102857	0	N.A.	0
POTEJ		0,275189364	0	N.A.	0
POTEM		0,126983831	0	N.A.	0
POU3F2			0	N.A.	0
POU3F3		-0,044032151	0	N.A.	0
POU6F2-AS2		0,025571874	0	N.A.	0
PP12613			0	N.A.	0
PP2D1		-0,033964715	0	N.A.	0
PPAN-P2RY11			0	N.A.	0
PPAPDC3			0	N.A.	0
PPEF1			0	N.A.	0
PPEF2			0	N.A.	0
PPFIA2			0	N.A.	0
PPIAL4B			0	N.A.	0
PPIAL4C		0,078294522	0	N.A.	0
PPIAL4E			0	N.A.	0
PPM1E			0	N.A.	0
PPP1R17			0	N.A.	0
PPP1R3C			0	N.A.	0
PPP2R2B			0	N.A.	0
PPY			0	N.A.	0
PRAM1			0	N.A.	0
PRB2		-0,01125164	0	N.A.	0
PRB3		0,005267169	0	N.A.	0
PRC1-AS1		-0,025280353	0	N.A.	0
PRCD			0	N.A.	0
PRDM14			0	N.A.	0
PREX2			0	N.A.	0
PRG2		-0,14073666	0	N.A.	0
PRG4			0	N.A.	0
PRH1-PRR4			0	N.A.	0
PRICKLE2-AS3			0	N.A.	0

PRIMA1			0	N.A.	0
PRKCA-AS1		0,13744339	0	N.A.	0
PRKG1-AS1			0	N.A.	0
PRL			0	N.A.	0
PRLHR			0	N.A.	0
PRND			0	N.A.	0
PROK1			0	N.A.	0
PROK2		-0,22700888	0	N.A.	0
PROKR1			0	N.A.	0
PROSER2-AS1			0	N.A.	0
PROX2			0	N.A.	0
PRPH2			0	N.A.	0
PRR23C			0	N.A.	0
PRR29			0	N.A.	0
PRR35		0,192749775	0	N.A.	0
PRRG3		-0,087746453	0	N.A.	0
PRSS38			0	N.A.	0
PRSS42		0,196547295	0	N.A.	0
PRSS48			0	N.A.	0
PRSS50			0	N.A.	0
PRSS51		0,069225508	0	N.A.	0
PRSS55			0	N.A.	0
PRTG			0	N.A.	0
PSD2		0,243777137	0	N.A.	0
PSG1			0	N.A.	0
PSG3			0	N.A.	0
PSG4			0	N.A.	0
PSG8			0	N.A.	0
PSMA8			0	N.A.	0
PTCHD1-AS			0	N.A.	0
PTCRA			0	N.A.	0
PTCSC3			0	N.A.	0
PTENP1-AS			0	N.A.	0

PTF1A		-0,024375969	0	N.A.	0
PTGER3		0,073126338	0	N.A.	0
PTGES3L-AARSD1			0	N.A.	0
PTGFR			0	N.A.	0
PTH1R			0	N.A.	0
PTH2R		-0,053698773	0	N.A.	0
PTOV1-AS2			0	N.A.	0
PTPN5			0	N.A.	0
PTPRCAP		-0,225163244	0	N.A.	0
PTPRQ			0	N.A.	0
PTPRZ1			0	N.A.	0
PURG			0	N.A.	0
PVALB			0	N.A.	0
PVRIG			0	N.A.	0
PWRN1			0	N.A.	0
PXT1			0	N.A.	0
PYDC1			0	N.A.	0
PYDC2		-0,083023222	0	N.A.	0
PYGO1			0	N.A.	0
PYY			0	N.A.	0
PZP			0	N.A.	0
QRFP			0	N.A.	0
QRFPR		0,029803283	0	N.A.	0
R3HDML		0,059290145	0	N.A.	0
RAB19		-0,382749673	0	N.A.	0
RAB40A		-0,090753943	0	N.A.	0
RAB4B-EGLN2			0	N.A.	0
RANBP3L			0	N.A.	0
RAPGEF4-AS1			0	N.A.	0
RASGEF1C			0	N.A.	0
RASGRF2-AS1		-0,068586	0	N.A.	0
RASGRP4			0	N.A.	0
RAX			0	N.A.	0

RBAKDN		-0,034147717	0	N.A.	0
RBFOX3		-0,000164059	0	N.A.	0
RBM20			0	N.A.	0
RBM46			0	N.A.	0
RBM5-AS1			0	N.A.	0
RBMS3-AS2			0	N.A.	0
RBMS3-AS3		-0,220132295	0	N.A.	0
RBMXL2			0	N.A.	0
RBP3		0,211220851	0	N.A.	0
RBP5		0,083283446	0	N.A.	0
RBPMS2			0	N.A.	0
RCSD1		-0,181822692	0	N.A.	0
RCVRN			0	N.A.	0
RDH8			0	N.A.	0
REC114			0	N.A.	0
RELN			0	N.A.	0
RERGL		0,161501152	0	N.A.	0
RFPL1S			0	N.A.	0
RFPL2			0	N.A.	0
RFTN2		0,024597141	0	N.A.	0
RFX4		-0,062707975	0	N.A.	0
RFX8			0	N.A.	0
RGAG1			0	N.A.	0
RGPD2		-0,015674807	0	N.A.	0
RGPD3			0	N.A.	0
RGPD4			0	N.A.	0
RGPD4-AS1		0,120332208	0	N.A.	0
RGPD6		-0,020463653	0	N.A.	0
RGS18			0	N.A.	0
RGS22		0,021957073	0	N.A.	0
RGS7BP			0	N.A.	0
RGSL1		-0,025234084	0	N.A.	0
RHD			0	N.A.	0

RHO			0	N.A.	0
RIMBP3C			0	N.A.	0
RIMS1			0	N.A.	0
RIMS4			0	N.A.	0
RIPPLY2		0,107473348	0	N.A.	0
RIT2			0	N.A.	0
RMDN2-AS1			0	N.A.	0
RNASE10			0	N.A.	0
RNASE2			0	N.A.	0
RNASE3			0	N.A.	0
RNF103-CHMP3		0,036189464	0	N.A.	0
RNF150			0	N.A.	0
RNF17			0	N.A.	0
RNF175			0	N.A.	0
RNF212B			0	N.A.	0
RNF219-AS1		0,151043365	0	N.A.	0
RNF224		0,090361061	0	N.A.	0
ROPN1			0	N.A.	0
ROPN1B		-0,133291339	0	N.A.	0
ROPN1L		-0,066373777	0	N.A.	0
RORB			0	N.A.	0
ROS1		0,057866813	0	N.A.	0
RP1		0,281968515	0	N.A.	0
RP1-101G11.3		0,329926764	0	N.A.	0
RP1-101K10.6			0	N.A.	0
RP1-102D24.5			0	N.A.	0
RP1-102G20.5			0	N.A.	0
RP1-102K2.6		0,030490663	0	N.A.	0
RP1-111B22.3			0	N.A.	0
RP1-111C20.3			0	N.A.	0
RP1-111D6.2			0	N.A.	0
RP1-111D6.3			0	N.A.	0
RP1-118J21.5		-0,149805262	0	N.A.	0

RP1-122K4.3		0	N.A.	0
RP1-122O8.7	-0,21966926	0	N.A.	0
RP1-124C6.1		0	N.A.	0
RP1-127D3.4		0	N.A.	0
RP1-127L4.7		0	N.A.	0
RP1-12G14.9	-0,079793303	0	N.A.	0
RP1-135L22.1		0	N.A.	0
RP1-136B1.1	-0,088807712	0	N.A.	0
RP1-137D17.1		0	N.A.	0
RP1-137D17.2	0,091423349	0	N.A.	0
RP1-137K2.2	0,037302611	0	N.A.	0
RP1-142L7.5		0	N.A.	0
RP1-142L7.9	0,27530734	0	N.A.	0
RP1-148H17.1		0	N.A.	0
RP1-149A16.3		0	N.A.	0
RP1-149L1.1		0	N.A.	0
RP1-151B14.6		0	N.A.	0
RP1-153P14.5		0	N.A.	0
RP1-153P14.8		0	N.A.	0
RP1-154K9.2		0	N.A.	0
RP1-155D22.2		0	N.A.	0
RP1-15D23.2		0	N.A.	0
RP1-163M9.7		0	N.A.	0
RP1-166H4.2		0	N.A.	0
RP1-167A14.2	-0,216713575	0	N.A.	0
RP1-17K7.2		0	N.A.	0
RP1-193H18.3		0	N.A.	0
RP1-197B17.4		0	N.A.	0
RP1-207H1.3		0	N.A.	0
RP1-212P9.3		0	N.A.	0
RP1-225E12.2		0	N.A.	0
RP1-232P20.1		0	N.A.	0
RP1-23E21.2		0	N.A.	0

RP1-241P17.1		0	N.A.	0
RP1-249F5.3		0	N.A.	0
RP1-253P7.4	-0,028490556	0	N.A.	0
RP1-256G22.2		0	N.A.	0
RP1-265C24.8		0	N.A.	0
RP1-267L14.3		0	N.A.	0
RP1-278C19.8	0,305838193	0	N.A.	0
RP1-27K12.4		0	N.A.	0
RP1-283K11.3		0	N.A.	0
RP1-286D6.5	-0,112237429	0	N.A.	0
RP1-28O10.1	-0,284561521	0	N.A.	0
RP1-290I10.7		0	N.A.	0
RP1-292B18.3		0	N.A.	0
RP1-29C18.10		0	N.A.	0
RP1-29C18.9		0	N.A.	0
RP1-309F20.4	-0,087716437	0	N.A.	0
RP1-30E17.2	0,341393322	0	N.A.	0
RP1-30M3.6		0	N.A.	0
RP1-310O13.7		0	N.A.	0
RP1-313L4.3		0	N.A.	0
RP1-34H18.1		0	N.A.	0
RP1-35C21.1		0	N.A.	0
RP1-37E16.12	0,256013796	0	N.A.	0
RP1-38C16.2		0	N.A.	0
RP1-41P2.7		0	N.A.	0
RP1-45C12.1		0	N.A.	0
RP1-45N11.1	0,147060278	0	N.A.	0
RP1-55C23.7		0	N.A.	0
RP1-56K13.3		0	N.A.	0
RP1-56K13.5		0	N.A.	0
RP1-59D14.10	0,255234424	0	N.A.	0
RP1-59D14.5	-0,025234084	0	N.A.	0
RP1-5O6.5	0,096817909	0	N.A.	0

RP1-60O19.2		0	N.A.	0
RP1-65P5.5		0	N.A.	0
RP1-69D17.3	-0,002393069	0	N.A.	0
RP1-69D17.4		0	N.A.	0
RP1-69M21.2	-0,257169163	0	N.A.	0
RP1-78O14.1	-0,005496725	0	N.A.	0
RP1-81P11.1		0	N.A.	0
RP1-90G24.11		0	N.A.	0
RP1-90J20.8		0	N.A.	0
RP1-90J4.1		0	N.A.	0
RP1-90K10.4		0	N.A.	0
RP1-90L14.1		0	N.A.	0
RP1-90L6.3		0	N.A.	0
RP1-91J24.3		0	N.A.	0
RP1-92O14.6	-0,073596332	0	N.A.	0
RP1-93H18.1	0,098657128	0	N.A.	0
RP1-97G4.1		0	N.A.	0
RP11-1006G14.2	0,142124569	0	N.A.	0
RP11-1008C21.1		0	N.A.	0
RP11-100L22.1		0	N.A.	0
RP11-100N20.1	0,09592698	0	N.A.	0
RP11-1012A1.4		0	N.A.	0
RP11-1018N14.5		0	N.A.	0
RP11-101C11.1		0	N.A.	0
RP11-101E5.1		0	N.A.	0
RP11-1026M7.2	0,010413208	0	N.A.	0
RP11-1029J19.4	0,254986171	0	N.A.	0
RP11-1029J19.5	0,187771762	0	N.A.	0
RP11-102G14.1	0,089814695	0	N.A.	0
RP11-102L12.2		0	N.A.	0
RP11-102N11.1		0	N.A.	0
RP11-1030E3.1		0	N.A.	0
RP11-1035H13.3		0	N.A.	0

RP11-1038A11.2	0,273065756	0	N.A.	0
RP11-1038A11.3		0	N.A.	0
RP11-103C16.2		0	N.A.	0
RP11-103H7.2		0	N.A.	0
RP11-103H7.5	-0,157360424	0	N.A.	0
RP11-104E19.1		0	N.A.	0
RP11-104H15.8		0	N.A.	0
RP11-104H15.9		0	N.A.	0
RP11-1055B8.3	0,113042075	0	N.A.	0
RP11-105N14.3		0	N.A.	0
RP11-1060G2.1	-0,040945974	0	N.A.	0
RP11-106M3.2		0	N.A.	0
RP11-106M3.3	-0,085459169	0	N.A.	0
RP11-106M7.1	0,001047933	0	N.A.	0
RP11-1070N10.3		0	N.A.	0
RP11-1072C15.4	0,02755381	0	N.A.	0
RP11-1079K10.3		0	N.A.	0
RP11-107F6.3		0	N.A.	0
RP11-1080G15.1		0	N.A.	0
RP11-1081L13.4	0,322029999	0	N.A.	0
RP11-1084A12.2		0	N.A.	0
RP11-1084E5.1	0,096481216	0	N.A.	0
RP11-108E14.1	0,057294996	0	N.A.	0
RP11-108K14.12		0	N.A.	0
RP11-108K14.8		0	N.A.	0
RP11-108L7.14	-0,121763428	0	N.A.	0
RP11-108L7.4	0,226462937	0	N.A.	0
RP11-108M9.3		0	N.A.	0
RP11-108O10.2		0	N.A.	0
RP11-108P20.4		0	N.A.	0
RP11-1090M7.1	0,178645822	0	N.A.	0
RP11-1094M14.12		0	N.A.	0
RP11-1096G20.5		0	N.A.	0

RP11-109A6.3	0,04178844	0	N.A.	0
RP11-109E24.1		0	N.A.	0
RP11-109E24.2		0	N.A.	0
RP11-109M19.1		0	N.A.	0
RP11-109P14.10		0	N.A.	0
RP11-109P14.9	0,014194687	0	N.A.	0
RP11-10A14.9	-0,111240054	0	N.A.	0
RP11-10C8.2		0	N.A.	0
RP11-10J21.4		0	N.A.	0
RP11-10J21.5		0	N.A.	0
RP11-10J5.1		0	N.A.	0
RP11-10K17.6		0	N.A.	0
RP11-10L7.1	0,014722007	0	N.A.	0
RP11-10N23.2		0	N.A.	0
RP11-10N23.5	0,017038737	0	N.A.	0
RP11-1102P16.1	-0,118437992	0	N.A.	0
RP11-1105G2.4	-0,120232798	0	N.A.	0
RP11-1109F11.3		0	N.A.	0
RP11-110I1.12	0,056783282	0	N.A.	0
RP11-111A22.1		0	N.A.	0
RP11-111E14.1		0	N.A.	0
RP11-111I12.1	-0,153881635	0	N.A.	0
RP11-111M22.5		0	N.A.	0
RP11-112L6.3	0,223442761	0	N.A.	0
RP11-1134I14.8		0	N.A.	0
RP11-1136G4.1	0,342649228	0	N.A.	0
RP11-1136J12.1	0,070361615	0	N.A.	0
RP11-113K21.4		0	N.A.	0
RP11-113O24.3		0	N.A.	0
RP11-1144P22.1		0	N.A.	0
RP11-1149M10.1		0	N.A.	0
RP11-1149M10.2		0	N.A.	0
RP11-114B7.6	-0,270373881	0	N.A.	0

RP11-114F10.2		0	N.A.	0
RP11-114G11.5	-0,242626229	0	N.A.	0
RP11-114G22.1		0	N.A.	0
RP11-114H23.1	0,138491791	0	N.A.	0
RP11-114H23.2	-0,055873899	0	N.A.	0
RP11-114H24.6		0	N.A.	0
RP11-114J13.1		0	N.A.	0
RP11-114M1.1	-0,038099692	0	N.A.	0
RP11-114M1.2		0	N.A.	0
RP11-114N19.3	-0,021143245	0	N.A.	0
RP11-114O18.1	0,322102716	0	N.A.	0
RP11-1151B14.3		0	N.A.	0
RP11-1152H15.1		0	N.A.	0
RP11-115D19.2	0,062658061	0	N.A.	0
RP11-115H18.1		0	N.A.	0
RP11-115J16.1		0	N.A.	0
RP11-115J16.2	0,2878873	0	N.A.	0
RP11-115J23.1	0,257463765	0	N.A.	0
RP11-115N4.1		0	N.A.	0
RP11-1167A19.2		0	N.A.	0
RP11-116D17.3	0,091636392	0	N.A.	0
RP11-116D17.4		0	N.A.	0
RP11-117D22.2		0	N.A.	0
RP11-117L5.4	0,260547928	0	N.A.	0
RP11-118B18.2		0	N.A.	0
RP11-118B22.4		0	N.A.	0
RP11-118F19.1		0	N.A.	0
RP11-1191J2.2		0	N.A.	0
RP11-1191J2.5		0	N.A.	0
RP11-119D18.1		0	N.A.	0
RP11-119J18.1	-0,235849003	0	N.A.	0
RP11-11C20.3		0	N.A.	0
RP11-11N7.5	0,053219496	0	N.A.	0

RP11-120C12.3		0	N.A.	0
RP11-120K9.2		0	N.A.	0
RP11-1212A22.4	0,113661337	0	N.A.	0
RP11-121A14.2		0	N.A.	0
RP11-121A8.1		0	N.A.	0
RP11-1228E12.1		0	N.A.	0
RP11-1228E12.2	0,056217302	0	N.A.	0
RP11-122K13.12	-0,083651207	0	N.A.	0
RP11-122L4.1		0	N.A.	0
RP11-122M14.1	-0,145824683	0	N.A.	0
RP11-123O22.1		0	N.A.	0
RP11-124N2.1	0,197796069	0	N.A.	0
RP11-1250I15.3		0	N.A.	0
RP11-125O18.1		0	N.A.	0
RP11-1260E13.1	-0,016227897	0	N.A.	0
RP11-1260E13.3		0	N.A.	0
RP11-126H7.4	-0,051717513	0	N.A.	0
RP11-126K1.8		0	N.A.	0
RP11-126K15.1		0	N.A.	0
RP11-126O1.2		0	N.A.	0
RP11-126O1.6		0	N.A.	0
RP11-127B16.1	-0,159945608	0	N.A.	0
RP11-127I20.5	0,241254325	0	N.A.	0
RP11-128A17.2	0,082131203	0	N.A.	0
RP11-129I19.2		0	N.A.	0
RP11-129J12.1		0	N.A.	0
RP11-129K12.1		0	N.A.	0
RP11-129M6.1		0	N.A.	0
RP11-12A2.3		0	N.A.	0
RP11-12D24.10		0	N.A.	0
RP11-12J10.4		0	N.A.	0
RP11-12M5.3		0	N.A.	0
RP11-12M5.4	0,034357583	0	N.A.	0

RP11-130C6.1	-0,231788321	0	N.A.	0
RP11-131K5.1		0	N.A.	0
RP11-131K5.2		0	N.A.	0
RP11-131L12.4	0,07146445	0	N.A.	0
RP11-132N15.2	0,111156675	0	N.A.	0
RP11-132N15.3		0	N.A.	0
RP11-1336O20.2	0,271802512	0	N.A.	0
RP11-133F8.2		0	N.A.	0
RP11-133K1.6	-0,081060161	0	N.A.	0
RP11-133L14.5		0	N.A.	0
RP11-134N1.2		0	N.A.	0
RP11-134O21.1	-0,176074872	0	N.A.	0
RP11-135A1.3		0	N.A.	0
RP11-135A24.4	0,285222157	0	N.A.	0
RP11-135D11.2		0	N.A.	0
RP11-136F16.1		0	N.A.	0
RP11-136H19.1		0	N.A.	0
RP11-1376P16.1		0	N.A.	0
RP11-138B4.1		0	N.A.	0
RP11-138H10.2		0	N.A.	0
RP11-138H8.2		0	N.A.	0
RP11-138I18.1		0	N.A.	0
RP11-13A1.1		0	N.A.	0
RP11-13A1.3		0	N.A.	0
RP11-13J8.1	-0,037106033	0	N.A.	0
RP11-13K12.2		0	N.A.	0
RP11-13P5.1		0	N.A.	0
RP11-13P5.2		0	N.A.	0
RP11-1406H17.1		0	N.A.	0
RP11-141M1.3	-0,001925526	0	N.A.	0
RP11-142A12.1		0	N.A.	0
RP11-142A23.1		0	N.A.	0
RP11-142C4.6		0	N.A.	0

RP11-142G1.3		0	N.A.	0
RP11-142M10.2		0	N.A.	0
RP11-143I21.1	0,08109109	0	N.A.	0
RP11-143J12.3		0	N.A.	0
RP11-143K11.7		0	N.A.	0
RP11-143M1.3		0	N.A.	0
RP11-144F15.1		0	N.A.	0
RP11-144G7.2		0	N.A.	0
RP11-145A3.1		0	N.A.	0
RP11-145E17.2		0	N.A.	0
RP11-145G20.1		0	N.A.	0
RP11-145M9.5		0	N.A.	0
RP11-145M9.6	-0,011449508	0	N.A.	0
RP11-146F11.5	0,045074285	0	N.A.	0
RP11-146I2.1		0	N.A.	0
RP11-146N23.4		0	N.A.	0
RP11-147L13.8		0	N.A.	0
RP11-148B6.1	-0,09039104	0	N.A.	0
RP11-148K1.10		0	N.A.	0
RP11-148K1.12	-0,189416607	0	N.A.	0
RP11-148L24.1		0	N.A.	0
RP11-149I23.3		0	N.A.	0
RP11-14C22.3		0	N.A.	0
RP11-14C22.6		0	N.A.	0
RP11-150D20.5		0	N.A.	0
RP11-150O12.1		0	N.A.	0
RP11-150O12.4		0	N.A.	0
RP11-152H18.3		0	N.A.	0
RP11-152P23.2	0,32078436	0	N.A.	0
RP11-153M7.5		0	N.A.	0
RP11-154B12.3		0	N.A.	0
RP11-154D17.1		0	N.A.	0
RP11-154H12.2		0	N.A.	0

RP11-154H12.3		0	N.A.	0
RP11-155L15.1		0	N.A.	0
RP11-155O18.6		0	N.A.	0
RP11-156K13.3		0	N.A.	0
RP11-157B13.7		0	N.A.	0
RP11-157D23.2		0	N.A.	0
RP11-157E16.1	0,064575337	0	N.A.	0
RP11-157E21.1		0	N.A.	0
RP11-157I4.4	-0,080264754	0	N.A.	0
RP11-157J24.2	-0,015387699	0	N.A.	0
RP11-158D2.2	0,025583232	0	N.A.	0
RP11-158I9.5		0	N.A.	0
RP11-158M2.2		0	N.A.	0
RP11-158M2.3	-0,077666804	0	N.A.	0
RP11-158M2.4		0	N.A.	0
RP11-159D12.10		0	N.A.	0
RP11-159F24.6	0,044112527	0	N.A.	0
RP11-159H22.2	0,254233288	0	N.A.	0
RP11-15A1.2		0	N.A.	0
RP11-15B17.1		0	N.A.	0
RP11-15B24.4		0	N.A.	0
RP11-15B24.5		0	N.A.	0
RP11-15E1.5		0	N.A.	0
RP11-15F12.3	0,018448765	0	N.A.	0
RP11-15I11.2		0	N.A.	0
RP11-15I20.1		0	N.A.	0
RP11-15L13.4	0,214937481	0	N.A.	0
RP11-160E2.11		0	N.A.	0
RP11-160E2.17		0	N.A.	0
RP11-160E2.19	-0,173304306	0	N.A.	0
RP11-160N1.10		0	N.A.	0
RP11-161D15.1		0	N.A.	0
RP11-161D15.2		0	N.A.	0

RP11-161H23.5	0,236858659	0	N.A.	0
RP11-161H23.9		0	N.A.	0
RP11-161I2.1	-0,03847024	0	N.A.	0
RP11-161I6.2	0,36768246	0	N.A.	0
RP11-164O23.8		0	N.A.	0
RP11-165F24.3	-0,189787438	0	N.A.	0
RP11-166A12.1		0	N.A.	0
RP11-166B2.5		0	N.A.	0
RP11-166B2.7		0	N.A.	0
RP11-166N17.1		0	N.A.	0
RP11-167B3.3		0	N.A.	0
RP11-167H9.4		0	N.A.	0
RP11-167J8.3	0,066328557	0	N.A.	0
RP11-167N4.2		0	N.A.	0
RP11-168K11.2		0	N.A.	0
RP11-168L22.2		0	N.A.	0
RP11-169D4.2		0	N.A.	0
RP11-169K16.4	0,008710349	0	N.A.	0
RP11-169K17.3		0	N.A.	0
RP11-169K17.4		0	N.A.	0
RP11-16B13.1		0	N.A.	0
RP11-16C1.1	0,072565575	0	N.A.	0
RP11-16C1.3		0	N.A.	0
RP11-16E23.5		0	N.A.	0
RP11-16L21.7		0	N.A.	0
RP11-16N2.1	0,142027372	0	N.A.	0
RP11-16P20.3		0	N.A.	0
RP11-170M17.1		0	N.A.	0
RP11-170N16.3	0,044789428	0	N.A.	0
RP11-171I2.1		0	N.A.	0
RP11-171I2.3		0	N.A.	0
RP11-171I2.4	0,070361615	0	N.A.	0
RP11-172E10.1		0	N.A.	0

RP11-172E9.2		0	N.A.	0
RP11-173A16.1		0	N.A.	0
RP11-173M1.4	-0,248262729	0	N.A.	0
RP11-173P15.3	-0,023656369	0	N.A.	0
RP11-174G6.1	0,005283039	0	N.A.	0
RP11-174J11.1		0	N.A.	0
RP11-175D17.3	-0,139420794	0	N.A.	0
RP11-175K6.1		0	N.A.	0
RP11-175P13.2		0	N.A.	0
RP11-177F15.1	0,055374917	0	N.A.	0
RP11-177G23.2	0,136220507	0	N.A.	0
RP11-178F10.2		0	N.A.	0
RP11-178L8.3		0	N.A.	0
RP11-178L8.9		0	N.A.	0
RP11-179A16.1		0	N.A.	0
RP11-179B15.6		0	N.A.	0
RP11-17A1.3	0,027887752	0	N.A.	0
RP11-17A4.2	-0,142826893	0	N.A.	0
RP11-180C16.1	-0,028103058	0	N.A.	0
RP11-180I4.2		0	N.A.	0
RP11-180M15.4		0	N.A.	0
RP11-180O5.2		0	N.A.	0
RP11-180P8.3		0	N.A.	0
RP11-181B11.2		0	N.A.	0
RP11-181G12.4		0	N.A.	0
RP11-181K12.2	0,462789983	0	N.A.	0
RP11-181K3.4	0,378514221	0	N.A.	0
RP11-182J1.1		0	N.A.	0
RP11-182J1.13		0	N.A.	0
RP11-182J1.17		0	N.A.	0
RP11-182M20.2		0	N.A.	0
RP11-182N22.8	0,125515091	0	N.A.	0
RP11-184A2.2	-0,092226719	0	N.A.	0

RP11-184A2.3		0	N.A.	0
RP11-184M15.1	0,348770047	0	N.A.	0
RP11-184M15.2	0,237871634	0	N.A.	0
RP11-185E12.2		0	N.A.	0
RP11-185E8.1	0,03281444	0	N.A.	0
RP11-187A9.3		0	N.A.	0
RP11-187O7.3		0	N.A.	0
RP11-188C12.3		0	N.A.	0
RP11-188D8.1	0,044323418	0	N.A.	0
RP11-18B16.2		0	N.A.	0
RP11-18I14.11	0,065282108	0	N.A.	0
RP11-190P13.2		0	N.A.	0
RP11-191N8.2		0	N.A.	0
RP11-192H23.5		0	N.A.	0
RP11-192H23.7	-0,007192592	0	N.A.	0
RP11-192P3.4		0	N.A.	0
RP11-193M21.1		0	N.A.	0
RP11-195M16.3		0	N.A.	0
RP11-196E1.3		0	N.A.	0
RP11-196H14.2		0	N.A.	0
RP11-197K3.1	0,096295576	0	N.A.	0
RP11-197N18.7		0	N.A.	0
RP11-19N8.4		0	N.A.	0
RP11-1B20.1		0	N.A.	0
RP11-1C8.6		0	N.A.	0
RP11-1E22.1		0	N.A.	0
RP11-1H15.2		0	N.A.	0
RP11-1M18.1		0	N.A.	0
RP11-200A1.1	0,042118838	0	N.A.	0
RP11-201E8.1		0	N.A.	0
RP11-202G11.2	0,41735398	0	N.A.	0
RP11-202G18.1		0	N.A.	0
RP11-203B7.2		0	N.A.	0

RP11-203E8.1		0	N.A.	0
RP11-203J24.8	0,177497689	0	N.A.	0
RP11-203M5.2		0	N.A.	0
RP11-203M5.7		0	N.A.	0
RP11-204L24.2	0,119636986	0	N.A.	0
RP11-205K6.1		0	N.A.	0
RP11-205M3.3		0	N.A.	0
RP11-206M11.7		0	N.A.	0
RP11-209D14.2		0	N.A.	0
RP11-209E8.1	0,236229665	0	N.A.	0
RP11-20B24.2		0	N.A.	0
RP11-20B24.4		0	N.A.	0
RP11-20B24.5		0	N.A.	0
RP11-20B24.6		0	N.A.	0
RP11-20B24.7		0	N.A.	0
RP11-20B7.1		0	N.A.	0
RP11-20G13.2		0	N.A.	0
RP11-20G6.2		0	N.A.	0
RP11-20I23.3		0	N.A.	0
RP11-20J15.3		0	N.A.	0
RP11-20J15.5		0	N.A.	0
RP11-210K20.2		0	N.A.	0
RP11-211G3.2		0	N.A.	0
RP11-211G3.3		0	N.A.	0
RP11-211N11.5		0	N.A.	0
RP11-212D19.4	0,226985603	0	N.A.	0
RP11-212I21.2		0	N.A.	0
RP11-212I21.5		0	N.A.	0
RP11-213G6.2		0	N.A.	0
RP11-213H15.4		0	N.A.	0
RP11-214O1.2		0	N.A.	0
RP11-214O1.3		0	N.A.	0
RP11-215P8.4		0	N.A.	0

RP11-216B9.9		0	N.A.	0
RP11-216L13.17		0	N.A.	0
RP11-216N14.9		0	N.A.	0
RP11-217B7.3		0	N.A.	0
RP11-218C14.5		0	N.A.	0
RP11-218C14.8	0,223328552	0	N.A.	0
RP11-218E20.3		0	N.A.	0
RP11-218I7.2	0,142027372	0	N.A.	0
RP11-218L14.4	0,004287949	0	N.A.	0
RP11-218M22.2		0	N.A.	0
RP11-219B4.3		0	N.A.	0
RP11-219E7.1		0	N.A.	0
RP11-219E7.3		0	N.A.	0
RP11-21A7A.4		0	N.A.	0
RP11-21C4.1		0	N.A.	0
RP11-21K12.3		0	N.A.	0
RP11-21L19.1		0	N.A.	0
RP11-221J22.1		0	N.A.	0
RP11-222K16.1		0	N.A.	0
RP11-222K16.2	-0,225987027	0	N.A.	0
RP11-223P11.3	-0,078534737	0	N.A.	0
RP11-225H22.4		0	N.A.	0
RP11-225N10.1		0	N.A.	0
RP11-227D13.1		0	N.A.	0
RP11-227D13.4	0,047932409	0	N.A.	0
RP11-227D13.5		0	N.A.	0
RP11-227G15.11	0,051650015	0	N.A.	0
RP11-227G15.9		0	N.A.	0
RP11-227H4.5		0	N.A.	0
RP11-228B15.4	0,03158775	0	N.A.	0
RP11-229E13.4		0	N.A.	0
RP11-229P13.15		0	N.A.	0
RP11-229P13.22	0,070250011	0	N.A.	0

RP11-22P4.2		0	N.A.	0
RP11-230B22.1		0	N.A.	0
RP11-230C9.2		0	N.A.	0
RP11-230C9.3	0,266497112	0	N.A.	0
RP11-231C14.4	-0,060494999	0	N.A.	0
RP11-231N3.1	0,272497937	0	N.A.	0
RP11-234G16.4		0	N.A.	0
RP11-234G16.5		0	N.A.	0
RP11-234K19.1		0	N.A.	0
RP11-234K24.3	0,285224053	0	N.A.	0
RP11-235C23.5		0	N.A.	0
RP11-235G24.1		0	N.A.	0
RP11-236B18.2		0	N.A.	0
RP11-236B18.5		0	N.A.	0
RP11-236L14.2		0	N.A.	0
RP11-238K6.1		0	N.A.	0
RP11-239E10.2		0	N.A.	0
RP11-239E10.3		0	N.A.	0
RP11-239H6.2		0	N.A.	0
RP11-239L20.6	0,131601708	0	N.A.	0
RP11-23E10.3		0	N.A.	0
RP11-23N2.4	0,211323737	0	N.A.	0
RP11-23P13.4		0	N.A.	0
RP11-240G22.4		0	N.A.	0
RP11-240M16.1		0	N.A.	0
RP11-242F24.1	0,097264344	0	N.A.	0
RP11-242G20.1	-0,170847167	0	N.A.	0
RP11-242J7.1	-0,059055527	0	N.A.	0
RP11-242P2.1		0	N.A.	0
RP11-243J16.7	0,042909635	0	N.A.	0
RP11-244F12.2	0,180184152	0	N.A.	0
RP11-244N9.4	0,160506141	0	N.A.	0
RP11-245P10.4	0,006801153	0	N.A.	0

RP11-245P10.6	0,211323737	0	N.A.	0
RP11-246A10.1		0	N.A.	0
RP11-246K15.1		0	N.A.	0
RP11-247C2.2	-0,201814824	0	N.A.	0
RP11-247I13.11		0	N.A.	0
RP11-247L20.3		0	N.A.	0
RP11-248G5.9		0	N.A.	0
RP11-248J23.7		0	N.A.	0
RP11-249C24.10		0	N.A.	0
RP11-249C24.12		0	N.A.	0
RP11-24C3.2		0	N.A.	0
RP11-24N18.1		0	N.A.	0
RP11-252C15.1		0	N.A.	0
RP11-252E2.1	-0,092369789	0	N.A.	0
RP11-253A20.1		0	N.A.	0
RP11-253D19.1		0	N.A.	0
RP11-253I19.3		0	N.A.	0
RP11-253M7.4	0,016508802	0	N.A.	0
RP11-254F7.3		0	N.A.	0
RP11-254I22.1	0,394520592	0	N.A.	0
RP11-254I22.3	0,138707048	0	N.A.	0
RP11-255C15.3	0,072923356	0	N.A.	0
RP11-255G12.3		0	N.A.	0
RP11-255H23.4		0	N.A.	0
RP11-255P5.3		0	N.A.	0
RP11-256I23.1		0	N.A.	0
RP11-256I23.2	0,011204681	0	N.A.	0
RP11-256I23.3		0	N.A.	0
RP11-256L11.3	-0,152118175	0	N.A.	0
RP11-256L6.2		0	N.A.	0
RP11-257O5.4	0,300528089	0	N.A.	0
RP11-259O2.1		0	N.A.	0
RP11-25I15.3		0	N.A.	0

RP11-25L3.3		0	N.A.	0
RP11-260E18.1		0	N.A.	0
RP11-261P24.2	-0,157360424	0	N.A.	0
RP11-263K19.6	-0,146560342	0	N.A.	0
RP11-263K4.3		0	N.A.	0
RP11-263K4.5		0	N.A.	0
RP11-264B14.1		0	N.A.	0
RP11-264B17.5		0	N.A.	0
RP11-264E18.1		0	N.A.	0
RP11-264E20.1		0	N.A.	0
RP11-264E20.2		0	N.A.	0
RP11-264I13.2		0	N.A.	0
RP11-264L1.4		0	N.A.	0
RP11-264M12.2		0	N.A.	0
RP11-265D17.2	0,062151335	0	N.A.	0
RP11-265D19.6		0	N.A.	0
RP11-265N6.2	-0,323634211	0	N.A.	0
RP11-265N7.2		0	N.A.	0
RP11-265P11.1	0,2878873	0	N.A.	0
RP11-266A24.1		0	N.A.	0
RP11-266K4.13		0	N.A.	0
RP11-266K4.14	0,000299764	0	N.A.	0
RP11-266N13.2		0	N.A.	0
RP11-267A15.3		0	N.A.	0
RP11-268F1.3		0	N.A.	0
RP11-268G12.3		0	N.A.	0
RP11-268P4.5	0,179732487	0	N.A.	0
RP11-269C23.5		0	N.A.	0
RP11-269F21.2		0	N.A.	0
RP11-269G24.4		0	N.A.	0
RP11-269M20.3		0	N.A.	0
RP11-26J3.4	-0,015440016	0	N.A.	0
RP11-26M5.3		0	N.A.	0

RP11-271C24.2	0,105420048	0	N.A.	0
RP11-271M24.2		0	N.A.	0
RP11-274H2.3	0,2878873	0	N.A.	0
RP11-274H24.1		0	N.A.	0
RP11-274J16.5		0	N.A.	0
RP11-275I4.1		0	N.A.	0
RP11-275I4.2		0	N.A.	0
RP11-276H1.3		0	N.A.	0
RP11-276H19.1		0	N.A.	0
RP11-276M12.1	0,026520097	0	N.A.	0
RP11-277P12.10		0	N.A.	0
RP11-277P12.6		0	N.A.	0
RP11-278A23.4		0	N.A.	0
RP11-278H7.1		0	N.A.	0
RP11-278I4.2	0,095121315	0	N.A.	0
RP11-281A20.2	-0,04457274	0	N.A.	0
RP11-281O15.4		0	N.A.	0
RP11-282A11.3	-0,084834724	0	N.A.	0
RP11-282I1.1		0	N.A.	0
RP11-282O18.3	0,375782278	0	N.A.	0
RP11-283C24.1	-0,01444344	0	N.A.	0
RP11-283G6.5		0	N.A.	0
RP11-283I3.2		0	N.A.	0
RP11-284F21.8		0	N.A.	0
RP11-284G10.1		0	N.A.	0
RP11-284H18.1		0	N.A.	0
RP11-284H19.1		0	N.A.	0
RP11-285E23.2	0,064051713	0	N.A.	0
RP11-285J16.1		0	N.A.	0
RP11-286B14.2		0	N.A.	0
RP11-286H15.1		0	N.A.	0
RP11-286N3.1		0	N.A.	0
RP11-286N3.2		0	N.A.	0

RP11-286O18.1		0	N.A.	0
RP11-287D1.3		0	N.A.	0
RP11-287D1.4		0	N.A.	0
RP11-287F9.2		0	N.A.	0
RP11-288I21.1		0	N.A.	0
RP11-288L9.4		0	N.A.	0
RP11-289H16.1		0	N.A.	0
RP11-28G8.1		0	N.A.	0
RP11-290C10.1	-0,029872433	0	N.A.	0
RP11-290H9.5		0	N.A.	0
RP11-290L1.5	0,05926896	0	N.A.	0
RP11-290M5.2		0	N.A.	0
RP11-290O12.2		0	N.A.	0
RP11-291B21.2		0	N.A.	0
RP11-292E2.1		0	N.A.	0
RP11-293M10.1		0	N.A.	0
RP11-293M10.2		0	N.A.	0
RP11-295I5.4		0	N.A.	0
RP11-295K2.3	0,047152119	0	N.A.	0
RP11-295M18.6		0	N.A.	0
RP11-296A18.6		0	N.A.	0
RP11-297B17.3		0	N.A.	0
RP11-297C4.3	0,015366641	0	N.A.	0
RP11-297H3.3		0	N.A.	0
RP11-297J22.1		0	N.A.	0
RP11-298E2.2		0	N.A.	0
RP11-298H24.1		0	N.A.	0
RP11-298J23.5		0	N.A.	0
RP11-298O21.2		0	N.A.	0
RP11-298O21.3		0	N.A.	0
RP11-299D14.2	0,256972614	0	N.A.	0
RP11-299L17.3		0	N.A.	0
RP11-29B2.6	0,092928954	0	N.A.	0

RP11-29P20.1		0	N.A.	0
RP11-2A4.4	0,047874308	0	N.A.	0
RP11-2E11.5	-0,111916395	0	N.A.	0
RP11-2H8.2	0,113879707	0	N.A.	0
RP11-2N1.2		0	N.A.	0
RP11-2N1.3		0	N.A.	0
RP11-300E4.2	0,342649228	0	N.A.	0
RP11-300J18.2		0	N.A.	0
RP11-301G19.1		0	N.A.	0
RP11-301L8.2		0	N.A.	0
RP11-302F12.3	-0,104526445	0	N.A.	0
RP11-302M6.5		0	N.A.	0
RP11-303E16.3	0,177567937	0	N.A.	0
RP11-303E16.6	-0,1885341	0	N.A.	0
RP11-304L19.2	0,181068383	0	N.A.	0
RP11-304L19.4		0	N.A.	0
RP11-304M2.5	0,155612844	0	N.A.	0
RP11-305D15.8		0	N.A.	0
RP11-305F18.1	-0,29087456	0	N.A.	0
RP11-305O4.3		0	N.A.	0
RP11-306O13.1	0,117704282	0	N.A.	0
RP11-307C18.1	0,185868719	0	N.A.	0
RP11-307C19.2		0	N.A.	0
RP11-307L3.4		0	N.A.	0
RP11-308B5.2	0,273895799	0	N.A.	0
RP11-308N19.1		0	N.A.	0
RP11-309N24.1		0	N.A.	0
RP11-30J20.1		0	N.A.	0
RP11-30K9.5		0	N.A.	0
RP11-310E22.4		0	N.A.	0
RP11-310H4.1		0	N.A.	0
RP11-310H4.3		0	N.A.	0
RP11-310N16.1	0,267086423	0	N.A.	0

RP11-310P5.1	0,223442761	0	N.A.	0
RP11-311F12.1		0	N.A.	0
RP11-313A24.1		0	N.A.	0
RP11-313F23.4	-0,051768428	0	N.A.	0
RP11-313P22.1	-0,002911468	0	N.A.	0
RP11-314B1.2	0,224410612	0	N.A.	0
RP11-315A16.1		0	N.A.	0
RP11-315D16.2	-0,276397522	0	N.A.	0
RP11-315D16.4	0,081092198	0	N.A.	0
RP11-315I20.3	0,253520133	0	N.A.	0
RP11-316M1.12	-0,092706521	0	N.A.	0
RP11-316M1.3		0	N.A.	0
RP11-316M20.1		0	N.A.	0
RP11-316M21.6		0	N.A.	0
RP11-317G6.1		0	N.A.	0
RP11-317J19.1		0	N.A.	0
RP11-317N8.3		0	N.A.	0
RP11-318A15.8		0	N.A.	0
RP11-318C2.1		0	N.A.	0
RP11-318G21.4	-0,059028607	0	N.A.	0
RP11-318K12.2	0,21872104	0	N.A.	0
RP11-319G6.3		0	N.A.	0
RP11-31L22.3		0	N.A.	0
RP11-320G10.1		0	N.A.	0
RP11-320G24.1		0	N.A.	0
RP11-320M16.2		0	N.A.	0
RP11-320N21.1	0,226082094	0	N.A.	0
RP11-320N21.2	0,213660211	0	N.A.	0
RP11-320P7.2		0	N.A.	0
RP11-321A17.4		0	N.A.	0
RP11-321E2.4		0	N.A.	0
RP11-321P16.1		0	N.A.	0
RP11-322D14.2		0	N.A.	0

RP11-322J23.1		0	N.A.	0
RP11-323C15.2		0	N.A.	0
RP11-323I15.5	-0,007987439	0	N.A.	0
RP11-323N12.5	0,326952629	0	N.A.	0
RP11-324E6.6		0	N.A.	0
RP11-324I22.2		0	N.A.	0
RP11-324L17.1	-0,111558622	0	N.A.	0
RP11-325L12.6		0	N.A.	0
RP11-325L12.7		0	N.A.	0
RP11-325N19.3		0	N.A.	0
RP11-326A19.4		0	N.A.	0
RP11-326C3.13		0	N.A.	0
RP11-326C3.15		0	N.A.	0
RP11-326I11.4	-0,149906808	0	N.A.	0
RP11-326N17.2		0	N.A.	0
RP11-327F22.1		0	N.A.	0
RP11-327F22.2	0,070361615	0	N.A.	0
RP11-327F22.4		0	N.A.	0
RP11-327I22.5		0	N.A.	0
RP11-327J17.9		0	N.A.	0
RP11-327L3.3		0	N.A.	0
RP11-328J14.1	0,093457013	0	N.A.	0
RP11-328N19.1	-0,002060108	0	N.A.	0
RP11-329B9.1		0	N.A.	0
RP11-329B9.3	-0,086888337	0	N.A.	0
RP11-329J18.3		0	N.A.	0
RP11-329N22.1		0	N.A.	0
RP11-32B5.8		0	N.A.	0
RP11-330A16.1		0	N.A.	0
RP11-331F9.4		0	N.A.	0
RP11-332H14.1	-0,09608963	0	N.A.	0
RP11-332H18.5		0	N.A.	0
RP11-332K15.1		0	N.A.	0

RP11-333A23.4	0,063724129	0	N.A.	0
RP11-333O1.1		0	N.A.	0
RP11-334A14.5		0	N.A.	0
RP11-334C17.6		0	N.A.	0
RP11-334E6.10		0	N.A.	0
RP11-334E6.3		0	N.A.	0
RP11-335K5.2		0	N.A.	0
RP11-335L23.5		0	N.A.	0
RP11-335O4.3	-0,050539792	0	N.A.	0
RP11-336A10.2		0	N.A.	0
RP11-336A10.4		0	N.A.	0
RP11-336K24.5	0,043355161	0	N.A.	0
RP11-337N6.1		0	N.A.	0
RP11-338C15.3	0,281308341	0	N.A.	0
RP11-338E21.3		0	N.A.	0
RP11-338K13.1		0	N.A.	0
RP11-338L22.2		0	N.A.	0
RP11-338N10.1		0	N.A.	0
RP11-33A14.1		0	N.A.	0
RP11-33B1.3		0	N.A.	0
RP11-33B1.4		0	N.A.	0
RP11-33N14.3	0,135297074	0	N.A.	0
RP11-33O4.2	0,064713371	0	N.A.	0
RP11-340E6.1		0	N.A.	0
RP11-340F14.6		0	N.A.	0
RP11-340I6.8		0	N.A.	0
RP11-342D11.2		0	N.A.	0
RP11-342D11.3		0	N.A.	0
RP11-342K6.3		0	N.A.	0
RP11-342M3.5		0	N.A.	0
RP11-343B18.2		0	N.A.	0
RP11-343H19.1		0	N.A.	0
RP11-343K8.3		0	N.A.	0

RP11-344A16.2		0	N.A.	0
RP11-344B5.2		0	N.A.	0
RP11-344B5.3		0	N.A.	0
RP11-344B5.4		0	N.A.	0
RP11-344F13.1		0	N.A.	0
RP11-344P13.4	0,061936771	0	N.A.	0
RP11-344P13.6		0	N.A.	0
RP11-345J4.3	0,342684972	0	N.A.	0
RP11-346C20.4		0	N.A.	0
RP11-346C4.3		0	N.A.	0
RP11-346D19.1	0,144093466	0	N.A.	0
RP11-347C12.3	0,18760206	0	N.A.	0
RP11-347C18.5	0,046070765	0	N.A.	0
RP11-348F1.2		0	N.A.	0
RP11-348F1.3		0	N.A.	0
RP11-348J12.2		0	N.A.	0
RP11-348N5.9	-0,11784272	0	N.A.	0
RP11-349A8.3		0	N.A.	0
RP11-34A14.3		0	N.A.	0
RP11-34C15.2		0	N.A.	0
RP11-34D15.2		0	N.A.	0
RP11-34F13.2		0	N.A.	0
RP11-350G8.9		0	N.A.	0
RP11-350N15.3		0	N.A.	0
RP11-350N15.4		0	N.A.	0
RP11-350N15.6		0	N.A.	0
RP11-351A11.1		0	N.A.	0
RP11-351I24.1	-0,00080375	0	N.A.	0
RP11-352D13.5		0	N.A.	0
RP11-352D13.6	-0,247219715	0	N.A.	0
RP11-352D3.2		0	N.A.	0
RP11-353N14.3	0,21760698	0	N.A.	0
RP11-353N14.7	-0,133570526	0	N.A.	0

RP11-353N4.5		0	N.A.	0
RP11-353N4.6	-0,081774836	0	N.A.	0
RP11-354A14.1		0	N.A.	0
RP11-354K1.1		0	N.A.	0
RP11-354K4.2		0	N.A.	0
RP11-354M1.2		0	N.A.	0
RP11-354M20.3		0	N.A.	0
RP11-354P11.2		0	N.A.	0
RP11-354P11.4		0	N.A.	0
RP11-354P11.8		0	N.A.	0
RP11-355F16.1	0,310729108	0	N.A.	0
RP11-355F22.1	0,062890705	0	N.A.	0
RP11-356I2.1		0	N.A.	0
RP11-356K23.1		0	N.A.	0
RP11-356K23.2		0	N.A.	0
RP11-356N1.2	-0,28915815	0	N.A.	0
RP11-356O9.1		0	N.A.	0
RP11-357N13.3		0	N.A.	0
RP11-358B23.5		0	N.A.	0
RP11-358H9.1		0	N.A.	0
RP11-359D14.2		0	N.A.	0
RP11-359G22.2		0	N.A.	0
RP11-359I18.5	0,062135647	0	N.A.	0
RP11-359J14.2		0	N.A.	0
RP11-359J6.1		0	N.A.	0
RP11-359P5.1	0,219262558	0	N.A.	0
RP11-35G9.5	0,042534969	0	N.A.	0
RP11-35J1.2	0,361765386	0	N.A.	0
RP11-360F5.3		0	N.A.	0
RP11-360I2.1		0	N.A.	0
RP11-360L9.8		0	N.A.	0
RP11-360O19.4		0	N.A.	0
RP11-361D15.2		0	N.A.	0

RP11-362A1.1		0	N.A.	0
RP11-362A9.3		0	N.A.	0
RP11-362J17.1		0	N.A.	0
RP11-362K14.5	0,154461644	0	N.A.	0
RP11-362K14.7		0	N.A.	0
RP11-362K2.2		0	N.A.	0
RP11-363E6.4		0	N.A.	0
RP11-363J20.2		0	N.A.	0
RP11-363N22.2	0,217112093	0	N.A.	0
RP11-365N19.2	-0,02882133	0	N.A.	0
RP11-365O16.6	0,222541272	0	N.A.	0
RP11-366H4.3	-0,000867714	0	N.A.	0
RP11-366L20.2	-0,222511887	0	N.A.	0
RP11-367F23.2		0	N.A.	0
RP11-367H1.1	0,398677535	0	N.A.	0
RP11-367J11.2	-0,119628571	0	N.A.	0
RP11-367J7.3		0	N.A.	0
RP11-369E15.3		0	N.A.	0
RP11-36B15.1		0	N.A.	0
RP11-370F5.4		0	N.A.	0
RP11-370I10.10		0	N.A.	0
RP11-370I10.11	0,121080697	0	N.A.	0
RP11-370I10.6	0,119017588	0	N.A.	0
RP11-371A19.2		0	N.A.	0
RP11-371E8.4		0	N.A.	0
RP11-372H2.1		0	N.A.	0
RP11-373N22.3	-0,184791168	0	N.A.	0
RP11-374A4.1	0,188289691	0	N.A.	0
RP11-374M1.5	-0,102629604	0	N.A.	0
RP11-375A5.1		0	N.A.	0
RP11-375H19.2		0	N.A.	0
RP11-375O18.2		0	N.A.	0
RP11-377G16.2		0	N.A.	0

RP11-378E13.4		0	N.A.	0
RP11-378I6.1		0	N.A.	0
RP11-379C10.1	0,03644643	0	N.A.	0
RP11-379C10.4	0,315932116	0	N.A.	0
RP11-379F12.3		0	N.A.	0
RP11-379F12.4		0	N.A.	0
RP11-379F4.6	0,044848866	0	N.A.	0
RP11-379K17.4		0	N.A.	0
RP11-379K22.3	0,262194424	0	N.A.	0
RP11-37N22.1	-0,019819473	0	N.A.	0
RP11-380B4.3	0,278477694	0	N.A.	0
RP11-380F14.2		0	N.A.	0
RP11-380I10.4		0	N.A.	0
RP11-380L11.4		0	N.A.	0
RP11-380P13.1	0,269706999	0	N.A.	0
RP11-381N20.1	0,020152359	0	N.A.	0
RP11-381N20.2		0	N.A.	0
RP11-381P6.1		0	N.A.	0
RP11-382A20.4		0	N.A.	0
RP11-382A20.5		0	N.A.	0
RP11-382A20.7		0	N.A.	0
RP11-383C6.2	0,122970203	0	N.A.	0
RP11-383I23.2		0	N.A.	0
RP11-384F7.1	-0,235068607	0	N.A.	0
RP11-384K6.8		0	N.A.	0
RP11-384P7.7		0	N.A.	0
RP11-386G11.8		0	N.A.	0
RP11-386M24.10		0	N.A.	0
RP11-386M24.9		0	N.A.	0
RP11-387A1.5	0,229168662	0	N.A.	0
RP11-387D10.4	-0,03847024	0	N.A.	0
RP11-387H17.4	-0,0008111	0	N.A.	0
RP11-388P9.2		0	N.A.	0

RP11-38G5.2		0	N.A.	0
RP11-38L15.3		0	N.A.	0
RP11-38L15.8		0	N.A.	0
RP11-390F4.10		0	N.A.	0
RP11-392O1.4		0	N.A.	0
RP11-392O17.1		0	N.A.	0
RP11-393I23.4		0	N.A.	0
RP11-393J16.4		0	N.A.	0
RP11-394I13.1		0	N.A.	0
RP11-394I13.3		0	N.A.	0
RP11-394J1.2	-0,099938085	0	N.A.	0
RP11-394O2.3		0	N.A.	0
RP11-394O4.3		0	N.A.	0
RP11-394O4.6		0	N.A.	0
RP11-395E19.6		0	N.A.	0
RP11-395L14.3		0	N.A.	0
RP11-397A16.1		0	N.A.	0
RP11-397O8.7		0	N.A.	0
RP11-398A8.5		0	N.A.	0
RP11-398E10.1		0	N.A.	0
RP11-398J10.2		0	N.A.	0
RP11-399E6.1	0,043898844	0	N.A.	0
RP11-399K21.14		0	N.A.	0
RP11-39M21.1		0	N.A.	0
RP11-3B7.1		0	N.A.	0
RP11-3D4.4	0,21760698	0	N.A.	0
RP11-3L21.2		0	N.A.	0
RP11-400K9.4		0	N.A.	0
RP11-400L8.2	-0,082307229	0	N.A.	0
RP11-400N13.1	0,042972404	0	N.A.	0
RP11-401O9.3	-0,07273132	0	N.A.	0
RP11-401P9.1		0	N.A.	0
RP11-401P9.6	0,018904876	0	N.A.	0

RP11-402P6.11		0	N.A.	0
RP11-403A21.1		0	N.A.	0
RP11-403A3.2		0	N.A.	0
RP11-403A3.3		0	N.A.	0
RP11-403B2.7		0	N.A.	0
RP11-403I13.7	-0,054258389	0	N.A.	0
RP11-404E16.1		0	N.A.	0
RP11-404O13.1		0	N.A.	0
RP11-404P21.6		0	N.A.	0
RP11-405M12.3		0	N.A.	0
RP11-405M12.4		0	N.A.	0
RP11-406H21.2	0,006599937	0	N.A.	0
RP11-406O23.2		0	N.A.	0
RP11-407G23.1	0,001047933	0	N.A.	0
RP11-407G23.7	0,365523305	0	N.A.	0
RP11-407H12.8		0	N.A.	0
RP11-407N17.3		0	N.A.	0
RP11-408B11.2	0,020829773	0	N.A.	0
RP11-408N14.1		0	N.A.	0
RP11-408O19.5		0	N.A.	0
RP11-409I10.2		0	N.A.	0
RP11-409K20.6		0	N.A.	0
RP11-40E6.2	-0,047984723	0	N.A.	0
RP11-410K21.2		0	N.A.	0
RP11-411H5.1	0,143010787	0	N.A.	0
RP11-412B14.1		0	N.A.	0
RP11-412P11.1		0	N.A.	0
RP11-413P11.1		0	N.A.	0
RP11-415D17.3		0	N.A.	0
RP11-415D17.4		0	N.A.	0
RP11-415F23.4		0	N.A.	0
RP11-415I12.3	0,119636986	0	N.A.	0
RP11-415J8.3	-0,111020608	0	N.A.	0

RP11-415J8.5	-0,012605976	0	N.A.	0
RP11-417E7.2	0,105420048	0	N.A.	0
RP11-417F21.1		0	N.A.	0
RP11-417L19.4	-0,065845984	0	N.A.	0
RP11-418H16.1		0	N.A.	0
RP11-419I17.1	0,153907623	0	N.A.	0
RP11-420K14.8		0	N.A.	0
RP11-421F16.3	-0,023311432	0	N.A.	0
RP11-422N16.3		0	N.A.	0
RP11-423H2.4		0	N.A.	0
RP11-424G14.1		0	N.A.	0
RP11-424I19.2	0,255630337	0	N.A.	0
RP11-425A23.1		0	N.A.	0
RP11-425D17.1		0	N.A.	0
RP11-426A6.5		0	N.A.	0
RP11-426C22.4	0,088023239	0	N.A.	0
RP11-426C22.6		0	N.A.	0
RP11-426C22.8		0	N.A.	0
RP11-426L16.10		0	N.A.	0
RP11-427J23.1		0	N.A.	0
RP11-428C19.4		0	N.A.	0
RP11-428C19.5		0	N.A.	0
RP11-428G5.5	-0,238373313	0	N.A.	0
RP11-428J1.4		0	N.A.	0
RP11-429E11.2		0	N.A.	0
RP11-429E11.3		0	N.A.	0
RP11-429J17.4		0	N.A.	0
RP11-429J17.7	-0,255001554	0	N.A.	0
RP11-429J17.8	0,108407135	0	N.A.	0
RP11-429P3.3		0	N.A.	0
RP11-430C1.1	0,175441588	0	N.A.	0
RP11-430C7.4		0	N.A.	0
RP11-431K24.3	0,394520592	0	N.A.	0

RP11-431K24.4		0	N.A.	0
RP11-431M7.3		0	N.A.	0
RP11-432I5.8	-0,166730128	0	N.A.	0
RP11-432J24.3		0	N.A.	0
RP11-432J9.6		0	N.A.	0
RP11-433A10.3		0	N.A.	0
RP11-433J20.1		0	N.A.	0
RP11-433M22.2		0	N.A.	0
RP11-434D11.4	-0,086161026	0	N.A.	0
RP11-434H14.1		0	N.A.	0
RP11-434I12.2	0,000653148	0	N.A.	0
RP11-434P11.2		0	N.A.	0
RP11-435D7.3		0	N.A.	0
RP11-435O5.7	-0,131570781	0	N.A.	0
RP11-436D23.1		0	N.A.	0
RP11-436I24.1		0	N.A.	0
RP11-437J19.1		0	N.A.	0
RP11-438F14.3		0	N.A.	0
RP11-439A17.9		0	N.A.	0
RP11-439C15.4	0,001656132	0	N.A.	0
RP11-439H8.4	0,194152528	0	N.A.	0
RP11-439K3.3	0,083669845	0	N.A.	0
RP11-439L18.1		0	N.A.	0
RP11-439L18.2		0	N.A.	0
RP11-43D2.2		0	N.A.	0
RP11-43F13.3	0,146691391	0	N.A.	0
RP11-43N5.1	0,082367129	0	N.A.	0
RP11-440G9.1		0	N.A.	0
RP11-440I14.2		0	N.A.	0
RP11-440L14.3		0	N.A.	0
RP11-441F2.5	0,333407989	0	N.A.	0
RP11-442J21.2		0	N.A.	0
RP11-442O1.3		0	N.A.	0

RP11-442O1.4	0,01704278	0	N.A.	0
RP11-443C10.1		0	N.A.	0
RP11-444D3.1		0	N.A.	0
RP11-444E17.6		0	N.A.	0
RP11-445F12.1		0	N.A.	0
RP11-445F6.2	-0,225163244	0	N.A.	0
RP11-445K13.2		0	N.A.	0
RP11-445L6.3		0	N.A.	0
RP11-445N18.5	0,071290923	0	N.A.	0
RP11-445N20.3	0,117760074	0	N.A.	0
RP11-445O16.3		0	N.A.	0
RP11-445P17.8		0	N.A.	0
RP11-445P19.3		0	N.A.	0
RP11-446E24.4		0	N.A.	0
RP11-446F17.3		0	N.A.	0
RP11-446H18.5		0	N.A.	0
RP11-446H18.6		0	N.A.	0
RP11-446J8.1		0	N.A.	0
RP11-448G15.1		0	N.A.	0
RP11-449J1.1		0	N.A.	0
RP11-44B19.1		0	N.A.	0
RP11-44F21.3	0,064431072	0	N.A.	0
RP11-44K6.3		0	N.A.	0
RP11-44K6.4		0	N.A.	0
RP11-44N12.2	-0,09039104	0	N.A.	0
RP11-44N12.5		0	N.A.	0
RP11-452C13.1	0,03408571	0	N.A.	0
RP11-452C8.1		0	N.A.	0
RP11-452H21.1		0	N.A.	0
RP11-452I5.2	-0,087482772	0	N.A.	0
RP11-452K12.7	0,419325582	0	N.A.	0
RP11-452L6.8		0	N.A.	0
RP11-454K7.3		0	N.A.	0

RP11-455F5.6	0,012206386	0	N.A.	0
RP11-455J20.3	0,223414127	0	N.A.	0
RP11-455O6.8		0	N.A.	0
RP11-455P21.3	-0,344885361	0	N.A.	0
RP11-456D7.1		0	N.A.	0
RP11-456K23.1		0	N.A.	0
RP11-456O19.2		0	N.A.	0
RP11-457I16.2		0	N.A.	0
RP11-457M11.7		0	N.A.	0
RP11-459F6.3		0	N.A.	0
RP11-459I19.1	-0,006775496	0	N.A.	0
RP11-45A12.1		0	N.A.	0
RP11-45A17.4		0	N.A.	0
RP11-45L9.1		0	N.A.	0
RP11-45M22.3		0	N.A.	0
RP11-460I13.2		0	N.A.	0
RP11-461A8.5	0,191296245	0	N.A.	0
RP11-461L13.3	0,089323976	0	N.A.	0
RP11-461M2.2		0	N.A.	0
RP11-461O7.1		0	N.A.	0
RP11-462B18.2		0	N.A.	0
RP11-462P6.1		0	N.A.	0
RP11-463D19.1		0	N.A.	0
RP11-463J10.3		0	N.A.	0
RP11-463J10.4	0,140134487	0	N.A.	0
RP11-463O12.3	-0,091218544	0	N.A.	0
RP11-463O9.9		0	N.A.	0
RP11-464C19.3		0	N.A.	0
RP11-464F9.22	0,156819904	0	N.A.	0
RP11-465L10.10		0	N.A.	0
RP11-466A19.1	0,067312438	0	N.A.	0
RP11-466F5.10	-0,035438338	0	N.A.	0
RP11-466F5.8	-0,007271558	0	N.A.	0

RP11-466L17.1		0	N.A.	0
RP11-466P24.6	-0,107483555	0	N.A.	0
RP11-467I17.1		0	N.A.	0
RP11-467J12.4	-0,010486578	0	N.A.	0
RP11-467L19.16		0	N.A.	0
RP11-468E2.2		0	N.A.	0
RP11-469A15.2	-0,059431967	0	N.A.	0
RP11-469N6.1		0	N.A.	0
RP11-46F15.2	-0,060783053	0	N.A.	0
RP11-470M17.2		0	N.A.	0
RP11-472G21.2		0	N.A.	0
RP11-472N13.3		0	N.A.	0
RP11-472N19.3		0	N.A.	0
RP11-473C18.3	0,253107376	0	N.A.	0
RP11-473E2.3		0	N.A.	0
RP11-473E2.4		0	N.A.	0
RP11-473O4.3	-0,106226008	0	N.A.	0
RP11-473O4.4	-0,082307229	0	N.A.	0
RP11-474B12.1		0	N.A.	0
RP11-474C8.8		0	N.A.	0
RP11-474D1.4	-0,050932012	0	N.A.	0
RP11-474I16.8	0,115491748	0	N.A.	0
RP11-474N24.6	0,237871634	0	N.A.	0
RP11-474P2.4		0	N.A.	0
RP11-475B2.1		0	N.A.	0
RP11-476B1.1		0	N.A.	0
RP11-478K15.6		0	N.A.	0
RP11-479J7.2		0	N.A.	0
RP11-47I22.1		0	N.A.	0
RP11-47J17.3	0,182392106	0	N.A.	0
RP11-47P18.1		0	N.A.	0
RP11-47P18.2	-0,054183333	0	N.A.	0
RP11-480D4.6		0	N.A.	0

RP11-481G8.2		0	N.A.	0
RP11-481J13.1	-0,067456333	0	N.A.	0
RP11-482D24.3		0	N.A.	0
RP11-482M8.1		0	N.A.	0
RP11-483P21.2		0	N.A.	0
RP11-484D2.2	0,118354217	0	N.A.	0
RP11-484L8.1		0	N.A.	0
RP11-484N16.1	0,097793156	0	N.A.	0
RP11-484O2.1		0	N.A.	0
RP11-485G7.6		0	N.A.	0
RP11-486F17.1	0,215322647	0	N.A.	0
RP11-486L19.2		0	N.A.	0
RP11-486M23.1		0	N.A.	0
RP11-486M23.2		0	N.A.	0
RP11-488C13.6		0	N.A.	0
RP11-489O18.1		0	N.A.	0
RP11-48G14.1		0	N.A.	0
RP11-48G14.3	0,249967027	0	N.A.	0
RP11-490E15.2		0	N.A.	0
RP11-492I21.1	0,235248939	0	N.A.	0
RP11-493E12.1		0	N.A.	0
RP11-493L12.5		0	N.A.	0
RP11-493L12.7		0	N.A.	0
RP11-493P1.2		0	N.A.	0
RP11-494O16.4		0	N.A.	0
RP11-495K9.3		0	N.A.	0
RP11-495O10.1		0	N.A.	0
RP11-495P10.3	0,252066648	0	N.A.	0
RP11-495P10.6		0	N.A.	0
RP11-497E19.1		0	N.A.	0
RP11-497E19.2		0	N.A.	0
RP11-497H17.1		0	N.A.	0
RP11-497K21.1		0	N.A.	0

RP11-498C9.16	-0,239476269	0	N.A.	0
RP11-498M15.1	0,325137988	0	N.A.	0
RP11-499O7.7	0,026295128	0	N.A.	0
RP11-49I4.3		0	N.A.	0
RP11-49K24.8		0	N.A.	0
RP11-4B16.3	0,051967137	0	N.A.	0
RP11-4C20.3		0	N.A.	0
RP11-4K16.2		0	N.A.	0
RP11-4L24.3		0	N.A.	0
RP11-4M23.3		0	N.A.	0
RP11-500G10.5		0	N.A.	0
RP11-500G22.5	0,22741539	0	N.A.	0
RP11-500M8.7	0,258208655	0	N.A.	0
RP11-501J20.5		0	N.A.	0
RP11-503C24.2		0	N.A.	0
RP11-503C24.4	-0,03847024	0	N.A.	0
RP11-503L19.1		0	N.A.	0
RP11-503P10.1	-0,072942913	0	N.A.	0
RP11-504I13.3		0	N.A.	0
RP11-504P24.9	0,35147641	0	N.A.	0
RP11-505E24.2		0	N.A.	0
RP11-506H21.5	0,239362366	0	N.A.	0
RP11-506M12.1	0,177497689	0	N.A.	0
RP11-507B12.2		0	N.A.	0
RP11-507K2.2	0,070218576	0	N.A.	0
RP11-507K2.3	-0,017118869	0	N.A.	0
RP11-508N12.2	-0,047107715	0	N.A.	0
RP11-508N22.12	0,091697578	0	N.A.	0
RP11-508N22.13	0,014959771	0	N.A.	0
RP11-508N22.9		0	N.A.	0
RP11-50B3.4	0,140822044	0	N.A.	0
RP11-50D16.4		0	N.A.	0
RP11-50D9.3	-0,054793905	0	N.A.	0

RP11-510M2.2		0	N.A.	0
RP11-510M2.6	-0,211213254	0	N.A.	0
RP11-513D5.2	-0,112269064	0	N.A.	0
RP11-513D5.5		0	N.A.	0
RP11-513G11.4	0,034357583	0	N.A.	0
RP11-513G19.1		0	N.A.	0
RP11-513H8.1		0	N.A.	0
RP11-513M1.1		0	N.A.	0
RP11-513N24.1		0	N.A.	0
RP11-513O17.2	0,189648562	0	N.A.	0
RP11-514D23.3		0	N.A.	0
RP11-515O17.3		0	N.A.	0
RP11-516C1.1	0,146193425	0	N.A.	0
RP11-517A5.5		0	N.A.	0
RP11-517A5.7		0	N.A.	0
RP11-517C16.2		0	N.A.	0
RP11-517C16.4		0	N.A.	0
RP11-517I3.1	-0,021734083	0	N.A.	0
RP11-517O13.3		0	N.A.	0
RP11-51J9.4		0	N.A.	0
RP11-520A21.1	0,082359308	0	N.A.	0
RP11-521B24.5		0	N.A.	0
RP11-521C20.5	0,184091022	0	N.A.	0
RP11-521D12.1		0	N.A.	0
RP11-521D12.5		0	N.A.	0
RP11-521O16.1		0	N.A.	0
RP11-521O16.2		0	N.A.	0
RP11-523H20.3	0,233204855	0	N.A.	0
RP11-523H24.3	0,153076372	0	N.A.	0
RP11-524D16--A.3	0,399090323	0	N.A.	0
RP11-524N5.1		0	N.A.	0
RP11-525G13.2	-0,103598713	0	N.A.	0
RP11-527F13.1		0	N.A.	0

RP11-527N22.2	0,228744416	0	N.A.	0
RP11-528G1.2	0,160213458	0	N.A.	0
RP11-529E10.7		0	N.A.	0
RP11-529E15.1		0	N.A.	0
RP11-529G21.2		0	N.A.	0
RP11-529K1.4		0	N.A.	0
RP11-530C5.1		0	N.A.	0
RP11-530C5.2		0	N.A.	0
RP11-530N7.3		0	N.A.	0
RP11-531H8.2		0	N.A.	0
RP11-532E4.2	0,201200549	0	N.A.	0
RP11-532F6.4	-0,081060161	0	N.A.	0
RP11-532N4.2		0	N.A.	0
RP11-535M15.1	0,242866184	0	N.A.	0
RP11-536C5.2	-0,025234084	0	N.A.	0
RP11-536I6.2		0	N.A.	0
RP11-536K7.5		0	N.A.	0
RP11-536O18.1		0	N.A.	0
RP11-537A6.9		0	N.A.	0
RP11-537H15.3		0	N.A.	0
RP11-538D16.2		0	N.A.	0
RP11-538I12.3		0	N.A.	0
RP11-539E17.5		0	N.A.	0
RP11-53B2.4		0	N.A.	0
RP11-53B5.1		0	N.A.	0
RP11-540O11.1	-0,022286942	0	N.A.	0
RP11-541P9.3	-0,223041116	0	N.A.	0
RP11-542A14.1		0	N.A.	0
RP11-542A14.2		0	N.A.	0
RP11-542B15.1		0	N.A.	0
RP11-542C16.1		0	N.A.	0
RP11-542C16.2		0	N.A.	0
RP11-542G1.1	0,252359688	0	N.A.	0

RP11-542K23.7	-0,045506036	0	N.A.	0
RP11-542M13.2		0	N.A.	0
RP11-542M13.3		0	N.A.	0
RP11-543C4.1	0,021255301	0	N.A.	0
RP11-543D10.2		0	N.A.	0
RP11-543D5.1	-0,267728939	0	N.A.	0
RP11-544D21.2		0	N.A.	0
RP11-544L8--B.4		0	N.A.	0
RP11-544M22.8		0	N.A.	0
RP11-545J16.1	0,196712056	0	N.A.	0
RP11-545N8.3	0,094912245	0	N.A.	0
RP11-545P7.4	0,303217865	0	N.A.	0
RP11-546B15.1	-0,114832045	0	N.A.	0
RP11-546M21.6	-0,068586	0	N.A.	0
RP11-546O6.4	-0,111916395	0	N.A.	0
RP11-547D23.1		0	N.A.	0
RP11-548P2.2		0	N.A.	0
RP11-549L6.3		0	N.A.	0
RP11-54A9.1		0	N.A.	0
RP11-54O15.3		0	N.A.	0
RP11-54O7.11		0	N.A.	0
RP11-54O7.16		0	N.A.	0
RP11-54O7.18	0,129694057	0	N.A.	0
RP11-54O7.2		0	N.A.	0
RP11-54O7.3		0	N.A.	0
RP11-550P17.5		0	N.A.	0
RP11-552D4.1		0	N.A.	0
RP11-552F3.10		0	N.A.	0
RP11-552F3.12		0	N.A.	0
RP11-552F3.13		0	N.A.	0
RP11-553A10.1	-0,021674945	0	N.A.	0
RP11-553K8.2		0	N.A.	0
RP11-553K8.5		0	N.A.	0

RP11-553L6.2		0	N.A.	0
RP11-553P9.2	0,015297525	0	N.A.	0
RP11-554A11.7		0	N.A.	0
RP11-554A11.9	-0,01079708	0	N.A.	0
RP11-554D13.1		0	N.A.	0
RP11-554D14.8		0	N.A.	0
RP11-554D15.1		0	N.A.	0
RP11-554D15.3	0,042126362	0	N.A.	0
RP11-554E23.2		0	N.A.	0
RP11-554F20.1		0	N.A.	0
RP11-554I8.1		0	N.A.	0
RP11-554I8.2	0,081232597	0	N.A.	0
RP11-556I14.1		0	N.A.	0
RP11-556O15.1	0,200508491	0	N.A.	0
RP11-557H15.4		0	N.A.	0
RP11-557L19.1		0	N.A.	0
RP11-560I19.1	-0,047696839	0	N.A.	0
RP11-561O23.5		0	N.A.	0
RP11-561O23.8		0	N.A.	0
RP11-561P12.5		0	N.A.	0
RP11-562A8.4	-0,072506321	0	N.A.	0
RP11-563D10.1		0	N.A.	0
RP11-563N12.2		0	N.A.	0
RP11-563P16.1		0	N.A.	0
RP11-565N2.2		0	N.A.	0
RP11-566K11.4		0	N.A.	0
RP11-567G11.1	0,313378805	0	N.A.	0
RP11-567I13.1		0	N.A.	0
RP11-567J20.2		0	N.A.	0
RP11-567J20.3		0	N.A.	0
RP11-567M16.2		0	N.A.	0
RP11-569A11.1	0,017447498	0	N.A.	0
RP11-56B16.4		0	N.A.	0

RP11-56B16.5	-0,085469688	0	N.A.	0
RP11-56G10.2		0	N.A.	0
RP11-56N19.5		0	N.A.	0
RP11-571E6.3		0	N.A.	0
RP11-571I18.5	0,116204999	0	N.A.	0
RP11-571M6.15		0	N.A.	0
RP11-571M6.7	0,156388106	0	N.A.	0
RP11-572C15.5		0	N.A.	0
RP11-572C21.1		0	N.A.	0
RP11-572M11.3		0	N.A.	0
RP11-572M11.4	-0,003416197	0	N.A.	0
RP11-572O6.1	0,145422675	0	N.A.	0
RP11-573G6.6		0	N.A.	0
RP11-573G6.8		0	N.A.	0
RP11-573G6.9		0	N.A.	0
RP11-574F21.3	0,119861328	0	N.A.	0
RP11-574H6.1		0	N.A.	0
RP11-574K11.31		0	N.A.	0
RP11-574K11.5		0	N.A.	0
RP11-574O7.1		0	N.A.	0
RP11-575L7.4		0	N.A.	0
RP11-576D8.4		0	N.A.	0
RP11-576I22.2	-0,016228904	0	N.A.	0
RP11-578F21.6		0	N.A.	0
RP11-579D7.2		0	N.A.	0
RP11-57C13.3	-0,006340534	0	N.A.	0
RP11-582J16.3	0,105962394	0	N.A.	0
RP11-583F2.2	0,13570527	0	N.A.	0
RP11-586D19.1	-0,000626971	0	N.A.	0
RP11-586D19.2		0	N.A.	0
RP11-586K2.1		0	N.A.	0
RP11-587P21.2	0,025295251	0	N.A.	0
RP11-589B3.6	0,044112527	0	N.A.	0

RP11-589C21.5		0	N.A.	0
RP11-589M4.1		0	N.A.	0
RP11-58K22.5		0	N.A.	0
RP11-593F23.1		0	N.A.	0
RP11-594N15.2		0	N.A.	0
RP11-597D13.8	-0,087091051	0	N.A.	0
RP11-597M12.2		0	N.A.	0
RP11-598F7.4	0,085710832	0	N.A.	0
RP11-598F7.5		0	N.A.	0
RP11-599B13.3	0,220915941	0	N.A.	0
RP11-59N23.1		0	N.A.	0
RP11-600L4.1	0,014501726	0	N.A.	0
RP11-603J24.14	0,083333248	0	N.A.	0
RP11-603J24.4		0	N.A.	0
RP11-603J24.5	0,216279624	0	N.A.	0
RP11-60I3.4		0	N.A.	0
RP11-611O2.5	-0,238862676	0	N.A.	0
RP11-613C6.2		0	N.A.	0
RP11-613D13.8	0,107473348	0	N.A.	0
RP11-613M10.6		0	N.A.	0
RP11-613M10.8		0	N.A.	0
RP11-613M10.9	-0,265781989	0	N.A.	0
RP11-614F17.2		0	N.A.	0
RP11-615I2.1		0	N.A.	0
RP11-616M22.11		0	N.A.	0
RP11-616M22.12		0	N.A.	0
RP11-616M22.3		0	N.A.	0
RP11-618K13.2		0	N.A.	0
RP11-618L22.1	-0,039671105	0	N.A.	0
RP11-619A14.3		0	N.A.	0
RP11-619L19.2		0	N.A.	0
RP11-61E11.2		0	N.A.	0
RP11-61F12.1		0	N.A.	0

RP11-61G19.1		0	N.A.	0
RP11-61I13.3		0	N.A.	0
RP11-61J19.3		0	N.A.	0
RP11-61K9.2		0	N.A.	0
RP11-61O1.2		0	N.A.	0
RP11-621L6.3		0	N.A.	0
RP11-622A1.2		0	N.A.	0
RP11-624C23.1	0,086912565	0	N.A.	0
RP11-624D11.2		0	N.A.	0
RP11-624J12.1	-0,067456333	0	N.A.	0
RP11-624L4.2	-0,086157723	0	N.A.	0
RP11-624M8.1		0	N.A.	0
RP11-625L16.1		0	N.A.	0
RP11-625L16.3		0	N.A.	0
RP11-626G11.4		0	N.A.	0
RP11-626H12.1	0,202715135	0	N.A.	0
RP11-626H12.3		0	N.A.	0
RP11-626I20.3		0	N.A.	0
RP11-627G18.1		0	N.A.	0
RP11-629G13.1	0,260618853	0	N.A.	0
RP11-629N8.3	0,232150643	0	N.A.	0
RP11-62C7.2		0	N.A.	0
RP11-62F24.1		0	N.A.	0
RP11-62F24.2	0,021619	0	N.A.	0
RP11-62H20.1		0	N.A.	0
RP11-62I21.1		0	N.A.	0
RP11-62J1.4	0,025924663	0	N.A.	0
RP11-632K5.3		0	N.A.	0
RP11-632L2.2		0	N.A.	0
RP11-634B7.4		0	N.A.	0
RP11-635L1.3		0	N.A.	0
RP11-638I2.4		0	N.A.	0
RP11-638I2.6	0,04890826	0	N.A.	0

RP11-63A1.1		0	N.A.	0
RP11-63A2.2		0	N.A.	0
RP11-63B13.1		0	N.A.	0
RP11-63G10.3		0	N.A.	0
RP11-63N8.3		0	N.A.	0
RP11-640I15.1		0	N.A.	0
RP11-640L9.1	0,092101135	0	N.A.	0
RP11-643C9.2		0	N.A.	0
RP11-644C3.1	0,208223365	0	N.A.	0
RP11-644F5.10		0	N.A.	0
RP11-644K8.1	0,043640545	0	N.A.	0
RP11-646E18.4	-0,004177906	0	N.A.	0
RP11-647K16.1	-0,177462839	0	N.A.	0
RP11-647P12.1		0	N.A.	0
RP11-649E7.5		0	N.A.	0
RP11-64B16.3		0	N.A.	0
RP11-64C1.1		0	N.A.	0
RP11-64D22.5	0,099417652	0	N.A.	0
RP11-64D24.4		0	N.A.	0
RP11-64P14.7		0	N.A.	0
RP11-650L12.1	0,182120548	0	N.A.	0
RP11-650L12.2		0	N.A.	0
RP11-651P23.5	0,360693745	0	N.A.	0
RP11-654D12.3	0,195904803	0	N.A.	0
RP11-655C2.3		0	N.A.	0
RP11-656D10.5		0	N.A.	0
RP11-656D10.6		0	N.A.	0
RP11-656E20.5		0	N.A.	0
RP11-65B7.2		0	N.A.	0
RP11-65D17.1		0	N.A.	0
RP11-65D24.2		0	N.A.	0
RP11-65G9.1	0,01262946	0	N.A.	0
RP11-65J21.1	0,129379736	0	N.A.	0

RP11-65J21.4		0	N.A.	0
RP11-65J3.2		0	N.A.	0
RP11-65J3.3		0	N.A.	0
RP11-65L19.4		0	N.A.	0
RP11-65M17.3		0	N.A.	0
RP11-660M5.1		0	N.A.	0
RP11-661A12.12	-5,82055E-05	0	N.A.	0
RP11-661A12.4	-0,072224274	0	N.A.	0
RP11-661A12.9	0,095441582	0	N.A.	0
RP11-661C8.3		0	N.A.	0
RP11-662G23.1		0	N.A.	0
RP11-662I13.2		0	N.A.	0
RP11-662J14.2		0	N.A.	0
RP11-663P9.1		0	N.A.	0
RP11-665C16.5		0	N.A.	0
RP11-665C16.6	0,13694583	0	N.A.	0
RP11-665G4.1		0	N.A.	0
RP11-665J16.1		0	N.A.	0
RP11-666A8.8	-0,074771848	0	N.A.	0
RP11-667K14.4	0,304138734	0	N.A.	0
RP11-667K14.5		0	N.A.	0
RP11-669M16.1		0	N.A.	0
RP11-66B24.1		0	N.A.	0
RP11-66B24.9		0	N.A.	0
RP11-66N24.7		0	N.A.	0
RP11-672A2.5		0	N.A.	0
RP11-672A2.6		0	N.A.	0
RP11-673E1.1		0	N.A.	0
RP11-673P17.4		0	N.A.	0
RP11-674P19.2		0	N.A.	0
RP11-675F6.3		0	N.A.	0
RP11-676J12.6	0,063444968	0	N.A.	0
RP11-676J12.7		0	N.A.	0

RP11-676J15.1		0	N.A.	0
RP11-677I18.3		0	N.A.	0
RP11-678G14.2	-0,312107551	0	N.A.	0
RP11-678G14.3		0	N.A.	0
RP11-678G14.4		0	N.A.	0
RP11-679B17.2		0	N.A.	0
RP11-679B19.1		0	N.A.	0
RP11-679B19.2		0	N.A.	0
RP11-679C8.2		0	N.A.	0
RP11-67H24.2		0	N.A.	0
RP11-67L3.5	-0,007771366	0	N.A.	0
RP11-680C21.1		0	N.A.	0
RP11-680F20.6		0	N.A.	0
RP11-680F20.9		0	N.A.	0
RP11-680G24.6	0,110328733	0	N.A.	0
RP11-684B21.1		0	N.A.	0
RP11-685M7.3	-0,210071283	0	N.A.	0
RP11-685N10.1		0	N.A.	0
RP11-686D22.4		0	N.A.	0
RP11-686D22.5		0	N.A.	0
RP11-686F15.2		0	N.A.	0
RP11-686G8.5		0	N.A.	0
RP11-686O6.1	-0,118104084	0	N.A.	0
RP11-687F6.5		0	N.A.	0
RP11-688I9.4		0	N.A.	0
RP11-689B22.2		0	N.A.	0
RP11-68I3.5		0	N.A.	0
RP11-68I3.7		0	N.A.	0
RP11-690P14.4		0	N.A.	0
RP11-693J15.5		0	N.A.	0
RP11-693J15.6		0	N.A.	0
RP11-693L9.2		0	N.A.	0
RP11-697E2.11		0	N.A.	0

RP11-697E22.1		0	N.A.	0
RP11-697E22.2	-0,133299686	0	N.A.	0
RP11-697H9.3		0	N.A.	0
RP11-698F20.3		0	N.A.	0
RP11-699L21.1	0,301406611	0	N.A.	0
RP11-69C13.1	0,086580496	0	N.A.	0
RP11-69G7.1		0	N.A.	0
RP11-69I8.2	0,107929843	0	N.A.	0
RP11-69L16.4		0	N.A.	0
RP11-6D1.3	0,064361634	0	N.A.	0
RP11-6L6.7		0	N.A.	0
RP11-6N17.2	0,29175436	0	N.A.	0
RP11-6O2.2	0,09102857	0	N.A.	0
RP11-6O2.3		0	N.A.	0
RP11-6O2.4		0	N.A.	0
RP11-700J17.1		0	N.A.	0
RP11-701P16.2		0	N.A.	0
RP11-702F3.1		0	N.A.	0
RP11-702F3.3		0	N.A.	0
RP11-702F3.4	-0,016863589	0	N.A.	0
RP11-702H23.4		0	N.A.	0
RP11-702H23.6		0	N.A.	0
RP11-703G6.1	0,268551018	0	N.A.	0
RP11-703I16.1		0	N.A.	0
RP11-703M24.5	-0,075276129	0	N.A.	0
RP11-704M14.1		0	N.A.	0
RP11-705C15.4		0	N.A.	0
RP11-705O24.1		0	N.A.	0
RP11-706C16.8		0	N.A.	0
RP11-706D8.3		0	N.A.	0
RP11-707P17.2	0,174649506	0	N.A.	0
RP11-709D24.5		0	N.A.	0
RP11-70D24.2		0	N.A.	0

RP11-70D24.3		0	N.A.	0
RP11-70F11.8	0,053561122	0	N.A.	0
RP11-70L8.4		0	N.A.	0
RP11-710C12.1		0	N.A.	0
RP11-710E1.2		0	N.A.	0
RP11-712L6.7	-0,082307229	0	N.A.	0
RP11-713D19.1		0	N.A.	0
RP11-713N11.5		0	N.A.	0
RP11-713P17.4		0	N.A.	0
RP11-714L20.1	0,260618853	0	N.A.	0
RP11-715J22.3	-0,011199851	0	N.A.	0
RP11-715J22.4		0	N.A.	0
RP11-718O11.1		0	N.A.	0
RP11-71E19.1		0	N.A.	0
RP11-71G12.1		0	N.A.	0
RP11-71N10.1		0	N.A.	0
RP11-720L2.4		0	N.A.	0
RP11-723D22.3	-0,115044285	0	N.A.	0
RP11-723O4.6	0,222388481	0	N.A.	0
RP11-724N1.1		0	N.A.	0
RP11-728F11.4	0,08690519	0	N.A.	0
RP11-728K20.3	0,060385538	0	N.A.	0
RP11-729L2.2		0	N.A.	0
RP11-72L22.1		0	N.A.	0
RP11-731D1.4	0,243666365	0	N.A.	0
RP11-731F5.2	-0,225163244	0	N.A.	0
RP11-731K22.1	-0,081774836	0	N.A.	0
RP11-732A19.5		0	N.A.	0
RP11-732A19.8	0,036877935	0	N.A.	0
RP11-732A19.9		0	N.A.	0
RP11-732A21.2		0	N.A.	0
RP11-733O18.1		0	N.A.	0
RP11-734K21.3	-0,091972288	0	N.A.	0

RP11-734K23.9		0	N.A.	0
RP11-735G4.1		0	N.A.	0
RP11-736N17.10		0	N.A.	0
RP11-737O24.2		0	N.A.	0
RP11-737O24.5		0	N.A.	0
RP11-739N20.2	-0,054302529	0	N.A.	0
RP11-73G16.2	0,237871634	0	N.A.	0
RP11-73M14.1		0	N.A.	0
RP11-73M7.6	0,346986164	0	N.A.	0
RP11-73M7.9	-0,099739463	0	N.A.	0
RP11-73O6.3		0	N.A.	0
RP11-742B18.1		0	N.A.	0
RP11-744J10.3		0	N.A.	0
RP11-745C15.2	0,044112527	0	N.A.	0
RP11-745O10.4		0	N.A.	0
RP11-746M1.1		0	N.A.	0
RP11-74E22.5		0	N.A.	0
RP11-74M11.2		0	N.A.	0
RP11-750H9.5	0,005824376	0	N.A.	0
RP11-752D24.3		0	N.A.	0
RP11-752L20.5		0	N.A.	0
RP11-753D20.3		0	N.A.	0
RP11-753H16.3		0	N.A.	0
RP11-753H16.5		0	N.A.	0
RP11-754B17.1		0	N.A.	0
RP11-755B10.2	0,044112527	0	N.A.	0
RP11-755F10.3		0	N.A.	0
RP11-755O11.2		0	N.A.	0
RP11-756G20.1		0	N.A.	0
RP11-756P10.2		0	N.A.	0
RP11-757A13.1	0,021619	0	N.A.	0
RP11-757G1.6		0	N.A.	0
RP11-758M4.4	0,033111108	0	N.A.	0

RP11-758P17.2	0,057650746	0	N.A.	0
RP11-75C10.9		0	N.A.	0
RP11-75L1.1		0	N.A.	0
RP11-761I4.5		0	N.A.	0
RP11-761N21.1		0	N.A.	0
RP11-762E8.1		0	N.A.	0
RP11-762H8.1		0	N.A.	0
RP11-762H8.3		0	N.A.	0
RP11-762I7.5	0,031294157	0	N.A.	0
RP11-764E7.1		0	N.A.	0
RP11-764K9.1		0	N.A.	0
RP11-767I20.1		0	N.A.	0
RP11-767N15.1		0	N.A.	0
RP11-768B22.2		0	N.A.	0
RP11-768G7.2		0	N.A.	0
RP11-769N22.1		0	N.A.	0
RP11-769O8.1	0,205487926	0	N.A.	0
RP11-769O8.2		0	N.A.	0
RP11-76E17.3	-0,087091051	0	N.A.	0
RP11-76E17.4		0	N.A.	0
RP11-76I14.1		0	N.A.	0
RP11-770E5.1		0	N.A.	0
RP11-770E5.3		0	N.A.	0
RP11-773H22.4	-0,169313749	0	N.A.	0
RP11-774D14.1		0	N.A.	0
RP11-775C24.3		0	N.A.	0
RP11-775H9.3		0	N.A.	0
RP11-77A13.1		0	N.A.	0
RP11-77I22.2	0,007570482	0	N.A.	0
RP11-77K12.1	0,1175721	0	N.A.	0
RP11-77K12.7	0,07734317	0	N.A.	0
RP11-780K2.1		0	N.A.	0
RP11-781A6.1		0	N.A.	0

RP11-789C2.1	-0,180700722	0	N.A.	0
RP11-78A19.4	0,242696037	0	N.A.	0
RP11-78F17.1		0	N.A.	0
RP11-791M20.1		0	N.A.	0
RP11-794M8.1		0	N.A.	0
RP11-794P6.6		0	N.A.	0
RP11-797D24.3	0,119613478	0	N.A.	0
RP11-797D24.4	0,160985156	0	N.A.	0
RP11-798G7.5		0	N.A.	0
RP11-798K3.3		0	N.A.	0
RP11-799B12.2		0	N.A.	0
RP11-799M12.2		0	N.A.	0
RP11-799N11.1		0	N.A.	0
RP11-79M19.2		0	N.A.	0
RP11-79N23.1		0	N.A.	0
RP11-7F17.3		0	N.A.	0
RP11-7F17.5		0	N.A.	0
RP11-7F17.7		0	N.A.	0
RP11-7I15.3	-0,019996083	0	N.A.	0
RP11-800A3.3		0	N.A.	0
RP11-804A23.4		0	N.A.	0
RP11-805I24.3		0	N.A.	0
RP11-806L2.2		0	N.A.	0
RP11-806L2.6		0	N.A.	0
RP11-807H22.7		0	N.A.	0
RP11-809H16.5		0	N.A.	0
RP11-809N8.2		0	N.A.	0
RP11-80A15.1	0,142867239	0	N.A.	0
RP11-80H5.9	0,006800455	0	N.A.	0
RP11-810P12.5		0	N.A.	0
RP11-814H16.2		0	N.A.	0
RP11-815J21.2		0	N.A.	0
RP11-815J21.3		0	N.A.	0

RP11-815J21.4	-0,087091051	0	N.A.	0
RP11-815M8.1	0,060602299	0	N.A.	0
RP11-816J6.3	0,076215755	0	N.A.	0
RP11-817G13.3		0	N.A.	0
RP11-817J15.3	0,295846442	0	N.A.	0
RP11-81H14.1		0	N.A.	0
RP11-81H3.2	0,271131357	0	N.A.	0
RP11-81K2.1		0	N.A.	0
RP11-820I16.1		0	N.A.	0
RP11-820I16.3	0,234723945	0	N.A.	0
RP11-829H16.3		0	N.A.	0
RP11-82L7.4		0	N.A.	0
RP11-82O19.1	-0,059298536	0	N.A.	0
RP11-830F9.5		0	N.A.	0
RP11-834C11.11	0,118826305	0	N.A.	0
RP11-834C11.4		0	N.A.	0
RP11-834C11.5		0	N.A.	0
RP11-834C11.6		0	N.A.	0
RP11-834C11.8		0	N.A.	0
RP11-837J7.4	0,147611564	0	N.A.	0
RP11-839D17.3		0	N.A.	0
RP11-839G9.1	0,275497721	0	N.A.	0
RP11-83M16.6		0	N.A.	0
RP11-83N9.6		0	N.A.	0
RP11-840I19.3		0	N.A.	0
RP11-843A23.1		0	N.A.	0
RP11-843B15.2		0	N.A.	0
RP11-844P9.2	-0,086628904	0	N.A.	0
RP11-845C23.3		0	N.A.	0
RP11-845M18.6		0	N.A.	0
RP11-845M18.7		0	N.A.	0
RP11-847H18.2	0,022160425	0	N.A.	0
RP11-847H18.3	0,005147662	0	N.A.	0

RP11-848P1.3		0	N.A.	0
RP11-849F2.5		0	N.A.	0
RP11-84A14.5		0	N.A.	0
RP11-84A19.2	0,230640844	0	N.A.	0
RP11-84C10.2		0	N.A.	0
RP11-84C13.2	-0,052794271	0	N.A.	0
RP11-84D1.1		0	N.A.	0
RP11-84D1.2		0	N.A.	0
RP11-854K16.3		0	N.A.	0
RP11-855A2.3		0	N.A.	0
RP11-855A2.5		0	N.A.	0
RP11-856M7.6	0,01456601	0	N.A.	0
RP11-857B24.5	0,044112527	0	N.A.	0
RP11-85B7.4		0	N.A.	0
RP11-85B7.5	0,041839506	0	N.A.	0
RP11-85E16.1		0	N.A.	0
RP11-85G18.6		0	N.A.	0
RP11-85I21.1		0	N.A.	0
RP11-860B13.1		0	N.A.	0
RP11-861E21.2		0	N.A.	0
RP11-862G15.2		0	N.A.	0
RP11-862P13.1		0	N.A.	0
RP11-863H1.1		0	N.A.	0
RP11-863P13.1		0	N.A.	0
RP11-863P13.4		0	N.A.	0
RP11-863P13.5		0	N.A.	0
RP11-864G5.3	-0,076351601	0	N.A.	0
RP11-864I4.1	-0,238862676	0	N.A.	0
RP11-864I4.4	0,041400317	0	N.A.	0
RP11-865I6.2		0	N.A.	0
RP11-867G2.5	0,146185406	0	N.A.	0
RP11-867G2.8	0,035222078	0	N.A.	0
RP11-867G23.2		0	N.A.	0

RP11-867G23.3		0	N.A.	0
RP11-867O8.5	0,301828534	0	N.A.	0
RP11-875O11.2		0	N.A.	0
RP11-876N24.2		0	N.A.	0
RP11-879F14.1		0	N.A.	0
RP11-879F14.2	-0,263525398	0	N.A.	0
RP11-879F14.3		0	N.A.	0
RP11-87E22.2		0	N.A.	0
RP11-87F15.2	0,286663739	0	N.A.	0
RP11-881L2.1	0,241452567	0	N.A.	0
RP11-881M11.1	-0,014715036	0	N.A.	0
RP11-881M11.2		0	N.A.	0
RP11-881M11.4		0	N.A.	0
RP11-883A18.3	0,069314768	0	N.A.	0
RP11-885B4.2		0	N.A.	0
RP11-885N19.6		0	N.A.	0
RP11-886P16.6		0	N.A.	0
RP11-887P2.5		0	N.A.	0
RP11-888D10.3		0	N.A.	0
RP11-88E10.4		0	N.A.	0
RP11-88G17.6		0	N.A.	0
RP11-88H9.2		0	N.A.	0
RP11-893F2.13		0	N.A.	0
RP11-893F2.14		0	N.A.	0
RP11-893F2.15		0	N.A.	0
RP11-893F2.5		0	N.A.	0
RP11-893F2.6		0	N.A.	0
RP11-894J14.5		0	N.A.	0
RP11-895M11.3		0	N.A.	0
RP11-89C3.4		0	N.A.	0
RP11-89H19.1		0	N.A.	0
RP11-89N17.1		0	N.A.	0
RP11-8L8.2		0	N.A.	0

RP11-900F13.2		0	N.A.	0
RP11-900F13.3	-0,093920636	0	N.A.	0
RP11-903H12.3	0,101926428	0	N.A.	0
RP11-903H12.5	0,287638428	0	N.A.	0
RP11-905K4.1		0	N.A.	0
RP11-90B22.1	0,272093854	0	N.A.	0
RP11-90C1.1		0	N.A.	0
RP11-90J7.2		0	N.A.	0
RP11-90J7.4		0	N.A.	0
RP11-90L20.2		0	N.A.	0
RP11-90P13.1		0	N.A.	0
RP11-90P5.2	0,319402286	0	N.A.	0
RP11-91A18.4		0	N.A.	0
RP11-91I20.2		0	N.A.	0
RP11-91I20.3		0	N.A.	0
RP11-91I20.4		0	N.A.	0
RP11-91I8.3		0	N.A.	0
RP11-91J19.3	-0,338316193	0	N.A.	0
RP11-91K8.5	0,284521957	0	N.A.	0
RP11-91K9.1		0	N.A.	0
RP11-91P24.5		0	N.A.	0
RP11-91P24.6	-0,105785875	0	N.A.	0
RP11-91P24.7	-0,030506586	0	N.A.	0
RP11-923I11.3		0	N.A.	0
RP11-923I11.5		0	N.A.	0
RP11-923I11.6		0	N.A.	0
RP11-923I11.8		0	N.A.	0
RP11-92C4.3	0,104529206	0	N.A.	0
RP11-92K15.1		0	N.A.	0
RP11-930P14.1		0	N.A.	0
RP11-932O9.7	-0,049240528	0	N.A.	0
RP11-932O9.8	0,013324742	0	N.A.	0
RP11-932O9.9		0	N.A.	0

RP11-93I21.3		0	N.A.	0
RP11-93K22.6	-0,040132196	0	N.A.	0
RP11-944L7.4		0	N.A.	0
RP11-946L16.2		0	N.A.	0
RP11-946L20.2	0,364379078	0	N.A.	0
RP11-94A24.1	0,401970305	0	N.A.	0
RP11-94B19.3		0	N.A.	0
RP11-94C24.13		0	N.A.	0
RP11-94H18.1		0	N.A.	0
RP11-950C14.3		0	N.A.	0
RP11-950C14.7	-0,179645805	0	N.A.	0
RP11-955H22.1		0	N.A.	0
RP11-956E11.1		0	N.A.	0
RP11-95H3.1		0	N.A.	0
RP11-95I16.6	0,089738762	0	N.A.	0
RP11-95M5.1	-0,108258475	0	N.A.	0
RP11-960L18.1		0	N.A.	0
RP11-961A15.1		0	N.A.	0
RP11-962G15.1	-0,209276001	0	N.A.	0
RP11-968A15.2		0	N.A.	0
RP11-96A15.1	0,194997311	0	N.A.	0
RP11-96B2.1	0,294749356	0	N.A.	0
RP11-96B5.3	0,018448765	0	N.A.	0
RP11-96C21.1	-0,23918289	0	N.A.	0
RP11-96K19.2	-0,225987027	0	N.A.	0
RP11-96P7.1		0	N.A.	0
RP11-973H7.1		0	N.A.	0
RP11-973H7.4		0	N.A.	0
RP11-976B16.1		0	N.A.	0
RP11-977G19.10	0,018448765	0	N.A.	0
RP11-977G19.14		0	N.A.	0
RP11-981G7.2		0	N.A.	0
RP11-982M15.2	0,17181073	0	N.A.	0

RP11-982M15.7		0	N.A.	0
RP11-982M15.8		0	N.A.	0
RP11-989F5.1		0	N.A.	0
RP11-989F5.3		0	N.A.	0
RP11-98D18.1	0,01926779	0	N.A.	0
RP11-98D18.15		0	N.A.	0
RP11-98F14.11	0,132468456	0	N.A.	0
RP11-991C1.1		0	N.A.	0
RP11-998D10.4		0	N.A.	0
RP11-99E15.2	0,314760534	0	N.A.	0
RP11-99J16--A.2	-0,094981875	0	N.A.	0
RP11-99L13.2		0	N.A.	0
RP11-9G1.3		0	N.A.	0
RP11-9M16.2		0	N.A.	0
RP11-9N20.3	0,126983831	0	N.A.	0
RP13-1016M1.2		0	N.A.	0
RP13-1039J1.3		0	N.A.	0
RP13-126P21.2		0	N.A.	0
RP13-143G15.4		0	N.A.	0
RP13-16H11.2	-0,266207112	0	N.A.	0
RP13-16H11.5	-0,088907486	0	N.A.	0
RP13-228J13.1		0	N.A.	0
RP13-270P17.2	0,047601491	0	N.A.	0
RP13-297E16.4		0	N.A.	0
RP13-349O20.2		0	N.A.	0
RP13-379O24.2		0	N.A.	0
RP13-436F16.1	-0,126200059	0	N.A.	0
RP13-444H2.1		0	N.A.	0
RP13-465B17.4		0	N.A.	0
RP13-465B17.5	-0,113438735	0	N.A.	0
RP13-476E20.1		0	N.A.	0
RP13-49I15.6	0,289394926	0	N.A.	0
RP13-514E23.2		0	N.A.	0

RP13-516M14.2		0	N.A.	0
RP13-578N3.3		0	N.A.	0
RP13-614K11.2		0	N.A.	0
RP13-631K18.2	0,335806186	0	N.A.	0
RP13-631K18.3		0	N.A.	0
RP13-631K18.5		0	N.A.	0
RP13-638C3.3	0,098314877	0	N.A.	0
RP13-672B3.2	0,282586412	0	N.A.	0
RP13-735L24.1		0	N.A.	0
RP13-766D20.2	-0,019422578	0	N.A.	0
RP13-820C6.2	-0,089380209	0	N.A.	0
RP13-884E18.2	0,161501152	0	N.A.	0
RP13-884E18.4		0	N.A.	0
RP13-895J2.2	0,085348244	0	N.A.	0
RP13-895J2.6	0,144277124	0	N.A.	0
RP13-941N14.1		0	N.A.	0
RP13-977J11.8		0	N.A.	0
RP13-977J11.9		0	N.A.	0
RP13-991F5.2		0	N.A.	0
RP3-322G13.7	-0,310739126	0	N.A.	0
RP3-323N1.2		0	N.A.	0
RP3-325F22.3		0	N.A.	0
RP3-329A5.8		0	N.A.	0
RP3-330M21.5		0	N.A.	0
RP3-331H24.5		0	N.A.	0
RP3-332B22.1	-0,025234084	0	N.A.	0
RP3-333A15.1		0	N.A.	0
RP3-333B15.5	-0,148589307	0	N.A.	0
RP3-337H4.8		0	N.A.	0
RP3-340N1.2		0	N.A.	0
RP3-355L5.4		0	N.A.	0
RP3-359N14.2		0	N.A.	0
RP3-369A17.4		0	N.A.	0

RP3-388M5.9		0	N.A.	0
RP3-395M20.2		0	N.A.	0
RP3-400B16.1		0	N.A.	0
RP3-400B16.4		0	N.A.	0
RP3-403L10.3		0	N.A.	0
RP3-405J10.4		0	N.A.	0
RP3-406P24.3	-0,030121414	0	N.A.	0
RP3-413H6.2		0	N.A.	0
RP3-425P12.1		0	N.A.	0
RP3-425P12.5		0	N.A.	0
RP3-428L16.1		0	N.A.	0
RP3-429O6.1		0	N.A.	0
RP3-430N8.11	0,212849304	0	N.A.	0
RP3-434P1.6	0,084366803	0	N.A.	0
RP3-438O4.4	0,456794633	0	N.A.	0
RP3-439F8.1	0,02386129	0	N.A.	0
RP3-441A12.1	-0,063223559	0	N.A.	0
RP3-448I9.2		0	N.A.	0
RP3-449H6.1		0	N.A.	0
RP3-449M8.6		0	N.A.	0
RP3-462D8.2		0	N.A.	0
RP3-466P17.2		0	N.A.	0
RP3-467K16.7		0	N.A.	0
RP3-468B3.2		0	N.A.	0
RP3-470B24.5		0	N.A.	0
RP3-471M13.2	0,322723805	0	N.A.	0
RP3-483K16.4		0	N.A.	0
RP3-486I3.5		0	N.A.	0
RP3-486I3.7	-0,080325017	0	N.A.	0
RP3-495K2.2		0	N.A.	0
RP3-508I15.10		0	N.A.	0
RP3-508I15.19		0	N.A.	0
RP3-510H16.3		0	N.A.	0

RP3-512E2.2		0	N.A.	0
RP3-522J7.6	0,057897758	0	N.A.	0
RP3-522P13.3		0	N.A.	0
RP3-523C21.2	0,077261407	0	N.A.	0
RP3-525N10.2		0	N.A.	0
RP4-529N6.1		0	N.A.	0
RP4-530I15.6		0	N.A.	0
RP4-530I15.9		0	N.A.	0
RP4-535B20.4	-0,102742458	0	N.A.	0
RP4-536B24.2		0	N.A.	0
RP4-536B24.3		0	N.A.	0
RP4-537K23.4	0,382408213	0	N.A.	0
RP4-539M6.14		0	N.A.	0
RP4-539M6.19		0	N.A.	0
RP4-543J13.1		0	N.A.	0
RP4-549F15.1		0	N.A.	0
RP4-550H1.4		0	N.A.	0
RP4-553F4.2		0	N.A.	0
RP4-555D20.2		0	N.A.	0
RP4-555D20.4		0	N.A.	0
RP4-555L14.4		0	N.A.	0
RP4-575N6.4		0	N.A.	0
RP4-580N22.2		0	N.A.	0
RP4-583P15.16		0	N.A.	0
RP4-584D14.5	-0,220869649	0	N.A.	0
RP4-584D14.6		0	N.A.	0
RP4-585I14.3		0	N.A.	0
RP4-594A5.1	0,05426266	0	N.A.	0
RP4-598P13.1		0	N.A.	0
RP4-601P9.2		0	N.A.	0
RP4-604G5.1		0	N.A.	0
RP4-604K5.3		0	N.A.	0
RP4-607I7.1		0	N.A.	0

RP4-613B23.5		0	N.A.	0
RP4-614O4.11	0,35469583	0	N.A.	0
RP4-616B8.5	0,042809058	0	N.A.	0
RP4-620F22.2		0	N.A.	0
RP4-621B10.8	0,311341217	0	N.A.	0
RP4-633I8.4		0	N.A.	0
RP4-633O19--A.1	0,023762925	0	N.A.	0
RP4-639J15.3		0	N.A.	0
RP4-640H8.2		0	N.A.	0
RP4-644L1.2		0	N.A.	0
RP4-647C14.2		0	N.A.	0
RP4-655C5.4		0	N.A.	0
RP4-660H19.1	0,273853928	0	N.A.	0
RP4-663N10.1		0	N.A.	0
RP4-668J24.2		0	N.A.	0
RP4-669P10.16	0,133483091	0	N.A.	0
RP4-669P10.20		0	N.A.	0
RP4-676J13.2		0	N.A.	0
RP4-678D15.1	0,03776502	0	N.A.	0
RP4-680D5.9		0	N.A.	0
RP4-681N20.5		0	N.A.	0
RP4-682C21.5		0	N.A.	0
RP4-684O24.5		0	N.A.	0
RP4-686C3.7		0	N.A.	0
RP4-694A7.2		0	N.A.	0
RP4-706G24.1		0	N.A.	0
RP4-712E4.1	0,107001707	0	N.A.	0
RP4-712E4.2	0,087656925	0	N.A.	0
RP4-715N11.2		0	N.A.	0
RP4-724E16.2	0,02498731	0	N.A.	0
RP4-734C18.1		0	N.A.	0
RP4-735C1.4		0	N.A.	0
RP4-736L20.3	-0,168168316	0	N.A.	0

RP4-738P15.1		0	N.A.	0
RP4-742J24.2		0	N.A.	0
RP4-745E8.2		0	N.A.	0
RP4-751H13.7		0	N.A.	0
RP4-752I6.1		0	N.A.	0
RP4-760C5.5		0	N.A.	0
RP4-765H13.1		0	N.A.	0
RP4-769N13.7	-0,082307229	0	N.A.	0
RP4-771M4.3		0	N.A.	0
RP4-777L9.3		0	N.A.	0
RP4-777O23.1		0	N.A.	0
RP4-777O23.3	-0,330646628	0	N.A.	0
RP4-778K6.3		0	N.A.	0
RP4-781K5.5		0	N.A.	0
RP4-781K5.6		0	N.A.	0
RP4-784A16.5		0	N.A.	0
RP4-791M13.3		0	N.A.	0
RP4-798A10.4		0	N.A.	0
RP4-799D16.1		0	N.A.	0
RP4-806M20.3		0	N.A.	0
RP4-806M20.4		0	N.A.	0
RP4-809F18.2		0	N.A.	0
RP4-813D12.3	-0,234142143	0	N.A.	0
RP4-816N1.7	0,107840651	0	N.A.	0
RP5-1011O1.3		0	N.A.	0
RP5-1017F8.2		0	N.A.	0
RP5-1018K9.1	0,14797175	0	N.A.	0
RP5-1021I20.2		0	N.A.	0
RP5-1021I20.4	-0,070338456	0	N.A.	0
RP5-1021I20.5	0,175796347	0	N.A.	0
RP5-1021I20.8	0,128298425	0	N.A.	0
RP5-1022P6.5		0	N.A.	0
RP5-1022P6.7	-0,153902306	0	N.A.	0

RP5-1024C24.1		0	N.A.	0
RP5-1024G6.2		0	N.A.	0
RP5-1024N4.2		0	N.A.	0
RP5-1024N4.4	0,011169857	0	N.A.	0
RP5-1029F21.3		0	N.A.	0
RP5-1031D4.2		0	N.A.	0
RP5-1039K5.17	-0,299354089	0	N.A.	0
RP5-1039K5.18		0	N.A.	0
RP5-1042K10.13		0	N.A.	0
RP5-1050D4.2		0	N.A.	0
RP5-1050D4.4	0,339502795	0	N.A.	0
RP5-1050E16.2		0	N.A.	0
RP5-1052I5.2		0	N.A.	0
RP5-1056L3.1		0	N.A.	0
RP5-1057B20.3		0	N.A.	0
RP5-1065P14.2		0	N.A.	0
RP5-1066H13.4		0	N.A.	0
RP5-1068H6.6		0	N.A.	0
RP5-1069C8.2		0	N.A.	0
RP5-1071N3.1		0	N.A.	0
RP5-1077I2.3	0,105416771	0	N.A.	0
RP5-1087E8.3	-0,100625426	0	N.A.	0
RP5-1092A11.5		0	N.A.	0
RP5-1092A3.5		0	N.A.	0
RP5-1096D14.3		0	N.A.	0
RP5-1096J16.1		0	N.A.	0
RP5-1101C3.1	0,251966183	0	N.A.	0
RP5-1102E8.3		0	N.A.	0
RP5-1103G7.10		0	N.A.	0
RP5-1113E3.3		0	N.A.	0
RP5-1115A15.1	0,143789501	0	N.A.	0
RP5-1116H23.3	0,044112527	0	N.A.	0
RP5-1116H23.5		0	N.A.	0

RP5-1116H23.6		0	N.A.	0
RP5-1120P11.3		0	N.A.	0
RP5-1121H13.4		0	N.A.	0
RP5-1142A6.10		0	N.A.	0
RP5-1142A6.2		0	N.A.	0
RP5-1142A6.9		0	N.A.	0
RP5-1142J19.1		0	N.A.	0
RP5-1172A22.1		0	N.A.	0
RP5-1172N10.2	-0,088928456	0	N.A.	0
RP5-1180E21.4	0,097617444	0	N.A.	0
RP5-1182A14.5		0	N.A.	0
RP5-1184F4.5	-0,100152379	0	N.A.	0
RP5-1184F4.7		0	N.A.	0
RP5-1185H19.2	-0,136494867	0	N.A.	0
RP5-823G15.5	-0,048863145	0	N.A.	0
RP5-828H9.1		0	N.A.	0
RP5-836J3.1		0	N.A.	0
RP5-837I24.4		0	N.A.	0
RP5-839B4.8	-0,050139777	0	N.A.	0
RP5-855D21.1		0	N.A.	0
RP5-856G1.1	-0,150347513	0	N.A.	0
RP5-864K19.6	0,360634656	0	N.A.	0
RP5-867C24.5		0	N.A.	0
RP5-881P19.7		0	N.A.	0
RP5-884C9.2		0	N.A.	0
RP5-887A10.1		0	N.A.	0
RP5-890E16.5		0	N.A.	0
RP5-899B16.1	0,101741538	0	N.A.	0
RP5-901A4.1		0	N.A.	0
RP5-902P8.10	0,030548322	0	N.A.	0
RP5-902P8.12	-0,120597876	0	N.A.	0
RP5-905N1.2		0	N.A.	0
RP5-906C1.1		0	N.A.	0

RP5-907D15.3		0	N.A.	0
RP5-907D15.4	0,121777708	0	N.A.	0
RP5-919F19.5		0	N.A.	0
RP5-921G16.1	0,214110073	0	N.A.	0
RP5-921G16.2		0	N.A.	0
RP5-930J4.2		0	N.A.	0
RP5-940J5.3		0	N.A.	0
RP5-940J5.8		0	N.A.	0
RP5-947P14.1		0	N.A.	0
RP5-952N6.1		0	N.A.	0
RP5-956O18.3		0	N.A.	0
RP5-965F6.2		0	N.A.	0
RP5-966M1.6	-0,195763393	0	N.A.	0
RP5-968D22.1		0	N.A.	0
RP5-971N18.3		0	N.A.	0
RP5-973N23.4		0	N.A.	0
RP5-981O7.2	-0,055489061	0	N.A.	0
RP5-991G20.2	-0,072500795	0	N.A.	0
RP5-991G20.6	-0,178047786	0	N.A.	0
RP5-997D24.3		0	N.A.	0
RP5-998N21.4		0	N.A.	0
RP6-109B7.2		0	N.A.	0
RP6-114E22.1	-0,042302686	0	N.A.	0
RP6-159A1.4	0,251277806	0	N.A.	0
RP6-191P20.4		0	N.A.	0
RP6-74O6.2		0	N.A.	0
RP6-91H8.2		0	N.A.	0
RP6-91H8.5		0	N.A.	0
RPEL1	-0,150198417	0	N.A.	0
RPGRIP1		0	N.A.	0
RPH3A		0	N.A.	0
RRH		0	N.A.	0
RRM1-AS1		0	N.A.	0

RSPH4A			0	N.A.	0
RSPO1		0,047658527	0	N.A.	0
RSPO4		-0,05083931	0	N.A.	0
RTCA-AS1		-0,066195532	0	N.A.	0
RTEL1		0,055551195	0	N.A.	0
RTP1			0	N.A.	0
RTP5			0	N.A.	0
RUFY4		0,04909427	0	N.A.	0
RUVBL1-AS1			0	N.A.	0
RXFP1			0	N.A.	0
RXRG			0	N.A.	0
RYR1			0	N.A.	0
RYR3			0	N.A.	0
S100G		-0,008452583	0	N.A.	0
S1PR5			0	N.A.	0
SAA2-SAA4		0,301828534	0	N.A.	0
SACS-AS1			0	N.A.	0
SAGE1			0	N.A.	0
SALL1			0	N.A.	0
SALL2			0	N.A.	0
SAMD7			0	N.A.	0
SAMSN1-AS1			0	N.A.	0
SAPCD1		-0,123447259	0	N.A.	0
SARDH			0	N.A.	0
SBK3			0	N.A.	0
SCARA5			0	N.A.	0
SCARNA9		-0,192592473	0	N.A.	0
SCGB1A1			0	N.A.	0
SCGB1D2			0	N.A.	0
SCGB2A2			0	N.A.	0
SCGB2B2			0	N.A.	0
SCGB3A1		0,262738574	0	N.A.	0
SCHIP1			0	N.A.	0

SCHLAP1			0	N.A.	0
SCIMP		-0,238678317	0	N.A.	0
SCN2A			0	N.A.	0
SCN2B			0	N.A.	0
SCN4B		0,221072751	0	N.A.	0
SCN5A			0	N.A.	0
SCN7A			0	N.A.	0
SCUBE1			0	N.A.	0
SDK1			0	N.A.	0
SDPR		0,178485334	0	N.A.	0
SDR9C7		0,012371259	0	N.A.	0
SEC14L5		0,139011887	0	N.A.	0
SEC14L6			0	N.A.	0
SEC62-AS1		0,04509252	0	N.A.	0
SEL1L2		0,275184814	0	N.A.	0
SELP			0	N.A.	0
SELV			0	N.A.	0
SEMA3E			0	N.A.	0
SEMA3F-AS1			0	N.A.	0
SEMA5B			0	N.A.	0
SENCR		-0,338316193	0	N.A.	0
SENP3-EIF4A1			0	N.A.	0
SEPT4-AS1		-0,282912902	0	N.A.	0
SERPINA3			0	N.A.	0
SERPINA5			0	N.A.	0
SERPINA9			0	N.A.	0
SERPINB13			0	N.A.	0
SERPINC1		0,04503565	0	N.A.	0
SERPINE3			0	N.A.	0
SERPINI2			0	N.A.	0
SERTM1			0	N.A.	0
SEZ6L			0	N.A.	0
SFMBT2		0,279288119	0	N.A.	0

SFTPB		0,085086587	0	N.A.	0
SFTPD			0	N.A.	0
SGCA		-0,168610581	0	N.A.	0
SGCD			0	N.A.	0
SGCG			0	N.A.	0
SGIP1		0,042606676	0	N.A.	0
SH2D1B			0	N.A.	0
SH2D7		-0,174508826	0	N.A.	0
SH3GL3			0	N.A.	0
SH3RF3-AS1			0	N.A.	0
SHANK1			0	N.A.	0
SHCBP1L			0	N.A.	0
SHE		-0,110941701	0	N.A.	0
SHOX		0,020125584	0	N.A.	0
SIAH2-AS1			0	N.A.	0
SIAH3			0	N.A.	0
SIGLEC11			0	N.A.	0
SIGLEC14			0	N.A.	0
SIGLEC5			0	N.A.	0
SIGLEC6		-0,080170565	0	N.A.	0
SIGLEC8			0	N.A.	0
SIM1			0	N.A.	0
SIRPB2			0	N.A.	0
SIRPD			0	N.A.	0
SIRPG-AS1			0	N.A.	0
SLC10A1			0	N.A.	0
SLC10A4		-0,073585913	0	N.A.	0
SLC10A6			0	N.A.	0
SLC12A1			0	N.A.	0
SLC12A3			0	N.A.	0
SLC14A2-AS1			0	N.A.	0
SLC16A6		-0,048345425	0	N.A.	0
SLC16A8			0	N.A.	0

SLC17A3			0	N.A.	0
SLC1A6			0	N.A.	0
SLC22A10			0	N.A.	0
SLC22A13			0	N.A.	0
SLC22A16			0	N.A.	0
SLC22A9			0	N.A.	0
SLC24A2		0,344148805	0	N.A.	0
SLC24A4		0,154953718	0	N.A.	0
SLC24A5			0	N.A.	0
SLC25A2			0	N.A.	0
SLC25A31			0	N.A.	0
SLC25A52			0	N.A.	0
SLC26A4-AS1			0	N.A.	0
SLC26A5			0	N.A.	0
SLC27A6			0	N.A.	0
SLC28A1			0	N.A.	0
SLC2A7			0	N.A.	0
SLC30A10		0,157179603	0	N.A.	0
SLC30A8			0	N.A.	0
SLC32A1			0	N.A.	0
SLC34A1		0,063444968	0	N.A.	0
SLC34A2			0	N.A.	0
SLC35F1		-0,159945608	0	N.A.	0
SLC35F3			0	N.A.	0
SLC35F4			0	N.A.	0
SLC35G3			0	N.A.	0
SLC35G6			0	N.A.	0
SLC36A3			0	N.A.	0
SLC39A12			0	N.A.	0
SLC46A2		0,143789501	0	N.A.	0
SLC47A2			0	N.A.	0
SLC4A5		0,070301307	0	N.A.	0
SLC5A10			0	N.A.	0

SLC5A11		-0,102180935	0	N.A.	0
SLC5A4			0	N.A.	0
SLC5A5			0	N.A.	0
SLC5A7			0	N.A.	0
SLC5A8		0,035672988	0	N.A.	0
SLC6A1		0,312425052	0	N.A.	0
SLC6A11			0	N.A.	0
SLC6A13			0	N.A.	0
SLC6A17			0	N.A.	0
SLC6A2			0	N.A.	0
SLC6A3			0	N.A.	0
SLC7A3			0	N.A.	0
SLC8A1-AS1			0	N.A.	0
SLC8A3			0	N.A.	0
SLC9A4		0,238671282	0	N.A.	0
SLC9A9		-0,245427736	0	N.A.	0
SLCO1A2		-0,002060108	0	N.A.	0
SLCO1B7		0,09102857	0	N.A.	0
SLCO1C1			0	N.A.	0
SLCO4C1		-0,112667106	0	N.A.	0
SLFN12		-0,116348445	0	N.A.	0
SLFN12L		-0,088758096	0	N.A.	0
SLIT2		0,063174968	0	N.A.	0
SLIT3		-0,020100757	0	N.A.	0
SLITRK1			0	N.A.	0
SLITRK3			0	N.A.	0
SLITRK4			0	N.A.	0
SMAD1-AS1		0,253088171	0	N.A.	0
SMC1B		0,021220447	0	N.A.	0
SMCO3			0	N.A.	0
SMCR2		0,016520309	0	N.A.	0
SMIM17			0	N.A.	0
SMIM18			0	N.A.	0

SMIM2			0	N.A.	0
SMLR1		0,238271556	0	N.A.	0
SNAP25-AS1			0	N.A.	0
SNAP91			0	N.A.	0
SNCB		0,373156579	0	N.A.	0
SNHG24			0	N.A.	0
SNTG2			0	N.A.	0
SNX31		0,068107124	0	N.A.	0
SOAT2		0,043898844	0	N.A.	0
SOGA3			0	N.A.	0
SOHLH1			0	N.A.	0
SOHLH2			0	N.A.	0
SORCS1			0	N.A.	0
SORCS2			0	N.A.	0
SORCS3			0	N.A.	0
SOST			0	N.A.	0
SOX11		0,120081917	0	N.A.	0
SOX21			0	N.A.	0
SOX21-AS1			0	N.A.	0
SP7			0	N.A.	0
SPACA6P-AS		0,167970965	0	N.A.	0
SPAG17		0,175991341	0	N.A.	0
SPAG6			0	N.A.	0
SPANXN3		-0,240700833	0	N.A.	0
SPATA21		-0,316757176	0	N.A.	0
SPATA22			0	N.A.	0
SPATA25		-0,020118889	0	N.A.	0
SPATA3-AS1			0	N.A.	0
SPATA32		-0,060300726	0	N.A.	0
SPATA4			0	N.A.	0
SPATA42			0	N.A.	0
SPATA45			0	N.A.	0
SPATA8			0	N.A.	0

SPATC1			0	N.A.	0
SPECC1L-ADORA2A			0	N.A.	0
SPEM1			0	N.A.	0
SPG20-AS1			0	N.A.	0
SPHKAP			0	N.A.	0
SPIC			0	N.A.	0
SPIN4-AS1		0,119636986	0	N.A.	0
SPINK13		0,014204854	0	N.A.	0
SPOCK3			0	N.A.	0
SPP2			0	N.A.	0
SPRN			0	N.A.	0
SPRR2F			0	N.A.	0
SPSB4			0	N.A.	0
SPTA1			0	N.A.	0
SPX			0	N.A.	0
SRD5A2		0,292571474	0	N.A.	0
SRGAP2-AS1			0	N.A.	0
SRGAP3-AS4			0	N.A.	0
SRL			0	N.A.	0
SRRM4			0	N.A.	0
SSBP3-AS1			0	N.A.	0
SSPO			0	N.A.	0
SSTR3			0	N.A.	0
SSTR5		0,07310785	0	N.A.	0
SSX5		0,067041463	0	N.A.	0
ST3GAL5-AS1		-0,080170565	0	N.A.	0
ST8SIA2			0	N.A.	0
ST8SIA5			0	N.A.	0
STAB2		0,264286056	0	N.A.	0
STAC			0	N.A.	0
STAR		0,027369755	0	N.A.	0
STARD6		0,176752677	0	N.A.	0
STAU2-AS1		0,016298992	0	N.A.	0

STEAP1B			0	N.A.	0
STEAP3-AS1		0,096295576	0	N.A.	0
STK32B			0	N.A.	0
STMN2		-0,110127447	0	N.A.	0
STON1-GTF2A1L			0	N.A.	0
STOX2		-0,146865157	0	N.A.	0
STPG2		-0,023499093	0	N.A.	0
STT3A-AS1		-0,073931452	0	N.A.	0
STXBP5L		0,033170905	0	N.A.	0
SUCNR1			0	N.A.	0
SULT1C3		-0,108009806	0	N.A.	0
SUSD5			0	N.A.	0
SV2B			0	N.A.	0
SVEP1		-0,175273273	0	N.A.	0
SYCE1			0	N.A.	0
SYCP2L		0,222525733	0	N.A.	0
SYNDIG1			0	N.A.	0
SYNDIG1L		0,294969809	0	N.A.	0
SYNE1-AS1			0	N.A.	0
SYNPO2		0,21227799	0	N.A.	0
SYP-AS1		-0,003948478	0	N.A.	0
SYPL2		0,188762453	0	N.A.	0
SYS1-DBNDD2		-0,356418153	0	N.A.	0
SYT10			0	N.A.	0
SYT14			0	N.A.	0
SYT16			0	N.A.	0
SYT2		-0,260957727	0	N.A.	0
SYT3			0	N.A.	0
SYT4			0	N.A.	0
SYT6			0	N.A.	0
SYT9			0	N.A.	0
SZT2-AS1		0,342555136	0	N.A.	0
T			0	N.A.	0

TAB3-AS1			0	N.A.	0
TAB3-AS2			0	N.A.	0
TAC4			0	N.A.	0
TACR1			0	N.A.	0
TAF1L			0	N.A.	0
TARID			0	N.A.	0
TAS2R10		-0,012313719	0	N.A.	0
TAS2R13			0	N.A.	0
TAS2R19			0	N.A.	0
TAS2R20			0	N.A.	0
TAS2R31		-0,037004223	0	N.A.	0
TAS2R43		0,218441627	0	N.A.	0
TAS2R46			0	N.A.	0
TAS2R5		-0,025275429	0	N.A.	0
TAT			0	N.A.	0
TBC1D29		0,207348597	0	N.A.	0
TBC1D3C		0,394769127	0	N.A.	0
TBC1D3P1-DHX40P1		-0,13735241	0	N.A.	0
TBL1XR1-AS1		0,156038902	0	N.A.	0
TBL1Y		-0,186192533	0	N.A.	0
TBPL2			0	N.A.	0
TBX4		0,240580767	0	N.A.	0
TBX5			0	N.A.	0
TBX5-AS1			0	N.A.	0
TCEAL2		0,217722414	0	N.A.	0
TCEAL3-AS1		0,033784266	0	N.A.	0
TCEAL5			0	N.A.	0
TCEAL6			0	N.A.	0
TCERG1L			0	N.A.	0
TCERG1L-AS1			0	N.A.	0
TCF15			0	N.A.	0
TCF23			0	N.A.	0
TCL1B			0	N.A.	0

TCP10		-0,165555393	0	N.A.	0
TDRD1			0	N.A.	0
TDRD10			0	N.A.	0
TDRD12			0	N.A.	0
TDRD15			0	N.A.	0
TDRD9			0	N.A.	0
TECTA		0,005882012	0	N.A.	0
TEK		-0,140628863	0	N.A.	0
TEN1		-0,107750357	0	N.A.	0
TENM1			0	N.A.	0
TENM2			0	N.A.	0
TENM3		0,255547056	0	N.A.	0
TENM4			0	N.A.	0
TEPP			0	N.A.	0
TESC-AS1			0	N.A.	0
TET2-AS1			0	N.A.	0
TEX19		-0,087085477	0	N.A.	0
TEX26		-0,025234084	0	N.A.	0
TEX26-AS1			0	N.A.	0
TEX28			0	N.A.	0
TEX35			0	N.A.	0
TEX38			0	N.A.	0
TEX41			0	N.A.	0
TEX43		0,031578778	0	N.A.	0
TF		0,330854972	0	N.A.	0
TGFB2-AS1			0	N.A.	0
TGM5			0	N.A.	0
TGM7			0	N.A.	0
THEGL			0	N.A.	0
THEM5			0	N.A.	0
THPO			0	N.A.	0
THRIL			0	N.A.	0
THSD7B			0	N.A.	0

TICAM2		0,265803224	0	N.A.	0
TIFAB			0	N.A.	0
TIMD4			0	N.A.	0
TISP43			0	N.A.	0
TKTL2		0,100954369	0	N.A.	0
TLL1			0	N.A.	0
TLR7		-0,027339268	0	N.A.	0
TLR8			0	N.A.	0
TLR8-AS1			0	N.A.	0
TLX2		0,383735275	0	N.A.	0
TLX3		-0,084677314	0	N.A.	0
TM4SF19		-0,021493375	0	N.A.	0
TM4SF19-TCTEX1D2		0,212035923	0	N.A.	0
TMCO2			0	N.A.	0
TMED7-TICAM2		0,085400064	0	N.A.	0
TMEFF2			0	N.A.	0
TMEM100			0	N.A.	0
TMEM108			0	N.A.	0
TMEM114			0	N.A.	0
TMEM132E			0	N.A.	0
TMEM155			0	N.A.	0
TMEM169		-0,211175551	0	N.A.	0
TMEM190		0,093704124	0	N.A.	0
TMEM200C			0	N.A.	0
TMEM212			0	N.A.	0
TMEM215			0	N.A.	0
TMEM229A			0	N.A.	0
TMEM233		-0,073352714	0	N.A.	0
TMEM244			0	N.A.	0
TMEM246-AS1			0	N.A.	0
TMEM247			0	N.A.	0
TMEM255A		0,315932116	0	N.A.	0
TMEM26			0	N.A.	0

TMEM26-AS1		0	N.A.	0
TMEM262	0,211993442	0	N.A.	0
TMEM5-AS1		0	N.A.	0
TMEM52B		0	N.A.	0
TMEM72-AS1		0	N.A.	0
TMEM78	-0,083023222	0	N.A.	0
TMEM8C		0	N.A.	0
TMIGD1	0,272838829	0	N.A.	0
TMLHE-AS1	-0,031426129	0	N.A.	0
TMPRSS11A		0	N.A.	0
TMPRSS11B		0	N.A.	0
TMPRSS12		0	N.A.	0
TMX2-CTNND1		0	N.A.	0
TNFAIP8L2	0,151392207	0	N.A.	0
TNFRSF8		0	N.A.	0
TNFSF18		0	N.A.	0
TNNT3		0	N.A.	0
TNR		0	N.A.	0
TP53TG3D		0	N.A.	0
TP63		0	N.A.	0
TP73-AS1		0	N.A.	0
TPBGL		0	N.A.	0
TPH2	-0,001750179	0	N.A.	0
TPPP2	-0,188003524	0	N.A.	0
TPRG1-AS1	0,009364661	0	N.A.	0
TPSD1		0	N.A.	0
TRAPPC3L	0,314760534	0	N.A.	0
TRAV1-1		0	N.A.	0
TRAV1-2		0	N.A.	0
TRAV10		0	N.A.	0
TRAV11		0	N.A.	0
TRAV12-1		0	N.A.	0
TRAV12-2	-0,088758096	0	N.A.	0

TRAV12-3			0	N.A.	0
TRAV13-1			0	N.A.	0
TRAV13-2		-0,081466483	0	N.A.	0
TRAV14DV4			0	N.A.	0
TRAV16			0	N.A.	0
TRAV17			0	N.A.	0
TRAV18			0	N.A.	0
TRAV19			0	N.A.	0
TRAV2			0	N.A.	0
TRAV20			0	N.A.	0
TRAV21			0	N.A.	0
TRAV22			0	N.A.	0
TRAV23DV6			0	N.A.	0
TRAV24			0	N.A.	0
TRAV25			0	N.A.	0
TRAV26-1			0	N.A.	0
TRAV26-2			0	N.A.	0
TRAV27			0	N.A.	0
TRAV29DV5			0	N.A.	0
TRAV3			0	N.A.	0
TRAV30		-0,087163387	0	N.A.	0
TRAV33		0,201319142	0	N.A.	0
TRAV34			0	N.A.	0
TRAV35			0	N.A.	0
TRAV36DV7		-0,225163244	0	N.A.	0
TRAV38-1		0,136022464	0	N.A.	0
TRAV38-2DV8		0,152179561	0	N.A.	0
TRAV4			0	N.A.	0
TRAV40			0	N.A.	0
TRAV41		0,243316397	0	N.A.	0
TRAV5			0	N.A.	0
TRAV6			0	N.A.	0
TRAV8-1			0	N.A.	0

TRAV8-2			0	N.A.	0
TRAV8-3			0	N.A.	0
TRAV8-4			0	N.A.	0
TRAV8-5			0	N.A.	0
TRAV8-6			0	N.A.	0
TRAV9-2			0	N.A.	0
TRBV10-1			0	N.A.	0
TRBV10-3		-0,088758096	0	N.A.	0
TRBV11-2			0	N.A.	0
TRBV11-3			0	N.A.	0
TRBV12-3			0	N.A.	0
TRBV12-4			0	N.A.	0
TRBV13			0	N.A.	0
TRBV14			0	N.A.	0
TRBV15			0	N.A.	0
TRBV16			0	N.A.	0
TRBV18			0	N.A.	0
TRBV19			0	N.A.	0
TRBV2			0	N.A.	0
TRBV20-1		-0,029872433	0	N.A.	0
TRBV21-1			0	N.A.	0
TRBV23-1			0	N.A.	0
TRBV24-1			0	N.A.	0
TRBV27			0	N.A.	0
TRBV28			0	N.A.	0
TRBV29-1			0	N.A.	0
TRBV29OR9-2			0	N.A.	0
TRBV3-1			0	N.A.	0
TRBV30			0	N.A.	0
TRBV4-1			0	N.A.	0
TRBV4-2			0	N.A.	0
TRBV5-1			0	N.A.	0
TRBV5-3			0	N.A.	0

TRBV5-4			0	N.A.	0
TRBV5-6			0	N.A.	0
TRBV5-7			0	N.A.	0
TRBV6-1			0	N.A.	0
TRBV6-5			0	N.A.	0
TRBV6-8			0	N.A.	0
TRBV7-3		0,058015713	0	N.A.	0
TRBV7-4			0	N.A.	0
TRBV7-6			0	N.A.	0
TRBV7-7			0	N.A.	0
TRBV7-9			0	N.A.	0
TRBV9			0	N.A.	0
TRBVB			0	N.A.	0
TRDN		0,272093854	0	N.A.	0
TRDV1			0	N.A.	0
TRDV2			0	N.A.	0
TRDV3			0	N.A.	0
TREML1			0	N.A.	0
TREML4			0	N.A.	0
TRERNA1		0,142027372	0	N.A.	0
TREX2		-0,102934901	0	N.A.	0
TRG-AS1			0	N.A.	0
TRGV11			0	N.A.	0
TRGV2			0	N.A.	0
TRGV3			0	N.A.	0
TRGV4			0	N.A.	0
TRGV6			0	N.A.	0
TRGV7			0	N.A.	0
TRGV8			0	N.A.	0
TRGV9			0	N.A.	0
TRH			0	N.A.	0
TRIM17			0	N.A.	0
TRIM34		0,134810663	0	N.A.	0

TRIM50			0	N.A.	0
TRIM55			0	N.A.	0
TRIM6			0	N.A.	0
TRIM61		-0,249101905	0	N.A.	0
TRIM63			0	N.A.	0
TRIM67			0	N.A.	0
TRIM71			0	N.A.	0
TRIM74		0,141239978	0	N.A.	0
TRIML2			0	N.A.	0
TRO			0	N.A.	0
TRPC3			0	N.A.	0
TRPC5OS		0,285224053	0	N.A.	0
TRPC6			0	N.A.	0
TRPM1			0	N.A.	0
TRPM8		0,03119994	0	N.A.	0
TRPV5		0,164490215	0	N.A.	0
TSGA10IP			0	N.A.	0
TSGA13			0	N.A.	0
TSHR		-0,236901237	0	N.A.	0
TSKS			0	N.A.	0
TSPAN11			0	N.A.	0
TSPAN16			0	N.A.	0
TSPAN19		0,271802512	0	N.A.	0
TSSK1B			0	N.A.	0
TSSK4		0,078334459	0	N.A.	0
TTC23L		-0,032524591	0	N.A.	0
TTC24			0	N.A.	0
TTLL10-AS1			0	N.A.	0
TTLL11-IT1		-0,071246	0	N.A.	0
TTLL9		0,285607377	0	N.A.	0
TTTY10			0	N.A.	0
TUB		-0,097966469	0	N.A.	0
TUBA3D			0	N.A.	0

TUBA4B			0	N.A.	0
TUBB8		0,301868235	0	N.A.	0
TULP1			0	N.A.	0
TULP2			0	N.A.	0
TUNAR			0	N.A.	0
TUSC5			0	N.A.	0
TXNDC2			0	N.A.	0
U51244.2		-0,021494531	0	N.A.	0
U52111.14		-0,09943123	0	N.A.	0
U82695.9			0	N.A.	0
U91324.1			0	N.A.	0
U91328.21			0	N.A.	0
U91328.22		0,075381666	0	N.A.	0
UBE2E1-AS1		-0,120898451	0	N.A.	0
UBE2E2-AS1			0	N.A.	0
UBE2Q1-AS1			0	N.A.	0
UBE2Q2L			0	N.A.	0
UBE2U			0	N.A.	0
UCP1			0	N.A.	0
UFL1-AS1		-0,016092559	0	N.A.	0
UG0898H09		0,001658786	0	N.A.	0
UGT1A10		-0,042508007	0	N.A.	0
UGT1A6			0	N.A.	0
UGT1A7		0,276829026	0	N.A.	0
UGT1A8		-0,064556059	0	N.A.	0
UGT3A2		-0,099084565	0	N.A.	0
UNC13C		-0,057449403	0	N.A.	0
UNC45B			0	N.A.	0
UNC5C			0	N.A.	0
UNC5D			0	N.A.	0
UNC80			0	N.A.	0
UPB1			0	N.A.	0
UPK1A			0	N.A.	0

UPK1A-AS1			0	N.A.	0
USH2A		0,014722007	0	N.A.	0
USP12-AS1			0	N.A.	0
USP12-AS2			0	N.A.	0
USP29			0	N.A.	0
USP50			0	N.A.	0
UST		-0,061197777	0	N.A.	0
UTF1			0	N.A.	0
UTS2B		-0,154530481	0	N.A.	0
VAC14-AS1		0,275972871	0	N.A.	0
VAT1L			0	N.A.	0
VAV3-AS1			0	N.A.	0
VAX2			0	N.A.	0
VCX3B		-0,094981875	0	N.A.	0
VEGFC		-0,021037317	0	N.A.	0
VGLL2			0	N.A.	0
VIP			0	N.A.	0
VIPR1-AS1		0,002728852	0	N.A.	0
VIT		0,192628106	0	N.A.	0
VN1R1			0	N.A.	0
VN1R2		-0,09118431	0	N.A.	0
VPREB1			0	N.A.	0
VPS13A-AS1			0	N.A.	0
VRTN		0,014333731	0	N.A.	0
VSTM1			0	N.A.	0
VSTM2A		-0,009828198	0	N.A.	0
VWA3B		-0,297876844	0	N.A.	0
VWA5B1		0,063417205	0	N.A.	0
VWA8-AS1		-0,153404062	0	N.A.	0
VWC2			0	N.A.	0
WARS2-IT1			0	N.A.	0
WASF3-AS1			0	N.A.	0
WASIR1			0	N.A.	0

WDR17			0	N.A.	0
WDR38		0,046907151	0	N.A.	0
WDR49			0	N.A.	0
WDR64		-0,025057886	0	N.A.	0
WDR86			0	N.A.	0
WDR88			0	N.A.	0
WEE2			0	N.A.	0
WFDC10A		0,259901402	0	N.A.	0
WFDC11		0,093276329	0	N.A.	0
WFDC6			0	N.A.	0
WFDC9		-0,081836753	0	N.A.	0
WFIKKN2		-0,025234084	0	N.A.	0
WI2-1959D15.1			0	N.A.	0
WI2-80269A6.1			0	N.A.	0
WNK3			0	N.A.	0
WNT1			0	N.A.	0
WNT16			0	N.A.	0
WNT2		0,038478195	0	N.A.	0
WNT3A			0	N.A.	0
WNT5A-AS1			0	N.A.	0
WNT9B			0	N.A.	0
WSCD2			0	N.A.	0
WT1-AS			0	N.A.	0
WWC3-AS1			0	N.A.	0
XAGE3		-0,220082512	0	N.A.	0
XIAP-AS1			0	N.A.	0
XIRP1		0,196335573	0	N.A.	0
XIRP2		0,075400901	0	N.A.	0
XX-C00717C00720L.1			0	N.A.	0
XX-C283C717.1			0	N.A.	0
XX-CR54.1			0	N.A.	0
XX-FW83128A1.2		-0,004232293	0	N.A.	0
XXbac-B33L19.12		-0,231788321	0	N.A.	0

XXbac-B33L19.4			0	N.A.	0
XXbac-B444P24.8		0,000112058	0	N.A.	0
XXbac-BPG13B8.10			0	N.A.	0
XXbac-BPG181M17.6			0	N.A.	0
XXbac-BPG249D20.9		0,114314435	0	N.A.	0
XXbac-BPGBPG55C20.3			0	N.A.	0
XXyac-YM21GA2.7			0	N.A.	0
XXyac-YR29IB3.1			0	N.A.	0
XXyac-YX155B6.5		0,284263809	0	N.A.	0
XXYLT1-AS1			0	N.A.	0
YEATS2-AS1		0,082587823	0	N.A.	0
YIPF7			0	N.A.	0
Z69666.2		-0,119640818	0	N.A.	0
Z83001.1			0	N.A.	0
Z99756.1			0	N.A.	0
ZAR1			0	N.A.	0
ZBBX		-0,16424845	0	N.A.	0
ZBTB12		0,062238835	0	N.A.	0
ZBTB16			0	N.A.	0
ZBTB20-AS1			0	N.A.	0
ZBTB20-AS3			0	N.A.	0
ZBTB8B			0	N.A.	0
ZCCHC5			0	N.A.	0
ZDHHC15			0	N.A.	0
ZDHHC19			0	N.A.	0
ZFHX4-AS1			0	N.A.	0
ZFP42			0	N.A.	0
ZFP92			0	N.A.	0
ZFR2			0	N.A.	0
ZFY-AS1		0,383735275	0	N.A.	0
ZIC4			0	N.A.	0
ZIK1		-0,0220966	0	N.A.	0
ZIM2		-0,056663581	0	N.A.	0

ZIM2-AS1			0	N.A.	0
ZIM3			0	N.A.	0
ZMAT4			0	N.A.	0
ZMYM4-AS1		-0,16210987	0	N.A.	0
ZMYM6NB		0,071583608	0	N.A.	0
ZNF132		-0,225163244	0	N.A.	0
ZNF135			0	N.A.	0
ZNF154			0	N.A.	0
ZNF157			0	N.A.	0
ZNF197-AS1			0	N.A.	0
ZNF208			0	N.A.	0
ZNF221		0,245616325	0	N.A.	0
ZNF229		-0,086157723	0	N.A.	0
ZNF295-AS1		0,100954369	0	N.A.	0
ZNF30-AS1			0	N.A.	0
ZNF32-AS1		-0,045646278	0	N.A.	0
ZNF32-AS2		-0,094078587	0	N.A.	0
ZNF350-AS1			0	N.A.	0
ZNF354C			0	N.A.	0
ZNF366		-0,097593405	0	N.A.	0
ZNF382		-0,092677992	0	N.A.	0
ZNF385B			0	N.A.	0
ZNF418			0	N.A.	0
ZNF474			0	N.A.	0
ZNF536			0	N.A.	0
ZNF559-ZNF177			0	N.A.	0
ZNF560			0	N.A.	0
ZNF582-AS1			0	N.A.	0
ZNF625-ZNF20			0	N.A.	0
ZNF649-AS1			0	N.A.	0
ZNF667		-0,025850064	0	N.A.	0
ZNF667-AS1		0,029062871	0	N.A.	0
ZNF676		-0,216067753	0	N.A.	0

ZNF705A			0	N.A.	0
ZNF705G		0,009265806	0	N.A.	0
ZNF716		0,337693264	0	N.A.	0
ZNF727		-0,234497338	0	N.A.	0
ZNF732		-0,086310764	0	N.A.	0
ZNF781			0	N.A.	0
ZNF790-AS1		0,081138471	0	N.A.	0
ZNF804A		-0,154574615	0	N.A.	0
ZNF812			0	N.A.	0
ZNF829			0	N.A.	0
ZNF831		0,136022464	0	N.A.	0
ZNF835			0	N.A.	0
ZNF888		-0,043498837	0	N.A.	0
ZNF98			0	N.A.	0
ZNF99			0	N.A.	0
ZP1		0,14117977	0	N.A.	0
ZPBP			0	N.A.	0
ZPBP2			0	N.A.	0
ZRANB2-AS1			0	N.A.	0
ZSCAN1			0	N.A.	0
ZSCAN23			0	N.A.	0
ZSCAN4		-0,057605904	0	N.A.	0
ZSCAN5B			0	N.A.	0
ZSWIM2			0	N.A.	0
ZYG11A			0	N.A.	0

Supplementary Table 3. Correlation of basal pancreatic genes with Ep

gene	Mean_SMC_Cohort	Mean_KUL_Cohort
EMP1	0,869356026	0,853861678
ITGA3	0,76369857	0,922465825
AHNAK2	0,720598585	0,794526483
SLC2A1	0,711780311	0,850531314
KRT19	0,662218606	0,839990568
KRT7	0,62052748	0,49344304
CAST	0,603443421	0,72383654
DHRS9	0,577439569	0,62814721
ANXA1	0,577071676	0,335263196
F3	0,504995258	0,75042491
KRT15	0,504451152	0,432809164
S100A2	0,498329069	0,354499252
KRT17	0,493230731	0,568491862
SCEL	0,473439109	0,631357019
SPRR1B	0,469394784	0,452490938
ANXA2	0,448203252	0,452065675
SEMA3C	0,436193003	0,309821044
LY6D	0,406274892	0,336809208
CAP1	0,369989178	0,504539576
KRT6A	0,348487892	0,538086096
CAV1	0,333017431	0,000136606
GPR87	0,332369568	0,260654866
CD109	0,33109085	0,521307369
CST6	0,328104392	0,415487183
FAM83A	0,297027805	0,110919615
FLNA	0,2863217	0,281339764
GJB5	0,163833734	0,570084996
KRT13	0,154437221	0,124386989
DDIT4	0,153804183	0,625657038
PTPN13	0,141352241	0,129005636
KRT5	0,136223892	
COL7A1	0,041286735	0,277151037
PKP1	0,025231937	0,305685037
FRMD6	0,023146377	0,143794203
SLITRK6	0,014825454	0,02057934
AQP3	-0,005496947	0,03482334
MTSS1	-0,02568104	-0,052508472
CSTA	-0,047702527	-0,245026463
MT2A	-0,066201053	-0,20710425
PVRL1	-0,0977537	-0,071148221
YBX3	-0,184074128	-0,025807553
CD44	-0,18524605	-0,321441415
GAPDH	-0,324296261	-0,275150248
HSP90AA1	-0,615830604	-0,088997784
LDHB	-0,702396149	-0,6815864

Descripives of clinical info by dataset

		TCGA (538)	GSE38832 (122)	GSE44076 (98)	GSE33113 (90)	GSE14333 (290)	GSE39582 (562)	GSE37892 (130)	TOTAL (1830)
Gender	Male	280 (52.0%)		71 (72.4%)	42 (46.7%)	164 (56.6%)	307 (54.6%)	69 (53.1%)	933 (54.6%)
	Female	258 (48.0%)		27 (27.6%)	48 (53.3%)	126 (43.4%)	255 (45.4%)	61 (46.9%)	775 (45.4%)
	Miss.	0 (0.0%)	122 (100.0%)	0 (0.0%)	0 (0.0%)	0 (0.0%)	0 (0.0%)	0 (0.0%)	122 (6.7%)
Age at diagnosis	Mean (Sd)	65.36 (12.87)		70.54 (9.02)	70.39 (12.95)	65.96 (12.52)	66.84 (13.28)	68.25 (12.69)	66.73 (12.83)
	Miss.	0 (0.0%)	122 (100.0%)	0 (0.0%)	1 (1.1%)	0 (0.0%)	1 (0.2%)	0 (0.0%)	124 (6.8%)
Tumor location	Right	191 (41.9%)		38 (38.8%)		125 (43.7%)	222 (39.5%)	57 (44.2%)	633 (41.3%)
	Left	117 (25.7%)		60 (61.2%)		122 (42.7%)	340 (60.5%)	72 (55.8%)	711 (46.4%)
	Rectum	148 (32.5%)		0 (0.0%)		39 (13.6%)	0 (0.0%)	0 (0.0%)	187 (12.2%)
	Miss.	82 (15.2%)	122 (100.0%)	0 (0.0%)	90 (100.0%)	4 (1.4%)	0 (0.0%)	1 (0.8%)	299 (16.3%)
Stage	Stage I	93 (17.9%)	18 (14.8%)	0 (0.0%)	0 (0.0%)	44 (15.2%)	33 (5.9%)	0 (0.0%)	188 (10.4%)
	Stage II	198 (38.1%)	35 (28.7%)	98 (100.0%)	90 (100.0%)	94 (32.4%)	264 (47.0%)	73 (56.2%)	852 (47.0%)
	Stage III	157 (30.2%)	39 (32.0%)	0 (0.0%)	0 (0.0%)	91 (31.4%)	205 (36.5%)	57 (43.8%)	549 (30.3%)
	Stage IV	72 (13.8%)	30 (24.6%)	0 (0.0%)	0 (0.0%)	61 (21.0%)	60 (10.7%)	0 (0.0%)	223 (12.3%)
	Miss.	18 (3.3%)	0 (0.0%)	0 (0.0%)	0 (0.0%)	0 (0.0%)	0 (0.0%)	0 (0.0%)	18 (1.0%)
MSI status	MSS	462 (85.9%)	99 (81.1%)	98 (100.0%)	61 (67.8%)	240 (82.8%)	483 (85.9%)	130 (100.0%)	1573 (86.0%)
	MSI	76 (14.1%)	23 (18.9%)	0 (0.0%)	29 (32.2%)	50 (17.2%)	79 (14.1%)	0 (0.0%)	257 (14.0%)
Time of follow-up (years, not events)	Median (MAD)	1.63 (1.28)	3.34 (2.44)		3.64 (1.62)	3.96 (2.22)	5.33 (2.72)	4.17 (1.45)	3.83 (2.85)
Recurrence	No recurrence	214 (53.4%)	89 (96.7%)		71 (79.8%)	176 (77.9%)	353 (71.6%)	93 (71.5%)	996 (69.6%)
	Recurrence	187 (46.6%)	3 (3.3%)		18 (20.2%)	50 (22.1%)	140 (28.4%)	37 (28.5%)	435 (30.4%)
	Miss.	137 (25.5%)	30 (24.6%)	98 (100.0%)	1 (1.1%)	64 (22.1%)	69 (12.3%)	0 (0.0%)	399 (21.8%)

Supplemtary Table 4. Functional enrichment of cell population across metastatic relapse

	HRCs Krt20-	HRCs	Krt20+	Lgr5+ Ki67+	Lgr5+ Ki67-	Ki67+
HALLMARK_HYPOXIA	30,77551	28,51489	24,49159	0,00000953	0,365992236	0,00000013
HALLMARK_INTERFERON_GAMMA_RESPON	27,24515	0,11966	2,392422	1,96519	7,252677237	10,05199
HALLMARK_INTERFERON_ALPHA_RESPONS	27,08662	0,401881	3,995807	0,319878	2,933395069	8,80403
HALLMARK_PROTEIN_SECRETION	22,67438	15,56462	10,9052	3,123357	4,558567337	0,772295
HALLMARK_P53_PATHWAY	18,93139	25,27526	17,88514	1,778619	2,870020336	0,645644
HALLMARK_TNFA_SIGNALING_VIA_NFKB	16,59663	16,20497	13,53301	1,703204	3,2177386	0,00000013
HALLMARK_MTORC1_SIGNALING	14,88045	6,63137	4,64339	21,25053	7,277966985	24,52802
HALLMARK_HEME_METABOLISM	11,34406	16,20497	4,674632	5,592432	5,311753414	0,645644
HALLMARK_PI3K_AKT_MTOR_SIGNALING	11,27813	6,387765	1,453124	7,845002	2,701581061	5,015559
HALLMARK_APOPTOSIS	10,80282	9,180745	11,42646	1,4938	0,987132255	0,570294
HALLMARK_GLYCOLYSIS	9,019813	4,354255	14,36281	7,590663	6,126240202	4,014588
HALLMARK_CHOLESTEROL_HOMEOSTASIS	8,625716	4,585903	6,37011	2,994341	2,526837643	4,361533
HALLMARK_ESTROGEN_RESPONSE_EARLY	8,557877	10,28148	12,46381	5,03259	4,740939108	0,098036
HALLMARK_MITOTIC_SPINDLE	7,668918	7,230265	3,552531	20,55275	8,059405272	17,07332
HALLMARK_IL6_JAK_STAT3_SIGNALING	7,668918	3,331595	2,237264	0,535035	1,959874483	0,00000013
HALLMARK_TGF_BETA_SIGNALING	7,042241	16,20497	11,69304	0,660831	1,353016091	0,09087
HALLMARK_IL2_STAT5_SIGNALING	6,645344	5,213776	9,269553	0,98998	1,485501807	0,922308
HALLMARK_COMPLEMENT	6,368495	3,695182	6,340621	0,606537	0,922220161	0,00000013
HALLMARK_UV_RESPONSE_UP	5,505433	5,397965	2,934072	10,0022	3,877119378	6,020873
HALLMARK_ADIPOGENESIS	5,249682	2,868029	5,259809	17,30318	24,36033264	10,76306
HALLMARK_INFLAMMATORY_RESPONSE	4,4299	1,400033	1,684921	0,14728	0,392267326	0,00000013
HALLMARK_PEROXISOME	4,055169	0,366073	3,756669	5,055622	3,205736293	2,362712
HALLMARK_ESTROGEN_RESPONSE_LATE	3,951758	5,748746	6,813605	5,728767	1,618858153	0,911483
HALLMARK_UV_RESPONSE_DN	3,903646	8,424758	6,591048	1,122393	2,785328073	0,00000013
HALLMARK_APICAL_JUNCTION	3,533321	3,986106	8,154383	0,054254	1,027041775	0,00000013
HALLMARK_ANDROGEN_RESPONSE	2,995932	7,787059	3,756669	3,346517	2,526837643	1,881757
HALLMARK_ALLOGRAFT_REJECTION	2,685821	0,04328	0,3748	0,701148	1,197507354	0,563962
HALLMARK_UNFOLDED_PROTEIN_RESPONS	2,576666	4,792946	0,227096	25,0262	11,18767232	15,61567
HALLMARK_MYOGENESIS	2,456178	4,463978	3,076006	0,001661	0,052043943	0,00000013
HALLMARK_REACTIVE_OXIGEN_SPECIES_P	2,423762	0,589527	4,064399	3,307276	2,872328616	4,006558
HALLMARK_DNA_REPAIR	2,368222	2,946959	5,260527	12,23167	5,122950873	29,83106
HALLMARK_KRAS_SIGNALING_UP	2,242744	3,618786	6,961996	0,070233	0,309559477	0,00000013

HALLMARK_NOTCH_SIGNALING	2,142473	1,642963	3,579575	1,246696	1,412167843	0,098036
HALLMARK_XENOBIOTIC_METABOLISM	2,102266	2,406661	5,479666	1,793864	2,084393126	0,019761
HALLMARK_COAGULATION	2,046873	3,041721	4,929165	0,001661	0,194433174	0,00000013
HALLMARK_FATTY_ACID_METABOLISM	1,902755	0,208192	4,326749	13,50998	11,11401738	3,659594
HALLMARK_BILE_ACID_METABOLISM	1,343289	0,372908	3,141794	0,918788	1,795154356	0,00000013
HALLMARK_APICAL_SURFACE	0,85983	1,172019	0,361201	1,131127	0,553831016	0,123749
HALLMARK_EPITHELIAL_MESENCHYMAL_TF	0,636127	2,83392	4,315474	0,00000953	0,000240402	0,00000013
HALLMARK_OXIDATIVE_PHOSPHORYLATION	0,37211	0,407994	2,850124	35,31482	54,13666626	48,62552
HALLMARK_WNT_BETA_CATENIN_SIGNALIN	0,251852	0,401881	1,050288	2,507028	2,933395069	0,448749
HALLMARK_HEDGEHOG_SIGNALING	0,086565	0,18715	0,361201	0,27066	0,76134047	0,00000013
HALLMARK_ANGIOGENESIS	0,080679	1,097841	3,776017	0,054254	0,117023297	0,00000013
HALLMARK_SPERMATOGENESIS	0,001514	0,04328	0,148403	3,044301	0,434988119	2,432845
HALLMARK_PANCREAS_BETA_CELLS	0,000683	0,18715	0,361201	0,001661	0,028567473	0,00000013
HALLMARK_KRAS_SIGNALING_DN	0,000683	0,099957	0,103034	0,00000953	0,000240402	0,00000013
HALLMARK_G2M_CHECKPOINT	0,000683	0,052428	1,68E-10	69,47117	1,28069982	74,42111
HALLMARK_E2F_TARGETS	0	0,0000367	1,68E-10	96,74523	0,194433174	103,056
HALLMARK_MYC_TARGETS_V1	0	0,0000367	1,68E-10	78,9164	30,09305613	95,51818
HALLMARK_MYC_TARGETS_V2	0	0,0000367	1,68E-10	26,75196	18,55265426	24,62721

Supplementary Table 5. Genotyping primers

Gene	Site	Sequence
mEmp1	5’ specific integration PCR	F: CTAATGCTCCTGGTTCTGTCTTG
		R: AAGACGGCAATATGGTGGAAAAT
	3’ specific integration PCR	F: GCATTGTCTGAGTAGGTGTCATT
		R: AGTGTGATGAAAGCTGTTGAGAG
mLgr5	5’ specific integration PCR	F: GCACCTATTTGGTAGCTGGCTGATCC
		R: GCTACAGACGTTGTTTGTCTTCAA
	3’ specific integration PCR	F: ATGCTGGGGATGCGGTGGGCTCTAT
		R: AGAAGCATGCTCCTACTCAGAAATAA

Supplementary Table 6. Immunofluorescence protocol

Antibody	Company	Reference	Antigen retrieval	Dilution	Time inc.	Host
tdTomato	Rockland	600-401-379	Tris-EDTA	1/100	ON 4ºC	Rabbit
tdTomato	Sicgen	AB8181-200	Tris-EDTA	1/100	ON 4ºC	Goat
GFP	Abcam	ab6673	Tris-EDTA	1/200	2h RT	Goat
E-Cadherin	BD	610182	Tris-EDTA	1/300	ON 4ºC	Mouse
CD31	Abcam	ab28364	Tris-EDTA	1/300	ON 4ºC	Rabbit
KRT20	Sigma	HPA024309	Tris-EDTA	1/100	ON 4ºC	Rabbit
KRT17	Sigma	HPA000453	Tris-EDTA	1/100	ON 4ºC	Rabbit
a-SMA	Abcam	ab7817	Tris-EDTA	1/200	ON 4ºC	Mouse
CD3	ABCAM	ab16669	Tris-EDTA	1/500 + T	ON 4ºC	Rabbit
EpCAM	R&D (Bio-techne)	AF960	Tris-EDTA	1/50	ON 4ºC	Goat
LAMC2	Sigma	AMAB91098	Tris-EDTA	1/100	ON 4ºC	Mouse
Alexa Fluor 647 anti-Rabbit IgG	Thermo Fisher	A31573		1/400	1h RT	Donkey
Alexa Fluor 647 anti-Mouse IgG	Thermo Fisher	A31571		1/400	1h RT	Donkey

Supplementary Table 7. Gene signatures used in this study

All-HR	TME-HR	EpiHR	Lgr5 signature	Wnt ON signature (Morral et al. 2020)	mKi67 (Basak et al. 2014)
PABPC5	PABPC5	NGRN	BCL11B	ASCL2	CDKN1B
RBM46	RBM46	FBXL13	AXIN2	AXIN2	PIF1
TAS2R8	TAS2R8	ITGA3	LGR5	LGR5	CCNA1
IFNA5	IFNA5	SLC25A25	ASCL2	KIAA1199	GTSE1
COL1A1	COL1A1	SEMA4B	LRIG1	SP5	CEP55
LUM	LUM	MAPK3		AXIN2	KIF20B
TPT1	TPT1	RIN1		CACHD1	CENPE
ZDHHC15	ZDHHC15	PNPLA2		CST1	KIF18A
C11orf96	C11orf96	RHOD		LGR5	BUB1
VCAN	VCAN	MID1IP1		SMOC2	CDC25B
DCN	DCN	DDR1		FAM216A	GJB3
IGFBP5	IGFBP5	LAMB3		LEF1	KNSTRN
IGFBP7	IGFBP7	FLRT1		CYP4X1	CDCA3
USP44	USP44	PRSS22		SLC12A2	KIF18B
SRP14	SRP14	LAMC2		ASCL2	MELK
ANTXR1	ANTXR1	HOXA4		DPEP1	TROAP
CTSK	CTSK	FAM83A		NKD1	DDIAS
COL3A1	COL3A1	EXOC7		LRP4	CKAP2
HYAL2	HYAL2	TNFRSF21		ZNRF3	KIF20A
COL5A1	COL5A1	RIPK4		PTPRO	MIS18BP1
CALD1	CALD1	PRPH		APCDD1	CCNB2
POSTN	POSTN	KRT19		TNFRSF19	CDC20
FXR2	FXR2	COLEC10		SLC12A2	CKAP2L
SPARC	SPARC	SPINT1		SOX2	CCNB1
ADAMTS2	ADAMTS2	VGLL1		PTCH1	CRYM
THBS2	THBS2	ZNF185		TSPAN5	TPX2
COL1A2	COL1A2	GJB3		MYRIP	DLGAP5
CYFIP1	CYFIP1	PAEP		CDK6	BORA
TIMP2	TIMP2	RIMBP2		ITGA9	NEK2
PLAT	PLAT	WFDC3		H19	CDKN3
C1S	C1S	GAS6		PPP2R2C	ASPM

VAMP2	VAMP2	HTR2C	IGFL2	PSRC1
CCDC80	CCDC80	MROH6	CHI3L1	KIF23
TWF2	TWF2	CAPN12	FRY	PCSK6
FAM129B	FAM129B	FBXL14	CXCR4	OSCP1
ISLR	ISLR	MAGI2	SLC28A3	SLC28A3
CDH10	CDH10	ANXA3	SACS	NRARP
ING4	ING4	PPL	HAS2	CENPA
GPNMB	GPNMB	BAIAP2	SP8	ALPP
TSG101	TSG101	LINGO1	NKD1	ALPP
AXL	AXL	KLK7	AXIN2	ALPP
COL8A1	COL8A1	EPHA2	LOC100505730	SHCBP1
TNS1	TNS1	KRT17	AXIN2	ARHGEF39
LINC00472	LINC00472	SCGN	CDCA7	ANLN
ABHD4	ABHD4	KLK5	LOC100134361	HMMR
MXRA5	MXRA5	LAMA3	CDCA7	PARPBP
COL6A1	COL6A1	EPS8L1	CDK6	TLR1
GREM1	GREM1	FXYD4	ETS2	CDC25C
CERS5	CERS5	SNX24	SOX4	CCNF
PRRX2	PRRX2	SLC22A18	VSNL1	AURKA
NRG3	NRG3	KLK8	CADM1	PRC1
EFEMP1	EFEMP1	KCNN4	PTCH1	CENPF
NXN	NXN	RASAL1	SLC7A8	GSDMC
CTHRC1	CTHRC1	LEMD1	ZNF704	SPC25
FBN1	FBN1	KCNK3	FAM46C	KIF2C
CAV1	CAV1	RNF39	FAM43A	KIF14
C1R	C1R	HIST2H2BE	PROX1	EFCAB11
COL5A2	COL5A2	LMO7	NRXN3	PRR11
MMP2	MMP2	C1orf116	FGF18	KIF22
CRISPLD2	CRISPLD2	PSORS1C1	LYZ	GSDMC
COL12A1	COL12A1	GPRC5A	BAMBI	PLK1
CYGB	CYGB	SEZ6L2	CADM1	
TAGLN	TAGLN	C19orf33	AMACR	
MGP	MGP	GDPD3	LRIG1	

TCF4	TCF4	KLK10	NAV2
RORB	RORB	SLC20A1	TSPAN5
FSTL1	FSTL1	KLK11	KREMEN1
LOXL4	LOXL4	KLK6	ETS2
DKK3	DKK3	SCEL	HAS2
SPON2	SPON2	KCNJ16	KCNJ2
COL6A3	COL6A3	KRT80	PROX1
PDGFC	PDGFC	CAMK2N1	SLC16A10
BASP1	BASP1	KRT6A	TSPAN5
BGN	BGN	DISP2	PRSS23
TMEM51	TMEM51	SLC2A1	C21orf63
PMP22	PMP22	SCNN1A	RGMB
SEMA3D	SEMA3D	TM4SF4	PROX1
LGALS1	LGALS1	MSLN	RTKN2
NNMT	NNMT	COL17A1	FREM2
FN1	FN1	FAM155A	KCNJ2
IL1R1	IL1R1	KRT6B	OXGR1
PDGFRA	PDGFRA	PLA2G10	ABCC4
CCNDBP1	CCNDBP1	MYBPC1	SLC7A8
LRAT	LRAT	SFTA2	MME
CES5A	CES5A	SLPI	VSNL1
MMP19	MMP19	VSIG2	KCNJ8
SFRP2	SFRP2	RHBDL2	RGMB
CYR61	CYR61	SERPINB5	RGMB
SERPINF1	SERPINF1	S100A2	ZNF704
ARHGEF2	ARHGEF2	MMP7	ASB4
INHBA	INHBA	CTSE	
FIBIN	FIBIN	NXF3	
GAS7	GAS7	GSTA2	
STARD6	STARD6	GSTA1	
PHC1	PHC1	CLSTN2	
STAC	STAC	HOXB8	
GAS1	GAS1	GABRP	

SDC2	SDC2	TACSTD2
COL15A1	COL15A1	DUSP27
LOXL2	LOXL2	
ARL8A	ARL8A	
THY1	THY1	
NOTCH4	NOTCH4	
ALDH3B1	ALDH3B1	
TNC	TNC	
ASPN	ASPN	
GJA1	GJA1	
ARMCX6	ARMCX6	
SPAG7	SPAG7	
VIM	VIM	
SLC24A3	SLC24A3	
TRIM55	TRIM55	
PLAU	PLAU	
WBP2	WBP2	
TNFAIP6	TNFAIP6	
UNC5B	UNC5B	
RAB31	RAB31	
COL4A2	COL4A2	
EFEMP2	EFEMP2	
MRC2	MRC2	
CYBRD1	CYBRD1	
GNPTG	GNPTG	
DNASE1L1	DNASE1L1	
ITGBL1	ITGBL1	
CTGF	CTGF	
PALLD	PALLD	
INPP5K	INPP5K	
PDGFB	PDGFB	
IGFBP3	IGFBP3	
ANGPTL2	ANGPTL2	

FBLN1	FBLN1
PDPN	PDPN
ITGAV	ITGAV
PRKAG1	PRKAG1
AEBP1	AEBP1
SPARCL1	SPARCL1
WDFY3-AS	WDFY3-AS2
FNDC1	FNDC1
PXDN	PXDN
MCAM	MCAM
POLD4	POLD4
SERPING1	SERPING1
COL4A1	COL4A1
NFAM1	NFAM1
TMEM119	TMEM119
PRRX1	PRRX1
CLMP	CLMP
BAALC	BAALC
TMEM63B	TMEM63B
MFAP4	MFAP4
ACTA2	ACTA2
NOTCH3	NOTCH3
TWIST1	TWIST1
LHFP	LHFP
RELB	RELB
SUSD5	SUSD5
COL14A1	COL14A1
LRRN4CL	LRRN4CL
NGRN	COL5A3
COL5A3	PLD2
PLD2	SERTAD2
SERTAD2	TIMP1
TIMP1	PTPRS

PTPRS	ALDH1A3
ALDH1A3	TFPI
TFPI	STOM
STOM	EDNRA
EDNRA	CCL2
CCL2	S100A11
S100A11	A2M
A2M	COL6A2
COL6A2	IFI27L2
IFI27L2	PLXDC1
PLXDC1	OLFML2A
OLFML2A	COL18A1
COL18A1	FERMT2
FERMT2	SPHK1
SPHK1	DCBLD1
FBXL13	MYO9B
DCBLD1	TUBB6
MYO9B	ZFP92
TUBB6	SLC27A1
ZFP92	ADAMTS1
SLC27A1	FEZ2
ADAMTS1	HTRA1
FEZ2	PTGDS
HTRA1	S1PR3
PTGDS	PPP1R11
ITGA3	OLFML3
S1PR3	MXRA8
PPP1R11	EFHD2
OLFML3	SLIT2
MXRA8	MAFB
EFHD2	PCOLCE
SLIT2	LRCH4
MAFB	LAMC1

PCOLCE	FZD8
LRCH4	TIMP3
LAMC1	IL4R
FZD8	EDIL3
TIMP3	PDLIM7
IL4R	PTPN14
EDIL3	MYH10
PDLIM7	RNF167
PTPN14	ITGB5
MYH10	DRAM1
RNF167	FILIP1L
ITGB5	SLITRK3
DRAM1	KCNQ5
FILIP1L	SULF1
SLITRK3	EPAS1
KCNQ5	LAMA2
SULF1	FAP
EPAS1	FHL3
LAMA2	GEM
FAP	GPX8
FHL3	LMBRD1
GEM	EVA1B
GPX8	CHST15
LMBRD1	NID1
EVA1B	GIPC1
CHST15	WISP1-OT1 ||| WISP1
NID1	C10orf10
GIPC1	IL6ST
WISP1-OT1	TJP1
C10orf10	GPSM3
IL6ST	COL11A1
TJP1	LAMB2
GPSM3	TMEM204

COL11A1	SALL2
LAMB2	LOX
TMEM204	FSCN1
SALL2	PDLIM3
LOX	PPARD
FSCN1	MMP11
PDLIM3	GPX3
PPARD	GPR124
MMP11	LATS2
GPX3	NTM
GPR124	HEYL
LATS2	ZCCHC24
NTM	GLIPR2
HEYL	HSPG2
ZCCHC24	OBSL1
GLIPR2	FZD1
SLC25A25	PTRF
HSPG2	LOXL1
OBSL1	AP1B1
FZD1	JAM3
PTRF	KLF9
LOXL1	COL10A1
AP1B1	LAMA4
JAM3	MSN
KLF9	COX7A1
COL10A1	MFAP5
LAMA4	COLEC12
MSN	DIO2
COX7A1	MEX3B
MFAP5	HOXB4
COLEC12	SOX13
DIO2	CCDC28A
MEX3B	RAB34

HOXB4	GLIS2
SOX13	AKT3
CCDC28A	CILP
RAB34	ITIH5
GLIS2	CDH11
AKT3	FGFR1
CILP	CCDC107
ITIH5	DYSF
CDH11	NRP2
FGFR1	SFRP4
SEMA4B	OSMR
CCDC107	UNC5D
DYSF	ABTB1
NRP2	OLFML2B
SFRP4	GSN
OSMR	ATP6V0C
UNC5D	FAM198B
ABTB1	DPYSL3
OLFML2B	SNED1
GSN	ELN
ATP6V0C	RGS5
FAM198B	THBS1
DPYSL3	NDN
SNED1	ADAP2
ELN	RARRES2
RGS5	ROGDI
THBS1	CBX6
NDN	RUNX1T1
ADAP2	SSPN
RARRES2	CD34
ROGDI	NLGN4Y
CBX6	DLC1
RUNX1T1	RBMS1

SSPN	FHL1
CD34	RFTN1
NLGN4Y	SPOCK1
DLC1	CLCF1
RBMS1	SH2B1
FHL1	PFDN1
RFTN1	IKBKG
SPOCK1	DACT1
CLCF1	COPZ2
SH2B1	PDLIM4
PFDN1	MSRA
IKBKG	FLNA
DACT1	TMEM45A
COPZ2	PPP1R14A
PDLIM4	CCDC160
MSRA	BTBD19
FLNA	SNAI2
TMEM45A	UBE2E2
PPP1R14A	NRP1
CCDC160	LBH
BTBD19	PHLDB2
SNAI2	GUCY1B3
UBE2E2	APLP1
NRP1	SLC43A3
LBH	RNF111
PHLDB2	A4GALT
GUCY1B3	ZNF132
APLP1	VIPAS39
SLC43A3	SESN2
RNF111	BNC2
A4GALT	PHLDA1
ZNF132	PPAP2A
VIPAS39	ZEB1

SESN2	FAM20C
BNC2	RGS16
PHLDA1	ARMCX1
PPAP2A	CTSL
ZEB1	KCNE2
FAM20C	GNB4
RGS16	EFS
ARMCX1	UBTD1
CTSL	SHISA2
KCNE2	C15orf52
GNB4	SETBP1
EFS	FAM101B
UBTD1	CSPG4
SHISA2	CACNB3
C15orf52	LEF1
SETBP1	SVEP1
FAM101B	MED11
CSPG4	ZMAT3
CACNB3	CHSY1
LEF1	LAMB1
SVEP1	MOAP1
MED11	IFFO1
ZMAT3	MSRB3
CHSY1	FBLN5
LAMB1	BTBD10
MOAP1	TMBIM1
IFFO1	TPM2
MSRB3	CTSO
FBLN5	GGT5
BTBD10	CERCAM
TMBIM1	TSPAN4
TPM2	KANK2
CTSO	PTPLAD2

GGT5	MFAP2
CERCAM	GLT8D2
TSPAN4	CCL13
KANK2	TNFAIP2
PTPLAD2	ABCA1
MFAP2	ANXA5
GLT8D2	CTNNBIP1
CCL13	GPR176
TNFAIP2	CLIC4
ABCA1	GLIPR1
ANXA5	PER1
CTNNBIP1	ECM1
GPR176	BEST1
CLIC4	ADAM28
GLIPR1	GJC1
PER1	MRVI1
ECM1	NOP10
BEST1	CHST3
ADAM28	ELK3
GJC1	DSE
MRVI1	KIAA0430
NOP10	YIPF3
CHST3	LMO2
ELK3	SLC8A1
MAPK3	PLXDC2
DSE	SCN1B
KIAA0430	SPRED3
YIPF3	LRRC32
LMO2	MYLK
SLC8A1	SH3PXD2B
PLXDC2	IGF1
SCN1B	MEIS1
SPRED3	VCAM1

LRRC32	TUBA1A
MYLK	RGS2
SH3PXD2B	NOG
IGF1	IGSF11
MEIS1	CD248
VCAM1	PIAS1
TUBA1A	KIRREL
RGS2	TRO
NOG	CTSC
IGSF11	ADAMTSL4
CD248	ABCA8
PIAS1	CALU
KIRREL	COMP
TRO	BCL6B
CTSC	FAM3C
ADAMTSL4	CYB5R1
ABCA8	EGR3
CALU	GPX7
COMP	TCN2
BCL6B	PDGFRB
FAM3C	ATP6AP1
CYB5R1	ADAM12
EGR3	MAN1C1
GPX7	AKAP12
TCN2	BTG1
PDGFRB	GUCY1A3
ATP6AP1	JOSD1
ADAM12	VAMP8
MAN1C1	C16orf86
AKAP12	AATK
BTG1	TSC22D3
GUCY1A3	IFI16
JOSD1	MYD88

VAMP8	BCAM
C16orf86	SOBP
AATK	DSEL
TSC22D3	B3GALT4
IFI16	FBLN2
MYD88	MITF
BCAM	BCAT1
SOBP	SGCE
DSEL	DNM1
B3GALT4	TAGLN3
FBLN2	MIR143HG
MITF	C4orf3
BCAT1	NES
SGCE	PTAFR
DNM1	MAP6
TAGLN3	PPAP2B
MIR143HG	LMO4
C4orf3	AP2A2
NES	EMP1
PTAFR	CTNNA3
MAP6	FZD4
PPAP2B	BICC1
LMO4	GNAO1
AP2A2	OLFML1
EMP1	SPSB3
CTNNA3	SRPX
FZD4	DEDD2
BICC1	ATP8A2
GNAO1	RGL2
OLFML1	EMP3
SPSB3	DNAJC4
SRPX	CD68
DEDD2	MYOF

ATP8A2	ZNF410
RGL2	TRIM17
EMP3	RAB5B
DNAJC4	ACTG2
CD68	TBC1D25
MYOF	LDB2
ZNF410	ST3GAL1
TRIM17	HIC1
RAB5B	C16orf62
ACTG2	LHPP
TBC1D25	PDGFD
LDB2	RAI14
ST3GAL1	ADAM19
HIC1	DPYSL2
C16orf62	GRIPAP1
LHPP	DBN1
PDGFD	CERS2
RAI14	THBD
ADAM19	NFIC
DPYSL2	STX1A
GRIPAP1	ST5
DBN1	COL8A2
CERS2	MMP14
THBD	RHOJ
NFIC	SPG20
RIN1	GPR108
STX1A	RILP
ST5	SIM1
COL8A2	HIF1A
MMP14	NR2F1
RHOJ	ZDHHC7
SPG20	TUSC3
GPR108	UCHL1

RILP	ROR2
SIM1	LRRC15
HIF1A	ARHGEF17
NR2F1	TTC28
ZDHHC7	ARL4C
TUSC3	APCDD1L
UCHL1	PPP1R18
ROR2	TGFB1
LRRC15	FLRT2
ARHGEF17	CRMP1
TTC28	ITGA1
ARL4C	CAV2
APCDD1L	HGSNAT
PPP1R18	HEG1
TGFB1	TSHZ3
FLRT2	PTGIS
CRMP1	SH3BP5
ITGA1	ZNF428
CAV2	PTPRM
HGSNAT	LTBP1
HEG1	FAM196B
TSHZ3	FUT11
PTGIS	TMEM242
SH3BP5	CRLF1
ZNF428	DDR2
PTPRM	QKI
LTBP1	LSP1
FAM196B	ATP2B4
FUT11	PLD3
TMEM242	RSPH3
CRLF1	PCDHGC5 /// PCDHGA8 /// PCDHGA11 /// PCDHGC3
DDR2	PRELP
QKI	FRMD6

LSP1	PAMR1
ATP2B4	KCNE4
PLD3	SERPINE1
RSPH3	RBMS3
PCDHGC5	NR2F2
PRELP	TRIP4
FRMD6	MOXD1
PAMR1	NID2
KCNE4	GNG11
SERPINE1	PPIC
PNPLA2	ZSCAN18
RBMS3	DAB2
NR2F2	MXRA7
TRIP4	TCF25
MOXD1	ACVR1
NID2	WWTR1
GNG11	PLXNC1
PPIC	CCDC102B
ZSCAN18	EVC
DAB2	KIF3C
MXRA7	FOLR2
TCF25	RCN3
ACVR1	ARHGAP24
WWTR1	PIAS3
PLXNC1	PLEKHG2
CCDC102B	ENTPD1
EVC	DPT
KIF3C	ANGPT2
FOLR2	CLEC2B
RCN3	TCEAL3
ARHGAP24	DNAJB2
PIAS3	THSD7B
PLEKHG2	KAL1

ENTPD1	DDA1
DPT	SLC12A6
ANGPT2	CLU
CLEC2B	VAMP3
TCEAL3	ATG14
DNAJB2	MYL9
THSD7B	PRICKLE1
KAL1	CSGALNACT2
DDA1	SYDE1
SLC12A6	EHD2
CLU	ZNF521
VAMP3	LRP1
ATG14	LGI1
MYL9	SLITRK4
PRICKLE1	SEPP1
CSGALNAC	ZMIZ1
SYDE1	SMIM3
EHD2	PLBD2
ZNF521	CCDC3
LRP1	CLTB
LGI1	CMTM3
SLITRK4	PRDM1
SEPP1	EID1
ZMIZ1	CDKN1C
SMIM3	ABCA6
PLBD2	LYPD3
CCDC3	NOTCH2
CLTB	SULF2
CMTM3	GXYLT2
PRDM1	FBXL7
EID1	WLS
CDKN1C	CRYAB
ABCA6	KLHDC8A

LYPD3	PKD2
NOTCH2	PAM
SULF2	BAI3
GXYLT2	PPAPDC1A
FBXL7	MSC
WLS	PRR24
CRYAB	DACT3
KLHDC8A	PCBP4
PKD2	NLGN2
PAM	ASAP1
BAI3	ADAMTS12
PPAPDC1A	AKR1B1
MSC	MAB21L2
PRR24	CHPF
DACT3	RENBP
PCBP4	G0S2
NLGN2	TSPYL4
ASAP1	AMOTL1
ADAMTS12	IGFBP4
AKR1B1	RGL1
MAB21L2	ITPRIP
CHPF	CSRP2
RENBP	ANK2
G0S2	SHISA4
TSPYL4	MPPE1
RHOD	LINC00839
AMOTL1	LRRTM1
IGFBP4	GPR68
RGL1	38231
ITPRIP	NEXN
CSRP2	CXCL12
ANK2	FAM218A
SHISA4	FBLIM1

MPPE1	ANXA6
LINC00839	GALNT10
LRRTM1	SPATS2L
GPR68	KLK13
sep-04	BACE1
NEXN	MAP1A
CXCL12	GPSM1
FAM218A	PTGER3
FBLIM1	RCAN2
ANXA6	GPR4
GALNT10	SMARCD3
SPATS2L	ARHGEF3
KLK13	CNKSR2
BACE1	ZFYVE19
MAP1A	LTBP2
GPSM1	LMCD1
PTGER3	MDFI
RCAN2	WDR13
GPR4	SPON1
SMARCD3	PEA15
ARHGEF3	ZNF438
CNKSR2	MYH11
ZFYVE19	MAML2
LTBP2	FLT1
	HLX
LMCD1	MAFF
MDFI	PRKCDBP
WDR13	FYN
MID1IP1	NFATC1
SPON1	TENM1
PEA15	CD109
ZNF438	BHLHE41
MYH11	CLIP4

MAML2	HDAC9
FLT1	RNF144A
HLX	SGCB
MAFF	PORCN
PRKCDBP	TRIB2
FYN	SHC1
NFATC1	PJA2
TENM1	TACC1
CD109	SKI
BHLHE41	TMEM175
CLIP4	FRY
HDAC9	ATXN1
RNF144A	MEDAG
SGCB	CNRIP1
PORCN	RELA
TRIB2	GFPT2
SHC1	PBX3
PJA2	MRAS
TACC1	UBE2R2
SKI	CD151
TMEM175	CPQ
FRY	PTPN21
ATXN1	TPST1
MEDAG	TPP1
CNRIP1	TXNDC15
DDR1	PCDHB11
RELA	PLCD1
GFPT2	PLK3
PBX3	C7orf57
MRAS	ULK3
UBE2R2	EBF1
CD151	CLSTN3
CPQ	MCC

PTPN21	MPDZ
TPST1	GADD45B
TPP1	ATP10A
TXNDC15	CMTM7
PCDHB11	YIPF1
PLCD1	GINM1
PLK3	UGCG
C7orf57	HOPX
ULK3	ADIPOR1
EBF1	DEGS1
CLSTN3	ADM
MCC	PEAK1
MPDZ	ZNF469
GADD45B	ADAM8
ATP10A	YPEL3
LAMB3	UPP1
CMTM7	EVL
YIPF1	SERPINH1
GINM1	ANKRD50
UGCG	SNAPC2
HOPX	DNAJB5
ADIPOR1	C1QTNF6
DEGS1	EGR2
ADM	NR4A3
PEAK1	METRNL
ZNF469	CEP170
ADAM8	CEBPB
YPEL3	ARPP19
UPP1	C1orf85
EVL	AHNAK2
SERPINH1	BHLHE40
ANKRD50	FAM110D
SNAPC2	MEA1

DNAJB5	WIPF1
C1QTNF6	SCD5
EGR2	ADAMTS10
NR4A3	SLC9A1
METRNL	DCBLD2
CEP170	TMEM108
CEBPB	PLEKHM3
ARPP19	SYNPO2
C1orf85	CHST11
AHNAK2	CTSB
BHLHE40	TOM1L2
FAM110D	ENPEP
MEA1	CAPG
WIPF1	TRPS1
SCD5	PRNP
ADAMTS1(	EPHA3
SLC9A1	FAM110B
DCBLD2	SLFN11
TMEM108	EML1
PLEKHM3	FAM129A
SYNPO2	STAT6
CHST11	LAYN
CTSB	NPTX1
TOM1L2	HSPB8
ENPEP	BCAR3
CAPG	TMEM245
TRPS1	TGFB3
PRNP	KIF26B
EPHA3	VSTM4
FAM110B	C6orf226
SLFN11	MAGEH1
EML1	CYTH3
FAM129A	TECPR1

STAT6	SNX18
LAYN	HIVEP2
NPTX1	IGFBP6
HSPB8	C20orf194
BCAR3	CDK14
TMEM245	TGFB1I1
TGFB3	RNF217
KIF26B	MR1
VSTM4	SAT1
C6orf226	POLR3GL
MAGEH1	SPSB1
CYTH3	KIAA1467
TECPR1	LY86
FLRT1	MSRB2
SNX18	CD99L2
HIVEP2	ITM2B
IGFBP6	APOE
C20orf194	HDAC6
CDK14	MAP1B
TGFB1I1	RIN2
RNF217	FAM19A5
MR1	UACA
SAT1	TGFB2
POLR3GL	AKT1
SPSB1	RASA3
KIAA1467	FAM127A
LY86	TM2D2
MSRB2	CD276
CD99L2	FTO
ITM2B	JDP2
APOE	BRD2
HDAC6	STX4
MAP1B	STX7

RIN2	LIMS1
FAM19A5	PLOD1
UACA	RNF112
TGFB2	PIK3IP1
AKT1	CLDN11
RASA3	RXFP1
FAM127A	HEY1
TM2D2	PCDH7
CD276	PRKCH
FTO	CYB5R3
JDP2	SPP1
BRD2	RANGRF
STX4	TSPYL5
STX7	LPHN2
LIMS1	ARL4D
PLOD1	GNG2
RNF112	VASP
PIK3IP1	VAMP5
CLDN11	ANTXR2
RXFP1	MARCKS
HEY1	PABPC4L
PCDH7	INSIG2
PRKCH	KIAA2022
CYB5R3	WFDC1
SPP1	BPGM
RANGRF	MAP7D1
TSPYL5	PGR
LPHN2	DTX3
ARL4D	AAK1
GNG2	LRRC8C
VASP	TSHZ1
VAMP5	VPS9D1
ANTXR2	CSRP1

MARCKS	FAM126A
PABPC4L	FAM114A1
INSIG2	ARID5A
KIAA2022	APBB2
WFDC1	FAM171B
BPGM	CLEC14A
MAP7D1	DENND5A
PGR	AKTIP
DTX3	ATL3
AAK1	LEPR ||| LEPROT
LRRC8C	RAB9A
TSHZ1	TAF7
VPS9D1	FGD5
CSRP1	RUNX2
FAM126A	DUSP1
FAM114A1	AP1S2
ARID5A	SELPLG
APBB2	ARMC9
FAM171B	KDM6B
CLEC14A	AFAP1L2
PRSS22	RAP2B
DENND5A	KCTD7
AKTIP	ZBTB47
ATL3	SLC38A2
LEPR ||| L	CYP1B1
RAB9A	RASSF8
TAF7	FKBP10
FGD5	SGSM2
RUNX2	PRKACA
LAMC2	CFL2
DUSP1	MATK
AP1S2	STAB1
SELPLG	CBLN3

ARMC9	DENND3
KDM6B	RGMA
AFAP1L2	RABAC1
RAP2B	MYADM
KCTD7	DCTN1
ZBTB47	DFNA5
SLC38A2	FAM20A
CYP1B1	TPBG
RASSF8	PVRL2
FKBP10	CCDC155
SGSM2	FKBP7
PRKACA	DIP2C
CFL2	SLC10A3
MATK	ARHGAP23
STAB1	WWC2
CBLN3	PDLIM2
DENND3	THBS3
RGMA	MINK1
RABAC1	HDLBP
MYADM	ACTN1
DCTN1	NFKBIA
DFNA5	C12orf57
FAM20A	ETS1
TPBG	ZFP36L1
PVRL2	MAF
CCDC155	ARHGAP15
FKBP7	L3MBTL3
DIP2C	BAG3
SLC10A3	RPS6KA2
ARHGAP23	PNRC1
WWC2	EXTL2
PDLIM2	ATG4A
THBS3	OGN

MINK1	TLDC1
HDLBP	AOC3
ACTN1	CNPY4
NFKBIA	CXorf40A
C12orf57	GABRG2
ETS1	PPEF1
ZFP36L1	CRHBP
MAF	GATS
ARHGAP15	BOC
L3MBTL3	THBS4
BAG3	HS3ST2
RPS6KA2	AP1M1
PNRC1	RAP2C
EXTL2	PCDHGA11
ATG4A	KLHDC8B
OGN	GLT8D1
TLDC1	MFGE8
AOC3	SERINC1
CNPY4	SVIL
CXorf40A	PXDC1
GABRG2	DGKA
PPEF1	OSBPL1A
CRHBP	JUNB
GATS	KCND2
BOC	PPP1R13L
THBS4	MTSS1L
HS3ST2	PAFAH1B1
AP1M1	VAT1
RAP2C	FMOD
PCDHGA11	SYNE1
KLHDC8B	PAPPA
GLT8D1	EHBP1L1
MFGE8	RORA

SERINC1	SSBP2
SVIL	UROD
PXDC1	ID4
DGKA	ENG
OSBPL1A	KAT6A
HOXA4	NAGK
JUNB	HERC1
KCND2	NUAK1
PPP1R13L	CBLB
MTSS1L	LTC4S
PAFAH1B1	KLHL5
FAM83A	MOSPD2
VAT1	STOML1
FMOD	PODXL
SYNE1	XYLT1
PAPPA	DAAM2
EHBP1L1	RASL12
RORA	MN1
SSBP2	SIGIRR
UROD	DNAJB4
ID4	PDK4
ENG	ITGA5
KAT6A	ZBBX
NAGK	BMPR2
HERC1	DCHS1
NUAK1	CLDN5
CBLB	KLF8
LTC4S	ZBTB20
KLHL5	SCARA3
MOSPD2	ARL2BP
EXOC7	TGFBR2
STOML1	SMARCA1
PODXL	SH2D3C

XYLT1	FAM131A
DAAM2	MORF4L1
RASL12	SPRY1
MN1	SNX33
SIGIRR	KCNMB1
DNAJB4	ZNF362
PDK4	ITGA4
ITGA5	GLI2
ZBBX	RGS10
BMPR2	VGLL4
DCHS1	SCGB3A2
CLDN5	WSB1
KLF8	STC1
ZBTB20	CXorf36
SCARA3	SGIP1
ARL2BP	S100A4
TGFBR2	INPP5F
SMARCA1	S100A16
SH2D3C	CDKN1A
FAM131A	MFSD1
MORF4L1	DHRS3
SPRY1	LDOC1
SNX33	VEGFC
KCNMB1	SYNPO
TNFRSF21	BNIP3L
ZNF362	ADAMTS6
ITGA4	PIGB
GLI2	KDR
RGS10	ITGB1
VGLL4	FRMD4B
SCGB3A2	ARMCX2
WSB1	PLEKHM2
STC1	PNMA1

CXorf36	MYO5A
SGIP1	PFKFB3
S100A4	BNIP2
INPP5F	BEND6
S100A16	SERTAD1
CDKN1A	TMEM88
MFSD1	PARVA
DHRS3	EGR1
LDOC1	PCDHGA3
VEGFC	SPIN1
SYNPO	GNAI2
BNIP3L	SMTN
ADAMTS6	FAT4
PIGB	SWI5
KDR	ZBTB46
RIPK4	UBQLN2
ITGB1	PHLDA3
FRMD4B	AKAP13
ARMCX2	ZNF281
PLEKHM2	LAT2
PNMA1	MID2
MYO5A	MYCT1
PFKFB3	GRIA2
BNIP2	ERRFI1
BEND6	TEK
SERTAD1	CLSTN1
TMEM88	KIAA0247
PARVA	STEAP4
EGR1	EFHC1
PCDHGA3	TPRG1L
SPIN1	RASSF4
GNAI2	AMPH
SMTN	ADAMTSL3

FAT4	C1QTNF7
SWI5	PLCD3
ZBTB46	SLC27A6
UBQLN2	KCNMB2
PRPH	C16orf45
PHLDA3	PPP2R2B
AKAP13	GALNT15
ZNF281	UST
LAT2	ZNF331
MID2	SLC39A13
MYCT1	KIAA1462
GRIA2	LIMS2
ERRFI1	SEC31A
TEK	VCL
CLSTN1	FAM63B
KIAA0247	GPC1
STEAP4	CCL8
EFHC1	DUSP5
KRT19	MAP1LC3B
TPRG1L	PIEZO2
RASSF4	APOD
AMPH	GLG1
COLEC10	TMEM8A
ADAMTSL3	UNC93B1
C1QTNF7	CNTNAP1
PLCD3	CD36
SPINT1	SGCD
SLC27A6	TXNIP
KCNMB2	DYNLT3
C16orf45	TNFSF8
PPP2R2B	IGSF5
GALNT15	SLC24A2
UST	THSD4

ZNF331	C7
SLC39A13	CLEC1A
VGLL1	GLI3
KIAA1462	PPP1R15A
LIMS2	NALCN
SEC31A	TBC1D9
VCL	CHST14
FAM63B	ADD1
GPC1	PELO
CCL8	GJB2
DUSP5	LILRA2
MAP1LC3B	CD4
PIEZO2	CITED2
APOD	C1orf216
GLG1	TP73-AS1
TMEM8A	WDR45
UNC93B1	LRRC4C
CNTNAP1	USP11
CD36	DDIT3
ZNF185	PCDHB7
SGCD	TMED4
TXNIP	PLVAP
DYNLT3	LY96
TNFSF8	CXCR4
IGSF5	PAX8
SLC24A2	ISCU
THSD4	TM4SF1
C7	NDST1
CLEC1A	SOX15
GLI3	JAKMIP2
PPP1R15A	NCKAP5L
NALCN	LPL
TBC1D9	FHL2

CHST14	FEZ1
ADD1	NR3C1
PELO	EFNB2
GJB2	ADCYAP1R1
LILRA2	FOSL2
CD4	CLEC11A
CITED2	ALDH1L2
C1orf216	CACNA2D4
TP73-AS1	MAPKAPK2
WDR45	FRMD4A
LRRC4C	PBX1
USP11	AHR
DDIT3	RBPMS
PCDHB7	MURC
TMED4	PSAP
PLVAP	CAPZB
LY96	DYRK3
CXCR4	EYA4
PAX8	PACS1
ISCU	BMP4
TM4SF1	NRBF2
NDST1	GOLGA2
SOX15	TRPV2
JAKMIP2	NPTN
NCKAP5L	CCDC88A
LPL	ZNF365
FHL2	TLN1
FEZ1	ADAMTS3
NR3C1	SAMD4A
EFNB2	MICAL2
ADCYAP1R	NEFM
FOSL2	PURA
CLEC11A	ACSS3

GJB3	CRABP2
ALDH1L2	CREM
CACNA2D4	ENDOD1
MAPKAPK2	SH3PXD2A
FRMD4A	PCED1B
PBX1	TCEA2
AHR	HTR7P1
RBPMS	TFEB
MURC	PDZD2
PSAP	LRRC17
CAPZB	SUSD4
PAEP	LRCH3
DYRK3	GPRC5B
EYA4	ACADVL
PACS1	NTAN1
BMP4	ABHD2
NRBF2	YAP1
GOLGA2	EBF4
TRPV2	NAV1
NPTN	NDEL1
CCDC88A	TNFSF4
ZNF365	TMEM30A
TLN1	SNN
ADAMTS3	FOSB
SAMD4A	HMOX1
MICAL2	SDCCAG8
NEFM	CCNA1
PURA	PLAUR
ACSS3	GABARAPL1
CRABP2	KLF2
CREM	ZNRF1
ENDOD1	BCL9L
SH3PXD2A	MEF2D

PCED1B	RGCC
TCEA2	CNGA3
HTR7P1	TNFRSF12A
TFEB	GRAMD1B
PDZD2	HTR1F
LRRC17	PCDHB10
SUSD4	TSPAN2
LRCH3	TMEM136
GPRC5B	SASH1
ACADVL	GPR161
NTAN1	UNC5C
ABHD2	LAMA5
YAP1	NGFRAP1
EBF4	AP3M2
NAV1	NTSR1
NDEL1	GPRASP1
TNFSF4	ZNF532
TMEM30A	BRI3
SNN	MBD5
FOSB	TP53BP1
HMOX1	LIX1L
SDCCAG8	PKM
CCNA1	CALHM2
PLAUR	MAGED2
GABARAPL	SLC2A10
KLF2	SLC35G2
ZNRF1	COL13A1
BCL9L	CASC1
MEF2D	BEX4
RGCC	CD93
CNGA3	STON1
TNFRSF12/	ISYNA1
GRAMD1B	IGSF10

HTR1F	PRPF8
PCDHB10	AIF1
TSPAN2	PRKD1
TMEM136	ARHGAP29
SASH1	GPR116
GPR161	GAD2
UNC5C	RBFOX2
LAMA5	ALCAM
NGFRAP1	PTHLH
AP3M2	ISG15
NTSR1	YIPF5
GPRASP1	CFH
ZNF532	ARHGEF6
BRI3	PTX3
MBD5	BBX
TP53BP1	GRP
LIX1L	FJX1
PKM	PLXND1
CALHM2	OAZ2
RIMBP2	SLIT3
MAGED2	CNIH3
SLC2A10	LIF
SLC35G2	LRRC8A
COL13A1	SOX5
CASC1	SLC15A3
WFDC3	MAP3K6
BEX4	CAMTA2
CD93	RHOQ
STON1	LSAMP
ISYNA1	CNTN4
IGSF10	PRPH2
PRPF8	FBXL5
AIF1	TUBB2A

PRKD1	LPAR1
ARHGAP29	RNH1
GPR116	STK17A
GAD2	ECSCR
RBFOX2	BCL6
ALCAM	ZBTB4
PTHLH	ZNF275
GAS6	PRRG1
ISG15	HEXA
YIPF5	DZIP1
CFH	NPR3
ARHGEF6	SSH1
PTX3	HSD17B2
BBX	CHD3
GRP	CHODL
FJX1	TBX15
PLXND1	CCM2L
OAZ2	EZR
SLIT3	PRDM6
CNIH3	ASAP2
LIF	METRN
LRRC8A	NEFH
SOX5	ZNF436
SLC15A3	DOK5
MAP3K6	TK2
CAMTA2	CTF1
RHOQ	ITPKB
LSAMP	PPP2CB
CNTN4	PDE2A
PRPH2	MEGF10
FBXL5	FOXF2
TUBB2A	CAMSAP2
LPAR1	SUGCT

RNH1	LHFPL2
STK17A	PLEKHO2
ECSCR	NCK2
BCL6	ADORA3
ZBTB4	AHNAK
ZNF275	ACYP2
PRRG1	HSPA12B
HEXA	PDGFRL
DZIP1	KLHL21
NPR3	JAZF1
SSH1	TMTC1
HTR2C	ZFHX4
HSD17B2	ITPRIPL2
CHD3	DUSP6
CHODL	ASPHD1
TBX15	RIT1
CCM2L	ARHGAP33
EZR	TEAD2
PRDM6	ZNF134
ASAP2	ARHGAP1
METRN	VKORC1
NEFH	PPP1R12B
ZNF436	SRGAP2
DOK5	MAP4K4
TK2	NRXN1
CTF1	P4HA3
ITPKB	NT5E
PPP2CB	RARB
PDE2A	ZHX2
MEGF10	COL16A1
FOXF2	RIMKLB
CAMSAP2	C1orf198
SUGCT	NREP

LHFPL2	SPTAN1
PLEKHO2	ETV1
NCK2	TGFA
ADORA3	CRIP2
AHNAK	ANO2
ACYP2	UBE2E3
HSPA12B	CDH6
PDGFRL	FAM124B
KLHL21	FAM49A
JAZF1	LTBP4
TMTC1	ABCC9
ZFHX4	PTN
ITPRIPL2	CPNE8
DUSP6	STX2
MROH6	ZFP28
ASPHD1	MAST4
RIT1	PARVG
ARHGAP33	FAM46A
TEAD2	BACH1-IT1 ||| BACH1
ZNF134	TCEAL1
ARHGAP1	TBX2
VKORC1	STOX2
PPP1R12B	TEAD1
SRGAP2	SLC7A7
MAP4K4	CDKN2C
NRXN1	IQGAP1
P4HA3	INHBB
NT5E	C1RL
RARB	KIAA1644
ZHX2	VSIG4
COL16A1	WBP5
RIMKLB	ERG
C1orf198	SYT11

NREP	TENM4
SPTAN1	ELFN2
ETV1	S100A13
TGFA	NAB2
CRIP2	GULP1
ANO2	PLA2G5
UBE2E3	PPP1R3C
CDH6	ST3GAL6
FAM124B	MEIS2
FAM49A	PTPRZ1
LTBP4	NFATC4
ABCC9	ENOX1
PTN	C1orf54
CPNE8	HAO2
STX2	TOM1
ZFP28	C6orf15
MAST4	ZNF449
PARVG	LONRF2
CAPN12	HES4
FAM46A	PCDHB4
BACH1-IT1	RASSF5
TCEAL1	TLE4
TBX2	POLM
STOX2	EGFLAM
TEAD1	ELTD1
SLC7A7	CCDC8
CDKN2C	PLEKHO1
IQGAP1	ALPL
INHBB	MYH9
C1RL	PCDHB15
KIAA1644	PBXIP1
VSIG4	PTGFRN
WBP5	ANGPT1

ERG	CYYR1
SYT11	TMEM47
TENM4	RDX
ELFN2	ZNF677
S100A13	TRIM8
NAB2	SCARF1
GULP1	NKX3-2
PLA2G5	IDS
PPP1R3C	GNS
ST3GAL6	RECK
MEIS2	ACE
PTPRZ1	CDC42BPA
NFATC4	TMEM200B
ENOX1	RNF207
C1orf54	PTMS
HAO2	HOXB2
TOM1	ZIM2 ||| PEG3
C6orf15	ERC1
ZNF449	C1QTNF3
LONRF2	PRMT2
HES4	EHBP1
PCDHB4	PREX2
RASSF5	ZNF423
TLE4	RBPMS2
POLM	NAV3
EGFLAM	LRCH1
ELTD1	FAM195B
CCDC8	SCN4B
PLEKHO1	MSANTD3
ALPL	CCDC85A
FBXL14	VWF
MYH9	SCARF2
PCDHB15	TCEAL7

PBXIP1	GPR87
PTGFRN	TSHZ2
ANGPT1	NDUFAF1
CYYR1	CSTB
TMEM47	PAPSS1
RDX	PCSK5
ZNF677	SFXN3
TRIM8	INMT ||| INMT-FAM188B
SCARF1	MTMR9
NKX3-2	PIP4K2A
IDS	RASIP1
GNS	MMRN2
RECK	IRF2BPL
ACE	IL10RB
CDC42BPA	TREM2
TMEM200I	HTRA3
RNF207	SDS
PTMS	VAT1L
MAGI2	LETM2
HOXB2	KIF7
ZIM2 ||| P	NAP1L3
ERC1	MARCO
C1QTNF3	MAPKBP1
PRMT2	PLSCR4
EHBP1	MEF2A
PREX2	LRP10
ZNF423	CD99
RBPMS2	FXYD1
NAV3	CHST7
LRCH1	ACKR3
FAM195B	ROBO4
SCN4B	KANK4
MSANTD3	SPTBN5

CCDC85A	COL22A1
VWF	PLS3
SCARF2	TUBG2
TCEAL7	SPRY4
GPR87	CHRD
TSHZ2	LINC00478
NDUFAF1	SH3GLB1
CSTB	CDC42EP3
PAPSS1	ANXA1
PCSK5	PCDH9
SFXN3	TLR5
INMT ||| I	C4orf47
MTMR9	PHYHD1
PIP4K2A	FBXL3
RASIP1	FAM65A
MMRN2	ANKRD35
IRF2BPL	PPM1F
IL10RB	CREBRF
TREM2	RAB8B
HTRA3	MLLT11
SDS	PCGF2
VAT1L	ICMT
LETM2	MED15
KIF7	RIOK3
NAP1L3	TFE3
MARCO	CDKN2B
MAPKBP1	C19orf66
PLSCR4	OR1A1
MEF2A	RANBP9
LRP10	ILK
CD99	NEGR1
FXYD1	UNC13D
CHST7	ISG20

ACKR3	KCNJ15
ROBO4	ITGB1BP2
KANK4	CCDC50
SPTBN5	MAN2B1
ANXA3	SLFN12
COL22A1	AGPAT4
PLS3	TMEM55A
TUBG2	ARSI
SPRY4	BICD2
CHRD	RASGRP3
LINC00478	HAND2-AS1
SH3GLB1	SLC20A2
PPL	FAM19A2
CDC42EP3	TMEM140
ANXA1	PRKG1
PCDH9	CD59
TLR5	CYP27C1
C4orf47	SNCA
PHYHD1	GYPC
FBXL3	FHOD3
FAM65A	CHSY3
ANKRD35	ROR1
PPM1F	SH3KBP1
CREBRF	TREM1
RAB8B	CD33
MLLT11	APLNR
PCGF2	ZFYVE1
ICMT	DVL3
MED15	PLK2
RIOK3	GPC6
BAIAP2	GNB5
TFE3	SYN1
CDKN2B	36951

C19orf66	PLCD4
OR1A1	ECE1
RANBP9	VASH1
ILK	REEP3
NEGR1	RNF144B
UNC13D	STX12
ISG20	ARHGEF37
KCNJ15	MFNG
ITGB1BP2	KCNJ8
CCDC50	SMAD7
MAN2B1	SLC9A9
SLFN12	PRND
AGPAT4	WDFY3
TMEM55A	SIRPB1
ARSI	ARRDC3
BICD2	HIGD1B
RASGRP3	SLC11A1
HAND2-AS	SERPINA5
SLC20A2	SUSD2
FAM19A2	CD81
TMEM140	LYRM5
PRKG1	UBXN6
CD59	LAPTM4A
CYP27C1	CDYL2
SNCA	DDAH2
GYPC	NCOR2
FHOD3	SV2A
CHSY3	CPXM2
	SH3BGRL
ROR1	CRAT
SH3KBP1	RHBDD2
TREM1	CREB3
CD33	MAGED1

APLNR	ADAM9
ZFYVE1	FKBP8
DVL3	SOX11
PLK2	VTCN1
GPC6	CDK2AP1
GNB5	SMPD1
SYN1	GRIK2
mar-01	SGK1
PLCD4	CDKN2A
ECE1	DDHD2
VASH1	KIAA1024
REEP3	IBSP
RNF144B	PPAPDC3
STX12	MIR22HG
ARHGEF37	WT1-AS
MFNG	SCG5
KCNJ8	FAM46B
SMAD7	HSPB1
SLC9A9	ARMCX3
PRND	NR1D2
WDFY3	UBE2QL1
SIRPB1	ZYX
ARRDC3	AVPR1A
HIGD1B	PLEKHA5
SLC11A1	RNF11
SERPINA5	NR2F1-AS1
SUSD2	SLC17A5
CD81	RNF7
LYRM5	RND2
UBXN6	SH3BGRL3
LAPTM4A	AGRN
CDYL2	FLNC
DDAH2	CARD6

NCOR2	NAP1L2
SV2A	EHD3
CPXM2	FXYD5
SH3BGRL	RRAS
CRAT	FGD6
RHBDD2	SNX9
CREB3	CDC42BPB
MAGED1	TMEM106A
ADAM9	IP6K3
FKBP8	PEAR1
SOX11	TSPYL2
VTCN1	FCN1
CDK2AP1	WWC3
LINGO1	PLA2G16
SMPD1	RELL1
GRIK2	RUFY3
SGK1	ITSN1
CDKN2A	LRFN5
DDHD2	LEPRE1
KIAA1024	FAT3
IBSP	SCG2
PPAPDC3	PI15
MIR22HG	LZTS1
WT1-AS	BATF
SCG5	RSPO3
FAM46B	ARL6IP5
HSPB1	CBX4
ARMCX3	MRGPRF
NR1D2	LY6H
UBE2QL1	LYPD1
ZYX	TPM1
AVPR1A	TRAPPC6B
PLEKHA5	KLF7

RNF11	SKAP1
NR2F1-AS1	SH2B3
SLC17A5	ALPP
RNF7	C10orf54
RND2	TRIOBP
SH3BGRL3	ARL10
AGRN	TUB
FLNC	AAED1
CARD6	ARHGDIB
NAP1L2	USP51
EHD3	FAM107A
FXYD5	C10orf128
RRAS	SIGLEC7
FGD6	THEMIS2
SNX9	BTBD16
CDC42BPB	C10orf25
TMEM106/	FAXDC2
IP6K3	VIMP
PEAR1	FAM198A
TSPYL2	SLC39A1
FCN1	C14orf37
WWC3	NPAS3
PLA2G16	NOS1
RELL1	SNX29
RUFY3	AP3B2
ITSN1	VGLL3
LRFN5	FAM50A
LEPRE1	KCTD1
FAT3	LPP
SCG2	SYNGR1
PI15	MARK4
LZTS1	LYST
BATF	RASGRF2

RSPO3	ATF3
ARL6IP5	CD14
CBX4	NPC2
MRGPRF	CCR1
LY6H	SGMS2
LYPD1	RASSF2
TPM1	BCL2L1
TRAPPC6B	SLC46A3
KLF7	CCPG1
SKAP1	PTPN12
SH2B3	C19orf59
ALPP	FBXO32
C10orf54	GLI1
TRIOBP	FLT4
ARL10	CTNNA1
TUB	DCLK1
AAED1	PDE1A
ARHGDIB	MAP4
USP51	YWHAE
FAM107A	P2RX7
C10orf128	VEGFB
SIGLEC7	CCDC92
THEMIS2	ITGAM
BTBD16	MEF2C
C10orf25	HCK
FAXDC2	WBP1L
VIMP	ZNF404
FAM198A	ZFPM2
SLC39A1	APOC1
C14orf37	SOX17
NPAS3	ADAMTS5
NOS1	PECAM1
SNX29	PHF1

AP3B2	TCEAL4
VGLL3	LZTS2
FAM50A	NCF2
KCTD1	SLA
LPP	ZFAND3
SYNGR1	ISM1
MARK4	HMCN1
LYST	ANGPTL4
RASGRF2	PPP2R4
ATF3	FAM89B
CD14	FILIP1
NPC2	CSF1R
	TCEAL8
CCR1	VAMP4
SGMS2	SLC16A3
RASSF2	FAM229B
BCL2L1	C5AR1
SLC46A3	TSPAN9
CCPG1	RNF150
PTPN12	KRAS
KLK7	CD163L1
C19orf59	CECR1
FBXO32	FKBP9
EPHA2	GALNT1
GLI1	LEPR
FLT4	NLRP1
CTNNA1	SNX3
DCLK1	RGS4
PDE1A	GLRB
MAP4	LAIR1
YWHAE	LMNA
P2RX7	IL10
VEGFB	CRISPLD1

CCDC92	SIGLEC1
ITGAM	LIMCH1
MEF2C	NFKB2
HCK	ZEB2
WBP1L	ITGA7
ZNF404	NPC1
ZFPM2	NCALD
APOC1	GJA4
SOX17	THSD7A
ADAMTS5	TNFRSF1A
PECAM1	HIP1
PHF1	TRPC1
TCEAL4	SLC38A6
LZTS2	FBP1
NCF2	NGFR
SLA	SYNC
ZFAND3	SYCP2
ISM1	LAMP5
HMCN1	ABCA9
ANGPTL4	ZNF25
PPP2R4	EBF2
FAM89B	TBC1D1
KRT17	PIM1
FILIP1	TEAD3
CSF1R	SLC37A2
TCEAL8	GNA15
VAMP4	GUCY1A2
SLC16A3	CORO1A
FAM229B	FGD1
C5AR1	JAM2
TSPAN9	MOSPD1
RNF150	TRERF1
KRAS	CPEB4

CD163L1	LRRC49
SCGN	WDR81
CECR1	CSRNP1
FKBP9	F8
GALNT1	PLCL1
LEPR	CD55
NLRP1	SERPINB2
SNX3	S100A3
RGS4	RERGL
GLRB	PHACTR1
LAIR1	MPRIP
LMNA	CAP2
IL10	IL20RB
CRISPLD1	ITPR1
SIGLEC1	UBE2G1
LIMCH1	PARP8
NFKB2	TPM4
ZEB2	SEMA3A
ITGA7	DCAF5
NPC1	IKBIP
NCALD	TTC9
GJA4	MS4A7
THSD7A	HSPA6
KLK5	POLR2A
TNFRSF1A	PPM1M
HIP1	SIGLEC10
TRPC1	CAPN2
SLC38A6	LPXN
FBP1	ENO2
NGFR	DST
SYNC	RING1
LAMA3	GAA
SYCP2	AP2M1

LAMP5	NTN1
ABCA9	TENC1
ZNF25	ST6GALNAC5
EBF2	KIF1B
TBC1D1	PTTG1IP
PIM1	ETV5
TEAD3	TYROBP
SLC37A2	ALOX5AP
EPS8L1	DOCK4
GNA15	FES
GUCY1A2	MDFIC
CORO1A	SNHG14
FGD1	SPI1
JAM2	GAP43
MOSPD1	CSF3R
TRERF1	SPRED1
CPEB4	MEGF6
LRRC49	TTC7B
WDR81	ZNF385A
FXYD4	RBMS2
CSRNP1	ZNF83
F8	TNKS1BP1
PLCL1	PRKAB2
CD55	TINF2
SERPINB2	ENPP2
S100A3	CACNA2D1
RERGL	COPS7A
PHACTR1	PRICKLE2
MPRIP	MGAT1
CAP2	WDR7
IL20RB	PLN
ITPR1	KLC1
SNX24	TP53INP2

UBE2G1	OSBPL8
PARP8	PKN1
TPM4	37316
SEMA3A	ESAM
DCAF5	CSRNP2
IKBIP	CCNI
TTC9	ULK2
MS4A7	SRPX2
HSPA6	PFDN5
POLR2A	RRAGA
PPM1M	PCDHB13
SIGLEC10	MYOT
CAPN2	QSOX1
LPXN	ARF4
SLC22A18	CTDNEP1
ENO2	WDR47
DST	ZNF697
RING1	VWDE
GAA	PINK1
AP2M1	COL24A1
NTN1	NOX4
TENC1	GLIS3
ST6GALNA	ADCY2
KIF1B	RBP7
PTTG1IP	SCN3B
ETV5	C3AR1
TYROBP	RNF185
ALOX5AP	ITGA11
DOCK4	SAT2
FES	PRRG3
MDFIC	NRSN2
SNHG14	DAP
SPI1	C2orf74

GAP43	CYTL1
CSF3R	KLHL15
SPRED1	SCUBE2
MEGF6	TIE1
TTC7B	FST
ZNF385A	CPM
RBMS2	OR51E2
ZNF83	HBEGF
TNKS1BP1	DMPK
PRKAB2	ZFAND2A
TINF2	CD63
ENPP2	KCNB1
CACNA2D1	ESM1
COPS7A	FOSL1
PRICKLE2	TDRP
MGAT1	CDR2L
WDR7	CD300LF
PLN	PHLDB1
KLC1	HCFC2
TP53INP2	RRAGC
OSBPL8	CYLD
PKN1	TTYH3
mar-02	IVNS1ABP
ESAM	MAOB
KLK8	KLRF1
CSRNP2	CDH5
CCNI	PI4KB
ULK2	ARAP3
SRPX2	TCP11L1
PFDN5	HOOK3
RRAGA	B3GNT9
KCNN4	MPPED2
PCDHB13	ARHGAP31

MYOT	PDZRN3
QSOX1	ANO5
ARF4	SNAP25
CTDNEP1	SCUBE3
WDR47	MS4A6A
ZNF697	SLMAP
VWDE	C2orf40
PINK1	PNMAL1
COL24A1	CCR10
NOX4	ZFHX3
GLIS3	CALCRL
ADCY2	PPFIBP1
RBP7	WASF1
SCN3B	OPTN
C3AR1	APBB1
RNF185	CAND2
ITGA11	FAM120C
SAT2	TMEM107
PRRG3	ABI3BP
NRSN2	ZNF117
DAP	SOX4
C2orf74	ATP6V1D
CYTL1	LAMP2
KLHL15	TRPC4
SCUBE2	ANKDD1A
TIE1	CCBE1
FST	PRAF2
CPM	FOXC1
OR51E2	NLGN4X
HBEGF	PCOLCE2
RASAL1	C11orf68
DMPK	PJA1
ZFAND2A	FTH1

CD63	PPP4R1
KCNB1	GPR137B
ESM1	NDUFA4L2
FOSL1	ARHGAP10
TDRP	RRAGB
CDR2L	RHOC
CD300LF	ECM2
PHLDB1	TRIP10
HCFC2	ZSWIM4
RRAGC	DAPK1
CYLD	STX8
TTYH3	AR
IVNS1ABP	TMEM255A
MAOB	SDPR
KLRF1	DNAJC1
LEMD1	C1QC
CDH5	JPH2
PI4KB	KIAA1377
ARAP3	DCTN2
KCNK3	ABCA13
TCP11L1	GPBAR1
HOOK3	HSPB7
RNF39	CX3CR1
B3GNT9	DNER
MPPED2	CCNG2
ARHGAP31	SLC16A6
PDZRN3	CD72
ANO5	SRGAP1
SNAP25	CALB2
SCUBE3	NDFIP1
MS4A6A	RTN2
SLMAP	MMP16
C2orf40	TNIP1

PNMAL1	KLF12
CCR10	HIVEP3
ZFHX3	DMXL2
	TTLL7
HIST2H2BE	PCDHGA4
CALCRL	SYNDIG1
PPFIBP1	BACH2
WASF1	SLC16A2
OPTN	DYNC1LI2
APBB1	SOX18
LMO7	RFTN2
CAND2	SLCO2A1
FAM120C	KIDINS220
TMEM107	PCDH17
ABI3BP	GADD45A
ZNF117	PDZRN4
SOX4	VASN
ATP6V1D	TM4SF18
LAMP2	LMOD1
TRPC4	EMCN
ANKDD1A	KCTD11
CCBE1	ABLIM3
C1orf116	EVI2B
PSORS1C1	MAPRE2
PRAF2	VPS18
FOXC1	AQP1
NLGN4X	NEK7
PCOLCE2	PLEC
C11orf68	EFHD1
PJA1	TMSB15A ||| TMSB15B
FTH1	EPHB6
PPP4R1	ZNF382
GPR137B	ABCG2

NDUFA4L2	EMC4
ARHGAP1(	FNDC4
RRAGB	CORIN
RHOC	RERG
ECM2	IGFL1
GPRC5A	MDK
SEZ6L2	SMAD3
TRIP10	CTDSP1
ZSWIM4	DPYD
DAPK1	PILRA
STX8	HAS2
AR	GRN
TMEM255/	GYG1
SDPR	C6orf89
DNAJC1	CD84
C1QC	BEAN1
JPH2	TLR7
KIAA1377	ZNF580
DCTN2	GGTA1P
ABCA13	MYO1C
GPBAR1	C1QA
HSPB7	PDE7B
CX3CR1	DIXDC1
DNER	FLI1
CCNG2	ANKRD6
SLC16A6	CADM1
CD72	RAB38
SRGAP1	CLIC2
CALB2	YPEL5
NDFIP1	SLC44A2
RTN2	KCNT2
MMP16	CORO2B
TNIP1	ARF3

KLF12	RILPL1
HIVEP3	C1orf51
DMXL2	HDAC7
TTLL7	ZNF667
PCDHGA4	SEC14L1
SYNDIG1	FIGN
C19orf33	RYR1
BACH2	TERF2IP
SLC16A2	HECW2
DYNC1LI2	CHRNB1
SOX18	EPYC
RFTN2	ARNT2
SLCO2A1	SYT17
KIDINS220	PLP1
PCDH17	BVES
GADD45A	PHF23
PDZRN4	KIAA0040
VASN	ZNF641
TM4SF18	LAPTM5
LMOD1	WNK1
EMCN	NRIP3
KCTD11	AKR1C1
ABLIM3	PALMD
EVI2B	CLIP3
MAPRE2	COL21A1
VPS18	NHSL2
AQP1	RAB2B
NEK7	PGM2L1
PLEC	MATN3
EFHD1	CALCOCO1
TMSB15A	TM6SF1
EPHB6	KLF6
ZNF382	ZNF542

ABCG2	XKR4
GDPD3	HAS1
EMC4	PPM1L
FNDC4	ACTG1 ||| ACTB
CORIN	CSGALNACT1
RERG	ACTC1
IGFL1	ZNF829
MDK	OS9
SMAD3	ERMN
KLK10	ARHGAP20
CTDSP1	KIAA1549L
DPYD	OSCAR
PILRA	MCOLN1
HAS2	AVEN
GRN	NR1H2
GYG1	LCA5
C6orf89	ANKRD52
CD84	AK5
BEAN1	RCBTB2
TLR7	APBB1IP
SLC20A1	HPGDS
ZNF580	PHF13
GGTA1P	TNIK
MYO1C	PCDH19
C1QA	ZNF853
PDE7B	FAM219B
DIXDC1	RNASE6
FLI1	RIMS2
ANKRD6	OBFC1
CADM1	SLC7A2
RAB38	COLGALT2
CLIC2	NDP
YPEL5	HCFC1R1

SLC44A2	LARP6
KCNT2	ZNF304
CORO2B	SOX7
ARF3	TANC2
RILPL1	INPP4B
C1orf51	SLC22A17
HDAC7	ATP8B2
ZNF667	ORAI3
SEC14L1	SOCS5
FIGN	PALM2
RYR1	TPRG1
TERF2IP	LTBP3
HECW2	NFE2L1
CHRNB1	MMP8
KLK11	CDA
KLK6	HECTD2
EPYC	LYVE1
ARNT2	DUSP4
SCEL	GPM6B
SYT17	WAS
PLP1	GAL3ST4
KCNJ16	C1orf123
BVES	NINJ2
PHF23	ZBED2
KIAA0040	CD163
ZNF641	CNTN1
LAPTM5	SIPA1
WNK1	MIDN
NRIP3	MMP28
AKR1C1	PLAC9
PALMD	FGF1
KRT80	FMO1
CLIP3	SECISBP2L

COL21A1	PCDHB2
NHSL2	GCNT2
RAB2B	ZNF257
PGM2L1	DOK6
MATN3	HOMER3
CALCOCO1	TENM3
TM6SF1	ZFP82
KLF6	AK1
ZNF542	GNB1
XKR4	PI4K2A
HAS1	UBR4
PPM1L	STX11
ACTG1 |||	ENTPD3
CSGALNAC	FPR3
ACTC1	LPAR6
ZNF829	SCG3
OS9	CHRNA3
ERMN	FOXC2
ARHGAP2(	PCMTD1
KIAA1549L	DTWD1
OSCAR	PMEPA1
MCOLN1	RNF180
CAMK2N1	EFNA5
AVEN	CR1
NR1H2	MAP2
LCA5	PELI2
ANKRD52	TBC1D19
AK5	ST6GALNAC2
RCBTB2	RAB3B
APBB1IP	SLC22A15
HPGDS	C1orf122
PHF13	TCEAL2
TNIK	HAVCR2

PCDH19	IER5L
ZNF853	CDH19
FAM219B	PCDHB18
RNASE6	FSTL3
RIMS2	GREM2
OBFC1	HSD17B14
SLC7A2	RAB5C
COLGALT2	FAM214B
NDP	DOC2B
HCFC1R1	KCNMA1
LARP6	DOCK5
ZNF304	NRK
SOX7	DDIT4L
TANC2	ACTR1A
INPP4B	LRCH2
SLC22A17	GIMAP4
ATP8B2	HEY2
ORAI3	DDIT4
SOCS5	THRSP
PALM2	CNN1
TPRG1	AMIGO2
LTBP3	C1orf162
NFE2L1	VLDLR
MMP8	TCF7L1
CDA	POU6F1
HECTD2	CERK
LYVE1	STMN2
DUSP4	SCRG1
GPM6B	C3orf18
WAS	RHBDF1
GAL3ST4	F13A1
C1orf123	SAV1
KRT6A	KDM5B

NINJ2	LGMN
ZBED2	EPB41L4A
CD163	OMD
CNTN1	GPAT2
SIPA1	ITGB2
MIDN	SEC11A
MMP28	KCNAB1
PLAC9	FGF7
FGF1	MS4A4A
FMO1	AKR1C2
SECISBP2L	MSR1
PCDHB2	RASSF9
GCNT2	ZNF366
ZNF257	VAV2
DOK6	MAN2B2
HOMER3	IL10RA
TENM3	FKBP1B
ZFP82	PLIN3
DISP2	FGF2
AK1	FCER1G
GNB1	FAM13C
PI4K2A	MRC1
UBR4	PLCG2
STX11	GNPDA2
ENTPD3	NOV
FPR3	GPR183
LPAR6	EPHA4
SCG3	HIST1H2AC
CHRNA3	GABARAP
FOXC2	CTSZ
PCMTD1	ANO1
DTWD1	TRAPPC1
PMEPA1	TRNP1

RNF180	BHMT2
EFNA5	EMX2
CR1	MUC16
MAP2	SLC16A4
PELI2	C1QTNF2
TBC1D19	EMILIN1
ST6GALNA	TMEM35
RAB3B	RILPL2
SLC22A15	ZMAT1
C1orf122	MMP13
TCEAL2	MEOX2
HAVCR2	DKK2
IER5L	SIX1
CDH19	C1QB
PCDHB18	DOK3
FSTL3	CACNA1C
GREM2	RAI2
HSD17B14	FPR1
RAB5C	CST6
FAM214B	PYGM
DOC2B	ANGPTL1
KCNMA1	TNFSF12
DOCK5	IL1RN
NRK	TULP3
DDIT4L	DCTN3
ACTR1A	AFAP1L1
LRCH2	CHL1
GIMAP4	GUK1
HEY2	GSTM3
DDIT4	RHOG
THRSP	SNX1
CNN1	FMO2
AMIGO2	DOCK10

C1orf162	TMEM127
VLDLR	LRP12
TCF7L1	CD86
POU6F1	SSC5D
CERK	PRIMA1
STMN2	MALL
SCRG1	ZNF805
C3orf18	ULBP2
RHBDF1	CGNL1
F13A1	FAM214A
SAV1	HOXA5
KDM5B	RGS19
LGMN	FBXO10
EPB41L4A	CCL7
OMD	ST8SIA4
GPAT2	OLR1
ITGB2	PLEKHG1
SEC11A	HOXC10
KCNAB1	SERF2
FGF7	CTSD
MS4A4A	FCGR2B
AKR1C2	CA11
MSR1	SPOCD1
RASSF9	IGF1R
ZNF366	GABARAPL2
VAV2	PKHD1
MAN2B2	RNF115
IL10RA	SORCS1
FKBP1B	ADAMTS16
PLIN3	KCNA2
SLC2A1	ATP6AP2
FGF2	PELI1
FCER1G	CDIPT

FAM13C	CD53
MRC1	PCDHB6
PLCG2	SMG6
GNPDA2	KIAA1279
NOV	RHOA
GPR183	GSTM5
EPHA4	ZNF781
HIST1H2A(	IFNGR2
GABARAP	HTR2B
CTSZ	TTC7A
ANO1	UBE2H
TRAPPC1	PCDHB12
TRNP1	ADRB2
BHMT2	KCTD10
EMX2	FGF13
MUC16	CD37
SLC16A4	ZNF655
C1QTNF2	RMDN3
EMILIN1	CDKL5
TMEM35	BMF
RILPL2	DCUN1D3
ZMAT1	ANXA2
MMP13	ZNF471
MEOX2	LIFR
DKK2	NOVA1
SIX1	DIRAS3
C1QB	AGPAT1
DOK3	PODN
CACNA1C	GPR110
RAI2	MICU3
FPR1	RGAG4
CST6	RGS17
PYGM	NCKAP1L

ANGPTL1	ARPC2
TNFSF12	PITPNM3
IL1RN	ADCY7
TULP3	AGTR1
DCTN3	KIFC3
AFAP1L1	LMO3
CHL1	GLDN
GUK1	FAM168A
GSTM3	ADPRM
RHOG	GPR158
SNX1	HLA-DPA1
FMO2	SLC14A1
DOCK10	CAP1
TMEM127	MFSD7
LRP12	CRYZL1
CD86	IL17RD
SSC5D	ANKRD65
PRIMA1	LMF1
MALL	BICD1
SCNN1A	FCER1A
ZNF805	HIST1H2BC ||| HIST1H2BE ||| HIST1H2BF ||| HIST1H2BG ||| HIST1H2BI
ULBP2	RTN1
CGNL1	SMOC2
FAM214A	DOCK2
HOXA5	KRT7
RGS19	CD74
FBXO10	KATNAL1
CCL7	ARHGEF25
ST8SIA4	SERPINA3
TM4SF4	ATP1A2
OLR1	CHGB
MSLN	RNF181
PLEKHG1	ST6GALNAC6

HOXC10	LRRK2
SERF2	TYRP1
COL17A1	LACTB
CTSD	MORN4
FCGR2B	BMPR1B
CA11	CPEB1
SPOCD1	STXBP1
IGF1R	FOXP2
GABARAPL	CDO1
PKHD1	MFSD11
RNF115	MICU1
SORCS1	TNNT1
ADAMTS16	FOXJ2
KCNA2	AADAC
ATP6AP2	GPR34
PELI1	CD82
CDIPT	OR51E1
CD53	ZFP90
PCDHB6	EPHA7
SMG6	UBASH3B
KIAA1279	DTNA
RHOA	RAMP1
GSTM5	OLFM1
ZNF781	GJB5
IFNGR2	ADRA2A
HTR2B	MAMDC2
FAM155A	C5orf46
TTC7A	EVI2A
UBE2H	HIST1H1C
PCDHB12	SRGN
ADRB2	FGFR2
KCTD10	PCDHB16
FGF13	ANXA2P2

CD37	TRIM6
ZNF655	DKK1
RMDN3	MAP2K1
CDKL5	IGFL2
BMF	PTPRN2
DCUN1D3	RAI1
ANXA2	TLR1
ZNF471	SWAP70
LIFR	CACHD1
NOVA1	CPE
DIRAS3	ATL1
AGPAT1	MUM1L1
PODN	MYOCD
GPR110	PCDHB5
MICU3	SUN2
RGAG4	ACOT9
RGS17	PRKACB
NCKAP1L	POPDC2
ARPC2	PALM
PITPNM3	LDB3
ADCY7	NPY1R
AGTR1	FAM26E
KIFC3	ALOX5
LMO3	DARC
GLDN	CDH2
FAM168A	RAB11FIP5
ADPRM	SLC4A11
GPR158	SEMA4C
HLA-DPA1	DDX24
SLC14A1	DHRS9
CAP1	CLDN10
MFSD7	GNAI1
CRYZL1	FCGR1A /// FCGR1B

IL17RD	RXRB
ANKRD65	FOLR1
LMF1	CLEC7A
KRT6B	C6orf1
BICD1	C2orf76
FCER1A	STX5
HIST1H2BC	ANXA10
RTN1	PKIA
SMOC2	CMAHP
DOCK2	FLOT1
KRT7	COMMD1
CD74	HOXC6
KATNAL1	PIP5K1C
ARHGEF25	ARPC1B
SERPINA3	NECAP2
ATP1A2	TMEM71
CHGB	MAGEL2
RNF181	RRAGD
ST6GALNA	HBP1
LRRK2	FLII
TYRP1	ELOVL4
LACTB	CIC
MORN4	FRMD5
BMPR1B	CLEC5A
CPEB1	BCHE
STXBP1	FLRT3
FOXP2	AGPAT9
CDO1	KLHDC1
MFSD11	CYS1
MICU1	FABP4
TNNT1	CPA3
FOXJ2	GSTA2 ||| GSTA1
AADAC	CLIC3

GPR34	GATM
CD82	DPP4
OR51E1	FLYWCH1
ZFP90	C3orf14
EPHA7	SYT1
UBASH3B	PSMB6
DTNA	FRAS1
RAMP1	TFF2
OLFM1	UBC
GJB5	SPRR1B
ADRA2A	SIX4
MAMDC2	ADIPOQ
C5orf46	ST6GAL2
EVI2A	GHR
HIST1H1C	TMPRSS3
SRGN	L1CAM
FGFR2	SNX27
PCDHB16	GDI1
PLA2G10	PKIB
ANXA2P2	NLRP2
TRIM6	CALM3 ||| CALM2 ||| CALM1
DKK1	NTRK2
MAP2K1	ATP6V1E1
IGFL2	MAGEA12
PTPRN2	
RAI1	
MYBPC1	
TLR1	
SWAP70	
CACHD1	
CPE	
SFTA2	
ATL1	

MUM1L1
MYOCD
SLPI
PCDHB5
SUN2
ACOT9
PRKACB
POPDC2
PALM
LDB3
NPY1R
FAM26E
ALOX5
DARC
VSIG2
CDH2
RAB11FIP5
SLC4A11
SEMA4C
DDX24
DHRS9
CLDN10
GNAI1
FCGR1A /// FCGR1B
RHBDL2
RXRB
FOLR1
CLEC7A
C6orf1
C2orf76
STX5
ANXA10
PKIA

CMAHP
FLOT1
COMMD1
HOXC6
PIP5K1C
ARPC1B
NECAP2
SERPINB5
TMEM71
MAGEL2
RRAGD
HBP1
S100A2
FLII
ELOVL4
CIC
FRMD5
CLEC5A
BCHE
FLRT3
AGPAT9
KLHDC1
MMP7
CTSE
CYS1
NXF3
FABP4
CPA3
GSTA2 ||| GSTA1
CLIC3
GATM
DPP4
FLYWCH1

CLSTN2
C3orf14
SYT1
PSMB6
FRAS1
TFF2
UBC
SPRR1B
SIX4
ADIPOQ
ST6GAL2
GHR
TMPRSS3
L1CAM
SNX27
GDI1
HOXB8
PKIB
NLRP2
CALM3 ||| CALM2 ||| CALM1
NTRK2
ATP6V1E1
GABRP
MAGEA12
TACSTD2
DUSP27

Basal pancreatic cancer cells (Raghavan et al. 202	YAP_22 (Wang et a	Fetal (Mustata et al. 2013)	coreHRC
KRT6A	CYR61	SPP1	LAMB3
S100A2	CTGF	GJA1	SDCBP2
KRT13	AMOTL2	BASP1	EMP1
KRT17	ANKRD1	TACSTD2	EPS8L1
LY6D	IGFBP3	SFTPD	JUP
KRT7	F3	MAL	LMO7
KRT5	FJX1	CLU	MYOF
FAM83A	NUAK2	WFDC2	ITGB4
CD109	LATS2	MSLN	GPRC5A
GAPDH	CRIM1	VSIG1	AHNAK
CSTA	GADD45A	TUBB6	CAMK2N1
PTPN13	TGFB2	CRIP2	C19orf33
MTSS1	PTPN14	ANXA1	MYH14
SPRR1B	NT5E	COL4A1	CD55
SLC2A1	FOXF2	IL33	RHOF
CAV1	AXL	COL4A2	TMBIM1
EMP1	DOCK5	KRT4	SEMA3B
SEMA3C	ASAP1	IGFBP7	TINAGL1
DHRS9	RBMS3	AKAP13	MISP
CAST	MYOF	ECSCR	COL17A1
FLNA	ARHGEF17	GKN1	PLEC
SLITRK6	CCDC80	CDH13	TSPAN1
COL7A1		SLC45A3	ABHD11-AS1
AQP3		EFNA5	PTPRH
MT2A		ANXA8	CRB3
AHNAK2		ACSM3	ITGA3
KRT19		PPIC	RIOK3
PKP1		ANXA3	LAMC2
YBX3		EDA2R	CYP3A5
GJB5		S100A6	FA2H
		EFEMP1	RRAS

DDIT4L	MUC13
PDZK1IP1	CEACAM1
LY6D	TMPRSS2
LAPTM5	SERINC2
PLEKHS1	SLC44A4
HS6ST2	PCDH1
SLC4A11	USP53
CRYAB	C1orf106
SH3KBP1	FLNB
SAMD5	CXCL16
PHLDA3	EZR
KRT7	TNFRSF21
IL1RN	CDA
CCND1	ADAM9
PLAUR	RNF103
SYT8	KRT80
TMEM40	EFNB1
PTP4A3	SMAD3
GPX2	PDZK1IP1
PSAPL1	LRP10
S100A11	PRSS22
PSCA	AHNAK2
SOX21	B4GALT1
FABP5	ANXA11
COL18A1	BAIAP2L1
CD14	F11R
PMM1	DUSP5
PLK2	DDR1
CLIC3	ITGB1
NCMAP	SLC2A1
EREG	CDKN2B
ICAM1	PLAUR
CCNG1	ITGA2

RBMS1	CDCP1
MGAT4C	CD59
RDH10	DSG2
ENAH	KLK10
VNN1	ARFGAP3
EPHA4	CD68
PDLIM4	CGN
TNNI2	TM4SF1
IER3	PPP2CB
GLIPR1	HEBP2
IL17RE	UGCG
PRSS22	GBP3
TSPAN6	TIMP2
TNS4	PINK1
S100A14	CYSTM1
LAMC2	SLC6A8
ECM1	PPARD
SCEL	LIPH
OSBPL5	CLIC3
TLR4	SPINT1
TIMP2	PTK6
TREX2	TJP3
GATM	STK24
ZIC4	AC009133.12
ARL4C	CEACAM5
WWTR1	MXD1
AQP5	SNX9
CAPN2	ERO1L
CCP110	SSFA2
EMP2	TSPAN3
ABCC4	SLPI
HYAL1	BMP2
IFT57	GJB3

SLC25A48
PSRC1
SLC44A2
HSPA1A
PPL
MGMT
CXCL16
P2RY6
DDAH1
PMEPA1
GCNT1
CGNL1
EMP1
BOK
ILDR1
ARHGEF3
MSN
DCXR
SPATA6
ARHGAP44
RGS19
NOXO1
VANGL1
S100A4
TSPAN4
GPR137B
CCND2
TTC9
PDRG1
VAMP5
FAR1
SERPINB5
CTSH
LAMA3
TMPRSS4
B4GALNT3

AKR1B10
RAB15
ZMAT3
AHNAK
KRT18
DAPK1
FLOT2
AVPI1
RAB11FIP5
PAQR8
SERPINB11
PRKAR1A
LAMA3
TINAGL1
MCAM
NFE2L3
SH3BGRL2
LMNA
SCMH1
PDLIM7
FOXQ1
GCNT3
TMEM43
MDM2
GTSE1
GPC1
CAMSAP2
HSPB1
EPS8L1
CKAP2
GOLGA8S
PLAT
WWC2

INF2
MRAS
CHRNB1
ANXA5
AK1
VAX1
TEAD4
DYRK3
ITGB4
BAX
ARL1
RHOD
MSANTD3
KLRG2
CLCF1
ID2
SGCB
AEN
ABCC5
FLNC
PIR
FMNL2
CD44
SLC39A10
ENDOD1
MBOAT1
KIFC3
CCDC92
SUSD4
CNN2
GNAI1
RAB27B
TSPAN2

USP46
P2RY2
CD55
KRT23
GSN
S100A16
TBC1D2
SORL1
FKBP9
AAAS
EPN3
FRMD6
ALCAM
ABI3
B9D1
ZBTB32
GJB3
PLCD3
BTG2
SLC19A2
SLCO4A1
BAIAP2
BIVM-ERCC5
ABHD4
KCNN4
DHH
AP1S3
WNT4
GLIS3
TNFAIP8
MLKL
PHLDA2
FOSL1

DUSP22
HSPA2
WTIP
KLHL13
CAMKK1
CAPN5
ICA1
TNFRSF10B
GADD45B
EXOC4
RGS17
STX11
RPS19
GUSB
SERPINH1
CPT1C
BTBD11
FAM149A
